# Automated mass spectrometry imaging of over 2000 proteins from tissue sections at 100-μm spatial resolution

**DOI:** 10.1038/s41467-019-13858-z

**Published:** 2020-01-07

**Authors:** Paul D. Piehowski, Ying Zhu, Lisa M. Bramer, Kelly G. Stratton, Rui Zhao, Daniel J. Orton, Ronald J. Moore, Jia Yuan, Hugh D. Mitchell, Yuqian Gao, Bobbie-Jo M. Webb-Robertson, Sudhansu K. Dey, Ryan T. Kelly, Kristin E. Burnum-Johnson

**Affiliations:** 1 Biological Sciences Division, Pacific Northwest National Laboratory, Richland, WA USA; 2 The Environmental Molecular Sciences Laboratory, Pacific Northwest National Laboratory, Richland, WA USA; 3 National Security Directorate, Pacific Northwest National Laboratory, Richland, WA USA; 4 Cincinnati Children’s Hospital Medical Center, Cincinnati, OH USA; 5 Department of Chemistry and Biochemistry, Brigham Young University, Provo, UT USA

**Keywords:** Analytical biochemistry, Microfluidics, Proteomics

## Abstract

Biological tissues exhibit complex spatial heterogeneity that directs the functions of multicellular organisms. Quantifying protein expression is essential for elucidating processes within complex biological assemblies. Imaging mass spectrometry (IMS) is a powerful emerging tool for mapping the spatial distribution of metabolites and lipids across tissue surfaces, but technical challenges have limited the application of IMS to the analysis of proteomes. Methods for probing the spatial distribution of the proteome have generally relied on the use of labels and/or antibodies, which limits multiplexing and requires a priori knowledge of protein targets. Past efforts to make spatially resolved proteome measurements across tissues have had limited spatial resolution and proteome coverage and have relied on manual workflows. Here, we demonstrate an automated approach to imaging that utilizes label-free nanoproteomics to analyze tissue voxels, generating quantitative cell-type-specific images for >2000 proteins with 100-µm spatial resolution across mouse uterine tissue sections preparing for blastocyst implantation.

## Abstract (web summary)

Imaging mass spectrometry is a powerful emerging tool for mapping the spatial distribution of biomolecules across tissue surfaces. Here the authors showcase an automated technology for deep proteome imaging that utilizes ultrasensitive microfluidics and a mass spectrometry workflow to analyze tissue voxels, generating quantitative cell-type-specific images.

## Introduction

Imaging mass spectrometry (IMS, also termed mass spectrometry imaging (MSI)) is a powerful tool for mapping the spatial distribution of biomolecules across a tissue of interest. In an IMS experiment, a probe, which may be a laser, ion beam, or liquid junction, is rastered across a surface to desorb or extract biomolecules that are then directly analyzed by mass spectrometry (MS). This allows for the creation of detailed spatial maps that reveal the native distribution of biomolecules at the surface without labels or pretreatment. However, there are limitations to these approaches, particularly for the analysis of proteins^1,2^. First, molecules are transmitted directly from the sample to the mass spectrometer without separation, limiting the dynamic range of observed analyte concentrations and restricting detection to the most abundant species. As a result, IMS experiments as applied to proteome profiling are limited to the most abundant 5% of proteins present in the tissue or cell model^3–5^. Second, the ionization efficiency for a given analyte is strongly impacted by the other constituents in the mixture, making quantitative comparisons challenging.

A single matrix-assisted laser deposition/ionization (MALDI) IMS experiment can produce thousands of ion images, providing molecular context to classical histological analysis, yet in order to identify the proteins, fragmentation data are often collected in separate experiments (reviewed in ref. ^6^) either directly from tissue or by liquid chromatography-tandem MS (LC-MS/MS) following extraction^6–8^. Protein coverage using IMS can be improved through on-tissue digestion, but confident in situ MS/MS peptide identification remains challenging due to low signal-to-noise ratios and high spectral complexity that impede database identifications. To increase the number of identified peptides, researchers have coupled IMS with LC-MS, where one tissue section is analyzed by IMS, while an adjacent section is homogenized and analyzed by LC-MS/MS^9–11^. However, linking these two MS modalities is challenging due to the high complexity of mammalian tissue sections, which has led to false-positive assignments^12^. As a result, there is currently no IMS technology capable of in-depth proteome imaging.

Proteomics methods based on LC-MS/MS analysis have become an indispensable tool for biological research^13,14^. Significant investment has been made in developing robust methodologies for quantitative proteomics to monitor changes in the proteome between different patients and/or treatment conditions^15–17^. This powerful approach offers a highly comprehensive and quantitative molecular profile of the specimen of interest. To achieve this in-depth coverage and measurement accuracy, proteins need to be extracted, digested into peptides, and separated by LC for effective MS analysis^18^. Analyte losses during this multistep processing due to surface adsorption can lead to larger sample requirements than would otherwise be necessary. Consequently, the requisite bulk extraction process blurs spatial information about differing cell types and tissue contexts, which are critical to obtaining a systems-level understanding of the specimen. The approach has been extended to proteome mapping using a “voxelation” approach, though the lateral resolution was limited to 1 mm due to sample handling and technical constraints^19^. To address this challenge, proteomic approaches have been combined with isolation techniques such as laser capture microdissection (LCM) and fluorescence-activated cell sorting (FACS); however, these applications are narrow to date, due to the limited amount of sample mass obtainable^8,20–24^.

To address the sensitivity limitations of existing proteomics workflows, we have developed a microfluidic sample preparation platform termed nanoPOTS (Nanodroplet Processing in One pot for Trace Samples), which dramatically increases proteome coverage for small samples, extending to single mammalian cells^25^. The combination of robotic nanopipetting, a microfabricated glass nanowell chip, and a one-pot processing workflow enable all sample preparation steps to take place in a ~200-nL volume, thereby reducing adsorptive losses to the surface of the reaction vessel and maintaining sufficient protein concentrations for efficient in-solution proteolytic digestion. In combination with FACS and ultrasensitive nanoLC-MS/MS, nanoPOTS has enabled nearly 700 proteins to be identified from single mammalian cells^26^. In addition, nanoPOTS has been combined with LCM to isolate and profile proteins within regions of interest in pancreas, brain and liver thin sections, as well as plant tissues^8,25,27,28^. Following sample processing of biological material into digested peptides, the samples were collected into capillary columns and concentrated onto solid-phase extraction (SPE) columns. These columns were manually inserted at the head of a nanoLC column and then injected for LC-MS/MS analysis. While in principle the previously described workflow could have been extended to high-resolution proteome imaging, the manual analysis and the lack of informatics tools precluded the generation and interpretation of in-depth proteome images.

In this work, we demonstrate high-resolution and in-depth proteome imaging using an automated workflow. First, we have coupled the nanoPOTS sample-processing platform with LCM, which provides automated sample collection and processing with unprecedented sensitivity^25,29^. Second, we paired these nanogram-quantity samples with a custom-designed LC system to achieve sensitive, reproducible analysis with robust, automated data capture, allowing confident analysis of the large sample sets required to create proteome maps.

In this first-of-its-kind application, we analyzed uterine cross sections from pregnant mice prior to the adhesion of early embryos. The luminal epithelial (LE) cells lining the uterine cavity are surrounded by stromal (S) cells and dispersed glandular epithelial (GE) cells. These cells show unique cell-type-specific protein expression in preparation for the attachment of early embryos to the LE, and subsequent invasion into the S. Distinct molecular signatures across the heterogeneous landscape of the mouse uterus during early pregnancy has made this an ideal model system for evaluating other imaging techniques such as matrix-assisted laser desorption ionization (MALDI) IMS^30,31^ and nanodesorption electrospray ionization (NanoDESI) IMS^32–35^ in previous studies. In this study, our proteomic imaging platform was capable of mapping >2000 proteins with 100-µm spatial resolution, thereby capturing the unique protein expression patterns of the LE, S, and GE cell types. Visualization of this large dataset was made possible through the development of a custom implementation of the powerful, open-source platform Trelliscope^36^, which also serves as an interactive, web-based interface for facile data dissemination. The application of this innovative platform to proteome mapping in a mouse uterus model system clearly demonstrates the exciting potential of proteome imaging to advance biomedical research.

## Results

### NanoPOTS imaging platform workflow

**Fig. 1:**
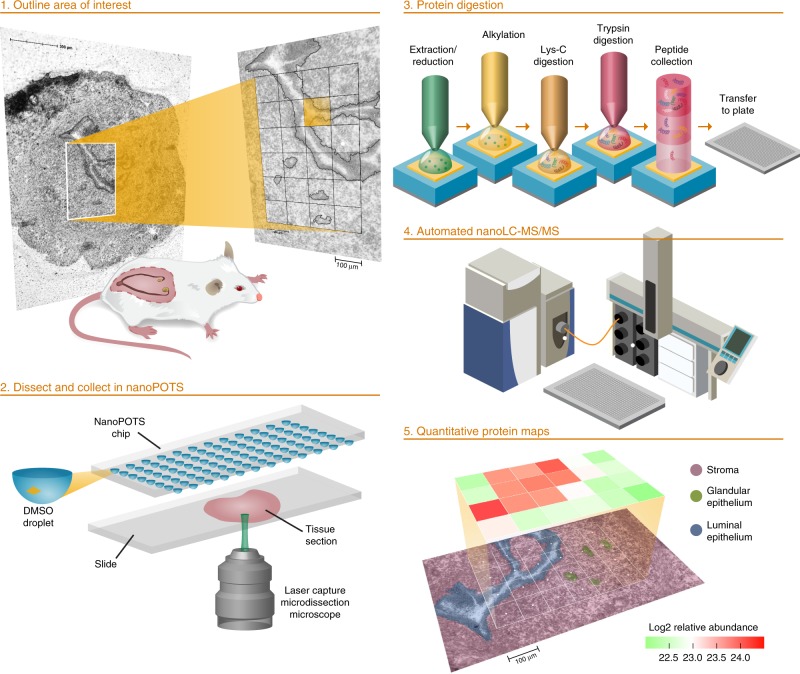
Schematic workflow for high-throughput, spatially resolved proteomics using the nanoPOTS imaging platform. The authors thank PNNL Graphic Designer Nathan Johnson for preparing the figure.

Our approach combines existing technology with a suite of technologies recently developed in our lab to achieve the robustness, sensitivity, and throughput that are essential for proteome-level imaging. A schematic of the workflow is shown in Fig. 1. Briefly, tissue voxels are created using LCM and captured directly into the nanoPOTS chip by pre-populating the nanowells with DMSO “capture solvent”^8^. Automated proteomic sample preparation is then carried out on-chip to minimize surface area exposure as was described previously^25^. The digested peptides are then transferred to a 96-well plate that has been prepopulated with 20 µL of LC buffer A (0.1% formic acid in water) in each well, which aids in reproducible droplet transfer while minimizing peptide losses. Samples are then transferred to a custom LC system equipped with a zero-dead-volume injection needle (Supplementary Fig. 1) to ensure full sample injection. Peptide separation is achieved using an in-house-packed capillary column with a 50-µm internal diameter and an integrated electrospray emitter tip to maximize sensitivity while maintaining the robust operation required for increased throughput. MS analysis was done using a QExactive Orbitrap in data-dependent mode with a 150-ms maximum ion time to allow for longer accumulation to accommodate lower ion fluxes. Datasets are then processed with MaxQuant utilizing the match-between-runs (MBR) option to reduce missing data. Sample datasets are then registered with coordinates from LCM dissection and protein and peptide data are visualized using a Trelliscope.

### Platform sensitivity and reproducibility

**Fig. 2:**
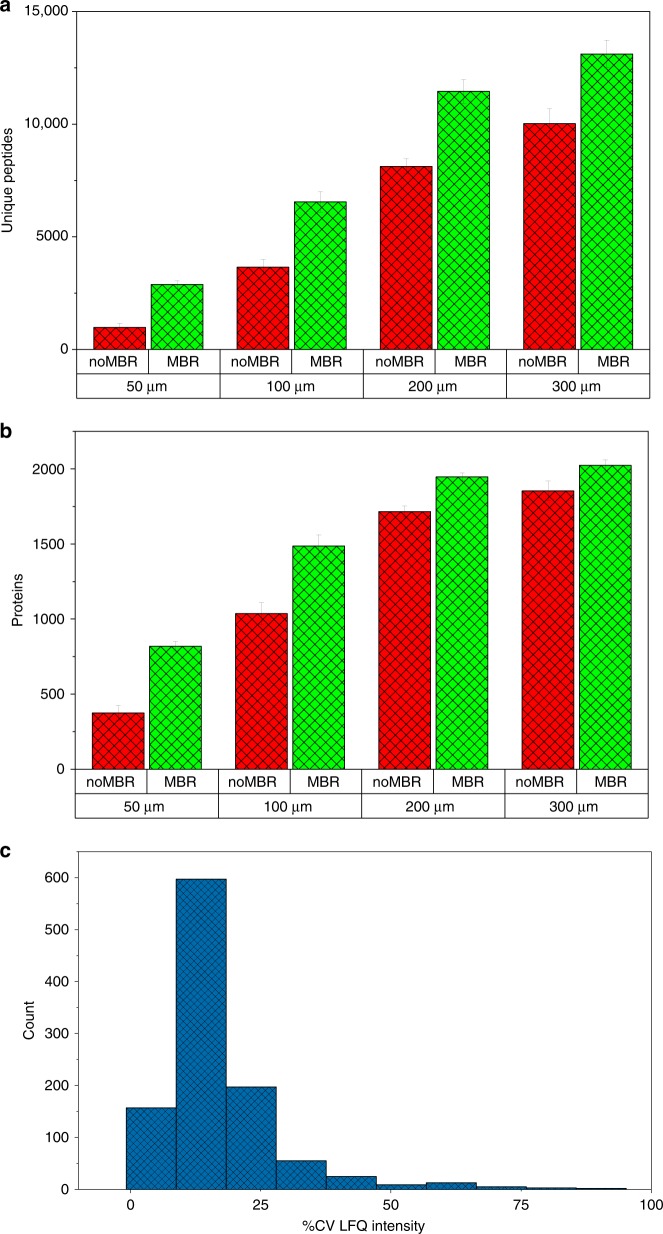
Reproducibility of nanoPOTS analysis on liver tissue. **a** Mean number of peptide identifications from four replicate analyses of liver tissue voxels at different lateral resolutions, with and without MBR enabled. **b** Mean number of protein identifications from four replicate analyses of liver tissue voxels at different lateral resolutions, with and without MBR enabled. **c** Histogram of protein LFQ intensity coefficient of variation (CV) for 20 replicate voxels from homogeneous tissue sections. Error bars, standard deviation.

To demonstrate the reproducibility and sensitivity achievable with this platform, we used mouse liver tissue as a model system. Liver was chosen due to its relative homogeneity on the size scale used in this study. First, square tissue voxels of decreasing area were cut from the liver tissue, with four replicate voxels analyzed at each size. Figure 2a, b shows the peptide and protein coverage, respectively, as a function of lateral resolution. As expected, protein coverage decreases as voxel area is decreased due to the resulting reduction in protein-loading mass. However, when MaxQuant MBR is employed, >800 proteins can still be identified with two unique peptides at 50-µm lateral resolution. Second, reproducibility is critical to producing quantitative protein maps. To establish the reproducibility of our imaging platform, 20 replicate voxels were dissected from a homogeneous region of liver tissue and analyzed using the nanoPOTS imaging platform. Figure 2c shows coefficients of variation (CVs) for protein quantification across the 20 datasets. Using normalized MaxQuant LFQ intensity gives a median CV of 14.4%, which indicates that robust quantification is achievable with this platform.

### Proteome analysis of cell types in *Wnt5a*-null uterine tissue

To validate the findings in our proteomic images, we performed a complementary study in which we used LCM, nanoPOTS, and LC-MS/MS analyses to isolate, characterize, and statistically compare LE, S, and GE cells across multiple *Wnt5a*-null mouse uterine tissue sections. This dominant cell population study contained 15 LC-MS/MS instrument runs associated with 15 unique biological samples, 5 S samples, 5 LE samples, and 5 GE samples, in which 100–200 ng of these unique cell populations were captured from three to five sections for each of the 15 samples. From the MaxQuant MBR search, 19,952 peptides had at least two observations across the 15 analyses. The algorithm RMD-PAV was used to identify any outlier biological samples^37^. Samples were also examined via Pearson correlation and no samples were identified as outliers. Peptides found to have inadequate data for either qualitative or quantitative statistical tests were also removed from the dataset, resulting in a final dataset for normalization that included 15 unique biological samples and 17,387 measured unique peptides corresponding to 2940 unique proteins. Median centering based on rank-invariant peptides (with *p* value threshold for rank invariance of 0.2) was used for normalization^38^. Protein quantification was performed using R-rollup, which scales the peptides associated with each protein by a reference peptide and then sets their median as the protein abundance^39^. The peptide having the least missing data is selected as the reference peptide. Pairwise-univariate statistical comparisons were carried out between each of the three cell types using a Tukey-adjusted ANOVA or a Holm-adjusted *g* test to compare each pair of dominant cell types for each of the 2940 proteins^38^. The three statistical comparisons of interest were (1) LE vs. GE, (2) S vs. GE, and (3) S vs. LE. The number of significant proteins (adjusted *p* value <0.05) for each of the three comparisons based on the ANOVA-adjusted *p* values were (1) 1220 proteins increasing in the LE and 46 proteins increasing in the GE, (2) 1673 proteins increasing in the S and 42 proteins increasing in the GE, and (3) 777 proteins increasing in the S and 196 proteins increasing in the LE.

### Proteome imaging of *Wnt5a*-null uterine tissue

**Fig. 3:**
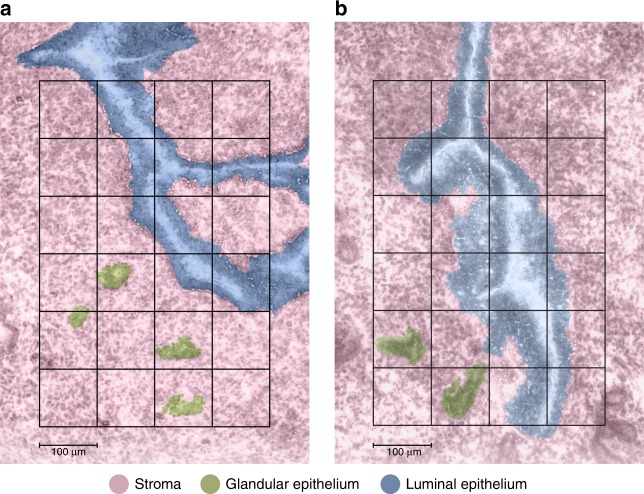
Pseudocolor optical micrographs of the imaged tissue sections with voxel pattern overlay. **a** Stromal-dominant image and **b** luminal epithelium-dominant image. Scale bar, 100 µm. The authors thank PNNL Graphic Designer Nathan Johnson for preparing the figure.

The nanoPOTS proteomic imaging platform was then used to create 2D protein images of tissue sections comprising the three cell types of interest. Pseudocolor optical images of the imaging area and voxel boundaries are shown in Fig. 3a, b. To display these 2D protein images, we developed a Trelliscope software platform that allowed us to explore the images and correlate them to the statistically significant results from the dominant cell population study. This software platform enabled us to share all results from this study in a searchable and customizable approach (**see** Supplementary Trelliscope Video Tutorial).

**Fig. 4:**
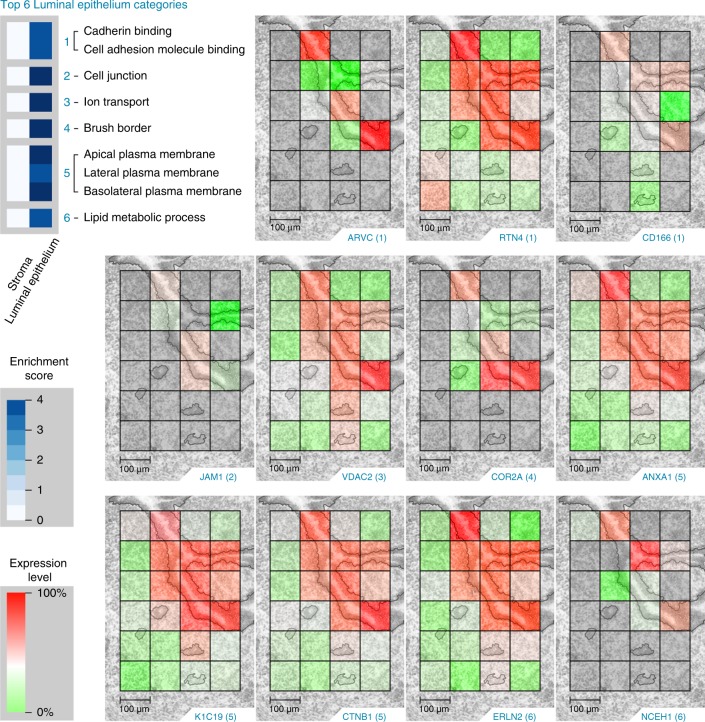
The top six luminal epithelium (LE) Gene Ontology categories (top, left) enriched in the statistically significant (Tukey-adjusted ANOVA or a Holm-adjusted *g* test, *p* value <0.05) proteins from the dominant cell population study and the corresponding protein images. (1) Armadillo repeat protein deleted in velo-cardio-facial syndrome homolog (ARVC), reticulon-4 (RTN4), and CD166 antigen (CD166); (2) junctional adhesion molecule A (JAM1); (3) voltage-dependent anion-selective channel protein 2 (VDAC2); (4) coronin-2A (COR2A); (5) annexin A1 (ANXA1), keratin type I cytoskeletal 19 (K1C19), and catenin beta-1 (CTNB1); (6) erlin-2 (ERLN2), a neutral cholesterol ester hydrolase 1 (NCEH1). Scale bars, 100 µm. The authors thank PNNL Graphic Designer Nathan Johnson for preparing the figure.

**Fig. 5:**
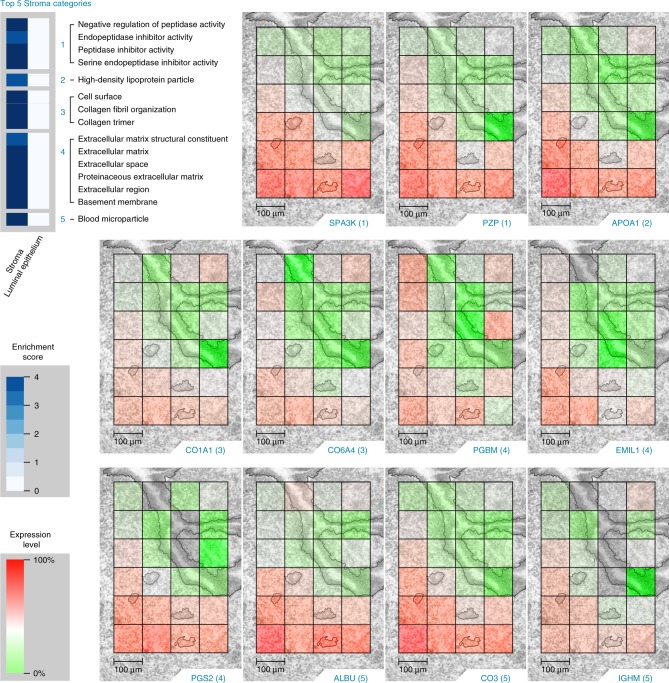
The top 5 stroma (S) Gene Ontology categories (top, left) enriched in the statistically significant (Tukey-adjusted ANOVA or a Holm-adjusted *g* test, *p* value <0.05) proteins from the dominant cell population study and the corresponding protein images. (1) Serine protease inhibitor A3K (SPA3K), pregnancy zone protein (PZP); (2) apolipoprotein A-I (APOA1); (3) collagen alpha-1(I) chain (CO1A1), collagen alpha-4(VI) chain (CO6A4); (4) basement membrane-specific heparan sulfate proteoglycan core protein (PGBM), EMILIN-1 (EMIL1), Decorin (PGS2); (5) serum albumin (ALBU), complement C3 (CO3), and immunoglobulin heavy constant mu (IGHM). Scale bars, 100 µm. The authors thank PNNL Graphic Designer Nathan Johnson for preparing the figure.

**Fig. 6:**
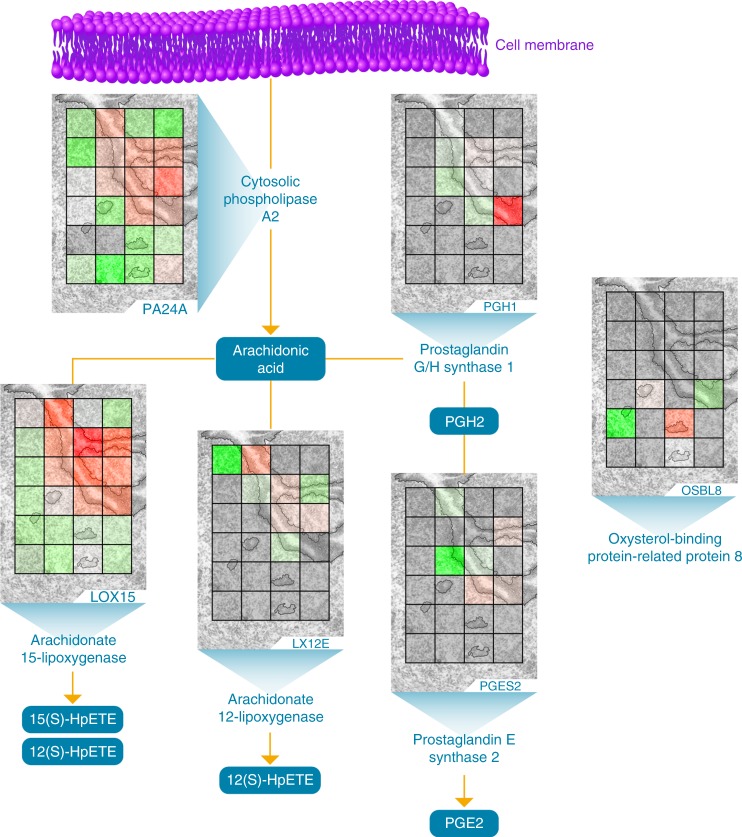
Arachidonic acid metabolism localizes to the luminal epithelium. Prostaglandin H2 (PGH2), prostaglandin E2 (PGE2), 12(S)-hydroperoxyeicosatetraenoic acid (12(S)-HpETE), 15(S)-hydroperoxyeicosatetraenoic acid (15(S)-HpETE). The authors thank PNNL Graphic Designer Nathan Johnson for preparing the figure.

Imaged areas were taken from the center of uterine sections, enabling visualization of the proteomic landscape of the uterus orchestrating embryo implantation. The S-dominant tissue section (depicted in Figs. 4–6) comprises 24 LC-MS/MS instrument runs associated with 24 unique voxels, 4 containing GE and S, 8 containing LE, and 12 containing S (Fig. 3a). The LE-dominant tissue section (depicted in Supplementary Figs. 2 and 3) also contains 24 LC-MS/MS instrument runs associated with 24 voxels, in this case with 2 containing GE and S, 14 containing LE, and 8 containing S (Fig. 3b). MaxQuant analysis of our S-dominant section characterized 8065 unique peptides corresponding to 1658 unique proteins that had at least two observations across the 24 analyses. Employing the MBR feature characterized 9411 unique peptides corresponding to 1764 unique proteins that had at least two observations across the 24 runs. MaxQuant analysis of the LE-dominant section characterized 11,803 unique peptides corresponding to 2212 unique proteins that had at least two observations across the 24 runs. Employing MBR characterized 13,797 unique peptides corresponding to 2357 unique proteins that had at least two observations across the 24 runs. Median centering based on rank-invariant peptides (0.2) was used for normalization. Our searchable Trelliscope software platform ([http://msc-viz.emsl.pnnl.gov/nanoPOTS_PI_MS/]) contains images of all 2298 and 2447 quantifiable MaxQuant and MaxQuant MBR proteins, respectively, and 12,495 and 14,673 quantifiable MaxQuant and MaxQuant MBR peptides, respectively, from the S- and LE-dominant sections.

### Functional analysis of tissue-type differences

Proteins of interest discussed in the paper were statistically significant (<0.05 adjusted *p* value) in our dominant cell-type data and had complementary spatial distributions in our proteome-imaging data. Of these proteins of interest, 149 are enriched in the LE and 175 are enriched in the S (Supplementary Tables 1 and 2). Supplementary Data 1 and 2 contain the images, from both MaxQuant and MaxQuant MBR, for these proteins in addition to the associated box plots from the dominant cell population study. Although we provide both MaxQuant and MaxQuant MBR data in Supplementary Data 1 and 2, the images in the paper are from MaxQuant unless otherwise specified.

Proteins detected in the GE exhibit a high degree of overlap with the LE and S-expression patterns since 50 of the 149 proteins enriched in the LE were also enriched in the GE and 27 of the 175 proteins enriched in the S were also enriched in the GE (Supplementary Tables 1 and 2). An important goal of this study was to characterize the unique proteomic landscapes of LE cells, which are the first cells to attract and make contact with the early embryo (blastocyst), and the S cells, which support embryo growth during early pregnancy. Our 100-µm voxel size was sufficient to capture the LE cells lining on both sides of the uterine cavity. The LE-localized protein images in Fig. 4 and S-localized protein images in Fig. 5 were selected by correlating gene ontology (GO) categories between our images and our statistically significant results. Negative log10 *p* values from these tests are indicated by white-to-blue color intensity enrichment scores in Figs. 4 and 5.

The top six LE GO categories and the corresponding images depicted in Fig. 4 include (1) cadherin binding and cell adhesion molecule binding with protein images armadillo repeat protein deleted in velo-cardio-facial syndrome homolog (“ARVC [https://www.uniprot.org/uniprot/P98203]”), reticulon-4 (“RTN4 [https://www.uniprot.org/uniprot/Q99P72]”), and CD166 antigen (“CD166 [https://www.uniprot.org/uniprot/Q61490]”); (2) cell junction with protein image junctional adhesion molecule A (“JAM1 [https://www.uniprot.org/uniprot/O88792]”); (3) ion transport with protein image voltage-dependent anion-selective channel protein 2 (“VDAC2 [https://www.uniprot.org/uniprot/Q60930]” with MBR); (4) brush border with protein image coronin-2A (“COR2A [https://www.uniprot.org/uniprot/Q8C0P5]” with MBR); (5) apical, lateral, and basolateral plasma membrane with protein images annexin A1 (“ANXA1 [https://www.uniprot.org/uniprot/P10107]”), keratin type I cytoskeletal 19 (“K1C19 [https://www.uniprot.org/uniprot/P19001]”), and catenin beta-1 (“CTNB1 [https://www.uniprot.org/uniprot/Q02248]”); (6) lipid metabolic process with protein images erlin-2 (“ERLN2 [https://www.uniprot.org/uniprot/Q8BFZ9]” with MBR) and neutral cholesterol ester hydrolase 1 (“NCEH1 [https://www.uniprot.org/uniprot/Q8BLF1]” with MBR). The corresponding protein images for the LE-dominant tissue section can be found in Supplementary Fig. 2. These proteins have functional roles molecularly linked to epithelial cell crypt formation such as actin cytoskeleton remodeling, cell polarization, and cell migration. In addition, proteins such as “CTNB1 [https://www.uniprot.org/uniprot/Q02248]” are molecularly linked to our *Wnt5a*-null phenotype^40^.

The top 5 S GO categories and the corresponding images depicted in Fig. 5 include (1) peptidase inhibitor activity with protein images serine protease inhibitor A3K (“SPA3K [https://www.uniprot.org/uniprot/P07759]”) and pregnancy zone protein (“PZP [https://www.uniprot.org/uniprot/Q61838]”); (2) high-density lipoprotein particle with protein image apolipoprotein A-I (“APOA1 [https://www.uniprot.org/uniprot/Q00623]”); (3) collagen organization with protein images collagen alpha-1(I) chain (“CO1A1 [https://www.uniprot.org/uniprot/P11087]”) and collagen alpha-4(VI) chain (“CO6A4 [https://www.uniprot.org/uniprot/A2AX52]” with MBR); (4) extracellular matrix and basement membrane with protein images basement membrane-specific heparan sulfate proteoglycan core protein (“PGBM [https://www.uniprot.org/uniprot/Q05793]” with MBR), EMILIN-1 (“EMIL1 [https://www.uniprot.org/uniprot/Q99K41]” with MBR), Decorin (“PGS2 [https://www.uniprot.org/uniprot/P28654]” with MBR); (5) blood microparticle with protein images serum albumin (“ALBU [https://www.uniprot.org/uniprot/P07724]”), complement C3 (“CO3 [https://www.uniprot.org/uniprot/P01027]”), and immunoglobulin heavy constant mu (“IGHM [https://www.uniprot.org/uniprot/P01872]” with MBR). The corresponding protein images for the LE-dominant tissue section can be found in Supplementary Fig. 3. Molecular epithelial–stromal cell crosstalk is essential for successful embryo implantation, and many of these significantly changing S proteins have functional roles molecularly linked to remodeling of the extracellular matrix. In addition, blood-associated proteins, including immune proteins IGHM and CO3, significantly increased in S cells compared with LE cells where they were not detected or detected at low levels; the avascular LE cells aid the blastocyst in escaping the maternal immune surveillance at the time of implantation.

### Comparison to known lipid-mediated metabolic processes

Corroborating our LE-specific increase in lipid metabolic processes (Fig. 4), arachidonic acid-derived lipid mediators are known to play an essential role in embryo implantation^41–43^. We have recently shown, using in situ metabolome-imaging techniques, that prostaglandins (PG) including PGE2 localize to the LE in uterine sections obtained from the same *Wnt5a*-null mouse analyzed in this study^32^. As illustrated in Fig. 6, our nanoPOTS proteomic images mapped cytosolic phospholipase A2 (“PA24A [https://www.uniprot.org/uniprot/P47713]”), prostaglandin G/H synthase 1 (“PGH1 [https://www.uniprot.org/uniprot/P22437]”, also named cyclooxygenase-1), and prostaglandin E synthase 2 (“PGES2 [https://www.uniprot.org/uniprot/Q8BWM0]” with MBR) expression to LE cells. PA24A hydrolyzes arachidonic acid from the sn-2 position of phospholipids; free arachidonic acid is then metabolized into prostaglandins such as PGH2 and PGE2. In addition, our nanoPOTS proteomic images also captured the LE localization of arachidonate 12-lipoxygenase (“LX12E [https://www.uniprot.org/uniprot/P55249]”) and arachidonate 15-lipoxygenase (“LOX15 [https://www.uniprot.org/uniprot/P39654]”), which metabolize arachidonic acid into bioactive lipid mediators, 12(S)-hydroperoxyeicosatetraenoic acid (12(S)-HpETE) and 15(S)-hydroperoxyeicosatetraenoic acid (15(S)-HpETE). Progesterone-induced synthesis of the 12/15-LOX-derived lipid mediators in LE cells activates a critical regulator of embryo implantation, the nuclear receptor peroxisome proliferator-activated receptor γ (PPARγ) and its downstream gene networks^44^. In addition to an LE-specific increase in lipid metabolic processes, our nanoPOTS proteomic images mapped oxysterol-binding protein-related protein 8 (“OSBL8 [https://www.uniprot.org/uniprot/B9EJ86]”) expression to voxels containing GE (Fig. 6). OSBL8 plays an essential role in lipid transfer between cell membrane bilayers at contacts between the endoplasmic reticulum and other membranes to aid in maintaining membrane lipid homeostasis^45^.

## Discussion

Herein, we establish the potential for in-depth, high-lateral-resolution imaging of the proteome across tissues using an automated nanoPOTS workflow. The approach combines the high sensitivity of the nanoPOTS approach with LCM and a custom, automated sample transfer and analysis platform that enables complete sample utilization and robust operation. When taken together, this platform enables quantitative mapping of >2000 proteins with 100-µm spatial resolution. The sensitivity of this approach was demonstrated through replicate analysis of liver tissue voxels of decreasing size. Further, analyzing 20 replicate tissue voxels from this relatively homogeneous tissue produced CVs similar to bulk analysis, indicating platform reproducibility and stability. We then verified the ability of our imaging platform to find meaningful differences using a mouse embryo implantation model system. Many of the proteins quantified in the proteome images showed differential expression across different tissue features. These differences were then confirmed by adjusted *p* values from multiple comparison testing of highly enriched cell-type pools from the same tissues using LCM. The high level of agreement between these analyses indicates the great potential of this approach as a discovery tool. The depth of proteome coverage and robust quantitation achievable with this platform greatly exceed those of existing IMS platforms.

This nanoPOTS proteomic imaging analytical platform enabled us to visualize proteome-level cell-type-specific alterations across mouse uterine tissue sections preparing for blastocyst implantation. A reciprocal interaction between a blastocyst and the receptive uterus is critical to successful pregnancy. In mice, LE cells undergo extensive remodeling to create implantation chambers (crypts) formed by the evagination of these epithelial cells; during the initial apposition, adhesion, and attachment steps of embryo implantation, blastocysts are positioned within these crypts^46^. Mice with uterine-specific deletion of *Wnt5a*, which exhibit haphazard crypt formation and enhanced molecular transformation across all LE cells, were imaged in this study. Thus, proteins detected represent samples of deleted uteri and may vary in normal uteri. The nanoPOTS imaging platform generated quantitative images for >2000 proteins across cellular regions in the *Wnt5a*-null uterus with 100-µm spatial resolution. An important goal of this study was to characterize the unique proteomic landscapes of LE cells, the first cells to attract and make direct contact with the blastocyst, and the S cells, which support embryo growth during early pregnancy. Images of proteins localizing to the LE had functional roles molecularly linked to epithelial cell crypt formation, such as actin cytoskeleton remodeling, cell polarization, and cell migration. Images of proteins localizing to the S had functional roles molecularly linked to remodeling of the extracellular matrix, a primary component of the S. In addition to visualizing tissue-type-specific expression with our protein images, we were also able to visualize region-specific bioactivity. We have recently shown that prostaglandins, including prostaglandin E2, localize to the LE in uterine sections obtained from the same *Wnt5a*-null mouse used in this study^32^. Our protein images were able to visualize the metabolism of arachidonic acid into bioactive lipid mediators where prostaglandin G/H synthase 1 and prostaglandin E synthase 2 localized to the LE. In addition, our protein images were able to characterize unique tissue microenvironments within the same cell populations by visualizing the gradient expression increase of stroma proteins along the mesometrial (top)–antimesometrial (bottom) axis (Fig. 5).

The in-depth proteome-mapping results from the innovative nanoPOTS imaging platform clearly demonstrate the exciting potential of spatially resolved proteomics to provide previously unobtainable insights into tissue proteomes. Further, the utilization of Trelliscope for data visualization makes these powerful datasets quickly and easily accessible to the broader research community, greatly increasing their impact.

## Methods

### Mouse liver tissue

All mice used in this study were housed at PNNL according to NIH and institutional guidelines for the use of laboratory animals. All protocols for this study were reviewed and approved by the Institutional Animal Care and Use Committee of Battelle, Pacific Northwest Division. C57BL/6J mice were obtained from Jackson Labs and tissues were prepared for LCM as previously described^8^. Briefly, harvested mouse livers were washed with phosphate-buffered saline (PBS) before being snap frozen and stored at –80 °C until analysis. Samples were placed in the cryostat (NX-70; Thermo Fisher Scientific) and allowed to warm to cutting temperature, approximately –15 °C. At that point, a lobe was separated from the rest of the liver with a sterile scalpel and mounted with water onto a chuck. Sections were obtained at 10 µm, thaw-mounted onto PEN membrane slides (Carl Zeiss Microscopy, Germany), and stored at −80 °C until use.

### Mouse uterine tissue

All mice used in this study were housed in the Cincinnati Children’s Animal Care Facility according to NIH and institutional guidelines for the use of laboratory animals. All protocols for this study were reviewed and approved by the Cincinnati Children’s Research Foundation Institutional Animal Care and Use Committee. Utilizing the LoxP-Cre system, mice with uterine-specific inactivation of *Wnt5a*^*d/d*^ (*Wnt5a*^*loxP/loxP*^) were generated as previously described^46^. Uterine tissues were collected on day 4 of pregnancy. This transgenic mouse model of impaired embryo implantation contains cellular and molecular changes in the uterus, including disrupted luminal epithelial evaginations (crypts) at the antimesometrial and mesometrial domains^46^. These crypts are an essential step in the receptive uterus prior to embryo attachment; in wild-type mice these luminal epithelial projections localize only to the antimesometrial pole. Wnt5*a*^*d/d*^ mice were chosen for these foundational nanoPOTS proteomic imaging experiments because of these exaggerated morphological changes. Uterine tissue from one *Wnt5a*^*d/d*^ mouse was sectioned with a thickness of 12 µm using a cryostat (NX-70). The temperatures of chuck and blade were set at −16 and −20 °C for liver tissues and −16 and −20 °C for uterus tissues. The tissue sections were deposited on Zeiss PEN membrane slides and stored at −80 °C.

Tissue fixative solution (70% ethanol) was precooled to 4 °C before use. Tissue sections were immediately immersed into 70% ethanol for 15 s after removal from the −80 °C freezer or dry-ice box. Rehydration was performed for 30 s in deionized water. Next, the tissue sections were immersed in Mayer’s hematoxylin solution (Sigma-Aldrich, St. Louis, USA) for 1 min, dipped twice in deionized water to remove excess dye solution, and immersed in Scott’s Tap Water Substitute (Sigma-Aldrich) for 15 s to dye the tissues. Finally, tissue dehydration was performed by sequentially immersing the tissue sections in 70% ethanol for 1 min, 95% ethanol for 1 min, 100% ethanol for 1 min, and xylene for 2 min. The sections were dried in a fume hood for 10 min; subsequently fixed tissue sections can be directly used or stored at −80 °C for future use.

### Nanowell chip fabrication

Nanowell chips were fabricated on glass slides with precoated chromium and photoresist layers (Telic company, Valencia, USA) using standard photolithography and wet chemical-etching procedures. An array of 3 × 9 nanowells with a diameter of 1.2 mm and a center-to-center spacing of 4.5 mm was designed in AutoCAD and printed with a Direct-Write Lithography System (SF-100; Intelligent Micro Patterning LLC, St. Petersburg, USA). After exposure, development, and chromium etching, the slides were etched in a solution of 2:4:4 (v:v:v) buffered hydrofluoric acid, hydrochloric acid, and water at an etch rate of 1 µm/min for 10 min. After drying at 120 °C for 2 h, the slides were treated with 2% (v/v) heptadecafluoro-1,1,2,2-tetrahydrodecyldimethylchlorosilane in 2,2,4-trimethylpentane. After removing the remaining chromium layer, an array of hydrophilic spots was formed on a hydrophobic background. A glass frame (machined by Coherent Inc., Santa Clara, CA) with a thickness of 1 mm and a width of 5 mm was affixed to the nanowell slide using silicone adhesive. Finally, a sealing cover plate was fabricated by spin-coating a layer of polydimethylsiloxane (30 µm in thickness). The sealing cover slide was used to reversibly seal the nanowell chip during reaction incubation.

### Laser capture microdissection of tissue sections

Before experiments, nanowells were prepopulated with 200-nL DMSO droplets that served as a low-vapor-pressure capture medium. Laser capture microdissection (LCM) was performed on a PALM MicroBeam system (Carl Zeiss MicroImaging, Munich, Germany). A slide adapter (SlideCollector 48, Carl Zeiss MicroImaging) was used to mount a nanowell chip on the LCM microscope. Voxelation of the tissue section was achieved by first drawing a grid on the tissue using PalmRobo software, followed by tissue cutting and catapulting. Both liver and uterine tissues were cut at an energy level of 42 and with an iteration cycle of 2 to completely separate 100 × 100 µm tissue voxels. The “CenterRoboLPC” function with an energy level of delta 10 and a focus level of delta 5 was used to catapult tissue voxels into DMSO droplets. The “CapCheck” function was activated to confirm successful sample collection from tissue sections to DMSO droplets. The collected samples can be processed directly or stored at −20 °C for weeks until use.

### Proteomic sample processing

The nanowell chip was heated to 70 °C for 10 min to evaporate the DMSO droplet. A nanoliter-resolution robotic liquid-handling platform was employed to dispense reagents into nanowells. First, a cell lysis buffer containing 0.2% (w/v) n-dodecyl-β-d-maltoside (DDM; Sigma-Aldrich), 5 mM dithiothreitol (DTT), and 1× PBS was applied into each nanowell with a volume of 100 nL. The chip was incubated at 70 °C for 1 h for cell lysis, protein extraction, and denaturation. Next, 50 nL of 30 mM iodoacetamide in 50 mM ammonium bicarbonate (ABC) buffer (pH 8.0) was added to each well and incubated in the dark for 30 min. Protein digestion was performed by dispensing 50 nL of 0.01 ng/nL Lys-C (MS grade, Promega, Madison, USA) and trypsin (Promega) in ABC buffer, and incubated for 4 and 8 h, respectively. Finally, the enzymatic reaction was terminated by adding 50 nL of 0.5% trifluoroacetic acid in aqueous buffer and incubated for 30 min.

The processed samples were transferred into 96-well PCR plates (twin.tec PCR Plates; Eppendorf, Hauppauge, USA) for LC-MS analysis. The 96-well plate was prefilled with 25 µL of 0.1% FA and 0.02% DDM aqueous buffer. The robotic platform was used to aspirate nanoliter samples from nanowells and dispense into the 25-µL buffer. Each nanowell was washed twice with 200 nL of the same buffer to maximize sample recovery. Finally, the 96-well plates were sealed with sealing tape (Nunc; Thermo Scientific) and stored at −20 °C.

### Sample analysis with SPE-LC-MS/MS

A homebuilt LC system was employed to automatically perform sample injection, sample cleanup, and LC separation. The platform consisted of a PAL autosampler (CTC Analytics AG, Zwingen, Switzerland), two Cheminert six-port injection valves (Valco Instruments, Houston, USA), a binary nanoUPLC pump (Dionex UltiMate NCP-3200RS; Thermo Scientific), and a HPLC sample loading pump (1200 Series; Agilent, Santa Clara, USA). Both SPE precolumn (150 µm i.d., 4-cm length) and LC column (50 µm i.d., 70-cm Self-Pack PicoFrit column, New Objective, Woburn, USA) were slurry-packed with Jupiter C18 packing material (300-Å pore size, trapping column 5 µm, and analytical column 3-µm particle sizes; Phenomenex, Terrence, USA). The sample was injected into a 20-µL loop and loaded onto the trapping column using Buffer A (0.1% formic acid in water) at 3 µL/min for 20 min. After trapping, the sample was reverse-flow eluted onto the analytical column at 150 nL/min and separated by a gradient of 5–8% (0–2 min), 8–12% (2–20 min), 12–35% (20–75 min), and 35–60% (75–97 min) of Buffer B (0.1% formic acid in acetonitrile). The LC column was then washed using 75% Buffer B for 10 min and re-equilibrated using 5% Buffer B for 50 min.

A QExactive Plus Orbitrap MS (Thermo Scientific) was used to analyze the separated peptides. A 2.2-kV high voltage was applied at the ionization source to generate electrospray and ionize peptides. The ion transfer capillary was heated to 250 °C to desolvate droplets. The S-lens RF level was set at 70. Data-dependent mode was employed to automatically trigger precursor scan and MS/MS scans. Precursors were scanned at a resolution of 35,000, an AGC target of 3E6, a maximum ion trap time of 50 ms, and mass range of 375–1800. Top-12 precursors were isolated with an isolation window of 2, an AGC target of 1E5, and a maximum ion trap time of 150 ms, and then fragmented by high-energy collision with an energy level of 32%. A dynamic exclusion of 30 s was used to minimize repeated sequencing. MS/MS spectra were scanned at a resolution of 17,500.

### Data analysis

All raw files were processed using MaxQuant^47,48^ (version 1.5.3.30) for feature detection, database searching, and protein/peptide quantification. Mass spectra were searched against the Uniprot *Mus Musculus* database downloaded in October 2016, containing 16,825 sequence entries. Carbamidomethylation of cysteine was set as a fixed modification and N-terminal acetylation and oxidation of methionine were allowed as variable modifications. A peptide length > 6 was required with a maximum of two missed cleavages allowed, and a false discovery rate of 0.01. The searches were completed twice with these settings, first with the match-between-runs (MBR) feature enabled and then without, for comparison. Contaminants and reverse sequences were removed from the peptides.txt file prior to use for downstream statistical analysis and image display.

### Statistical analysis

In the quantified data, values that were not observed were indicated by NA and data were then log2 transformed. Peptides not observed in at least two samples across all instrument runs within a study were removed. Biological outliers were identified via RMD-PAV^37^ (*p* value threshold 0.001) and Pearson correlation. Data were normalized by median centering based on rank-invariant peptides^38^, where rank invariance was determined by a *p* value threshold of 0.2. Protein quantification was performed using R-rollup^49^, which scaled the peptides associated with each protein by a reference peptide (the peptide with the least missing data) and then set the median of the scaled peptides as the protein abundance. Pairwise-univariate statistical comparisons were carried out between each of the three cell types using a Tukey-adjusted ANOVA or a Holm-adjusted *g* test to compare each pair of dominant cell types^50^.

### Gene Ontology Enrichment Analysis

Uniprot IDs were converted to gene symbols using the ID conversion service at www.uniprot.org. Using the R statistical programming environment, lists of proteins identified as differentially abundant in each experiment were subjected to the EASE-adjusted Fisher exact test^51^ using the complete human GO categories available at [http://geneontology.org/].

### Reporting summary

Further information on research design is available in the Nature Research Reporting Summary linked to this article.

## Supplementary information

Supplementary Information

Peer Review File

Reporting Summary

Description of Additional Supplementary Files

Supplementary Data 1

Supplementary Data 2

Supplementary Movie 1

## Data Availability

The data supporting this study are available from the corresponding authors upon reasonable request. Mass Spectrometry Interactive Virtual Environment (MassIVE; [https://massive.ucsd.edu]) accession number MSV000084421 contains raw proteomics datasets and their corresponding MaxQuant searching results. Visualization of these large datasets is possible through our searchable Trelliscope software platform ([http://msc-viz.emsl.pnnl.gov/nanoPOTS_PI_MS/]).
