# Supplementary material for: Automated mass spectrometry imaging of over 2000 proteins from tissue sections at 100-μm spatial resolution
Source: Nat Commun. 2020 Jan 7;11:8. doi: 10.1038/s41467-019-13858-z (PMC6946663; doi:10.1038/s41467-019-13858-z)
Supplement: Supplementary file 1 — Supplementary Information [file 41467_2019_13858_MOESM1_ESM.pdf]

Supplementary Information

Automated mass spectrometry imaging of over 2000 proteins from tissue sections at 100-µm spatial resolution

Paul D. Piehowski[1]*, Ying Zhu[2]*, Lisa M. Bramer[3], Kelly G. Stratton[3], Rui Zhao[1], Daniel J. Orton[1], Ronald J. Moore[1], Jia Yuan[4], Hugh D. Mitchell[1], Yuqian Gao[1], Bobbie-Jo M. Webb-Robertson[3], Sudhansu. K. Dey[4], Ryan T. Kelly[2,5,‡], Kristin E. Burnum-Johnson[1,‡]

[1]Biological Sciences Division, [2]The Environmental Molecular Sciences Laboratory, [3]National Security Directorate, Pacific Northwest National Laboratory, Richland, WA
[4]Cincinnati Children's Hospital, Cincinnati, OH
[5]Department of Chemistry and Biochemistry, Brigham Young University, Provo, UT
**These authors contributed equally to this work*
‡Corresponding authors

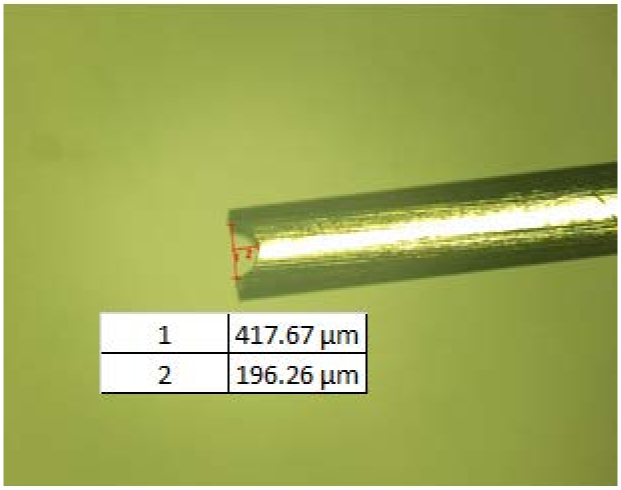

Supplementary Figure 1. Pacific Northwest National Laboratory’s improved high sample recovery custom LC system is equipped with the pictured zero-dead-volume injection needle.

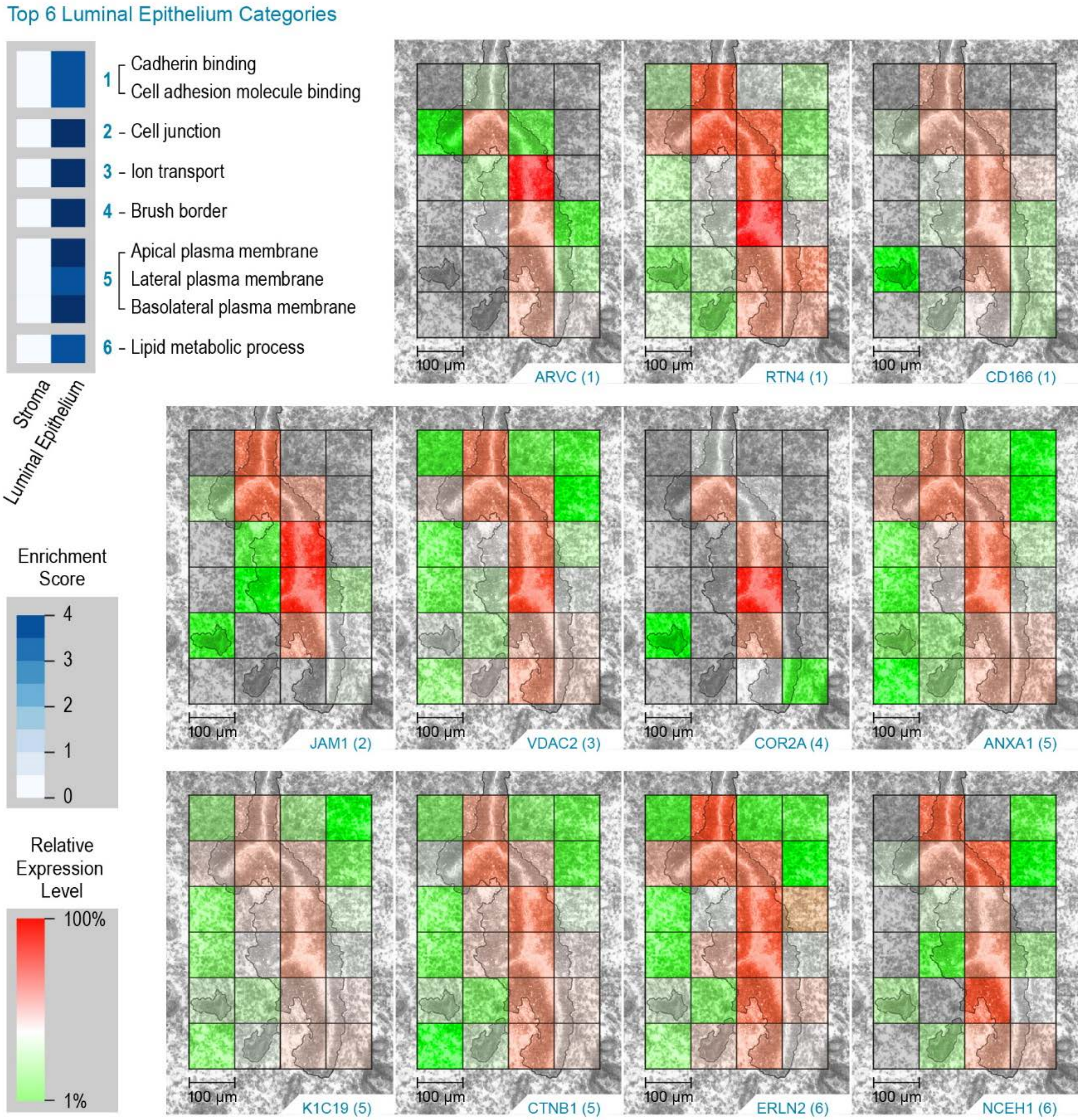

Supplementary Figure 2. The top 6 Luminal Epithelium (LE) Gene Ontology categories (top, left) enriched in the statistically significant (Tukey-adjusted ANOVA or a Holm-adjusted g-test, adjusted p-value <0.05) proteins from the dominant cell population study and corresponding protein images. (1) armadillo repeat protein deleted in velo-cardio-facial syndrome homolog (ARVC), reticulon-4 (RTN4), CD166 antigen (CD166); (2) junctional adhesion molecule A (JAM1); (3) voltage-dependent anion-selective channel protein 2 (VDAC2); (4) coronin-2A (COR2A); (5) annexin A1 (ANXA1), keratin type I cytoskeletal 19 (K1C19), catenin beta-1 (CTNB1); (6) erlin-2 (ERLN2), neutral cholesterol ester hydrolase 1 (NCEH1). Scale bars, 100 µm. The authors would like to thank PNNL Graphic Designer Nathan Johnson for preparing the figure.

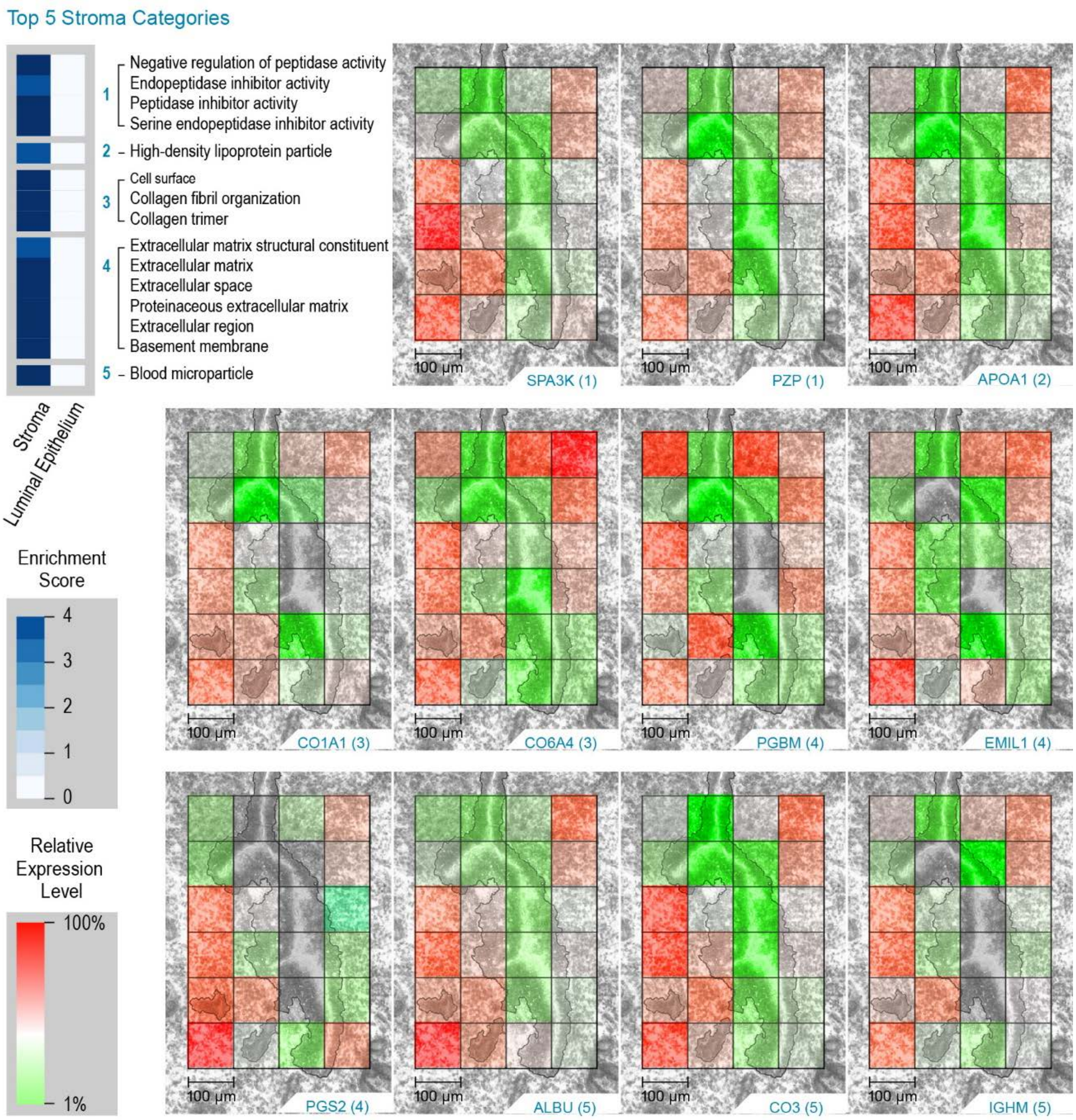

Supplementary Figure 3. The top 5 Stroma (S) Gene Ontology categories (top, left) enriched in the statistically significant (Tukey-adjusted ANOVA or a Holm-adjusted g-test, adjusted p-value <0.05) proteins from the dominant cell population study and corresponding protein images. (1) serine protease inhibitor A3K (SPA3K), pregnancy zone protein (PZP); (2) apolipoprotein A-I (APOA1); (3) collagen alpha-1(I) chain (CO1A1), collagen alpha-4(VI) chain (CO6A4); (4) basement membrane-specific heparan sulfate proteoglycan core protein (PGBM), EMILIN-1 (EMIL1), Decorin (PGS2); (5) serum albumin (ALBU), complement C3 (CO3), immunoglobulin heavy constant mu (IGHM). Scale bars, 100 µm. The authors would like to thank PNNL Graphic Designer Nathan Johnson for preparing the figure.

Supplementary Table 1. 149 Proteins significantly increased in the Luminal Epithelium (LE) and/or Glandular Epithelium (GE) vs. Stroma (S)

Gene Ontology terms From **Figure 4**

	Cell type with significantly increased expression	Uniprot link	Uniprot Accession Number	Uniprot Entry Name	Uniprot Entry Name Extended	GeneName	Protein Name	cadherin binding	cell adhesion	cell adhesion molecule binding	cell junction	ion transport	brush border	apical plasma membrane	lateral plasma membrane	basolateral plasma membrane	lipid metabolic process
1	Up in Luminal Epithelium & Glandular Epithelium compared to Stroma	https://www.uniprot.org/uniprot/P48193	P48193	41_MOUSE	sp|P48193|41_MOUSE	Epb41	erythrocyte protein band 4.1				X						
2	Up in Luminal Epithelium compared to Stroma	https://www.uniprot.org/uniprot/Q5SWU9	Q5SWU9	ACACA_MOUSE	sp|Q5SWU9|ACACA_MOUSE	Acaca	acetyl-Coenzyme A carboxylase alpha										X
3	Up in Luminal Epithelium compared to Stroma	https://www.uniprot.org/uniprot/Q9QUJ7	Q9QUJ7	ACSL4_MOUSE	sp|Q9QUJ7|ACSL4_MOUSE	Acsl4	acyl-CoA synthetase long-chain family member 4										X
4	Up in Luminal Epithelium compared to Stroma	https://www.uniprot.org/uniprot/Q9QZQ1	Q9QZQ1	AFAD_MOUSE	sp|Q9QZQ1|AFAD_MOUSE	Afdn	Afadin	X	X	X	X						
5	Up in Luminal Epithelium & Glandular Epithelium compared to Stroma	https://www.uniprot.org/uniprot/P97449	P97449	AMPN_MOUSE	sp|P97449|AMPN_MOUSE	Anpep	alanyl (membrane) aminopeptidase										
6	Up in Luminal Epithelium compared to Stroma	https://www.uniprot.org/uniprot/P55302	P55302	AMRP_MOUSE	sp|P55302|AMRP_MOUSE	Lrpap1	low density lipoprotein receptor-related protein associated protein 1										
7	Up in Luminal Epithelium compared to Stroma	https://www.uniprot.org/uniprot/Q9JHZ2	Q9JHZ2	ANKH_MOUSE	sp|Q9JHZ2|ANKH_MOUSE	Ank	progressive ankylosis										
8	Up in Luminal Epithelium compared to Stroma	https://www.uniprot.org/uniprot/P10107	P10107	ANXA1_MOUSE	sp|P10107|ANXA1_MOUSE	Anxa1	annexin A1	X	X	X				X	X		
9	Up in Luminal Epithelium & Glandular Epithelium compared to Stroma	https://www.uniprot.org/uniprot/P97429	P97429	ANXA4_MOUSE	sp|P97429|ANXA4_MOUSE	Anxa4	annexin A4							X			
10	Up in Luminal Epithelium compared to Stroma	https://www.uniprot.org/uniprot/P14824	P14824	ANXA6_MOUSE	sp|P14824|ANXA6_MOUSE	Anxa6	annexin A6							X			
11	Up in Luminal Epithelium compared to Stroma	https://www.uniprot.org/uniprot/P22892	P22892	AP1G1_MOUSE	sp|P22892|AP1G1_MOUSE	Ap1g1	adaptor protein complex AP-1, gamma 1 subunit										
12	Up in Luminal Epithelium compared to Stroma	https://www.uniprot.org/uniprot/Q9WVP1	Q9WVP1	AP1M2_MOUSE	sp|Q9WVP1|AP1M2_MOUSE	Ap1m2	adaptor protein complex AP-1, mu 2 subunit										
13	Up in Luminal Epithelium compared to Stroma	https://www.uniprot.org/uniprot/Q8VBT6	Q8VBT6	APOBR_MOUSE	sp|Q8VBT6|APOBR_MOUSE	Apob48r	apolipoprotein B48 receptor										X
14	Up in Luminal Epithelium compared to Stroma	https://www.uniprot.org/uniprot/Q9D0L7	Q9D0L7	ARM10_MOUSE	sp|Q9D0L7|ARM10_MOUSE	Armc10	armadillo repeat containing 10										
15	Up in Luminal Epithelium compared to Stroma	https://www.uniprot.org/uniprot/P98203	P98203	ARVC_MOUSE	sp|P98203|ARVC_MOUSE	Arvcf	Armadillo repeat protein deleted in velo-cardio-facial syndrome homolog		X	X							
16	Up in Luminal Epithelium & Glandular Epithelium compared to Stroma	https://www.uniprot.org/uniprot/Q8VDN2	Q8VDN2	AT1A1_MOUSE	sp|Q8VDN2|AT1A1_MOUSE	Atp1a1	Sodium/potassium-transporting ATPase subunit alpha-1							X	X	X	
17	Up in Luminal Epithelium & Glandular Epithelium compared to Stroma	https://www.uniprot.org/uniprot/P14094	P14094	AT1B1_MOUSE	sp|P14094|AT1B1_MOUSE	Atp1b1	Sodium/potassium-transporting ATPase subunit beta-1							X	X	X	
18	Up in Luminal Epithelium compared to Stroma	https://www.uniprot.org/uniprot/O55143	O55143	AT2A2_MOUSE	sp|O55143|AT2A2_MOUSE	Atp2a2	ATPase, Ca++ transporting, cardiac muscle, slow twitch 2					X					
19	Up in Luminal Epithelium compared to Stroma	https://www.uniprot.org/uniprot/Q91V12	Q91V12	BACH_MOUSE	sp|Q91V12|BACH_MOUSE	Acot7	acyl-CoA thioesterase 7										
20	Up in Luminal Epithelium compared to Stroma	https://www.uniprot.org/uniprot/O08734	O08734	BAK_MOUSE	sp|O08734|BAK_MOUSE	Bak1	BCL2-antagonist/killer 1										
21	Up in Luminal Epithelium compared to Stroma	https://www.uniprot.org/uniprot/Q61335	Q61335	BAP31_MOUSE	sp|Q61335|BAP31_MOUSE	Bcap31	B-cell receptor-associated protein 31										
22	Up in Luminal Epithelium compared to Stroma	https://www.uniprot.org/uniprot/P09803	P09803	CADH1_MOUSE	sp|P09803|CADH1_MOUSE	Cdh1	cadherin 1	X	X	X	X				X	X	
23	Up in Luminal Epithelium compared to Stroma	https://www.uniprot.org/uniprot/O35350	O35350	CAN1_MOUSE	sp|O35350|CAN1_MOUSE	Capn1	calpain 1										
24	Up in Luminal Epithelium compared to Stroma	https://www.uniprot.org/uniprot/P18242	P18242	CATD_MOUSE	sp|P18242|CATD_MOUSE	Ctsd	cathepsin D										
25	Up in Luminal Epithelium & Glandular Epithelium compared to Stroma	https://www.uniprot.org/uniprot/P08074	P08074	CBR2_MOUSE	sp|P08074|CBR2_MOUSE	Cbr2	carbonyl reductase 2										
26	Up in Luminal Epithelium compared to Stroma	https://www.uniprot.org/uniprot/Q61490	Q61490	CD166_MOUSE	sp|Q61490|CD166_MOUSE	Alcam	CD166 antigen		X	X							
27	Up in Luminal Epithelium & Glandular Epithelium compared to Stroma	https://www.uniprot.org/uniprot/Q8BJ64	Q8BJ64	CHDH_MOUSE	sp|Q8BJ64|CHDH_MOUSE	Chdh	choline dehydrogenase										
28	Up in Luminal Epithelium compared to Stroma	https://www.uniprot.org/uniprot/P61022	P61022	CHP1_MOUSE	sp|P61022|CHP1_MOUSE	Chp1	Calcineurin B homologous protein 1										
29	Up in Luminal Epithelium compared to Stroma	https://www.uniprot.org/uniprot/Q9CZU6	Q9CZU6	CISY_MOUSE	sp|Q9CZU6|CISY_MOUSE	Cs	citrate synthase										
30	Up in Luminal Epithelium & Glandular Epithelium compared to Stroma	https://www.uniprot.org/uniprot/Q9Z0G9	Q9Z0G9	CLD3_MOUSE	sp|Q9Z0G9|CLD3_MOUSE	Cldn3	claudin 3				X				X		
31	Up in Luminal Epithelium compared to Stroma	https://www.uniprot.org/uniprot/Q9QXX4	Q9QXX4	CMC2_MOUSE	sp|Q9QXX4|CMC2_MOUSE	Slc25a13	solute carrier family 25 (mitochondrial carrier, adenine nucleotide translocator), member 13										
32	Up in Luminal Epithelium & Glandular Epithelium compared to Stroma	https://www.uniprot.org/uniprot/Q8C0P5	Q8C0P5	COR2A_MOUSE	sp|Q8C0P5|COR2A_MOUSE	Coro2a	Coronin-2A						X				
33	Up in Luminal Epithelium compared to Stroma	https://www.uniprot.org/uniprot/P56546	P56546	CTBP2_MOUSE	sp|P56546|CTBP2_MOUSE	Ctbp2	C-terminal binding protein 2										
34	Up in Luminal Epithelium compared to Stroma	https://www.uniprot.org/uniprot/P26231	P26231	CTNA1_MOUSE	sp|P26231|CTNA1_MOUSE	Ctnna1	Catenin alpha-1	X	X	X	X						
35	Up in Luminal Epithelium compared to Stroma	https://www.uniprot.org/uniprot/Q61301	Q61301	CTNA2_MOUSE	sp|Q61301|CTNA2_MOUSE	Ctnna2	Catenin alpha-2	X	X	X					X	X	
36	Up in Luminal Epithelium compared to Stroma	https://www.uniprot.org/uniprot/Q02248	Q02248	CTNB1_MOUSE	sp|Q02248|CTNB1_MOUSE	Ctnnb1	Catenin beta-1	X	X	X	X				X	X	
37	Up in Luminal Epithelium compared to Stroma	https://www.uniprot.org/uniprot/P30999	P30999	CTND1_MOUSE	sp|P30999|CTND1_MOUSE	Ctnnd1	Catenin delta-1	X	X	X	X						
38	Up in Luminal Epithelium & Glandular Epithelium compared to Stroma	https://www.uniprot.org/uniprot/Q9CQX2	Q9CQX2	CYB5B_MOUSE	sp|Q9CQX2|CYB5B_MOUSE	Cyb5b	cytochrome b5 type B										
39	Up in Luminal Epithelium & Glandular Epithelium compared to Stroma	https://www.uniprot.org/uniprot/E9Q557	E9Q557	DESP_MOUSE	sp|E9Q557|DESP_MOUSE	Dsp	desmoplakin		X	X	X				X	X	
40	Up in Luminal Epithelium compared to Stroma	https://www.uniprot.org/uniprot/O70503	O70503	DHB12_MOUSE	sp|O70503|DHB12_MOUSE	Hsd17b12	hydroxysteroid (17-beta) dehydrogenase 12										X
41	Up in Luminal Epithelium & Glandular Epithelium compared to Stroma	https://www.uniprot.org/uniprot/Q99LB2	Q99LB2	DHRS4_MOUSE	sp|Q99LB2|DHRS4_MOUSE	Dhrs4	dehydrogenase/reductase (SDR family) member 4										
42	Up in Luminal Epithelium compared to Stroma	https://www.uniprot.org/uniprot/Q9QZD8	Q9QZD8	DIC_MOUSE	sp|Q9QZD8|DIC_MOUSE	Slc25a10	solute carrier family 25 (mitochondrial carrier, dicarboxylate transporter), member 10										
43	Up in Luminal Epithelium & Glandular Epithelium compared to Stroma	https://www.uniprot.org/uniprot/P28843	P28843	DPP4_MOUSE	sp|P28843|DPP4_MOUSE	Dpp4	dipeptidylpeptidase 4				X			X			
44	Up in Luminal Epithelium & Glandular Epithelium compared to Stroma	https://www.uniprot.org/uniprot/O55111	O55111	DSG2_MOUSE	sp|O55111|DSG2_MOUSE	Dsg2	desmoglein 2		X	X	X			X	X		
45	Up in Luminal Epithelium compared to Stroma	https://www.uniprot.org/uniprot/Q8BH95	Q8BH95	ECHM_MOUSE	sp|Q8BH95|ECHM_MOUSE	Echs1	enoyl Coenzyme A hydratase, short chain, 1, mitochondrial										X
46	Up in Luminal Epithelium compared to Stroma	https://www.uniprot.org/uniprot/Q8BHI7	Q8BHI7	ELOV5_MOUSE	sp|Q8BHI7|ELOV5_MOUSE	Elovl5	Elongation of very long chain fatty acids protein 5										X
47	Up in Luminal Epithelium compared to Stroma	https://www.uniprot.org/uniprot/Q3UMY5	Q3UMY5	EMAL4_MOUSE	sp|Q3UMY5|EMAL4_MOUSE	Eml4	echinoderm microtubule associated protein like 4										
48	Up in Luminal Epithelium compared to Stroma	https://www.uniprot.org/uniprot/Q8C522	Q8C522	ENDD1_MOUSE	sp|Q8C522|ENDD1_MOUSE	Endod1	endonuclease domain containing 1										
49	Up in Luminal Epithelium compared to Stroma	https://www.uniprot.org/uniprot/P08113	P08113	ENPL_MOUSE	sp|P08113|ENPL_MOUSE	Hsp90b1	heat shock protein 90, beta (Grp94), member 1										
50	Up in Luminal Epithelium compared to Stroma	https://www.uniprot.org/uniprot/Q6DYE8	Q6DYE8	ENPP3_MOUSE	sp|Q6DYE8|ENPP3_MOUSE	Enpp3	ectonucleotide pyrophosphatase/phosphodiesterase 3										
51	Up in Luminal Epithelium & Glandular Epithelium compared to Stroma	https://www.uniprot.org/uniprot/Q99JW5	Q99JW5	EPCAM_MOUSE	sp|Q99JW5|EPCAM_MOUSE	Epcam	epithelial cell adhesion molecule				X			X	X	X	
52	Up in Luminal Epithelium compared to Stroma	https://www.uniprot.org/uniprot/Q8BFZ9	Q8BFZ9	ERLN2_MOUSE	sp|Q8BFZ9|ERLN2_MOUSE	Erlin2	Erlin-2										X
53	Up in Luminal Epithelium compared to Stroma	https://www.uniprot.org/uniprot/P26040	P26040	EZRI_MOUSE	sp|P26040|EZRI_MOUSE	Ezr	ezrin		X	X			X	X	X	X	
54	Up in Luminal Epithelium & Glandular Epithelium compared to Stroma	https://www.uniprot.org/uniprot/Q9CYH2	Q9CYH2	F213A_MOUSE	sp|Q9CYH2|F213A_MOUSE	Fam213a	Redox-regulatory protein FAM213A										
55	Up in Luminal Epithelium compared to Stroma	https://www.uniprot.org/uniprot/Q8BYI8	Q8BYI8	F234B_MOUSE	sp|Q8BYI8|F234B_MOUSE	Fam234b	Protein FAM234B										
56	Up in Luminal Epithelium & Glandular Epithelium compared to Stroma	https://www.uniprot.org/uniprot/Q80W54	Q80W54	FACE1_MOUSE	sp|Q80W54|FACE1_MOUSE	Zmpste24	CAAX prenyl protease 1 homolog										
57	Up in Luminal Epithelium compared to Stroma	https://www.uniprot.org/uniprot/Q922J9	Q922J9	FACR1_MOUSE	sp|Q922J9|FACR1_MOUSE	Far1	fatty acyl CoA reductase 1										
58	Up in Luminal Epithelium & Glandular Epithelium compared to Stroma	https://www.uniprot.org/uniprot/Q91VU0	Q91VU0	FAM3C_MOUSE	sp|Q91VU0|FAM3C_MOUSE	Fam3c	family with sequence similarity 3, member C										
59	Up in Luminal Epithelium compared to Stroma	https://www.uniprot.org/uniprot/Q80X90	Q80X90	FLNB_MOUSE	sp|Q80X90|FLNB_MOUSE	Flnb	filamin, beta	X	X	X							
60	Up in Luminal Epithelium & Glandular Epithelium compared to Stroma	https://www.uniprot.org/uniprot/Q0VAX3	Q0VAX3	FS2P1_MOUSE	sp|Q0VAX3|FS2P1_MOUSE	Fads2p1	Fatty acid desaturase 2-like protein FADS2P1										
61	Up in Luminal Epithelium compared to Stroma	https://www.uniprot.org/uniprot/O08912	O08912	GALT1_MOUSE	sp|O08912|GALT1_MOUSE	Galnt1	UDP-N-acetyl-alpha-D-galactosamine:polypeptide N-acetylgalactosaminyltransferase 1										
62	Up in Luminal Epithelium & Glandular Epithelium compared to Stroma	https://www.uniprot.org/uniprot/P15105	P15105	GLNA_MOUSE	sp|P15105|GLNA_MOUSE	Glul	glutamate-ammonia ligase (glutamine synthetase)										
63	Up in Luminal Epithelium & Glandular Epithelium compared to Stroma	https://www.uniprot.org/uniprot/Q9QYE6	Q9QYE6	GOGA5_MOUSE	sp|Q9QYE6|GOGA5_MOUSE	Golga5	golgi autoantigen, golgin subfamily a, 5										
64	Up in Luminal Epithelium compared to Stroma	https://www.uniprot.org/uniprot/Q64521	Q64521	GPDM_MOUSE	sp|Q64521|GPDM_MOUSE	Gpd2	glycerol phosphate dehydrogenase 2, mitochondrial										
65	Up in Luminal Epithelium compared to Stroma	https://www.uniprot.org/uniprot/Q6PEM6	Q6PEM6	GRAM3_MOUSE	sp|Q6PEM6|GRAM3_MOUSE	Gramd3	GRAM domain containing 3										
66	Up in Luminal Epithelium compared to Stroma	https://www.uniprot.org/uniprot/Q8K2C9	Q8K2C9	HACD3_MOUSE	sp|Q8K2C9|HACD3_MOUSE	Ptplad1	Very-long-chain (3R)-3-hydroxyacyl-CoA dehydratase 3										X
67	Up in Luminal Epithelium compared to Stroma	https://www.uniprot.org/uniprot/O70252	O70252	HMOX2_MOUSE	sp|O70252|HMOX2_MOUSE	Hmox2	heme oxygenase (decycling) 2										
68	Up in Luminal Epithelium compared to Stroma	https://www.uniprot.org/uniprot/Q61696	Q61696	HS71A_MOUSE	sp|Q61696|HS71A_MOUSE	Hspa1a	heat shock protein 1A	X	X	X				X	X	X	
69	Up in Luminal Epithelium compared to Stroma	https://www.uniprot.org/uniprot/Q2TPA8	Q2TPA8	HSDL2_MOUSE	sp|Q2TPA8|HSDL2_MOUSE	Hsdl2	hydroxysteroid dehydrogenase like 2										
70	Up in Luminal Epithelium compared to Stroma	https://www.uniprot.org/uniprot/O08528	O08528	HXK2_MOUSE	sp|O08528|HXK2_MOUSE	Hk2	hexokinase 2										
71	Up in Luminal Epithelium compared to Stroma	https://www.uniprot.org/uniprot/Q8BU33	Q8BU33	ILVBL_MOUSE	sp|Q8BU33|ILVBL_MOUSE	Ilvbl	Acetolactate synthase-like protein										
72	Up in Luminal Epithelium compared to Stroma	https://www.uniprot.org/uniprot/P97431	P97431	IRF6_MOUSE	sp|P97431|IRF6_MOUSE	Irf6	interferon regulatory factor 6										
73	Up in Luminal Epithelium & Glandular Epithelium compared to Stroma	https://www.uniprot.org/uniprot/P54987	P54987	IRG1_MOUSE	sp|P54987|IRG1_MOUSE	Irg1	immunoresponsive gene 1										
74	Up in Luminal Epithelium & Glandular Epithelium compared to Stroma	https://www.uniprot.org/uniprot/O88792	O88792	JAM1_MOUSE	sp|O88792|JAM1_MOUSE	F11r	FJunctional adhesion molecule A	X	X	X	X						
75	Up in Luminal Epithelium & Glandular Epithelium compared to Stroma	https://www.uniprot.org/uniprot/P05784	P05784	K1C18_MOUSE	sp|P05784|K1C18_MOUSE	Krt18	Keratin, type I cytoskeletal 18										
76	Up in Luminal Epithelium compared to Stroma	https://www.uniprot.org/uniprot/P19001	P19001	K1C19_MOUSE	sp|P19001|K1C19_MOUSE	Krt19	Keratin, type I cytoskeletal 19								X		
77	Up in Luminal Epithelium compared to Stroma	https://www.uniprot.org/uniprot/Q9D312	Q9D312	K1C20_MOUSE	sp|Q9D312|K1C20_MOUSE	Krt20	Keratin, type I cytoskeletal 20										
78	Up in Luminal Epithelium & Glandular Epithelium compared to Stroma	https://www.uniprot.org/uniprot/Q9DCV7	Q9DCV7	K2C7_MOUSE	sp|Q9DCV7|K2C7_MOUSE	Krt7	Keratin, type II cytoskeletal 7										
79	Up in Luminal Epithelium & Glandular Epithelium compared to Stroma	https://www.uniprot.org/uniprot/P11679	P11679	K2C8_MOUSE	sp|P11679|K2C8_MOUSE	Krt8	Keratin, type II cytoskeletal 8								X		
80	Up in Luminal Epithelium compared to Stroma	https://www.uniprot.org/uniprot/P30275	P30275	KCRU_MOUSE	sp|P30275|KCRU_MOUSE	Ckmt1	mitochondrial creatine kinase contact site complex										
81	Up in Luminal Epithelium compared to Stroma	https://www.uniprot.org/uniprot/Q61087	Q61087	LAMB3_MOUSE	sp|Q61087|LAMB3_MOUSE	Lamb3	laminin, beta 3		X	X							
82	Up in Luminal Epithelium compared to Stroma	https://www.uniprot.org/uniprot/P17047	P17047	LAMP2_MOUSE	sp|P17047|LAMP2_MOUSE	Lamp2	lysosomal-associated membrane protein 2										
83	Up in Luminal Epithelium compared to Stroma	https://www.uniprot.org/uniprot/Q9ERG0	Q9ERG0	LIMA1_MOUSE	sp|Q9ERG0|LIMA1_MOUSE	Lima1	LIM domain and actin binding 1	X	X	X							
84	Up in Luminal Epithelium & Glandular Epithelium compared to Stroma	https://www.uniprot.org/uniprot/P39654	P39654	LOX15_MOUSE	sp|P39654|LOX15_MOUSE	Alox15	arachidonate 15-lipoxygenase										
85	Up in Luminal Epithelium & Glandular Epithelium compared to Stroma	https://www.uniprot.org/uniprot/Q9ESE1	Q9ESE1	LRBA_MOUSE	sp|Q9ESE1|LRBA_MOUSE	Lrba	LPS-responsive beige-like anchor										
86	Up in Luminal Epithelium compared to Stroma	https://www.uniprot.org/uniprot/A2ARV4	A2ARV4	LRP2_MOUSE	sp|A2ARV4|LRP2_MOUSE	Lrp2	low density lipoprotein receptor-related protein 2							X			
87	Up in Luminal Epithelium compared to Stroma	https://www.uniprot.org/uniprot/P55249	P55249	LX12E_MOUSE	sp|P55249|LX12E_MOUSE	Alox12e	Arachidonate 12-lipoxygenase, epidermal-type										X
88	Up in Luminal Epithelium compared to Stroma	https://www.uniprot.org/uniprot/Q9CR62	Q9CR62	M2OM_MOUSE	sp|Q9CR62|M2OM_MOUSE	Slc25a11	solute carrier family 25 (mitochondrial carrier oxoglutarate carrier), member 11										
89	Up in Luminal Epithelium compared to Stroma	https://www.uniprot.org/uniprot/Q8BI08	Q8BI08	MAL2_MOUSE	sp|Q8BI08|MAL2_MOUSE	Mal2	mal, T-cell differentiation protein 2										
90	Up in Luminal Epithelium compared to Stroma	https://www.uniprot.org/uniprot/Q99KE1	Q99KE1	MAOM_MOUSE	sp|Q99KE1|MAOM_MOUSE	Me2	malic enzyme 2, NAD(+)-dependent, mitochondrial										
91	Up in Luminal Epithelium compared to Stroma	https://www.uniprot.org/uniprot/Q8BP48	Q8BP48	MAP11_MOUSE	sp|Q8BP48|MAP11_MOUSE	Metap1	methionyl aminopeptidase 1										
92	Up in Luminal Epithelium compared to Stroma	https://www.uniprot.org/uniprot/Q91V01	Q91V01	MBOA5_MOUSE	sp|Q91V01|MBOA5_MOUSE	Lpcat3	lysophosphatidylcholine acyltransferase 3										X
93	Up in Luminal Epithelium compared to Stroma	https://www.uniprot.org/uniprot/Q6ZQI3	Q6ZQI3	MLEC_MOUSE	sp|Q6ZQI3|MLEC_MOUSE	Mlec	Malectin										
94	Up in Luminal Epithelium & Glandular Epithelium compared to Stroma	https://www.uniprot.org/uniprot/Q64331	Q64331	MYO6_MOUSE	sp|Q64331|MYO6_MOUSE	Myo6	myosin VI						X				
95	Up in Luminal Epithelium & Glandular Epithelium compared to Stroma	https://www.uniprot.org/uniprot/Q9DCN2	Q9DCN2	NB5R3_MOUSE	sp|Q9DCN2|NB5R3_MOUSE	Cyb5r3	cytochrome b5 reductase 3										
96	Up in Luminal Epithelium compared to Stroma	https://www.uniprot.org/uniprot/Q8BLF1	Q8BLF1	NCEH1_MOUSE	sp|Q8BLF1|NCEH1_MOUSE	Aadacl1	Neutral cholesterol ester hydrolase 1										X
97	Up in Luminal Epithelium compared to Stroma	https://www.uniprot.org/uniprot/P37040	P37040	NCPR_MOUSE	sp|P37040|NCPR_MOUSE	Por	P450 (cytochrome) oxidoreductase										
98	Up in Luminal Epithelium compared to Stroma	https://www.uniprot.org/uniprot/Q62425	Q62425	NDUA4_MOUSE	sp|Q62425|NDUA4_MOUSE	Ndufa4	NADH-ubiquinone reductase										
99	Up in Luminal Epithelium & Glandular Epithelium compared to Stroma	https://www.uniprot.org/uniprot/Q61391	Q61391	NEP_MOUSE	sp|Q61391|NEP_MOUSE	Mme	membrane metallo endopeptidase						X				
100	Up in Luminal Epithelium compared to Stroma	https://www.uniprot.org/uniprot/P11930	P11930	NUD19_MOUSE	sp|P11930|NUD19_MOUSE	Nudt19	nudix (nucleoside diphosphate linked moiety X)-type motif 19										
101	Up in Luminal Epithelium compared to Stroma	https://www.uniprot.org/uniprot/P47713	P47713	PA24A_MOUSE	sp|P47713|PA24A_MOUSE	Pla2g4a	Cytosolic phospholipase A2										X
102	Up in Luminal Epithelium & Glandular Epithelium compared to Stroma	https://www.uniprot.org/uniprot/Q99JB8	Q99JB8	PACN3_MOUSE	sp|Q99JB8|PACN3_MOUSE	Pacsin3	protein kinase C and casein kinase substrate in neurons 3										
103	Up in Luminal Epithelium compared to Stroma	https://www.uniprot.org/uniprot/Q9Z183	Q9Z183	PADI4_MOUSE	sp|Q9Z183|PADI4_MOUSE	Padi4	peptidyl arginine deiminase, type IV										
104	Up in Luminal Epithelium compared to Stroma	https://www.uniprot.org/uniprot/Q922H2	Q922H2	PDK3_MOUSE	sp|Q922H2|PDK3_MOUSE	Pdk3	pyruvate dehydrogenase kinase, isoenzyme 3										
105	Up in Luminal Epithelium compared to Stroma	https://www.uniprot.org/uniprot/O70400	O70400	PDLI1_MOUSE	sp|O70400|PDLI1_MOUSE	Pdlim1	PDZ and LIM domain 1 (elfin)	X	X	X							
106	Up in Luminal Epithelium compared to Stroma	https://www.uniprot.org/uniprot/Q99K01	Q99K01	PDXD1_MOUSE	sp|Q99K01|PDXD1_MOUSE	Pdxdc1	pyridoxal-dependent decarboxylase domain containing 1	X	X	X							
107	Up in Luminal Epithelium compared to Stroma	https://www.uniprot.org/uniprot/P22437	P22437	PGH1_MOUSE	sp|P22437|PGH1_MOUSE	Ptgs1	Prostaglandin G/H synthase 1										X
108	Up in Luminal Epithelium & Glandular Epithelium compared to Stroma	https://www.uniprot.org/uniprot/Q02257	Q02257	PLAK_MOUSE	sp|Q02257|PLAK_MOUSE	Jup	junction plakoglobin		X	X	X				X		
109	Up in Luminal Epithelium compared to Stroma	https://www.uniprot.org/uniprot/P43883	P43883	PLIN2_MOUSE	sp|P43883|PLIN2_MOUSE	Adfp	adipose differentiation related protein										
110	Up in Luminal Epithelium compared to Stroma	https://www.uniprot.org/uniprot/Q9DCU2	Q9DCU2	PLLP_MOUSE	sp|Q9DCU2|PLLP_MOUSE	Pllp	plasma membrane proteolipid					X					
111	Up in Luminal Epithelium compared to Stroma	https://www.uniprot.org/uniprot/Q9R1Q7	Q9R1Q7	PLP2_MOUSE	sp|Q9R1Q7|PLP2_MOUSE	Plp2	proteolipid protein 2										
112	Up in Luminal Epithelium & Glandular Epithelium compared to Stroma	https://www.uniprot.org/uniprot/Q9JJ00	Q9JJ00	PLS1_MOUSE	sp|Q9JJ00|PLS1_MOUSE	Plscr1	phospholipid scramblase 1										
113	Up in Luminal Epithelium compared to Stroma	https://www.uniprot.org/uniprot/Q9QY81	Q9QY81	PO210_MOUSE	sp|Q9QY81|PO210_MOUSE	Nup210	nucleoporin 210										
114	Up in Luminal Epithelium compared to Stroma	https://www.uniprot.org/uniprot/P20108	P20108	PRDX3_MOUSE	sp|P20108|PRDX3_MOUSE	Prdx3	peroxiredoxin 3										
115	Up in Luminal Epithelium & Glandular Epithelium compared to Stroma	https://www.uniprot.org/uniprot/Q8BHD7	Q8BHD7	PTBP3_MOUSE	sp|Q8BHD7|PTBP3_MOUSE	Ptbp3	Polypyrimidine tract-binding protein 3										
116	Up in Luminal Epithelium compared to Stroma	https://www.uniprot.org/uniprot/Q05920	Q05920	PYC_MOUSE	sp|Q05920|PYC_MOUSE	Pcx	pyruvate carboxylase										
117	Up in Luminal Epithelium compared to Stroma	https://www.uniprot.org/uniprot/Q9CZE3	Q9CZE3	RAB32_MOUSE	sp|Q9CZE3|RAB32_MOUSE	Rab32	RAB32, member RAS oncogene family										
118	Up in Luminal Epithelium compared to Stroma	https://www.uniprot.org/uniprot/P35279	P35279	RAB6A_MOUSE	sp|P35279|RAB6A_MOUSE	Rab6	RAB6, member RAS oncogene family										
119	Up in Luminal Epithelium compared to Stroma	https://www.uniprot.org/uniprot/P51150	P51150	RAB7A_MOUSE	sp|P51150|RAB7A_MOUSE	Rab7	RAB7, member RAS oncogene family										
120	Up in Luminal Epithelium compared to Stroma	https://www.uniprot.org/uniprot/P26043	P26043	RADI_MOUSE	sp|P26043|RADI_MOUSE	Rdx	radixin	X	X	X				X			
121	Up in Luminal Epithelium & Glandular Epithelium compared to Stroma	https://www.uniprot.org/uniprot/Q91WT8	Q91WT8	RBM47_MOUSE	sp|Q91WT8|RBM47_MOUSE	Rbm47	RNA binding motif protein 47										
122	Up in Luminal Epithelium & Glandular Epithelium compared to Stroma	https://www.uniprot.org/uniprot/Q8VCH7	Q8VCH7	RDH10_MOUSE	sp|Q8VCH7|RDH10_MOUSE	Rdh10	retinol dehydrogenase 10 (all-trans)										
123	Up in Luminal Epithelium compared to Stroma	https://www.uniprot.org/uniprot/Q9ES97	Q9ES97	RTN3_MOUSE	sp|Q9ES97|RTN3_MOUSE	Rtn3	reticulon 3										
124	Up in Luminal Epithelium compared to Stroma	https://www.uniprot.org/uniprot/Q99P72	Q99P72	RTN4_MOUSE	sp|Q99P72|RTN4_MOUSE	Rtn4	reticulon 4	X	X	X							
125	Up in Luminal Epithelium compared to Stroma	https://www.uniprot.org/uniprot/P55012	P55012	S12A2_MOUSE	sp|P55012|S12A2_MOUSE	Slc12a2	solute carrier family 12, member 2					X		X	X	X	
126	Up in Luminal Epithelium & Glandular Epithelium compared to Stroma	https://www.uniprot.org/uniprot/Q78IQ7	Q78IQ7	S39A4_MOUSE	sp|Q78IQ7|S39A4_MOUSE	Slc39a4	solute carrier family 39 (zinc transporter), member 4							X			
127	Up in Luminal Epithelium compared to Stroma	https://www.uniprot.org/uniprot/Q9D662	Q9D662	SC23B_MOUSE	sp|Q9D662|SC23B_MOUSE	Sec23b	Protein transport protein Sec23B										
128	Up in Luminal Epithelium & Glandular Epithelium compared to Stroma	https://www.uniprot.org/uniprot/Q9Z2G6	Q9Z2G6	SE1L1_MOUSE	sp|Q9Z2G6|SE1L1_MOUSE	Sel1l	Protein sel-1 homolog 1										
129	Up in Luminal Epithelium & Glandular Epithelium compared to Stroma	https://www.uniprot.org/uniprot/O88307	O88307	SORL_MOUSE	sp|O88307|SORL_MOUSE	Sorl1	sortilin-related receptor, LDLR class A repeats-containing										
130	Up in Luminal Epithelium & Glandular Epithelium compared to Stroma	https://www.uniprot.org/uniprot/Q6PHU5	Q6PHU5	SORT_MOUSE	sp|Q6PHU5|SORT_MOUSE	Sort1	sortilin 1										
131	Up in Luminal Epithelium & Glandular Epithelium compared to Stroma	https://www.uniprot.org/uniprot/Q61473	Q61473	SOX17_MOUSE	sp|Q61473|SOX17_MOUSE	Sox17	SRY-box containing gene 17										
132	Up in Luminal Epithelium compared to Stroma	https://www.uniprot.org/uniprot/P05480	P05480	SRC_MOUSE	sp|P05480|SRC_MOUSE	Src	Rous sarcoma oncogene	X	X	X							
133	Up in Luminal Epithelium compared to Stroma	https://www.uniprot.org/uniprot/P56677	P56677	ST14_MOUSE	sp|P56677|ST14_MOUSE	St14	suppression of tumorigenicity 14 (colon carcinoma)								X	X	
134	Up in Luminal Epithelium compared to Stroma	https://www.uniprot.org/uniprot/P70302	P70302	STIM1_MOUSE	sp|P70302|STIM1_MOUSE	Stim1	stromal interaction molecule 1					X					
135	Up in Luminal Epithelium compared to Stroma	https://www.uniprot.org/uniprot/Q3TBT3	Q3TBT3	STING_MOUSE	sp|Q3TBT3|STING_MOUSE	Tmem173	Stimulator of interferon genes protein										
136	Up in Luminal Epithelium compared to Stroma	https://www.uniprot.org/uniprot/Q9ER00	Q9ER00	STX12_MOUSE	sp|Q9ER00|STX12_MOUSE	Stx12	syntaxin 12										
137	Up in Luminal Epithelium compared to Stroma	https://www.uniprot.org/uniprot/Q9D666	Q9D666	SUN1_MOUSE	sp|Q9D666|SUN1_MOUSE	Sun1	SUN domain-containing protein 1										
138	Up in Luminal Epithelium & Glandular Epithelium compared to Stroma	https://www.uniprot.org/uniprot/Q8BGV3	Q8BGV3	TACD2_MOUSE	sp|Q8BGV3|TACD2_MOUSE	Tacstd2	tumor-associated calcium signal transducer 2								X		
139	Up in Luminal Epithelium compared to Stroma	https://www.uniprot.org/uniprot/P68368	P68368	TBA4A_MOUSE	sp|P68368|TBA4A_MOUSE	Tuba4a	tubulin, alpha 4A										
140	Up in Luminal Epithelium compared to Stroma	https://www.uniprot.org/uniprot/Q921H8	Q921H8	THIKA_MOUSE	sp|Q921H8|THIKA_MOUSE	Acaa1a	acetyl-Coenzyme A acyltransferase 1A										X
141	Up in Luminal Epithelium compared to Stroma	https://www.uniprot.org/uniprot/Q9CQN6	Q9CQN6	TM14C_MOUSE	sp|Q9CQN6|TM14C_MOUSE	Tmem14c	transmembrane protein 14C										
142	Up in Luminal Epithelium & Glandular Epithelium compared to Stroma	https://www.uniprot.org/uniprot/Q99JY4	Q99JY4	TRABD_MOUSE	sp|Q99JY4|TRABD_MOUSE	Trabd	TraB domain containing										
143	Up in Luminal Epithelium & Glandular Epithelium compared to Stroma	https://www.uniprot.org/uniprot/Q8R3G9	Q8R3G9	TSN8_MOUSE	sp|Q8R3G9|TSN8_MOUSE	Tspan8	tetraspanin 8										
144	Up in Luminal Epithelium compared to Stroma	https://www.uniprot.org/uniprot/Q6ZQM8	Q6ZQM8	UD17C_MOUSE	sp|Q6ZQM8|UD17C_MOUSE	Ugt1a7c	UDP glucuronosyltransferase 1 family, polypeptide A7C										
145	Up in Luminal Epithelium compared to Stroma	https://www.uniprot.org/uniprot/P63024	P63024	VAMP3_MOUSE	sp|P63024|VAMP3_MOUSE	Vamp3	vesicle-associated membrane protein 3							X			
146	Up in Luminal Epithelium compared to Stroma	https://www.uniprot.org/uniprot/P57746	P57746	VATD_MOUSE	sp|P57746|VATD_MOUSE	Atp6v1d	vacuolar ATPase					X					
147	Up in Luminal Epithelium compared to Stroma	https://www.uniprot.org/uniprot/Q60930	Q60930	VDAC2_MOUSE	sp|Q60930|VDAC2_MOUSE	Vdac2	Voltage-dependent anion-selective channel protein 2					X					
148	Up in Luminal Epithelium compared to Stroma	https://www.uniprot.org/uniprot/Q8BX70	Q8BX70	VP13C_MOUSE	sp|Q8BX70|VP13C_MOUSE	Vps13c	vacuolar protein sorting 13C (yeast)										
149	Up in Luminal Epithelium compared to Stroma	https://www.uniprot.org/uniprot/Q9Z0U1	Q9Z0U1	ZO2_MOUSE	sp|Q9Z0U1|ZO2_MOUSE	Tjp2	tight junction protein 2	X	X	X	X						

Supplementary Table 2. 177 Proteins significantly increased in the Stroma (S) and/or Glandular Epithelium (GE) vs. Luminal Epithelium (LE)

Note: 175 proteins are enriched in the S compared to LE (27 of these proteins are also enriched in the GE compared to LE); 2 proteins are only enriched in the GE compared to LE

Gene Ontology (GO) terms from **Figure 5**

	Cell type with significantly increased expression	Uniprot link	Uniprot Accession Number	Uniprot Entry Name	Uniprot Entry Name Extended	GeneName	Protein Name	negative regulation of peptidase activity	endopeptidase inhibitor activity	peptidase inhibitor activity	serine-type endopeptidase inhibitor activity	high-density lipoprotein particle	cell surface	collagen fibril organization	collagen trimer	extracellular matrix structural constituent	extracellular matrix	extracellular space	proteinaceous extracellular matrix	extracellular region	basement membrane	blood microparticle
1	Up in Stroma compared to Luminal Epithelium	https://www.uniprot.org/uniprot/P22599	P22599	A1AT2_MOUSE	sp|P22599|A1AT2_MOUSE	Serpina1b	serine (or cysteine) preptidase inhibitor, clade A, member 1B	X	X	X	X	X	X					X		X		
2	Up in Stroma compared to Luminal Epithelium	https://www.uniprot.org/uniprot/Q00896	Q00896	A1AT3_MOUSE	sp|Q00896|A1AT3_MOUSE	Serpina1c	serine (or cysteine) peptidase inhibitor, clade A, member 1C	X	X	X	X	X	X					X		X		
3	Up in Stroma compared to Luminal Epithelium	https://www.uniprot.org/uniprot/Q00897	Q00897	A1AT4_MOUSE	sp|Q00897|A1AT4_MOUSE	Serpina1d	serine (or cysteine) peptidase inhibitor, clade A, member 1D	X	X	X	X	X	X					X		X		
4	Up in Stroma compared to Luminal Epithelium	https://www.uniprot.org/uniprot/Q00898	Q00898	A1AT5_MOUSE	sp|Q00898|A1AT5_MOUSE	Serpina1e	serine (or cysteine) peptidase inhibitor, clade A, member 1E	X	X	X	X	X	X					X		X		
5	Up in Stroma compared to Luminal Epithelium	https://www.uniprot.org/uniprot/Q61247	Q61247	A2AP_MOUSE	sp|Q61247|A2AP_MOUSE	Serpinf2	serine (or cysteine) peptidase inhibitor, clade F, member 2	X	X	X	X	X	X	X				X		X		X
6	Up in Stroma compared to Luminal Epithelium	https://www.uniprot.org/uniprot/Q9JJ59	Q9JJ59	ABCB9_MOUSE	sp|Q9JJ59|ABCB9_MOUSE	Abcb9	ATP-binding cassette, sub-family B (MDR/TAP), member 9															
7	Up in Stroma compared to Luminal Epithelium	https://www.uniprot.org/uniprot/O55137	O55137	ACOT1_MOUSE	sp|O55137|ACOT1_MOUSE	Acot1	acyl-CoA thioesterase 1															
8	Up in Stroma compared to Luminal Epithelium	https://www.uniprot.org/uniprot/Q9QYR9	Q9QYR9	ACOT2_MOUSE	sp|Q9QYR9|ACOT2_MOUSE	Acot2	acyl-CoA thioesterase 2															
9	Up in Stroma & Glandular Epithelium compared to Luminal Epithelium	https://www.uniprot.org/uniprot/Q8VCW8	Q8VCW8	ACSF2_MOUSE	sp|Q8VCW8|ACSF2_MOUSE	Acsf2	acyl-CoA synthetase family member 2															
10	Up in Stroma compared to Luminal Epithelium	https://www.uniprot.org/uniprot/P68033	P68033	ACTC_MOUSE	sp|P68033|ACTC_MOUSE	Actc1	actin, alpha, cardiac muscle 1											X				X
11	Up in Stroma compared to Luminal Epithelium	https://www.uniprot.org/uniprot/P00329	P00329	ADH1_MOUSE	sp|P00329|ADH1_MOUSE	Adh1	alcohol dehydrogenase 1 (class I)															
12	Up in Stroma compared to Luminal Epithelium	https://www.uniprot.org/uniprot/P48962	P48962	ADT1_MOUSE	sp|P48962|ADT1_MOUSE	Slc25a4	mitochondrial creatine kinase contact site complex															
13	Up in Stroma compared to Luminal Epithelium	https://www.uniprot.org/uniprot/P07724	P07724	ALBU_MOUSE	sp|P07724|ALBU_MOUSE	Alb	Serum albumin											X		X	X	X
14	Up in Stroma compared to Luminal Epithelium	https://www.uniprot.org/uniprot/P47738	P47738	ALDH2_MOUSE	sp|P47738|ALDH2_MOUSE	Aldh2	aldehyde dehydrogenase 2, mitochondrial															
15	Up in Stroma compared to Luminal Epithelium	https://www.uniprot.org/uniprot/Q9EST5	Q9EST5	AN32B_MOUSE	sp|Q9EST5|AN32B_MOUSE	Anp32b	acidic (leucine-rich) nuclear phosphoprotein 32 family, member B															
16	Up in Stroma compared to Luminal Epithelium	https://www.uniprot.org/uniprot/O35639	O35639	ANXA3_MOUSE	sp|O35639|ANXA3_MOUSE	Anxa3	annexin A3															
17	Up in Stroma & Glandular Epithelium compared to Luminal Epithelium	https://www.uniprot.org/uniprot/Q64133	Q64133	AOFA_MOUSE	sp|Q64133|AOFA_MOUSE	Maoa	monoamine oxidase A															
18	Up in Stroma & Glandular Epithelium compared to Luminal Epithelium	https://www.uniprot.org/uniprot/Q8BW75	Q8BW75	AOFB_MOUSE	sp|Q8BW75|AOFB_MOUSE	Maob	amine oxidase activity															
19	Up in Stroma compared to Luminal Epithelium	https://www.uniprot.org/uniprot/Q00623	Q00623	APOA1_MOUSE	sp|Q00623|APOA1_MOUSE	Apoa1	apolipoprotein A-I					X	X					X		X		X
20	Up in Stroma compared to Luminal Epithelium	https://www.uniprot.org/uniprot/P09813	P09813	APOA2_MOUSE	sp|P09813|APOA2_MOUSE	Apoa2	apolipoprotein A-II					X						X		X		X
21	Up in Stroma compared to Luminal Epithelium	https://www.uniprot.org/uniprot/P06728	P06728	APOA4_MOUSE	sp|P06728|APOA4_MOUSE	Apoa4	apolipoprotein A-IV					X	X					X		X		X
22	Up in Stroma & Glandular Epithelium compared to Luminal Epithelium	https://www.uniprot.org/uniprot/P33622	P33622	APOC3_MOUSE	sp|P33622|APOC3_MOUSE	Apoc3	apolipoprotein C-III											X		X		
23	Up in Stroma compared to Luminal Epithelium	https://www.uniprot.org/uniprot/P08226	P08226	APOE_MOUSE	sp|P08226|APOE_MOUSE	Apoe	apolipoprotein E					X	X				X	X		X		X
24	Up in Stroma compared to Luminal Epithelium	https://www.uniprot.org/uniprot/Q91YI0	Q91YI0	ARLY_MOUSE	sp|Q91YI0|ARLY_MOUSE	Asl	argininosuccinate lyase															
25	Up in Stroma & Glandular Epithelium compared to Luminal Epithelium	https://www.uniprot.org/uniprot/Q8BSY0	Q8BSY0	ASPH_MOUSE	sp|Q8BSY0|ASPH_MOUSE	Asph	aspartate-beta-hydroxylase															
26	Up in Stroma compared to Luminal Epithelium	https://www.uniprot.org/uniprot/P97370	P97370	AT1B3_MOUSE	sp|P97370|AT1B3_MOUSE	Atp1b3	Sodium/potassium-transporting ATPase subunit beta-3															
27	Up in Stroma compared to Luminal Epithelium	https://www.uniprot.org/uniprot/Q923D2	Q923D2	BLVRB_MOUSE	sp|Q923D2|BLVRB_MOUSE	Blvrb	biliverdin reductase B (flavin reductase (NADPH))															
28	Up in Stroma compared to Luminal Epithelium	https://www.uniprot.org/uniprot/P35564	P35564	CALX_MOUSE	sp|P35564|CALX_MOUSE	Canx	calnexin										X					
29	Up in Stroma compared to Luminal Epithelium	https://www.uniprot.org/uniprot/Q64314	Q64314	CD34_MOUSE	sp|Q64314|CD34_MOUSE	Cd34	CD34 antigen						X							X		
30	Up in Stroma compared to Luminal Epithelium	https://www.uniprot.org/uniprot/P15379	P15379	CD44_MOUSE	sp|P15379|CD44_MOUSE	Cd44	CD44 antigen						X									
31	Up in Stroma & Glandular Epithelium compared to Luminal Epithelium	https://www.uniprot.org/uniprot/Q06890	Q06890	CLUS_MOUSE	sp|Q06890|CLUS_MOUSE	Clu	clusterin										X	X		X		X
32	Up in Stroma & Glandular Epithelium compared to Luminal Epithelium	https://www.uniprot.org/uniprot/P11087	P11087	CO1A1_MOUSE	sp|P11087|CO1A1_MOUSE	Col1a1	Collagen alpha-1(I) chain							X	X		X	X	X	X		
33	Up in Stroma & Glandular Epithelium compared to Luminal Epithelium	https://www.uniprot.org/uniprot/Q01149	Q01149	CO1A2_MOUSE	sp|Q01149|CO1A2_MOUSE	Col1a2	collagen, type I, alpha 2							X	X		X	X	X	X		
34	Up in Stroma compared to Luminal Epithelium	https://www.uniprot.org/uniprot/P01027	P01027	CO3_MOUSE	sp|P01027|CO3_MOUSE	C3	complement 3	X	X	X	X	X	X					X		X		X
35	Up in Stroma compared to Luminal Epithelium	https://www.uniprot.org/uniprot/P02463	P02463	CO4A1_MOUSE	sp|P02463|CO4A1_MOUSE	Col4a1	collagen, type IV, alpha 1									X	X		X	X	X	
36	Up in Stroma compared to Luminal Epithelium	https://www.uniprot.org/uniprot/P08122	P08122	CO4A2_MOUSE	sp|P08122|CO4A2_MOUSE	Col4a2	collagen, type IV, alpha 2									X	X		X	X	X	
37	Up in Stroma & Glandular Epithelium compared to Luminal Epithelium	https://www.uniprot.org/uniprot/Q04857	Q04857	CO6A1_MOUSE	sp|Q04857|CO6A1_MOUSE	Col6a1	collagen, type VI, alpha 1								X		X		X	X		
38	Up in Stroma & Glandular Epithelium compared to Luminal Epithelium	https://www.uniprot.org/uniprot/Q02788	Q02788	CO6A2_MOUSE	sp|Q02788|CO6A2_MOUSE	Col6a2	collagen, type VI, alpha 2								X		X	X	X	X		
39	Up in Stroma & Glandular Epithelium compared to Luminal Epithelium	https://www.uniprot.org/uniprot/A2AX52	A2AX52	CO6A4_MOUSE	sp|A2AX52|CO6A4_MOUSE	Col6a4	Collagen alpha-4(VI) chain								X		X		X	X		
40	Up in Stroma compared to Luminal Epithelium	https://www.uniprot.org/uniprot/Q8C6K9	Q8C6K9	CO6A6_MOUSE	sp|Q8C6K9|CO6A6_MOUSE	Col6a6	Collagen, type VI, alpha 6										X		X	X		
41	Up in Stroma & Glandular Epithelium compared to Luminal Epithelium	https://www.uniprot.org/uniprot/O35206	O35206	COFA1_MOUSE	sp|O35206|COFA1_MOUSE	Col15a1	collagen, type XV, alpha 1								X		X	X	X	X		
42	Up in Stroma compared to Luminal Epithelium	https://www.uniprot.org/uniprot/Q8K4Q8	Q8K4Q8	COL12_MOUSE	sp|Q8K4Q8|COL12_MOUSE	Colec12	collectin sub-family member 12															
43	Up in Stroma & Glandular Epithelium compared to Luminal Epithelium	https://www.uniprot.org/uniprot/O35348	O35348	COLQ_MOUSE	sp|O35348|COLQ_MOUSE	Colq	collagen-like tail subunit (single strand of homotrimer) of asymmetric acetylcholinesterase								X							
44	Up in Stroma compared to Luminal Epithelium	https://www.uniprot.org/uniprot/O89053	O89053	COR1A_MOUSE	sp|O89053|COR1A_MOUSE	Coro1a	coronin, actin binding protein 1A															
45	Up in Stroma compared to Luminal Epithelium	https://www.uniprot.org/uniprot/P43135	P43135	COT2_MOUSE	sp|P43135|COT2_MOUSE	Nr2f2	nuclear receptor subfamily 2, group F, member 2															
46	Up in Stroma compared to Luminal Epithelium	https://www.uniprot.org/uniprot/Q8C166	Q8C166	CPNE1_MOUSE	sp|Q8C166|CPNE1_MOUSE	Cpne1	copine I															
47	Up in Stroma compared to Luminal Epithelium	https://www.uniprot.org/uniprot/Q99LD8	Q99LD8	DDAH2_MOUSE	sp|Q99LD8|DDAH2_MOUSE	Ddah2	dimethylarginine dimethylaminohydrolase 2															
48	Up in Stroma compared to Luminal Epithelium	https://www.uniprot.org/uniprot/Q501J6	Q501J6	DDX17_MOUSE	sp|Q501J6|DDX17_MOUSE	Ddx17	DEAD (Asp-Glu-Ala-Asp) box polypeptide 17															
49	Up in Stroma compared to Luminal Epithelium	https://www.uniprot.org/uniprot/Q9JIK5	Q9JIK5	DDX21_MOUSE	sp|Q9JIK5|DDX21_MOUSE	Ddx21	DEAD (Asp-Glu-Ala-Asp) box polypeptide 21															
50	Up in Stroma compared to Luminal Epithelium	https://www.uniprot.org/uniprot/Q9QZZ6	Q9QZZ6	DERM_MOUSE	sp|Q9QZZ6|DERM_MOUSE	Dpt	dermatopontin							X			X	X	X	X		
51	Up in Stroma & Glandular Epithelium compared to Luminal Epithelium	https://www.uniprot.org/uniprot/P31001	P31001	DESM_MOUSE	sp|P31001|DESM_MOUSE	Des	desmin															
52	Up in Stroma & Glandular Epithelium compared to Luminal Epithelium	https://www.uniprot.org/uniprot/P51661	P51661	DHI2_MOUSE	sp|P51661|DHI2_MOUSE	Hsd11b2	hydroxysteroid 11-beta dehydrogenase 2															
53	Up in Stroma compared to Luminal Epithelium	https://www.uniprot.org/uniprot/Q9ESX5	Q9ESX5	DKC1_MOUSE	sp|Q9ESX5|DKC1_MOUSE	Dkc1	dyskeratosis congenita 1, dyskerin homolog (human)															
54	Up in Stroma compared to Luminal Epithelium	https://www.uniprot.org/uniprot/O08553	O08553	DPYL2_MOUSE	sp|O08553|DPYL2_MOUSE	Dpysl2	dihydropyrimidinase-like 2															
55	Up in Stroma compared to Luminal Epithelium	https://www.uniprot.org/uniprot/Q62188	Q62188	DPYL3_MOUSE	sp|Q62188|DPYL3_MOUSE	Dpysl3	dihydropyrimidinase-like 3											X				
56	Up in Stroma compared to Luminal Epithelium	https://www.uniprot.org/uniprot/Q9QXS6	Q9QXS6	DREB_MOUSE	sp|Q9QXS6|DREB_MOUSE	Dbn1	drebrin 1															
57	Up in Stroma compared to Luminal Epithelium	https://www.uniprot.org/uniprot/O70318	O70318	E41L2_MOUSE	sp|O70318|E41L2_MOUSE	Epb41l2	erythrocyte protein band 4.1-like 2															
58	Up in Stroma compared to Luminal Epithelium	https://www.uniprot.org/uniprot/Q01279	Q01279	EGFR_MOUSE	sp|Q01279|EGFR_MOUSE	Egfr	epidermal growth factor receptor						X									
59	Up in Stroma & Glandular Epithelium compared to Luminal Epithelium	https://www.uniprot.org/uniprot/Q8BH64	Q8BH64	EHD2_MOUSE	sp|Q8BH64|EHD2_MOUSE	Ehd2	EH-domain containing 2															
60	Up in Stroma compared to Luminal Epithelium	https://www.uniprot.org/uniprot/Q9EQP2	Q9EQP2	EHD4_MOUSE	sp|Q9EQP2|EHD4_MOUSE	Ehd4	EH-domain containing 4															
61	Up in Stroma & Glandular Epithelium compared to Luminal Epithelium	https://www.uniprot.org/uniprot/Q99K41	Q99K41	EMIL1_MOUSE	sp|Q99K41|EMIL1_MOUSE	Emilin1	EMILIN-1								X		X		X	X		
62	Up in Stroma compared to Luminal Epithelium	https://www.uniprot.org/uniprot/Q3U7R1	Q3U7R1	ESYT1_MOUSE	sp|Q3U7R1|ESYT1_MOUSE	Esyt1	Extended synaptotagmin-1															
63	Up in Stroma compared to Luminal Epithelium	https://www.uniprot.org/uniprot/Q3TZZ7	Q3TZZ7	ESYT2_MOUSE	sp|Q3TZZ7|ESYT2_MOUSE	Esyt2	Extended synaptotagmin-2															
64	Up in Stroma compared to Luminal Epithelium	https://www.uniprot.org/uniprot/Q61554	Q61554	FBN1_MOUSE	sp|Q61554|FBN1_MOUSE	Fbn1	fibrillin 1									X	X	X	X	X	X	
65	Up in Stroma compared to Luminal Epithelium	https://www.uniprot.org/uniprot/P35550	P35550	FBRL_MOUSE	sp|P35550|FBRL_MOUSE	Fbl	fibrillarin															
66	Up in Stroma compared to Luminal Epithelium	https://www.uniprot.org/uniprot/P29699	P29699	FETUA_MOUSE	sp|P29699|FETUA_MOUSE	Ahsg	alpha-2-HS-glycoprotein		X	X							X	X		X		X
67	Up in Stroma compared to Luminal Epithelium	https://www.uniprot.org/uniprot/E9PV24	E9PV24	FIBA_MOUSE	sp|E9PV24|FIBA_MOUSE	Fga	fibrinogen alpha chain						X					X		X		X
68	Up in Stroma & Glandular Epithelium compared to Luminal Epithelium	https://www.uniprot.org/uniprot/Q8K0E8	Q8K0E8	FIBB_MOUSE	sp|Q8K0E8|FIBB_MOUSE	Fgb	fibrinogen beta chain											X		X		X
69	Up in Stroma & Glandular Epithelium compared to Luminal Epithelium	https://www.uniprot.org/uniprot/Q8VCM7	Q8VCM7	FIBG_MOUSE	sp|Q8VCM7|FIBG_MOUSE	Fgg	fibrinogen gamma chain											X		X		X
70	Up in Stroma compared to Luminal Epithelium	https://www.uniprot.org/uniprot/P11276	P11276	FINC_MOUSE	sp|P11276|FINC_MOUSE	Fn1	fibronectin 1										X	X	X	X	X	X
71	Up in Stroma compared to Luminal Epithelium	https://www.uniprot.org/uniprot/Q8BTM8	Q8BTM8	FLNA_MOUSE	sp|Q8BTM8|FLNA_MOUSE	Flna	filamin, alpha										X					
72	Up in Stroma compared to Luminal Epithelium	https://www.uniprot.org/uniprot/Q8VHX6	Q8VHX6	FLNC_MOUSE	sp|Q8VHX6|FLNC_MOUSE	Flnc	filamin C, gamma															
73	Up in Stroma compared to Luminal Epithelium	https://www.uniprot.org/uniprot/P50285	P50285	FMO1_MOUSE	sp|P50285|FMO1_MOUSE	Fmo1	flavin containing monooxygenase 1															
74	Up in Stroma compared to Luminal Epithelium	https://www.uniprot.org/uniprot/P29391	P29391	FRIL1_MOUSE	sp|P29391|FRIL1_MOUSE	Ftl1	ferritin light chain 1															
75	Up in Stroma compared to Luminal Epithelium	https://www.uniprot.org/uniprot/Q61553	Q61553	FSCN1_MOUSE	sp|Q61553|FSCN1_MOUSE	Fscn1	fascin homolog 1, actin bundling protein (Strongylocentrotus purpuratus)															
76	Up in Stroma compared to Luminal Epithelium	https://www.uniprot.org/uniprot/Q9R0N0	Q9R0N0	GALK1_MOUSE	sp|Q9R0N0|GALK1_MOUSE	Galk1	galactokinase 1															
77	Up in Stroma compared to Luminal Epithelium	https://www.uniprot.org/uniprot/Q9Z2A9	Q9Z2A9	GGT5_MOUSE	sp|Q9Z2A9|GGT5_MOUSE	Ggt5	gamma-glutamyltransferase 5															
78	Up in Stroma compared to Luminal Epithelium	https://www.uniprot.org/uniprot/Q99JY3	Q99JY3	GIMA4_MOUSE	sp|Q99JY3|GIMA4_MOUSE	Gimap4	GTPase, IMAP family member 4															
79	Up in Stroma compared to Luminal Epithelium	https://www.uniprot.org/uniprot/P08752	P08752	GNAI2_MOUSE	sp|P08752|GNAI2_MOUSE	Gnai2	guanine nucleotide binding protein (G protein), alpha inhibiting 2															
80	Up in Stroma compared to Luminal Epithelium	https://www.uniprot.org/uniprot/Q9QZF2	Q9QZF2	GPC1_MOUSE	sp|Q9QZF2|GPC1_MOUSE	Gpc1	glypican 1										X		X	X		
81	Up in Stroma compared to Luminal Epithelium	https://www.uniprot.org/uniprot/Q9R087	Q9R087	GPC6_MOUSE	sp|Q9R087|GPC6_MOUSE	Gpc6	glypican 6										X		X	X		
82	Up in Stroma compared to Luminal Epithelium	https://www.uniprot.org/uniprot/Q8K297	Q8K297	GT251_MOUSE	sp|Q8K297|GT251_MOUSE	Glt25d1	glycosyltransferase 25 domain containing 1															
83	Up in Stroma compared to Luminal Epithelium	https://www.uniprot.org/uniprot/Q8CGP6	Q8CGP6	H2A1H_MOUSE	sp|Q8CGP6|H2A1H_MOUSE	Hist1h2ah	histone cluster 1, H2ah															
84	Up in Stroma compared to Luminal Epithelium	https://www.uniprot.org/uniprot/P84244	P84244	H33_MOUSE	sp|P84244|H33_MOUSE	H3f3a	H3 histone, family 3A															
85	Up in Stroma compared to Luminal Epithelium	https://www.uniprot.org/uniprot/P62806	P62806	H4_MOUSE	sp|P62806|H4_MOUSE	Hist1h4m	histone cluster 1, H4m															
86	Up in Stroma compared to Luminal Epithelium	https://www.uniprot.org/uniprot/O08756	O08756	HCD2_MOUSE	sp|O08756|HCD2_MOUSE	Hsd17b10	hydroxysteroid (17-beta) dehydrogenase 10															
87	Up in Stroma compared to Luminal Epithelium	https://www.uniprot.org/uniprot/P63158	P63158	HMGB1_MOUSE	sp|P63158|HMGB1_MOUSE	Hmgb1	high mobility group box 1											X		X		
88	Up in Stroma compared to Luminal Epithelium	https://www.uniprot.org/uniprot/Q3TEA8	Q3TEA8	HP1B3_MOUSE	sp|Q3TEA8|HP1B3_MOUSE	Hp1bp3	heterochromatin protein 1, binding protein 3															
89	Up in Stroma compared to Luminal Epithelium	https://www.uniprot.org/uniprot/P97290	P97290	IC1_MOUSE	sp|P97290|IC1_MOUSE	Serping1	serine (or cysteine) peptidase inhibitor, clade G, member 1	X	X	X	X	X	X					X		X		X
90	Up in Stroma compared to Luminal Epithelium	https://www.uniprot.org/uniprot/P0DOV1	P0DOV1	IFI5B_MOUSE	sp|P0DOV1|IFI5B_MOUSE	Mnda	myeloid cell nuclear differentiation antigen															
91	Up in Stroma compared to Luminal Epithelium	https://www.uniprot.org/uniprot/P01867	P01867	IGG2B_MOUSE	sp|P01867|IGG2B_MOUSE	Igh-3	immunoglobulin heavy chain 3 (serum IgG2b)															
92	Up in Stroma compared to Luminal Epithelium	https://www.uniprot.org/uniprot/P01872	P01872	IGHM_MOUSE	sp|P01872|IGHM_MOUSE	Ighmbp2	Immunoglobulin heavy constant mu						X					X		X		X
93	Up in Stroma compared to Luminal Epithelium	https://www.uniprot.org/uniprot/P01837	P01837	IGKC_MOUSE	sp|P01837|IGKC_MOUSE	Igk-C	immunoglobulin kappa chain, constant region															X
94	Up in Stroma compared to Luminal Epithelium	https://www.uniprot.org/uniprot/Q9D154	Q9D154	ILEUA_MOUSE	sp|Q9D154|ILEUA_MOUSE	Serpinb1a	serine (or cysteine) peptidase inhibitor, clade B, member 1a	X	X	X	X	X	X					X				
95	Up in Stroma compared to Luminal Epithelium	https://www.uniprot.org/uniprot/P70168	P70168	IMB1_MOUSE	sp|P70168|IMB1_MOUSE	Kpnb1	karyopherin (importin) beta 1															
96	Up in Stroma compared to Luminal Epithelium	https://www.uniprot.org/uniprot/P24547	P24547	IMDH2_MOUSE	sp|P24547|IMDH2_MOUSE	Impdh2	inosine 5'-phosphate dehydrogenase 2															
97	Up in Stroma compared to Luminal Epithelium	https://www.uniprot.org/uniprot/Q8VI75	Q8VI75	IPO4_MOUSE	sp|Q8VI75|IPO4_MOUSE	Ipo4	importin 4															
98	Up in Stroma compared to Luminal Epithelium	https://www.uniprot.org/uniprot/Q6GU68	Q6GU68	ISLR_MOUSE	sp|Q6GU68|ISLR_MOUSE	Islr	immunoglobulin superfamily containing leucine-rich repeat													X		
99	Up in Stroma compared to Luminal Epithelium	https://www.uniprot.org/uniprot/Q91V64	Q91V64	ISOC1_MOUSE	sp|Q91V64|ISOC1_MOUSE	Isoc1	isochorismatase domain containing 1															
100	Up in Stroma compared to Luminal Epithelium	https://www.uniprot.org/uniprot/Q61702	Q61702	ITIH1_MOUSE	sp|Q61702|ITIH1_MOUSE	Itih1	inter-alpha trypsin inhibitor, heavy chain 1	X	X	X	X	X	X							X		X
101	Up in Stroma & Glandular Epithelium compared to Luminal Epithelium	https://www.uniprot.org/uniprot/Q61703	Q61703	ITIH2_MOUSE	sp|Q61703|ITIH2_MOUSE	Itih2	inter-alpha trypsin inhibitor, heavy chain 2	X	X	X	X	X	X							X		X
102	Up in Stroma & Glandular Epithelium compared to Luminal Epithelium	https://www.uniprot.org/uniprot/Q61704	Q61704	ITIH3_MOUSE	sp|Q61704|ITIH3_MOUSE	Itih3	inter-alpha trypsin inhibitor, heavy chain 3	X	X	X	X	X	X							X		
103	Up in Stroma compared to Luminal Epithelium	https://www.uniprot.org/uniprot/P52480	P52480	KPYM_MOUSE	sp|P52480|KPYM_MOUSE	Pkm2	pyruvate kinase, muscle										X					
104	Up in Stroma compared to Luminal Epithelium	https://www.uniprot.org/uniprot/P01631	P01631	KV2A7_MOUSE	sp|P01631|KV2A7_MOUSE	Igh-1a	immunoglobulin heavy chain 1a (serum IgG2a)															
105	Up in Stroma compared to Luminal Epithelium	https://www.uniprot.org/uniprot/P97927	P97927	LAMA4_MOUSE	sp|P97927|LAMA4_MOUSE	Lama4	laminin, alpha 4										X		X	X	X	
106	Up in Stroma compared to Luminal Epithelium	https://www.uniprot.org/uniprot/P02469	P02469	LAMB1_MOUSE	sp|P02469|LAMB1_MOUSE	Lamb1-1	laminin B1 subunit 1									X	X	X	X	X	X	
107	Up in Stroma compared to Luminal Epithelium	https://www.uniprot.org/uniprot/O08573	O08573	LEG9_MOUSE	sp|O08573|LEG9_MOUSE	Lgals9	lectin, galactose binding, soluble 9													X		
108	Up in Stroma compared to Luminal Epithelium	https://www.uniprot.org/uniprot/Q99JW4	Q99JW4	LIMS1_MOUSE	sp|Q99JW4|LIMS1_MOUSE	Lims1	LIM and senescent cell antigen-like domains 1															
109	Up in Stroma compared to Luminal Epithelium	https://www.uniprot.org/uniprot/P21619	P21619	LMNB2_MOUSE	sp|P21619|LMNB2_MOUSE	Lmnb2	lamin B2															
110	Up in Stroma compared to Luminal Epithelium	https://www.uniprot.org/uniprot/Q91ZX7	Q91ZX7	LRP1_MOUSE	sp|Q91ZX7|LRP1_MOUSE	Lrp1	low density lipoprotein receptor-related protein 1															
111	Up in Stroma compared to Luminal Epithelium	https://www.uniprot.org/uniprot/P19973	P19973	LSP1_MOUSE	sp|P19973|LSP1_MOUSE	Lsp1	lymphocyte specific 1															
112	Up in Stroma & Glandular Epithelium compared to Luminal Epithelium	https://www.uniprot.org/uniprot/P51885	P51885	LUM_MOUSE	sp|P51885|LUM_MOUSE	Lum	lumican							X			X	X	X	X		
113	Up in Stroma compared to Luminal Epithelium	https://www.uniprot.org/uniprot/P14873	P14873	MAP1B_MOUSE	sp|P14873|MAP1B_MOUSE	Mtap1b	microtubule-associated protein 1B															
114	Up in Stroma compared to Luminal Epithelium	https://www.uniprot.org/uniprot/Q9Z2D6	Q9Z2D6	MECP2_MOUSE	sp|Q9Z2D6|MECP2_MOUSE	Mecp2	methyl CpG binding protein 2											X				
115	Up in Stroma compared to Luminal Epithelium	https://www.uniprot.org/uniprot/Q62000	Q62000	MIME_MOUSE	sp|Q62000|MIME_MOUSE	Ogn	osteoglycin										X	X	X	X		
116	Up in Stroma compared to Luminal Epithelium	https://www.uniprot.org/uniprot/Q64449	Q64449	MRC2_MOUSE	sp|Q64449|MRC2_MOUSE	Mrc2	mannose receptor, C type 2						X									
117	Up in Stroma compared to Luminal Epithelium	https://www.uniprot.org/uniprot/P28665	P28665	MUG1_MOUSE	sp|P28665|MUG1_MOUSE	Mug1	murinoglobulin 1	X	X	X	X	X	X					X		X		
118	Up in Stroma compared to Luminal Epithelium	https://www.uniprot.org/uniprot/Q61879	Q61879	MYH10_MOUSE	sp|Q61879|MYH10_MOUSE	Myh10	myosin, heavy polypeptide 10, non-muscle															
119	Up in Stroma compared to Luminal Epithelium	https://www.uniprot.org/uniprot/Q9CZR2	Q9CZR2	NALD2_MOUSE	sp|Q9CZR2|NALD2_MOUSE	Naalad2	N-acetylated alpha-linked acidic dipeptidase 2															
120	Up in Stroma compared to Luminal Epithelium	https://www.uniprot.org/uniprot/Q8CFI0	Q8CFI0	NED4L_MOUSE	sp|Q8CFI0|NED4L_MOUSE	Nedd4l	neural precursor cell expressed, developmentally down-regulated gene 4-like															
121	Up in Stroma compared to Luminal Epithelium	https://www.uniprot.org/uniprot/P46935	P46935	NEDD4_MOUSE	sp|P46935|NEDD4_MOUSE	Nedd4	neural precursor cell expressed, developmentally down-regulated 4															
122	Up in Stroma & Glandular Epithelium compared to Luminal Epithelium	https://www.uniprot.org/uniprot/P70255	P70255	NFIC_MOUSE	sp|P70255|NFIC_MOUSE	Nfic	nuclear factor I/C															
123	Up in Stroma compared to Luminal Epithelium	https://www.uniprot.org/uniprot/Q61941	Q61941	NNTM_MOUSE	sp|Q61941|NNTM_MOUSE	Nnt	nicotinamide nucleotide transhydrogenase															
124	Up in Stroma compared to Luminal Epithelium	https://www.uniprot.org/uniprot/Q9D6Z1	Q9D6Z1	NOP56_MOUSE	sp|Q9D6Z1|NOP56_MOUSE	Nop56	NOP56 ribonucleoprotein homolog (yeast)															
125	Up in Stroma compared to Luminal Epithelium	https://www.uniprot.org/uniprot/Q6DFW4	Q6DFW4	NOP58_MOUSE	sp|Q6DFW4|NOP58_MOUSE	Nop58	NOP58 ribonucleoprotein homolog (yeast)															
126	Up in Stroma compared to Luminal Epithelium	https://www.uniprot.org/uniprot/Q9CPP0	Q9CPP0	NPM3_MOUSE	sp|Q9CPP0|NPM3_MOUSE	Npm3	nucleoplasmin 3															
127	Up in Stroma compared to Luminal Epithelium	https://www.uniprot.org/uniprot/P09405	P09405	NUCL_MOUSE	sp|P09405|NUCL_MOUSE	Ncl	nucleolin						X									
128	Up in Stroma compared to Luminal Epithelium	https://www.uniprot.org/uniprot/Q60597	Q60597	ODO1_MOUSE	sp|Q60597|ODO1_MOUSE	Ogdh	oxoglutarate dehydrogenase (lipoamide)															
129	Up in Glandular Epithelium compared to Luminal Epithelium	https://www.uniprot.org/uniprot/B9EJ86	B9EJ86	OSBL8_MOUSE	sp|B9EJ86|OSBL8_MOUSE	Osbpl8	Oxysterol-binding protein-related protein 8															
130	Up in Stroma compared to Luminal Epithelium	https://www.uniprot.org/uniprot/Q9DCC4	Q9DCC4	P5CR3_MOUSE	sp|Q9DCC4|P5CR3_MOUSE	Pycrl	pyrroline-5-carboxylate reductase-like															
131	Up in Stroma compared to Luminal Epithelium	https://www.uniprot.org/uniprot/Q9ET54	Q9ET54	PALLD_MOUSE	sp|Q9ET54|PALLD_MOUSE	Palld	palladin, cytoskeletal associated protein															
132	Up in Stroma compared to Luminal Epithelium	https://www.uniprot.org/uniprot/P11103	P11103	PARP1_MOUSE	sp|P11103|PARP1_MOUSE	Parp1	poly (ADP-ribose) polymerase family, member 1															
133	Up in Stroma compared to Luminal Epithelium	https://www.uniprot.org/uniprot/Q91ZA3	Q91ZA3	PCCA_MOUSE	sp|Q91ZA3|PCCA_MOUSE	Pcca	propionyl-Coenzyme A carboxylase															
134	Up in Stroma compared to Luminal Epithelium	https://www.uniprot.org/uniprot/Q99MN9	Q99MN9	PCCB_MOUSE	sp|Q99MN9|PCCB_MOUSE	Pccb	propionyl-Coenzyme A carboxylase															
135	Up in Stroma compared to Luminal Epithelium	https://www.uniprot.org/uniprot/Q61823	Q61823	PDCD4_MOUSE	sp|Q61823|PDCD4_MOUSE	Pdcd4	programmed cell death 4															
136	Up in Stroma compared to Luminal Epithelium	https://www.uniprot.org/uniprot/O70209	O70209	PDLI3_MOUSE	sp|O70209|PDLI3_MOUSE	Pdlim3	PDZ and LIM domain 3															
137	Up in Stroma compared to Luminal Epithelium	https://www.uniprot.org/uniprot/Q05793	Q05793	PGBM_MOUSE	sp|Q05793|PGBM_MOUSE	Hspg2	Basement membrane-specific heparan sulfate proteoglycan core protein										X	X	X	X	X	
138	Up in Stroma compared to Luminal Epithelium	https://www.uniprot.org/uniprot/P26618	P26618	PGFRA_MOUSE	sp|P26618|PGFRA_MOUSE	Pdgfra	platelet derived growth factor receptor, alpha polypeptide						X									
139	Up in Stroma & Glandular Epithelium compared to Luminal Epithelium	https://www.uniprot.org/uniprot/P28653	P28653	PGS1_MOUSE	sp|P28653|PGS1_MOUSE	Bgn	biglycan										X		X	X		
140	Up in Stroma & Glandular Epithelium compared to Luminal Epithelium	https://www.uniprot.org/uniprot/P28654	P28654	PGS2_MOUSE	sp|P28654|PGS2_MOUSE	Dcn	decorin										X	X	X	X		
141	Up in Stroma compared to Luminal Epithelium	https://www.uniprot.org/uniprot/Q9JI48	Q9JI48	PLAC8_MOUSE	sp|Q9JI48|PLAC8_MOUSE	Plac8	placenta-specific 8															
142	Up in Stroma compared to Luminal Epithelium	https://www.uniprot.org/uniprot/Q9Z239	Q9Z239	PLM_MOUSE	sp|Q9Z239|PLM_MOUSE	Fxyd1	FXYD domain-containing ion transport regulator 1															
143	Up in Stroma compared to Luminal Epithelium	https://www.uniprot.org/uniprot/P20918	P20918	PLMN_MOUSE	sp|P20918|PLMN_MOUSE	Plg	plasminogen						X					X		X		X
144	Up in Stroma compared to Luminal Epithelium	https://www.uniprot.org/uniprot/Q9R0E2	Q9R0E2	PLOD1_MOUSE	sp|Q9R0E2|PLOD1_MOUSE	Plod1	procollagen-lysine, 2-oxoglutarate 5-dioxygenase 1															
145	Up in Stroma compared to Luminal Epithelium	https://www.uniprot.org/uniprot/Q9R0E1	Q9R0E1	PLOD3_MOUSE	sp|Q9R0E1|PLOD3_MOUSE	Plod3	procollagen-lysine, 2-oxoglutarate 5-dioxygenase 3							X								
146	Up in Stroma compared to Luminal Epithelium	https://www.uniprot.org/uniprot/Q61233	Q61233	PLSL_MOUSE	sp|Q61233|PLSL_MOUSE	Lcp1	lymphocyte cytosolic protein 1											X				
147	Up in Stroma compared to Luminal Epithelium	https://www.uniprot.org/uniprot/Q62009	Q62009	POSTN_MOUSE	sp|Q62009|POSTN_MOUSE	Postn	periostin, osteoblast specific factor										X	X	X	X		
148	Up in Stroma compared to Luminal Epithelium	https://www.uniprot.org/uniprot/O54724	O54724	PTRF_MOUSE	sp|O54724|PTRF_MOUSE	Ptrf	polymerase I and transcript release factor															
149	Up in Stroma & Glandular Epithelium compared to Luminal Epithelium	https://www.uniprot.org/uniprot/Q61838	Q61838	PZP_MOUSE	sp|Q61838|PZP_MOUSE	Pzp	pregnancy zone protein	X	X	X	X	X	X					X		X		
150	Up in Stroma compared to Luminal Epithelium	https://www.uniprot.org/uniprot/Q05186	Q05186	RCN1_MOUSE	sp|Q05186|RCN1_MOUSE	Rcn1	reticulocalbin 1															
151	Up in Stroma compared to Luminal Epithelium	https://www.uniprot.org/uniprot/Q01730	Q01730	RSU1_MOUSE	sp|Q01730|RSU1_MOUSE	Rsu1	Ras suppressor protein 1															
152	Up in Stroma compared to Luminal Epithelium	https://www.uniprot.org/uniprot/Q01405	Q01405	SC23A_MOUSE	sp|Q01405|SC23A_MOUSE	Sec23a	Protein transport protein Sec23A															
153	Up in Stroma compared to Luminal Epithelium	https://www.uniprot.org/uniprot/P42208	P42208	SEPT2_MOUSE	sp|P42208|SEPT2_MOUSE	Sept2	septin 2						X									
154	Up in Stroma compared to Luminal Epithelium	https://www.uniprot.org/uniprot/O55131	O55131	SEPT7_MOUSE	sp|O55131|SEPT7_MOUSE	Sept7	septin 7															
155	Up in Stroma compared to Luminal Epithelium	https://www.uniprot.org/uniprot/Q61753	Q61753	SERA_MOUSE	sp|Q61753|SERA_MOUSE	Phgdh	3-phosphoglycerate dehydrogenase															
156	Up in Stroma compared to Luminal Epithelium	https://www.uniprot.org/uniprot/P19324	P19324	SERPH_MOUSE	sp|P19324|SERPH_MOUSE	Serpinh1	serine (or cysteine) peptidase inhibitor, clade H, member 1	X	X	X	X	X	X	X				X				
157	Up in Stroma compared to Luminal Epithelium	https://www.uniprot.org/uniprot/Q9EQU5	Q9EQU5	SET_MOUSE	sp|Q9EQU5|SET_MOUSE	Set	SET translocation															
158	Up in Stroma compared to Luminal Epithelium	https://www.uniprot.org/uniprot/Q61235	Q61235	SNTB2_MOUSE	sp|Q61235|SNTB2_MOUSE	Sntb2	syntrophin, basic 2															
159	Up in Stroma compared to Luminal Epithelium	https://www.uniprot.org/uniprot/P07759	P07759	SPA3K_MOUSE	sp|P07759|SPA3K_MOUSE	Serpina3k	Serine protease inhibitor A3K	X	X	X	X	X	X					X		X		X
160	Up in Stroma compared to Luminal Epithelium	https://www.uniprot.org/uniprot/Q60854	Q60854	SPB6_MOUSE	sp|Q60854|SPB6_MOUSE	Serpinb6a	serine (or cysteine) peptidase inhibitor, clade B, member 6a															
161	Up in Stroma compared to Luminal Epithelium	https://www.uniprot.org/uniprot/Q62261	Q62261	SPTB2_MOUSE	sp|Q62261|SPTB2_MOUSE	Spnb2	spectrin beta 2															
162	Up in Stroma compared to Luminal Epithelium	https://www.uniprot.org/uniprot/P16546	P16546	SPTN1_MOUSE	sp|P16546|SPTN1_MOUSE	Spna2	spectrin alpha 2															
163	Up in Stroma compared to Luminal Epithelium	https://www.uniprot.org/uniprot/Q9CY50	Q9CY50	SSRA_MOUSE	sp|Q9CY50|SSRA_MOUSE	Ssr1	signal sequence receptor, alpha															
164	Up in Stroma compared to Luminal Epithelium	https://www.uniprot.org/uniprot/Q8CI59	Q8CI59	STEA3_MOUSE	sp|Q8CI59|STEA3_MOUSE	Steap3	STEAP family member 3															
165	Up in Glandular Epithelium compared to Luminal Epithelium	https://www.uniprot.org/uniprot/P32921	P32921	SYWC_MOUSE	sp|P32921|SYWC_MOUSE	Wars	tryptophanyl-tRNA synthetase															
166	Up in Stroma compared to Luminal Epithelium	https://www.uniprot.org/uniprot/P68369	P68369	TBA1A_MOUSE	sp|P68369|TBA1A_MOUSE	Tuba1a	tubulin, alpha 1A															
167	Up in Stroma compared to Luminal Epithelium	https://www.uniprot.org/uniprot/P21981	P21981	TGM2_MOUSE	sp|P21981|TGM2_MOUSE	Tgm2	transglutaminase 2, C polypeptide										X		X			
168	Up in Stroma compared to Luminal Epithelium	https://www.uniprot.org/uniprot/Q8QZT1	Q8QZT1	THIL_MOUSE	sp|Q8QZT1|THIL_MOUSE	Acat1	acetyl-Coenzyme A acetyltransferase 1															
169	Up in Stroma compared to Luminal Epithelium	https://www.uniprot.org/uniprot/Q8BWT1	Q8BWT1	THIM_MOUSE	sp|Q8BWT1|THIM_MOUSE	Acaa2	acetyl-Coenzyme A acyltransferase 2 (mitochondrial 3-oxoacyl-Coenzyme A thiolase)															
170	Up in Stroma compared to Luminal Epithelium	https://www.uniprot.org/uniprot/Q62318	Q62318	TIF1B_MOUSE	sp|Q62318|TIF1B_MOUSE	Trim28	tripartite motif-containing 28															
171	Up in Stroma compared to Luminal Epithelium	https://www.uniprot.org/uniprot/P26039	P26039	TLN1_MOUSE	sp|P26039|TLN1_MOUSE	Tln1	talin 1															
172	Up in Stroma compared to Luminal Epithelium	https://www.uniprot.org/uniprot/Q64511	Q64511	TOP2B_MOUSE	sp|Q64511|TOP2B_MOUSE	Top2b	topoisomerase (DNA) II beta															
173	Up in Stroma compared to Luminal Epithelium	https://www.uniprot.org/uniprot/P58771	P58771	TPM1_MOUSE	sp|P58771|TPM1_MOUSE	Tpm1	tropomyosin 1, alpha															
174	Up in Stroma compared to Luminal Epithelium	https://www.uniprot.org/uniprot/Q6IRU2	Q6IRU2	TPM4_MOUSE	sp|Q6IRU2|TPM4_MOUSE	Tpm4	tropomyosin 4															
175	Up in Stroma compared to Luminal Epithelium	https://www.uniprot.org/uniprot/Q9CRB6	Q9CRB6	TPPP3_MOUSE	sp|Q9CRB6|TPPP3_MOUSE	Tppp3	tubulin polymerization-promoting protein family member 3															
176	Up in Stroma compared to Luminal Epithelium	https://www.uniprot.org/uniprot/Q921I1	Q921I1	TRFE_MOUSE	sp|Q921I1|TRFE_MOUSE	Trf	transferrin															
177	Up in Stroma compared to Luminal Epithelium	https://www.uniprot.org/uniprot/Q62465	Q62465	VAT1_MOUSE	sp|Q62465|VAT1_MOUSE	Vat1	Synaptic vesicle membrane protein VAT-1 homolog															
