# Supplementary material for: Automated mass spectrometry imaging of over 2000 proteins from tissue sections at 100-μm spatial resolution
Source: Nat Commun. 2020 Jan 7;11:8. doi: 10.1038/s41467-019-13858-z (PMC6946663; doi:10.1038/s41467-019-13858-z)
Supplement: Supplementary file 3 — Reporting Summary [file 41467_2019_13858_MOESM3_ESM.pdf]

nature research

Corresponding author(s): Kristin Burnum-Johnson
Ryan Kelly

Last updated by author(s): Oct 2, 2019

Reporting Summary

Nature Research wishes to improve the reproducibility of the work that we publish. This form provides structure for consistency and transparency in reporting. For further information on Nature Research policies, see Authors & Referees and the Editorial Policy Checklist.

Statistics

For all statistical analyses, confirm that the following items are present in the figure legend, table legend, main text, or Methods section.

n/a	Confirmed	
☐	☒	The exact sample size (*n*) for each experimental group/condition, given as a discrete number and unit of measurement
☐	☒	A statement on whether measurements were taken from distinct samples or whether the same sample was measured repeatedly
☐	☒	The statistical test(s) used AND whether they are one- or two-sided<br>*Only common tests should be described solely by name; describe more complex techniques in the Methods section.*
☐	☒	A description of all covariates tested
☐	☒	A description of any assumptions or corrections, such as tests of normality and adjustment for multiple comparisons
☐	☒	A full description of the statistical parameters including central tendency (e.g. means) or other basic estimates (e.g. regression coefficient) AND variation (e.g. standard deviation) or associated estimates of uncertainty (e.g. confidence intervals)
☐	☒	For null hypothesis testing, the test statistic (e.g. *F*, *t*, *r*) with confidence intervals, effect sizes, degrees of freedom and *P* value noted<br>*Give P values as exact values whenever suitable.*
☒	☐	For Bayesian analysis, information on the choice of priors and Markov chain Monte Carlo settings
☒	☐	For hierarchical and complex designs, identification of the appropriate level for tests and full reporting of outcomes
☐	☒	Estimates of effect sizes (e.g. Cohen's *d*, Pearson's *r*), indicating how they were calculated

Our web collection on statistics for biologists contains articles on many of the points above.

Software and code

Policy information about availability of computer code

Data collection	All raw proteomic files were processed using MaxQuant (version 1.5.3.30) for feature detection, database searching and protein/peptide quantification. Mass spectra were searched against the Uniprot Mus Musculus database downloaded in October 2016, containing 16,825 sequence entries. Carbamidomethylation of cysteine was set as a fixed modification and N-terminal acetylation and oxidation of methionine were allowed as variable modifications. A peptide length > 6 was required with a maximum of two missed cleavages allowed, and a false discovery rate of 0.01. The searches were completed twice with these settings, first with the match-between-runs (MBR) feature enabled and then without, for comparison. Contaminants and reverse sequences were removed from the peptides.txt file prior to use for downstream statistical analysis and image display.
Data analysis	R 3.5.0 statistical software was used to analyze and plot data. All code packages used are publicly available on the Comprehensive R Archive Network (https://cran.r-project.org) with the exception of trelliscope, used to generate interactive figures, available on Github (https://github.com/hafen/trelliscope) and pmartR for proteomics data preprocessing available on Github (https://github.com/pmartR/pmartR)

For manuscripts utilizing custom algorithms or software that are central to the research but not yet described in published literature, software must be made available to editors/reviewers. We strongly encourage code deposition in a community repository (e.g. GitHub). See the Nature Research guidelines for submitting code & software for further information.

Data

Policy information about availability of data

All manuscripts must include a data availability statement. This statement should provide the following information, where applicable:

- Accession codes, unique identifiers, or web links for publicly available datasets
- A list of figures that have associated raw data
- A description of any restrictions on data availability

Our searchable Trelliscope software platform (http://msc-viz.emsl.pnnl.gov/nanoPOTS_PI_MS/) contains statistical metrics from the dominant cell population study and complementary images of all 2,298 and 2,447 quantifiable MaxQuant and MaxQuant MBR proteins, respectively, and 12,495 and 14,673 quantifiable MaxQuant

and MaxQuant MBR peptides, respectively, from the S- and LE-dominant sections. The raw datasets for this study are available on MassIVE accession number MSV000084421

Field-specific reporting

Please select the one below that is the best fit for your research. If you are not sure, read the appropriate sections before making your selection.

☒ Life sciences ☐ Behavioural & social sciences ☐ Ecological, evolutionary & environmental sciences

For a reference copy of the document with all sections, see nature.com/documents/nr-reporting-summary-flat.pdf

Life sciences study design

All studies must disclose on these points even when the disclosure is negative.

Sample size

To validate the findings in our proteomic images, we performed a complementary study in which we used LCM, nanoPOTS, and LC-MS/MS analyses to isolate, characterize, and statistically compare Luminal Epithelial (LE), Stroma (S), and Glandular Epithelial (GE) cells across multiple uterine tissue sections. This dominant cell population study contained 15 LC-MS/MS instrument runs associated with 15 unique biological samples, 5 S samples, 5 LE samples, 5 GE samples, in which 100–200 ng of these unique cell populations were captured from 3–5 sections for each of the 15 samples.

Data exclusions

No data were excluded from the analyses

Replication

Proteins of interest discussed in the manuscript were statistically significant (<0.05 adjusted p-value) in our dominant cell population study and had complimentary spatial distributions in our proteome imaging data. Proteome imaging was performed on a S-dominant tissue section and a LE-dominant tissue section. The S-dominant tissue section is comprised of 24 LC-MS/MS instrument runs associated with 24 unique voxels, 4 containing GE & S, 8 containing LE, and 12 containing S. The LE-dominant tissue section also contains 24 LC-MS/MS instrument runs associated with 24 voxels, in this case with 2 containing GE & S, 14 containing LE, and 8 containing S.

Randomization

LC-MS/MS instrument run orders were randomized across our three cell types (LE, S, and GE) in our dominant cell population study and our proteome imaging studies.

Blinding

Investigators were not blinded to group allocation during data collection or analysis

Reporting for specific materials, systems and methods

We require information from authors about some types of materials, experimental systems and methods used in many studies. Here, indicate whether each material, system or method listed is relevant to your study. If you are not sure if a list item applies to your research, read the appropriate section before selecting a response.

Materials & experimental systems

n/a	Involved in the study
☒	☐ Antibodies
☒	☐ Eukaryotic cell lines
☒	☐ Palaeontology
☐	☒ Animals and other organisms
☒	☐ Human research participants
☒	☐ Clinical data

Methods

n/a	Involved in the study
☒	☐ ChIP-seq
☒	☐ Flow cytometry
☒	☐ MRI-based neuroimaging

Animals and other organisms

Policy information about studies involving animals; ARRIVE guidelines recommended for reporting animal research

Laboratory animals

Mouse uterine tissue study, adult CD-1 uterine Wnt5a null female mice (housed at Cincinnati Children's Hospital, 20-25 g, 48-60 days old); Mouse liver tissue study C57BL/6J were obtained from Jackson Labs and housed at PNNL

Wild animals

did not involve wild animals

Field-collected samples

This study did not involve samples collected from the field

Ethics oversight

Mouse uterine tissue: All mice used in this study were housed in the Cincinnati Children’s Animal Care Facility according to NIH and institutional guidelines for the use of laboratory animals. All protocols for this study were reviewed and approved by the Cincinnati Children’s Research Foundation Institutional Animal Care and Use Committee.
Mouse liver tissue: All mice used in this study were housed at PNNL according to NIH and institutional guidelines for the use of laboratory animals. All protocols for this study were reviewed and approved by the Institutional Animal Care and Use Committee

of Battelle, Pacific Northwest Division.

Note that full information on the approval of the study protocol must also be provided in the manuscript.
