# Supplementary material for: Automated mass spectrometry imaging of over 2000 proteins from tissue sections at 100-μm spatial resolution
Source: Nat Commun. 2020 Jan 7;11:8. doi: 10.1038/s41467-019-13858-z (PMC6946663; doi:10.1038/s41467-019-13858-z)
Supplement: Supplementary file 4 — Description of Additional Supplementary Files [file 41467_2019_13858_MOESM4_ESM.docx]

**Description of Additional Supplementary Files**

File Name: Supplementary Movie 1

Description: This video tutorial provides details on how researchers can search the nanoPOTS proteomics imaging data with Trelliscope.

File Name: Supplementary Data 1

Description: For the proteins significantly increased in the Luminal Epithelium (LE) and/or Glandular Epithelium (GE) vs. Stroma this data file contains the images, from both MaxQuant and MaxQuant MBR, for these 149 proteins in addition to the associated box plots from the dominant cell population study. For the box plots the midline is the median of the data, with the upper and lower limits of the box being the third and first quartile (75th and 25th percentile), respectively. By default, the whiskers will extend to the minimum and maximum observed values up to 1.5 times the interquartile range from the top (bottom) of the box to the furthest datum within that distance. If there are any data beyond that distance, they are represented individually as points.

File Name: Supplementary Data 2

Description: For the proteins significantly increased in the stroma and/or glandular epithelium vs. luminal epithelium this data file contains the images, from both MaxQuant and MaxQuant MBR, for these 177 proteins in addition to the associated box plots from the dominant cell population study. For the box plots the midline is the median of the data, with the upper and lower limits of the box being the third and first quartile (75th and 25th percentile), respectively. By default, the whiskers will extend to the minimum and maximum observed values up to 1.5 times the interquartile range from the top (bottom) of the box to the furthest datum within that distance. If there are any data beyond that distance, they are represented individually as points.
