# Supplementary material for: Automated mass spectrometry imaging of over 2000 proteins from tissue sections at 100-μm spatial resolution
Source: Nat Commun. 2020 Jan 7;11:8. doi: 10.1038/s41467-019-13858-z (PMC6946663; doi:10.1038/s41467-019-13858-z)
Supplement: Supplementary file 5 — Supplementary Data 1 [file 41467_2019_13858_MOESM5_ESM.pdf]

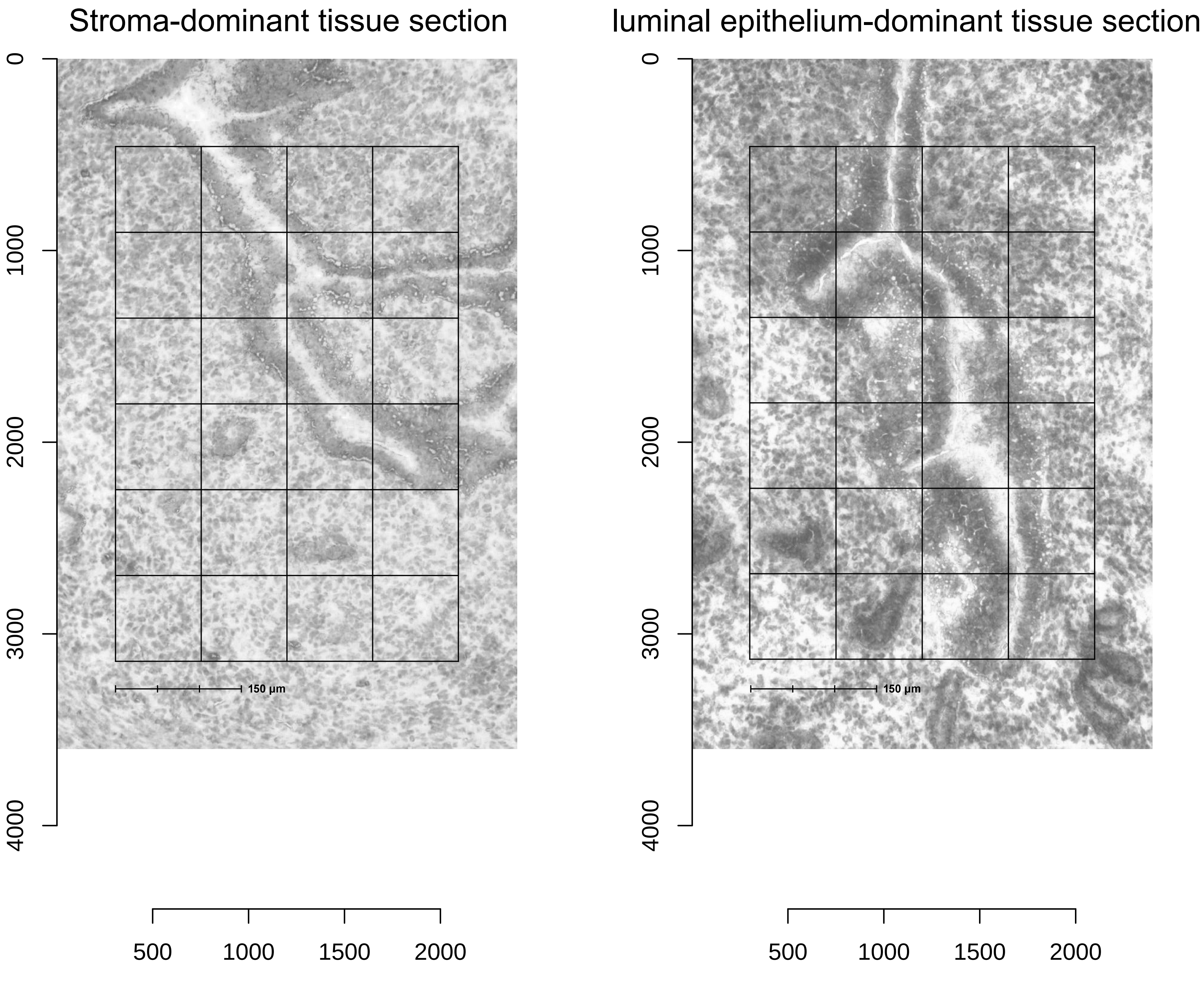

Stroma-dominant tissue section
luminal epithelium-dominant tissue section
0
1000
2000
3000
4000
150 µm
500
1000
1500
2000

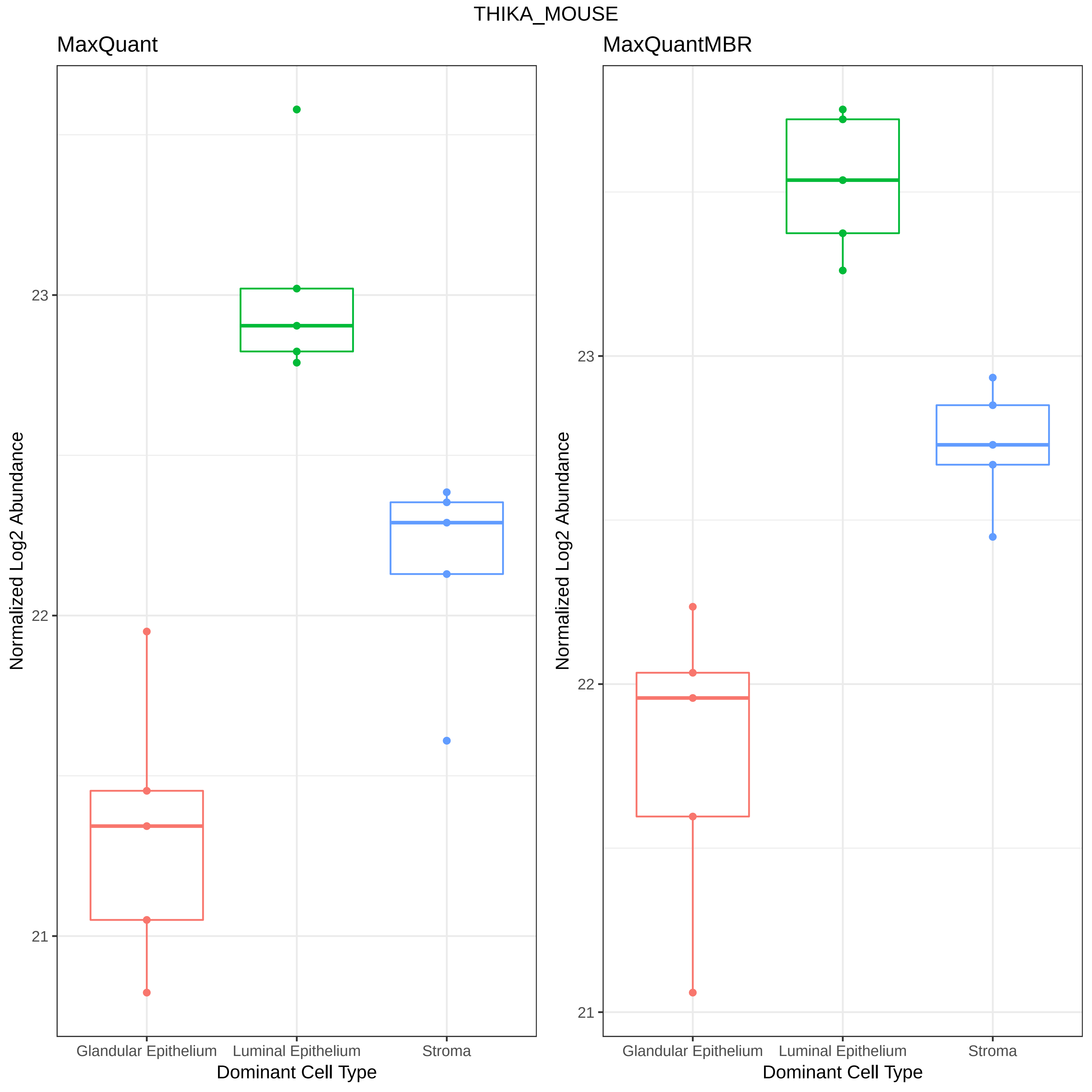
THIKA_MOUSE
MaxQuant
MaxQuantMBR
Normalized Log2 Abundance
23
22
21
Glandular Epithelium
Luminal Epithelium
Stroma
Dominant Cell Type

THIKA_MOUSE

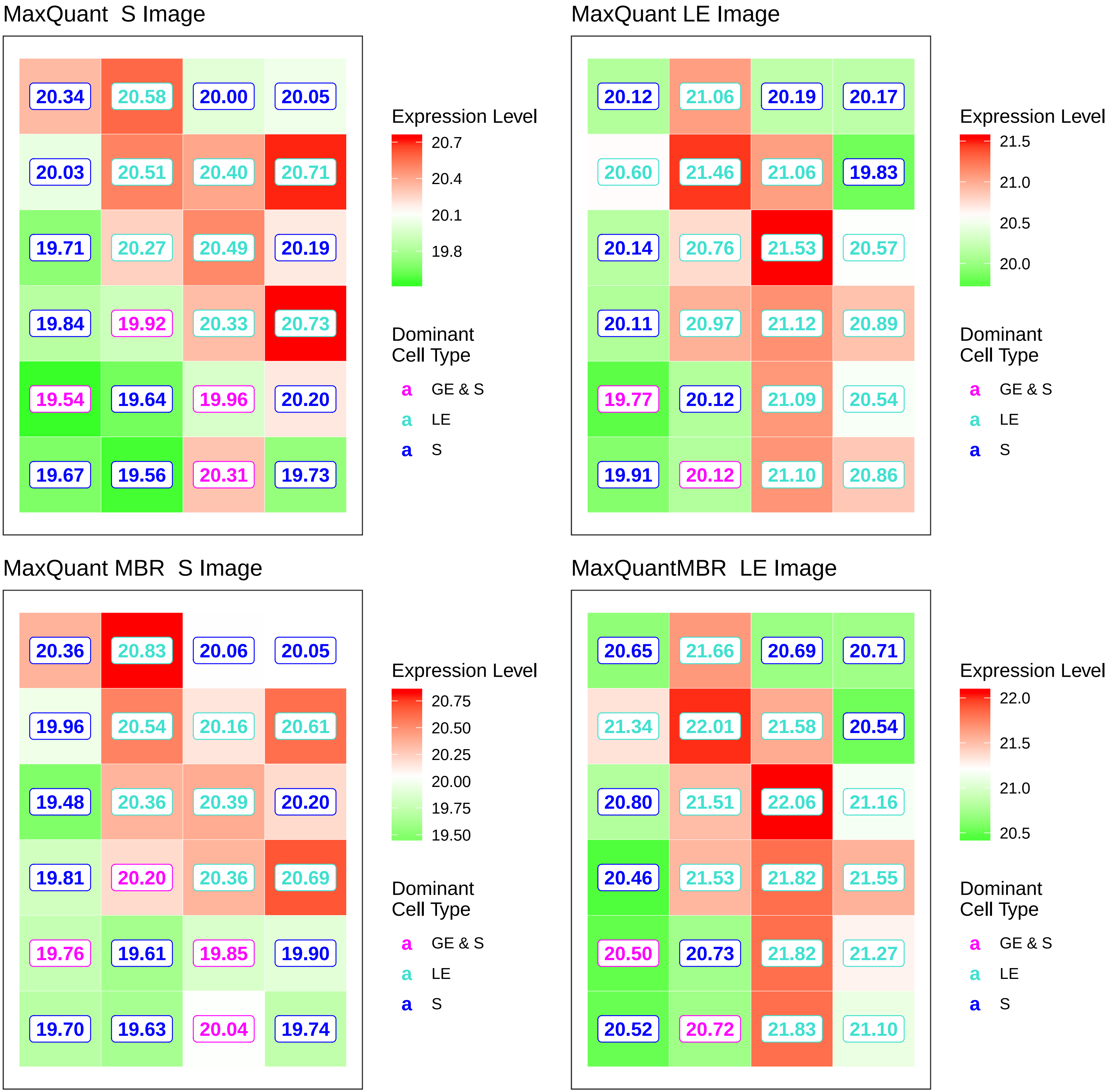
MaxQuant S Image
20.34
20.58
20.00
20.05
20.03
20.51
20.40
20.71
19.71
20.27
20.49
20.19
19.84
19.92
20.33
20.73
19.54
19.64
19.96
20.20
19.67
19.56
20.31
19.73
Expression Level
20.7
20.4
20.1
19.8
Dominant Cell Type
a GE & S
a LE
a S
MaxQuant LE Image
20.12
21.06
20.19
20.17
20.60
21.46
21.06
19.83
20.14
20.76
21.53
20.57
20.11
20.97
21.12
20.89
19.77
20.12
21.09
20.54
19.91
20.12
21.10
20.86
Expression Level
21.5
21.0
20.5
20.0
Dominant Cell Type
a GE & S
a LE
a S
MaxQuant MBR S Image
20.36
20.83
20.06
20.05
19.96
20.54
20.16
20.61
19.48
20.36
20.39
20.20
19.81
20.20
20.36
20.69
19.76
19.61
19.85
19.90
19.70
19.63
20.04
19.74
Expression Level
20.75
20.50
20.25
20.00
19.75
19.50
Dominant Cell Type
a GE & S
a LE
a S
MaxQuantMBR LE Image
20.65
21.66
20.69
20.71
21.34
22.01
21.58
20.54
20.80
21.51
22.06
21.16
20.46
21.53
21.82
21.55
20.50
20.73
21.82
21.27
20.52
20.72
21.83
21.10
Expression Level
22.0
21.5
21.0
20.5
Dominant Cell Type
a GE & S
a LE
a S

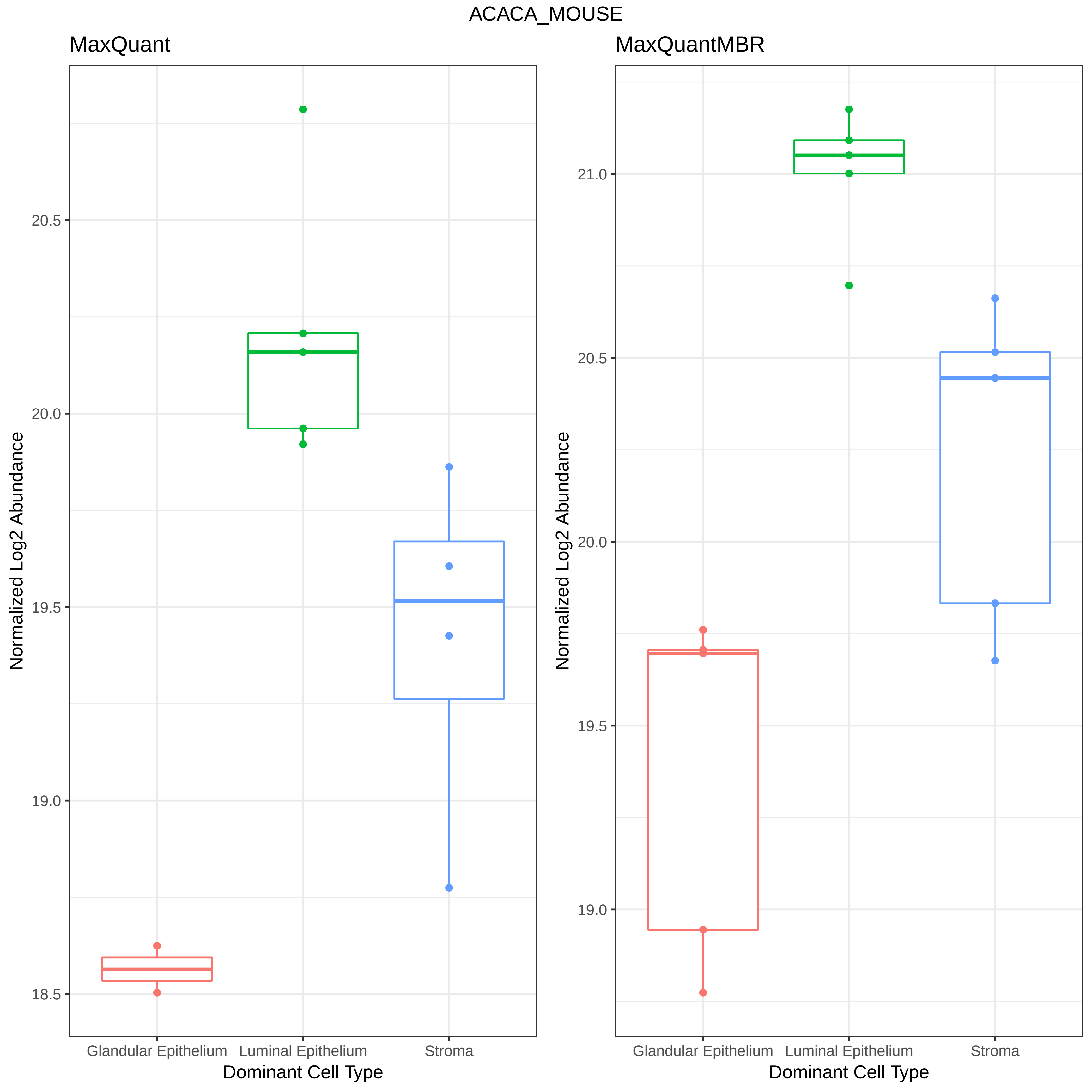
ACACA_MOUSE
MaxQuant
MaxQuantMBR
Normalized Log2 Abundance
20.5
20.0
19.5
19.0
18.5
21.0
Glandular Epithelium
Luminal Epithelium
Stroma
Dominant Cell Type

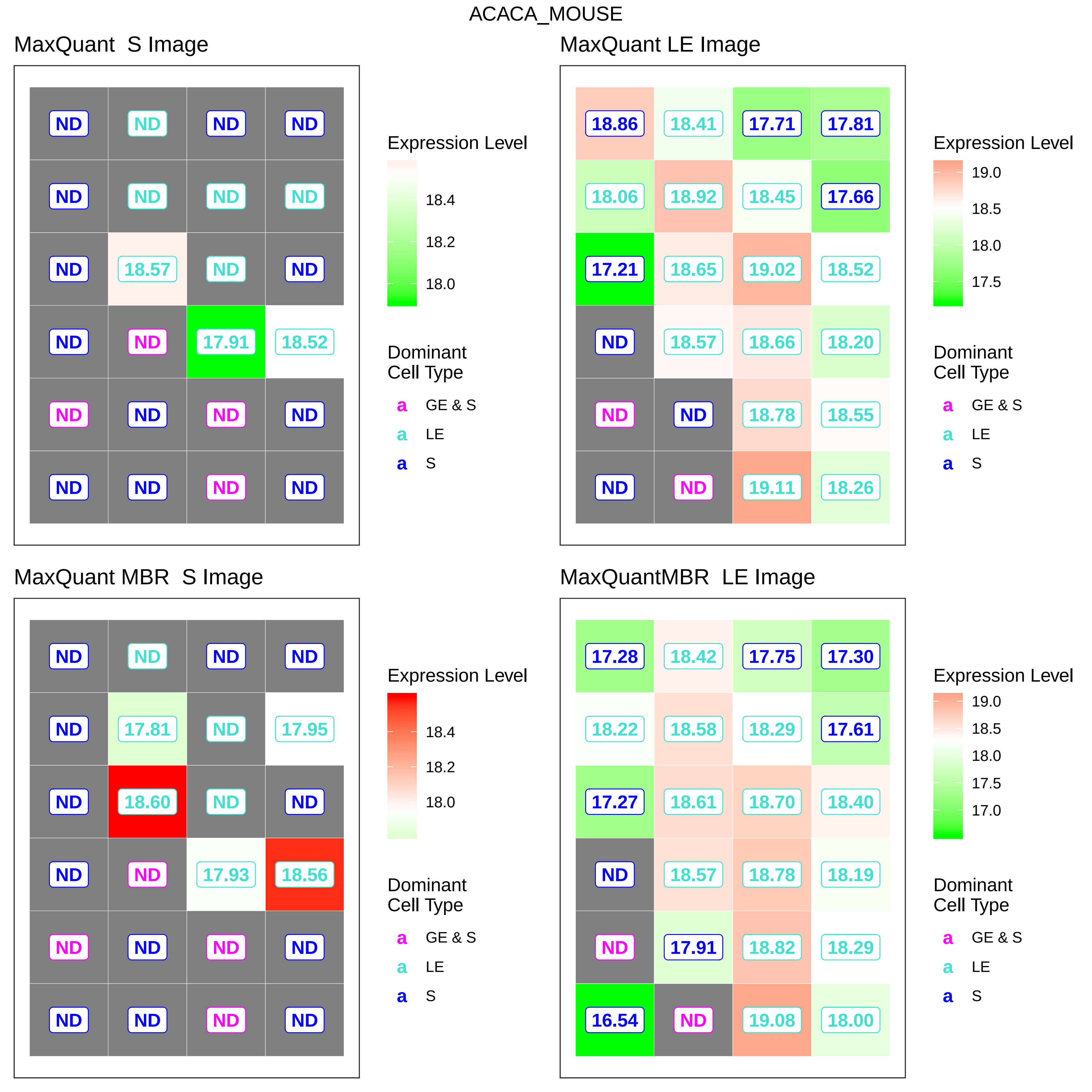
ACACA_MOUSE
MaxQuant S Image
MaxQuant LE Image
MaxQuant MBR S Image
MaxQuantMBR LE Image
Expression Level
Dominant Cell Type
GE & S
LE
S
ND
18.57
17.91
18.52
18.86
18.41
17.71
17.81
18.06
18.92
18.45
17.66
17.21
18.65
19.02
18.52
18.57
18.66
18.20
18.78
18.55
19.11
18.26
17.81
17.95
18.60
17.93
18.56
17.28
18.42
17.75
17.30
18.22
18.58
18.29
17.61
17.27
18.61
18.70
18.40
18.57
18.78
18.19
17.91
18.82
18.29
16.54
19.08
18.00

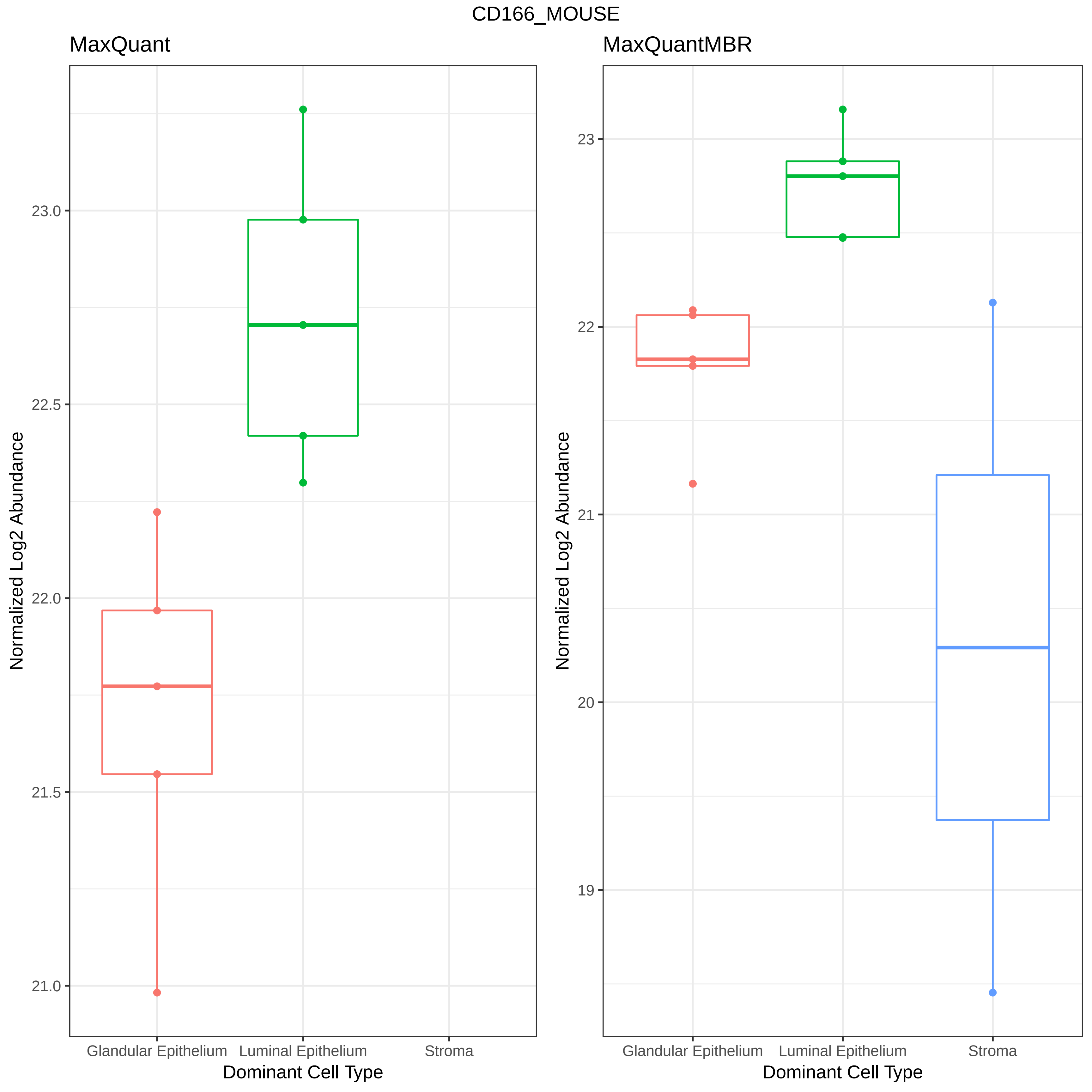
CD166_MOUSE
MaxQuant
MaxQuantMBR
Normalized Log2 Abundance
23.0
22.5
22.0
21.5
21.0
23
22
21
20
19
Glandular Epithelium
Luminal Epithelium
Stroma
Dominant Cell Type

CD166_MOUSE

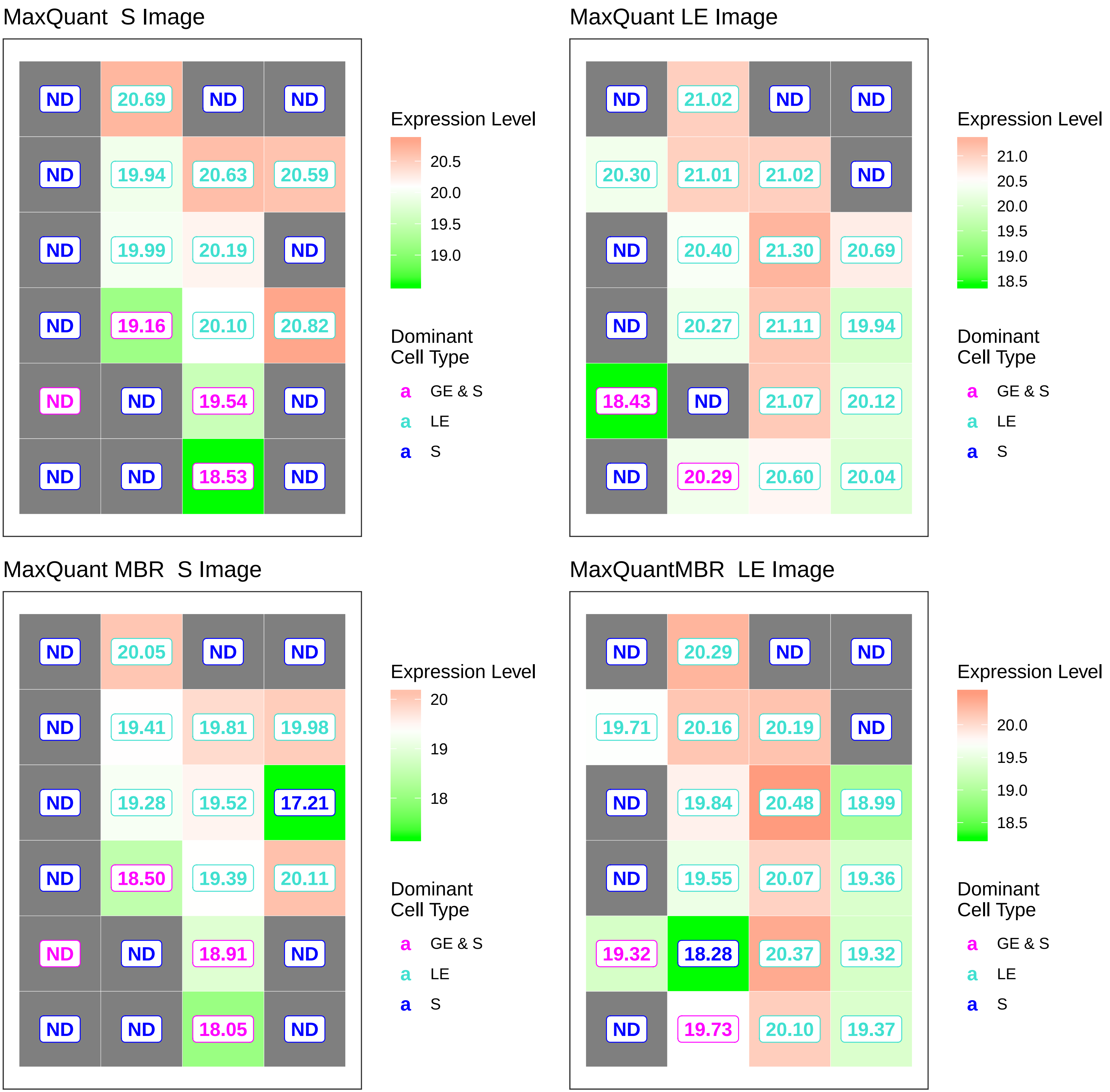
MaxQuant S Image
ND 20.69 ND ND
ND 19.94 20.63 20.59
ND 19.99 20.19 ND
ND 19.16 20.10 20.82
ND ND 19.54 ND
ND ND 18.53 ND
Expression Level
20.5
20.0
19.5
19.0
Dominant Cell Type
a GE & S
a LE
a S
MaxQuant LE Image
ND 21.02 ND ND
20.30 21.01 21.02 ND
ND 20.40 21.30 20.69
ND 20.27 21.11 19.94
18.43 ND 21.07 20.12
ND 20.29 20.60 20.04
Expression Level
21.0
20.5
20.0
19.5
19.0
18.5
Dominant Cell Type
a GE & S
a LE
a S
MaxQuant MBR S Image
ND 20.05 ND ND
ND 19.41 19.81 19.98
ND 19.28 19.52 17.21
ND 18.50 19.39 20.11
ND ND 18.91 ND
ND ND 18.05 ND
Expression Level
20
19
18
Dominant Cell Type
a GE & S
a LE
a S
MaxQuantMBR LE Image
ND 20.29 ND ND
19.71 20.16 20.19 ND
ND 19.84 20.48 18.99
ND 19.55 20.07 19.36
19.32 18.28 20.37 19.32
ND 19.73 20.10 19.37
Expression Level
20.0
19.5
19.0
18.5
Dominant Cell Type
a GE & S
a LE
a S

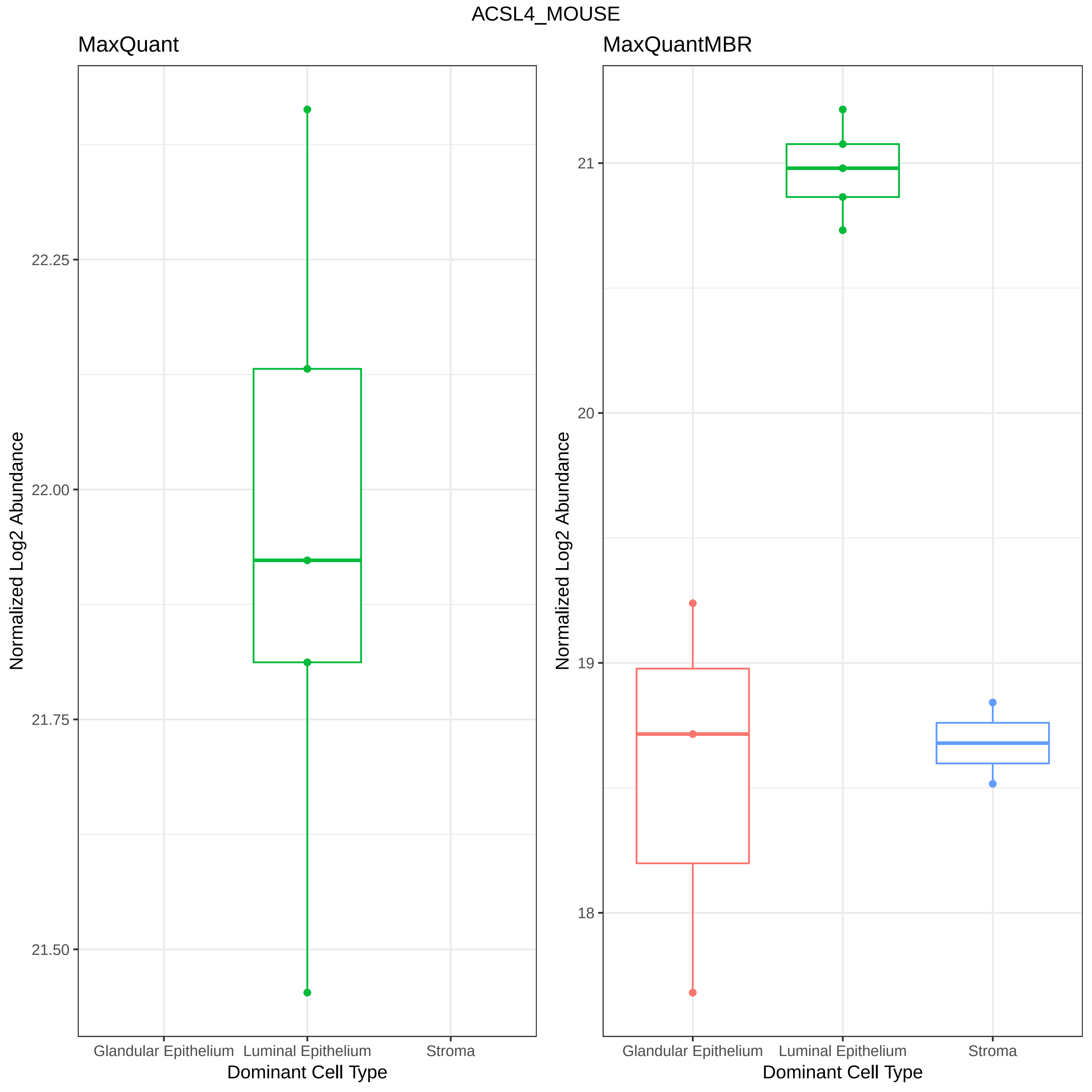
ACSL4_MOUSE
MaxQuant
MaxQuantMBR
Normalized Log2 Abundance
22.25
22.00
21.75
21.50
21
20
19
18
Glandular Epithelium
Luminal Epithelium
Stroma
Dominant Cell Type

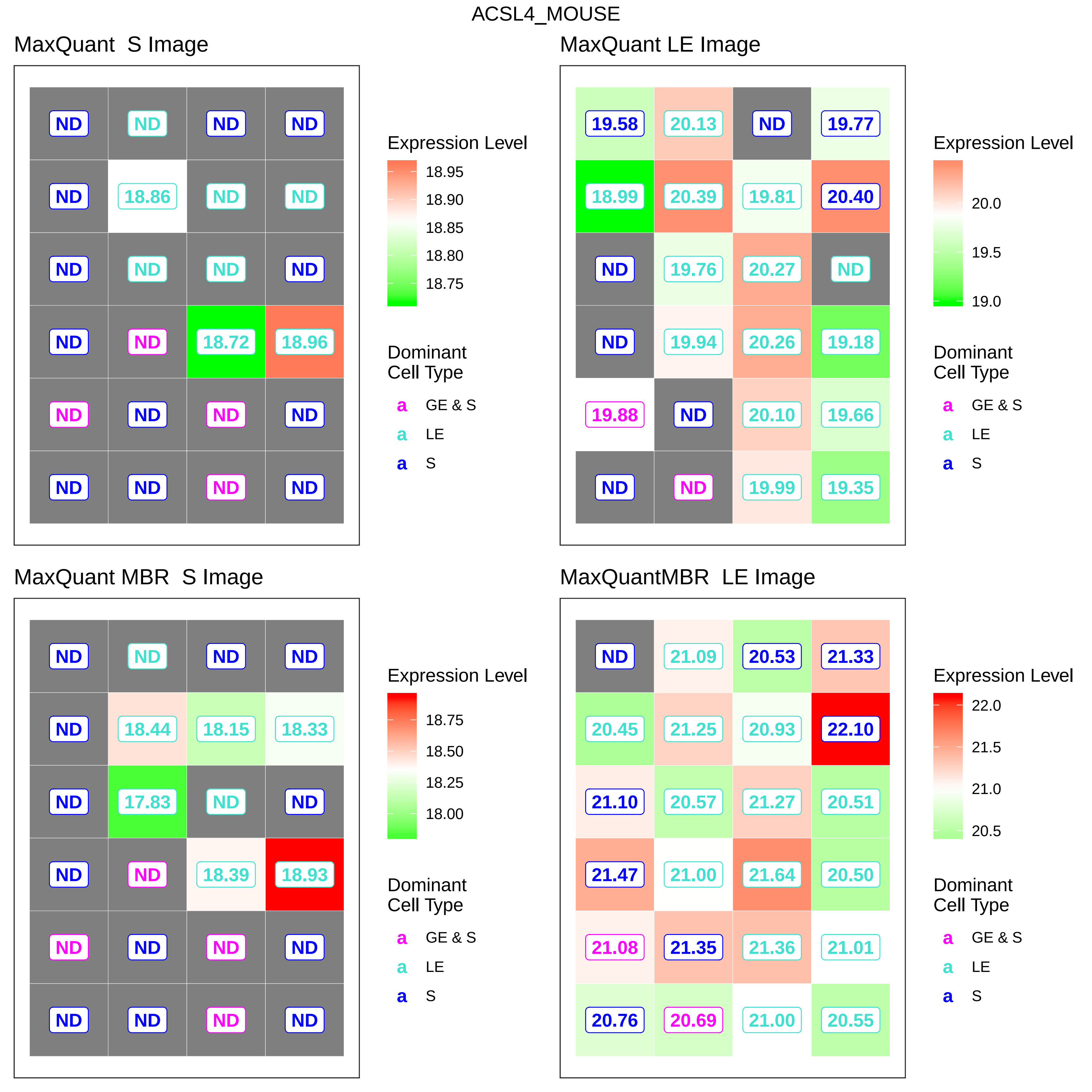
ACSL4_MOUSE
MaxQuant S Image
ND ND ND ND
ND 18.86 ND ND
ND ND ND ND
ND ND 18.72 18.96
ND ND ND ND
ND ND ND ND
Expression Level
18.95
18.90
18.85
18.80
18.75
Dominant Cell Type
a GE & S
a LE
a S
MaxQuant LE Image
19.58 20.13 ND 19.77
18.99 20.39 19.81 20.40
ND 19.76 20.27 ND
ND 19.94 20.26 19.18
19.88 ND 20.10 19.66
ND ND 19.99 19.35
Expression Level
20.0
19.5
19.0
Dominant Cell Type
a GE & S
a LE
a S
MaxQuant MBR S Image
ND ND ND ND
ND 18.44 18.15 18.33
ND 17.83 ND ND
ND ND 18.39 18.93
ND ND ND ND
ND ND ND ND
Expression Level
18.75
18.50
18.25
18.00
Dominant Cell Type
a GE & S
a LE
a S
MaxQuantMBR LE Image
ND 21.09 20.53 21.33
20.45 21.25 20.93 22.10
21.10 20.57 21.27 20.51
21.47 21.00 21.64 20.50
21.08 21.35 21.36 21.01
20.76 20.69 21.00 20.55
Expression Level
22.0
21.5
21.0
20.5
Dominant Cell Type
a GE & S
a LE
a S

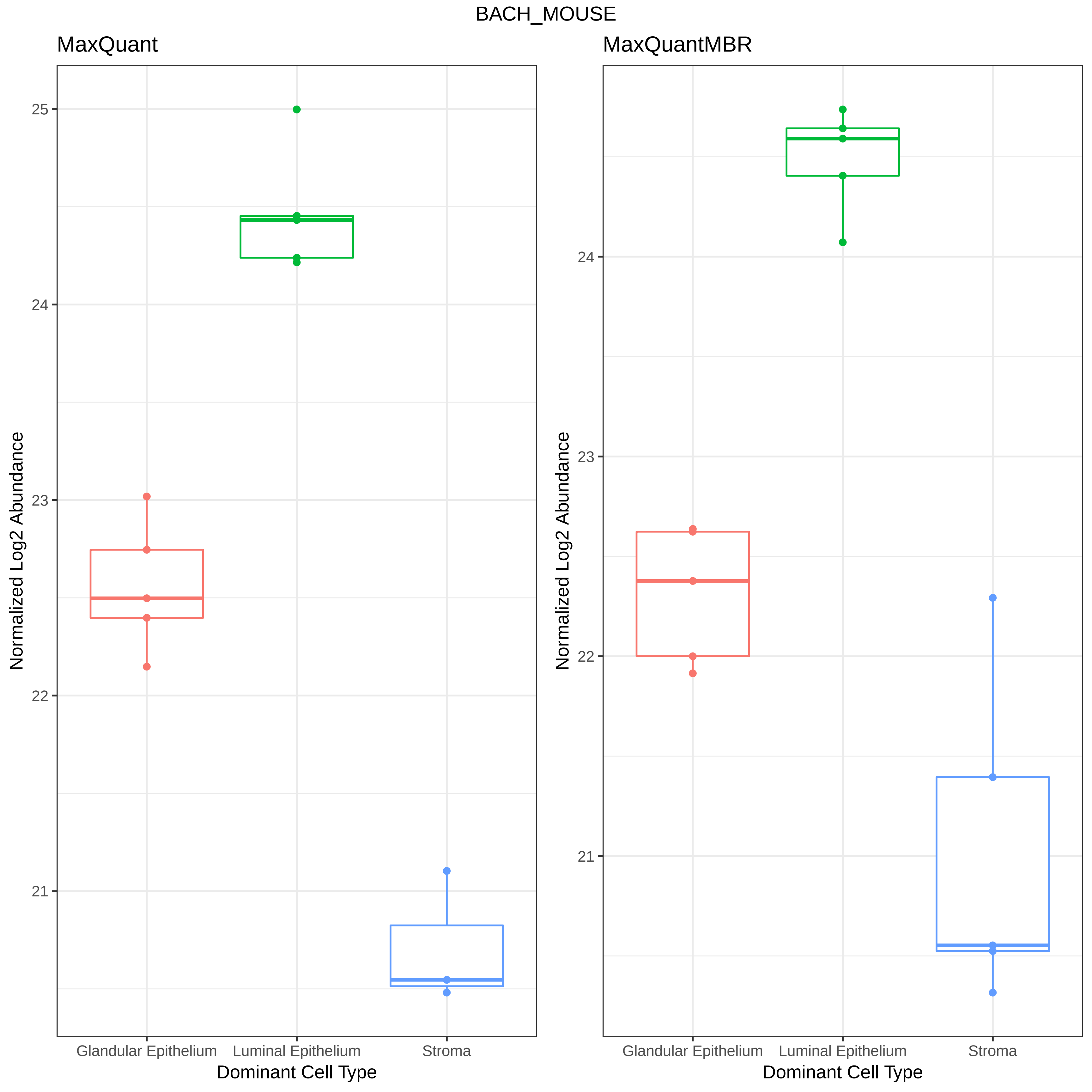
BACH_MOUSE
MaxQuant
MaxQuantMBR
Normalized Log2 Abundance
25
24
23
22
21
Glandular Epithelium
Luminal Epithelium
Stroma
Dominant Cell Type

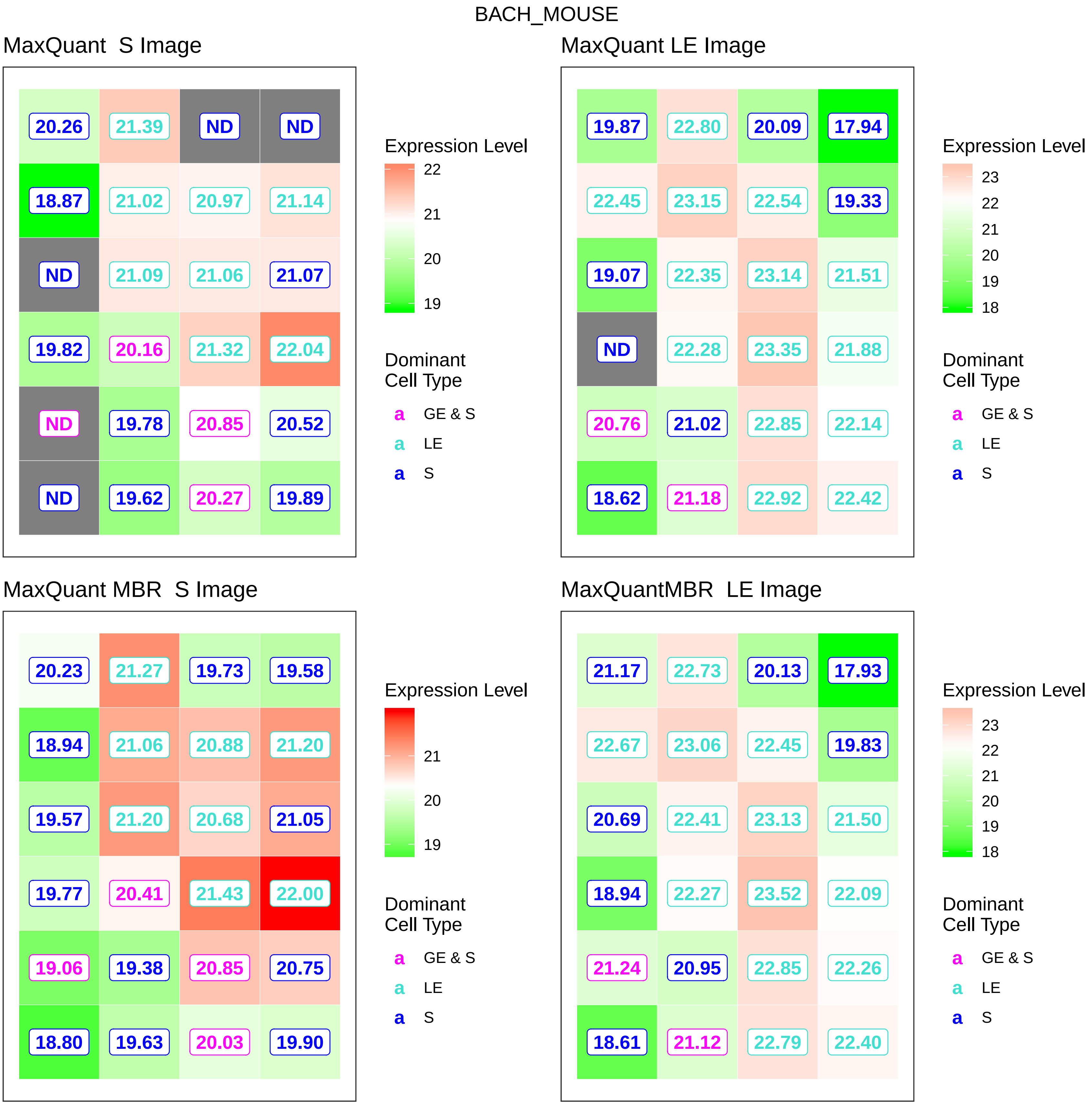
BACH_MOUSE
MaxQuant S Image
20.26 21.39 ND ND
18.87 21.02 20.97 21.14
ND 21.09 21.06 21.07
19.82 20.16 21.32 22.04
ND 19.78 20.85 20.52
ND 19.62 20.27 19.89
Expression Level
22
21
20
19
Dominant Cell Type
a GE & S
a LE
a S
MaxQuant LE Image
19.87 22.80 20.09 17.94
22.45 23.15 22.54 19.33
19.07 22.35 23.14 21.51
ND 22.28 23.35 21.88
20.76 21.02 22.85 22.14
18.62 21.18 22.92 22.42
Expression Level
23
22
21
20
19
18
Dominant Cell Type
a GE & S
a LE
a S
MaxQuant MBR S Image
20.23 21.27 19.73 19.58
18.94 21.06 20.88 21.20
19.57 21.20 20.68 21.05
19.77 20.41 21.43 22.00
19.06 19.38 20.85 20.75
18.80 19.63 20.03 19.90
Expression Level
21
20
19
Dominant Cell Type
a GE & S
a LE
a S
MaxQuantMBR LE Image
21.17 22.73 20.13 17.93
22.67 23.06 22.45 19.83
20.69 22.41 23.13 21.50
18.94 22.27 23.52 22.09
21.24 20.95 22.85 22.26
18.61 21.12 22.79 22.40
Expression Level
23
22
21
20
19
18
Dominant Cell Type
a GE & S
a LE
a S

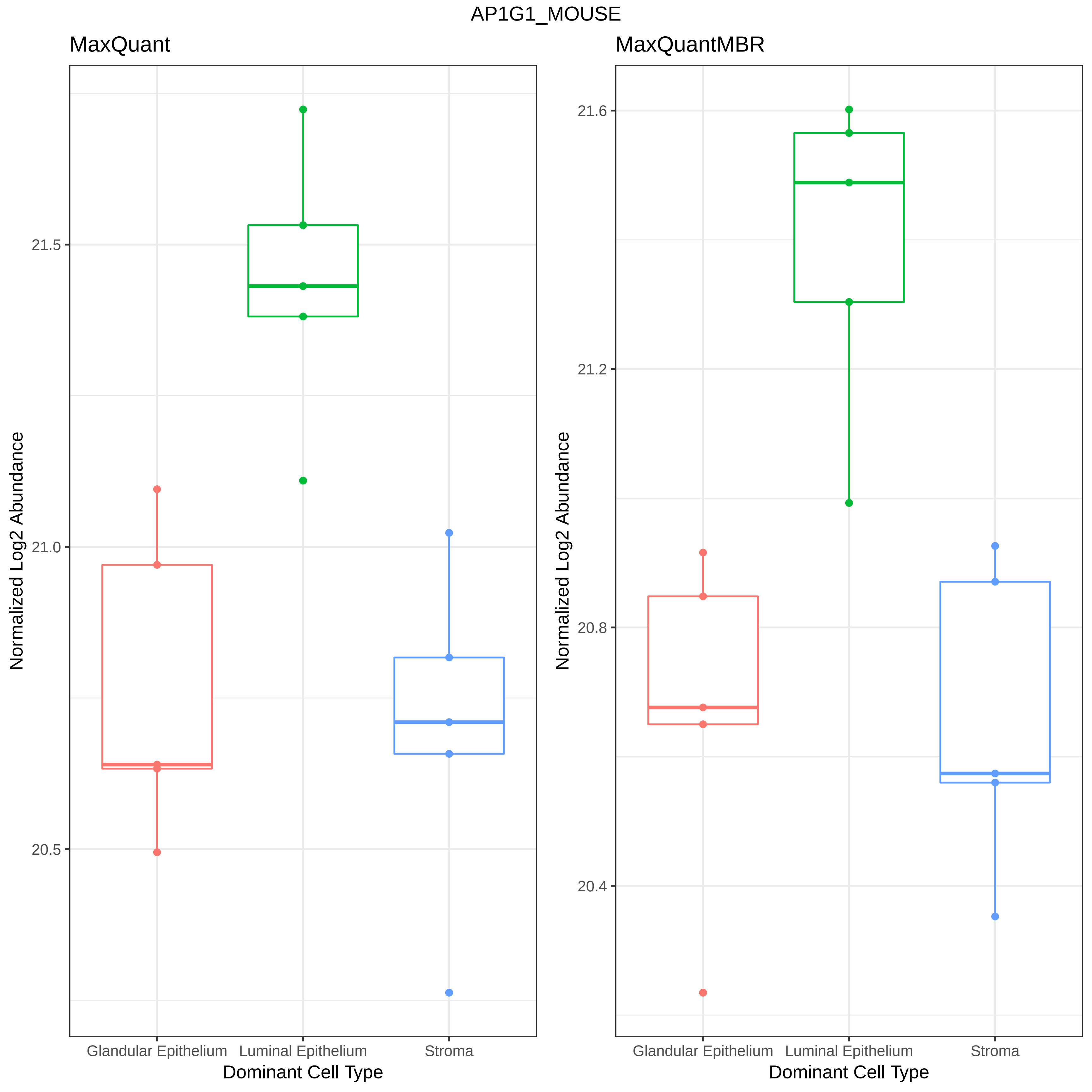
AP1G1_MOUSE
MaxQuant
MaxQuantMBR
Normalized Log2 Abundance
21.5
21.0
20.5
21.6
21.2
20.8
20.4
Glandular Epithelium
Luminal Epithelium
Stroma
Dominant Cell Type

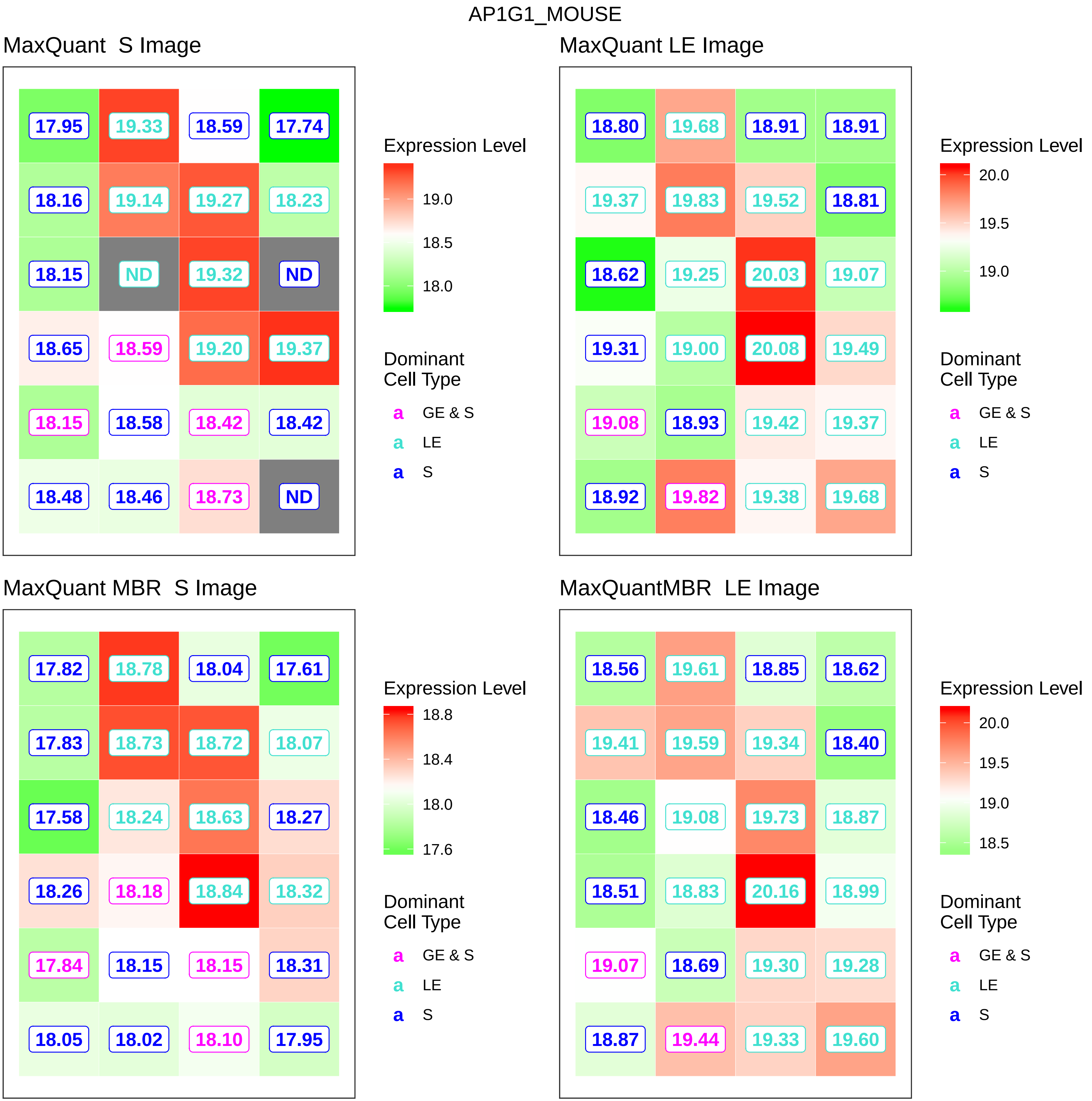
AP1G1_MOUSE
MaxQuant S Image
17.95 19.33 18.59 17.74
18.16 19.14 19.27 18.23
18.15 ND 19.32 ND
18.65 18.59 19.20 19.37
18.15 18.58 18.42 18.42
18.48 18.46 18.73 ND
Expression Level
19.0
18.5
18.0
Dominant Cell Type
a GE & S
a LE
a S
MaxQuant LE Image
18.80 19.68 18.91 18.91
19.37 19.83 19.52 18.81
18.62 19.25 20.03 19.07
19.31 19.00 20.08 19.49
19.08 18.93 19.42 19.37
18.92 19.82 19.38 19.68
Expression Level
20.0
19.5
19.0
Dominant Cell Type
a GE & S
a LE
a S
MaxQuant MBR S Image
17.82 18.78 18.04 17.61
17.83 18.73 18.72 18.07
17.58 18.24 18.63 18.27
18.26 18.18 18.84 18.32
17.84 18.15 18.15 18.31
18.05 18.02 18.10 17.95
Expression Level
18.8
18.4
18.0
17.6
Dominant Cell Type
a GE & S
a LE
a S
MaxQuantMBR LE Image
18.56 19.61 18.85 18.62
19.41 19.59 19.34 18.40
18.46 19.08 19.73 18.87
18.51 18.83 20.16 18.99
19.07 18.69 19.30 19.28
18.87 19.44 19.33 19.60
Expression Level
20.0
19.5
19.0
18.5
Dominant Cell Type
a GE & S
a LE
a S

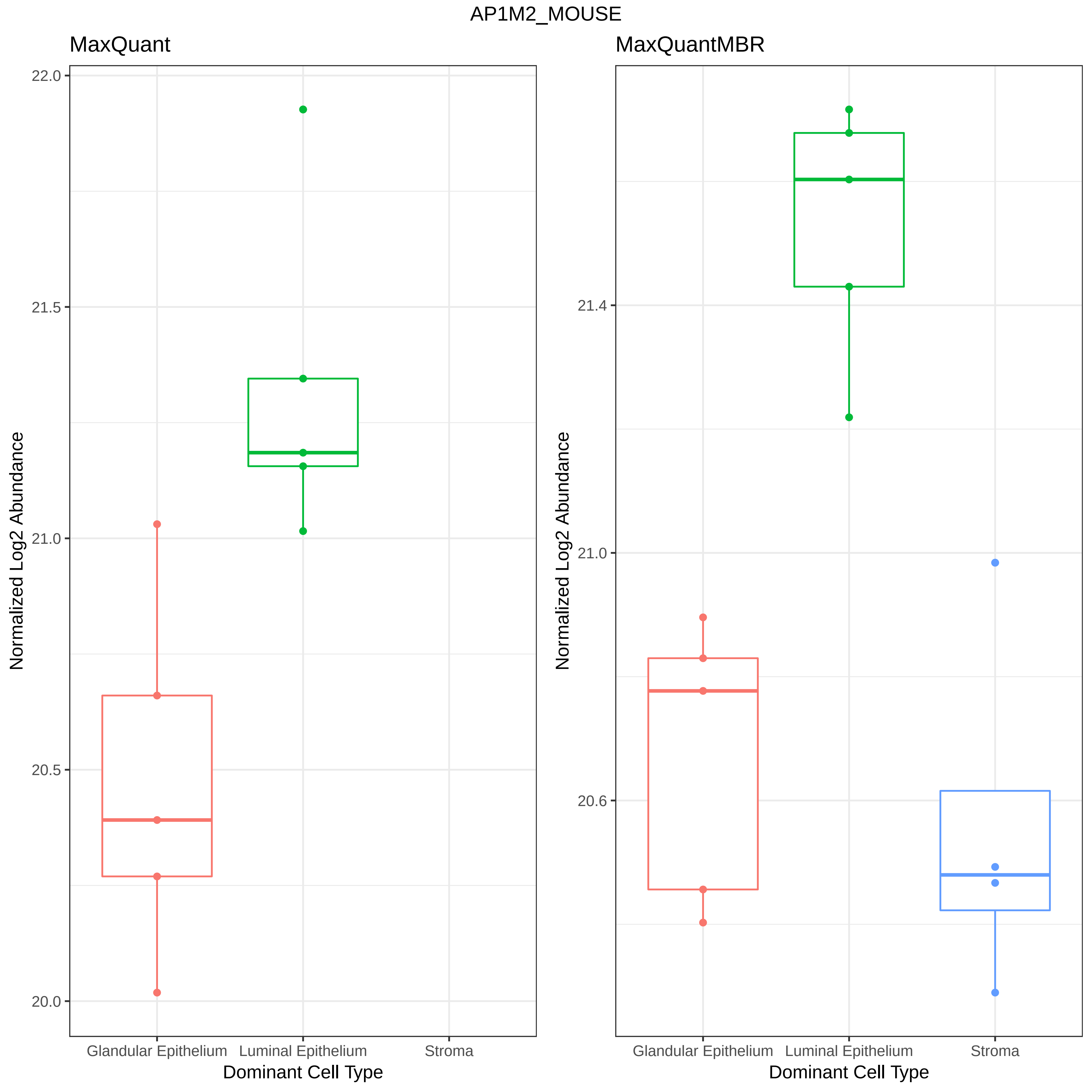
AP1M2_MOUSE
MaxQuant
MaxQuantMBR
Normalized Log2 Abundance
22.0
21.5
21.0
20.5
20.0
21.4
21.0
20.6
Glandular Epithelium
Luminal Epithelium
Stroma
Dominant Cell Type

AP1M2_MOUSE

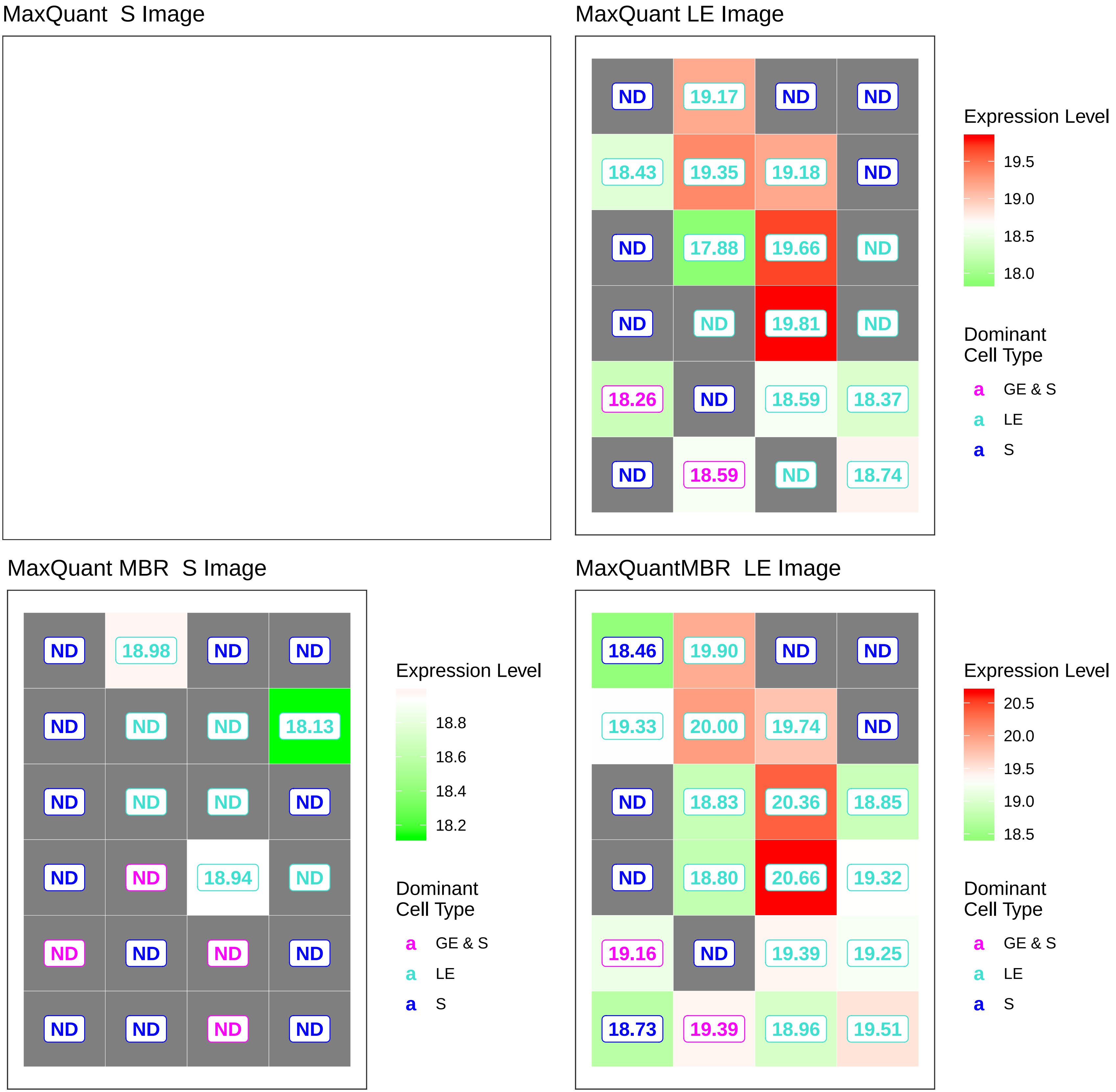

MaxQuant S Image
MaxQuant LE Image
ND
19.17
ND
ND
18.43
19.35
19.18
ND
ND
17.88
19.66
ND
ND
ND
19.81
ND
18.26
ND
18.59
18.37
ND
18.59
ND
18.74
Expression Level
19.5
19.0
18.5
18.0
Dominant Cell Type
a GE & S
a LE
a S
MaxQuant MBR S Image
ND
18.98
ND
ND
ND
ND
ND
18.13
ND
ND
ND
ND
ND
ND
18.94
ND
ND
ND
ND
ND
ND
ND
ND
ND
Expression Level
18.8
18.6
18.4
18.2
Dominant Cell Type
a GE & S
a LE
a S
MaxQuantMBR LE Image
18.46
19.90
ND
ND
19.33
20.00
19.74
ND
ND
18.83
20.36
18.85
ND
18.80
20.66
19.32
19.16
ND
19.39
19.25
18.73
19.39
18.96
19.51
Expression Level
20.5
20.0
19.5
19.0
18.5
Dominant Cell Type
a GE & S
a LE
a S

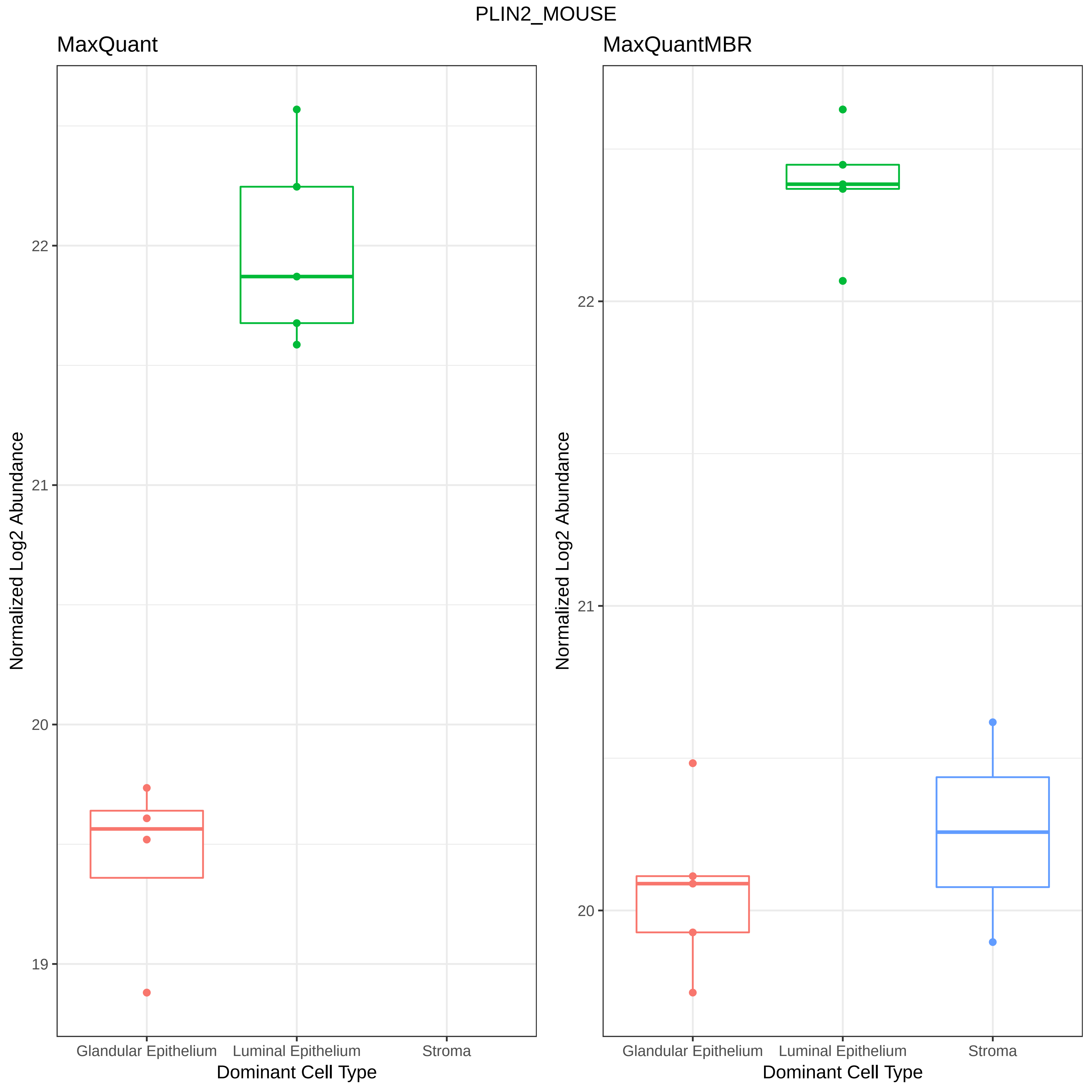

PLIN2_MOUSE
MaxQuant
MaxQuantMBR
Normalized Log2 Abundance
22
21
20
19
Glandular Epithelium
Luminal Epithelium
Stroma
Dominant Cell Type

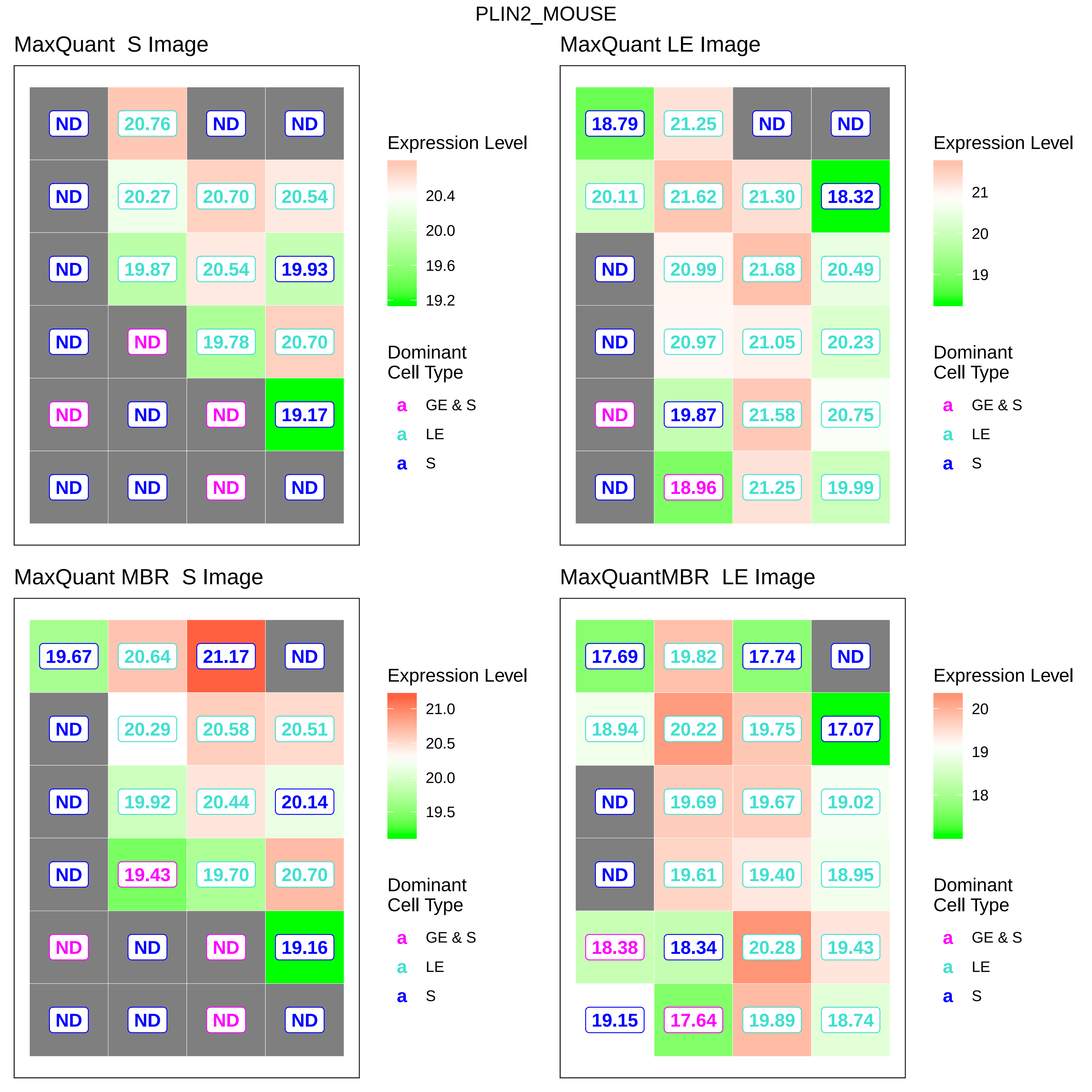
PLIN2_MOUSE
MaxQuant S Image
ND 20.76 ND ND
ND 20.27 20.70 20.54
ND 19.87 20.54 19.93
ND ND 19.78 20.70
ND ND ND 19.17
ND ND ND ND
Expression Level
20.4
20.0
19.6
19.2
Dominant Cell Type
a GE & S
a LE
a S
MaxQuant LE Image
18.79 21.25 ND ND
20.11 21.62 21.30 18.32
ND 20.99 21.68 20.49
ND 20.97 21.05 20.23
ND 19.87 21.58 20.75
ND 18.96 21.25 19.99
Expression Level
21
20
19
Dominant Cell Type
a GE & S
a LE
a S
MaxQuant MBR S Image
19.67 20.64 21.17 ND
ND 20.29 20.58 20.51
ND 19.92 20.44 20.14
ND 19.43 19.70 20.70
ND ND ND 19.16
ND ND ND ND
Expression Level
21.0
20.5
20.0
19.5
Dominant Cell Type
a GE & S
a LE
a S
MaxQuantMBR LE Image
17.69 19.82 17.74 ND
18.94 20.22 19.75 17.07
ND 19.69 19.67 19.02
ND 19.61 19.40 18.95
18.38 18.34 20.28 19.43
19.15 17.64 19.89 18.74
Expression Level
20
19
18
Dominant Cell Type
a GE & S
a LE
a S

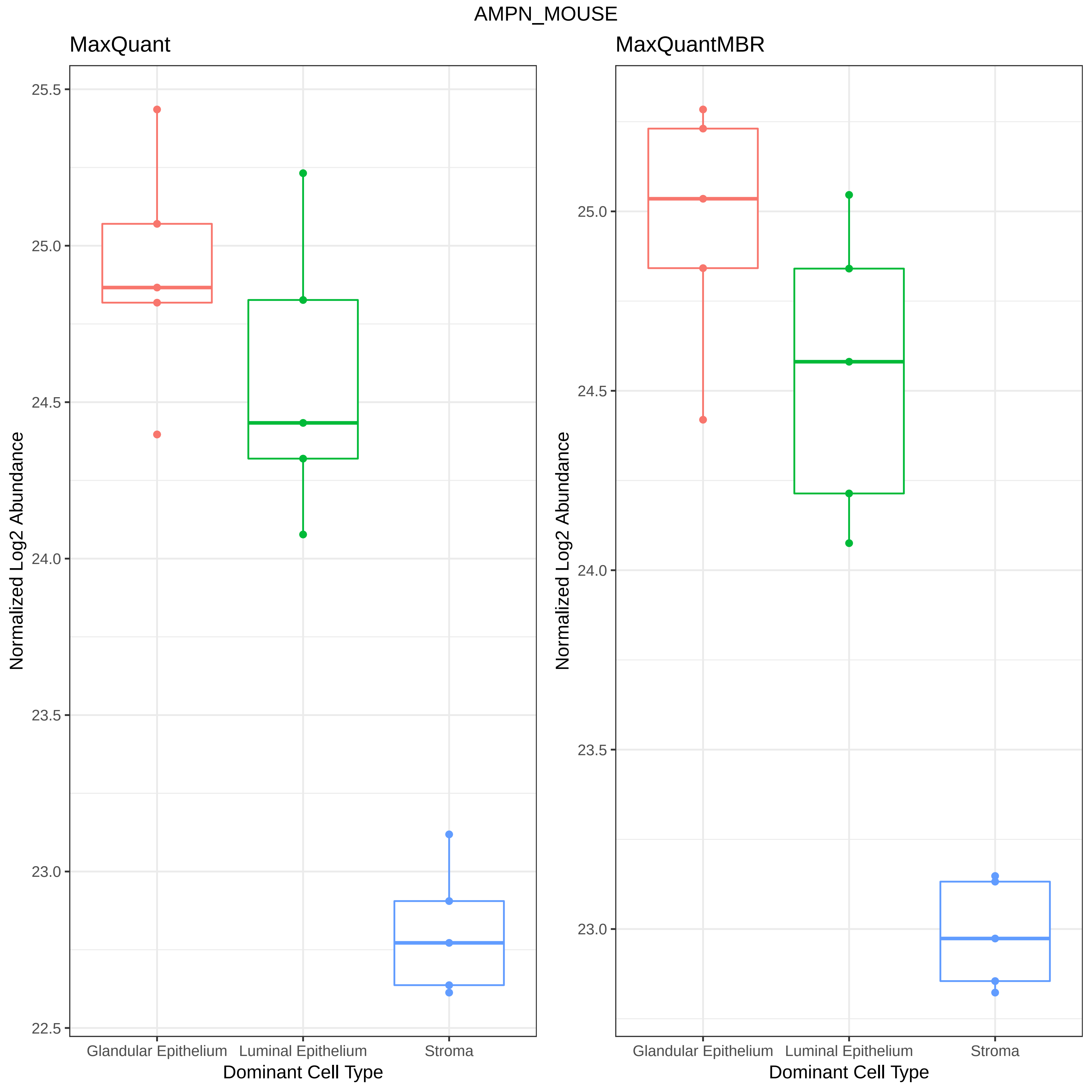
AMPN_MOUSE
MaxQuant
MaxQuantMBR
Normalized Log2 Abundance
25.5
25.0
24.5
24.0
23.5
23.0
22.5
Glandular Epithelium
Luminal Epithelium
Stroma
Dominant Cell Type

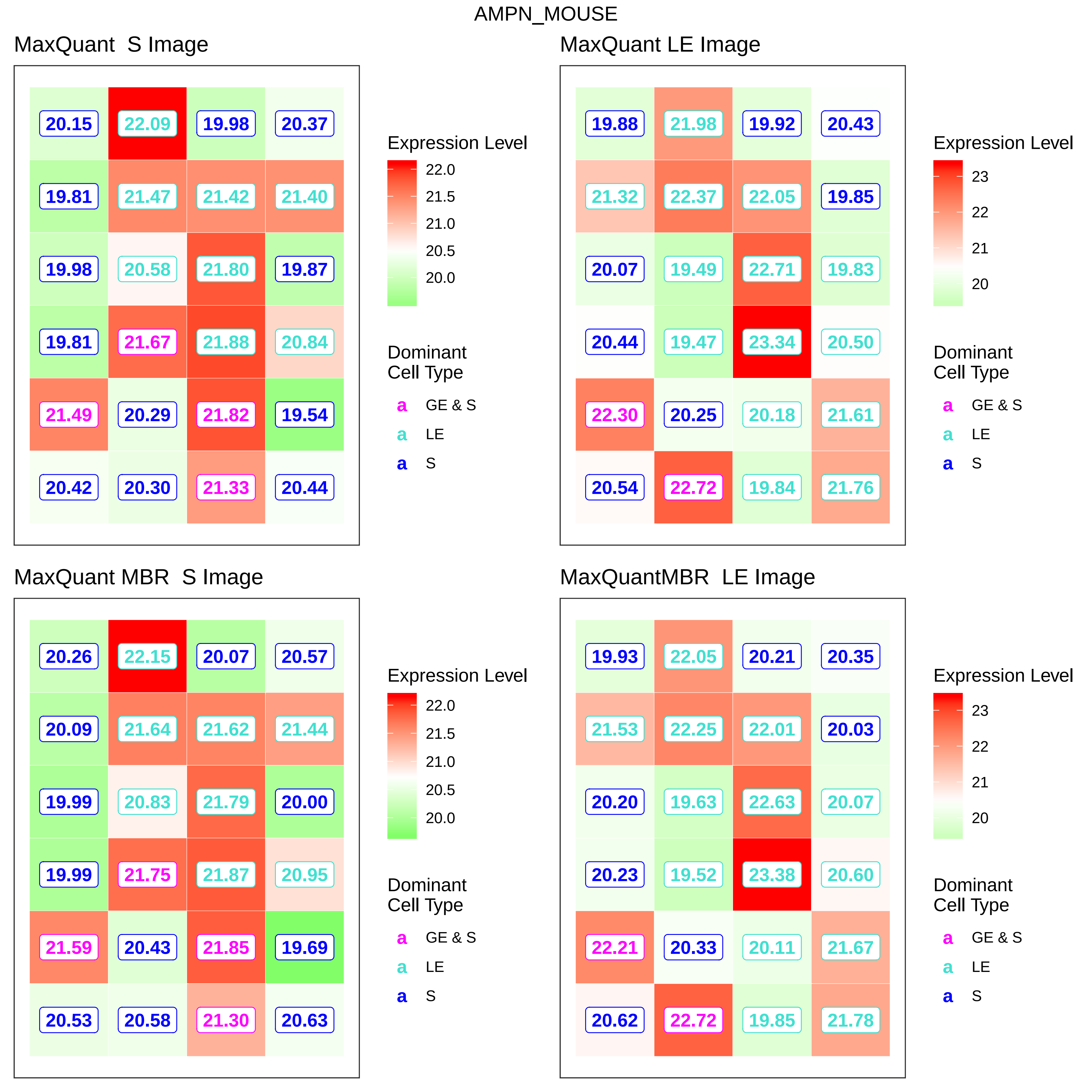
AMPN_MOUSE
MaxQuant S Image
20.15 22.09 19.98 20.37
19.81 21.47 21.42 21.40
19.98 20.58 21.80 19.87
19.81 21.67 21.88 20.84
21.49 20.29 21.82 19.54
20.42 20.30 21.33 20.44
Expression Level
22.0
21.5
21.0
20.5
20.0
Dominant Cell Type
a GE & S
a LE
a S
MaxQuant LE Image
19.88 21.98 19.92 20.43
21.32 22.37 22.05 19.85
20.07 19.49 22.71 19.83
20.44 19.47 23.34 20.50
22.30 20.25 20.18 21.61
20.54 22.72 19.84 21.76
Expression Level
23
22
21
20
Dominant Cell Type
a GE & S
a LE
a S
MaxQuant MBR S Image
20.26 22.15 20.07 20.57
20.09 21.64 21.62 21.44
19.99 20.83 21.79 20.00
19.99 21.75 21.87 20.95
21.59 20.43 21.85 19.69
20.53 20.58 21.30 20.63
Expression Level
22.0
21.5
21.0
20.5
20.0
Dominant Cell Type
a GE & S
a LE
a S
MaxQuantMBR LE Image
19.93 22.05 20.21 20.35
21.53 22.25 22.01 20.03
20.20 19.63 22.63 20.07
20.23 19.52 23.38 20.60
22.21 20.33 20.11 21.67
20.62 22.72 19.85 21.78
Expression Level
23
22
21
20
Dominant Cell Type
a GE & S
a LE
a S

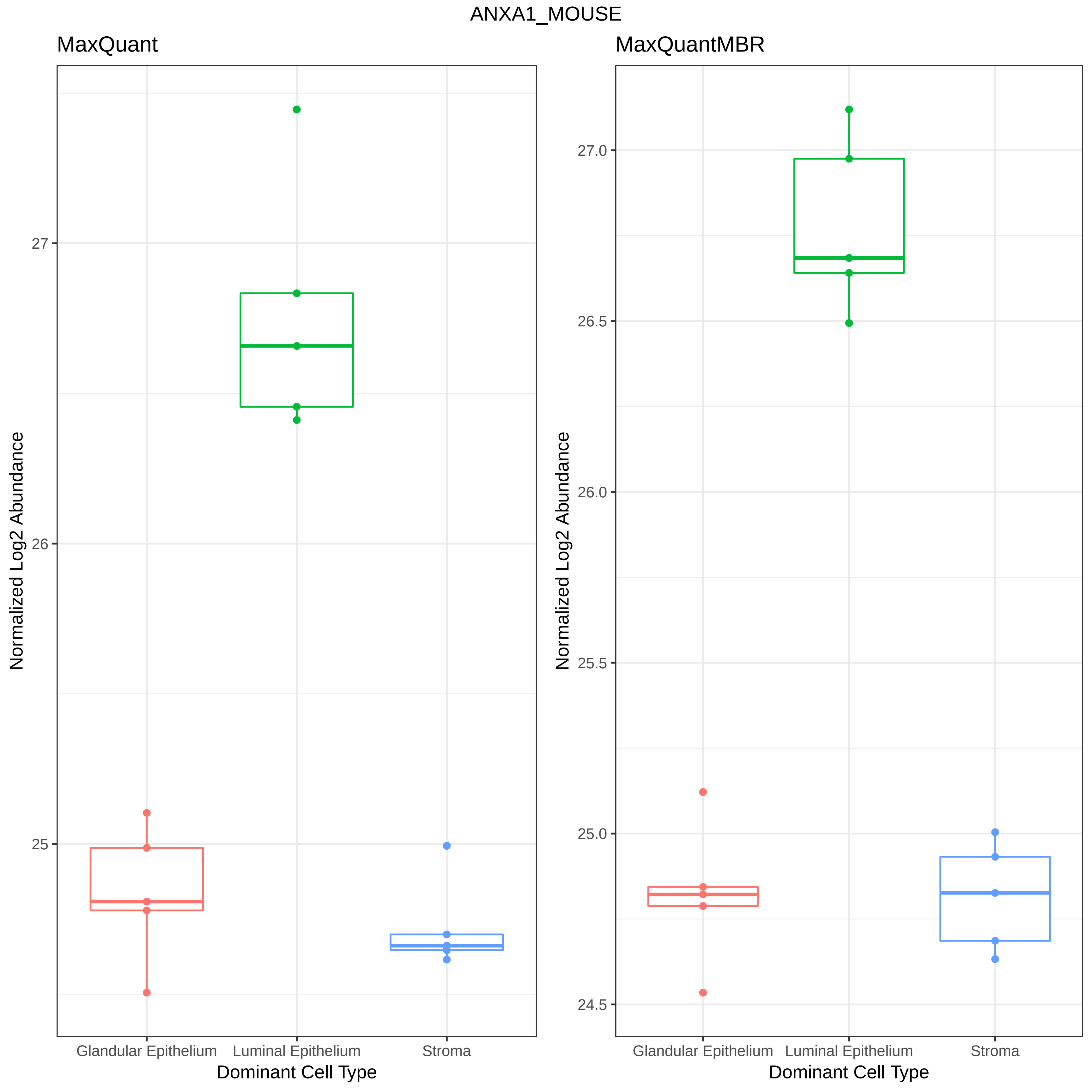
ANXA1_MOUSE
MaxQuant
MaxQuantMBR
Normalized Log2 Abundance
27
26
25
27.0
26.5
26.0
25.5
25.0
24.5
Glandular Epithelium
Luminal Epithelium
Stroma
Dominant Cell Type

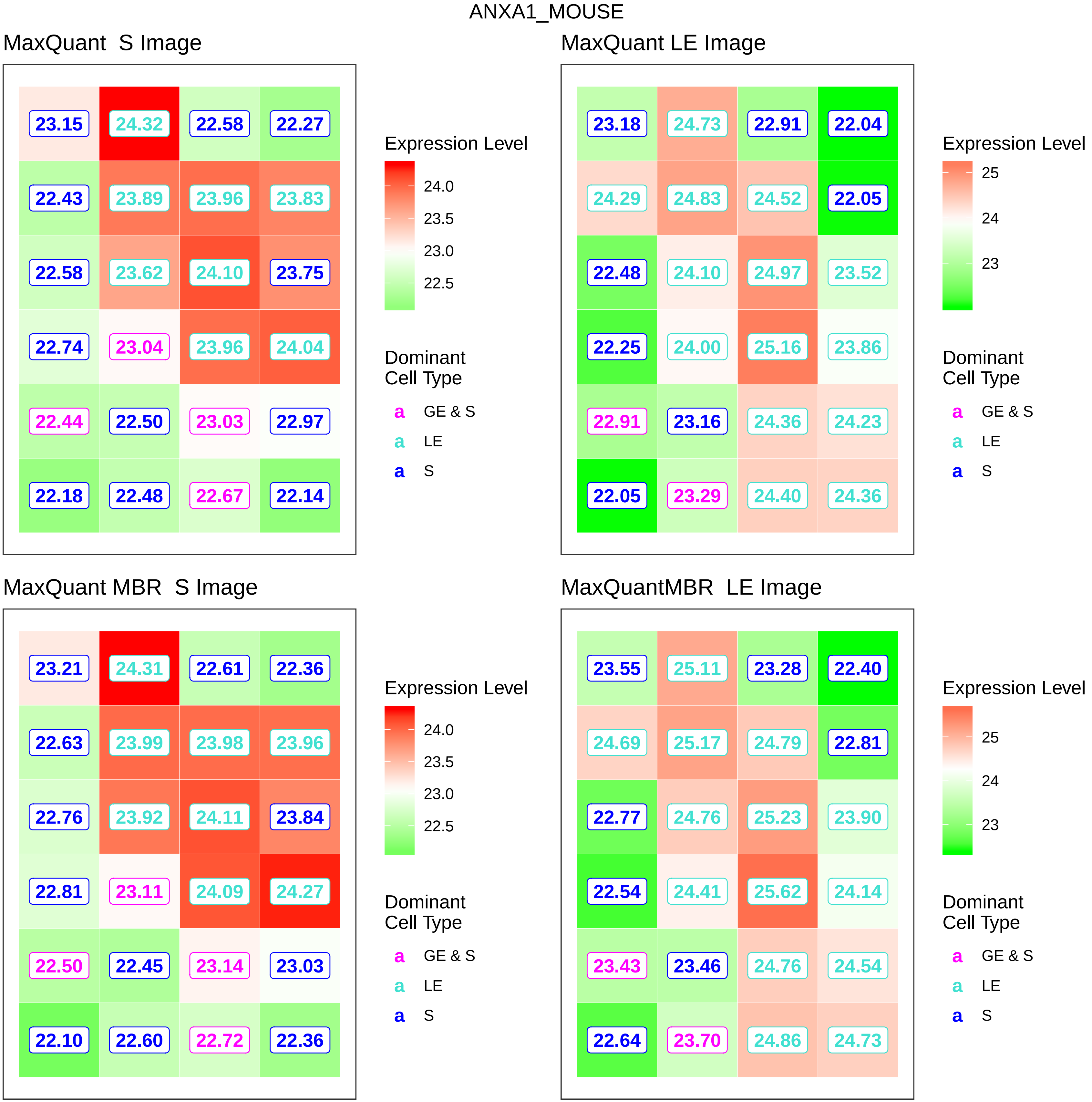

ANXA1_MOUSE
MaxQuant S Image
23.15 24.32 22.58 22.27
22.43 23.89 23.96 23.83
22.58 23.62 24.10 23.75
22.74 23.04 23.96 24.04
22.44 22.50 23.03 22.97
22.18 22.48 22.67 22.14
Expression Level
24.0
23.5
23.0
22.5
Dominant Cell Type
a GE & S
a LE
a S
MaxQuant LE Image
23.18 24.73 22.91 22.04
24.29 24.83 24.52 22.05
22.48 24.10 24.97 23.52
22.25 24.00 25.16 23.86
22.91 23.16 24.36 24.23
22.05 23.29 24.40 24.36
Expression Level
25
24
23
Dominant Cell Type
a GE & S
a LE
a S
MaxQuant MBR S Image
23.21 24.31 22.61 22.36
22.63 23.99 23.98 23.96
22.76 23.92 24.11 23.84
22.81 23.11 24.09 24.27
22.50 22.45 23.14 23.03
22.10 22.60 22.72 22.36
Expression Level
24.0
23.5
23.0
22.5
Dominant Cell Type
a GE & S
a LE
a S
MaxQuantMBR LE Image
23.55 25.11 23.28 22.40
24.69 25.17 24.79 22.81
22.77 24.76 25.23 23.90
22.54 24.41 25.62 24.14
23.43 23.46 24.76 24.54
22.64 23.70 24.86 24.73
Expression Level
25
24
23
Dominant Cell Type
a GE & S
a LE
a S

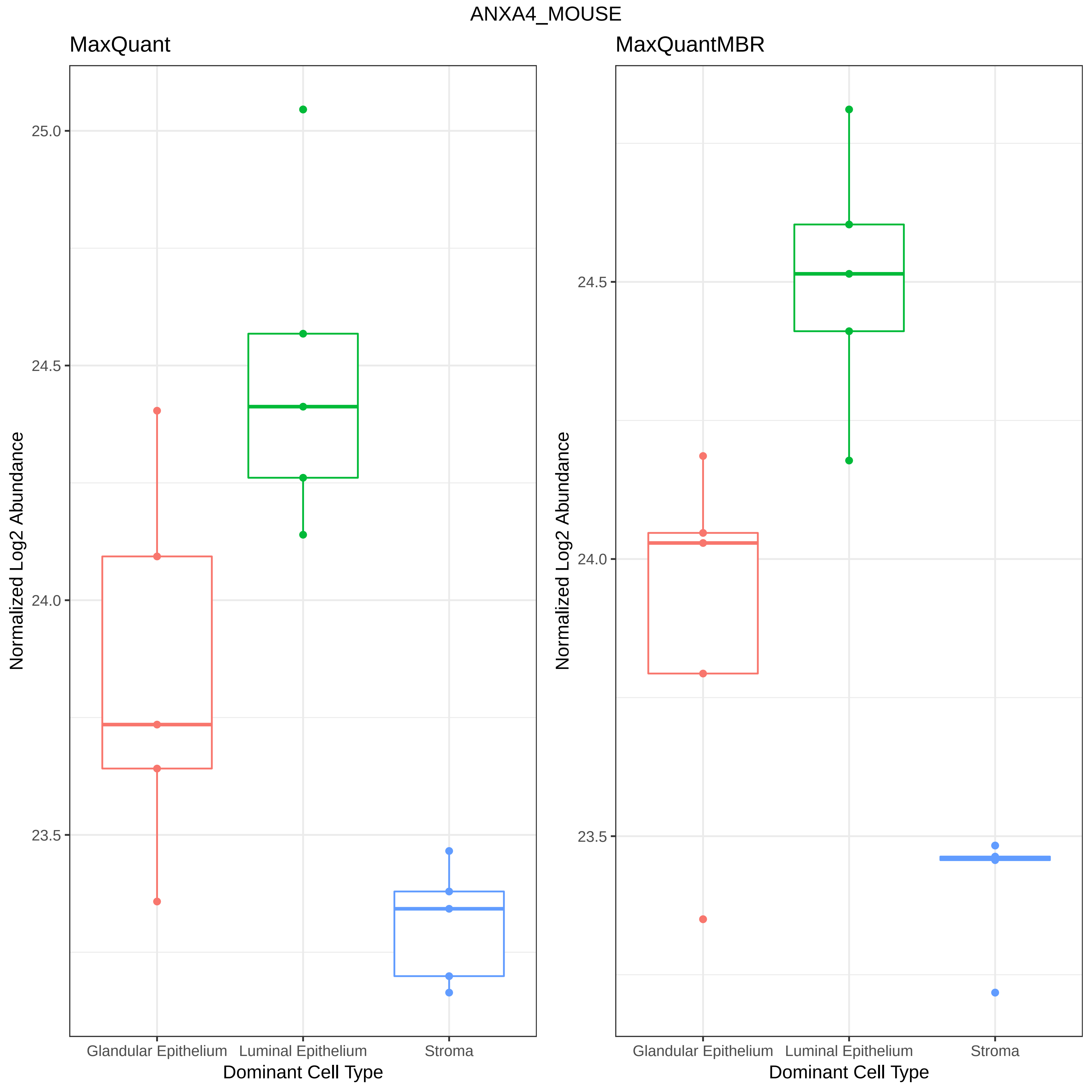
ANXA4_MOUSE
MaxQuant
MaxQuantMBR
Normalized Log2 Abundance
25.0
24.5
24.0
23.5
Glandular Epithelium
Luminal Epithelium
Stroma
Dominant Cell Type

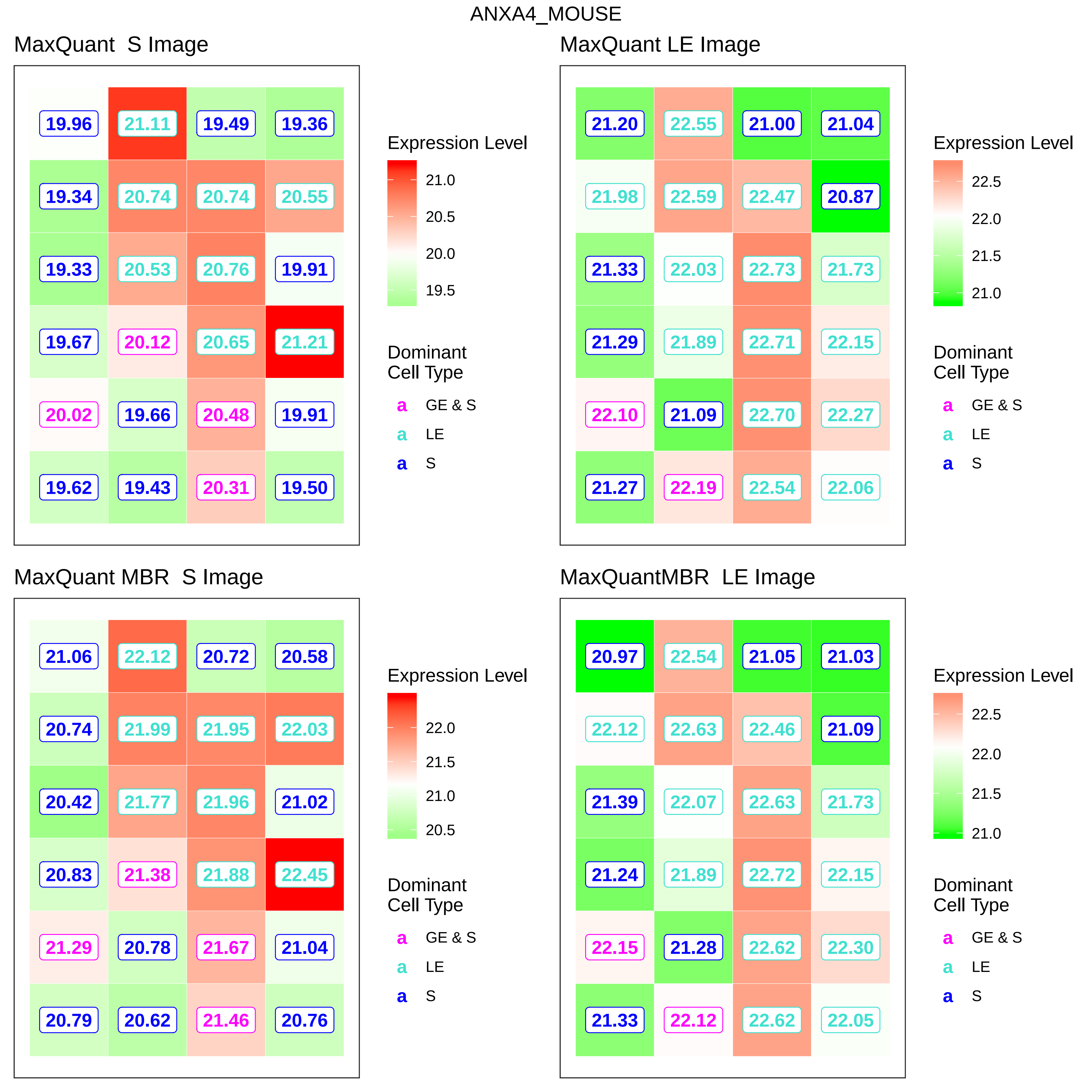
ANXA4_MOUSE
MaxQuant S Image
19.96
21.11
19.49
19.36
19.34
20.74
20.74
20.55
19.33
20.53
20.76
19.91
19.67
20.12
20.65
21.21
20.02
19.66
20.48
19.91
19.62
19.43
20.31
19.50
Expression Level
21.0
20.5
20.0
19.5
Dominant Cell Type
a GE & S
a LE
a S
MaxQuant LE Image
21.20
22.55
21.00
21.04
21.98
22.59
22.47
20.87
21.33
22.03
22.73
21.73
21.29
21.89
22.71
22.15
22.10
21.09
22.70
22.27
21.27
22.19
22.54
22.06
Expression Level
22.5
22.0
21.5
21.0
Dominant Cell Type
a GE & S
a LE
a S
MaxQuant MBR S Image
21.06
22.12
20.72
20.58
20.74
21.99
21.95
22.03
20.42
21.77
21.96
21.02
20.83
21.38
21.88
22.45
21.29
20.78
21.67
21.04
20.79
20.62
21.46
20.76
Expression Level
22.0
21.5
21.0
20.5
Dominant Cell Type
a GE & S
a LE
a S
MaxQuantMBR LE Image
20.97
22.54
21.05
21.03
22.12
22.63
22.46
21.09
21.39
22.07
22.63
21.73
21.24
21.89
22.72
22.15
22.15
21.28
22.62
22.30
21.33
22.12
22.62
22.05
Expression Level
22.5
22.0
21.5
21.0
Dominant Cell Type
a GE & S
a LE
a S

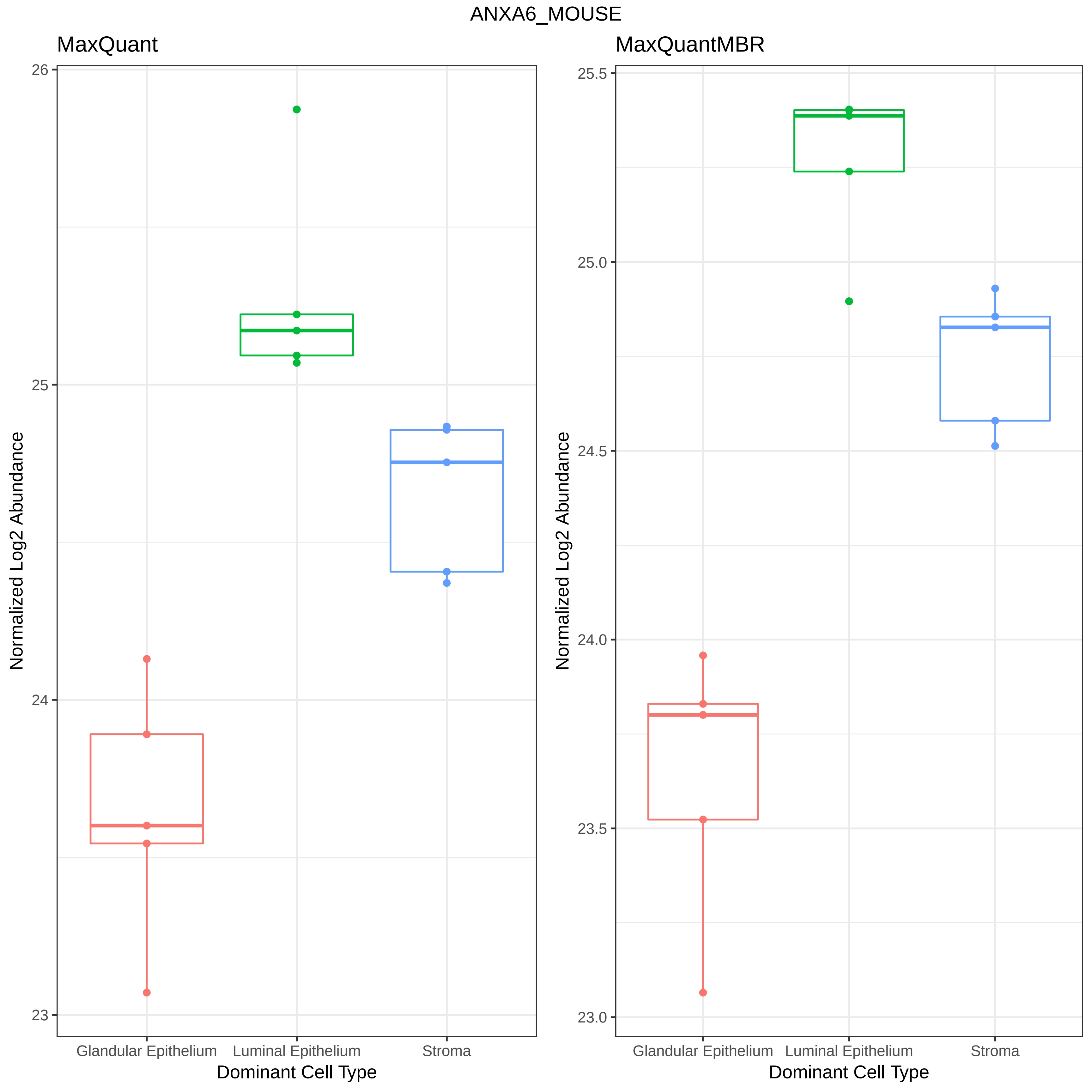

ANXA6_MOUSE
MaxQuant
MaxQuantMBR
Normalized Log2 Abundance
26
25
24
23
25.5
25.0
24.5
24.0
23.5
23.0
Glandular Epithelium
Luminal Epithelium
Stroma
Dominant Cell Type

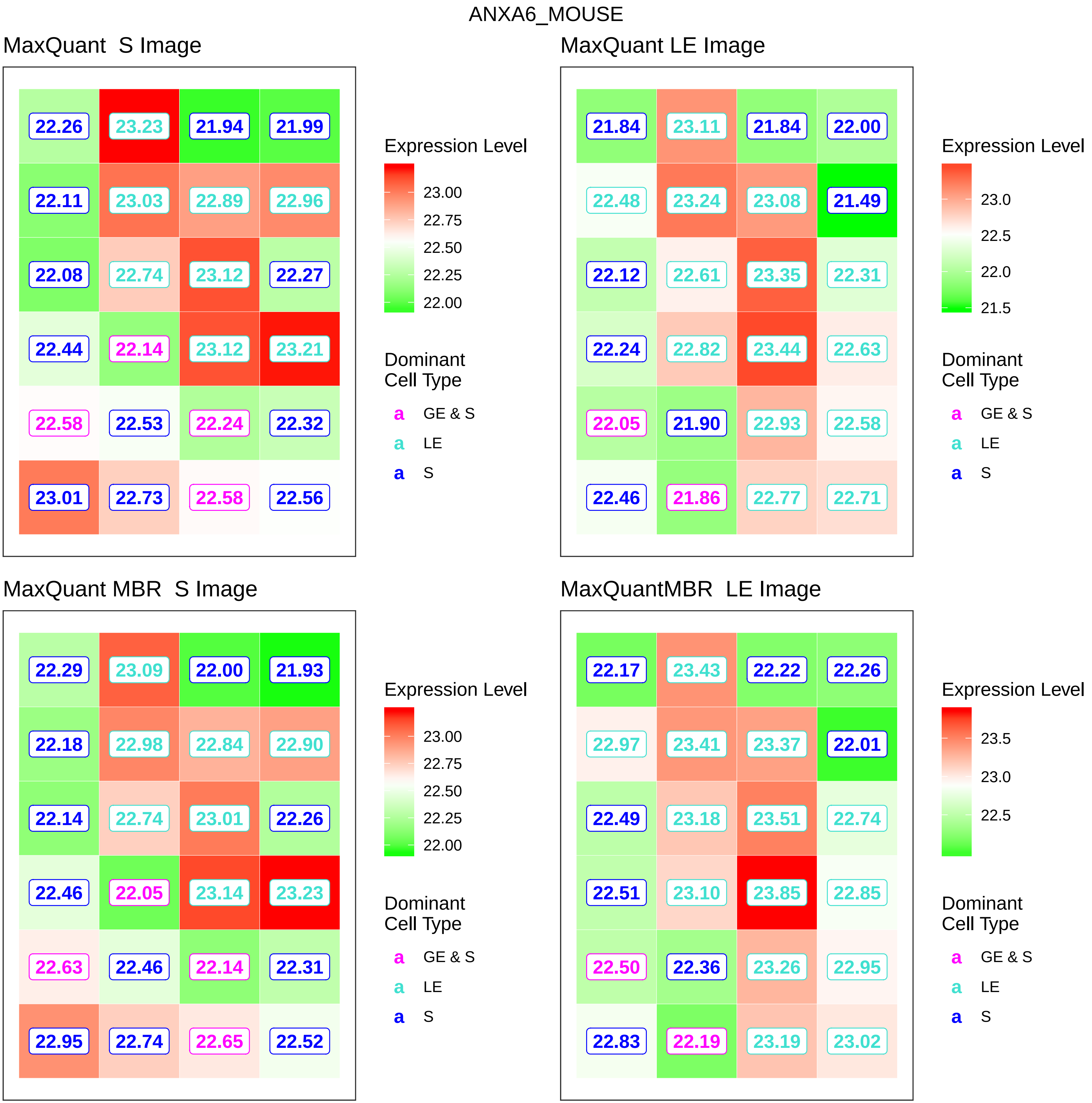
ANXA6_MOUSE
MaxQuant S Image
22.26 23.23 21.94 21.99
22.11 23.03 22.89 22.96
22.08 22.74 23.12 22.27
22.44 22.14 23.12 23.21
22.58 22.53 22.24 22.32
23.01 22.73 22.58 22.56
Expression Level
23.00
22.75
22.50
22.25
22.00
Dominant Cell Type
a GE & S
a LE
a S
MaxQuant LE Image
21.84 23.11 21.84 22.00
22.48 23.24 23.08 21.49
22.12 22.61 23.35 22.31
22.24 22.82 23.44 22.63
22.05 21.90 22.93 22.58
22.46 21.86 22.77 22.71
Expression Level
23.0
22.5
22.0
21.5
Dominant Cell Type
a GE & S
a LE
a S
MaxQuant MBR S Image
22.29 23.09 22.00 21.93
22.18 22.98 22.84 22.90
22.14 22.74 23.01 22.26
22.46 22.05 23.14 23.23
22.63 22.46 22.14 22.31
22.95 22.74 22.65 22.52
Expression Level
23.00
22.75
22.50
22.25
22.00
Dominant Cell Type
a GE & S
a LE
a S
MaxQuantMBR LE Image
22.17 23.43 22.22 22.26
22.97 23.41 23.37 22.01
22.49 23.18 23.51 22.74
22.51 23.10 23.85 22.85
22.50 22.36 23.26 22.95
22.83 22.19 23.19 23.02
Expression Level
23.5
23.0
22.5
Dominant Cell Type
a GE & S
a LE
a S

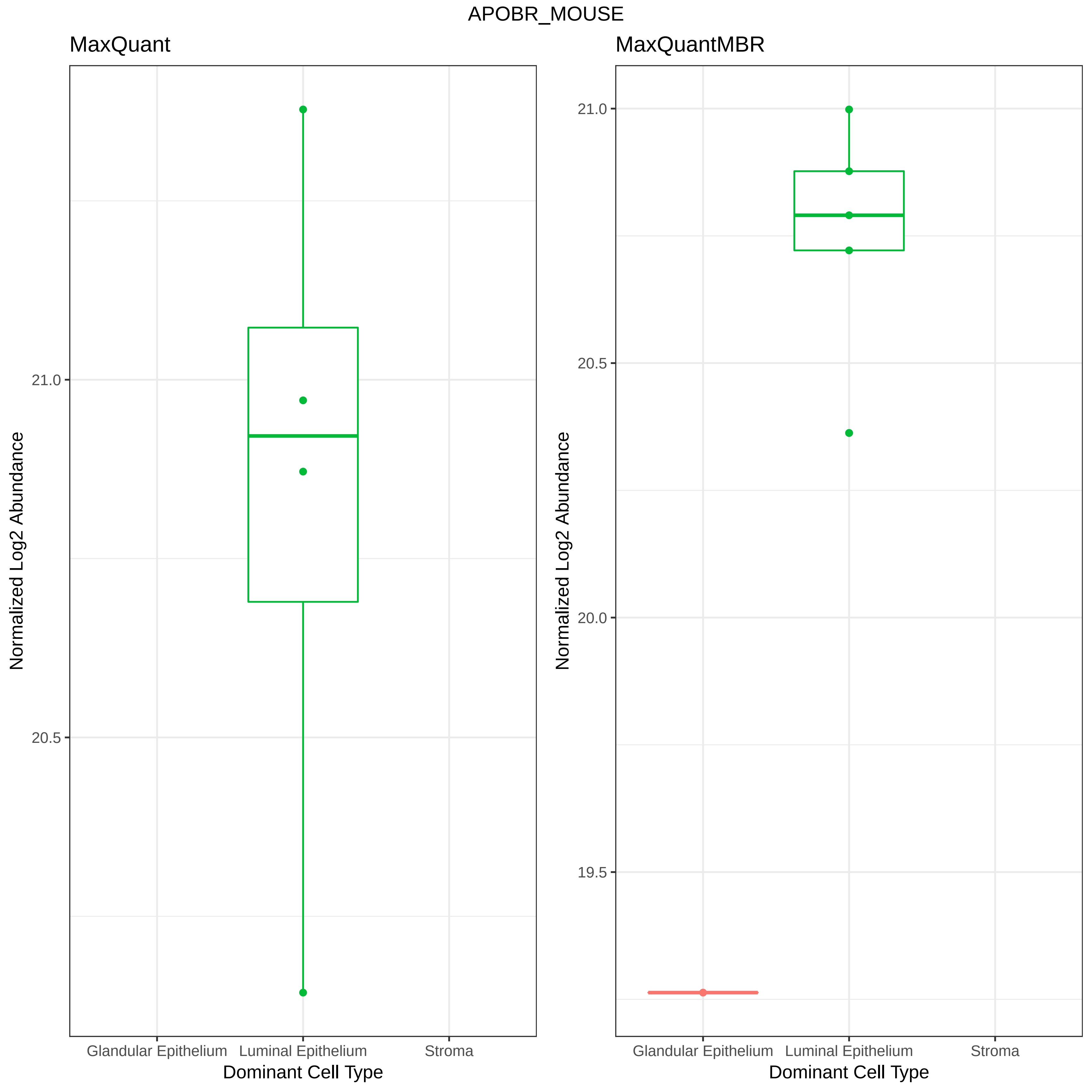
APOBR_MOUSE
MaxQuant
MaxQuantMBR
Normalized Log2 Abundance
21.0
20.5
20.0
19.5
Glandular Epithelium
Luminal Epithelium
Stroma
Dominant Cell Type

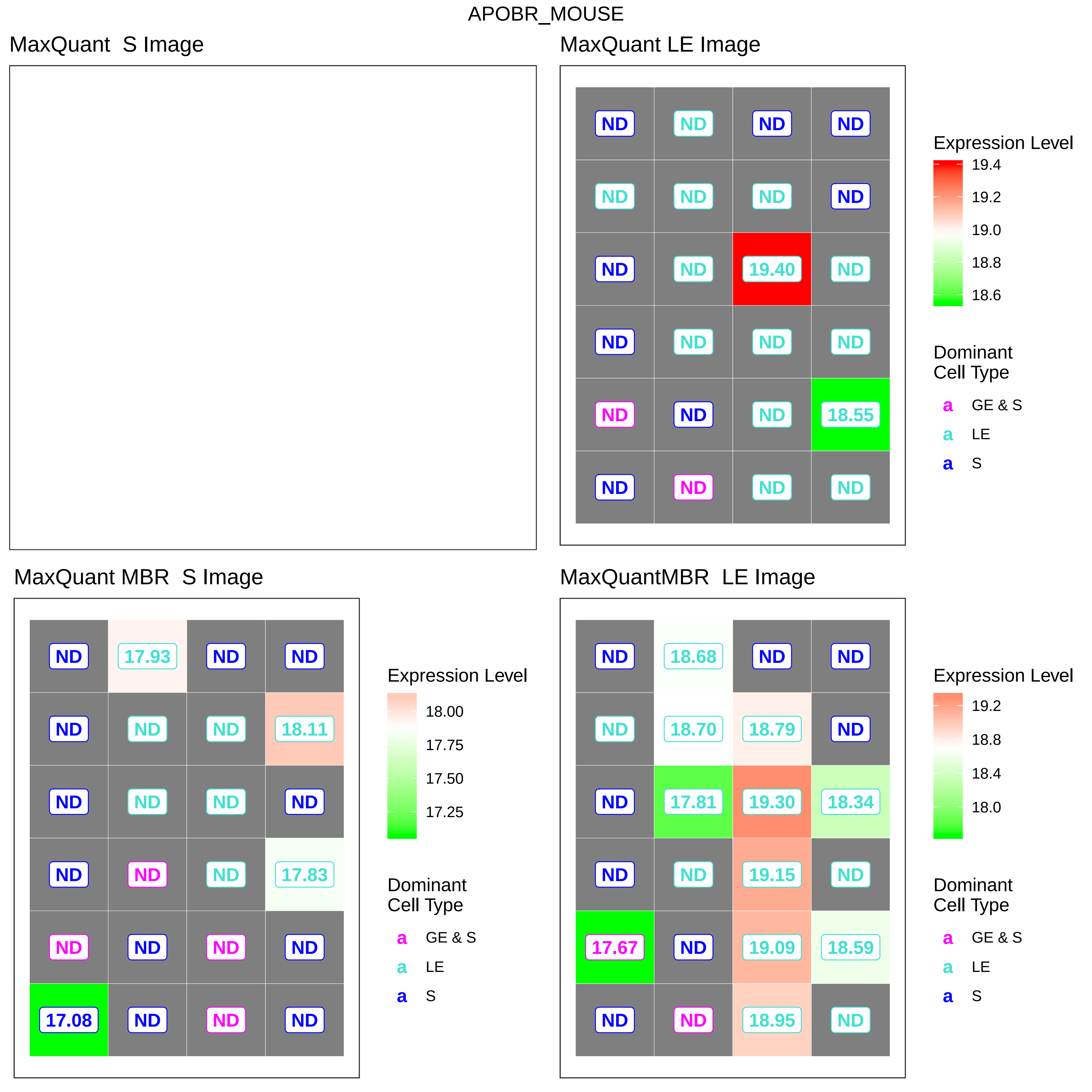
APOBR_MOUSE
MaxQuant S Image
MaxQuant LE Image
ND ND ND ND
ND ND ND ND
ND ND 19.40 ND
ND ND ND ND
ND ND ND 18.55
ND ND ND ND
Expression Level
19.4
19.2
19.0
18.8
18.6
Dominant Cell Type
a GE & S
a LE
a S
MaxQuant MBR S Image
ND 17.93 ND ND
ND ND ND 18.11
ND ND ND ND
ND ND ND 17.83
ND ND ND ND
17.08 ND ND ND
Expression Level
18.00
17.75
17.50
17.25
Dominant Cell Type
a GE & S
a LE
a S
MaxQuantMBR LE Image
ND 18.68 ND ND
ND 18.70 18.79 ND
ND 17.81 19.30 18.34
ND ND 19.15 ND
17.67 ND 19.09 18.59
ND ND 18.95 ND
Expression Level
19.2
18.8
18.4
18.0
Dominant Cell Type
a GE & S
a LE
a S

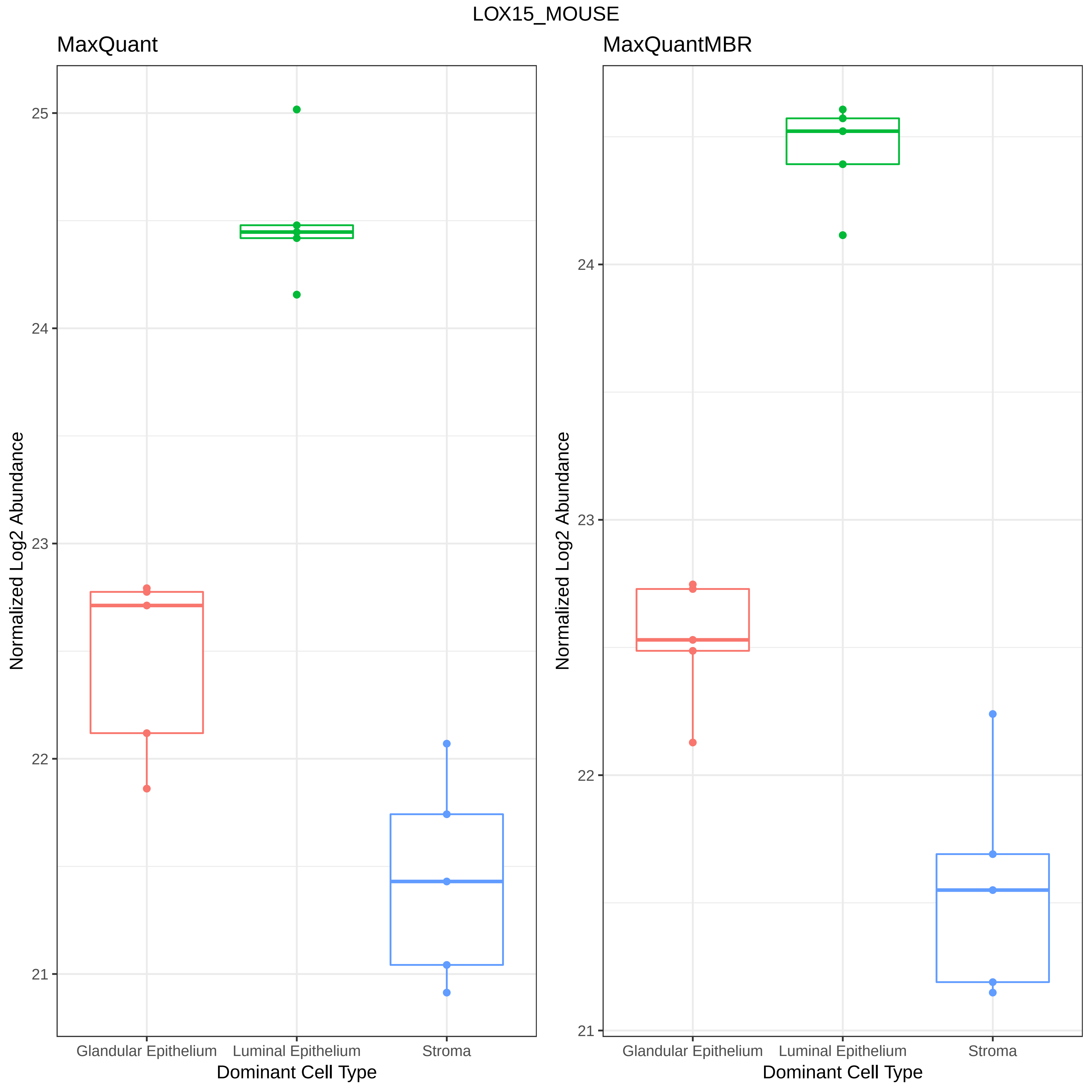

LOX15_MOUSE
MaxQuant
MaxQuantMBR
Normalized Log2 Abundance
Dominant Cell Type
Glandular Epithelium
Luminal Epithelium
Stroma
25
24
23
22
21

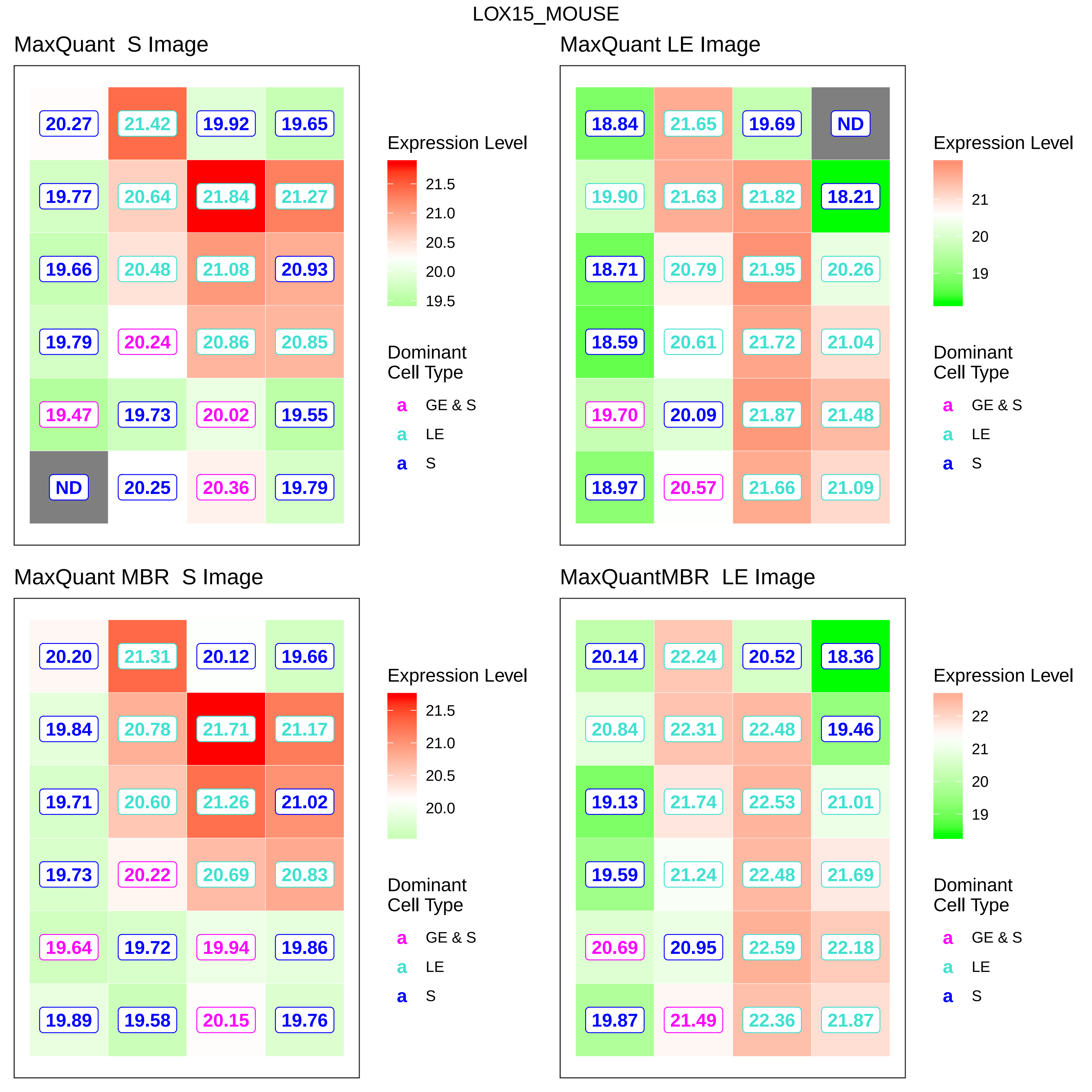
LOX15_MOUSE
MaxQuant S Image
20.27 21.42 19.92 19.65
19.77 20.64 21.84 21.27
19.66 20.48 21.08 20.93
19.79 20.24 20.86 20.85
19.47 19.73 20.02 19.55
ND 20.25 20.36 19.79
Expression Level
21.5
21.0
20.5
20.0
19.5
Dominant Cell Type
a GE & S
a LE
a S
MaxQuant LE Image
18.84 21.65 19.69 ND
19.90 21.63 21.82 18.21
18.71 20.79 21.95 20.26
18.59 20.61 21.72 21.04
19.70 20.09 21.87 21.48
18.97 20.57 21.66 21.09
Expression Level
21
20
19
Dominant Cell Type
a GE & S
a LE
a S
MaxQuant MBR S Image
20.20 21.31 20.12 19.66
19.84 20.78 21.71 21.17
19.71 20.60 21.26 21.02
19.73 20.22 20.69 20.83
19.64 19.72 19.94 19.86
19.89 19.58 20.15 19.76
Expression Level
21.5
21.0
20.5
20.0
Dominant Cell Type
a GE & S
a LE
a S
MaxQuantMBR LE Image
20.14 22.24 20.52 18.36
20.84 22.31 22.48 19.46
19.13 21.74 22.53 21.01
19.59 21.24 22.48 21.69
20.69 20.95 22.59 22.18
19.87 21.49 22.36 21.87
Expression Level
22
21
20
19
Dominant Cell Type
a GE & S
a LE
a S

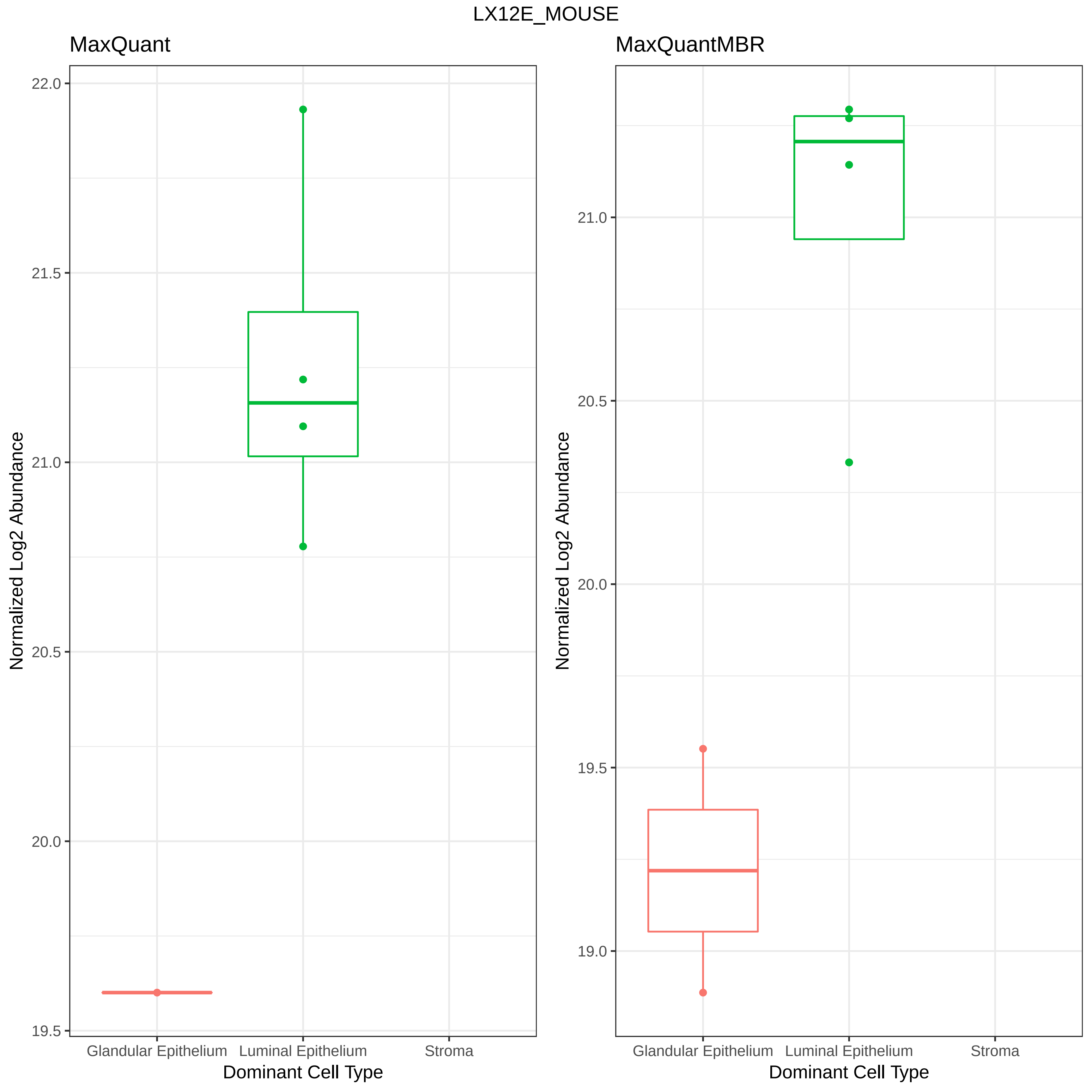

LX12E_MOUSE
MaxQuant
MaxQuantMBR
Normalized Log2 Abundance
22.0
21.5
21.0
20.5
20.0
19.5
19.0
Glandular Epithelium
Luminal Epithelium
Stroma
Dominant Cell Type

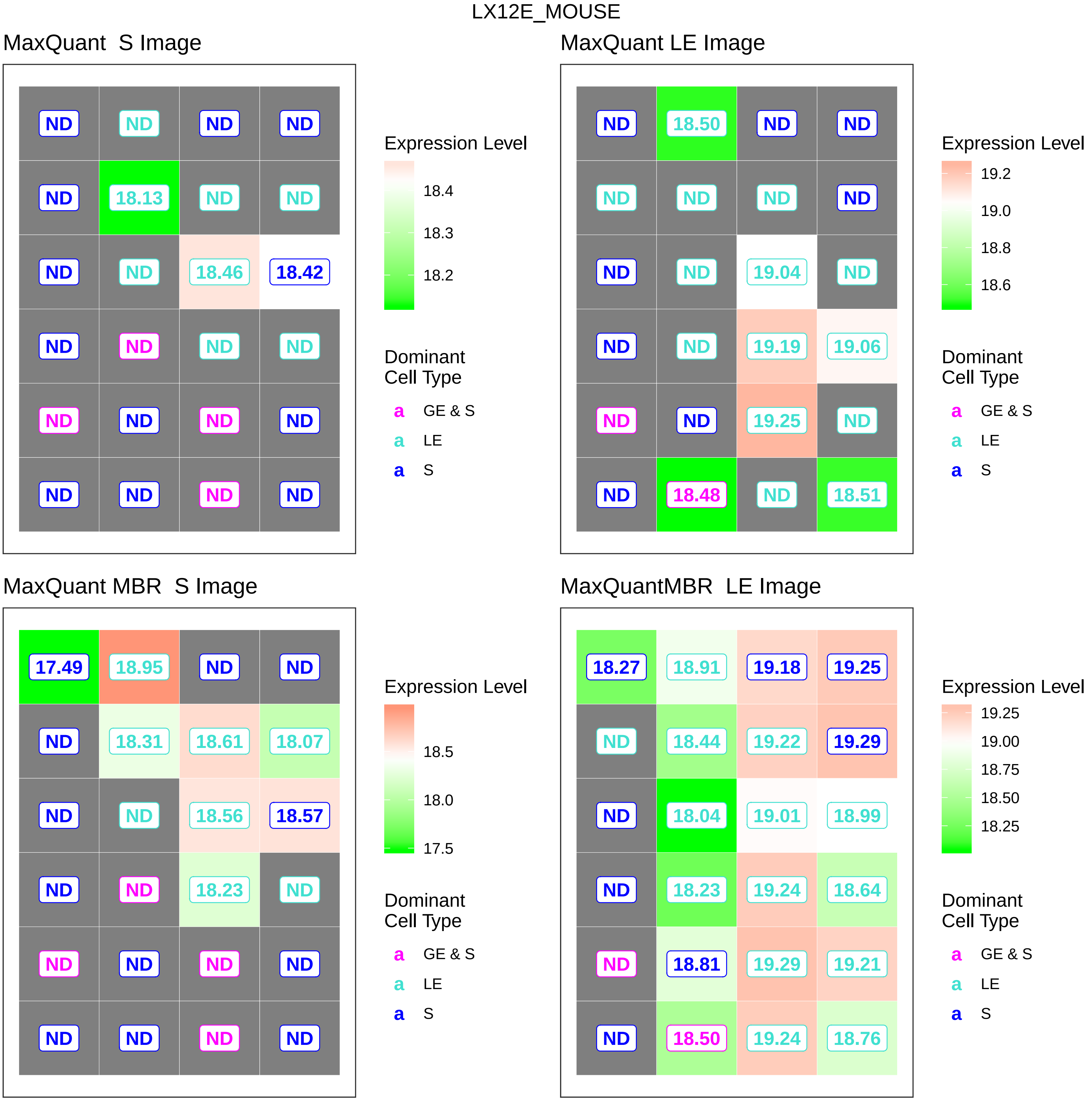
LX12E_MOUSE
MaxQuant S Image
ND ND ND ND
ND 18.13 ND ND
ND ND 18.46 18.42
ND ND ND ND
ND ND ND ND
ND ND ND ND
Expression Level
18.4
18.3
18.2
Dominant Cell Type
a GE & S
a LE
a S
MaxQuant LE Image
ND 18.50 ND ND
ND ND ND ND
ND ND 19.04 ND
ND ND 19.19 19.06
ND ND 19.25 ND
ND 18.48 ND 18.51
Expression Level
19.2
19.0
18.8
18.6
Dominant Cell Type
a GE & S
a LE
a S
MaxQuant MBR S Image
17.49 18.95 ND ND
ND 18.31 18.61 18.07
ND ND 18.56 18.57
ND ND 18.23 ND
ND ND ND ND
ND ND ND ND
Expression Level
18.5
18.0
17.5
Dominant Cell Type
a GE & S
a LE
a S
MaxQuantMBR LE Image
18.27 18.91 19.18 19.25
ND 18.44 19.22 19.29
ND 18.04 19.01 18.99
ND 18.23 19.24 18.64
ND 18.81 19.29 19.21
ND 18.50 19.24 18.76
Expression Level
19.25
19.00
18.75
18.50
18.25
Dominant Cell Type
a GE & S
a LE
a S

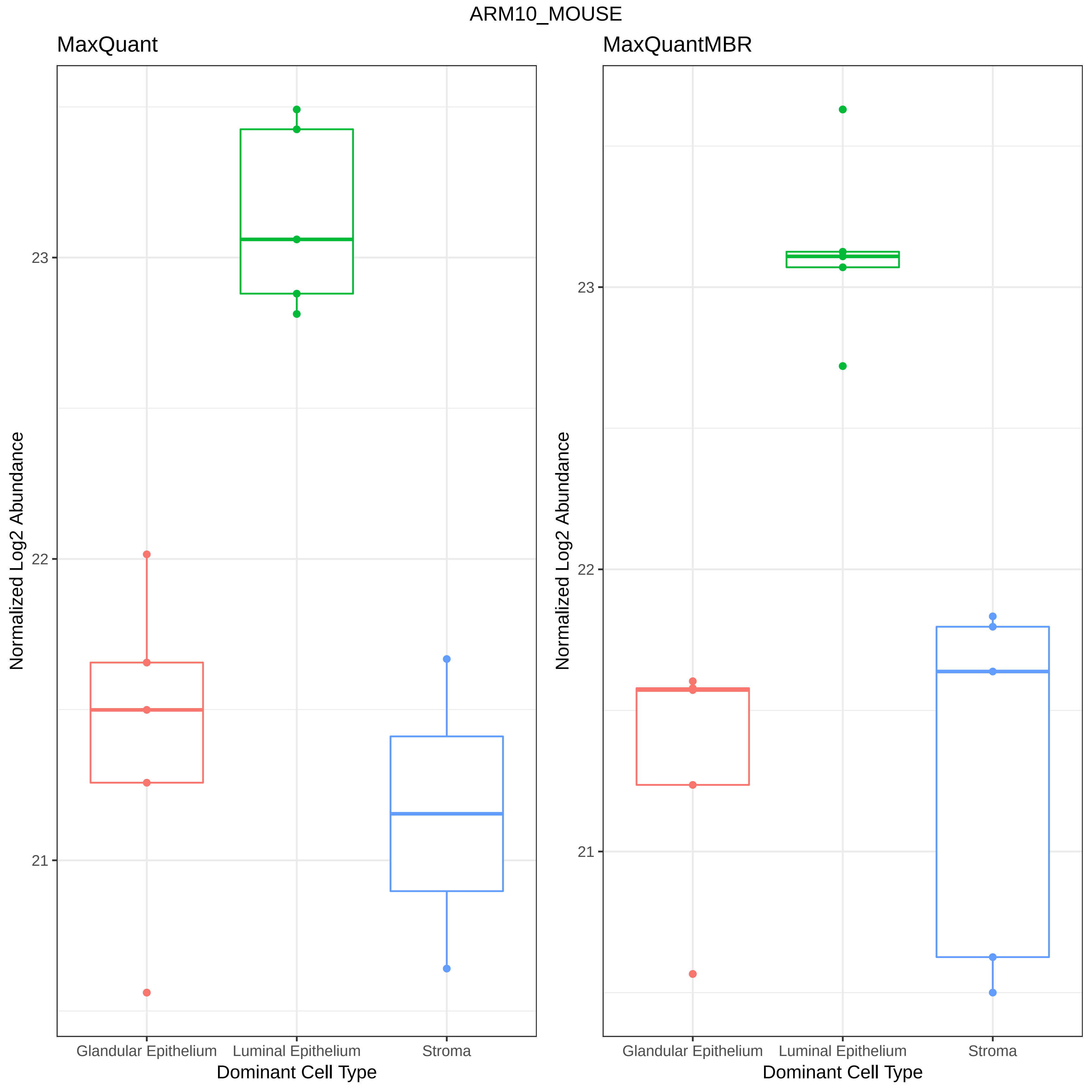

ARM10_MOUSE
MaxQuant
MaxQuantMBR
Normalized Log2 Abundance
23
22
21
Glandular Epithelium
Luminal Epithelium
Stroma
Dominant Cell Type

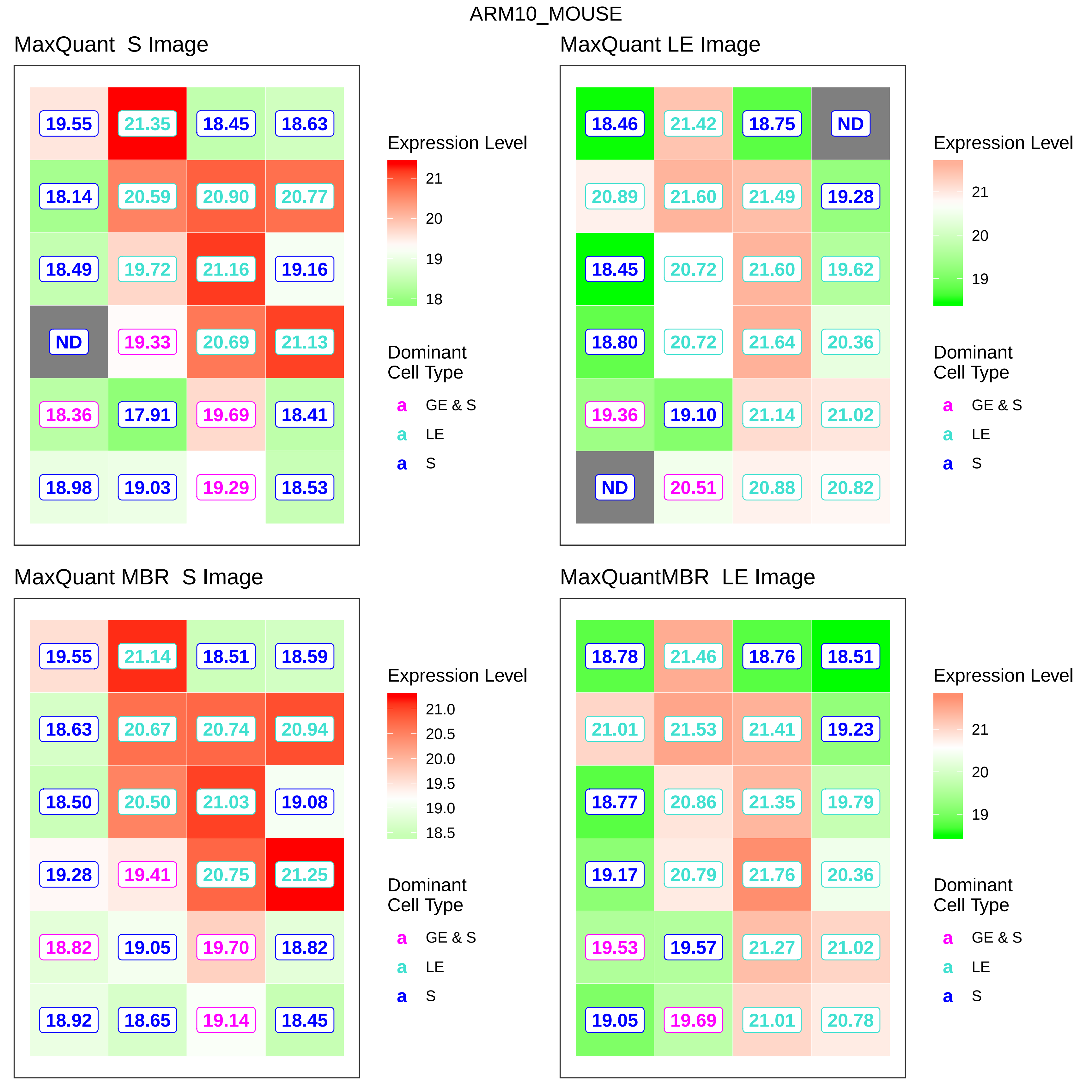
ARM10_MOUSE
MaxQuant S Image
19.55 21.35 18.45 18.63
18.14 20.59 20.90 20.77
18.49 19.72 21.16 19.16
ND 19.33 20.69 21.13
18.36 17.91 19.69 18.41
18.98 19.03 19.29 18.53
Expression Level
21
20
19
18
Dominant Cell Type
a GE & S
a LE
a S
MaxQuant LE Image
18.46 21.42 18.75 ND
20.89 21.60 21.49 19.28
18.45 20.72 21.60 19.62
18.80 20.72 21.64 20.36
19.36 19.10 21.14 21.02
ND 20.51 20.88 20.82
Expression Level
21
20
19
Dominant Cell Type
a GE & S
a LE
a S
MaxQuant MBR S Image
19.55 21.14 18.51 18.59
18.63 20.67 20.74 20.94
18.50 20.50 21.03 19.08
19.28 19.41 20.75 21.25
18.82 19.05 19.70 18.82
18.92 18.65 19.14 18.45
Expression Level
21.0
20.5
20.0
19.5
19.0
18.5
Dominant Cell Type
a GE & S
a LE
a S
MaxQuantMBR LE Image
18.78 21.46 18.76 18.51
21.01 21.53 21.41 19.23
18.77 20.86 21.35 19.79
19.17 20.79 21.76 20.36
19.53 19.57 21.27 21.02
19.05 19.69 21.01 20.78
Expression Level
21
20
19
Dominant Cell Type
a GE & S
a LE
a S

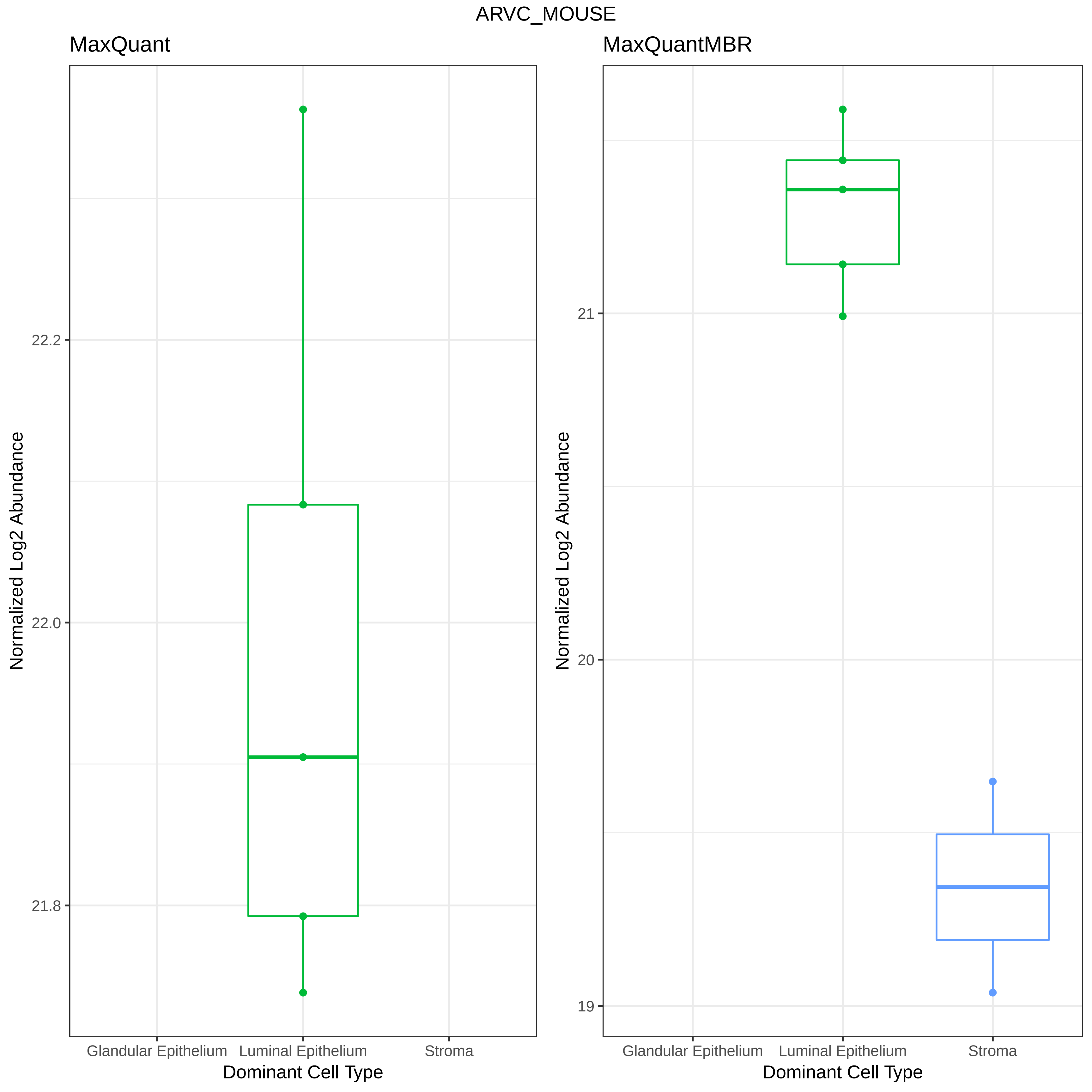

ARVC_MOUSE
MaxQuant
MaxQuantMBR
Normalized Log2 Abundance
22.2
22.0
21.8
21
20
19
Glandular Epithelium
Luminal Epithelium
Stroma
Dominant Cell Type

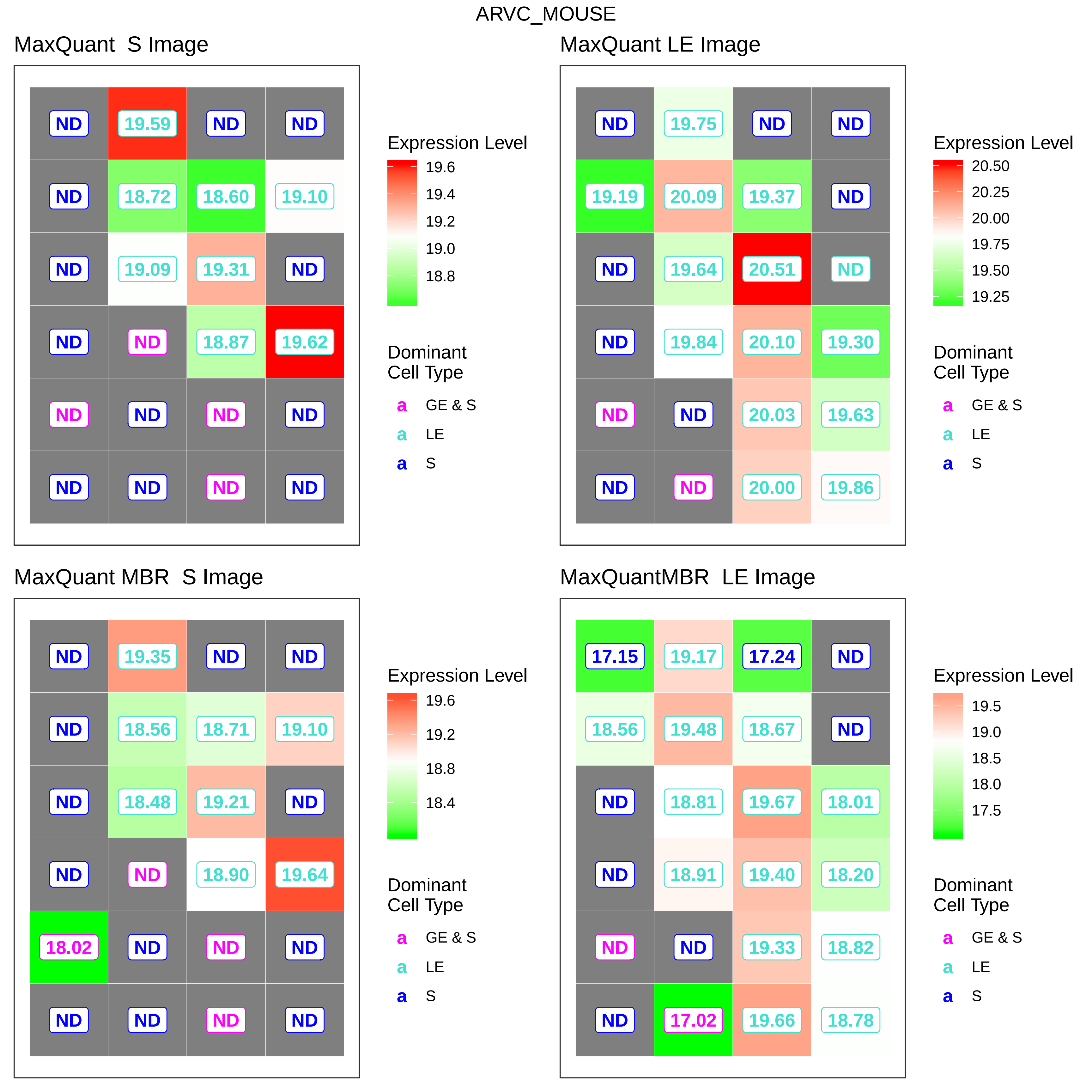

ARVC_MOUSE
MaxQuant S Image
ND
19.59
ND
ND
ND
18.72
18.60
19.10
ND
19.09
19.31
ND
ND
ND
18.87
19.62
ND
ND
ND
ND
ND
ND
ND
ND
Expression Level
19.6
19.4
19.2
19.0
18.8
Dominant Cell Type
a GE & S
a LE
a S
MaxQuant LE Image
ND
19.75
ND
ND
19.19
20.09
19.37
ND
ND
19.64
20.51
ND
ND
19.84
20.10
19.30
ND
ND
20.03
19.63
ND
ND
20.00
19.86
Expression Level
20.50
20.25
20.00
19.75
19.50
19.25
Dominant Cell Type
a GE & S
a LE
a S
MaxQuant MBR S Image
ND
19.35
ND
ND
ND
18.56
18.71
19.10
ND
18.48
19.21
ND
ND
ND
18.90
19.64
18.02
ND
ND
ND
ND
ND
ND
ND
Expression Level
19.6
19.2
18.8
18.4
Dominant Cell Type
a GE & S
a LE
a S
MaxQuantMBR LE Image
17.15
19.17
17.24
ND
18.56
19.48
18.67
ND
ND
18.81
19.67
18.01
ND
18.91
19.40
18.20
ND
ND
19.33
18.82
ND
17.02
19.66
18.78
Expression Level
19.5
19.0
18.5
18.0
17.5
Dominant Cell Type
a GE & S
a LE
a S

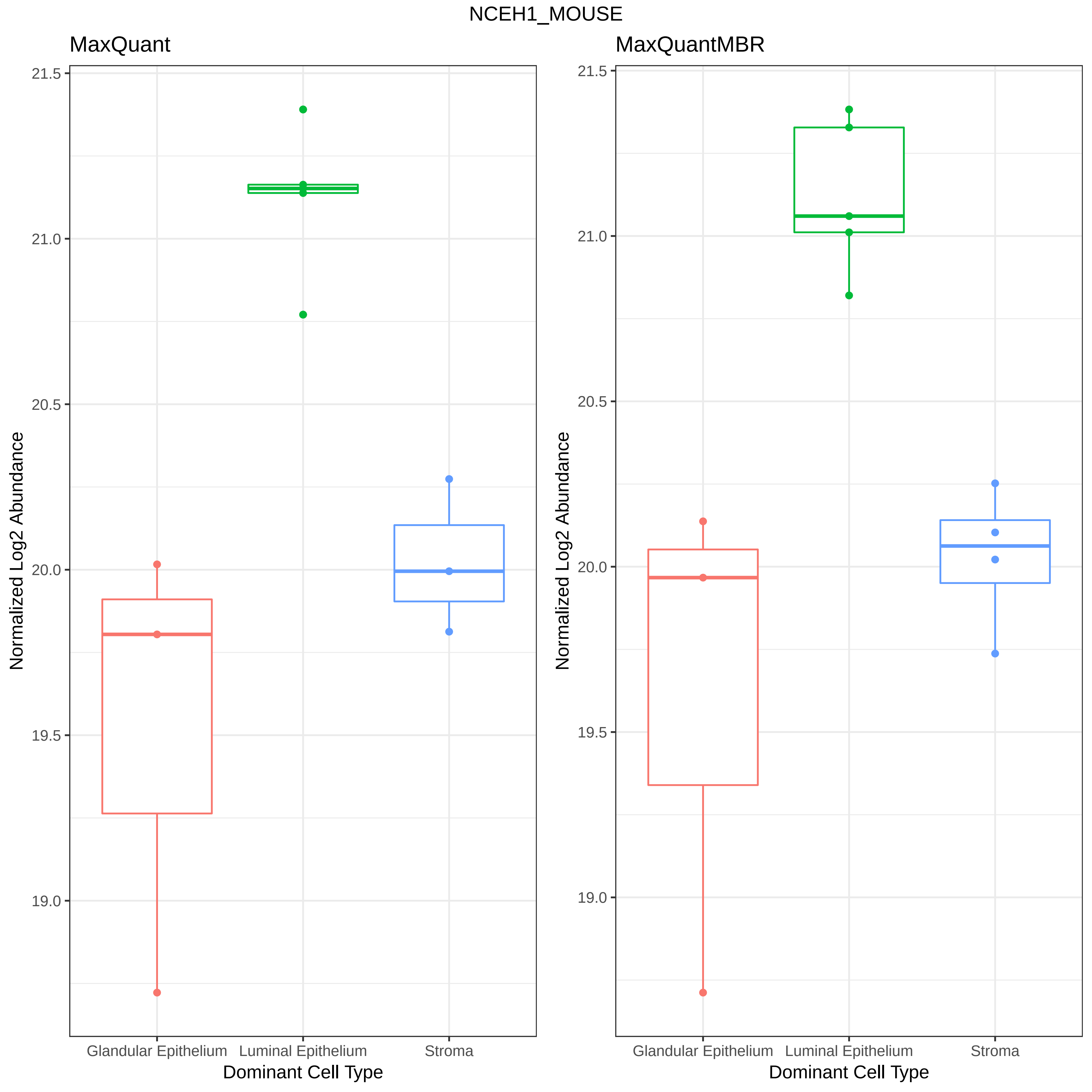

NCEH1_MOUSE
MaxQuant
MaxQuantMBR
Normalized Log2 Abundance
21.5
21.0
20.5
20.0
19.5
19.0
Glandular Epithelium
Luminal Epithelium
Stroma
Dominant Cell Type

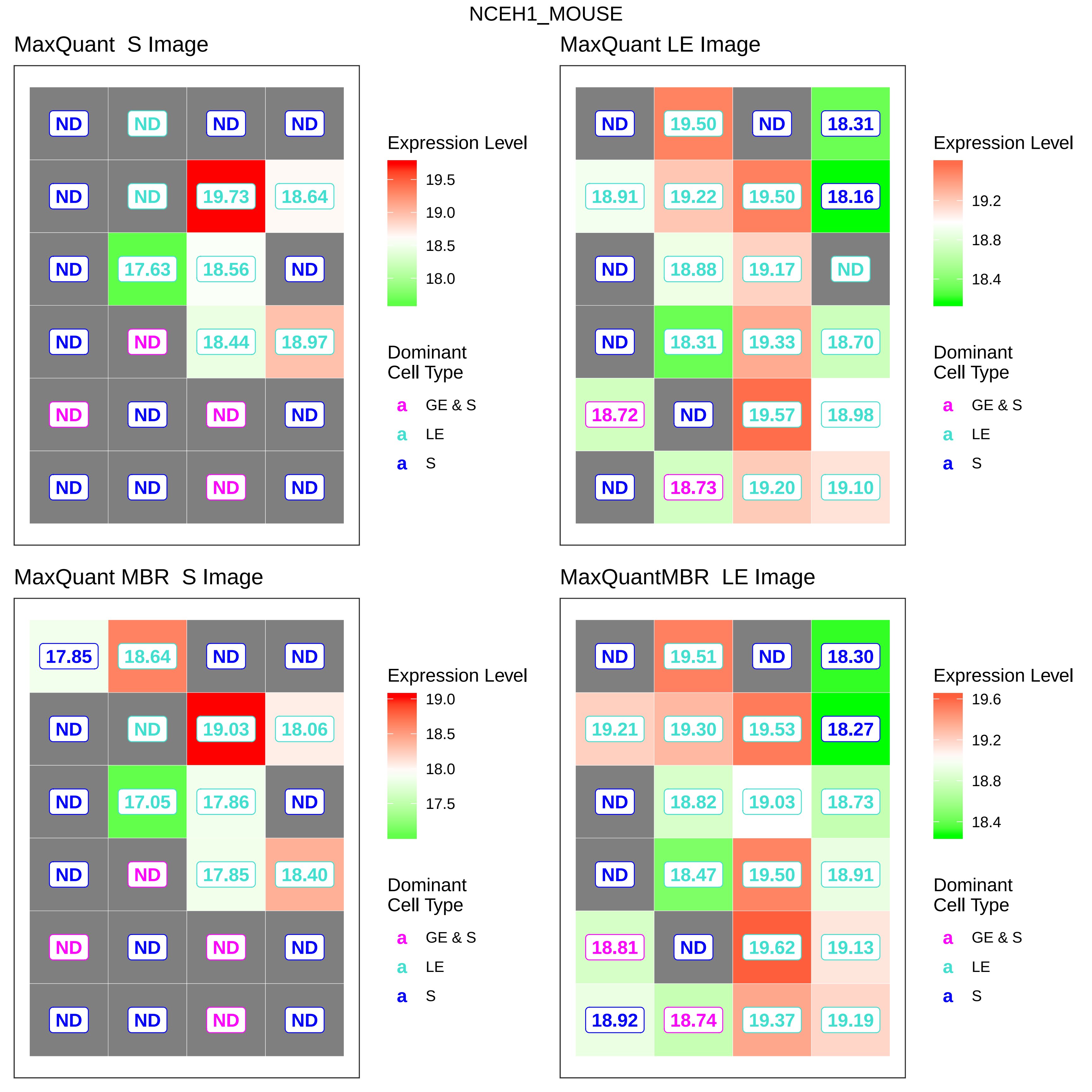
NCEH1_MOUSE
MaxQuant S Image
ND ND ND ND
ND ND 19.73 18.64
ND 17.63 18.56 ND
ND ND 18.44 18.97
ND ND ND ND
ND ND ND ND
Expression Level
19.5
19.0
18.5
18.0
Dominant Cell Type
a GE & S
a LE
a S
MaxQuant LE Image
ND 19.50 ND 18.31
18.91 19.22 19.50 18.16
ND 18.88 19.17 ND
ND 18.31 19.33 18.70
18.72 ND 19.57 18.98
ND 18.73 19.20 19.10
Expression Level
19.2
18.8
18.4
Dominant Cell Type
a GE & S
a LE
a S
MaxQuant MBR S Image
17.85 18.64 ND ND
ND ND 19.03 18.06
ND 17.05 17.86 ND
ND ND 17.85 18.40
ND ND ND ND
ND ND ND ND
Expression Level
19.0
18.5
18.0
17.5
Dominant Cell Type
a GE & S
a LE
a S
MaxQuantMBR LE Image
ND 19.51 ND 18.30
19.21 19.30 19.53 18.27
ND 18.82 19.03 18.73
ND 18.47 19.50 18.91
18.81 ND 19.62 19.13
18.92 18.74 19.37 19.19
Expression Level
19.6
19.2
18.8
18.4
Dominant Cell Type
a GE & S
a LE
a S

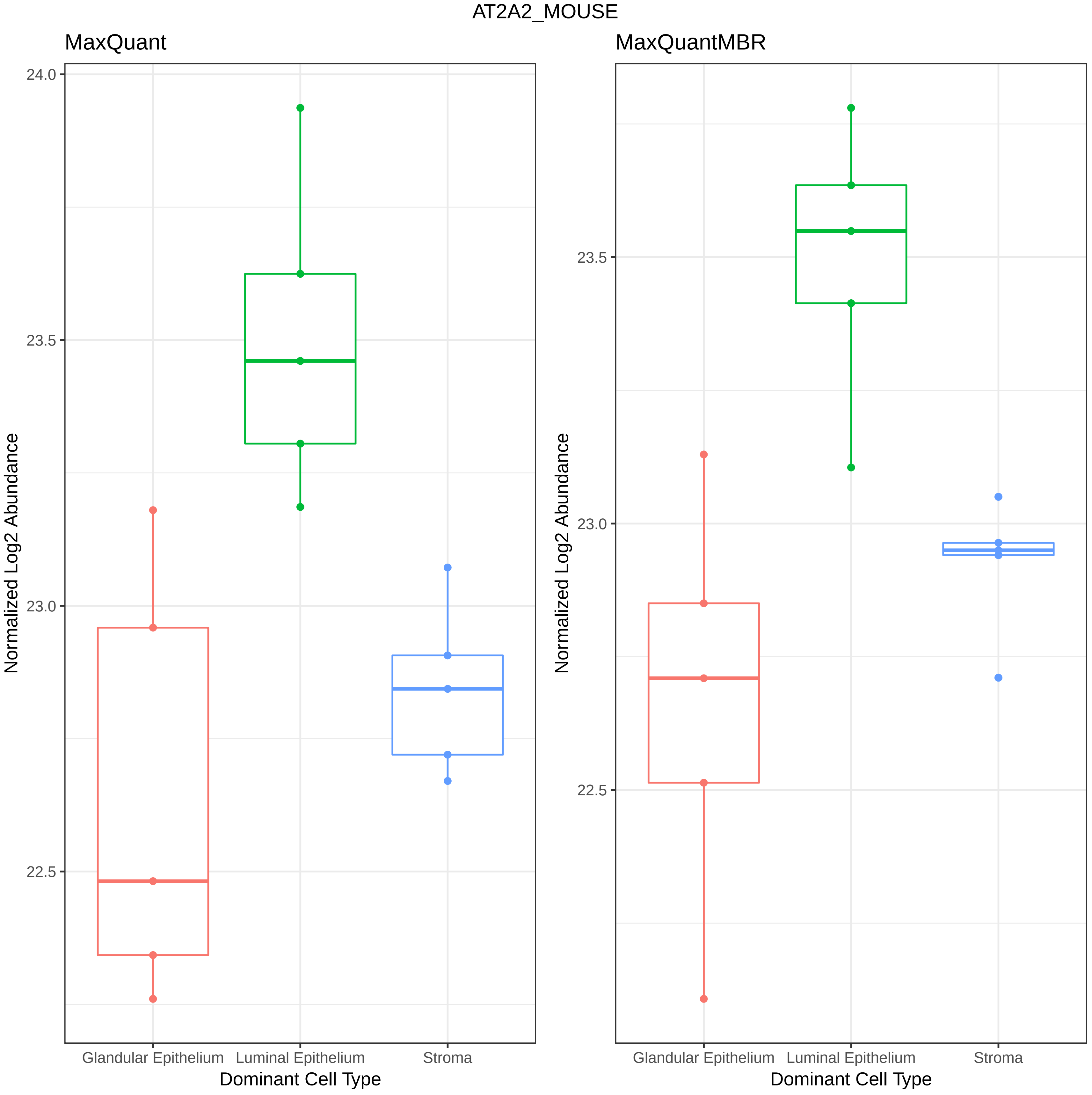

AT2A2_MOUSE
MaxQuant
MaxQuantMBR
24.0
23.5
23.0
22.5
Normalized Log2 Abundance
Glandular Epithelium
Luminal Epithelium
Stroma
Dominant Cell Type

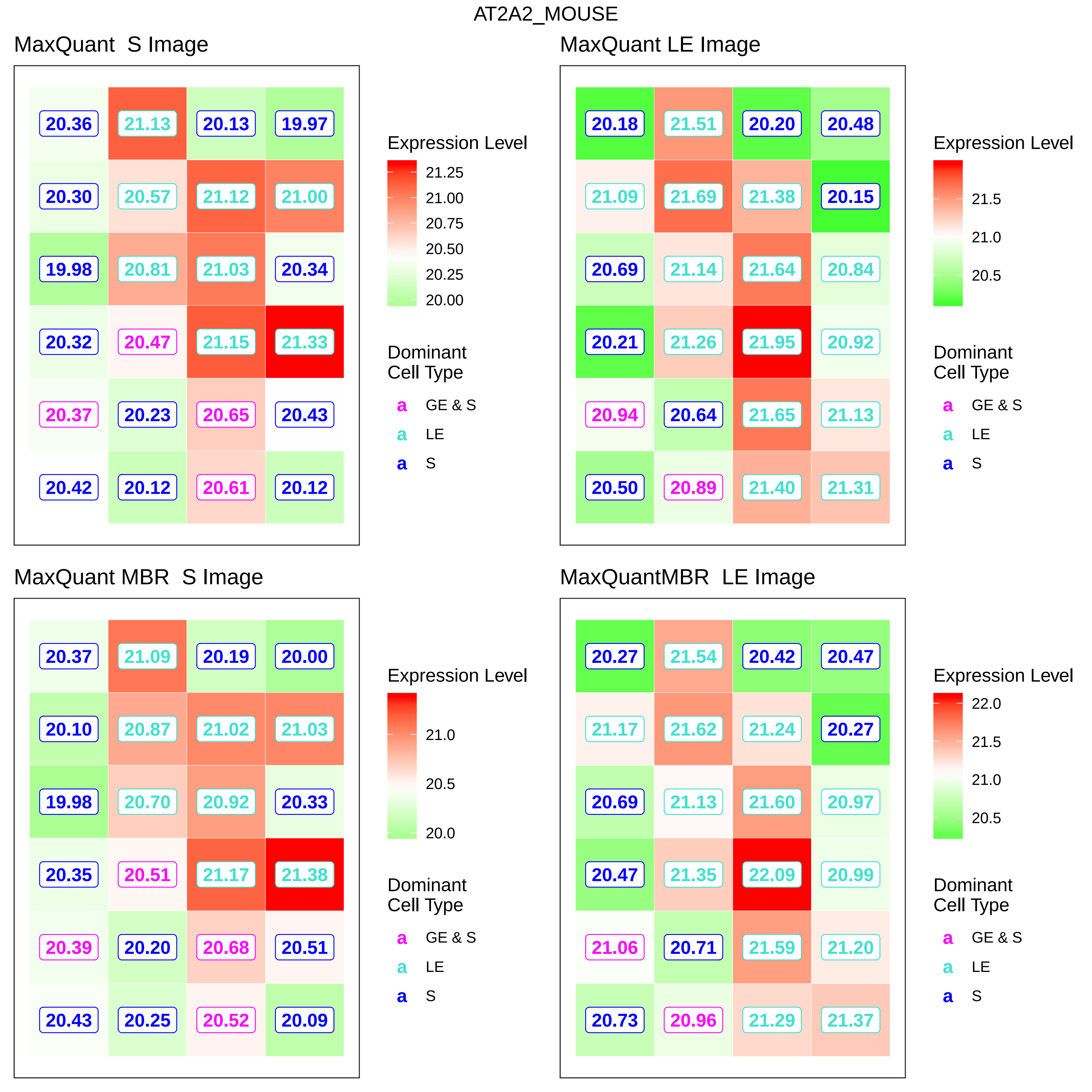
AT2A2_MOUSE
MaxQuant S Image
20.36 21.13 20.13 19.97
20.30 20.57 21.12 21.00
19.98 20.81 21.03 20.34
20.32 20.47 21.15 21.33
20.37 20.23 20.65 20.43
20.42 20.12 20.61 20.12
Expression Level
21.25
21.00
20.75
20.50
20.25
20.00
Dominant Cell Type
a GE & S
a LE
a S
MaxQuant LE Image
20.18 21.51 20.20 20.48
21.09 21.69 21.38 20.15
20.69 21.14 21.64 20.84
20.21 21.26 21.95 20.92
20.94 20.64 21.65 21.13
20.50 20.89 21.40 21.31
Expression Level
21.5
21.0
20.5
Dominant Cell Type
a GE & S
a LE
a S
MaxQuant MBR S Image
20.37 21.09 20.19 20.00
20.10 20.87 21.02 21.03
19.98 20.70 20.92 20.33
20.35 20.51 21.17 21.38
20.39 20.20 20.68 20.51
20.43 20.25 20.52 20.09
Expression Level
21.0
20.5
20.0
Dominant Cell Type
a GE & S
a LE
a S
MaxQuantMBR LE Image
20.27 21.54 20.42 20.47
21.17 21.62 21.24 20.27
20.69 21.13 21.60 20.97
20.47 21.35 22.09 20.99
21.06 20.71 21.59 21.20
20.73 20.96 21.29 21.37
Expression Level
22.0
21.5
21.0
20.5
Dominant Cell Type
a GE & S
a LE
a S

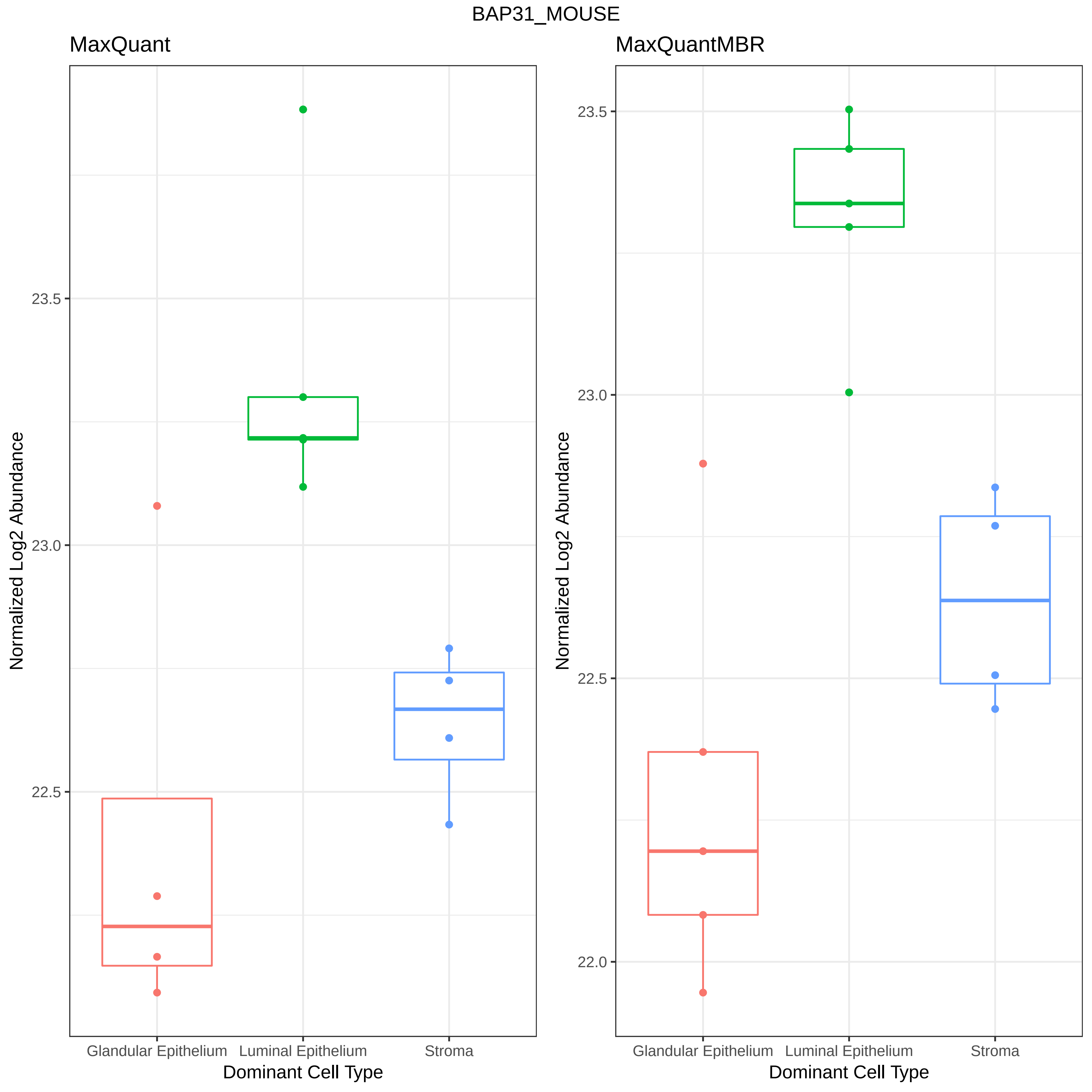

BAP31_MOUSE
MaxQuant
MaxQuantMBR
Normalized Log2 Abundance
23.5
23.0
22.5
22.0
Glandular Epithelium
Luminal Epithelium
Stroma
Dominant Cell Type

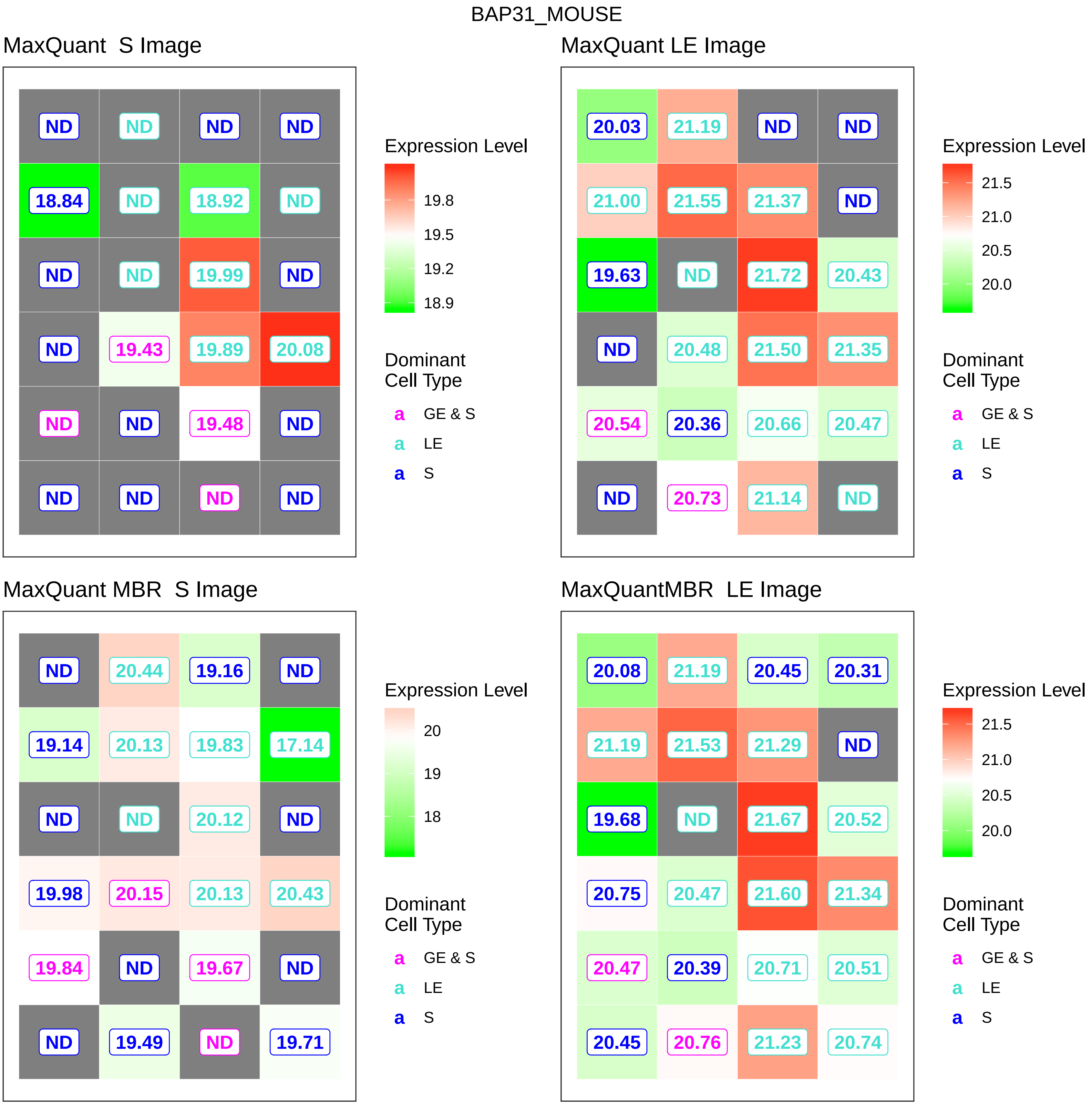
BAP31_MOUSE
MaxQuant S Image
ND ND ND ND
18.84 ND 18.92 ND
ND ND 19.99 ND
ND 19.43 19.89 20.08
ND ND 19.48 ND
ND ND ND ND
Expression Level
19.8
19.5
19.2
18.9
Dominant Cell Type
a GE & S
a LE
a S
MaxQuant LE Image
20.03 21.19 ND ND
21.00 21.55 21.37 ND
19.63 ND 21.72 20.43
ND 20.48 21.50 21.35
20.54 20.36 20.66 20.47
ND 20.73 21.14 ND
Expression Level
21.5
21.0
20.5
20.0
Dominant Cell Type
a GE & S
a LE
a S
MaxQuant MBR S Image
ND 20.44 19.16 ND
19.14 20.13 19.83 17.14
ND ND 20.12 ND
19.98 20.15 20.13 20.43
19.84 ND 19.67 ND
ND 19.49 ND 19.71
Expression Level
20
19
18
Dominant Cell Type
a GE & S
a LE
a S
MaxQuantMBR LE Image
20.08 21.19 20.45 20.31
21.19 21.53 21.29 ND
19.68 ND 21.67 20.52
20.75 20.47 21.60 21.34
20.47 20.39 20.71 20.51
20.45 20.76 21.23 20.74
Expression Level
21.5
21.0
20.5
20.0
Dominant Cell Type
a GE & S
a LE
a S

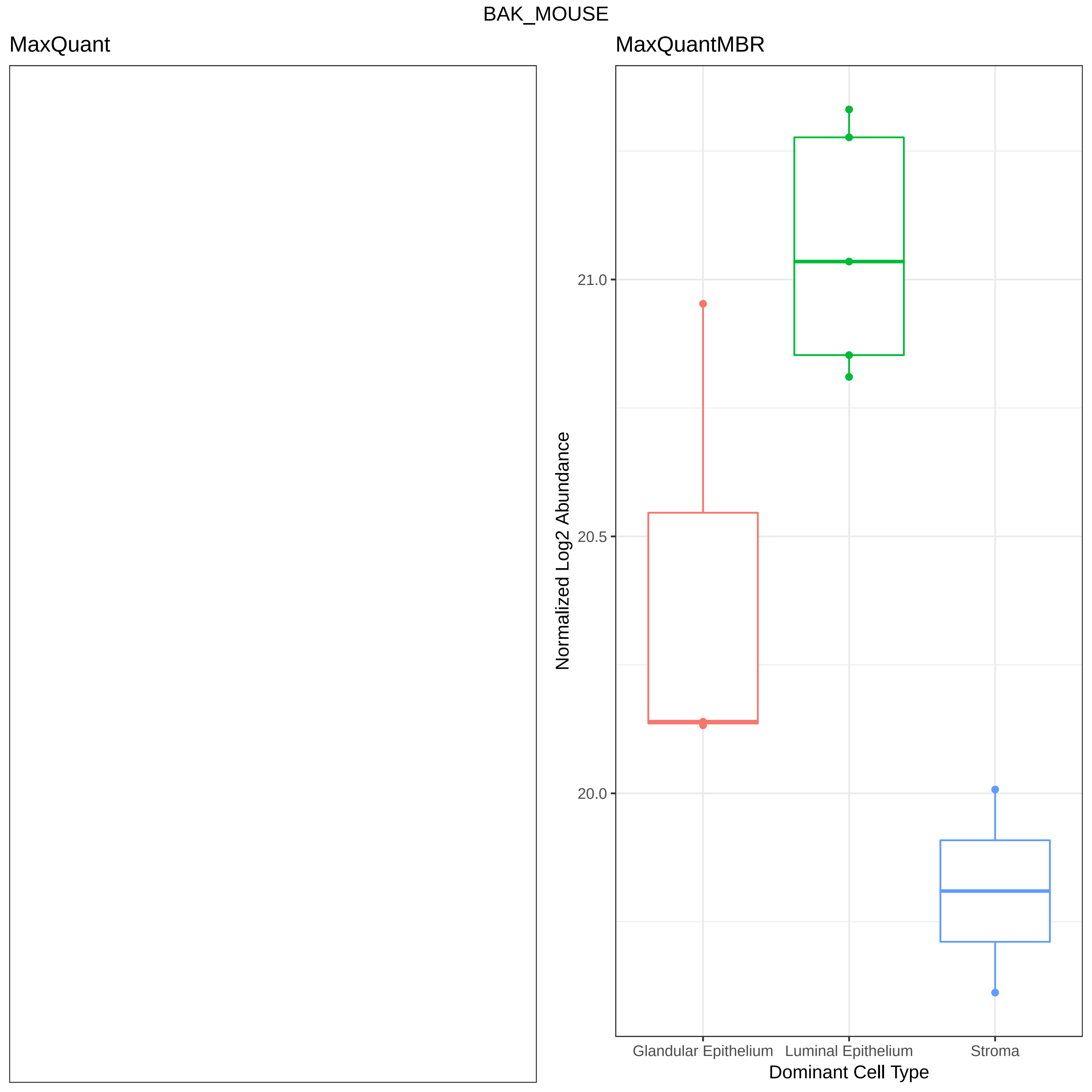
BAK_MOUSE
MaxQuant
MaxQuantMBR
Normalized Log2 Abundance
21.0
20.5
20.0
Glandular Epithelium
Luminal Epithelium
Stroma
Dominant Cell Type

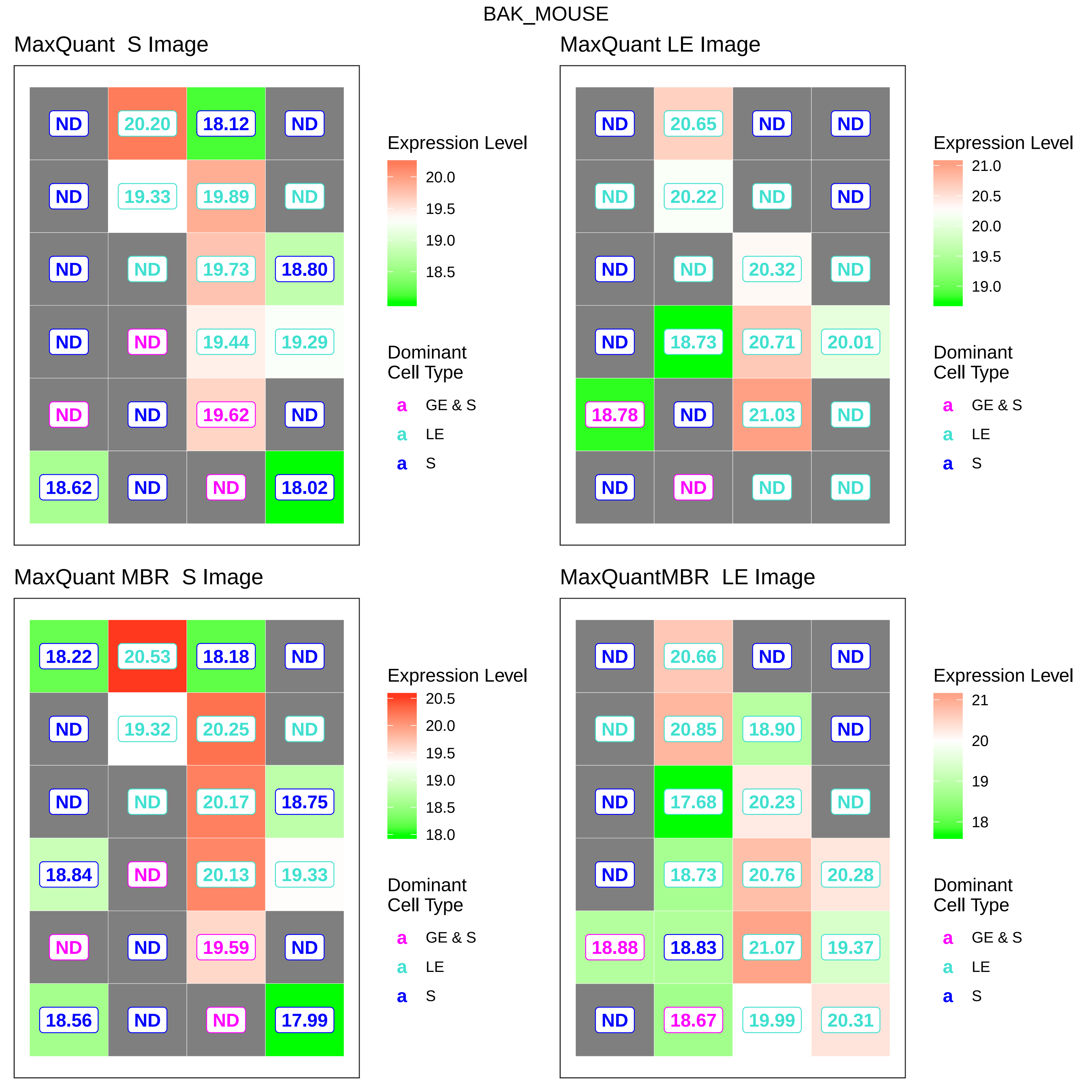
BAK_MOUSE
MaxQuant S Image
ND 20.20 18.12 ND
ND 19.33 19.89 ND
ND ND 19.73 18.80
ND ND 19.44 19.29
ND ND 19.62 ND
18.62 ND ND 18.02
Expression Level
20.0
19.5
19.0
18.5
Dominant Cell Type
a GE & S
a LE
a S
MaxQuant LE Image
ND 20.65 ND ND
ND 20.22 ND ND
ND ND 20.32 ND
ND 18.73 20.71 20.01
18.78 ND 21.03 ND
ND ND ND ND
Expression Level
21.0
20.5
20.0
19.5
19.0
Dominant Cell Type
a GE & S
a LE
a S
MaxQuant MBR S Image
18.22 20.53 18.18 ND
ND 19.32 20.25 ND
ND ND 20.17 18.75
18.84 ND 20.13 19.33
ND ND 19.59 ND
18.56 ND ND 17.99
Expression Level
20.5
20.0
19.5
19.0
18.5
18.0
Dominant Cell Type
a GE & S
a LE
a S
MaxQuantMBR LE Image
ND 20.66 ND ND
ND 20.85 18.90 ND
ND 17.68 20.23 ND
ND 18.73 20.76 20.28
18.88 18.83 21.07 19.37
ND 18.67 19.99 20.31
Expression Level
21
20
19
18
Dominant Cell Type
a GE & S
a LE
a S

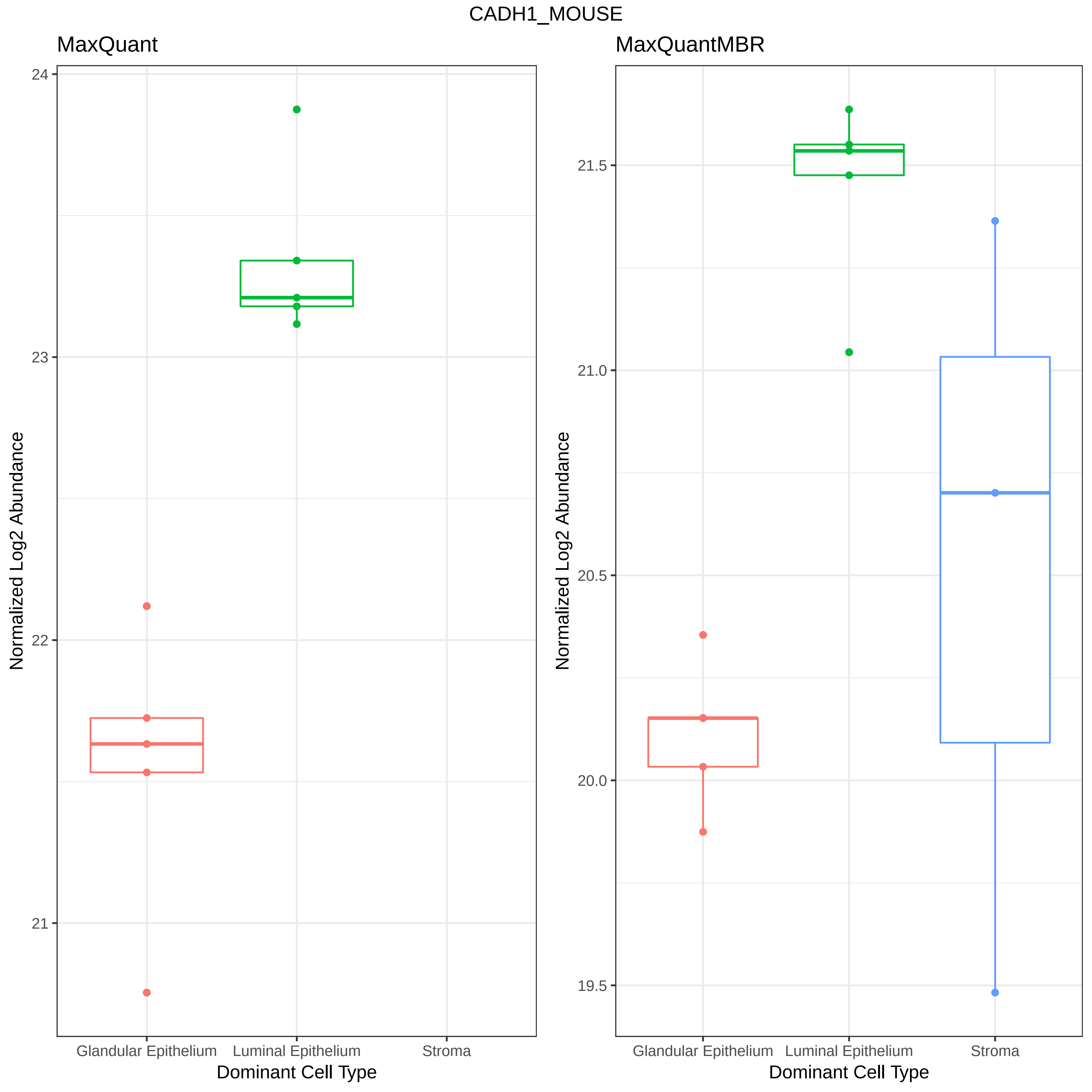
CADH1_MOUSE
MaxQuant
MaxQuantMBR
Normalized Log2 Abundance
24
23
22
21
21.5
21.0
20.5
20.0
19.5
Glandular Epithelium
Luminal Epithelium
Stroma
Dominant Cell Type

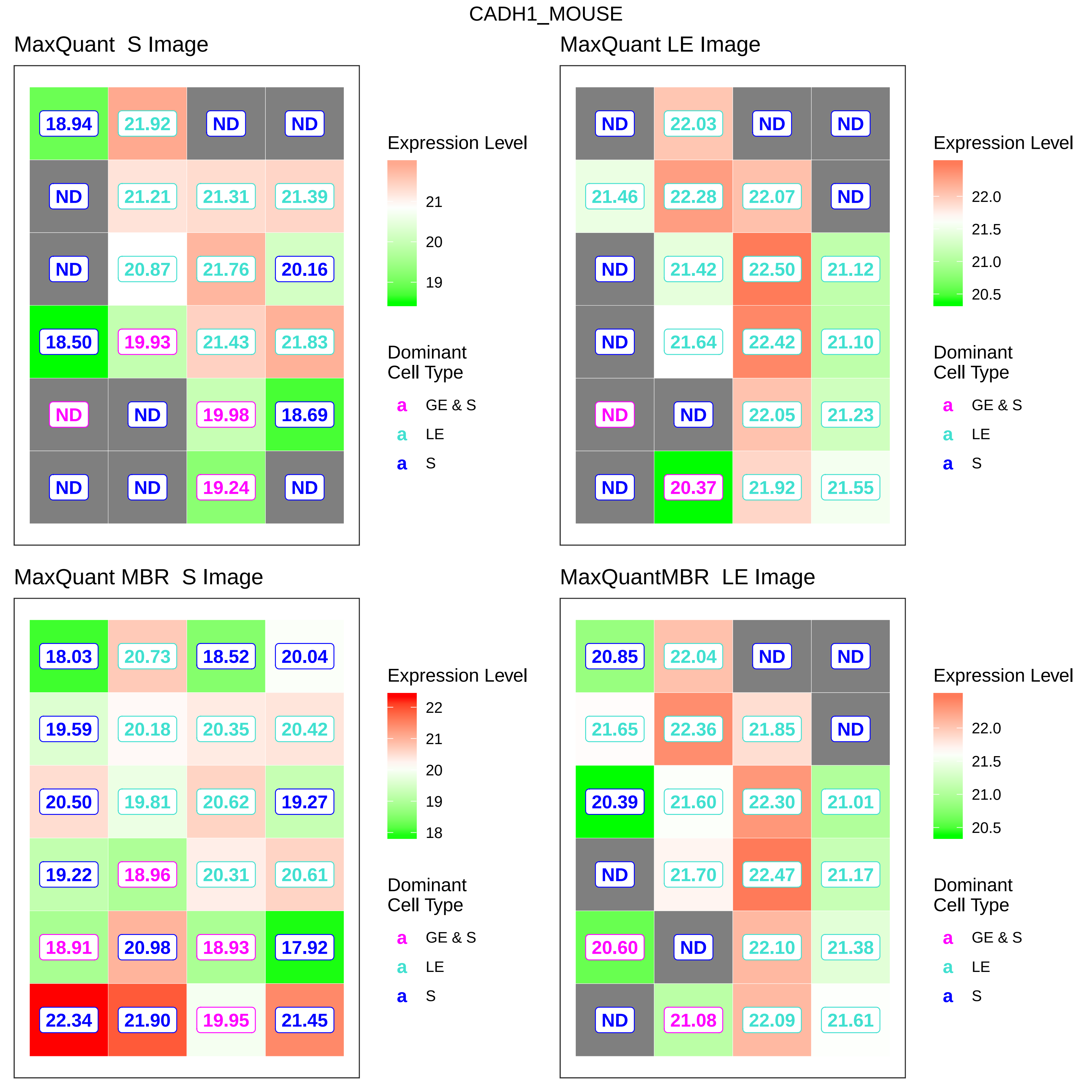

CADH1_MOUSE
MaxQuant S Image
18.94 21.92 ND ND
ND 21.21 21.31 21.39
ND 20.87 21.76 20.16
18.50 19.93 21.43 21.83
ND ND 19.98 18.69
ND ND 19.24 ND
Expression Level
21
20
19
Dominant Cell Type
a GE & S
a LE
a S
MaxQuant LE Image
ND 22.03 ND ND
21.46 22.28 22.07 ND
ND 21.42 22.50 21.12
ND 21.64 22.42 21.10
ND ND 22.05 21.23
ND 20.37 21.92 21.55
Expression Level
22.0
21.5
21.0
20.5
Dominant Cell Type
a GE & S
a LE
a S
MaxQuant MBR S Image
18.03 20.73 18.52 20.04
19.59 20.18 20.35 20.42
20.50 19.81 20.62 19.27
19.22 18.96 20.31 20.61
18.91 20.98 18.93 17.92
22.34 21.90 19.95 21.45
Expression Level
22
21
20
19
18
Dominant Cell Type
a GE & S
a LE
a S
MaxQuantMBR LE Image
20.85 22.04 ND ND
21.65 22.36 21.85 ND
20.39 21.60 22.30 21.01
ND 21.70 22.47 21.17
20.60 ND 22.10 21.38
ND 21.08 22.09 21.61
Expression Level
22.0
21.5
21.0
20.5
Dominant Cell Type
a GE & S
a LE
a S

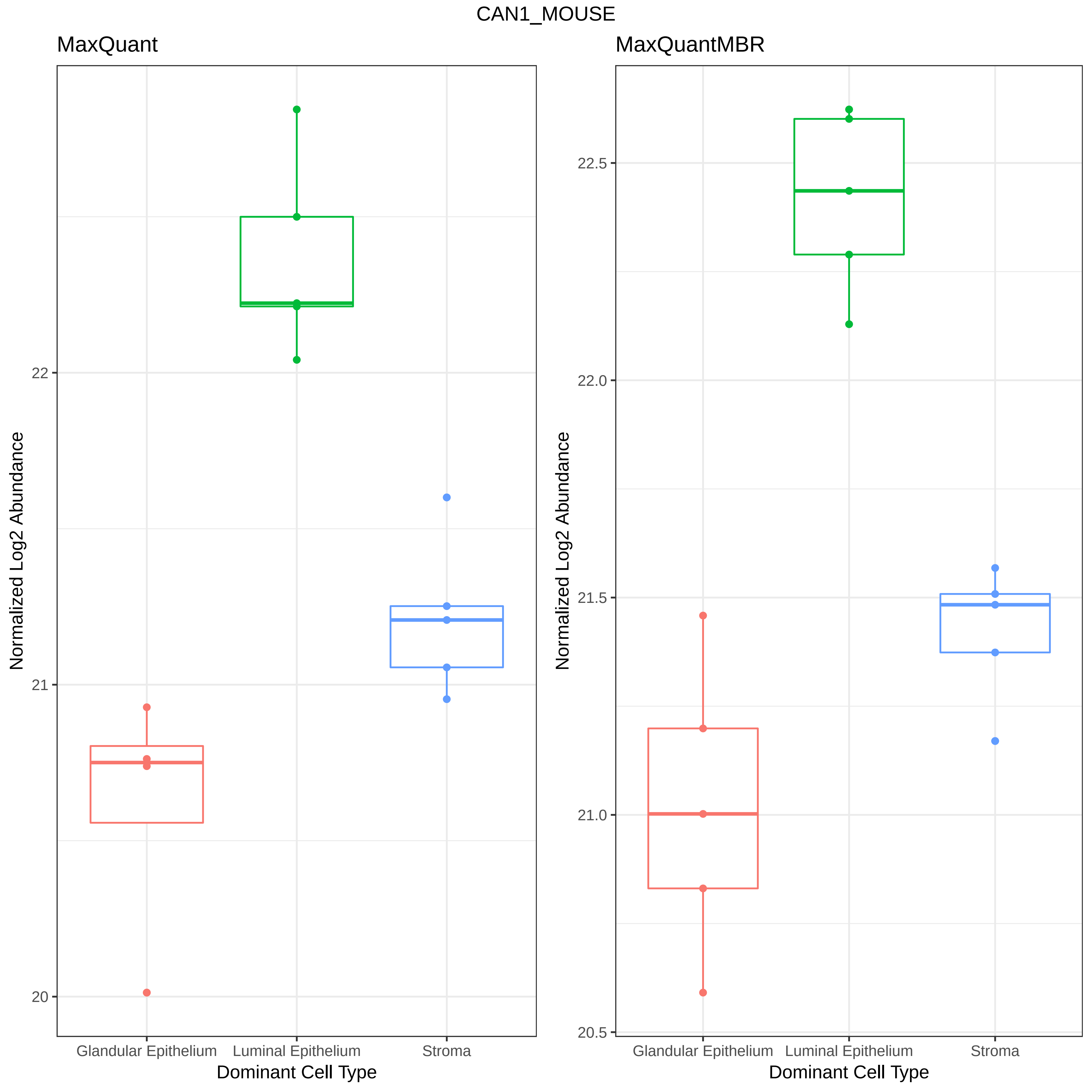
CAN1_MOUSE
MaxQuant
MaxQuantMBR
Normalized Log2 Abundance
22
21
20
22.5
22.0
21.5
21.0
20.5
Glandular Epithelium
Luminal Epithelium
Stroma
Dominant Cell Type

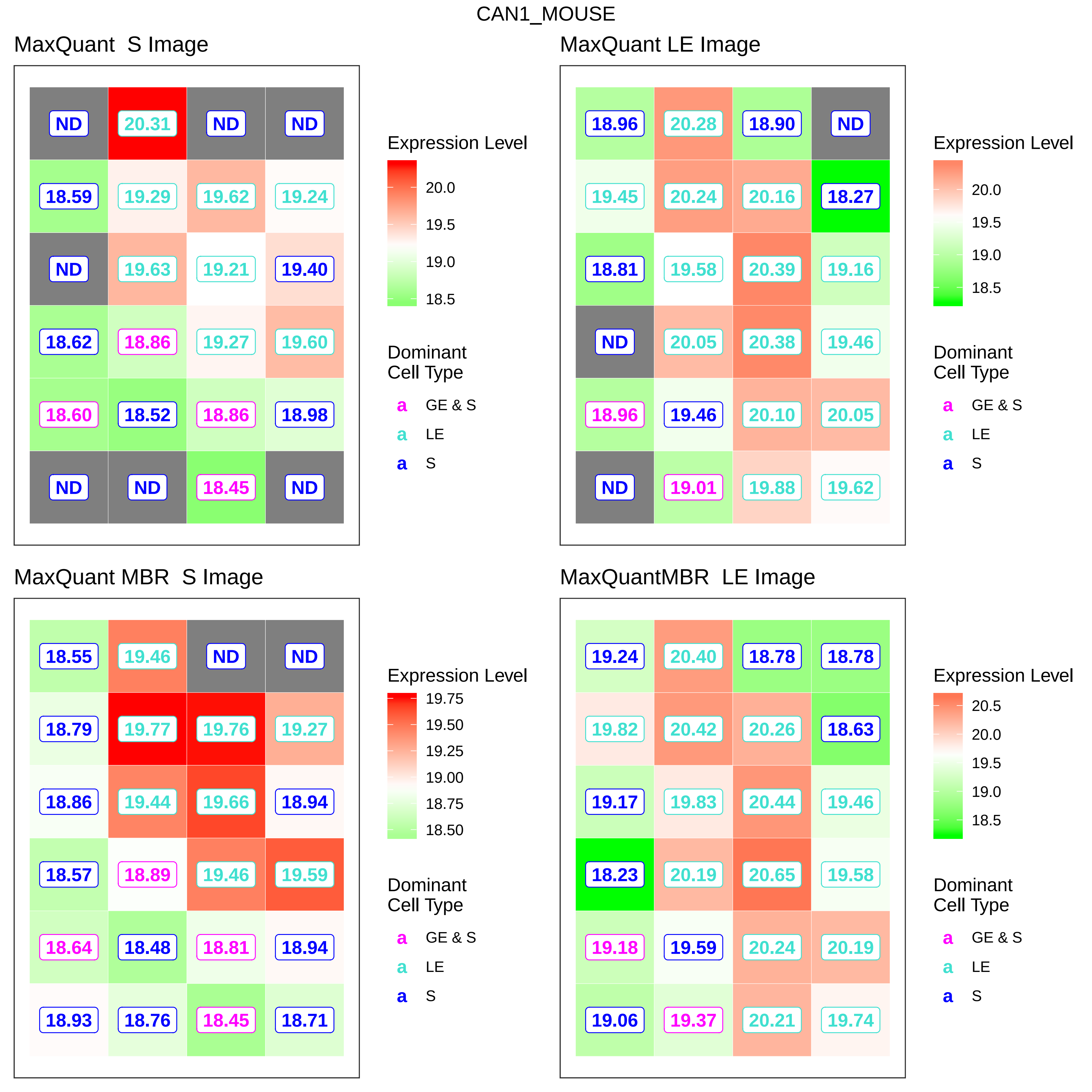
CAN1_MOUSE
MaxQuant S Image
ND 20.31 ND ND
18.59 19.29 19.62 19.24
ND 19.63 19.21 19.40
18.62 18.86 19.27 19.60
18.60 18.52 18.86 18.98
ND ND 18.45 ND
Expression Level
20.0
19.5
19.0
18.5
Dominant Cell Type
a GE & S
a LE
a S
MaxQuant LE Image
18.96 20.28 18.90 ND
19.45 20.24 20.16 18.27
18.81 19.58 20.39 19.16
ND 20.05 20.38 19.46
18.96 19.46 20.10 20.05
ND 19.01 19.88 19.62
Expression Level
20.0
19.5
19.0
18.5
Dominant Cell Type
a GE & S
a LE
a S
MaxQuant MBR S Image
18.55 19.46 ND ND
18.79 19.77 19.76 19.27
18.86 19.44 19.66 18.94
18.57 18.89 19.46 19.59
18.64 18.48 18.81 18.94
18.93 18.76 18.45 18.71
Expression Level
19.75
19.50
19.25
19.00
18.75
18.50
Dominant Cell Type
a GE & S
a LE
a S
MaxQuantMBR LE Image
19.24 20.40 18.78 18.78
19.82 20.42 20.26 18.63
19.17 19.83 20.44 19.46
18.23 20.19 20.65 19.58
19.18 19.59 20.24 20.19
19.06 19.37 20.21 19.74
Expression Level
20.5
20.0
19.5
19.0
18.5
Dominant Cell Type
a GE & S
a LE
a S

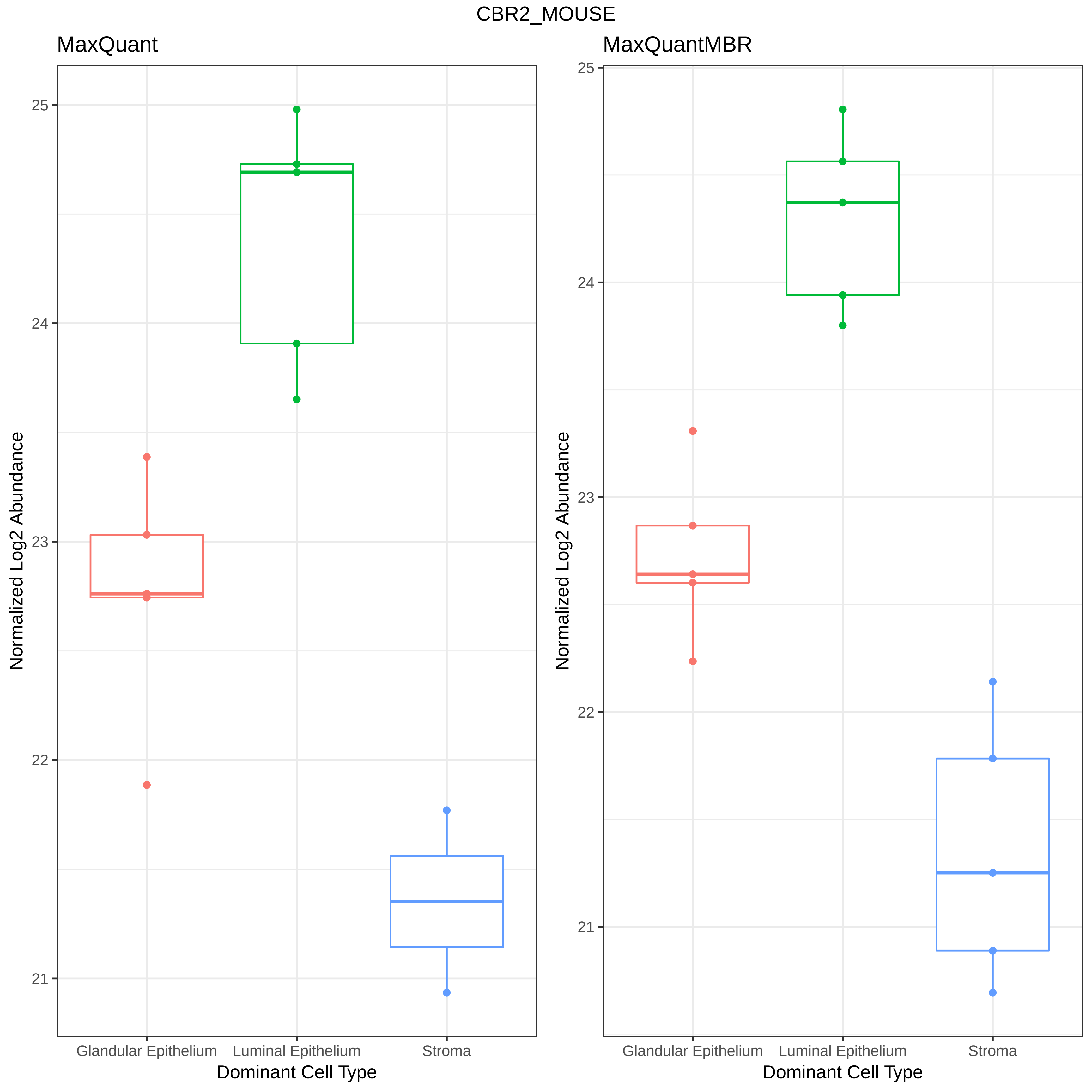
CBR2_MOUSE
MaxQuant
MaxQuantMBR
Normalized Log2 Abundance
Dominant Cell Type
Glandular Epithelium
Luminal Epithelium
Stroma
25
24
23
22
21

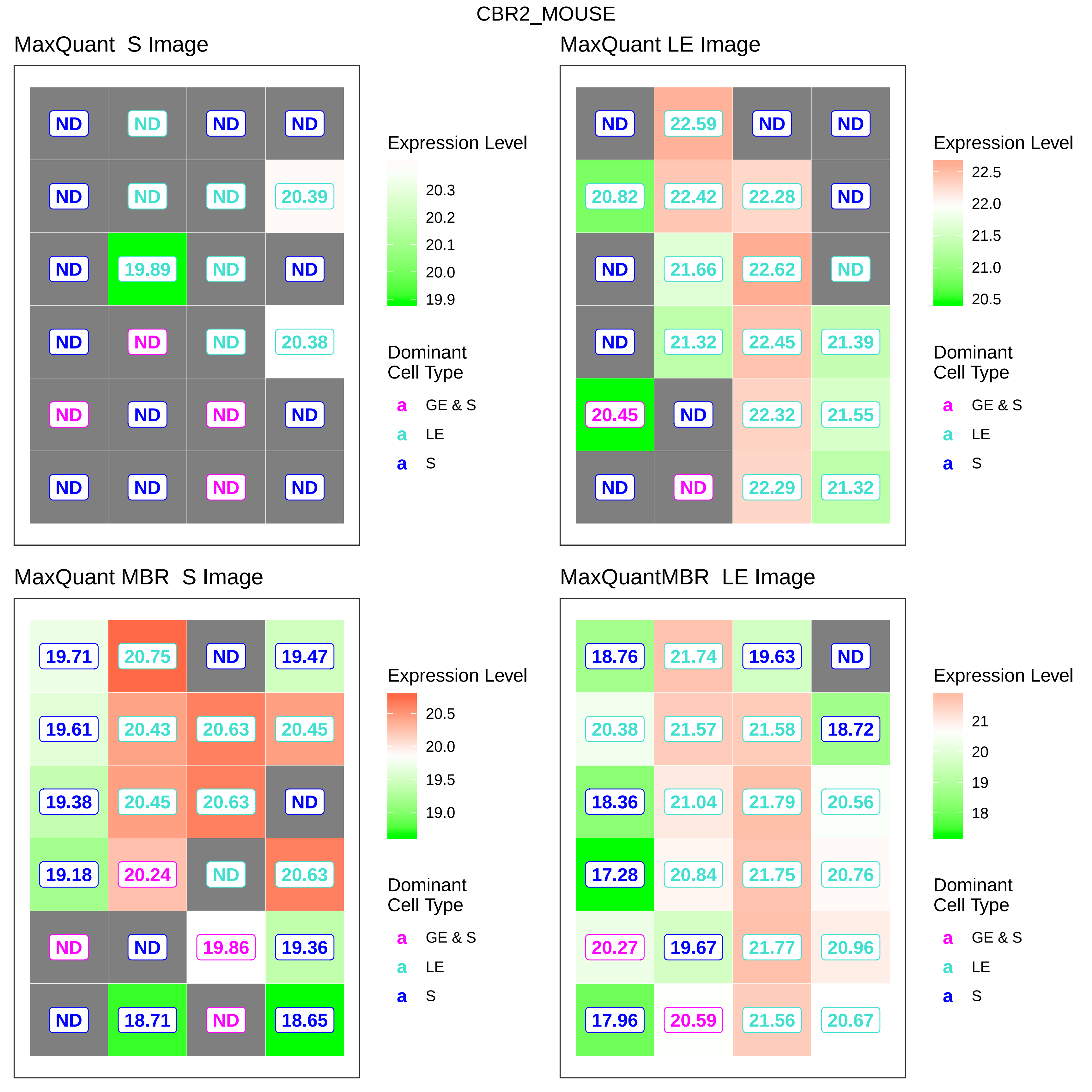
CBR2_MOUSE
MaxQuant S Image
ND ND ND ND
ND ND ND 20.39
ND 19.89 ND ND
ND ND ND 20.38
ND ND ND ND
ND ND ND ND
Expression Level
20.3
20.2
20.1
20.0
19.9
Dominant Cell Type
a GE & S
a LE
a S
MaxQuant LE Image
ND 22.59 ND ND
20.82 22.42 22.28 ND
ND 21.66 22.62 ND
ND 21.32 22.45 21.39
20.45 ND 22.32 21.55
ND ND 22.29 21.32
Expression Level
22.5
22.0
21.5
21.0
20.5
Dominant Cell Type
a GE & S
a LE
a S
MaxQuant MBR S Image
19.71 20.75 ND 19.47
19.61 20.43 20.63 20.45
19.38 20.45 20.63 ND
19.18 20.24 ND 20.63
ND ND 19.86 19.36
ND 18.71 ND 18.65
Expression Level
20.5
20.0
19.5
19.0
Dominant Cell Type
a GE & S
a LE
a S
MaxQuantMBR LE Image
18.76 21.74 19.63 ND
20.38 21.57 21.58 18.72
18.36 21.04 21.79 20.56
17.28 20.84 21.75 20.76
20.27 19.67 21.77 20.96
17.96 20.59 21.56 20.67
Expression Level
21
20
19
18
Dominant Cell Type
a GE & S
a LE
a S

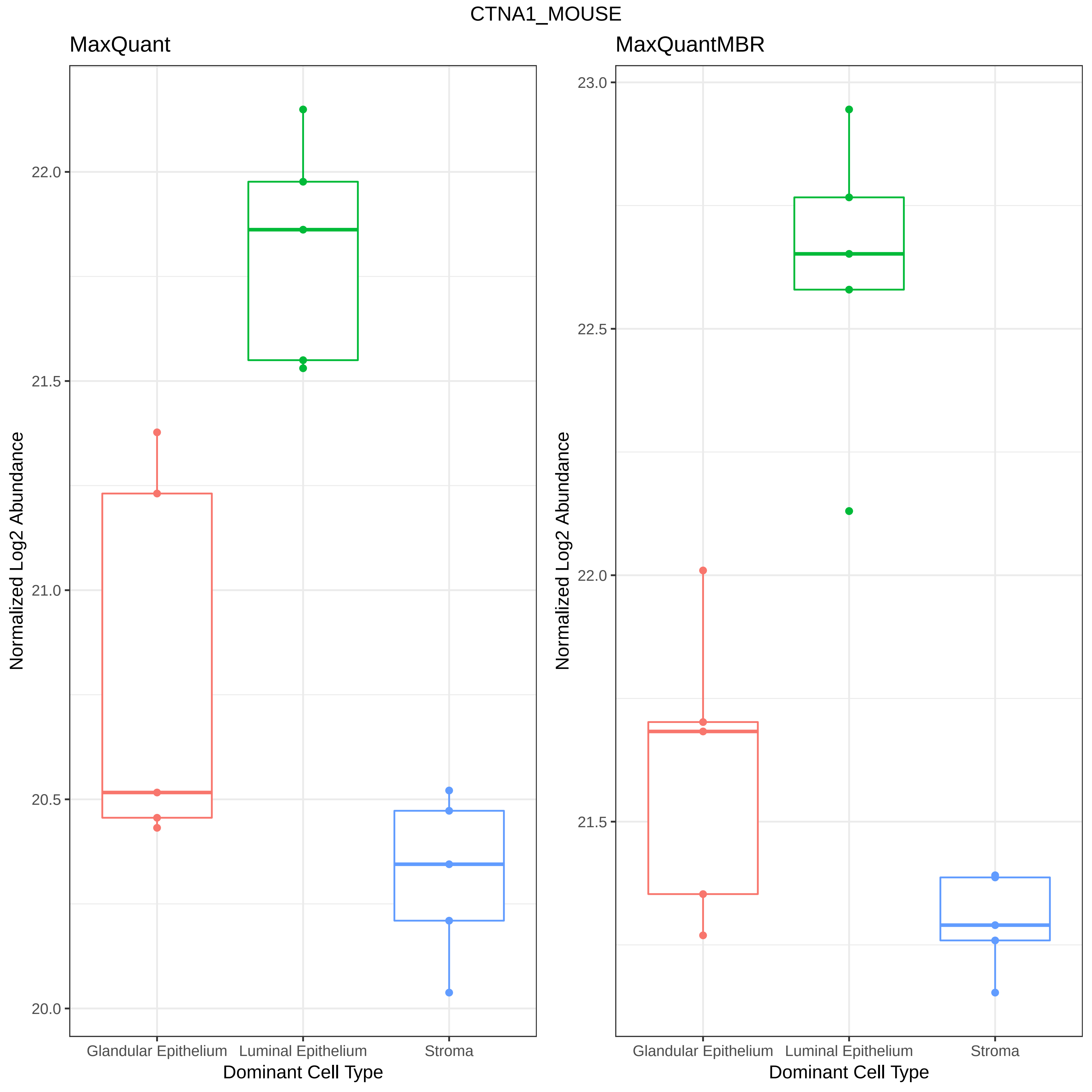

CTNA1_MOUSE
MaxQuant
MaxQuantMBR
Normalized Log2 Abundance
22.0
21.5
21.0
20.5
20.0
23.0
22.5
22.0
21.5
Glandular Epithelium
Luminal Epithelium
Stroma
Dominant Cell Type

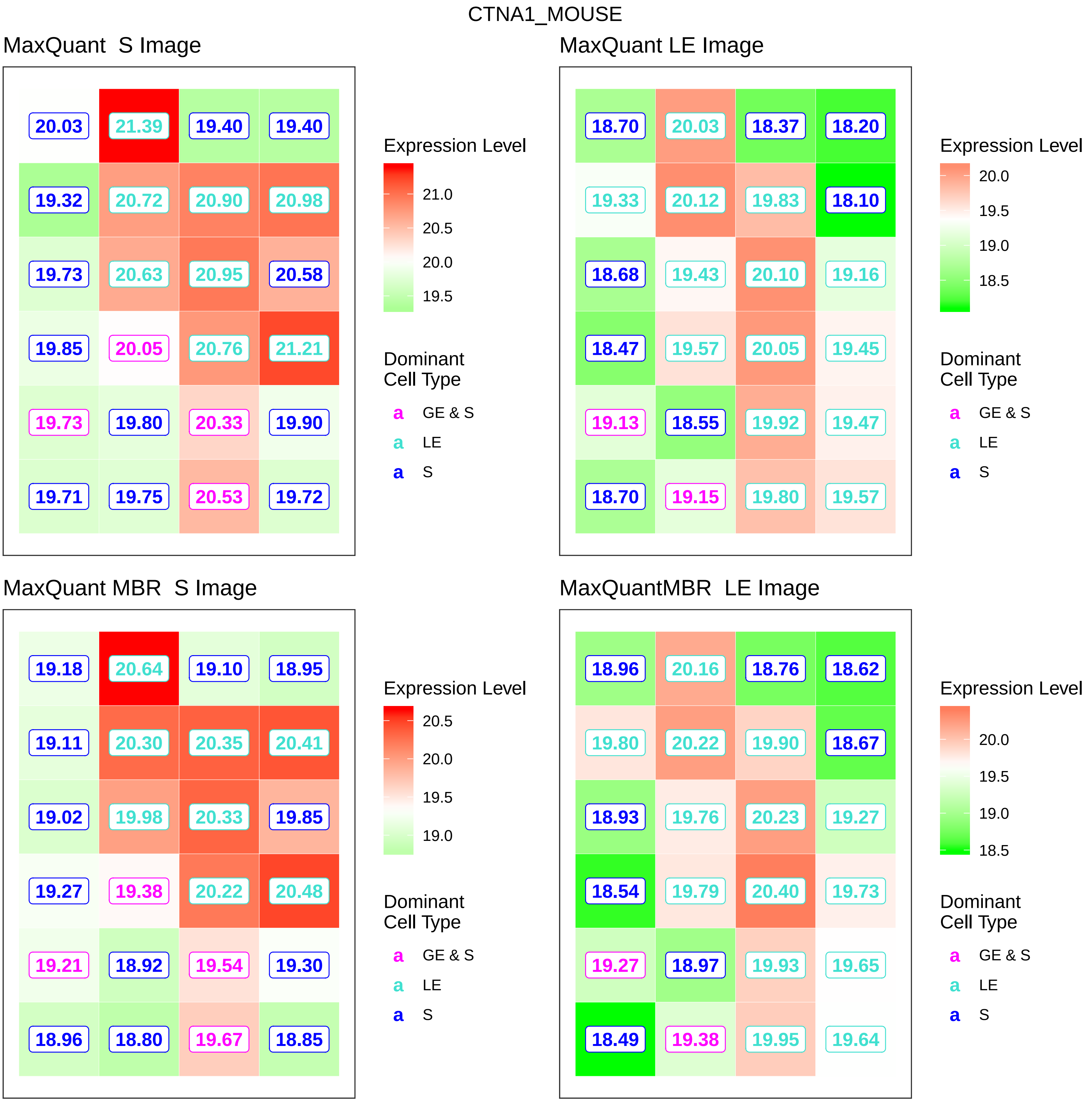

CTNA1_MOUSE
MaxQuant S Image
20.03 21.39 19.40 19.40
19.32 20.72 20.90 20.98
19.73 20.63 20.95 20.58
19.85 20.05 20.76 21.21
19.73 19.80 20.33 19.90
19.71 19.75 20.53 19.72
Expression Level
21.0
20.5
20.0
19.5
Dominant Cell Type
a GE & S
a LE
a S
MaxQuant LE Image
18.70 20.03 18.37 18.20
19.33 20.12 19.83 18.10
18.68 19.43 20.10 19.16
18.47 19.57 20.05 19.45
19.13 18.55 19.92 19.47
18.70 19.15 19.80 19.57
Expression Level
20.0
19.5
19.0
18.5
Dominant Cell Type
a GE & S
a LE
a S
MaxQuant MBR S Image
19.18 20.64 19.10 18.95
19.11 20.30 20.35 20.41
19.02 19.98 20.33 19.85
19.27 19.38 20.22 20.48
19.21 18.92 19.54 19.30
18.96 18.80 19.67 18.85
Expression Level
20.5
20.0
19.5
19.0
Dominant Cell Type
a GE & S
a LE
a S
MaxQuantMBR LE Image
18.96 20.16 18.76 18.62
19.80 20.22 19.90 18.67
18.93 19.76 20.23 19.27
18.54 19.79 20.40 19.73
19.27 18.97 19.93 19.65
18.49 19.38 19.95 19.64
Expression Level
20.0
19.5
19.0
18.5
Dominant Cell Type
a GE & S
a LE
a S

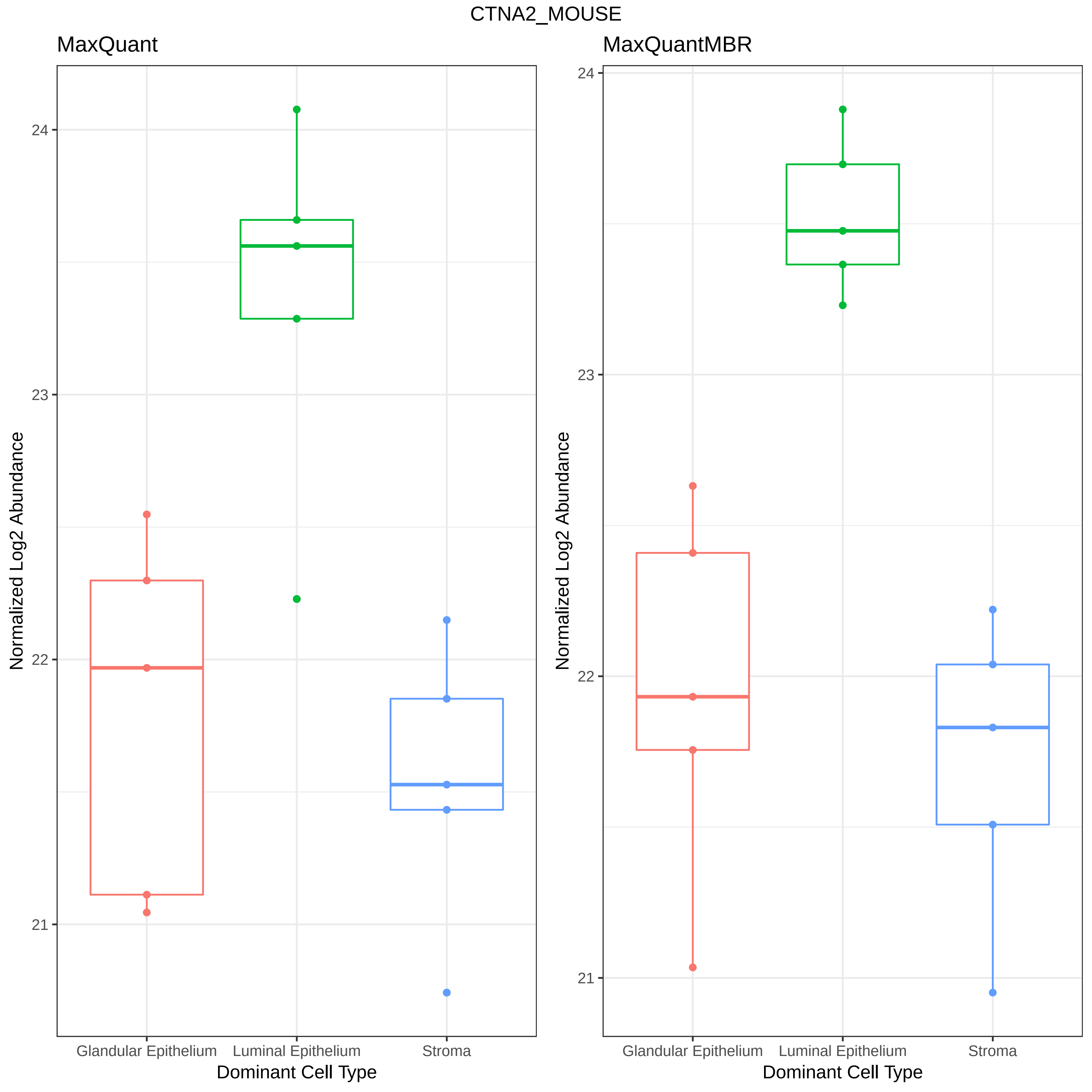
CTNA2_MOUSE
MaxQuant
MaxQuantMBR
Normalized Log2 Abundance
24
23
22
21
Glandular Epithelium
Luminal Epithelium
Stroma
Dominant Cell Type

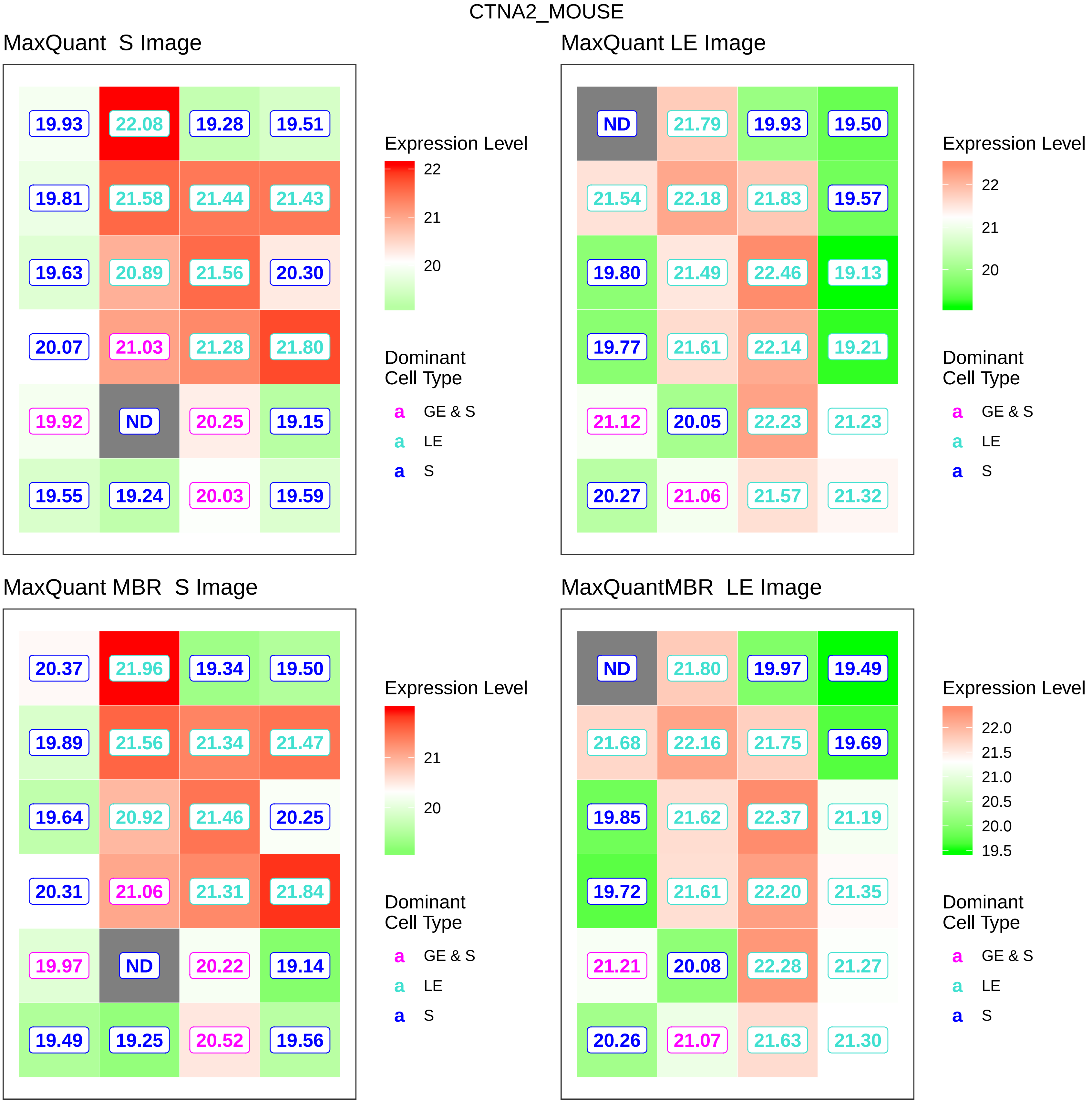
CTNA2_MOUSE
MaxQuant S Image
19.93 22.08 19.28 19.51
19.81 21.58 21.44 21.43
19.63 20.89 21.56 20.30
20.07 21.03 21.28 21.80
19.92 ND 20.25 19.15
19.55 19.24 20.03 19.59
Expression Level
22
21
20
Dominant Cell Type
a GE & S
a LE
a S
MaxQuant LE Image
ND 21.79 19.93 19.50
21.54 22.18 21.83 19.57
19.80 21.49 22.46 19.13
19.77 21.61 22.14 19.21
21.12 20.05 22.23 21.23
20.27 21.06 21.57 21.32
Expression Level
22
21
20
Dominant Cell Type
a GE & S
a LE
a S
MaxQuant MBR S Image
20.37 21.96 19.34 19.50
19.89 21.56 21.34 21.47
19.64 20.92 21.46 20.25
20.31 21.06 21.31 21.84
19.97 ND 20.22 19.14
19.49 19.25 20.52 19.56
Expression Level
21
20
Dominant Cell Type
a GE & S
a LE
a S
MaxQuantMBR LE Image
ND 21.80 19.97 19.49
21.68 22.16 21.75 19.69
19.85 21.62 22.37 21.19
19.72 21.61 22.20 21.35
21.21 20.08 22.28 21.27
20.26 21.07 21.63 21.30
Expression Level
22.0
21.5
21.0
20.5
20.0
19.5
Dominant Cell Type
a GE & S
a LE
a S

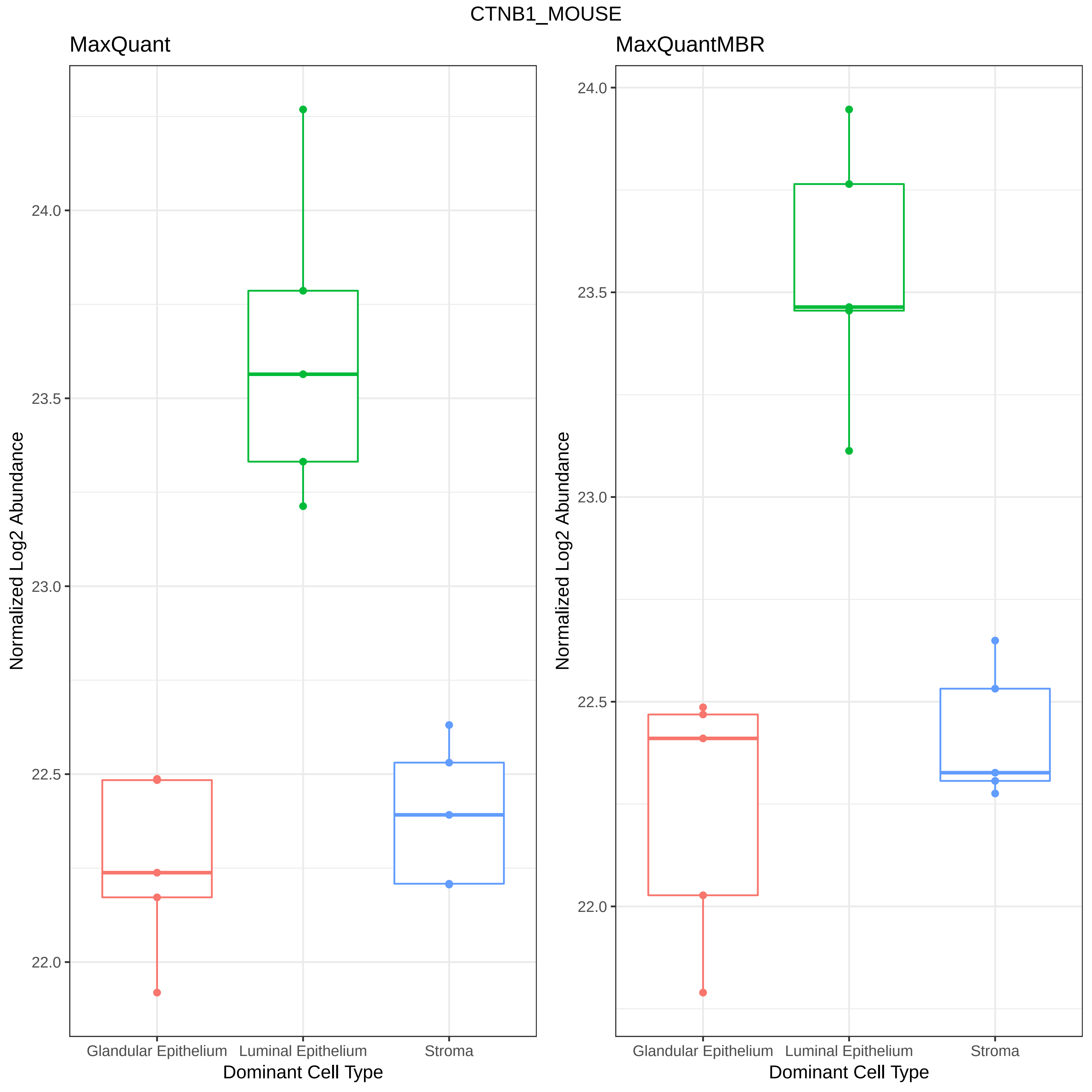
CTNB1_MOUSE
MaxQuant
MaxQuantMBR
Normalized Log2 Abundance
24.0
23.5
23.0
22.5
22.0
Glandular Epithelium
Luminal Epithelium
Stroma
Dominant Cell Type

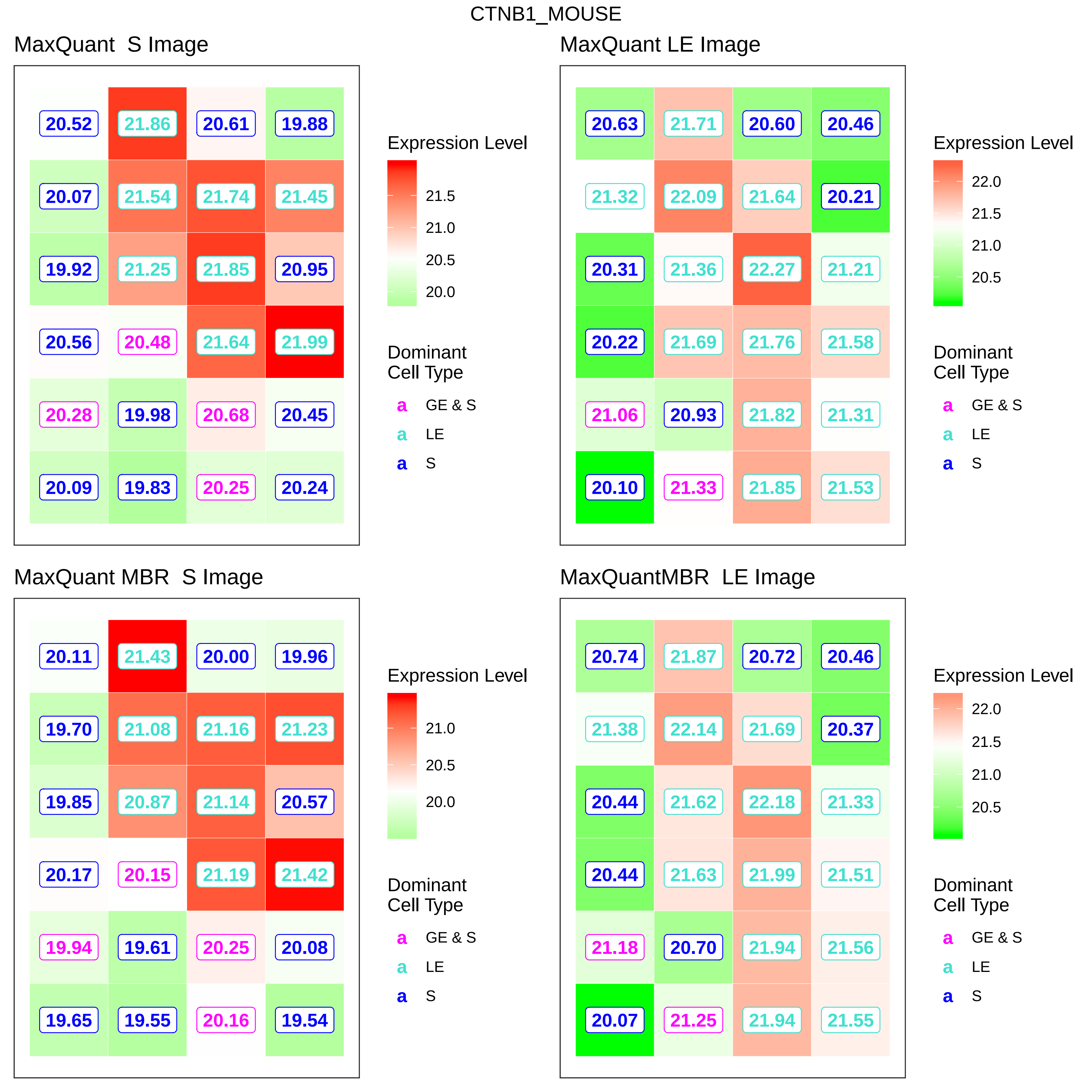

CTNB1_MOUSE
MaxQuant S Image
20.52 21.86 20.61 19.88
20.07 21.54 21.74 21.45
19.92 21.25 21.85 20.95
20.56 20.48 21.64 21.99
20.28 19.98 20.68 20.45
20.09 19.83 20.25 20.24
Expression Level
21.5
21.0
20.5
20.0
Dominant Cell Type
a GE & S
a LE
a S
MaxQuant LE Image
20.63 21.71 20.60 20.46
21.32 22.09 21.64 20.21
20.31 21.36 22.27 21.21
20.22 21.69 21.76 21.58
21.06 20.93 21.82 21.31
20.10 21.33 21.85 21.53
Expression Level
22.0
21.5
21.0
20.5
Dominant Cell Type
a GE & S
a LE
a S
MaxQuant MBR S Image
20.11 21.43 20.00 19.96
19.70 21.08 21.16 21.23
19.85 20.87 21.14 20.57
20.17 20.15 21.19 21.42
19.94 19.61 20.25 20.08
19.65 19.55 20.16 19.54
Expression Level
21.0
20.5
20.0
Dominant Cell Type
a GE & S
a LE
a S
MaxQuantMBR LE Image
20.74 21.87 20.72 20.46
21.38 22.14 21.69 20.37
20.44 21.62 22.18 21.33
20.44 21.63 21.99 21.51
21.18 20.70 21.94 21.56
20.07 21.25 21.94 21.55
Expression Level
22.0
21.5
21.0
20.5
Dominant Cell Type
a GE & S
a LE
a S

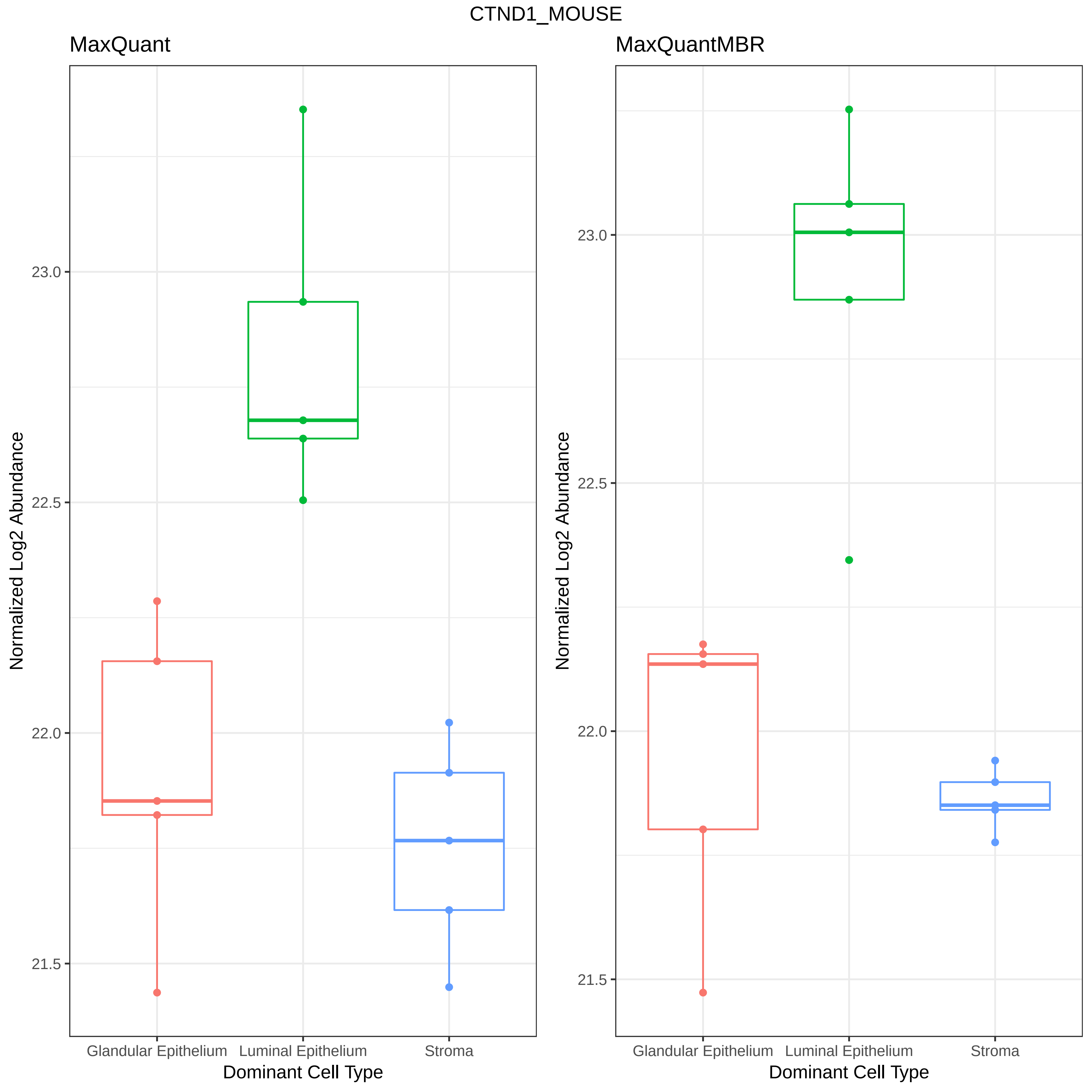

CTND1_MOUSE
MaxQuant
MaxQuantMBR
Normalized Log2 Abundance
23.0
22.5
22.0
21.5
Glandular Epithelium
Luminal Epithelium
Stroma
Dominant Cell Type

CTND1_MOUSE

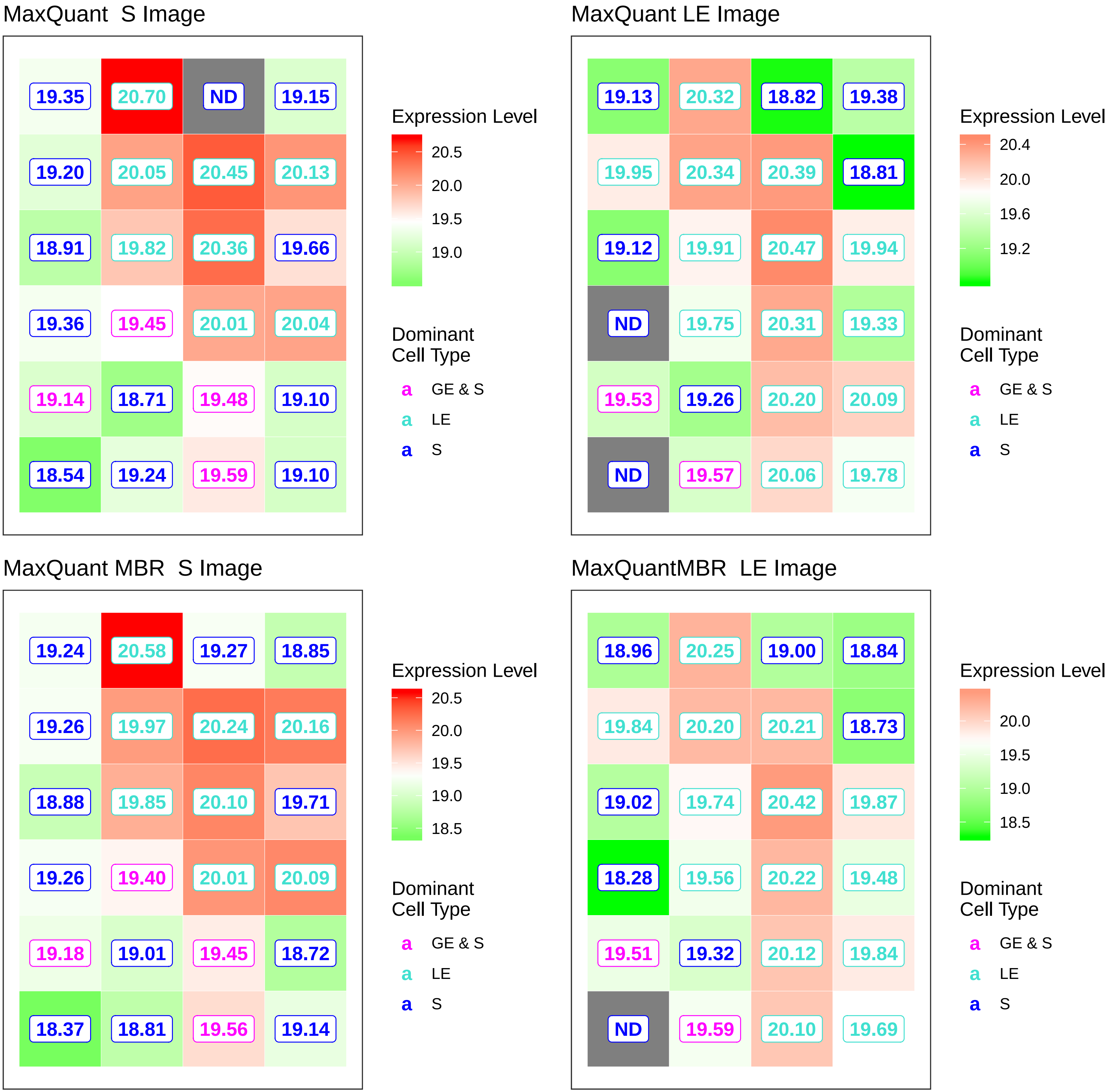
MaxQuant S Image
19.35 20.70 ND 19.15
19.20 20.05 20.45 20.13
18.91 19.82 20.36 19.66
19.36 19.45 20.01 20.04
19.14 18.71 19.48 19.10
18.54 19.24 19.59 19.10
Expression Level
20.5
20.0
19.5
19.0
Dominant Cell Type
a GE & S
a LE
a S
MaxQuant LE Image
19.13 20.32 18.82 19.38
19.95 20.34 20.39 18.81
19.12 19.91 20.47 19.94
ND 19.75 20.31 19.33
19.53 19.26 20.20 20.09
ND 19.57 20.06 19.78
Expression Level
20.4
20.0
19.6
19.2
Dominant Cell Type
a GE & S
a LE
a S
MaxQuant MBR S Image
19.24 20.58 19.27 18.85
19.26 19.97 20.24 20.16
18.88 19.85 20.10 19.71
19.26 19.40 20.01 20.09
19.18 19.01 19.45 18.72
18.37 18.81 19.56 19.14
Expression Level
20.5
20.0
19.5
19.0
18.5
Dominant Cell Type
a GE & S
a LE
a S
MaxQuantMBR LE Image
18.96 20.25 19.00 18.84
19.84 20.20 20.21 18.73
19.02 19.74 20.42 19.87
18.28 19.56 20.22 19.48
19.51 19.32 20.12 19.84
ND 19.59 20.10 19.69
Expression Level
20.0
19.5
19.0
18.5
Dominant Cell Type
a GE & S
a LE
a S

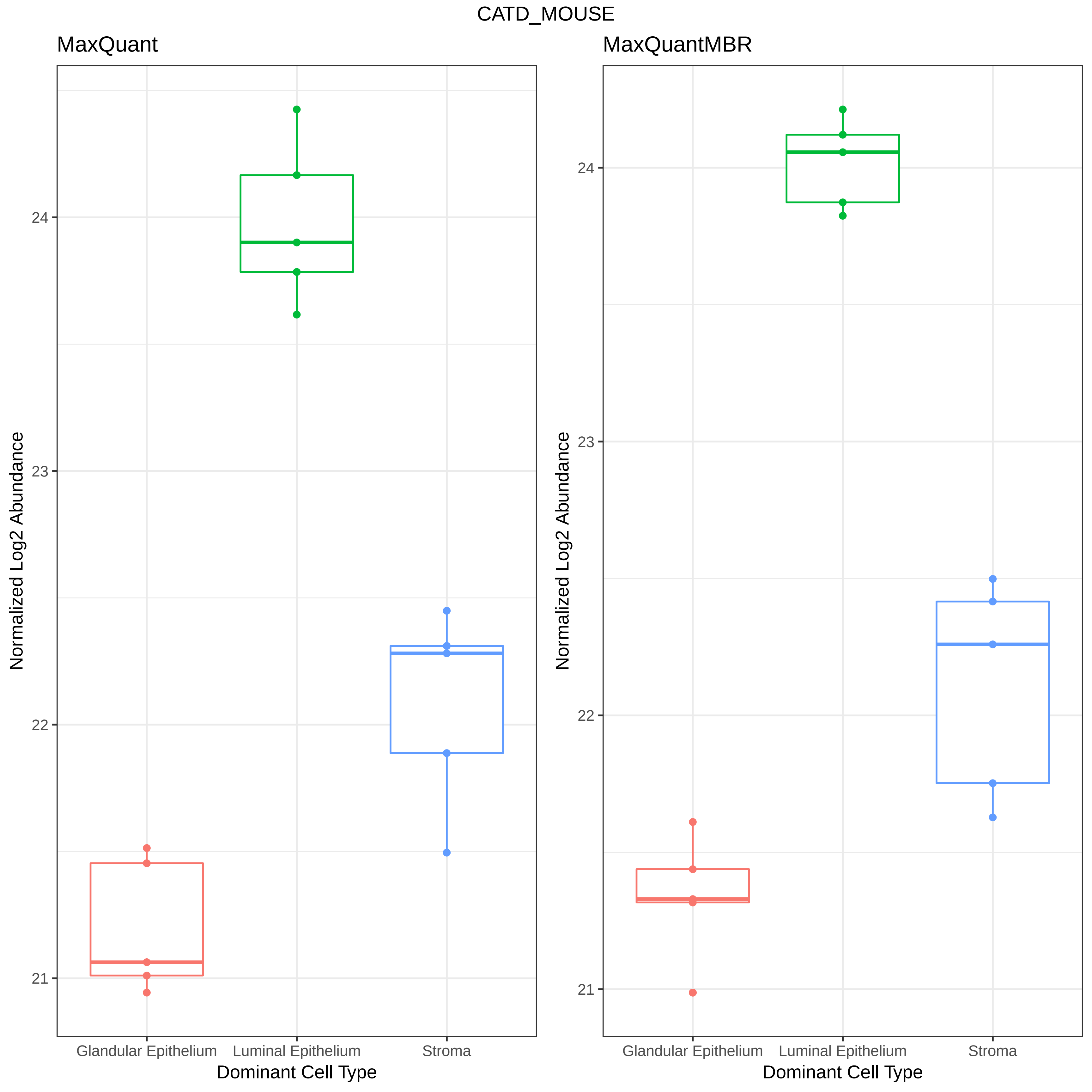
CATD_MOUSE
MaxQuant
MaxQuantMBR
Normalized Log2 Abundance
24
23
22
21
Glandular Epithelium
Luminal Epithelium
Stroma
Dominant Cell Type

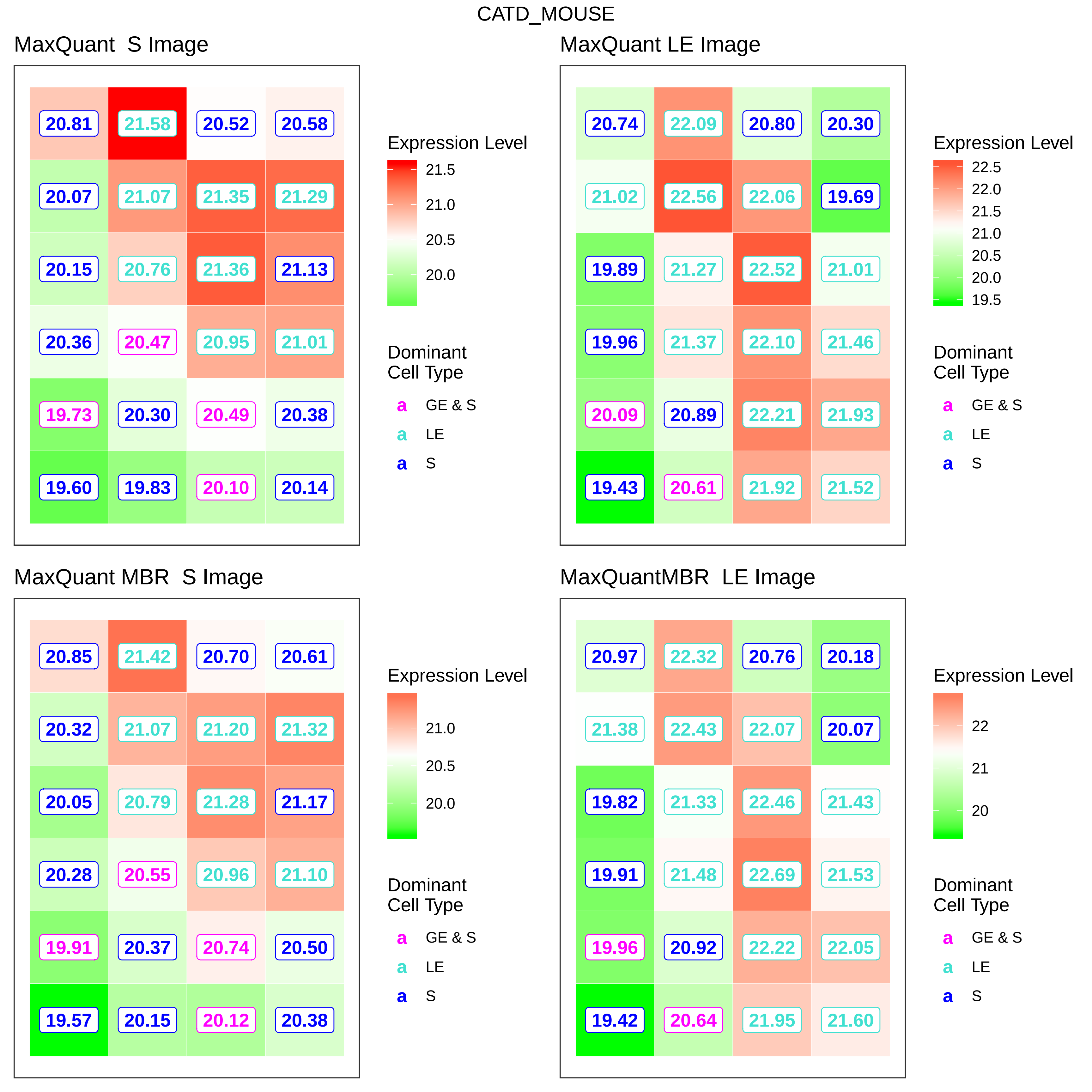
CATD_MOUSE
MaxQuant S Image
20.81 21.58 20.52 20.58
20.07 21.07 21.35 21.29
20.15 20.76 21.36 21.13
20.36 20.47 20.95 21.01
19.73 20.30 20.49 20.38
19.60 19.83 20.10 20.14
Expression Level
21.5
21.0
20.5
20.0
Dominant Cell Type
a GE & S
a LE
a S
MaxQuant LE Image
20.74 22.09 20.80 20.30
21.02 22.56 22.06 19.69
19.89 21.27 22.52 21.01
19.96 21.37 22.10 21.46
20.09 20.89 22.21 21.93
19.43 20.61 21.92 21.52
Expression Level
22.5
22.0
21.5
21.0
20.5
20.0
19.5
Dominant Cell Type
a GE & S
a LE
a S
MaxQuant MBR S Image
20.85 21.42 20.70 20.61
20.32 21.07 21.20 21.32
20.05 20.79 21.28 21.17
20.28 20.55 20.96 21.10
19.91 20.37 20.74 20.50
19.57 20.15 20.12 20.38
Expression Level
21.0
20.5
20.0
Dominant Cell Type
a GE & S
a LE
a S
MaxQuantMBR LE Image
20.97 22.32 20.76 20.18
21.38 22.43 22.07 20.07
19.82 21.33 22.46 21.43
19.91 21.48 22.69 21.53
19.96 20.92 22.22 22.05
19.42 20.64 21.95 21.60
Expression Level
22
21
20
Dominant Cell Type
a GE & S
a LE
a S

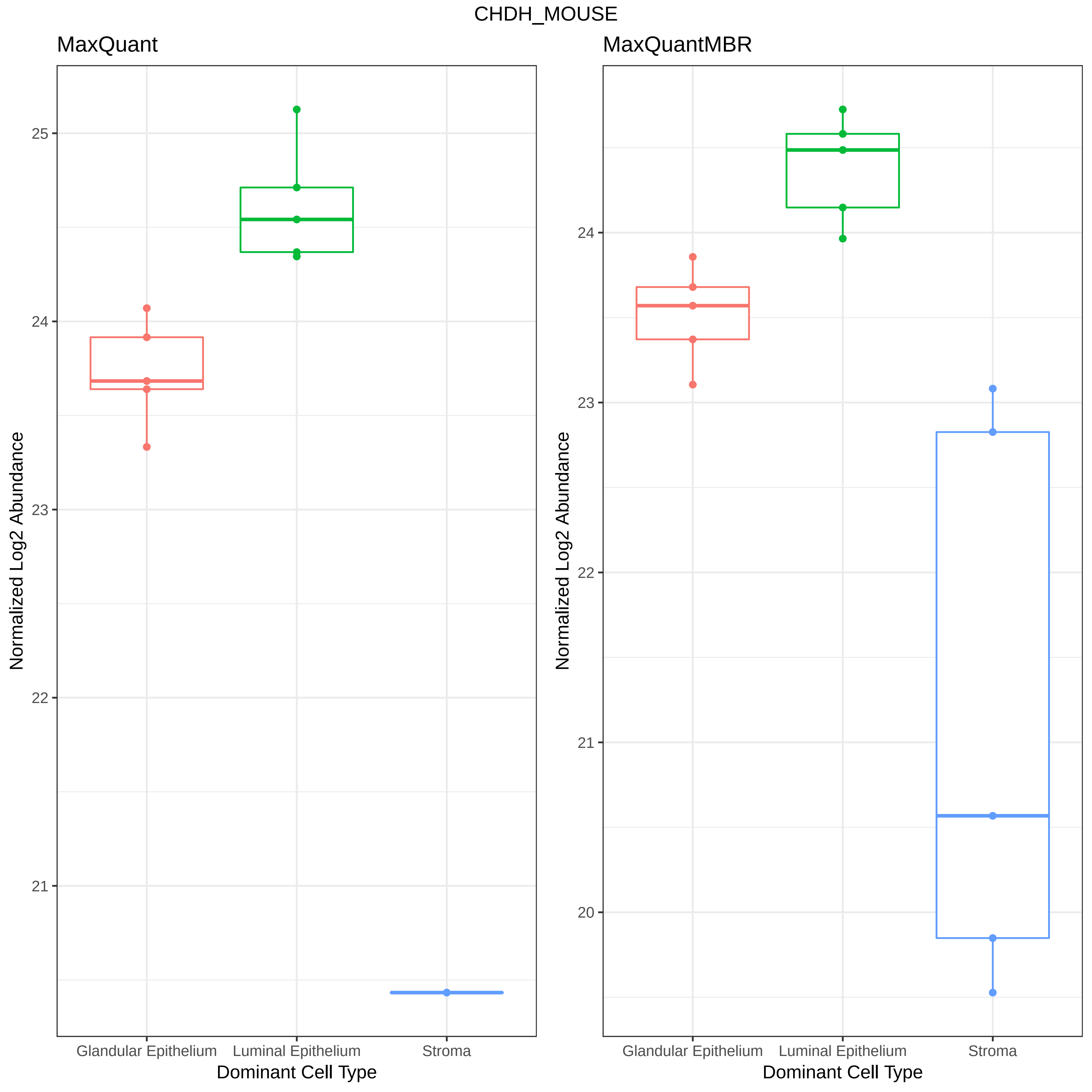
CHDH_MOUSE
MaxQuant
MaxQuantMBR
Normalized Log2 Abundance
25
24
23
22
21
20
Glandular Epithelium
Luminal Epithelium
Stroma
Dominant Cell Type

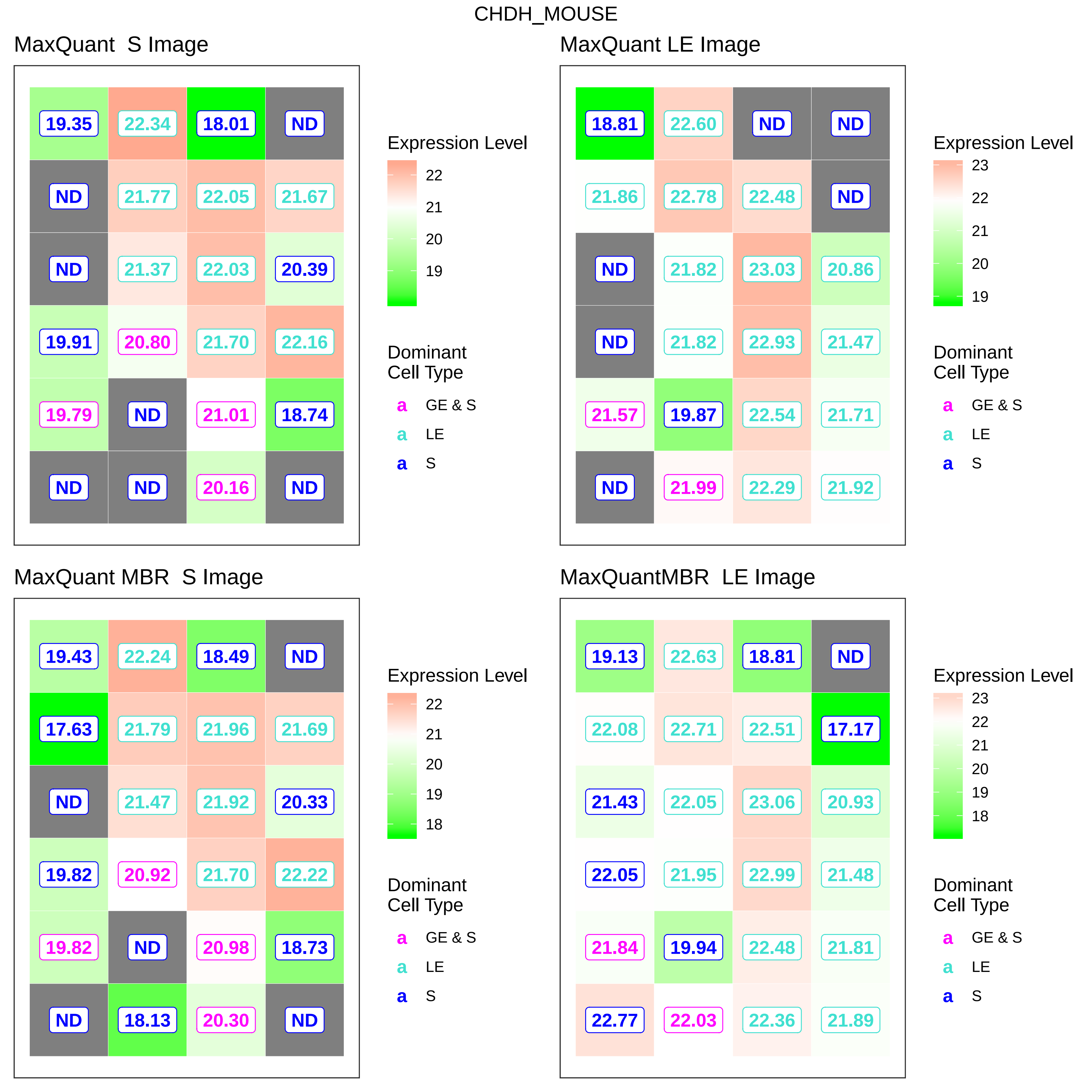
CHDH_MOUSE
MaxQuant S Image
19.35 22.34 18.01 ND
ND 21.77 22.05 21.67
ND 21.37 22.03 20.39
19.91 20.80 21.70 22.16
19.79 ND 21.01 18.74
ND ND 20.16 ND
Expression Level
22
21
20
19
Dominant Cell Type
a GE & S
a LE
a S
MaxQuant LE Image
18.81 22.60 ND ND
21.86 22.78 22.48 ND
ND 21.82 23.03 20.86
ND 21.82 22.93 21.47
21.57 19.87 22.54 21.71
ND 21.99 22.29 21.92
Expression Level
23
22
21
20
19
Dominant Cell Type
a GE & S
a LE
a S
MaxQuant MBR S Image
19.43 22.24 18.49 ND
17.63 21.79 21.96 21.69
ND 21.47 21.92 20.33
19.82 20.92 21.70 22.22
19.82 ND 20.98 18.73
ND 18.13 20.30 ND
Expression Level
22
21
20
19
18
Dominant Cell Type
a GE & S
a LE
a S
MaxQuantMBR LE Image
19.13 22.63 18.81 ND
22.08 22.71 22.51 17.17
21.43 22.05 23.06 20.93
22.05 21.95 22.99 21.48
21.84 19.94 22.48 21.81
22.77 22.03 22.36 21.89
Expression Level
23
22
21
20
19
18
Dominant Cell Type
a GE & S
a LE
a S

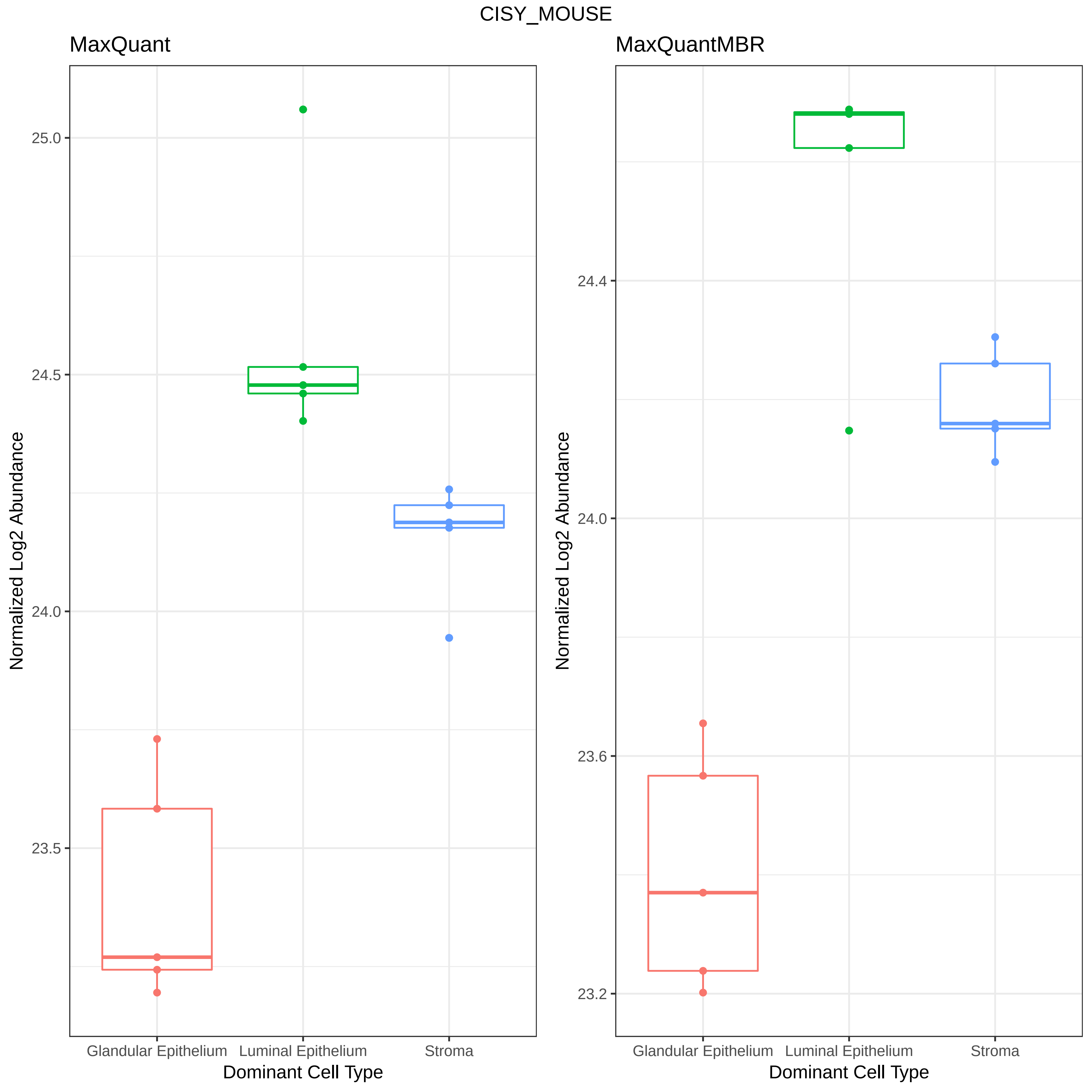

CISY_MOUSE
MaxQuant
MaxQuantMBR
Normalized Log2 Abundance
25.0
24.5
24.0
23.5
24.4
24.0
23.6
23.2
Glandular Epithelium
Luminal Epithelium
Stroma
Dominant Cell Type

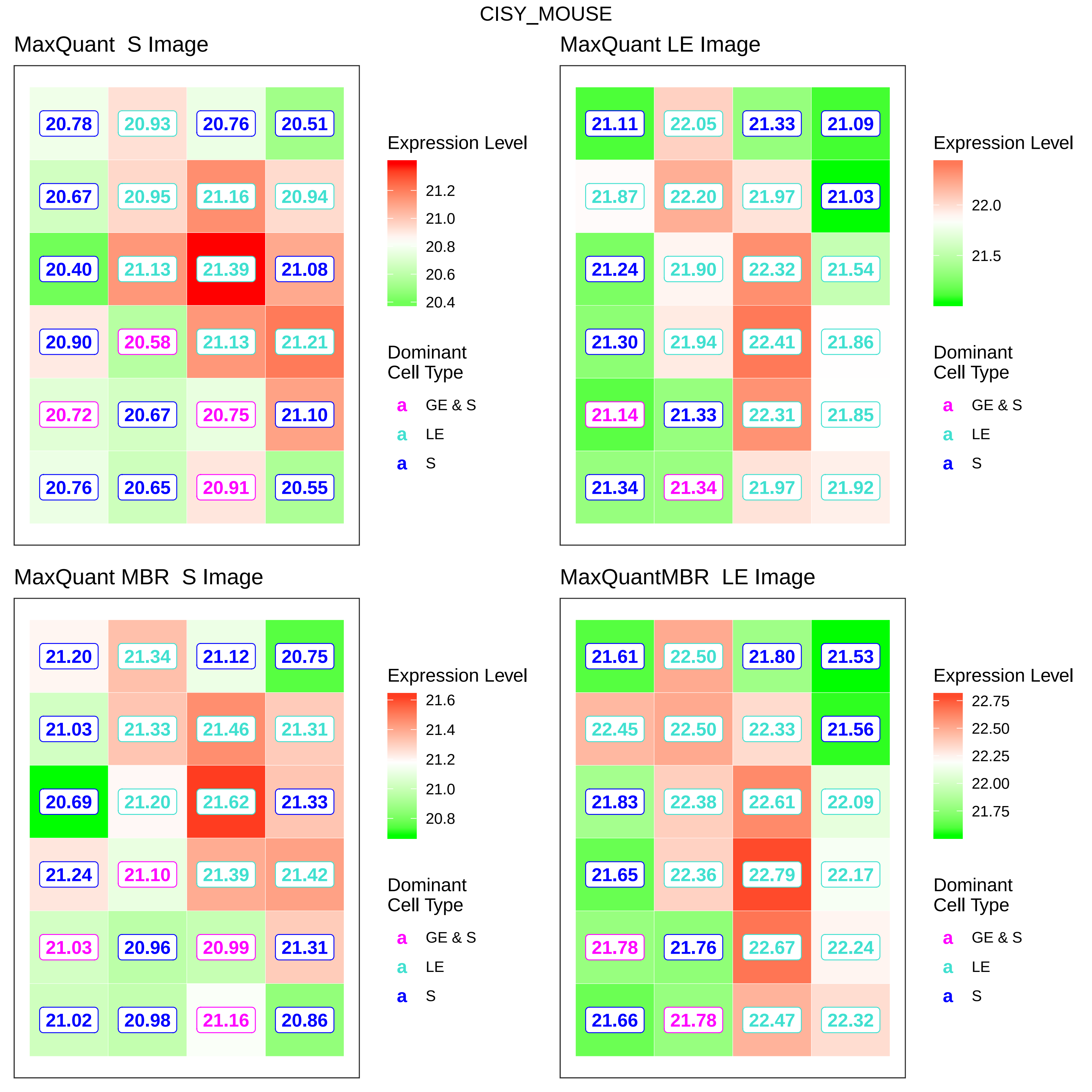
CISY_MOUSE
MaxQuant S Image
20.78 20.93 20.76 20.51
20.67 20.95 21.16 20.94
20.40 21.13 21.39 21.08
20.90 20.58 21.13 21.21
20.72 20.67 20.75 21.10
20.76 20.65 20.91 20.55
Expression Level
21.2
21.0
20.8
20.6
20.4
Dominant Cell Type
a GE & S
a LE
a S
MaxQuant LE Image
21.11 22.05 21.33 21.09
21.87 22.20 21.97 21.03
21.24 21.90 22.32 21.54
21.30 21.94 22.41 21.86
21.14 21.33 22.31 21.85
21.34 21.34 21.97 21.92
Expression Level
22.0
21.5
Dominant Cell Type
a GE & S
a LE
a S
MaxQuant MBR S Image
21.20 21.34 21.12 20.75
21.03 21.33 21.46 21.31
20.69 21.20 21.62 21.33
21.24 21.10 21.39 21.42
21.03 20.96 20.99 21.31
21.02 20.98 21.16 20.86
Expression Level
21.6
21.4
21.2
21.0
20.8
Dominant Cell Type
a GE & S
a LE
a S
MaxQuantMBR LE Image
21.61 22.50 21.80 21.53
22.45 22.50 22.33 21.56
21.83 22.38 22.61 22.09
21.65 22.36 22.79 22.17
21.78 21.76 22.67 22.24
21.66 21.78 22.47 22.32
Expression Level
22.75
22.50
22.25
22.00
21.75
Dominant Cell Type
a GE & S
a LE
a S

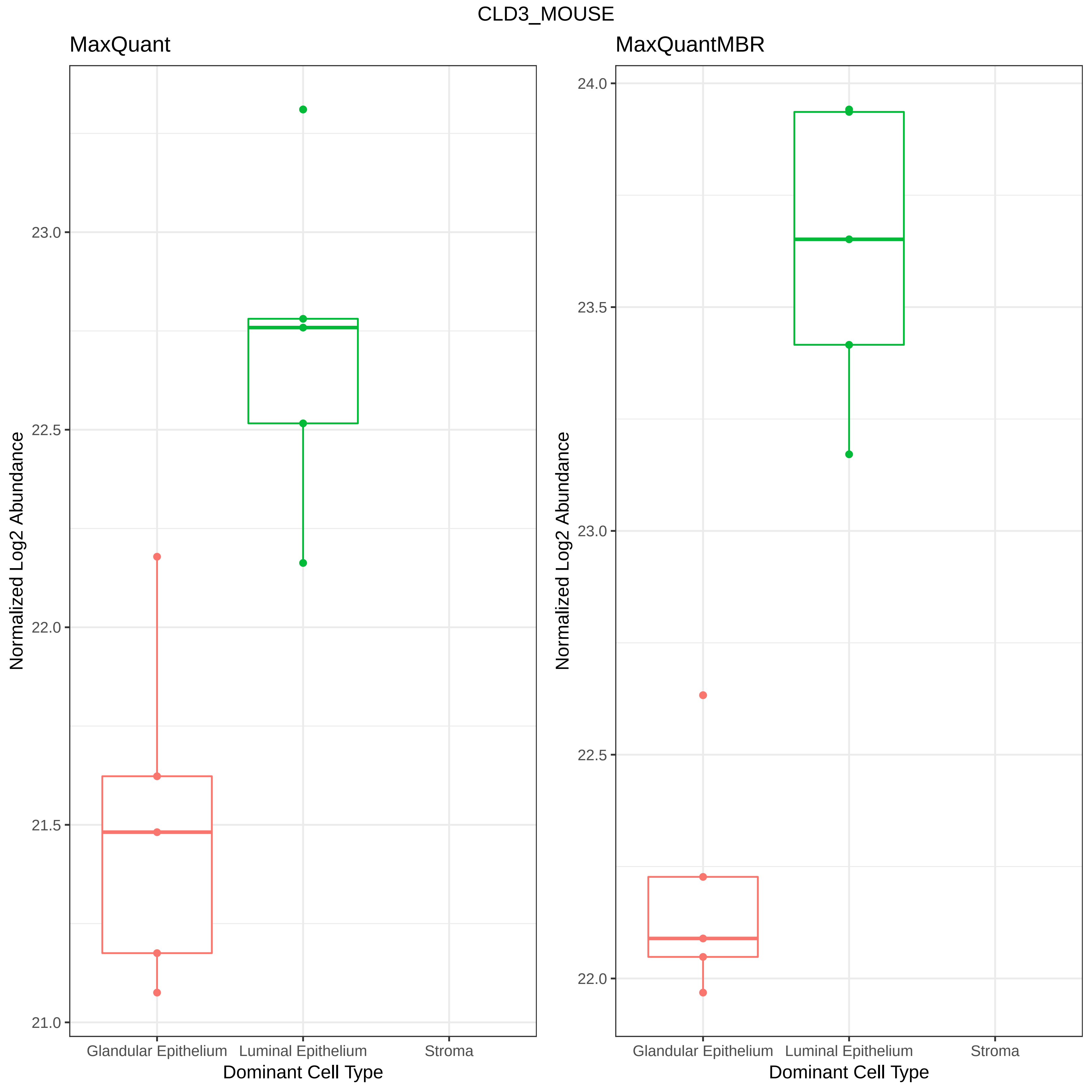

CLD3_MOUSE
MaxQuant
MaxQuantMBR
Normalized Log2 Abundance
21.0
21.5
22.0
22.5
23.0
23.5
24.0
Glandular Epithelium
Luminal Epithelium
Stroma
Dominant Cell Type

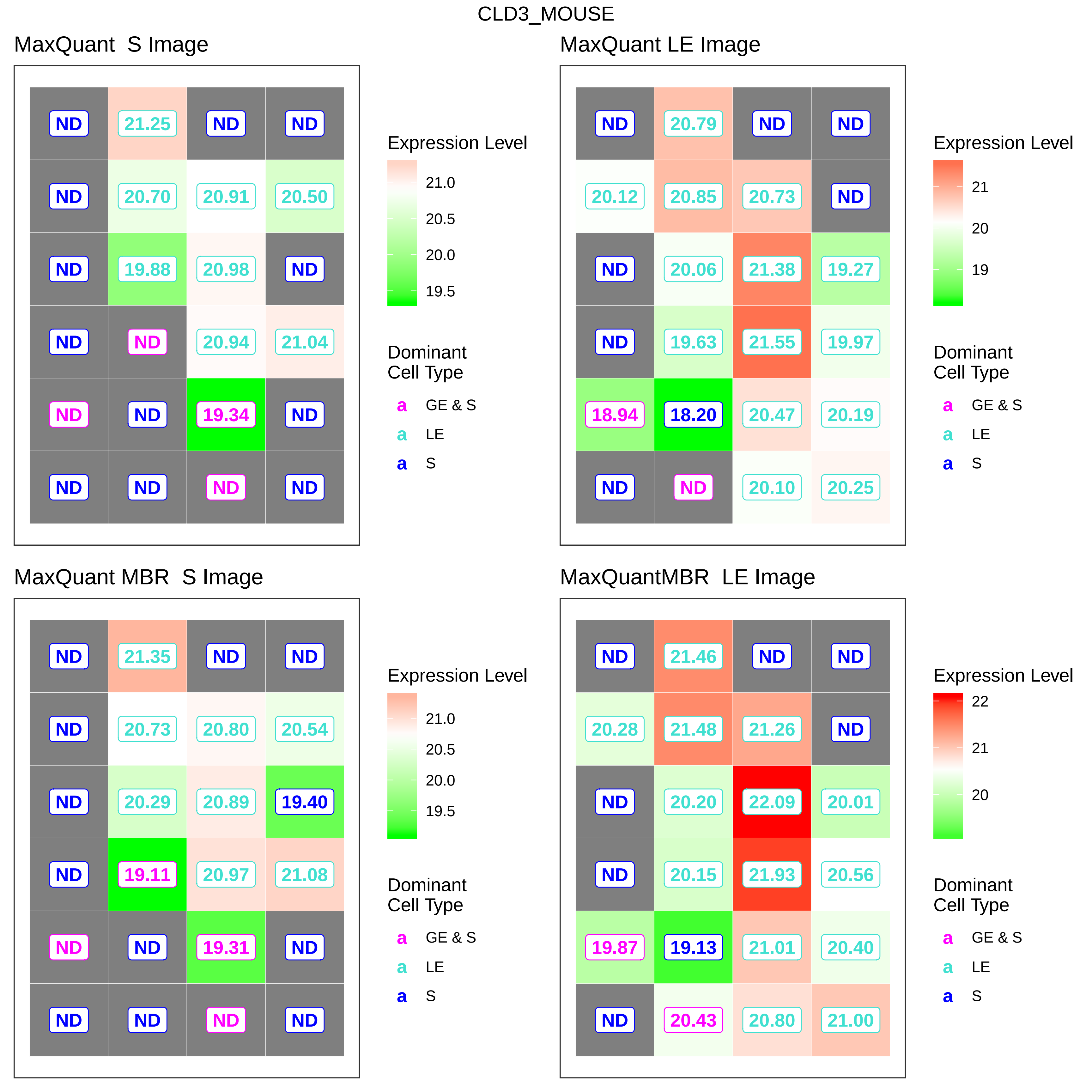
CLD3_MOUSE
MaxQuant S Image
ND 21.25 ND ND
ND 20.70 20.91 20.50
ND 19.88 20.98 ND
ND ND 20.94 21.04
ND ND 19.34 ND
ND ND ND ND
Expression Level
21.0
20.5
20.0
19.5
Dominant Cell Type
a GE & S
a LE
a S
MaxQuant LE Image
ND 20.79 ND ND
20.12 20.85 20.73 ND
ND 20.06 21.38 19.27
ND 19.63 21.55 19.97
18.94 18.20 20.47 20.19
ND ND 20.10 20.25
Expression Level
21
20
19
Dominant Cell Type
a GE & S
a LE
a S
MaxQuant MBR S Image
ND 21.35 ND ND
ND 20.73 20.80 20.54
ND 20.29 20.89 19.40
ND 19.11 20.97 21.08
ND ND 19.31 ND
ND ND ND ND
Expression Level
21.0
20.5
20.0
19.5
Dominant Cell Type
a GE & S
a LE
a S
MaxQuantMBR LE Image
ND 21.46 ND ND
20.28 21.48 21.26 ND
ND 20.20 22.09 20.01
ND 20.15 21.93 20.56
19.87 19.13 21.01 20.40
ND 20.43 20.80 21.00
Expression Level
22
21
20
Dominant Cell Type
a GE & S
a LE
a S

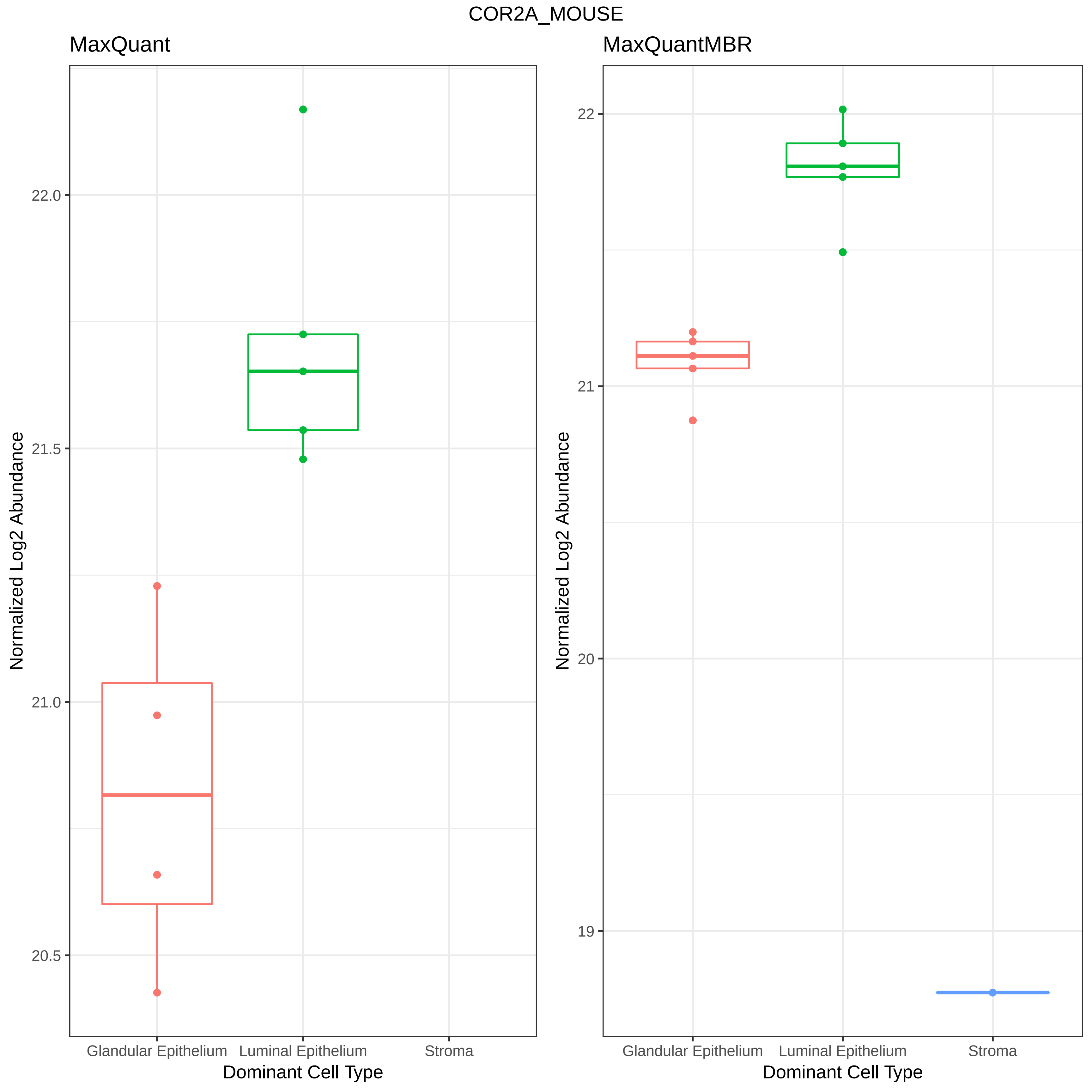
COR2A_MOUSE
MaxQuant
MaxQuantMBR
Normalized Log2 Abundance
Dominant Cell Type
Glandular Epithelium
Luminal Epithelium
Stroma
22.0
21.5
21.0
20.5
22
21
20
19

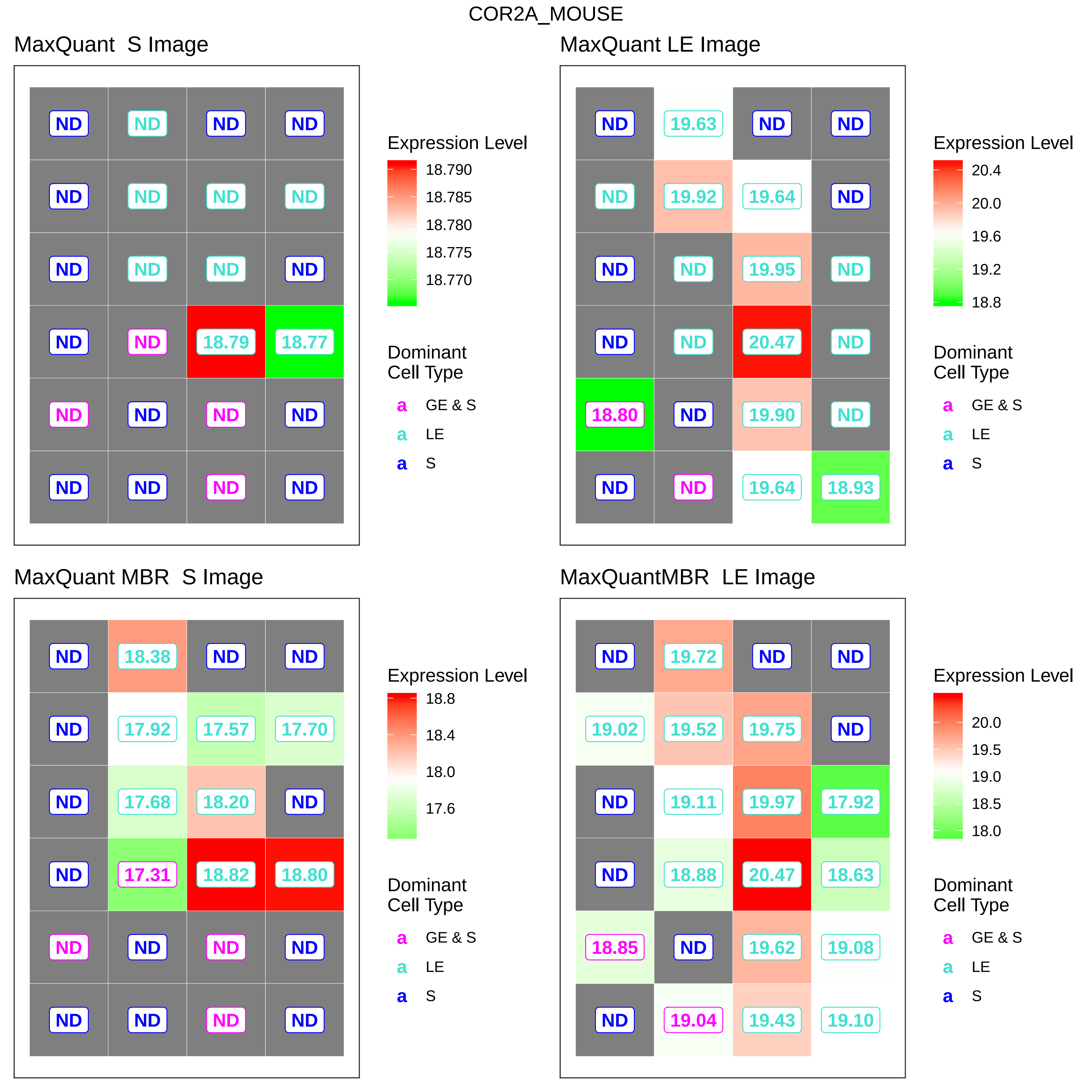

COR2A_MOUSE
MaxQuant S Image
ND ND ND ND
ND ND ND ND
ND ND ND ND
ND ND 18.79 18.77
ND ND ND ND
ND ND ND ND
Expression Level
18.790
18.785
18.780
18.775
18.770
Dominant Cell Type
a GE & S
a LE
a S
MaxQuant LE Image
ND 19.63 ND ND
ND 19.92 19.64 ND
ND ND 19.95 ND
ND ND 20.47 ND
18.80 ND 19.90 ND
ND ND 19.64 18.93
Expression Level
20.4
20.0
19.6
19.2
18.8
Dominant Cell Type
a GE & S
a LE
a S
MaxQuant MBR S Image
ND 18.38 ND ND
ND 17.92 17.57 17.70
ND 17.68 18.20 ND
ND 17.31 18.82 18.80
ND ND ND ND
ND ND ND ND
Expression Level
18.8
18.4
18.0
17.6
Dominant Cell Type
a GE & S
a LE
a S
MaxQuantMBR LE Image
ND 19.72 ND ND
19.02 19.52 19.75 ND
ND 19.11 19.97 17.92
ND 18.88 20.47 18.63
18.85 ND 19.62 19.08
ND 19.04 19.43 19.10
Expression Level
20.0
19.5
19.0
18.5
18.0
Dominant Cell Type
a GE & S
a LE
a S

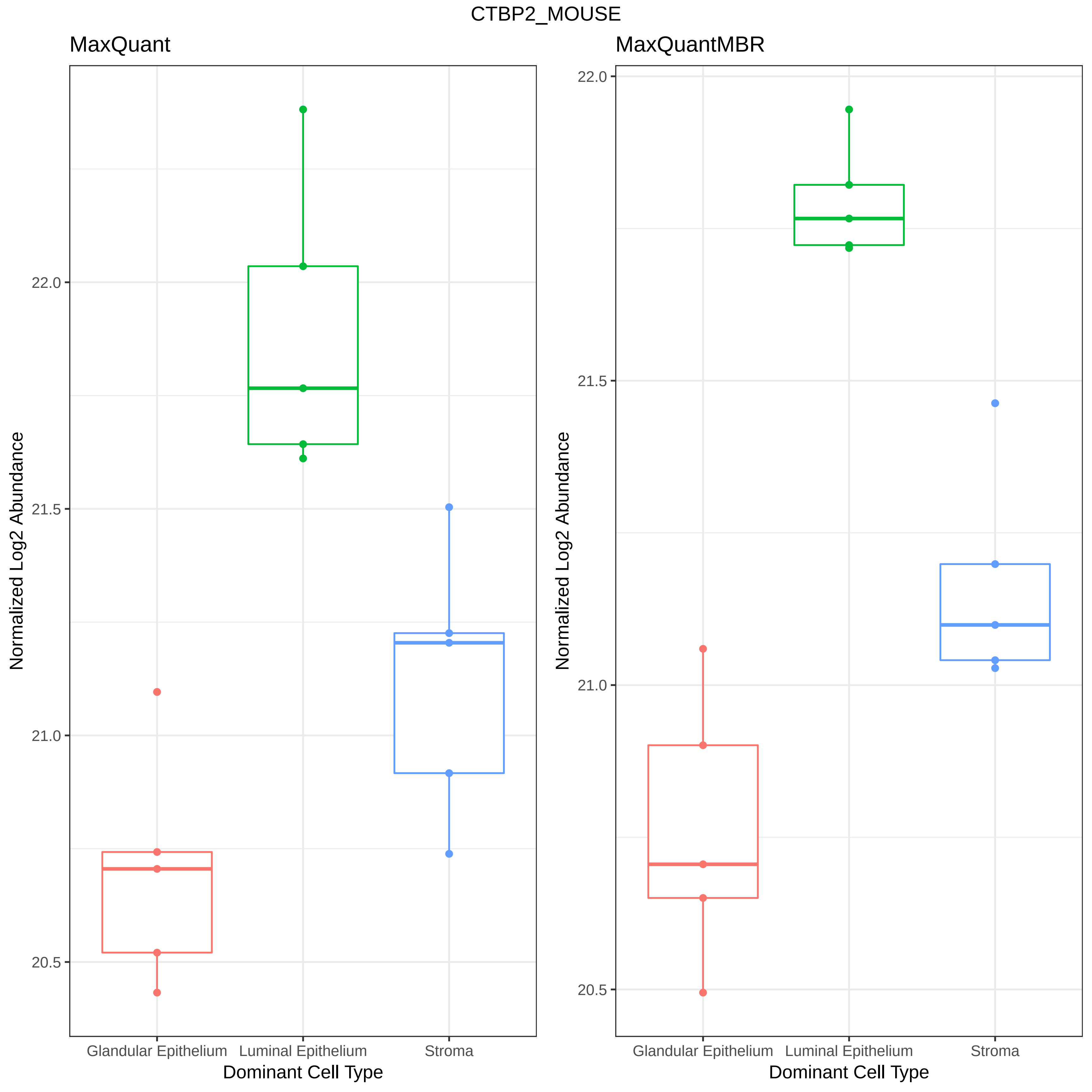

CTBP2_MOUSE
MaxQuant
MaxQuantMBR
Normalized Log2 Abundance
22.0
21.5
21.0
20.5
Glandular Epithelium
Luminal Epithelium
Stroma
Dominant Cell Type

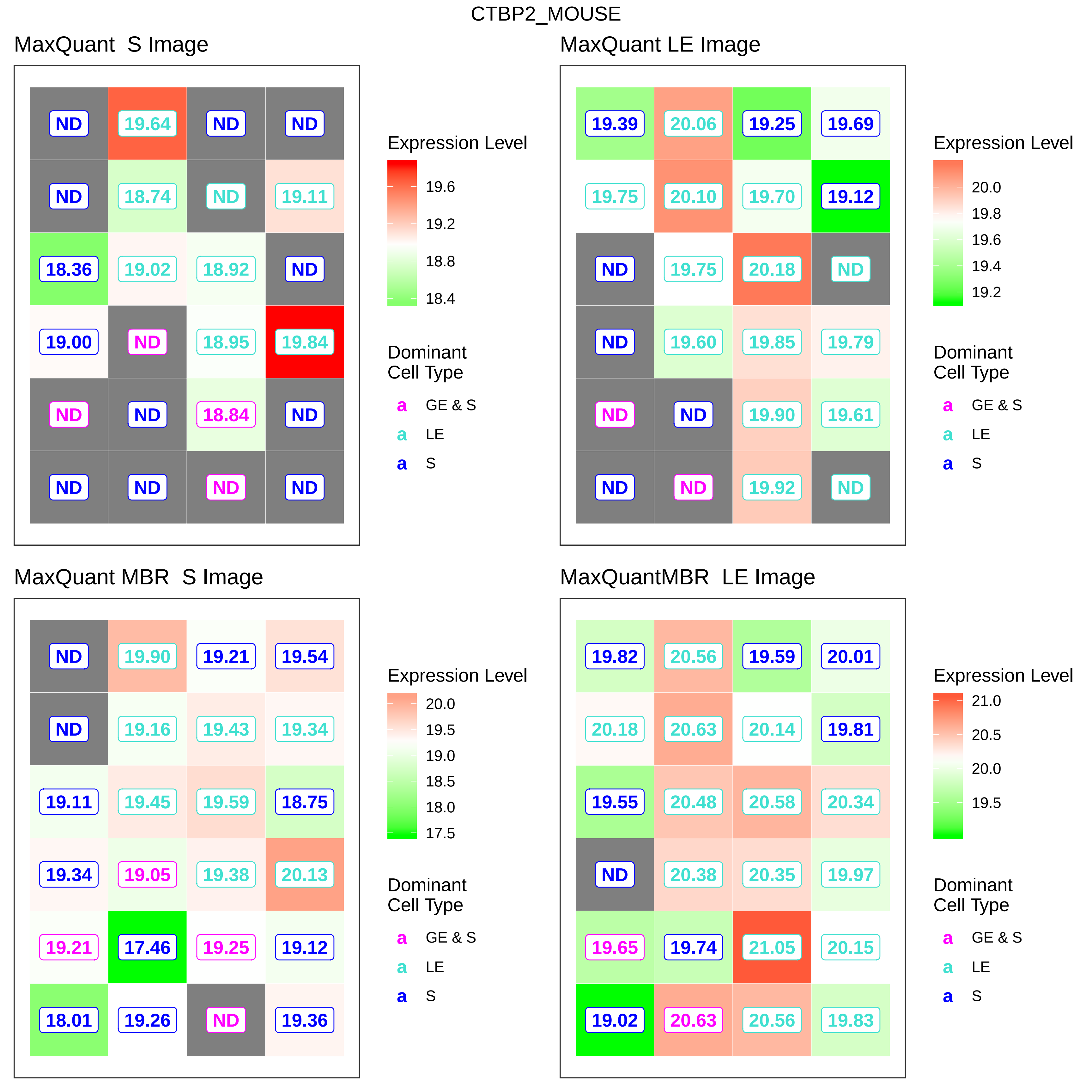
CTBP2_MOUSE
MaxQuant S Image
ND 19.64 ND ND
ND 18.74 ND 19.11
18.36 19.02 18.92 ND
19.00 ND 18.95 19.84
ND ND 18.84 ND
ND ND ND ND
Expression Level
19.6
19.2
18.8
18.4
Dominant Cell Type
a GE & S
a LE
a S
MaxQuant LE Image
19.39 20.06 19.25 19.69
19.75 20.10 19.70 19.12
ND 19.75 20.18 ND
ND 19.60 19.85 19.79
ND ND 19.90 19.61
ND ND 19.92 ND
Expression Level
20.0
19.8
19.6
19.4
19.2
Dominant Cell Type
a GE & S
a LE
a S
MaxQuant MBR S Image
ND 19.90 19.21 19.54
ND 19.16 19.43 19.34
19.11 19.45 19.59 18.75
19.34 19.05 19.38 20.13
19.21 17.46 19.25 19.12
18.01 19.26 ND 19.36
Expression Level
20.0
19.5
19.0
18.5
18.0
17.5
Dominant Cell Type
a GE & S
a LE
a S
MaxQuantMBR LE Image
19.82 20.56 19.59 20.01
20.18 20.63 20.14 19.81
19.55 20.48 20.58 20.34
ND 20.38 20.35 19.97
19.65 19.74 21.05 20.15
19.02 20.63 20.56 19.83
Expression Level
21.0
20.5
20.0
19.5
Dominant Cell Type
a GE & S
a LE
a S

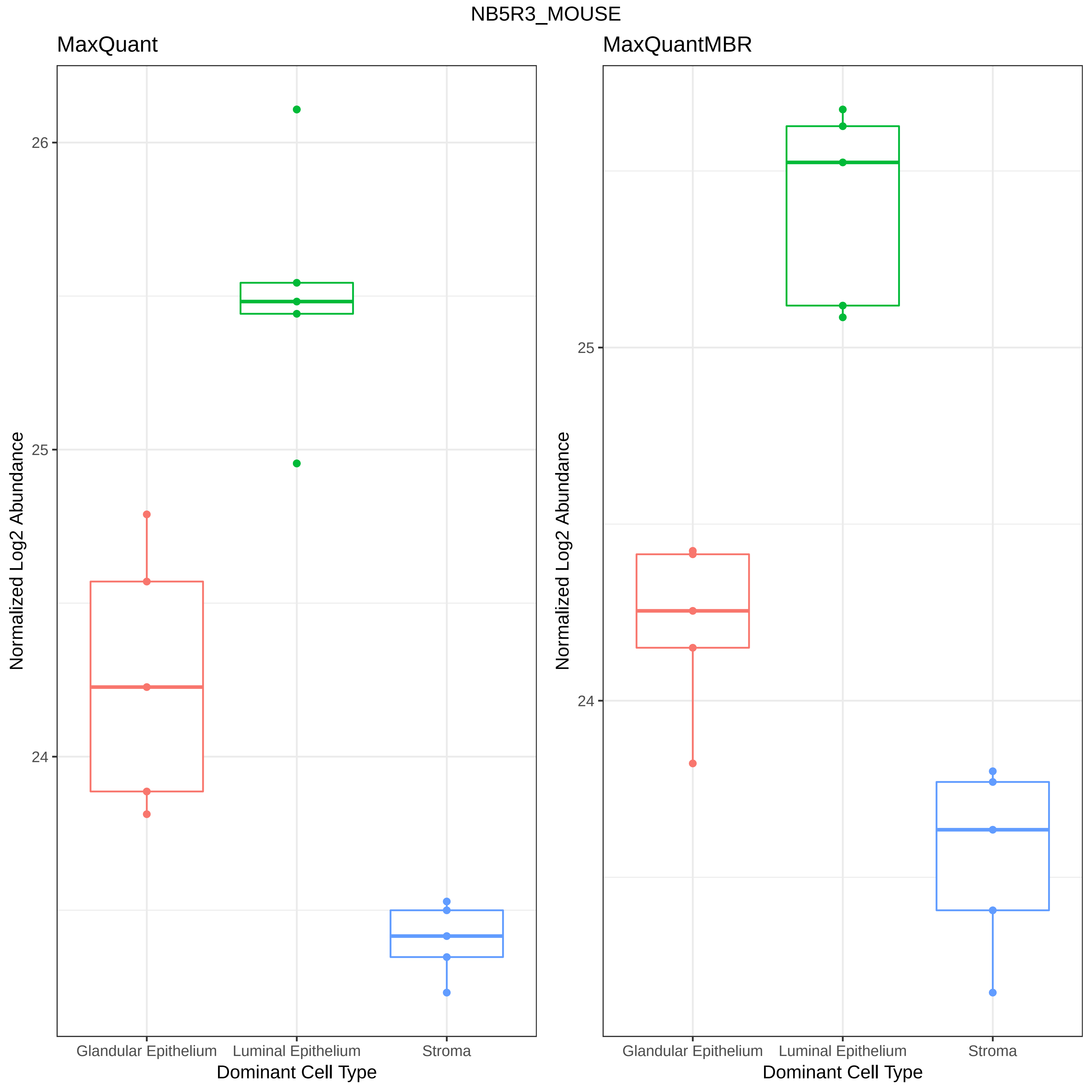

NB5R3_MOUSE
MaxQuant
MaxQuantMBR
Normalized Log2 Abundance
26
25
24
Dominant Cell Type
Glandular Epithelium
Luminal Epithelium
Stroma

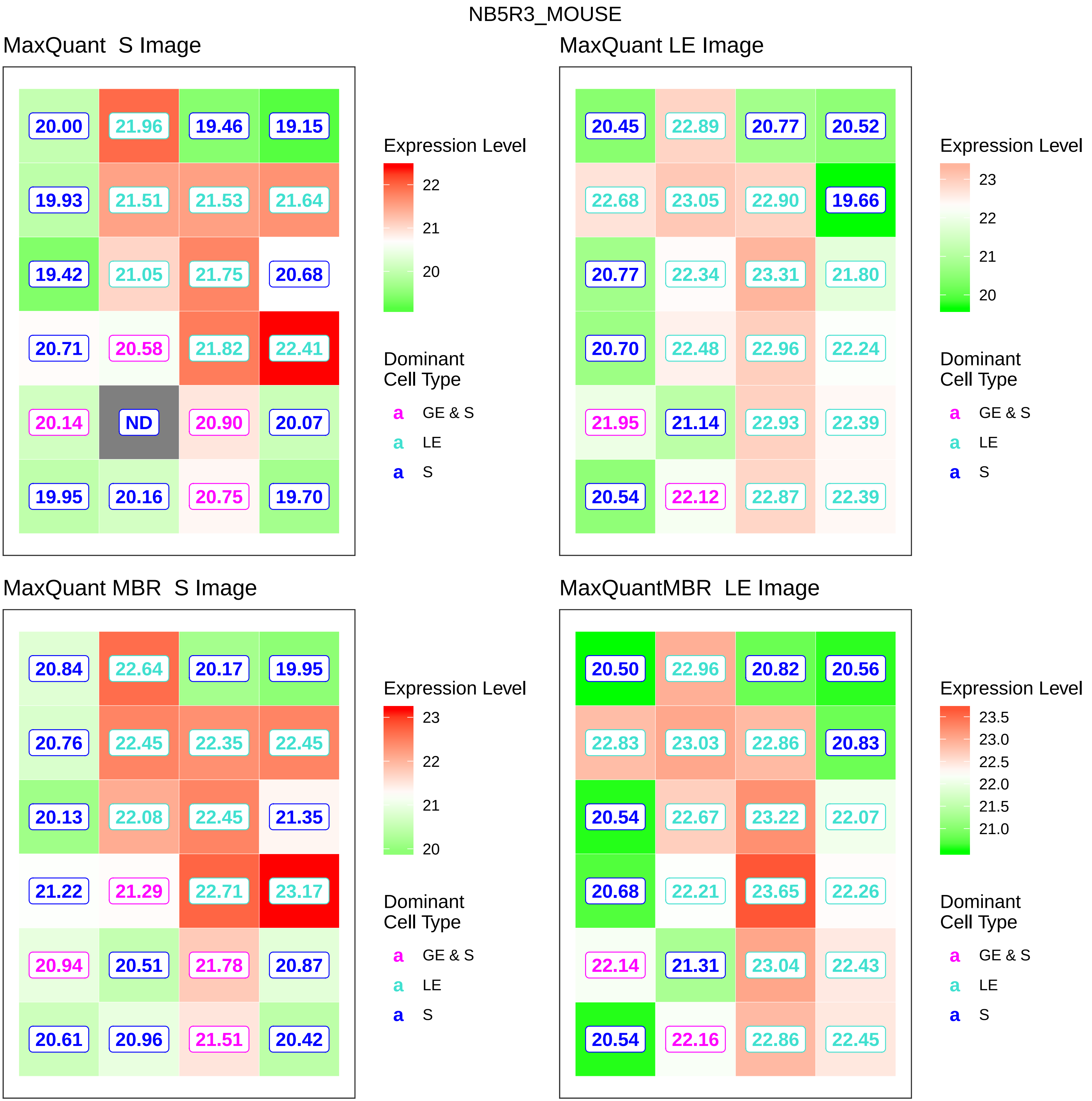
NB5R3_MOUSE
MaxQuant S Image
20.00 21.96 19.46 19.15
19.93 21.51 21.53 21.64
19.42 21.05 21.75 20.68
20.71 20.58 21.82 22.41
20.14 ND 20.90 20.07
19.95 20.16 20.75 19.70
Expression Level
22
21
20
Dominant Cell Type
a GE & S
a LE
a S
MaxQuant LE Image
20.45 22.89 20.77 20.52
22.68 23.05 22.90 19.66
20.77 22.34 23.31 21.80
20.70 22.48 22.96 22.24
21.95 21.14 22.93 22.39
20.54 22.12 22.87 22.39
Expression Level
23
22
21
20
Dominant Cell Type
a GE & S
a LE
a S
MaxQuant MBR S Image
20.84 22.64 20.17 19.95
20.76 22.45 22.35 22.45
20.13 22.08 22.45 21.35
21.22 21.29 22.71 23.17
20.94 20.51 21.78 20.87
20.61 20.96 21.51 20.42
Expression Level
23
22
21
20
Dominant Cell Type
a GE & S
a LE
a S
MaxQuantMBR LE Image
20.50 22.96 20.82 20.56
22.83 23.03 22.86 20.83
20.54 22.67 23.22 22.07
20.68 22.21 23.65 22.26
22.14 21.31 23.04 22.43
20.54 22.16 22.86 22.45
Expression Level
23.5
23.0
22.5
22.0
21.5
21.0
Dominant Cell Type
a GE & S
a LE
a S

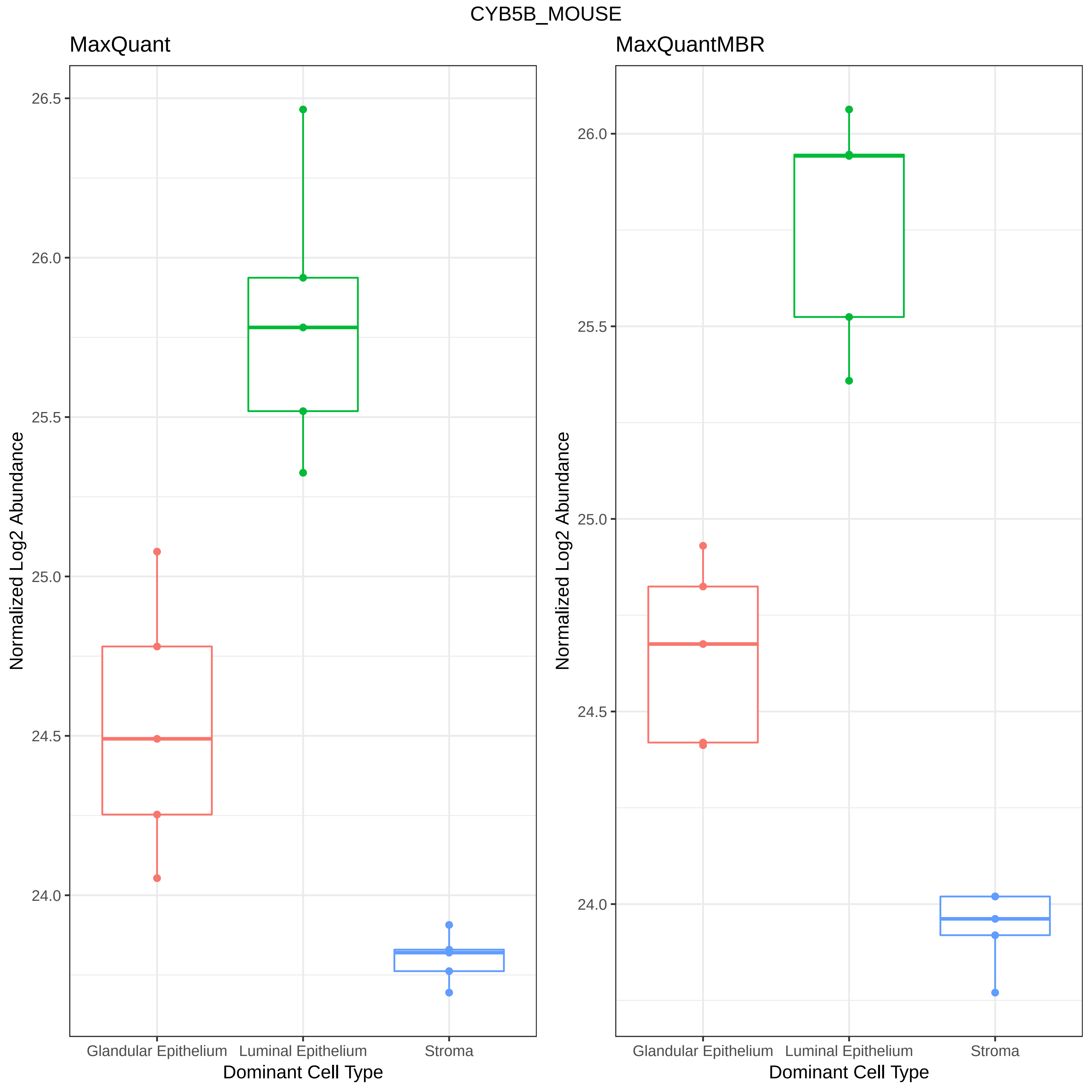

CYB5B_MOUSE
MaxQuant
MaxQuantMBR
Normalized Log2 Abundance
26.5
26.0
25.5
25.0
24.5
24.0
Glandular Epithelium
Luminal Epithelium
Stroma
Dominant Cell Type

CYB5B_MOUSE

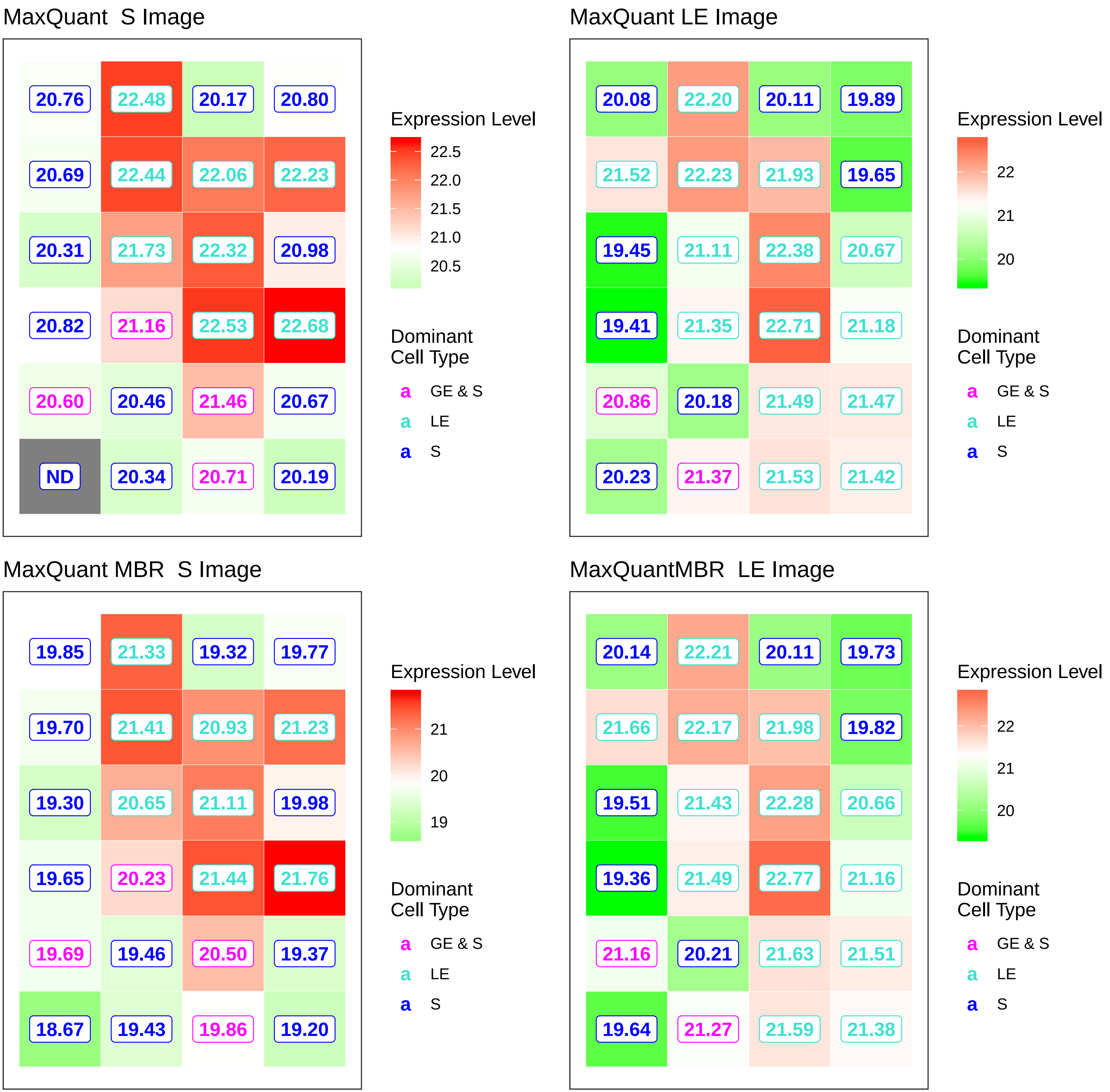
MaxQuant S Image
20.76 22.48 20.17 20.80
20.69 22.44 22.06 22.23
20.31 21.73 22.32 20.98
20.82 21.16 22.53 22.68
20.60 20.46 21.46 20.67
ND 20.34 20.71 20.19
Expression Level
22.5
22.0
21.5
21.0
20.5
Dominant Cell Type
a GE & S
a LE
a S
MaxQuant LE Image
20.08 22.20 20.11 19.89
21.52 22.23 21.93 19.65
19.45 21.11 22.38 20.67
19.41 21.35 22.71 21.18
20.86 20.18 21.49 21.47
20.23 21.37 21.53 21.42
Expression Level
22
21
20
Dominant Cell Type
a GE & S
a LE
a S
MaxQuant MBR S Image
19.85 21.33 19.32 19.77
19.70 21.41 20.93 21.23
19.30 20.65 21.11 19.98
19.65 20.23 21.44 21.76
19.69 19.46 20.50 19.37
18.67 19.43 19.86 19.20
Expression Level
21
20
19
Dominant Cell Type
a GE & S
a LE
a S
MaxQuantMBR LE Image
20.14 22.21 20.11 19.73
21.66 22.17 21.98 19.82
19.51 21.43 22.28 20.66
19.36 21.49 22.77 21.16
21.16 20.21 21.63 21.51
19.64 21.27 21.59 21.38
Expression Level
22
21
20
Dominant Cell Type
a GE & S
a LE
a S

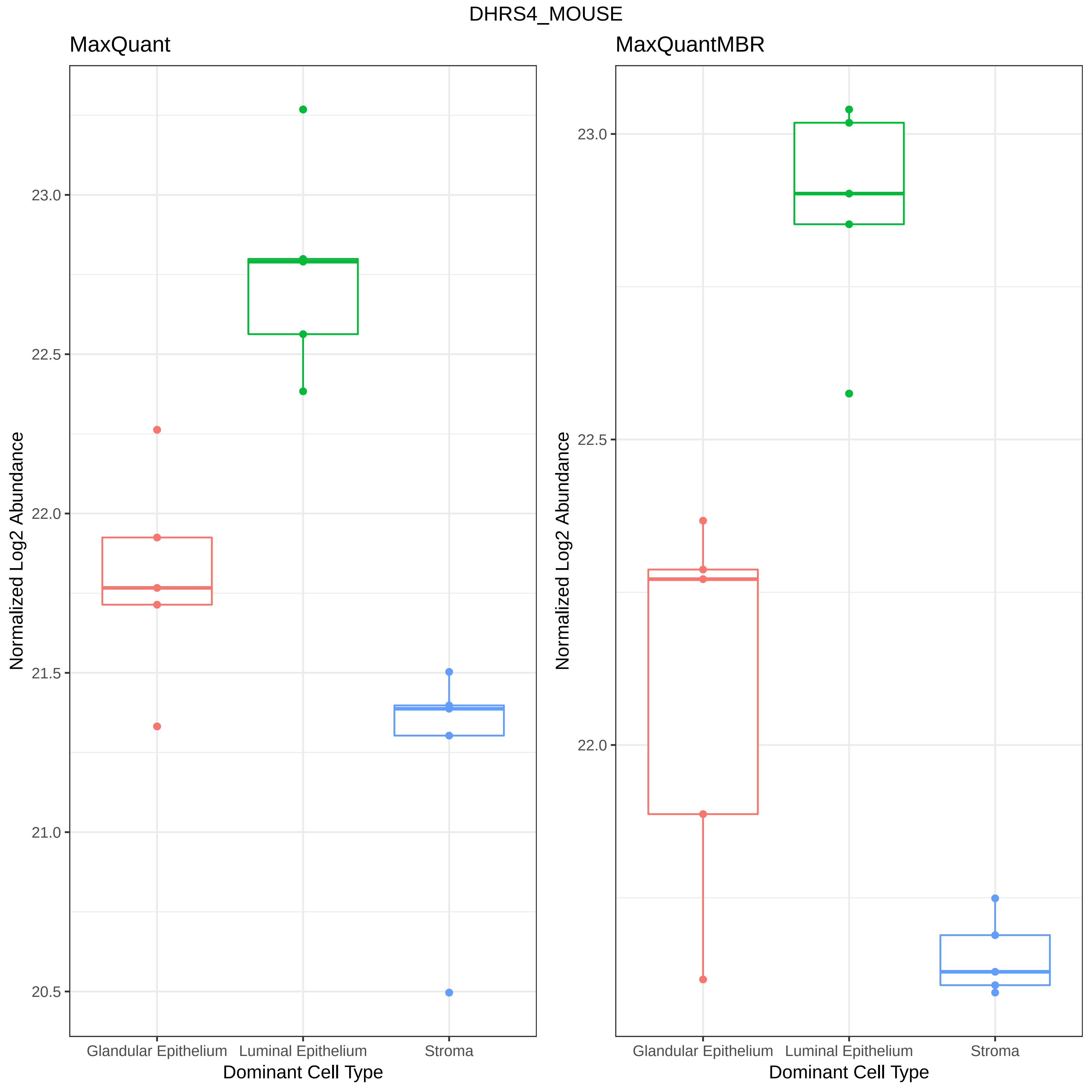
DHRS4_MOUSE
MaxQuant
MaxQuantMBR
Normalized Log2 Abundance
23.0
22.5
22.0
21.5
21.0
20.5
Glandular Epithelium
Luminal Epithelium
Stroma
Dominant Cell Type

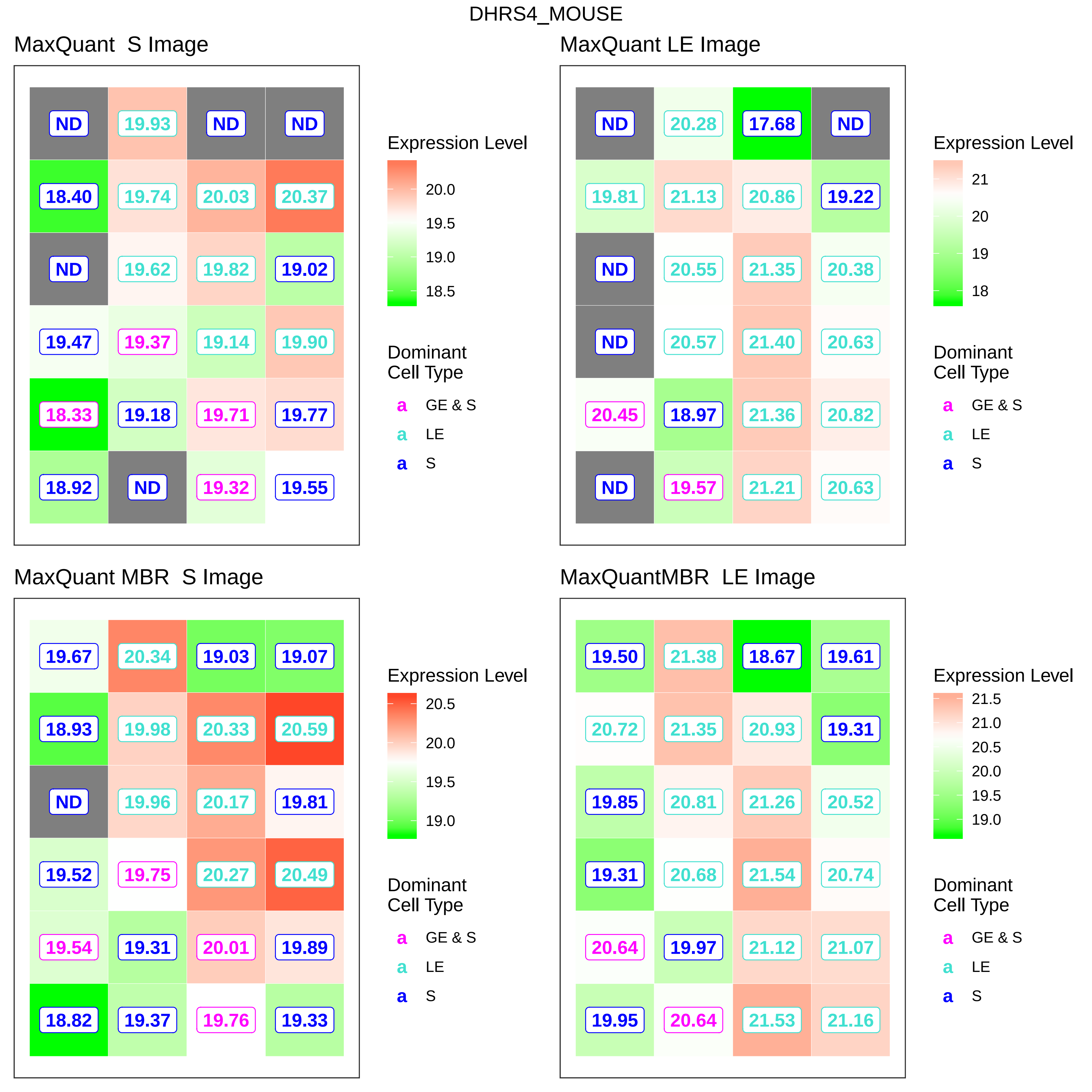
DHRS4_MOUSE
MaxQuant S Image
ND 19.93 ND ND
18.40 19.74 20.03 20.37
ND 19.62 19.82 19.02
19.47 19.37 19.14 19.90
18.33 19.18 19.71 19.77
18.92 ND 19.32 19.55
Expression Level
20.0
19.5
19.0
18.5
Dominant Cell Type
a GE & S
a LE
a S
MaxQuant LE Image
ND 20.28 17.68 ND
19.81 21.13 20.86 19.22
ND 20.55 21.35 20.38
ND 20.57 21.40 20.63
20.45 18.97 21.36 20.82
ND 19.57 21.21 20.63
Expression Level
21
20
19
18
Dominant Cell Type
a GE & S
a LE
a S
MaxQuant MBR S Image
19.67 20.34 19.03 19.07
18.93 19.98 20.33 20.59
ND 19.96 20.17 19.81
19.52 19.75 20.27 20.49
19.54 19.31 20.01 19.89
18.82 19.37 19.76 19.33
Expression Level
20.5
20.0
19.5
19.0
Dominant Cell Type
a GE & S
a LE
a S
MaxQuantMBR LE Image
19.50 21.38 18.67 19.61
20.72 21.35 20.93 19.31
19.85 20.81 21.26 20.52
19.31 20.68 21.54 20.74
20.64 19.97 21.12 21.07
19.95 20.64 21.53 21.16
Expression Level
21.5
21.0
20.5
20.0
19.5
19.0
Dominant Cell Type
a GE & S
a LE
a S

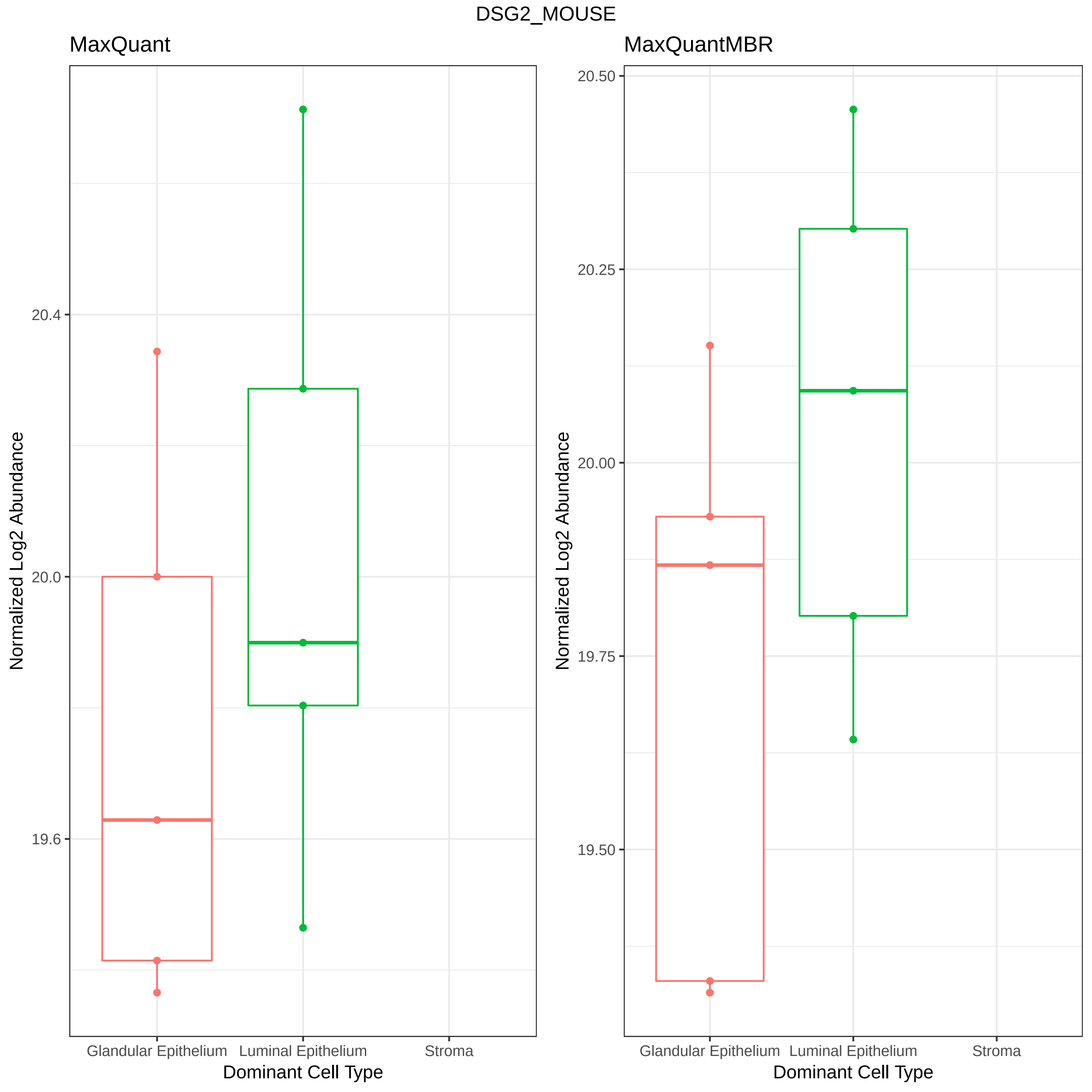
DSG2_MOUSE
MaxQuant
MaxQuantMBR
Normalized Log2 Abundance
20.4
20.0
19.6
20.50
20.25
20.00
19.75
19.50
Glandular Epithelium
Luminal Epithelium
Stroma
Dominant Cell Type

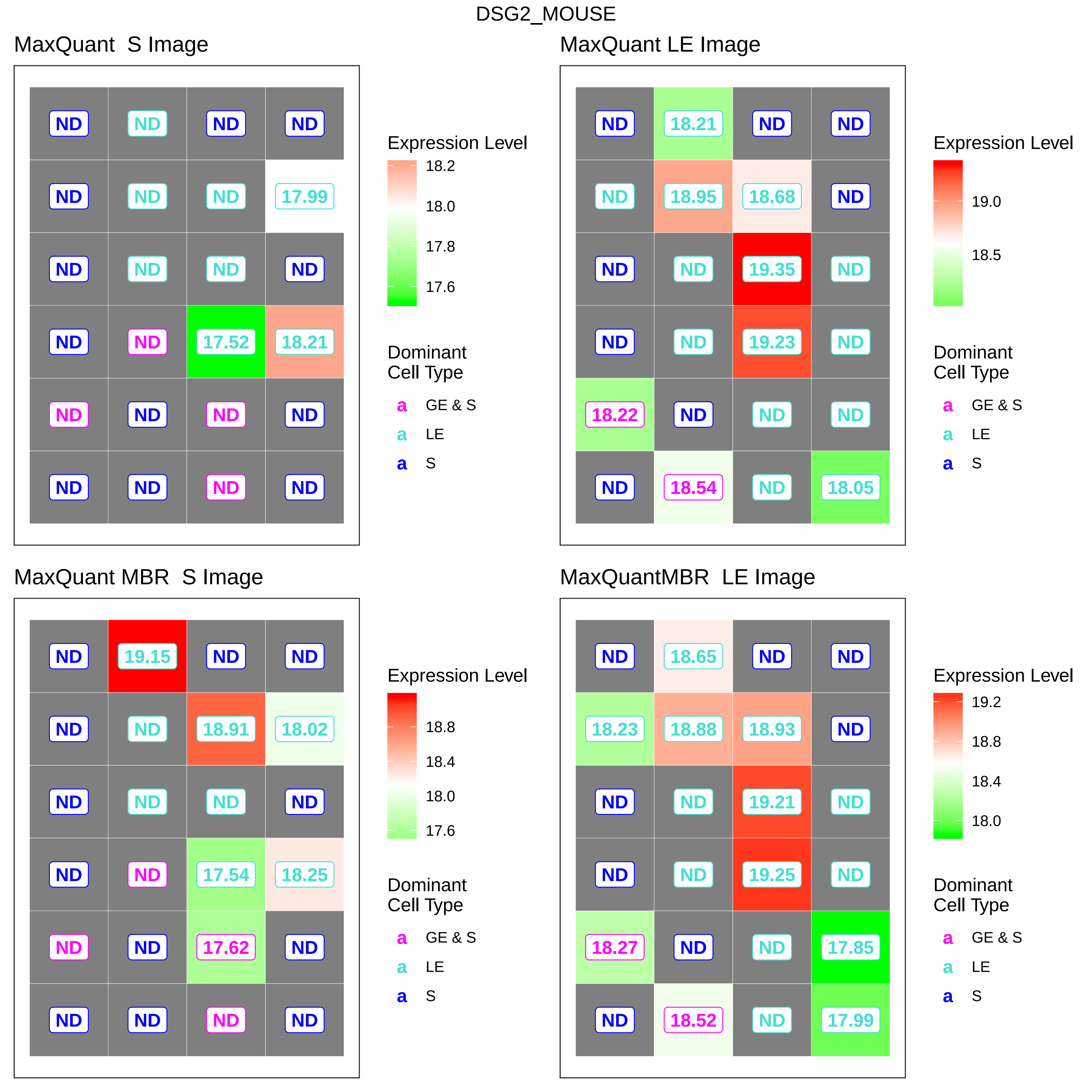
DSG2_MOUSE
MaxQuant S Image
ND ND ND ND
ND ND ND 17.99
ND ND ND ND
ND ND 17.52 18.21
ND ND ND ND
ND ND ND ND
Expression Level
18.2
18.0
17.8
17.6
Dominant Cell Type
a GE & S
a LE
a S
MaxQuant LE Image
ND 18.21 ND ND
ND 18.95 18.68 ND
ND ND 19.35 ND
ND ND 19.23 ND
18.22 ND ND ND
ND 18.54 ND 18.05
Expression Level
19.0
18.5
Dominant Cell Type
a GE & S
a LE
a S
MaxQuant MBR S Image
ND 19.15 ND ND
ND ND 18.91 18.02
ND ND ND ND
ND ND 17.54 18.25
ND ND 17.62 ND
ND ND ND ND
Expression Level
18.8
18.4
18.0
17.6
Dominant Cell Type
a GE & S
a LE
a S
MaxQuantMBR LE Image
ND 18.65 ND ND
18.23 18.88 18.93 ND
ND ND 19.21 ND
ND ND 19.25 ND
18.27 ND ND 17.85
ND 18.52 ND 17.99
Expression Level
19.2
18.8
18.4
18.0
Dominant Cell Type
a GE & S
a LE
a S

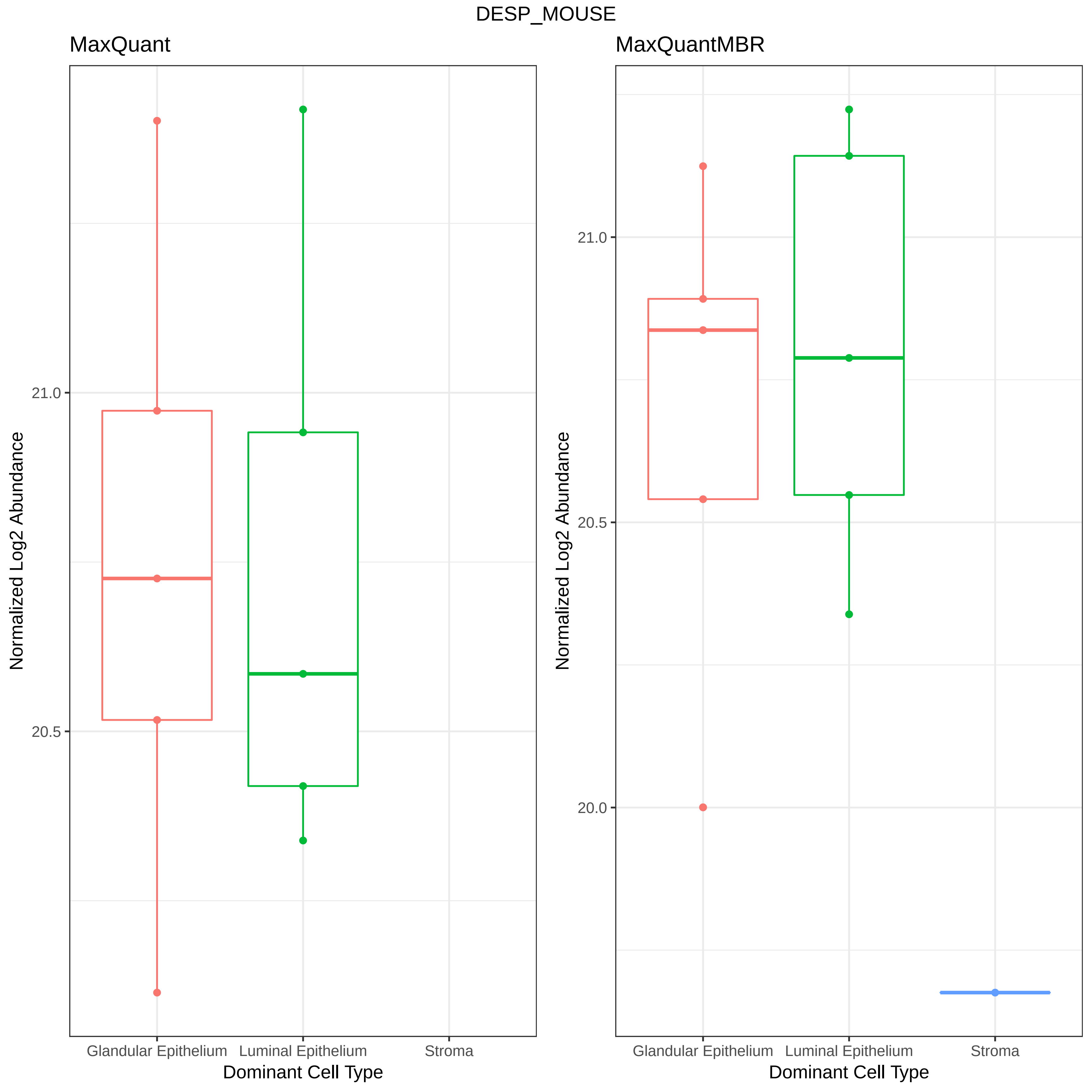
DESP_MOUSE
MaxQuant
MaxQuantMBR
Normalized Log2 Abundance
21.0
20.5
20.0
Glandular Epithelium
Luminal Epithelium
Stroma
Dominant Cell Type

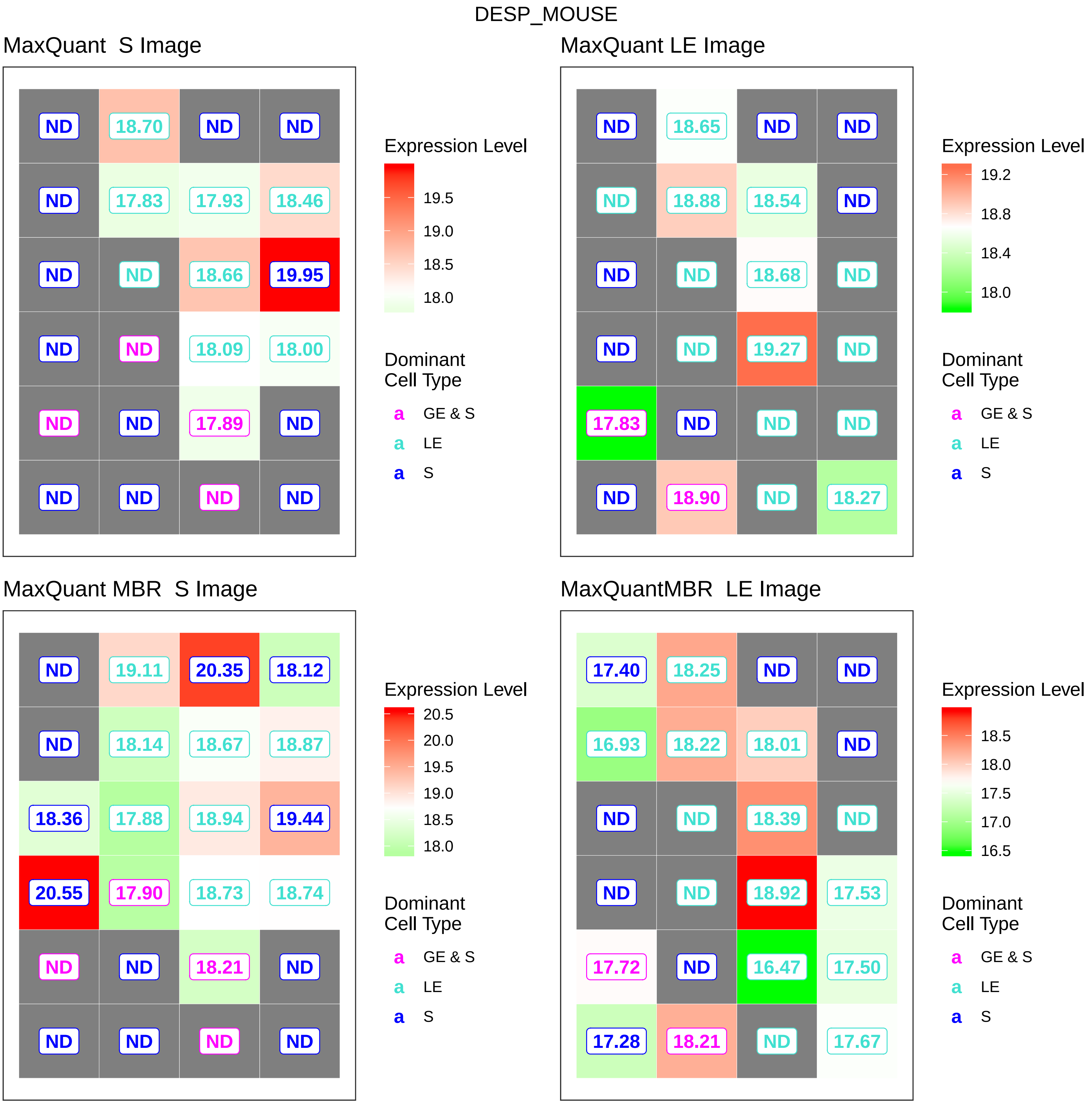
DESP_MOUSE
MaxQuant S Image
ND 18.70 ND ND
ND 17.83 17.93 18.46
ND ND 18.66 19.95
ND ND 18.09 18.00
ND ND 17.89 ND
ND ND ND ND
Expression Level
19.5
19.0
18.5
18.0
Dominant Cell Type
a GE & S
a LE
a S
MaxQuant LE Image
ND 18.65 ND ND
ND 18.88 18.54 ND
ND ND 18.68 ND
ND ND 19.27 ND
17.83 ND ND ND
ND 18.90 ND 18.27
Expression Level
19.2
18.8
18.4
18.0
Dominant Cell Type
a GE & S
a LE
a S
MaxQuant MBR S Image
ND 19.11 20.35 18.12
ND 18.14 18.67 18.87
18.36 17.88 18.94 19.44
20.55 17.90 18.73 18.74
ND ND 18.21 ND
ND ND ND ND
Expression Level
20.5
20.0
19.5
19.0
18.5
18.0
Dominant Cell Type
a GE & S
a LE
a S
MaxQuantMBR LE Image
17.40 18.25 ND ND
16.93 18.22 18.01 ND
ND ND 18.39 ND
ND ND 18.92 17.53
17.72 ND 16.47 17.50
17.28 18.21 ND 17.67
Expression Level
18.5
18.0
17.5
17.0
16.5
Dominant Cell Type
a GE & S
a LE
a S

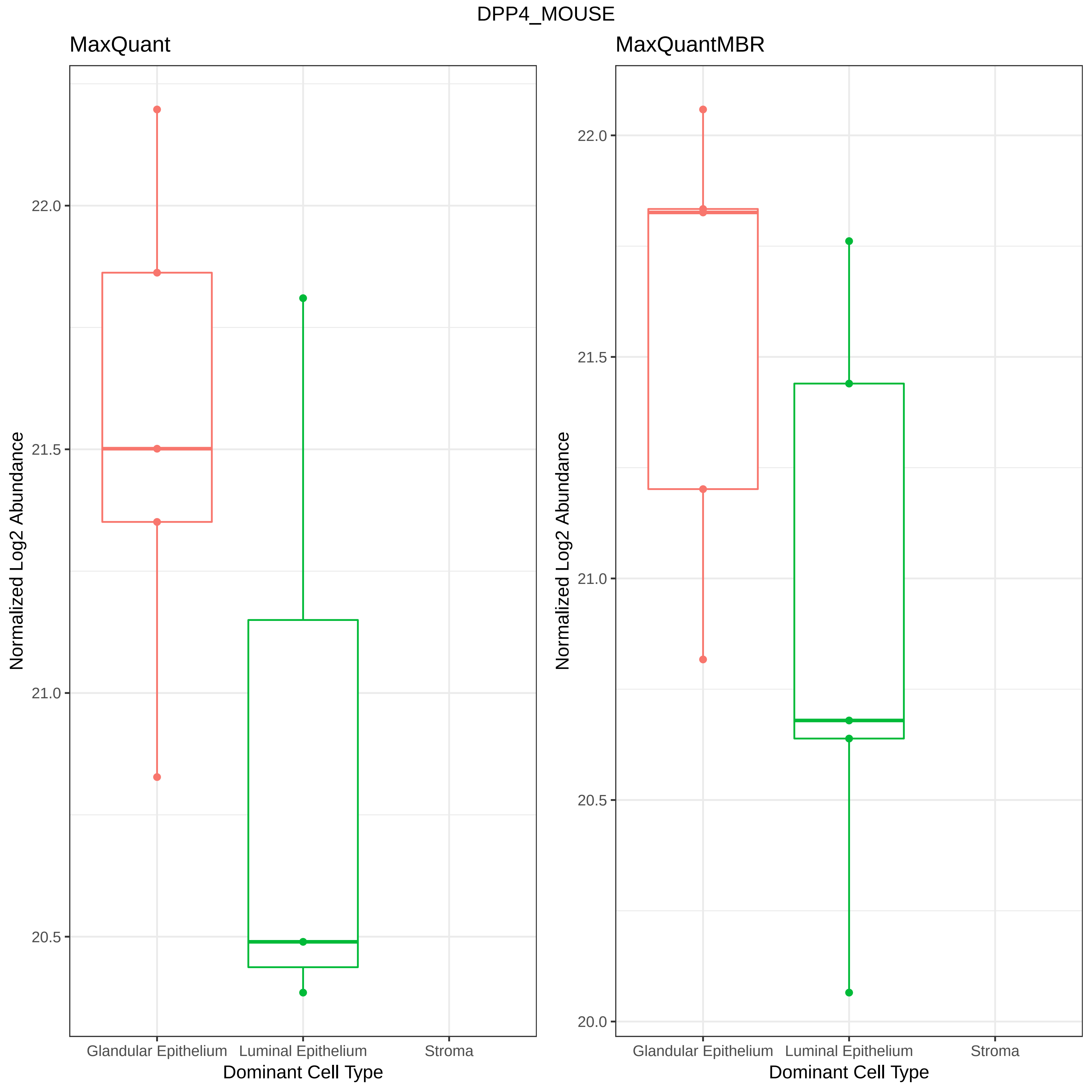
DPP4_MOUSE
MaxQuant
MaxQuantMBR
Normalized Log2 Abundance
22.0
21.5
21.0
20.5
20.0
Glandular Epithelium
Luminal Epithelium
Stroma
Dominant Cell Type

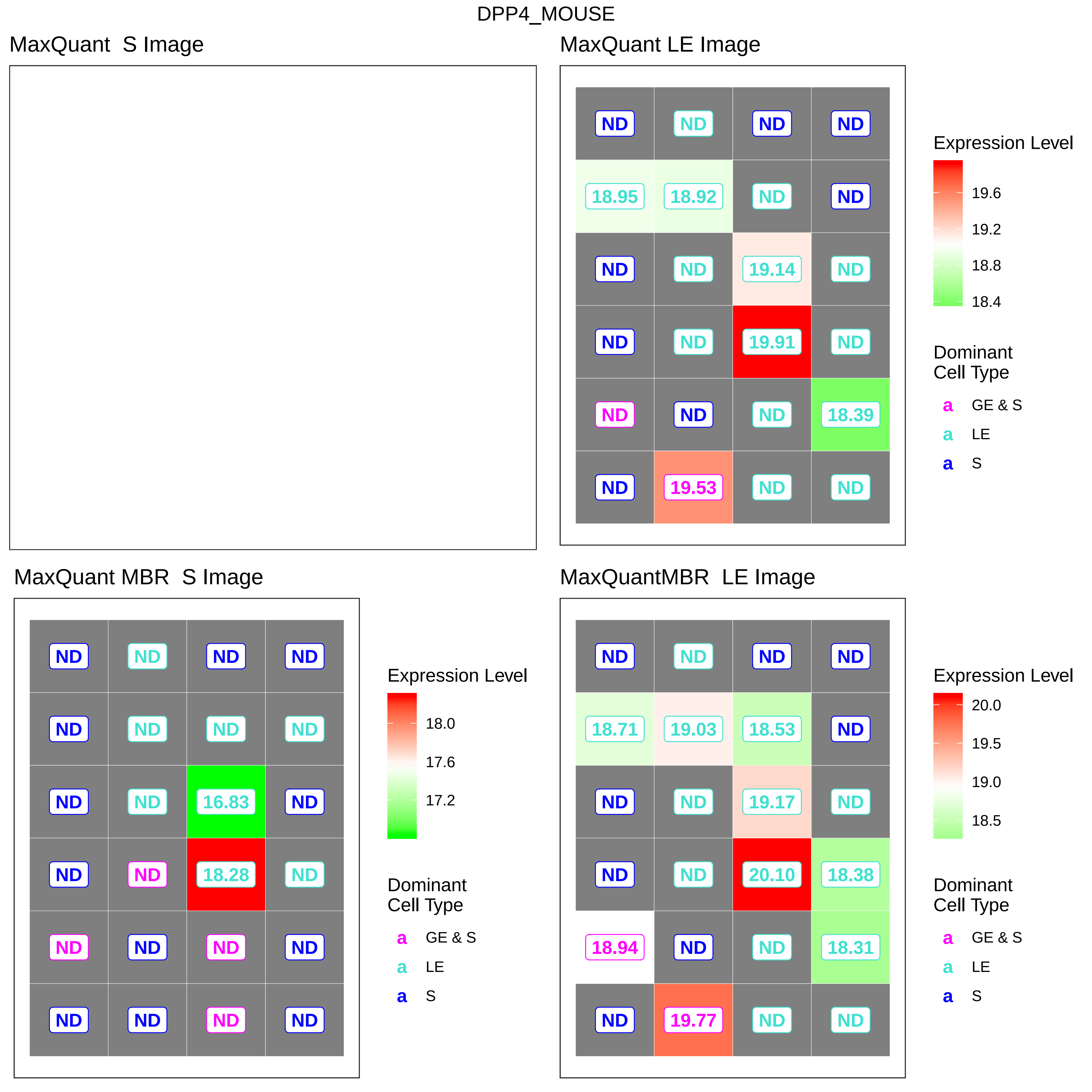
DPP4_MOUSE
MaxQuant S Image
MaxQuant LE Image
ND ND ND ND
18.95 18.92 ND ND
ND ND 19.14 ND
ND ND 19.91 ND
ND ND ND 18.39
ND 19.53 ND ND
Expression Level
19.6
19.2
18.8
18.4
Dominant Cell Type
a GE & S
a LE
a S
MaxQuant MBR S Image
ND ND ND ND
ND ND ND ND
ND ND 16.83 ND
ND ND 18.28 ND
ND ND ND ND
ND ND ND ND
Expression Level
18.0
17.6
17.2
Dominant Cell Type
a GE & S
a LE
a S
MaxQuantMBR LE Image
ND ND ND ND
18.71 19.03 18.53 ND
ND ND 19.17 ND
ND ND 20.10 18.38
18.94 ND ND 18.31
ND 19.77 ND ND
Expression Level
20.0
19.5
19.0
18.5
Dominant Cell Type
a GE & S
a LE
a S

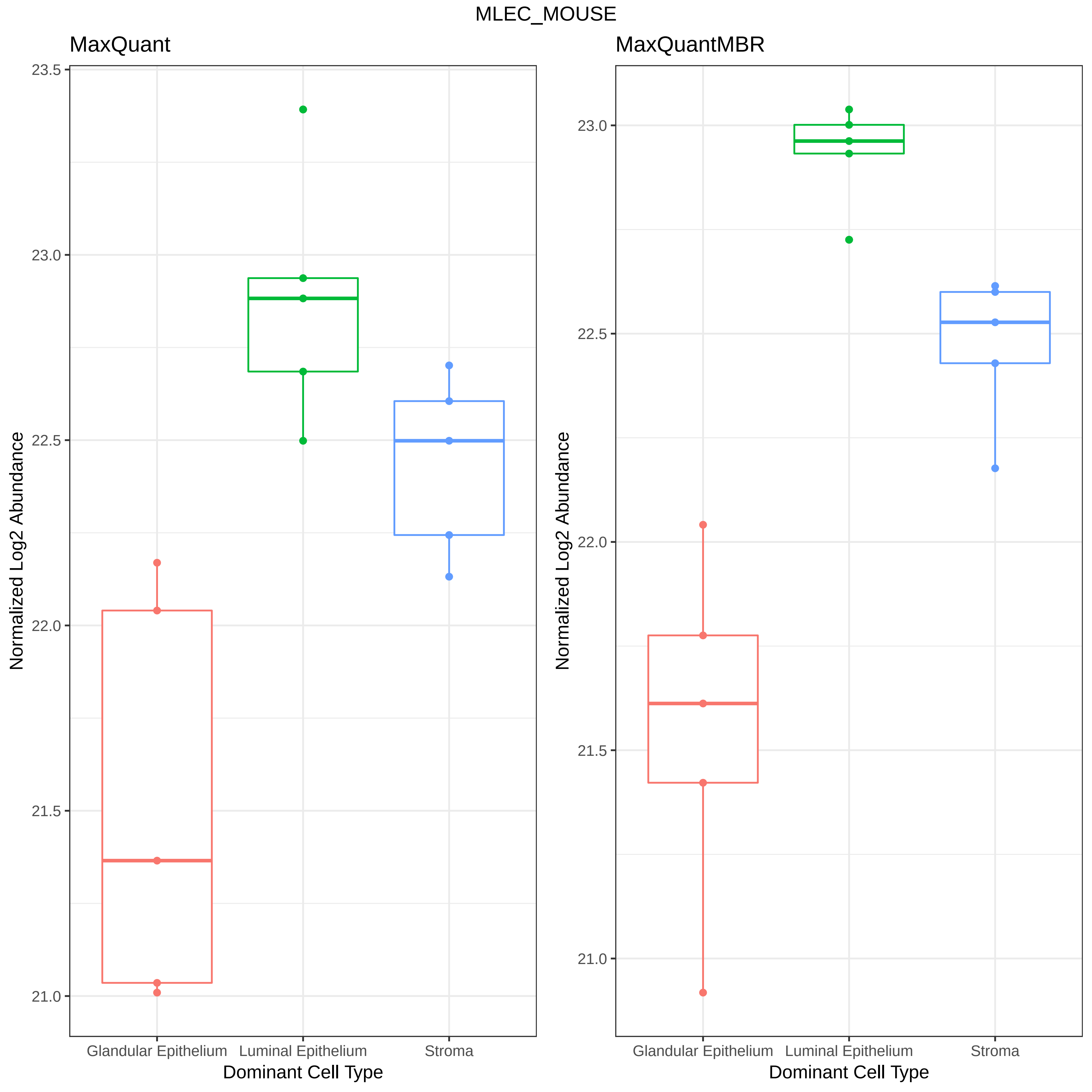
MLEC_MOUSE
MaxQuant
MaxQuantMBR
Normalized Log2 Abundance
Dominant Cell Type
Glandular Epithelium
Luminal Epithelium
Stroma
23.5
23.0
22.5
22.0
21.5
21.0

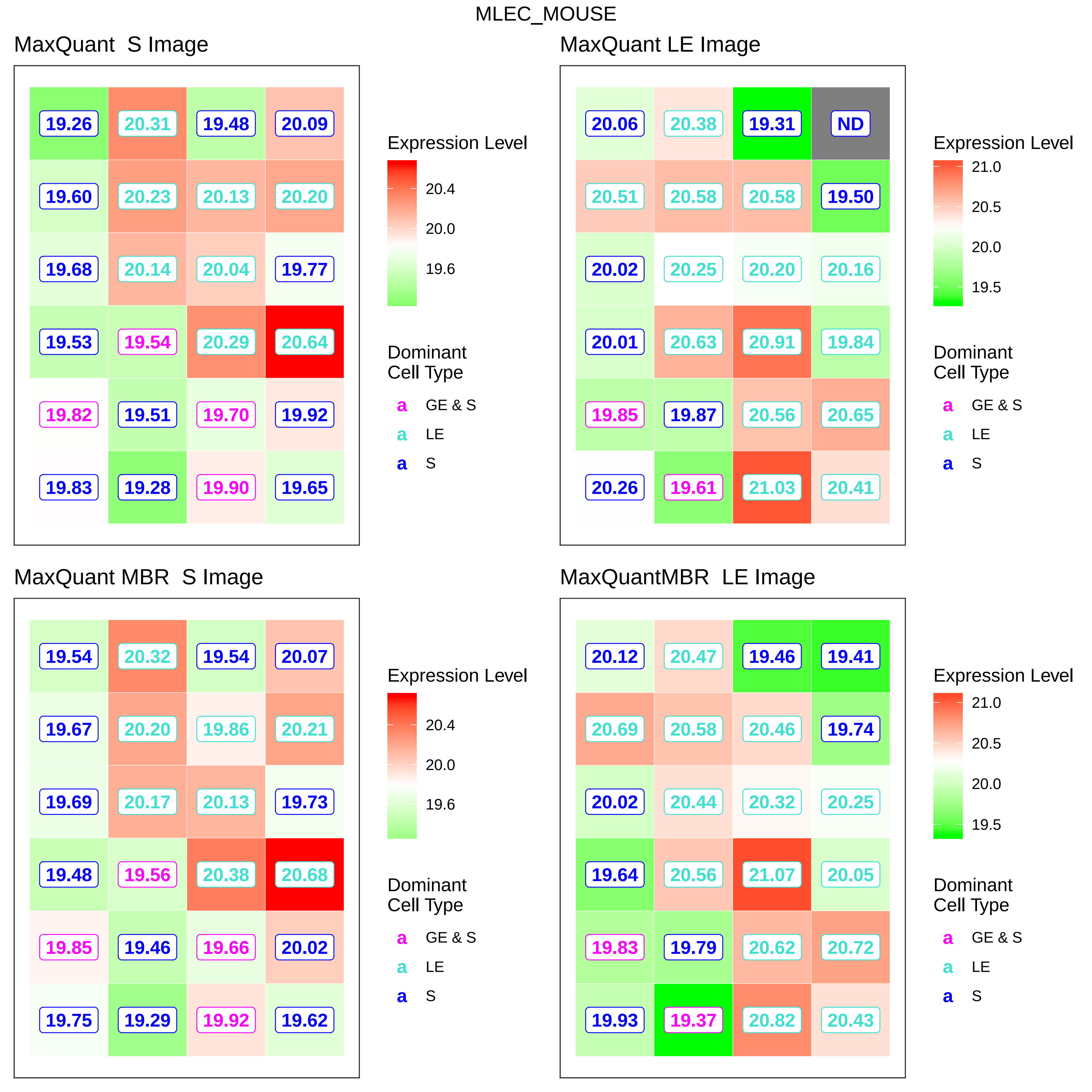
MLEC_MOUSE
MaxQuant S Image
19.26 20.31 19.48 20.09
19.60 20.23 20.13 20.20
19.68 20.14 20.04 19.77
19.53 19.54 20.29 20.64
19.82 19.51 19.70 19.92
19.83 19.28 19.90 19.65
Expression Level
20.4
20.0
19.6
Dominant Cell Type
a GE & S
a LE
a S
MaxQuant LE Image
20.06 20.38 19.31 ND
20.51 20.58 20.58 19.50
20.02 20.25 20.20 20.16
20.01 20.63 20.91 19.84
19.85 19.87 20.56 20.65
20.26 19.61 21.03 20.41
Expression Level
21.0
20.5
20.0
19.5
Dominant Cell Type
a GE & S
a LE
a S
MaxQuant MBR S Image
19.54 20.32 19.54 20.07
19.67 20.20 19.86 20.21
19.69 20.17 20.13 19.73
19.48 19.56 20.38 20.68
19.85 19.46 19.66 20.02
19.75 19.29 19.92 19.62
Expression Level
20.4
20.0
19.6
Dominant Cell Type
a GE & S
a LE
a S
MaxQuantMBR LE Image
20.12 20.47 19.46 19.41
20.69 20.58 20.46 19.74
20.02 20.44 20.32 20.25
19.64 20.56 21.07 20.05
19.83 19.79 20.62 20.72
19.93 19.37 20.82 20.43
Expression Level
21.0
20.5
20.0
19.5
Dominant Cell Type
a GE & S
a LE
a S

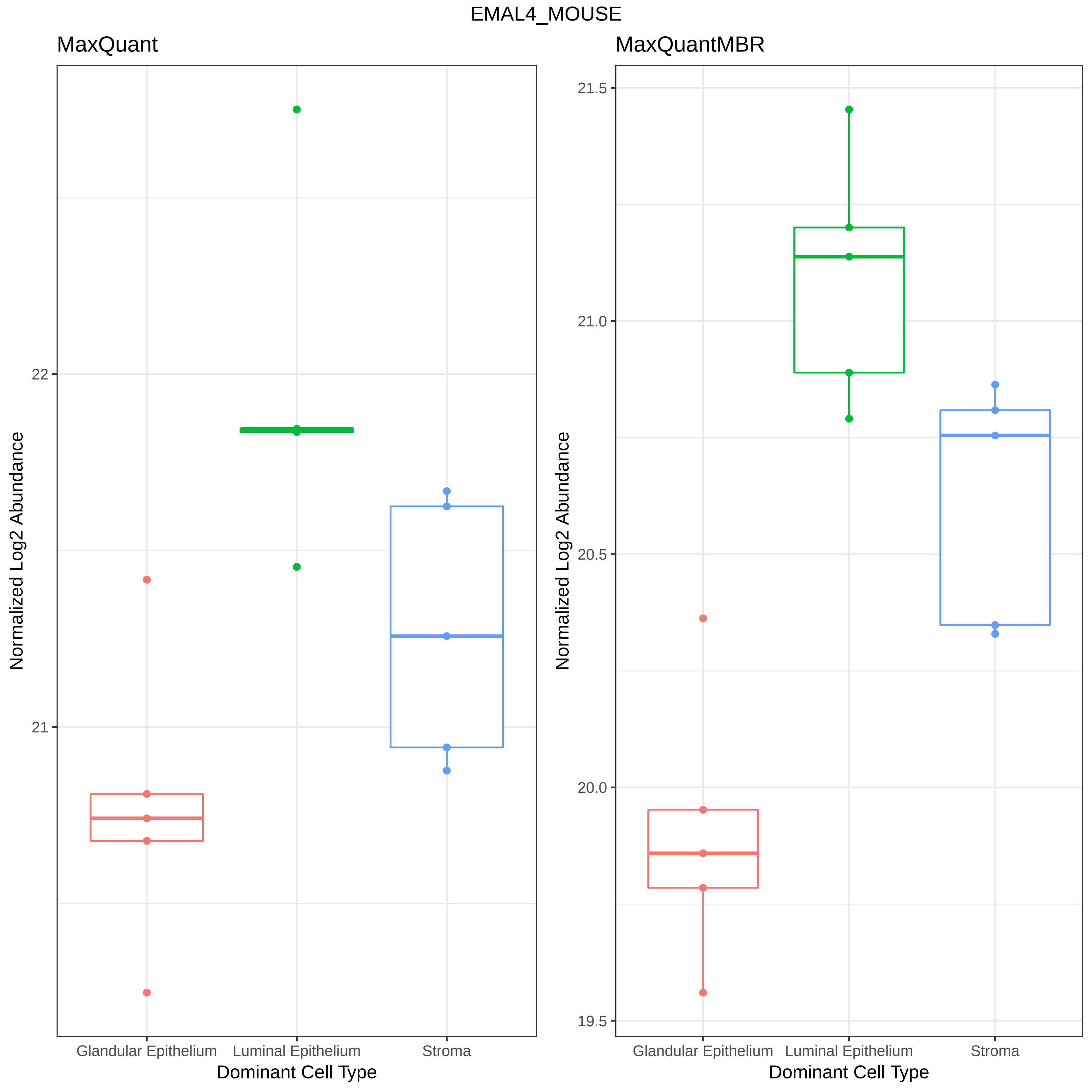
EMAL4_MOUSE
MaxQuant
MaxQuantMBR
Normalized Log2 Abundance
22
21
21.5
21.0
20.5
20.0
19.5
Glandular Epithelium
Luminal Epithelium
Stroma
Dominant Cell Type

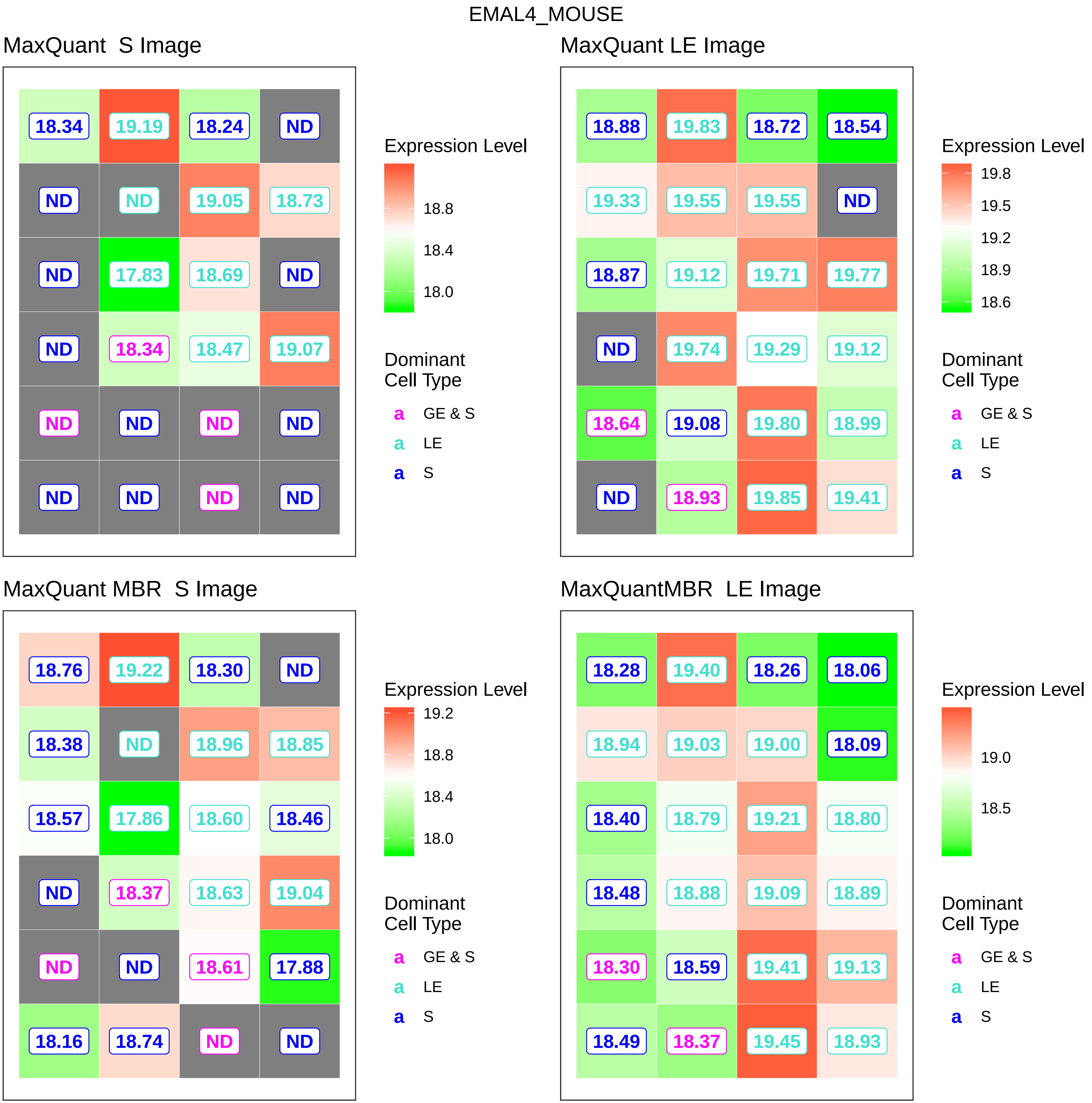
EMAL4_MOUSE
MaxQuant S Image
18.34 19.19 18.24 ND
ND ND 19.05 18.73
ND 17.83 18.69 ND
ND 18.34 18.47 19.07
ND ND ND ND
ND ND ND ND
Expression Level
18.8
18.4
18.0
Dominant Cell Type
a GE & S
a LE
a S
MaxQuant LE Image
18.88 19.83 18.72 18.54
19.33 19.55 19.55 ND
18.87 19.12 19.71 19.77
ND 19.74 19.29 19.12
18.64 19.08 19.80 18.99
ND 18.93 19.85 19.41
Expression Level
19.8
19.5
19.2
18.9
18.6
Dominant Cell Type
a GE & S
a LE
a S
MaxQuant MBR S Image
18.76 19.22 18.30 ND
18.38 ND 18.96 18.85
18.57 17.86 18.60 18.46
ND 18.37 18.63 19.04
ND ND 18.61 17.88
18.16 18.74 ND ND
Expression Level
19.2
18.8
18.4
18.0
Dominant Cell Type
a GE & S
a LE
a S
MaxQuantMBR LE Image
18.28 19.40 18.26 18.06
18.94 19.03 19.00 18.09
18.40 18.79 19.21 18.80
18.48 18.88 19.09 18.89
18.30 18.59 19.41 19.13
18.49 18.37 19.45 18.93
Expression Level
19.0
18.5
Dominant Cell Type
a GE & S
a LE
a S

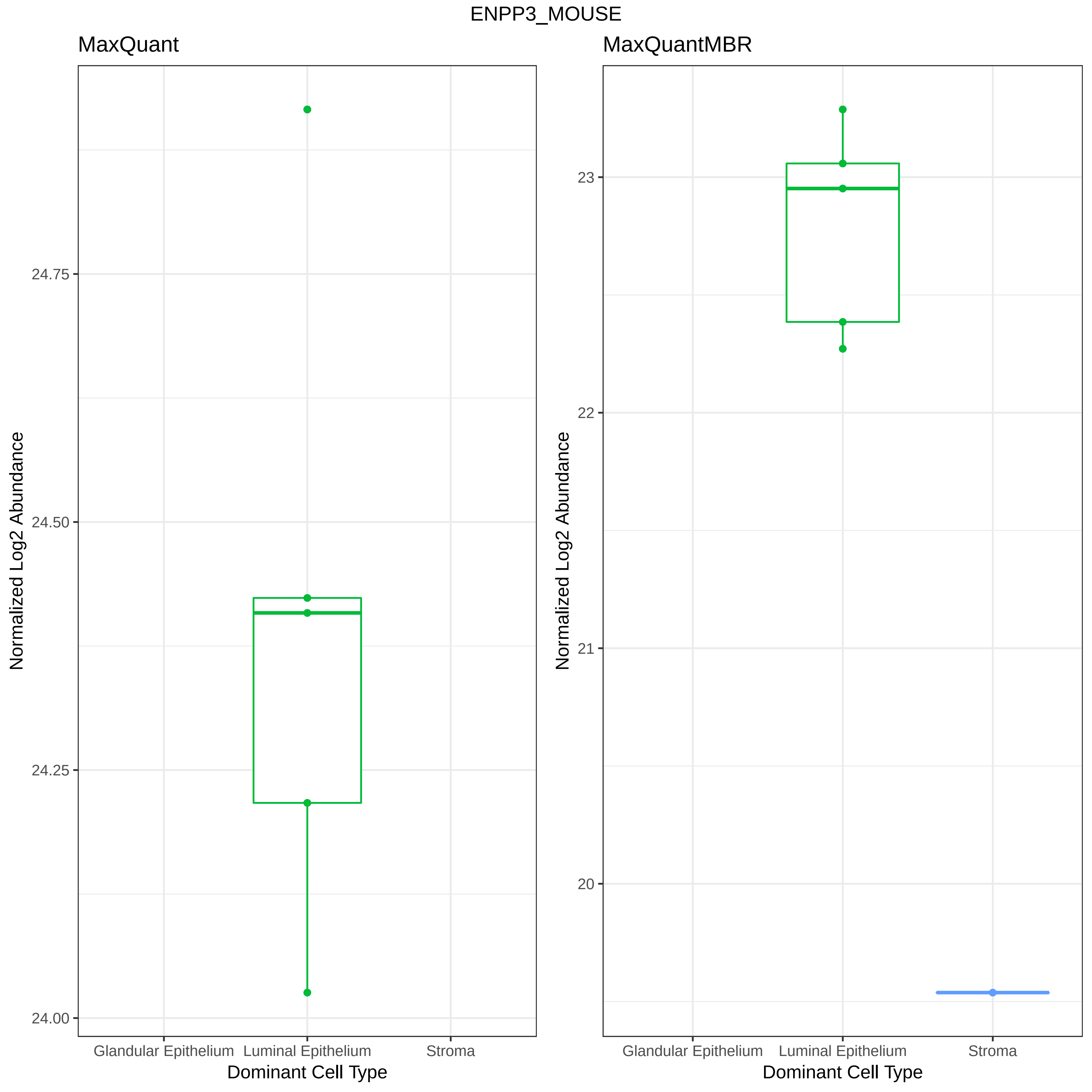
ENPP3_MOUSE
MaxQuant
MaxQuantMBR
Normalized Log2 Abundance
24.75
24.50
24.25
24.00
23
22
21
20
Glandular Epithelium
Luminal Epithelium
Stroma
Dominant Cell Type

ENPP3_MOUSE

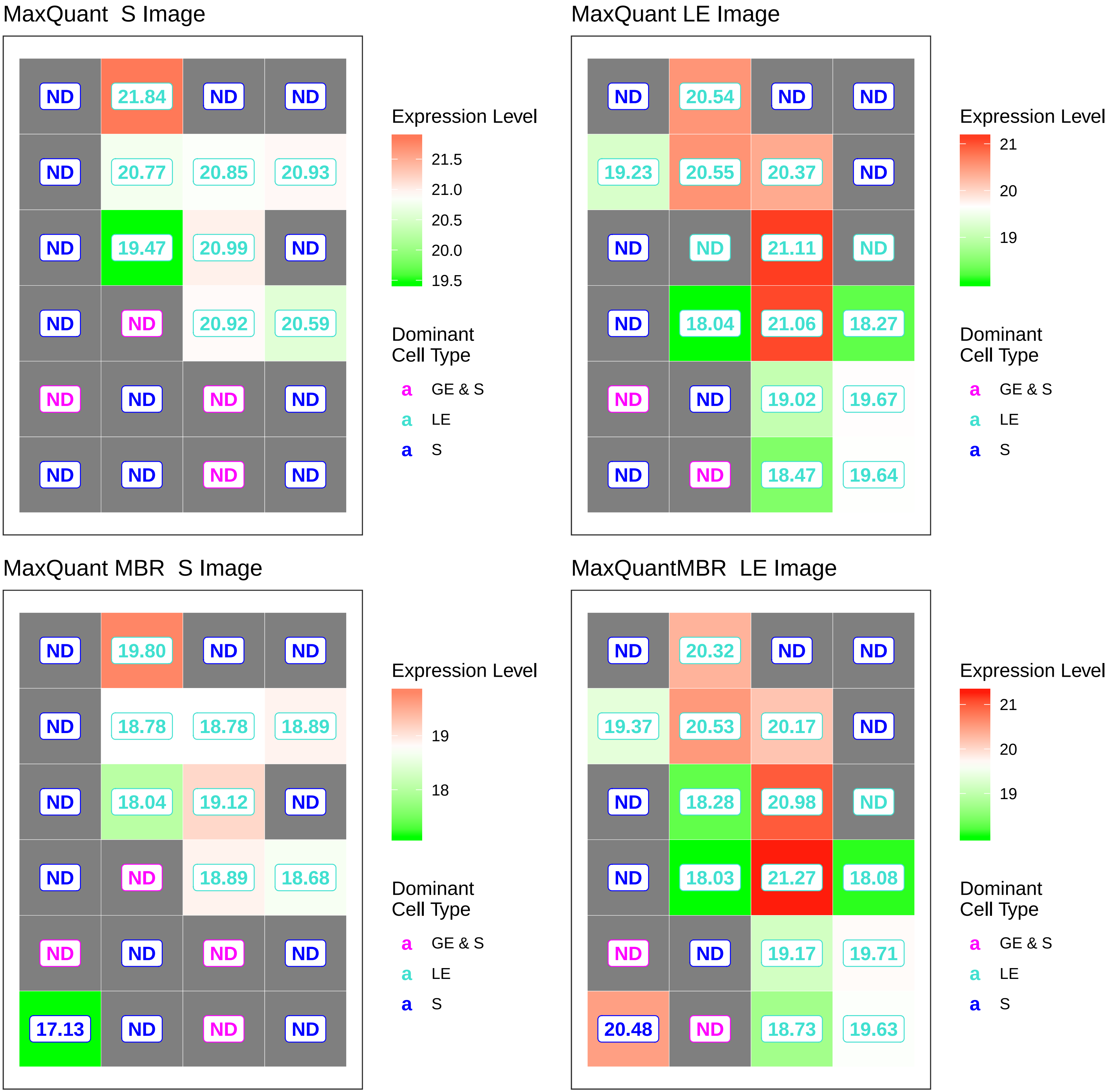

MaxQuant S Image
ND 21.84 ND ND
ND 20.77 20.85 20.93
ND 19.47 20.99 ND
ND ND 20.92 20.59
ND ND ND ND
ND ND ND ND
Expression Level
21.5
21.0
20.5
20.0
19.5
Dominant Cell Type
a GE & S
a LE
a S
MaxQuant LE Image
ND 20.54 ND ND
19.23 20.55 20.37 ND
ND ND 21.11 ND
ND 18.04 21.06 18.27
ND ND 19.02 19.67
ND ND 18.47 19.64
Expression Level
21
20
19
Dominant Cell Type
a GE & S
a LE
a S
MaxQuant MBR S Image
ND 19.80 ND ND
ND 18.78 18.78 18.89
ND 18.04 19.12 ND
ND ND 18.89 18.68
ND ND ND ND
17.13 ND ND ND
Expression Level
19
18
Dominant Cell Type
a GE & S
a LE
a S
MaxQuantMBR LE Image
ND 20.32 ND ND
19.37 20.53 20.17 ND
ND 18.28 20.98 ND
ND 18.03 21.27 18.08
ND ND 19.17 19.71
20.48 ND 18.73 19.63
Expression Level
21
20
19
Dominant Cell Type
a GE & S
a LE
a S

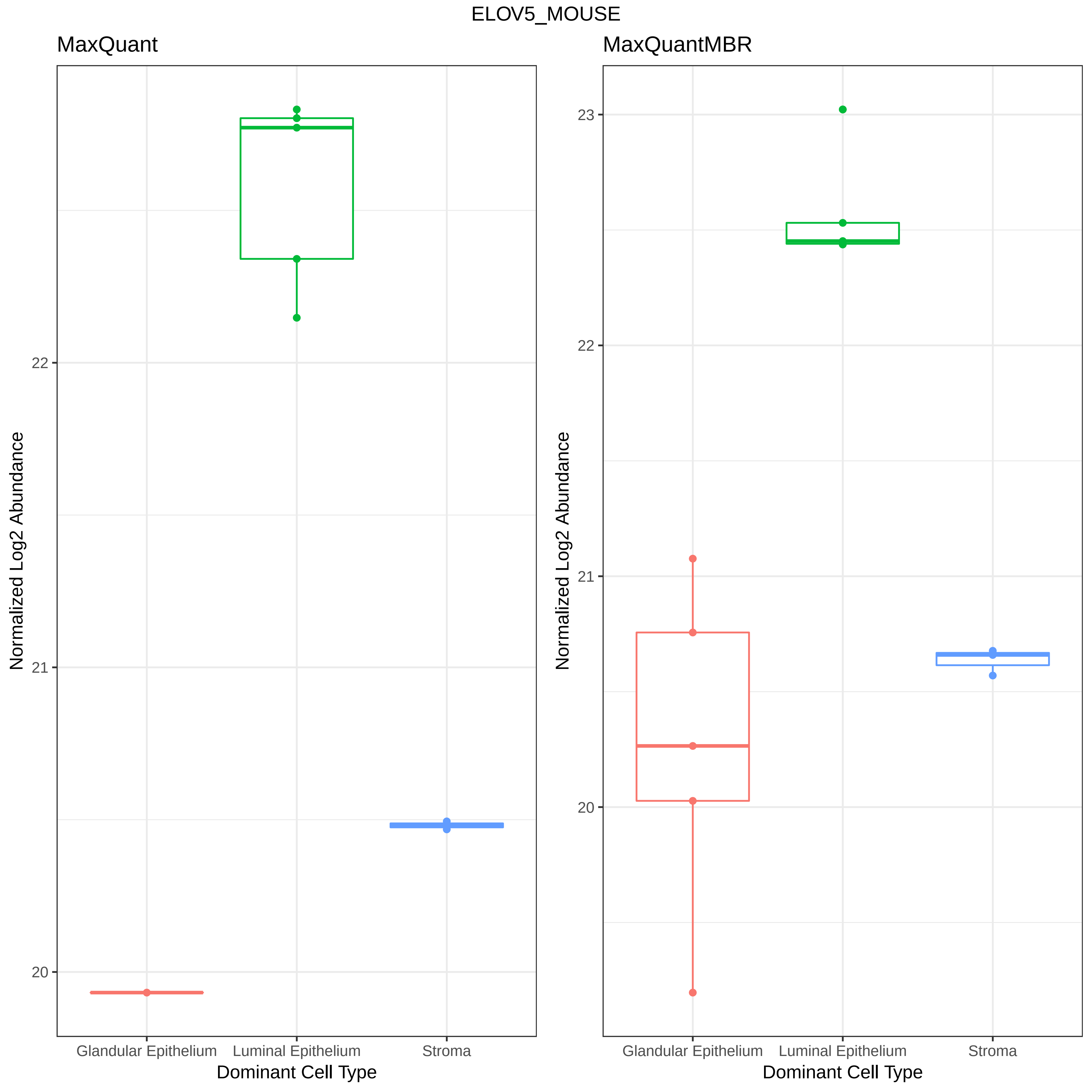
ELOV5_MOUSE
MaxQuant
MaxQuantMBR
Normalized Log2 Abundance
22
21
20
23
Glandular Epithelium
Luminal Epithelium
Stroma
Dominant Cell Type

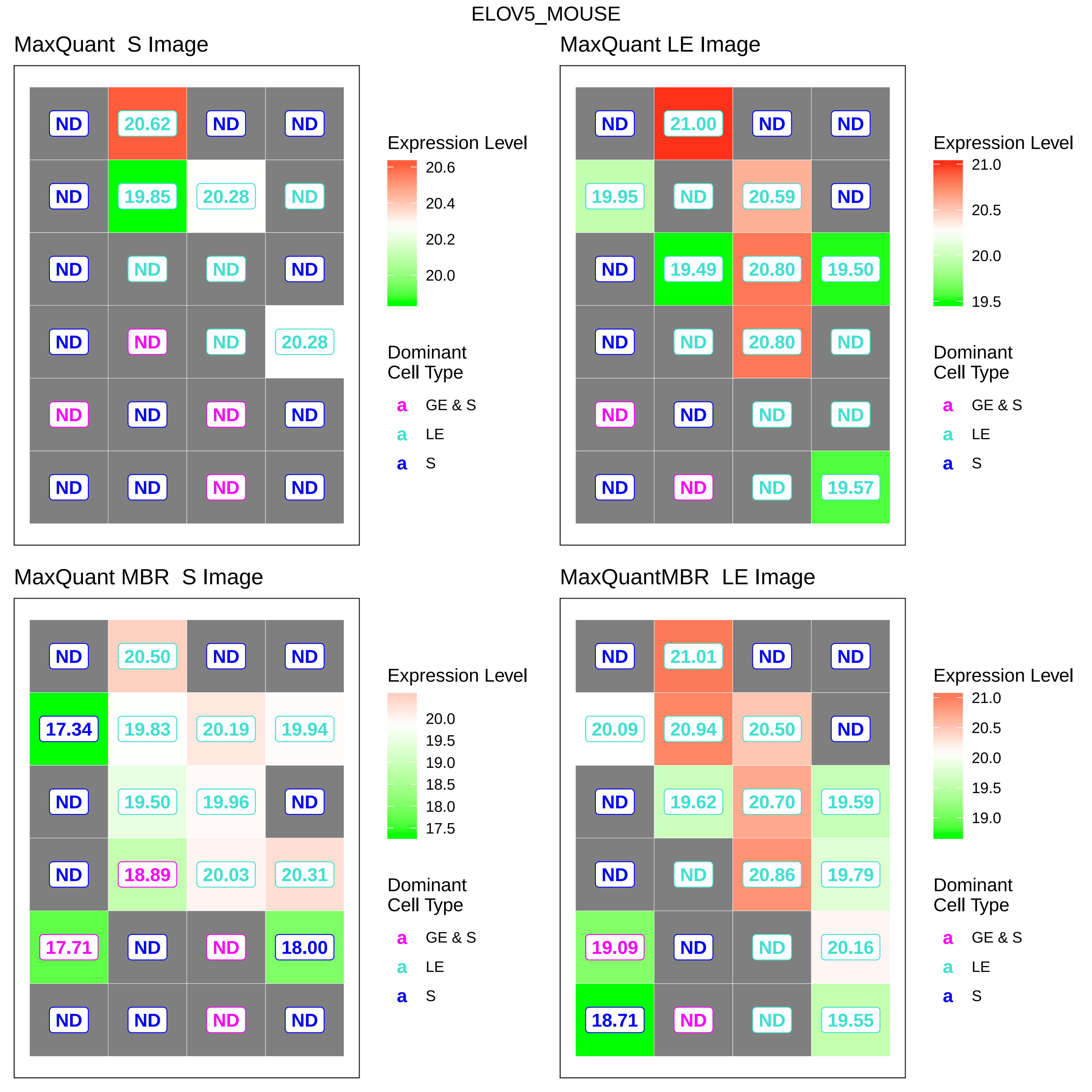
ELOV5_MOUSE
MaxQuant S Image
ND
20.62
ND
ND
ND
19.85
20.28
ND
ND
ND
ND
ND
ND
ND
ND
20.28
ND
ND
ND
ND
ND
ND
ND
ND
Expression Level
20.6
20.4
20.2
20.0
Dominant Cell Type
a GE & S
a LE
a S
MaxQuant LE Image
ND
21.00
ND
ND
19.95
ND
20.59
ND
ND
19.49
20.80
19.50
ND
ND
20.80
ND
ND
ND
ND
ND
ND
ND
ND
19.57
Expression Level
21.0
20.5
20.0
19.5
Dominant Cell Type
a GE & S
a LE
a S
MaxQuant MBR S Image
ND
20.50
ND
ND
17.34
19.83
20.19
19.94
ND
19.50
19.96
ND
ND
18.89
20.03
20.31
17.71
ND
ND
18.00
ND
ND
ND
ND
Expression Level
20.0
19.5
19.0
18.5
18.0
17.5
Dominant Cell Type
a GE & S
a LE
a S
MaxQuantMBR LE Image
ND
21.01
ND
ND
20.09
20.94
20.50
ND
ND
19.62
20.70
19.59
ND
ND
20.86
19.79
19.09
ND
ND
20.16
18.71
ND
ND
19.55
Expression Level
21.0
20.5
20.0
19.5
19.0
Dominant Cell Type
a GE & S
a LE
a S

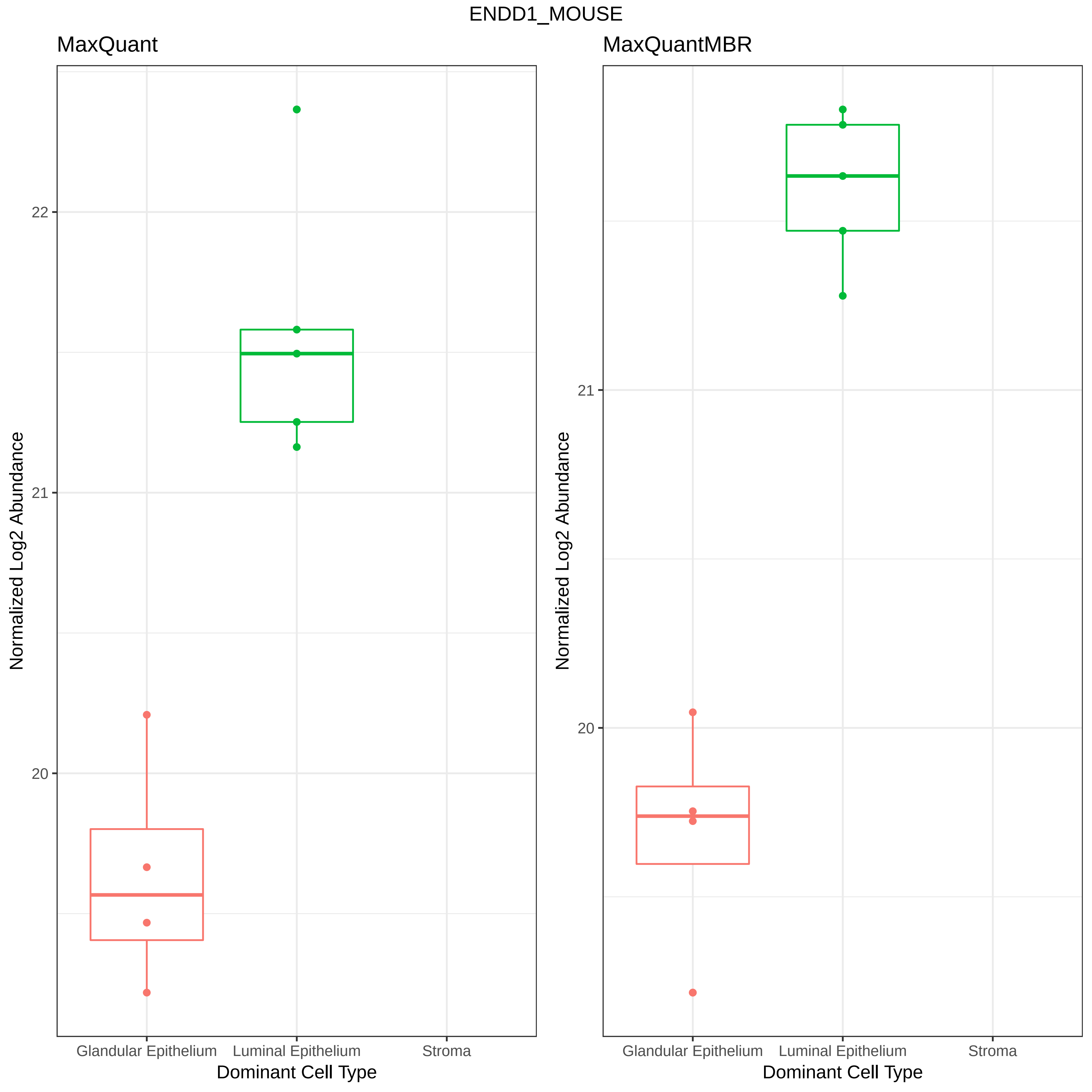

ENDD1_MOUSE
MaxQuant
MaxQuantMBR
Normalized Log2 Abundance
22
21
20
Glandular Epithelium
Luminal Epithelium
Stroma
Dominant Cell Type

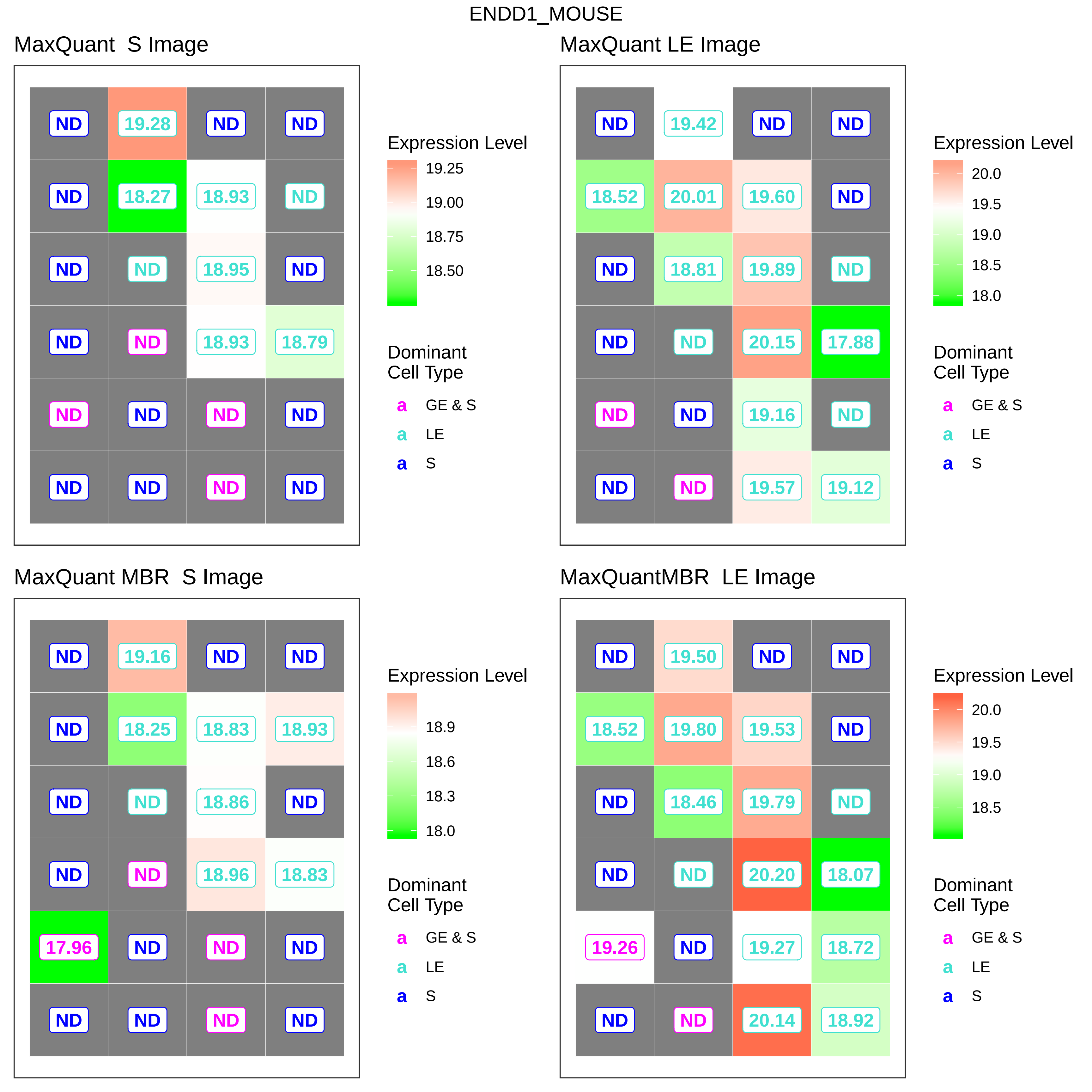
ENDD1_MOUSE
MaxQuant S Image
ND 19.28 ND ND
ND 18.27 18.93 ND
ND ND 18.95 ND
ND ND 18.93 18.79
ND ND ND ND
ND ND ND ND
Expression Level
19.25
19.00
18.75
18.50
Dominant Cell Type
a GE & S
a LE
a S
MaxQuant LE Image
ND 19.42 ND ND
18.52 20.01 19.60 ND
ND 18.81 19.89 ND
ND ND 20.15 17.88
ND ND 19.16 ND
ND ND 19.57 19.12
Expression Level
20.0
19.5
19.0
18.5
18.0
Dominant Cell Type
a GE & S
a LE
a S
MaxQuant MBR S Image
ND 19.16 ND ND
ND 18.25 18.83 18.93
ND ND 18.86 ND
ND ND 18.96 18.83
17.96 ND ND ND
ND ND ND ND
Expression Level
18.9
18.6
18.3
18.0
Dominant Cell Type
a GE & S
a LE
a S
MaxQuantMBR LE Image
ND 19.50 ND ND
18.52 19.80 19.53 ND
ND 18.46 19.79 ND
ND ND 20.20 18.07
19.26 ND 19.27 18.72
ND ND 20.14 18.92
Expression Level
20.0
19.5
19.0
18.5
Dominant Cell Type
a GE & S
a LE
a S

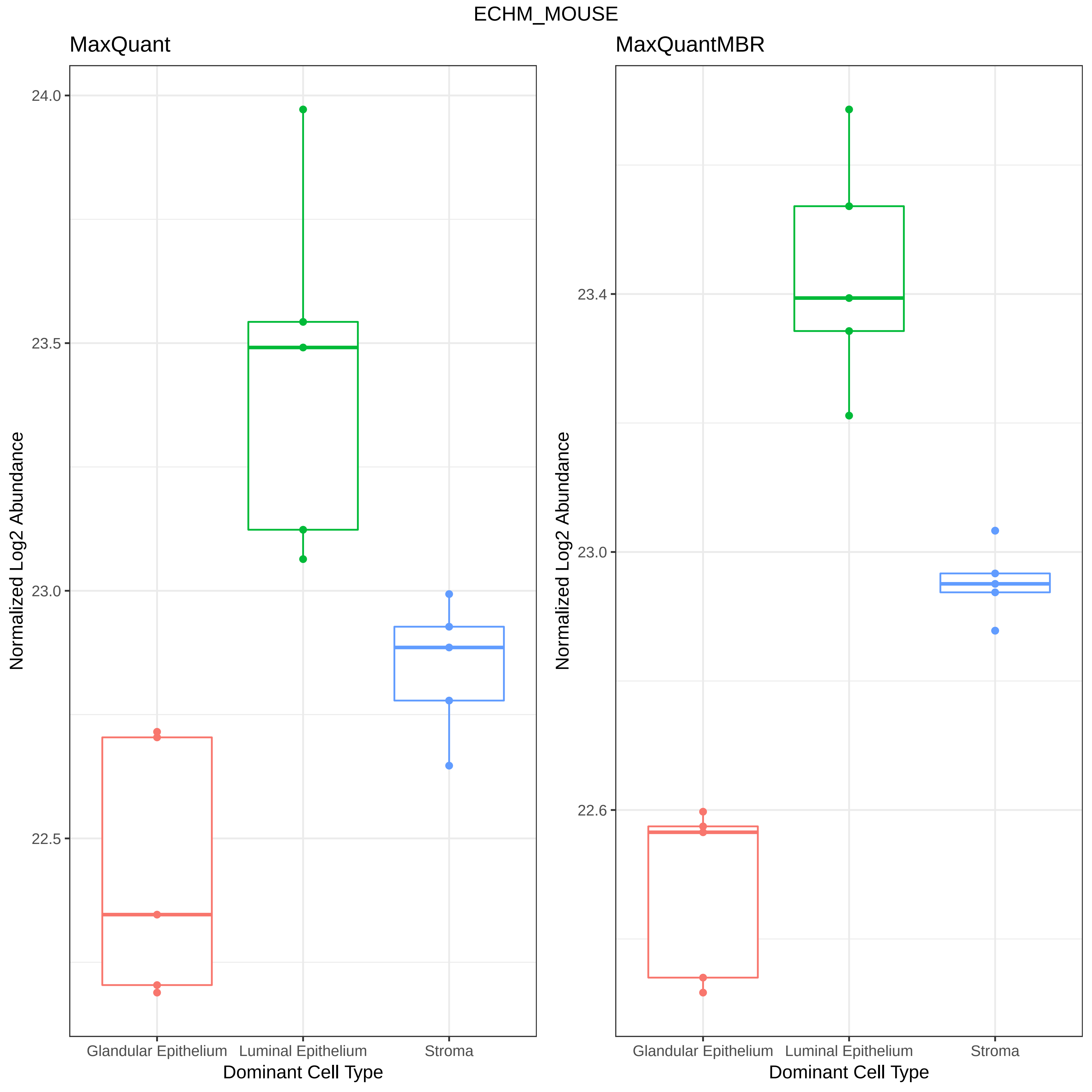

ECHM_MOUSE
MaxQuant
MaxQuantMBR
24.0
23.5
23.0
22.5
23.4
23.0
22.6
Normalized Log2 Abundance
Glandular Epithelium
Luminal Epithelium
Stroma
Dominant Cell Type

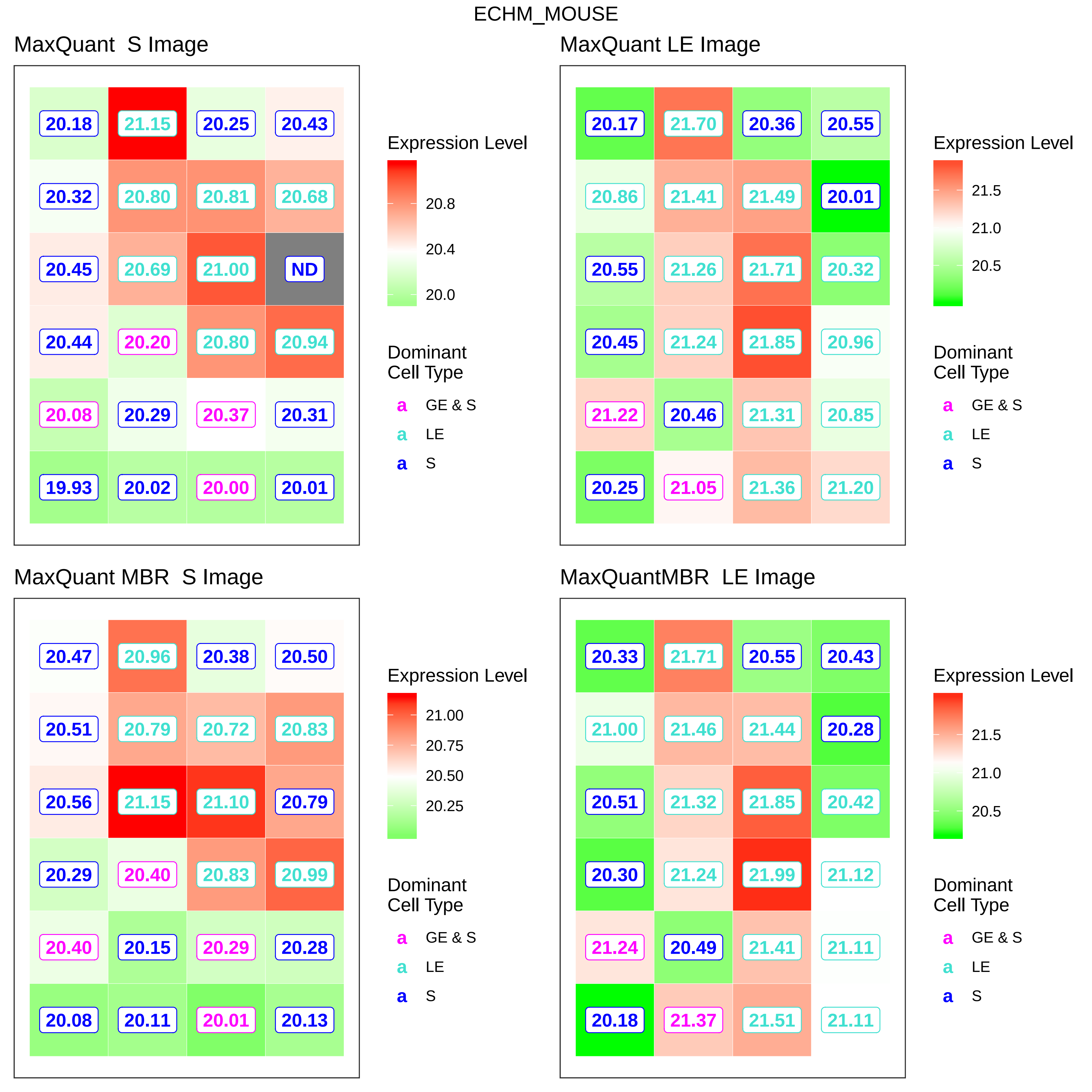
ECHM_MOUSE
MaxQuant S Image
20.18 21.15 20.25 20.43
20.32 20.80 20.81 20.68
20.45 20.69 21.00 ND
20.44 20.20 20.80 20.94
20.08 20.29 20.37 20.31
19.93 20.02 20.00 20.01
Expression Level
20.8
20.4
20.0
Dominant Cell Type
a GE & S
a LE
a S
MaxQuant LE Image
20.17 21.70 20.36 20.55
20.86 21.41 21.49 20.01
20.55 21.26 21.71 20.32
20.45 21.24 21.85 20.96
21.22 20.46 21.31 20.85
20.25 21.05 21.36 21.20
Expression Level
21.5
21.0
20.5
Dominant Cell Type
a GE & S
a LE
a S
MaxQuant MBR S Image
20.47 20.96 20.38 20.50
20.51 20.79 20.72 20.83
20.56 21.15 21.10 20.79
20.29 20.40 20.83 20.99
20.40 20.15 20.29 20.28
20.08 20.11 20.01 20.13
Expression Level
21.00
20.75
20.50
20.25
Dominant Cell Type
a GE & S
a LE
a S
MaxQuantMBR LE Image
20.33 21.71 20.55 20.43
21.00 21.46 21.44 20.28
20.51 21.32 21.85 20.42
20.30 21.24 21.99 21.12
21.24 20.49 21.41 21.11
20.18 21.37 21.51 21.11
Expression Level
21.5
21.0
20.5
Dominant Cell Type
a GE & S
a LE
a S

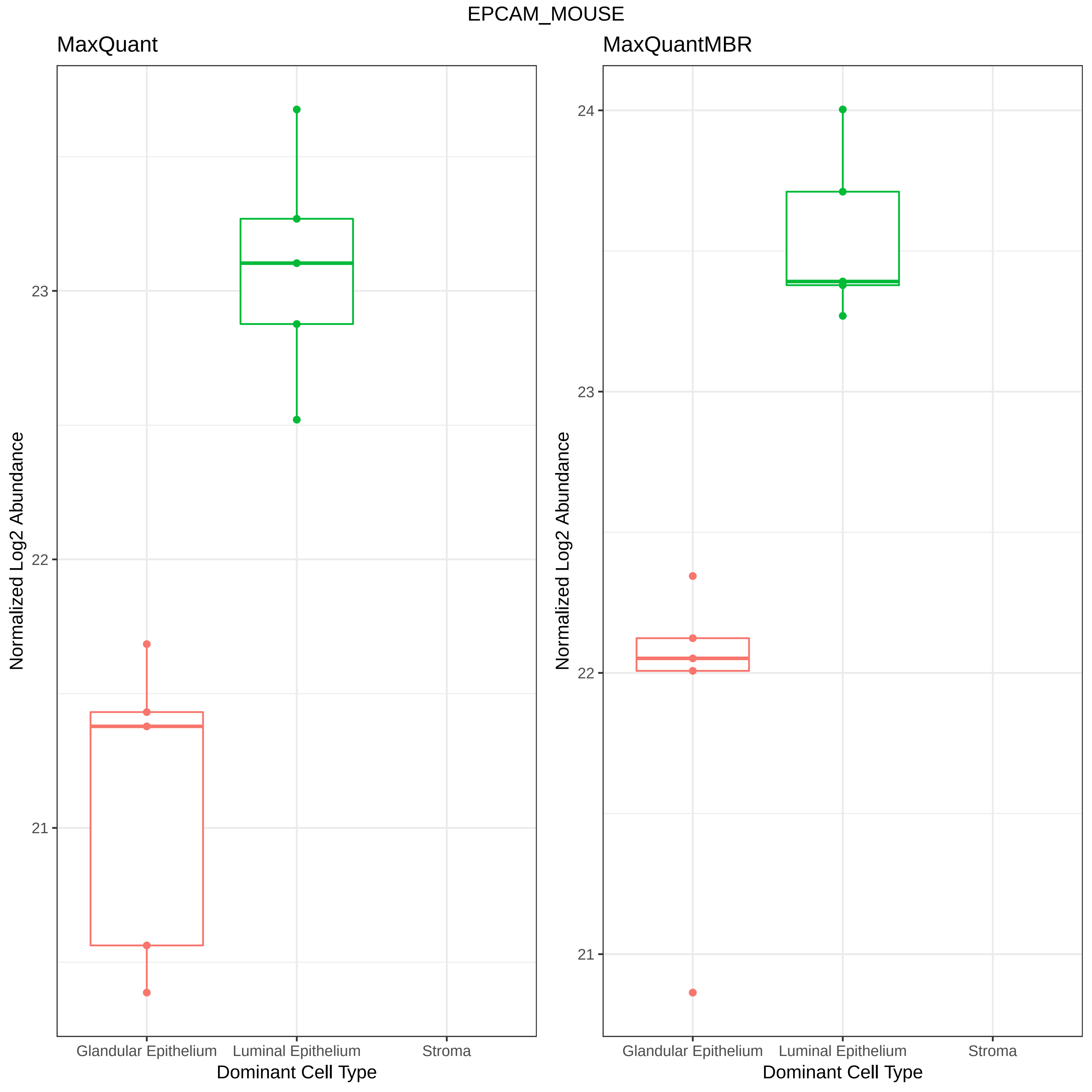

EPCAM_MOUSE
MaxQuant
MaxQuantMBR
Normalized Log2 Abundance
21
22
23
24
Glandular Epithelium
Luminal Epithelium
Stroma
Dominant Cell Type

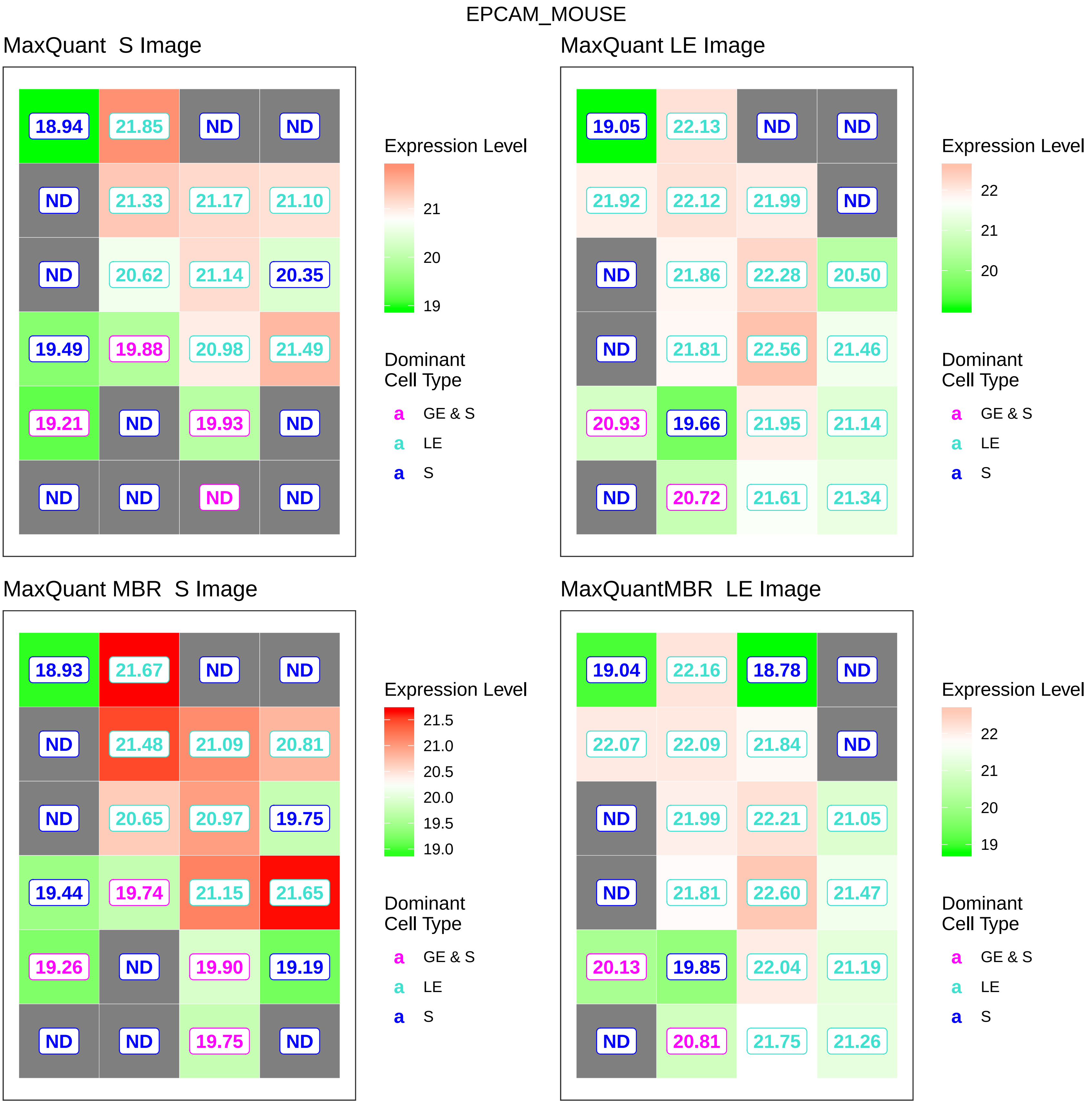
EPCAM_MOUSE
MaxQuant S Image
18.94 21.85 ND ND
ND 21.33 21.17 21.10
ND 20.62 21.14 20.35
19.49 19.88 20.98 21.49
19.21 ND 19.93 ND
ND ND ND ND
Expression Level
21
20
19
Dominant Cell Type
a GE & S
a LE
a S
MaxQuant LE Image
19.05 22.13 ND ND
21.92 22.12 21.99 ND
ND 21.86 22.28 20.50
ND 21.81 22.56 21.46
20.93 19.66 21.95 21.14
ND 20.72 21.61 21.34
Expression Level
22
21
20
Dominant Cell Type
a GE & S
a LE
a S
MaxQuant MBR S Image
18.93 21.67 ND ND
ND 21.48 21.09 20.81
ND 20.65 20.97 19.75
19.44 19.74 21.15 21.65
19.26 ND 19.90 19.19
ND ND 19.75 ND
Expression Level
21.5
21.0
20.5
20.0
19.5
19.0
Dominant Cell Type
a GE & S
a LE
a S
MaxQuantMBR LE Image
19.04 22.16 18.78 ND
22.07 22.09 21.84 ND
ND 21.99 22.21 21.05
ND 21.81 22.60 21.47
20.13 19.85 22.04 21.19
ND 20.81 21.75 21.26
Expression Level
22
21
20
19
Dominant Cell Type
a GE & S
a LE
a S

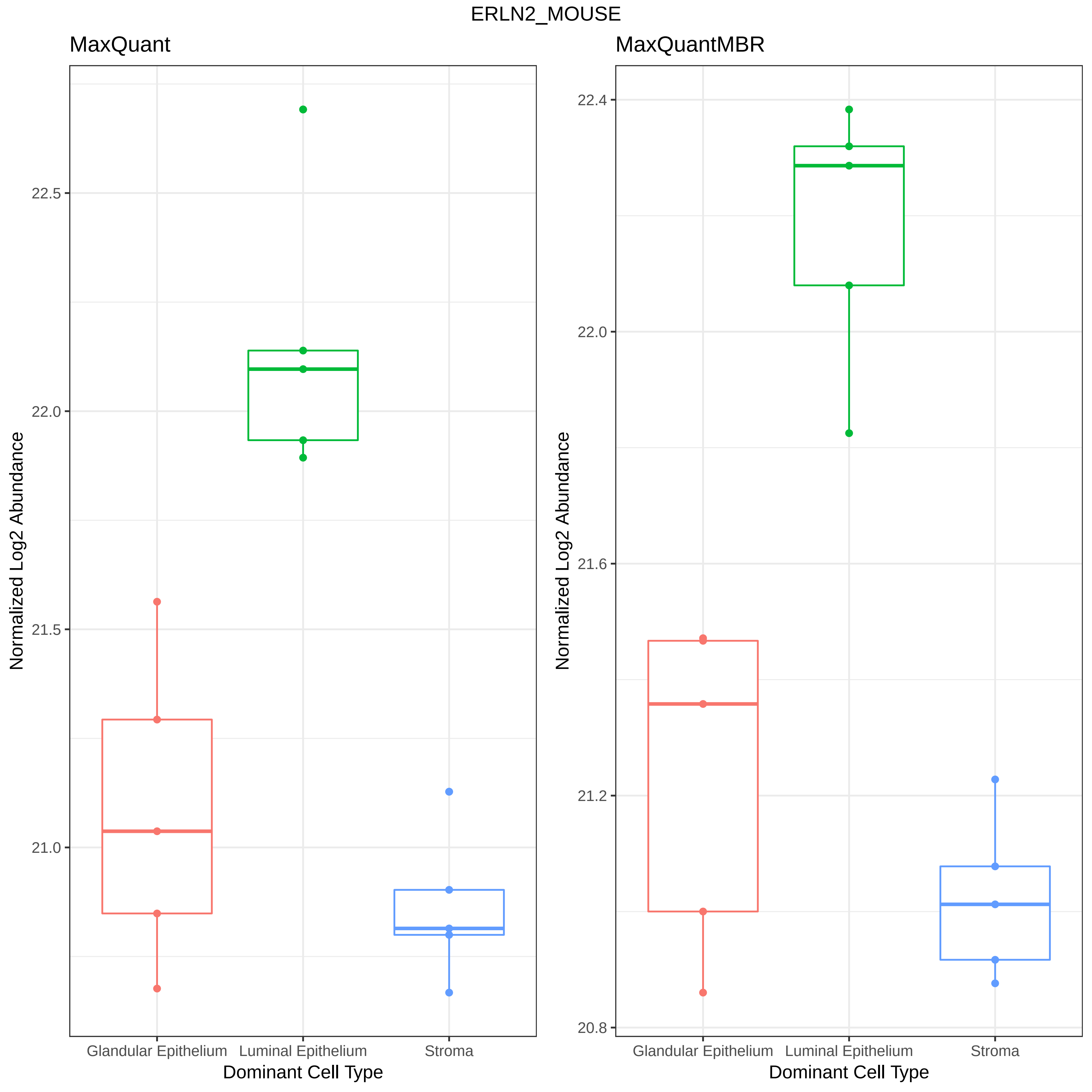
ERLN2_MOUSE
MaxQuant
MaxQuantMBR
Normalized Log2 Abundance
22.5
22.0
21.5
21.0
22.4
22.0
21.6
21.2
20.8
Glandular Epithelium
Luminal Epithelium
Stroma
Dominant Cell Type

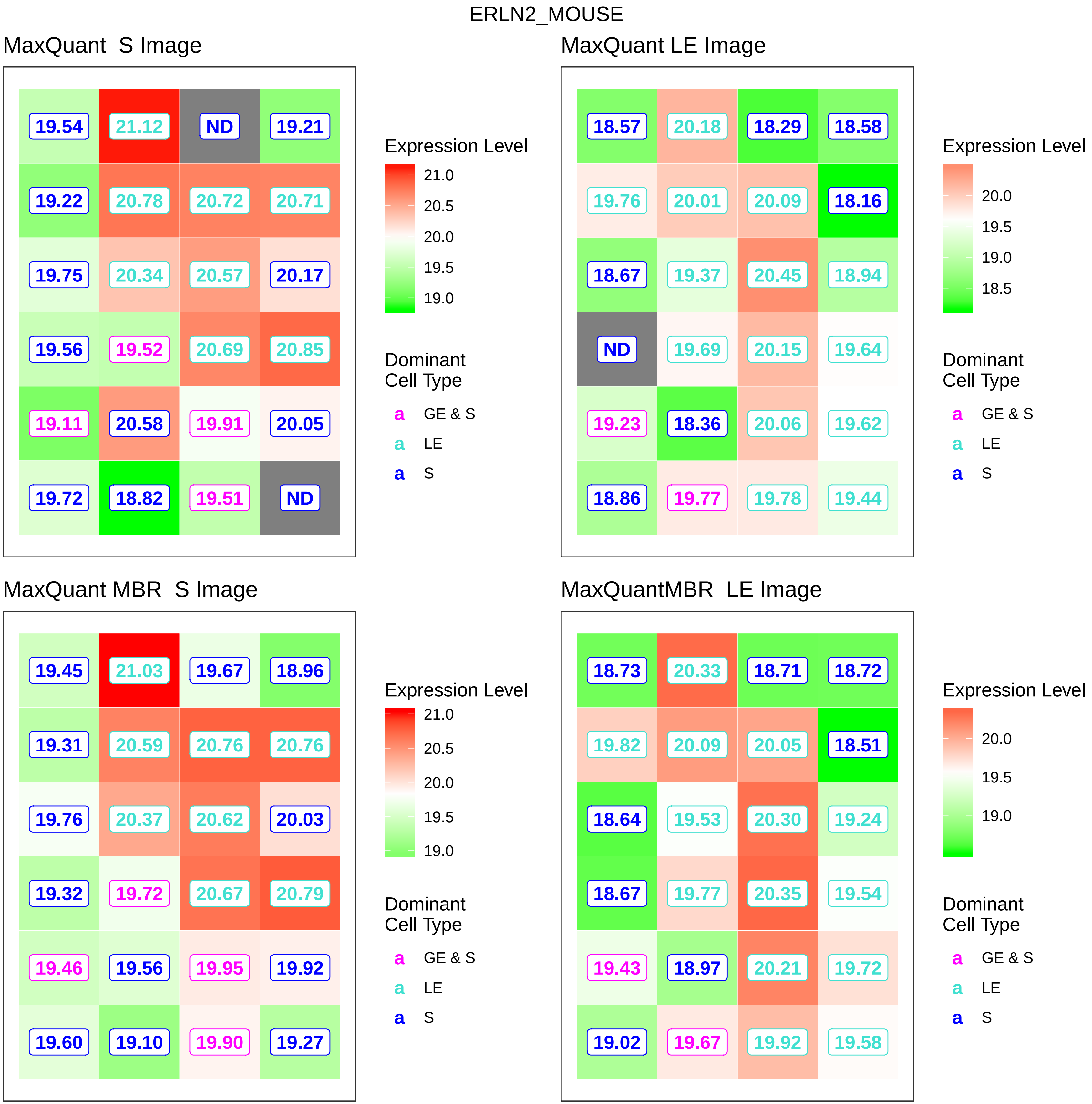

ERLN2_MOUSE
MaxQuant S Image
19.54 21.12 ND 19.21
19.22 20.78 20.72 20.71
19.75 20.34 20.57 20.17
19.56 19.52 20.69 20.85
19.11 20.58 19.91 20.05
19.72 18.82 19.51 ND
Expression Level
21.0
20.5
20.0
19.5
19.0
Dominant Cell Type
a GE & S
a LE
a S
MaxQuant LE Image
18.57 20.18 18.29 18.58
19.76 20.01 20.09 18.16
18.67 19.37 20.45 18.94
ND 19.69 20.15 19.64
19.23 18.36 20.06 19.62
18.86 19.77 19.78 19.44
Expression Level
20.0
19.5
19.0
18.5
Dominant Cell Type
a GE & S
a LE
a S
MaxQuant MBR S Image
19.45 21.03 19.67 18.96
19.31 20.59 20.76 20.76
19.76 20.37 20.62 20.03
19.32 19.72 20.67 20.79
19.46 19.56 19.95 19.92
19.60 19.10 19.90 19.27
Expression Level
21.0
20.5
20.0
19.5
19.0
Dominant Cell Type
a GE & S
a LE
a S
MaxQuantMBR LE Image
18.73 20.33 18.71 18.72
19.82 20.09 20.05 18.51
18.64 19.53 20.30 19.24
18.67 19.77 20.35 19.54
19.43 18.97 20.21 19.72
19.02 19.67 19.92 19.58
Expression Level
20.0
19.5
19.0
Dominant Cell Type
a GE & S
a LE
a S

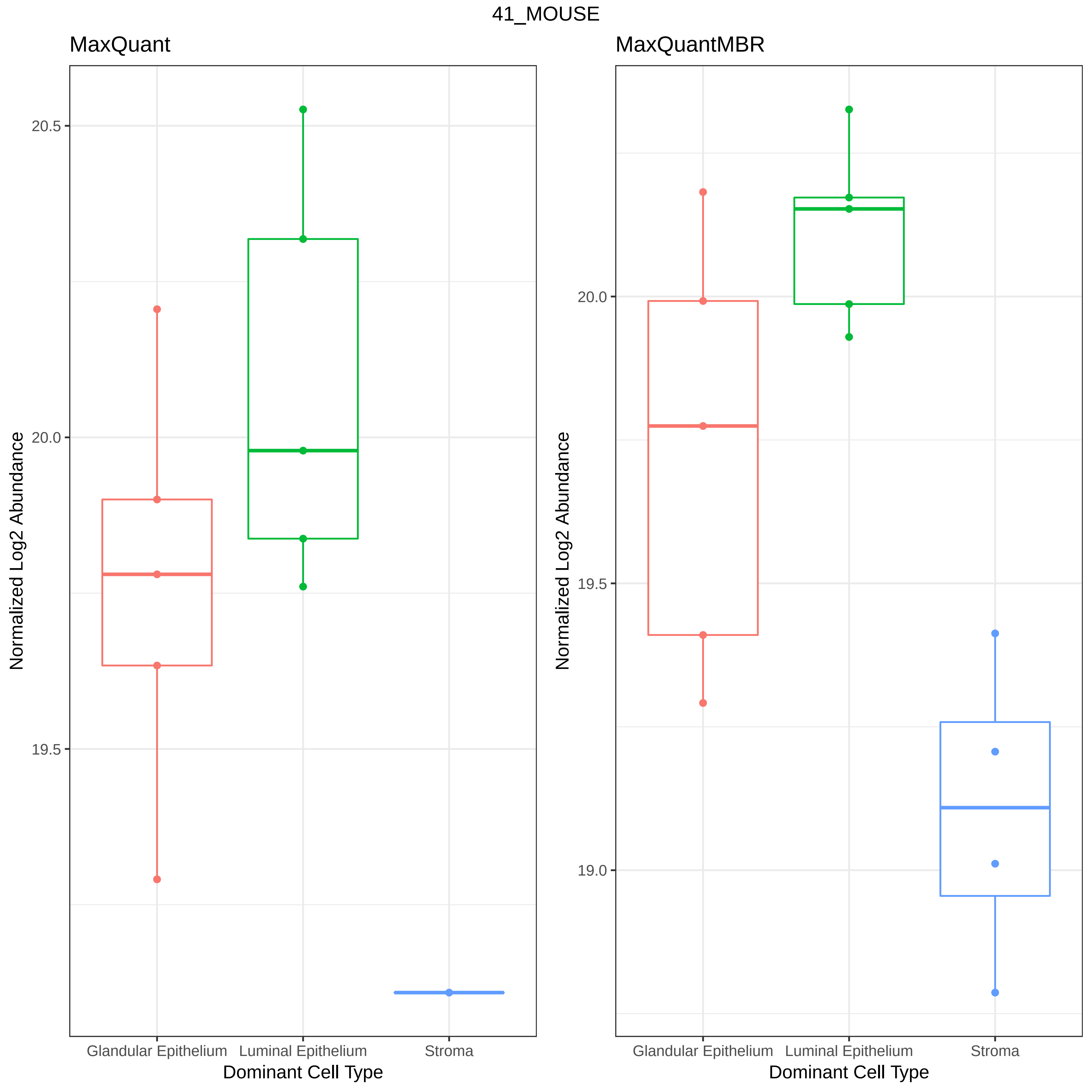
41_MOUSE
MaxQuant
MaxQuantMBR
Normalized Log2 Abundance
20.5
20.0
19.5
19.0
Glandular Epithelium
Luminal Epithelium
Stroma
Dominant Cell Type

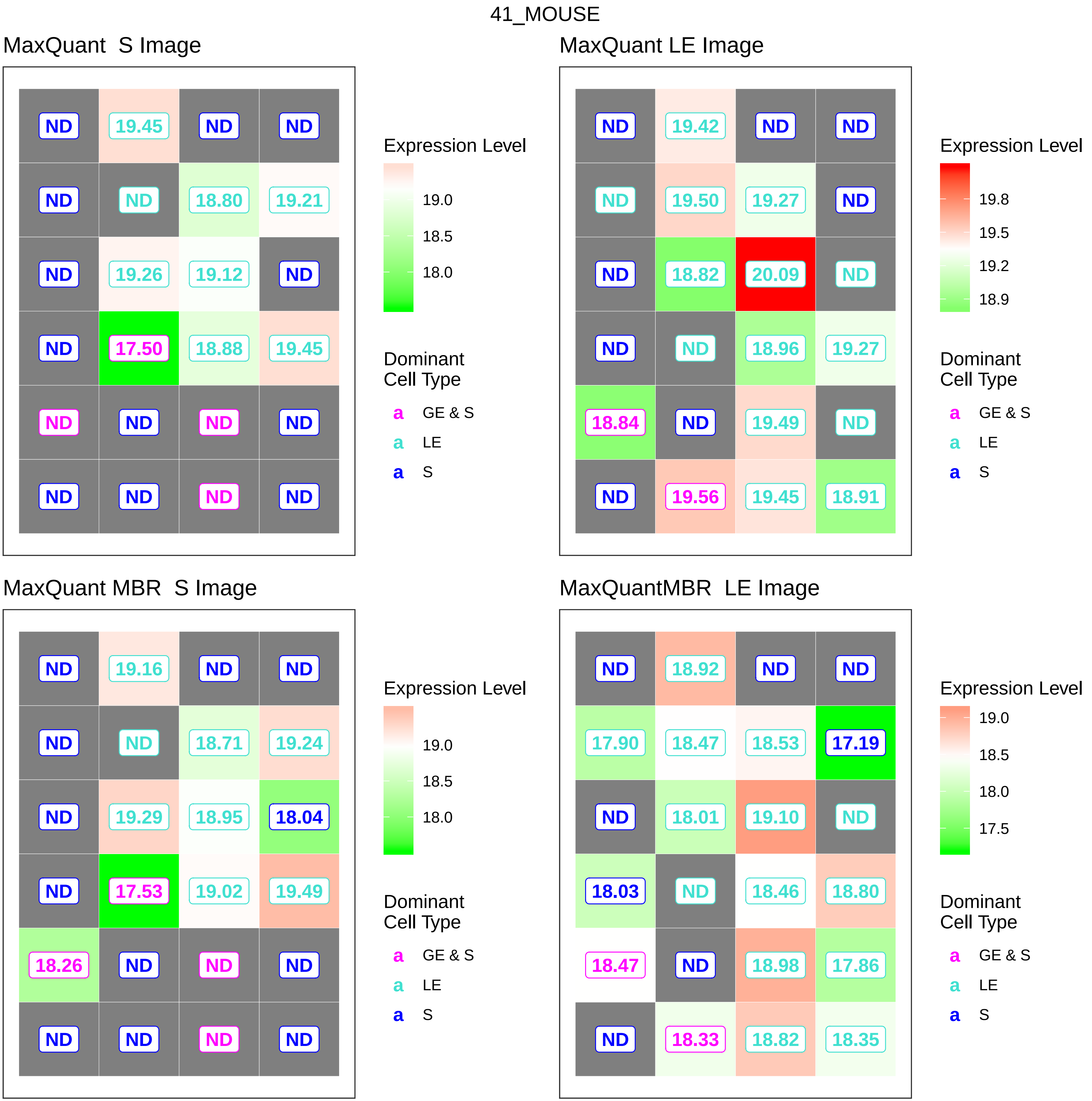
41_MOUSE
MaxQuant S Image
ND 19.45 ND ND
ND ND 18.80 19.21
ND 19.26 19.12 ND
ND 17.50 18.88 19.45
ND ND ND ND
ND ND ND ND
Expression Level
19.0
18.5
18.0
Dominant Cell Type
a GE & S
a LE
a S
MaxQuant LE Image
ND 19.42 ND ND
ND 19.50 19.27 ND
ND 18.82 20.09 ND
ND ND 18.96 19.27
18.84 ND 19.49 ND
ND 19.56 19.45 18.91
Expression Level
19.8
19.5
19.2
18.9
Dominant Cell Type
a GE & S
a LE
a S
MaxQuant MBR S Image
ND 19.16 ND ND
ND ND 18.71 19.24
ND 19.29 18.95 18.04
ND 17.53 19.02 19.49
18.26 ND ND ND
ND ND ND ND
Expression Level
19.0
18.5
18.0
Dominant Cell Type
a GE & S
a LE
a S
MaxQuantMBR LE Image
ND 18.92 ND ND
17.90 18.47 18.53 17.19
ND 18.01 19.10 ND
18.03 ND 18.46 18.80
18.47 ND 18.98 17.86
ND 18.33 18.82 18.35
Expression Level
19.0
18.5
18.0
17.5
Dominant Cell Type
a GE & S
a LE
a S

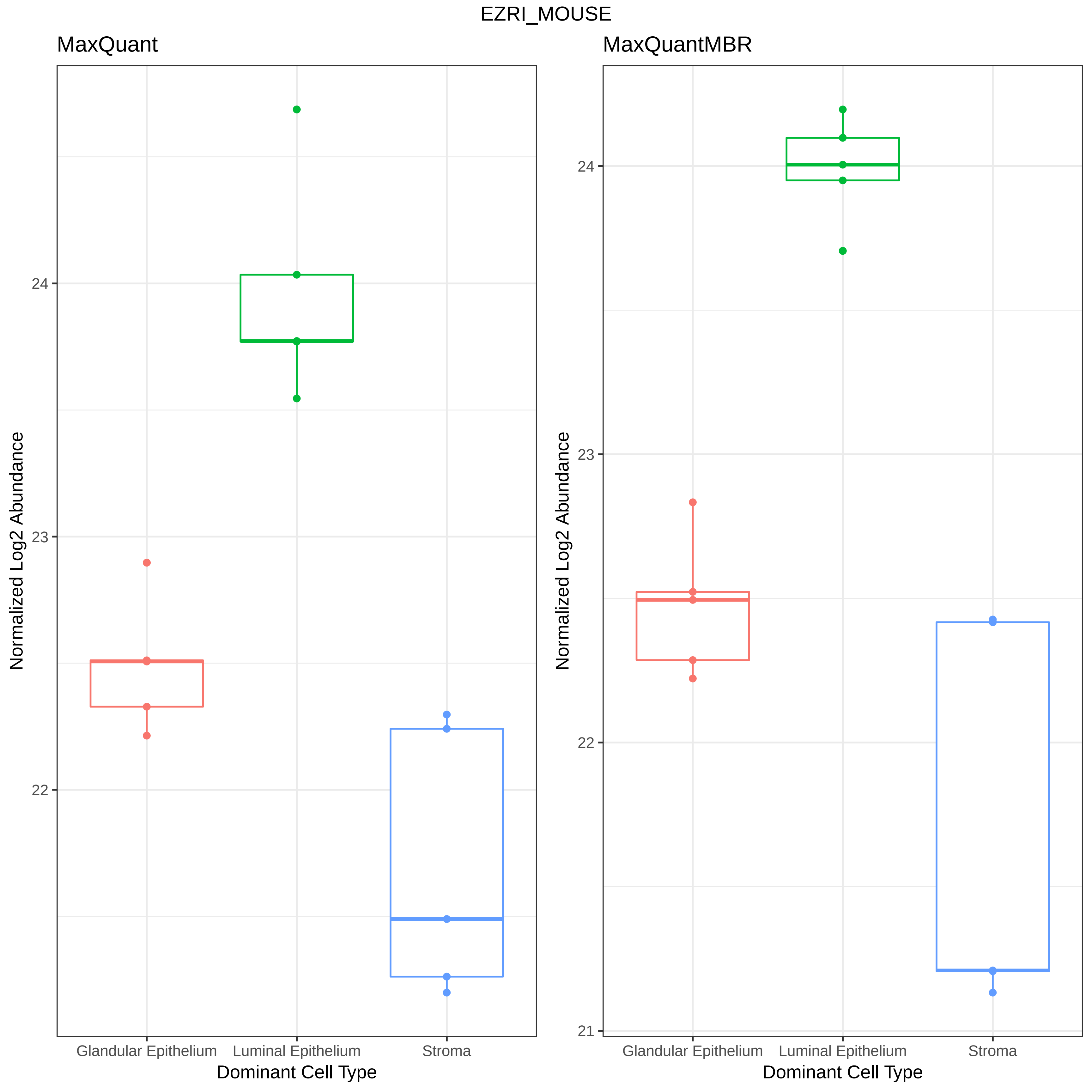
EZRI_MOUSE
MaxQuant
MaxQuantMBR
Normalized Log2 Abundance
24
23
22
21
Glandular Epithelium
Luminal Epithelium
Stroma
Dominant Cell Type

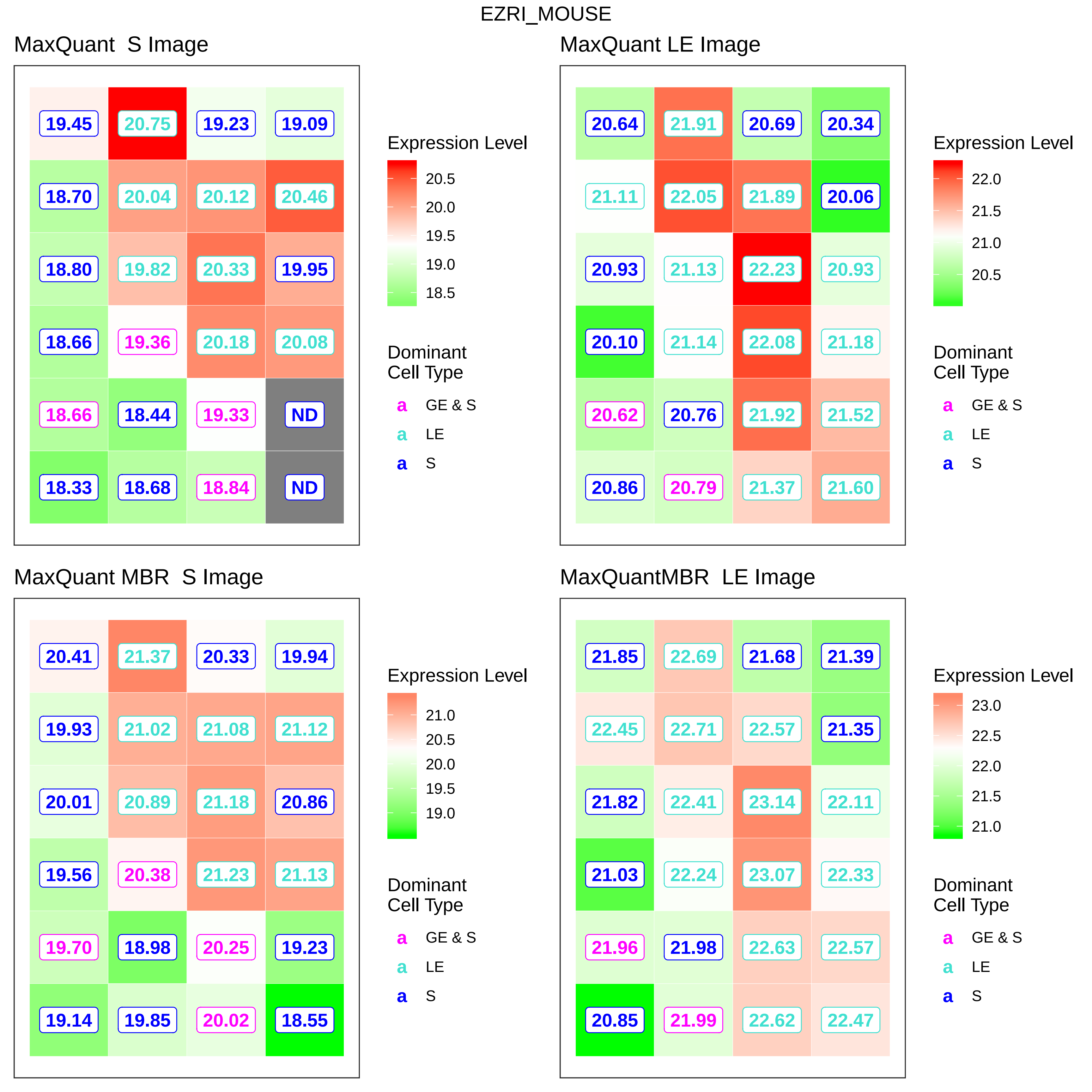
EZRI_MOUSE
MaxQuant S Image
19.45 20.75 19.23 19.09
18.70 20.04 20.12 20.46
18.80 19.82 20.33 19.95
18.66 19.36 20.18 20.08
18.66 18.44 19.33 ND
18.33 18.68 18.84 ND
Expression Level
20.5
20.0
19.5
19.0
18.5
Dominant Cell Type
a GE & S
a LE
a S
MaxQuant LE Image
20.64 21.91 20.69 20.34
21.11 22.05 21.89 20.06
20.93 21.13 22.23 20.93
20.10 21.14 22.08 21.18
20.62 20.76 21.92 21.52
20.86 20.79 21.37 21.60
Expression Level
22.0
21.5
21.0
20.5
Dominant Cell Type
a GE & S
a LE
a S
MaxQuant MBR S Image
20.41 21.37 20.33 19.94
19.93 21.02 21.08 21.12
20.01 20.89 21.18 20.86
19.56 20.38 21.23 21.13
19.70 18.98 20.25 19.23
19.14 19.85 20.02 18.55
Expression Level
21.0
20.5
20.0
19.5
19.0
Dominant Cell Type
a GE & S
a LE
a S
MaxQuantMBR LE Image
21.85 22.69 21.68 21.39
22.45 22.71 22.57 21.35
21.82 22.41 23.14 22.11
21.03 22.24 23.07 22.33
21.96 21.98 22.63 22.57
20.85 21.99 22.62 22.47
Expression Level
23.0
22.5
22.0
21.5
21.0
Dominant Cell Type
a GE & S
a LE
a S

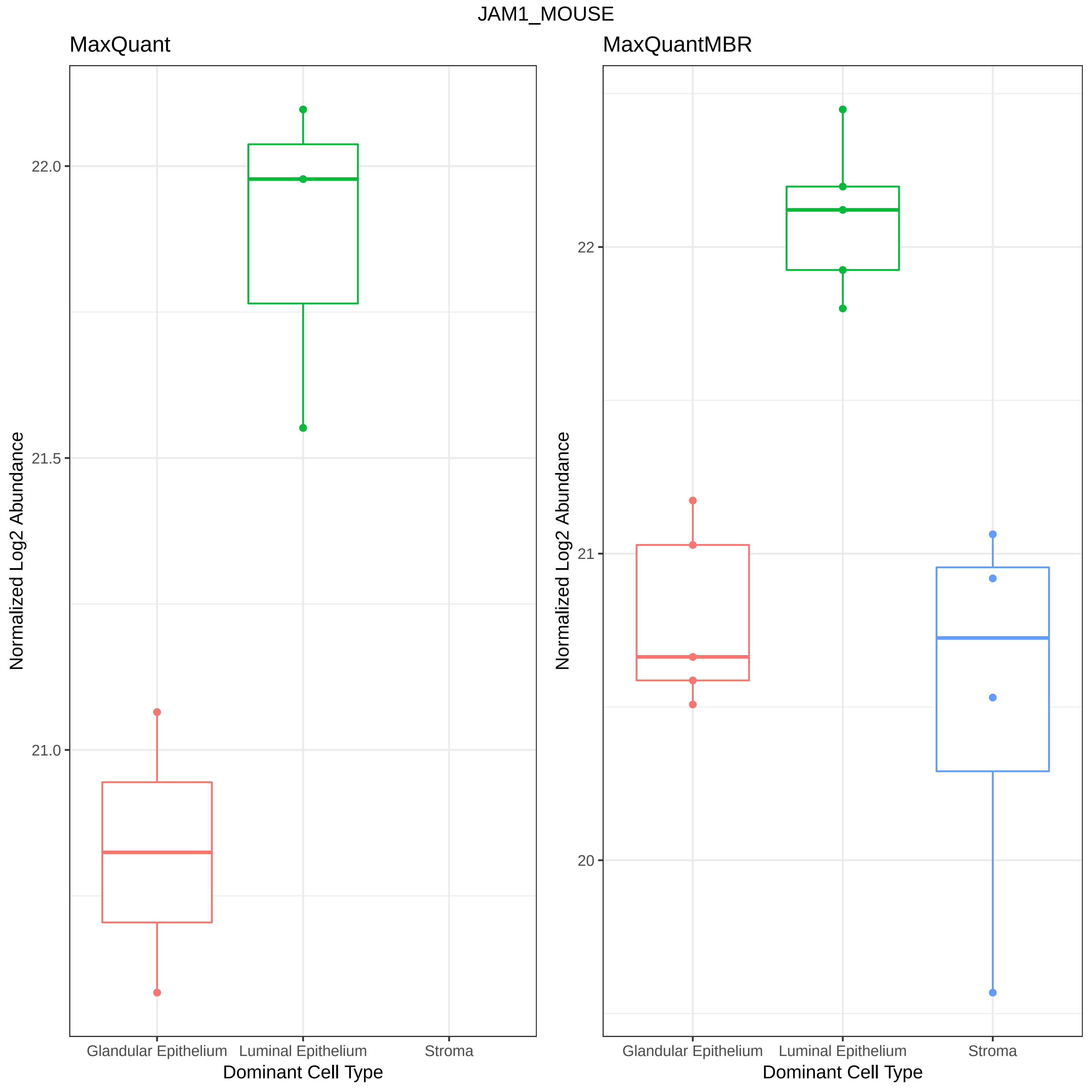
JAM1_MOUSE
MaxQuant
MaxQuantMBR
Normalized Log2 Abundance
22.0
21.5
21.0
22
21
20
Glandular Epithelium
Luminal Epithelium
Stroma
Dominant Cell Type

JAM1_MOUSE

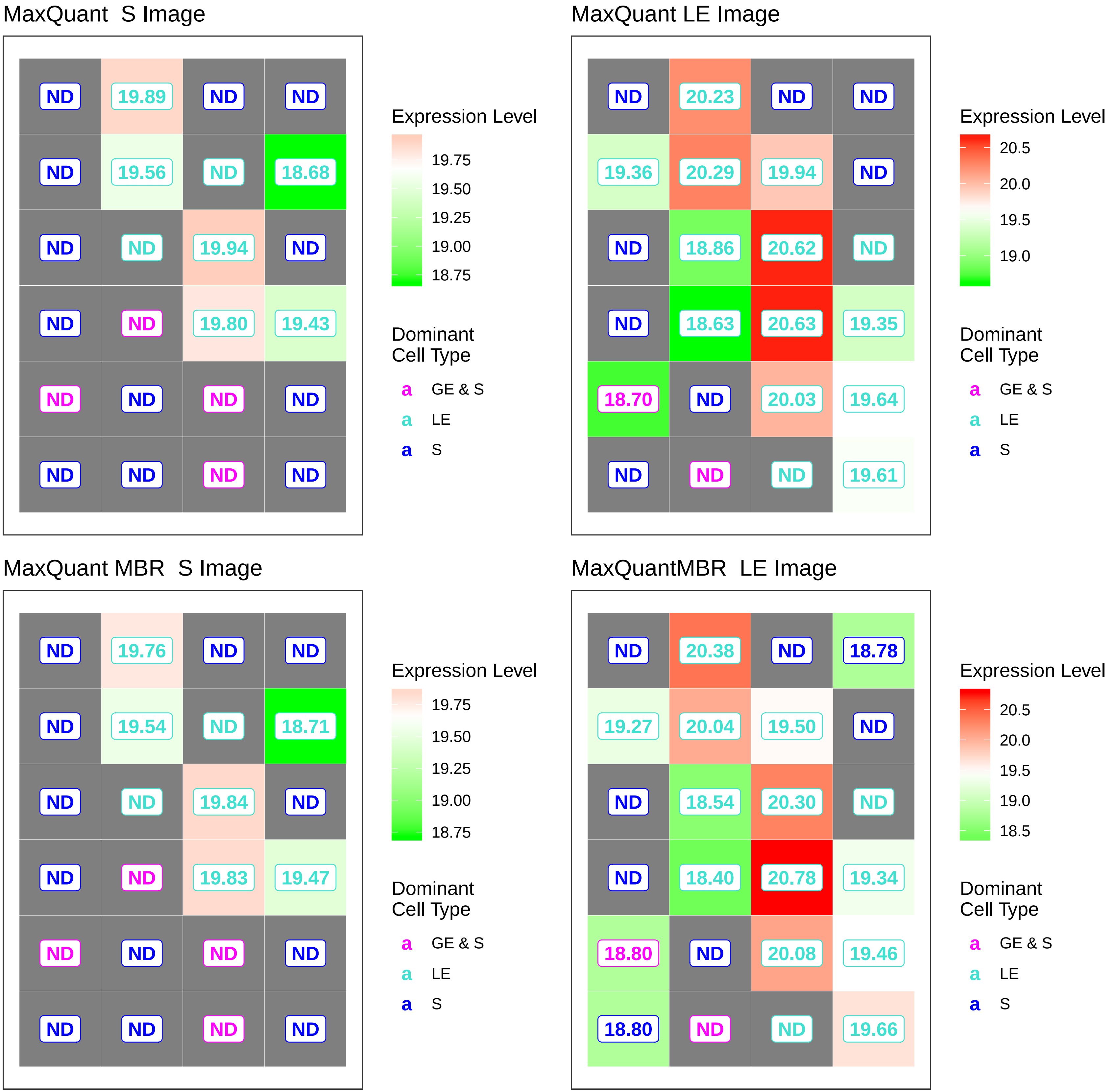
MaxQuant S Image
ND
19.89
ND
ND
ND
19.56
ND
18.68
ND
ND
19.94
ND
ND
ND
19.80
19.43
ND
ND
ND
ND
ND
ND
ND
ND
Expression Level
19.75
19.50
19.25
19.00
18.75
Dominant Cell Type
a GE & S
a LE
a S
MaxQuant LE Image
ND
20.23
ND
ND
19.36
20.29
19.94
ND
ND
18.86
20.62
ND
ND
18.63
20.63
19.35
18.70
ND
20.03
19.64
ND
ND
ND
19.61
Expression Level
20.5
20.0
19.5
19.0
Dominant Cell Type
a GE & S
a LE
a S
MaxQuant MBR S Image
ND
19.76
ND
ND
ND
19.54
ND
18.71
ND
ND
19.84
ND
ND
ND
19.83
19.47
ND
ND
ND
ND
ND
ND
ND
ND
Expression Level
19.75
19.50
19.25
19.00
18.75
Dominant Cell Type
a GE & S
a LE
a S
MaxQuantMBR LE Image
ND
20.38
ND
18.78
19.27
20.04
19.50
ND
ND
18.54
20.30
ND
ND
18.40
20.78
19.34
18.80
ND
20.08
19.46
18.80
ND
ND
19.66
Expression Level
20.5
20.0
19.5
19.0
18.5
Dominant Cell Type
a GE & S
a LE
a S

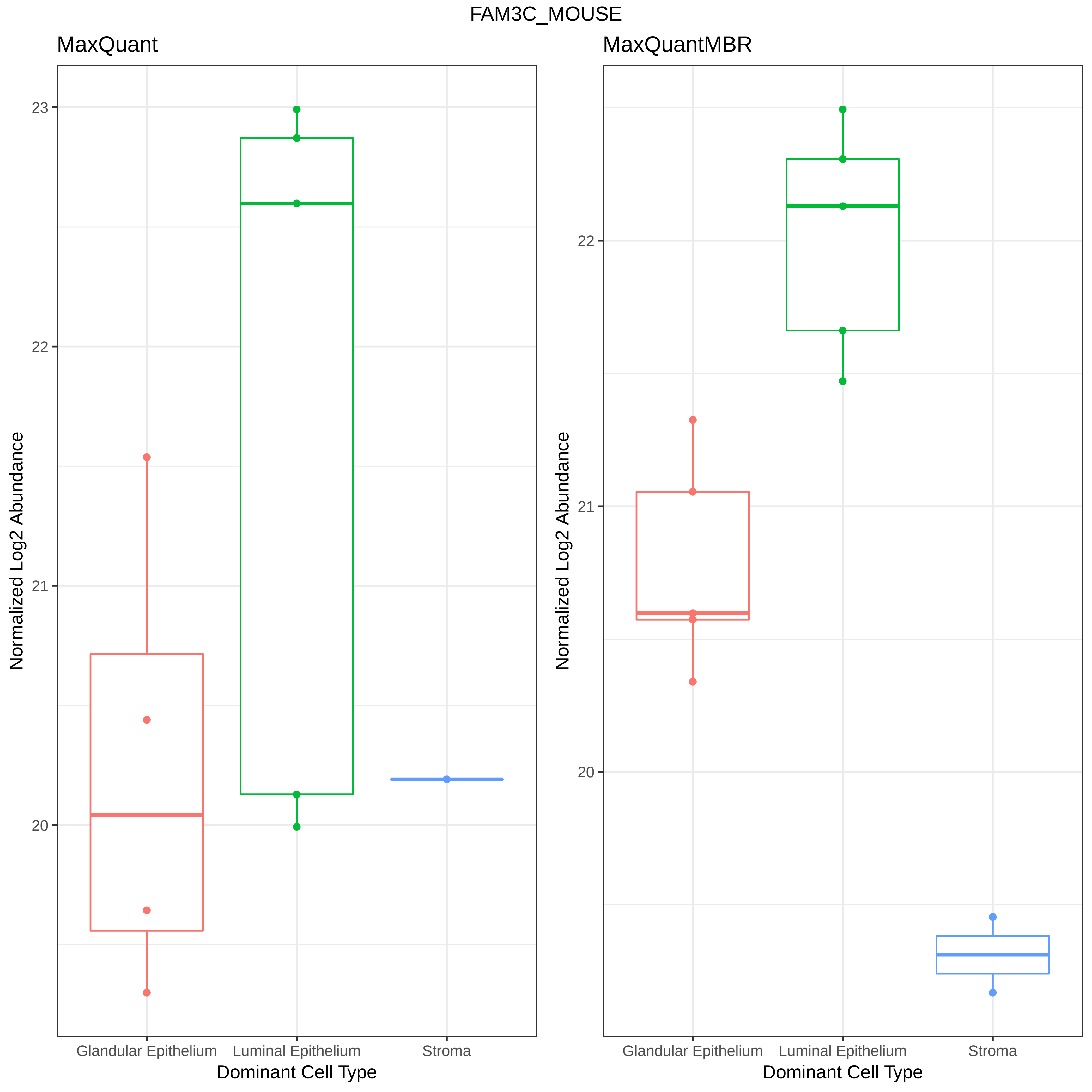
FAM3C_MOUSE
MaxQuant
MaxQuantMBR
Normalized Log2 Abundance
23
22
21
20
Glandular Epithelium
Luminal Epithelium
Stroma
Dominant Cell Type

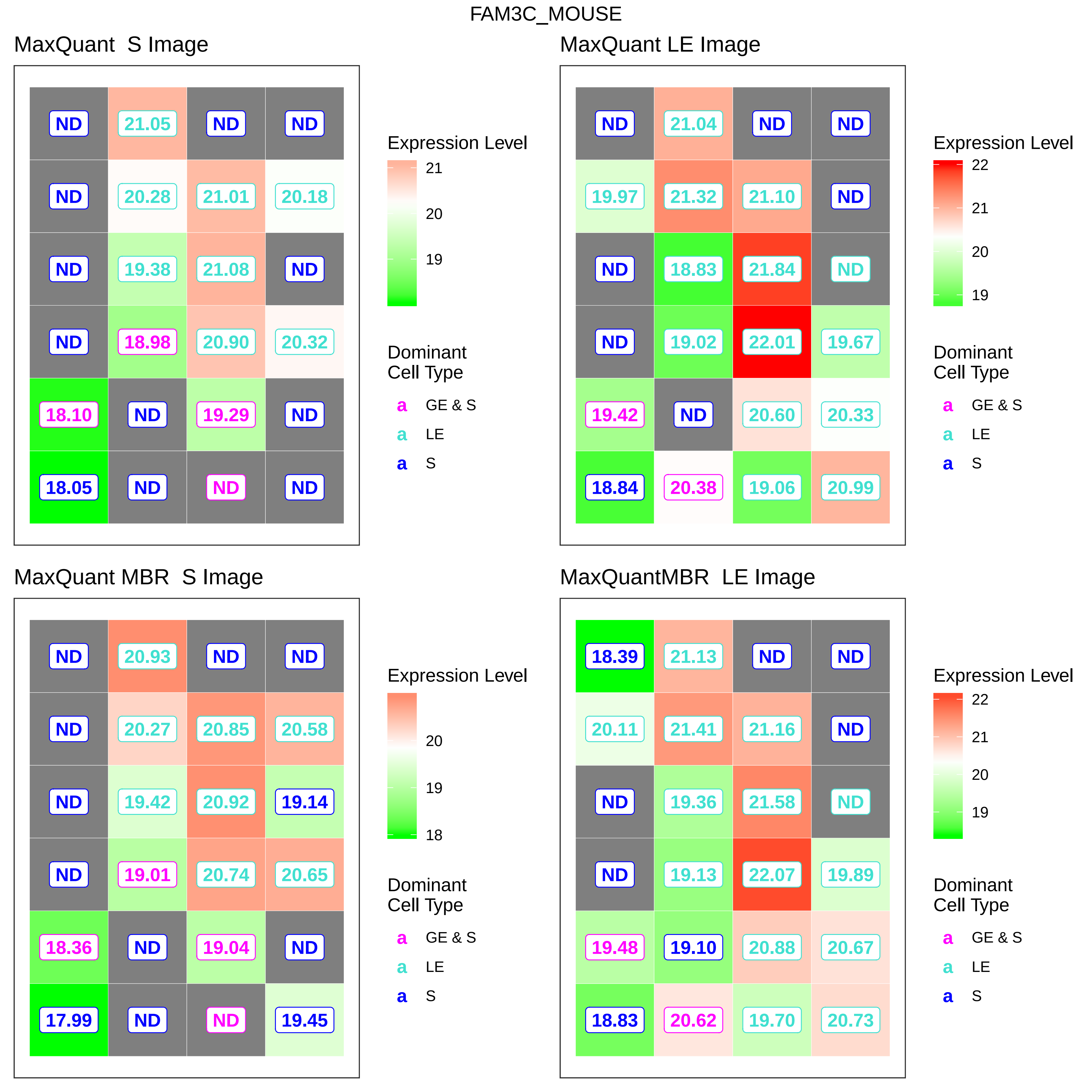
FAM3C_MOUSE
MaxQuant S Image
ND 21.05 ND ND
ND 20.28 21.01 20.18
ND 19.38 21.08 ND
ND 18.98 20.90 20.32
18.10 ND 19.29 ND
18.05 ND ND ND
Expression Level
21
20
19
Dominant Cell Type
a GE & S
a LE
a S
MaxQuant LE Image
ND 21.04 ND ND
19.97 21.32 21.10 ND
ND 18.83 21.84 ND
ND 19.02 22.01 19.67
19.42 ND 20.60 20.33
18.84 20.38 19.06 20.99
Expression Level
22
21
20
19
Dominant Cell Type
a GE & S
a LE
a S
MaxQuant MBR S Image
ND 20.93 ND ND
ND 20.27 20.85 20.58
ND 19.42 20.92 19.14
ND 19.01 20.74 20.65
18.36 ND 19.04 ND
17.99 ND ND 19.45
Expression Level
20
19
18
Dominant Cell Type
a GE & S
a LE
a S
MaxQuantMBR LE Image
18.39 21.13 ND ND
20.11 21.41 21.16 ND
ND 19.36 21.58 ND
ND 19.13 22.07 19.89
19.48 19.10 20.88 20.67
18.83 20.62 19.70 20.73
Expression Level
22
21
20
19
Dominant Cell Type
a GE & S
a LE
a S

FACR1_MOUSE

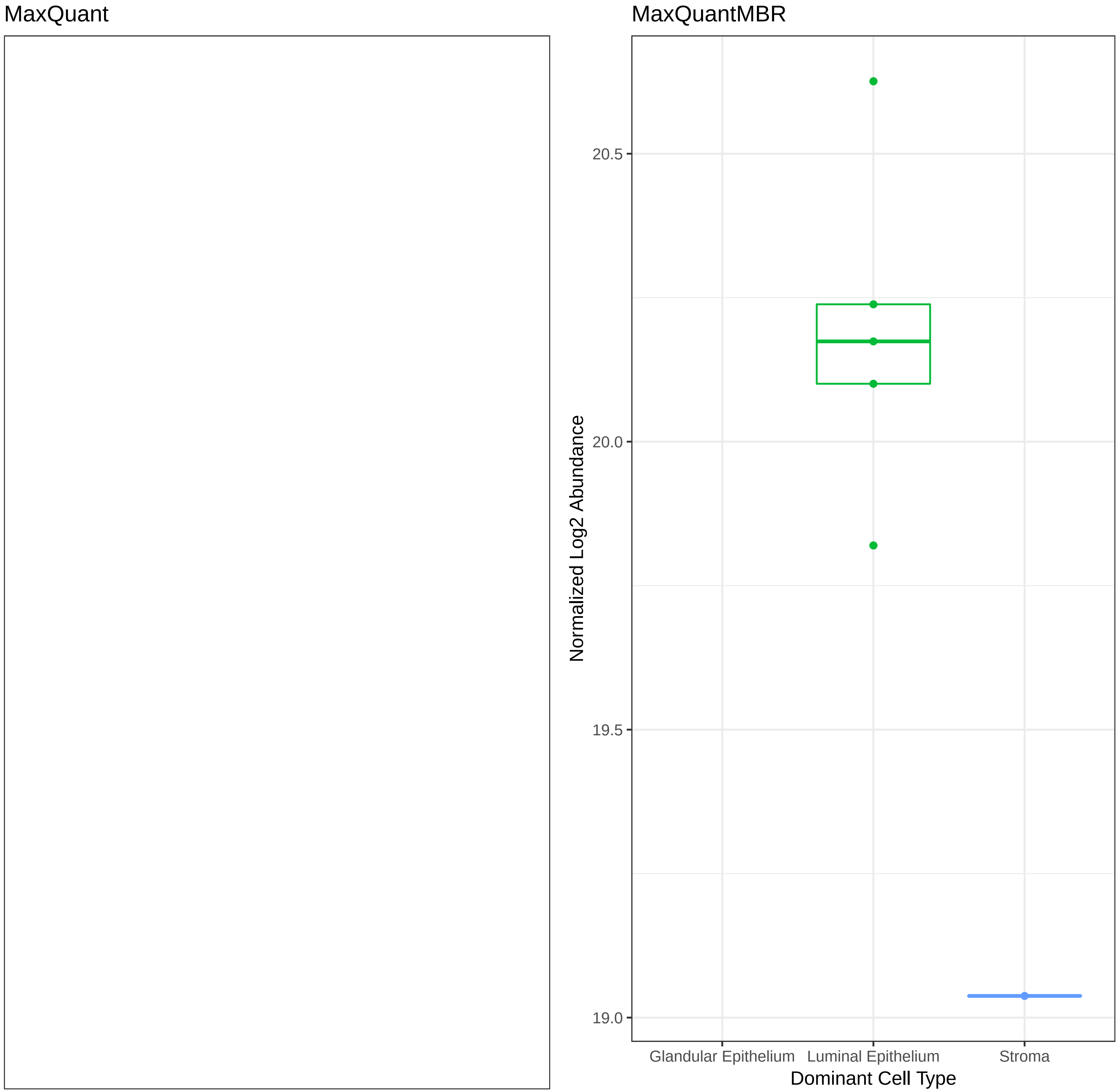
MaxQuant
MaxQuantMBR
Normalized Log2 Abundance
20.5
20.0
19.5
19.0
Glandular Epithelium
Luminal Epithelium
Stroma
Dominant Cell Type

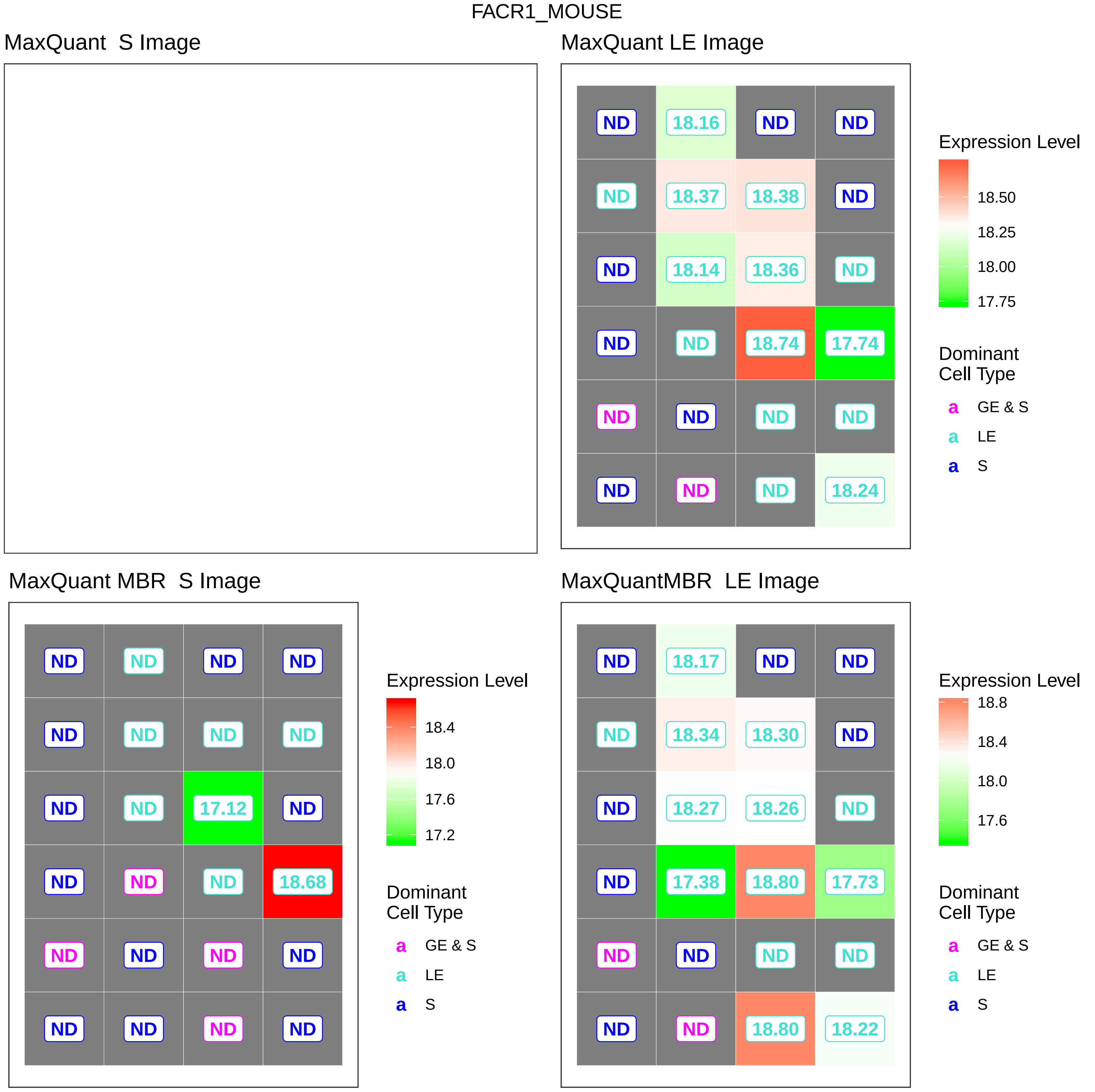
FACR1_MOUSE
MaxQuant S Image
MaxQuant LE Image
ND
18.16
ND
ND
ND
18.37
18.38
ND
ND
18.14
18.36
ND
ND
ND
18.74
17.74
ND
ND
ND
ND
ND
ND
ND
18.24
Expression Level
18.50
18.25
18.00
17.75
Dominant Cell Type
a GE & S
a LE
a S
MaxQuant MBR S Image
ND
ND
ND
ND
ND
ND
ND
ND
ND
ND
17.12
ND
ND
ND
ND
18.68
ND
ND
ND
ND
ND
ND
ND
ND
Expression Level
18.4
18.0
17.6
17.2
Dominant Cell Type
a GE & S
a LE
a S
MaxQuantMBR LE Image
ND
18.17
ND
ND
ND
18.34
18.30
ND
ND
18.27
18.26
ND
ND
17.38
18.80
17.73
ND
ND
ND
ND
ND
ND
18.80
18.22
Expression Level
18.8
18.4
18.0
17.6
Dominant Cell Type
a GE & S
a LE
a S

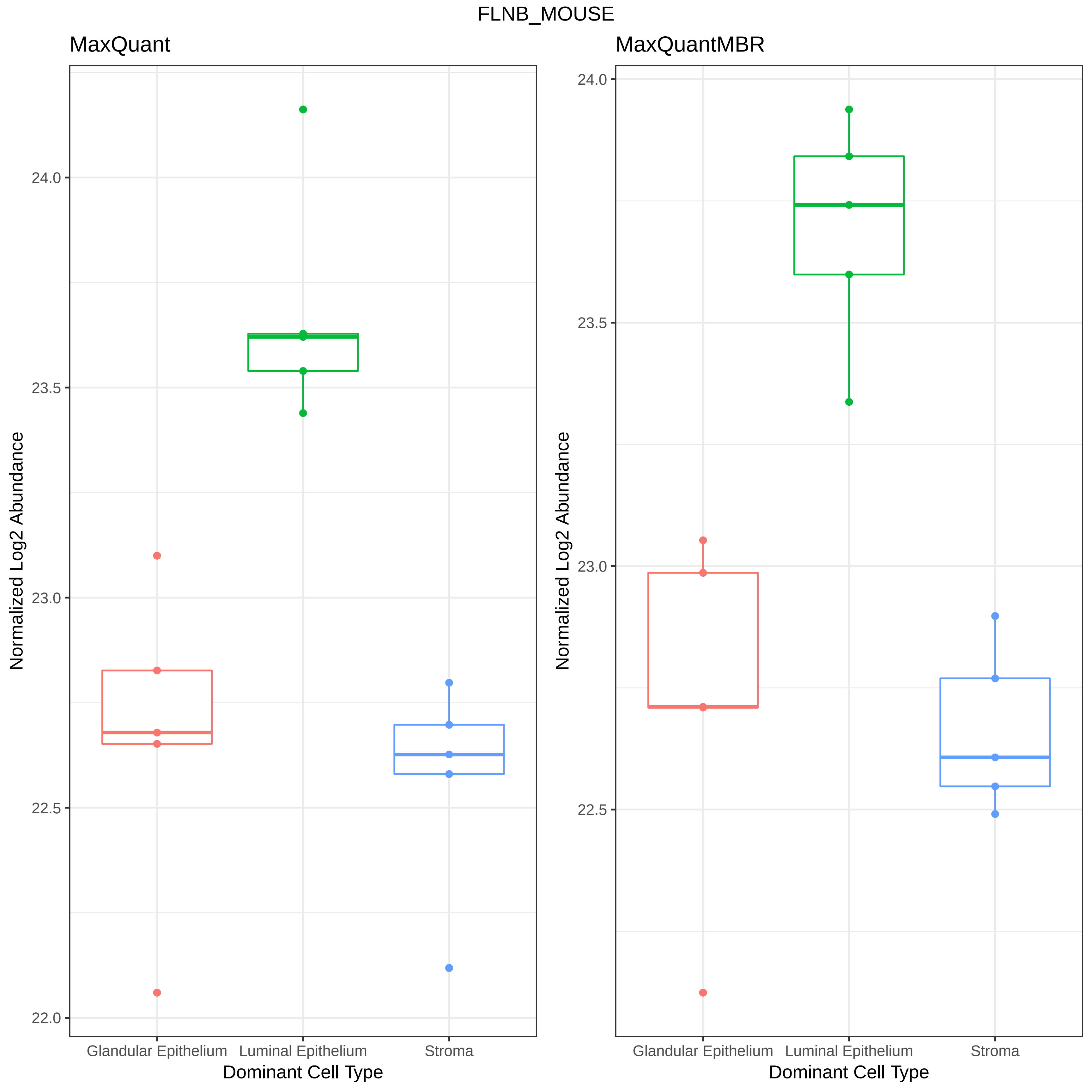
FLNB_MOUSE
MaxQuant
MaxQuantMBR
Normalized Log2 Abundance
24.0
23.5
23.0
22.5
22.0
Glandular Epithelium
Luminal Epithelium
Stroma
Dominant Cell Type

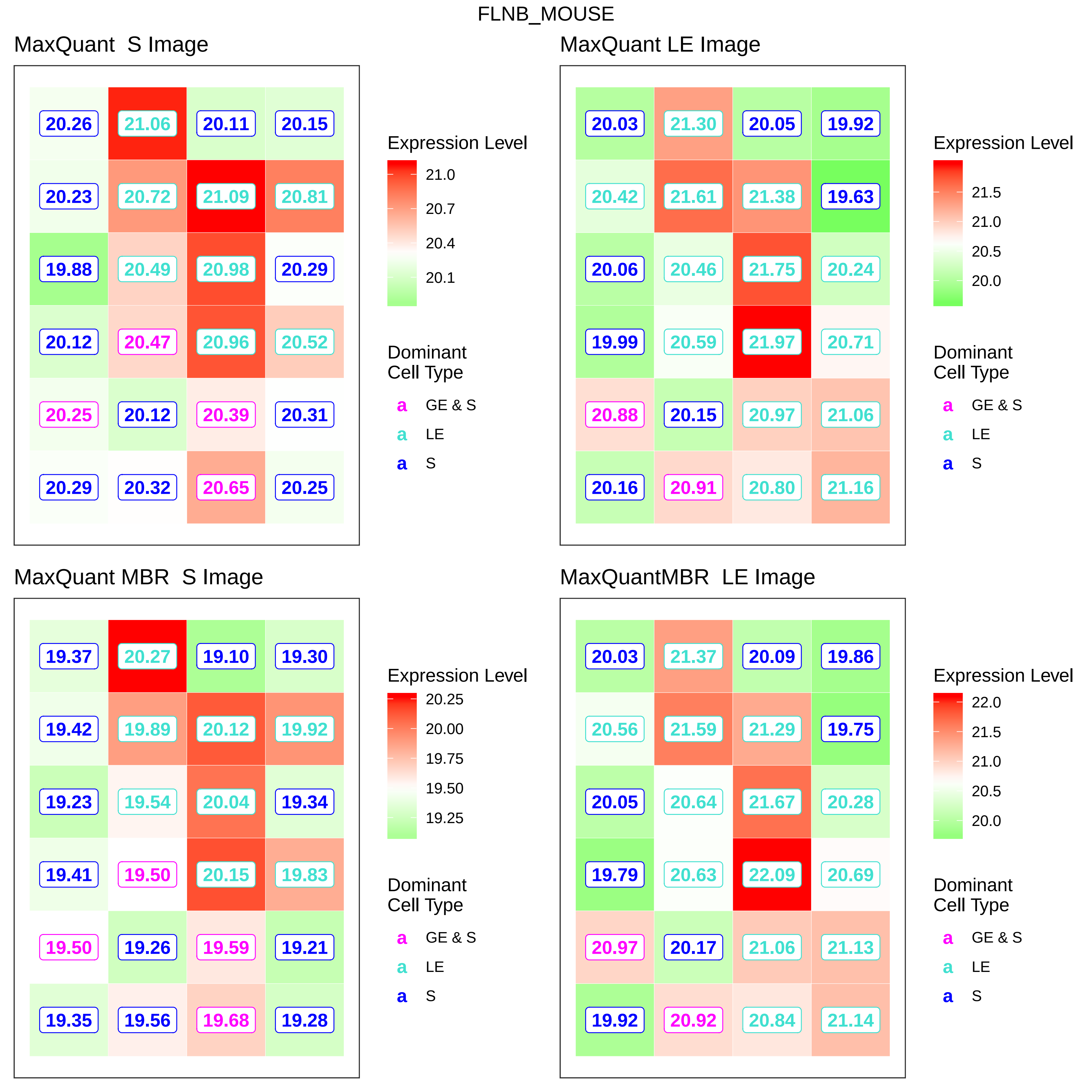
FLNB_MOUSE
MaxQuant S Image
20.26 21.06 20.11 20.15
20.23 20.72 21.09 20.81
19.88 20.49 20.98 20.29
20.12 20.47 20.96 20.52
20.25 20.12 20.39 20.31
20.29 20.32 20.65 20.25
Expression Level
21.0
20.7
20.4
20.1
Dominant Cell Type
a GE & S
a LE
a S
MaxQuant LE Image
20.03 21.30 20.05 19.92
20.42 21.61 21.38 19.63
20.06 20.46 21.75 20.24
19.99 20.59 21.97 20.71
20.88 20.15 20.97 21.06
20.16 20.91 20.80 21.16
Expression Level
21.5
21.0
20.5
20.0
Dominant Cell Type
a GE & S
a LE
a S
MaxQuant MBR S Image
19.37 20.27 19.10 19.30
19.42 19.89 20.12 19.92
19.23 19.54 20.04 19.34
19.41 19.50 20.15 19.83
19.50 19.26 19.59 19.21
19.35 19.56 19.68 19.28
Expression Level
20.25
20.00
19.75
19.50
19.25
Dominant Cell Type
a GE & S
a LE
a S
MaxQuantMBR LE Image
20.03 21.37 20.09 19.86
20.56 21.59 21.29 19.75
20.05 20.64 21.67 20.28
19.79 20.63 22.09 20.69
20.97 20.17 21.06 21.13
19.92 20.92 20.84 21.14
Expression Level
22.0
21.5
21.0
20.5
20.0
Dominant Cell Type
a GE & S
a LE
a S

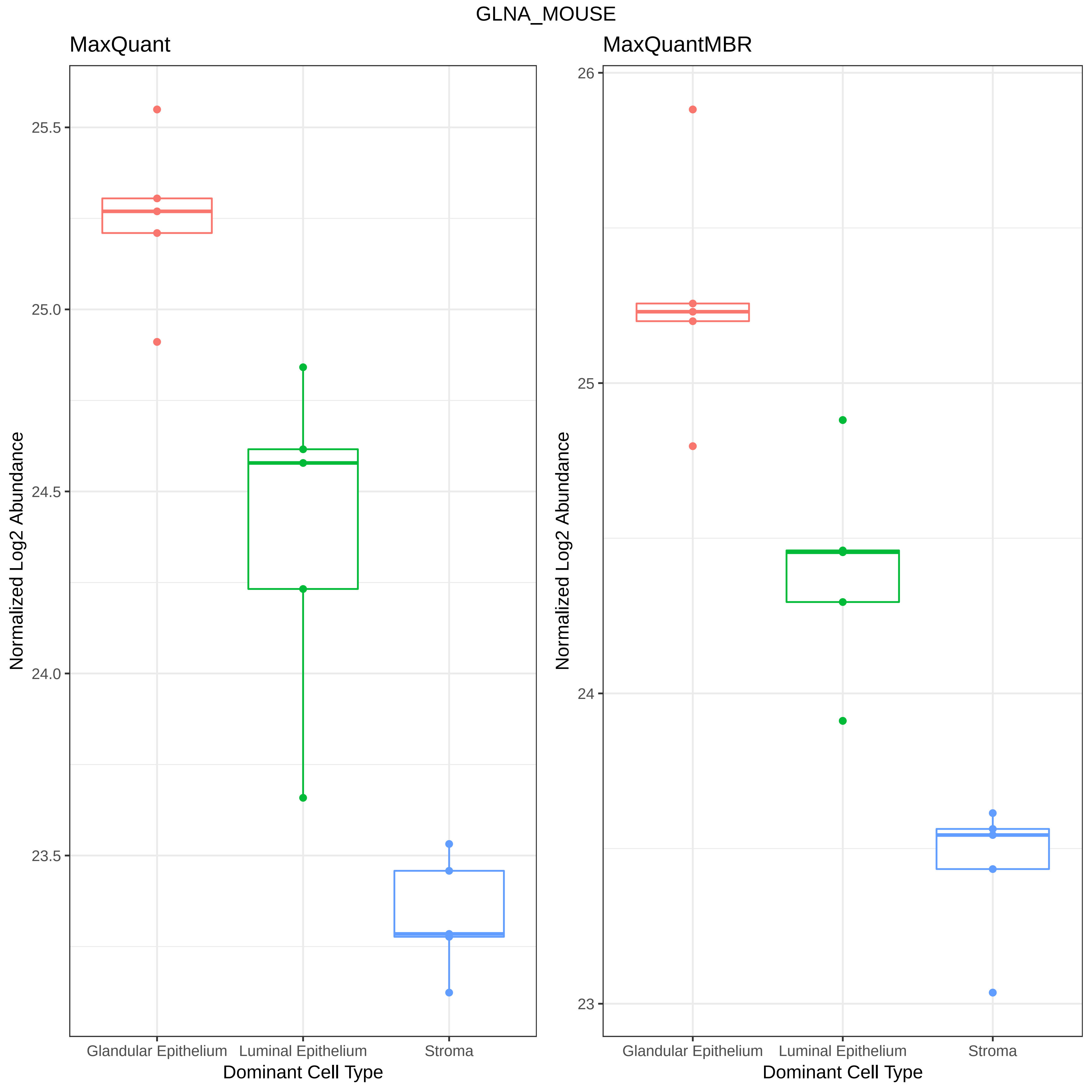

GLNA_MOUSE
MaxQuant
MaxQuantMBR
Normalized Log2 Abundance
25.5
25.0
24.5
24.0
23.5
26
25
24
23
Glandular Epithelium
Luminal Epithelium
Stroma
Dominant Cell Type

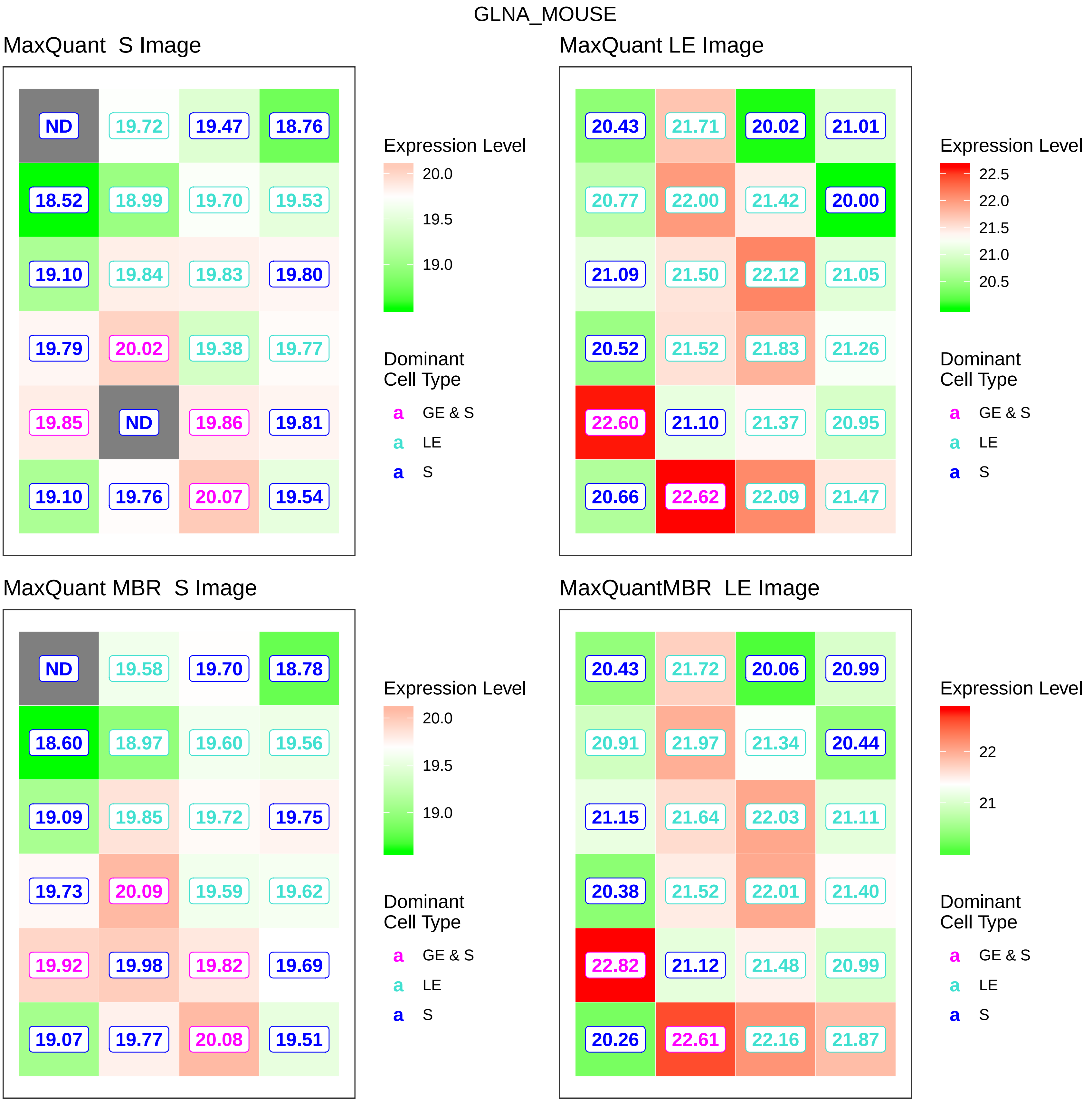
GLNA_MOUSE
MaxQuant S Image
ND 19.72 19.47 18.76
18.52 18.99 19.70 19.53
19.10 19.84 19.83 19.80
19.79 20.02 19.38 19.77
19.85 ND 19.86 19.81
19.10 19.76 20.07 19.54
Expression Level
20.0
19.5
19.0
Dominant Cell Type
a GE & S
a LE
a S
MaxQuant LE Image
20.43 21.71 20.02 21.01
20.77 22.00 21.42 20.00
21.09 21.50 22.12 21.05
20.52 21.52 21.83 21.26
22.60 21.10 21.37 20.95
20.66 22.62 22.09 21.47
Expression Level
22.5
22.0
21.5
21.0
20.5
Dominant Cell Type
a GE & S
a LE
a S
MaxQuant MBR S Image
ND 19.58 19.70 18.78
18.60 18.97 19.60 19.56
19.09 19.85 19.72 19.75
19.73 20.09 19.59 19.62
19.92 19.98 19.82 19.69
19.07 19.77 20.08 19.51
Expression Level
20.0
19.5
19.0
Dominant Cell Type
a GE & S
a LE
a S
MaxQuantMBR LE Image
20.43 21.72 20.06 20.99
20.91 21.97 21.34 20.44
21.15 21.64 22.03 21.11
20.38 21.52 22.01 21.40
22.82 21.12 21.48 20.99
20.26 22.61 22.16 21.87
Expression Level
22
21
Dominant Cell Type
a GE & S
a LE
a S

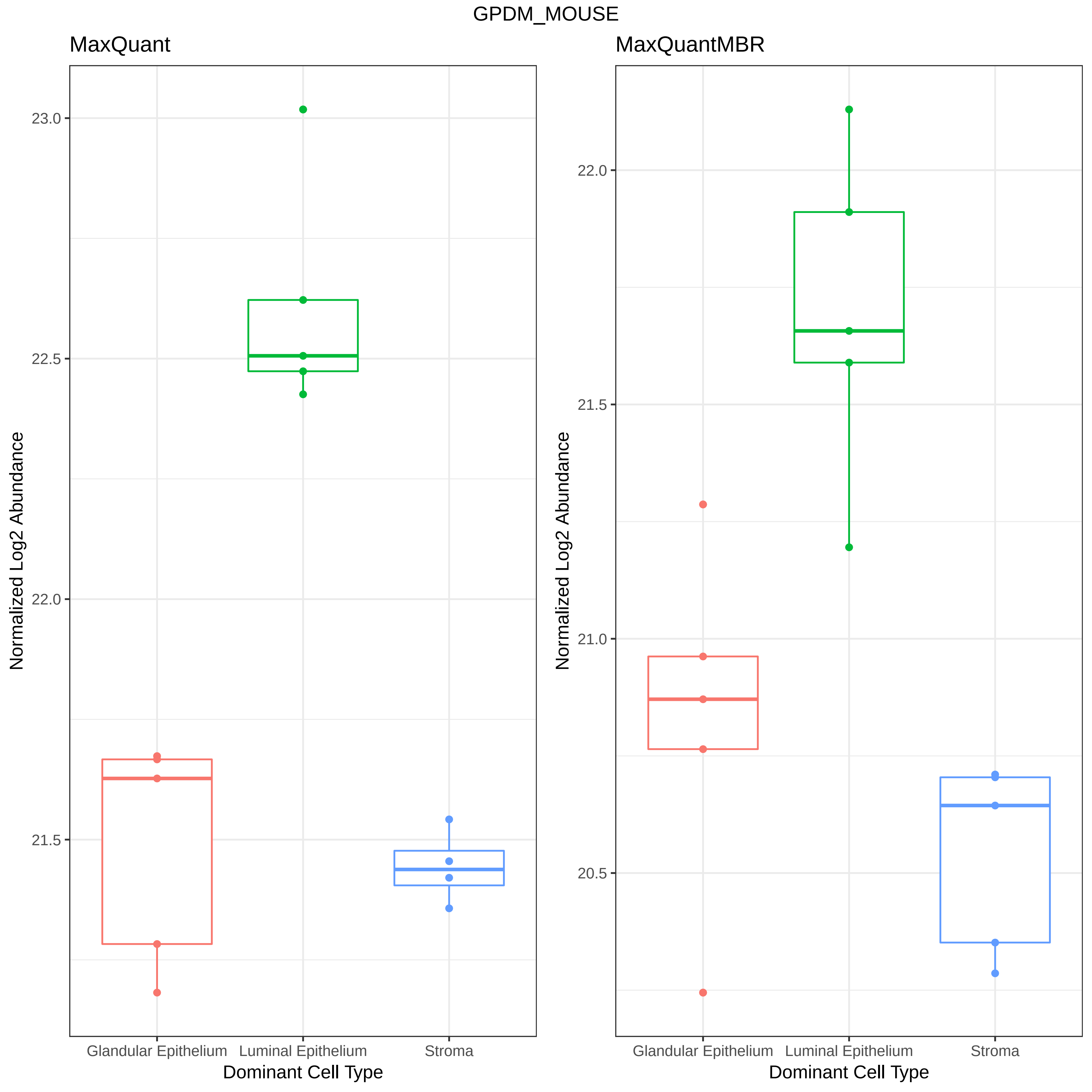

GPDM_MOUSE
MaxQuant
MaxQuantMBR
Normalized Log2 Abundance
23.0
22.5
22.0
21.5
21.0
20.5
Glandular Epithelium
Luminal Epithelium
Stroma
Dominant Cell Type

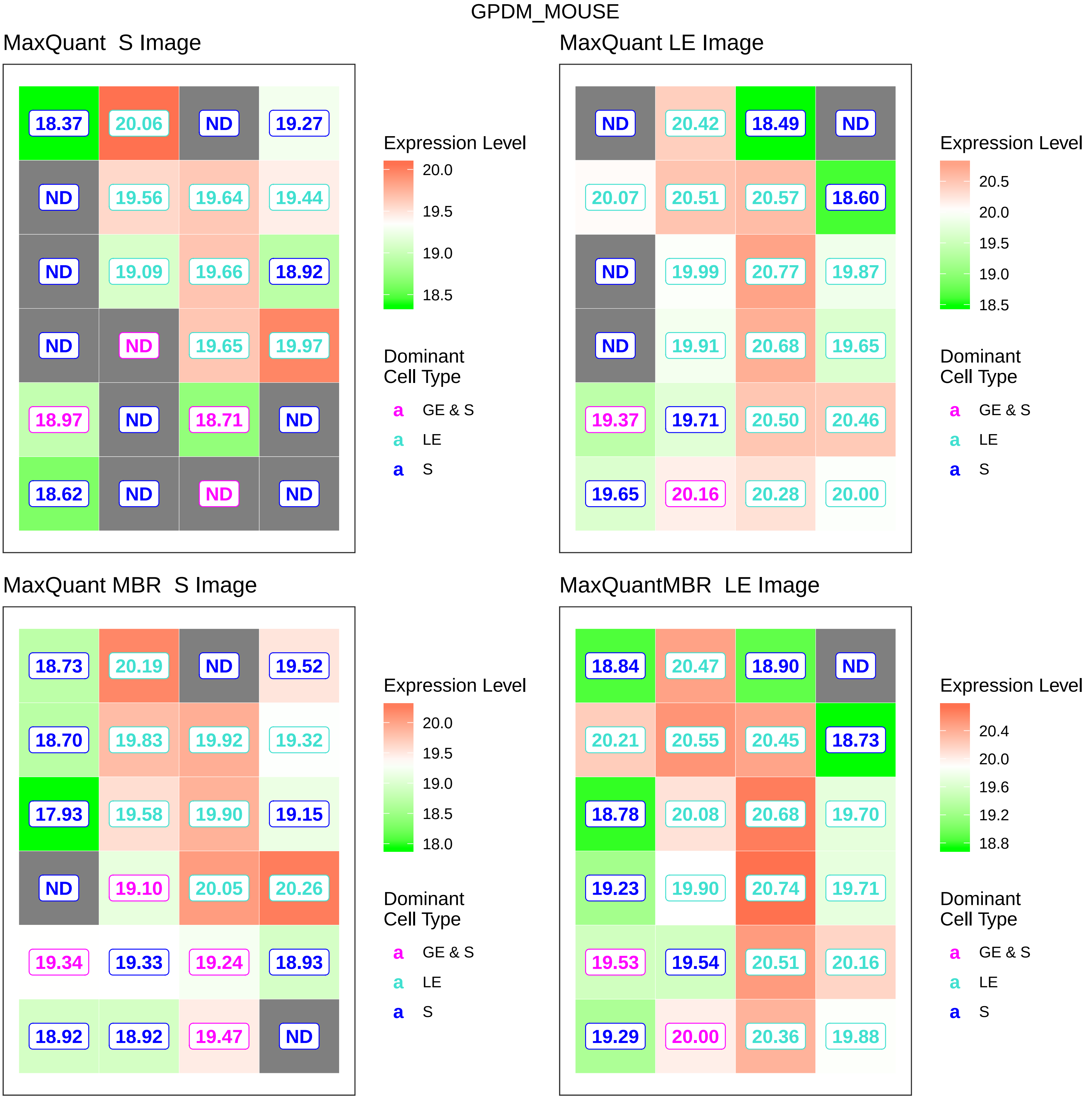
GPDM_MOUSE
MaxQuant S Image
18.37 20.06 ND 19.27
ND 19.56 19.64 19.44
ND 19.09 19.66 18.92
ND ND 19.65 19.97
18.97 ND 18.71 ND
18.62 ND ND ND
Expression Level
20.0
19.5
19.0
18.5
Dominant Cell Type
a GE & S
a LE
a S
MaxQuant LE Image
ND 20.42 18.49 ND
20.07 20.51 20.57 18.60
ND 19.99 20.77 19.87
ND 19.91 20.68 19.65
19.37 19.71 20.50 20.46
19.65 20.16 20.28 20.00
Expression Level
20.5
20.0
19.5
19.0
18.5
Dominant Cell Type
a GE & S
a LE
a S
MaxQuant MBR S Image
18.73 20.19 ND 19.52
18.70 19.83 19.92 19.32
17.93 19.58 19.90 19.15
ND 19.10 20.05 20.26
19.34 19.33 19.24 18.93
18.92 18.92 19.47 ND
Expression Level
20.0
19.5
19.0
18.5
18.0
Dominant Cell Type
a GE & S
a LE
a S
MaxQuantMBR LE Image
18.84 20.47 18.90 ND
20.21 20.55 20.45 18.73
18.78 20.08 20.68 19.70
19.23 19.90 20.74 19.71
19.53 19.54 20.51 20.16
19.29 20.00 20.36 19.88
Expression Level
20.4
20.0
19.6
19.2
18.8
Dominant Cell Type
a GE & S
a LE
a S

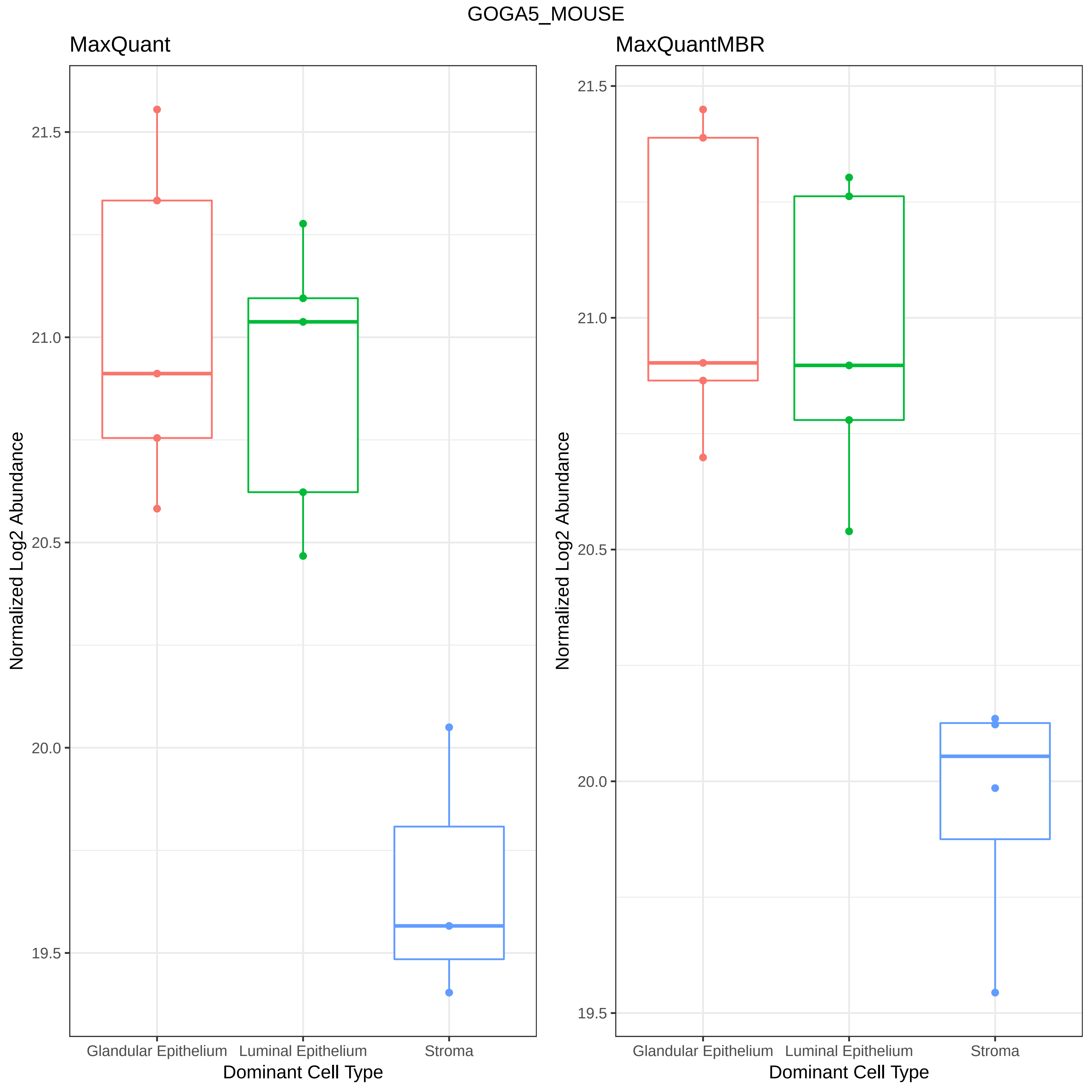
GOGA5_MOUSE
MaxQuant
MaxQuantMBR
Normalized Log2 Abundance
21.5
21.0
20.5
20.0
19.5
Glandular Epithelium
Luminal Epithelium
Stroma
Dominant Cell Type

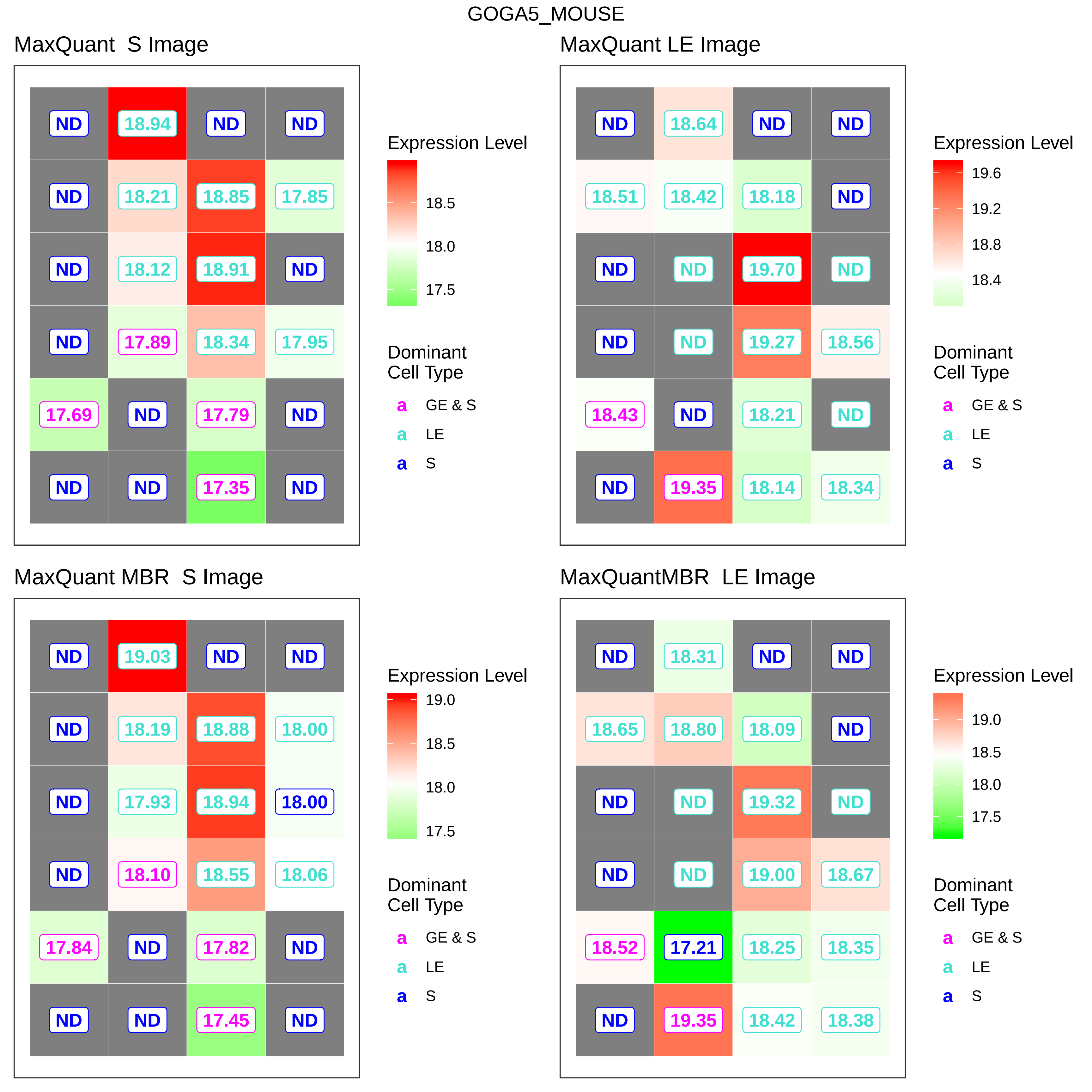
GOGA5_MOUSE
MaxQuant S Image
ND 18.94 ND ND
ND 18.21 18.85 17.85
ND 18.12 18.91 ND
ND 17.89 18.34 17.95
17.69 ND 17.79 ND
ND ND 17.35 ND
Expression Level
18.5
18.0
17.5
Dominant Cell Type
a GE & S
a LE
a S
MaxQuant LE Image
ND 18.64 ND ND
18.51 18.42 18.18 ND
ND ND 19.70 ND
ND ND 19.27 18.56
18.43 ND 18.21 ND
ND 19.35 18.14 18.34
Expression Level
19.6
19.2
18.8
18.4
Dominant Cell Type
a GE & S
a LE
a S
MaxQuant MBR S Image
ND 19.03 ND ND
ND 18.19 18.88 18.00
ND 17.93 18.94 18.00
ND 18.10 18.55 18.06
17.84 ND 17.82 ND
ND ND 17.45 ND
Expression Level
19.0
18.5
18.0
17.5
Dominant Cell Type
a GE & S
a LE
a S
MaxQuantMBR LE Image
ND 18.31 ND ND
18.65 18.80 18.09 ND
ND ND 19.32 ND
ND ND 19.00 18.67
18.52 17.21 18.25 18.35
ND 19.35 18.42 18.38
Expression Level
19.0
18.5
18.0
17.5
Dominant Cell Type
a GE & S
a LE
a S

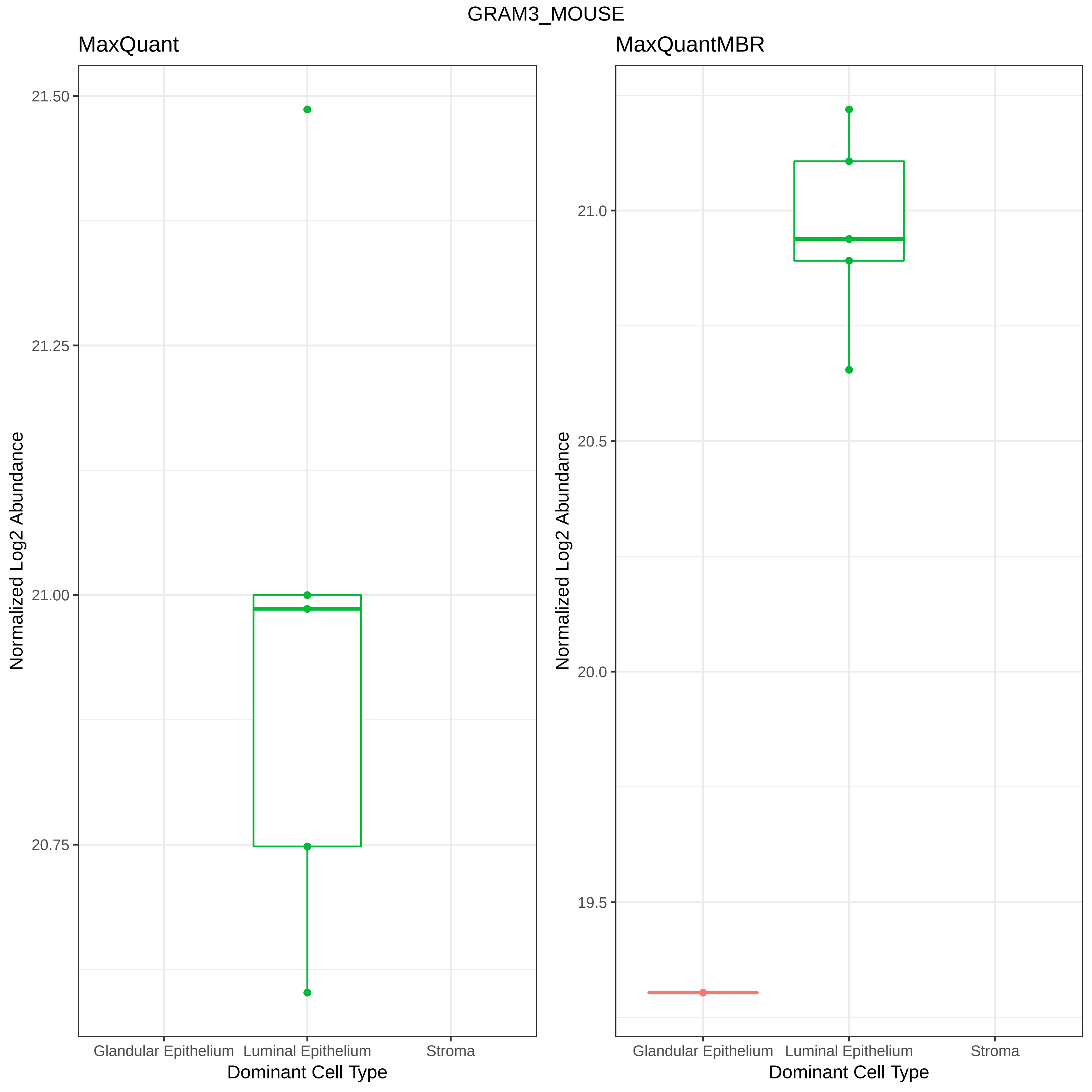
GRAM3_MOUSE
MaxQuant
MaxQuantMBR
Normalized Log2 Abundance
21.50
21.25
21.00
20.75
21.0
20.5
20.0
19.5
Glandular Epithelium
Luminal Epithelium
Stroma
Dominant Cell Type

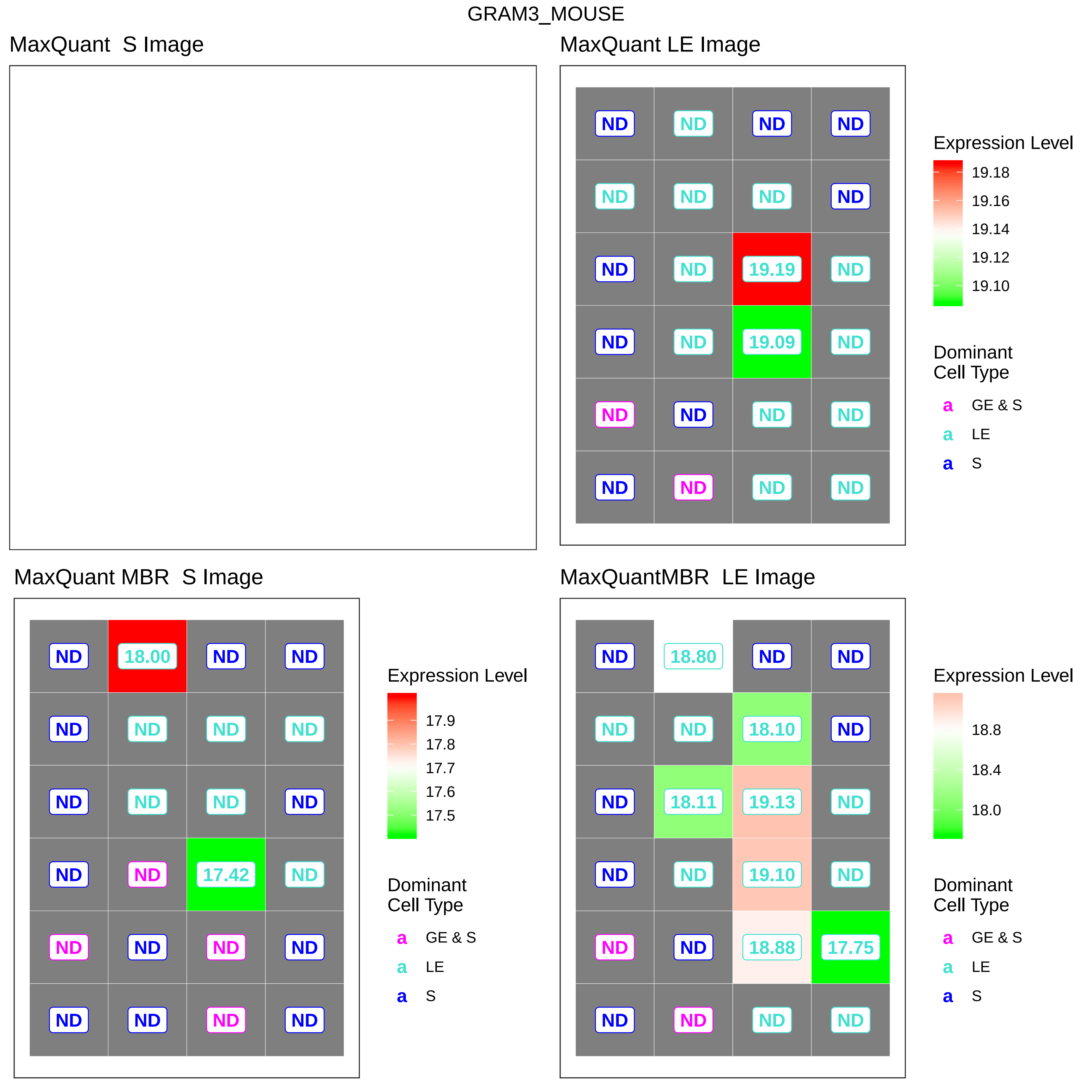
GRAM3_MOUSE
MaxQuant S Image
MaxQuant LE Image
ND
19.19
19.09
Expression Level
19.18
19.16
19.14
19.12
19.10
Dominant Cell Type
a GE & S
a LE
a S
MaxQuant MBR S Image
18.00
17.42
Expression Level
17.9
17.8
17.7
17.6
17.5
Dominant Cell Type
a GE & S
a LE
a S
MaxQuantMBR LE Image
18.80
18.10
18.11
19.13
19.10
18.88
17.75
Expression Level
18.8
18.4
18.0
Dominant Cell Type
a GE & S
a LE
a S

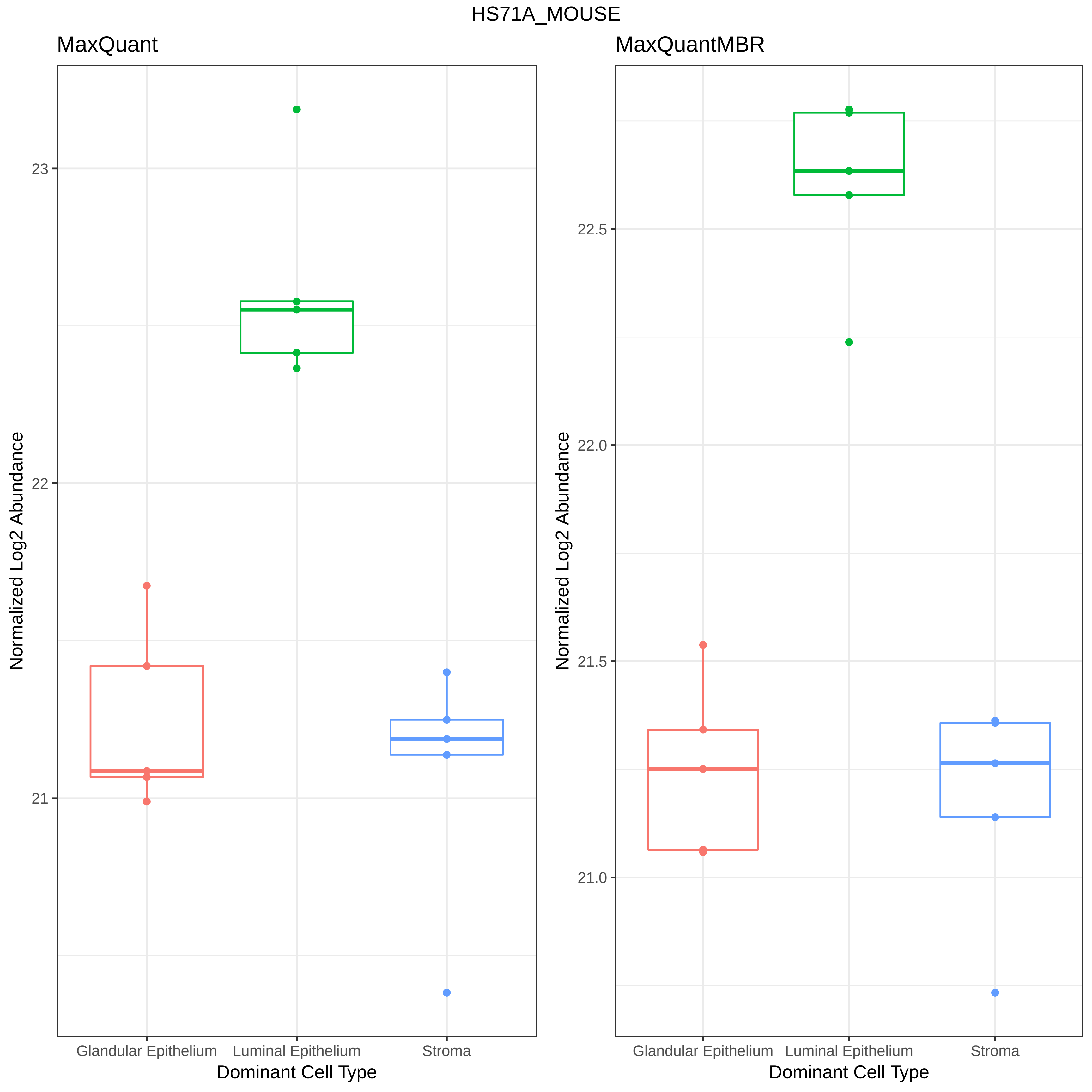

HS71A_MOUSE
MaxQuant
MaxQuantMBR
Normalized Log2 Abundance
23
22
21
22.5
22.0
21.5
21.0
Glandular Epithelium
Luminal Epithelium
Stroma
Dominant Cell Type

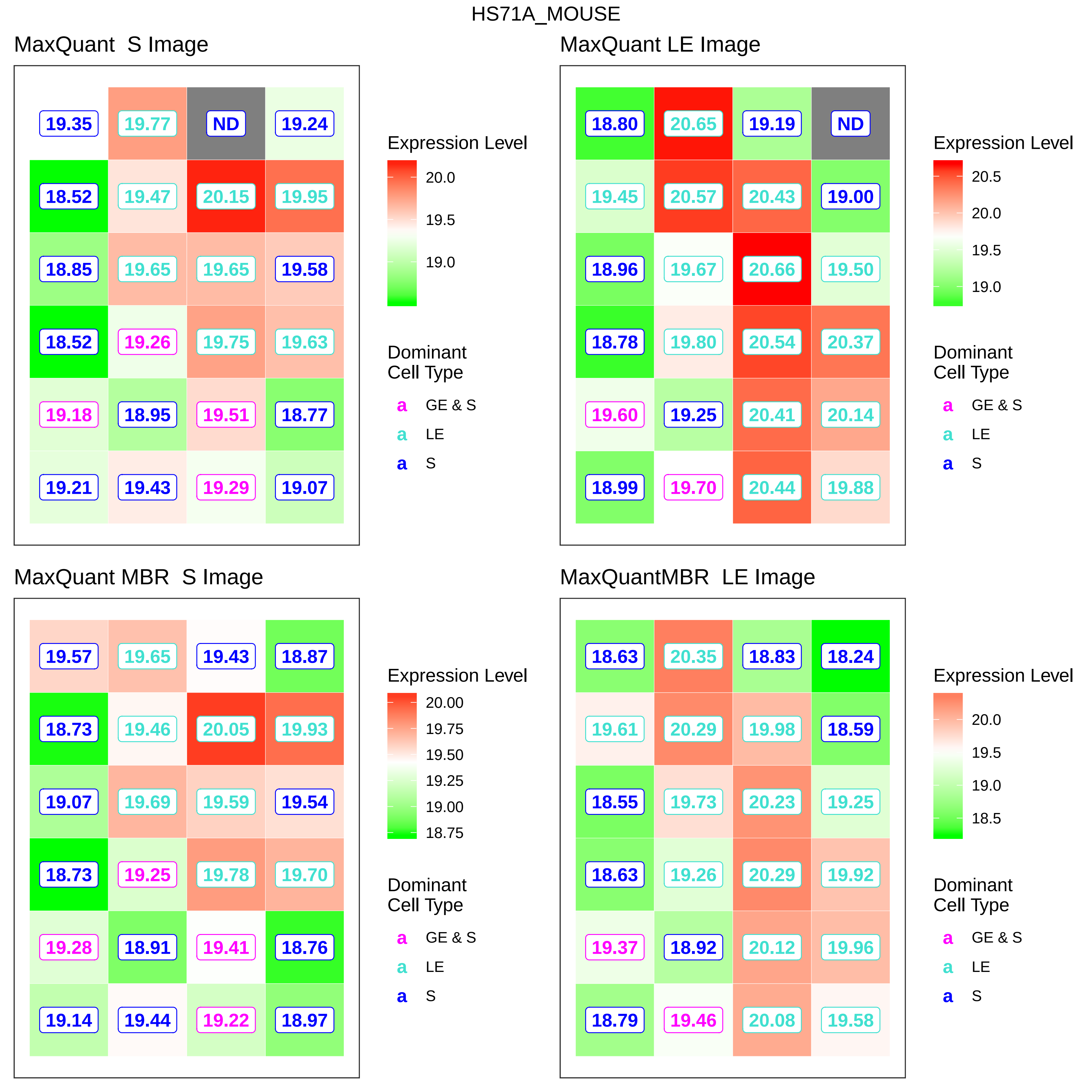

HS71A_MOUSE
MaxQuant S Image
19.35 19.77 ND 19.24
18.52 19.47 20.15 19.95
18.85 19.65 19.65 19.58
18.52 19.26 19.75 19.63
19.18 18.95 19.51 18.77
19.21 19.43 19.29 19.07
Expression Level
20.0
19.5
19.0
Dominant Cell Type
a GE & S
a LE
a S
MaxQuant LE Image
18.80 20.65 19.19 ND
19.45 20.57 20.43 19.00
18.96 19.67 20.66 19.50
18.78 19.80 20.54 20.37
19.60 19.25 20.41 20.14
18.99 19.70 20.44 19.88
Expression Level
20.5
20.0
19.5
19.0
Dominant Cell Type
a GE & S
a LE
a S
MaxQuant MBR S Image
19.57 19.65 19.43 18.87
18.73 19.46 20.05 19.93
19.07 19.69 19.59 19.54
18.73 19.25 19.78 19.70
19.28 18.91 19.41 18.76
19.14 19.44 19.22 18.97
Expression Level
20.00
19.75
19.50
19.25
19.00
18.75
Dominant Cell Type
a GE & S
a LE
a S
MaxQuantMBR LE Image
18.63 20.35 18.83 18.24
19.61 20.29 19.98 18.59
18.55 19.73 20.23 19.25
18.63 19.26 20.29 19.92
19.37 18.92 20.12 19.96
18.79 19.46 20.08 19.58
Expression Level
20.0
19.5
19.0
18.5
Dominant Cell Type
a GE & S
a LE
a S

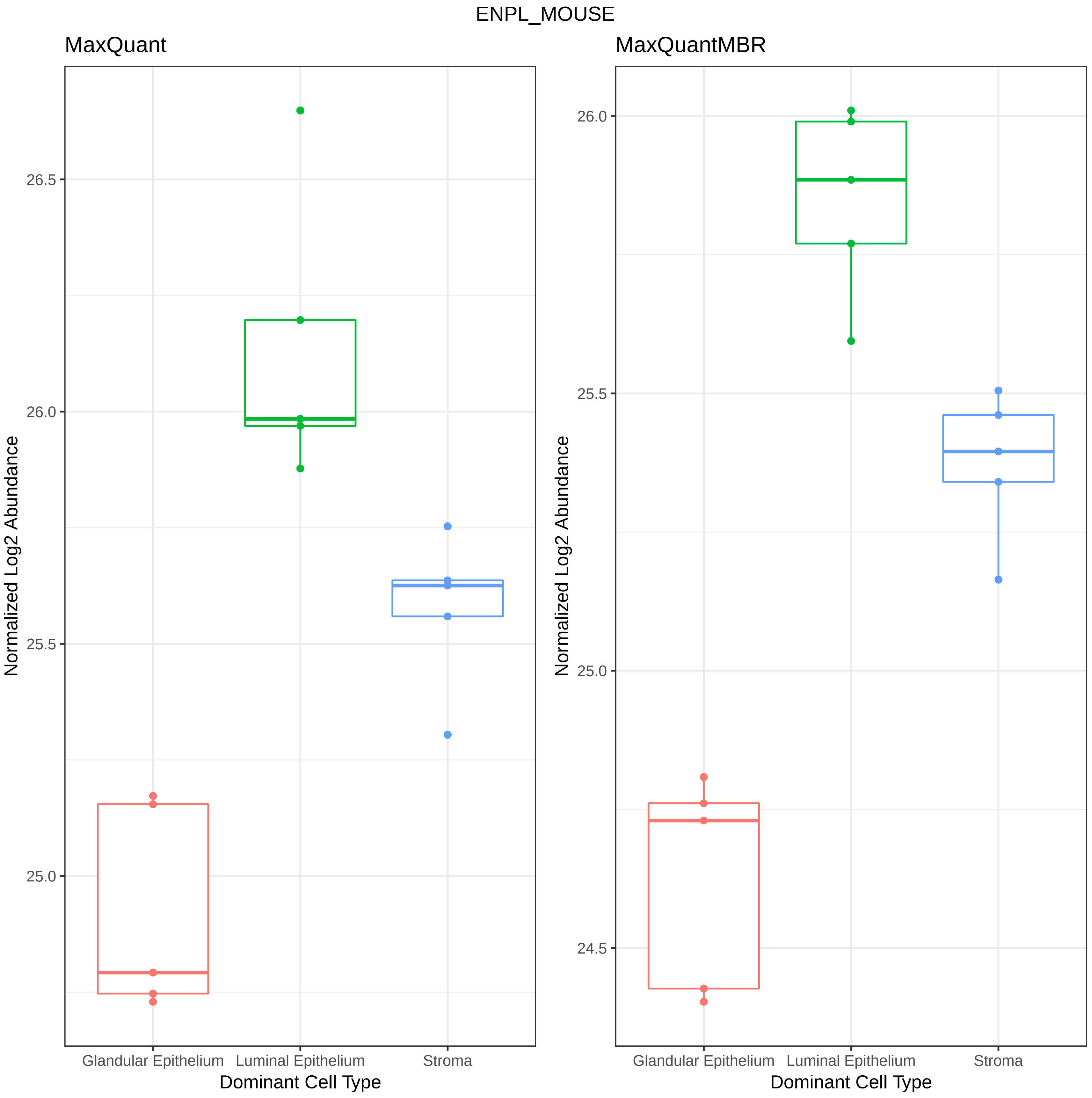
ENPL_MOUSE
MaxQuant
MaxQuantMBR
Normalized Log2 Abundance
26.5
26.0
25.5
25.0
24.5
Glandular Epithelium
Luminal Epithelium
Stroma
Dominant Cell Type

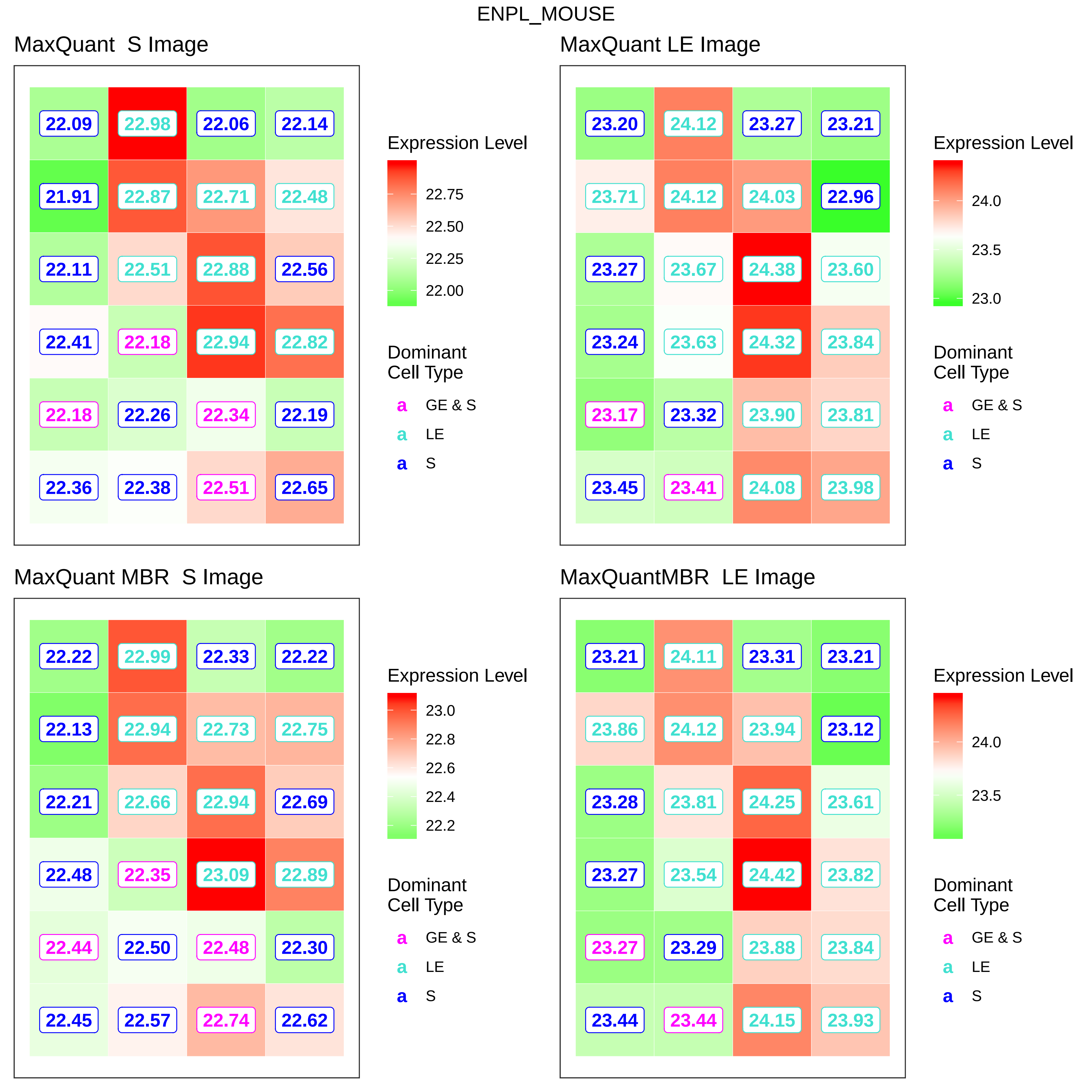
ENPL_MOUSE
MaxQuant S Image
22.09 22.98 22.06 22.14
21.91 22.87 22.71 22.48
22.11 22.51 22.88 22.56
22.41 22.18 22.94 22.82
22.18 22.26 22.34 22.19
22.36 22.38 22.51 22.65
Expression Level
22.75
22.50
22.25
22.00
Dominant Cell Type
a GE & S
a LE
a S
MaxQuant LE Image
23.20 24.12 23.27 23.21
23.71 24.12 24.03 22.96
23.27 23.67 24.38 23.60
23.24 23.63 24.32 23.84
23.17 23.32 23.90 23.81
23.45 23.41 24.08 23.98
Expression Level
24.0
23.5
23.0
Dominant Cell Type
a GE & S
a LE
a S
MaxQuant MBR S Image
22.22 22.99 22.33 22.22
22.13 22.94 22.73 22.75
22.21 22.66 22.94 22.69
22.48 22.35 23.09 22.89
22.44 22.50 22.48 22.30
22.45 22.57 22.74 22.62
Expression Level
23.0
22.8
22.6
22.4
22.2
Dominant Cell Type
a GE & S
a LE
a S
MaxQuantMBR LE Image
23.21 24.11 23.31 23.21
23.86 24.12 23.94 23.12
23.28 23.81 24.25 23.61
23.27 23.54 24.42 23.82
23.27 23.29 23.88 23.84
23.44 23.44 24.15 23.93
Expression Level
24.0
23.5
Dominant Cell Type
a GE & S
a LE
a S

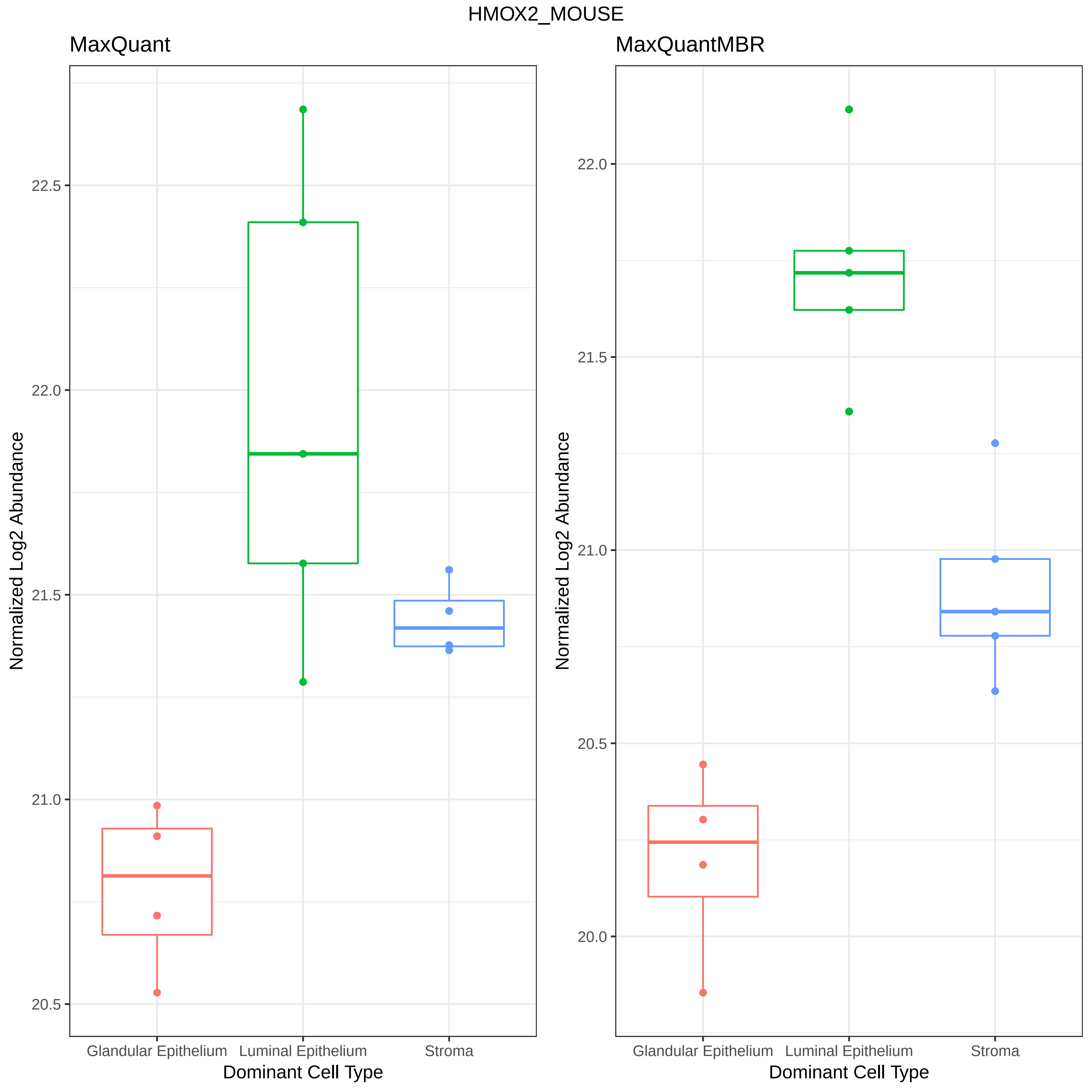

HMOX2_MOUSE
MaxQuant
MaxQuantMBR
Normalized Log2 Abundance
22.5
22.0
21.5
21.0
20.5
20.0
Glandular Epithelium
Luminal Epithelium
Stroma
Dominant Cell Type

HMOX2_MOUSE

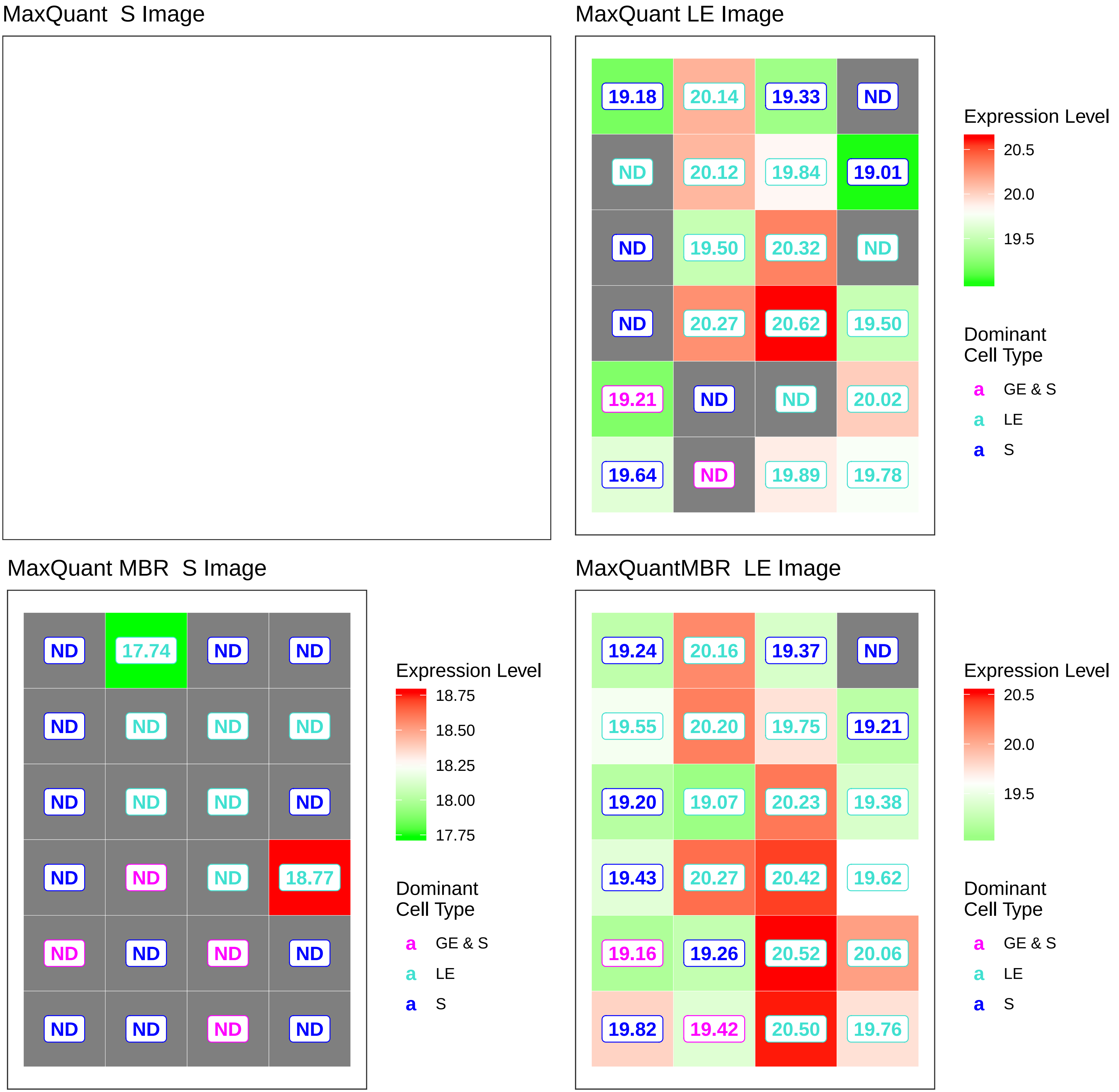

MaxQuant S Image
MaxQuant LE Image
19.18 20.14 19.33 ND
ND 20.12 19.84 19.01
ND 19.50 20.32 ND
ND 20.27 20.62 19.50
19.21 ND ND 20.02
19.64 ND 19.89 19.78
Expression Level
20.5
20.0
19.5
Dominant Cell Type
a GE & S
a LE
a S
MaxQuant MBR S Image
ND 17.74 ND ND
ND ND ND ND
ND ND ND ND
ND ND ND 18.77
ND ND ND ND
ND ND ND ND
Expression Level
18.75
18.50
18.25
18.00
17.75
Dominant Cell Type
a GE & S
a LE
a S
MaxQuantMBR LE Image
19.24 20.16 19.37 ND
19.55 20.20 19.75 19.21
19.20 19.07 20.23 19.38
19.43 20.27 20.42 19.62
19.16 19.26 20.52 20.06
19.82 19.42 20.50 19.76
Expression Level
20.5
20.0
19.5
Dominant Cell Type
a GE & S
a LE
a S

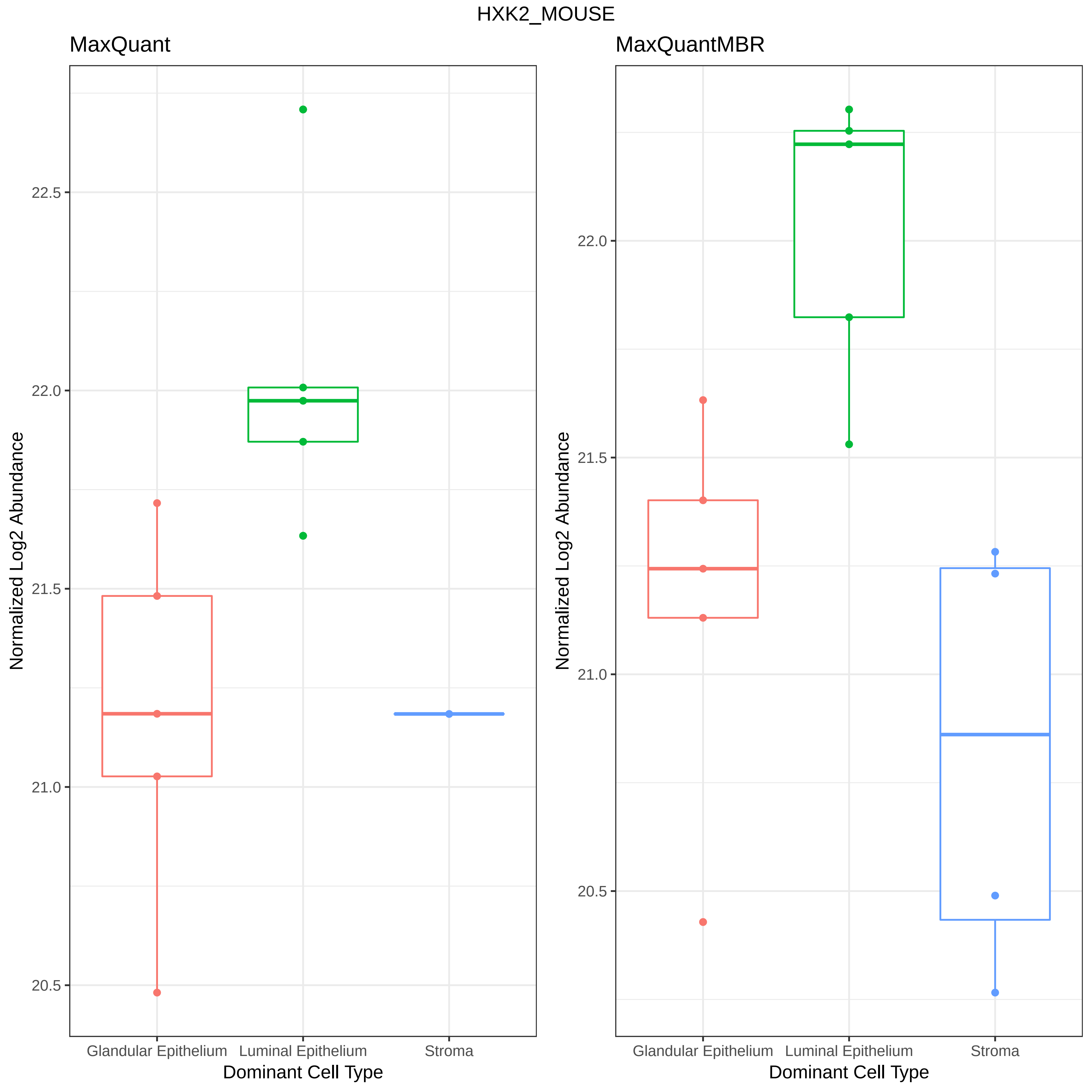

HXK2_MOUSE
MaxQuant
MaxQuantMBR
Normalized Log2 Abundance
22.5
22.0
21.5
21.0
20.5
Glandular Epithelium
Luminal Epithelium
Stroma
Dominant Cell Type

HXK2_MOUSE

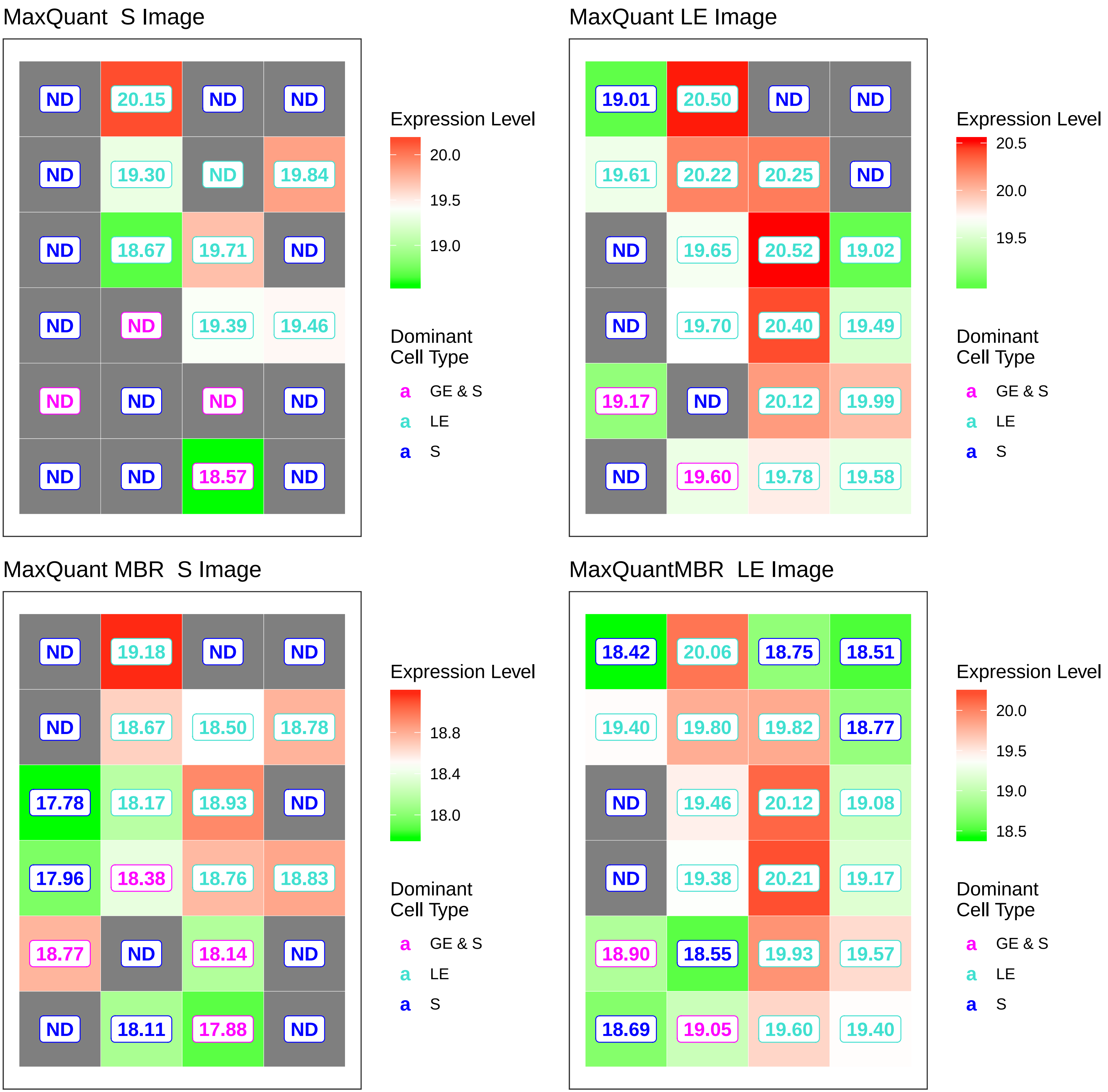
MaxQuant S Image
ND 20.15 ND ND
ND 19.30 ND 19.84
ND 18.67 19.71 ND
ND ND 19.39 19.46
ND ND ND ND
ND ND 18.57 ND
Expression Level
20.0
19.5
19.0
Dominant Cell Type
a GE & S
a LE
a S
MaxQuant LE Image
19.01 20.50 ND ND
19.61 20.22 20.25 ND
ND 19.65 20.52 19.02
ND 19.70 20.40 19.49
19.17 ND 20.12 19.99
ND 19.60 19.78 19.58
Expression Level
20.5
20.0
19.5
Dominant Cell Type
a GE & S
a LE
a S
MaxQuant MBR S Image
ND 19.18 ND ND
ND 18.67 18.50 18.78
17.78 18.17 18.93 ND
17.96 18.38 18.76 18.83
18.77 ND 18.14 ND
ND 18.11 17.88 ND
Expression Level
18.8
18.4
18.0
Dominant Cell Type
a GE & S
a LE
a S
MaxQuantMBR LE Image
18.42 20.06 18.75 18.51
19.40 19.80 19.82 18.77
ND 19.46 20.12 19.08
ND 19.38 20.21 19.17
18.90 18.55 19.93 19.57
18.69 19.05 19.60 19.40
Expression Level
20.0
19.5
19.0
18.5
Dominant Cell Type
a GE & S
a LE
a S

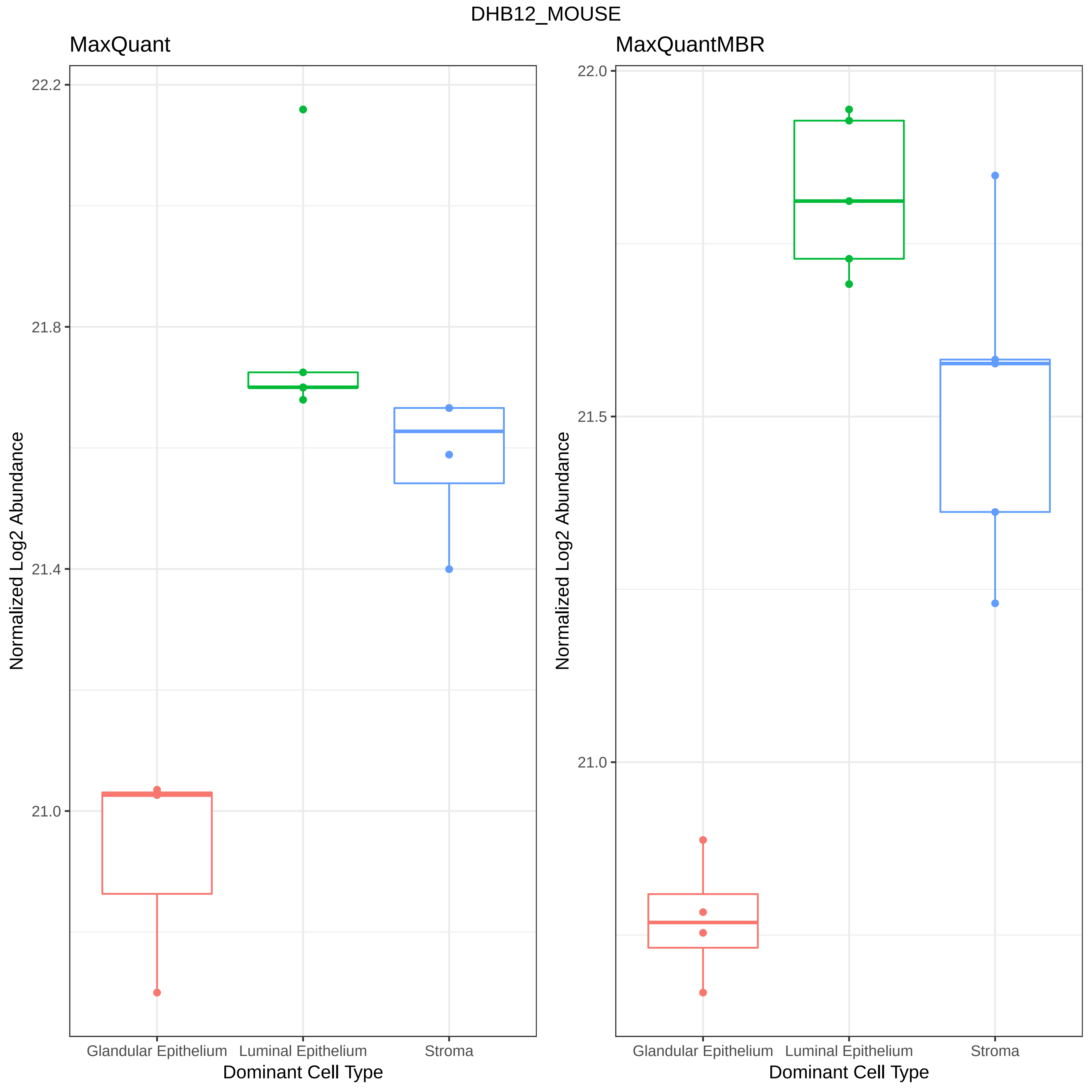

DHB12_MOUSE
MaxQuant
MaxQuantMBR
22.2
21.8
21.4
21.0
22.0
21.5
21.0
Normalized Log2 Abundance
Normalized Log2 Abundance
Glandular Epithelium
Luminal Epithelium
Stroma
Glandular Epithelium
Luminal Epithelium
Stroma
Dominant Cell Type
Dominant Cell Type

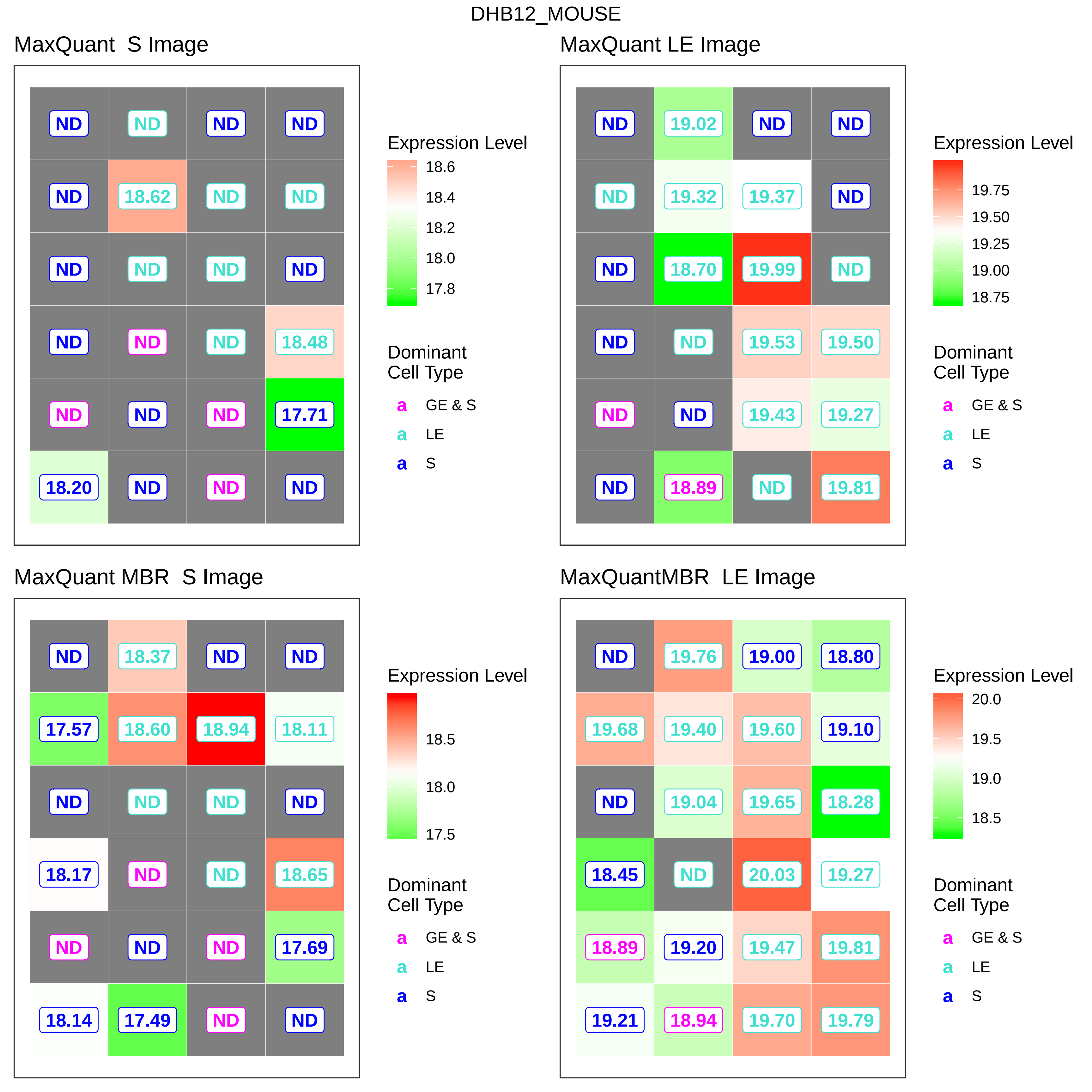
DHB12_MOUSE
MaxQuant S Image
ND ND ND ND
ND 18.62 ND ND
ND ND ND ND
ND ND ND 18.48
ND ND ND 17.71
18.20 ND ND ND
Expression Level
18.6
18.4
18.2
18.0
17.8
Dominant Cell Type
a GE & S
a LE
a S
MaxQuant LE Image
ND 19.02 ND ND
ND 19.32 19.37 ND
ND 18.70 19.99 ND
ND ND 19.53 19.50
ND ND 19.43 19.27
ND 18.89 ND 19.81
Expression Level
19.75
19.50
19.25
19.00
18.75
Dominant Cell Type
a GE & S
a LE
a S
MaxQuant MBR S Image
ND 18.37 ND ND
17.57 18.60 18.94 18.11
ND ND ND ND
18.17 ND ND 18.65
ND ND ND 17.69
18.14 17.49 ND ND
Expression Level
18.5
18.0
17.5
Dominant Cell Type
a GE & S
a LE
a S
MaxQuantMBR LE Image
ND 19.76 19.00 18.80
19.68 19.40 19.60 19.10
ND 19.04 19.65 18.28
18.45 ND 20.03 19.27
18.89 19.20 19.47 19.81
19.21 18.94 19.70 19.79
Expression Level
20.0
19.5
19.0
18.5
Dominant Cell Type
a GE & S
a LE
a S

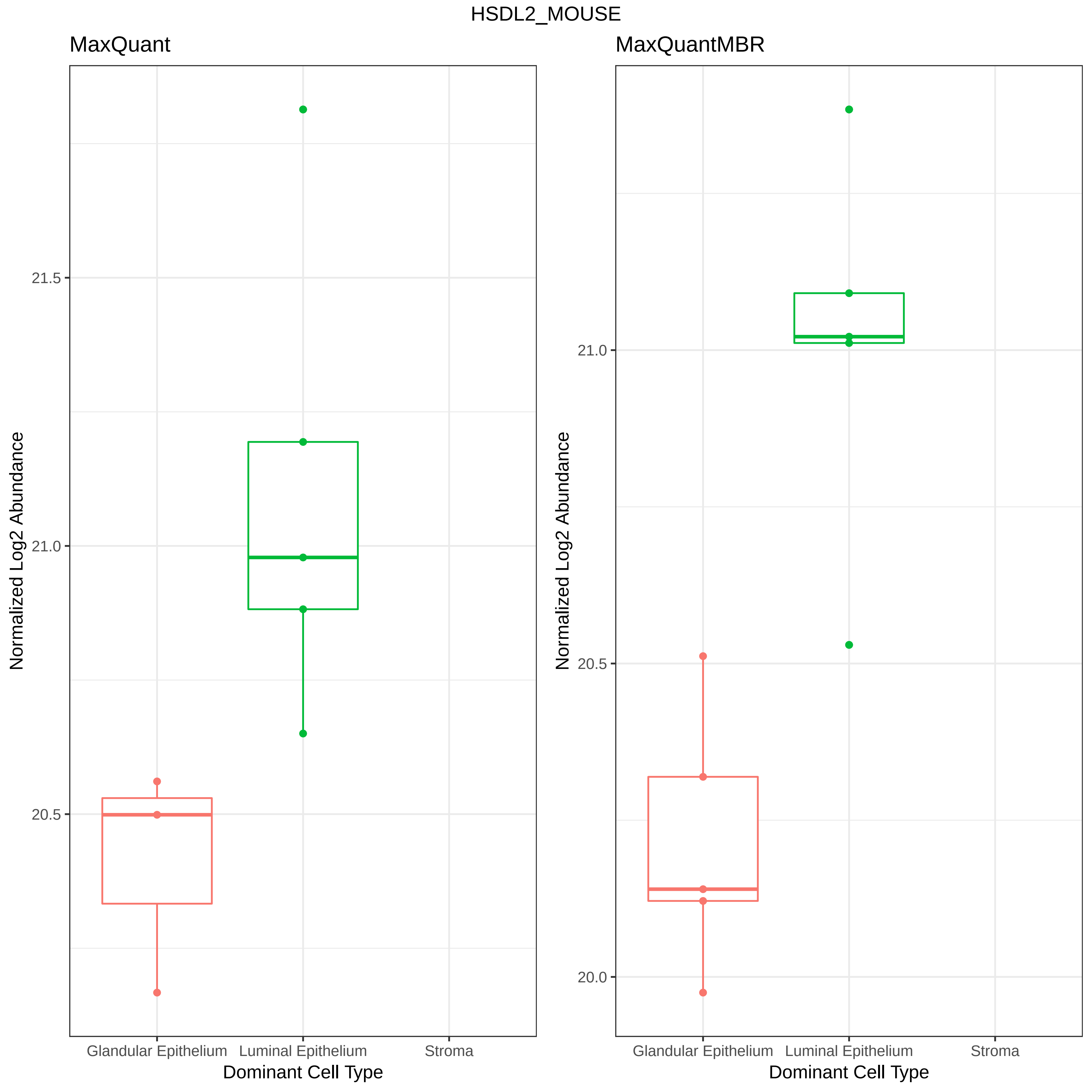
HSDL2_MOUSE
MaxQuant
MaxQuantMBR
Normalized Log2 Abundance
21.5
21.0
20.5
20.0
Glandular Epithelium
Luminal Epithelium
Stroma
Dominant Cell Type

HSDL2_MOUSE

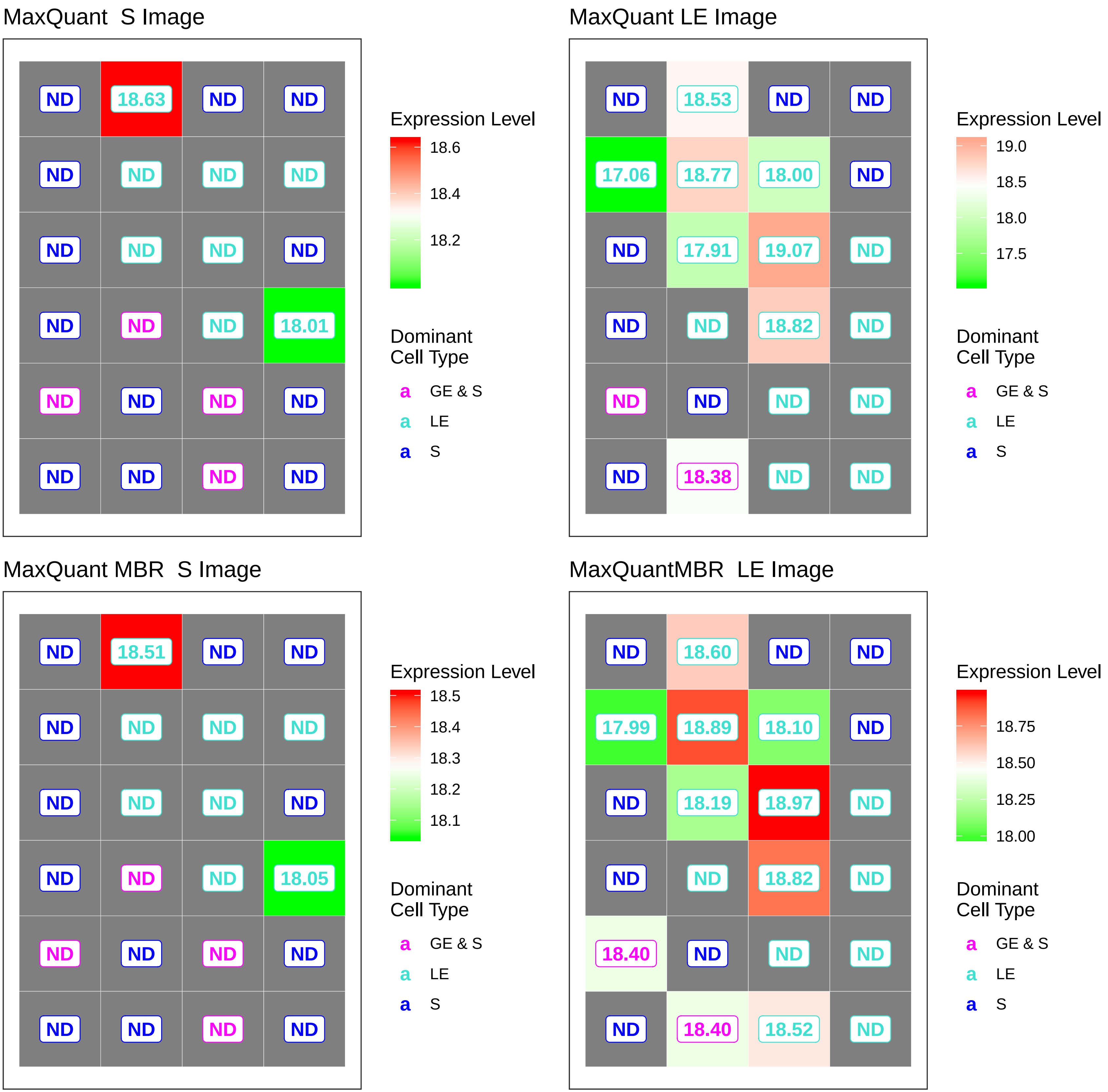
MaxQuant S Image
ND
18.63
ND
ND
ND
ND
ND
ND
ND
ND
ND
ND
ND
ND
ND
18.01
ND
ND
ND
ND
ND
ND
ND
ND
Expression Level
18.6
18.4
18.2
Dominant Cell Type
a GE & S
a LE
a S
MaxQuant LE Image
ND
18.53
ND
ND
17.06
18.77
18.00
ND
ND
17.91
19.07
ND
ND
ND
18.82
ND
ND
ND
ND
ND
ND
18.38
ND
ND
Expression Level
19.0
18.5
18.0
17.5
Dominant Cell Type
a GE & S
a LE
a S
MaxQuant MBR S Image
ND
18.51
ND
ND
ND
ND
ND
ND
ND
ND
ND
ND
ND
ND
ND
18.05
ND
ND
ND
ND
ND
ND
ND
ND
Expression Level
18.5
18.4
18.3
18.2
18.1
Dominant Cell Type
a GE & S
a LE
a S
MaxQuantMBR LE Image
ND
18.60
ND
ND
17.99
18.89
18.10
ND
ND
18.19
18.97
ND
ND
ND
18.82
ND
18.40
ND
ND
ND
ND
18.40
18.52
ND
Expression Level
18.75
18.50
18.25
18.00
Dominant Cell Type
a GE & S
a LE
a S

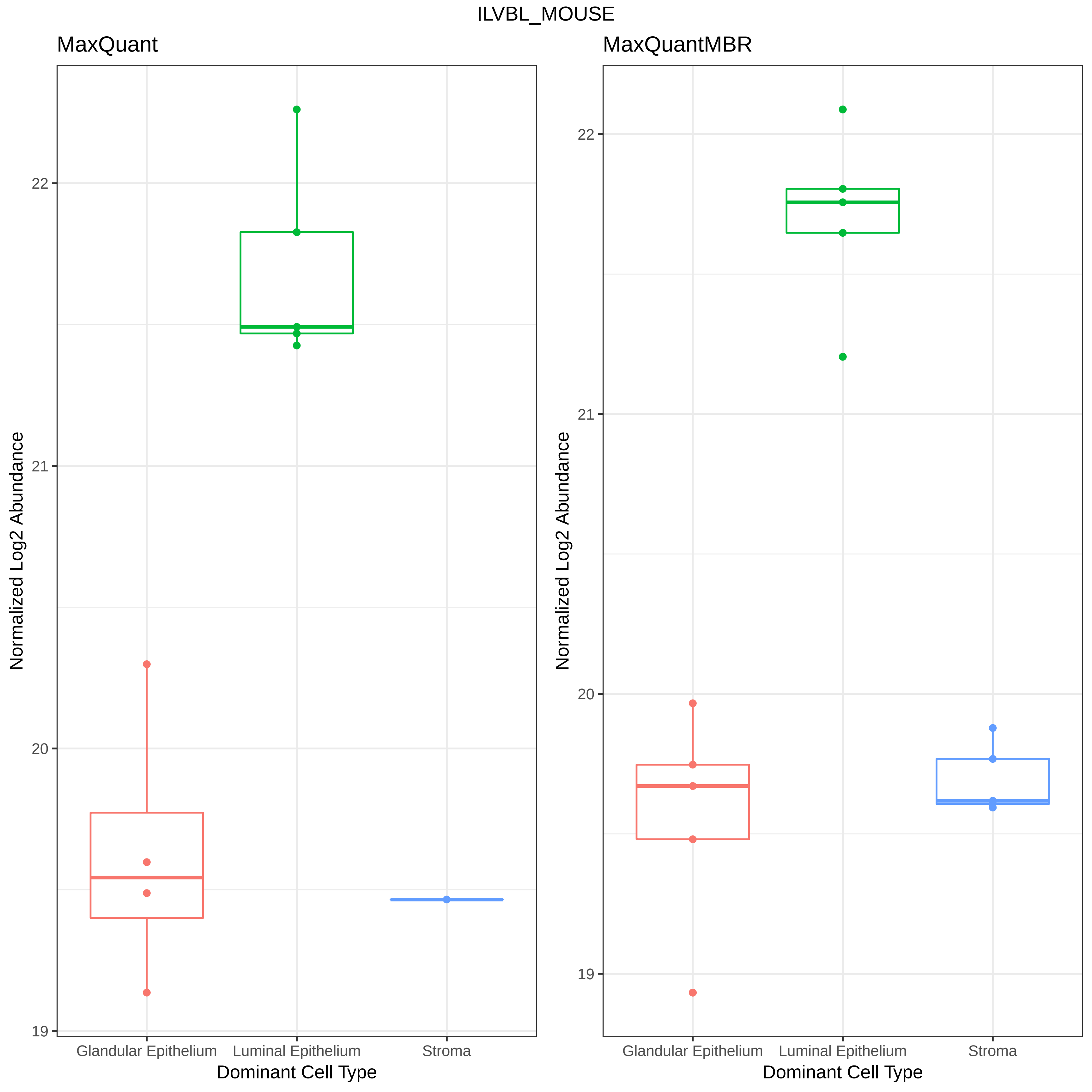

ILVBL_MOUSE
MaxQuant
MaxQuantMBR
Normalized Log2 Abundance
22
21
20
19
Glandular Epithelium
Luminal Epithelium
Stroma
Dominant Cell Type

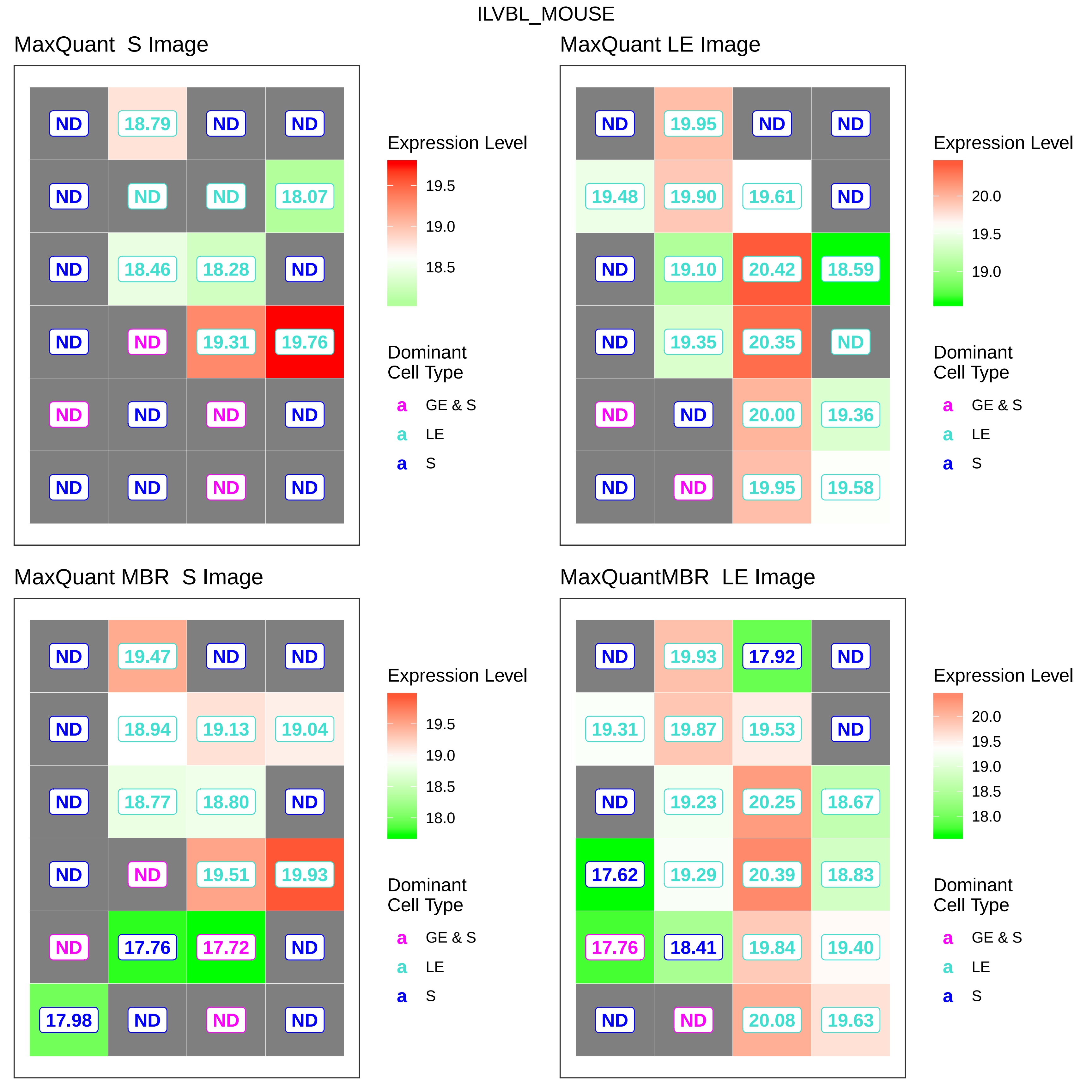
ILVBL_MOUSE
MaxQuant S Image
ND 18.79 ND ND
ND ND ND 18.07
ND 18.46 18.28 ND
ND ND 19.31 19.76
ND ND ND ND
ND ND ND ND
Expression Level
19.5
19.0
18.5
Dominant Cell Type
a GE & S
a LE
a S
MaxQuant LE Image
ND 19.95 ND ND
19.48 19.90 19.61 ND
ND 19.10 20.42 18.59
ND 19.35 20.35 ND
ND ND 20.00 19.36
ND ND 19.95 19.58
Expression Level
20.0
19.5
19.0
Dominant Cell Type
a GE & S
a LE
a S
MaxQuant MBR S Image
ND 19.47 ND ND
ND 18.94 19.13 19.04
ND 18.77 18.80 ND
ND ND 19.51 19.93
ND 17.76 17.72 ND
17.98 ND ND ND
Expression Level
19.5
19.0
18.5
18.0
Dominant Cell Type
a GE & S
a LE
a S
MaxQuantMBR LE Image
ND 19.93 17.92 ND
19.31 19.87 19.53 ND
ND 19.23 20.25 18.67
17.62 19.29 20.39 18.83
17.76 18.41 19.84 19.40
ND ND 20.08 19.63
Expression Level
20.0
19.5
19.0
18.5
18.0
Dominant Cell Type
a GE & S
a LE
a S

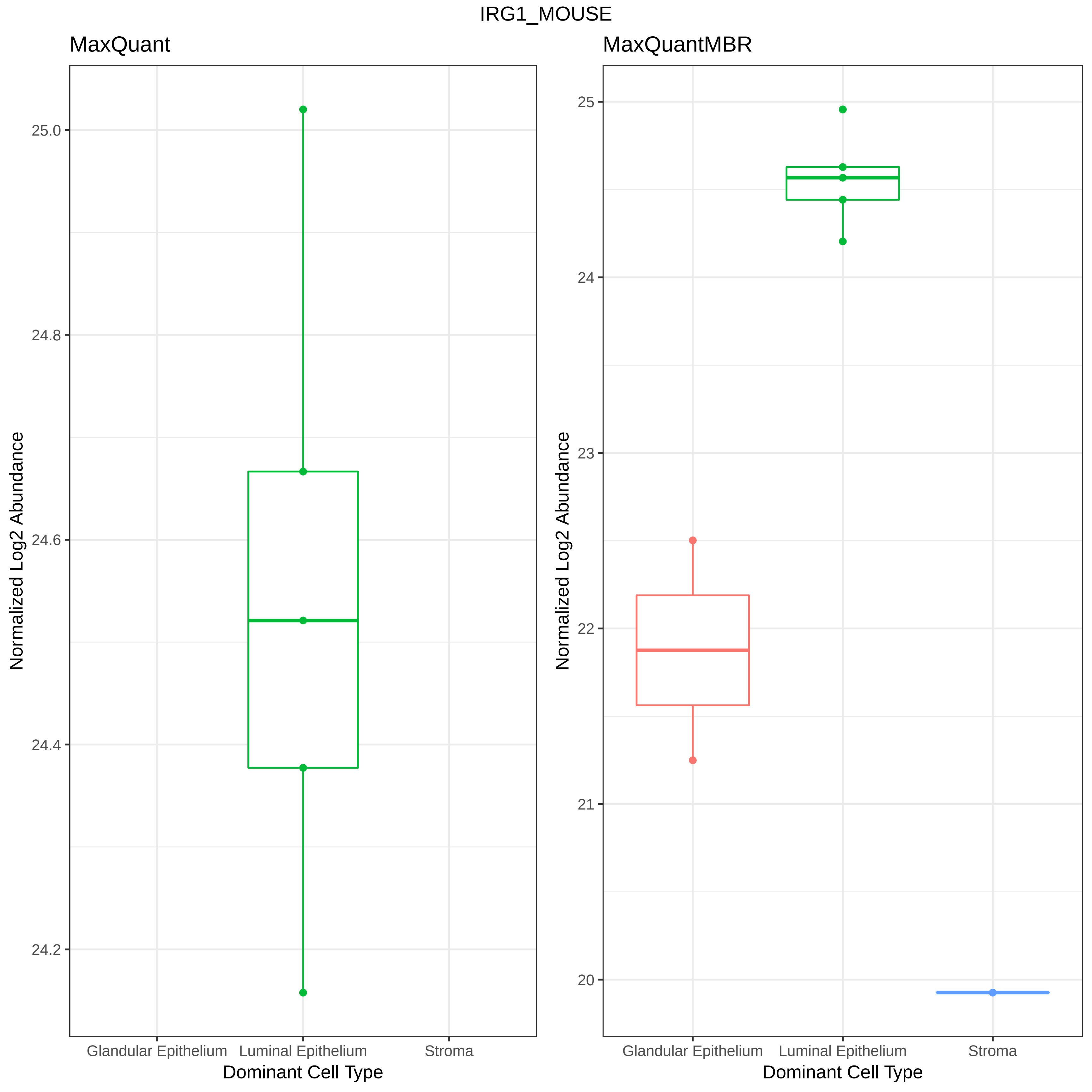
IRG1_MOUSE
MaxQuant
MaxQuantMBR
Normalized Log2 Abundance
25.0
24.8
24.6
24.4
24.2
25
24
23
22
21
20
Glandular Epithelium
Luminal Epithelium
Stroma
Dominant Cell Type

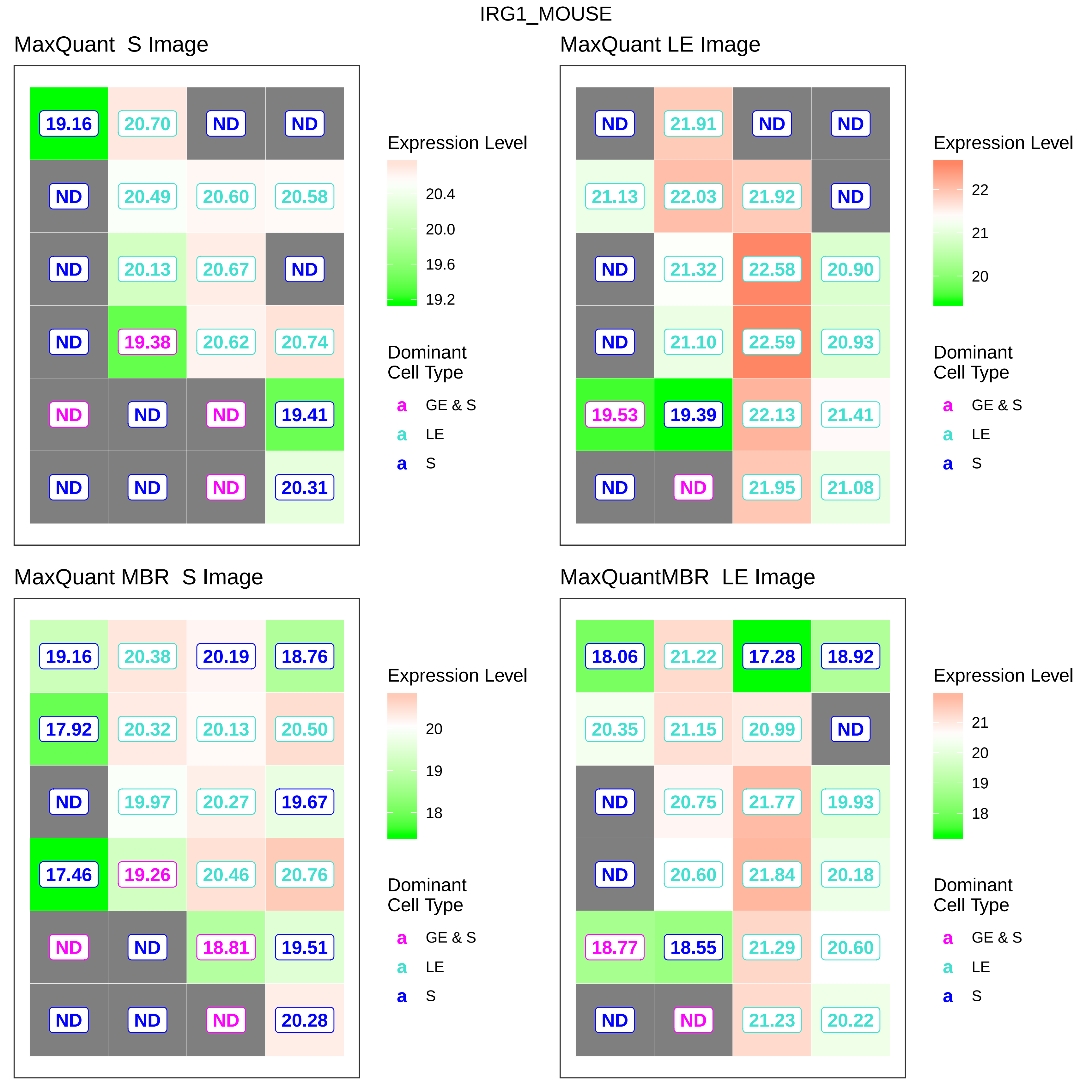

IRG1_MOUSE
MaxQuant S Image
19.16
20.70
ND
ND
ND
20.49
20.60
20.58
ND
20.13
20.67
ND
ND
19.38
20.62
20.74
ND
ND
ND
19.41
ND
ND
ND
20.31
Expression Level
20.4
20.0
19.6
19.2
Dominant Cell Type
a GE & S
a LE
a S
MaxQuant LE Image
ND
21.91
ND
ND
21.13
22.03
21.92
ND
ND
21.32
22.58
20.90
ND
21.10
22.59
20.93
19.53
19.39
22.13
21.41
ND
ND
21.95
21.08
Expression Level
22
21
20
Dominant Cell Type
a GE & S
a LE
a S
MaxQuant MBR S Image
19.16
20.38
20.19
18.76
17.92
20.32
20.13
20.50
ND
19.97
20.27
19.67
17.46
19.26
20.46
20.76
ND
ND
18.81
19.51
ND
ND
ND
20.28
Expression Level
20
19
18
Dominant Cell Type
a GE & S
a LE
a S
MaxQuantMBR LE Image
18.06
21.22
17.28
18.92
20.35
21.15
20.99
ND
ND
20.75
21.77
19.93
ND
20.60
21.84
20.18
18.77
18.55
21.29
20.60
ND
ND
21.23
20.22
Expression Level
21
20
19
18
Dominant Cell Type
a GE & S
a LE
a S

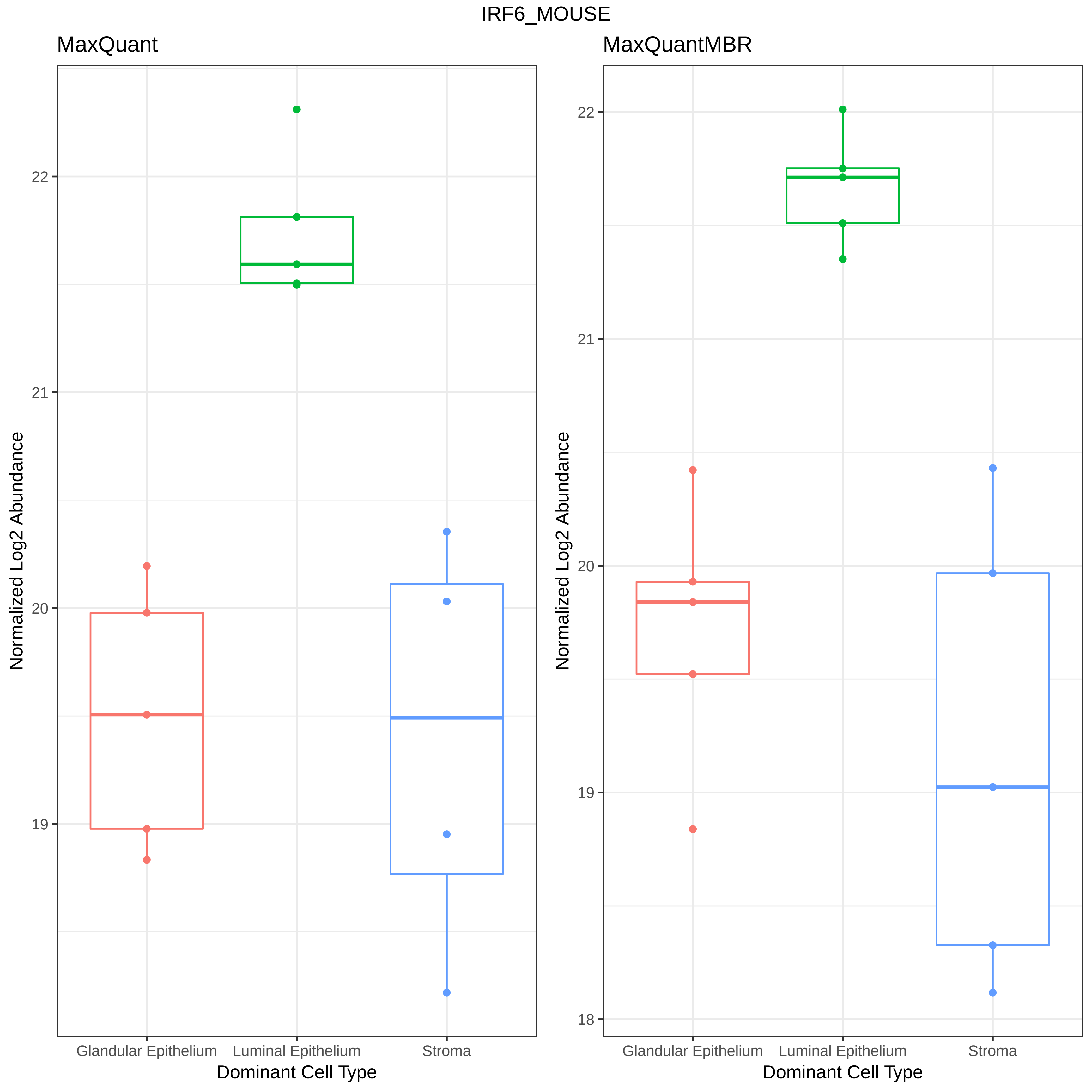

IRF6_MOUSE
MaxQuant
MaxQuantMBR
Normalized Log2 Abundance
Dominant Cell Type
Glandular Epithelium
Luminal Epithelium
Stroma
19
20
21
22
18

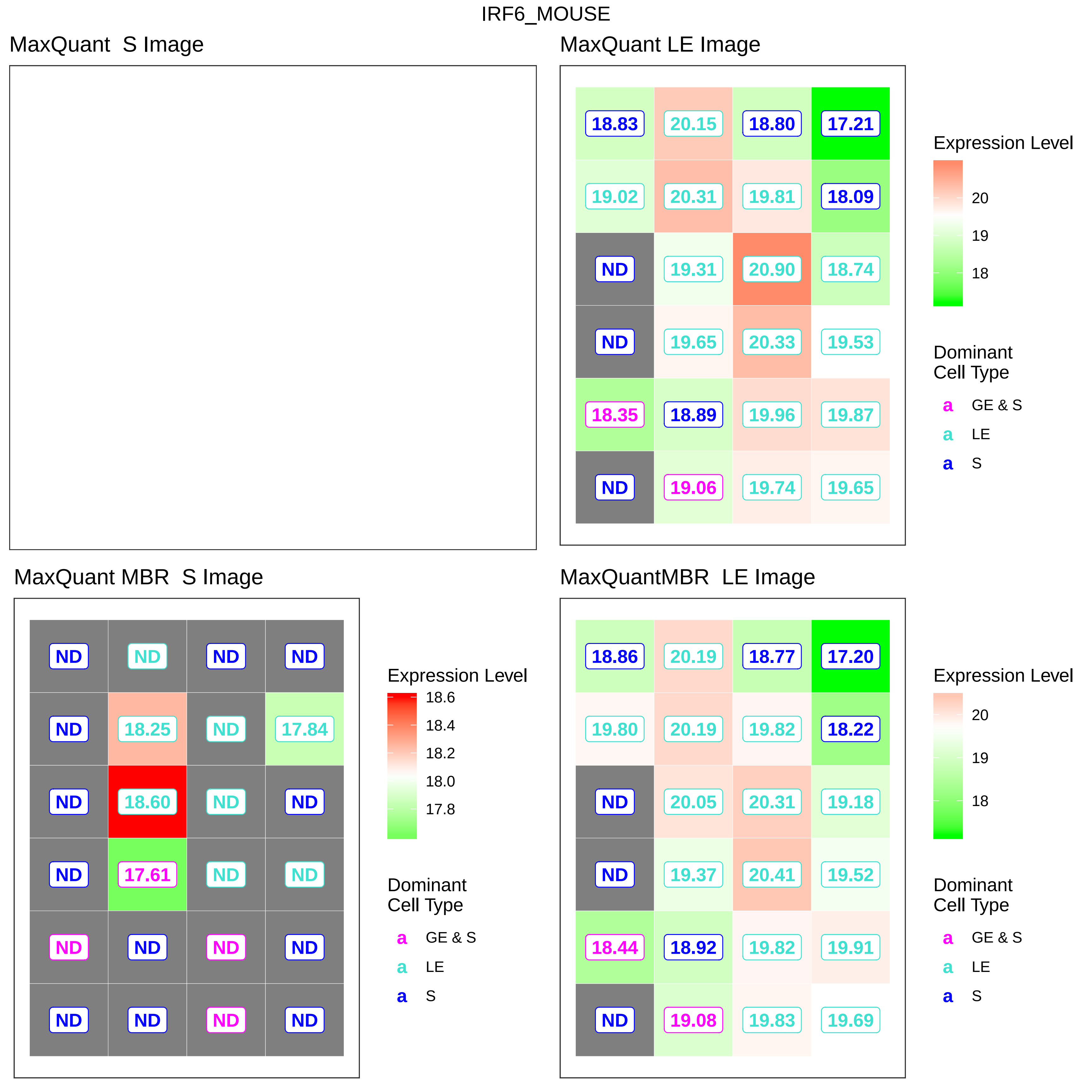
IRF6_MOUSE
MaxQuant S Image
MaxQuant LE Image
18.83 20.15 18.80 17.21
19.02 20.31 19.81 18.09
ND 19.31 20.90 18.74
ND 19.65 20.33 19.53
18.35 18.89 19.96 19.87
ND 19.06 19.74 19.65
Expression Level
20
19
18
Dominant Cell Type
a GE & S
a LE
a S
MaxQuant MBR S Image
ND ND ND ND
ND 18.25 ND 17.84
ND 18.60 ND ND
ND 17.61 ND ND
ND ND ND ND
ND ND ND ND
Expression Level
18.6
18.4
18.2
18.0
17.8
Dominant Cell Type
a GE & S
a LE
a S
MaxQuantMBR LE Image
18.86 20.19 18.77 17.20
19.80 20.19 19.82 18.22
ND 20.05 20.31 19.18
ND 19.37 20.41 19.52
18.44 18.92 19.82 19.91
ND 19.08 19.83 19.69
Expression Level
20
19
18
Dominant Cell Type
a GE & S
a LE
a S

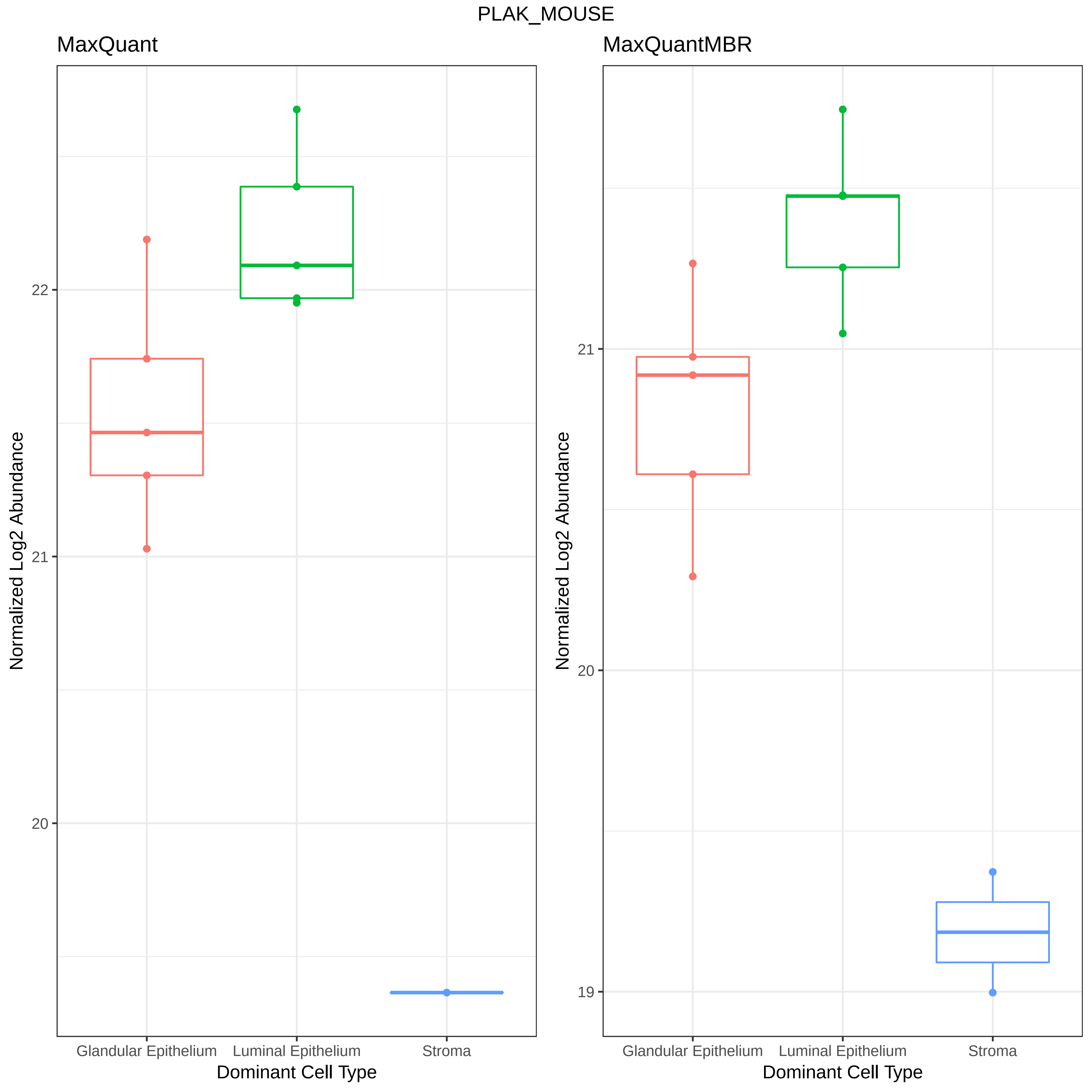
PLAK_MOUSE
MaxQuant
MaxQuantMBR
Normalized Log2 Abundance
22
21
20
19
Glandular Epithelium
Luminal Epithelium
Stroma
Dominant Cell Type

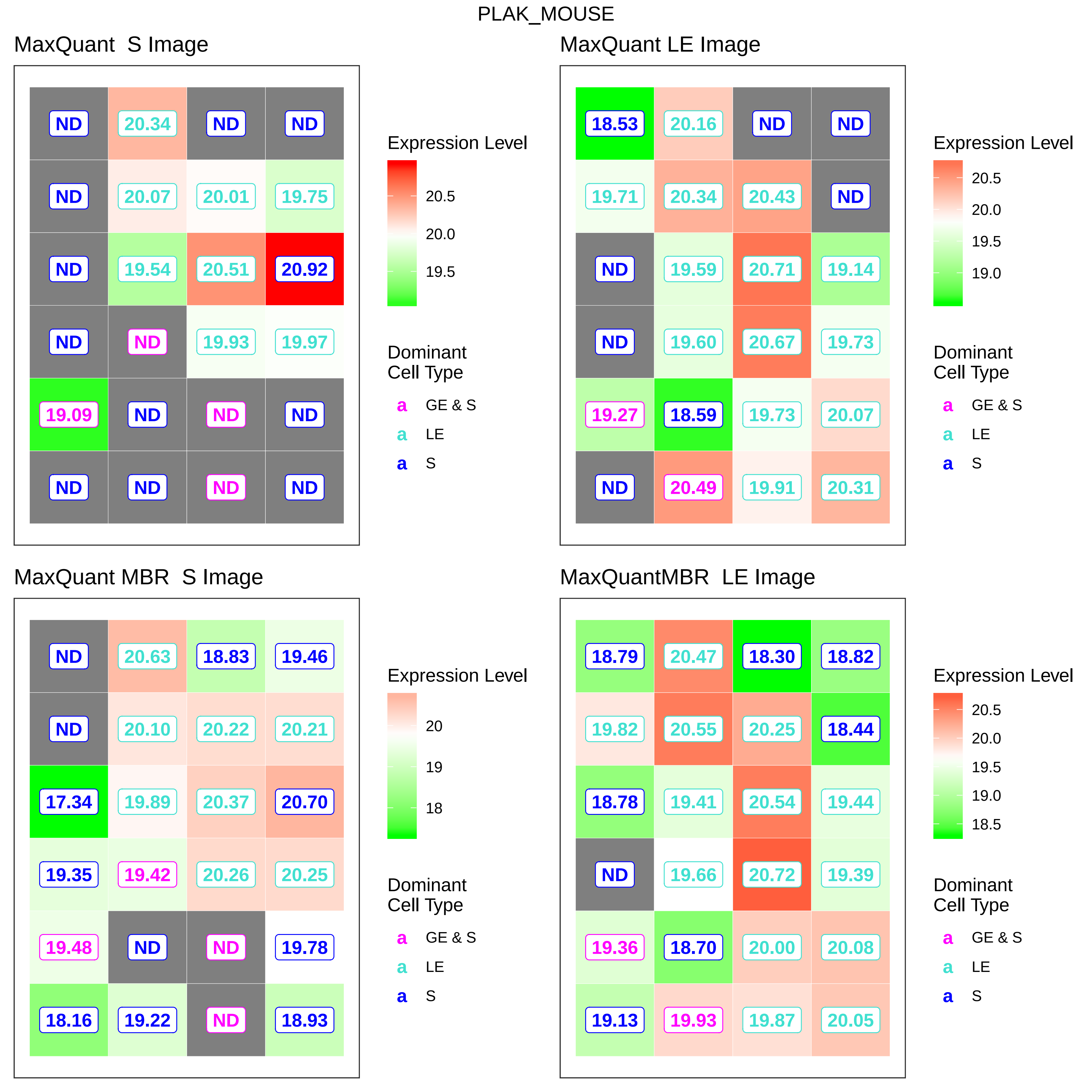

PLAK_MOUSE
MaxQuant S Image
ND 20.34 ND ND
ND 20.07 20.01 19.75
ND 19.54 20.51 20.92
ND ND 19.93 19.97
19.09 ND ND ND
ND ND ND ND
Expression Level
20.5
20.0
19.5
Dominant Cell Type
a GE & S
a LE
a S
MaxQuant LE Image
18.53 20.16 ND ND
19.71 20.34 20.43 ND
ND 19.59 20.71 19.14
ND 19.60 20.67 19.73
19.27 18.59 19.73 20.07
ND 20.49 19.91 20.31
Expression Level
20.5
20.0
19.5
19.0
Dominant Cell Type
a GE & S
a LE
a S
MaxQuant MBR S Image
ND 20.63 18.83 19.46
ND 20.10 20.22 20.21
17.34 19.89 20.37 20.70
19.35 19.42 20.26 20.25
19.48 ND ND 19.78
18.16 19.22 ND 18.93
Expression Level
20
19
18
Dominant Cell Type
a GE & S
a LE
a S
MaxQuantMBR LE Image
18.79 20.47 18.30 18.82
19.82 20.55 20.25 18.44
18.78 19.41 20.54 19.44
ND 19.66 20.72 19.39
19.36 18.70 20.00 20.08
19.13 19.93 19.87 20.05
Expression Level
20.5
20.0
19.5
19.0
18.5
Dominant Cell Type
a GE & S
a LE
a S

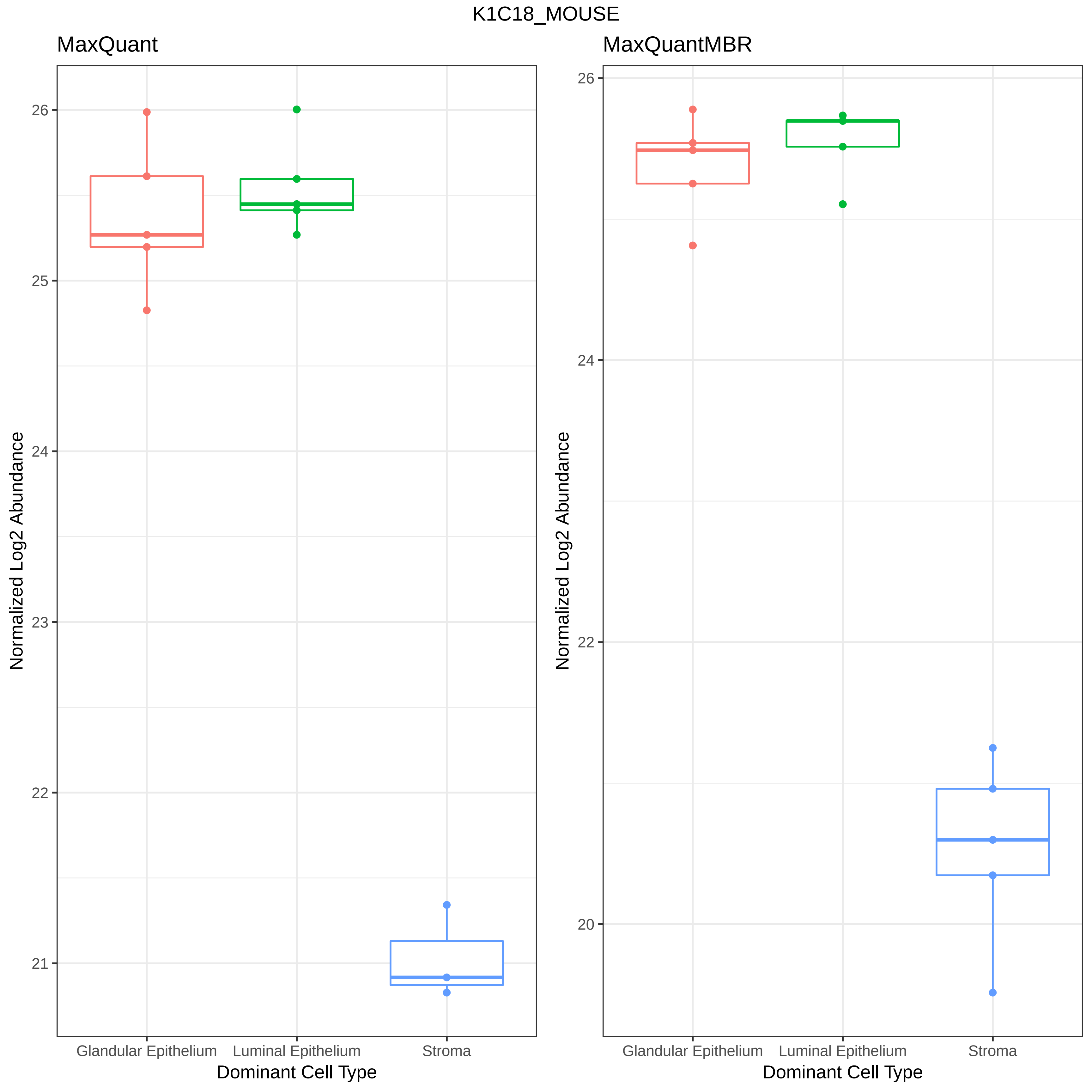

K1C18_MOUSE
MaxQuant
MaxQuantMBR
Normalized Log2 Abundance
26
25
24
23
22
21
20
Glandular Epithelium
Luminal Epithelium
Stroma
Dominant Cell Type

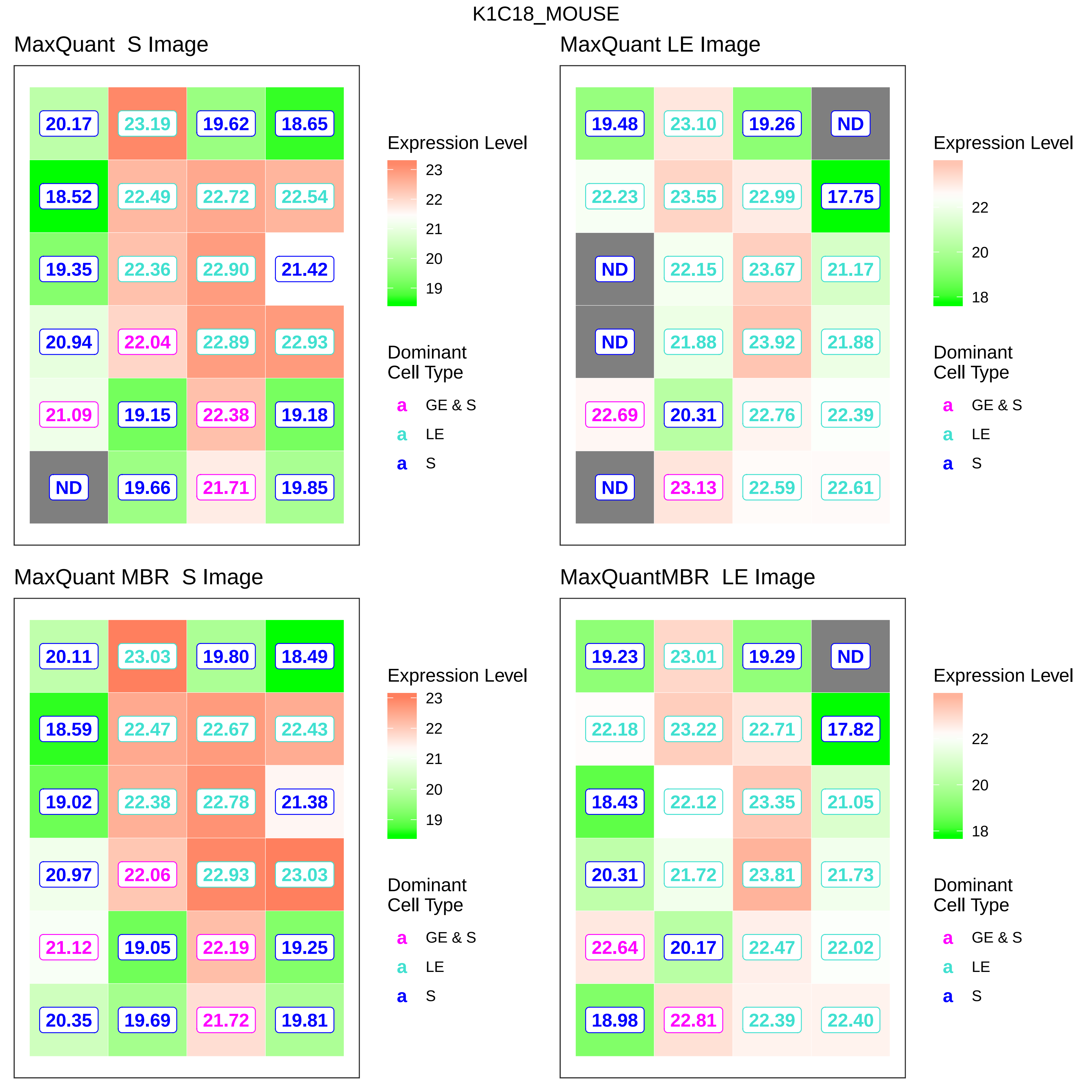
K1C18_MOUSE
MaxQuant S Image
20.17 23.19 19.62 18.65
18.52 22.49 22.72 22.54
19.35 22.36 22.90 21.42
20.94 22.04 22.89 22.93
21.09 19.15 22.38 19.18
ND 19.66 21.71 19.85
Expression Level
23
22
21
20
19
Dominant Cell Type
a GE & S
a LE
a S
MaxQuant LE Image
19.48 23.10 19.26 ND
22.23 23.55 22.99 17.75
ND 22.15 23.67 21.17
ND 21.88 23.92 21.88
22.69 20.31 22.76 22.39
ND 23.13 22.59 22.61
Expression Level
22
20
18
Dominant Cell Type
a GE & S
a LE
a S
MaxQuant MBR S Image
20.11 23.03 19.80 18.49
18.59 22.47 22.67 22.43
19.02 22.38 22.78 21.38
20.97 22.06 22.93 23.03
21.12 19.05 22.19 19.25
20.35 19.69 21.72 19.81
Expression Level
23
22
21
20
19
Dominant Cell Type
a GE & S
a LE
a S
MaxQuantMBR LE Image
19.23 23.01 19.29 ND
22.18 23.22 22.71 17.82
18.43 22.12 23.35 21.05
20.31 21.72 23.81 21.73
22.64 20.17 22.47 22.02
18.98 22.81 22.39 22.40
Expression Level
22
20
18
Dominant Cell Type
a GE & S
a LE
a S

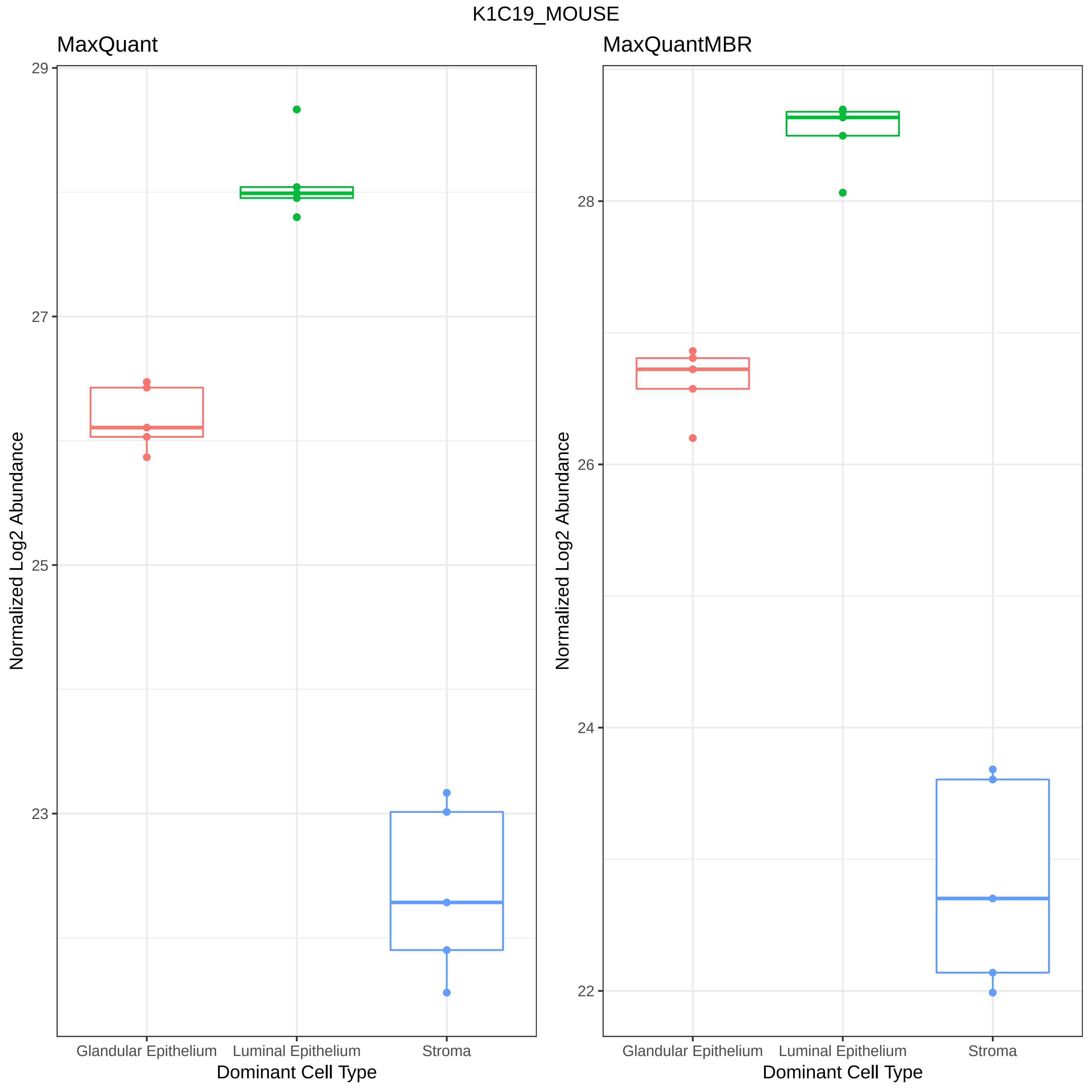

K1C19_MOUSE
MaxQuant
MaxQuantMBR
29
27
25
23
28
26
24
22
Normalized Log2 Abundance
Normalized Log2 Abundance
Glandular Epithelium
Luminal Epithelium
Stroma
Glandular Epithelium
Luminal Epithelium
Stroma
Dominant Cell Type
Dominant Cell Type

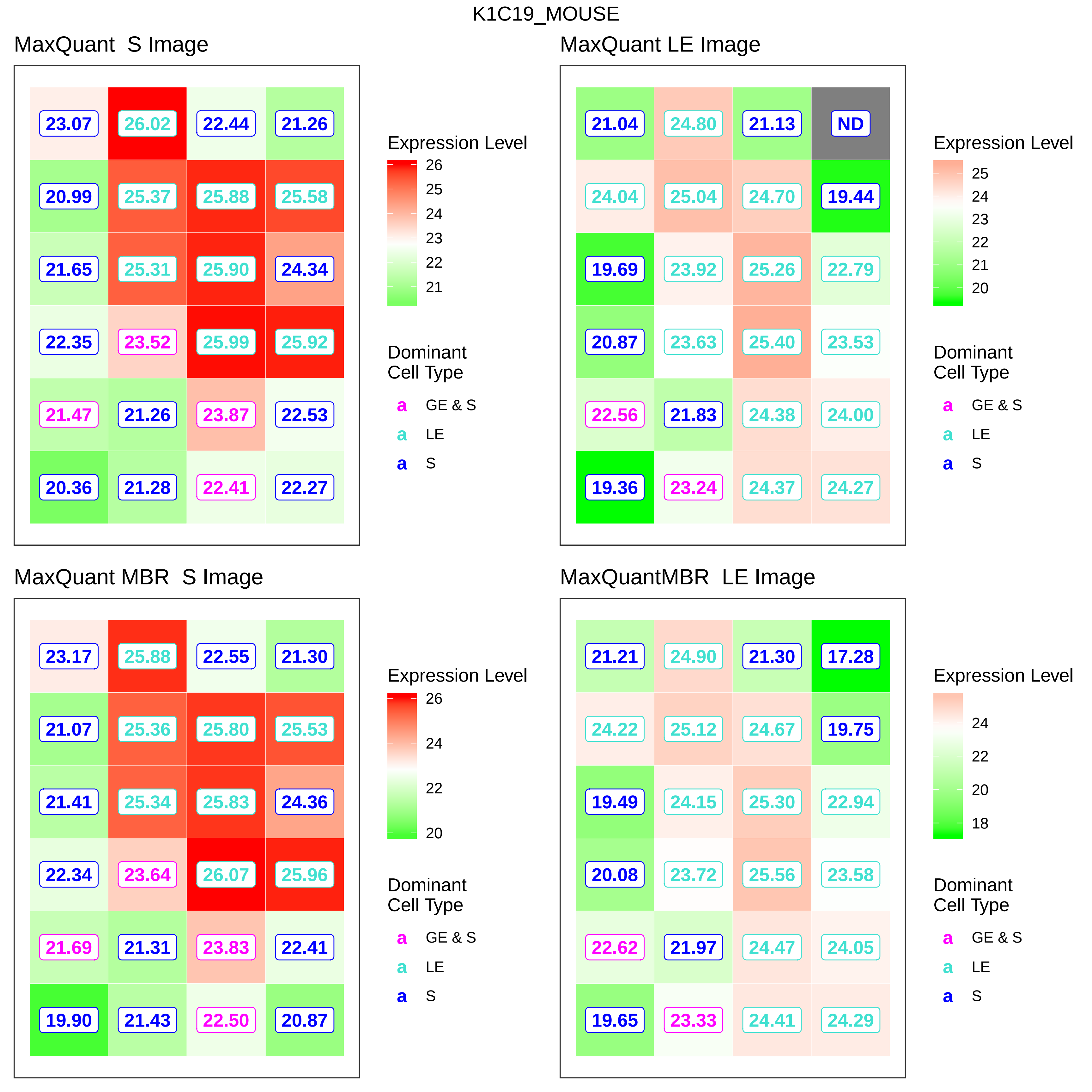
K1C19_MOUSE
MaxQuant S Image
23.07 26.02 22.44 21.26
20.99 25.37 25.88 25.58
21.65 25.31 25.90 24.34
22.35 23.52 25.99 25.92
21.47 21.26 23.87 22.53
20.36 21.28 22.41 22.27
Expression Level
26
25
24
23
22
21
Dominant Cell Type
a GE & S
a LE
a S
MaxQuant LE Image
21.04 24.80 21.13 ND
24.04 25.04 24.70 19.44
19.69 23.92 25.26 22.79
20.87 23.63 25.40 23.53
22.56 21.83 24.38 24.00
19.36 23.24 24.37 24.27
Expression Level
25
24
23
22
21
20
Dominant Cell Type
a GE & S
a LE
a S
MaxQuant MBR S Image
23.17 25.88 22.55 21.30
21.07 25.36 25.80 25.53
21.41 25.34 25.83 24.36
22.34 23.64 26.07 25.96
21.69 21.31 23.83 22.41
19.90 21.43 22.50 20.87
Expression Level
26
24
22
20
Dominant Cell Type
a GE & S
a LE
a S
MaxQuantMBR LE Image
21.21 24.90 21.30 17.28
24.22 25.12 24.67 19.75
19.49 24.15 25.30 22.94
20.08 23.72 25.56 23.58
22.62 21.97 24.47 24.05
19.65 23.33 24.41 24.29
Expression Level
24
22
20
18
Dominant Cell Type
a GE & S
a LE
a S

K1C20_MOUSE

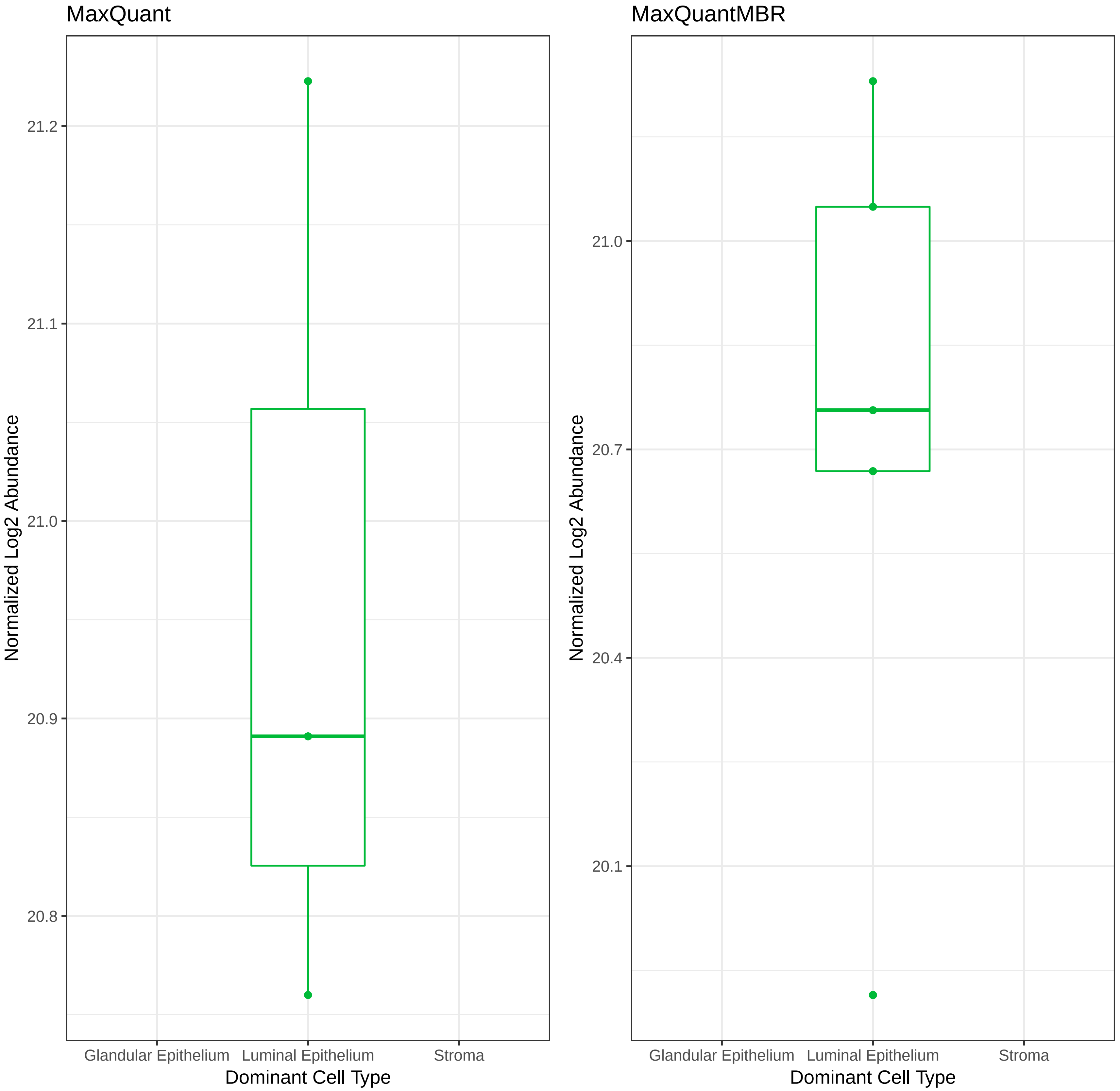
MaxQuant
MaxQuantMBR
Normalized Log2 Abundance
21.2
21.1
21.0
20.9
20.8
21.0
20.7
20.4
20.1
Glandular Epithelium
Luminal Epithelium
Stroma
Dominant Cell Type

K1C20_MOUSE

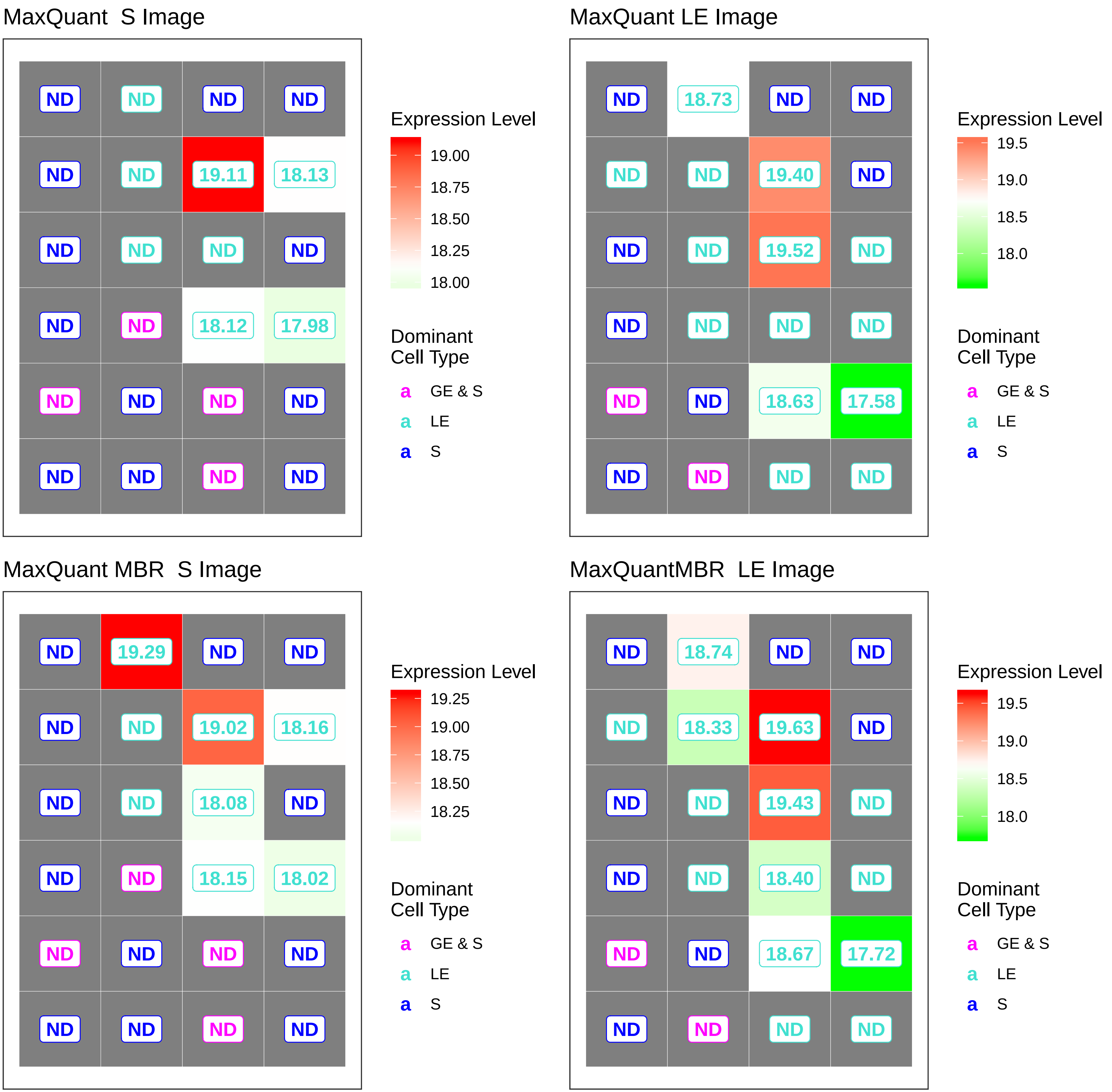
MaxQuant S Image
ND
19.11
18.13
18.12
17.98
Expression Level
19.00
18.75
18.50
18.25
18.00
Dominant Cell Type
a GE & S
a LE
a S
MaxQuant LE Image
18.73
19.40
19.52
18.63
17.58
Expression Level
19.5
19.0
18.5
18.0
MaxQuant MBR S Image
19.29
19.02
18.16
18.08
18.15
18.02
Expression Level
19.25
19.00
18.75
18.50
18.25
MaxQuantMBR LE Image
18.74
18.33
19.63
19.43
18.40
18.67
17.72
Expression Level
19.5
19.0
18.5
18.0

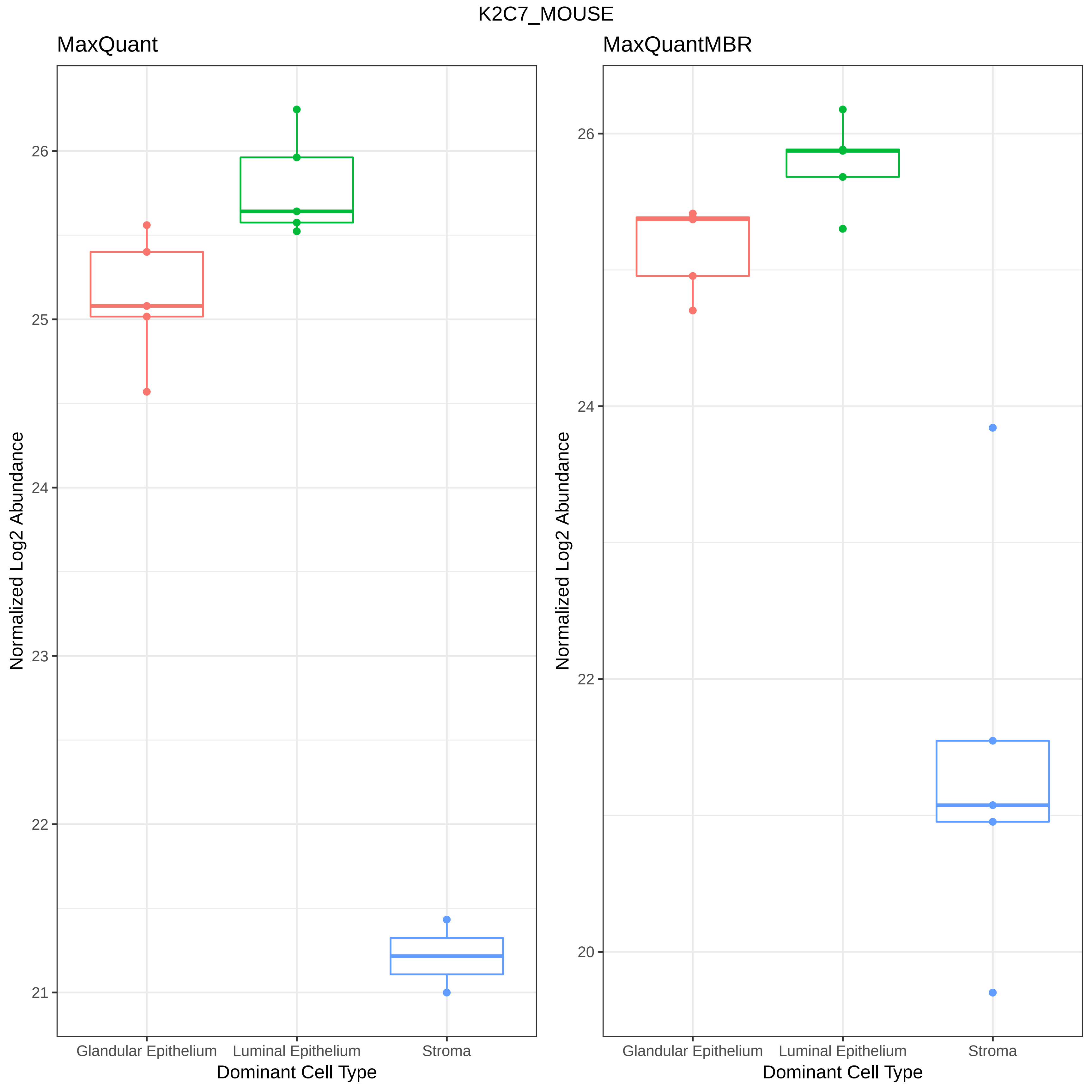

K2C7_MOUSE
MaxQuant
MaxQuantMBR
Normalized Log2 Abundance
26
25
24
23
22
21
20
Glandular Epithelium
Luminal Epithelium
Stroma
Dominant Cell Type

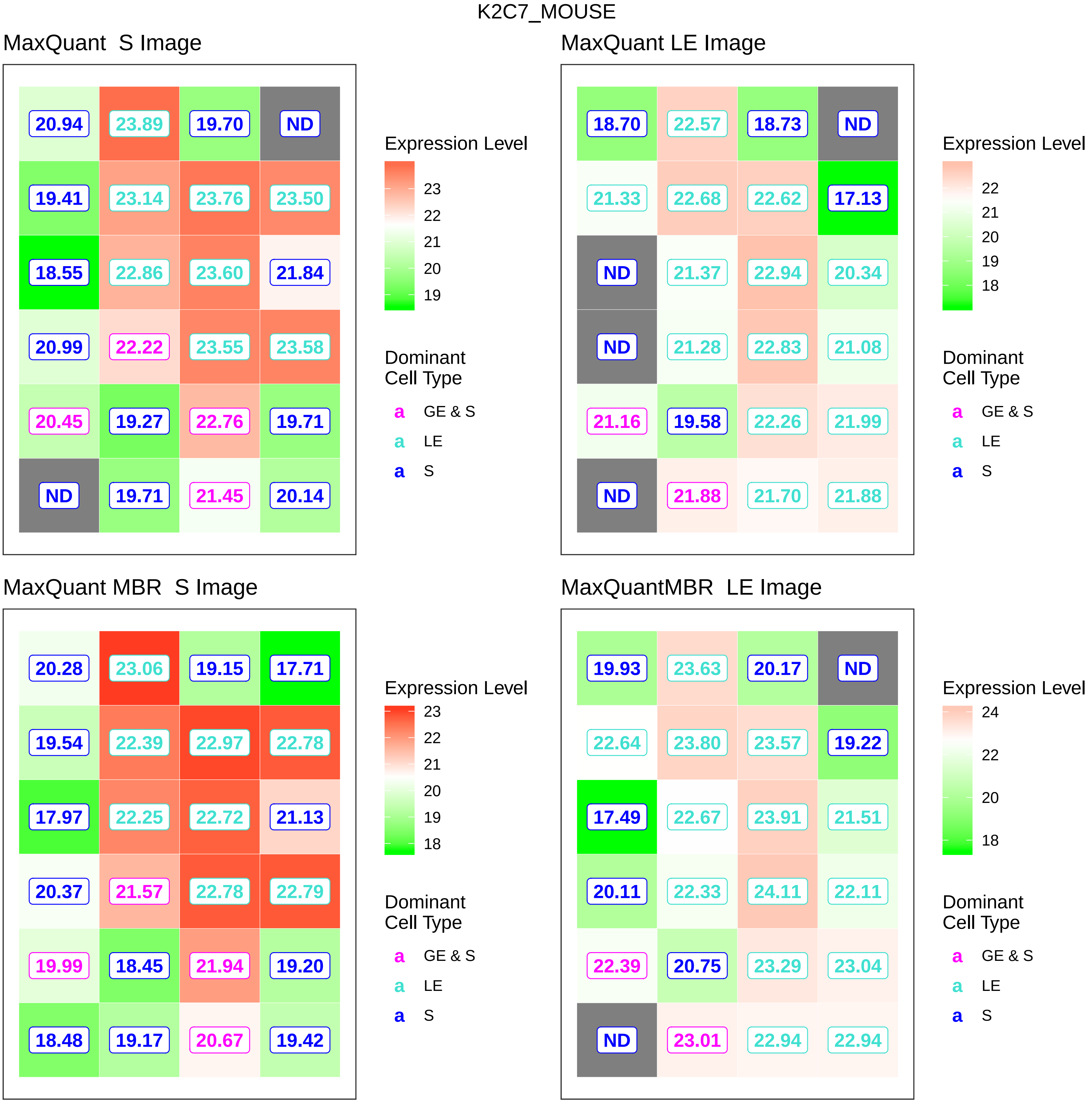
K2C7_MOUSE
MaxQuant S Image
20.94 23.89 19.70 ND
19.41 23.14 23.76 23.50
18.55 22.86 23.60 21.84
20.99 22.22 23.55 23.58
20.45 19.27 22.76 19.71
ND 19.71 21.45 20.14
Expression Level
23
22
21
20
19
Dominant Cell Type
a GE & S
a LE
a S
MaxQuant LE Image
18.70 22.57 18.73 ND
21.33 22.68 22.62 17.13
ND 21.37 22.94 20.34
ND 21.28 22.83 21.08
21.16 19.58 22.26 21.99
ND 21.88 21.70 21.88
Expression Level
22
21
20
19
18
Dominant Cell Type
a GE & S
a LE
a S
MaxQuant MBR S Image
20.28 23.06 19.15 17.71
19.54 22.39 22.97 22.78
17.97 22.25 22.72 21.13
20.37 21.57 22.78 22.79
19.99 18.45 21.94 19.20
18.48 19.17 20.67 19.42
Expression Level
23
22
21
20
19
18
Dominant Cell Type
a GE & S
a LE
a S
MaxQuantMBR LE Image
19.93 23.63 20.17 ND
22.64 23.80 23.57 19.22
17.49 22.67 23.91 21.51
20.11 22.33 24.11 22.11
22.39 20.75 23.29 23.04
ND 23.01 22.94 22.94
Expression Level
24
22
20
18
Dominant Cell Type
a GE & S
a LE
a S

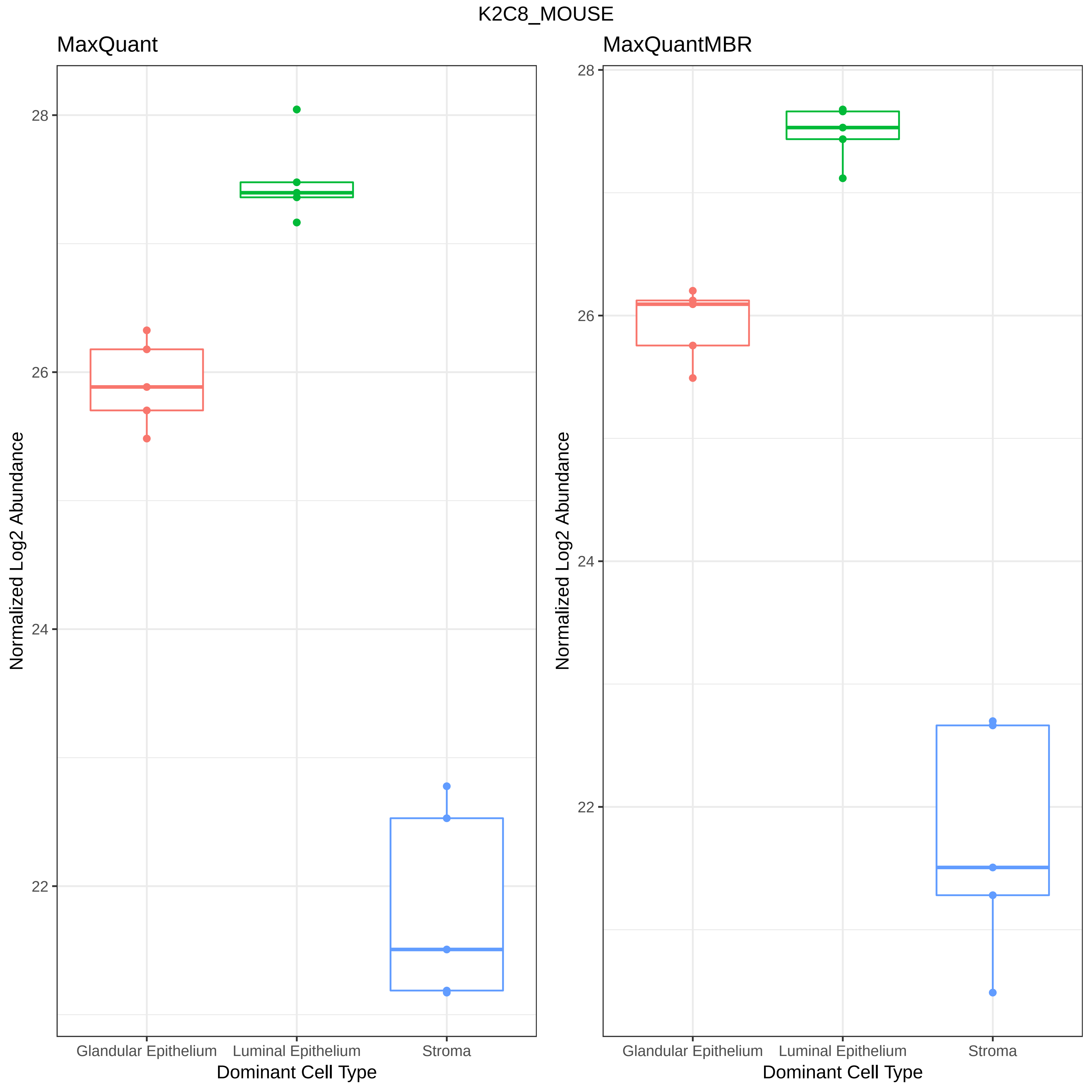

K2C8_MOUSE
MaxQuant
MaxQuantMBR
Normalized Log2 Abundance
28
26
24
22
Glandular Epithelium
Luminal Epithelium
Stroma
Dominant Cell Type

K2C8_MOUSE

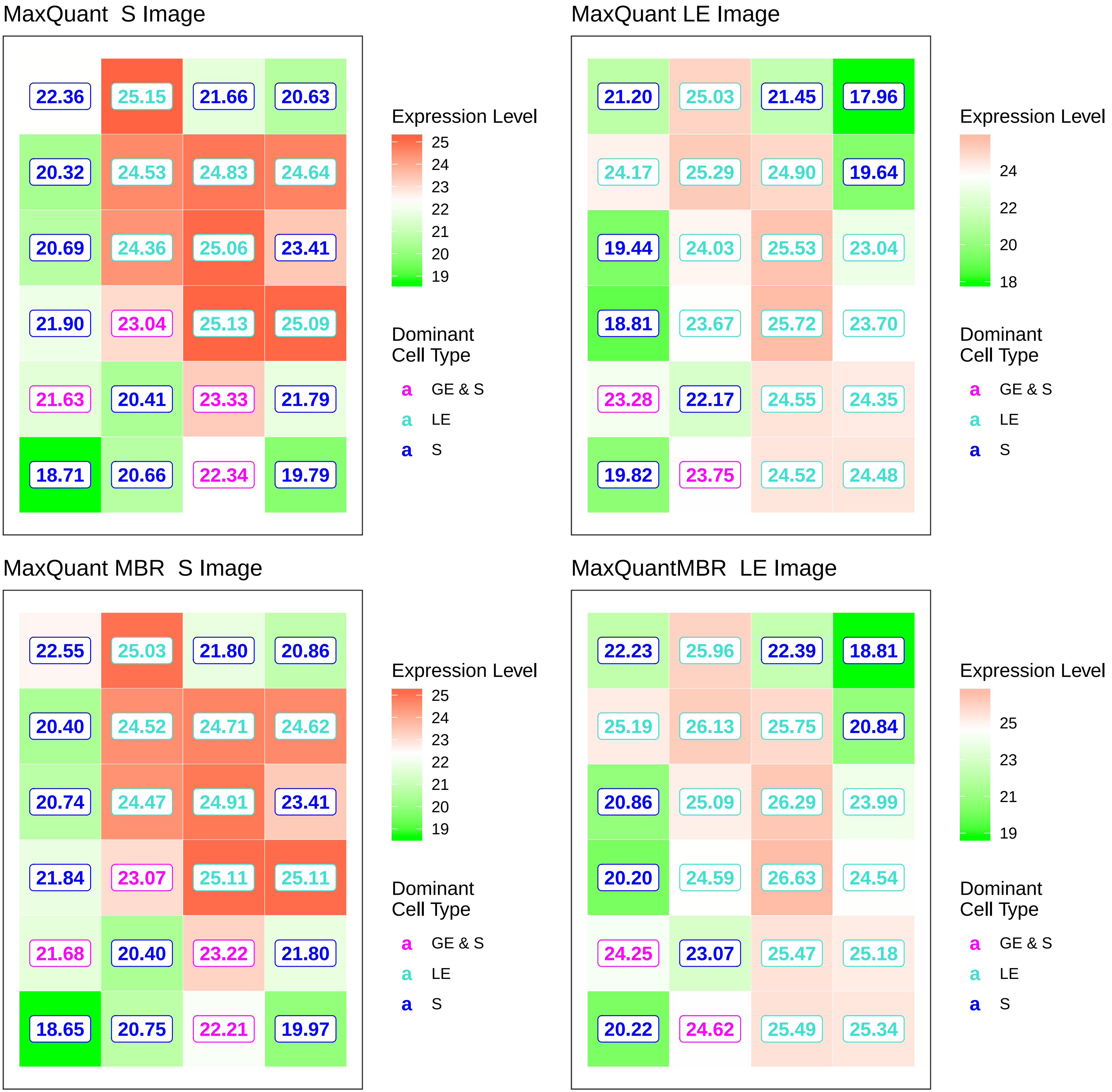

MaxQuant S Image
22.36 25.15 21.66 20.63
20.32 24.53 24.83 24.64
20.69 24.36 25.06 23.41
21.90 23.04 25.13 25.09
21.63 20.41 23.33 21.79
18.71 20.66 22.34 19.79
Expression Level
25 24 23 22 21 20 19
Dominant Cell Type
a GE & S
a LE
a S
MaxQuant LE Image
21.20 25.03 21.45 17.96
24.17 25.29 24.90 19.64
19.44 24.03 25.53 23.04
18.81 23.67 25.72 23.70
23.28 22.17 24.55 24.35
19.82 23.75 24.52 24.48
Expression Level
24 22 20 18
Dominant Cell Type
a GE & S
a LE
a S
MaxQuant MBR S Image
22.55 25.03 21.80 20.86
20.40 24.52 24.71 24.62
20.74 24.47 24.91 23.41
21.84 23.07 25.11 25.11
21.68 20.40 23.22 21.80
18.65 20.75 22.21 19.97
Expression Level
25 24 23 22 21 20 19
Dominant Cell Type
a GE & S
a LE
a S
MaxQuantMBR LE Image
22.23 25.96 22.39 18.81
25.19 26.13 25.75 20.84
20.86 25.09 26.29 23.99
20.20 24.59 26.63 24.54
24.25 23.07 25.47 25.18
20.22 24.62 25.49 25.34
Expression Level
25 23 21 19
Dominant Cell Type
a GE & S
a LE
a S

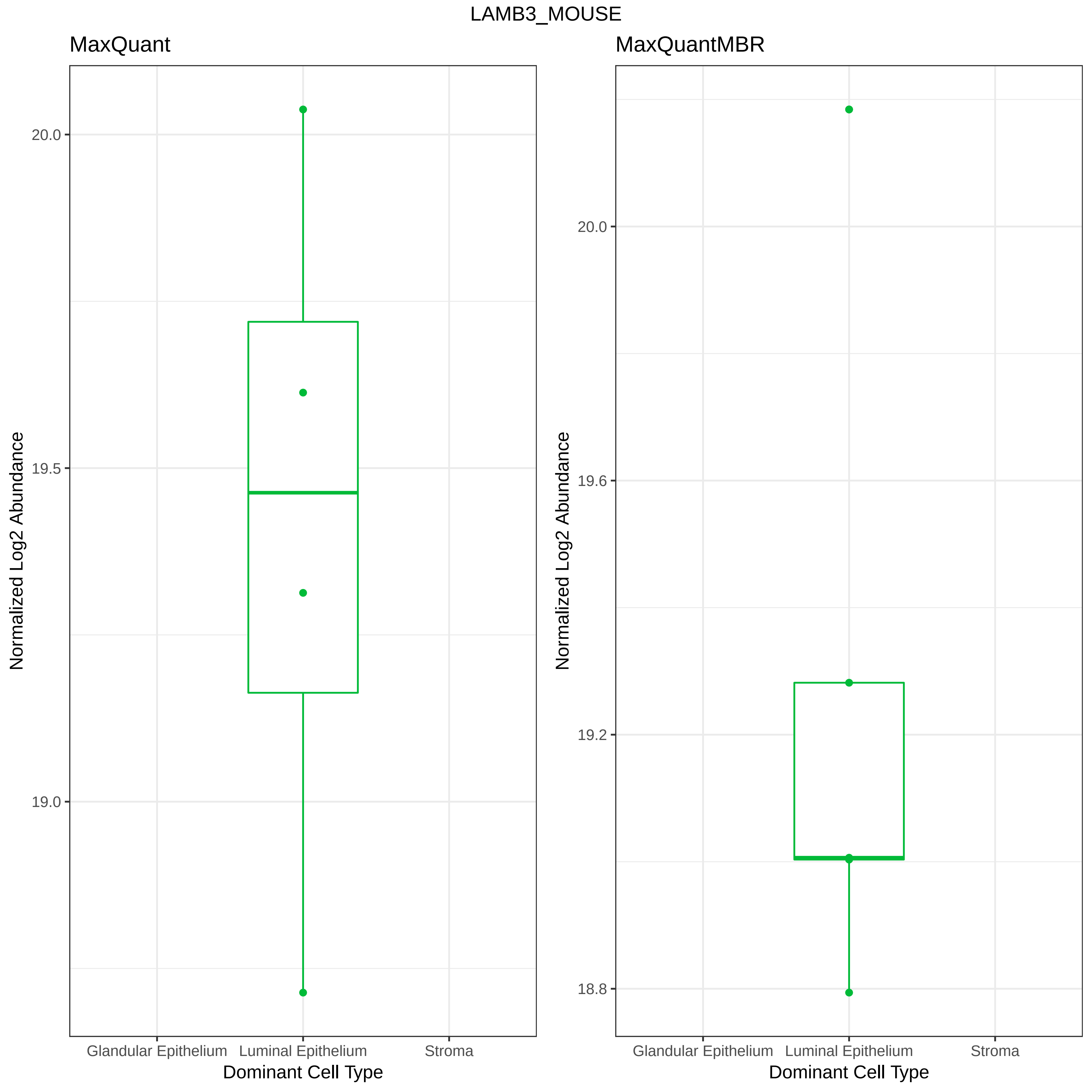
LAMB3_MOUSE
MaxQuant
MaxQuantMBR
Normalized Log2 Abundance
20.0
19.5
19.0
20.0
19.6
19.2
18.8
Glandular Epithelium
Luminal Epithelium
Stroma
Dominant Cell Type

LAMB3_MOUSE

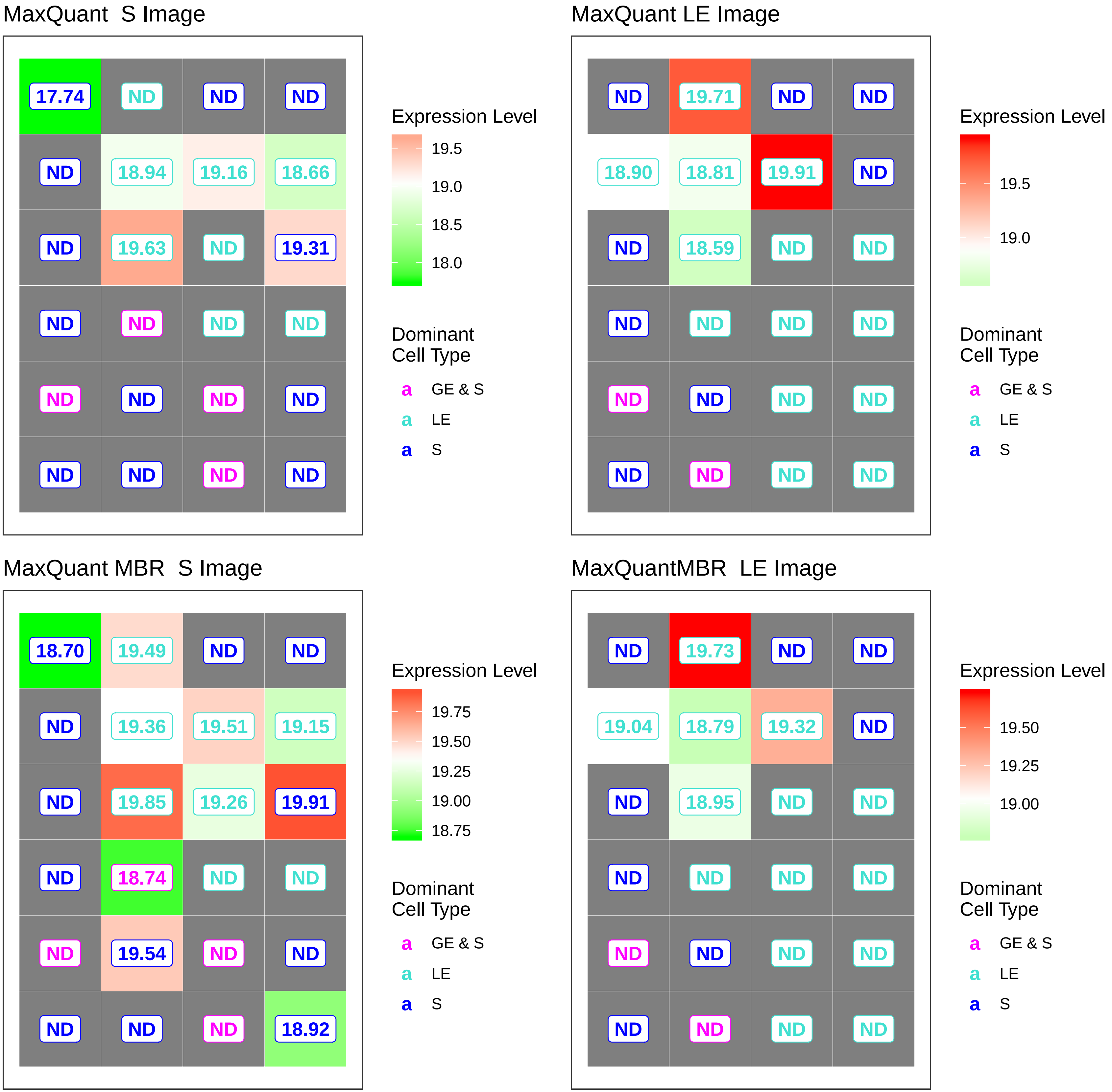

MaxQuant S Image
17.74 ND ND ND
ND 18.94 19.16 18.66
ND 19.63 ND 19.31
ND ND ND ND
ND ND ND ND
ND ND ND ND
Expression Level
19.5
19.0
18.5
18.0
Dominant Cell Type
a GE & S
a LE
a S
MaxQuant LE Image
ND 19.71 ND ND
18.90 18.81 19.91 ND
ND 18.59 ND ND
ND ND ND ND
ND ND ND ND
ND ND ND ND
Expression Level
19.5
19.0
Dominant Cell Type
a GE & S
a LE
a S
MaxQuant MBR S Image
18.70 19.49 ND ND
ND 19.36 19.51 19.15
ND 19.85 19.26 19.91
ND 18.74 ND ND
ND 19.54 ND ND
ND ND ND 18.92
Expression Level
19.75
19.50
19.25
19.00
18.75
Dominant Cell Type
a GE & S
a LE
a S
MaxQuantMBR LE Image
ND 19.73 ND ND
19.04 18.79 19.32 ND
ND 18.95 ND ND
ND ND ND ND
ND ND ND ND
ND ND ND ND
Expression Level
19.50
19.25
19.00
Dominant Cell Type
a GE & S
a LE
a S

LIMA1_MOUSE

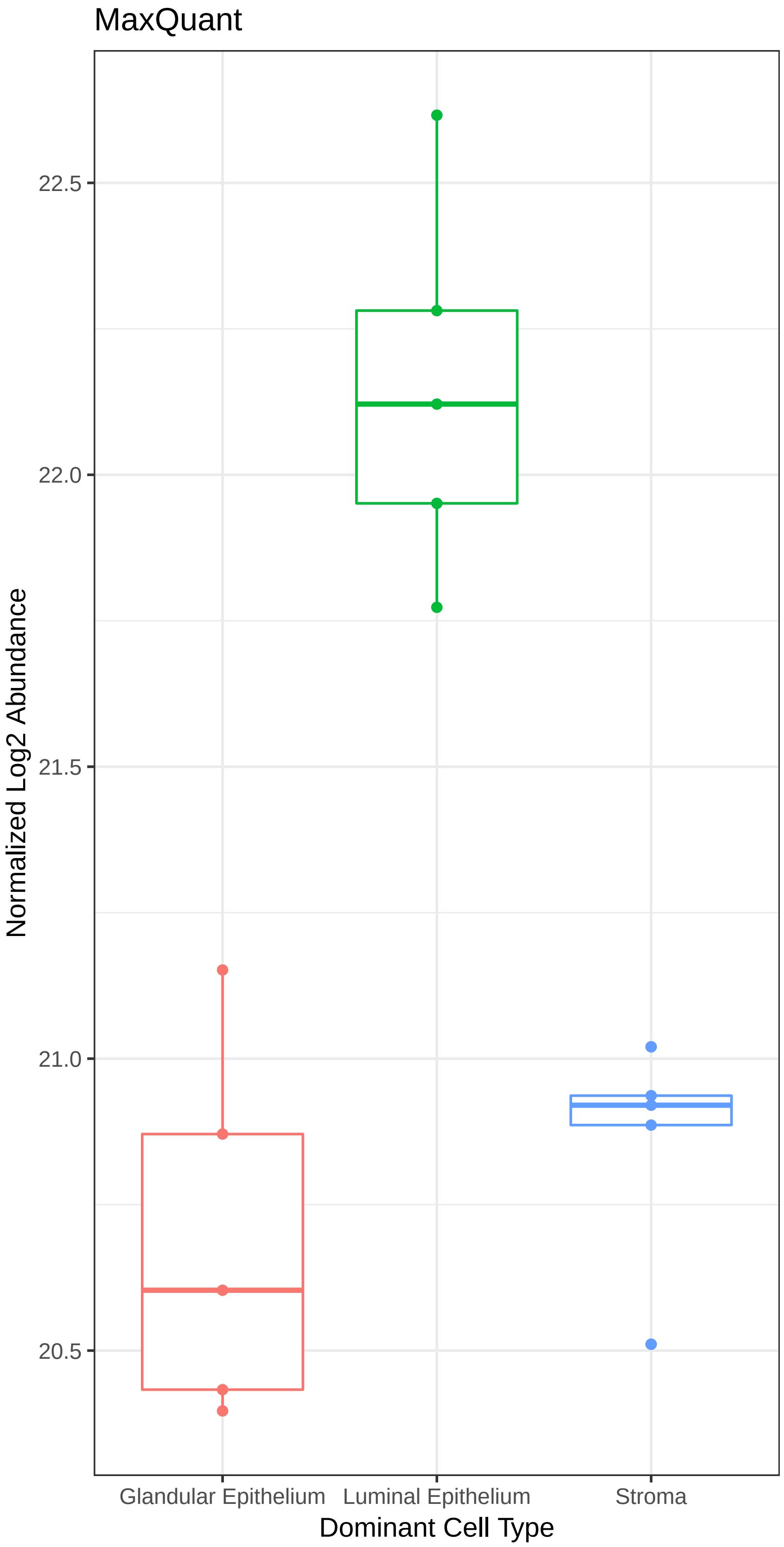

MaxQuant
Normalized Log2 Abundance
22.5
22.0
21.5
21.0
20.5
Glandular Epithelium
Luminal Epithelium
Stroma
Dominant Cell Type

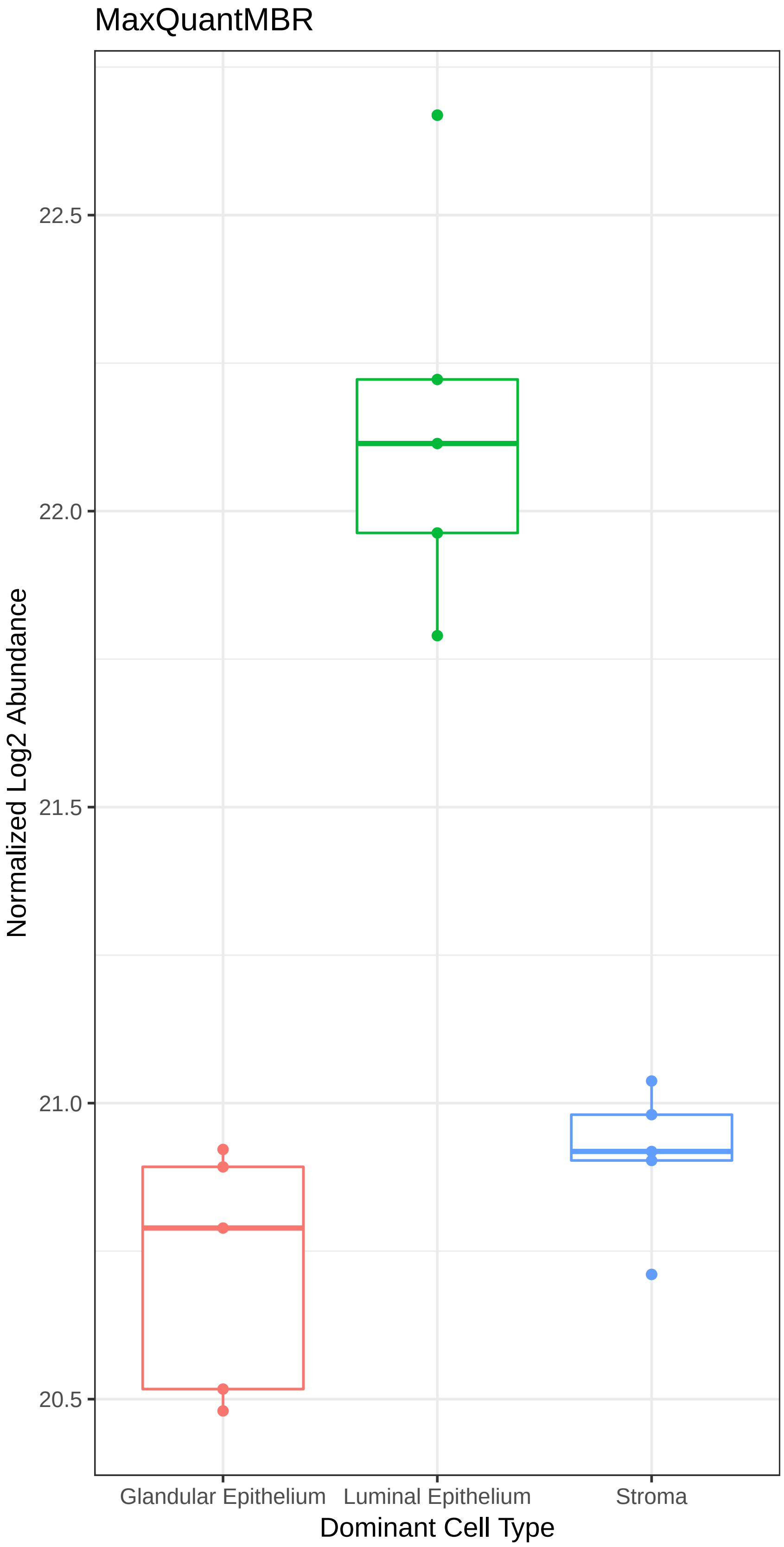

MaxQuantMBR
Normalized Log2 Abundance
22.5
22.0
21.5
21.0
20.5
Glandular Epithelium
Luminal Epithelium
Stroma
Dominant Cell Type

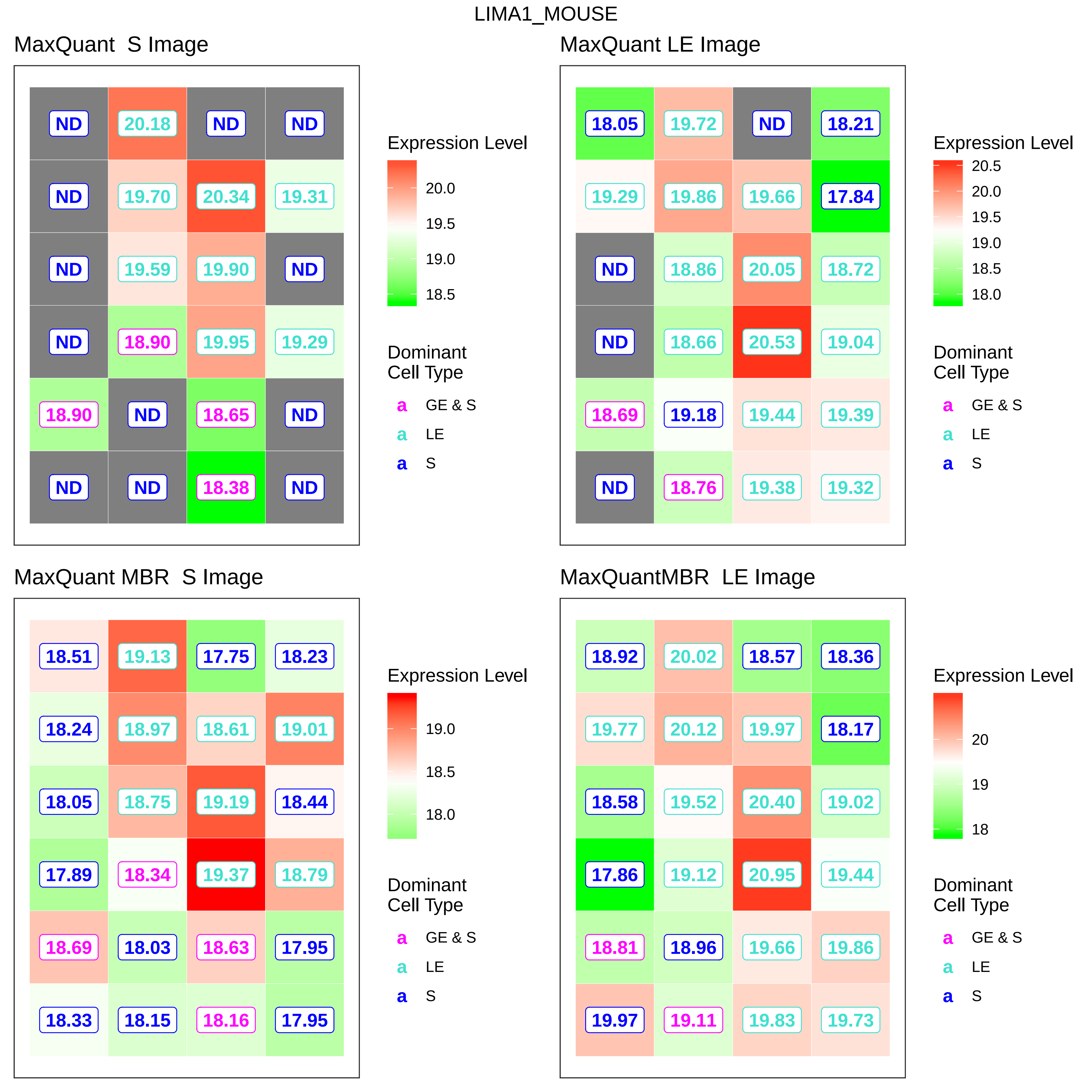
LIMA1_MOUSE
MaxQuant S Image
ND 20.18 ND ND
ND 19.70 20.34 19.31
ND 19.59 19.90 ND
ND 18.90 19.95 19.29
18.90 ND 18.65 ND
ND ND 18.38 ND
Expression Level
20.0
19.5
19.0
18.5
Dominant Cell Type
a GE & S
a LE
a S
MaxQuant LE Image
18.05 19.72 ND 18.21
19.29 19.86 19.66 17.84
ND 18.86 20.05 18.72
ND 18.66 20.53 19.04
18.69 19.18 19.44 19.39
ND 18.76 19.38 19.32
Expression Level
20.5
20.0
19.5
19.0
18.5
18.0
Dominant Cell Type
a GE & S
a LE
a S
MaxQuant MBR S Image
18.51 19.13 17.75 18.23
18.24 18.97 18.61 19.01
18.05 18.75 19.19 18.44
17.89 18.34 19.37 18.79
18.69 18.03 18.63 17.95
18.33 18.15 18.16 17.95
Expression Level
19.0
18.5
18.0
Dominant Cell Type
a GE & S
a LE
a S
MaxQuantMBR LE Image
18.92 20.02 18.57 18.36
19.77 20.12 19.97 18.17
18.58 19.52 20.40 19.02
17.86 19.12 20.95 19.44
18.81 18.96 19.66 19.86
19.97 19.11 19.83 19.73
Expression Level
20
19
18
Dominant Cell Type
a GE & S
a LE
a S

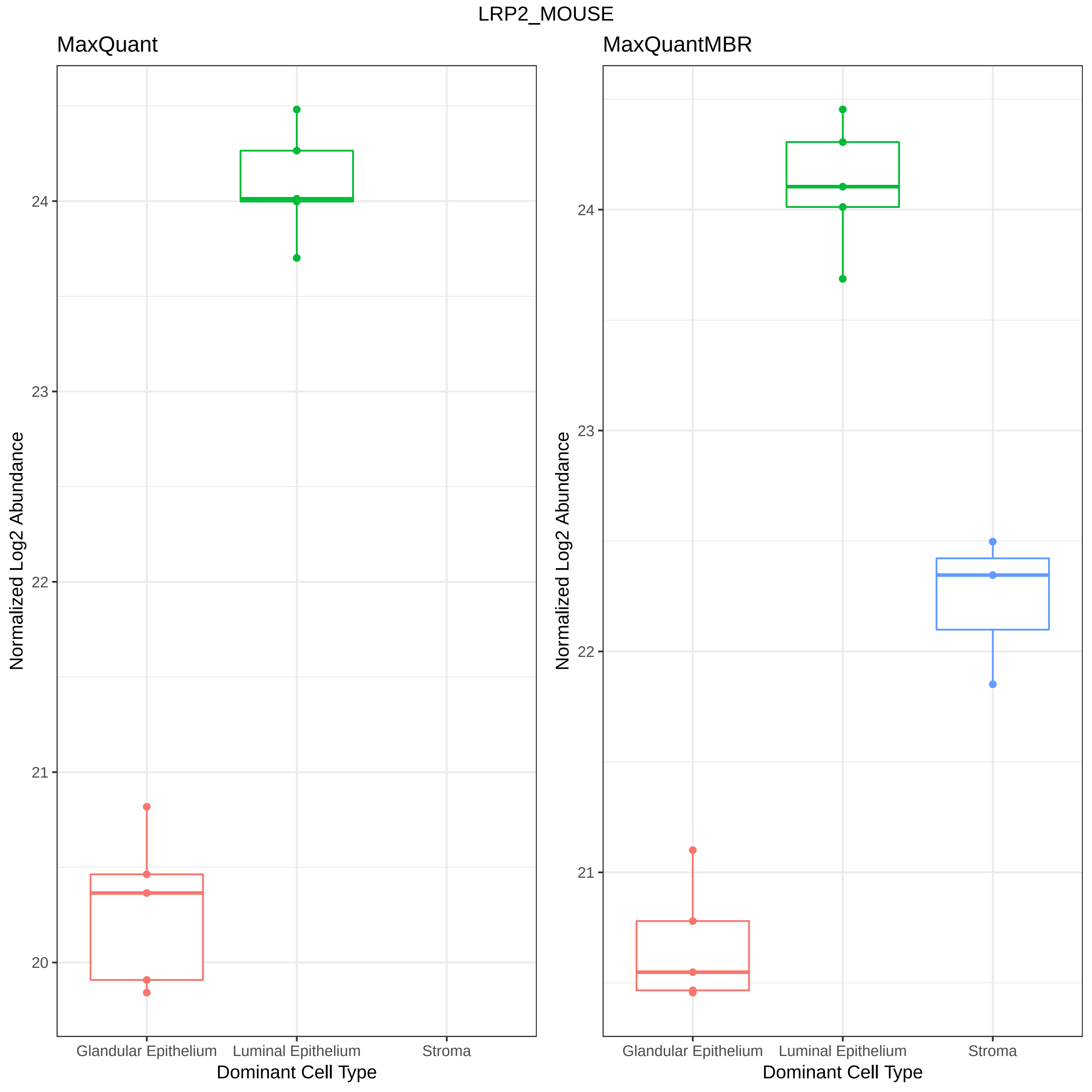

LRP2_MOUSE
MaxQuant
MaxQuantMBR
Normalized Log2 Abundance
24
23
22
21
20
Glandular Epithelium
Luminal Epithelium
Stroma
Dominant Cell Type

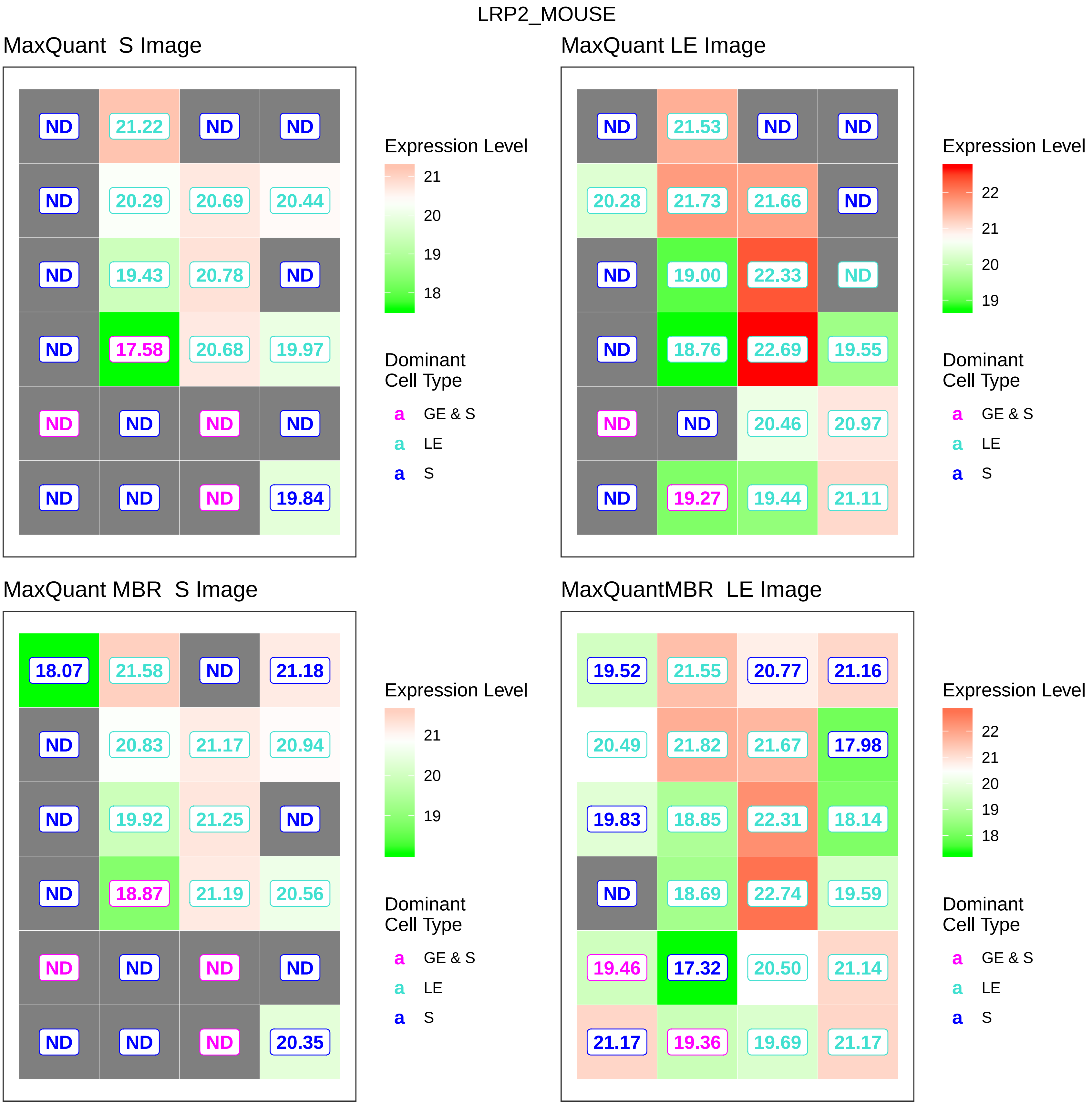
LRP2_MOUSE
MaxQuant S Image
ND 21.22 ND ND
ND 20.29 20.69 20.44
ND 19.43 20.78 ND
ND 17.58 20.68 19.97
ND ND ND ND
ND ND ND 19.84
Expression Level
21
20
19
18
Dominant Cell Type
a GE & S
a LE
a S
MaxQuant LE Image
ND 21.53 ND ND
20.28 21.73 21.66 ND
ND 19.00 22.33 ND
ND 18.76 22.69 19.55
ND ND 20.46 20.97
ND 19.27 19.44 21.11
Expression Level
22
21
20
19
Dominant Cell Type
a GE & S
a LE
a S
MaxQuant MBR S Image
18.07 21.58 ND 21.18
ND 20.83 21.17 20.94
ND 19.92 21.25 ND
ND 18.87 21.19 20.56
ND ND ND ND
ND ND ND 20.35
Expression Level
21
20
19
Dominant Cell Type
a GE & S
a LE
a S
MaxQuantMBR LE Image
19.52 21.55 20.77 21.16
20.49 21.82 21.67 17.98
19.83 18.85 22.31 18.14
ND 18.69 22.74 19.59
19.46 17.32 20.50 21.14
21.17 19.36 19.69 21.17
Expression Level
22
21
20
19
18
Dominant Cell Type
a GE & S
a LE
a S

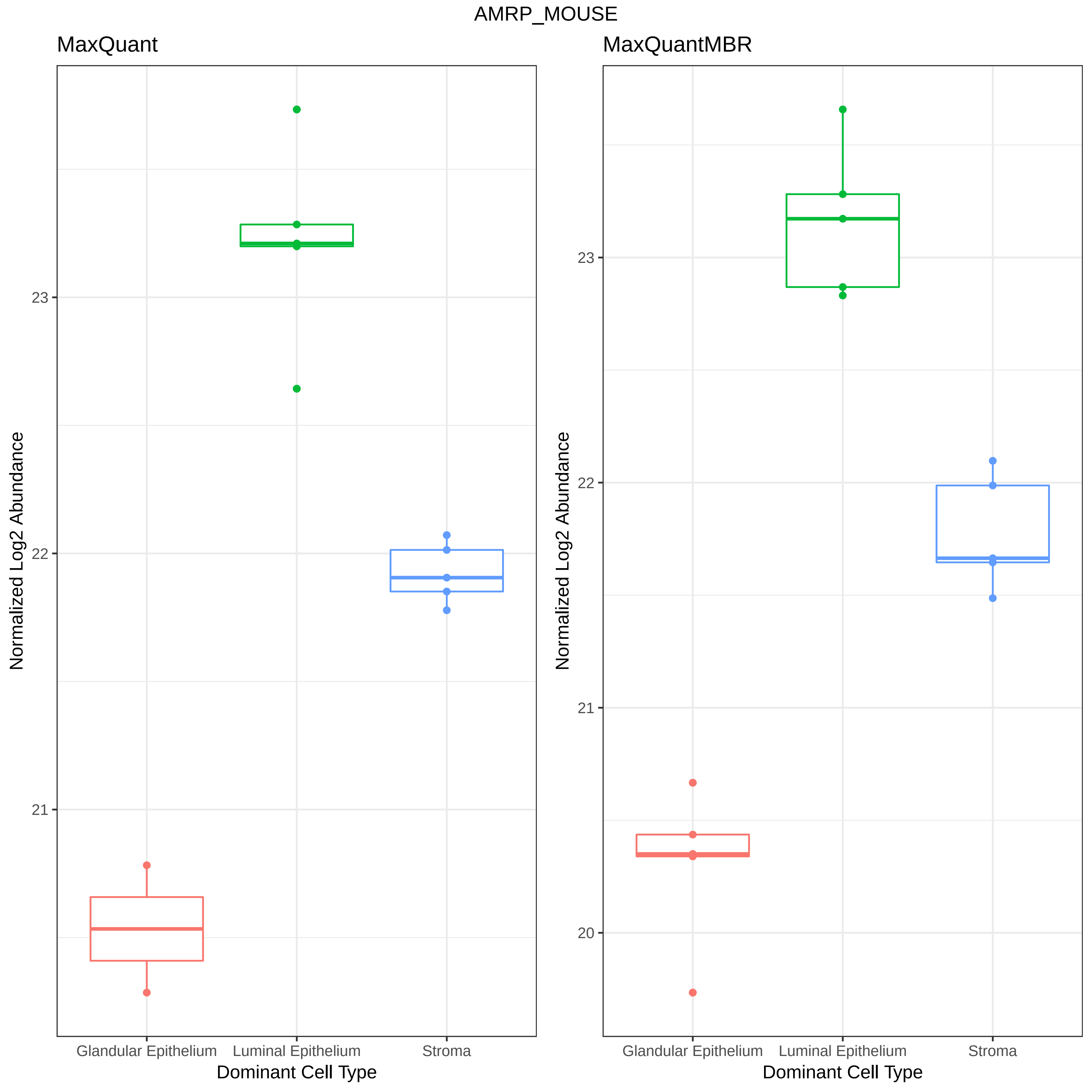

AMRP_MOUSE
MaxQuant
MaxQuantMBR
Normalized Log2 Abundance
23
22
21
20
Glandular Epithelium
Luminal Epithelium
Stroma
Dominant Cell Type

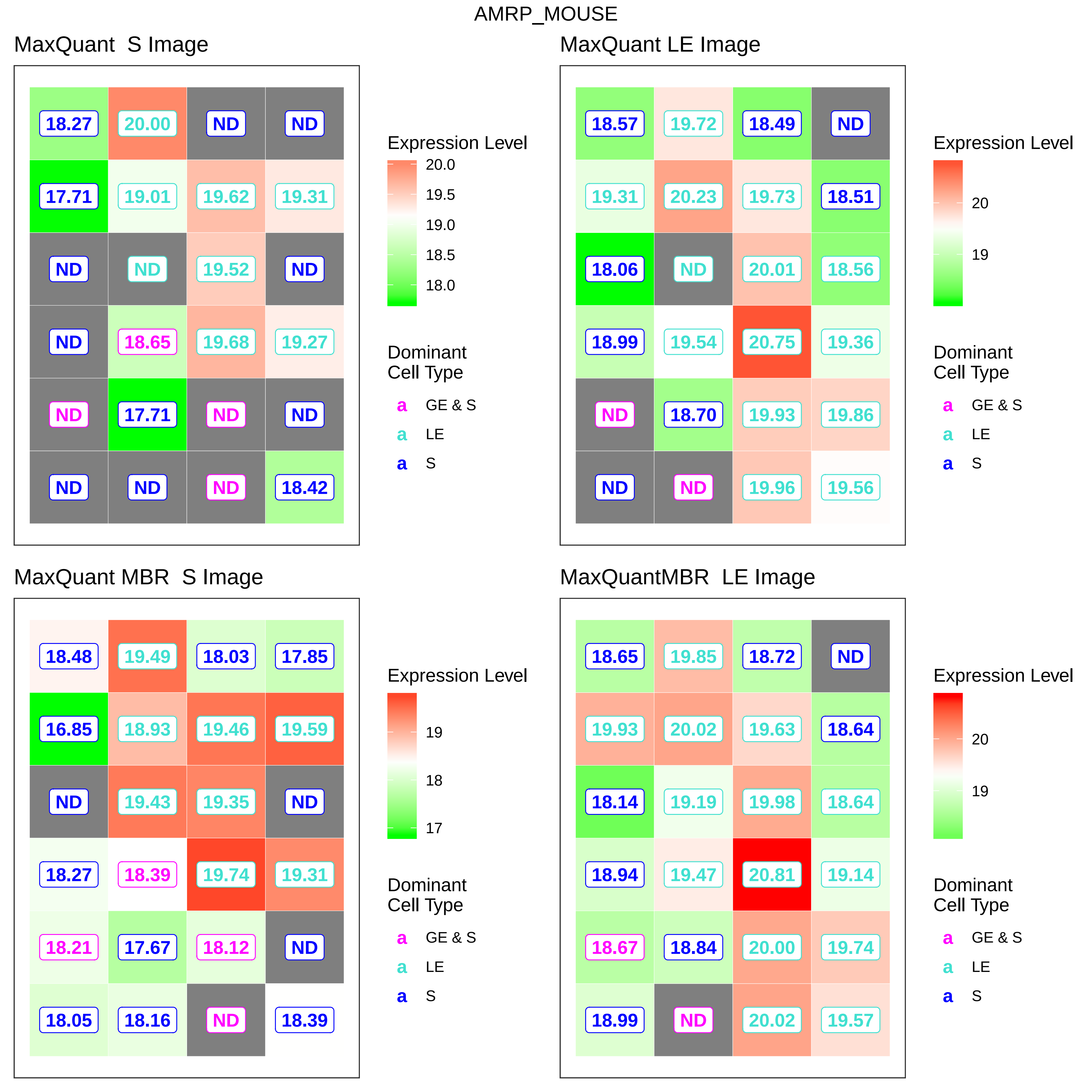

AMRP_MOUSE
MaxQuant S Image
18.27 20.00 ND ND
17.71 19.01 19.62 19.31
ND ND 19.52 ND
ND 18.65 19.68 19.27
ND 17.71 ND ND
ND ND ND 18.42
Expression Level
20.0
19.5
19.0
18.5
18.0
Dominant Cell Type
a GE & S
a LE
a S
MaxQuant LE Image
18.57 19.72 18.49 ND
19.31 20.23 19.73 18.51
18.06 ND 20.01 18.56
18.99 19.54 20.75 19.36
ND 18.70 19.93 19.86
ND ND 19.96 19.56
Expression Level
20
19
Dominant Cell Type
a GE & S
a LE
a S
MaxQuant MBR S Image
18.48 19.49 18.03 17.85
16.85 18.93 19.46 19.59
ND 19.43 19.35 ND
18.27 18.39 19.74 19.31
18.21 17.67 18.12 ND
18.05 18.16 ND 18.39
Expression Level
19
18
17
Dominant Cell Type
a GE & S
a LE
a S
MaxQuantMBR LE Image
18.65 19.85 18.72 ND
19.93 20.02 19.63 18.64
18.14 19.19 19.98 18.64
18.94 19.47 20.81 19.14
18.67 18.84 20.00 19.74
18.99 ND 20.02 19.57
Expression Level
20
19
Dominant Cell Type
a GE & S
a LE
a S

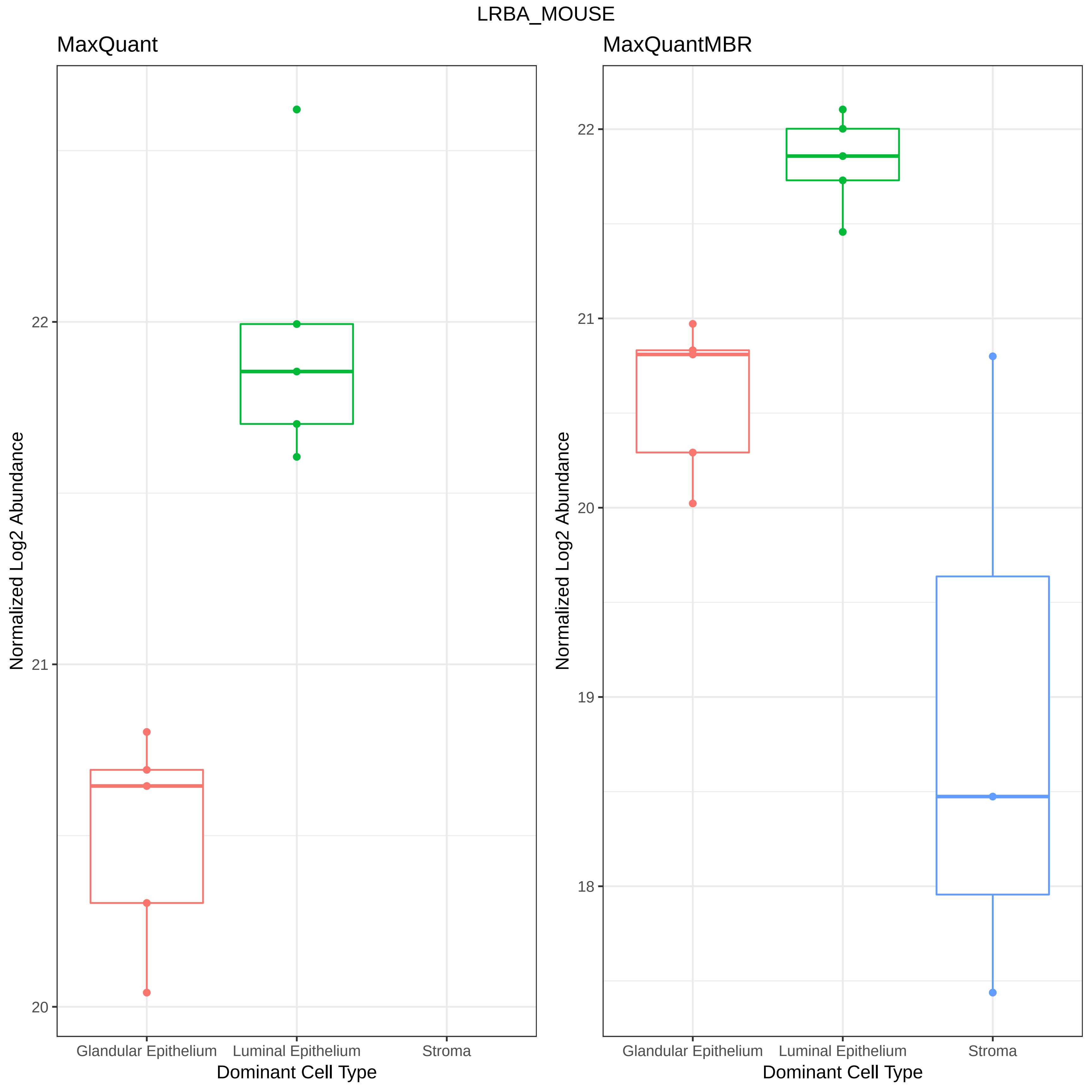

LRBA_MOUSE
MaxQuant
MaxQuantMBR
Normalized Log2 Abundance
22
21
20
19
18
Glandular Epithelium
Luminal Epithelium
Stroma
Dominant Cell Type

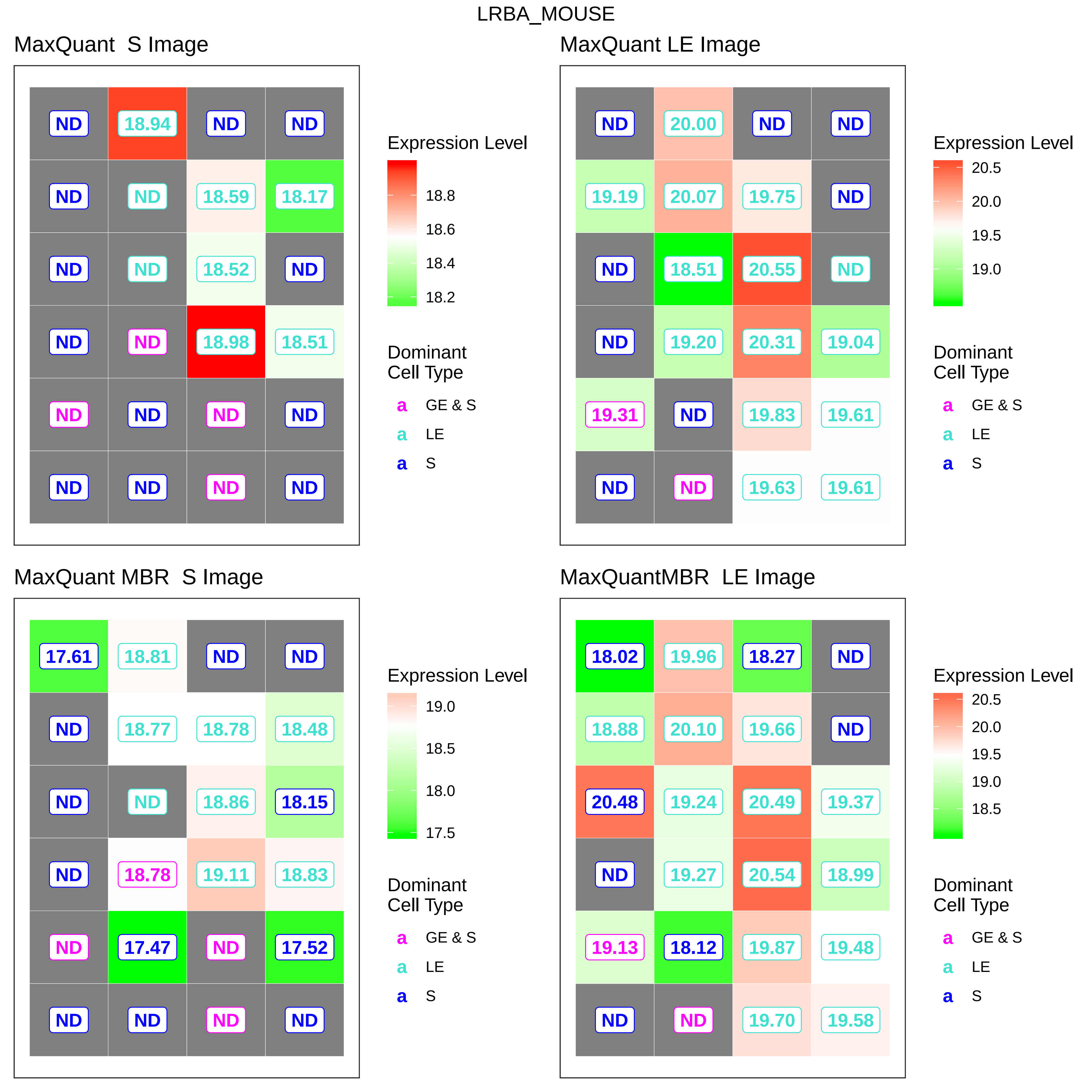
LRBA_MOUSE
MaxQuant S Image
ND 18.94 ND ND
ND ND 18.59 18.17
ND ND 18.52 ND
ND ND 18.98 18.51
ND ND ND ND
ND ND ND ND
Expression Level
18.8
18.6
18.4
18.2
Dominant Cell Type
a GE & S
a LE
a S
MaxQuant LE Image
ND 20.00 ND ND
19.19 20.07 19.75 ND
ND 18.51 20.55 ND
ND 19.20 20.31 19.04
19.31 ND 19.83 19.61
ND ND 19.63 19.61
Expression Level
20.5
20.0
19.5
19.0
Dominant Cell Type
a GE & S
a LE
a S
MaxQuant MBR S Image
17.61 18.81 ND ND
ND 18.77 18.78 18.48
ND ND 18.86 18.15
ND 18.78 19.11 18.83
ND 17.47 ND 17.52
ND ND ND ND
Expression Level
19.0
18.5
18.0
17.5
Dominant Cell Type
a GE & S
a LE
a S
MaxQuantMBR LE Image
18.02 19.96 18.27 ND
18.88 20.10 19.66 ND
20.48 19.24 20.49 19.37
ND 19.27 20.54 18.99
19.13 18.12 19.87 19.48
ND ND 19.70 19.58
Expression Level
20.5
20.0
19.5
19.0
18.5
Dominant Cell Type
a GE & S
a LE
a S

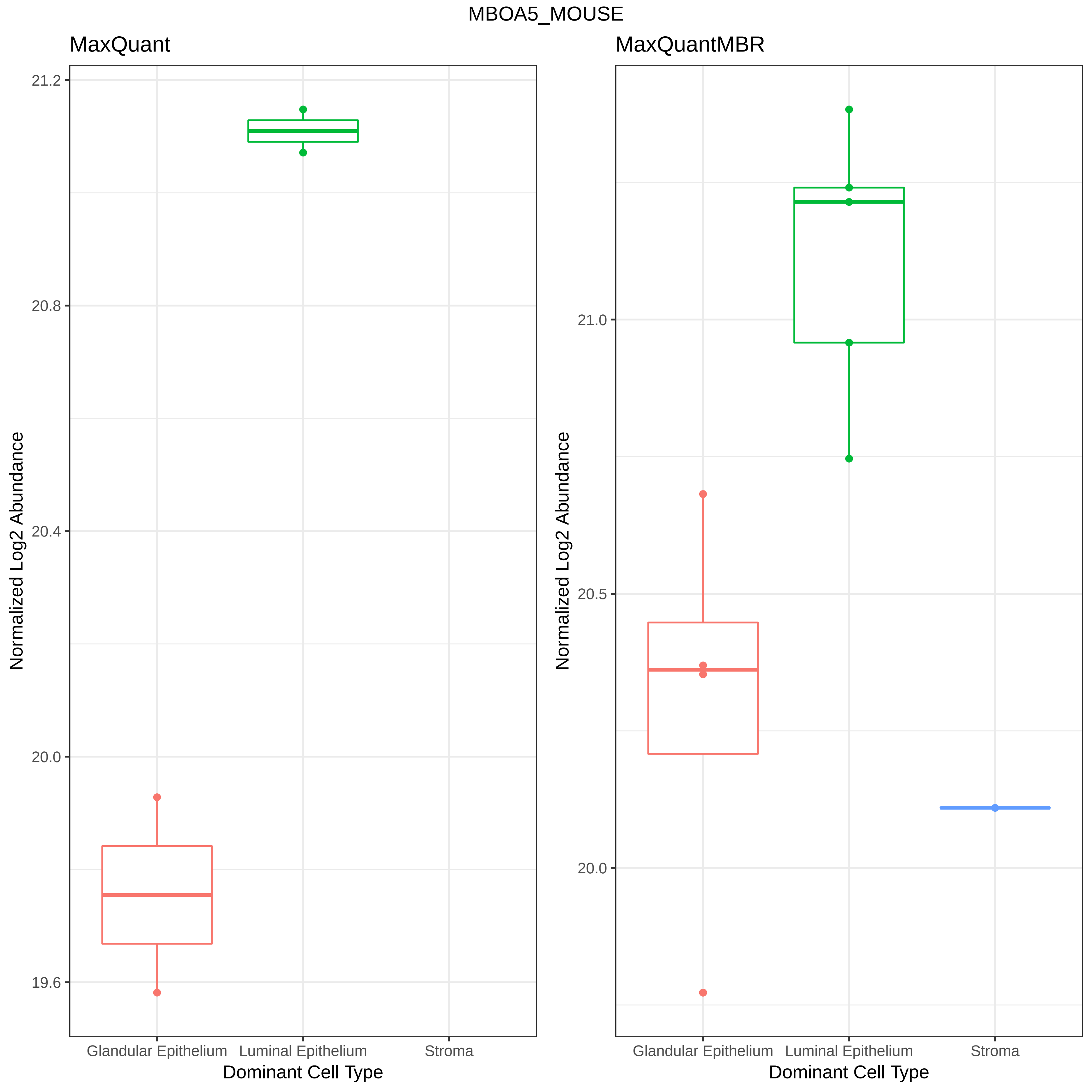

MBOA5_MOUSE
MaxQuant
MaxQuantMBR
21.2
20.8
20.4
20.0
19.6
21.0
20.5
20.0
Normalized Log2 Abundance
Glandular Epithelium
Luminal Epithelium
Stroma
Dominant Cell Type

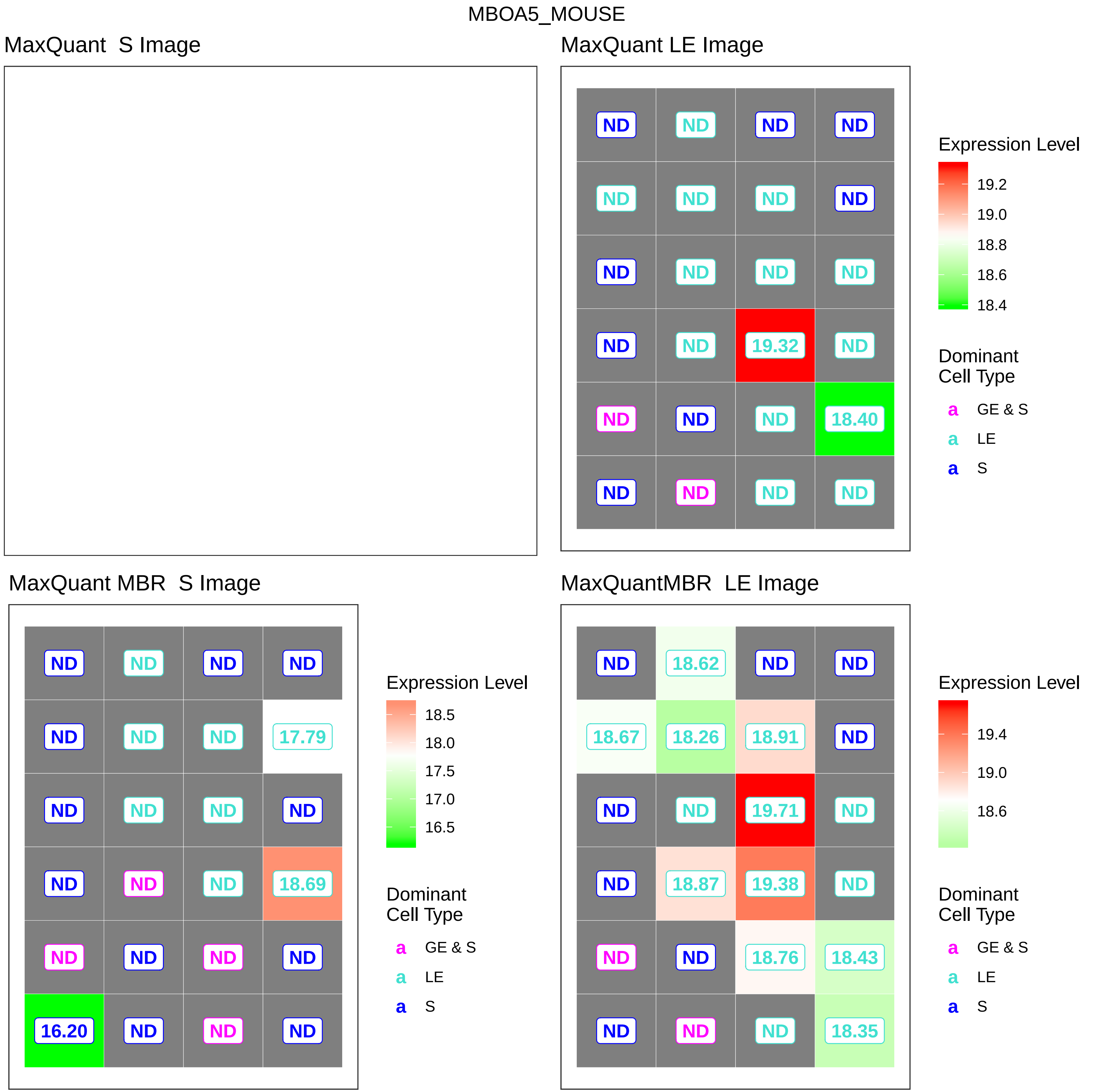
MBOA5_MOUSE
MaxQuant S Image
MaxQuant LE Image
ND ND ND ND
ND ND ND ND
ND ND ND ND
ND ND 19.32 ND
ND ND ND 18.40
ND ND ND ND
Expression Level
19.2
19.0
18.8
18.6
18.4
Dominant Cell Type
a GE & S
a LE
a S
MaxQuant MBR S Image
ND ND ND ND
ND ND ND 17.79
ND ND ND ND
ND ND ND 18.69
ND ND ND ND
16.20 ND ND ND
Expression Level
18.5
18.0
17.5
17.0
16.5
Dominant Cell Type
a GE & S
a LE
a S
MaxQuantMBR LE Image
ND 18.62 ND ND
18.67 18.26 18.91 ND
ND ND 19.71 ND
ND 18.87 19.38 ND
ND ND 18.76 18.43
ND ND ND 18.35
Expression Level
19.4
19.0
18.6
Dominant Cell Type
a GE & S
a LE
a S

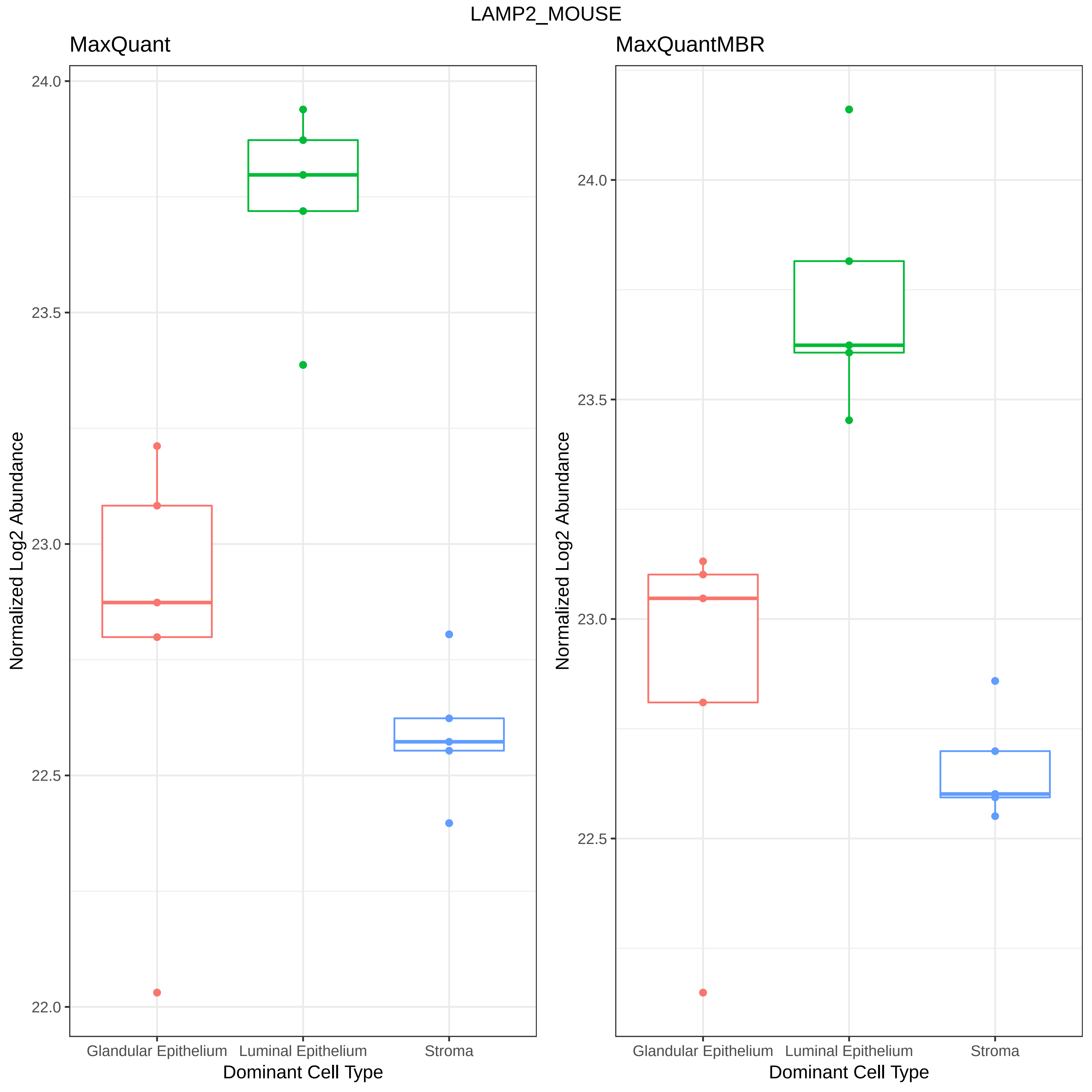

LAMP2_MOUSE
MaxQuant
MaxQuantMBR
Normalized Log2 Abundance
24.0
23.5
23.0
22.5
22.0
Glandular Epithelium
Luminal Epithelium
Stroma
Dominant Cell Type

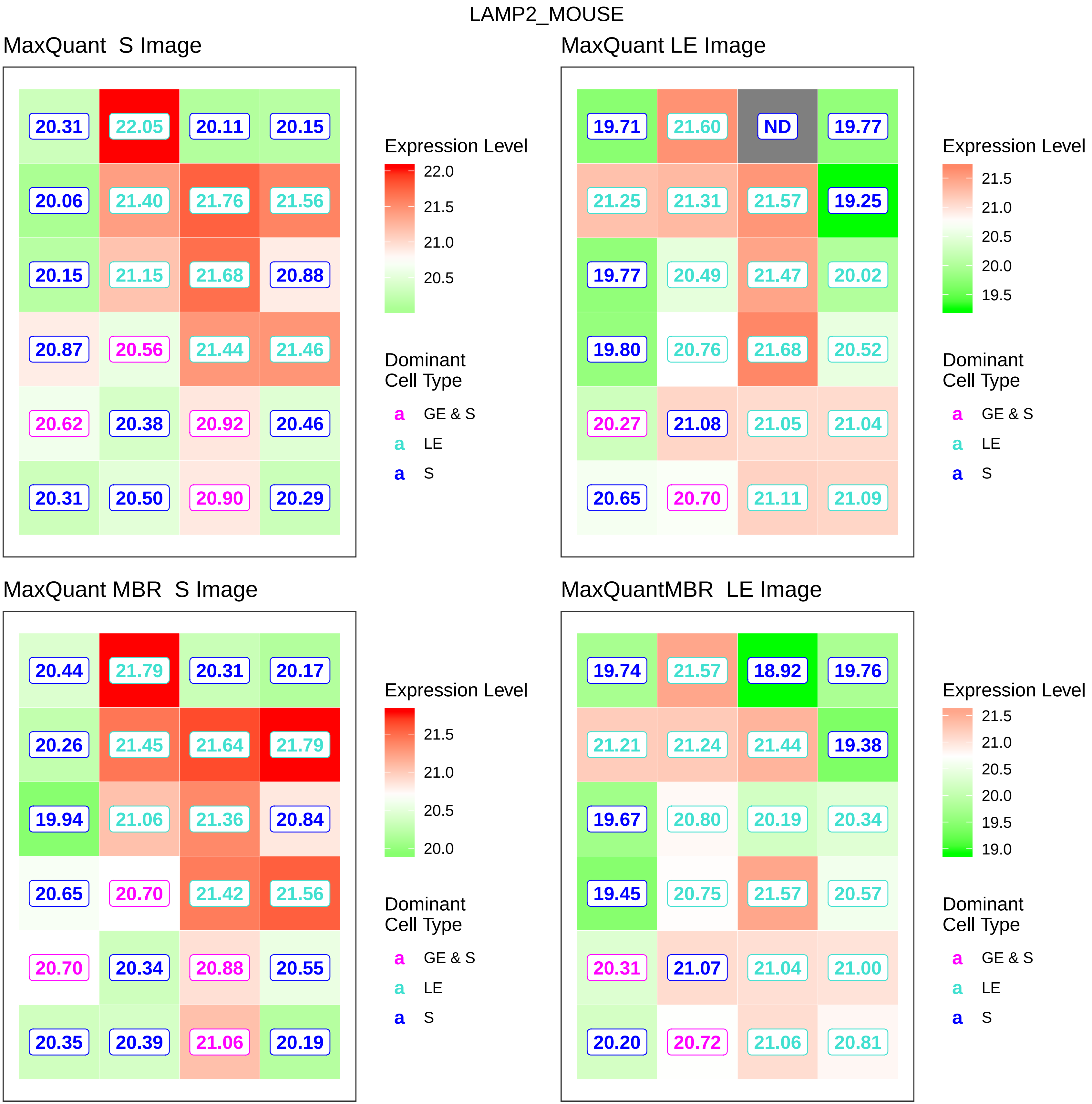
LAMP2_MOUSE
MaxQuant S Image
20.31 22.05 20.11 20.15
20.06 21.40 21.76 21.56
20.15 21.15 21.68 20.88
20.87 20.56 21.44 21.46
20.62 20.38 20.92 20.46
20.31 20.50 20.90 20.29
Expression Level
22.0
21.5
21.0
20.5
Dominant Cell Type
a GE & S
a LE
a S
MaxQuant LE Image
19.71 21.60 ND 19.77
21.25 21.31 21.57 19.25
19.77 20.49 21.47 20.02
19.80 20.76 21.68 20.52
20.27 21.08 21.05 21.04
20.65 20.70 21.11 21.09
Expression Level
21.5
21.0
20.5
20.0
19.5
Dominant Cell Type
a GE & S
a LE
a S
MaxQuant MBR S Image
20.44 21.79 20.31 20.17
20.26 21.45 21.64 21.79
19.94 21.06 21.36 20.84
20.65 20.70 21.42 21.56
20.70 20.34 20.88 20.55
20.35 20.39 21.06 20.19
Expression Level
21.5
21.0
20.5
20.0
Dominant Cell Type
a GE & S
a LE
a S
MaxQuantMBR LE Image
19.74 21.57 18.92 19.76
21.21 21.24 21.44 19.38
19.67 20.80 20.19 20.34
19.45 20.75 21.57 20.57
20.31 21.07 21.04 21.00
20.20 20.72 21.06 20.81
Expression Level
21.5
21.0
20.5
20.0
19.5
19.0
Dominant Cell Type
a GE & S
a LE
a S

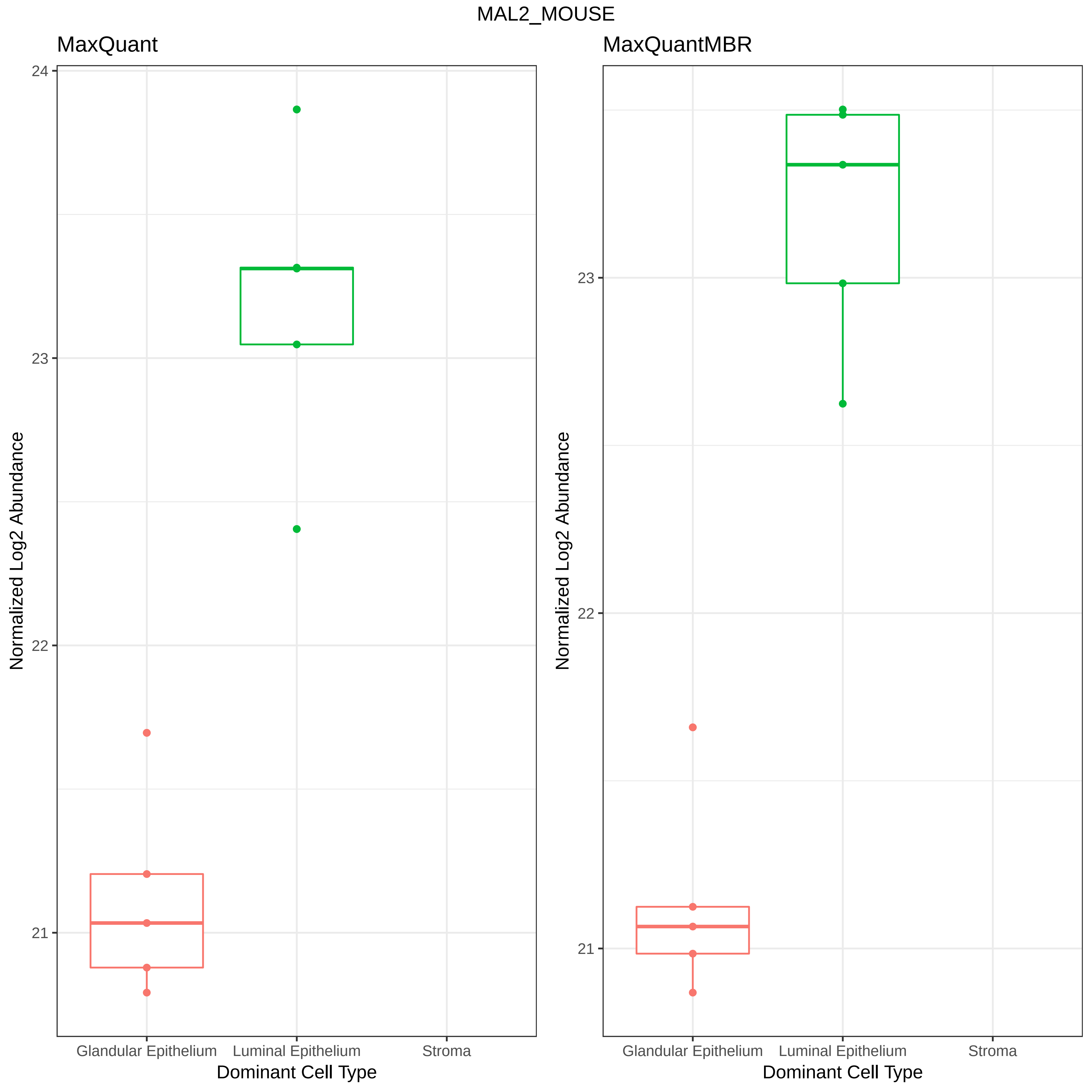
MAL2_MOUSE
MaxQuant
MaxQuantMBR
Normalized Log2 Abundance
24
23
22
21
Glandular Epithelium
Luminal Epithelium
Stroma
Dominant Cell Type

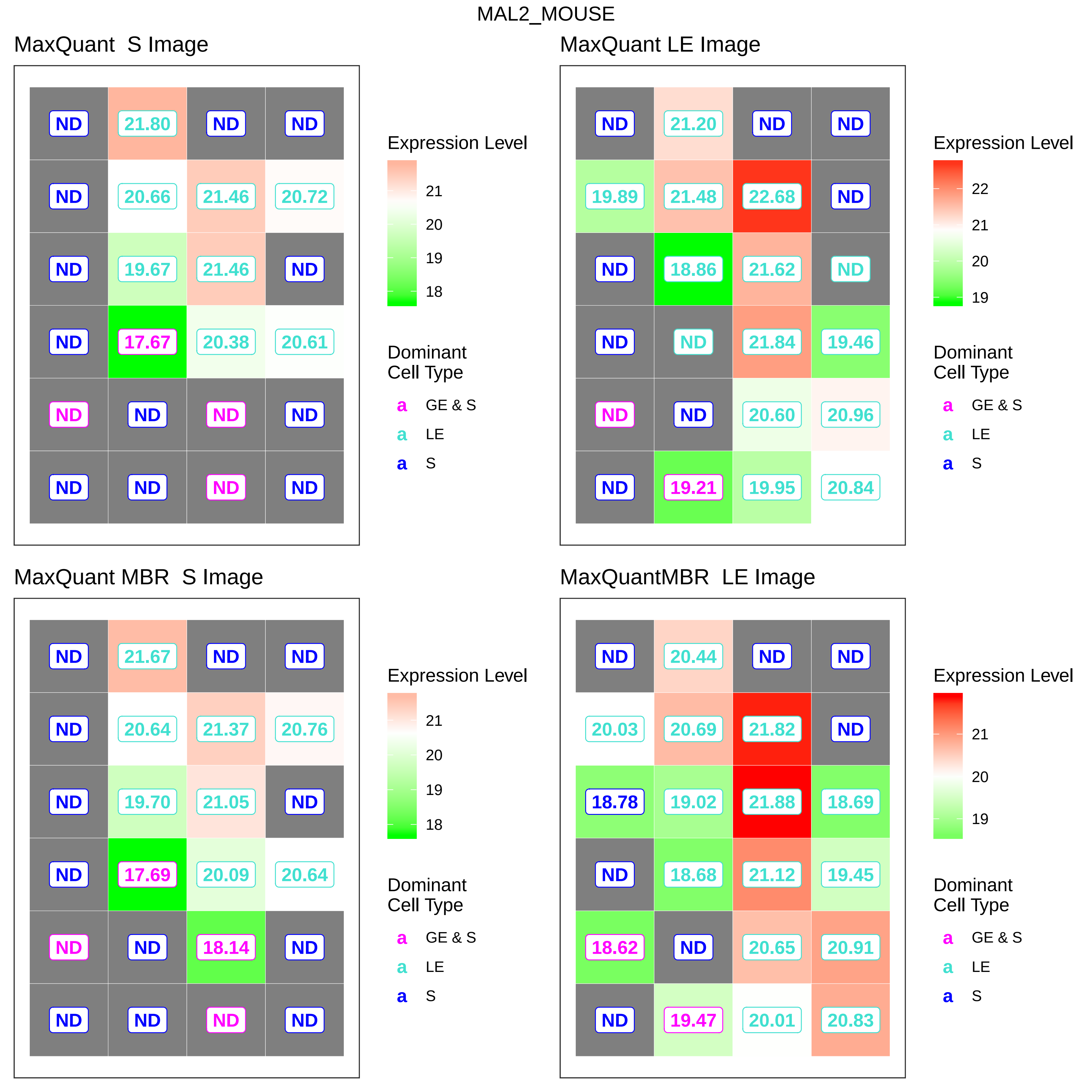
MAL2_MOUSE
MaxQuant S Image
ND
21.80
ND
ND
ND
20.66
21.46
20.72
ND
19.67
21.46
ND
ND
17.67
20.38
20.61
ND
ND
ND
ND
ND
ND
ND
ND
Expression Level
21
20
19
18
Dominant Cell Type
a GE & S
a LE
a S
MaxQuant LE Image
ND
21.20
ND
ND
19.89
21.48
22.68
ND
ND
18.86
21.62
ND
ND
ND
21.84
19.46
ND
ND
20.60
20.96
ND
19.21
19.95
20.84
Expression Level
22
21
20
19
Dominant Cell Type
a GE & S
a LE
a S
MaxQuant MBR S Image
ND
21.67
ND
ND
ND
20.64
21.37
20.76
ND
19.70
21.05
ND
ND
17.69
20.09
20.64
ND
ND
18.14
ND
ND
ND
ND
ND
Expression Level
21
20
19
18
Dominant Cell Type
a GE & S
a LE
a S
MaxQuantMBR LE Image
ND
20.44
ND
ND
20.03
20.69
21.82
ND
18.78
19.02
21.88
18.69
ND
18.68
21.12
19.45
18.62
ND
20.65
20.91
ND
19.47
20.01
20.83
Expression Level
21
20
19
Dominant Cell Type
a GE & S
a LE
a S

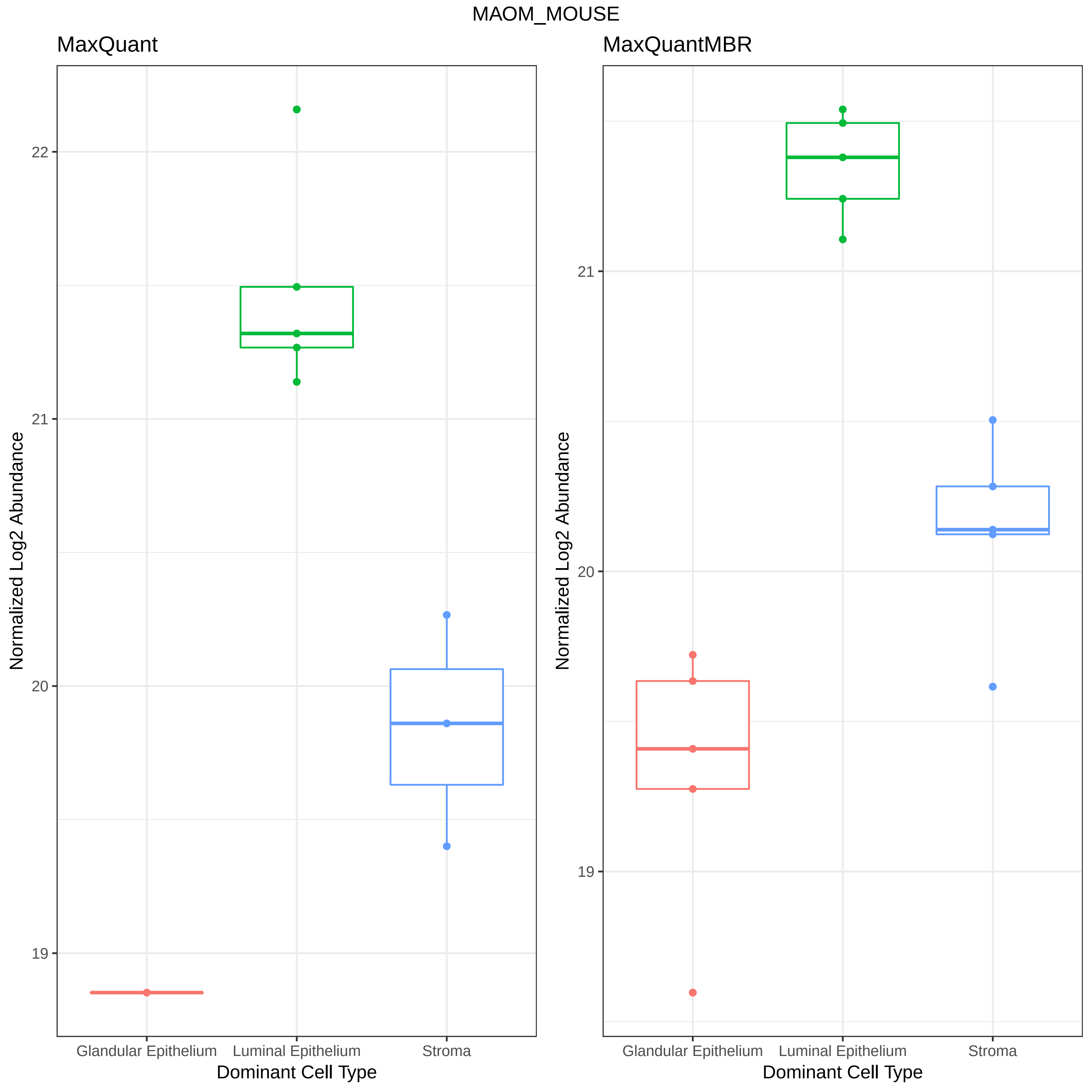

MAOM_MOUSE
MaxQuant
MaxQuantMBR
Normalized Log2 Abundance
22
21
20
19
Glandular Epithelium
Luminal Epithelium
Stroma
Dominant Cell Type

MAOM_MOUSE

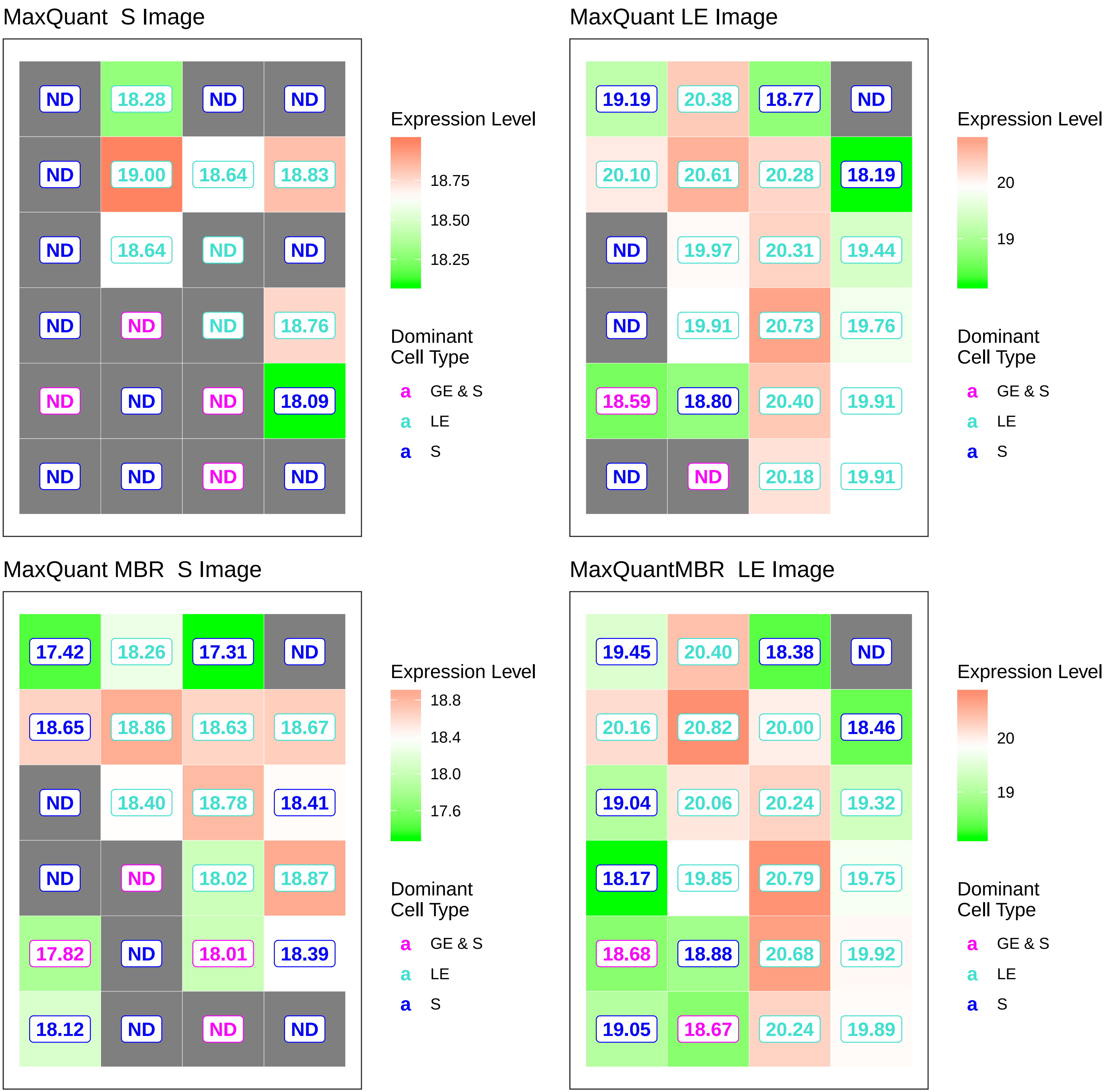

MaxQuant S Image
ND
18.28
ND
ND
ND
19.00
18.64
18.83
ND
18.64
ND
ND
ND
ND
ND
18.76
ND
ND
ND
18.09
ND
ND
ND
ND
Expression Level
18.75
18.50
18.25
Dominant Cell Type
a GE & S
a LE
a S
MaxQuant LE Image
19.19
20.38
18.77
ND
20.10
20.61
20.28
18.19
ND
19.97
20.31
19.44
ND
19.91
20.73
19.76
18.59
18.80
20.40
19.91
ND
ND
20.18
19.91
Expression Level
20
19
Dominant Cell Type
a GE & S
a LE
a S
MaxQuant MBR S Image
17.42
18.26
17.31
ND
18.65
18.86
18.63
18.67
ND
18.40
18.78
18.41
ND
ND
18.02
18.87
17.82
ND
18.01
18.39
18.12
ND
ND
ND
Expression Level
18.8
18.4
18.0
17.6
Dominant Cell Type
a GE & S
a LE
a S
MaxQuantMBR LE Image
19.45
20.40
18.38
ND
20.16
20.82
20.00
18.46
19.04
20.06
20.24
19.32
18.17
19.85
20.79
19.75
18.68
18.88
20.68
19.92
19.05
18.67
20.24
19.89
Expression Level
20
19
Dominant Cell Type
a GE & S
a LE
a S

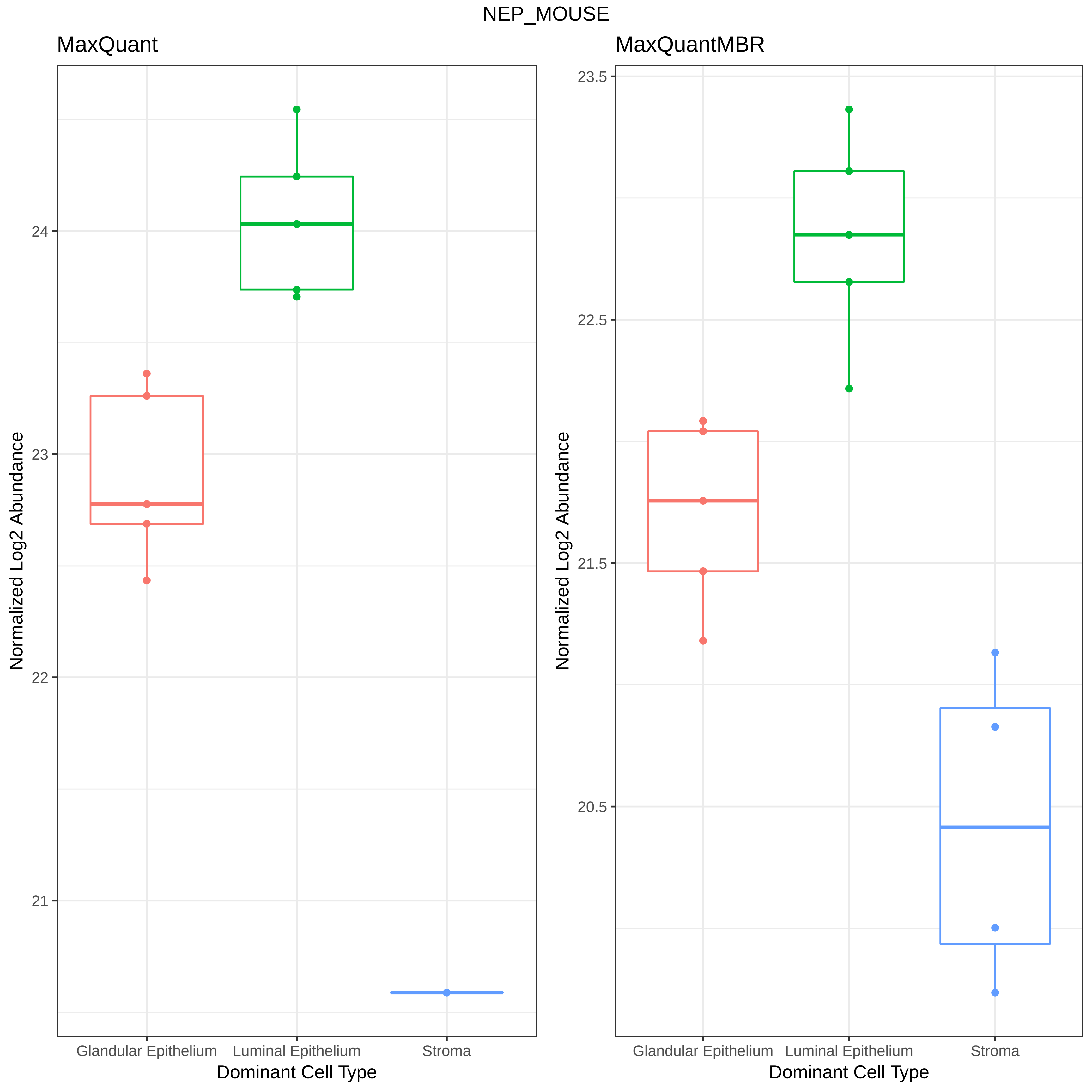

NEP_MOUSE
MaxQuant
MaxQuantMBR
Normalized Log2 Abundance
24
23
22
21
23.5
22.5
21.5
20.5
Glandular Epithelium
Luminal Epithelium
Stroma
Dominant Cell Type

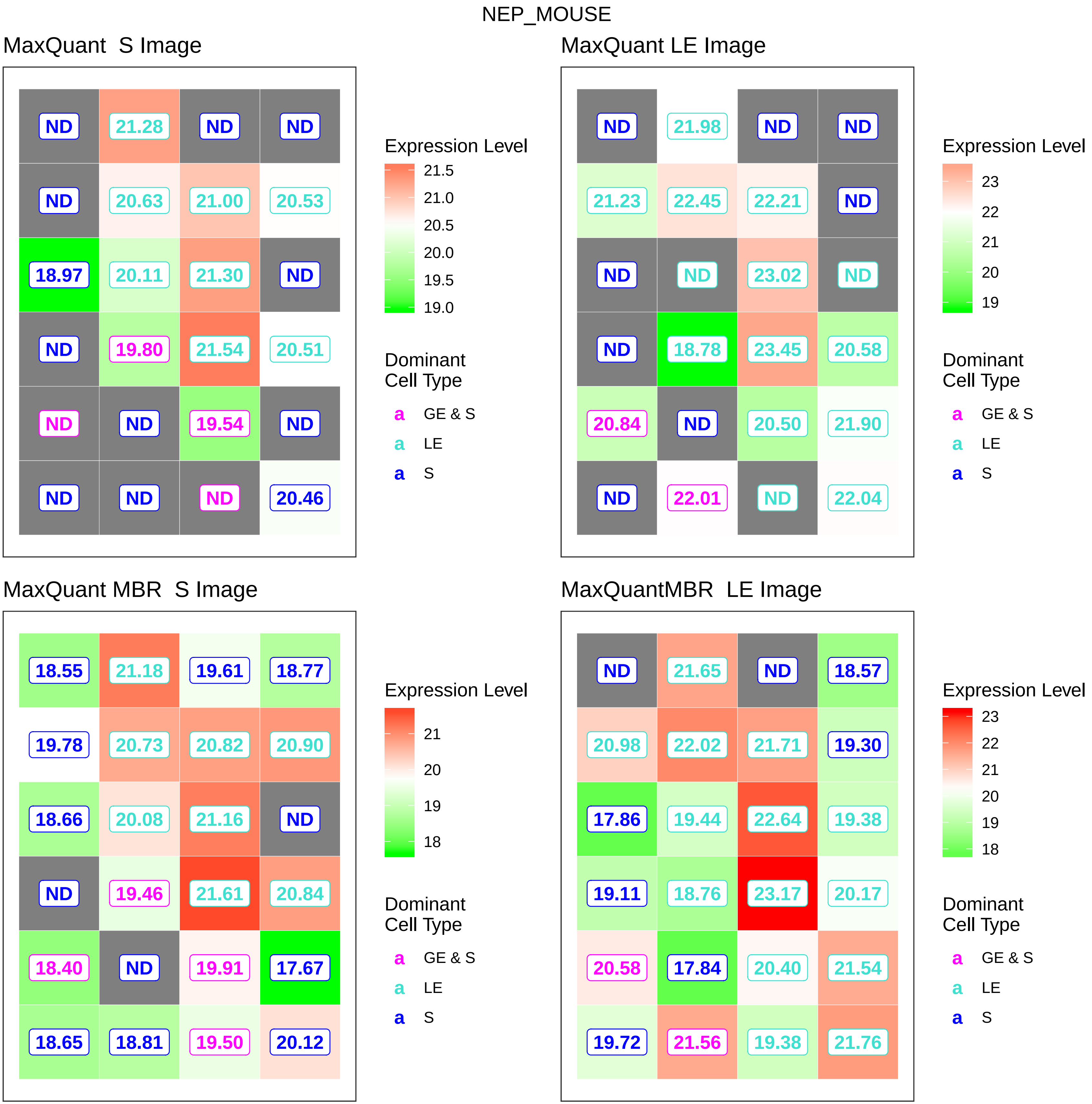
NEP_MOUSE
MaxQuant S Image
ND 21.28 ND ND
ND 20.63 21.00 20.53
18.97 20.11 21.30 ND
ND 19.80 21.54 20.51
ND ND 19.54 ND
ND ND ND 20.46
Expression Level
21.5
21.0
20.5
20.0
19.5
19.0
Dominant Cell Type
a GE & S
a LE
a S
MaxQuant LE Image
ND 21.98 ND ND
21.23 22.45 22.21 ND
ND ND 23.02 ND
ND 18.78 23.45 20.58
20.84 ND 20.50 21.90
ND 22.01 ND 22.04
Expression Level
23
22
21
20
19
Dominant Cell Type
a GE & S
a LE
a S
MaxQuant MBR S Image
18.55 21.18 19.61 18.77
19.78 20.73 20.82 20.90
18.66 20.08 21.16 ND
ND 19.46 21.61 20.84
18.40 ND 19.91 17.67
18.65 18.81 19.50 20.12
Expression Level
21
20
19
18
Dominant Cell Type
a GE & S
a LE
a S
MaxQuantMBR LE Image
ND 21.65 ND 18.57
20.98 22.02 21.71 19.30
17.86 19.44 22.64 19.38
19.11 18.76 23.17 20.17
20.58 17.84 20.40 21.54
19.72 21.56 19.38 21.76
Expression Level
23
22
21
20
19
18
Dominant Cell Type
a GE & S
a LE
a S

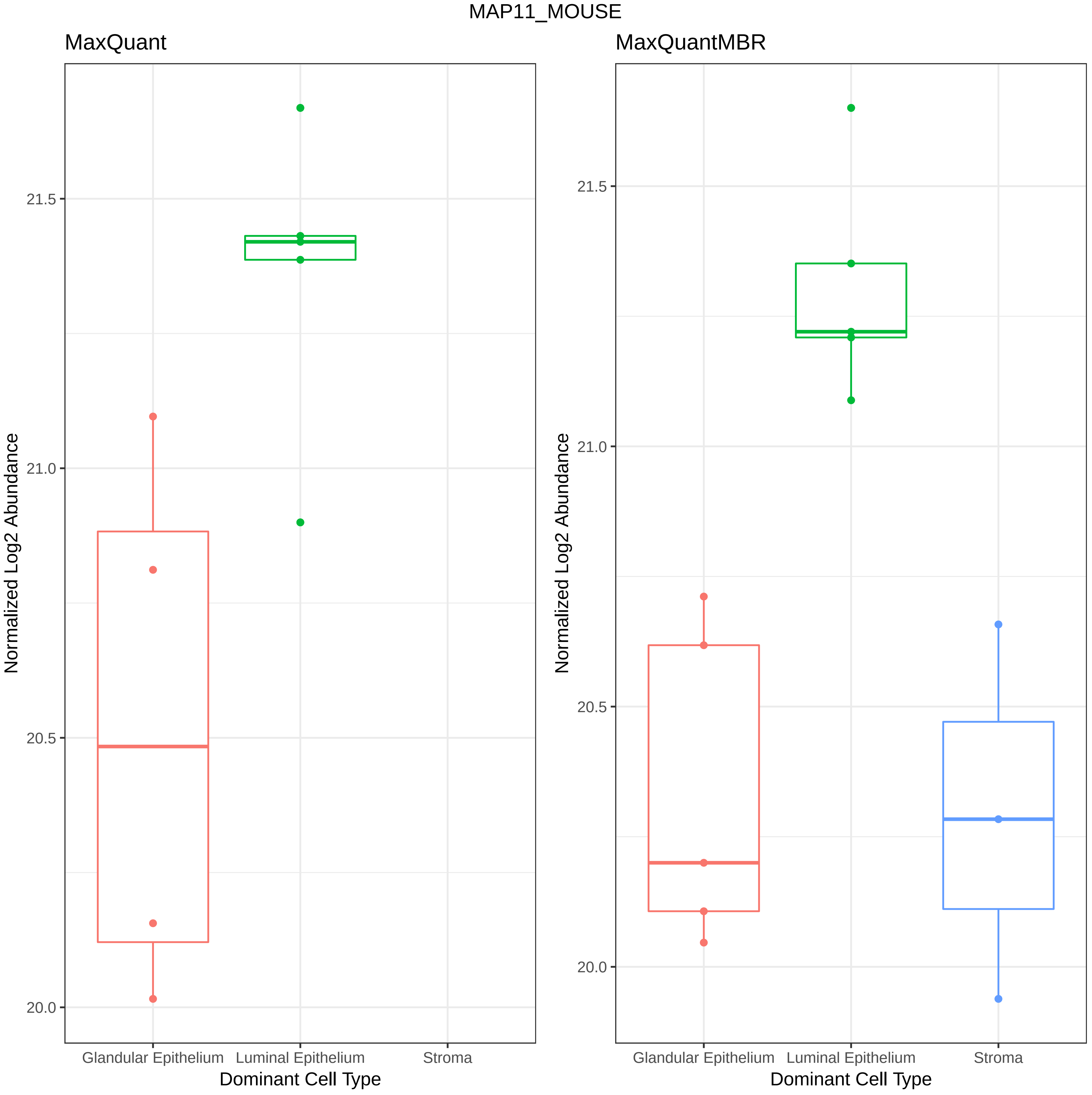

MAP11_MOUSE
MaxQuant
MaxQuantMBR
Normalized Log2 Abundance
21.5
21.0
20.5
20.0
Glandular Epithelium
Luminal Epithelium
Stroma
Dominant Cell Type

MAP11_MOUSE

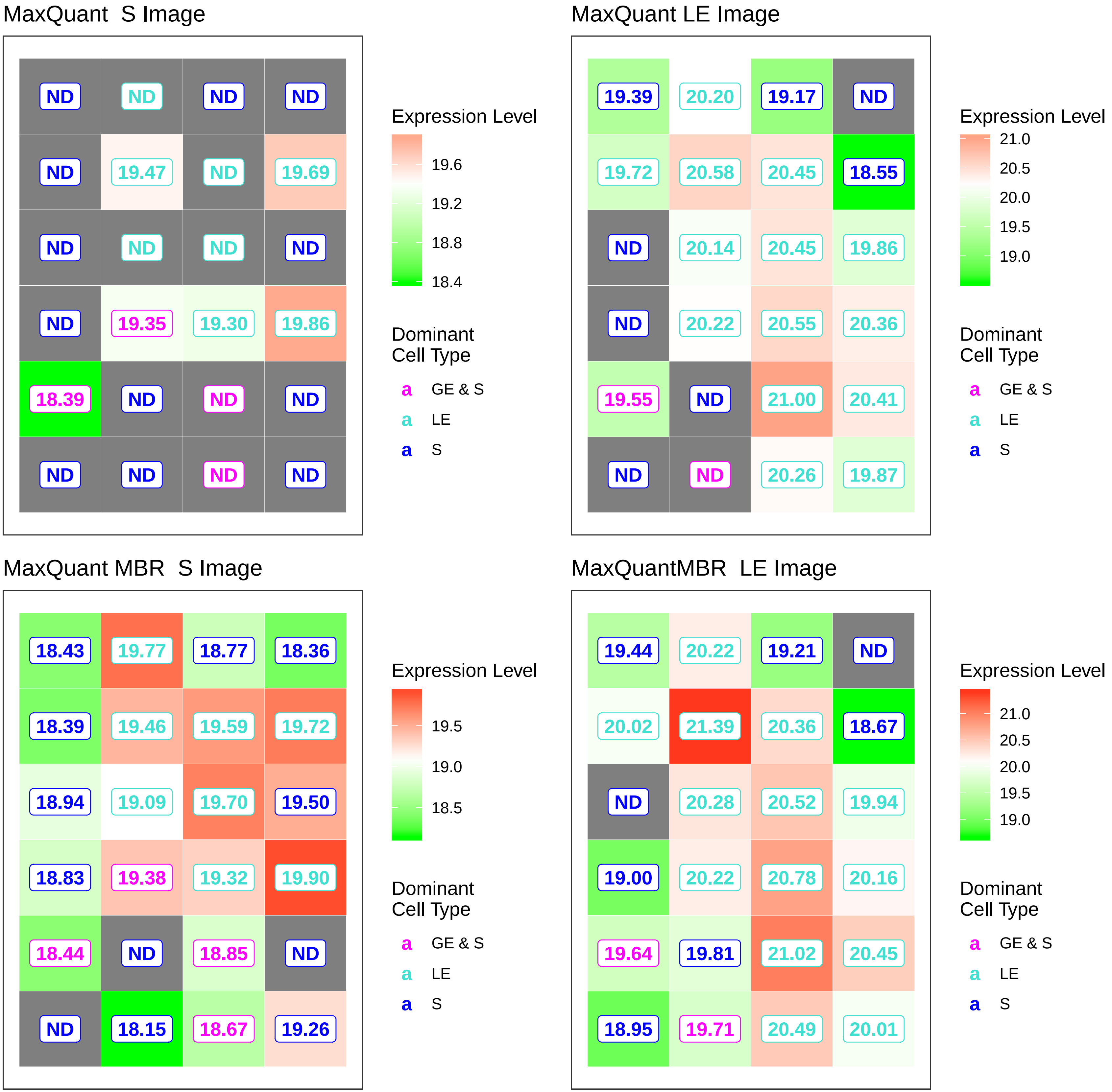

MaxQuant S Image
ND ND ND ND
ND 19.47 ND 19.69
ND ND ND ND
ND 19.35 19.30 19.86
18.39 ND ND ND
ND ND ND ND
Expression Level
19.6
19.2
18.8
18.4
Dominant Cell Type
a GE & S
a LE
a S
MaxQuant LE Image
19.39 20.20 19.17 ND
19.72 20.58 20.45 18.55
ND 20.14 20.45 19.86
ND 20.22 20.55 20.36
19.55 ND 21.00 20.41
ND ND 20.26 19.87
Expression Level
21.0
20.5
20.0
19.5
19.0
Dominant Cell Type
a GE & S
a LE
a S
MaxQuant MBR S Image
18.43 19.77 18.77 18.36
18.39 19.46 19.59 19.72
18.94 19.09 19.70 19.50
18.83 19.38 19.32 19.90
18.44 ND 18.85 ND
ND 18.15 18.67 19.26
Expression Level
19.5
19.0
18.5
Dominant Cell Type
a GE & S
a LE
a S
MaxQuantMBR LE Image
19.44 20.22 19.21 ND
20.02 21.39 20.36 18.67
ND 20.28 20.52 19.94
19.00 20.22 20.78 20.16
19.64 19.81 21.02 20.45
18.95 19.71 20.49 20.01
Expression Level
21.0
20.5
20.0
19.5
19.0
Dominant Cell Type
a GE & S
a LE
a S

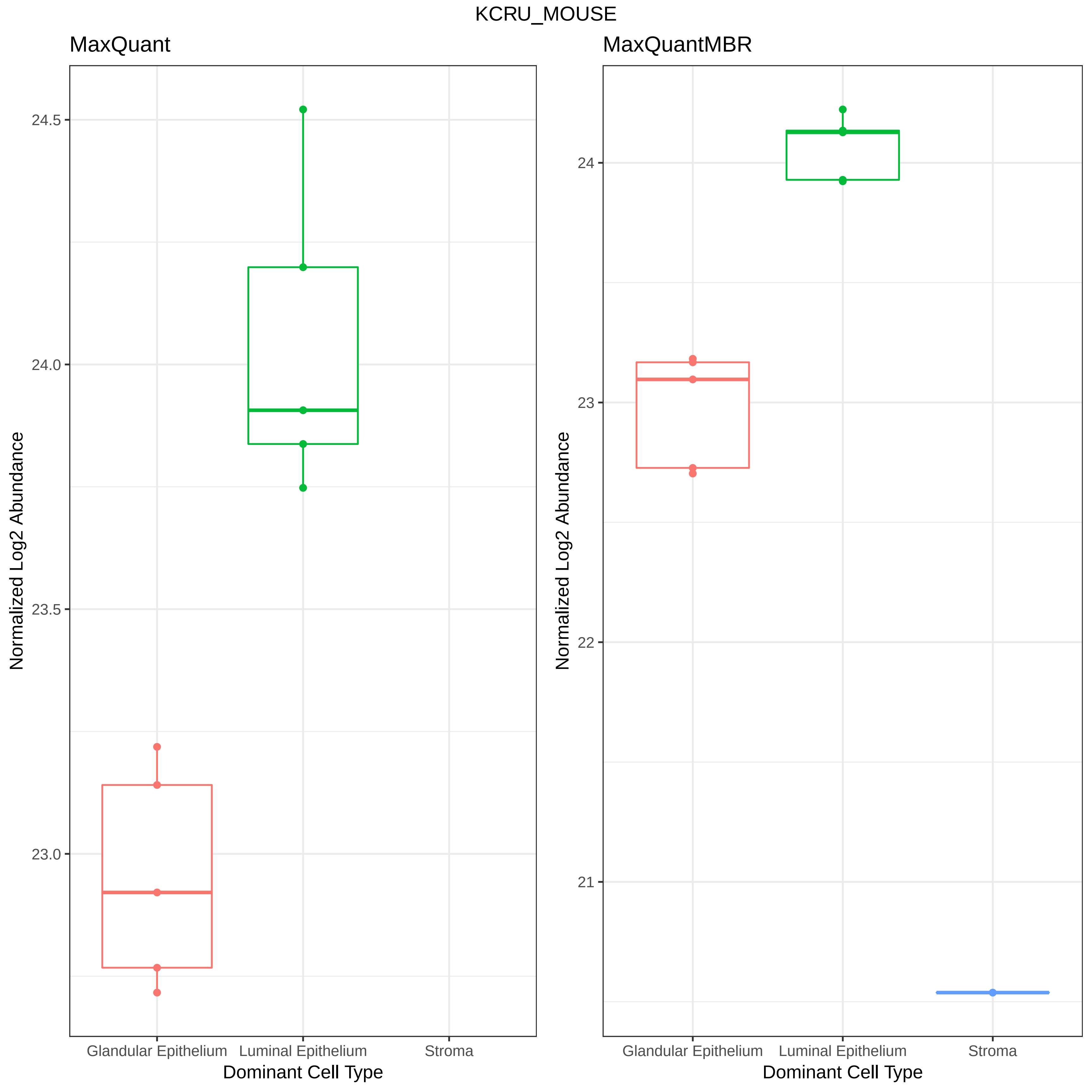
KCRU_MOUSE
MaxQuant
MaxQuantMBR
Normalized Log2 Abundance
24.5
24.0
23.5
23.0
24
23
22
21
Glandular Epithelium
Luminal Epithelium
Stroma
Dominant Cell Type

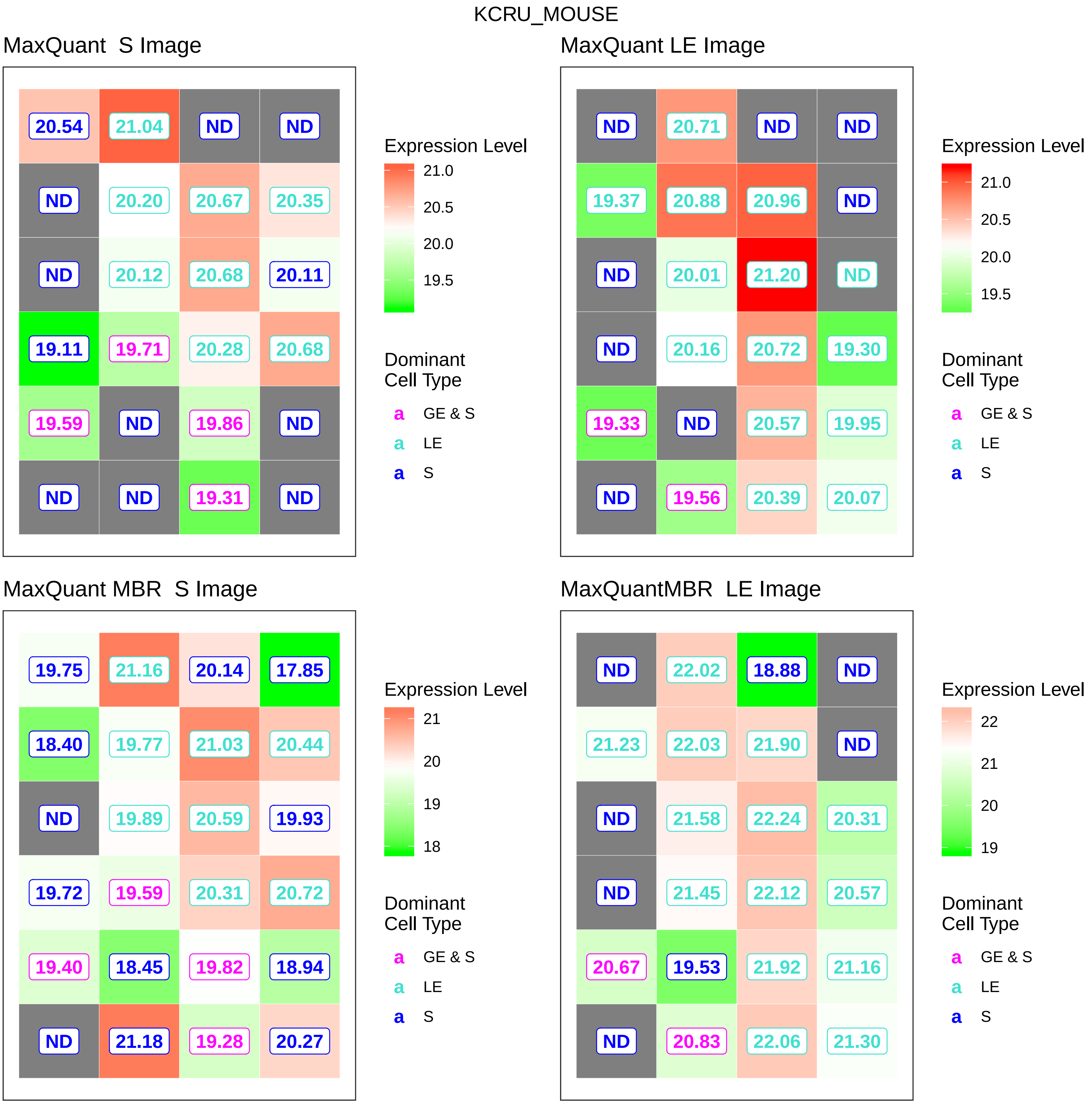

KCRU_MOUSE
MaxQuant S Image
20.54 21.04 ND ND
ND 20.20 20.67 20.35
ND 20.12 20.68 20.11
19.11 19.71 20.28 20.68
19.59 ND 19.86 ND
ND ND 19.31 ND
Expression Level
21.0
20.5
20.0
19.5
Dominant Cell Type
a GE & S
a LE
a S
MaxQuant LE Image
ND 20.71 ND ND
19.37 20.88 20.96 ND
ND 20.01 21.20 ND
ND 20.16 20.72 19.30
19.33 ND 20.57 19.95
ND 19.56 20.39 20.07
Expression Level
21.0
20.5
20.0
19.5
Dominant Cell Type
a GE & S
a LE
a S
MaxQuant MBR S Image
19.75 21.16 20.14 17.85
18.40 19.77 21.03 20.44
ND 19.89 20.59 19.93
19.72 19.59 20.31 20.72
19.40 18.45 19.82 18.94
ND 21.18 19.28 20.27
Expression Level
21
20
19
18
Dominant Cell Type
a GE & S
a LE
a S
MaxQuantMBR LE Image
ND 22.02 18.88 ND
21.23 22.03 21.90 ND
ND 21.58 22.24 20.31
ND 21.45 22.12 20.57
20.67 19.53 21.92 21.16
ND 20.83 22.06 21.30
Expression Level
22
21
20
19
Dominant Cell Type
a GE & S
a LE
a S

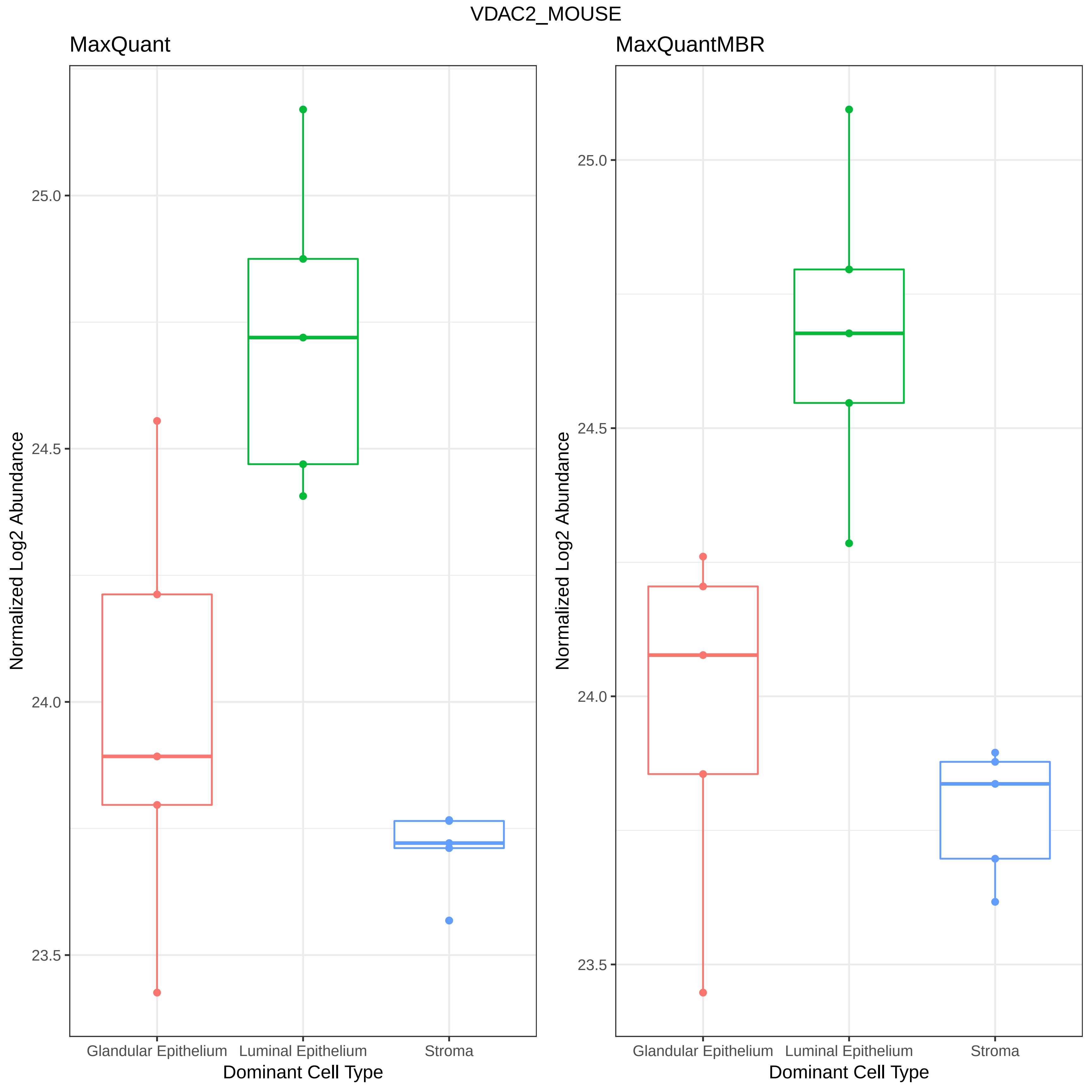

VDAC2_MOUSE
MaxQuant
MaxQuantMBR
Normalized Log2 Abundance
25.0
24.5
24.0
23.5
Glandular Epithelium
Luminal Epithelium
Stroma
Dominant Cell Type

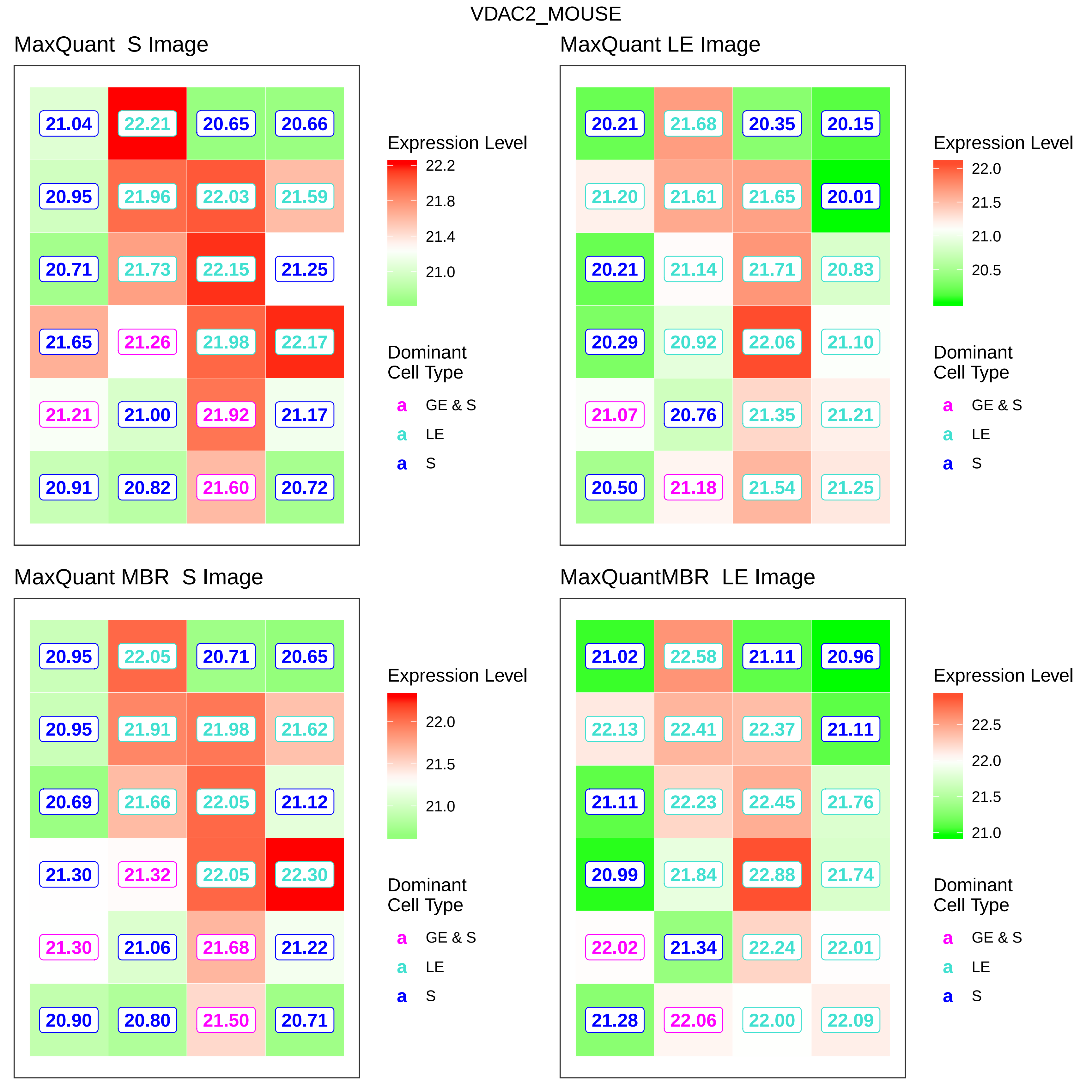
VDAC2_MOUSE
MaxQuant S Image
21.04 22.21 20.65 20.66
20.95 21.96 22.03 21.59
20.71 21.73 22.15 21.25
21.65 21.26 21.98 22.17
21.21 21.00 21.92 21.17
20.91 20.82 21.60 20.72
Expression Level
22.2
21.8
21.4
21.0
Dominant Cell Type
a GE & S
a LE
a S
MaxQuant LE Image
20.21 21.68 20.35 20.15
21.20 21.61 21.65 20.01
20.21 21.14 21.71 20.83
20.29 20.92 22.06 21.10
21.07 20.76 21.35 21.21
20.50 21.18 21.54 21.25
Expression Level
22.0
21.5
21.0
20.5
Dominant Cell Type
a GE & S
a LE
a S
MaxQuant MBR S Image
20.95 22.05 20.71 20.65
20.95 21.91 21.98 21.62
20.69 21.66 22.05 21.12
21.30 21.32 22.05 22.30
21.30 21.06 21.68 21.22
20.90 20.80 21.50 20.71
Expression Level
22.0
21.5
21.0
Dominant Cell Type
a GE & S
a LE
a S
MaxQuantMBR LE Image
21.02 22.58 21.11 20.96
22.13 22.41 22.37 21.11
21.11 22.23 22.45 21.76
20.99 21.84 22.88 21.74
22.02 21.34 22.24 22.01
21.28 22.06 22.00 22.09
Expression Level
22.5
22.0
21.5
21.0
Dominant Cell Type
a GE & S
a LE
a S

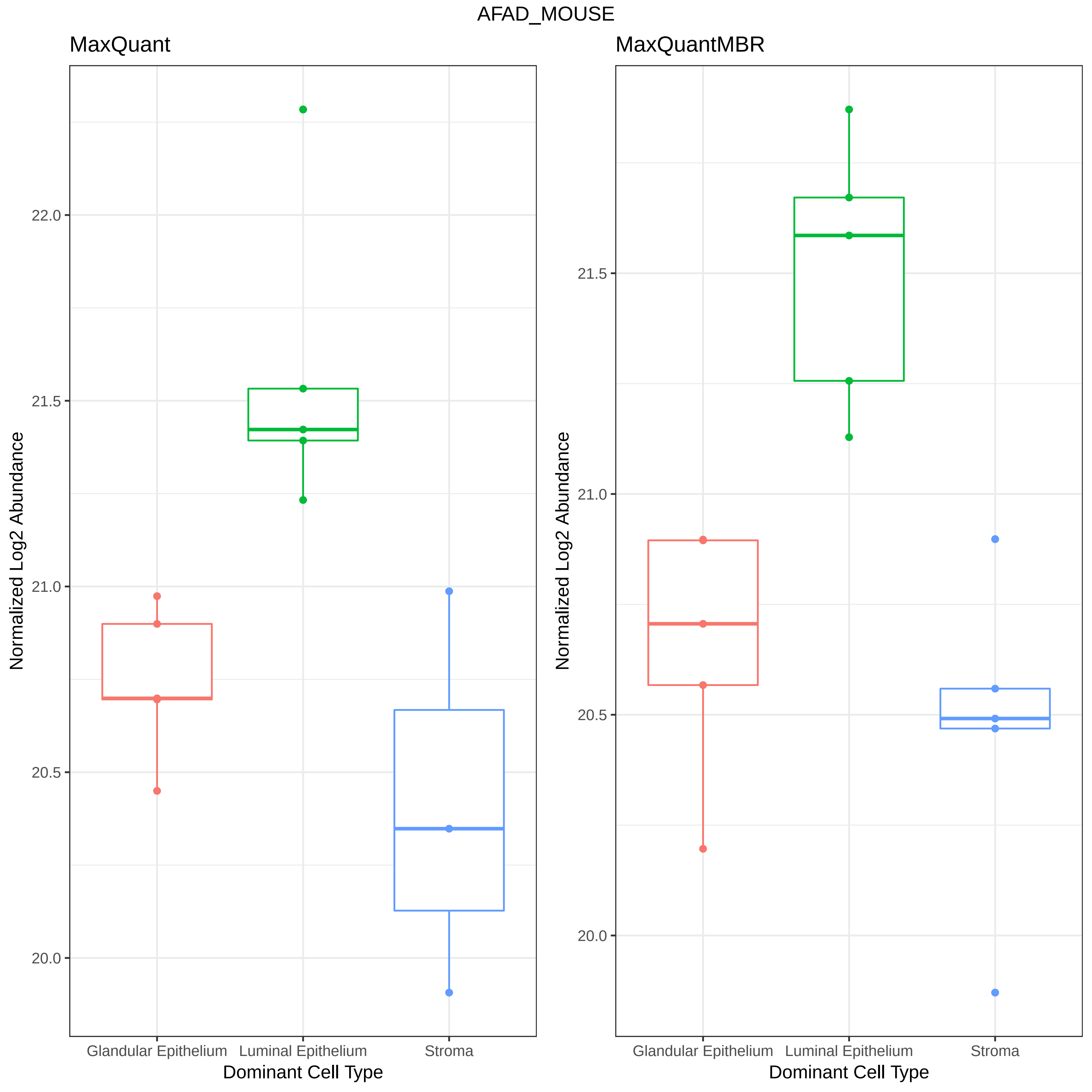
AFAD_MOUSE
MaxQuant
MaxQuantMBR
Normalized Log2 Abundance
22.0
21.5
21.0
20.5
20.0
Glandular Epithelium
Luminal Epithelium
Stroma
Dominant Cell Type

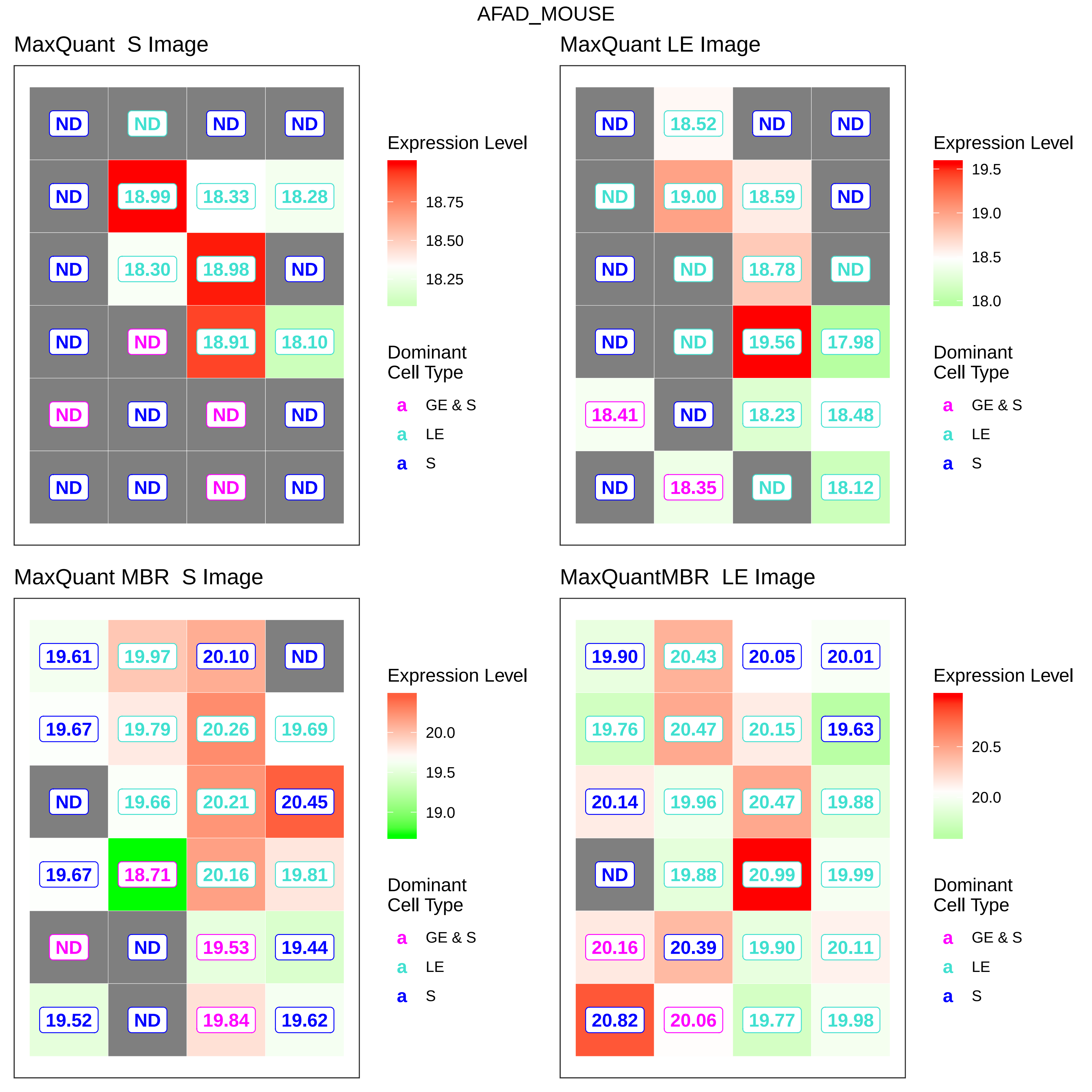

AFAD_MOUSE
MaxQuant S Image
ND ND ND ND
ND 18.99 18.33 18.28
ND 18.30 18.98 ND
ND ND 18.91 18.10
ND ND ND ND
ND ND ND ND
Expression Level
18.75
18.50
18.25
Dominant Cell Type
a GE & S
a LE
a S
MaxQuant LE Image
ND 18.52 ND ND
ND 19.00 18.59 ND
ND ND 18.78 ND
ND ND 19.56 17.98
18.41 ND 18.23 18.48
ND 18.35 ND 18.12
Expression Level
19.5
19.0
18.5
18.0
Dominant Cell Type
a GE & S
a LE
a S
MaxQuant MBR S Image
19.61 19.97 20.10 ND
19.67 19.79 20.26 19.69
ND 19.66 20.21 20.45
19.67 18.71 20.16 19.81
ND ND 19.53 19.44
19.52 ND 19.84 19.62
Expression Level
20.0
19.5
19.0
Dominant Cell Type
a GE & S
a LE
a S
MaxQuantMBR LE Image
19.90 20.43 20.05 20.01
19.76 20.47 20.15 19.63
20.14 19.96 20.47 19.88
ND 19.88 20.99 19.99
20.16 20.39 19.90 20.11
20.82 20.06 19.77 19.98
Expression Level
20.5
20.0
Dominant Cell Type
a GE & S
a LE
a S

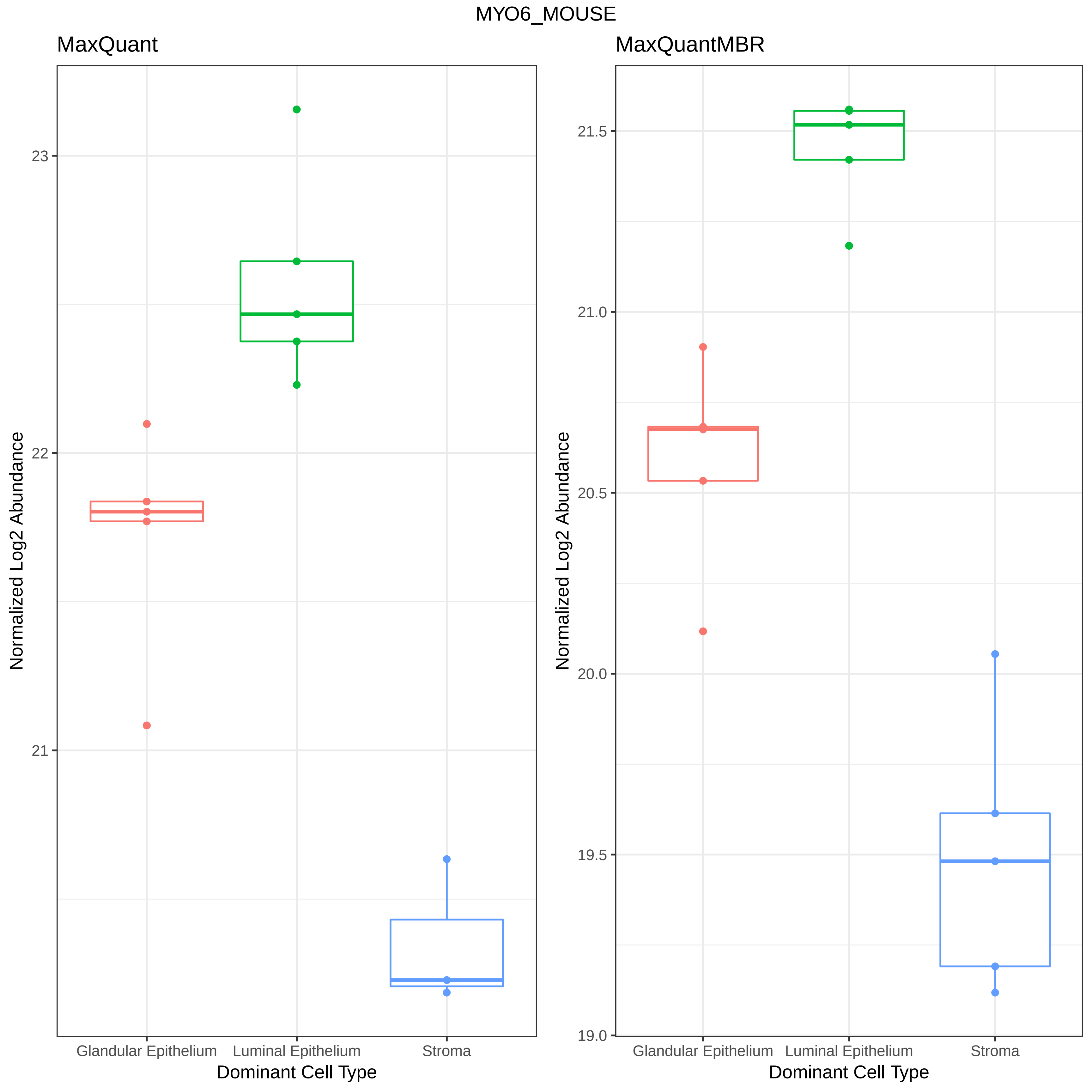

MYO6_MOUSE
MaxQuant
MaxQuantMBR
23
22
21
21.5
21.0
20.5
20.0
19.5
19.0
Normalized Log2 Abundance
Glandular Epithelium
Luminal Epithelium
Stroma
Dominant Cell Type

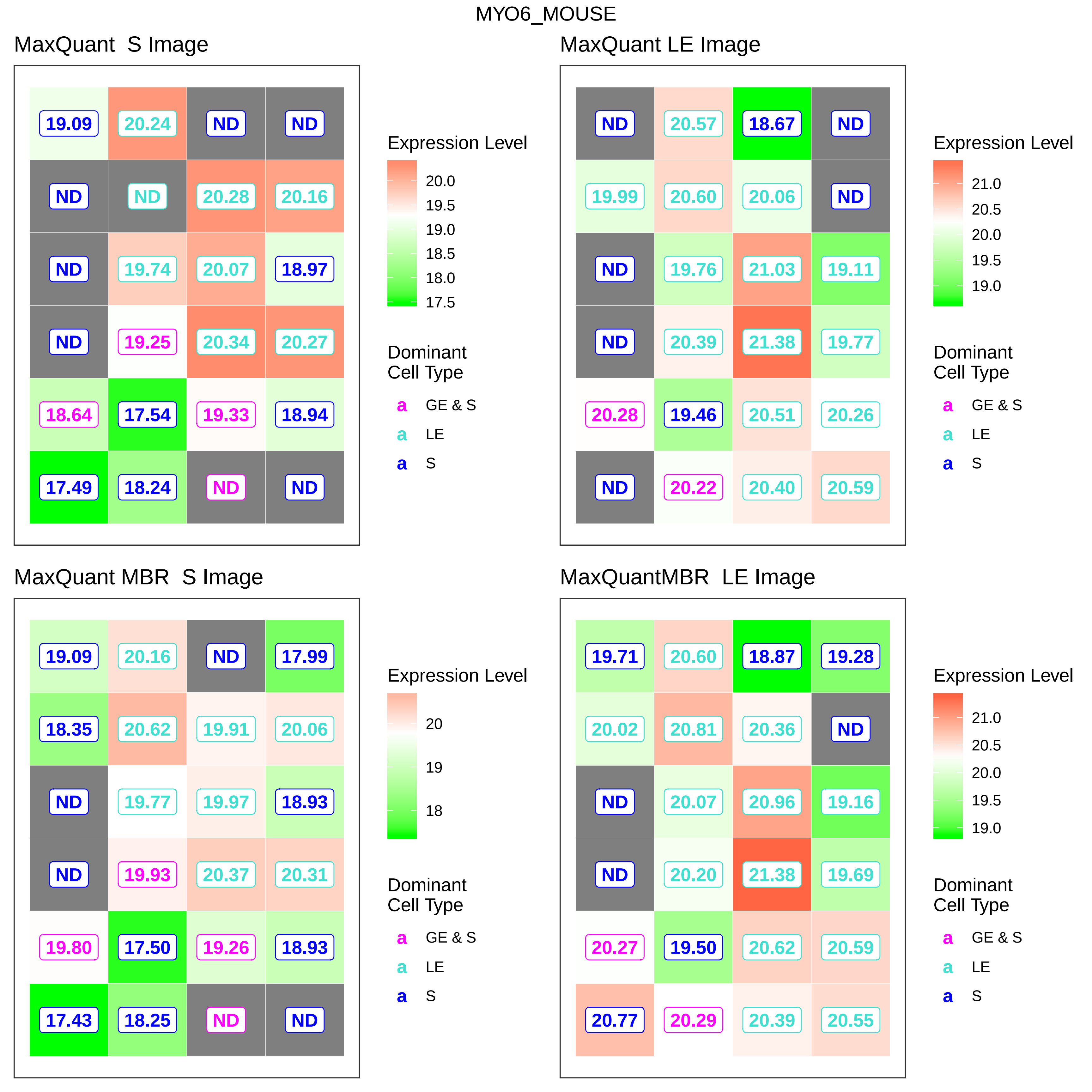
MYO6_MOUSE
MaxQuant S Image
19.09 20.24 ND ND
ND ND 20.28 20.16
ND 19.74 20.07 18.97
ND 19.25 20.34 20.27
18.64 17.54 19.33 18.94
17.49 18.24 ND ND
Expression Level
20.0
19.5
19.0
18.5
18.0
17.5
Dominant Cell Type
a GE & S
a LE
a S
MaxQuant LE Image
ND 20.57 18.67 ND
19.99 20.60 20.06 ND
ND 19.76 21.03 19.11
ND 20.39 21.38 19.77
20.28 19.46 20.51 20.26
ND 20.22 20.40 20.59
Expression Level
21.0
20.5
20.0
19.5
19.0
Dominant Cell Type
a GE & S
a LE
a S
MaxQuant MBR S Image
19.09 20.16 ND 17.99
18.35 20.62 19.91 20.06
ND 19.77 19.97 18.93
ND 19.93 20.37 20.31
19.80 17.50 19.26 18.93
17.43 18.25 ND ND
Expression Level
20
19
18
Dominant Cell Type
a GE & S
a LE
a S
MaxQuantMBR LE Image
19.71 20.60 18.87 19.28
20.02 20.81 20.36 ND
ND 20.07 20.96 19.16
ND 20.20 21.38 19.69
20.27 19.50 20.62 20.59
20.77 20.29 20.39 20.55
Expression Level
21.0
20.5
20.0
19.5
19.0
Dominant Cell Type
a GE & S
a LE
a S

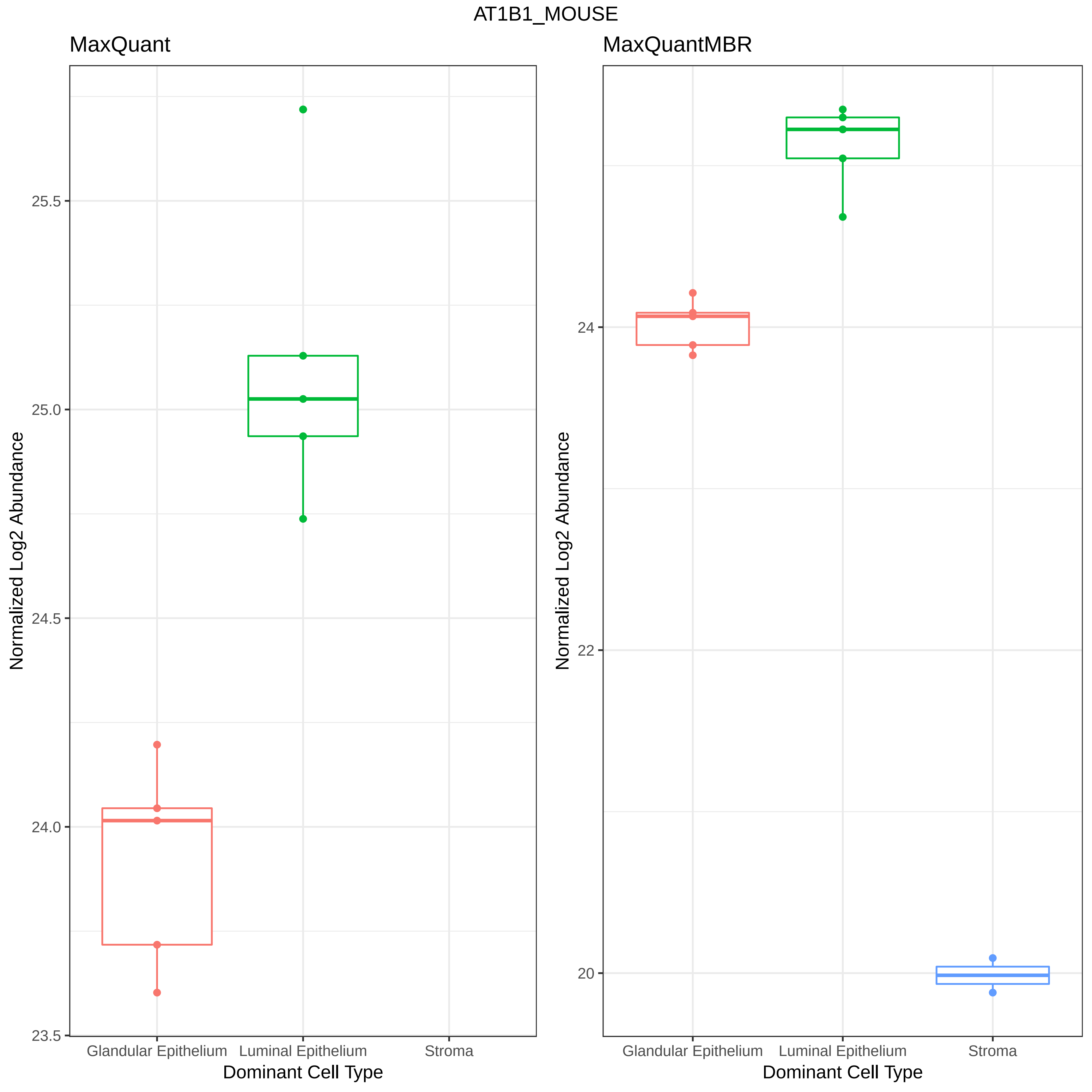

AT1B1_MOUSE
MaxQuant
MaxQuantMBR
Normalized Log2 Abundance
25.5
25.0
24.5
24.0
23.5
24
22
20
Glandular Epithelium
Luminal Epithelium
Stroma
Dominant Cell Type

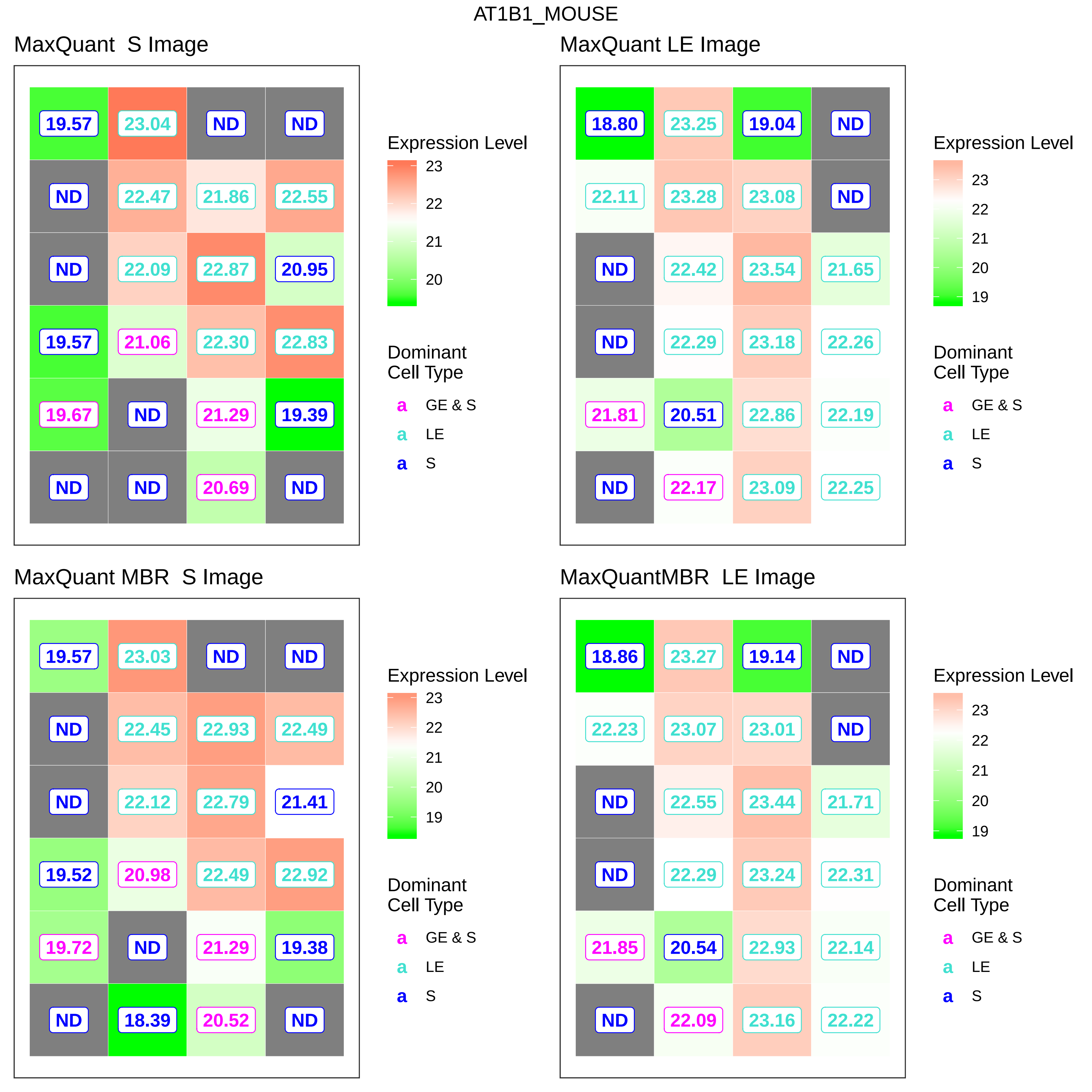
AT1B1_MOUSE
MaxQuant S Image
19.57
23.04
ND
ND
ND
22.47
21.86
22.55
ND
22.09
22.87
20.95
19.57
21.06
22.30
22.83
19.67
ND
21.29
19.39
ND
ND
20.69
ND
Expression Level
23
22
21
20
Dominant Cell Type
a GE & S
a LE
a S
MaxQuant LE Image
18.80
23.25
19.04
ND
22.11
23.28
23.08
ND
ND
22.42
23.54
21.65
ND
22.29
23.18
22.26
21.81
20.51
22.86
22.19
ND
22.17
23.09
22.25
Expression Level
23
22
21
20
19
Dominant Cell Type
a GE & S
a LE
a S
MaxQuant MBR S Image
19.57
23.03
ND
ND
ND
22.45
22.93
22.49
ND
22.12
22.79
21.41
19.52
20.98
22.49
22.92
19.72
ND
21.29
19.38
ND
18.39
20.52
ND
Expression Level
23
22
21
20
19
Dominant Cell Type
a GE & S
a LE
a S
MaxQuantMBR LE Image
18.86
23.27
19.14
ND
22.23
23.07
23.01
ND
ND
22.55
23.44
21.71
ND
22.29
23.24
22.31
21.85
20.54
22.93
22.14
ND
22.09
23.16
22.22
Expression Level
23
22
21
20
19
Dominant Cell Type
a GE & S
a LE
a S

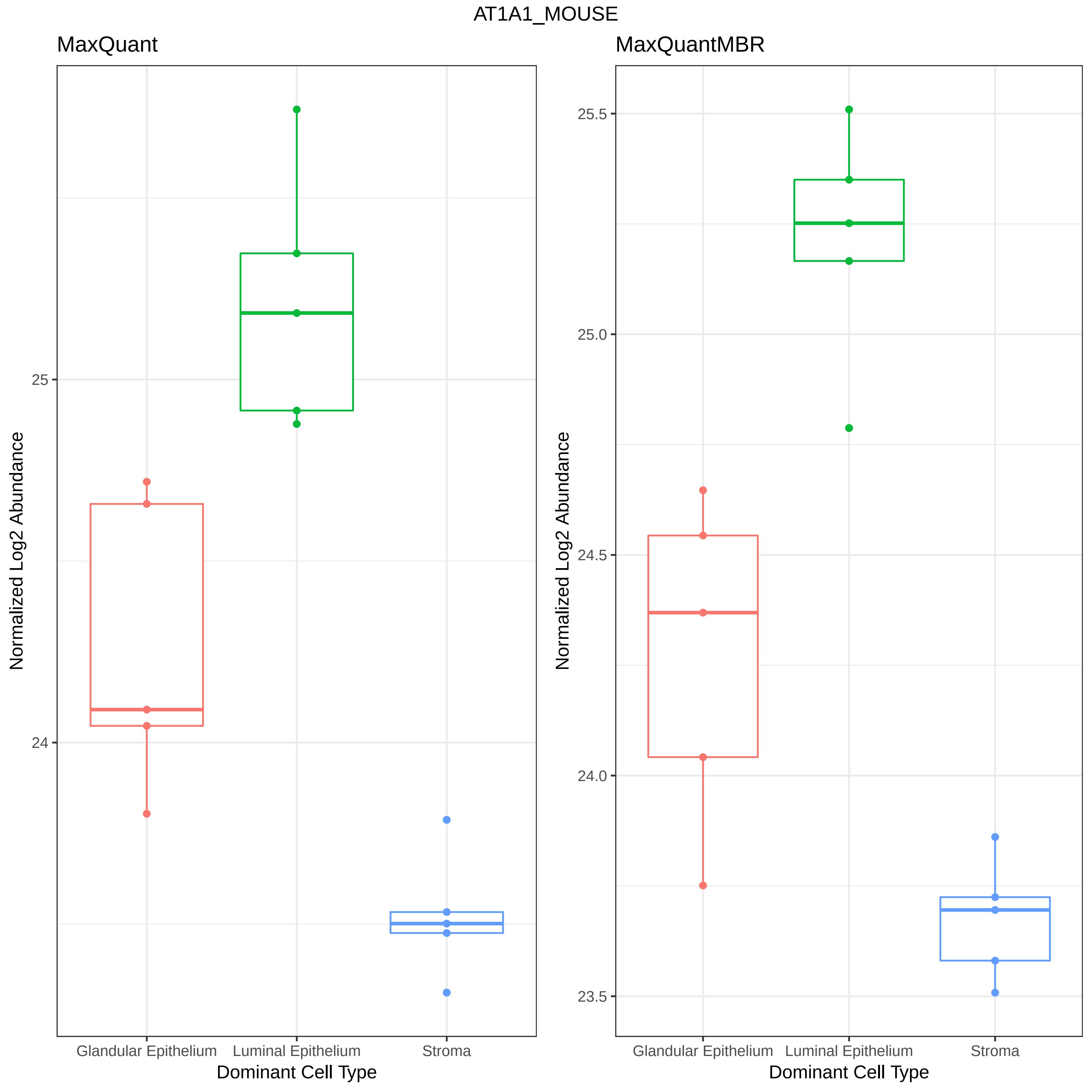
AT1A1_MOUSE
MaxQuant
MaxQuantMBR
Normalized Log2 Abundance
25
24
25.5
25.0
24.5
24.0
23.5
Glandular Epithelium
Luminal Epithelium
Stroma
Dominant Cell Type

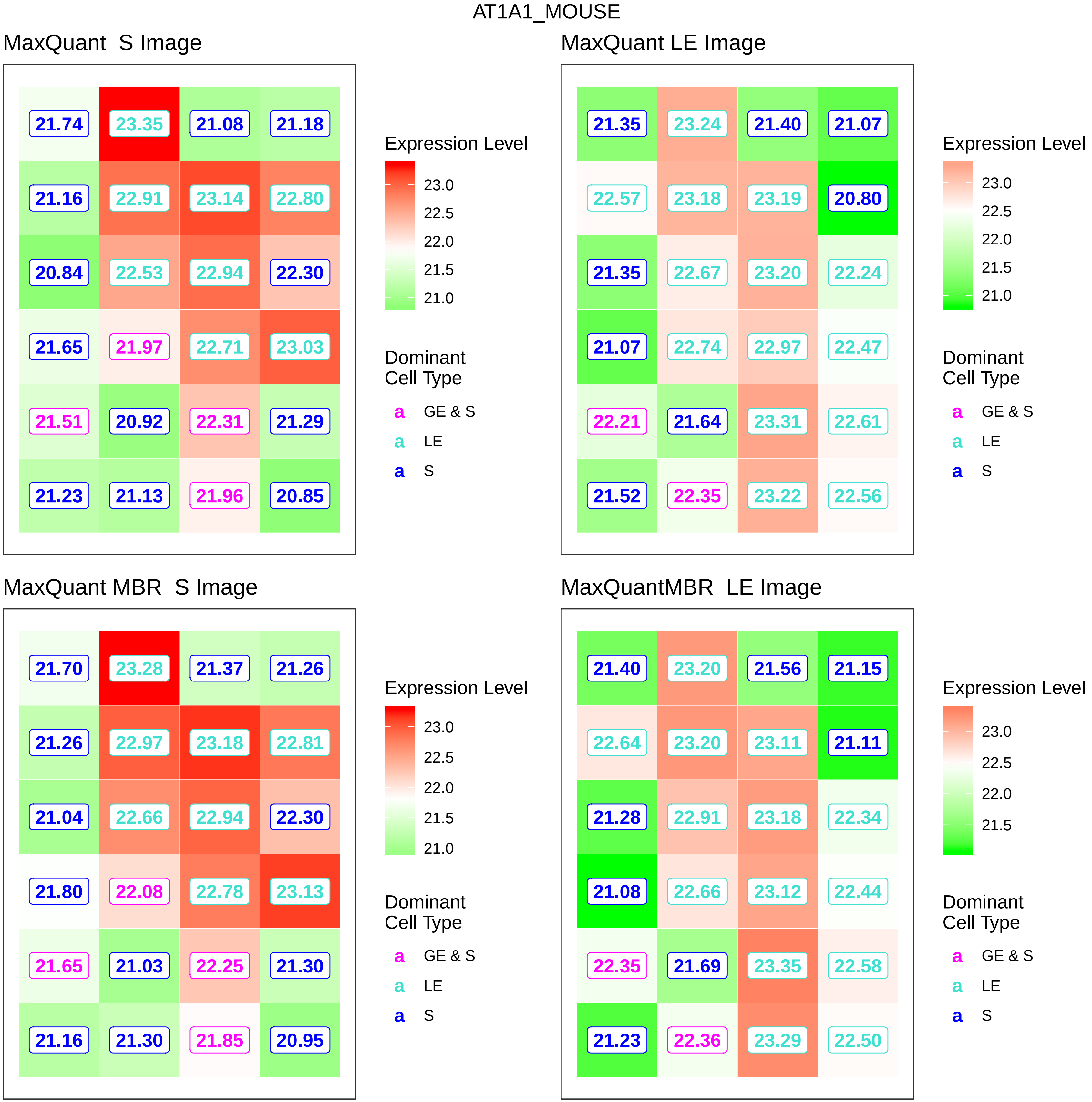
AT1A1_MOUSE
MaxQuant S Image
21.74 23.35 21.08 21.18
21.16 22.91 23.14 22.80
20.84 22.53 22.94 22.30
21.65 21.97 22.71 23.03
21.51 20.92 22.31 21.29
21.23 21.13 21.96 20.85
Expression Level
23.0
22.5
22.0
21.5
21.0
Dominant Cell Type
a GE & S
a LE
a S
MaxQuant LE Image
21.35 23.24 21.40 21.07
22.57 23.18 23.19 20.80
21.35 22.67 23.20 22.24
21.07 22.74 22.97 22.47
22.21 21.64 23.31 22.61
21.52 22.35 23.22 22.56
Expression Level
23.0
22.5
22.0
21.5
21.0
Dominant Cell Type
a GE & S
a LE
a S
MaxQuant MBR S Image
21.70 23.28 21.37 21.26
21.26 22.97 23.18 22.81
21.04 22.66 22.94 22.30
21.80 22.08 22.78 23.13
21.65 21.03 22.25 21.30
21.16 21.30 21.85 20.95
Expression Level
23.0
22.5
22.0
21.5
21.0
Dominant Cell Type
a GE & S
a LE
a S
MaxQuantMBR LE Image
21.40 23.20 21.56 21.15
22.64 23.20 23.11 21.11
21.28 22.91 23.18 22.34
21.08 22.66 23.12 22.44
22.35 21.69 23.35 22.58
21.23 22.36 23.29 22.50
Expression Level
23.0
22.5
22.0
21.5
Dominant Cell Type
a GE & S
a LE
a S

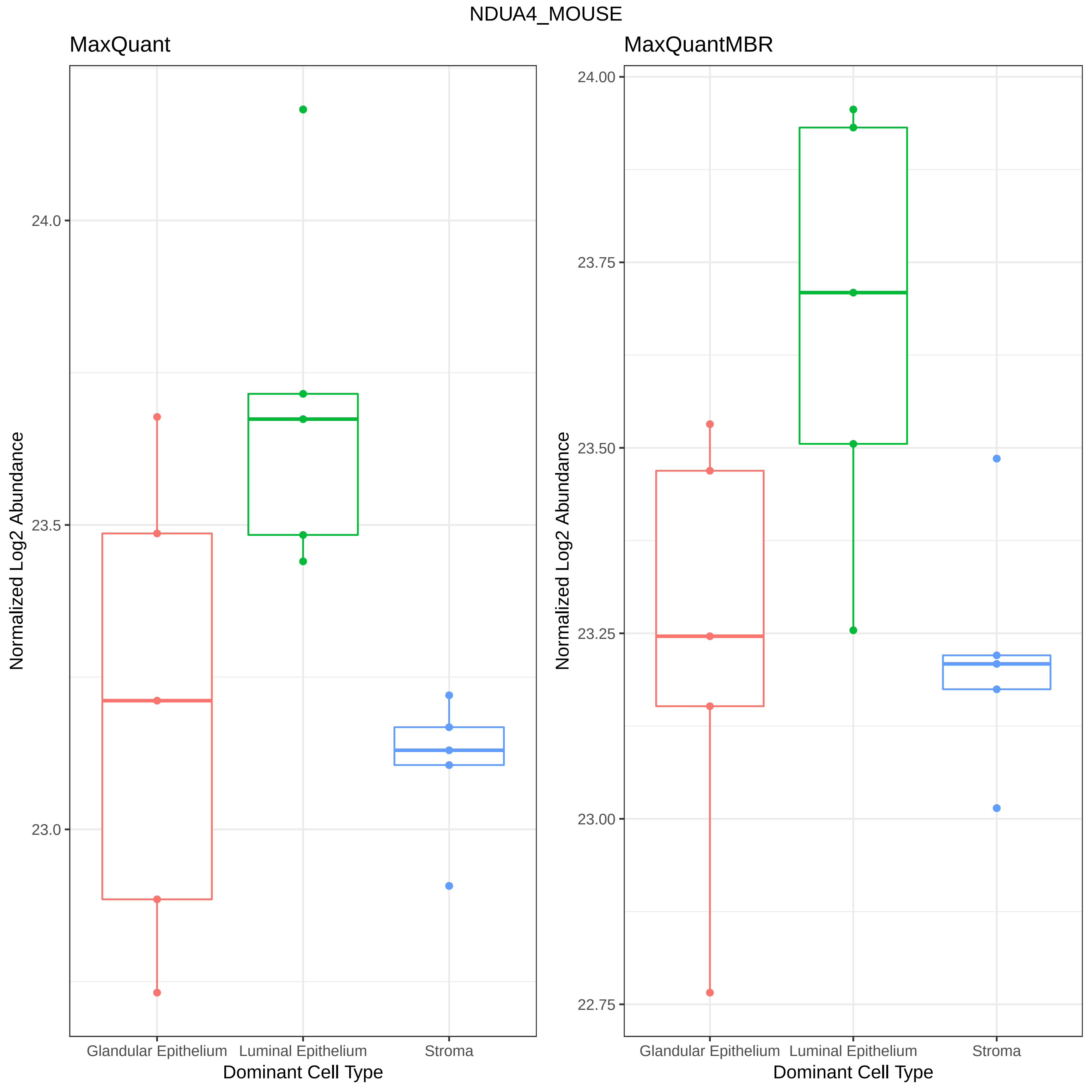
NDUA4_MOUSE
MaxQuant
MaxQuantMBR
Normalized Log2 Abundance
24.0
23.5
23.0
24.00
23.75
23.50
23.25
23.00
22.75
Glandular Epithelium
Luminal Epithelium
Stroma
Dominant Cell Type

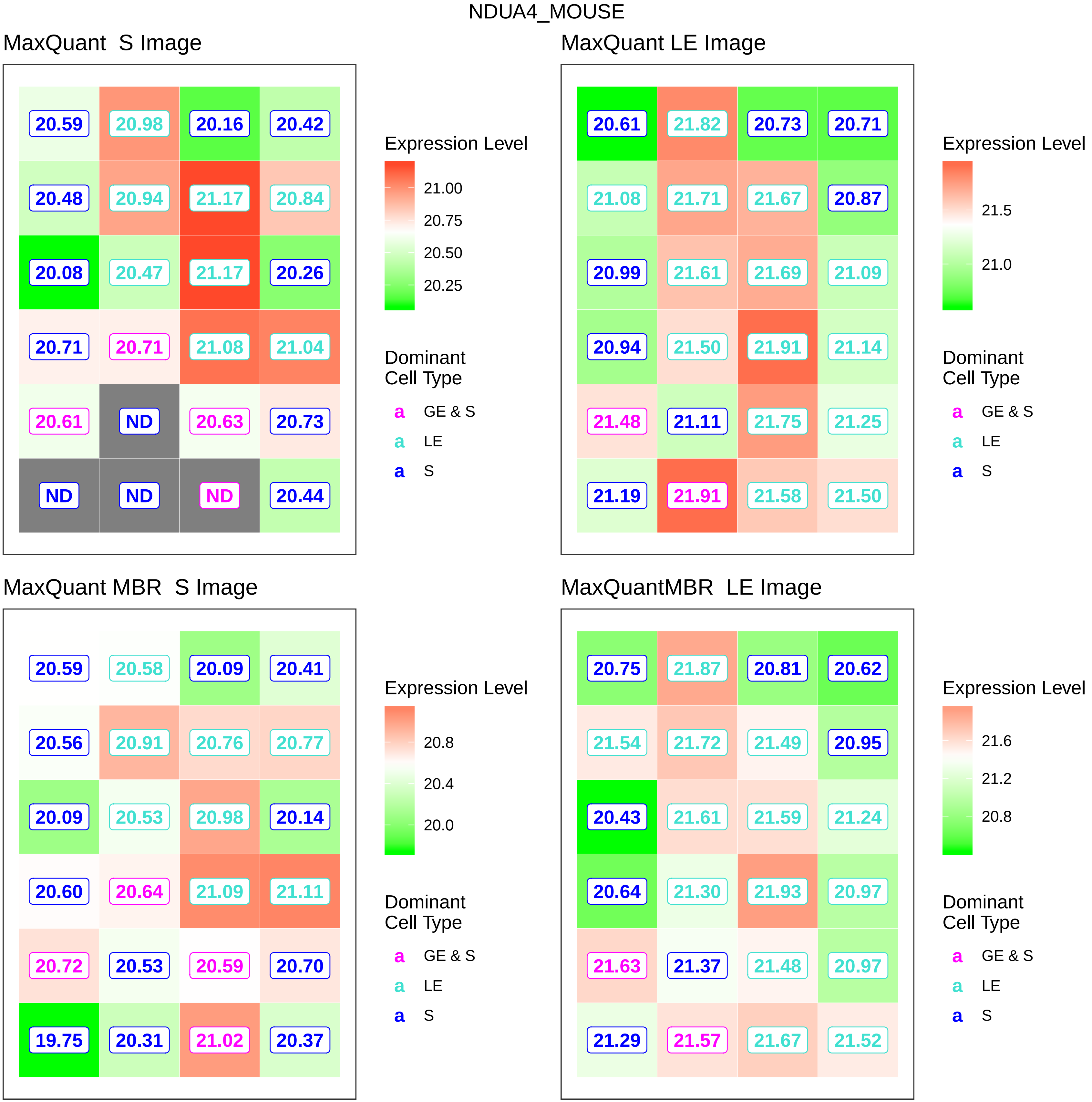

NDUA4_MOUSE
MaxQuant S Image
20.59 20.98 20.16 20.42
20.48 20.94 21.17 20.84
20.08 20.47 21.17 20.26
20.71 20.71 21.08 21.04
20.61 ND 20.63 20.73
ND ND ND 20.44
Expression Level
21.00
20.75
20.50
20.25
Dominant Cell Type
a GE & S
a LE
a S
MaxQuant LE Image
20.61 21.82 20.73 20.71
21.08 21.71 21.67 20.87
20.99 21.61 21.69 21.09
20.94 21.50 21.91 21.14
21.48 21.11 21.75 21.25
21.19 21.91 21.58 21.50
Expression Level
21.5
21.0
Dominant Cell Type
a GE & S
a LE
a S
MaxQuant MBR S Image
20.59 20.58 20.09 20.41
20.56 20.91 20.76 20.77
20.09 20.53 20.98 20.14
20.60 20.64 21.09 21.11
20.72 20.53 20.59 20.70
19.75 20.31 21.02 20.37
Expression Level
20.8
20.4
20.0
Dominant Cell Type
a GE & S
a LE
a S
MaxQuantMBR LE Image
20.75 21.87 20.81 20.62
21.54 21.72 21.49 20.95
20.43 21.61 21.59 21.24
20.64 21.30 21.93 20.97
21.63 21.37 21.48 20.97
21.29 21.57 21.67 21.52
Expression Level
21.6
21.2
20.8
Dominant Cell Type
a GE & S
a LE
a S

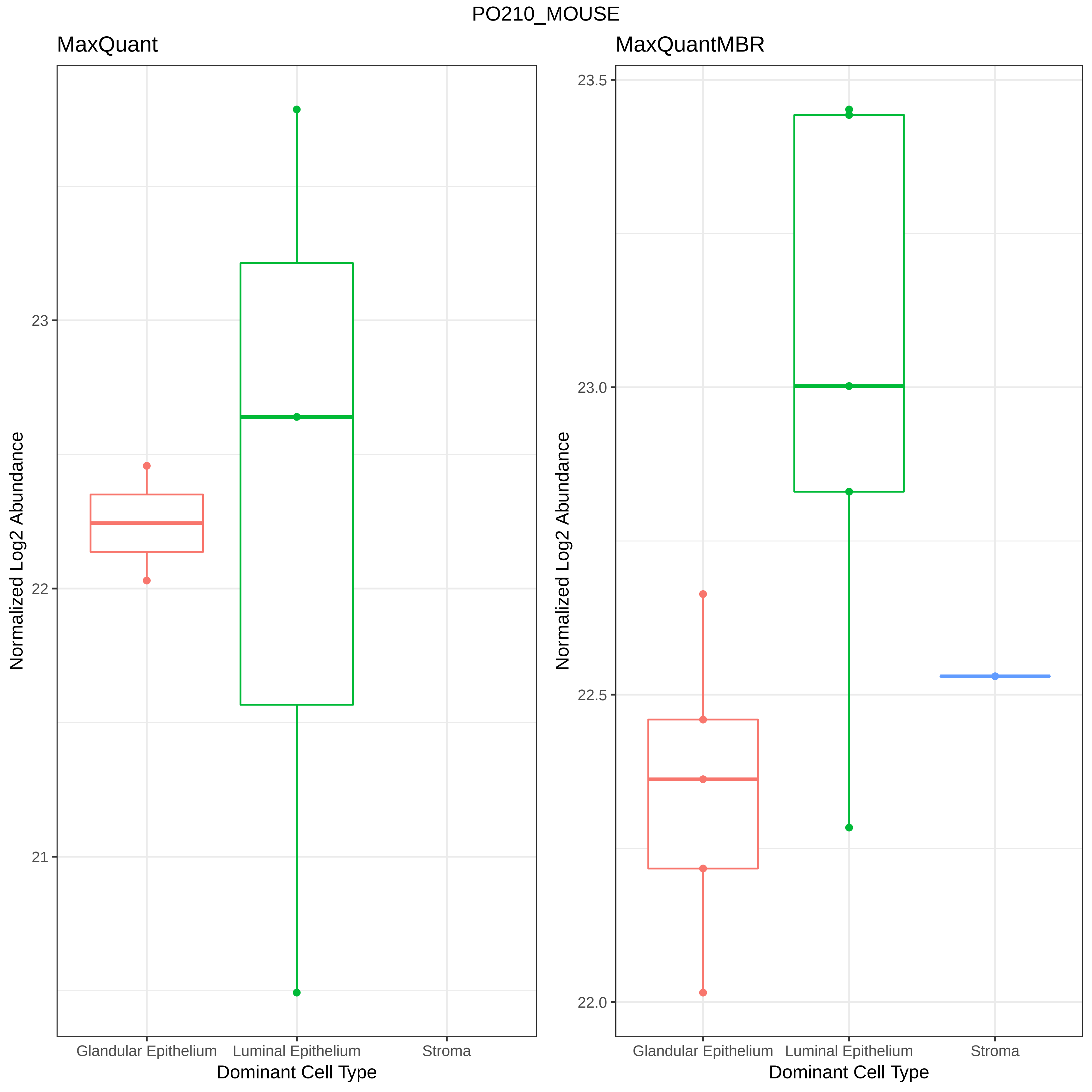
PO210_MOUSE
MaxQuant
MaxQuantMBR
Normalized Log2 Abundance
23
22
21
23.5
23.0
22.5
22.0
Glandular Epithelium
Luminal Epithelium
Stroma
Dominant Cell Type

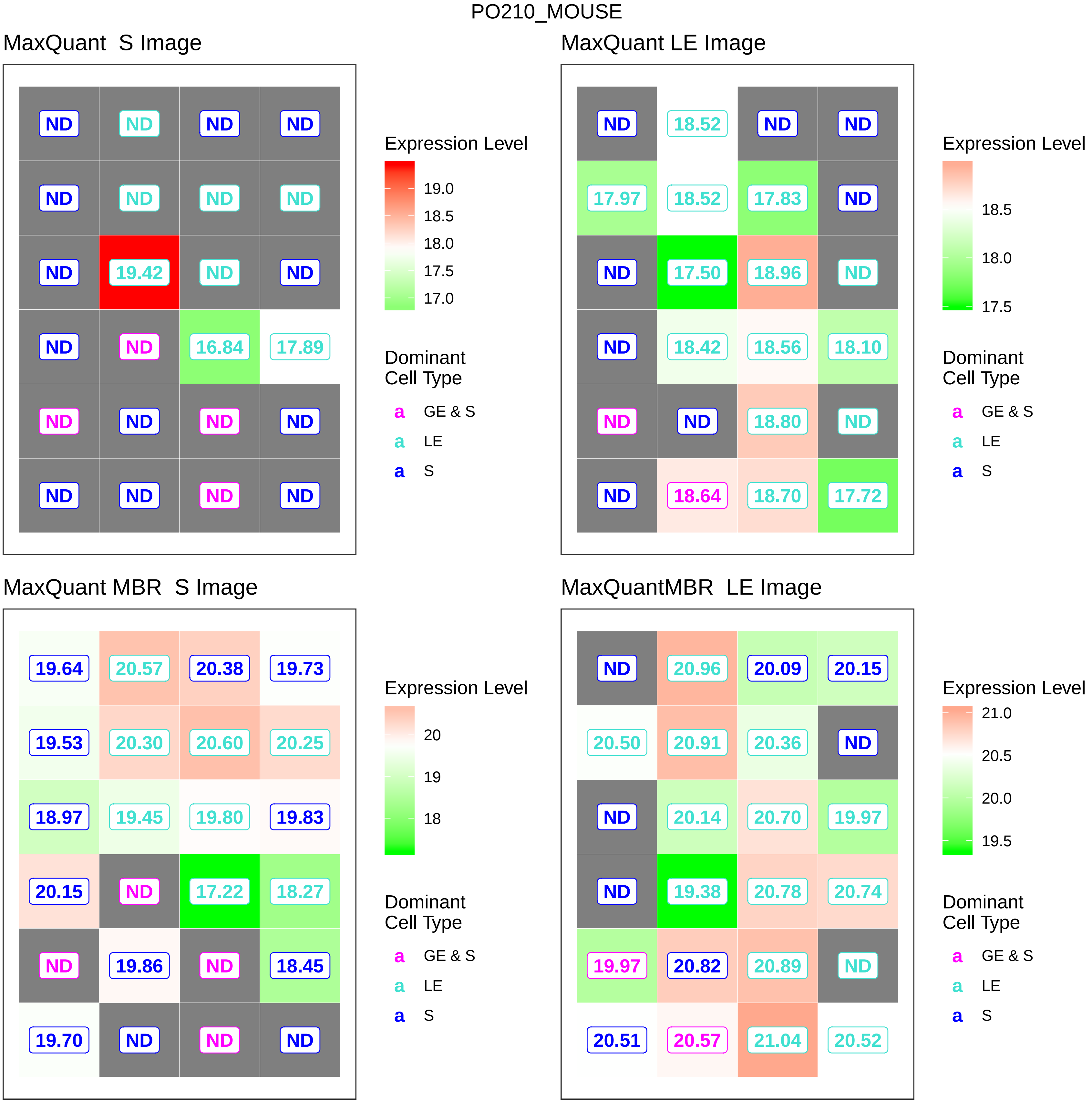
PO210_MOUSE
MaxQuant S Image
ND ND ND ND
ND ND ND ND
ND 19.42 ND ND
ND ND 16.84 17.89
ND ND ND ND
ND ND ND ND
Expression Level
19.0
18.5
18.0
17.5
17.0
Dominant Cell Type
a GE & S
a LE
a S
MaxQuant LE Image
ND 18.52 ND ND
17.97 18.52 17.83 ND
ND 17.50 18.96 ND
ND 18.42 18.56 18.10
ND ND 18.80 ND
ND 18.64 18.70 17.72
Expression Level
18.5
18.0
17.5
Dominant Cell Type
a GE & S
a LE
a S
MaxQuant MBR S Image
19.64 20.57 20.38 19.73
19.53 20.30 20.60 20.25
18.97 19.45 19.80 19.83
20.15 ND 17.22 18.27
ND 19.86 ND 18.45
19.70 ND ND ND
Expression Level
20
19
18
Dominant Cell Type
a GE & S
a LE
a S
MaxQuantMBR LE Image
ND 20.96 20.09 20.15
20.50 20.91 20.36 ND
ND 20.14 20.70 19.97
ND 19.38 20.78 20.74
19.97 20.82 20.89 ND
20.51 20.57 21.04 20.52
Expression Level
21.0
20.5
20.0
19.5
Dominant Cell Type
a GE & S
a LE
a S

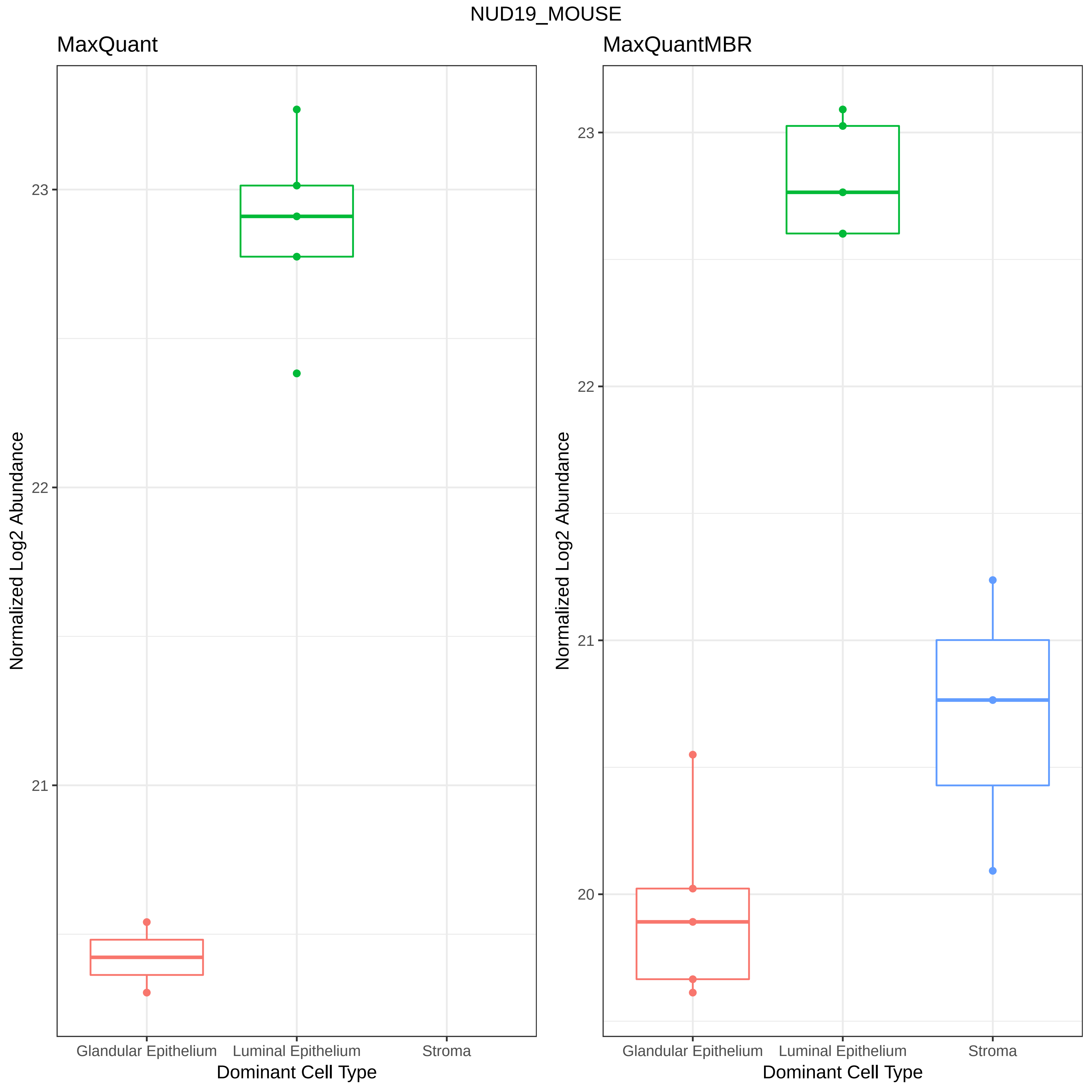
NUD19_MOUSE
MaxQuant
MaxQuantMBR
Normalized Log2 Abundance
23
22
21
20
Glandular Epithelium
Luminal Epithelium
Stroma
Dominant Cell Type

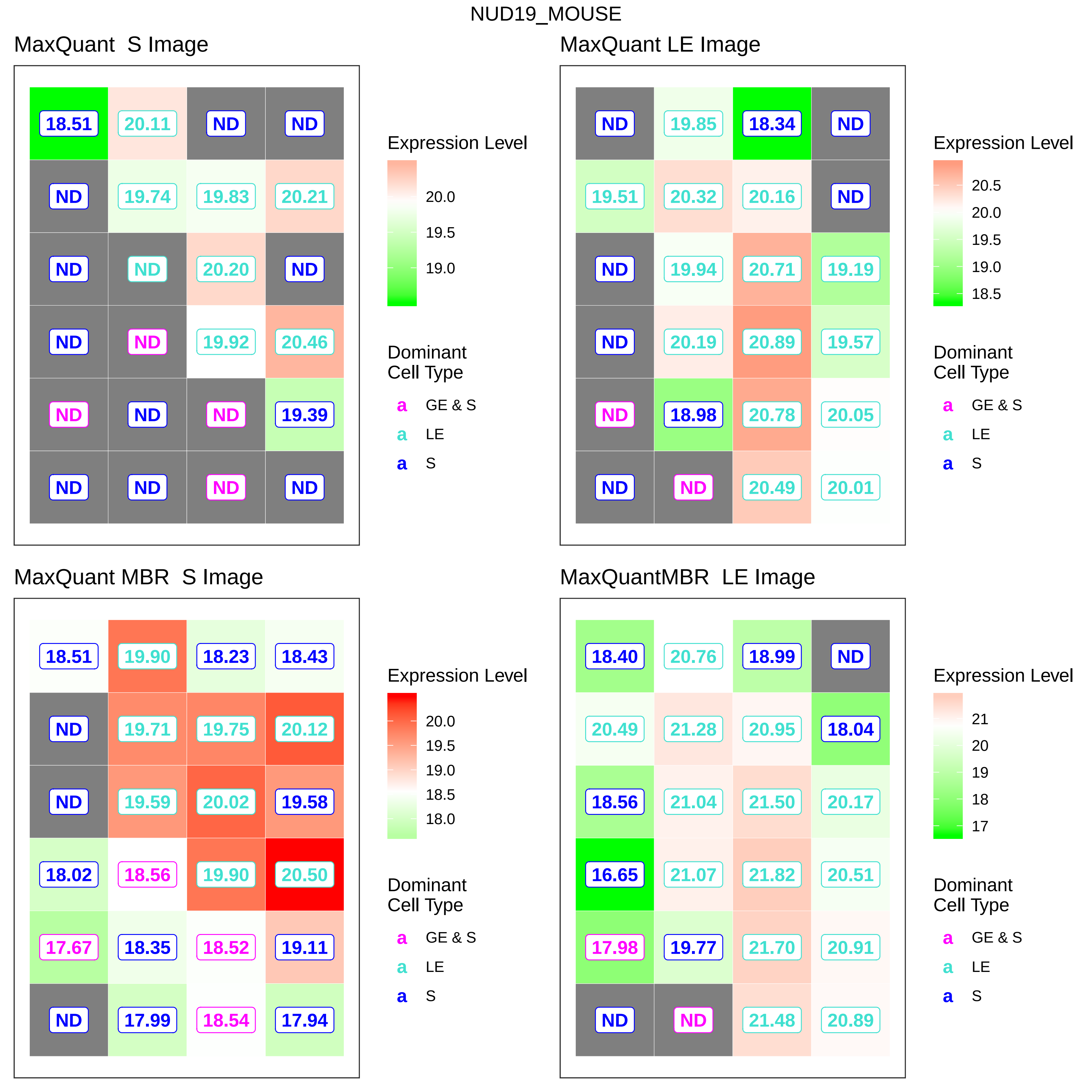
NUD19_MOUSE
MaxQuant S Image
18.51 20.11 ND ND
ND 19.74 19.83 20.21
ND ND 20.20 ND
ND ND 19.92 20.46
ND ND ND 19.39
ND ND ND ND
Expression Level
20.0
19.5
19.0
Dominant Cell Type
a GE & S
a LE
a S
MaxQuant LE Image
ND 19.85 18.34 ND
19.51 20.32 20.16 ND
ND 19.94 20.71 19.19
ND 20.19 20.89 19.57
ND 18.98 20.78 20.05
ND ND 20.49 20.01
Expression Level
20.5
20.0
19.5
19.0
18.5
Dominant Cell Type
a GE & S
a LE
a S
MaxQuant MBR S Image
18.51 19.90 18.23 18.43
ND 19.71 19.75 20.12
ND 19.59 20.02 19.58
18.02 18.56 19.90 20.50
17.67 18.35 18.52 19.11
ND 17.99 18.54 17.94
Expression Level
20.0
19.5
19.0
18.5
18.0
Dominant Cell Type
a GE & S
a LE
a S
MaxQuantMBR LE Image
18.40 20.76 18.99 ND
20.49 21.28 20.95 18.04
18.56 21.04 21.50 20.17
16.65 21.07 21.82 20.51
17.98 19.77 21.70 20.91
ND ND 21.48 20.89
Expression Level
21
20
19
18
17
Dominant Cell Type
a GE & S
a LE
a S

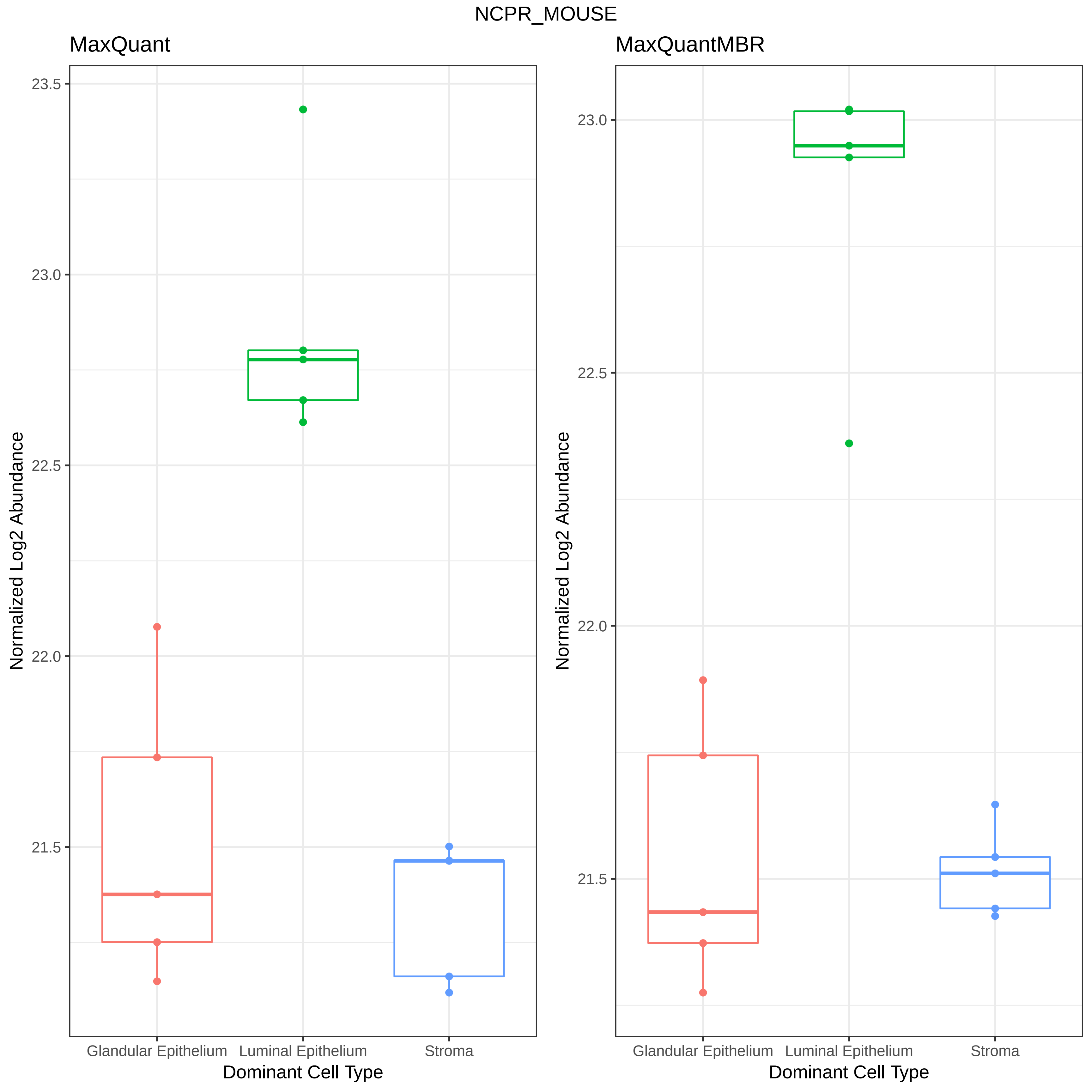

NCPR_MOUSE
MaxQuant
MaxQuantMBR
Normalized Log2 Abundance
23.5
23.0
22.5
22.0
21.5
Glandular Epithelium
Luminal Epithelium
Stroma
Dominant Cell Type

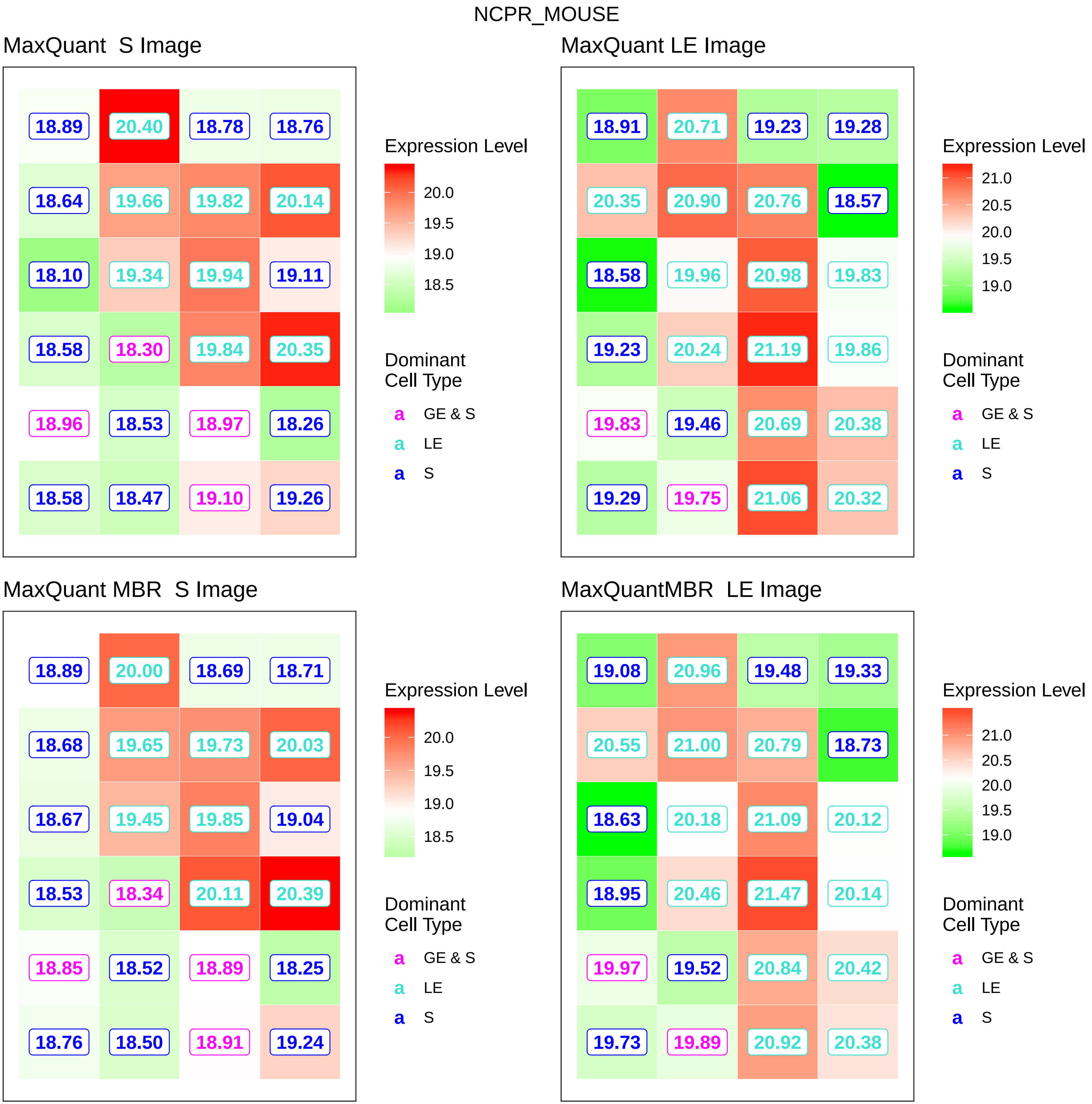
NCPR_MOUSE
MaxQuant S Image
18.89 20.40 18.78 18.76
18.64 19.66 19.82 20.14
18.10 19.34 19.94 19.11
18.58 18.30 19.84 20.35
18.96 18.53 18.97 18.26
18.58 18.47 19.10 19.26
Expression Level
20.0
19.5
19.0
18.5
Dominant Cell Type
a GE & S
a LE
a S
MaxQuant LE Image
18.91 20.71 19.23 19.28
20.35 20.90 20.76 18.57
18.58 19.96 20.98 19.83
19.23 20.24 21.19 19.86
19.83 19.46 20.69 20.38
19.29 19.75 21.06 20.32
Expression Level
21.0
20.5
20.0
19.5
19.0
Dominant Cell Type
a GE & S
a LE
a S
MaxQuant MBR S Image
18.89 20.00 18.69 18.71
18.68 19.65 19.73 20.03
18.67 19.45 19.85 19.04
18.53 18.34 20.11 20.39
18.85 18.52 18.89 18.25
18.76 18.50 18.91 19.24
Expression Level
20.0
19.5
19.0
18.5
Dominant Cell Type
a GE & S
a LE
a S
MaxQuantMBR LE Image
19.08 20.96 19.48 19.33
20.55 21.00 20.79 18.73
18.63 20.18 21.09 20.12
18.95 20.46 21.47 20.14
19.97 19.52 20.84 20.42
19.73 19.89 20.92 20.38
Expression Level
21.0
20.5
20.0
19.5
19.0
Dominant Cell Type
a GE & S
a LE
a S

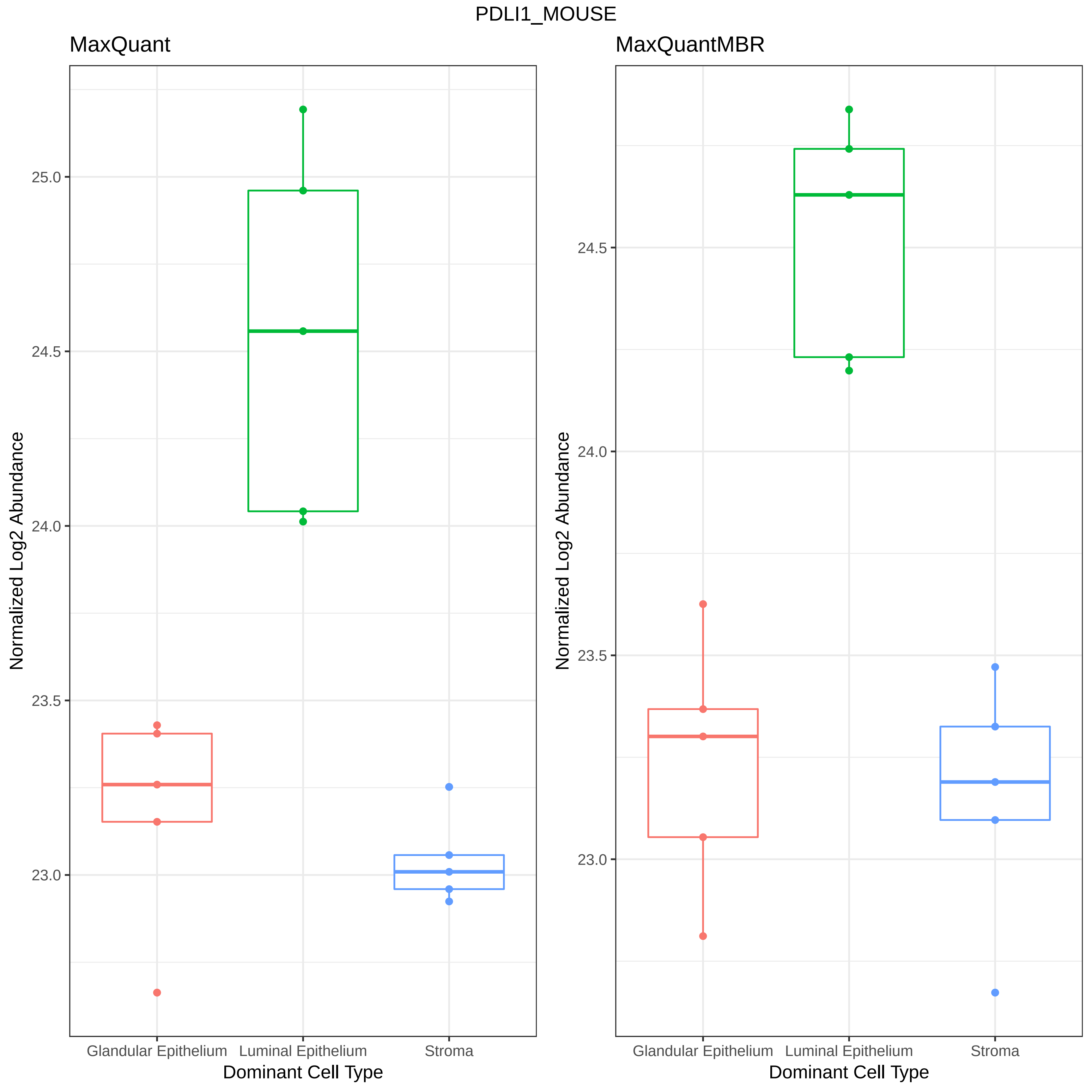

PDLI1_MOUSE
MaxQuant
MaxQuantMBR
Normalized Log2 Abundance
25.0
24.5
24.0
23.5
23.0
Glandular Epithelium
Luminal Epithelium
Stroma
Dominant Cell Type

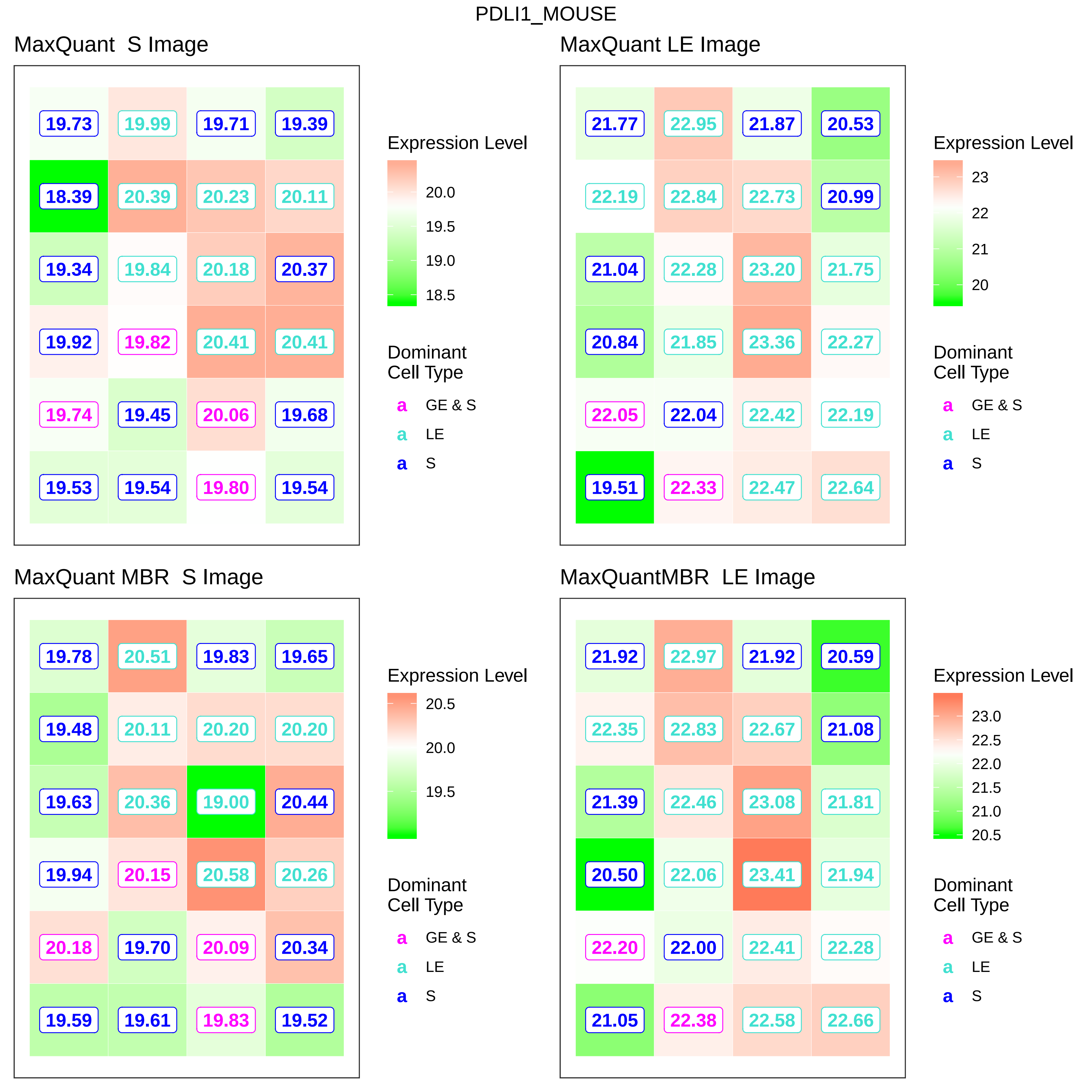
PDLI1_MOUSE
MaxQuant S Image
19.73 19.99 19.71 19.39
18.39 20.39 20.23 20.11
19.34 19.84 20.18 20.37
19.92 19.82 20.41 20.41
19.74 19.45 20.06 19.68
19.53 19.54 19.80 19.54
Expression Level
20.0
19.5
19.0
18.5
Dominant Cell Type
a GE & S
a LE
a S
MaxQuant LE Image
21.77 22.95 21.87 20.53
22.19 22.84 22.73 20.99
21.04 22.28 23.20 21.75
20.84 21.85 23.36 22.27
22.05 22.04 22.42 22.19
19.51 22.33 22.47 22.64
Expression Level
23
22
21
20
Dominant Cell Type
a GE & S
a LE
a S
MaxQuant MBR S Image
19.78 20.51 19.83 19.65
19.48 20.11 20.20 20.20
19.63 20.36 19.00 20.44
19.94 20.15 20.58 20.26
20.18 19.70 20.09 20.34
19.59 19.61 19.83 19.52
Expression Level
20.5
20.0
19.5
Dominant Cell Type
a GE & S
a LE
a S
MaxQuantMBR LE Image
21.92 22.97 21.92 20.59
22.35 22.83 22.67 21.08
21.39 22.46 23.08 21.81
20.50 22.06 23.41 21.94
22.20 22.00 22.41 22.28
21.05 22.38 22.58 22.66
Expression Level
23.0
22.5
22.0
21.5
21.0
20.5
Dominant Cell Type
a GE & S
a LE
a S

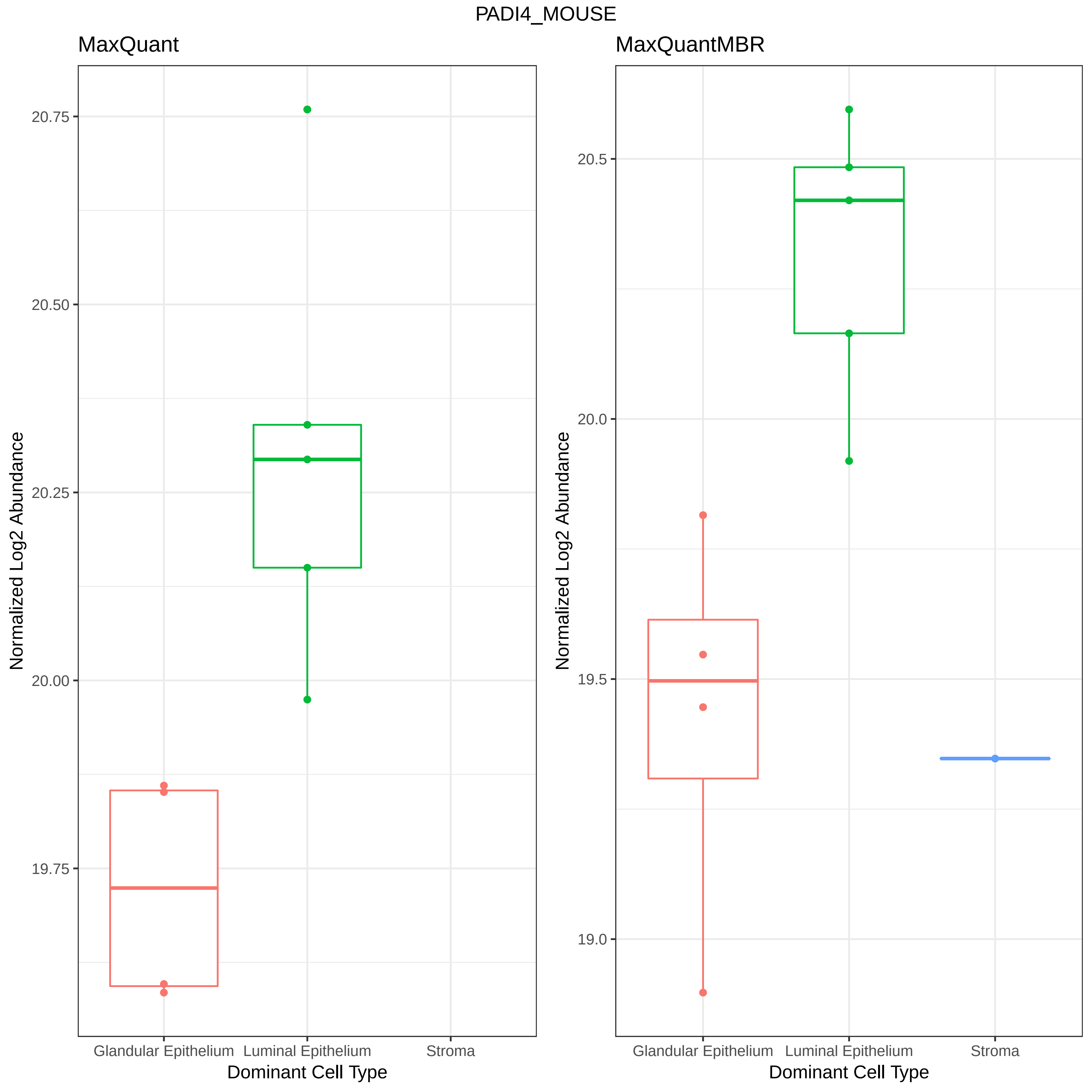

PADI4_MOUSE
MaxQuant
MaxQuantMBR
Normalized Log2 Abundance
20.75
20.50
20.25
20.00
19.75
20.5
20.0
19.5
19.0
Glandular Epithelium
Luminal Epithelium
Stroma
Dominant Cell Type

PADI4_MOUSE

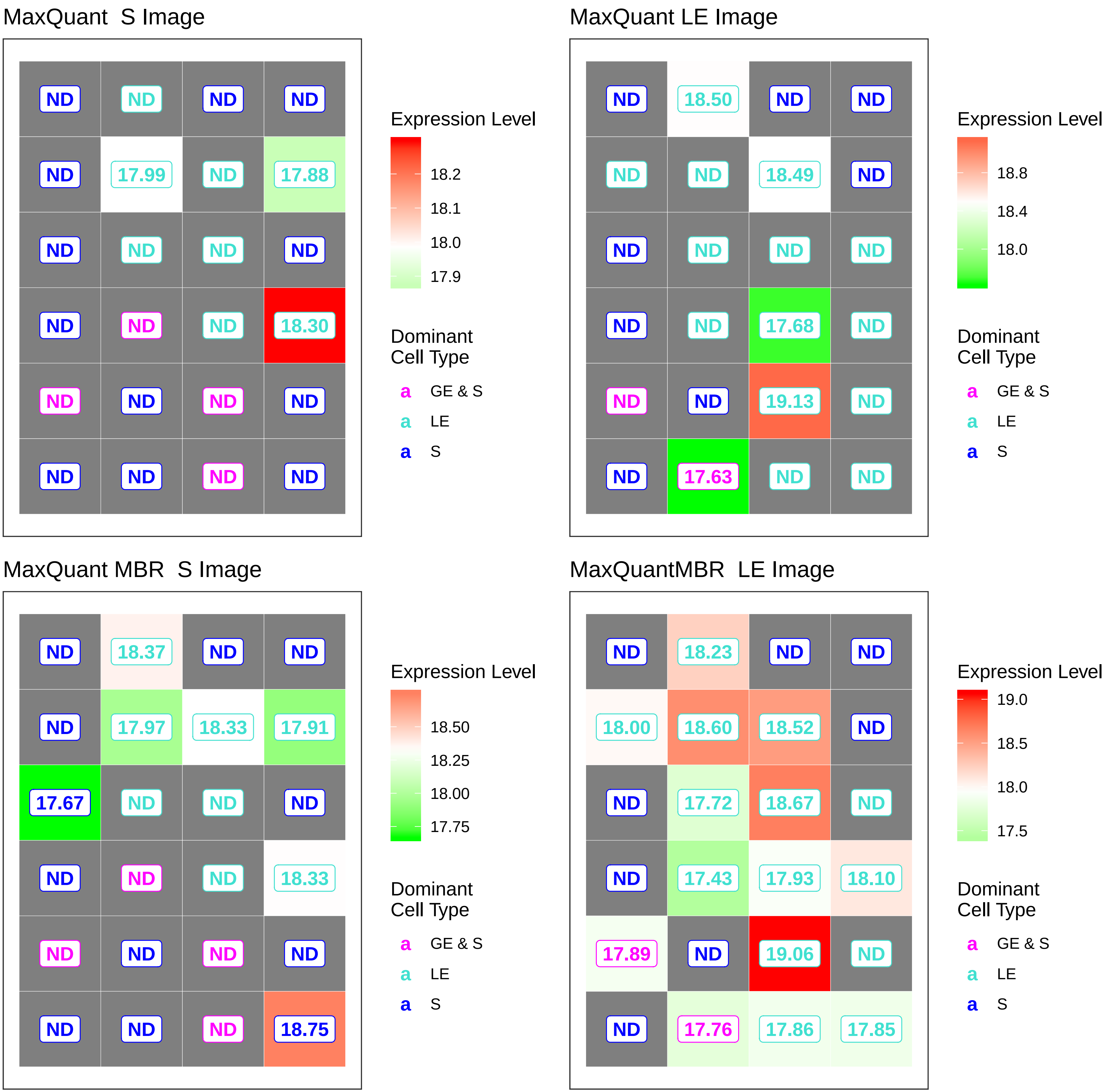

MaxQuant S Image
ND
ND
ND
ND
ND
17.99
ND
17.88
ND
ND
ND
ND
ND
ND
ND
18.30
ND
ND
ND
ND
ND
ND
ND
ND
Expression Level
18.2
18.1
18.0
17.9
Dominant Cell Type
a GE & S
a LE
a S
MaxQuant LE Image
ND
18.50
ND
ND
ND
ND
18.49
ND
ND
ND
ND
ND
ND
ND
17.68
ND
ND
ND
19.13
ND
ND
17.63
ND
ND
Expression Level
18.8
18.4
18.0
Dominant Cell Type
a GE & S
a LE
a S
MaxQuant MBR S Image
ND
18.37
ND
ND
ND
17.97
18.33
17.91
17.67
ND
ND
ND
ND
ND
ND
18.33
ND
ND
ND
ND
ND
ND
ND
18.75
Expression Level
18.50
18.25
18.00
17.75
Dominant Cell Type
a GE & S
a LE
a S
MaxQuantMBR LE Image
ND
18.23
ND
ND
18.00
18.60
18.52
ND
ND
17.72
18.67
ND
ND
17.43
17.93
18.10
17.89
ND
19.06
ND
ND
17.76
17.86
17.85
Expression Level
19.0
18.5
18.0
17.5
Dominant Cell Type
a GE & S
a LE
a S

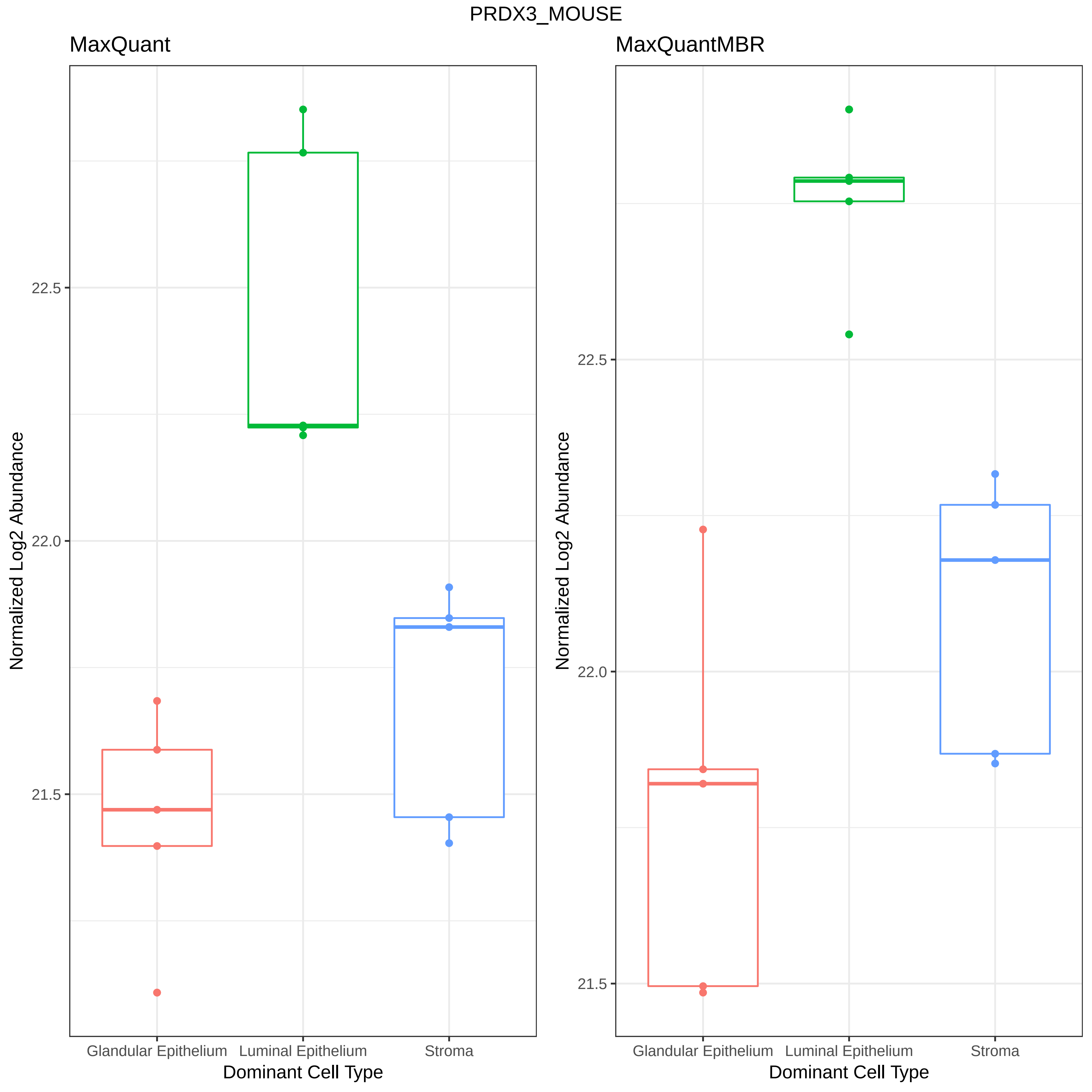

PRDX3_MOUSE
MaxQuant
MaxQuantMBR
Normalized Log2 Abundance
22.5
22.0
21.5
Glandular Epithelium
Luminal Epithelium
Stroma
Dominant Cell Type

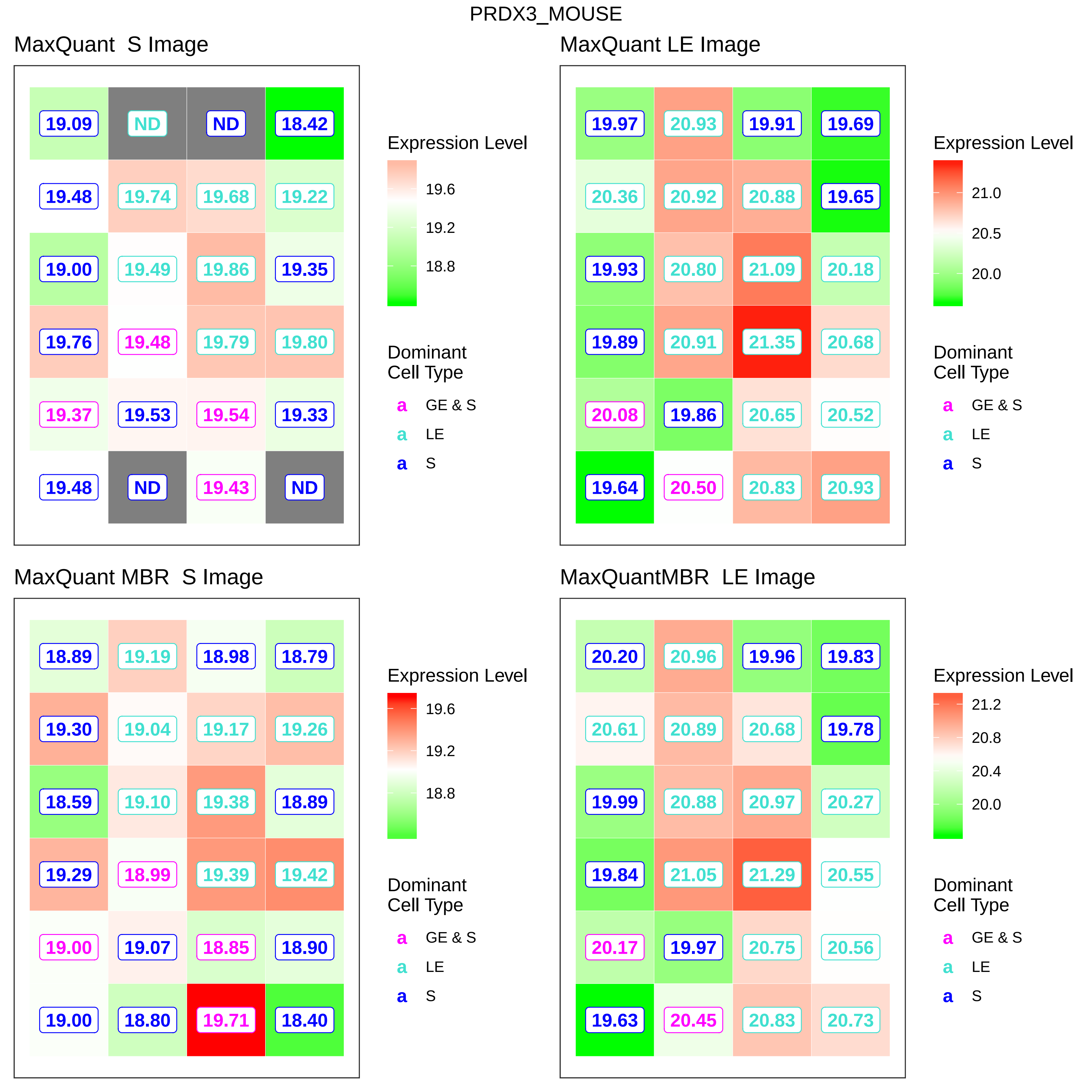
PRDX3_MOUSE
MaxQuant S Image
19.09 ND ND 18.42
19.48 19.74 19.68 19.22
19.00 19.49 19.86 19.35
19.76 19.48 19.79 19.80
19.37 19.53 19.54 19.33
19.48 ND 19.43 ND
Expression Level
19.6
19.2
18.8
Dominant Cell Type
a GE & S
a LE
a S
MaxQuant LE Image
19.97 20.93 19.91 19.69
20.36 20.92 20.88 19.65
19.93 20.80 21.09 20.18
19.89 20.91 21.35 20.68
20.08 19.86 20.65 20.52
19.64 20.50 20.83 20.93
Expression Level
21.0
20.5
20.0
Dominant Cell Type
a GE & S
a LE
a S
MaxQuant MBR S Image
18.89 19.19 18.98 18.79
19.30 19.04 19.17 19.26
18.59 19.10 19.38 18.89
19.29 18.99 19.39 19.42
19.00 19.07 18.85 18.90
19.00 18.80 19.71 18.40
Expression Level
19.6
19.2
18.8
Dominant Cell Type
a GE & S
a LE
a S
MaxQuantMBR LE Image
20.20 20.96 19.96 19.83
20.61 20.89 20.68 19.78
19.99 20.88 20.97 20.27
19.84 21.05 21.29 20.55
20.17 19.97 20.75 20.56
19.63 20.45 20.83 20.73
Expression Level
21.2
20.8
20.4
20.0
Dominant Cell Type
a GE & S
a LE
a S

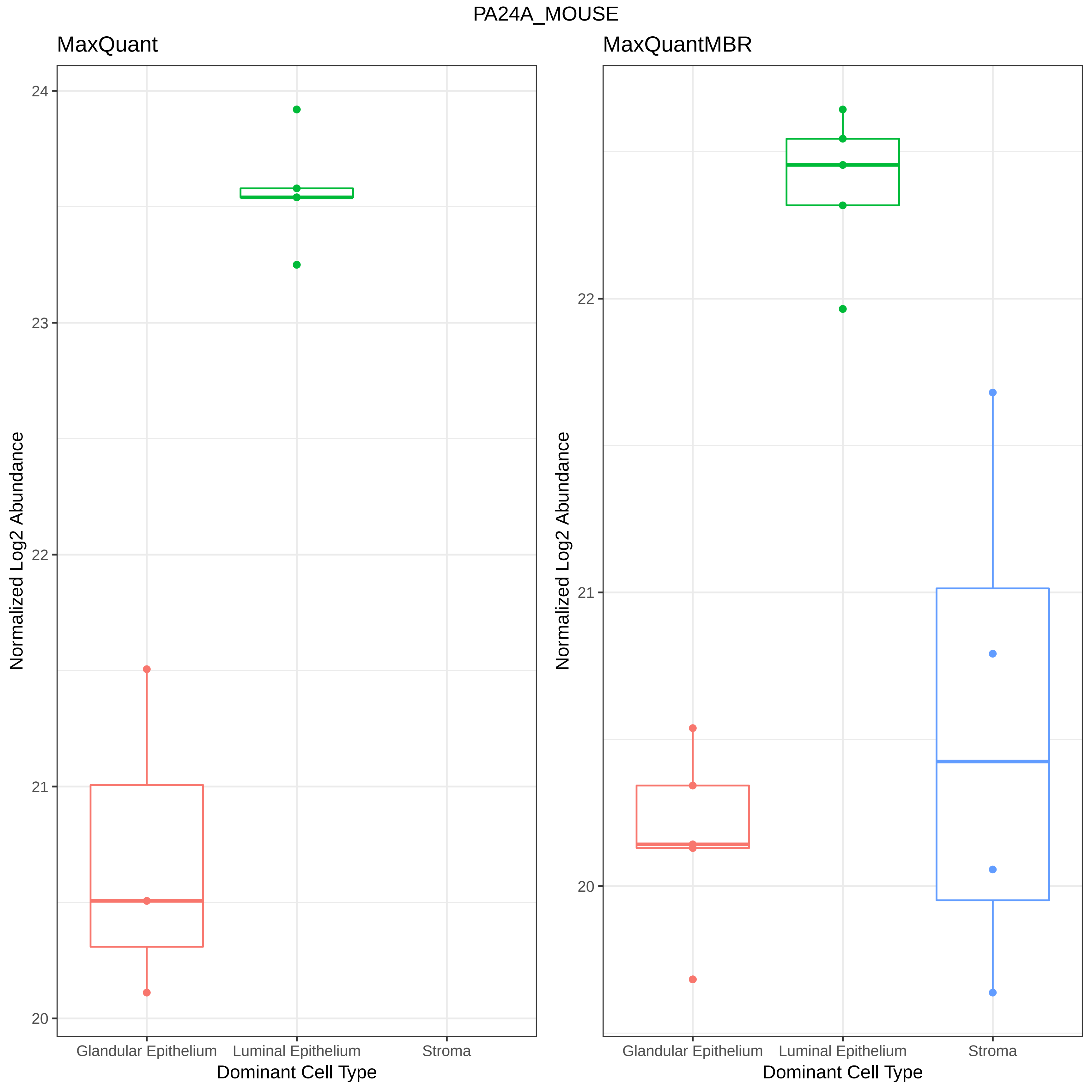

PA24A_MOUSE
MaxQuant
MaxQuantMBR
Normalized Log2 Abundance
24
23
22
21
20
Glandular Epithelium
Luminal Epithelium
Stroma
Dominant Cell Type

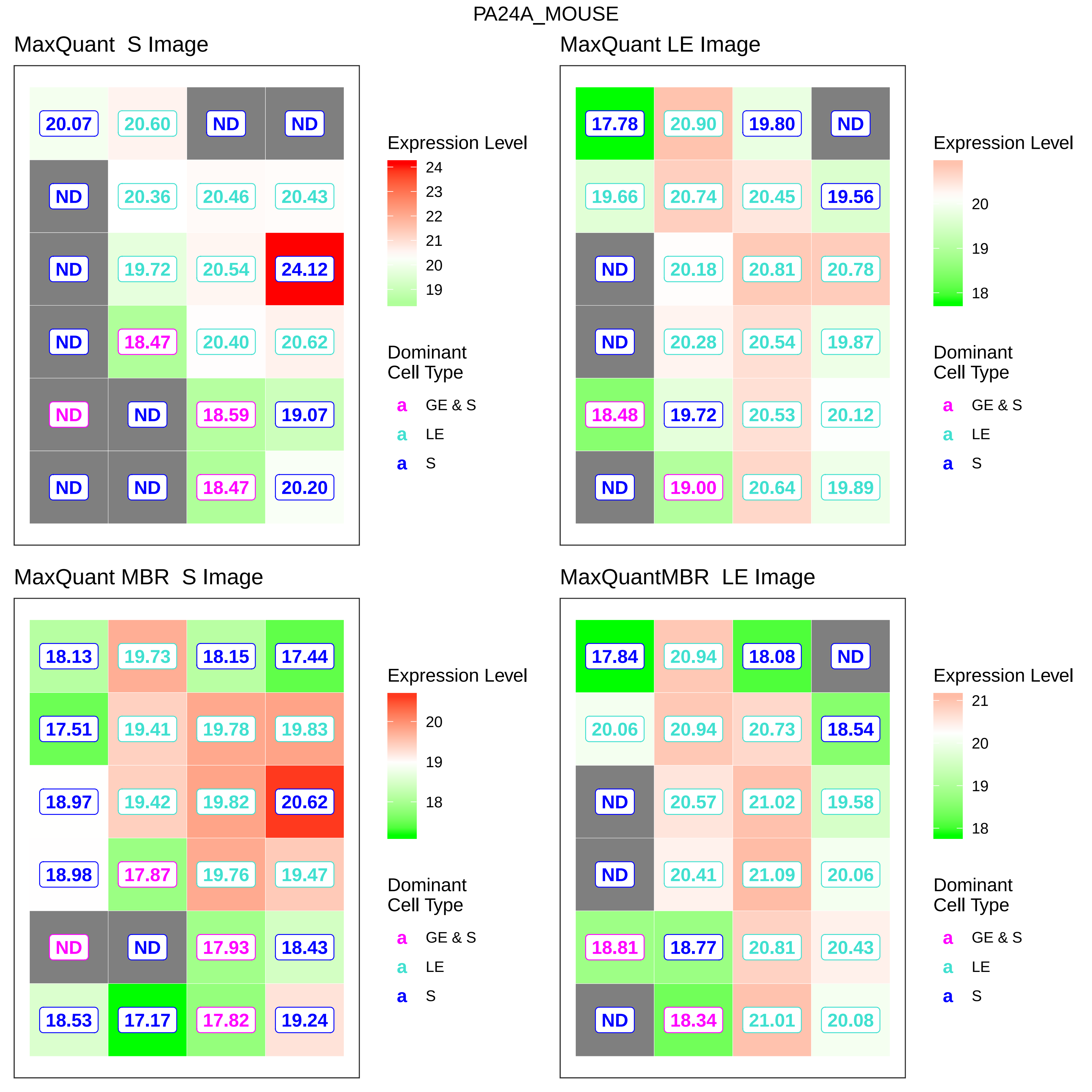
PA24A_MOUSE
MaxQuant S Image
20.07 20.60 ND ND
ND 20.36 20.46 20.43
ND 19.72 20.54 24.12
ND 18.47 20.40 20.62
ND ND 18.59 19.07
ND ND 18.47 20.20
Expression Level
24 23 22 21 20 19
Dominant Cell Type
a GE & S
a LE
a S
MaxQuant LE Image
17.78 20.90 19.80 ND
19.66 20.74 20.45 19.56
ND 20.18 20.81 20.78
ND 20.28 20.54 19.87
18.48 19.72 20.53 20.12
ND 19.00 20.64 19.89
Expression Level
20 19 18
Dominant Cell Type
a GE & S
a LE
a S
MaxQuant MBR S Image
18.13 19.73 18.15 17.44
17.51 19.41 19.78 19.83
18.97 19.42 19.82 20.62
18.98 17.87 19.76 19.47
ND ND 17.93 18.43
18.53 17.17 17.82 19.24
Expression Level
20 19 18
Dominant Cell Type
a GE & S
a LE
a S
MaxQuantMBR LE Image
17.84 20.94 18.08 ND
20.06 20.94 20.73 18.54
ND 20.57 21.02 19.58
ND 20.41 21.09 20.06
18.81 18.77 20.81 20.43
ND 18.34 21.01 20.08
Expression Level
21 20 19 18
Dominant Cell Type
a GE & S
a LE
a S

PLS1_MOUSE

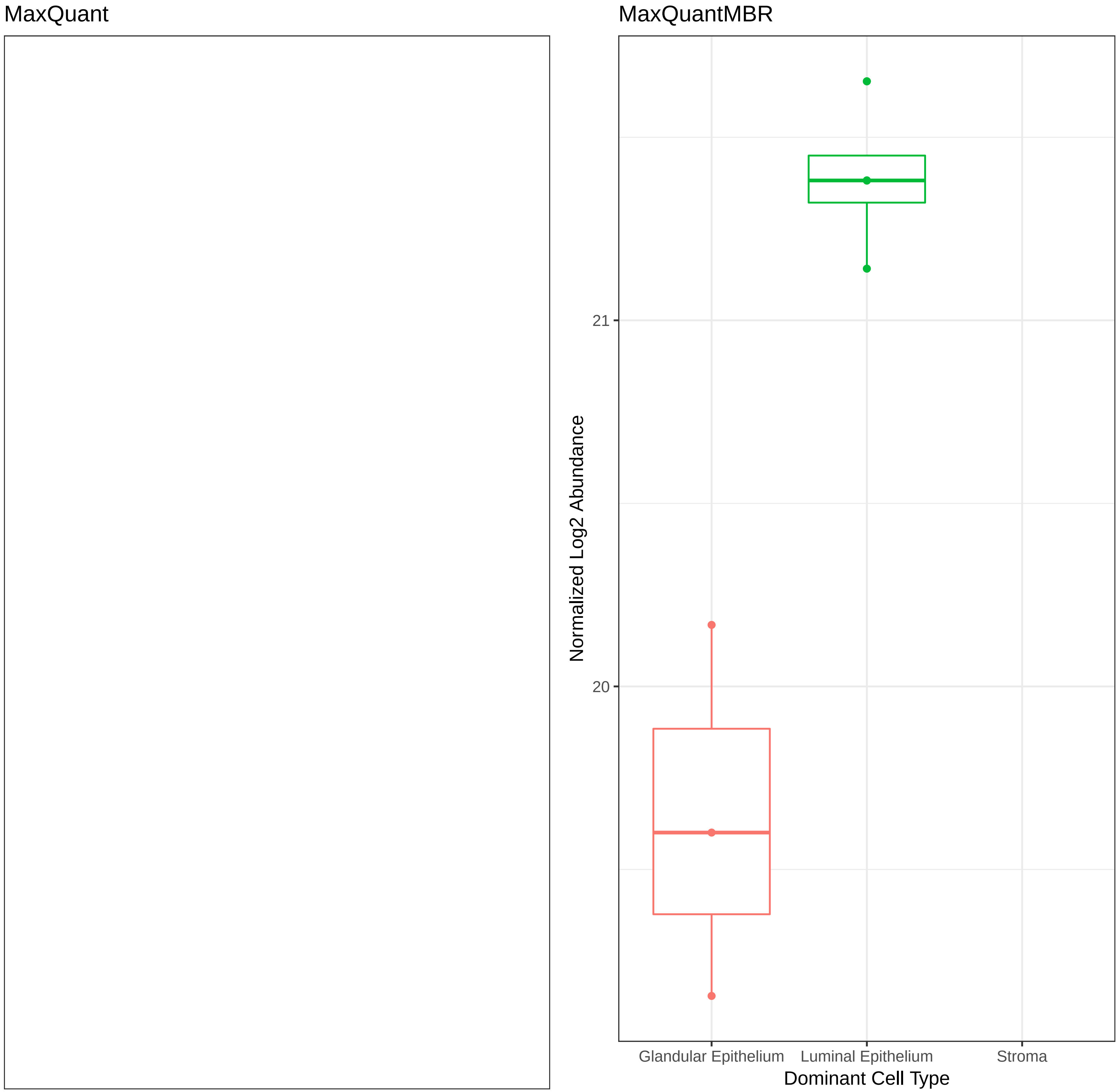

MaxQuant
MaxQuantMBR
Normalized Log2 Abundance
21
20
Glandular Epithelium
Luminal Epithelium
Stroma
Dominant Cell Type

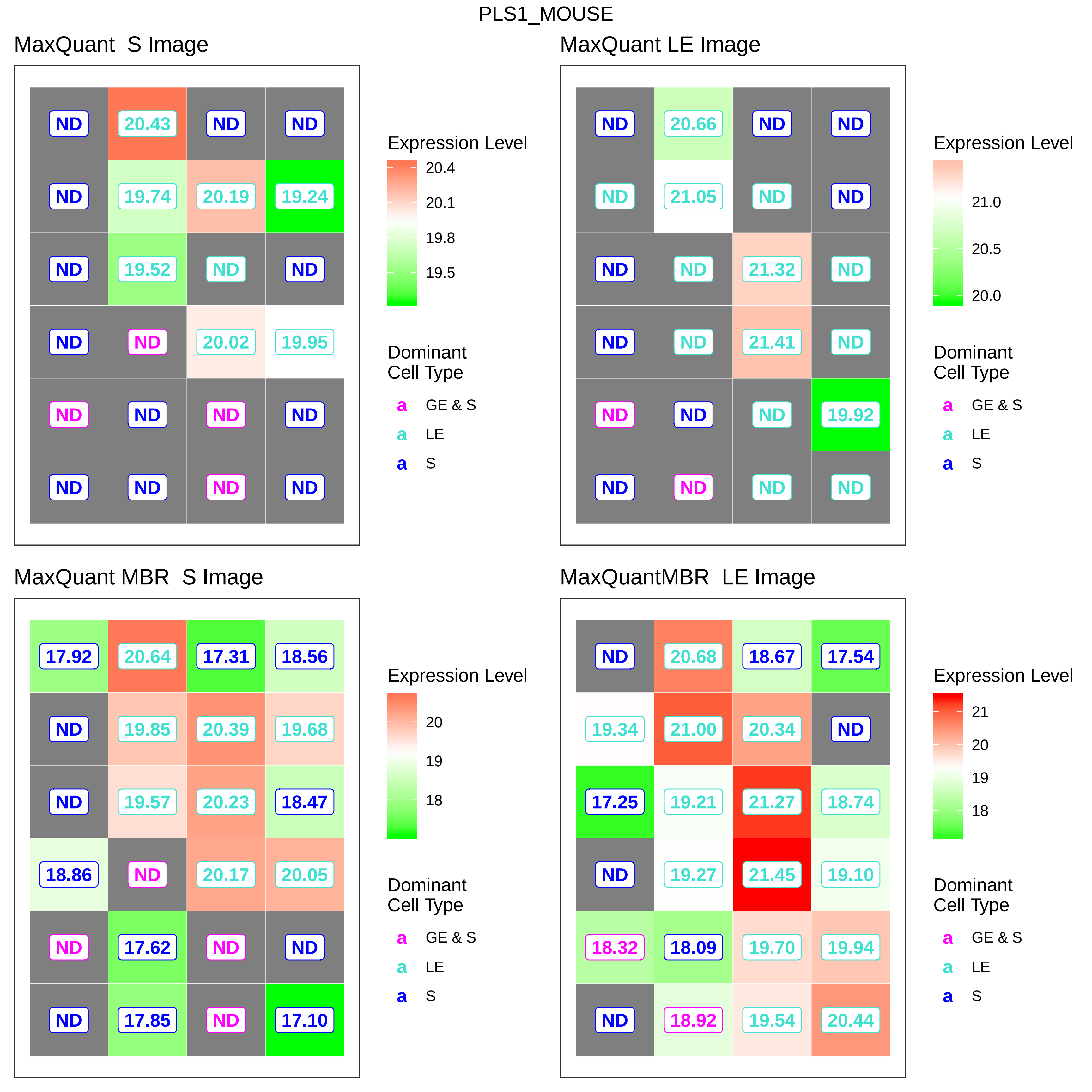
PLS1_MOUSE
MaxQuant S Image
ND 20.43 ND ND
ND 19.74 20.19 19.24
ND 19.52 ND ND
ND ND 20.02 19.95
ND ND ND ND
ND ND ND ND
Expression Level
20.4
20.1
19.8
19.5
Dominant Cell Type
a GE & S
a LE
a S
MaxQuant LE Image
ND 20.66 ND ND
ND 21.05 ND ND
ND ND 21.32 ND
ND ND 21.41 ND
ND ND ND 19.92
ND ND ND ND
Expression Level
21.0
20.5
20.0
Dominant Cell Type
a GE & S
a LE
a S
MaxQuant MBR S Image
17.92 20.64 17.31 18.56
ND 19.85 20.39 19.68
ND 19.57 20.23 18.47
18.86 ND 20.17 20.05
ND 17.62 ND ND
ND 17.85 ND 17.10
Expression Level
20
19
18
Dominant Cell Type
a GE & S
a LE
a S
MaxQuantMBR LE Image
ND 20.68 18.67 17.54
19.34 21.00 20.34 ND
17.25 19.21 21.27 18.74
ND 19.27 21.45 19.10
18.32 18.09 19.70 19.94
ND 18.92 19.54 20.44
Expression Level
21
20
19
18
Dominant Cell Type
a GE & S
a LE
a S

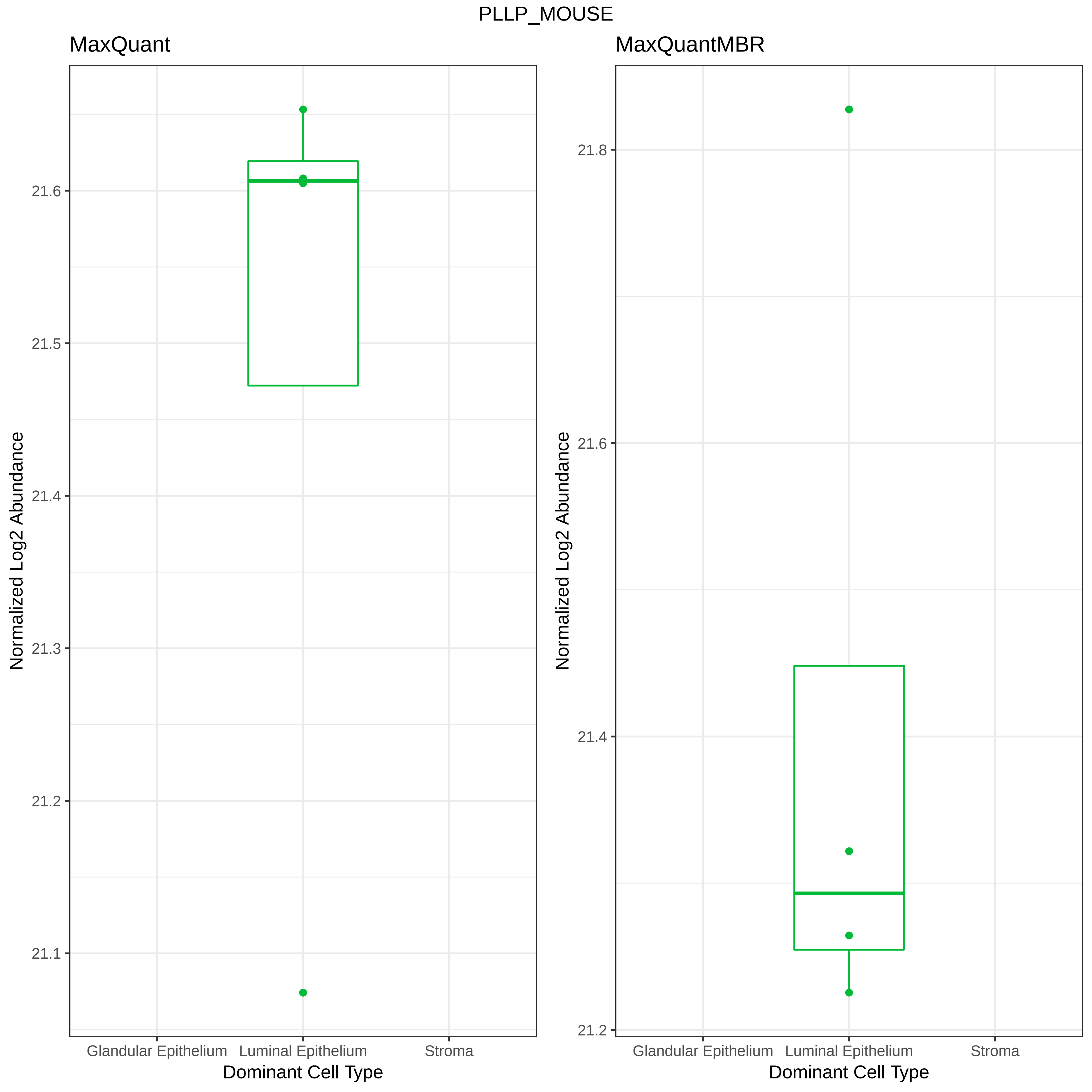
PLLP_MOUSE
MaxQuant
MaxQuantMBR
Normalized Log2 Abundance
21.1
21.2
21.3
21.4
21.5
21.6
21.8
Glandular Epithelium
Luminal Epithelium
Stroma
Dominant Cell Type

PLLP_MOUSE

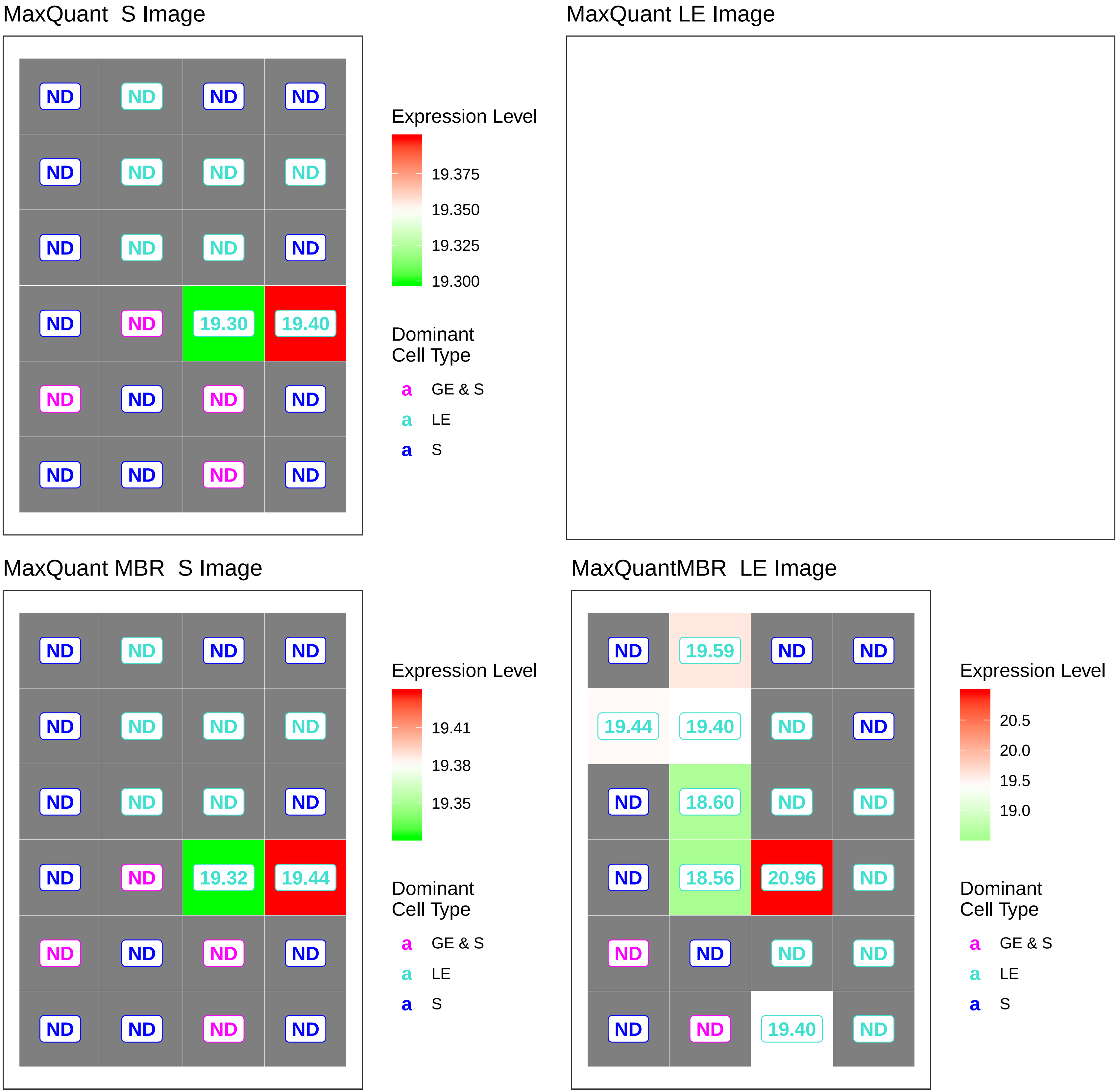
MaxQuant S Image
ND ND ND ND
ND ND ND ND
ND ND ND ND
ND ND 19.30 19.40
ND ND ND ND
ND ND ND ND
Expression Level
19.375
19.350
19.325
19.300
Dominant Cell Type
a GE & S
a LE
a S
MaxQuant LE Image
MaxQuant MBR S Image
ND ND ND ND
ND ND ND ND
ND ND ND ND
ND ND 19.32 19.44
ND ND ND ND
ND ND ND ND
Expression Level
19.41
19.38
19.35
Dominant Cell Type
a GE & S
a LE
a S
MaxQuantMBR LE Image
ND 19.59 ND ND
19.44 19.40 ND ND
ND 18.60 ND ND
ND 18.56 20.96 ND
ND ND ND ND
ND ND 19.40 ND
Expression Level
20.5
20.0
19.5
19.0
Dominant Cell Type
a GE & S
a LE
a S

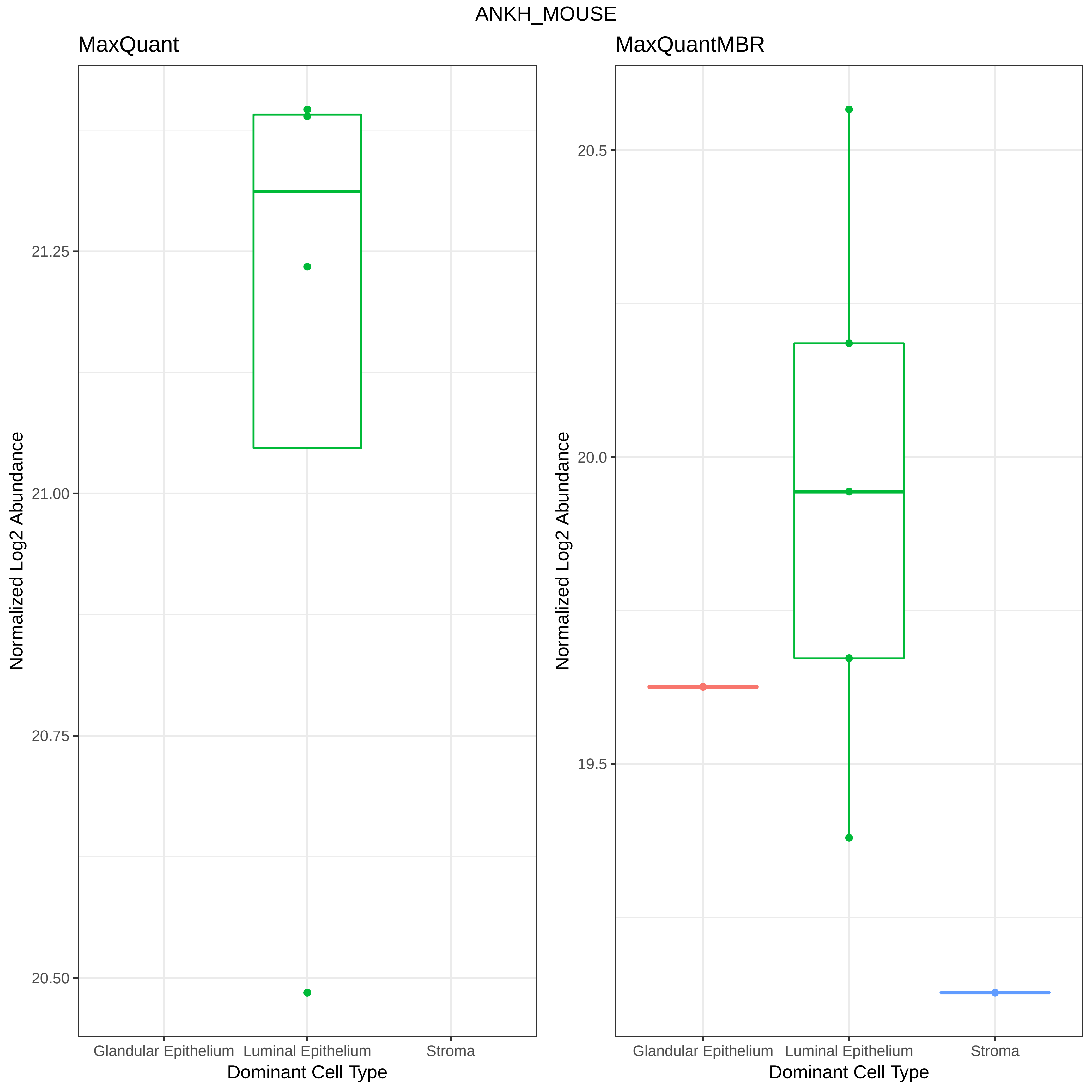

ANKH_MOUSE
MaxQuant
MaxQuantMBR
Normalized Log2 Abundance
21.25
21.00
20.75
20.50
20.5
20.0
19.5
Glandular Epithelium
Luminal Epithelium
Stroma
Dominant Cell Type

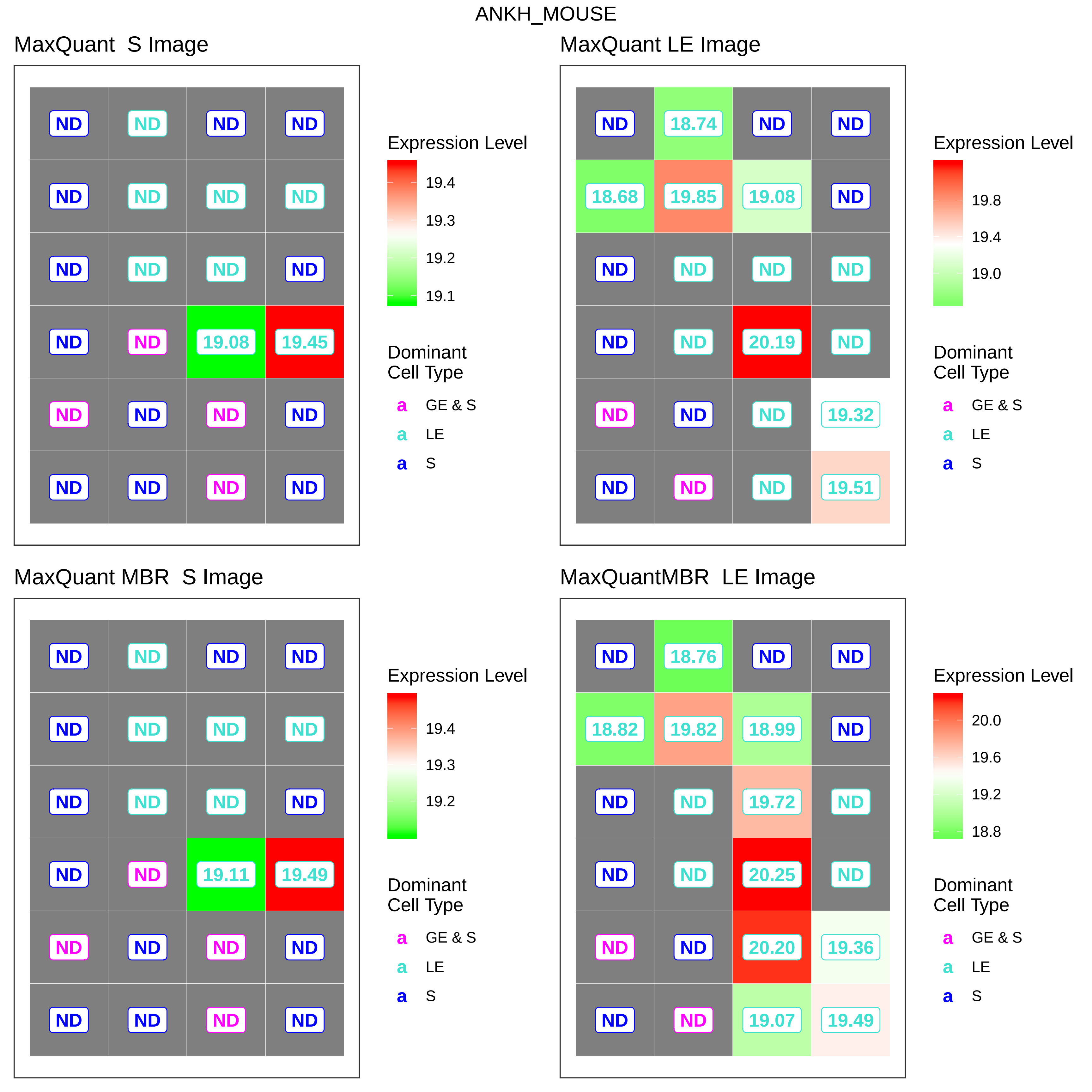
ANKH_MOUSE
MaxQuant S Image
MaxQuant LE Image
MaxQuant MBR S Image
MaxQuantMBR LE Image
Expression Level
Dominant Cell Type
GE & S
LE
S
ND
18.74
18.68
19.85
19.08
20.19
19.32
19.51
19.45
19.11
19.49
18.76
18.82
19.82
18.99
19.72
20.25
20.20
19.36
19.07

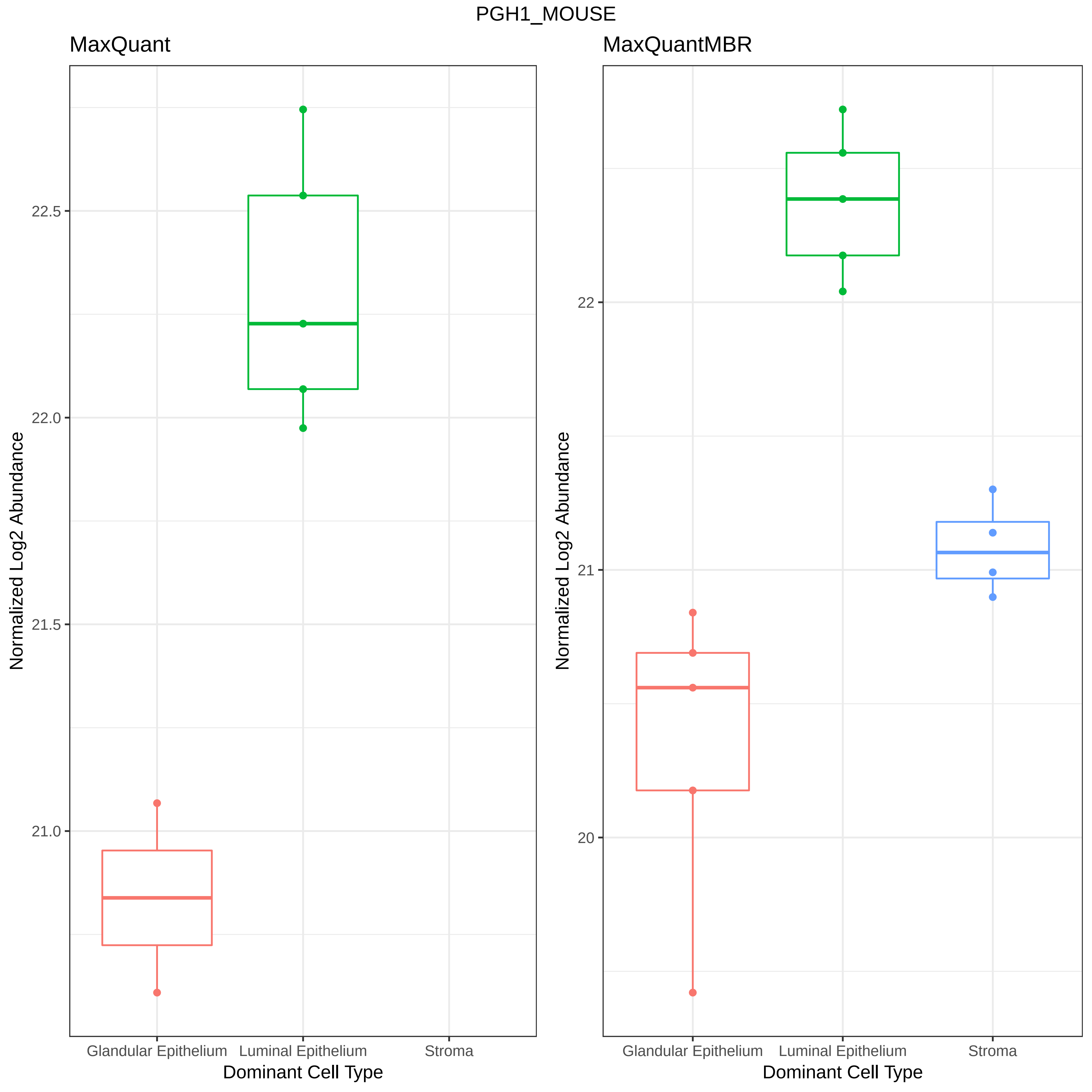

PGH1_MOUSE
MaxQuant
MaxQuantMBR
Normalized Log2 Abundance
22.5
22.0
21.5
21.0
22
21
20
Glandular Epithelium
Luminal Epithelium
Stroma
Dominant Cell Type

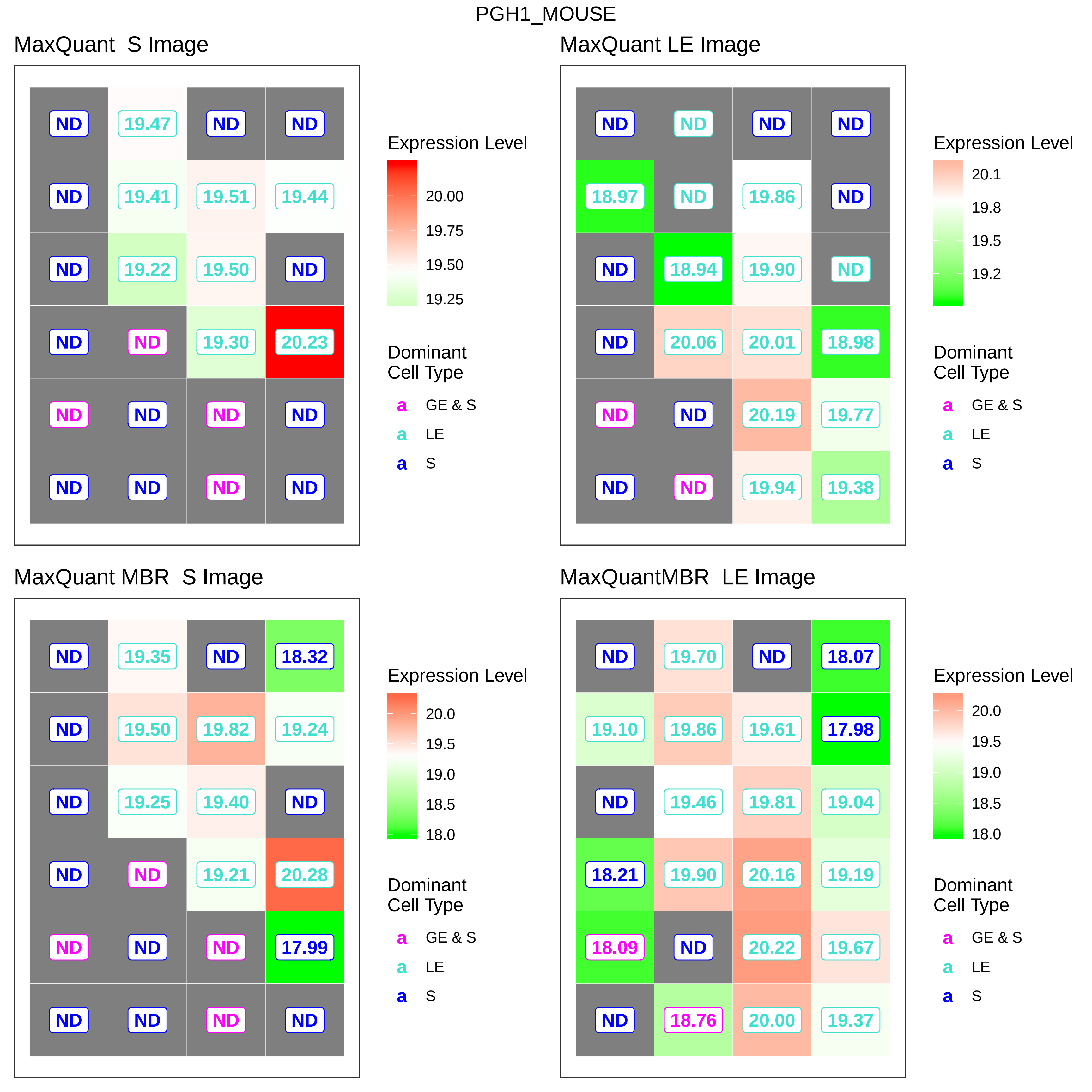
PGH1_MOUSE
MaxQuant S Image
ND
19.47
ND
ND
ND
19.41
19.51
19.44
ND
19.22
19.50
ND
ND
ND
19.30
20.23
ND
ND
ND
ND
ND
ND
ND
ND
Expression Level
20.00
19.75
19.50
19.25
Dominant Cell Type
a GE & S
a LE
a S
MaxQuant LE Image
ND
ND
ND
ND
18.97
ND
19.86
ND
ND
18.94
19.90
ND
ND
20.06
20.01
18.98
ND
ND
20.19
19.77
ND
ND
19.94
19.38
Expression Level
20.1
19.8
19.5
19.2
Dominant Cell Type
a GE & S
a LE
a S
MaxQuant MBR S Image
ND
19.35
ND
18.32
ND
19.50
19.82
19.24
ND
19.25
19.40
ND
ND
ND
19.21
20.28
ND
ND
ND
17.99
ND
ND
ND
ND
Expression Level
20.0
19.5
19.0
18.5
18.0
Dominant Cell Type
a GE & S
a LE
a S
MaxQuantMBR LE Image
ND
19.70
ND
18.07
19.10
19.86
19.61
17.98
ND
19.46
19.81
19.04
18.21
19.90
20.16
19.19
18.09
ND
20.22
19.67
ND
18.76
20.00
19.37
Expression Level
20.0
19.5
19.0
18.5
18.0
Dominant Cell Type
a GE & S
a LE
a S

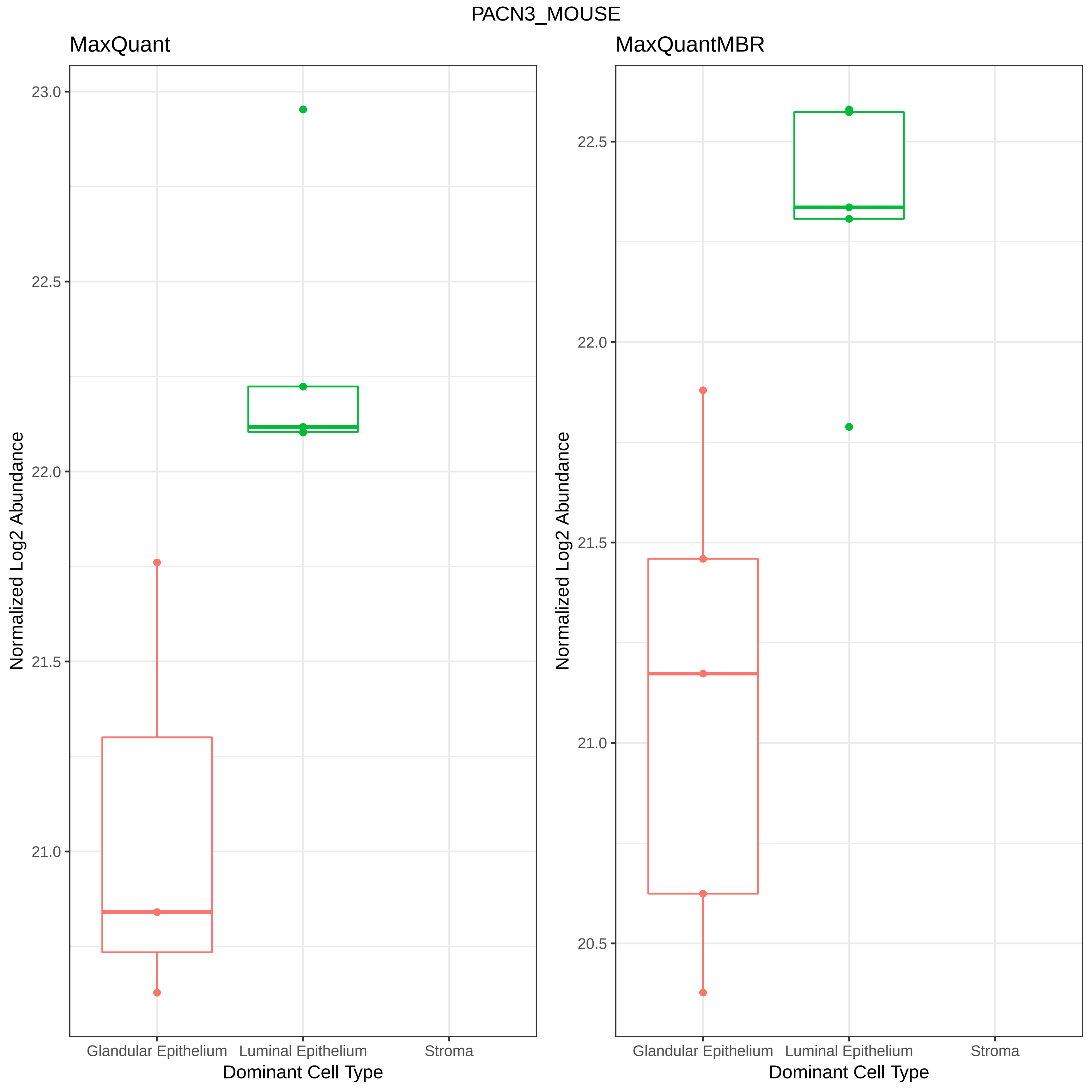

PACN3_MOUSE
MaxQuant
MaxQuantMBR
Normalized Log2 Abundance
23.0
22.5
22.0
21.5
21.0
20.5
Glandular Epithelium
Luminal Epithelium
Stroma
Dominant Cell Type

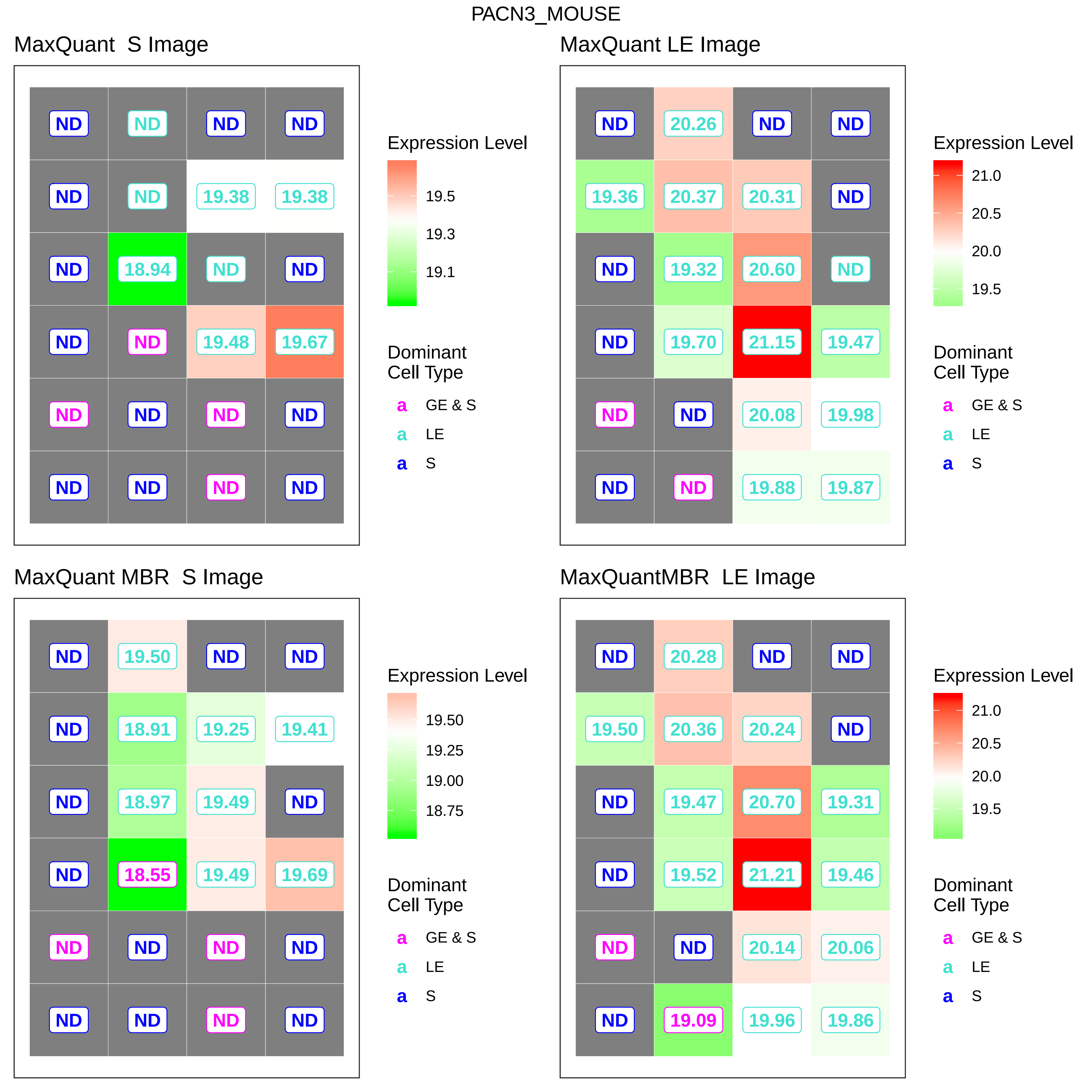

PACN3_MOUSE
MaxQuant S Image
ND ND ND ND
ND ND 19.38 19.38
ND 18.94 ND ND
ND ND 19.48 19.67
ND ND ND ND
ND ND ND ND
Expression Level
19.5
19.3
19.1
Dominant Cell Type
a GE & S
a LE
a S
MaxQuant LE Image
ND 20.26 ND ND
19.36 20.37 20.31 ND
ND 19.32 20.60 ND
ND 19.70 21.15 19.47
ND ND 20.08 19.98
ND ND 19.88 19.87
Expression Level
21.0
20.5
20.0
19.5
Dominant Cell Type
a GE & S
a LE
a S
MaxQuant MBR S Image
ND 19.50 ND ND
ND 18.91 19.25 19.41
ND 18.97 19.49 ND
ND 18.55 19.49 19.69
ND ND ND ND
ND ND ND ND
Expression Level
19.50
19.25
19.00
18.75
Dominant Cell Type
a GE & S
a LE
a S
MaxQuantMBR LE Image
ND 20.28 ND ND
19.50 20.36 20.24 ND
ND 19.47 20.70 19.31
ND 19.52 21.21 19.46
ND ND 20.14 20.06
ND 19.09 19.96 19.86
Expression Level
21.0
20.5
20.0
19.5
Dominant Cell Type
a GE & S
a LE
a S

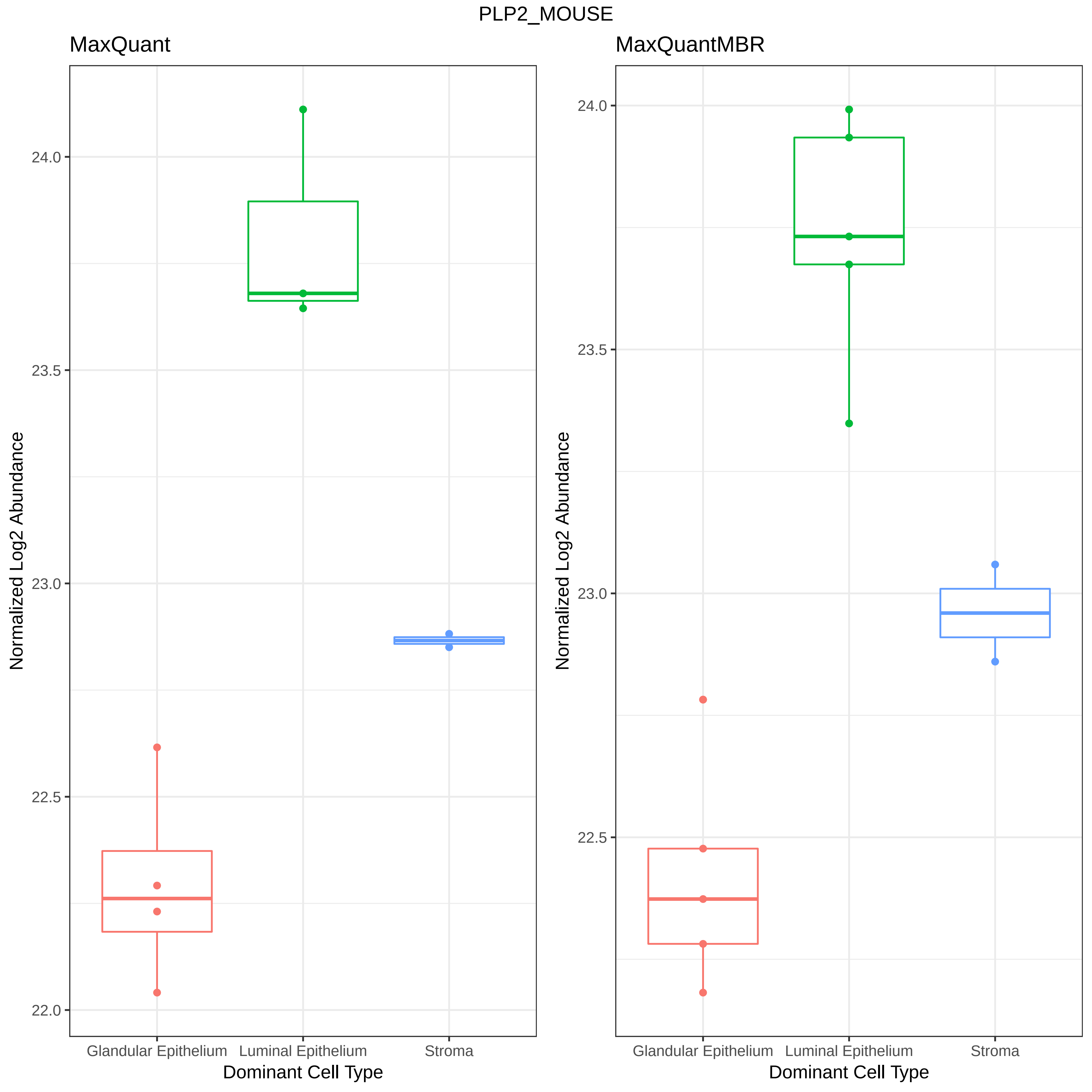

PLP2_MOUSE
MaxQuant
MaxQuantMBR
Normalized Log2 Abundance
24.0
23.5
23.0
22.5
22.0
Glandular Epithelium
Luminal Epithelium
Stroma
Dominant Cell Type

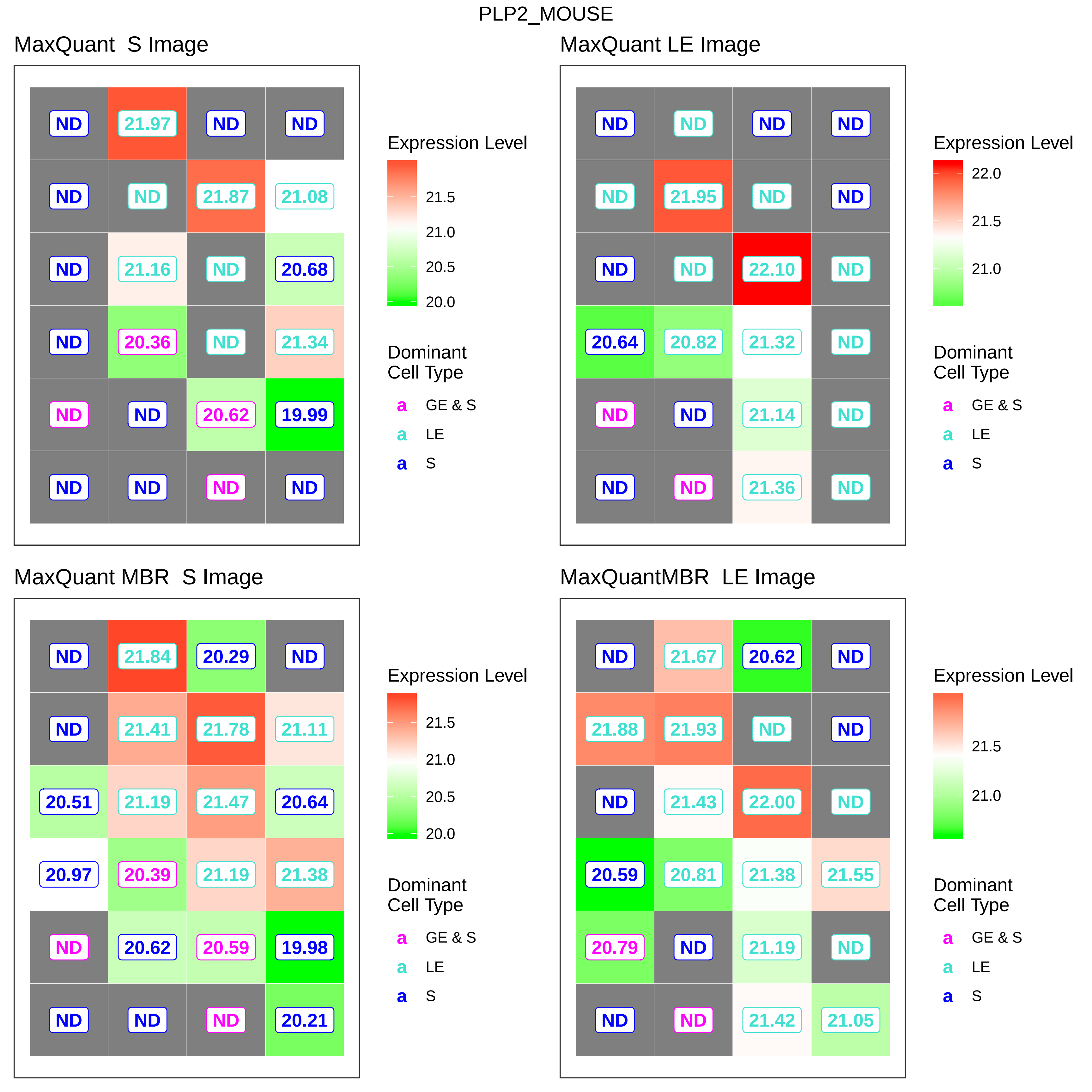
PLP2_MOUSE
MaxQuant S Image
ND 21.97 ND ND
ND ND 21.87 21.08
ND 21.16 ND 20.68
ND 20.36 ND 21.34
ND ND 20.62 19.99
ND ND ND ND
Expression Level
21.5
21.0
20.5
20.0
Dominant Cell Type
a GE & S
a LE
a S
MaxQuant LE Image
ND ND ND ND
ND 21.95 ND ND
ND ND 22.10 ND
20.64 20.82 21.32 ND
ND ND 21.14 ND
ND ND 21.36 ND
Expression Level
22.0
21.5
21.0
Dominant Cell Type
a GE & S
a LE
a S
MaxQuant MBR S Image
ND 21.84 20.29 ND
ND 21.41 21.78 21.11
20.51 21.19 21.47 20.64
20.97 20.39 21.19 21.38
ND 20.62 20.59 19.98
ND ND ND 20.21
Expression Level
21.5
21.0
20.5
20.0
Dominant Cell Type
a GE & S
a LE
a S
MaxQuantMBR LE Image
ND 21.67 20.62 ND
21.88 21.93 ND ND
ND 21.43 22.00 ND
20.59 20.81 21.38 21.55
20.79 ND 21.19 ND
ND ND 21.42 21.05
Expression Level
21.5
21.0
Dominant Cell Type
a GE & S
a LE
a S

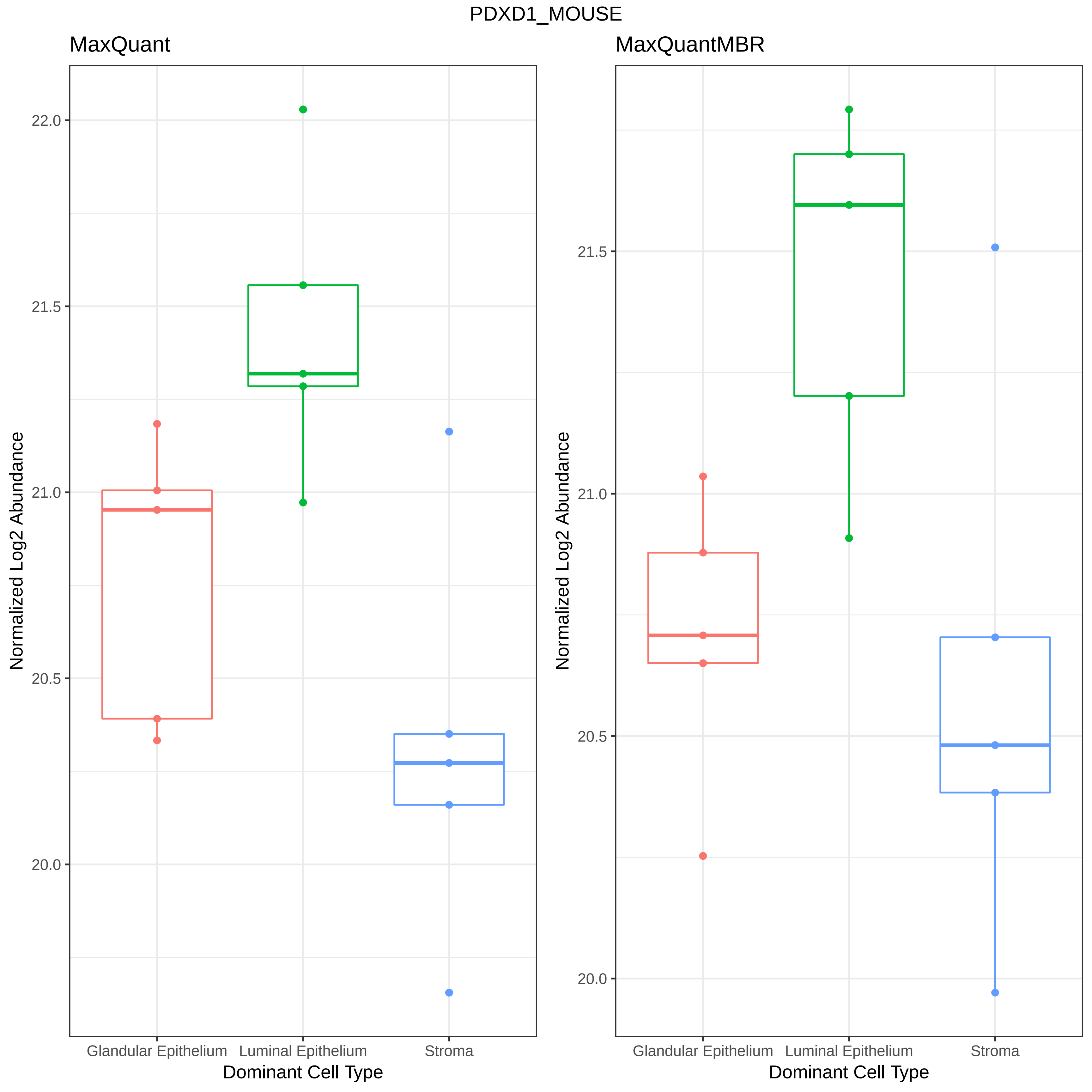
PDXD1_MOUSE
MaxQuant
MaxQuantMBR
Normalized Log2 Abundance
22.0
21.5
21.0
20.5
20.0
Glandular Epithelium
Luminal Epithelium
Stroma
Dominant Cell Type

PDXD1_MOUSE

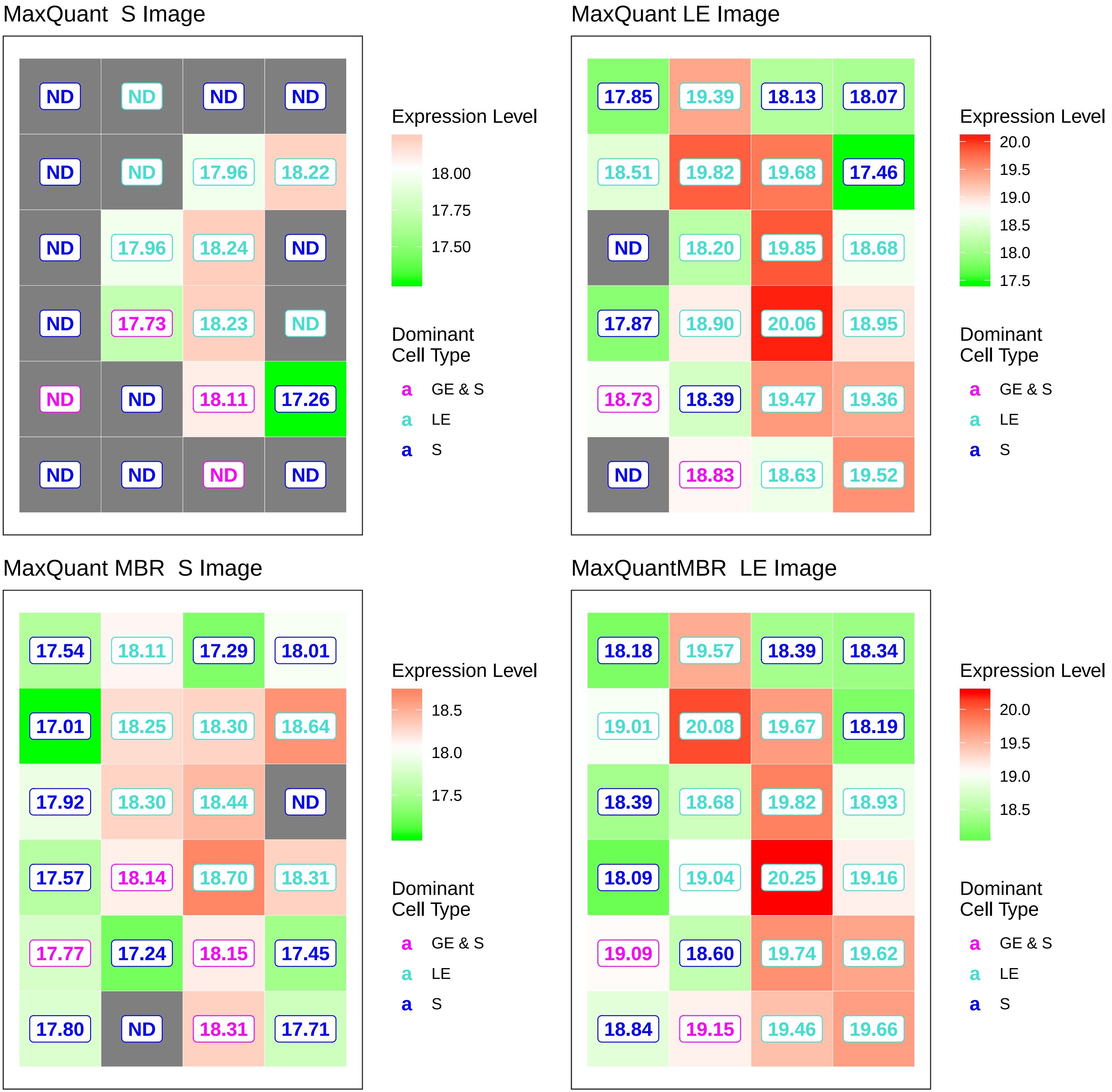
MaxQuant S Image
ND ND ND ND
ND ND 17.96 18.22
ND 17.96 18.24 ND
ND 17.73 18.23 ND
ND ND 18.11 17.26
ND ND ND ND
Expression Level
18.00
17.75
17.50
Dominant Cell Type
a GE & S
a LE
a S
MaxQuant LE Image
17.85 19.39 18.13 18.07
18.51 19.82 19.68 17.46
ND 18.20 19.85 18.68
17.87 18.90 20.06 18.95
18.73 18.39 19.47 19.36
ND 18.83 18.63 19.52
Expression Level
20.0
19.5
19.0
18.5
18.0
17.5
Dominant Cell Type
a GE & S
a LE
a S
MaxQuant MBR S Image
17.54 18.11 17.29 18.01
17.01 18.25 18.30 18.64
17.92 18.30 18.44 ND
17.57 18.14 18.70 18.31
17.77 17.24 18.15 17.45
17.80 ND 18.31 17.71
Expression Level
18.5
18.0
17.5
Dominant Cell Type
a GE & S
a LE
a S
MaxQuantMBR LE Image
18.18 19.57 18.39 18.34
19.01 20.08 19.67 18.19
18.39 18.68 19.82 18.93
18.09 19.04 20.25 19.16
19.09 18.60 19.74 19.62
18.84 19.15 19.46 19.66
Expression Level
20.0
19.5
19.0
18.5
Dominant Cell Type
a GE & S
a LE
a S

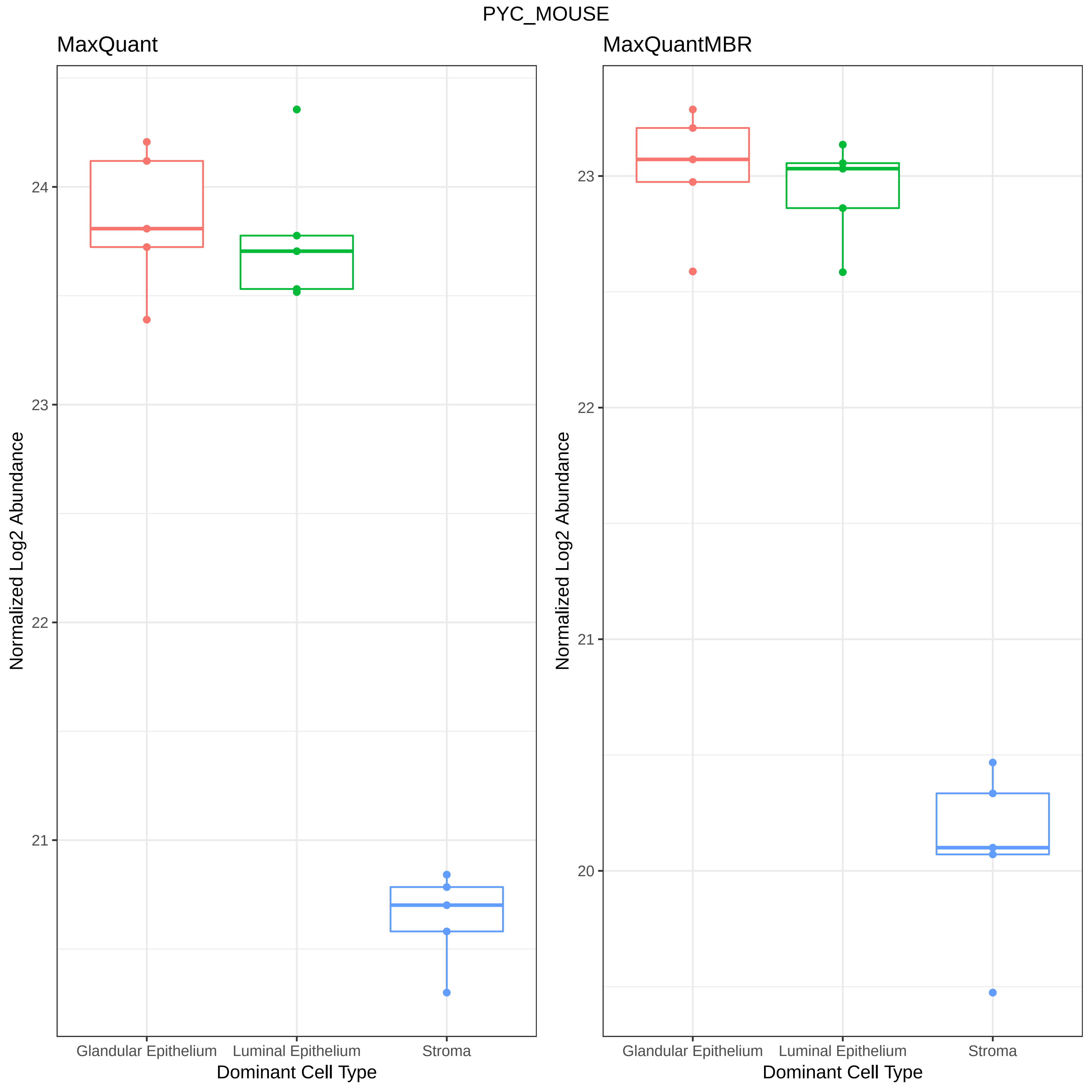
PYC_MOUSE
MaxQuant
MaxQuantMBR
Normalized Log2 Abundance
24
23
22
21
20
Glandular Epithelium
Luminal Epithelium
Stroma
Dominant Cell Type

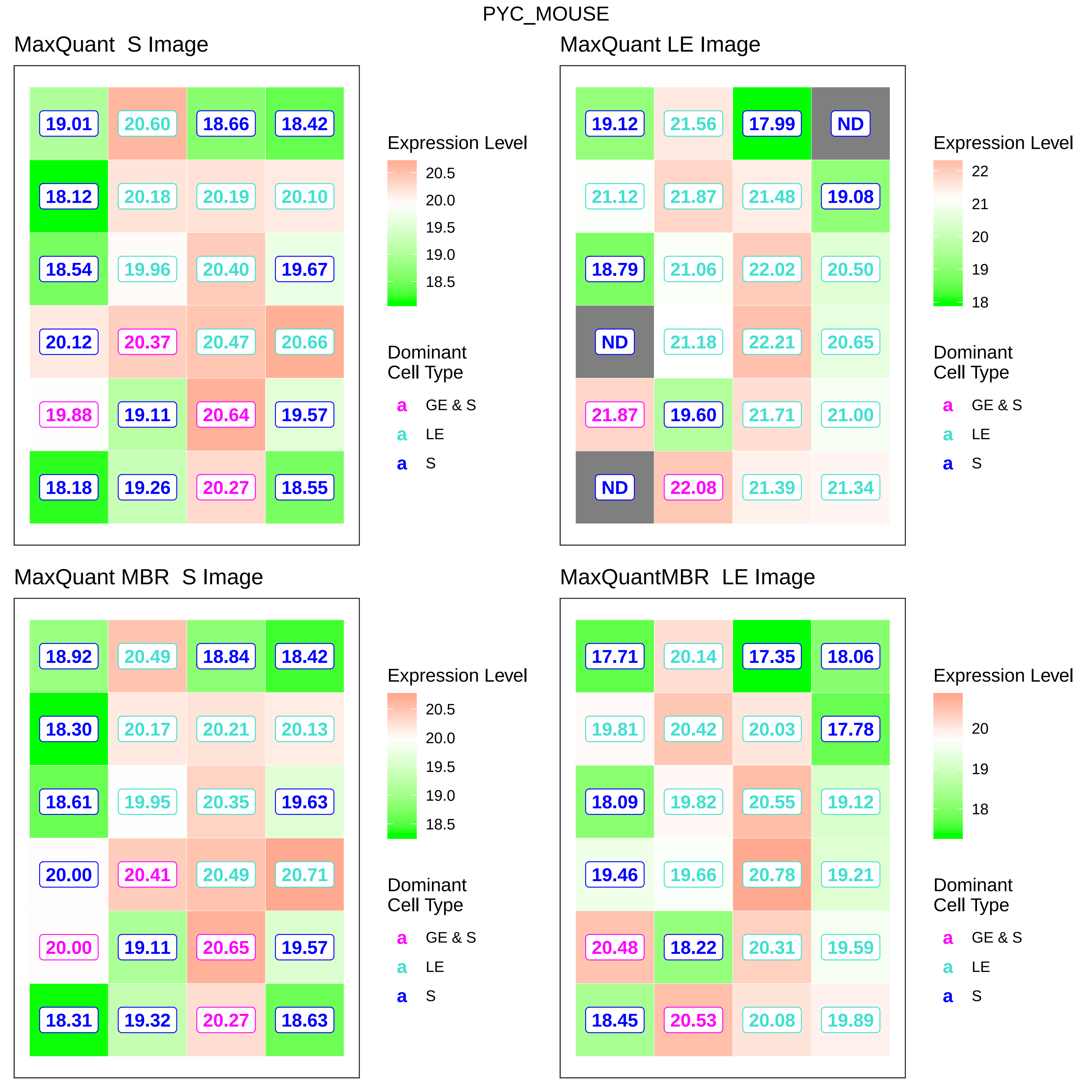
PYC_MOUSE
MaxQuant S Image
19.01 20.60 18.66 18.42
18.12 20.18 20.19 20.10
18.54 19.96 20.40 19.67
20.12 20.37 20.47 20.66
19.88 19.11 20.64 19.57
18.18 19.26 20.27 18.55
Expression Level
20.5
20.0
19.5
19.0
18.5
Dominant Cell Type
a GE & S
a LE
a S
MaxQuant LE Image
19.12 21.56 17.99 ND
21.12 21.87 21.48 19.08
18.79 21.06 22.02 20.50
ND 21.18 22.21 20.65
21.87 19.60 21.71 21.00
ND 22.08 21.39 21.34
Expression Level
22
21
20
19
18
Dominant Cell Type
a GE & S
a LE
a S
MaxQuant MBR S Image
18.92 20.49 18.84 18.42
18.30 20.17 20.21 20.13
18.61 19.95 20.35 19.63
20.00 20.41 20.49 20.71
20.00 19.11 20.65 19.57
18.31 19.32 20.27 18.63
Expression Level
20.5
20.0
19.5
19.0
18.5
Dominant Cell Type
a GE & S
a LE
a S
MaxQuantMBR LE Image
17.71 20.14 17.35 18.06
19.81 20.42 20.03 17.78
18.09 19.82 20.55 19.12
19.46 19.66 20.78 19.21
20.48 18.22 20.31 19.59
18.45 20.53 20.08 19.89
Expression Level
20
19
18
Dominant Cell Type
a GE & S
a LE
a S

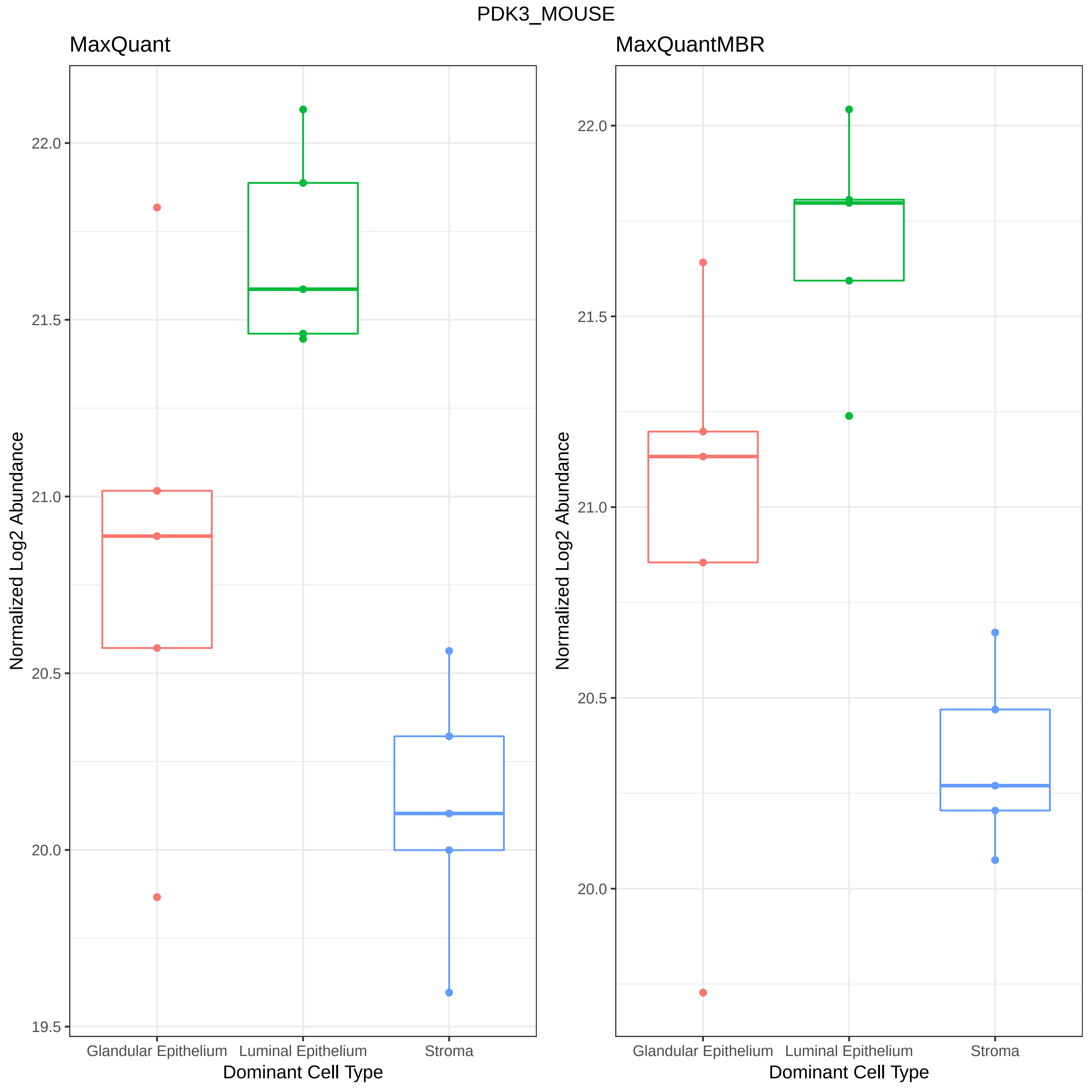

PDK3_MOUSE
MaxQuant
MaxQuantMBR
Normalized Log2 Abundance
22.0
21.5
21.0
20.5
20.0
19.5
Glandular Epithelium
Luminal Epithelium
Stroma
Dominant Cell Type

PDK3_MOUSE

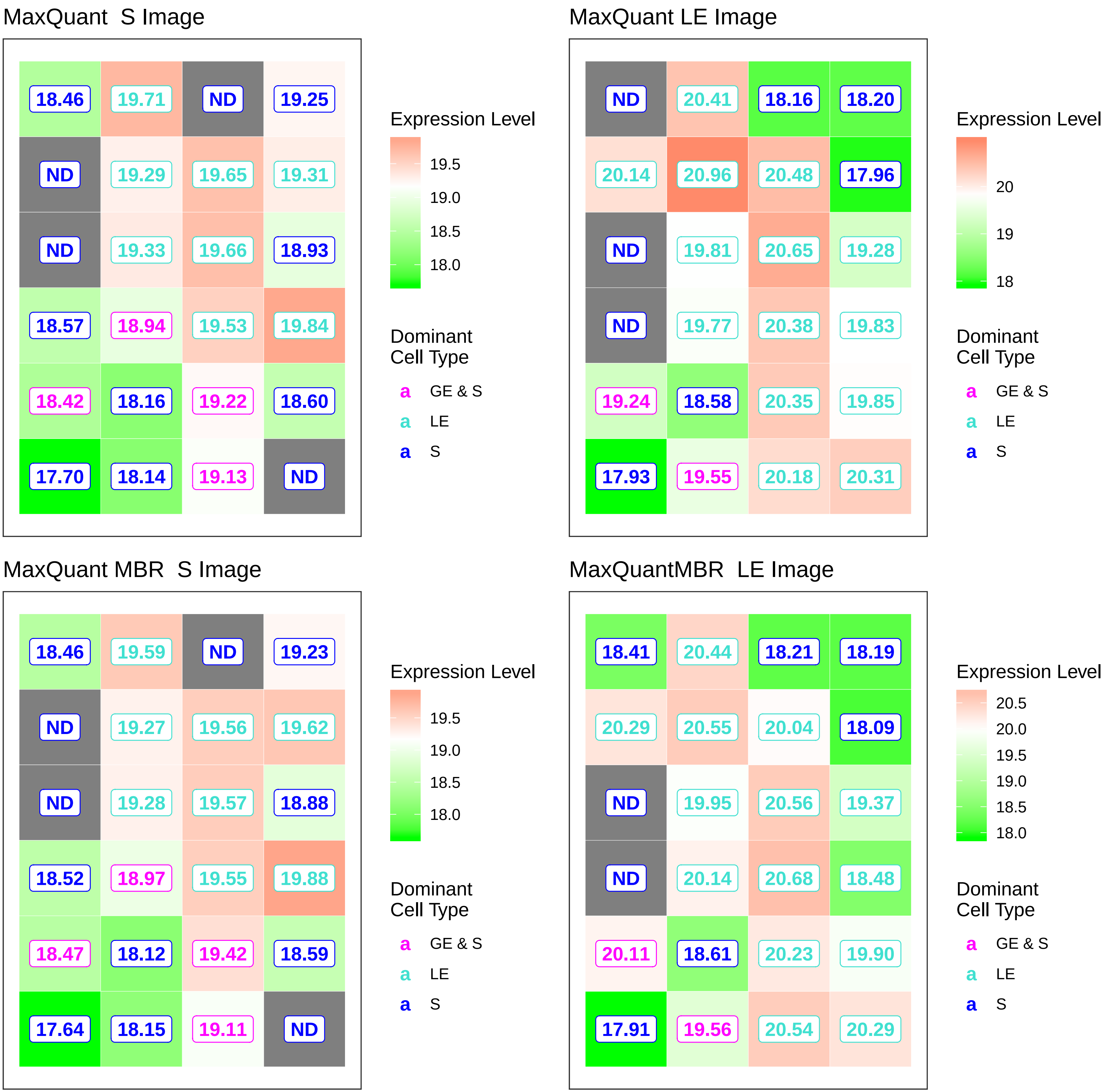

MaxQuant S Image
18.46
19.71
ND
19.25
ND
19.29
19.65
19.31
ND
19.33
19.66
18.93
18.57
18.94
19.53
19.84
18.42
18.16
19.22
18.60
17.70
18.14
19.13
ND
Expression Level
19.5
19.0
18.5
18.0
Dominant Cell Type
a GE & S
a LE
a S
MaxQuant LE Image
ND
20.41
18.16
18.20
20.14
20.96
20.48
17.96
ND
19.81
20.65
19.28
ND
19.77
20.38
19.83
19.24
18.58
20.35
19.85
17.93
19.55
20.18
20.31
Expression Level
20
19
18
Dominant Cell Type
a GE & S
a LE
a S
MaxQuant MBR S Image
18.46
19.59
ND
19.23
ND
19.27
19.56
19.62
ND
19.28
19.57
18.88
18.52
18.97
19.55
19.88
18.47
18.12
19.42
18.59
17.64
18.15
19.11
ND
Expression Level
19.5
19.0
18.5
18.0
Dominant Cell Type
a GE & S
a LE
a S
MaxQuantMBR LE Image
18.41
20.44
18.21
18.19
20.29
20.55
20.04
18.09
ND
19.95
20.56
19.37
ND
20.14
20.68
18.48
20.11
18.61
20.23
19.90
17.91
19.56
20.54
20.29
Expression Level
20.5
20.0
19.5
19.0
18.5
18.0
Dominant Cell Type
a GE & S
a LE
a S

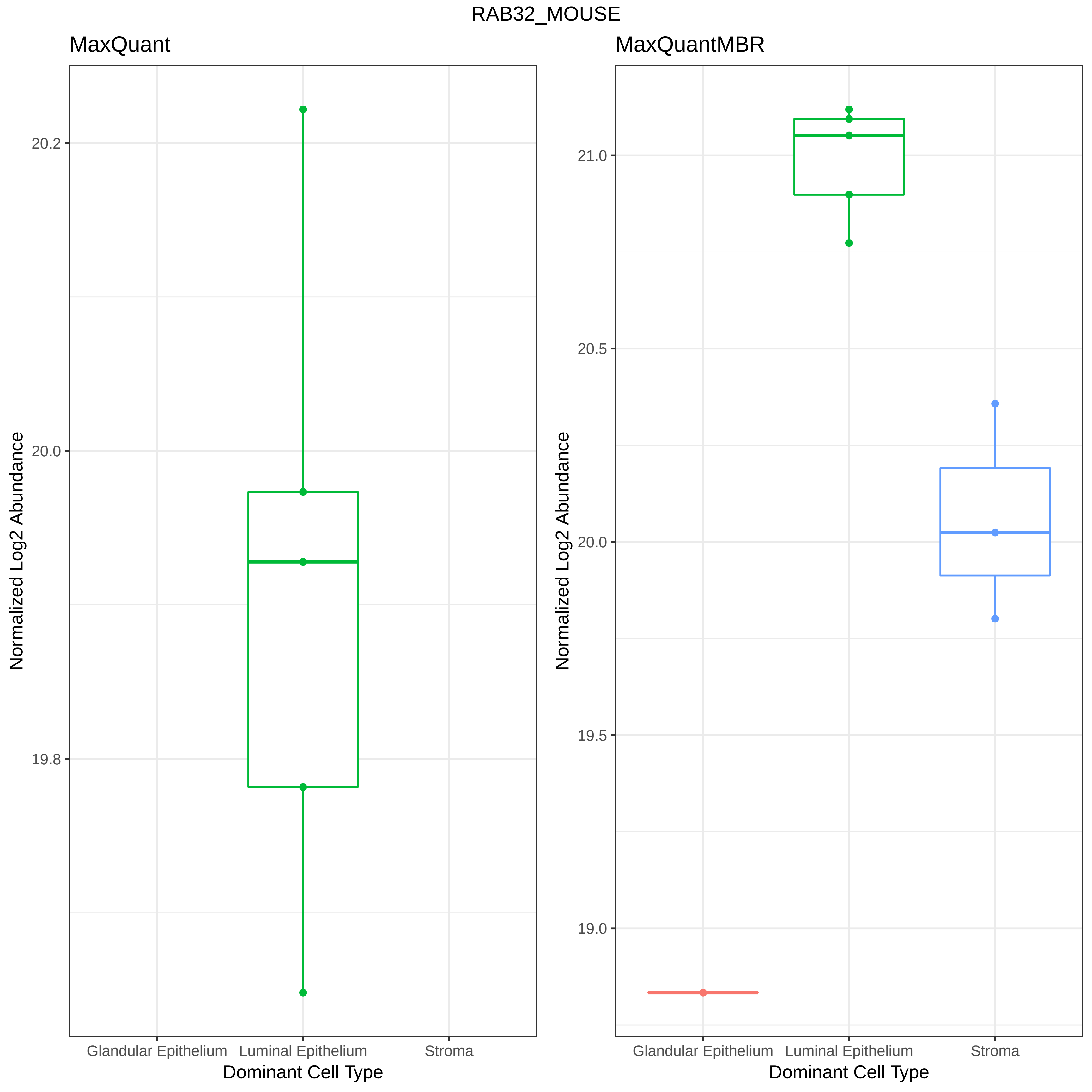
RAB32_MOUSE
MaxQuant
MaxQuantMBR
Normalized Log2 Abundance
20.2
20.0
19.8
21.0
20.5
20.0
19.5
19.0
Glandular Epithelium
Luminal Epithelium
Stroma
Dominant Cell Type

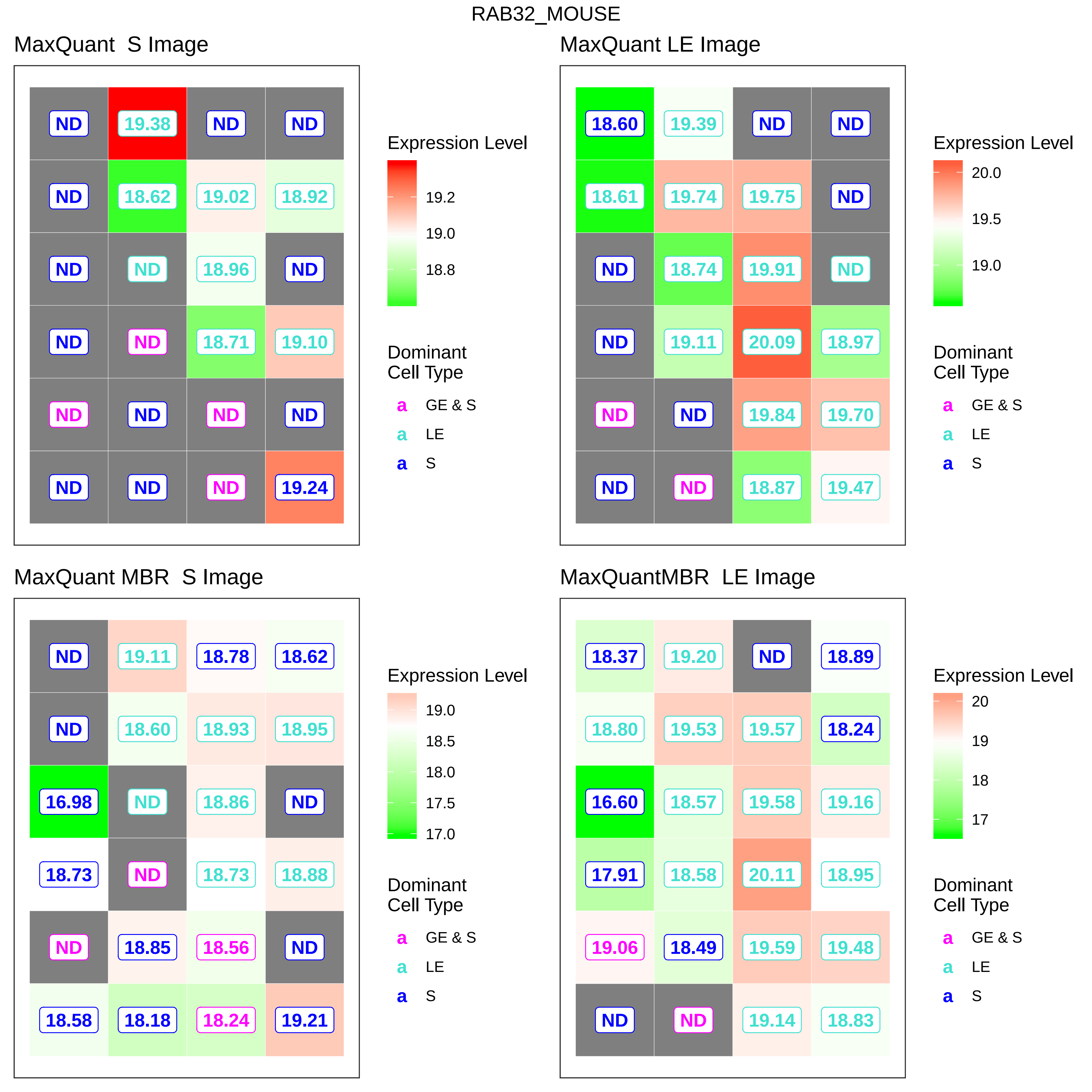

RAB32_MOUSE
MaxQuant S Image
ND
19.38
ND
ND
ND
18.62
19.02
18.92
ND
ND
18.96
ND
ND
ND
18.71
19.10
ND
ND
ND
ND
ND
ND
ND
19.24
Expression Level
19.2
19.0
18.8
Dominant Cell Type
a GE & S
a LE
a S
MaxQuant LE Image
18.60
19.39
ND
ND
18.61
19.74
19.75
ND
ND
18.74
19.91
ND
ND
19.11
20.09
18.97
ND
ND
19.84
19.70
ND
ND
18.87
19.47
Expression Level
20.0
19.5
19.0
Dominant Cell Type
a GE & S
a LE
a S
MaxQuant MBR S Image
ND
19.11
18.78
18.62
ND
18.60
18.93
18.95
16.98
ND
18.86
ND
18.73
ND
18.73
18.88
ND
18.85
18.56
ND
18.58
18.18
18.24
19.21
Expression Level
19.0
18.5
18.0
17.5
17.0
Dominant Cell Type
a GE & S
a LE
a S
MaxQuantMBR LE Image
18.37
19.20
ND
18.89
18.80
19.53
19.57
18.24
16.60
18.57
19.58
19.16
17.91
18.58
20.11
18.95
19.06
18.49
19.59
19.48
ND
ND
19.14
18.83
Expression Level
20
19
18
17
Dominant Cell Type
a GE & S
a LE
a S

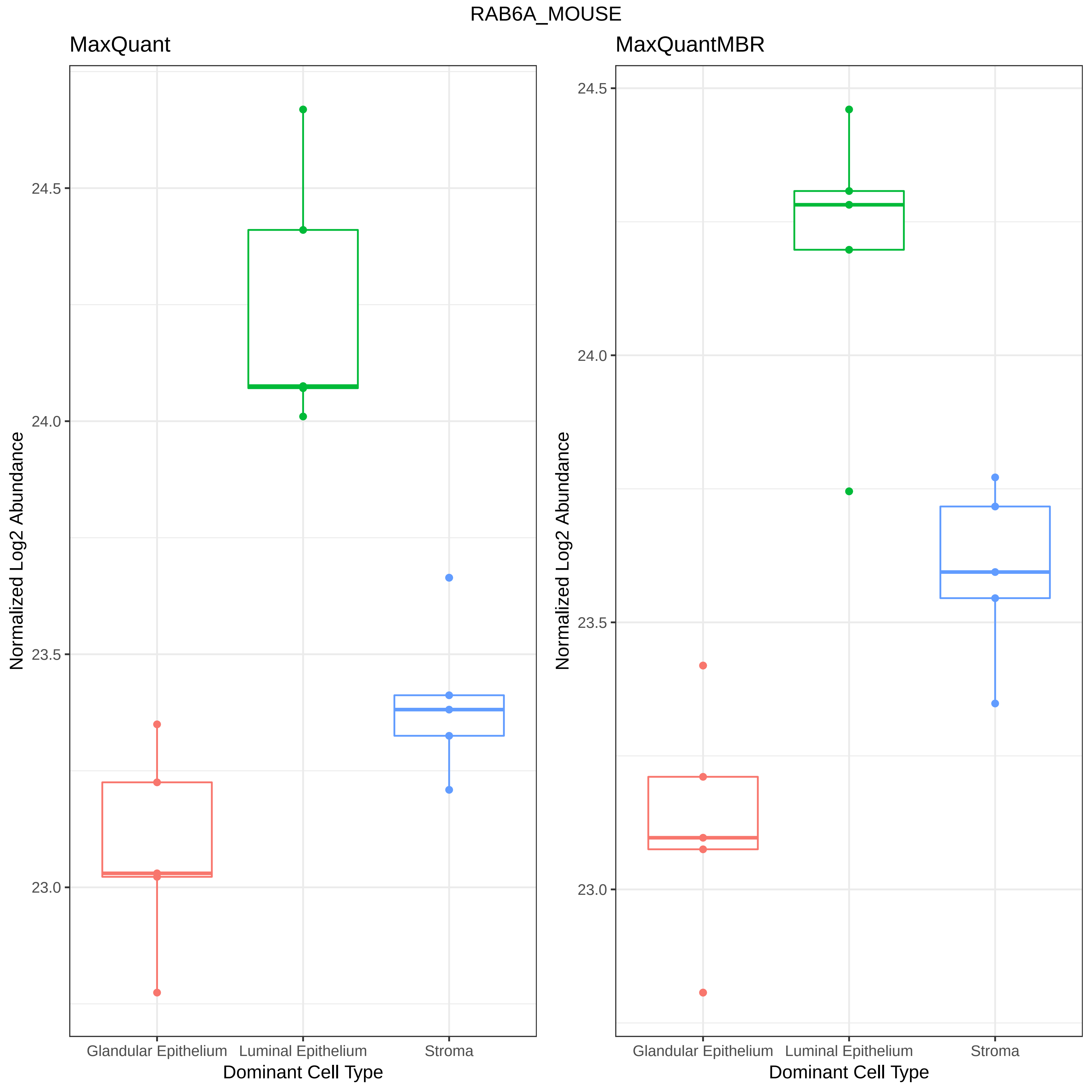
RAB6A_MOUSE
MaxQuant
MaxQuantMBR
Normalized Log2 Abundance
24.5
24.0
23.5
23.0
Glandular Epithelium
Luminal Epithelium
Stroma
Dominant Cell Type

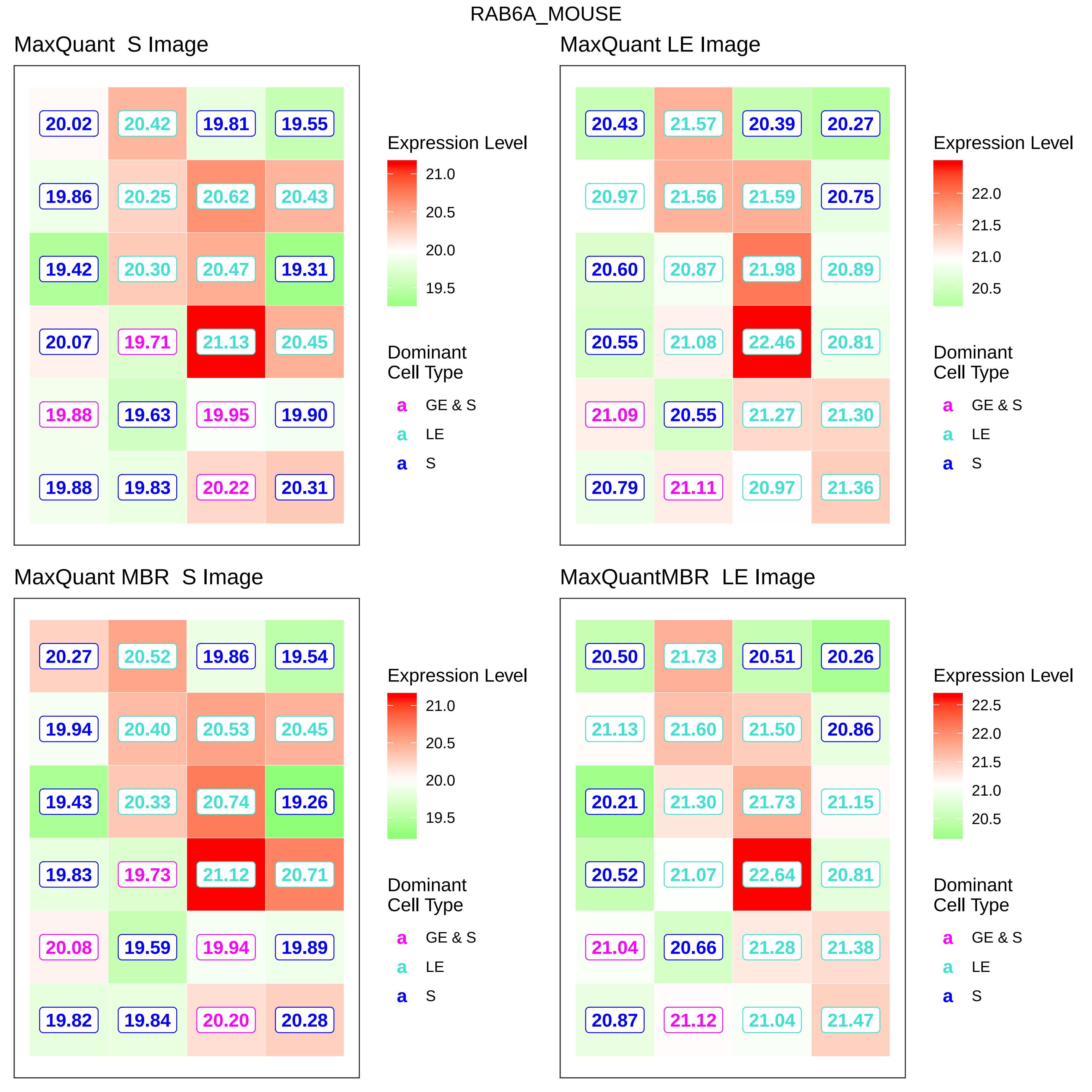
RAB6A_MOUSE
MaxQuant S Image
20.02 20.42 19.81 19.55
19.86 20.25 20.62 20.43
19.42 20.30 20.47 19.31
20.07 19.71 21.13 20.45
19.88 19.63 19.95 19.90
19.88 19.83 20.22 20.31
Expression Level
21.0
20.5
20.0
19.5
Dominant Cell Type
a GE & S
a LE
a S
MaxQuant LE Image
20.43 21.57 20.39 20.27
20.97 21.56 21.59 20.75
20.60 20.87 21.98 20.89
20.55 21.08 22.46 20.81
21.09 20.55 21.27 21.30
20.79 21.11 20.97 21.36
Expression Level
22.0
21.5
21.0
20.5
Dominant Cell Type
a GE & S
a LE
a S
MaxQuant MBR S Image
20.27 20.52 19.86 19.54
19.94 20.40 20.53 20.45
19.43 20.33 20.74 19.26
19.83 19.73 21.12 20.71
20.08 19.59 19.94 19.89
19.82 19.84 20.20 20.28
Expression Level
21.0
20.5
20.0
19.5
Dominant Cell Type
a GE & S
a LE
a S
MaxQuantMBR LE Image
20.50 21.73 20.51 20.26
21.13 21.60 21.50 20.86
20.21 21.30 21.73 21.15
20.52 21.07 22.64 20.81
21.04 20.66 21.28 21.38
20.87 21.12 21.04 21.47
Expression Level
22.5
22.0
21.5
21.0
20.5
Dominant Cell Type
a GE & S
a LE
a S

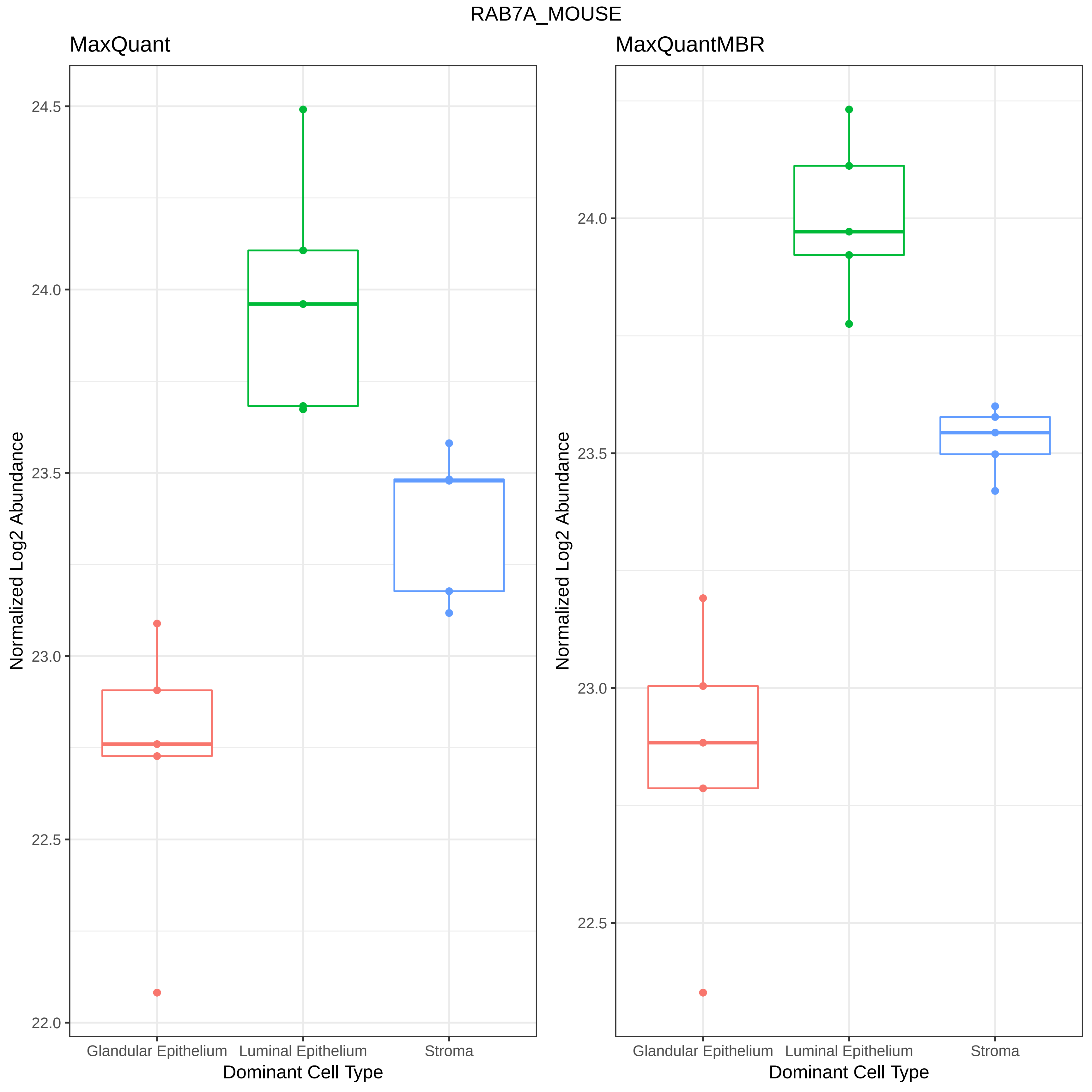
RAB7A_MOUSE
MaxQuant
MaxQuantMBR
Normalized Log2 Abundance
24.5
24.0
23.5
23.0
22.5
22.0
Glandular Epithelium
Luminal Epithelium
Stroma
Dominant Cell Type

RAB7A_MOUSE

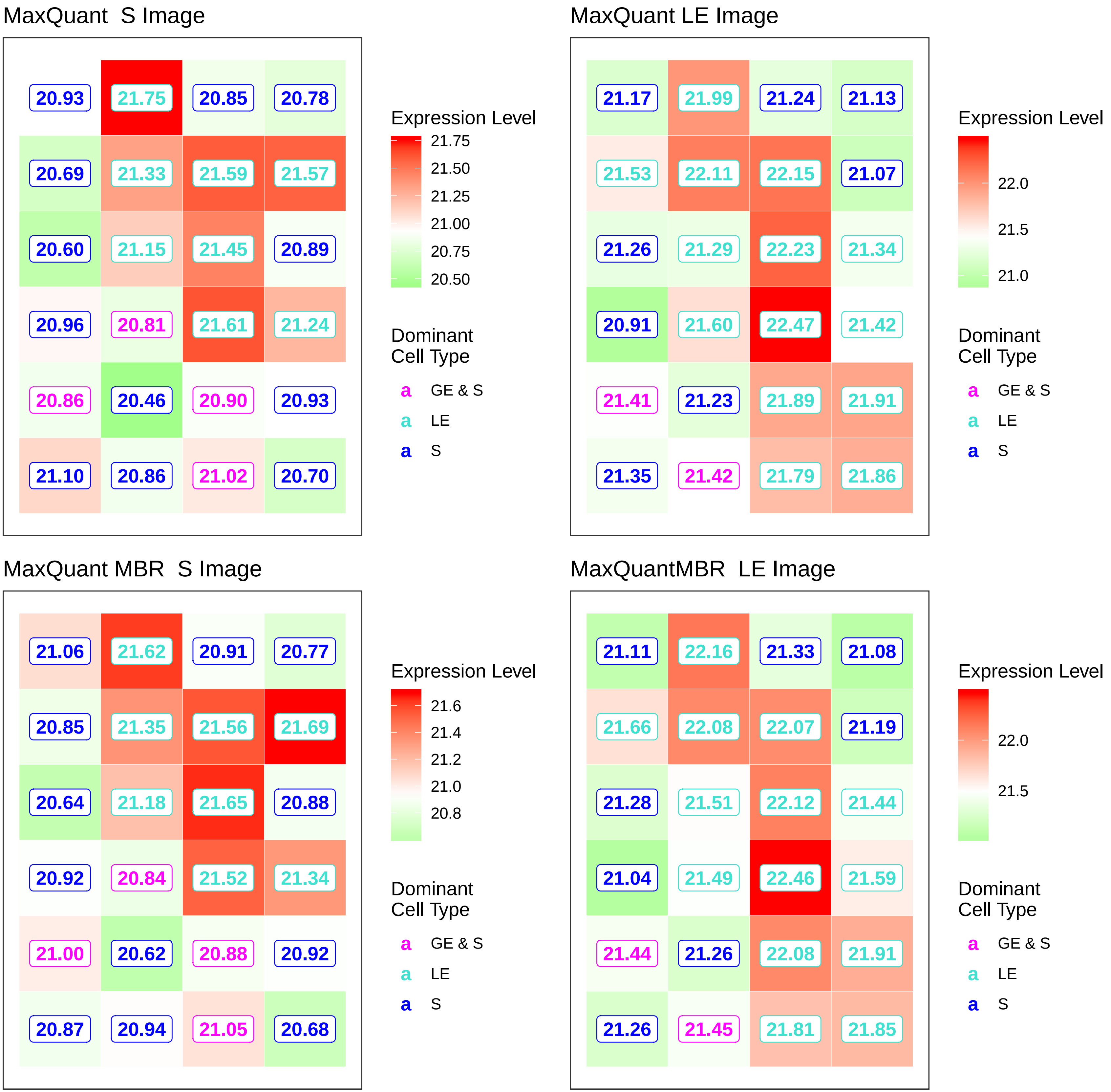

MaxQuant S Image
20.93 21.75 20.85 20.78
20.69 21.33 21.59 21.57
20.60 21.15 21.45 20.89
20.96 20.81 21.61 21.24
20.86 20.46 20.90 20.93
21.10 20.86 21.02 20.70
Expression Level
21.75
21.50
21.25
21.00
20.75
20.50
Dominant Cell Type
a GE & S
a LE
a S
MaxQuant LE Image
21.17 21.99 21.24 21.13
21.53 22.11 22.15 21.07
21.26 21.29 22.23 21.34
20.91 21.60 22.47 21.42
21.41 21.23 21.89 21.91
21.35 21.42 21.79 21.86
Expression Level
22.0
21.5
21.0
Dominant Cell Type
a GE & S
a LE
a S
MaxQuant MBR S Image
21.06 21.62 20.91 20.77
20.85 21.35 21.56 21.69
20.64 21.18 21.65 20.88
20.92 20.84 21.52 21.34
21.00 20.62 20.88 20.92
20.87 20.94 21.05 20.68
Expression Level
21.6
21.4
21.2
21.0
20.8
Dominant Cell Type
a GE & S
a LE
a S
MaxQuantMBR LE Image
21.11 22.16 21.33 21.08
21.66 22.08 22.07 21.19
21.28 21.51 22.12 21.44
21.04 21.49 22.46 21.59
21.44 21.26 22.08 21.91
21.26 21.45 21.81 21.85
Expression Level
22.0
21.5
Dominant Cell Type
a GE & S
a LE
a S

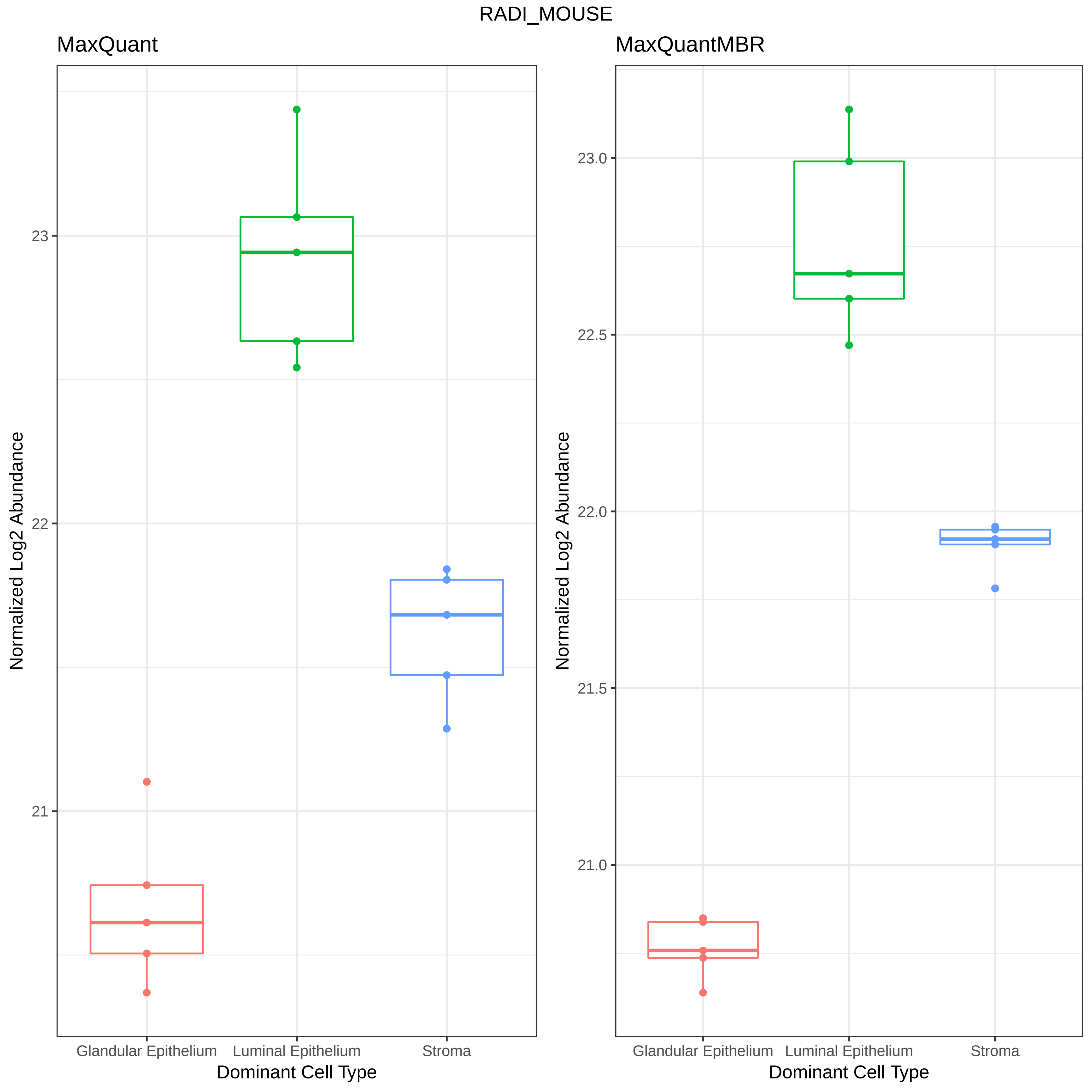

RADI_MOUSE
MaxQuant
MaxQuantMBR
Normalized Log2 Abundance
23
22
21
23.0
22.5
22.0
21.5
21.0
Glandular Epithelium
Luminal Epithelium
Stroma
Dominant Cell Type

RADI_MOUSE

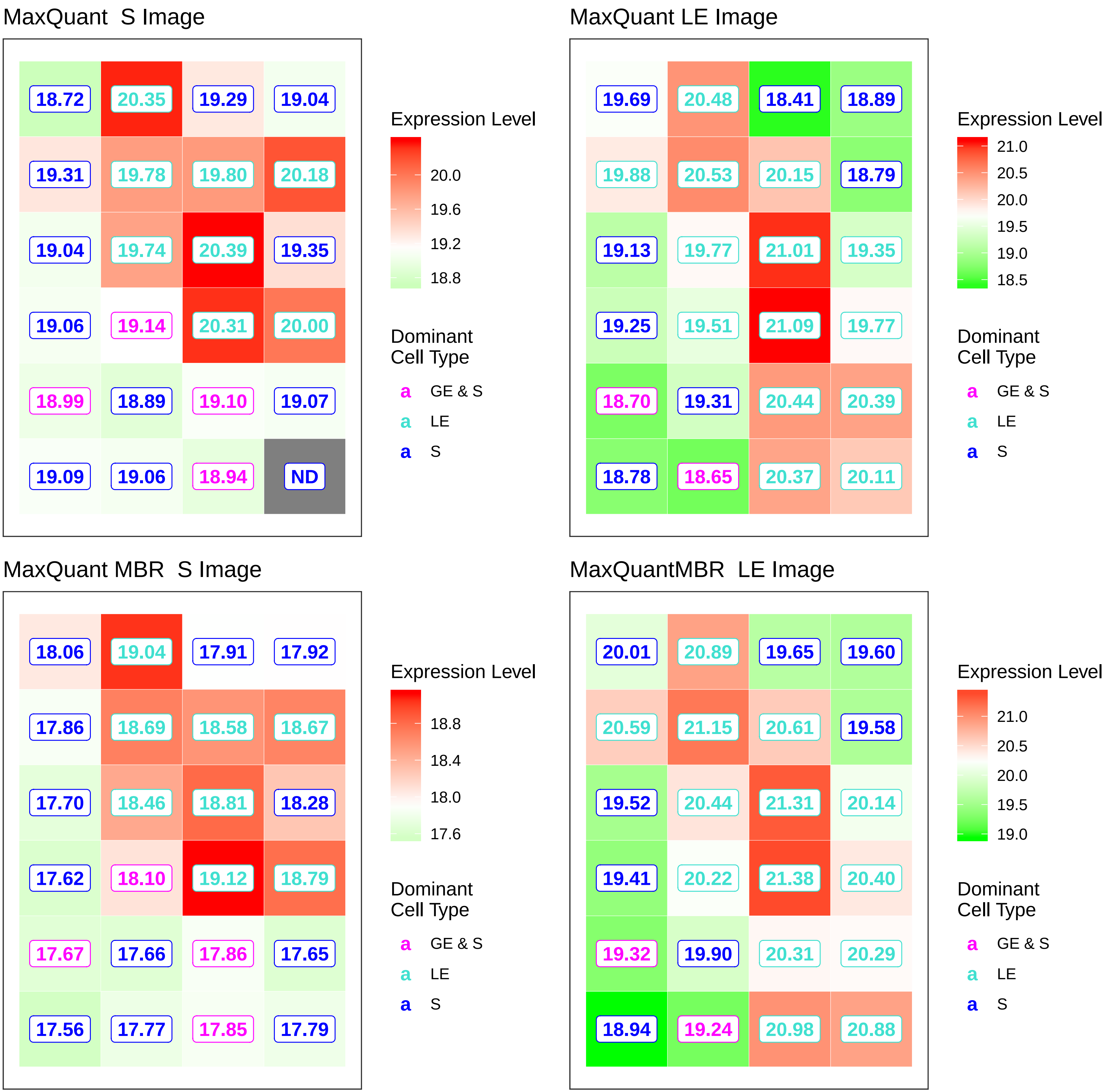
MaxQuant S Image
18.72
20.35
19.29
19.04
19.31
19.78
19.80
20.18
19.04
19.74
20.39
19.35
19.06
19.14
20.31
20.00
18.99
18.89
19.10
19.07
19.09
19.06
18.94
ND
Expression Level
20.0
19.6
19.2
18.8
Dominant Cell Type
a GE & S
a LE
a S
MaxQuant LE Image
19.69
20.48
18.41
18.89
19.88
20.53
20.15
18.79
19.13
19.77
21.01
19.35
19.25
19.51
21.09
19.77
18.70
19.31
20.44
20.39
18.78
18.65
20.37
20.11
Expression Level
21.0
20.5
20.0
19.5
19.0
18.5
Dominant Cell Type
a GE & S
a LE
a S
MaxQuant MBR S Image
18.06
19.04
17.91
17.92
17.86
18.69
18.58
18.67
17.70
18.46
18.81
18.28
17.62
18.10
19.12
18.79
17.67
17.66
17.86
17.65
17.56
17.77
17.85
17.79
Expression Level
18.8
18.4
18.0
17.6
Dominant Cell Type
a GE & S
a LE
a S
MaxQuantMBR LE Image
20.01
20.89
19.65
19.60
20.59
21.15
20.61
19.58
19.52
20.44
21.31
20.14
19.41
20.22
21.38
20.40
19.32
19.90
20.31
20.29
18.94
19.24
20.98
20.88
Expression Level
21.0
20.5
20.0
19.5
19.0
Dominant Cell Type
a GE & S
a LE
a S

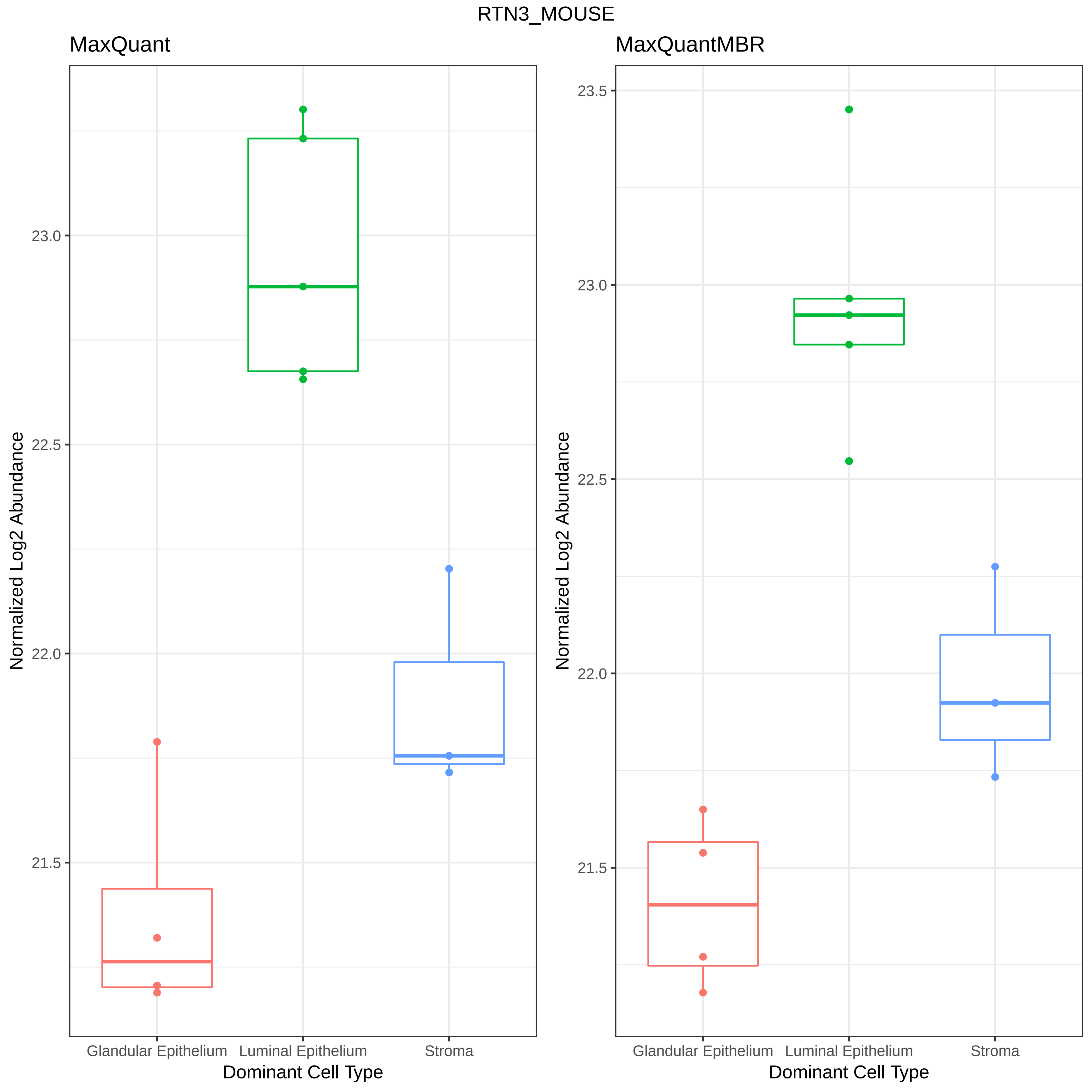

RTN3_MOUSE
MaxQuant
MaxQuantMBR
Normalized Log2 Abundance
23.5
23.0
22.5
22.0
21.5
Glandular Epithelium
Luminal Epithelium
Stroma
Dominant Cell Type

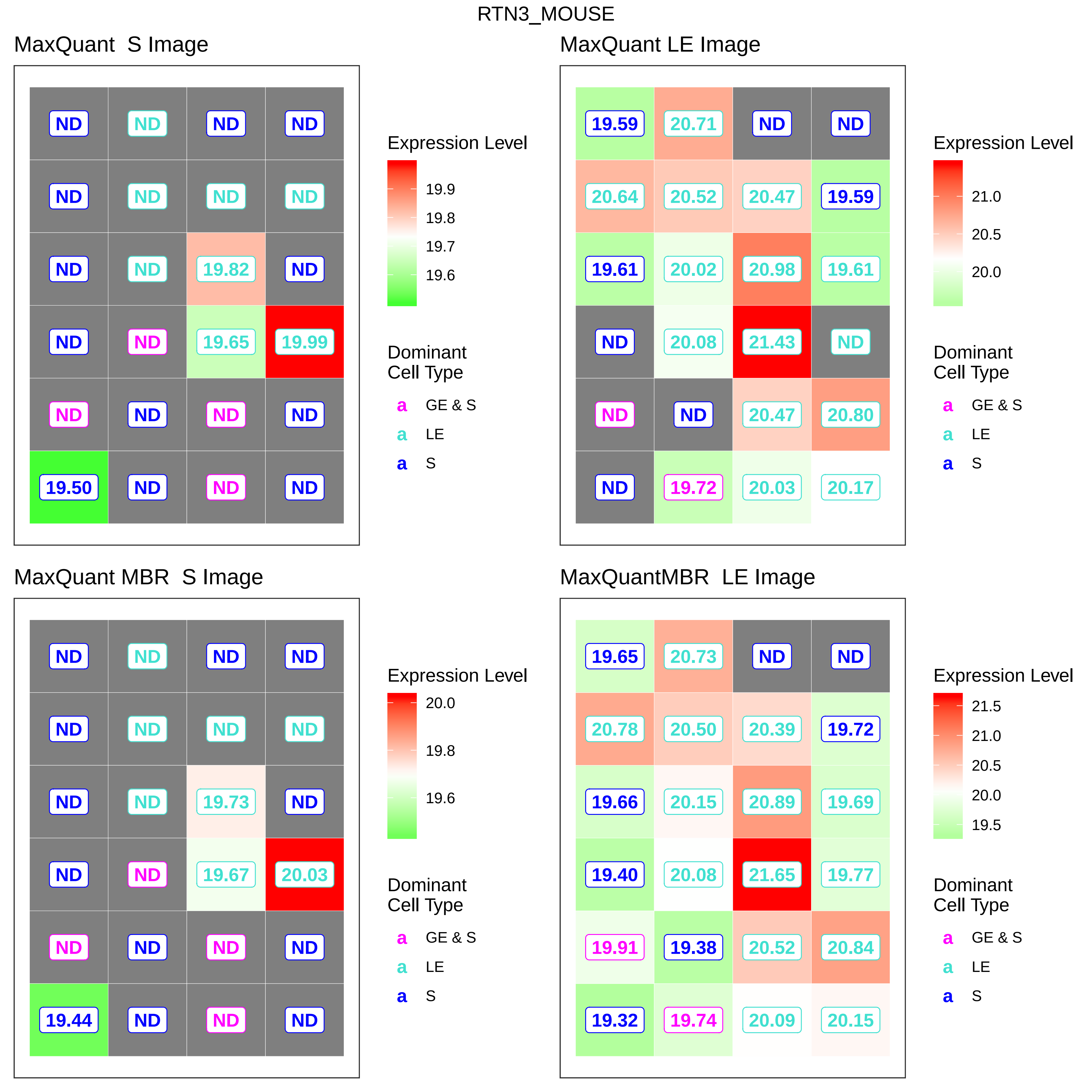
RTN3_MOUSE
MaxQuant S Image
ND ND ND ND
ND ND ND ND
ND ND 19.82 ND
ND ND 19.65 19.99
ND ND ND ND
19.50 ND ND ND
Expression Level
19.9
19.8
19.7
19.6
Dominant Cell Type
a GE & S
a LE
a S
MaxQuant LE Image
19.59 20.71 ND ND
20.64 20.52 20.47 19.59
19.61 20.02 20.98 19.61
ND 20.08 21.43 ND
ND ND 20.47 20.80
ND 19.72 20.03 20.17
Expression Level
21.0
20.5
20.0
Dominant Cell Type
a GE & S
a LE
a S
MaxQuant MBR S Image
ND ND ND ND
ND ND ND ND
ND ND 19.73 ND
ND ND 19.67 20.03
ND ND ND ND
19.44 ND ND ND
Expression Level
20.0
19.8
19.6
Dominant Cell Type
a GE & S
a LE
a S
MaxQuantMBR LE Image
19.65 20.73 ND ND
20.78 20.50 20.39 19.72
19.66 20.15 20.89 19.69
19.40 20.08 21.65 19.77
19.91 19.38 20.52 20.84
19.32 19.74 20.09 20.15
Expression Level
21.5
21.0
20.5
20.0
19.5
Dominant Cell Type
a GE & S
a LE
a S

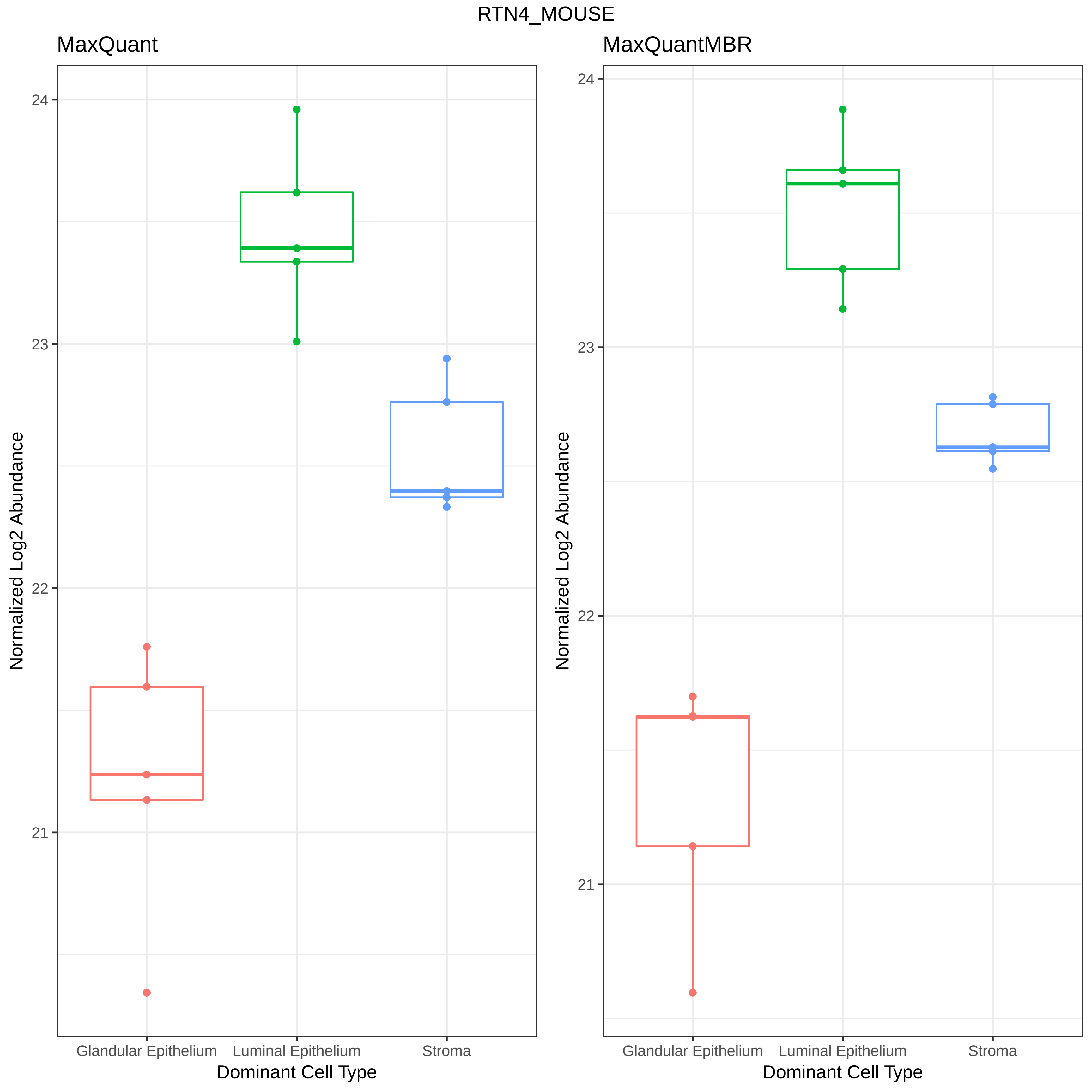

RTN4_MOUSE
MaxQuant
MaxQuantMBR
Normalized Log2 Abundance
24
23
22
21
Glandular Epithelium
Luminal Epithelium
Stroma
Dominant Cell Type

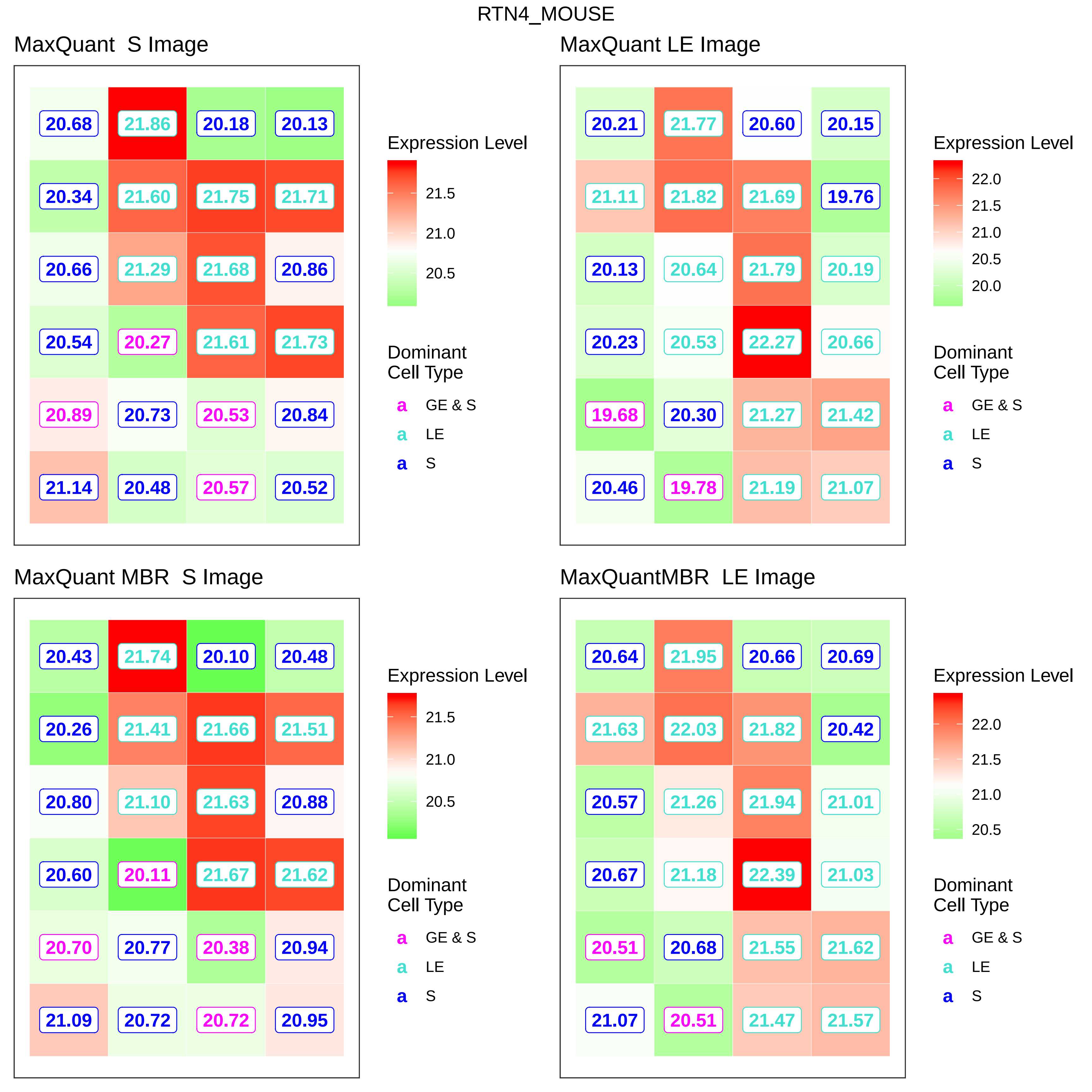
RTN4_MOUSE
MaxQuant S Image
20.68 21.86 20.18 20.13
20.34 21.60 21.75 21.71
20.66 21.29 21.68 20.86
20.54 20.27 21.61 21.73
20.89 20.73 20.53 20.84
21.14 20.48 20.57 20.52
Expression Level
21.5
21.0
20.5
Dominant Cell Type
a GE & S
a LE
a S
MaxQuant LE Image
20.21 21.77 20.60 20.15
21.11 21.82 21.69 19.76
20.13 20.64 21.79 20.19
20.23 20.53 22.27 20.66
19.68 20.30 21.27 21.42
20.46 19.78 21.19 21.07
Expression Level
22.0
21.5
21.0
20.5
20.0
Dominant Cell Type
a GE & S
a LE
a S
MaxQuant MBR S Image
20.43 21.74 20.10 20.48
20.26 21.41 21.66 21.51
20.80 21.10 21.63 20.88
20.60 20.11 21.67 21.62
20.70 20.77 20.38 20.94
21.09 20.72 20.72 20.95
Expression Level
21.5
21.0
20.5
Dominant Cell Type
a GE & S
a LE
a S
MaxQuantMBR LE Image
20.64 21.95 20.66 20.69
21.63 22.03 21.82 20.42
20.57 21.26 21.94 21.01
20.67 21.18 22.39 21.03
20.51 20.68 21.55 21.62
21.07 20.51 21.47 21.57
Expression Level
22.0
21.5
21.0
20.5
Dominant Cell Type
a GE & S
a LE
a S

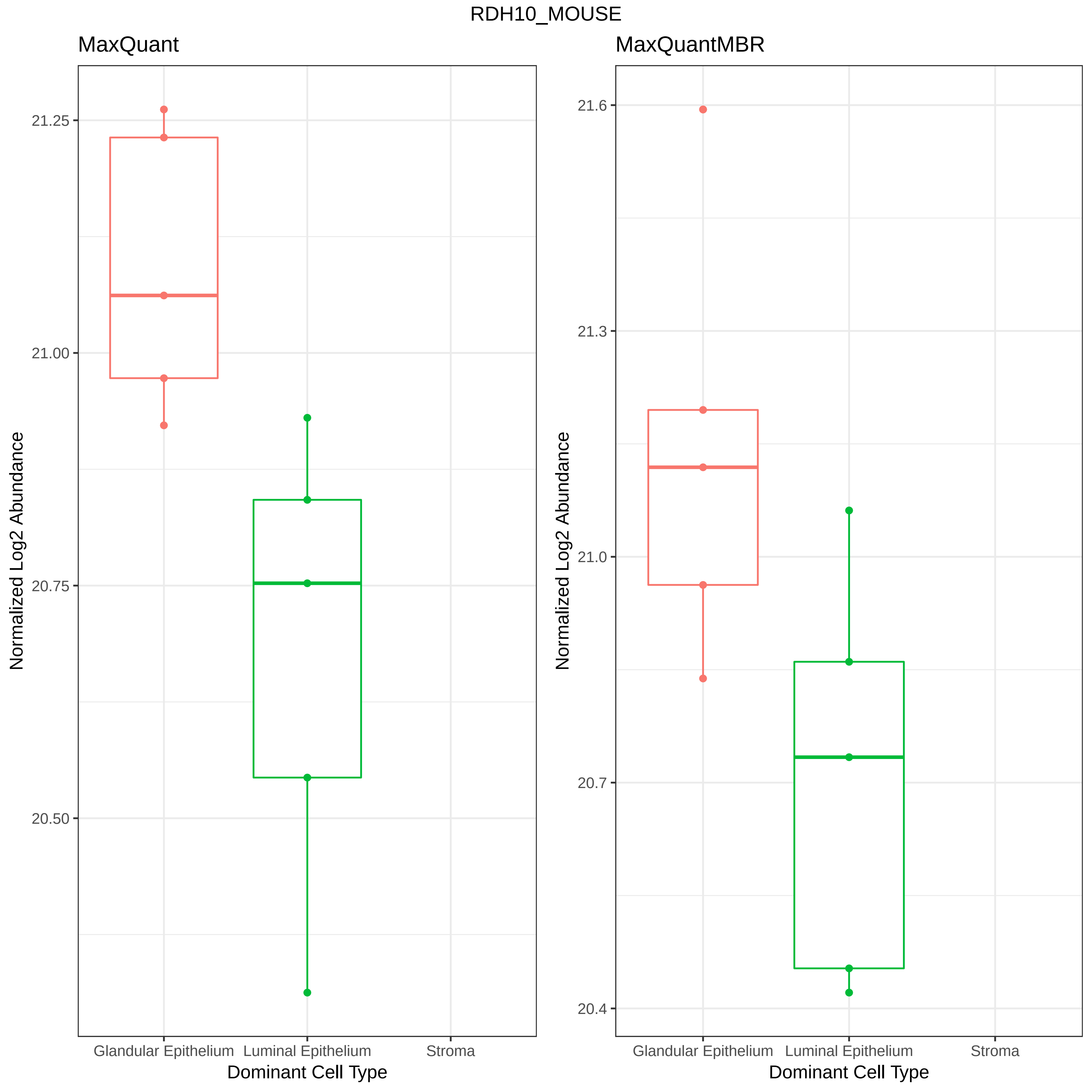
RDH10_MOUSE
MaxQuant
MaxQuantMBR
21.25
21.00
20.75
20.50
21.6
21.3
21.0
20.7
20.4
Normalized Log2 Abundance
Glandular Epithelium
Luminal Epithelium
Stroma
Dominant Cell Type

RDH10_MOUSE

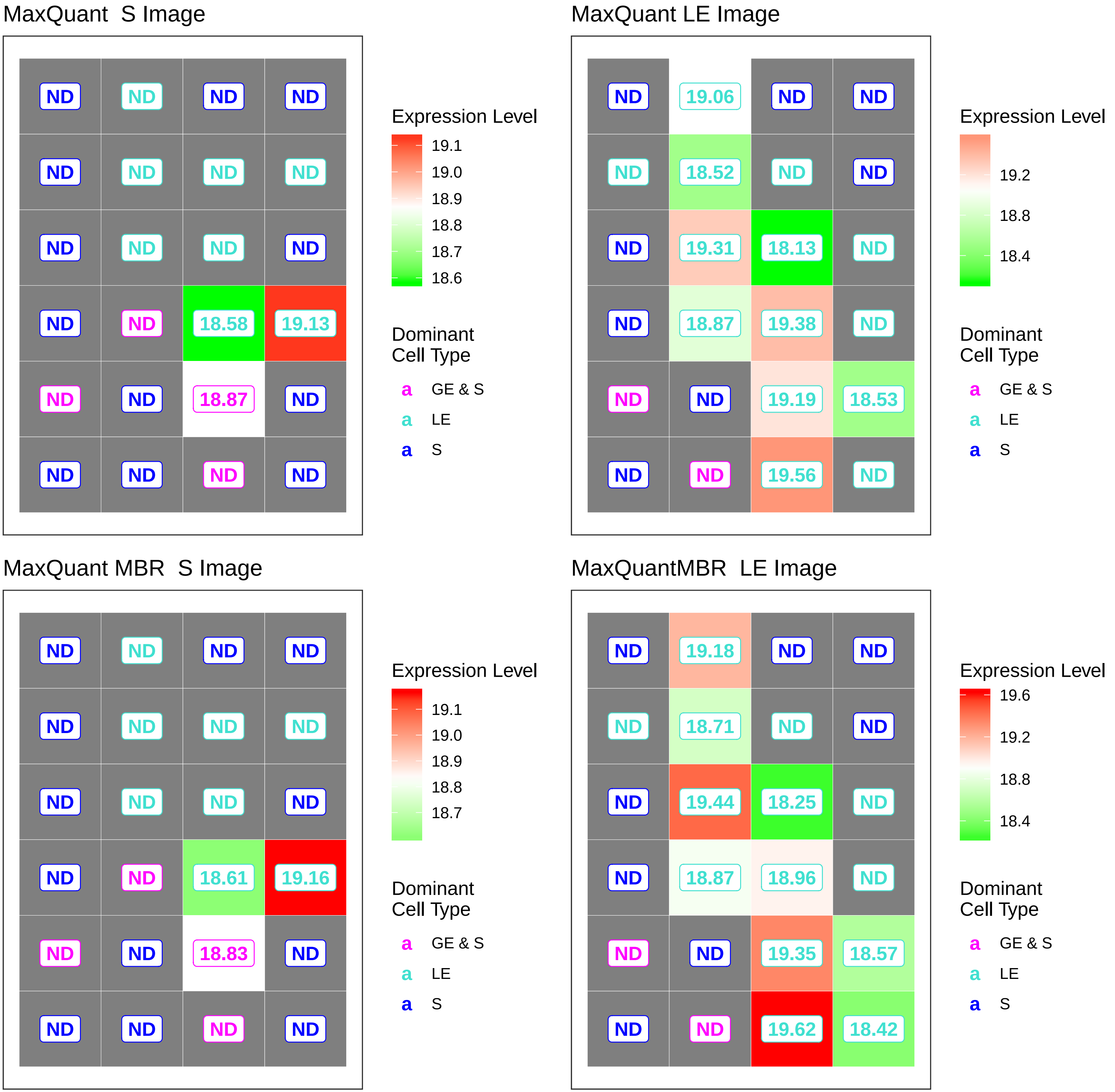
MaxQuant S Image
ND ND ND ND
ND ND ND ND
ND ND ND ND
ND ND 18.58 19.13
ND ND 18.87 ND
ND ND ND ND
Expression Level
19.1
19.0
18.9
18.8
18.7
18.6
Dominant Cell Type
a GE & S
a LE
a S
MaxQuant LE Image
ND 19.06 ND ND
ND 18.52 ND ND
ND 19.31 18.13 ND
ND 18.87 19.38 ND
ND ND 19.19 18.53
ND ND 19.56 ND
Expression Level
19.2
18.8
18.4
Dominant Cell Type
a GE & S
a LE
a S
MaxQuant MBR S Image
ND ND ND ND
ND ND ND ND
ND ND ND ND
ND ND 18.61 19.16
ND ND 18.83 ND
ND ND ND ND
Expression Level
19.1
19.0
18.9
18.8
18.7
Dominant Cell Type
a GE & S
a LE
a S
MaxQuantMBR LE Image
ND 19.18 ND ND
ND 18.71 ND ND
ND 19.44 18.25 ND
ND 18.87 18.96 ND
ND ND 19.35 18.57
ND ND 19.62 18.42
Expression Level
19.6
19.2
18.8
18.4
Dominant Cell Type
a GE & S
a LE
a S

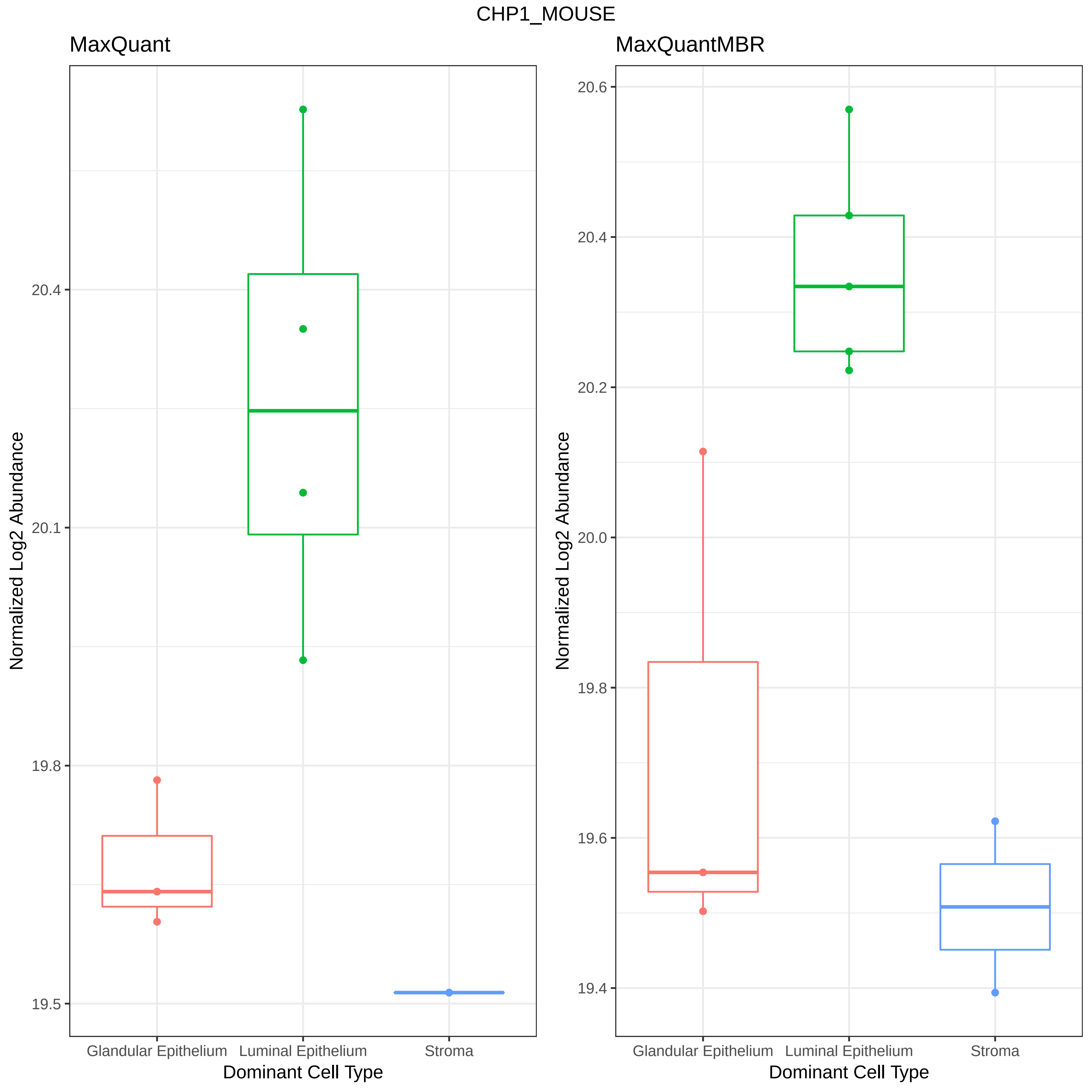

CHP1_MOUSE
MaxQuant
MaxQuantMBR
Normalized Log2 Abundance
20.4
20.1
19.8
19.5
20.6
20.2
20.0
19.6
19.4
Glandular Epithelium
Luminal Epithelium
Stroma
Dominant Cell Type

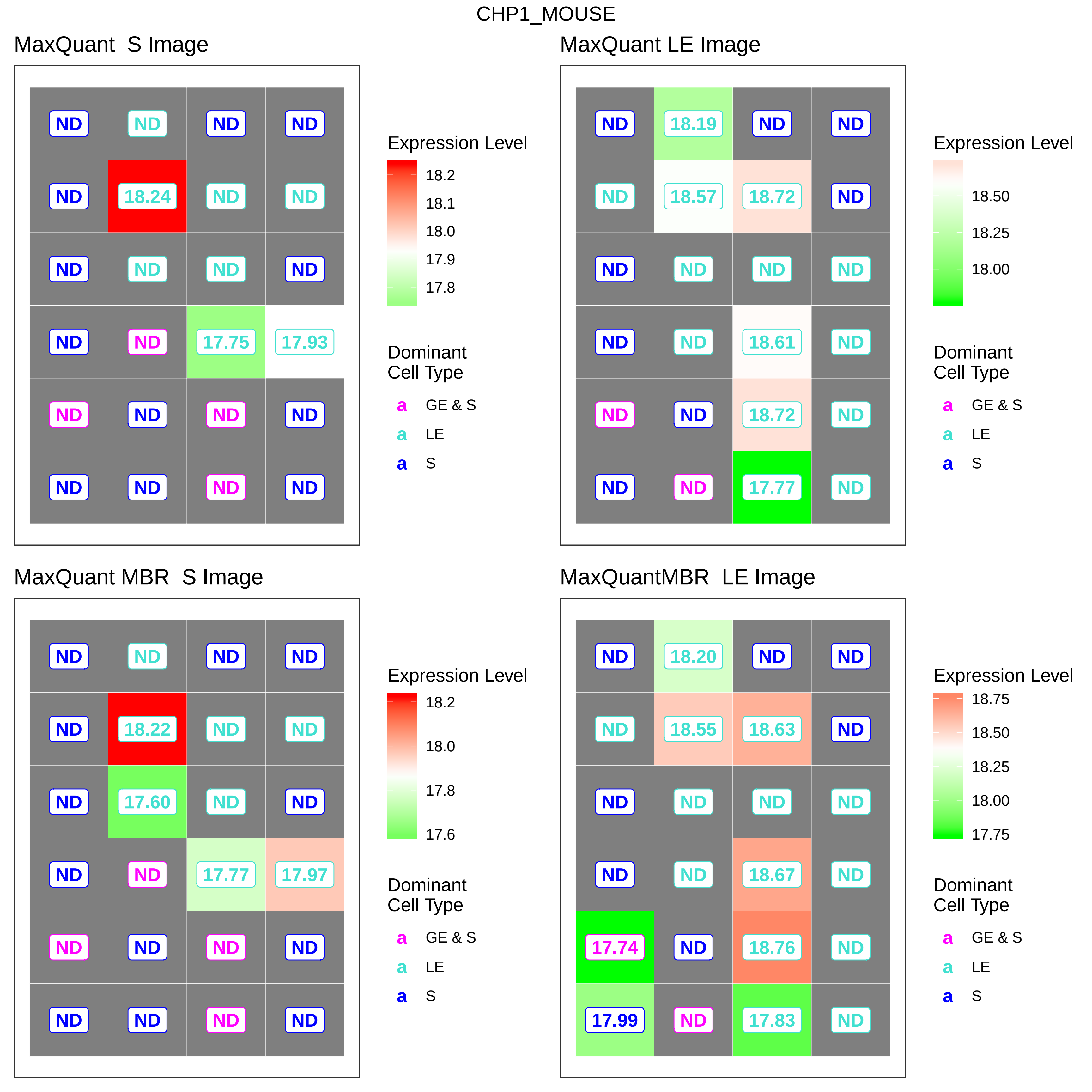
CHP1_MOUSE
MaxQuant S Image
MaxQuant LE Image
MaxQuant MBR S Image
MaxQuantMBR LE Image
Expression Level
Dominant Cell Type
GE & S
LE
S
18.24
17.75
17.93
18.19
18.57
18.72
18.61
18.72
17.77
18.22
17.60
17.77
17.97
18.20
18.55
18.63
18.67
17.74
18.76
17.99
17.83
ND

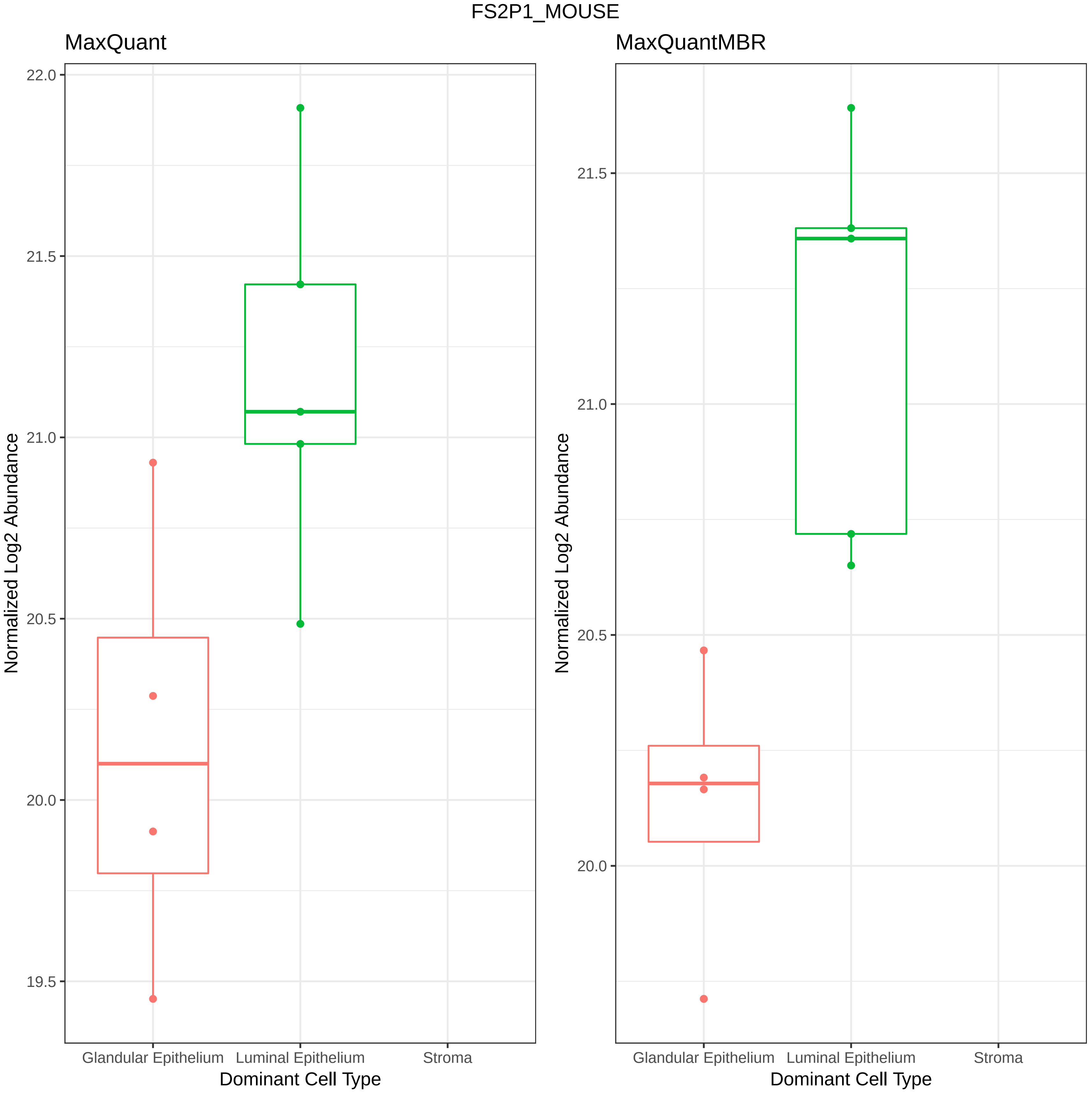
FS2P1_MOUSE
MaxQuant
MaxQuantMBR
Normalized Log2 Abundance
22.0
21.5
21.0
20.5
20.0
19.5
Glandular Epithelium
Luminal Epithelium
Stroma
Dominant Cell Type

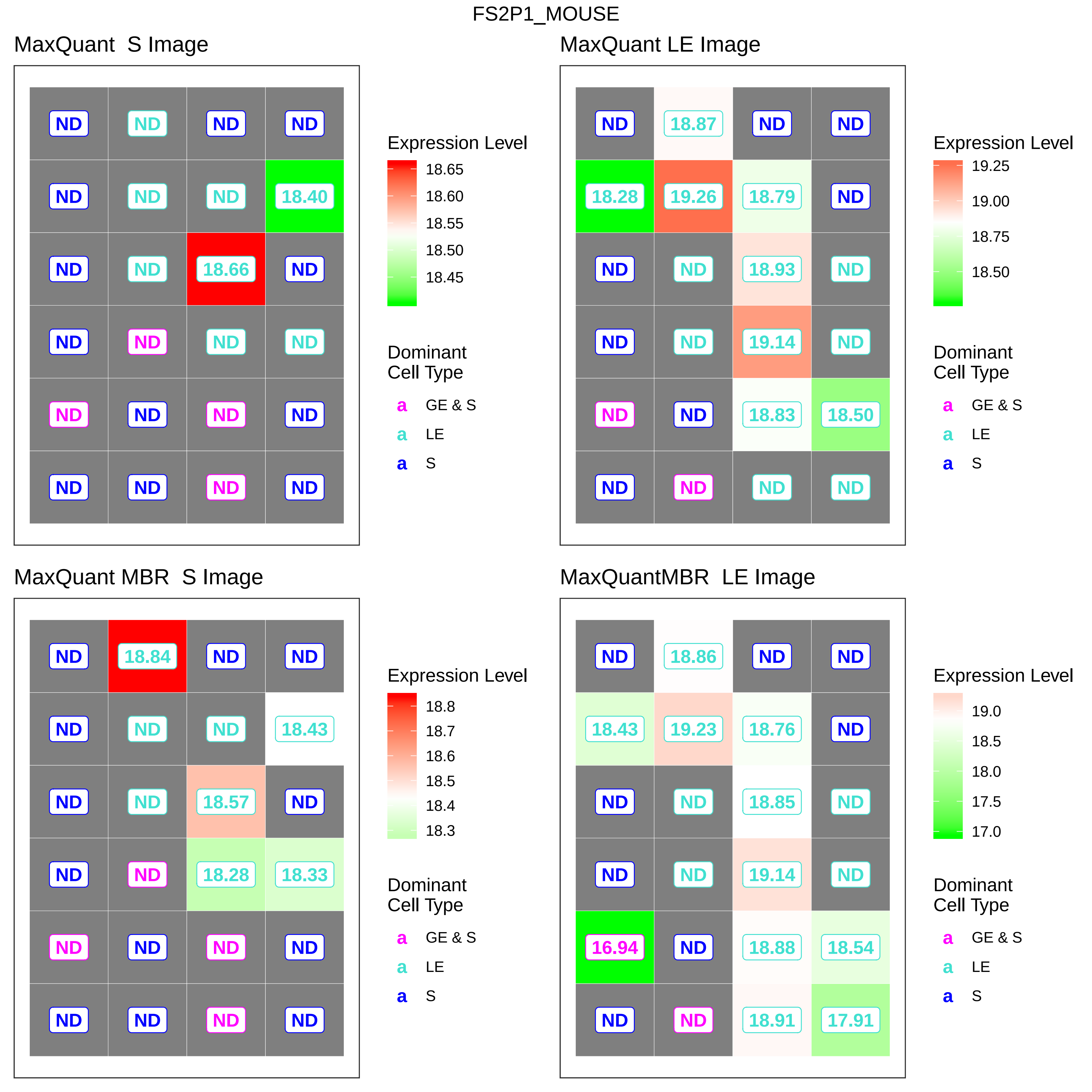
FS2P1_MOUSE
MaxQuant S Image
ND ND ND ND
ND ND ND 18.40
ND ND 18.66 ND
ND ND ND ND
ND ND ND ND
ND ND ND ND
Expression Level
18.65
18.60
18.55
18.50
18.45
Dominant Cell Type
a GE & S
a LE
a S
MaxQuant LE Image
ND 18.87 ND ND
18.28 19.26 18.79 ND
ND ND 18.93 ND
ND ND 19.14 ND
ND ND 18.83 18.50
ND ND ND ND
Expression Level
19.25
19.00
18.75
18.50
Dominant Cell Type
a GE & S
a LE
a S
MaxQuant MBR S Image
ND 18.84 ND ND
ND ND ND 18.43
ND ND 18.57 ND
ND ND 18.28 18.33
ND ND ND ND
ND ND ND ND
Expression Level
18.8
18.7
18.6
18.5
18.4
18.3
Dominant Cell Type
a GE & S
a LE
a S
MaxQuantMBR LE Image
ND 18.86 ND ND
18.43 19.23 18.76 ND
ND ND 18.85 ND
ND ND 19.14 ND
16.94 ND 18.88 18.54
ND ND 18.91 17.91
Expression Level
19.0
18.5
18.0
17.5
17.0
Dominant Cell Type
a GE & S
a LE
a S

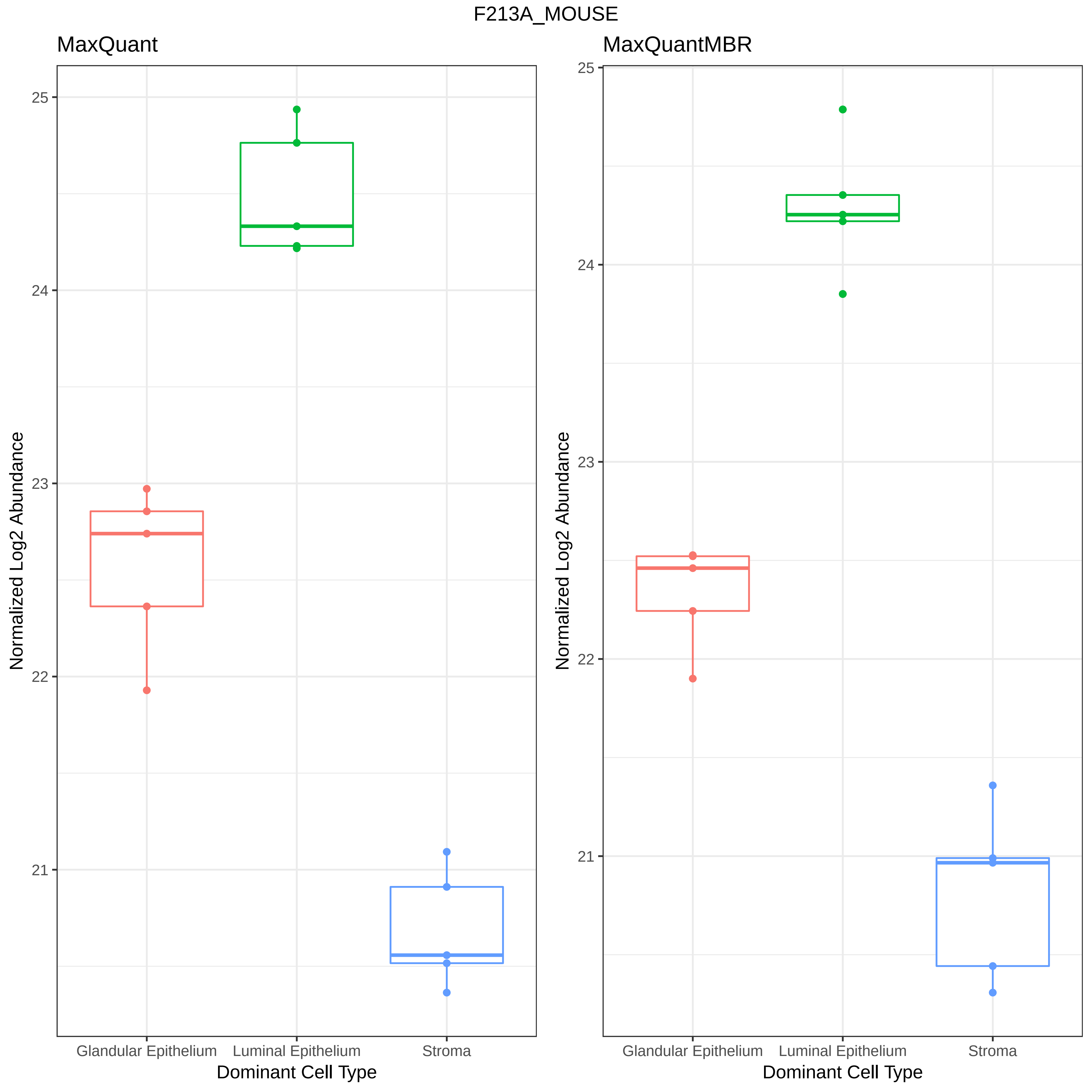
F213A_MOUSE
MaxQuant
MaxQuantMBR
Normalized Log2 Abundance
25
24
23
22
21
Glandular Epithelium
Luminal Epithelium
Stroma
Dominant Cell Type

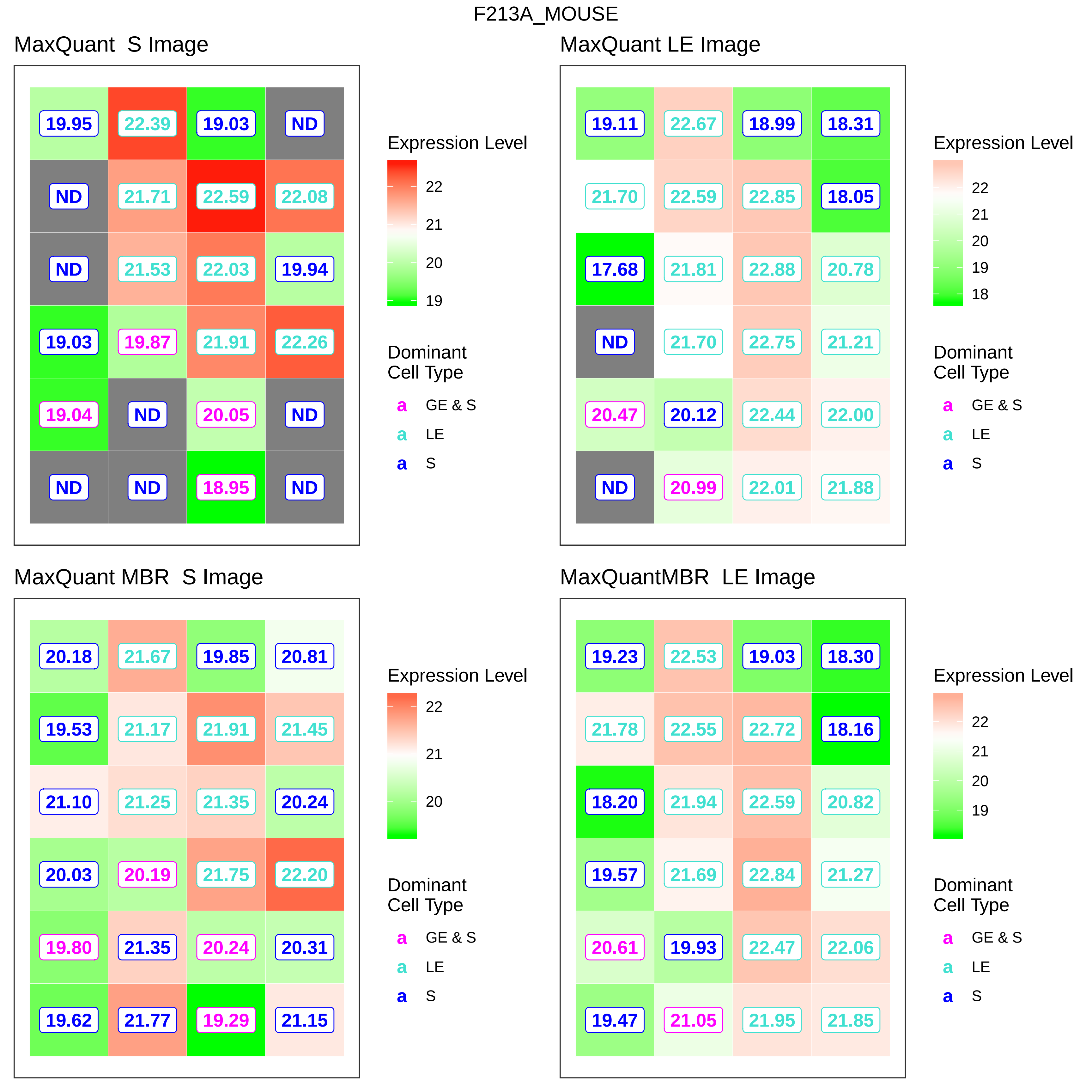
F213A_MOUSE
MaxQuant S Image
19.95 22.39 19.03 ND
ND 21.71 22.59 22.08
ND 21.53 22.03 19.94
19.03 19.87 21.91 22.26
19.04 ND 20.05 ND
ND ND 18.95 ND
Expression Level
22
21
20
19
Dominant Cell Type
a GE & S
a LE
a S
MaxQuant LE Image
19.11 22.67 18.99 18.31
21.70 22.59 22.85 18.05
17.68 21.81 22.88 20.78
ND 21.70 22.75 21.21
20.47 20.12 22.44 22.00
ND 20.99 22.01 21.88
Expression Level
22
21
20
19
18
Dominant Cell Type
a GE & S
a LE
a S
MaxQuant MBR S Image
20.18 21.67 19.85 20.81
19.53 21.17 21.91 21.45
21.10 21.25 21.35 20.24
20.03 20.19 21.75 22.20
19.80 21.35 20.24 20.31
19.62 21.77 19.29 21.15
Expression Level
22
21
20
Dominant Cell Type
a GE & S
a LE
a S
MaxQuantMBR LE Image
19.23 22.53 19.03 18.30
21.78 22.55 22.72 18.16
18.20 21.94 22.59 20.82
19.57 21.69 22.84 21.27
20.61 19.93 22.47 22.06
19.47 21.05 21.95 21.85
Expression Level
22
21
20
19
Dominant Cell Type
a GE & S
a LE
a S

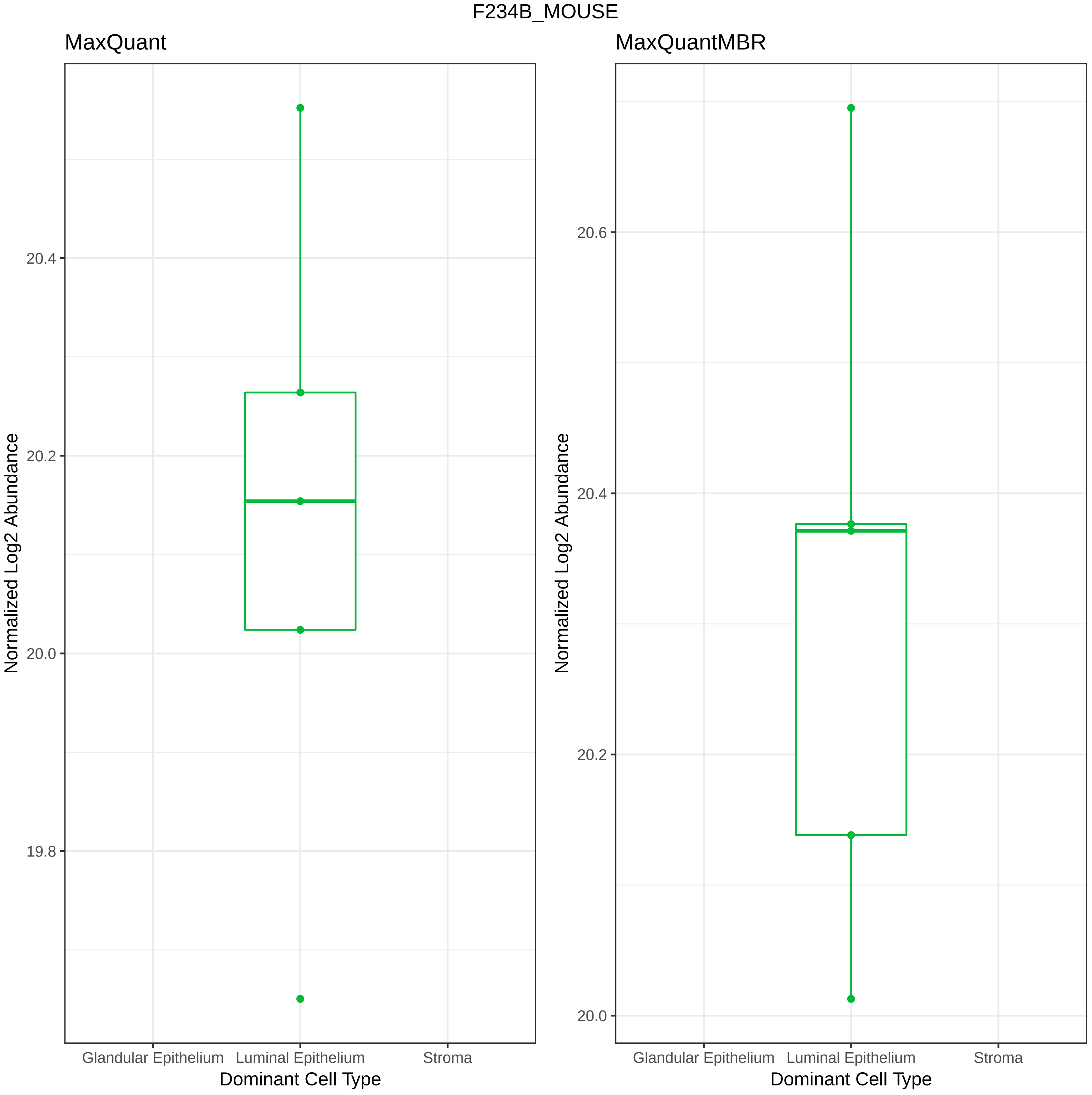

F234B_MOUSE
MaxQuant
MaxQuantMBR
Normalized Log2 Abundance
20.4
20.2
20.0
19.8
20.6
20.4
20.2
20.0
Glandular Epithelium
Luminal Epithelium
Stroma
Dominant Cell Type

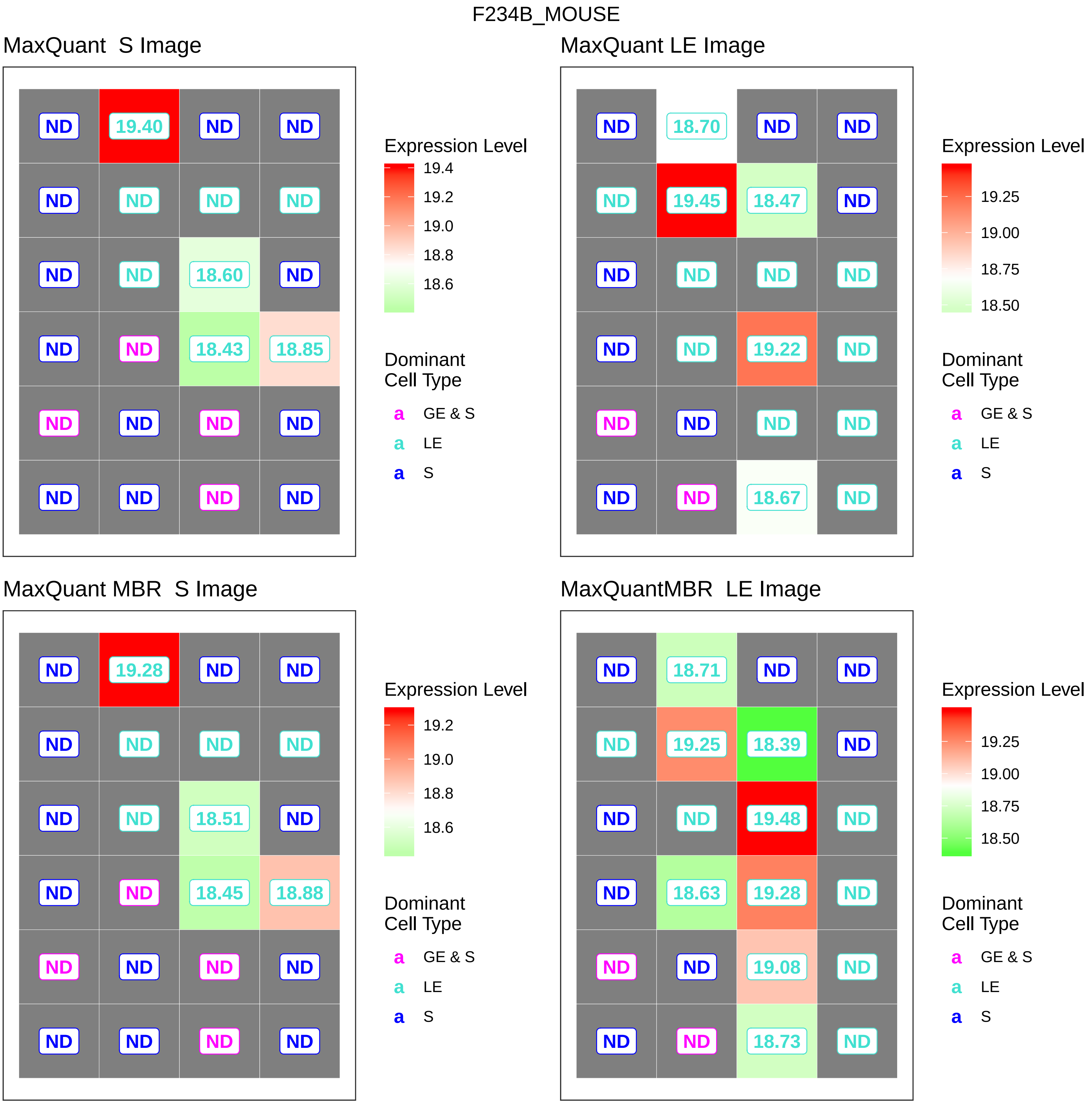
F234B_MOUSE
MaxQuant S Image
ND 19.40 ND ND
ND ND ND ND
ND ND 18.60 ND
ND ND 18.43 18.85
ND ND ND ND
ND ND ND ND
Expression Level
19.4
19.2
19.0
18.8
18.6
Dominant Cell Type
a GE & S
a LE
a S
MaxQuant LE Image
ND 18.70 ND ND
ND 19.45 18.47 ND
ND ND ND ND
ND ND 19.22 ND
ND ND ND ND
ND ND 18.67 ND
Expression Level
19.25
19.00
18.75
18.50
Dominant Cell Type
a GE & S
a LE
a S
MaxQuant MBR S Image
ND 19.28 ND ND
ND ND ND ND
ND ND 18.51 ND
ND ND 18.45 18.88
ND ND ND ND
ND ND ND ND
Expression Level
19.2
19.0
18.8
18.6
Dominant Cell Type
a GE & S
a LE
a S
MaxQuantMBR LE Image
ND 18.71 ND ND
ND 19.25 18.39 ND
ND ND 19.48 ND
ND 18.63 19.28 ND
ND ND 19.08 ND
ND ND 18.73 ND
Expression Level
19.25
19.00
18.75
18.50
Dominant Cell Type
a GE & S
a LE
a S

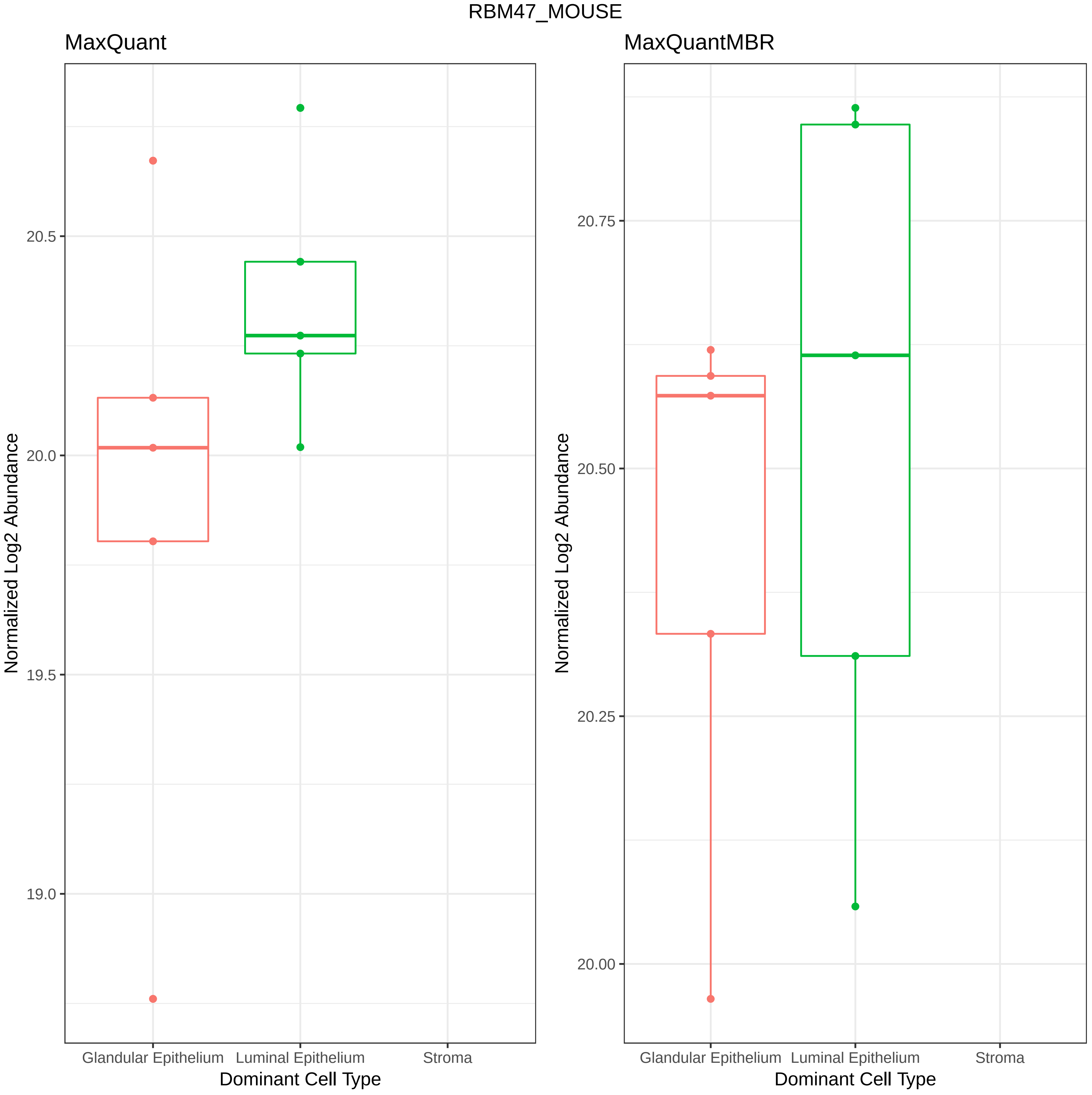
RBM47_MOUSE
MaxQuant
MaxQuantMBR
Normalized Log2 Abundance
20.5
20.0
19.5
19.0
20.75
20.50
20.25
20.00
Glandular Epithelium
Luminal Epithelium
Stroma
Dominant Cell Type

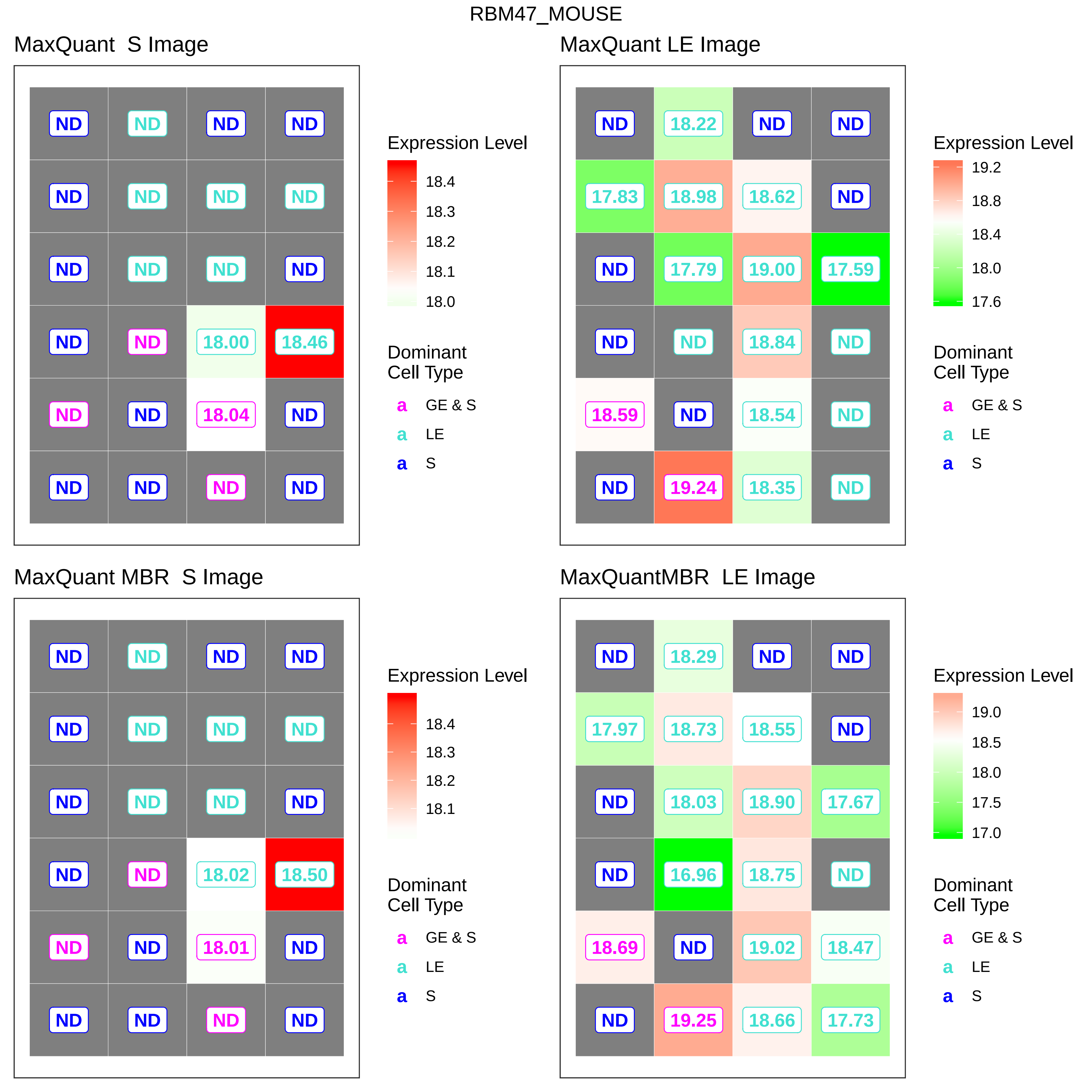

RBM47_MOUSE
MaxQuant S Image
ND ND ND ND
ND ND ND ND
ND ND ND ND
ND ND 18.00 18.46
ND ND 18.04 ND
ND ND ND ND
Expression Level
18.4
18.3
18.2
18.1
18.0
Dominant Cell Type
a GE & S
a LE
a S
MaxQuant LE Image
ND 18.22 ND ND
17.83 18.98 18.62 ND
ND 17.79 19.00 17.59
ND ND 18.84 ND
18.59 ND 18.54 ND
ND 19.24 18.35 ND
Expression Level
19.2
18.8
18.4
18.0
17.6
Dominant Cell Type
a GE & S
a LE
a S
MaxQuant MBR S Image
ND ND ND ND
ND ND ND ND
ND ND ND ND
ND ND 18.02 18.50
ND ND 18.01 ND
ND ND ND ND
Expression Level
18.4
18.3
18.2
18.1
Dominant Cell Type
a GE & S
a LE
a S
MaxQuantMBR LE Image
ND 18.29 ND ND
17.97 18.73 18.55 ND
ND 18.03 18.90 17.67
ND 16.96 18.75 ND
18.69 ND 19.02 18.47
ND 19.25 18.66 17.73
Expression Level
19.0
18.5
18.0
17.5
17.0
Dominant Cell Type
a GE & S
a LE
a S

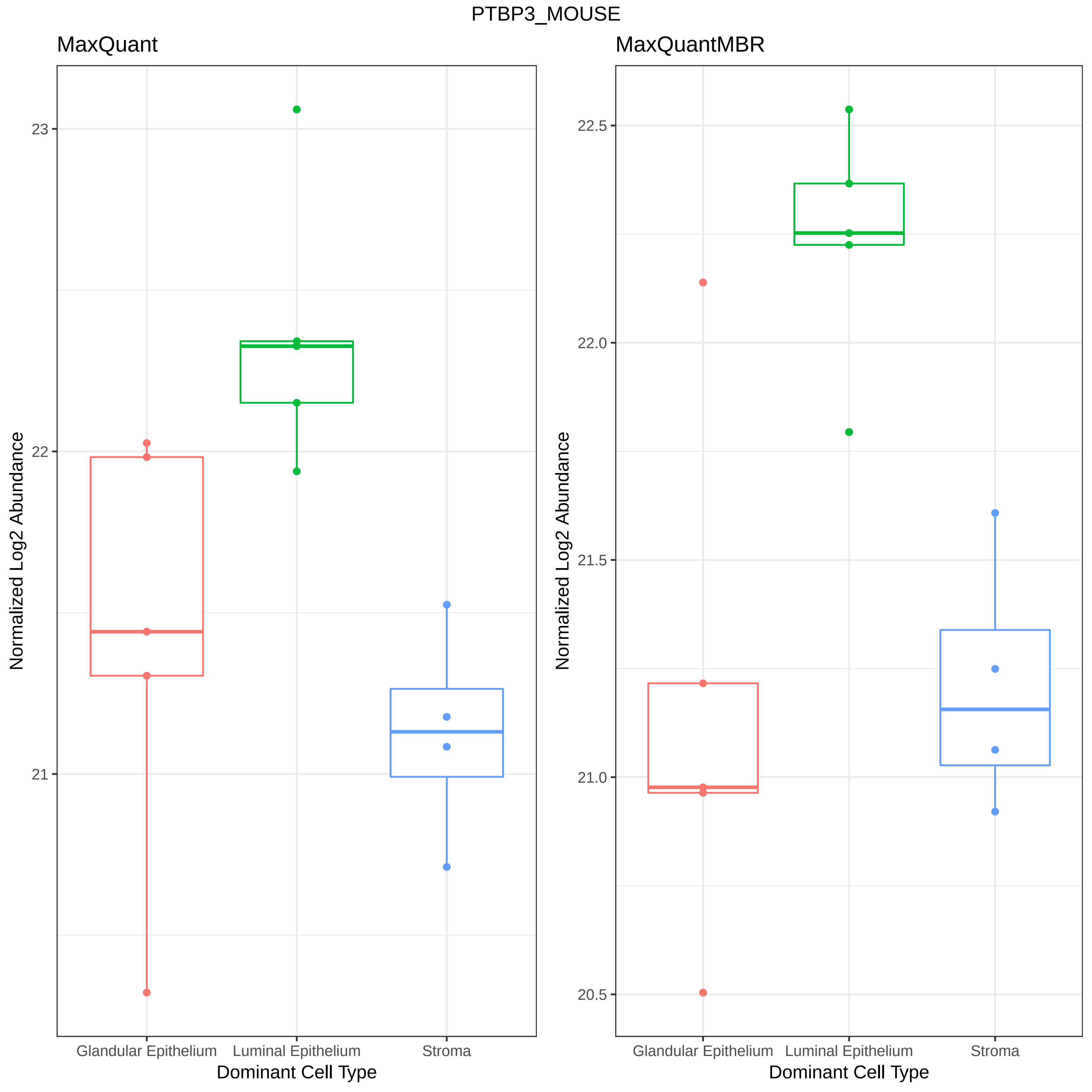
PTBP3_MOUSE
MaxQuant
MaxQuantMBR
Normalized Log2 Abundance
23
22
21
22.5
22.0
21.5
21.0
20.5
Glandular Epithelium
Luminal Epithelium
Stroma
Dominant Cell Type

PTBP3_MOUSE

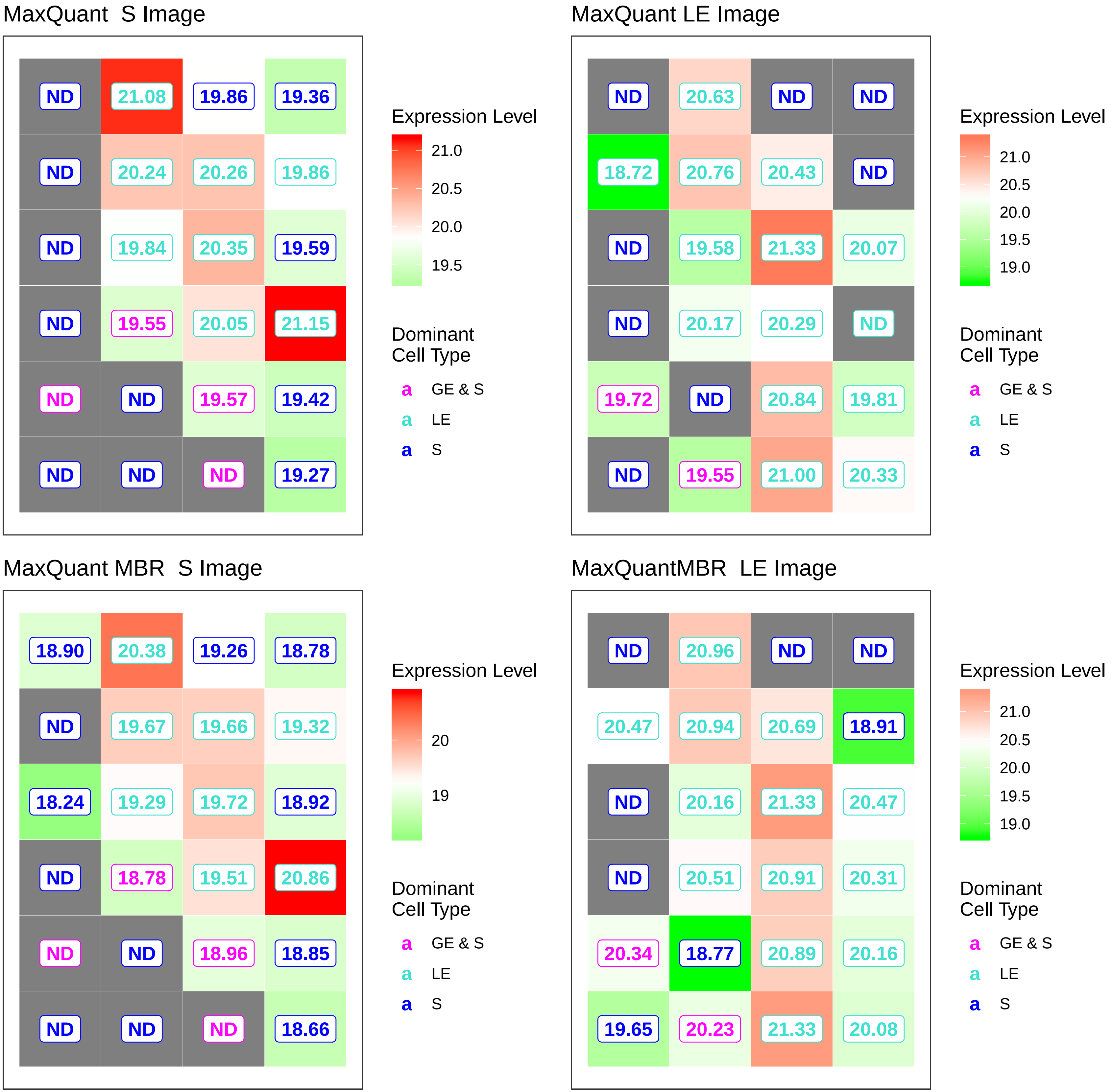
MaxQuant S Image
ND
21.08
19.86
19.36
ND
20.24
20.26
19.86
ND
19.84
20.35
19.59
ND
19.55
20.05
21.15
ND
ND
19.57
19.42
ND
ND
ND
19.27
Expression Level
21.0
20.5
20.0
19.5
Dominant Cell Type
a GE & S
a LE
a S
MaxQuant LE Image
ND
20.63
ND
ND
18.72
20.76
20.43
ND
ND
19.58
21.33
20.07
ND
20.17
20.29
ND
19.72
ND
20.84
19.81
ND
19.55
21.00
20.33
Expression Level
21.0
20.5
20.0
19.5
19.0
Dominant Cell Type
a GE & S
a LE
a S
MaxQuant MBR S Image
18.90
20.38
19.26
18.78
ND
19.67
19.66
19.32
18.24
19.29
19.72
18.92
ND
18.78
19.51
20.86
ND
ND
18.96
18.85
ND
ND
ND
18.66
Expression Level
20
19
Dominant Cell Type
a GE & S
a LE
a S
MaxQuantMBR LE Image
ND
20.96
ND
ND
20.47
20.94
20.69
18.91
ND
20.16
21.33
20.47
ND
20.51
20.91
20.31
20.34
18.77
20.89
20.16
19.65
20.23
21.33
20.08
Expression Level
21.0
20.5
20.0
19.5
19.0
Dominant Cell Type
a GE & S
a LE
a S

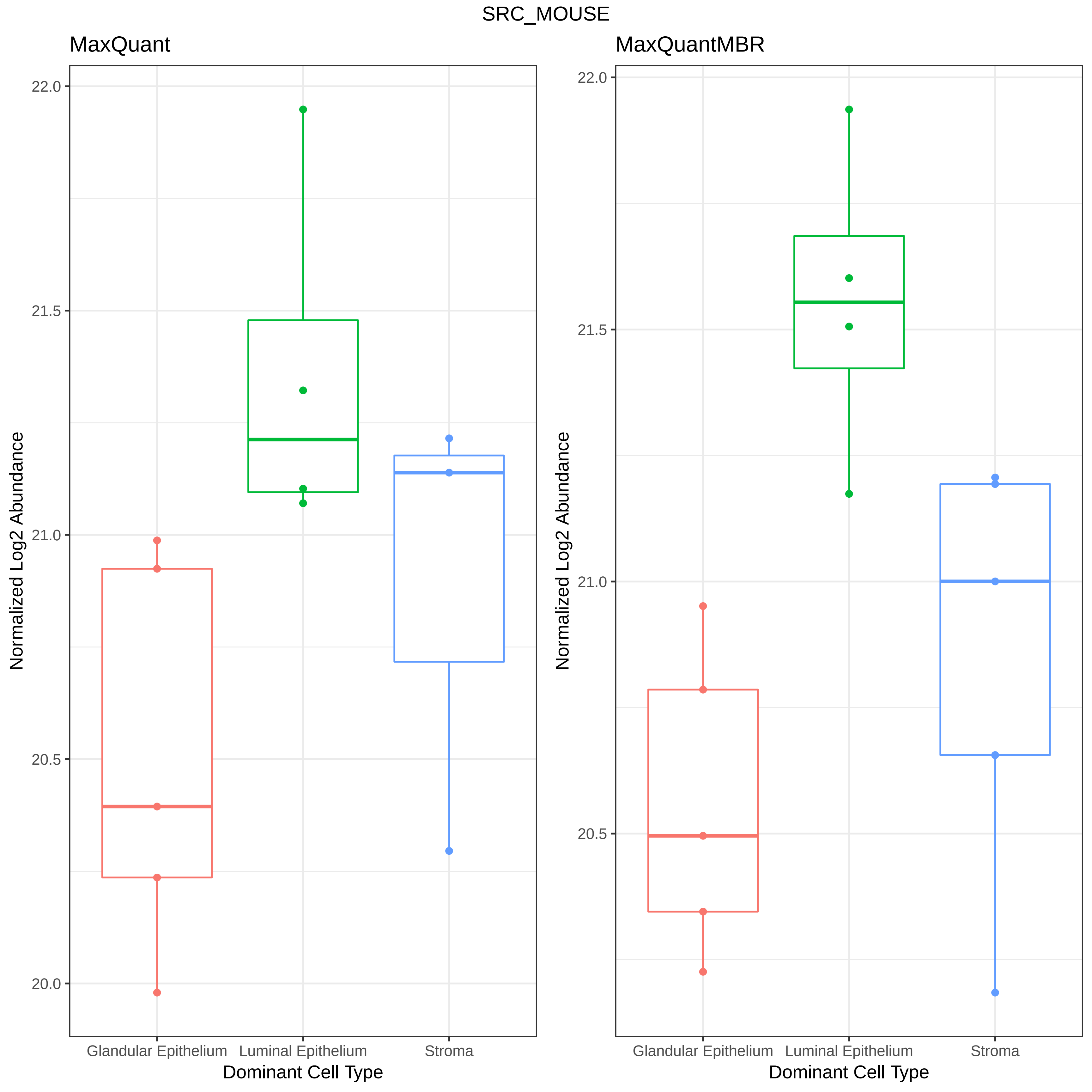
SRC_MOUSE
MaxQuant
MaxQuantMBR
Normalized Log2 Abundance
22.0
21.5
21.0
20.5
20.0
Glandular Epithelium
Luminal Epithelium
Stroma
Dominant Cell Type

SRC_MOUSE

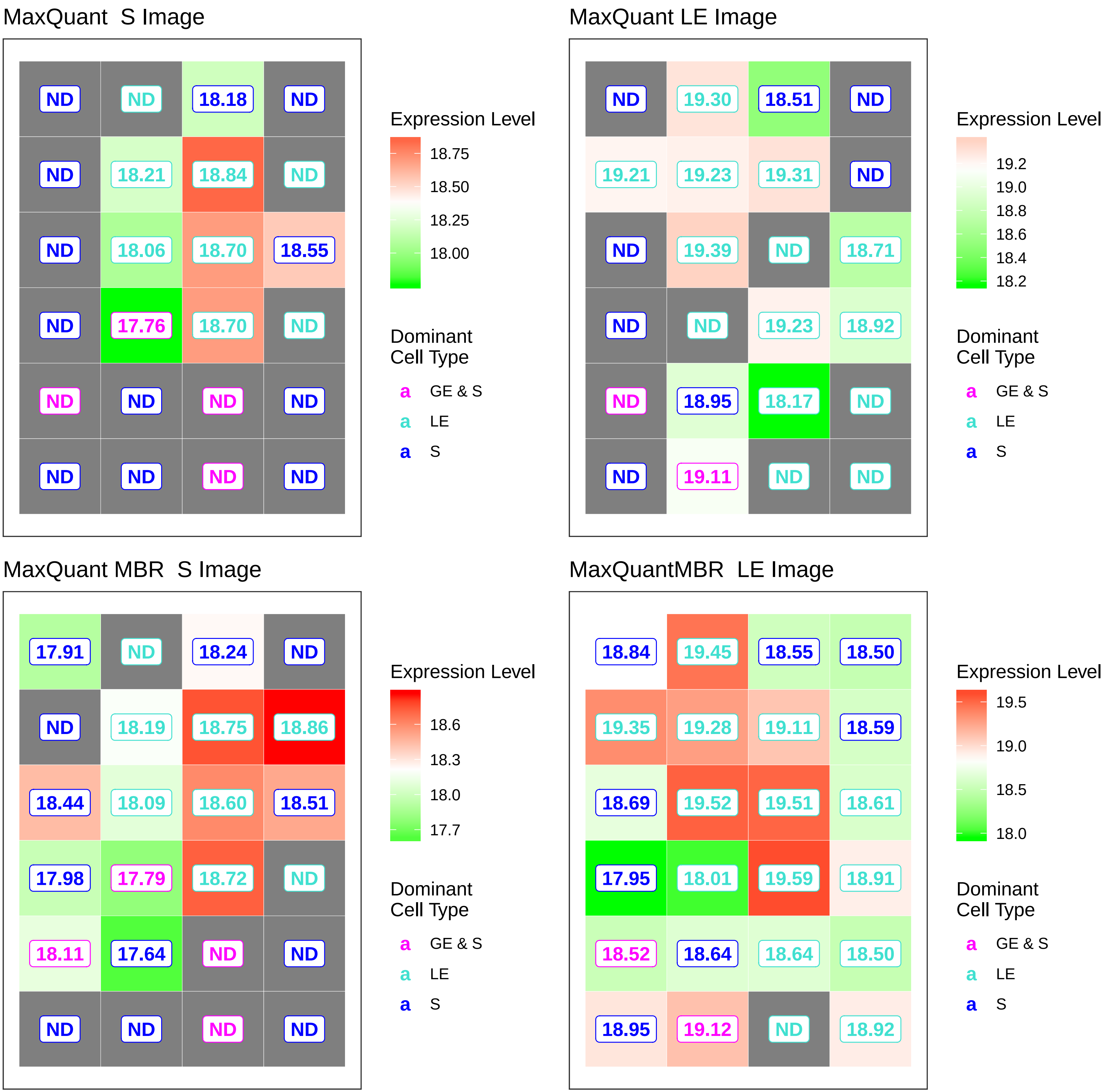
MaxQuant S Image
ND ND 18.18 ND
ND 18.21 18.84 ND
ND 18.06 18.70 18.55
ND 17.76 18.70 ND
ND ND ND ND
ND ND ND ND
Expression Level
18.75
18.50
18.25
18.00
Dominant Cell Type
a GE & S
a LE
a S
MaxQuant LE Image
ND 19.30 18.51 ND
19.21 19.23 19.31 ND
ND 19.39 ND 18.71
ND ND 19.23 18.92
ND 18.95 18.17 ND
ND 19.11 ND ND
Expression Level
19.2
19.0
18.8
18.6
18.4
18.2
Dominant Cell Type
a GE & S
a LE
a S
MaxQuant MBR S Image
17.91 ND 18.24 ND
ND 18.19 18.75 18.86
18.44 18.09 18.60 18.51
17.98 17.79 18.72 ND
18.11 17.64 ND ND
ND ND ND ND
Expression Level
18.6
18.3
18.0
17.7
Dominant Cell Type
a GE & S
a LE
a S
MaxQuantMBR LE Image
18.84 19.45 18.55 18.50
19.35 19.28 19.11 18.59
18.69 19.52 19.51 18.61
17.95 18.01 19.59 18.91
18.52 18.64 18.64 18.50
18.95 19.12 ND 18.92
Expression Level
19.5
19.0
18.5
18.0
Dominant Cell Type
a GE & S
a LE
a S

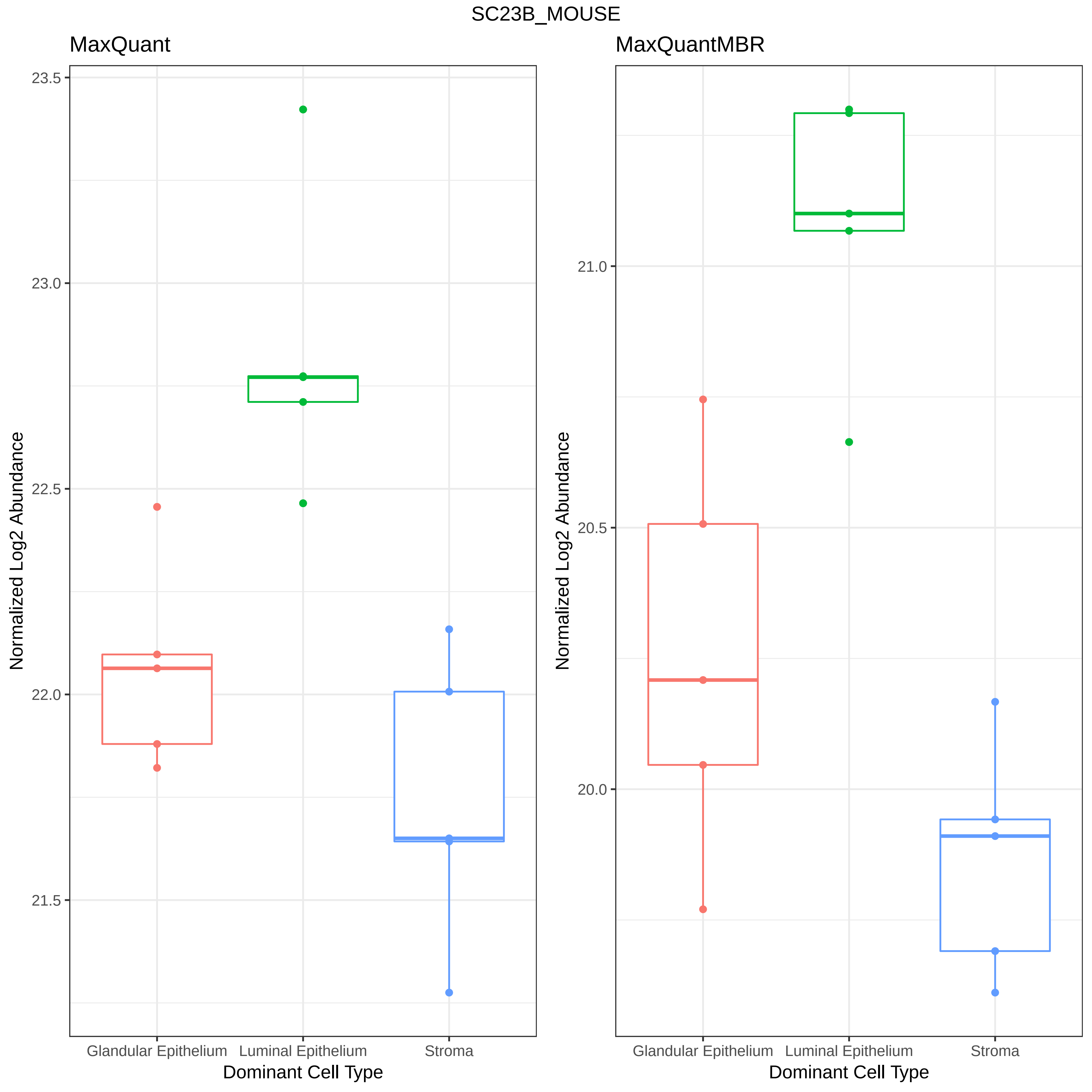

SC23B_MOUSE
MaxQuant
MaxQuantMBR
Normalized Log2 Abundance
23.5
23.0
22.5
22.0
21.5
21.0
20.5
20.0
Glandular Epithelium
Luminal Epithelium
Stroma
Dominant Cell Type

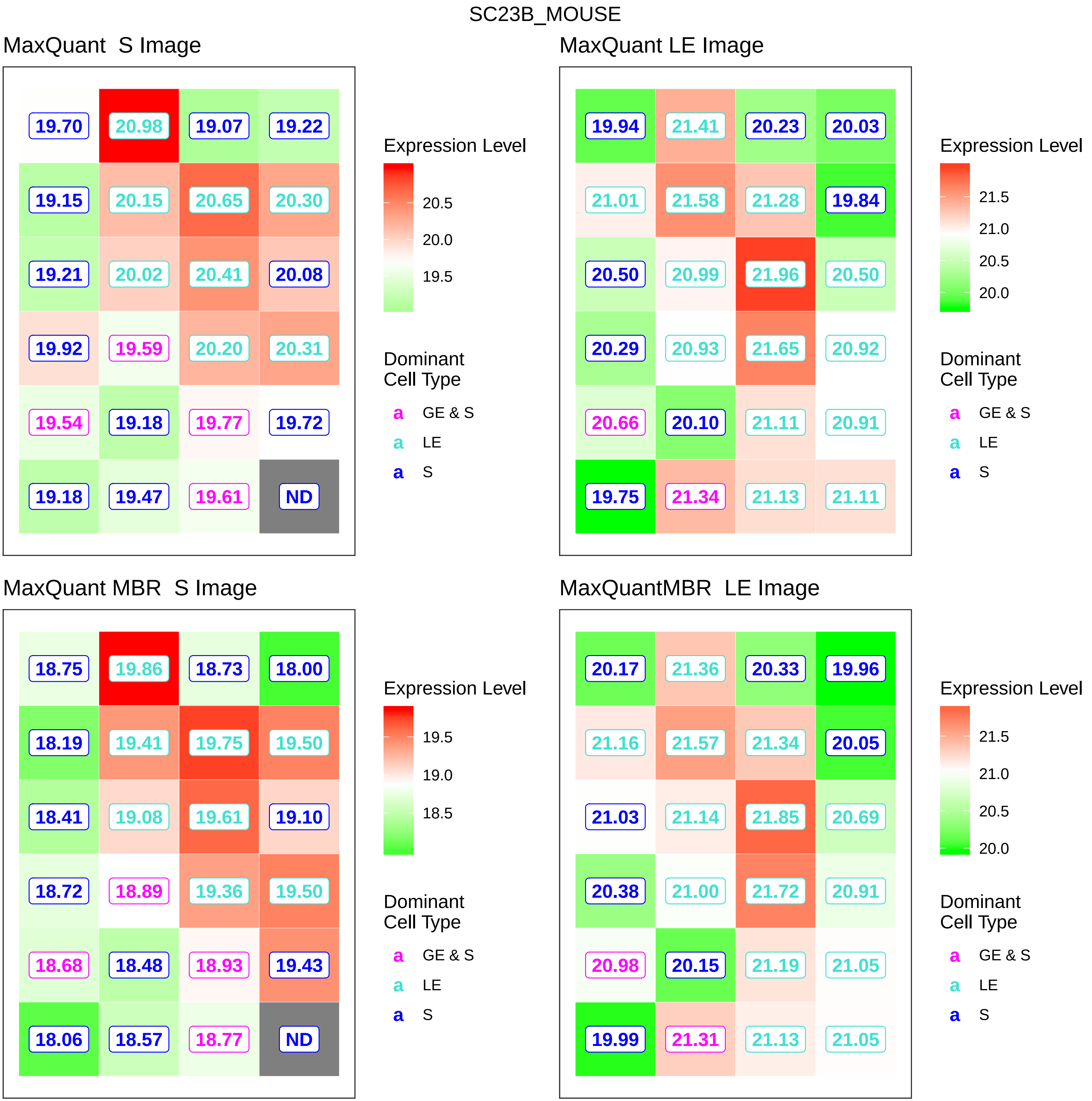
SC23B_MOUSE
MaxQuant S Image
19.70 20.98 19.07 19.22
19.15 20.15 20.65 20.30
19.21 20.02 20.41 20.08
19.92 19.59 20.20 20.31
19.54 19.18 19.77 19.72
19.18 19.47 19.61 ND
Expression Level
20.5
20.0
19.5
Dominant Cell Type
a GE & S
a LE
a S
MaxQuant LE Image
19.94 21.41 20.23 20.03
21.01 21.58 21.28 19.84
20.50 20.99 21.96 20.50
20.29 20.93 21.65 20.92
20.66 20.10 21.11 20.91
19.75 21.34 21.13 21.11
Expression Level
21.5
21.0
20.5
20.0
Dominant Cell Type
a GE & S
a LE
a S
MaxQuant MBR S Image
18.75 19.86 18.73 18.00
18.19 19.41 19.75 19.50
18.41 19.08 19.61 19.10
18.72 18.89 19.36 19.50
18.68 18.48 18.93 19.43
18.06 18.57 18.77 ND
Expression Level
19.5
19.0
18.5
Dominant Cell Type
a GE & S
a LE
a S
MaxQuantMBR LE Image
20.17 21.36 20.33 19.96
21.16 21.57 21.34 20.05
21.03 21.14 21.85 20.69
20.38 21.00 21.72 20.91
20.98 20.15 21.19 21.05
19.99 21.31 21.13 21.05
Expression Level
21.5
21.0
20.5
20.0
Dominant Cell Type
a GE & S
a LE
a S

SE1L1_MOUSE

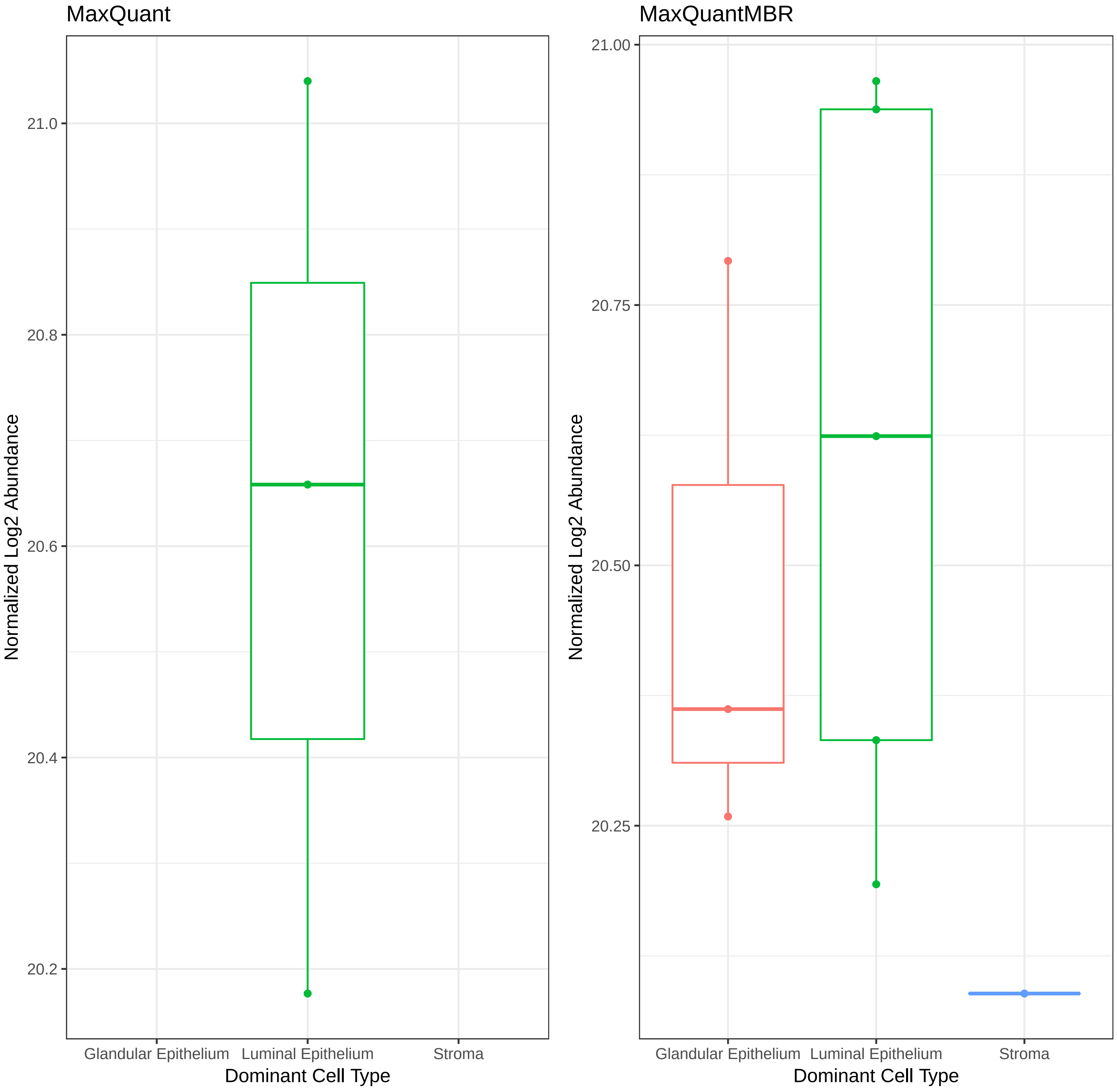

MaxQuant
MaxQuantMBR
Normalized Log2 Abundance
Dominant Cell Type
21.0
20.8
20.6
20.4
20.2
21.00
20.75
20.50
20.25
Glandular Epithelium
Luminal Epithelium
Stroma

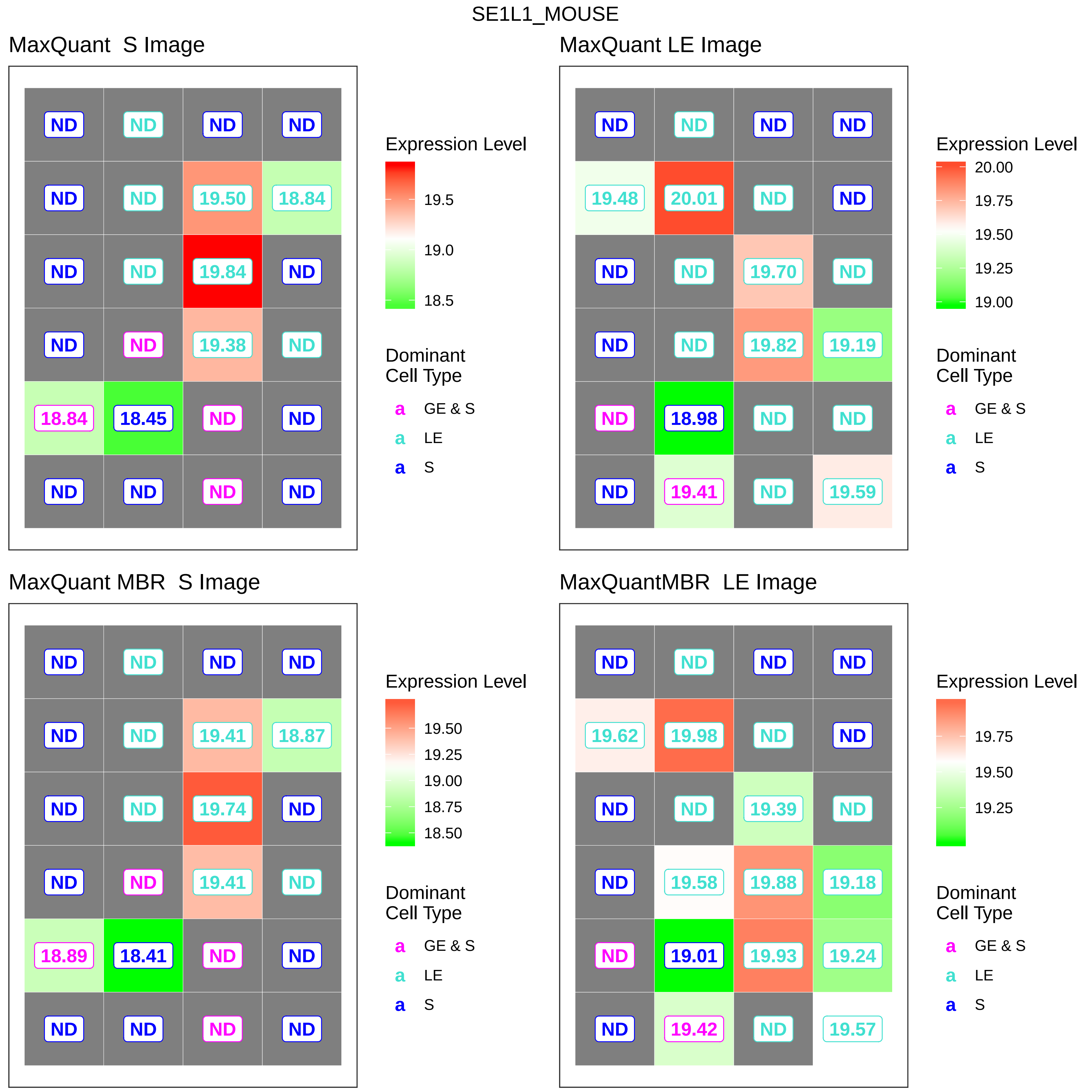
SE1L1_MOUSE
MaxQuant S Image
ND ND ND ND
ND ND 19.50 18.84
ND ND 19.84 ND
ND ND 19.38 ND
18.84 18.45 ND ND
ND ND ND ND
Expression Level
19.5
19.0
18.5
Dominant Cell Type
a GE & S
a LE
a S
MaxQuant LE Image
ND ND ND ND
19.48 20.01 ND ND
ND ND 19.70 ND
ND ND 19.82 19.19
ND 18.98 ND ND
ND 19.41 ND 19.59
Expression Level
20.00
19.75
19.50
19.25
19.00
Dominant Cell Type
a GE & S
a LE
a S
MaxQuant MBR S Image
ND ND ND ND
ND ND 19.41 18.87
ND ND 19.74 ND
ND ND 19.41 ND
18.89 18.41 ND ND
ND ND ND ND
Expression Level
19.50
19.25
19.00
18.75
18.50
Dominant Cell Type
a GE & S
a LE
a S
MaxQuantMBR LE Image
ND ND ND ND
19.62 19.98 ND ND
ND ND 19.39 ND
ND 19.58 19.88 19.18
ND 19.01 19.93 19.24
ND 19.42 ND 19.57
Expression Level
19.75
19.50
19.25
Dominant Cell Type
a GE & S
a LE
a S

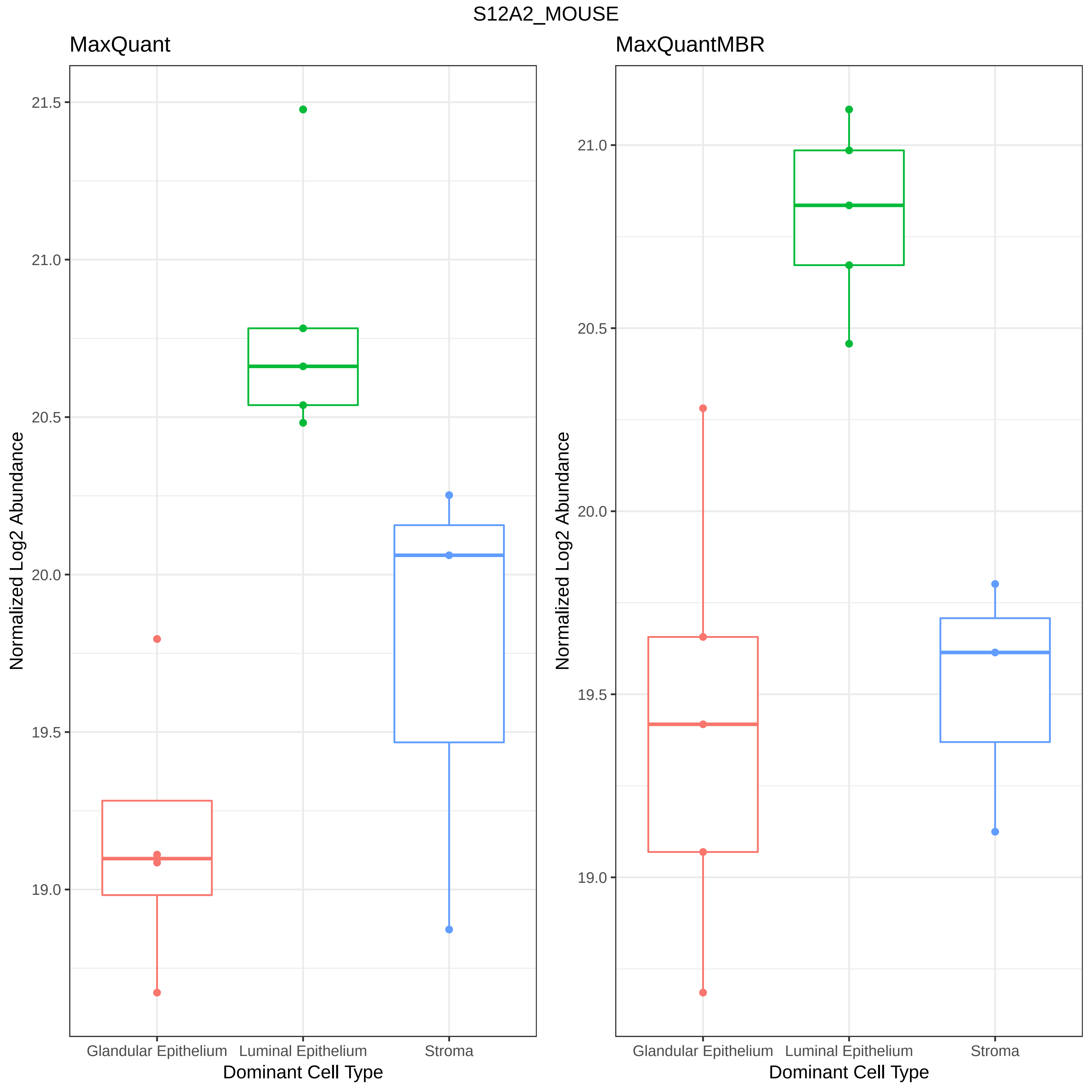

S12A2_MOUSE
MaxQuant
MaxQuantMBR
Normalized Log2 Abundance
21.5
21.0
20.5
20.0
19.5
19.0
Glandular Epithelium
Luminal Epithelium
Stroma
Dominant Cell Type

S12A2_MOUSE

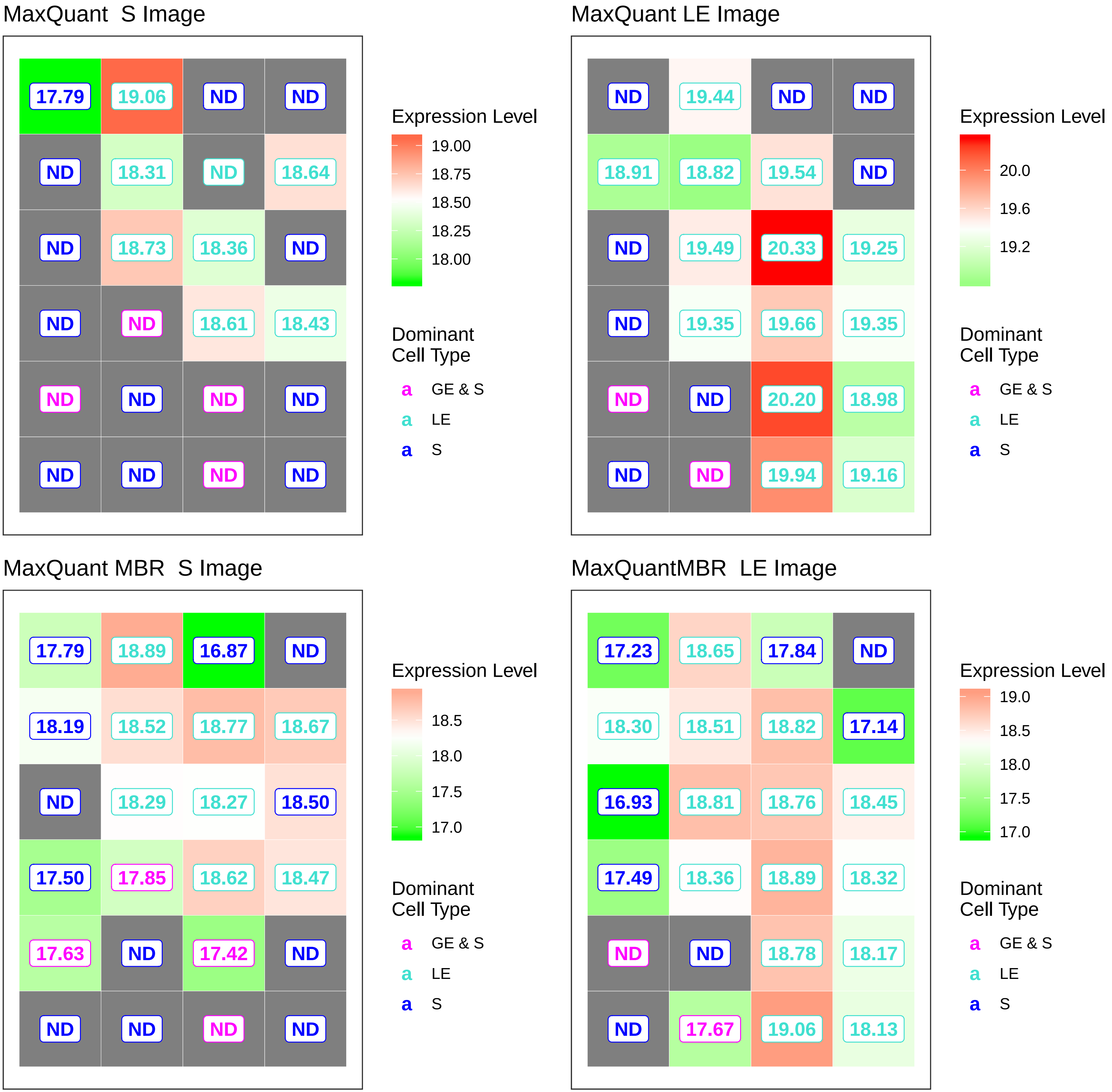
MaxQuant S Image
17.79 19.06 ND ND
ND 18.31 ND 18.64
ND 18.73 18.36 ND
ND ND 18.61 18.43
ND ND ND ND
ND ND ND ND
Expression Level
19.00
18.75
18.50
18.25
18.00
Dominant Cell Type
a GE & S
a LE
a S
MaxQuant LE Image
ND 19.44 ND ND
18.91 18.82 19.54 ND
ND 19.49 20.33 19.25
ND 19.35 19.66 19.35
ND ND 20.20 18.98
ND ND 19.94 19.16
Expression Level
20.0
19.6
19.2
Dominant Cell Type
a GE & S
a LE
a S
MaxQuant MBR S Image
17.79 18.89 16.87 ND
18.19 18.52 18.77 18.67
ND 18.29 18.27 18.50
17.50 17.85 18.62 18.47
17.63 ND 17.42 ND
ND ND ND ND
Expression Level
18.5
18.0
17.5
17.0
Dominant Cell Type
a GE & S
a LE
a S
MaxQuantMBR LE Image
17.23 18.65 17.84 ND
18.30 18.51 18.82 17.14
16.93 18.81 18.76 18.45
17.49 18.36 18.89 18.32
ND ND 18.78 18.17
ND 17.67 19.06 18.13
Expression Level
19.0
18.5
18.0
17.5
17.0
Dominant Cell Type
a GE & S
a LE
a S

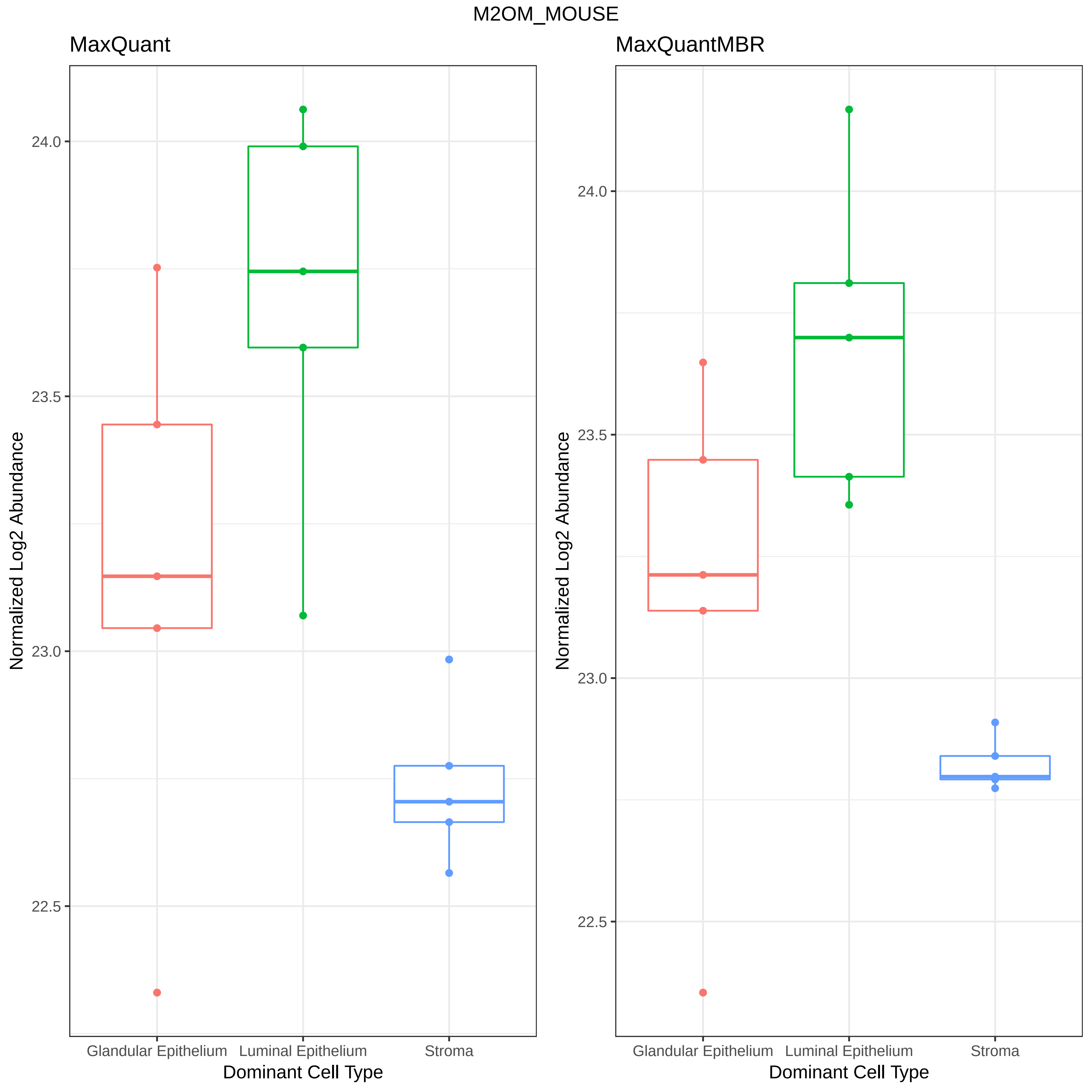
M2OM_MOUSE
MaxQuant
MaxQuantMBR
Normalized Log2 Abundance
24.0
23.5
23.0
22.5
Glandular Epithelium
Luminal Epithelium
Stroma
Dominant Cell Type

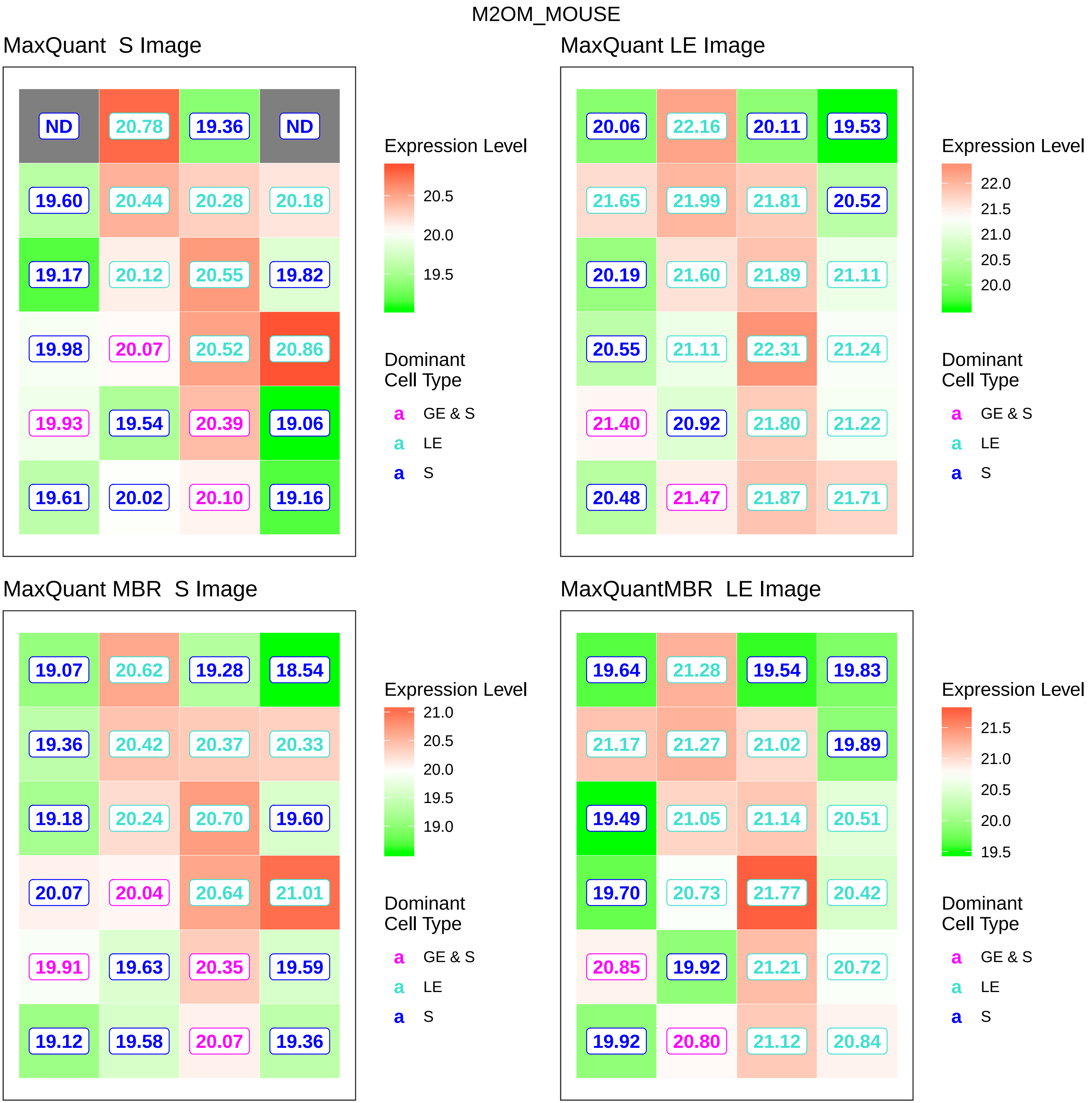
M2OM_MOUSE
MaxQuant S Image
ND 20.78 19.36 ND
19.60 20.44 20.28 20.18
19.17 20.12 20.55 19.82
19.98 20.07 20.52 20.86
19.93 19.54 20.39 19.06
19.61 20.02 20.10 19.16
Expression Level
20.5
20.0
19.5
Dominant Cell Type
a GE & S
a LE
a S
MaxQuant LE Image
20.06 22.16 20.11 19.53
21.65 21.99 21.81 20.52
20.19 21.60 21.89 21.11
20.55 21.11 22.31 21.24
21.40 20.92 21.80 21.22
20.48 21.47 21.87 21.71
Expression Level
22.0
21.5
21.0
20.5
20.0
Dominant Cell Type
a GE & S
a LE
a S
MaxQuant MBR S Image
19.07 20.62 19.28 18.54
19.36 20.42 20.37 20.33
19.18 20.24 20.70 19.60
20.07 20.04 20.64 21.01
19.91 19.63 20.35 19.59
19.12 19.58 20.07 19.36
Expression Level
21.0
20.5
20.0
19.5
19.0
Dominant Cell Type
a GE & S
a LE
a S
MaxQuantMBR LE Image
19.64 21.28 19.54 19.83
21.17 21.27 21.02 19.89
19.49 21.05 21.14 20.51
19.70 20.73 21.77 20.42
20.85 19.92 21.21 20.72
19.92 20.80 21.12 20.84
Expression Level
21.5
21.0
20.5
20.0
19.5
Dominant Cell Type
a GE & S
a LE
a S

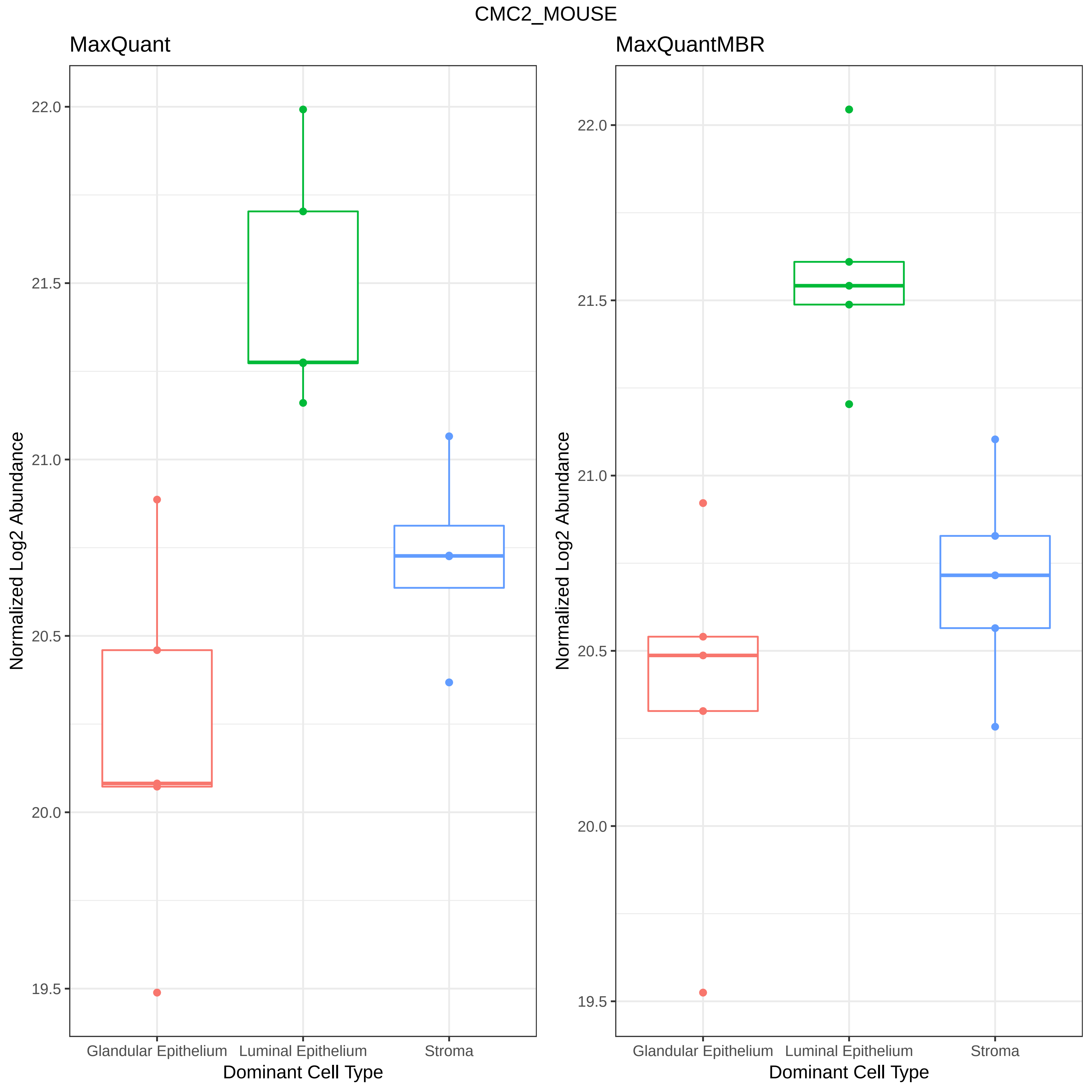
CMC2_MOUSE
MaxQuant
MaxQuantMBR
Normalized Log2 Abundance
22.0
21.5
21.0
20.5
20.0
19.5
Glandular Epithelium
Luminal Epithelium
Stroma
Dominant Cell Type

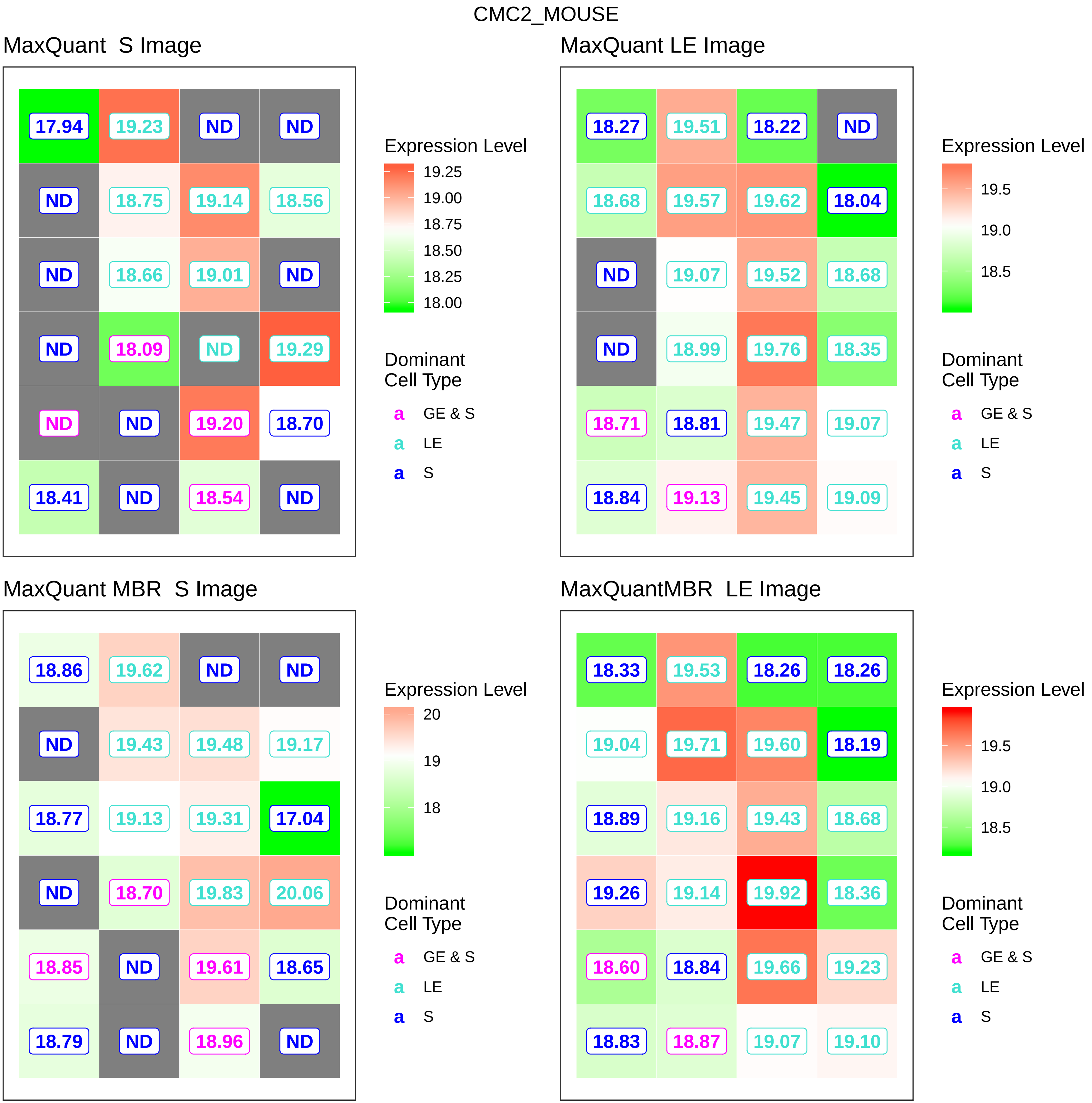
CMC2_MOUSE
MaxQuant S Image
17.94 19.23 ND ND
ND 18.75 19.14 18.56
ND 18.66 19.01 ND
ND 18.09 ND 19.29
ND ND 19.20 18.70
18.41 ND 18.54 ND
Expression Level
19.25
19.00
18.75
18.50
18.25
18.00
Dominant Cell Type
a GE & S
a LE
a S
MaxQuant LE Image
18.27 19.51 18.22 ND
18.68 19.57 19.62 18.04
ND 19.07 19.52 18.68
ND 18.99 19.76 18.35
18.71 18.81 19.47 19.07
18.84 19.13 19.45 19.09
Expression Level
19.5
19.0
18.5
Dominant Cell Type
a GE & S
a LE
a S
MaxQuant MBR S Image
18.86 19.62 ND ND
ND 19.43 19.48 19.17
18.77 19.13 19.31 17.04
ND 18.70 19.83 20.06
18.85 ND 19.61 18.65
18.79 ND 18.96 ND
Expression Level
20
19
18
Dominant Cell Type
a GE & S
a LE
a S
MaxQuantMBR LE Image
18.33 19.53 18.26 18.26
19.04 19.71 19.60 18.19
18.89 19.16 19.43 18.68
19.26 19.14 19.92 18.36
18.60 18.84 19.66 19.23
18.83 18.87 19.07 19.10
Expression Level
19.5
19.0
18.5
Dominant Cell Type
a GE & S
a LE
a S

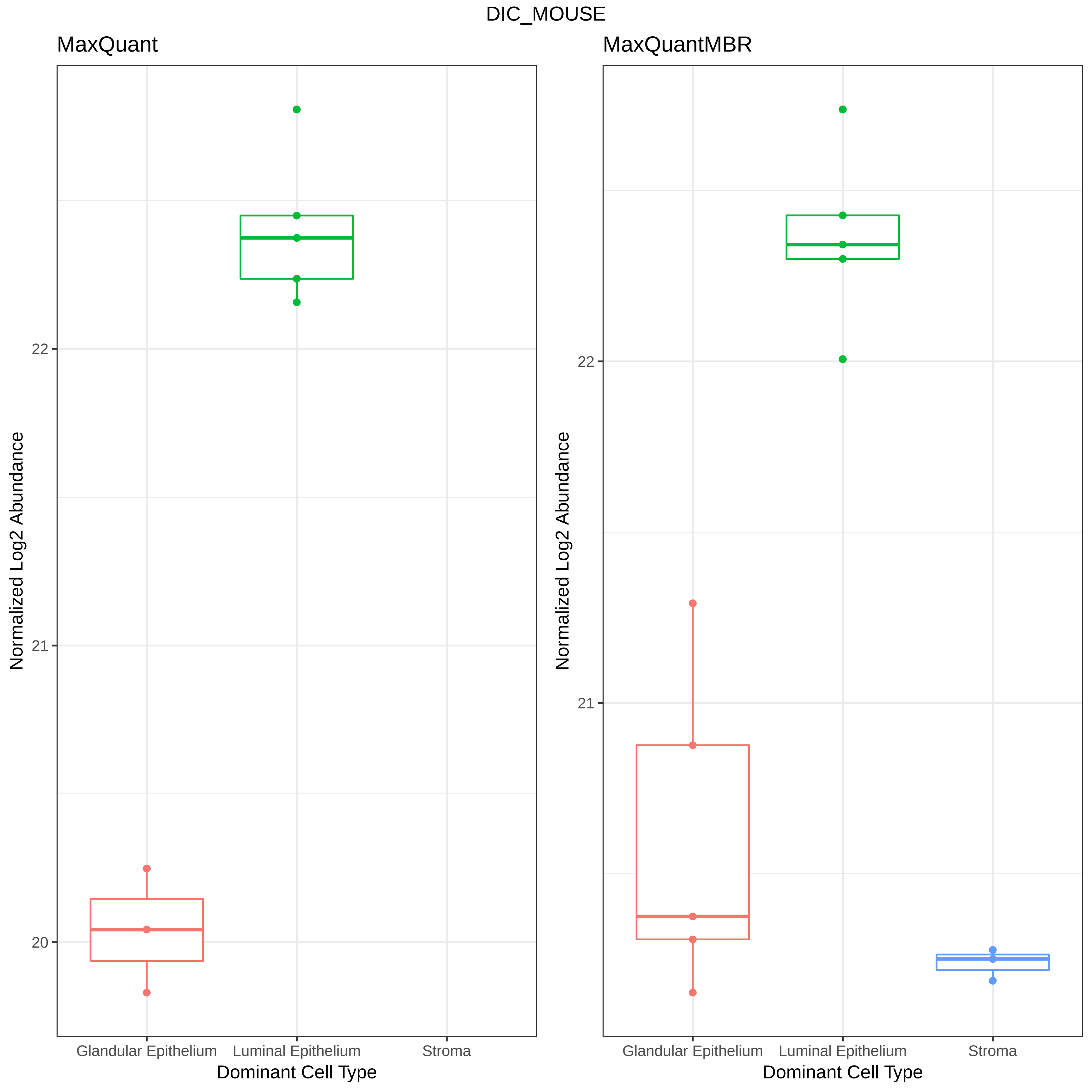
DIC_MOUSE
MaxQuant
MaxQuantMBR
Normalized Log2 Abundance
22
21
20
Glandular Epithelium
Luminal Epithelium
Stroma
Dominant Cell Type

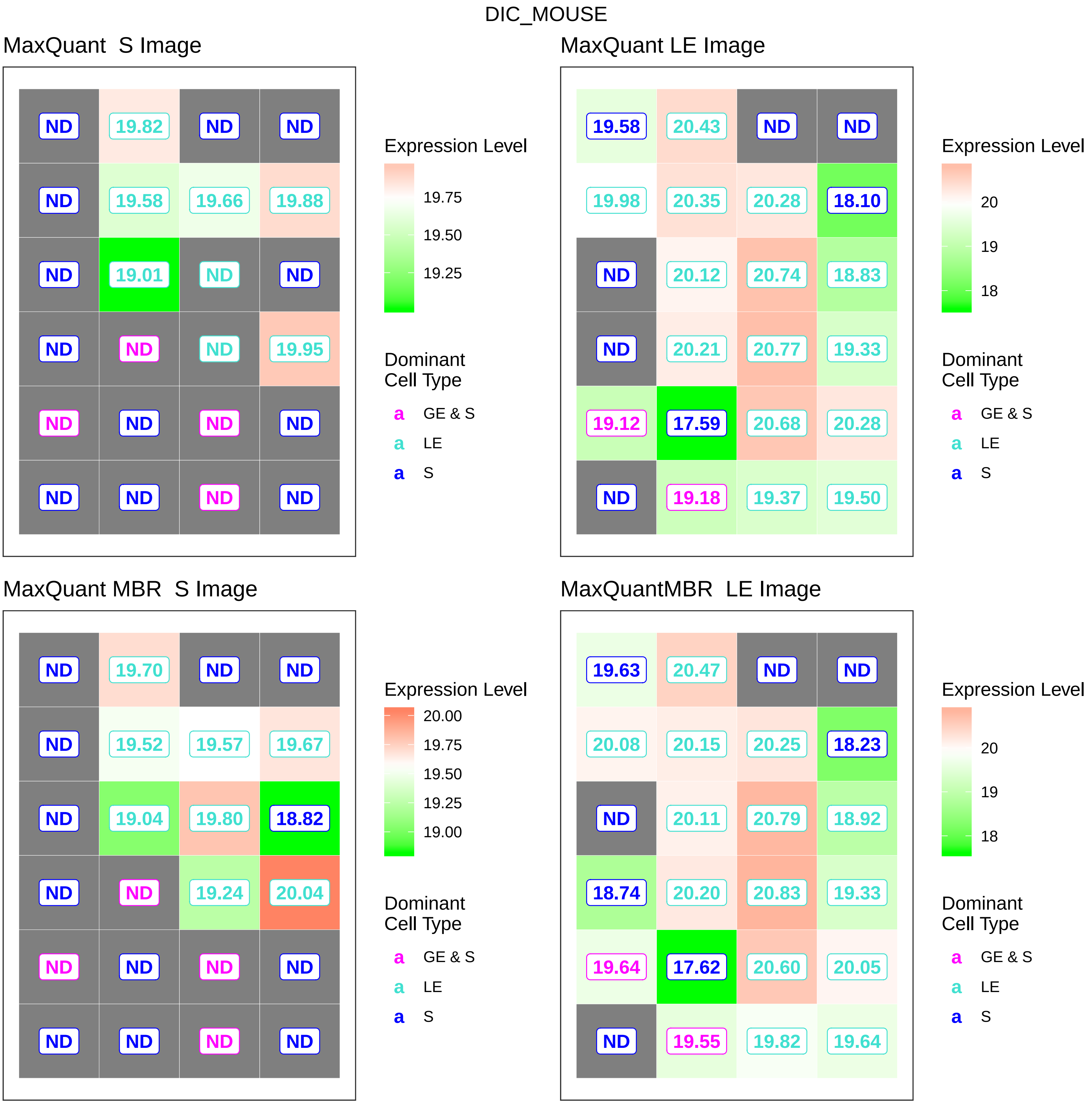
DIC_MOUSE
MaxQuant S Image
ND 19.82 ND ND
ND 19.58 19.66 19.88
ND 19.01 ND ND
ND ND ND 19.95
ND ND ND ND
ND ND ND ND
Expression Level
19.75
19.50
19.25
Dominant Cell Type
a GE & S
a LE
a S
MaxQuant LE Image
19.58 20.43 ND ND
19.98 20.35 20.28 18.10
ND 20.12 20.74 18.83
ND 20.21 20.77 19.33
19.12 17.59 20.68 20.28
ND 19.18 19.37 19.50
Expression Level
20
19
18
Dominant Cell Type
a GE & S
a LE
a S
MaxQuant MBR S Image
ND 19.70 ND ND
ND 19.52 19.57 19.67
ND 19.04 19.80 18.82
ND ND 19.24 20.04
ND ND ND ND
ND ND ND ND
Expression Level
20.00
19.75
19.50
19.25
19.00
Dominant Cell Type
a GE & S
a LE
a S
MaxQuantMBR LE Image
19.63 20.47 ND ND
20.08 20.15 20.25 18.23
ND 20.11 20.79 18.92
18.74 20.20 20.83 19.33
19.64 17.62 20.60 20.05
ND 19.55 19.82 19.64
Expression Level
20
19
18
Dominant Cell Type
a GE & S
a LE
a S

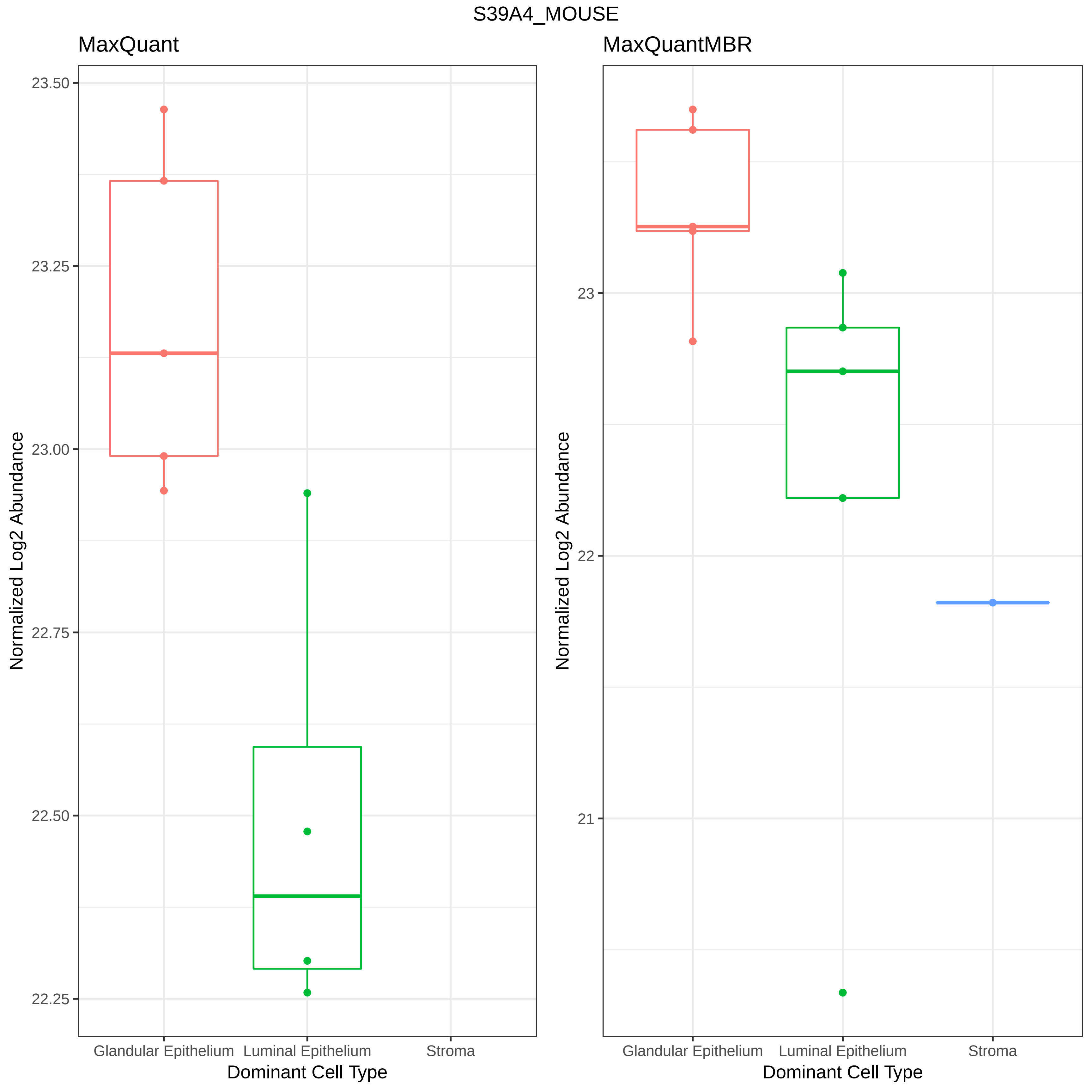

S39A4_MOUSE
MaxQuant
MaxQuantMBR
23.50
23.25
23.00
22.75
22.50
22.25
23
22
21
Normalized Log2 Abundance
Normalized Log2 Abundance
Glandular Epithelium
Luminal Epithelium
Stroma
Glandular Epithelium
Luminal Epithelium
Stroma
Dominant Cell Type
Dominant Cell Type

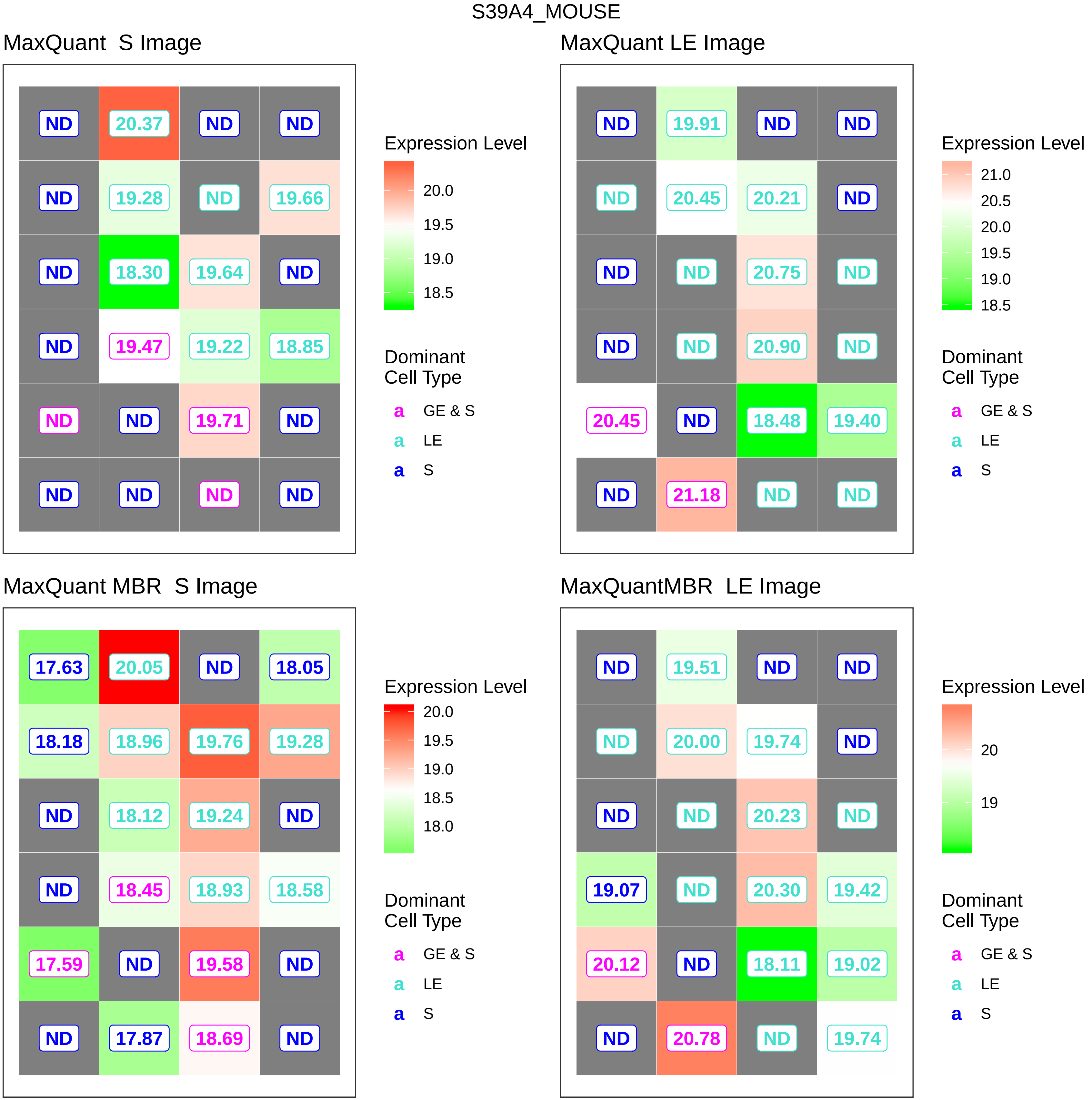
S39A4_MOUSE
MaxQuant S Image
ND 20.37 ND ND
ND 19.28 ND 19.66
ND 18.30 19.64 ND
ND 19.47 19.22 18.85
ND ND 19.71 ND
ND ND ND ND
Expression Level
20.0
19.5
19.0
18.5
Dominant Cell Type
a GE & S
a LE
a S
MaxQuant LE Image
ND 19.91 ND ND
ND 20.45 20.21 ND
ND ND 20.75 ND
ND ND 20.90 ND
20.45 ND 18.48 19.40
ND 21.18 ND ND
Expression Level
21.0
20.5
20.0
19.5
19.0
18.5
Dominant Cell Type
a GE & S
a LE
a S
MaxQuant MBR S Image
17.63 20.05 ND 18.05
18.18 18.96 19.76 19.28
ND 18.12 19.24 ND
ND 18.45 18.93 18.58
17.59 ND 19.58 ND
ND 17.87 18.69 ND
Expression Level
20.0
19.5
19.0
18.5
18.0
Dominant Cell Type
a GE & S
a LE
a S
MaxQuantMBR LE Image
ND 19.51 ND ND
ND 20.00 19.74 ND
ND ND 20.23 ND
19.07 ND 20.30 19.42
20.12 ND 18.11 19.02
ND 20.78 ND 19.74
Expression Level
20
19
Dominant Cell Type
a GE & S
a LE
a S

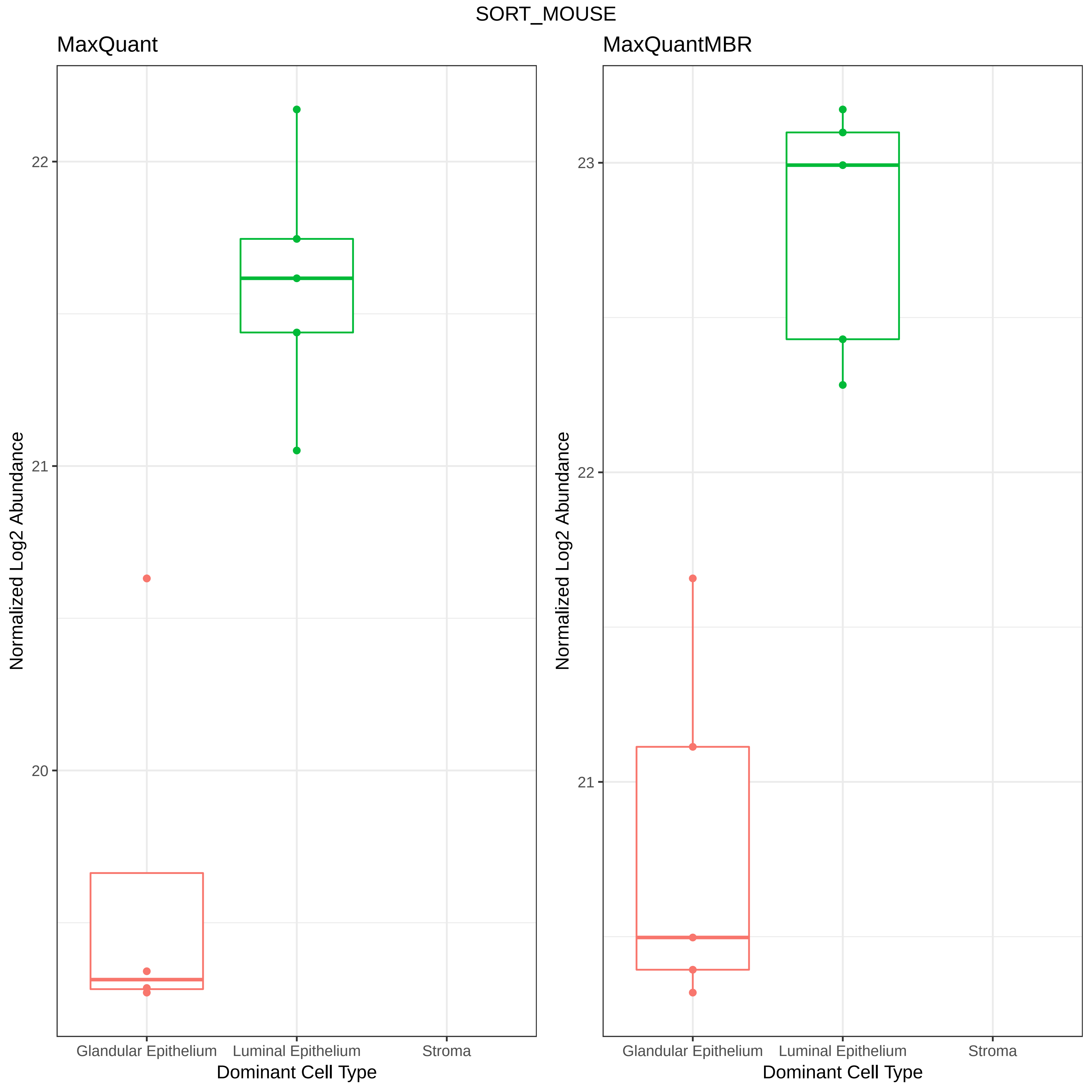
SORT_MOUSE
MaxQuant
MaxQuantMBR
22
21
20
23
22
21
Normalized Log2 Abundance
Normalized Log2 Abundance
Glandular Epithelium
Luminal Epithelium
Stroma
Glandular Epithelium
Luminal Epithelium
Stroma
Dominant Cell Type
Dominant Cell Type

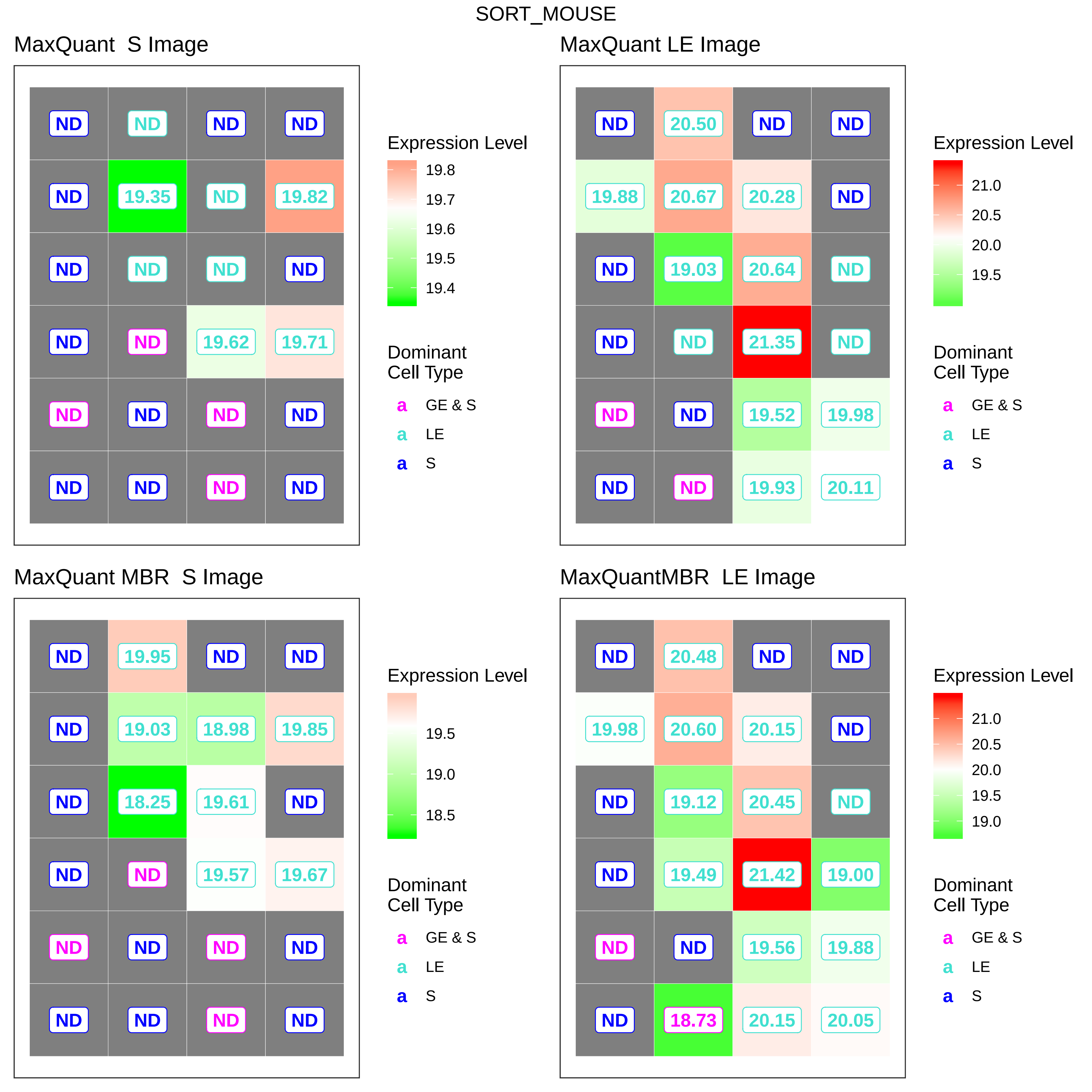
SORT_MOUSE
MaxQuant S Image
MaxQuant LE Image
MaxQuant MBR S Image
MaxQuantMBR LE Image
Expression Level
Dominant Cell Type
GE & S
LE
S
ND
19.35
19.82
19.62
19.71
20.50
19.88
20.67
20.28
19.03
20.64
21.35
19.52
19.98
19.93
20.11
19.95
19.03
18.98
19.85
18.25
19.61
19.57
19.67
20.48
19.98
20.60
20.15
19.12
20.45
19.49
21.42
19.00
19.56
19.88
18.73
20.15
20.05

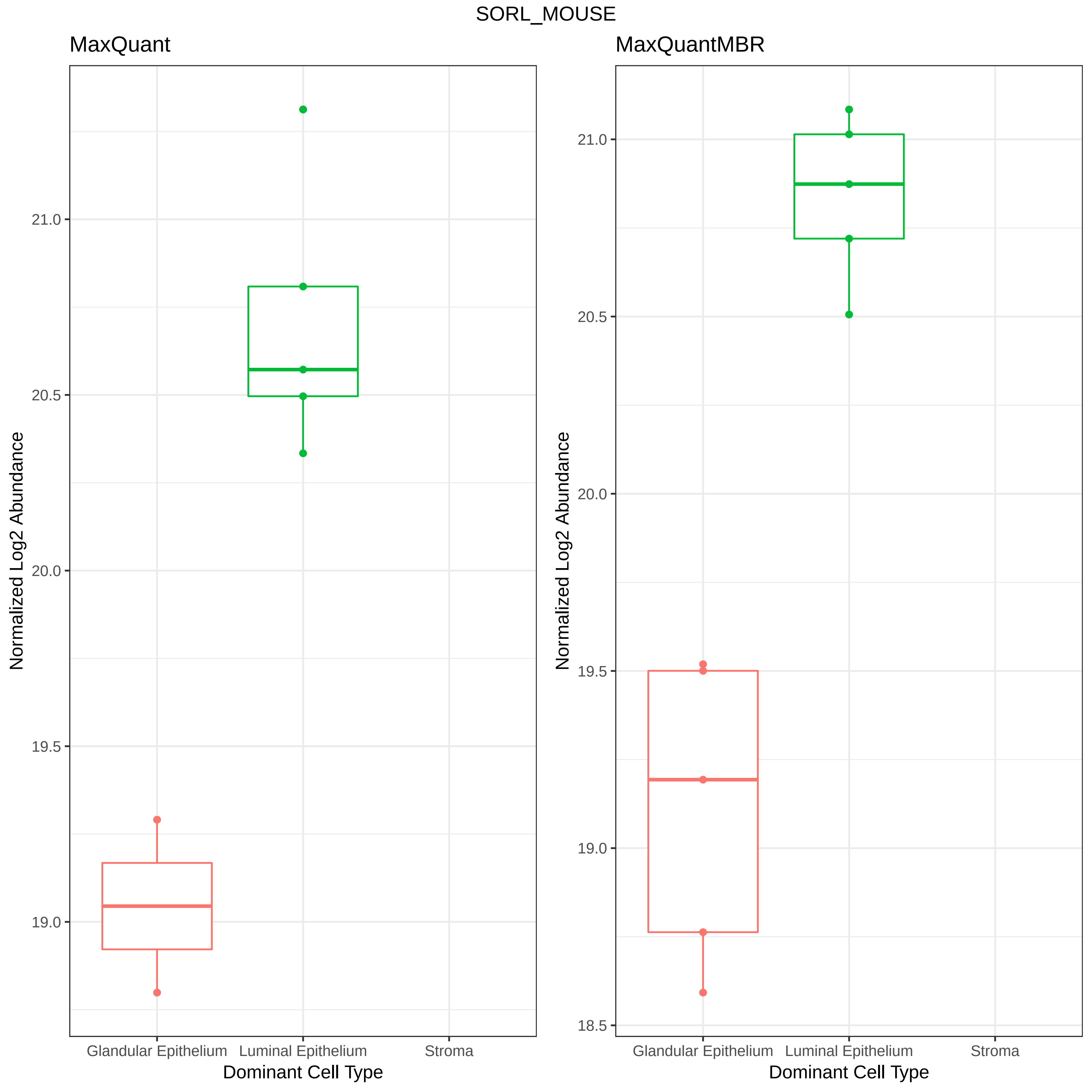

SORL_MOUSE
MaxQuant
MaxQuantMBR
Normalized Log2 Abundance
21.0
20.5
20.0
19.5
19.0
18.5
Glandular Epithelium
Luminal Epithelium
Stroma
Dominant Cell Type

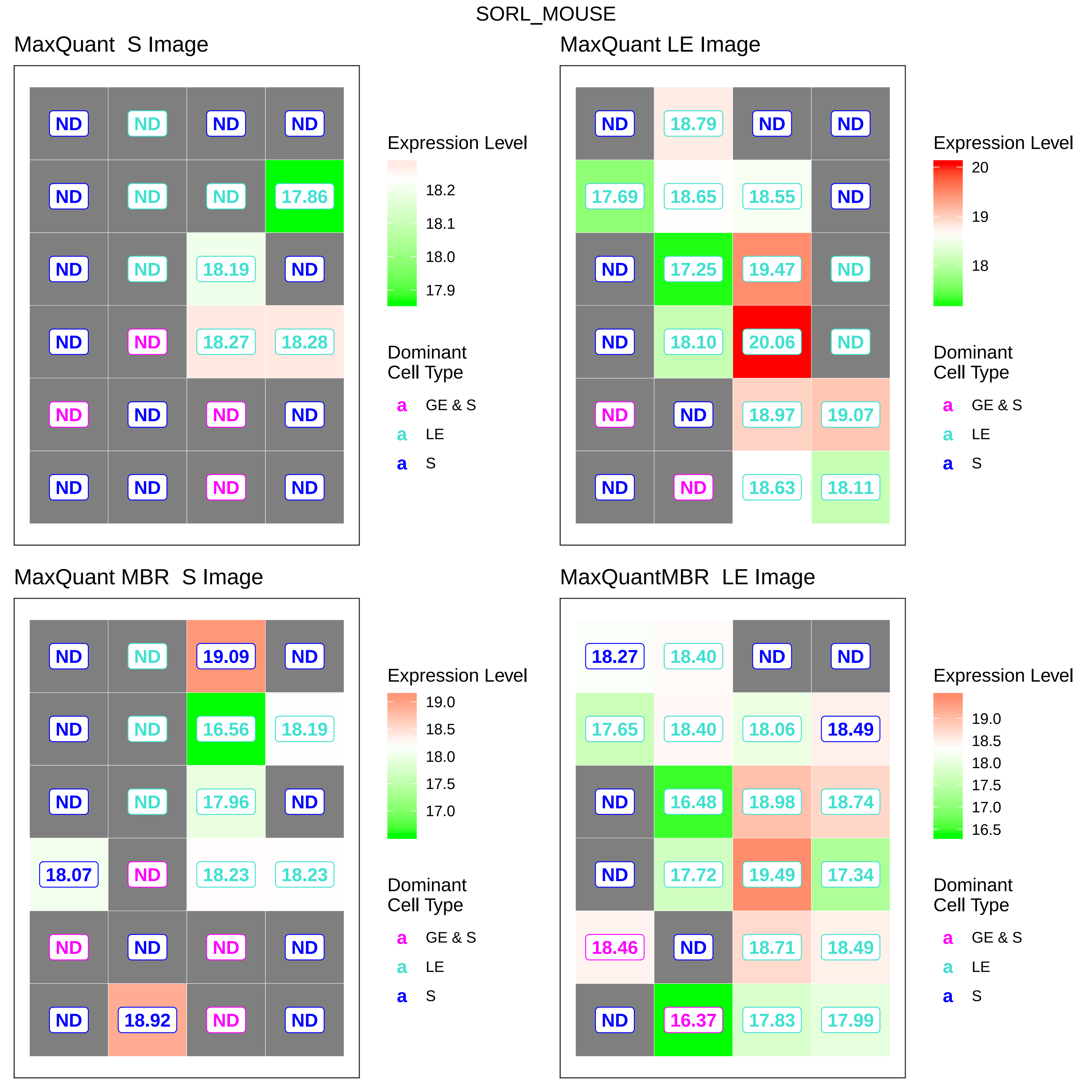
SORL_MOUSE
MaxQuant S Image
ND ND ND ND
ND ND ND 17.86
ND ND 18.19 ND
ND ND 18.27 18.28
ND ND ND ND
ND ND ND ND
Expression Level
18.2
18.1
18.0
17.9
Dominant Cell Type
a GE & S
a LE
a S
MaxQuant LE Image
ND 18.79 ND ND
17.69 18.65 18.55 ND
ND 17.25 19.47 ND
ND 18.10 20.06 ND
ND ND 18.97 19.07
ND ND 18.63 18.11
Expression Level
20
19
18
Dominant Cell Type
a GE & S
a LE
a S
MaxQuant MBR S Image
ND ND 19.09 ND
ND ND 16.56 18.19
ND ND 17.96 ND
18.07 ND 18.23 18.23
ND ND ND ND
ND 18.92 ND ND
Expression Level
19.0
18.5
18.0
17.5
17.0
Dominant Cell Type
a GE & S
a LE
a S
MaxQuantMBR LE Image
18.27 18.40 ND ND
17.65 18.40 18.06 18.49
ND 16.48 18.98 18.74
ND 17.72 19.49 17.34
18.46 ND 18.71 18.49
ND 16.37 17.83 17.99
Expression Level
19.0
18.5
18.0
17.5
17.0
16.5
Dominant Cell Type
a GE & S
a LE
a S

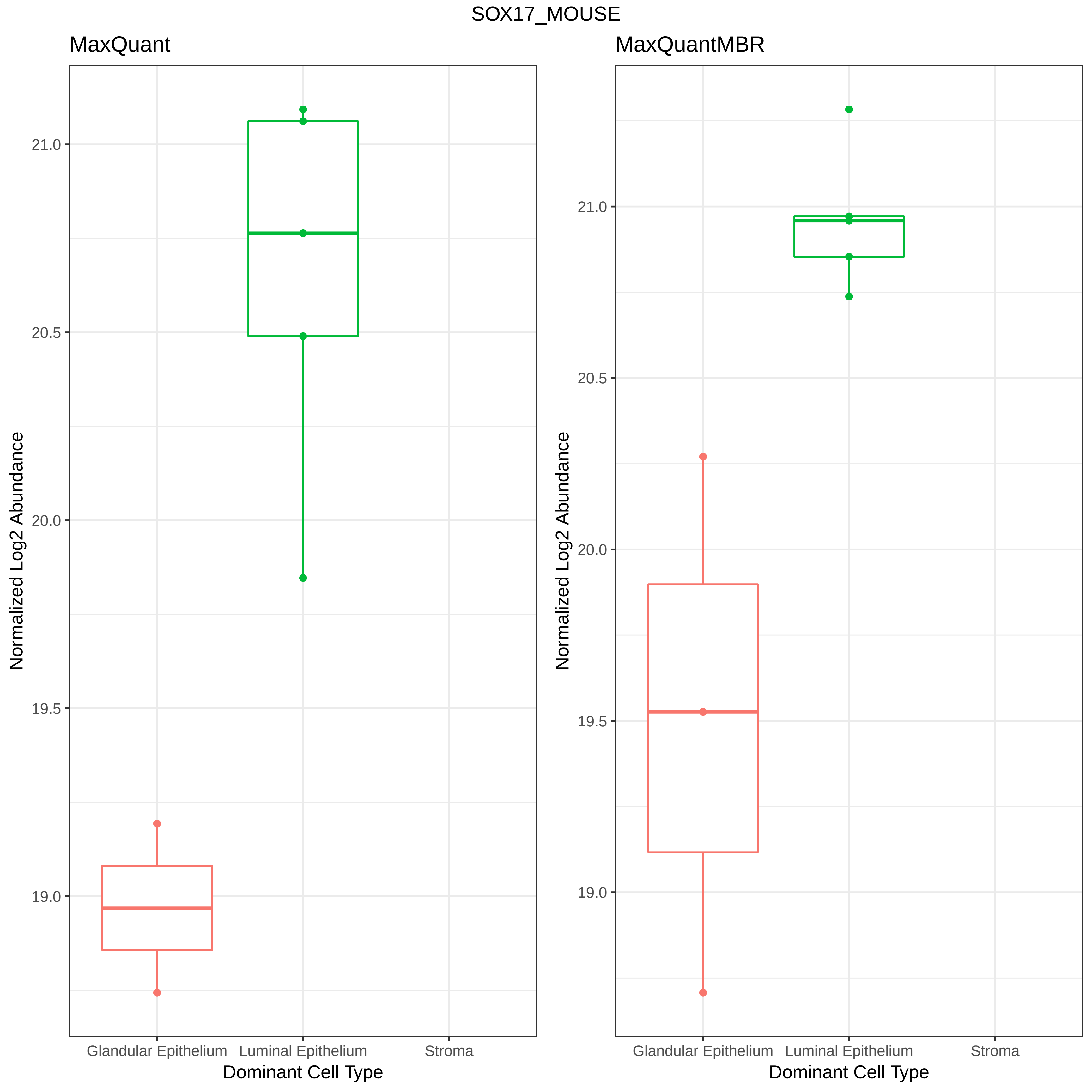

SOX17_MOUSE
MaxQuant
MaxQuantMBR
Normalized Log2 Abundance
21.0
20.5
20.0
19.5
19.0
Glandular Epithelium
Luminal Epithelium
Stroma
Dominant Cell Type

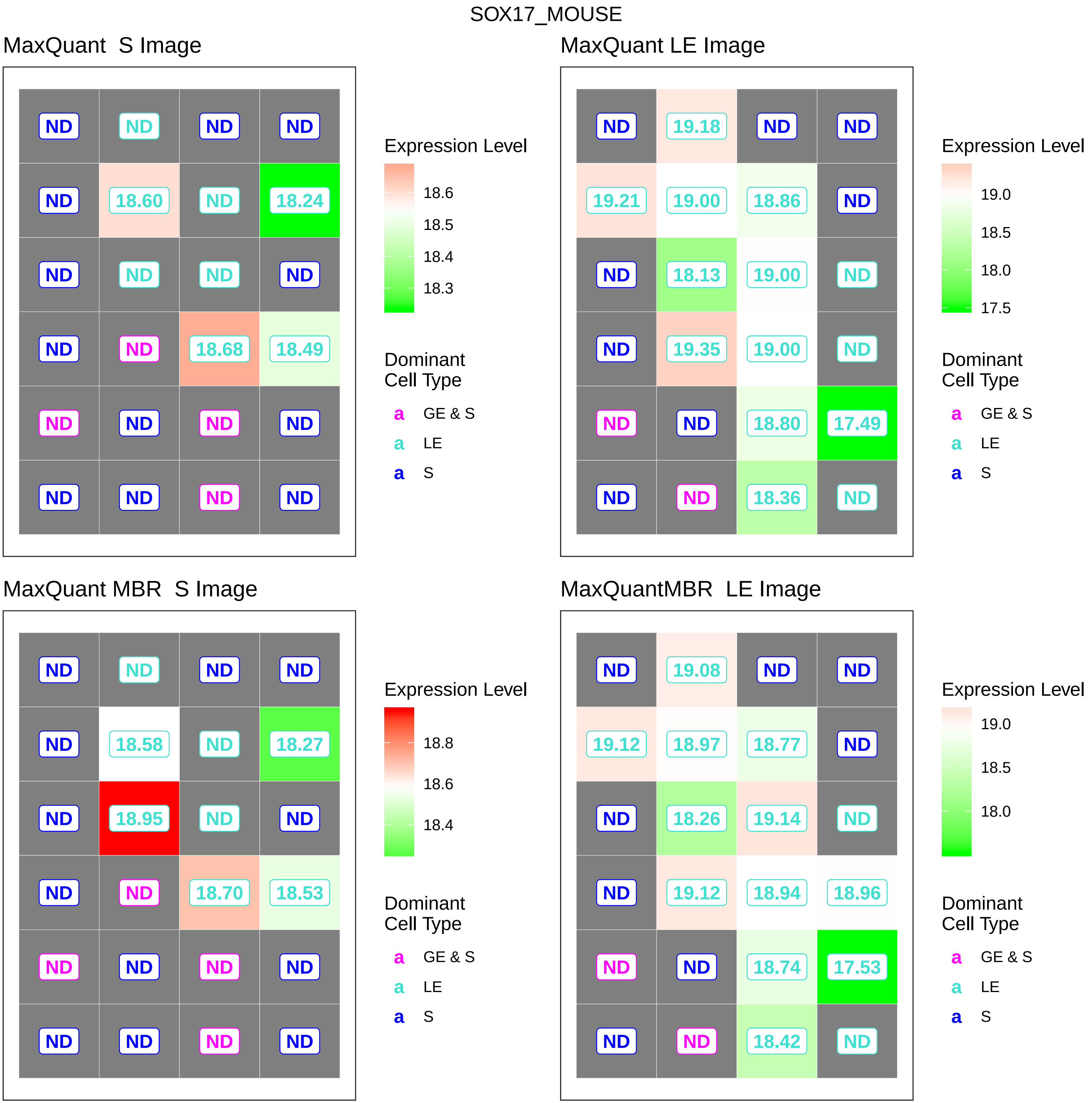
SOX17_MOUSE
MaxQuant S Image
ND ND ND ND
ND 18.60 ND 18.24
ND ND ND ND
ND ND 18.68 18.49
ND ND ND ND
ND ND ND ND
Expression Level
18.6
18.5
18.4
18.3
Dominant Cell Type
a GE & S
a LE
a S
MaxQuant LE Image
ND 19.18 ND ND
19.21 19.00 18.86 ND
ND 18.13 19.00 ND
ND 19.35 19.00 ND
ND ND 18.80 17.49
ND ND 18.36 ND
Expression Level
19.0
18.5
18.0
17.5
Dominant Cell Type
a GE & S
a LE
a S
MaxQuant MBR S Image
ND ND ND ND
ND 18.58 ND 18.27
ND 18.95 ND ND
ND ND 18.70 18.53
ND ND ND ND
ND ND ND ND
Expression Level
18.8
18.6
18.4
Dominant Cell Type
a GE & S
a LE
a S
MaxQuantMBR LE Image
ND 19.08 ND ND
19.12 18.97 18.77 ND
ND 18.26 19.14 ND
ND 19.12 18.94 18.96
ND ND 18.74 17.53
ND ND 18.42 ND
Expression Level
19.0
18.5
18.0
Dominant Cell Type
a GE & S
a LE
a S

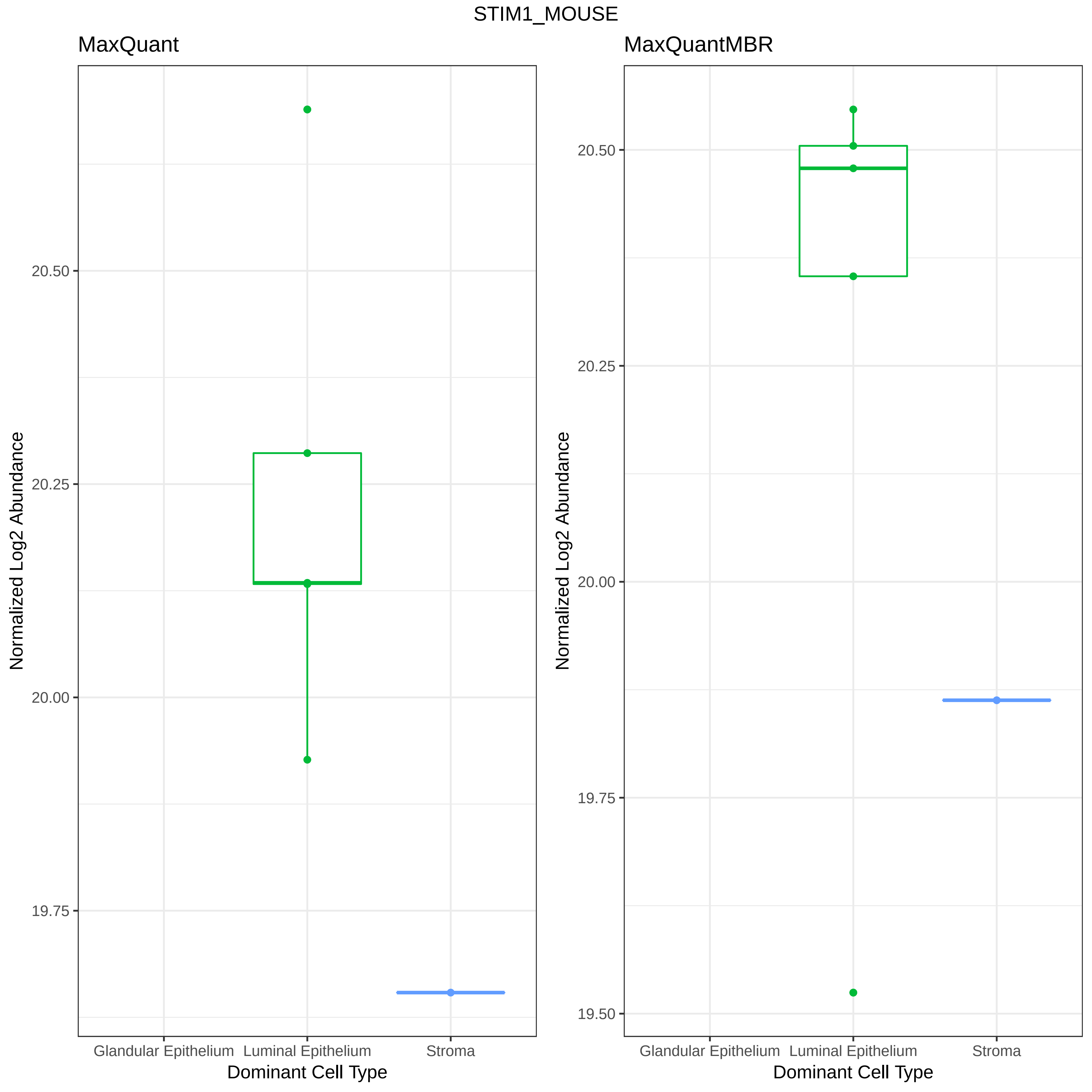
STIM1_MOUSE
MaxQuant
MaxQuantMBR
Normalized Log2 Abundance
20.50
20.25
20.00
19.75
19.50
Glandular Epithelium
Luminal Epithelium
Stroma
Dominant Cell Type

STIM1_MOUSE

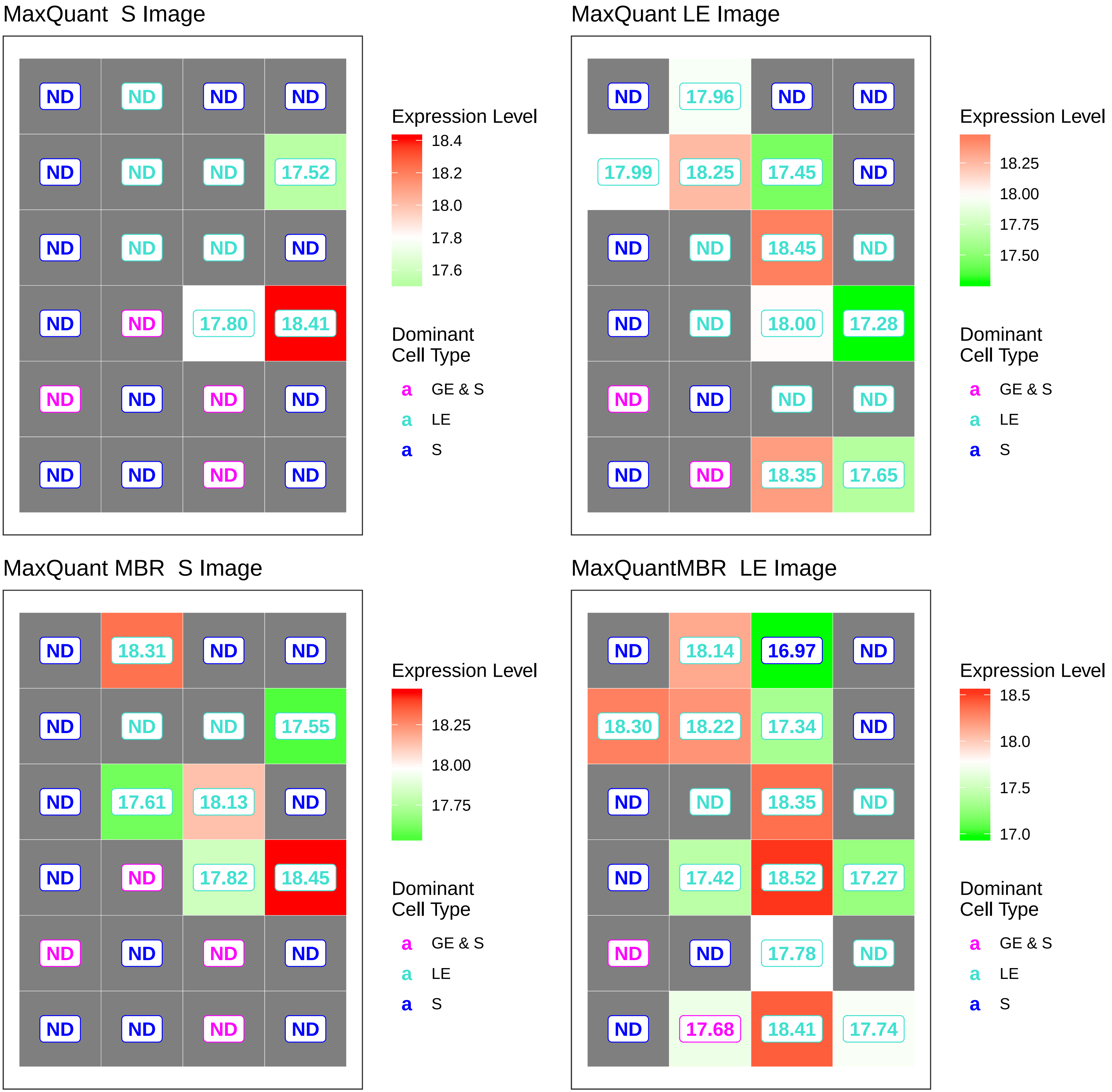
MaxQuant S Image
ND ND ND ND
ND ND ND 17.52
ND ND ND ND
ND ND 17.80 18.41
ND ND ND ND
ND ND ND ND
Expression Level
18.4
18.2
18.0
17.8
17.6
Dominant Cell Type
a GE & S
a LE
a S
MaxQuant LE Image
ND 17.96 ND ND
17.99 18.25 17.45 ND
ND ND 18.45 ND
ND ND 18.00 17.28
ND ND ND ND
ND ND 18.35 17.65
Expression Level
18.25
18.00
17.75
17.50
Dominant Cell Type
a GE & S
a LE
a S
MaxQuant MBR S Image
ND 18.31 ND ND
ND ND ND 17.55
ND 17.61 18.13 ND
ND ND 17.82 18.45
ND ND ND ND
ND ND ND ND
Expression Level
18.25
18.00
17.75
Dominant Cell Type
a GE & S
a LE
a S
MaxQuantMBR LE Image
ND 18.14 16.97 ND
18.30 18.22 17.34 ND
ND ND 18.35 ND
ND 17.42 18.52 17.27
ND ND 17.78 ND
ND 17.68 18.41 17.74
Expression Level
18.5
18.0
17.5
17.0
Dominant Cell Type
a GE & S
a LE
a S

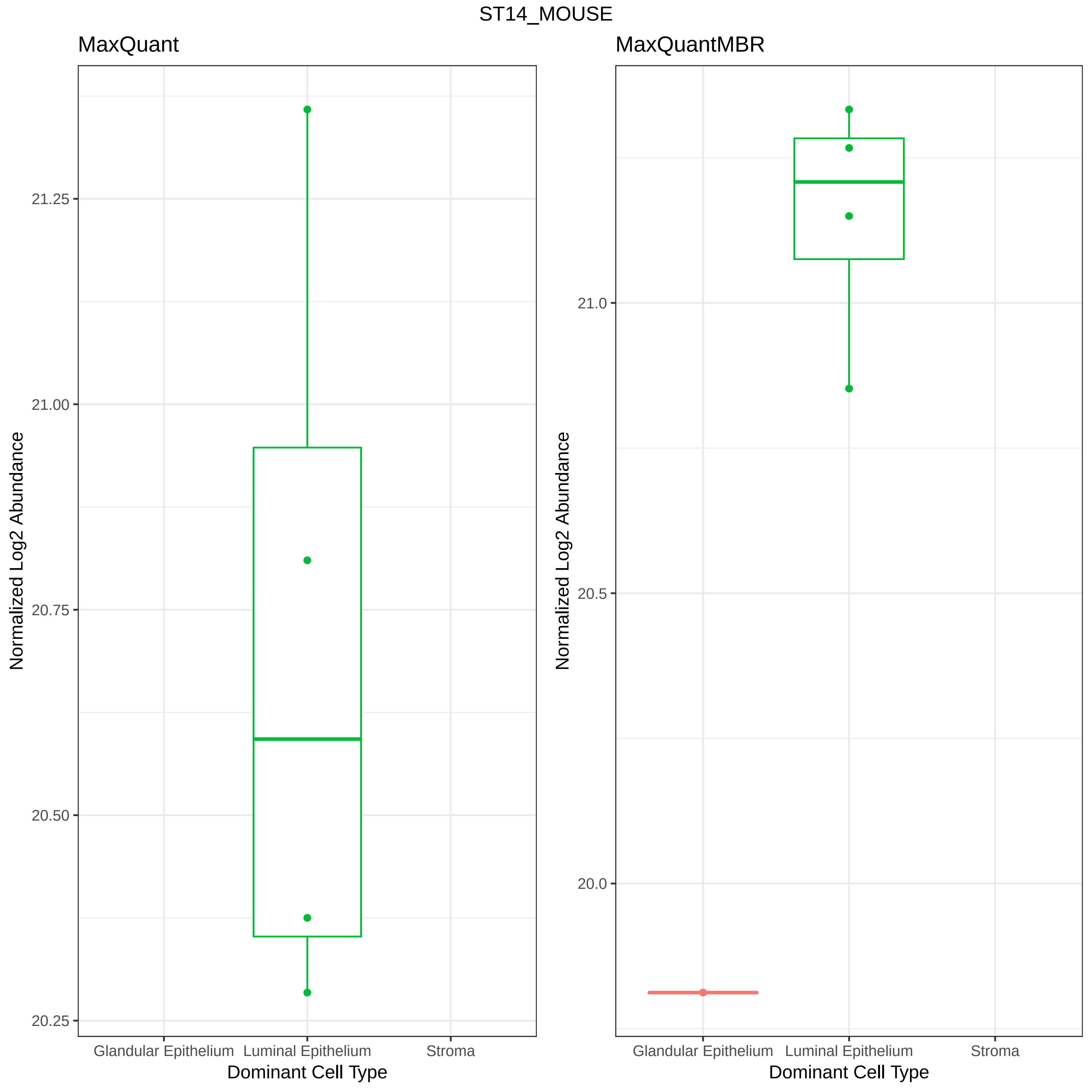

ST14_MOUSE
MaxQuant
MaxQuantMBR
Normalized Log2 Abundance
21.25
21.00
20.75
20.50
20.25
21.0
20.5
20.0
Glandular Epithelium
Luminal Epithelium
Stroma
Dominant Cell Type

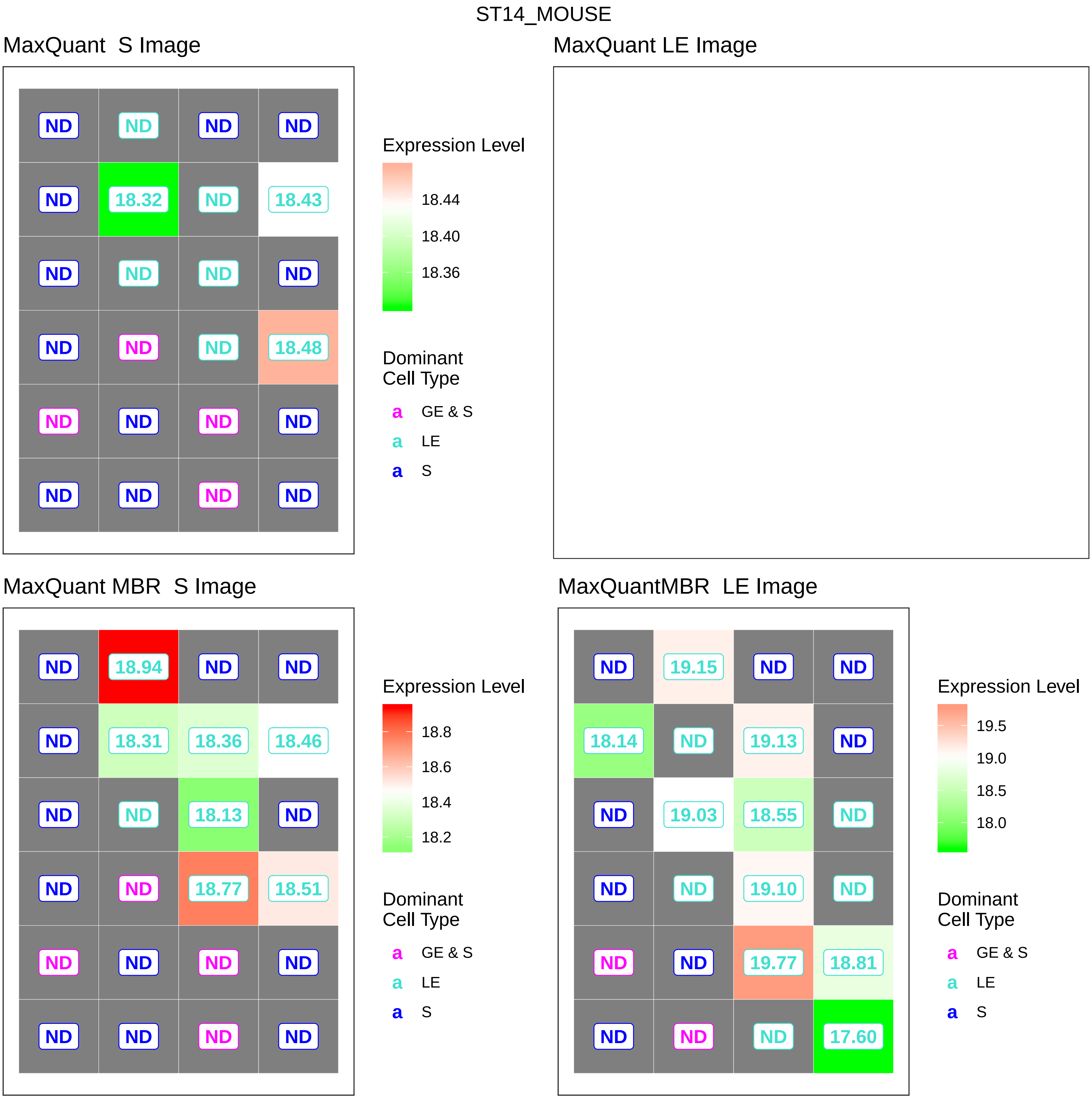
ST14_MOUSE
MaxQuant S Image
ND ND ND ND
ND 18.32 ND 18.43
ND ND ND ND
ND ND ND 18.48
ND ND ND ND
ND ND ND ND
Expression Level
18.44
18.40
18.36
Dominant Cell Type
a GE & S
a LE
a S
MaxQuant LE Image
MaxQuant MBR S Image
ND 18.94 ND ND
ND 18.31 18.36 18.46
ND ND 18.13 ND
ND ND 18.77 18.51
ND ND ND ND
ND ND ND ND
Expression Level
18.8
18.6
18.4
18.2
Dominant Cell Type
a GE & S
a LE
a S
MaxQuantMBR LE Image
ND 19.15 ND ND
18.14 ND 19.13 ND
ND 19.03 18.55 ND
ND ND 19.10 ND
ND ND 19.77 18.81
ND ND ND 17.60
Expression Level
19.5
19.0
18.5
18.0
Dominant Cell Type
a GE & S
a LE
a S

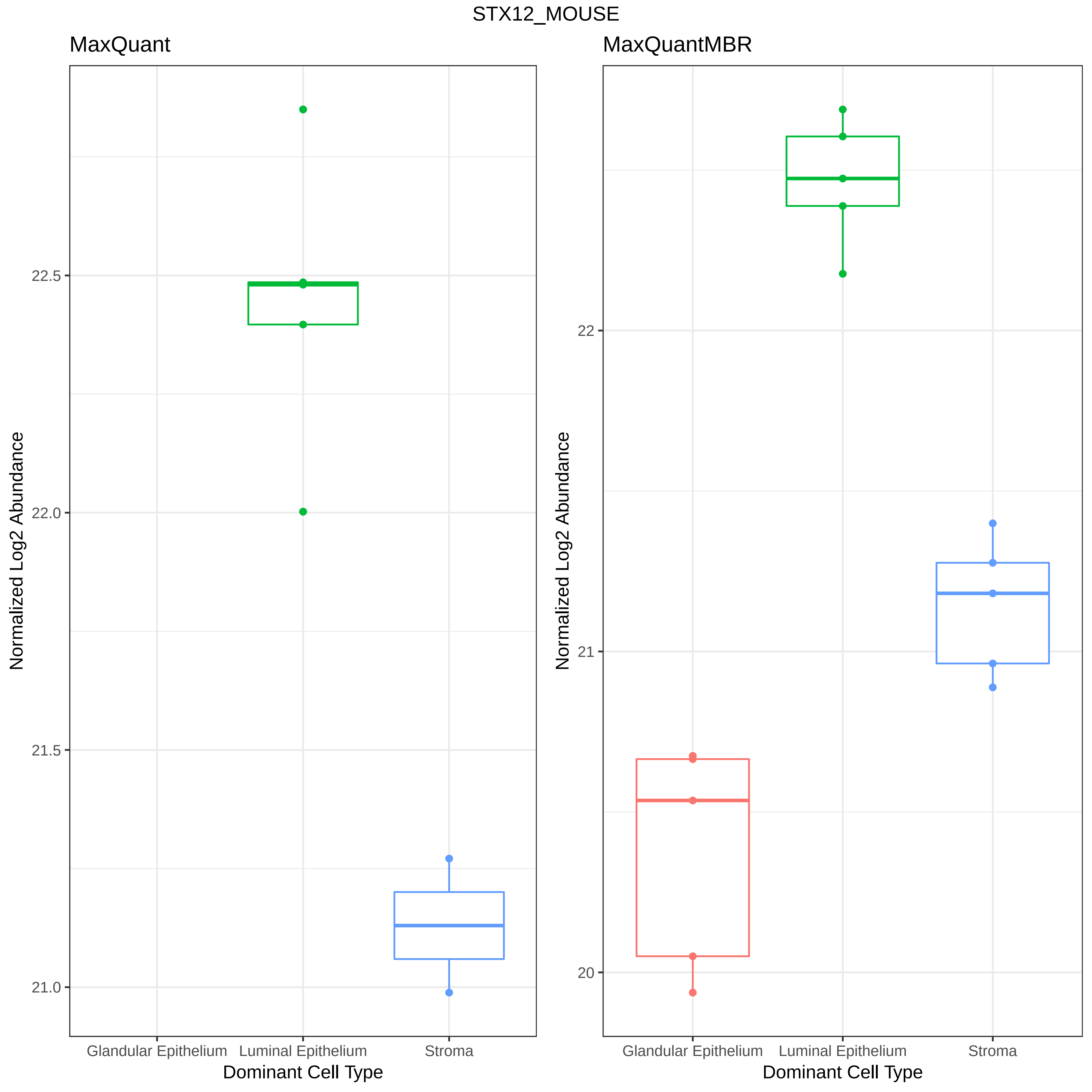

STX12_MOUSE
MaxQuant
MaxQuantMBR
Normalized Log2 Abundance
22.5
22.0
21.5
21.0
22
21
20
Glandular Epithelium
Luminal Epithelium
Stroma
Dominant Cell Type

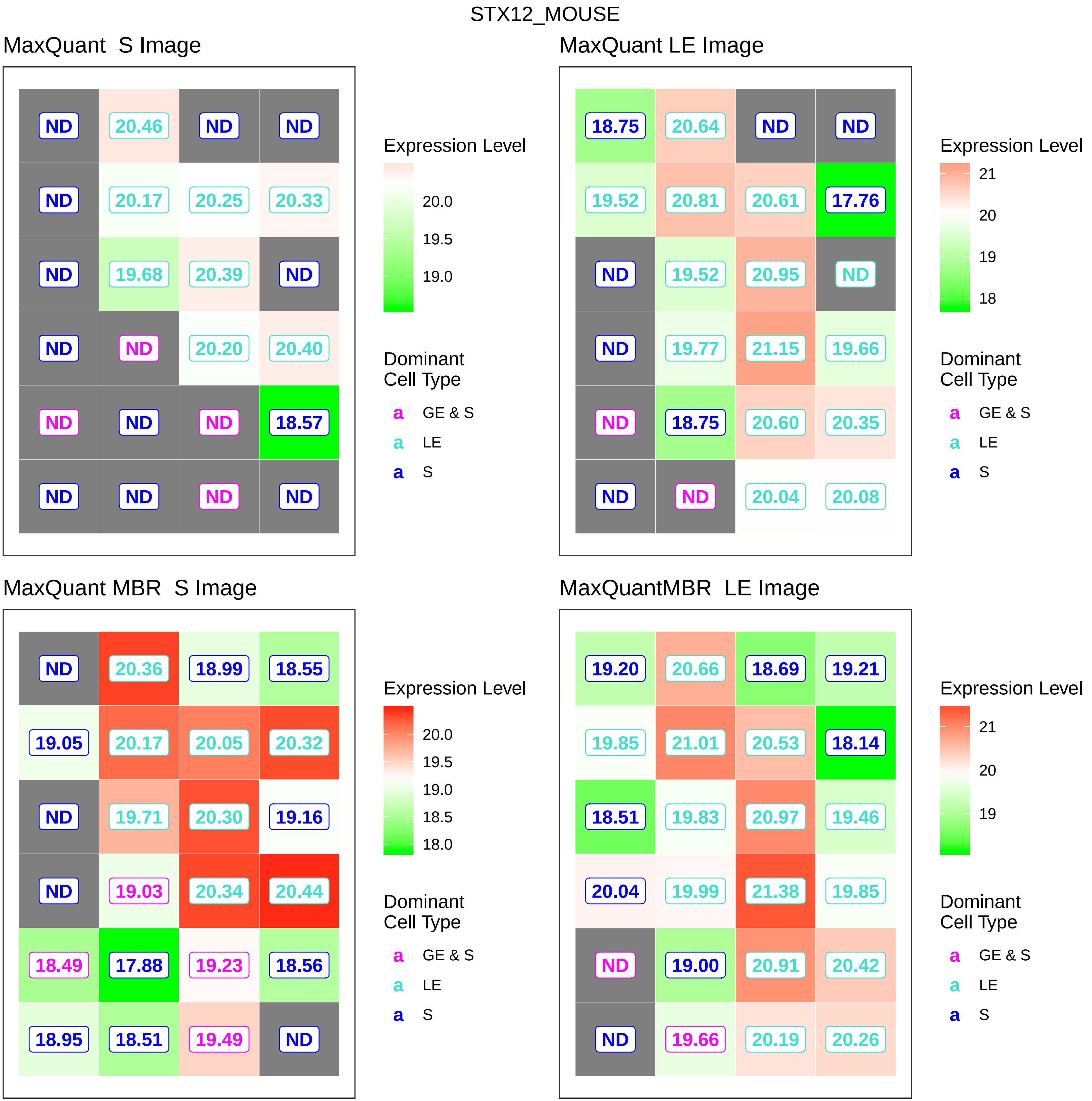
STX12_MOUSE
MaxQuant S Image
ND 20.46 ND ND
ND 20.17 20.25 20.33
ND 19.68 20.39 ND
ND ND 20.20 20.40
ND ND ND 18.57
ND ND ND ND
Expression Level
20.0
19.5
19.0
Dominant Cell Type
a GE & S
a LE
a S
MaxQuant LE Image
18.75 20.64 ND ND
19.52 20.81 20.61 17.76
ND 19.52 20.95 ND
ND 19.77 21.15 19.66
ND 18.75 20.60 20.35
ND ND 20.04 20.08
Expression Level
21
20
19
18
Dominant Cell Type
a GE & S
a LE
a S
MaxQuant MBR S Image
ND 20.36 18.99 18.55
19.05 20.17 20.05 20.32
ND 19.71 20.30 19.16
ND 19.03 20.34 20.44
18.49 17.88 19.23 18.56
18.95 18.51 19.49 ND
Expression Level
20.0
19.5
19.0
18.5
18.0
Dominant Cell Type
a GE & S
a LE
a S
MaxQuantMBR LE Image
19.20 20.66 18.69 19.21
19.85 21.01 20.53 18.14
18.51 19.83 20.97 19.46
20.04 19.99 21.38 19.85
ND 19.00 20.91 20.42
ND 19.66 20.19 20.26
Expression Level
21
20
19
Dominant Cell Type
a GE & S
a LE
a S

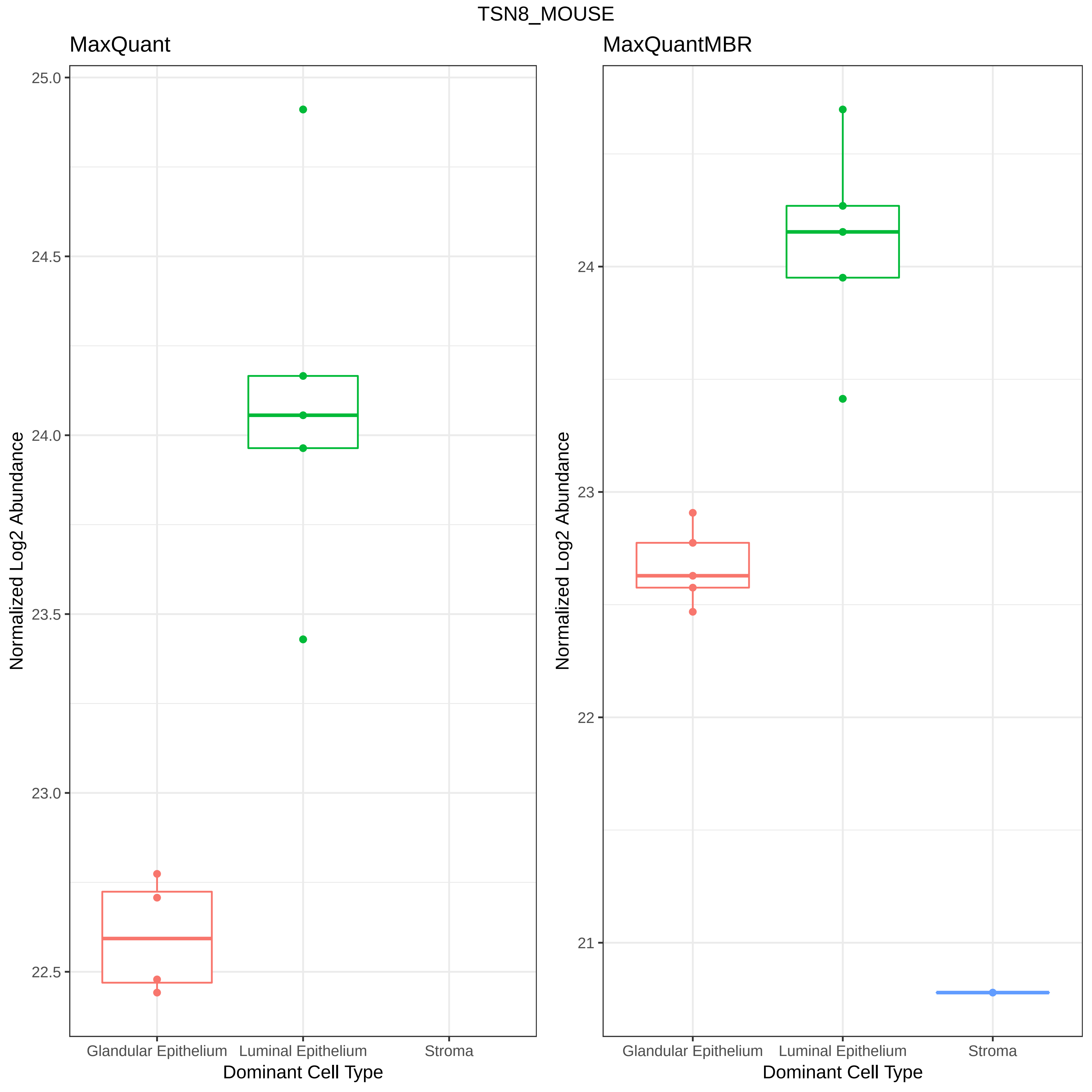
TSN8_MOUSE
MaxQuant
MaxQuantMBR
Normalized Log2 Abundance
25.0
24.5
24.0
23.5
23.0
22.5
24
23
22
21
Glandular Epithelium
Luminal Epithelium
Stroma
Dominant Cell Type

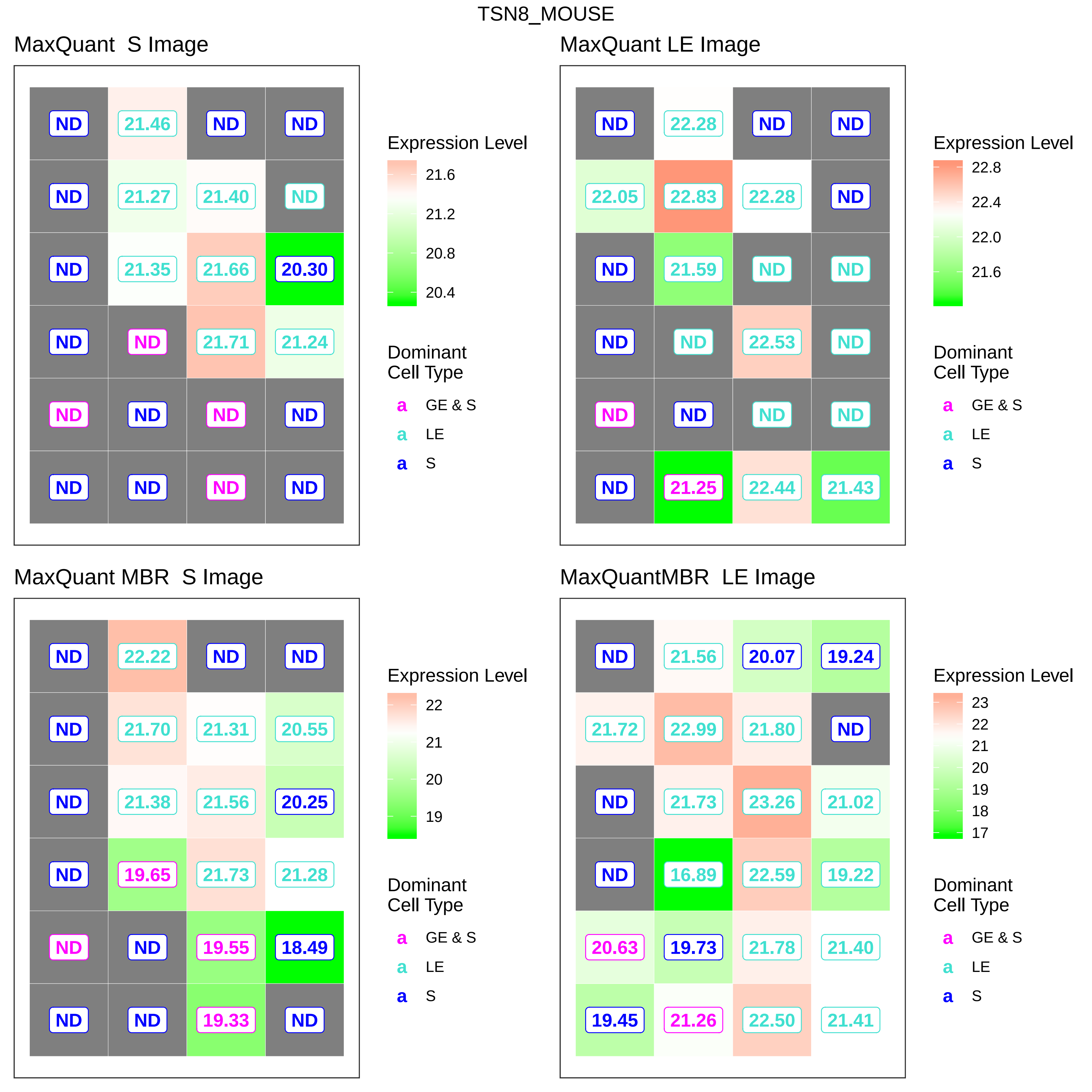

TSN8_MOUSE
MaxQuant S Image
ND 21.46 ND ND
ND 21.27 21.40 ND
ND 21.35 21.66 20.30
ND ND 21.71 21.24
ND ND ND ND
ND ND ND ND
Expression Level
21.6
21.2
20.8
20.4
Dominant Cell Type
a GE & S
a LE
a S
MaxQuant LE Image
ND 22.28 ND ND
22.05 22.83 22.28 ND
ND 21.59 ND ND
ND ND 22.53 ND
ND ND ND ND
ND 21.25 22.44 21.43
Expression Level
22.8
22.4
22.0
21.6
Dominant Cell Type
a GE & S
a LE
a S
MaxQuant MBR S Image
ND 22.22 ND ND
ND 21.70 21.31 20.55
ND 21.38 21.56 20.25
ND 19.65 21.73 21.28
ND ND 19.55 18.49
ND ND 19.33 ND
Expression Level
22
21
20
19
Dominant Cell Type
a GE & S
a LE
a S
MaxQuantMBR LE Image
ND 21.56 20.07 19.24
21.72 22.99 21.80 ND
ND 21.73 23.26 21.02
ND 16.89 22.59 19.22
20.63 19.73 21.78 21.40
19.45 21.26 22.50 21.41
Expression Level
23
22
21
20
19
18
17
Dominant Cell Type
a GE & S
a LE
a S

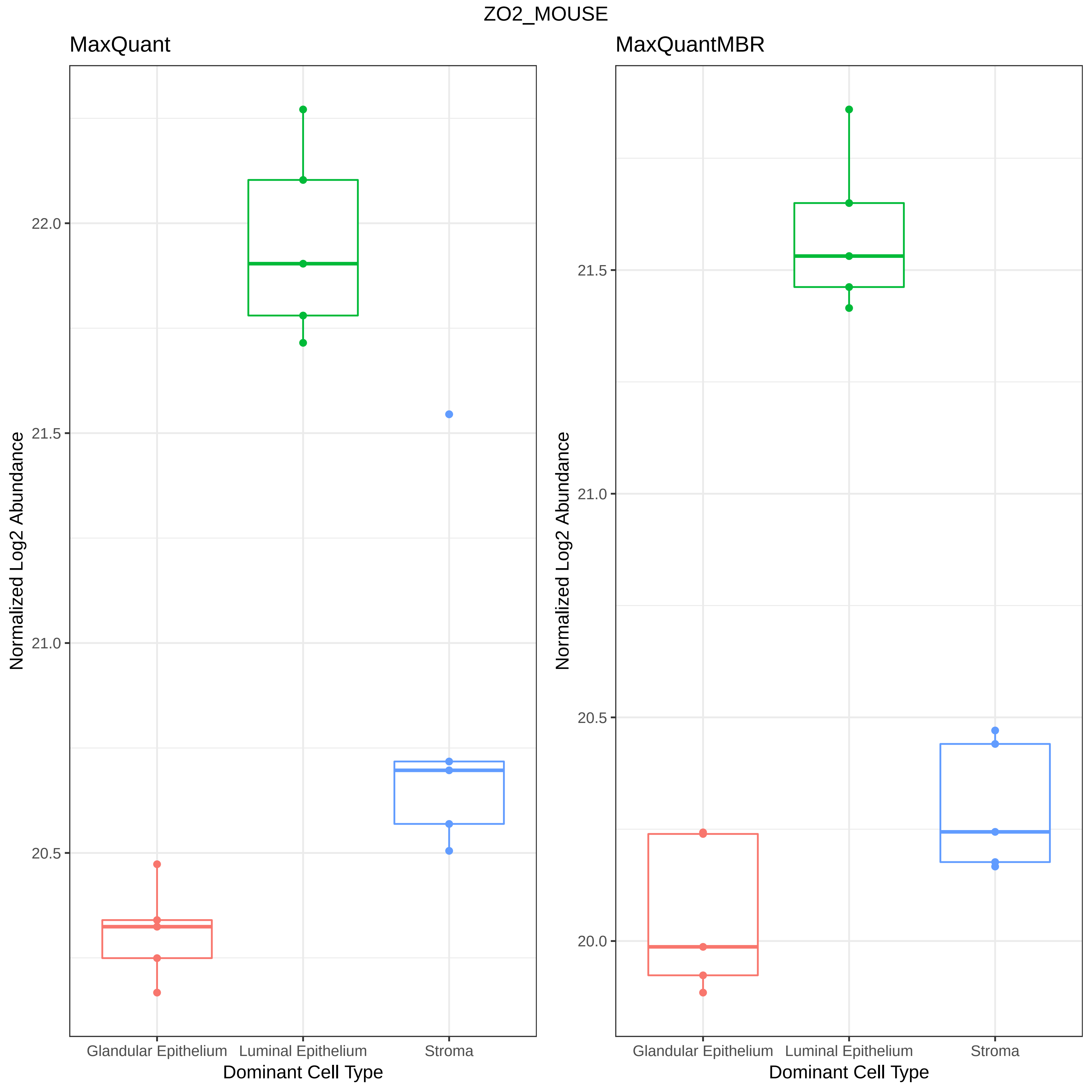
ZO2_MOUSE
MaxQuant
MaxQuantMBR
Normalized Log2 Abundance
22.0
21.5
21.0
20.5
20.0
Glandular Epithelium
Luminal Epithelium
Stroma
Dominant Cell Type

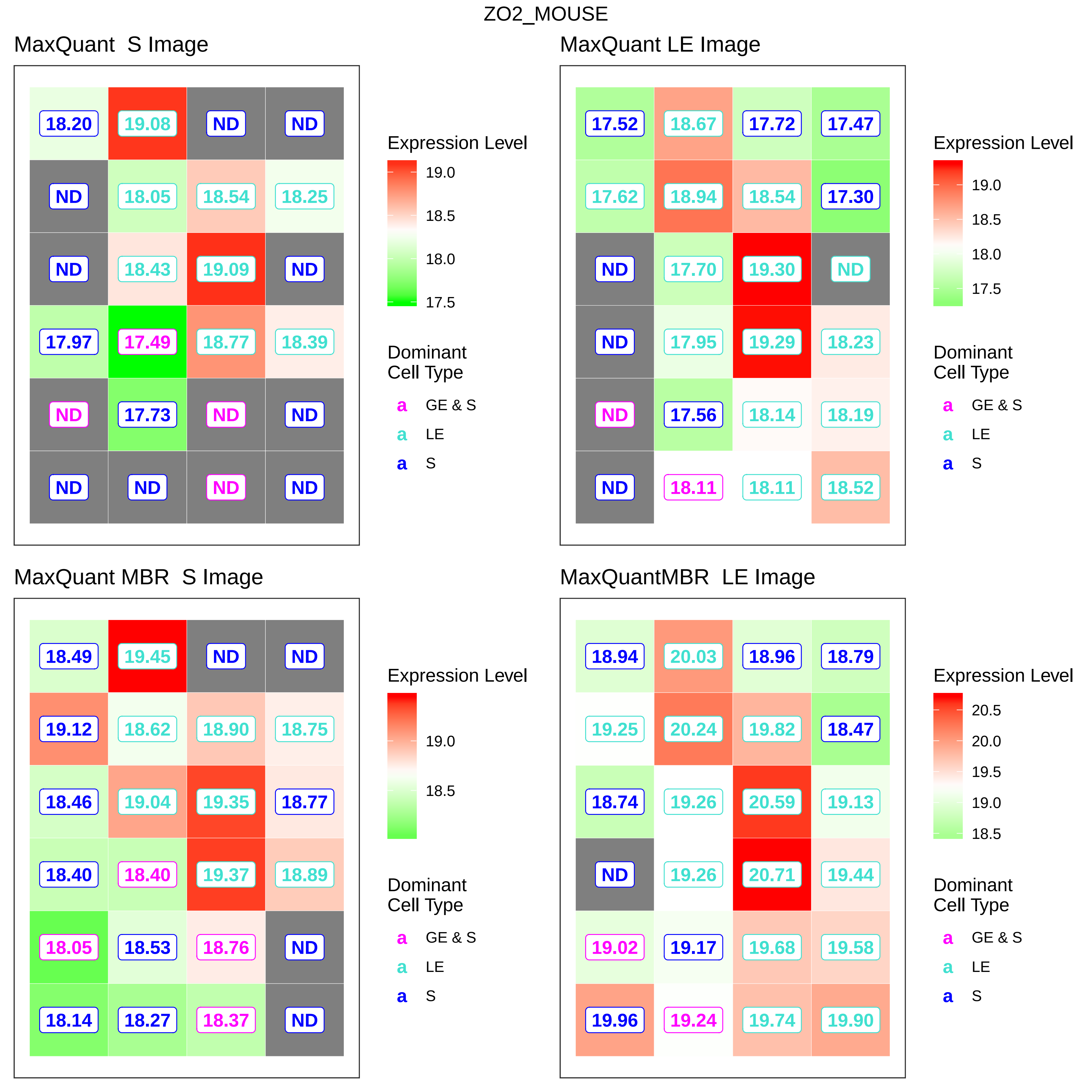
ZO2_MOUSE
MaxQuant S Image
18.20 19.08 ND ND
ND 18.05 18.54 18.25
ND 18.43 19.09 ND
17.97 17.49 18.77 18.39
ND 17.73 ND ND
ND ND ND ND
Expression Level
19.0
18.5
18.0
17.5
Dominant Cell Type
a GE & S
a LE
a S
MaxQuant LE Image
17.52 18.67 17.72 17.47
17.62 18.94 18.54 17.30
ND 17.70 19.30 ND
ND 17.95 19.29 18.23
ND 17.56 18.14 18.19
ND 18.11 18.11 18.52
Expression Level
19.0
18.5
18.0
17.5
Dominant Cell Type
a GE & S
a LE
a S
MaxQuant MBR S Image
18.49 19.45 ND ND
19.12 18.62 18.90 18.75
18.46 19.04 19.35 18.77
18.40 18.40 19.37 18.89
18.05 18.53 18.76 ND
18.14 18.27 18.37 ND
Expression Level
19.0
18.5
Dominant Cell Type
a GE & S
a LE
a S
MaxQuantMBR LE Image
18.94 20.03 18.96 18.79
19.25 20.24 19.82 18.47
18.74 19.26 20.59 19.13
ND 19.26 20.71 19.44
19.02 19.17 19.68 19.58
19.96 19.24 19.74 19.90
Expression Level
20.5
20.0
19.5
19.0
18.5
Dominant Cell Type
a GE & S
a LE
a S

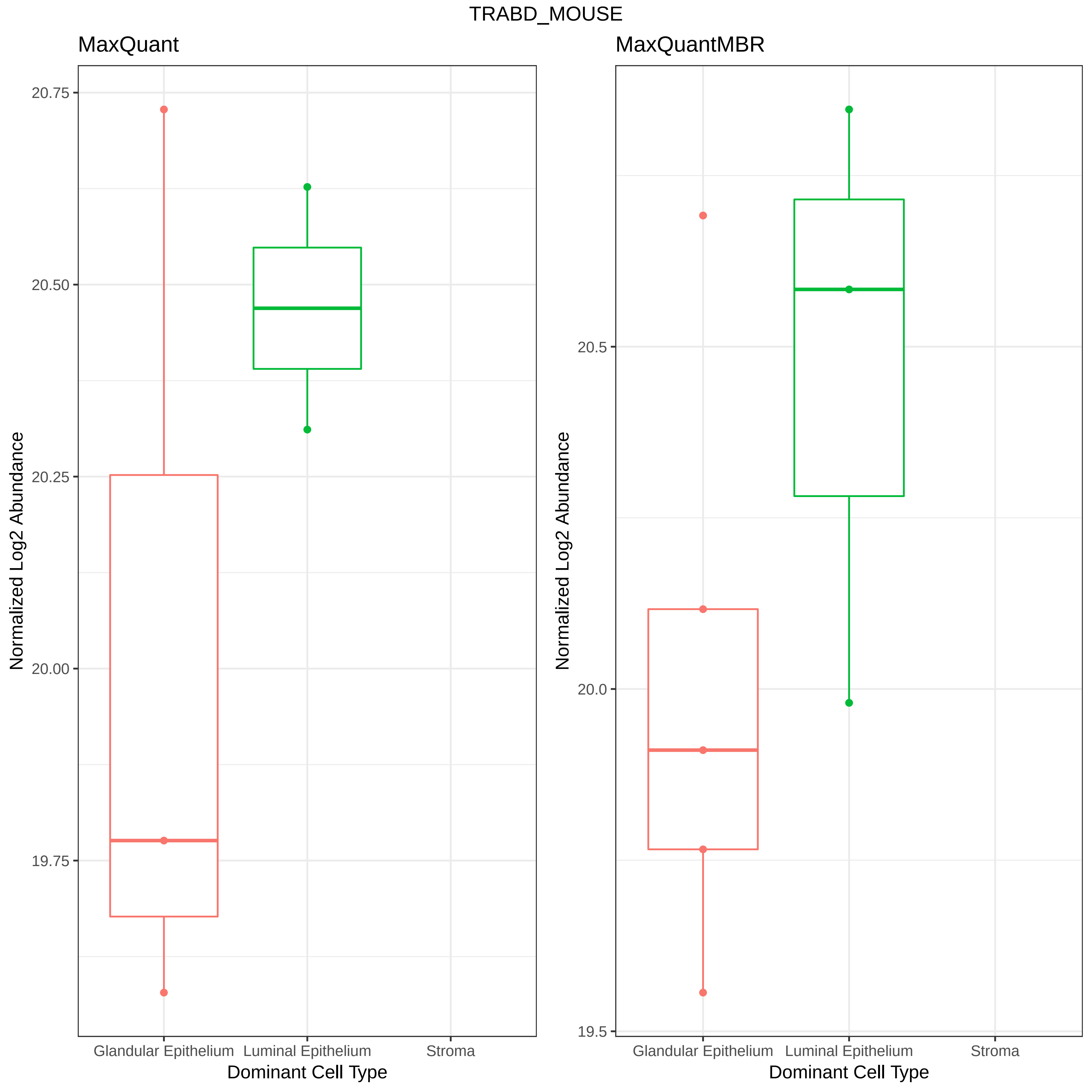

TRABD_MOUSE
MaxQuant
MaxQuantMBR
Normalized Log2 Abundance
20.75
20.50
20.25
20.00
19.75
20.5
20.0
19.5
Glandular Epithelium
Luminal Epithelium
Stroma
Dominant Cell Type

TRABD_MOUSE

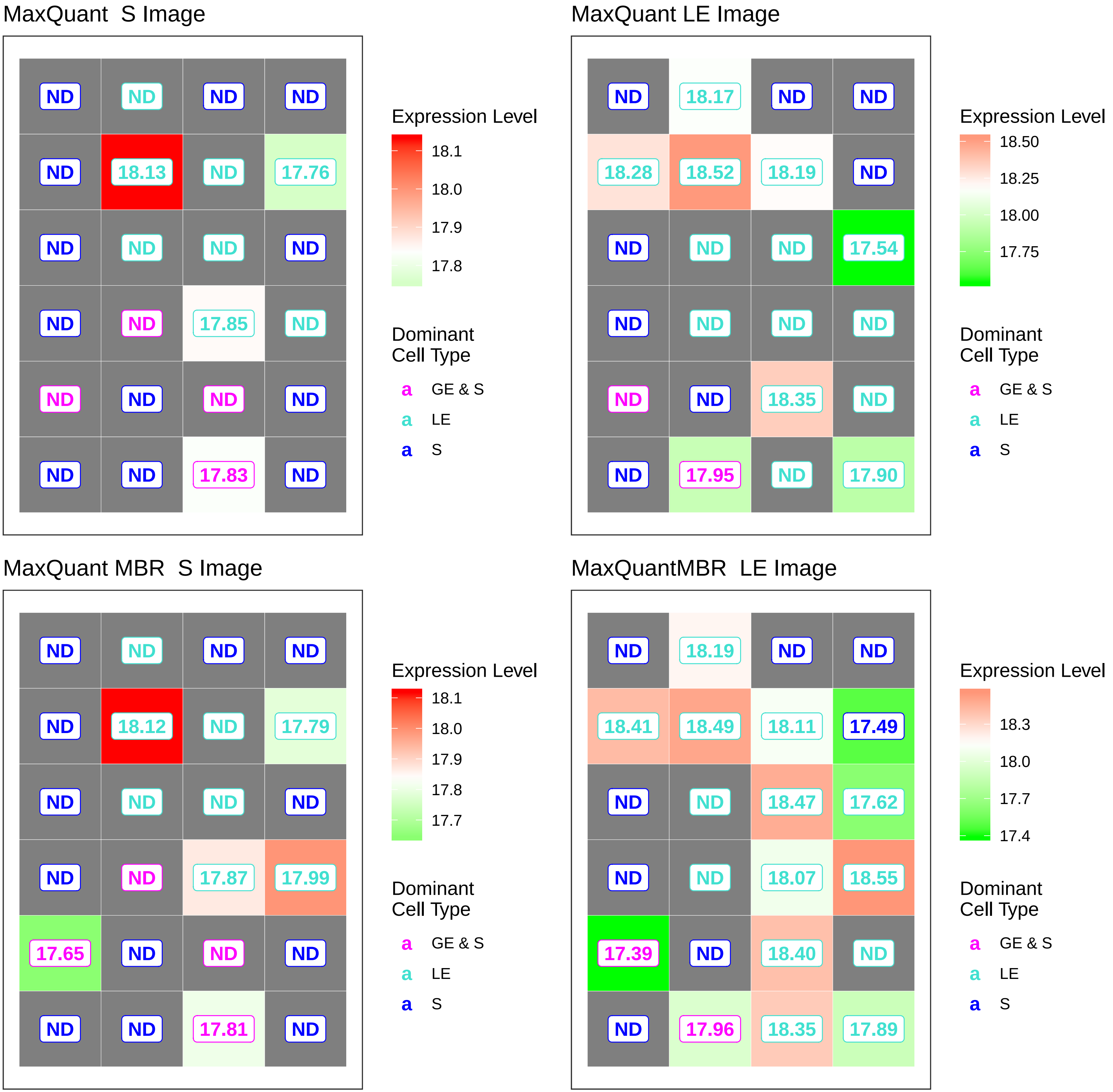
MaxQuant S Image
ND ND ND ND
ND 18.13 ND 17.76
ND ND ND ND
ND ND 17.85 ND
ND ND ND ND
ND ND 17.83 ND
Expression Level
18.1
18.0
17.9
17.8
Dominant Cell Type
a GE & S
a LE
a S
MaxQuant LE Image
ND 18.17 ND ND
18.28 18.52 18.19 ND
ND ND ND 17.54
ND ND ND ND
ND ND 18.35 ND
ND 17.95 ND 17.90
Expression Level
18.50
18.25
18.00
17.75
Dominant Cell Type
a GE & S
a LE
a S
MaxQuant MBR S Image
ND ND ND ND
ND 18.12 ND 17.79
ND ND ND ND
ND ND 17.87 17.99
17.65 ND ND ND
ND ND 17.81 ND
Expression Level
18.1
18.0
17.9
17.8
17.7
Dominant Cell Type
a GE & S
a LE
a S
MaxQuantMBR LE Image
ND 18.19 ND ND
18.41 18.49 18.11 17.49
ND ND 18.47 17.62
ND ND 18.07 18.55
17.39 ND 18.40 ND
ND 17.96 18.35 17.89
Expression Level
18.3
18.0
17.7
17.4
Dominant Cell Type
a GE & S
a LE
a S

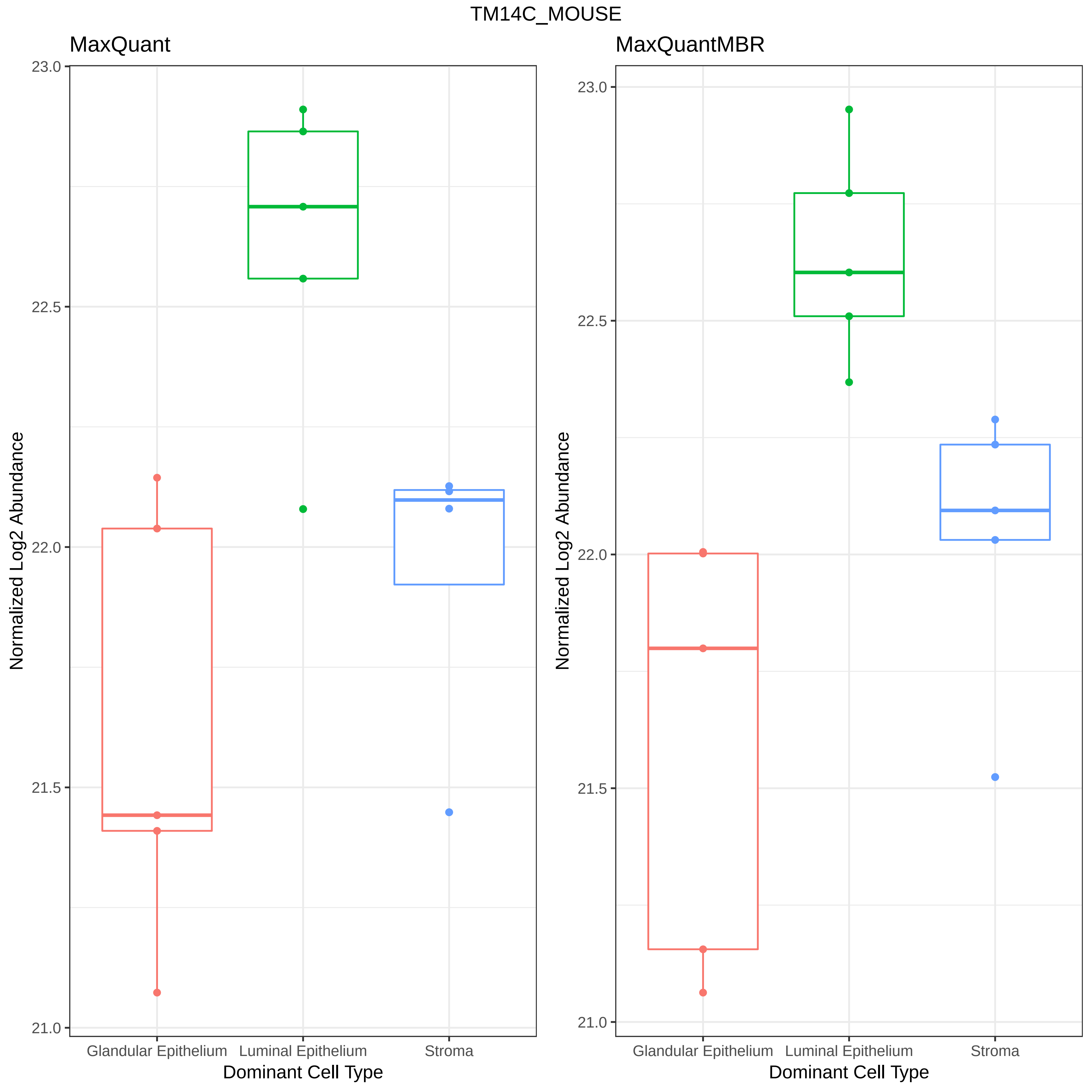

TM14C_MOUSE
MaxQuant
MaxQuantMBR
Normalized Log2 Abundance
23.0
22.5
22.0
21.5
21.0
Glandular Epithelium
Luminal Epithelium
Stroma
Dominant Cell Type

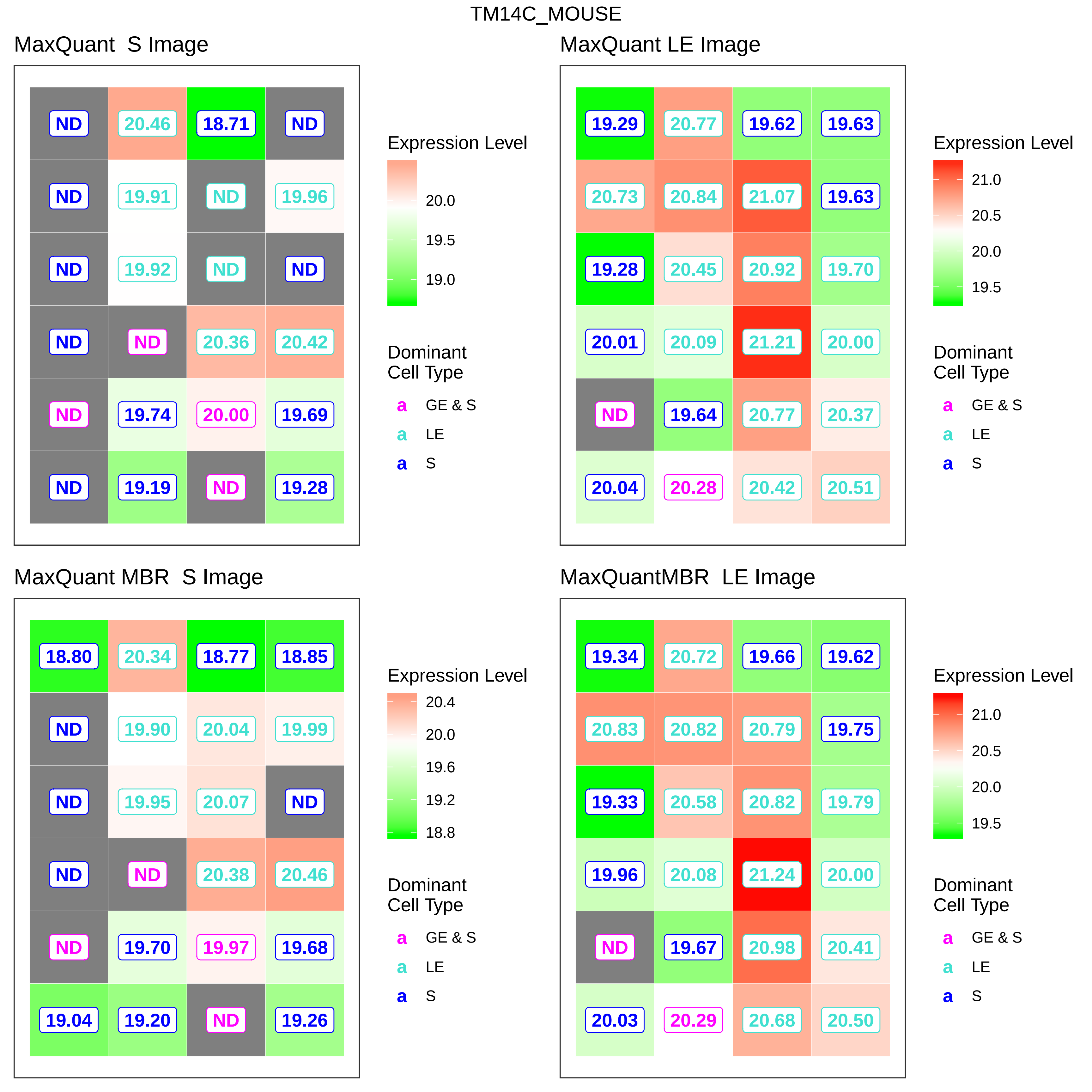
TM14C_MOUSE
MaxQuant S Image
ND 20.46 18.71 ND
ND 19.91 ND 19.96
ND 19.92 ND ND
ND ND 20.36 20.42
ND 19.74 20.00 19.69
ND 19.19 ND 19.28
Expression Level
20.0
19.5
19.0
Dominant Cell Type
a GE & S
a LE
a S
MaxQuant LE Image
19.29 20.77 19.62 19.63
20.73 20.84 21.07 19.63
19.28 20.45 20.92 19.70
20.01 20.09 21.21 20.00
ND 19.64 20.77 20.37
20.04 20.28 20.42 20.51
Expression Level
21.0
20.5
20.0
19.5
Dominant Cell Type
a GE & S
a LE
a S
MaxQuant MBR S Image
18.80 20.34 18.77 18.85
ND 19.90 20.04 19.99
ND 19.95 20.07 ND
ND ND 20.38 20.46
ND 19.70 19.97 19.68
19.04 19.20 ND 19.26
Expression Level
20.4
20.0
19.6
19.2
18.8
Dominant Cell Type
a GE & S
a LE
a S
MaxQuantMBR LE Image
19.34 20.72 19.66 19.62
20.83 20.82 20.79 19.75
19.33 20.58 20.82 19.79
19.96 20.08 21.24 20.00
ND 19.67 20.98 20.41
20.03 20.29 20.68 20.50
Expression Level
21.0
20.5
20.0
19.5
Dominant Cell Type
a GE & S
a LE
a S

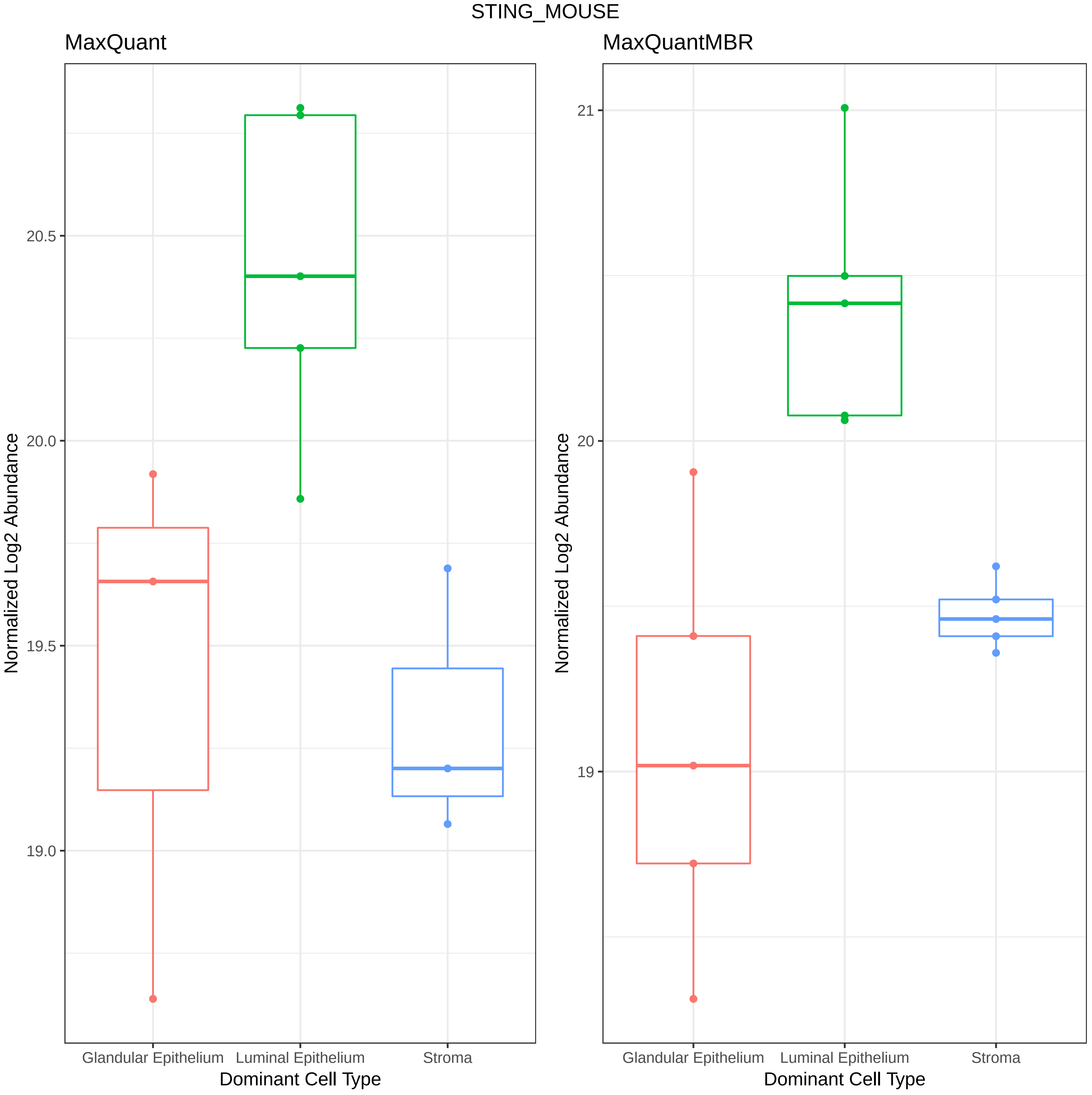
STING_MOUSE
MaxQuant
MaxQuantMBR
Normalized Log2 Abundance
20.5
20.0
19.5
19.0
21
20
19
Glandular Epithelium
Luminal Epithelium
Stroma
Dominant Cell Type

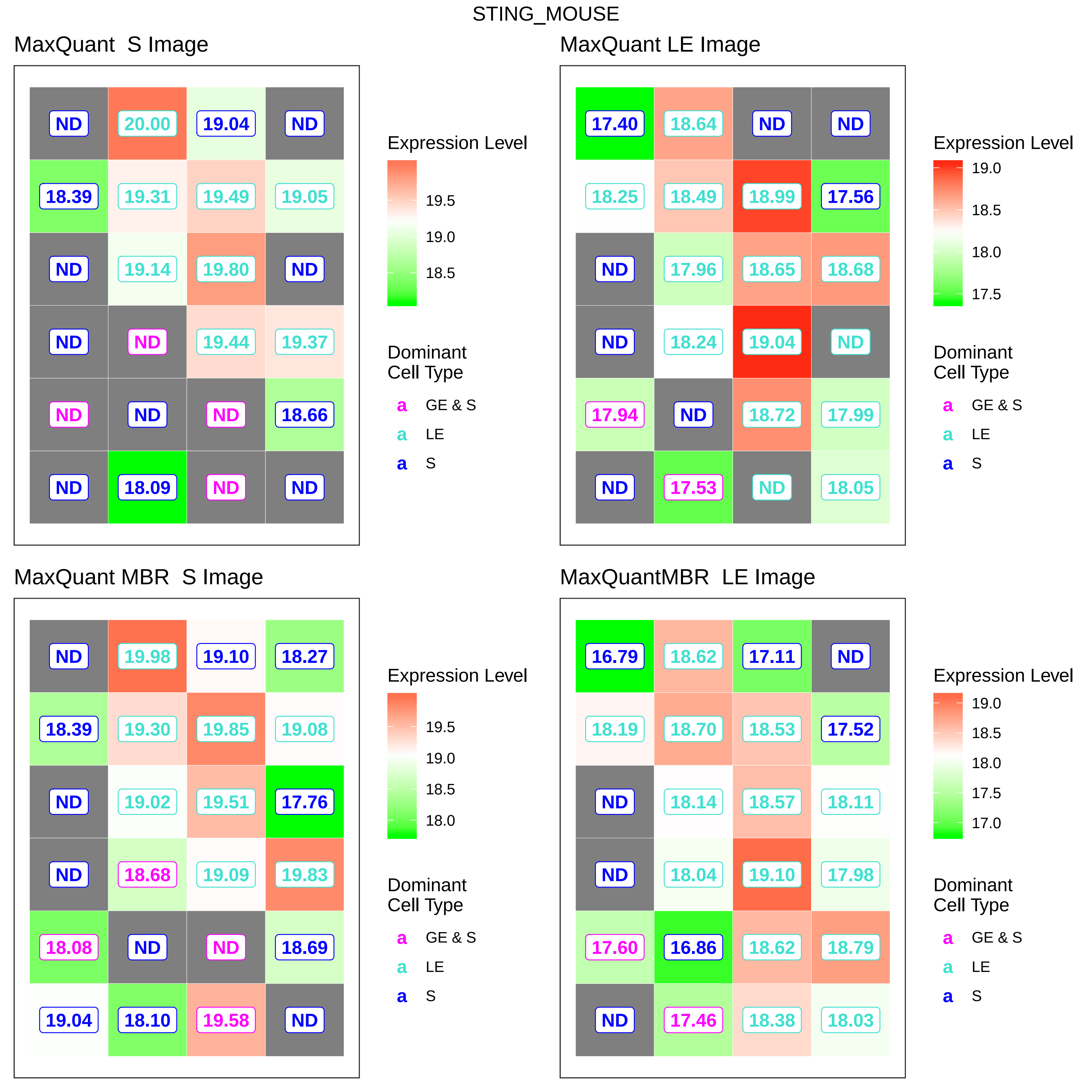
STING_MOUSE
MaxQuant S Image
ND 20.00 19.04 ND
18.39 19.31 19.49 19.05
ND 19.14 19.80 ND
ND ND 19.44 19.37
ND ND ND 18.66
ND 18.09 ND ND
Expression Level
19.5
19.0
18.5
Dominant Cell Type
a GE & S
a LE
a S
MaxQuant LE Image
17.40 18.64 ND ND
18.25 18.49 18.99 17.56
ND 17.96 18.65 18.68
ND 18.24 19.04 ND
17.94 ND 18.72 17.99
ND 17.53 ND 18.05
Expression Level
19.0
18.5
18.0
17.5
Dominant Cell Type
a GE & S
a LE
a S
MaxQuant MBR S Image
ND 19.98 19.10 18.27
18.39 19.30 19.85 19.08
ND 19.02 19.51 17.76
ND 18.68 19.09 19.83
18.08 ND ND 18.69
19.04 18.10 19.58 ND
Expression Level
19.5
19.0
18.5
18.0
Dominant Cell Type
a GE & S
a LE
a S
MaxQuantMBR LE Image
16.79 18.62 17.11 ND
18.19 18.70 18.53 17.52
ND 18.14 18.57 18.11
ND 18.04 19.10 17.98
17.60 16.86 18.62 18.79
ND 17.46 18.38 18.03
Expression Level
19.0
18.5
18.0
17.5
17.0
Dominant Cell Type
a GE & S
a LE
a S

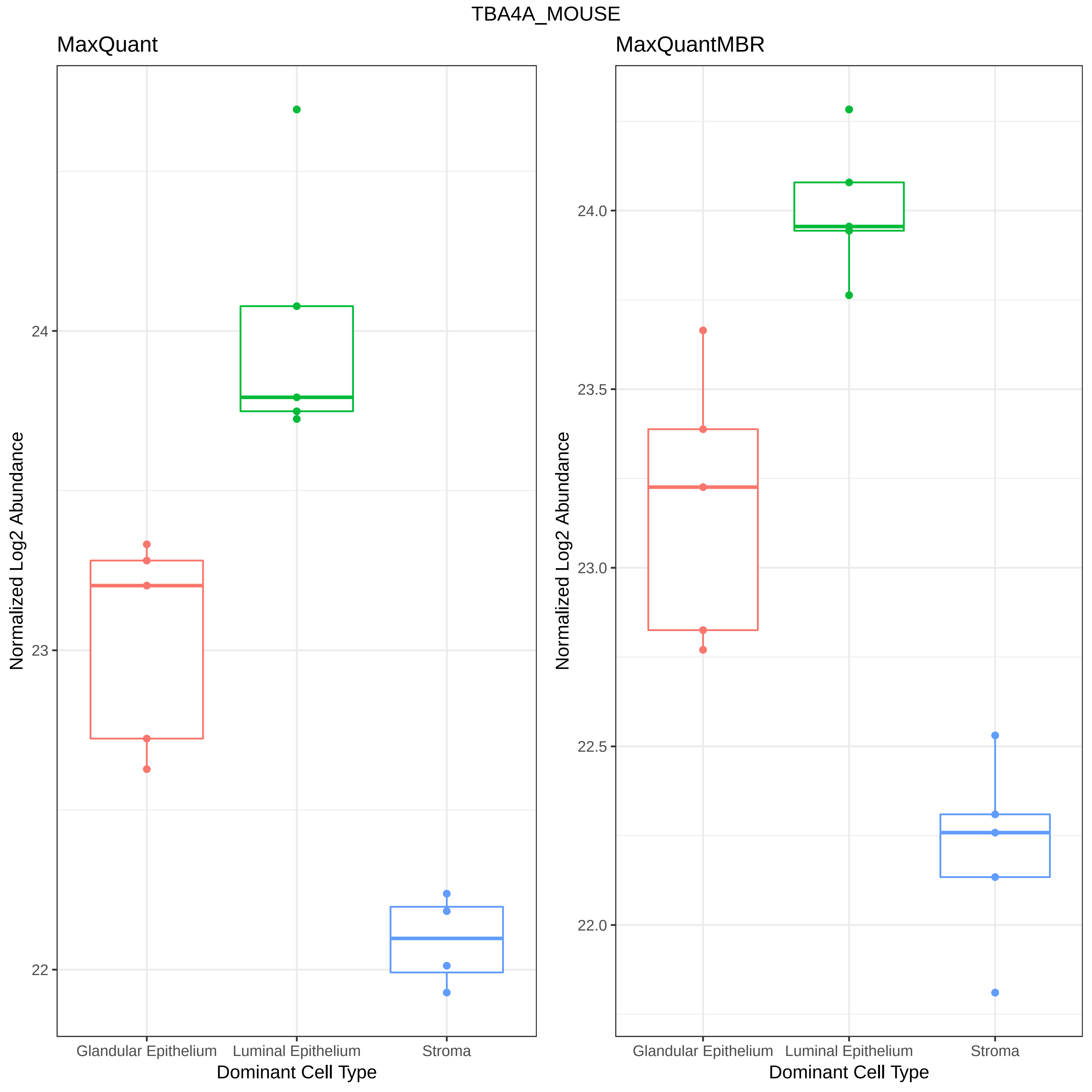
TBA4A_MOUSE
MaxQuant
MaxQuantMBR
Normalized Log2 Abundance
24
23
22
24.0
23.5
23.0
22.5
22.0
Glandular Epithelium
Luminal Epithelium
Stroma
Dominant Cell Type

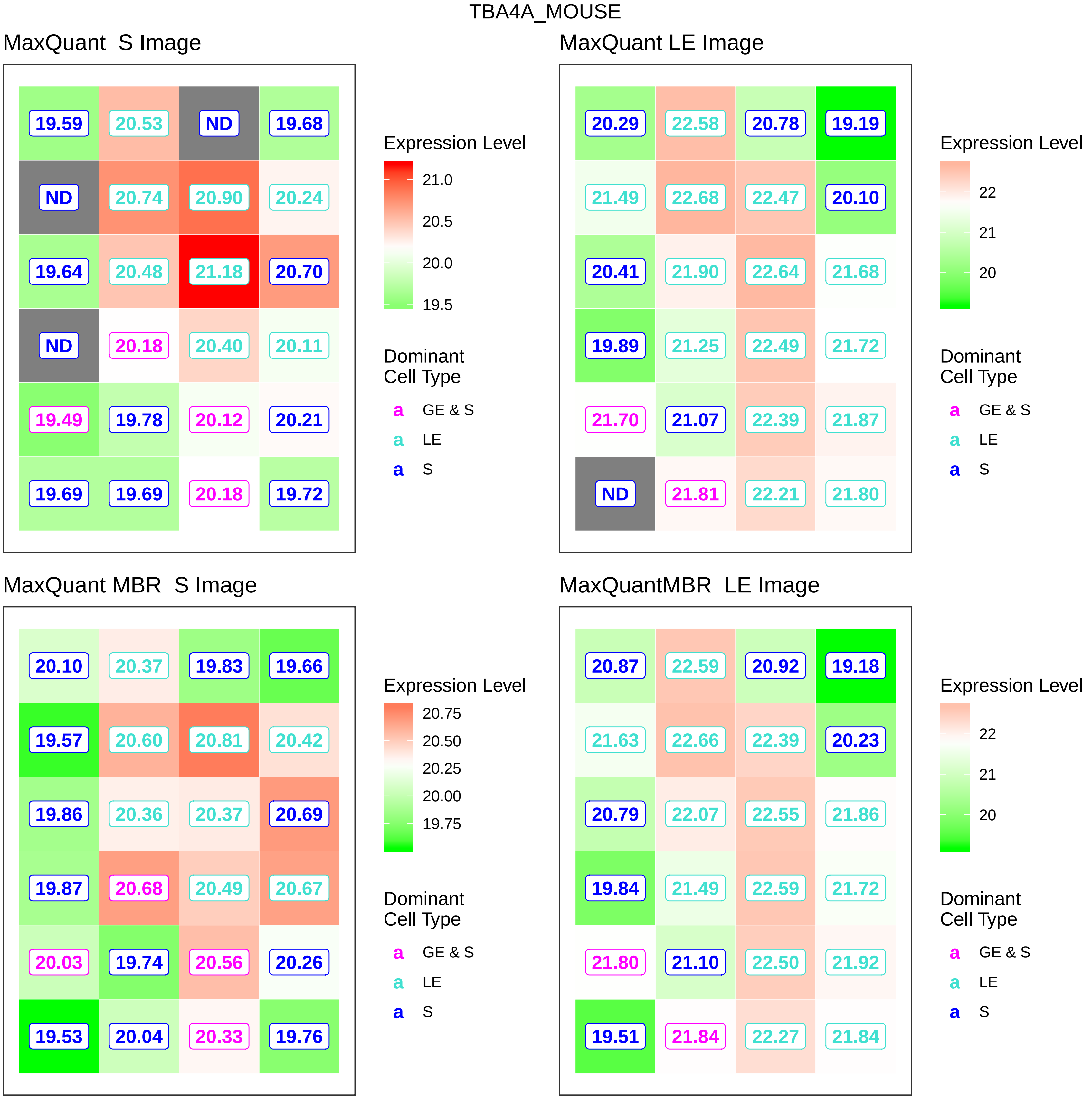
TBA4A_MOUSE
MaxQuant S Image
19.59 20.53 ND 19.68
ND 20.74 20.90 20.24
19.64 20.48 21.18 20.70
ND 20.18 20.40 20.11
19.49 19.78 20.12 20.21
19.69 19.69 20.18 19.72
Expression Level
21.0
20.5
20.0
19.5
Dominant Cell Type
a GE & S
a LE
a S
MaxQuant LE Image
20.29 22.58 20.78 19.19
21.49 22.68 22.47 20.10
20.41 21.90 22.64 21.68
19.89 21.25 22.49 21.72
21.70 21.07 22.39 21.87
ND 21.81 22.21 21.80
Expression Level
22
21
20
Dominant Cell Type
a GE & S
a LE
a S
MaxQuant MBR S Image
20.10 20.37 19.83 19.66
19.57 20.60 20.81 20.42
19.86 20.36 20.37 20.69
19.87 20.68 20.49 20.67
20.03 19.74 20.56 20.26
19.53 20.04 20.33 19.76
Expression Level
20.75
20.50
20.25
20.00
19.75
Dominant Cell Type
a GE & S
a LE
a S
MaxQuantMBR LE Image
20.87 22.59 20.92 19.18
21.63 22.66 22.39 20.23
20.79 22.07 22.55 21.86
19.84 21.49 22.59 21.72
21.80 21.10 22.50 21.92
19.51 21.84 22.27 21.84
Expression Level
22
21
20
Dominant Cell Type
a GE & S
a LE
a S

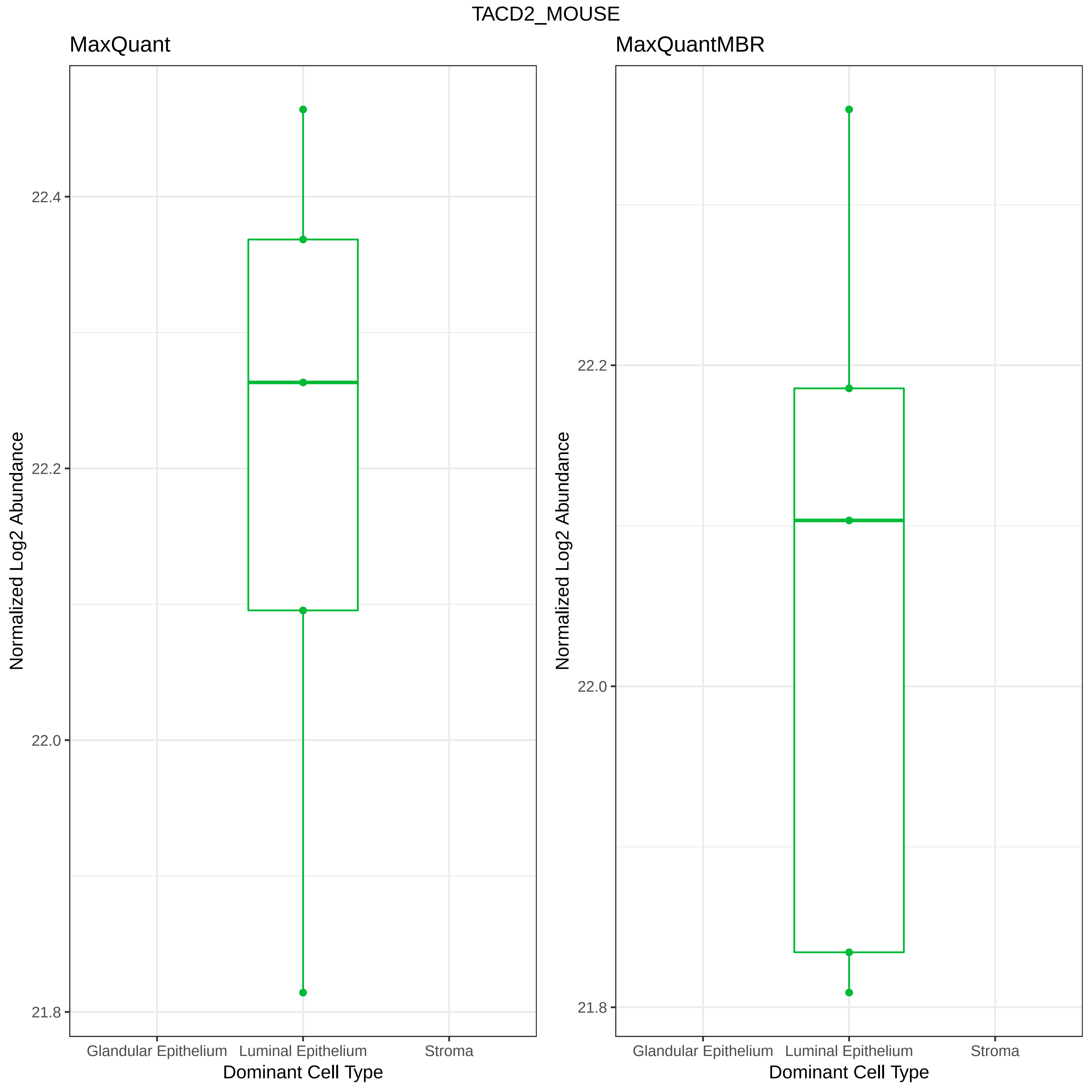

TACD2_MOUSE
MaxQuant
MaxQuantMBR
Normalized Log2 Abundance
22.4
22.2
22.0
21.8
Glandular Epithelium
Luminal Epithelium
Stroma
Dominant Cell Type

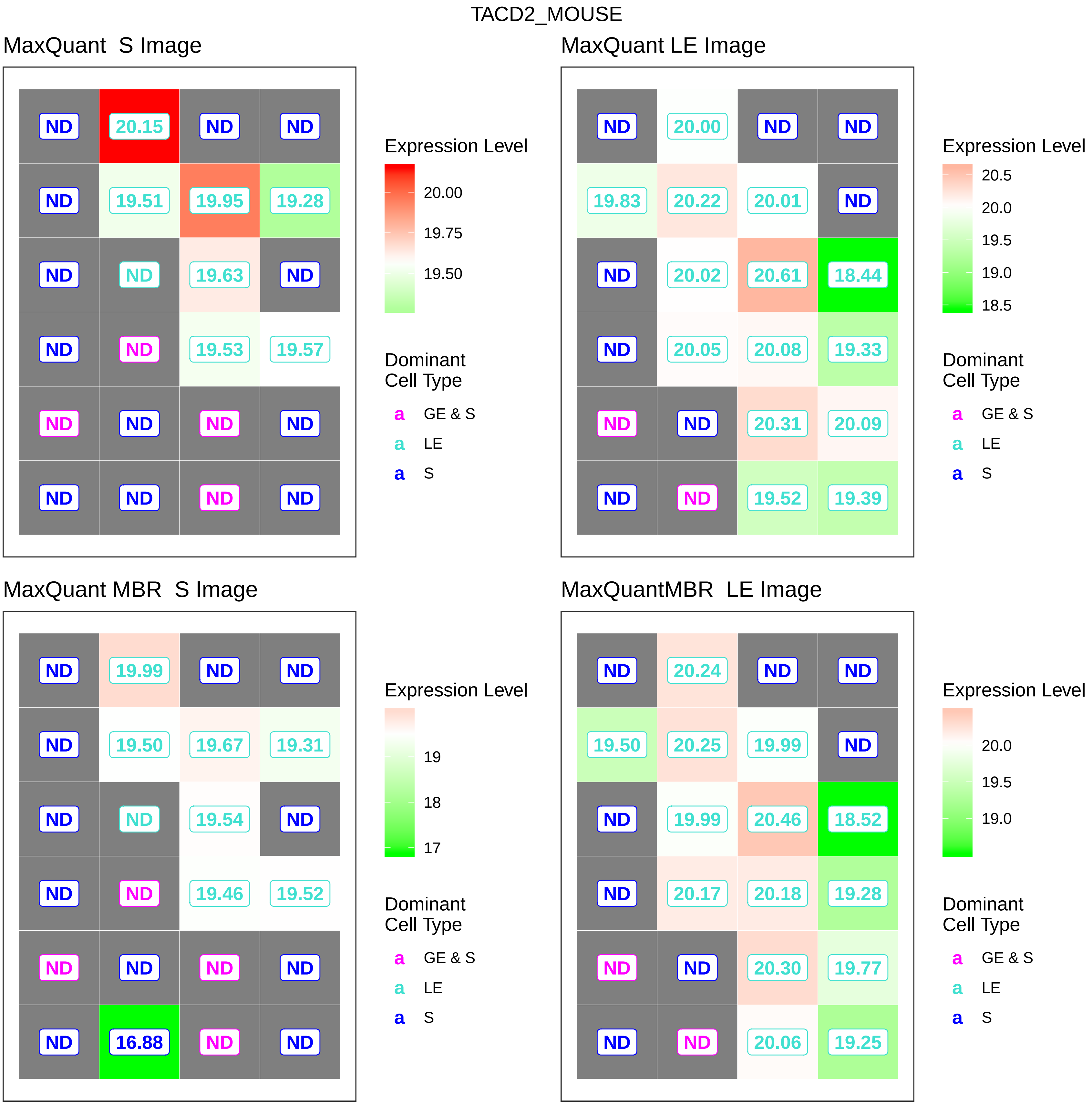
TACD2_MOUSE
MaxQuant S Image
ND
20.15
ND
ND
ND
19.51
19.95
19.28
ND
ND
19.63
ND
ND
ND
19.53
19.57
ND
ND
ND
ND
ND
ND
ND
ND
Expression Level
20.00
19.75
19.50
Dominant
Cell Type
a GE & S
a LE
a S
MaxQuant LE Image
ND
20.00
ND
ND
19.83
20.22
20.01
ND
ND
20.02
20.61
18.44
ND
20.05
20.08
19.33
ND
ND
20.31
20.09
ND
ND
19.52
19.39
Expression Level
20.5
20.0
19.5
19.0
18.5
Dominant
Cell Type
a GE & S
a LE
a S
MaxQuant MBR S Image
ND
19.99
ND
ND
ND
19.50
19.67
19.31
ND
ND
19.54
ND
ND
ND
19.46
19.52
ND
ND
ND
ND
ND
16.88
ND
ND
Expression Level
19
18
17
Dominant
Cell Type
a GE & S
a LE
a S
MaxQuantMBR LE Image
ND
20.24
ND
ND
19.50
20.25
19.99
ND
ND
19.99
20.46
18.52
ND
20.17
20.18
19.28
ND
ND
20.30
19.77
ND
ND
20.06
19.25
Expression Level
20.0
19.5
19.0
Dominant
Cell Type
a GE & S
a LE
a S

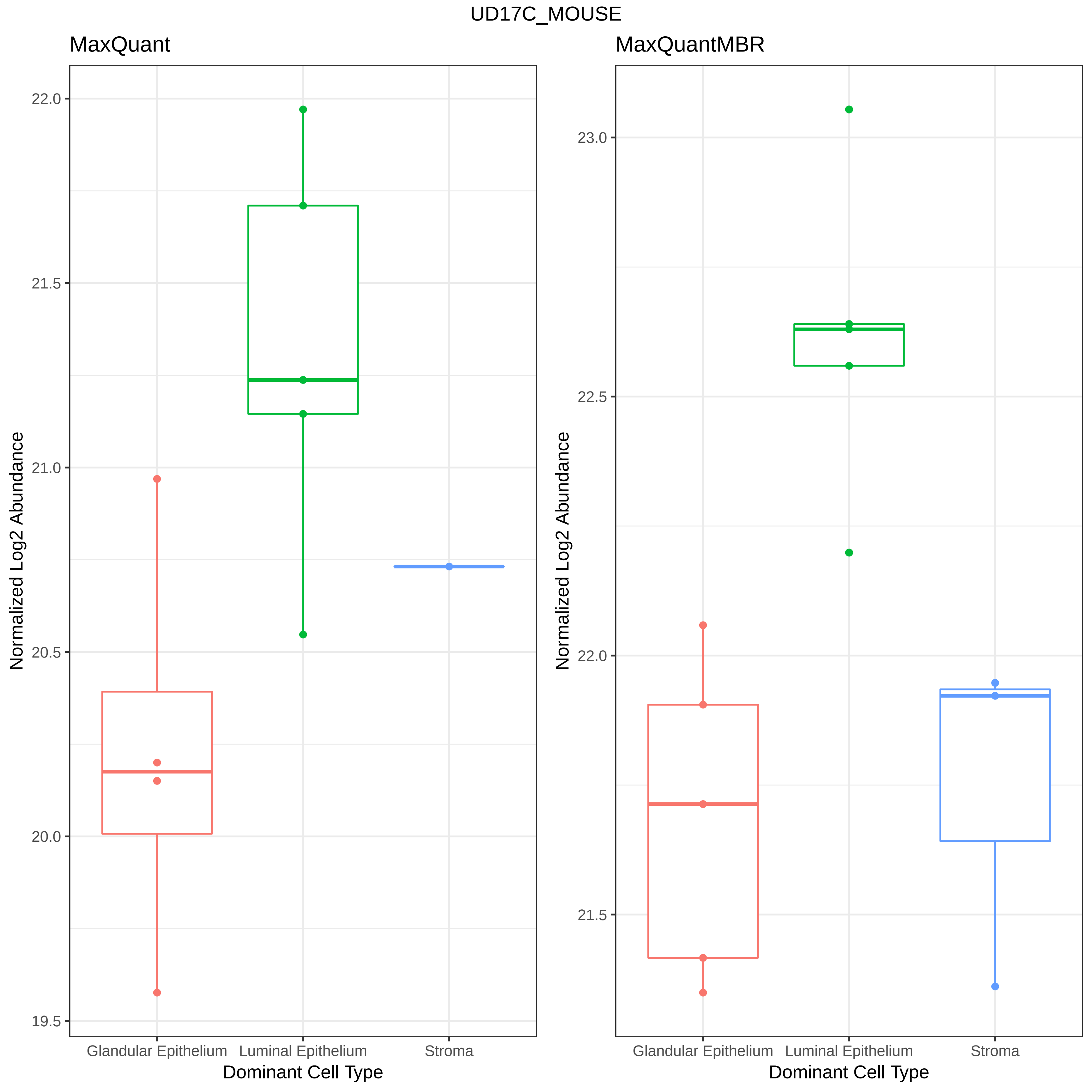
UD17C_MOUSE
MaxQuant
MaxQuantMBR
Normalized Log2 Abundance
22.0
21.5
21.0
20.5
20.0
19.5
23.0
22.5
22.0
21.5
Glandular Epithelium
Luminal Epithelium
Stroma
Dominant Cell Type

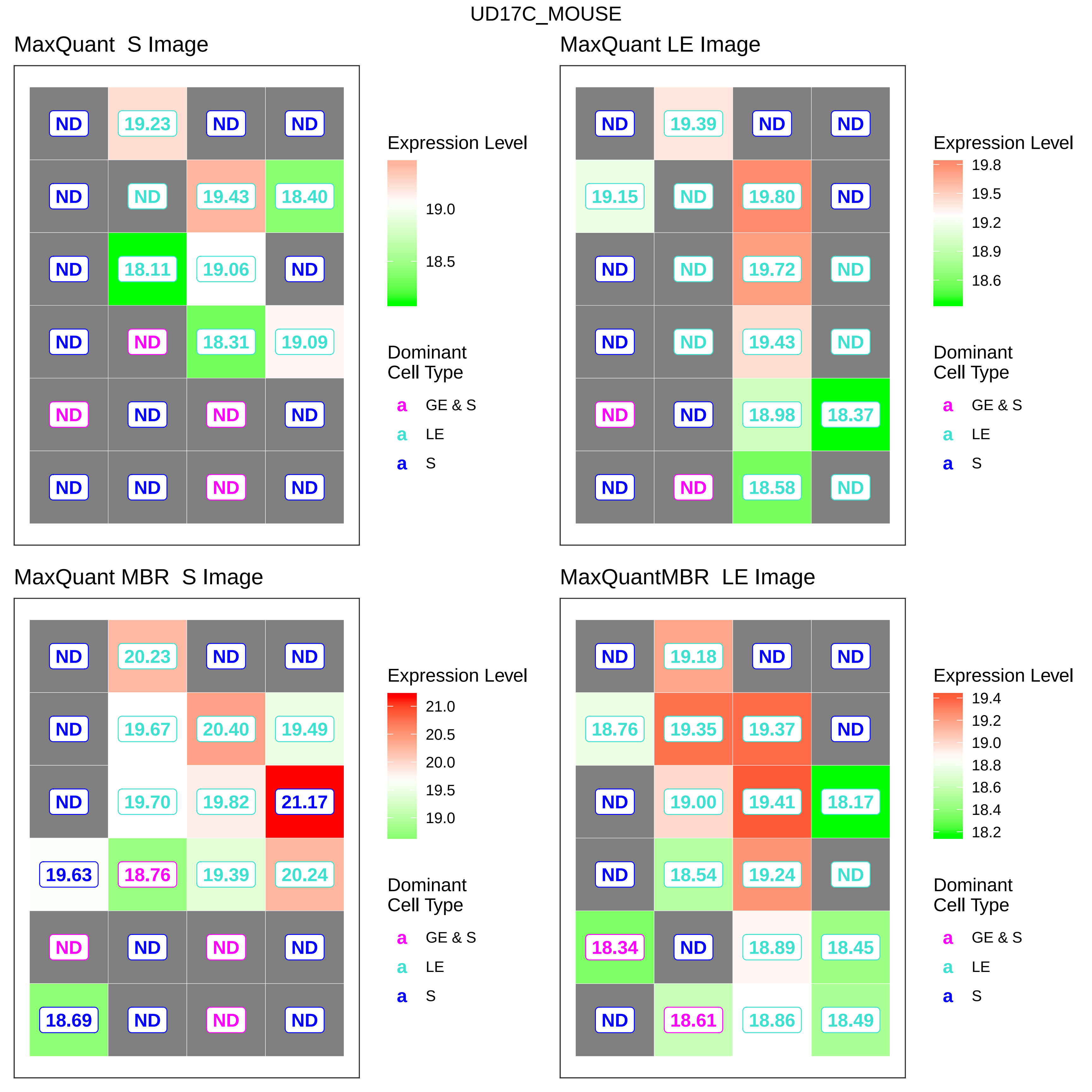

UD17C_MOUSE
MaxQuant S Image
ND 19.23 ND ND
ND ND 19.43 18.40
ND 18.11 19.06 ND
ND ND 18.31 19.09
ND ND ND ND
ND ND ND ND
Expression Level
19.0
18.5
Dominant Cell Type
a GE & S
a LE
a S
MaxQuant LE Image
ND 19.39 ND ND
19.15 ND 19.80 ND
ND ND 19.72 ND
ND ND 19.43 ND
ND ND 18.98 18.37
ND ND 18.58 ND
Expression Level
19.8
19.5
19.2
18.9
18.6
Dominant Cell Type
a GE & S
a LE
a S
MaxQuant MBR S Image
ND 20.23 ND ND
ND 19.67 20.40 19.49
ND 19.70 19.82 21.17
19.63 18.76 19.39 20.24
ND ND ND ND
18.69 ND ND ND
Expression Level
21.0
20.5
20.0
19.5
19.0
Dominant Cell Type
a GE & S
a LE
a S
MaxQuantMBR LE Image
ND 19.18 ND ND
18.76 19.35 19.37 ND
ND 19.00 19.41 18.17
ND 18.54 19.24 ND
18.34 ND 18.89 18.45
ND 18.61 18.86 18.49
Expression Level
19.4
19.2
19.0
18.8
18.6
18.4
18.2
Dominant Cell Type
a GE & S
a LE
a S

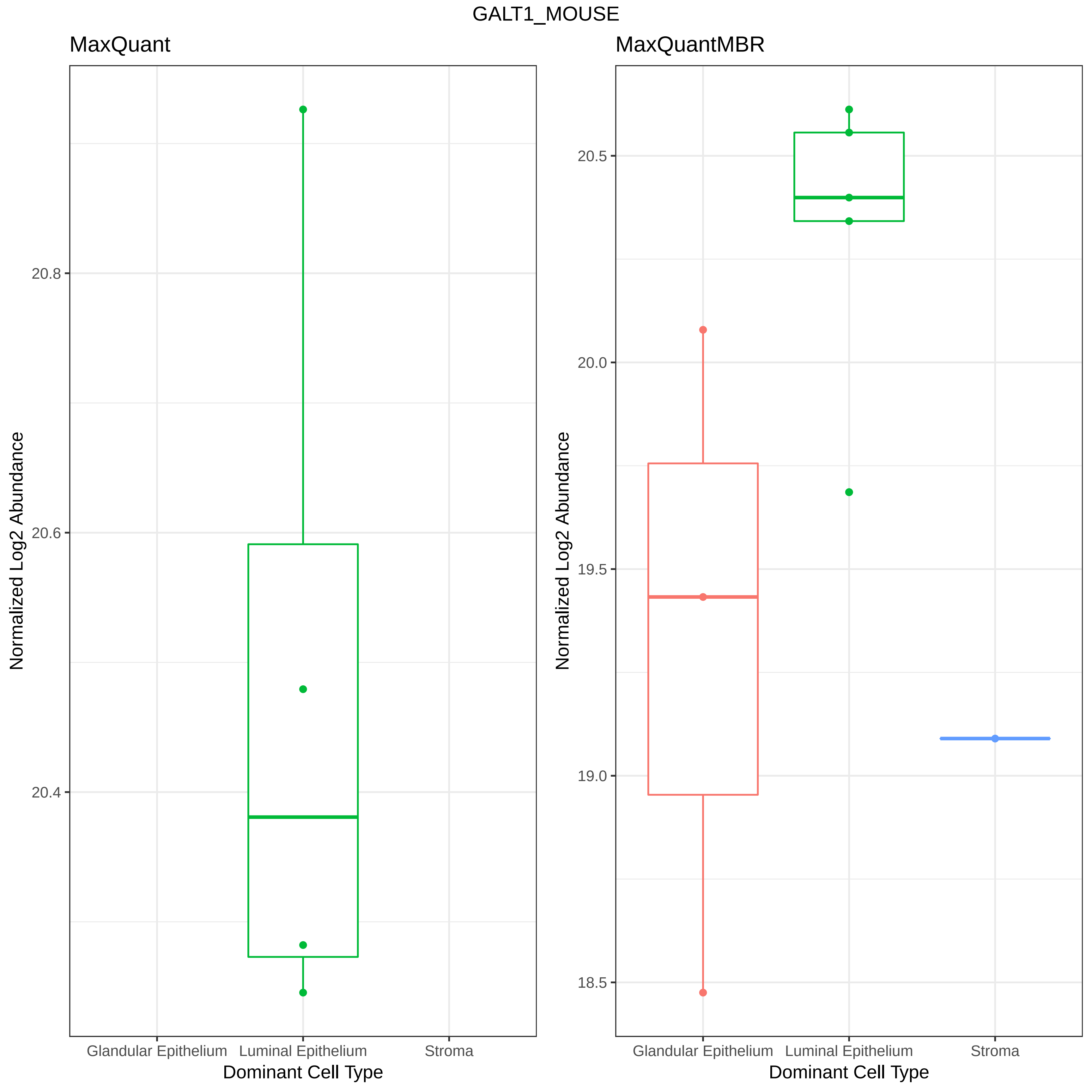

GALT1_MOUSE
MaxQuant
MaxQuantMBR
Normalized Log2 Abundance
20.8
20.6
20.4
20.5
20.0
19.5
19.0
18.5
Glandular Epithelium
Luminal Epithelium
Stroma
Dominant Cell Type

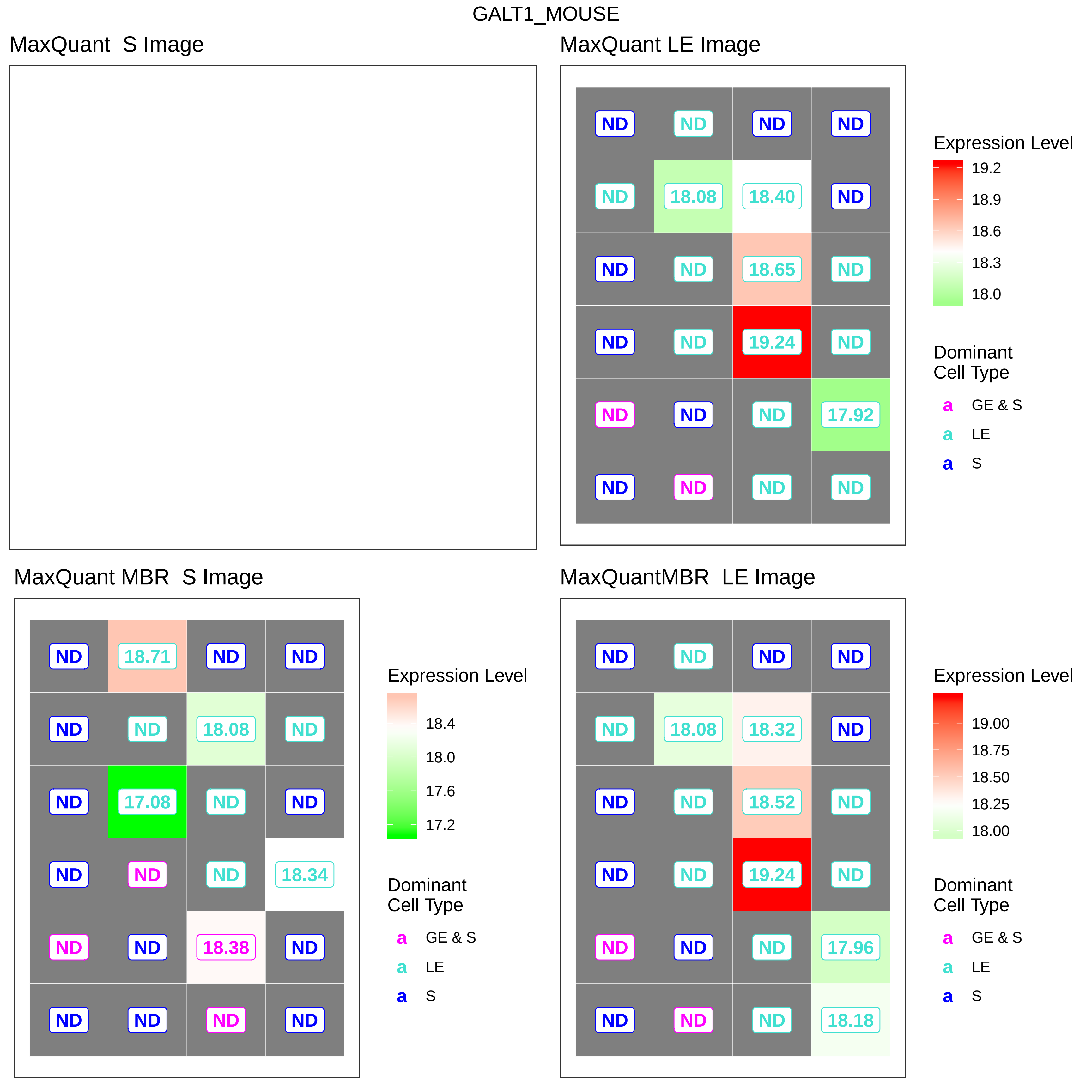
GALT1_MOUSE
MaxQuant S Image
MaxQuant LE Image
ND ND ND ND
ND 18.08 18.40 ND
ND ND 18.65 ND
ND ND 19.24 ND
ND ND ND 17.92
ND ND ND ND
Expression Level
19.2
18.9
18.6
18.3
18.0
Dominant Cell Type
a GE & S
a LE
a S
MaxQuant MBR S Image
ND 18.71 ND ND
ND ND 18.08 ND
ND 17.08 ND ND
ND ND ND 18.34
ND ND 18.38 ND
ND ND ND ND
Expression Level
18.4
18.0
17.6
17.2
Dominant Cell Type
a GE & S
a LE
a S
MaxQuantMBR LE Image
ND ND ND ND
ND 18.08 18.32 ND
ND ND 18.52 ND
ND ND 19.24 ND
ND ND ND 17.96
ND ND ND 18.18
Expression Level
19.00
18.75
18.50
18.25
18.00
Dominant Cell Type
a GE & S
a LE
a S

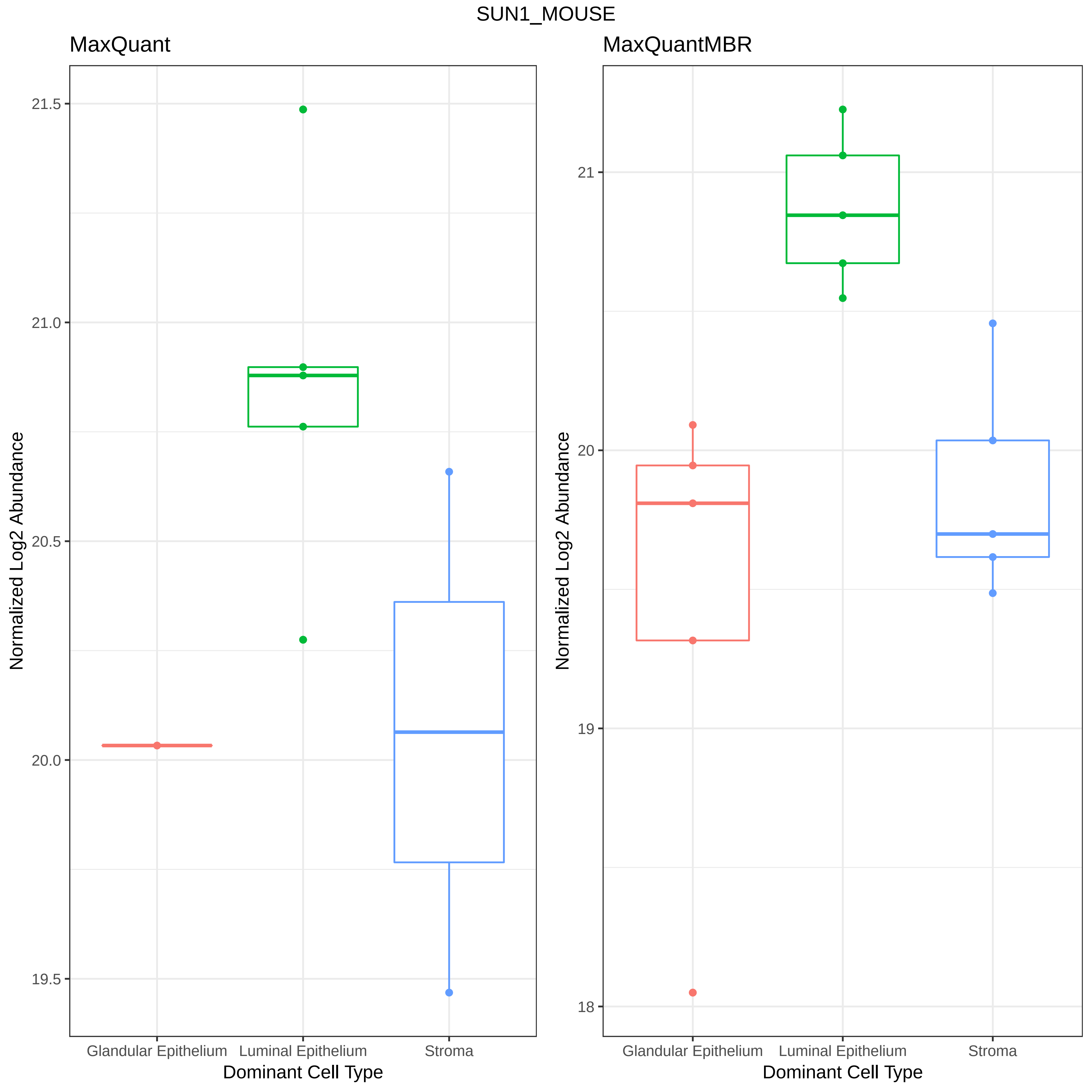
SUN1_MOUSE
MaxQuant
MaxQuantMBR
Normalized Log2 Abundance
21.5
21.0
20.5
20.0
19.5
21
20
19
18
Glandular Epithelium
Luminal Epithelium
Stroma
Dominant Cell Type

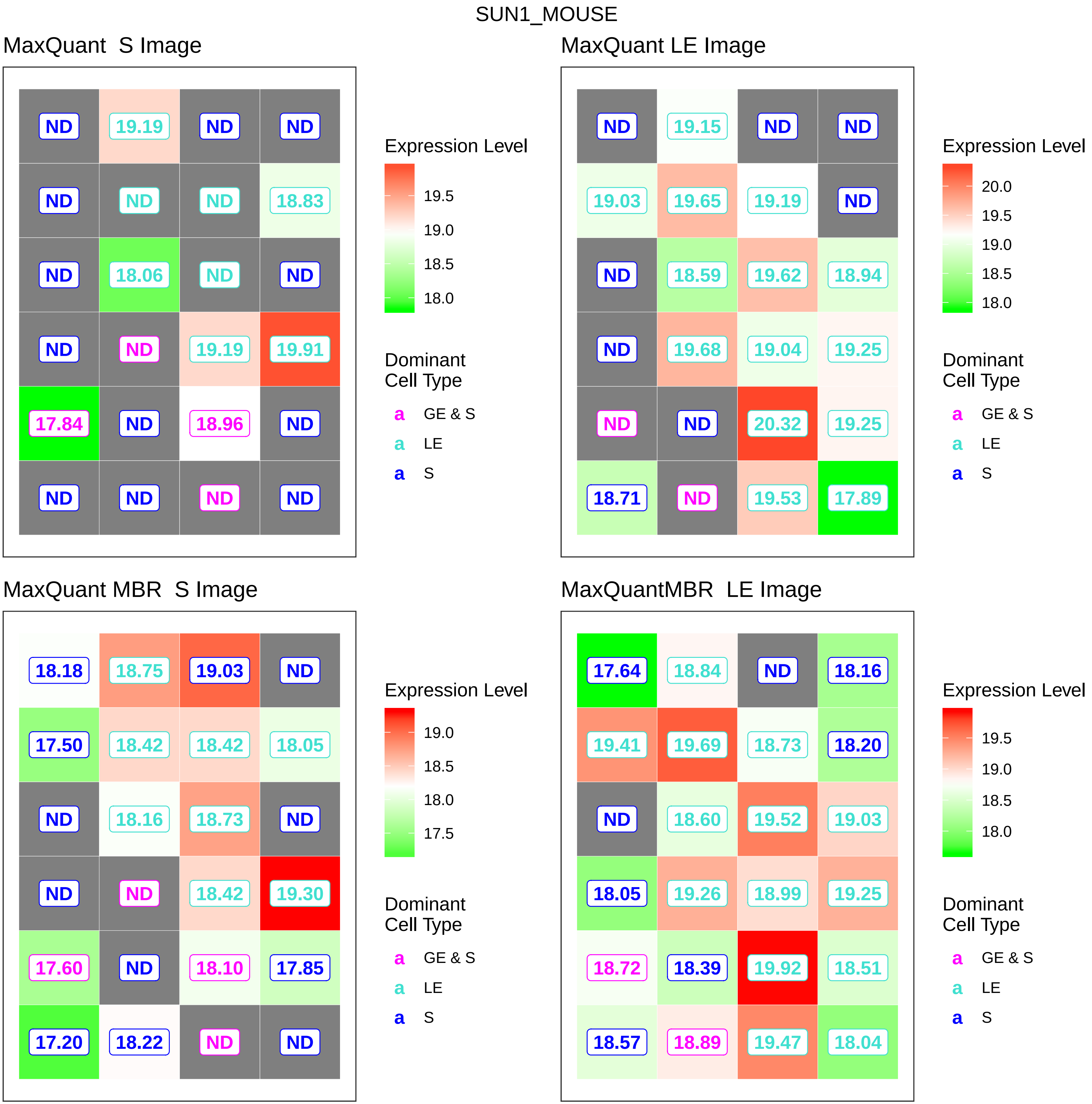

SUN1_MOUSE
MaxQuant S Image
ND 19.19 ND ND
ND ND ND 18.83
ND 18.06 ND ND
ND ND 19.19 19.91
17.84 ND 18.96 ND
ND ND ND ND
Expression Level
19.5
19.0
18.5
18.0
Dominant Cell Type
a GE & S
a LE
a S
MaxQuant LE Image
ND 19.15 ND ND
19.03 19.65 19.19 ND
ND 18.59 19.62 18.94
ND 19.68 19.04 19.25
ND ND 20.32 19.25
18.71 ND 19.53 17.89
Expression Level
20.0
19.5
19.0
18.5
18.0
Dominant Cell Type
a GE & S
a LE
a S
MaxQuant MBR S Image
18.18 18.75 19.03 ND
17.50 18.42 18.42 18.05
ND 18.16 18.73 ND
ND ND 18.42 19.30
17.60 ND 18.10 17.85
17.20 18.22 ND ND
Expression Level
19.0
18.5
18.0
17.5
Dominant Cell Type
a GE & S
a LE
a S
MaxQuantMBR LE Image
17.64 18.84 ND 18.16
19.41 19.69 18.73 18.20
ND 18.60 19.52 19.03
18.05 19.26 18.99 19.25
18.72 18.39 19.92 18.51
18.57 18.89 19.47 18.04
Expression Level
19.5
19.0
18.5
18.0
Dominant Cell Type
a GE & S
a LE
a S

VATD_MOUSE

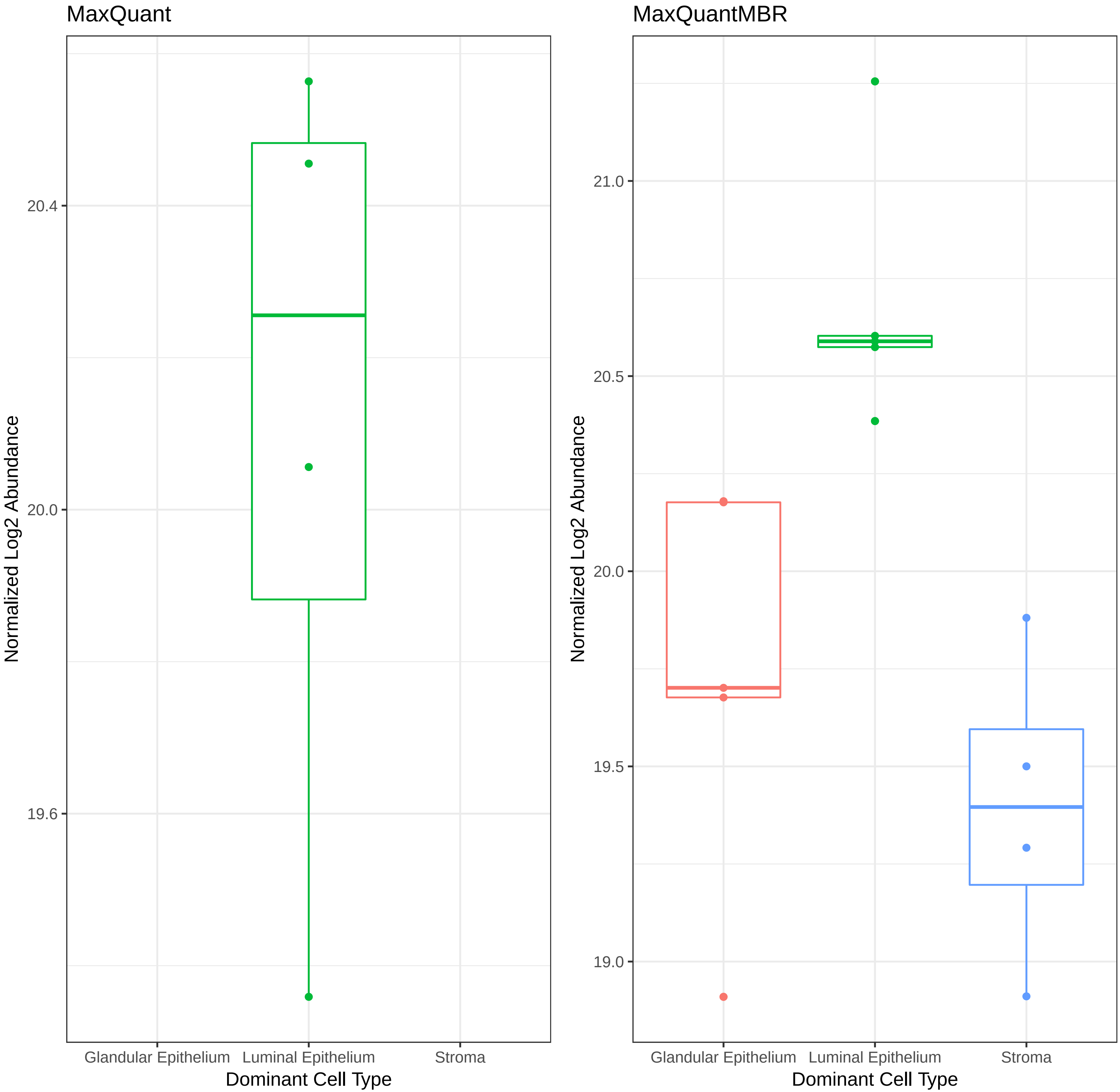

MaxQuant
MaxQuantMBR
20.4
20.0
19.6
21.0
20.5
20.0
19.5
19.0
Normalized Log2 Abundance
Normalized Log2 Abundance
Glandular Epithelium
Luminal Epithelium
Stroma
Glandular Epithelium
Luminal Epithelium
Stroma
Dominant Cell Type
Dominant Cell Type

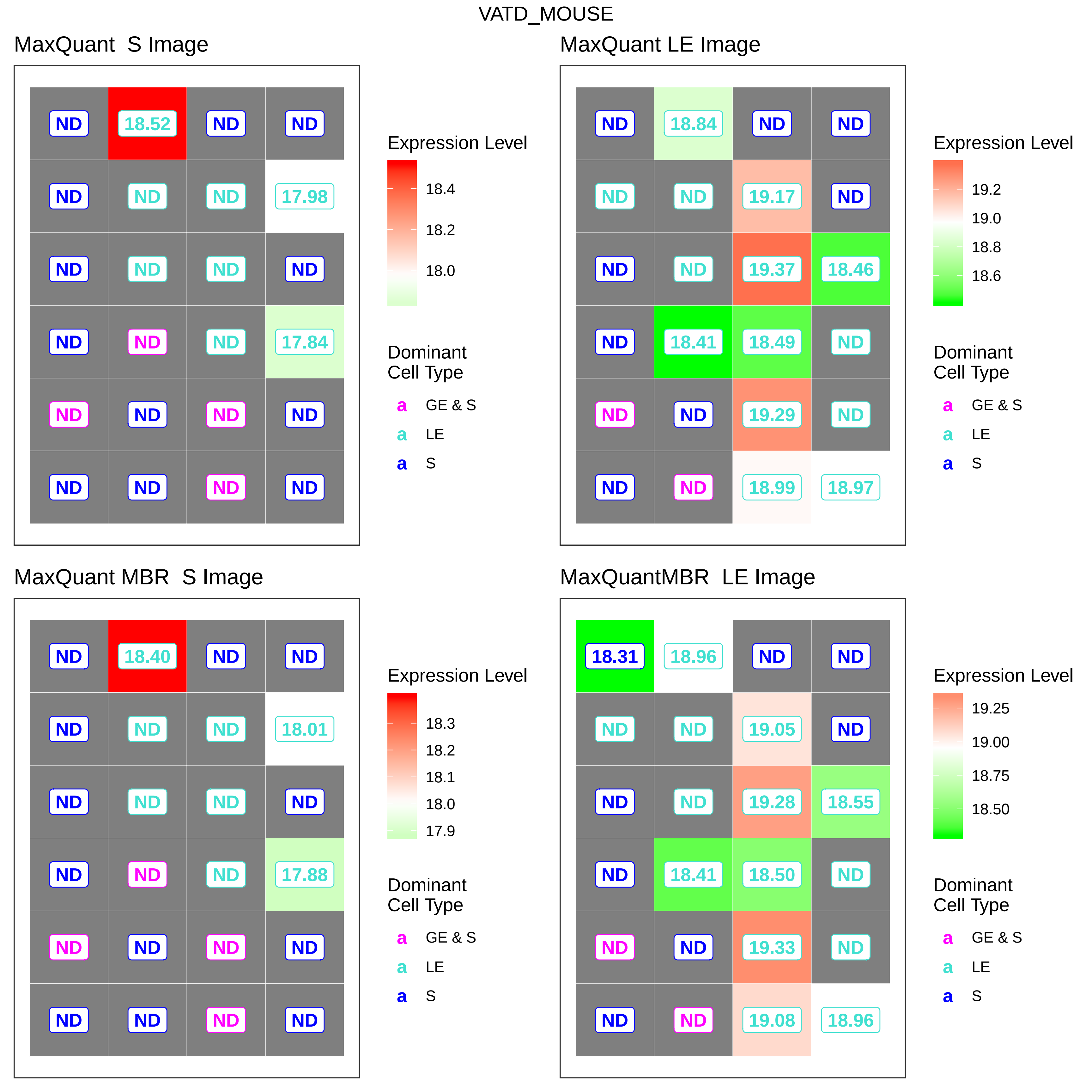

VATD_MOUSE
MaxQuant S Image
ND 18.52 ND ND
ND ND ND 17.98
ND ND ND ND
ND ND ND 17.84
ND ND ND ND
ND ND ND ND
Expression Level
18.4
18.2
18.0
Dominant Cell Type
a GE & S
a LE
a S
MaxQuant LE Image
ND 18.84 ND ND
ND ND 19.17 ND
ND ND 19.37 18.46
ND 18.41 18.49 ND
ND ND 19.29 ND
ND ND 18.99 18.97
Expression Level
19.2
19.0
18.8
18.6
Dominant Cell Type
a GE & S
a LE
a S
MaxQuant MBR S Image
ND 18.40 ND ND
ND ND ND 18.01
ND ND ND ND
ND ND ND 17.88
ND ND ND ND
ND ND ND ND
Expression Level
18.3
18.2
18.1
18.0
17.9
Dominant Cell Type
a GE & S
a LE
a S
MaxQuantMBR LE Image
18.31 18.96 ND ND
ND ND 19.05 ND
ND ND 19.28 18.55
ND 18.41 18.50 ND
ND ND 19.33 ND
ND ND 19.08 18.96
Expression Level
19.25
19.00
18.75
18.50
Dominant Cell Type
a GE & S
a LE
a S

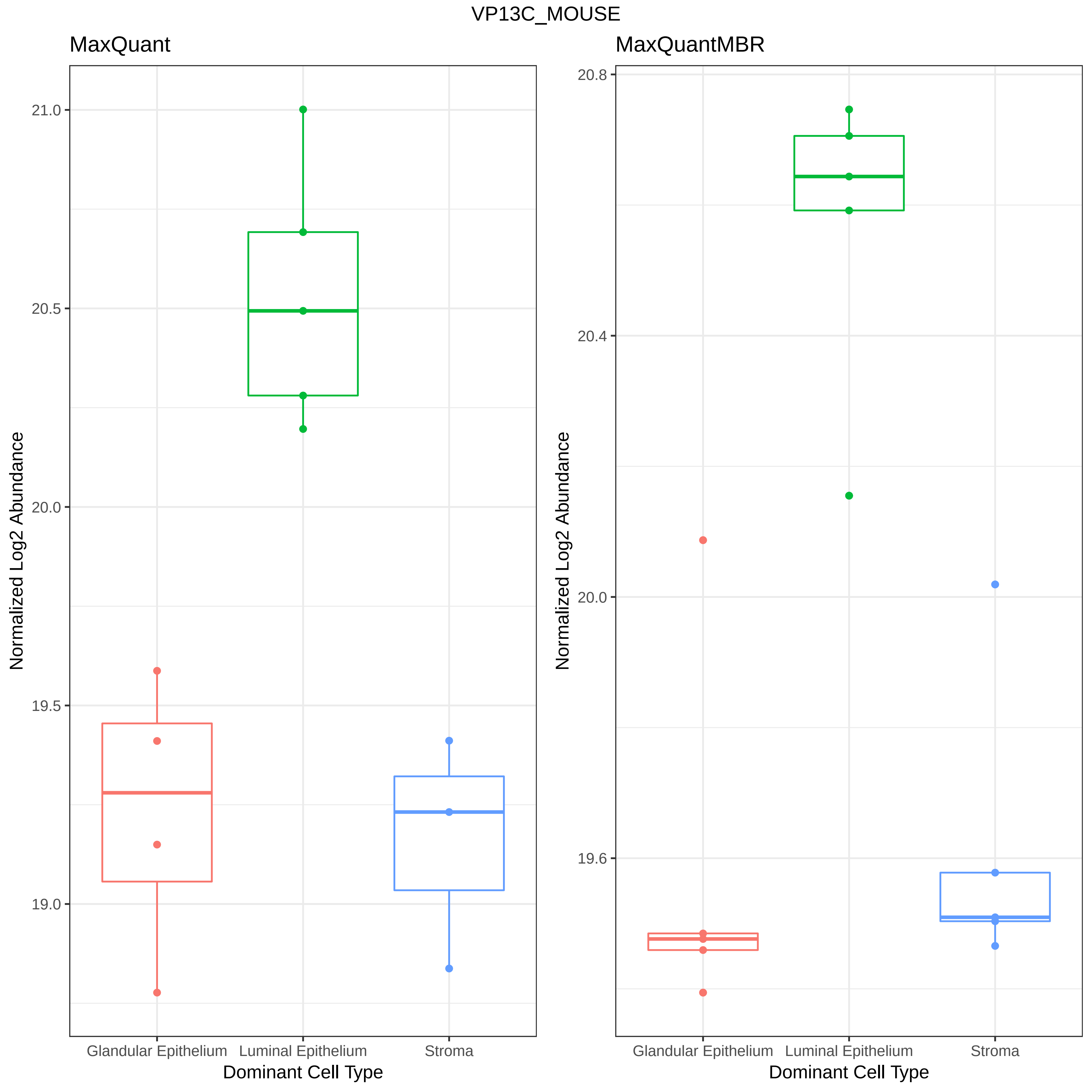
VP13C_MOUSE
MaxQuant
MaxQuantMBR
Normalized Log2 Abundance
21.0
20.5
20.0
19.5
19.0
20.8
20.4
20.0
19.6
Glandular Epithelium
Luminal Epithelium
Stroma
Dominant Cell Type

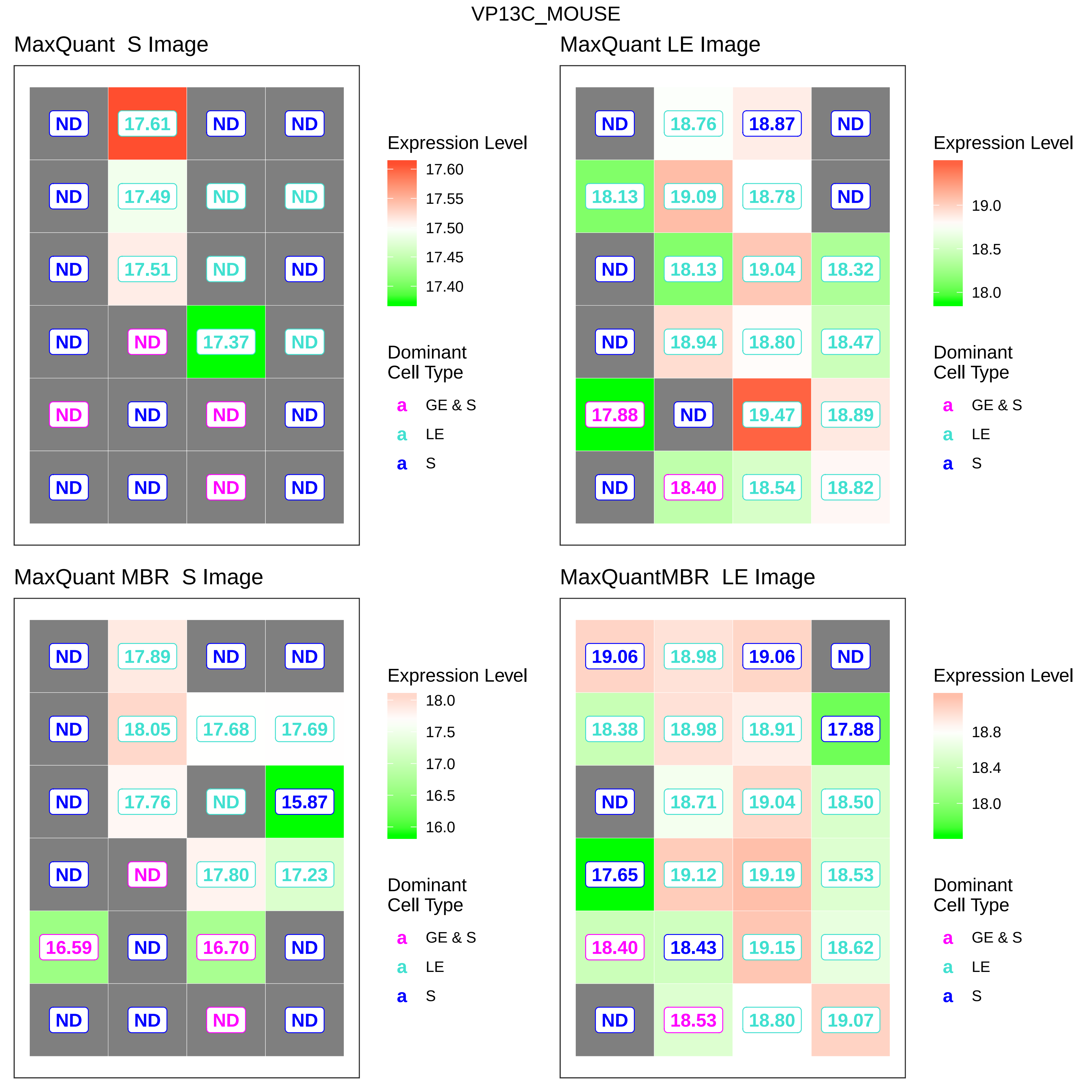

VP13C_MOUSE
MaxQuant S Image
ND
17.61
ND
ND
ND
17.49
ND
ND
ND
17.51
ND
ND
ND
ND
17.37
ND
ND
ND
ND
ND
ND
ND
ND
ND
Expression Level
17.60
17.55
17.50
17.45
17.40
Dominant Cell Type
a GE & S
a LE
a S
MaxQuant LE Image
ND
18.76
18.87
ND
18.13
19.09
18.78
ND
ND
18.13
19.04
18.32
ND
18.94
18.80
18.47
17.88
ND
19.47
18.89
ND
18.40
18.54
18.82
Expression Level
19.0
18.5
18.0
Dominant Cell Type
a GE & S
a LE
a S
MaxQuant MBR S Image
ND
17.89
ND
ND
ND
18.05
17.68
17.69
ND
17.76
ND
15.87
ND
ND
17.80
17.23
16.59
ND
16.70
ND
ND
ND
ND
ND
Expression Level
18.0
17.5
17.0
16.5
16.0
Dominant Cell Type
a GE & S
a LE
a S
MaxQuantMBR LE Image
19.06
18.98
19.06
ND
18.38
18.98
18.91
17.88
ND
18.71
19.04
18.50
17.65
19.12
19.19
18.53
18.40
18.43
19.15
18.62
ND
18.53
18.80
19.07
Expression Level
18.8
18.4
18.0
Dominant Cell Type
a GE & S
a LE
a S

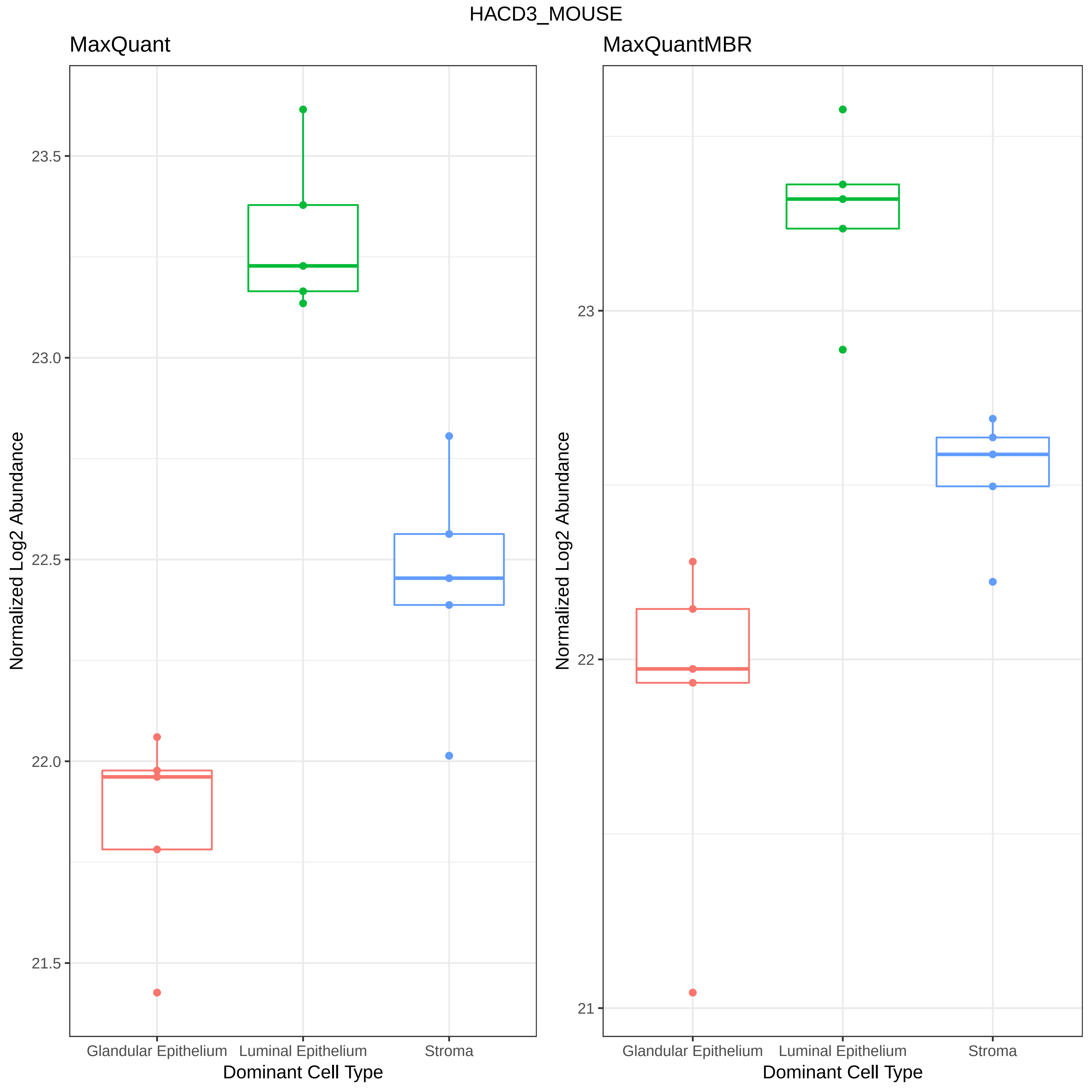

HACD3_MOUSE
MaxQuant
MaxQuantMBR
Normalized Log2 Abundance
23.5
23.0
22.5
22.0
21.5
23
22
21
Glandular Epithelium
Luminal Epithelium
Stroma
Dominant Cell Type

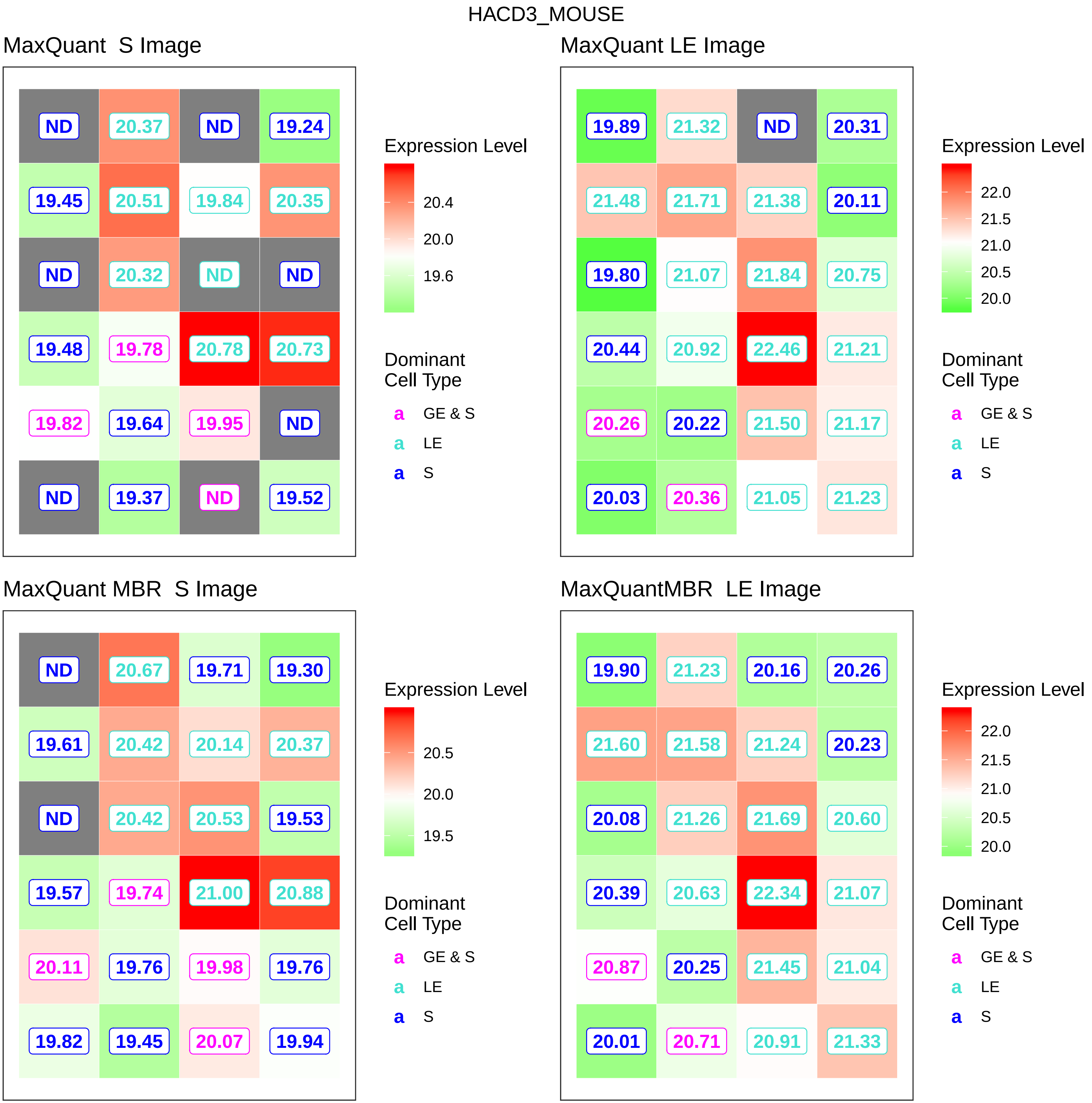
HACD3_MOUSE
MaxQuant S Image
ND 20.37 ND 19.24
19.45 20.51 19.84 20.35
ND 20.32 ND ND
19.48 19.78 20.78 20.73
19.82 19.64 19.95 ND
ND 19.37 ND 19.52
Expression Level
20.4
20.0
19.6
Dominant Cell Type
a GE & S
a LE
a S
MaxQuant LE Image
19.89 21.32 ND 20.31
21.48 21.71 21.38 20.11
19.80 21.07 21.84 20.75
20.44 20.92 22.46 21.21
20.26 20.22 21.50 21.17
20.03 20.36 21.05 21.23
Expression Level
22.0
21.5
21.0
20.5
20.0
Dominant Cell Type
a GE & S
a LE
a S
MaxQuant MBR S Image
ND 20.67 19.71 19.30
19.61 20.42 20.14 20.37
ND 20.42 20.53 19.53
19.57 19.74 21.00 20.88
20.11 19.76 19.98 19.76
19.82 19.45 20.07 19.94
Expression Level
20.5
20.0
19.5
Dominant Cell Type
a GE & S
a LE
a S
MaxQuantMBR LE Image
19.90 21.23 20.16 20.26
21.60 21.58 21.24 20.23
20.08 21.26 21.69 20.60
20.39 20.63 22.34 21.07
20.87 20.25 21.45 21.04
20.01 20.71 20.91 21.33
Expression Level
22.0
21.5
21.0
20.5
20.0
Dominant Cell Type
a GE & S
a LE
a S

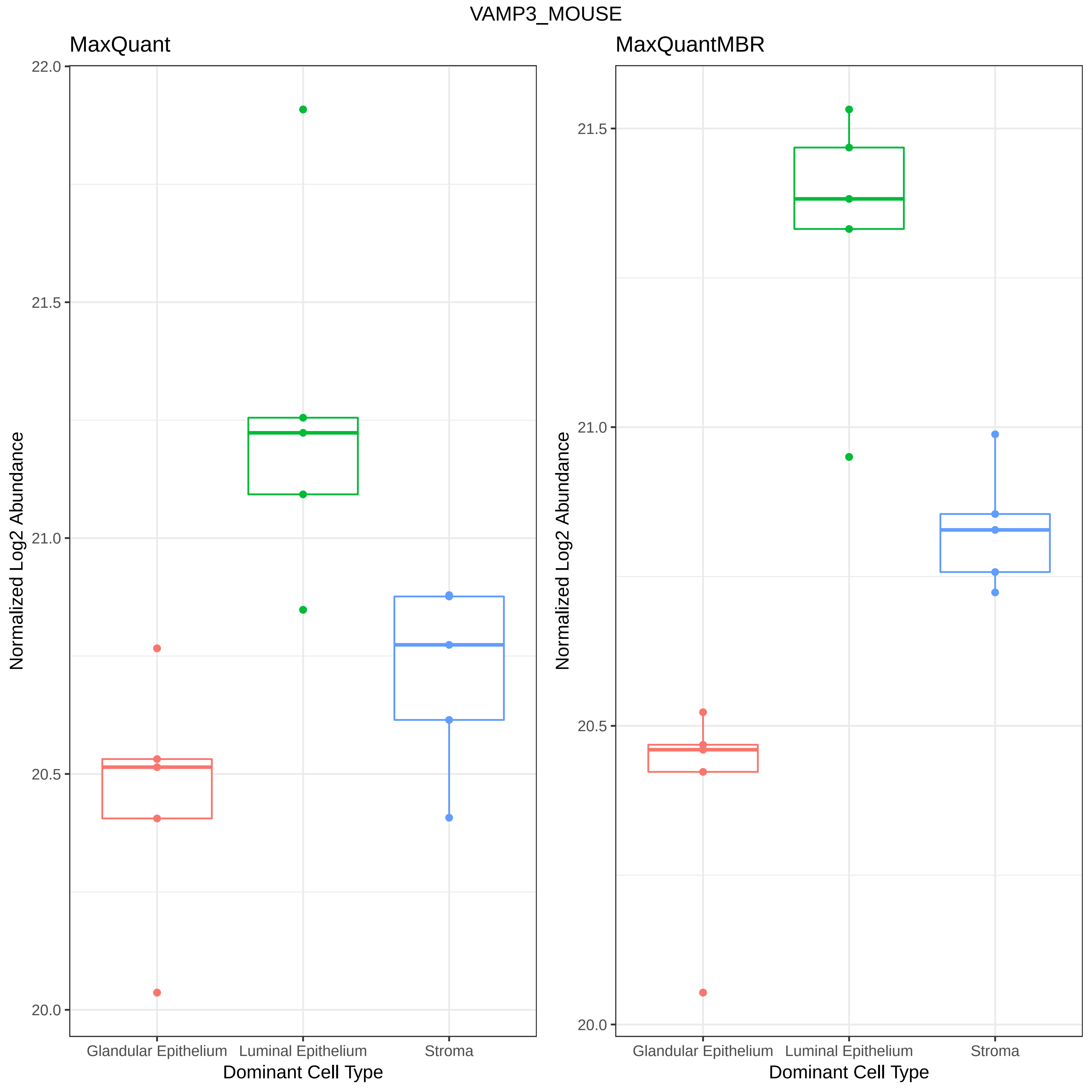
VAMP3_MOUSE
MaxQuant
MaxQuantMBR
Normalized Log2 Abundance
22.0
21.5
21.0
20.5
20.0
Glandular Epithelium
Luminal Epithelium
Stroma
Dominant Cell Type

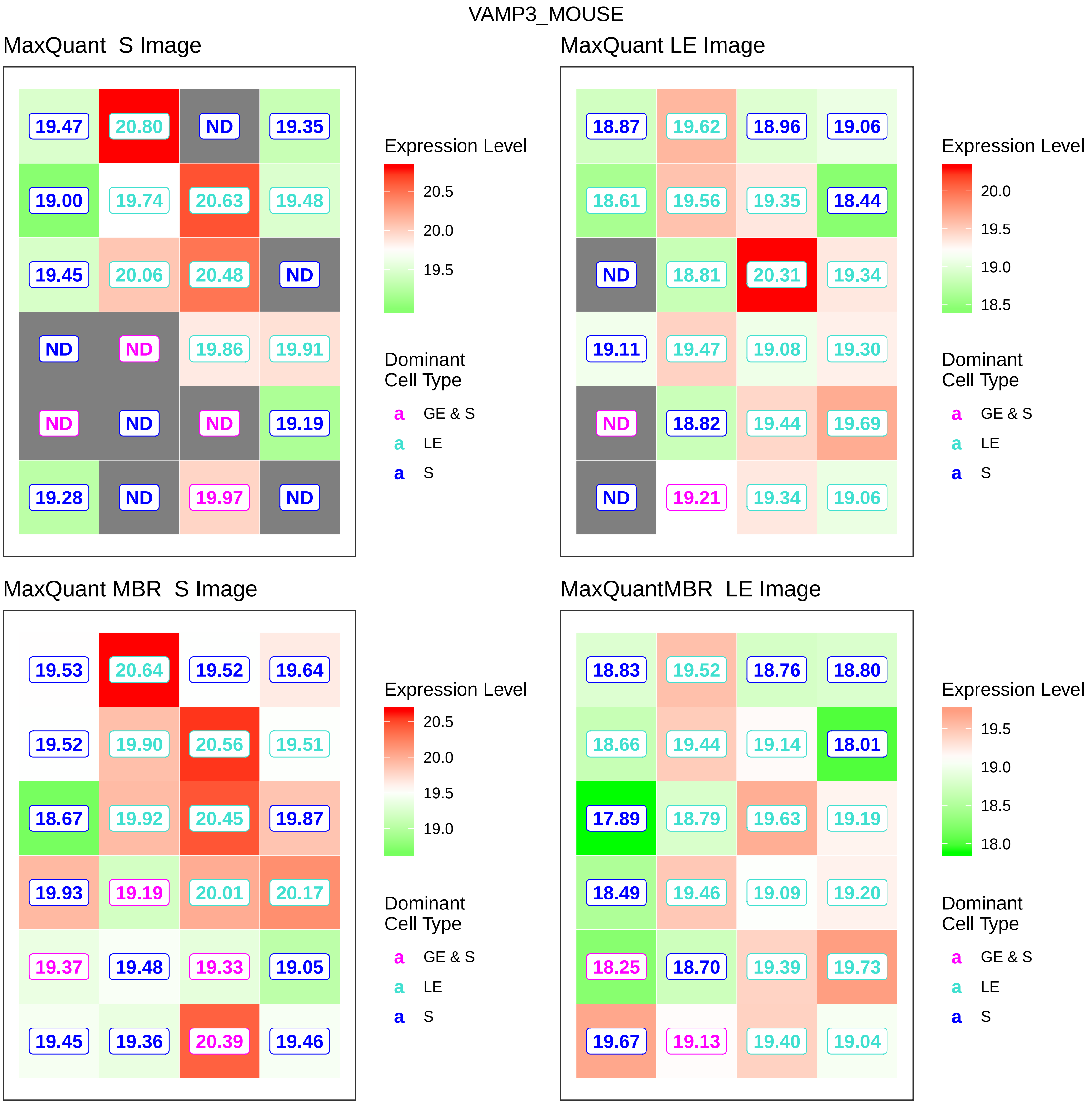
VAMP3_MOUSE
MaxQuant S Image
19.47 20.80 ND 19.35
19.00 19.74 20.63 19.48
19.45 20.06 20.48 ND
ND ND 19.86 19.91
ND ND ND 19.19
19.28 ND 19.97 ND
Expression Level
20.5
20.0
19.5
Dominant Cell Type
a GE & S
a LE
a S
MaxQuant LE Image
18.87 19.62 18.96 19.06
18.61 19.56 19.35 18.44
ND 18.81 20.31 19.34
19.11 19.47 19.08 19.30
ND 18.82 19.44 19.69
ND 19.21 19.34 19.06
Expression Level
20.0
19.5
19.0
18.5
Dominant Cell Type
a GE & S
a LE
a S
MaxQuant MBR S Image
19.53 20.64 19.52 19.64
19.52 19.90 20.56 19.51
18.67 19.92 20.45 19.87
19.93 19.19 20.01 20.17
19.37 19.48 19.33 19.05
19.45 19.36 20.39 19.46
Expression Level
20.5
20.0
19.5
19.0
Dominant Cell Type
a GE & S
a LE
a S
MaxQuantMBR LE Image
18.83 19.52 18.76 18.80
18.66 19.44 19.14 18.01
17.89 18.79 19.63 19.19
18.49 19.46 19.09 19.20
18.25 18.70 19.39 19.73
19.67 19.13 19.40 19.04
Expression Level
19.5
19.0
18.5
18.0
Dominant Cell Type
a GE & S
a LE
a S

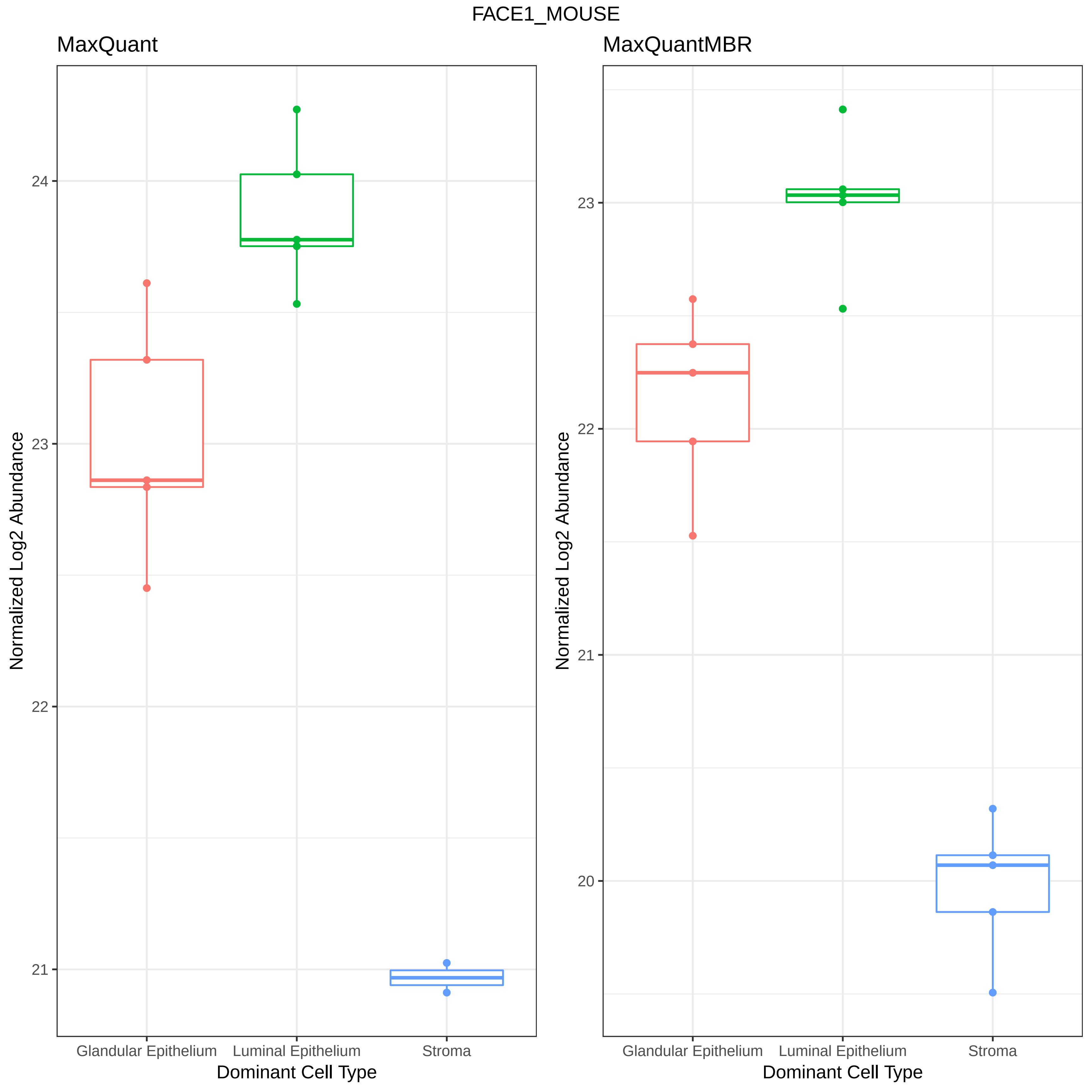

FACE1_MOUSE
MaxQuant
MaxQuantMBR
Normalized Log2 Abundance
24
23
22
21
20
Glandular Epithelium
Luminal Epithelium
Stroma
Dominant Cell Type

FACE1_MOUSE

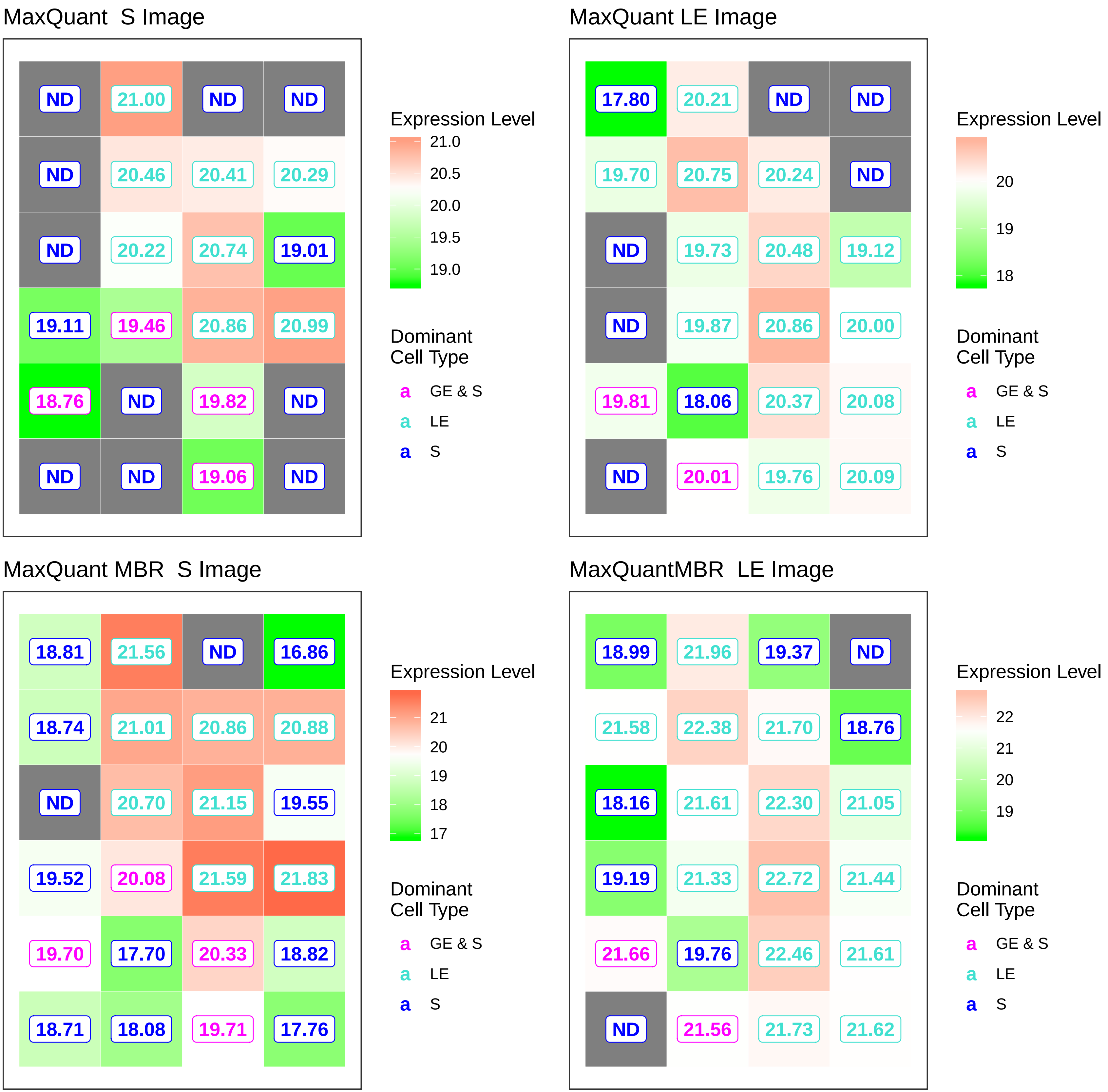
MaxQuant S Image
ND 21.00 ND ND
ND 20.46 20.41 20.29
ND 20.22 20.74 19.01
19.11 19.46 20.86 20.99
18.76 ND 19.82 ND
ND ND 19.06 ND
Expression Level
21.0
20.5
20.0
19.5
19.0
Dominant Cell Type
a GE & S
a LE
a S
MaxQuant LE Image
17.80 20.21 ND ND
19.70 20.75 20.24 ND
ND 19.73 20.48 19.12
ND 19.87 20.86 20.00
19.81 18.06 20.37 20.08
ND 20.01 19.76 20.09
Expression Level
20
19
18
Dominant Cell Type
a GE & S
a LE
a S
MaxQuant MBR S Image
18.81 21.56 ND 16.86
18.74 21.01 20.86 20.88
ND 20.70 21.15 19.55
19.52 20.08 21.59 21.83
19.70 17.70 20.33 18.82
18.71 18.08 19.71 17.76
Expression Level
21
20
19
18
17
Dominant Cell Type
a GE & S
a LE
a S
MaxQuantMBR LE Image
18.99 21.96 19.37 ND
21.58 22.38 21.70 18.76
18.16 21.61 22.30 21.05
19.19 21.33 22.72 21.44
21.66 19.76 22.46 21.61
ND 21.56 21.73 21.62
Expression Level
22
21
20
19
Dominant Cell Type
a GE & S
a LE
a S
