# Supplementary material for: Automated mass spectrometry imaging of over 2000 proteins from tissue sections at 100-μm spatial resolution
Source: Nat Commun. 2020 Jan 7;11:8. doi: 10.1038/s41467-019-13858-z (PMC6946663; doi:10.1038/s41467-019-13858-z)
Supplement: Supplementary file 6 — Supplementary Data 2 [file 41467_2019_13858_MOESM6_ESM.pdf]

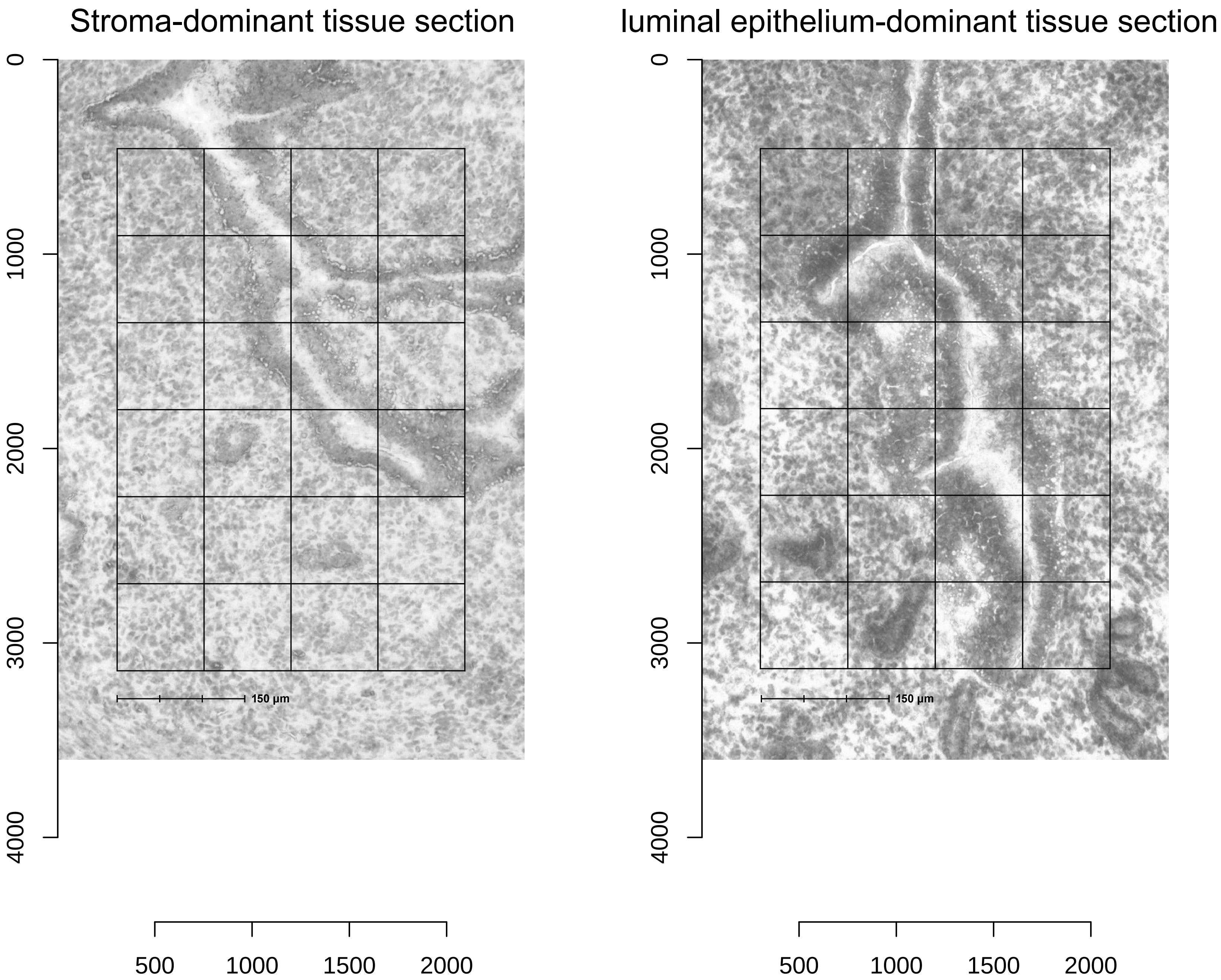

Stroma-dominant tissue section
luminal epithelium-dominant tissue section
0
1000
2000
3000
4000
500
1000
1500
2000
150 µm

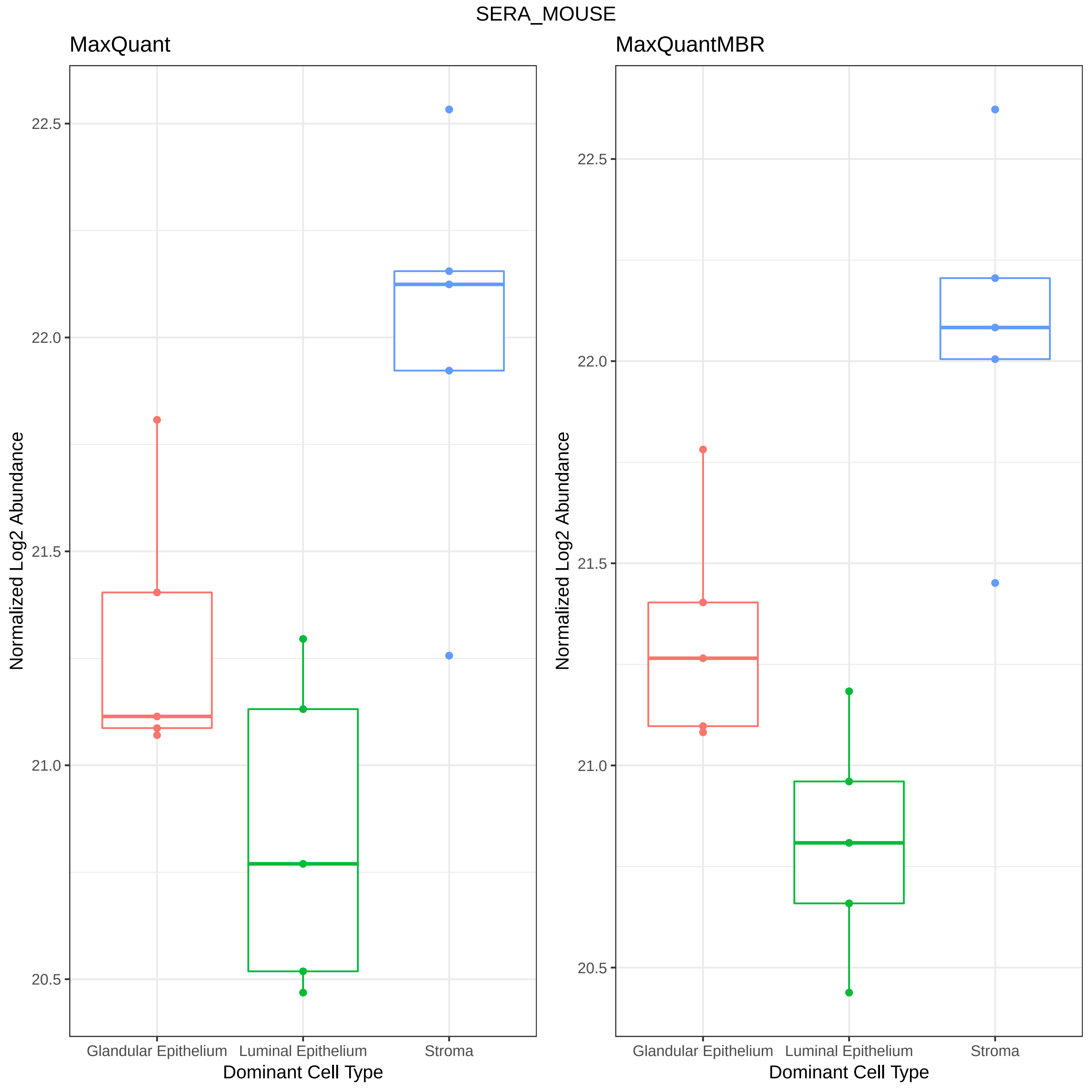
SERA_MOUSE
MaxQuant
MaxQuantMBR
Normalized Log2 Abundance
22.5
22.0
21.5
21.0
20.5
Glandular Epithelium
Luminal Epithelium
Stroma
Dominant Cell Type

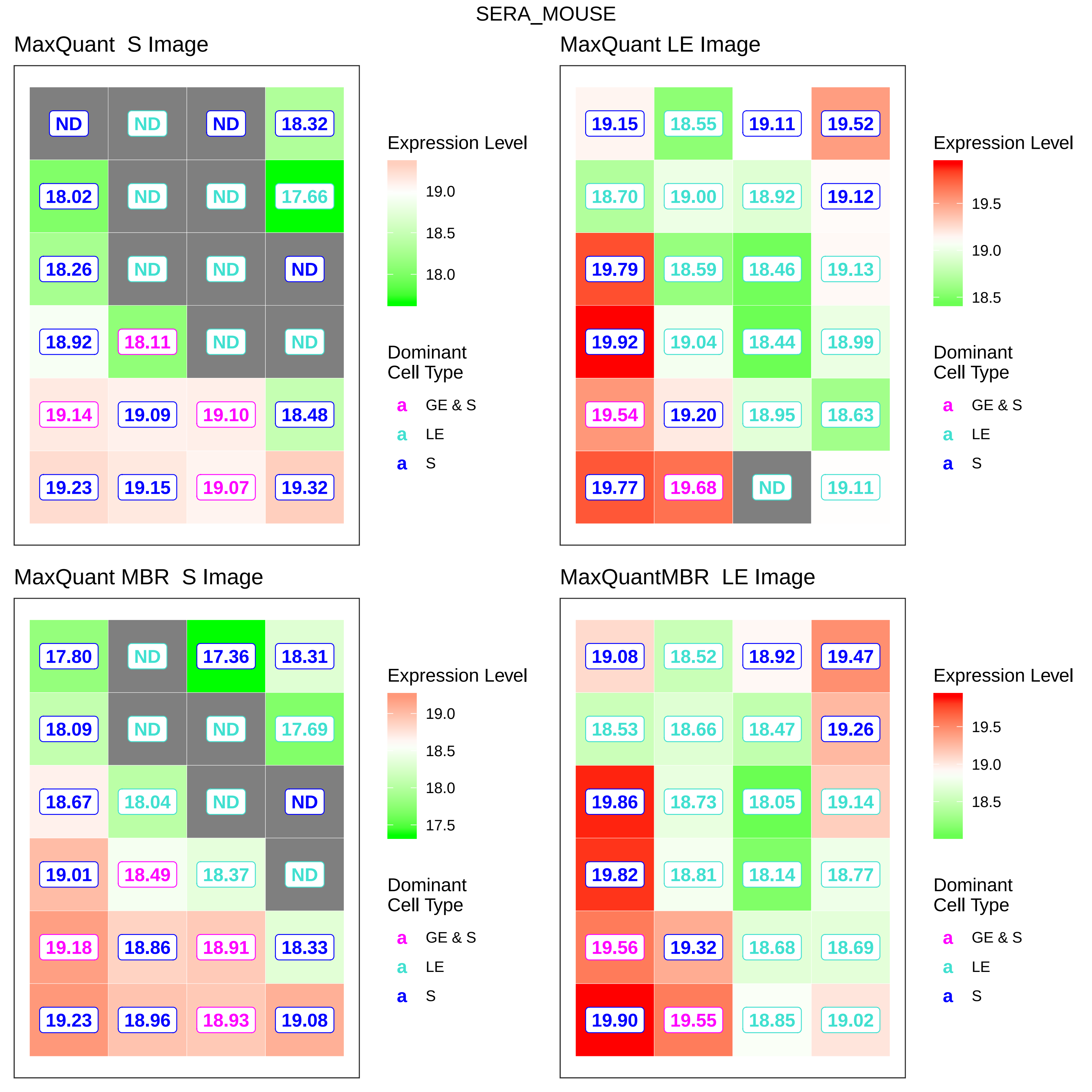
SERA_MOUSE
MaxQuant S Image
ND ND ND 18.32
18.02 ND ND 17.66
18.26 ND ND ND
18.92 18.11 ND ND
19.14 19.09 19.10 18.48
19.23 19.15 19.07 19.32
Expression Level
19.0
18.5
18.0
Dominant Cell Type
a GE & S
a LE
a S
MaxQuant LE Image
19.15 18.55 19.11 19.52
18.70 19.00 18.92 19.12
19.79 18.59 18.46 19.13
19.92 19.04 18.44 18.99
19.54 19.20 18.95 18.63
19.77 19.68 ND 19.11
Expression Level
19.5
19.0
18.5
Dominant Cell Type
a GE & S
a LE
a S
MaxQuant MBR S Image
17.80 ND 17.36 18.31
18.09 ND ND 17.69
18.67 18.04 ND ND
19.01 18.49 18.37 ND
19.18 18.86 18.91 18.33
19.23 18.96 18.93 19.08
Expression Level
19.0
18.5
18.0
17.5
Dominant Cell Type
a GE & S
a LE
a S
MaxQuantMBR LE Image
19.08 18.52 18.92 19.47
18.53 18.66 18.47 19.26
19.86 18.73 18.05 19.14
19.82 18.81 18.14 18.77
19.56 19.32 18.68 18.69
19.90 19.55 18.85 19.02
Expression Level
19.5
19.0
18.5
Dominant Cell Type
a GE & S
a LE
a S

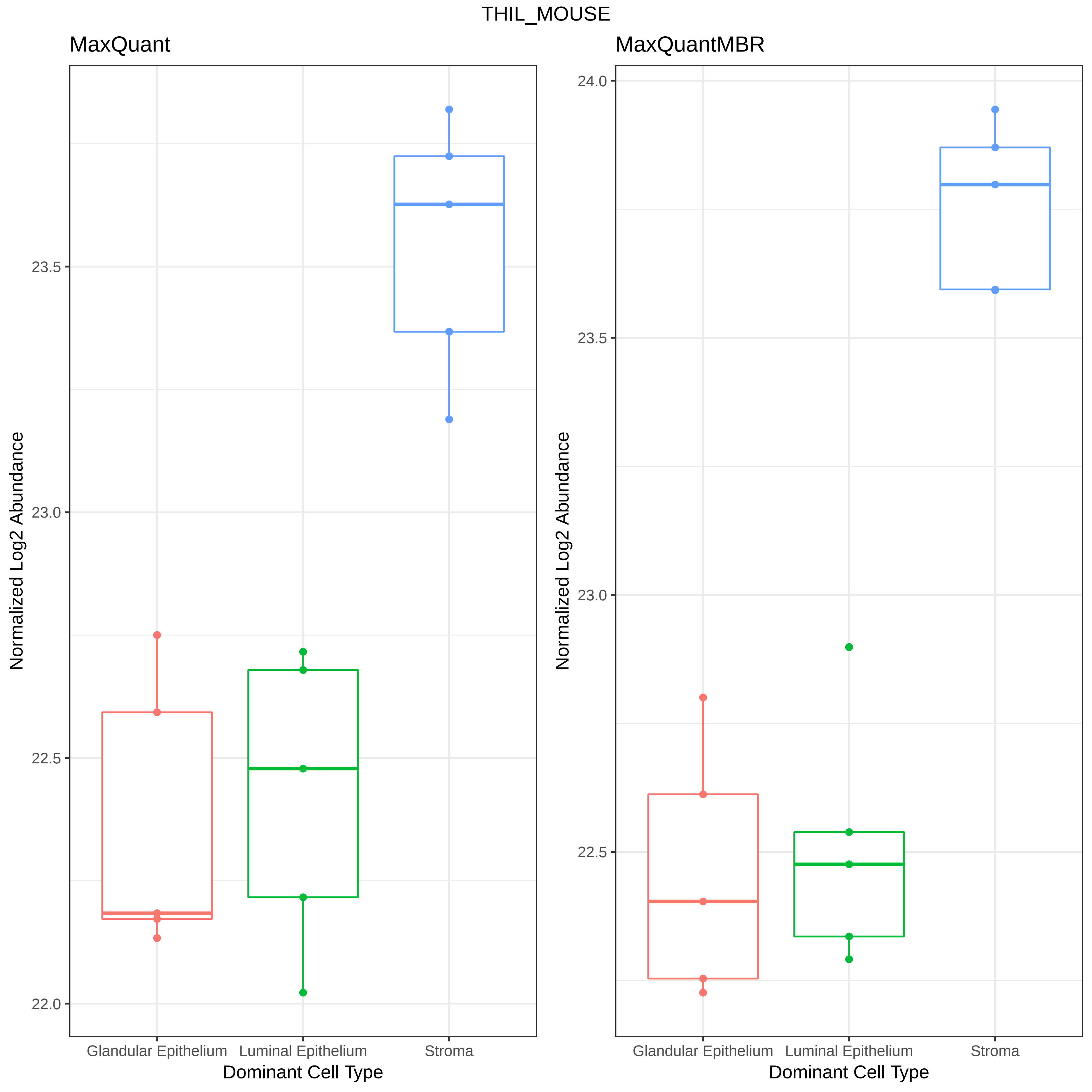
THIL_MOUSE
MaxQuant
MaxQuantMBR
Normalized Log2 Abundance
23.5
23.0
22.5
22.0
24.0
23.5
23.0
22.5
Glandular Epithelium
Luminal Epithelium
Stroma
Glandular Epithelium
Luminal Epithelium
Stroma
Dominant Cell Type
Dominant Cell Type

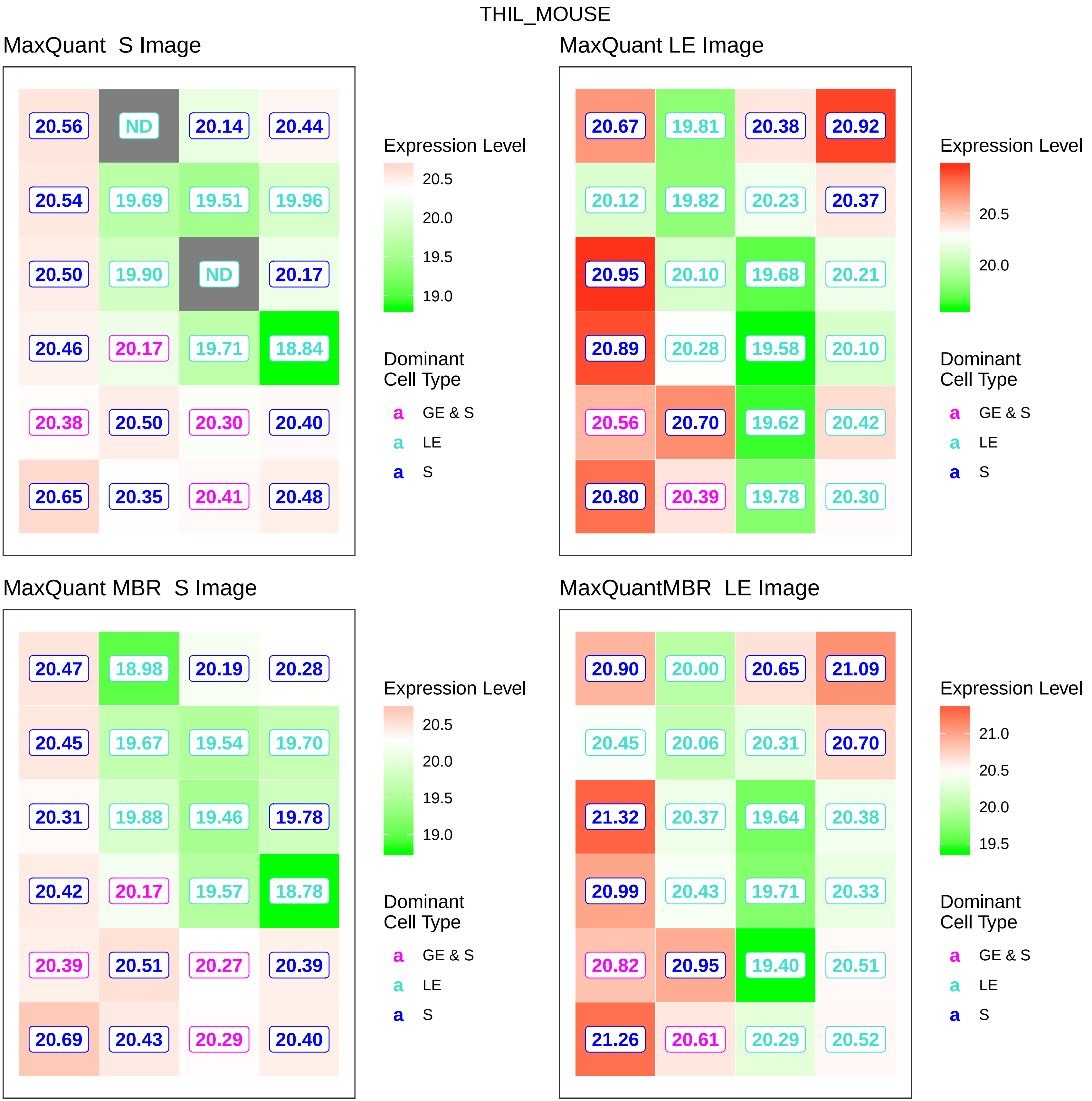
THIL_MOUSE
MaxQuant S Image
20.56 ND 20.14 20.44
20.54 19.69 19.51 19.96
20.50 19.90 ND 20.17
20.46 20.17 19.71 18.84
20.38 20.50 20.30 20.40
20.65 20.35 20.41 20.48
Expression Level
20.5
20.0
19.5
19.0
Dominant Cell Type
a GE & S
a LE
a S
MaxQuant LE Image
20.67 19.81 20.38 20.92
20.12 19.82 20.23 20.37
20.95 20.10 19.68 20.21
20.89 20.28 19.58 20.10
20.56 20.70 19.62 20.42
20.80 20.39 19.78 20.30
Expression Level
20.5
20.0
Dominant Cell Type
a GE & S
a LE
a S
MaxQuant MBR S Image
20.47 18.98 20.19 20.28
20.45 19.67 19.54 19.70
20.31 19.88 19.46 19.78
20.42 20.17 19.57 18.78
20.39 20.51 20.27 20.39
20.69 20.43 20.29 20.40
Expression Level
20.5
20.0
19.5
19.0
Dominant Cell Type
a GE & S
a LE
a S
MaxQuantMBR LE Image
20.90 20.00 20.65 21.09
20.45 20.06 20.31 20.70
21.32 20.37 19.64 20.38
20.99 20.43 19.71 20.33
20.82 20.95 19.40 20.51
21.26 20.61 20.29 20.52
Expression Level
21.0
20.5
20.0
19.5
Dominant Cell Type
a GE & S
a LE
a S

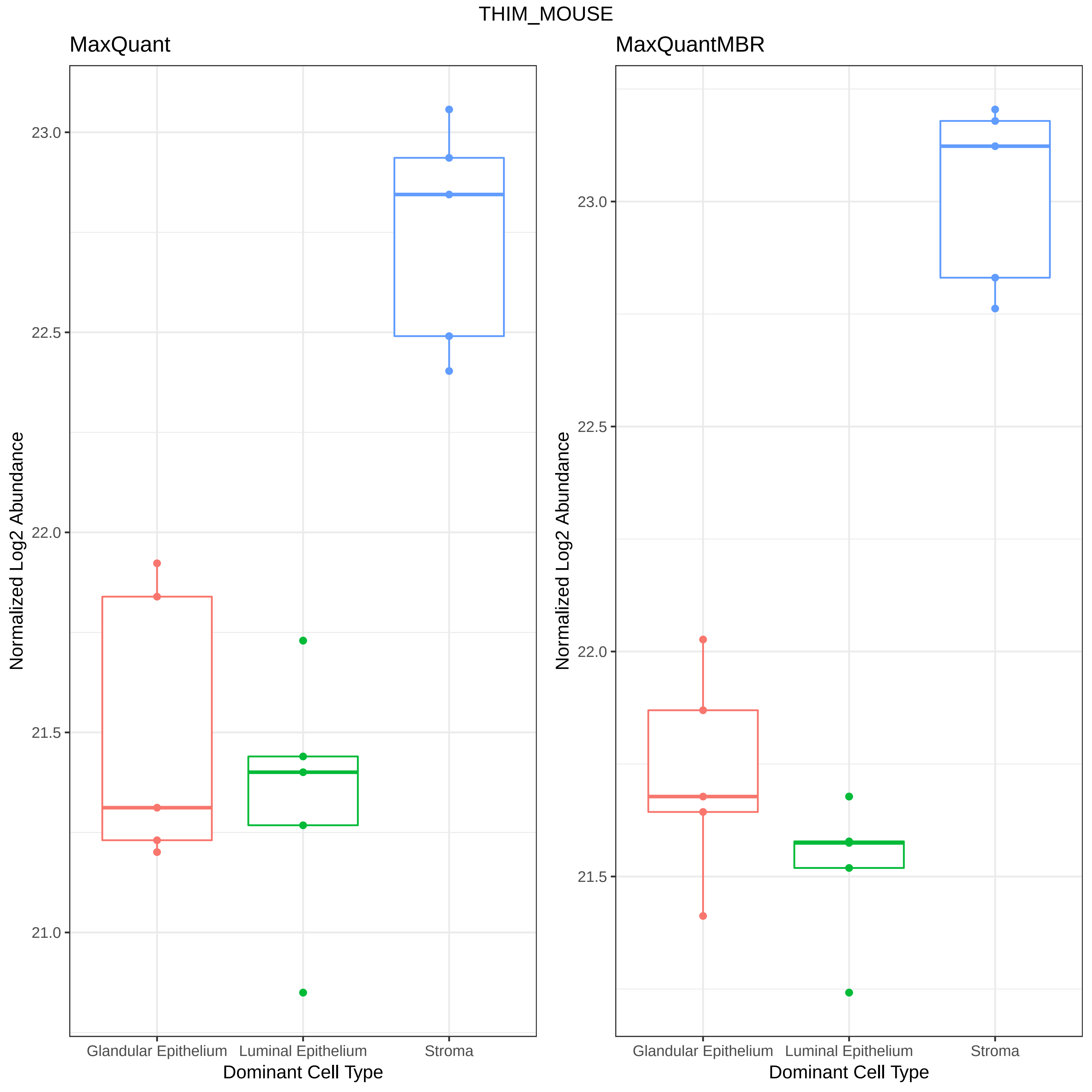

THIM_MOUSE
MaxQuant
MaxQuantMBR
Normalized Log2 Abundance
23.0
22.5
22.0
21.5
21.0
Glandular Epithelium
Luminal Epithelium
Stroma
Dominant Cell Type

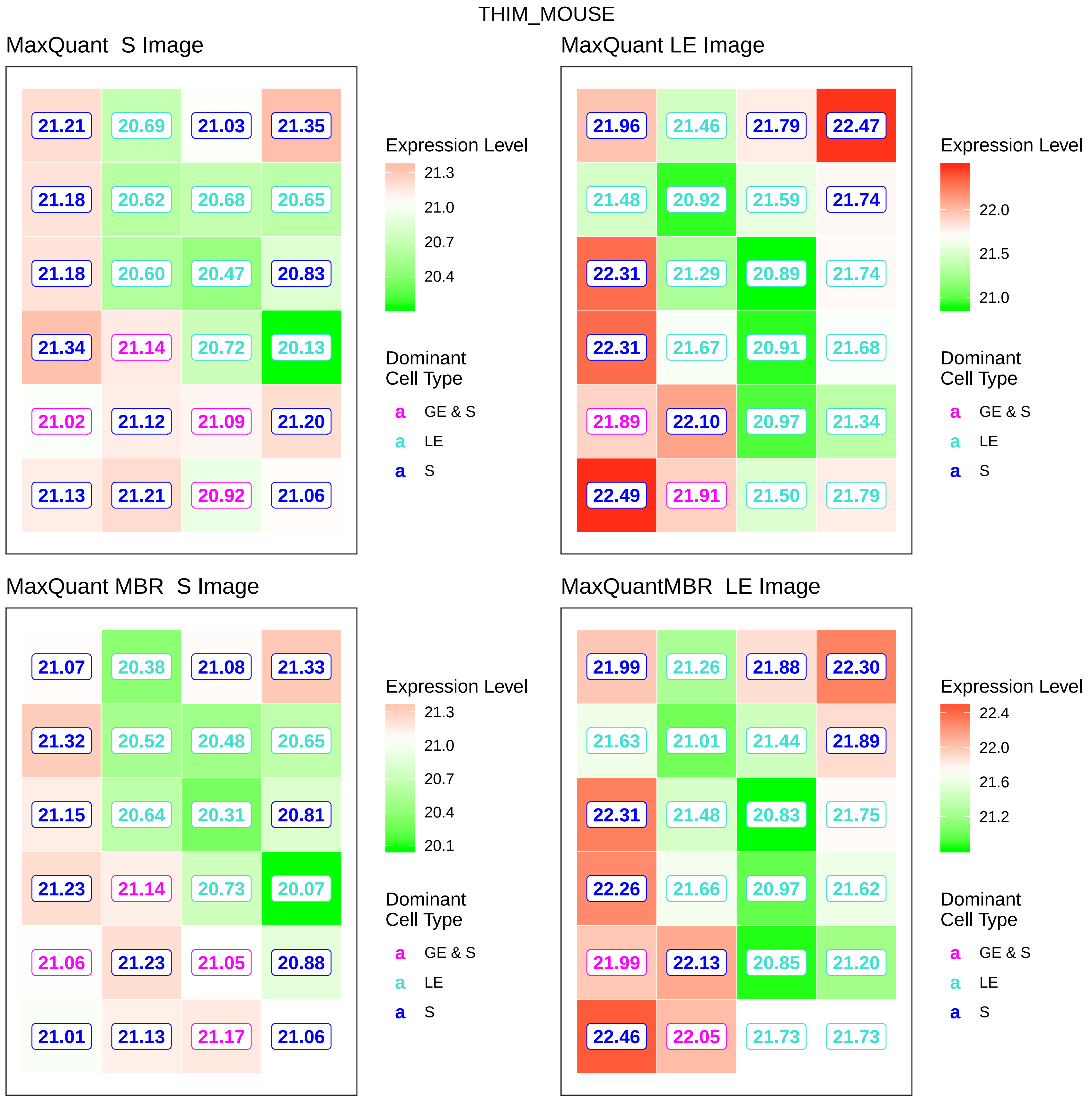
THIM_MOUSE
MaxQuant S Image
21.21 20.69 21.03 21.35
21.18 20.62 20.68 20.65
21.18 20.60 20.47 20.83
21.34 21.14 20.72 20.13
21.02 21.12 21.09 21.20
21.13 21.21 20.92 21.06
Expression Level
21.3
21.0
20.7
20.4
Dominant Cell Type
a GE & S
a LE
a S
MaxQuant LE Image
21.96 21.46 21.79 22.47
21.48 20.92 21.59 21.74
22.31 21.29 20.89 21.74
22.31 21.67 20.91 21.68
21.89 22.10 20.97 21.34
22.49 21.91 21.50 21.79
Expression Level
22.0
21.5
21.0
Dominant Cell Type
a GE & S
a LE
a S
MaxQuant MBR S Image
21.07 20.38 21.08 21.33
21.32 20.52 20.48 20.65
21.15 20.64 20.31 20.81
21.23 21.14 20.73 20.07
21.06 21.23 21.05 20.88
21.01 21.13 21.17 21.06
Expression Level
21.3
21.0
20.7
20.4
20.1
Dominant Cell Type
a GE & S
a LE
a S
MaxQuantMBR LE Image
21.99 21.26 21.88 22.30
21.63 21.01 21.44 21.89
22.31 21.48 20.83 21.75
22.26 21.66 20.97 21.62
21.99 22.13 20.85 21.20
22.46 22.05 21.73 21.73
Expression Level
22.4
22.0
21.6
21.2
Dominant Cell Type
a GE & S
a LE
a S

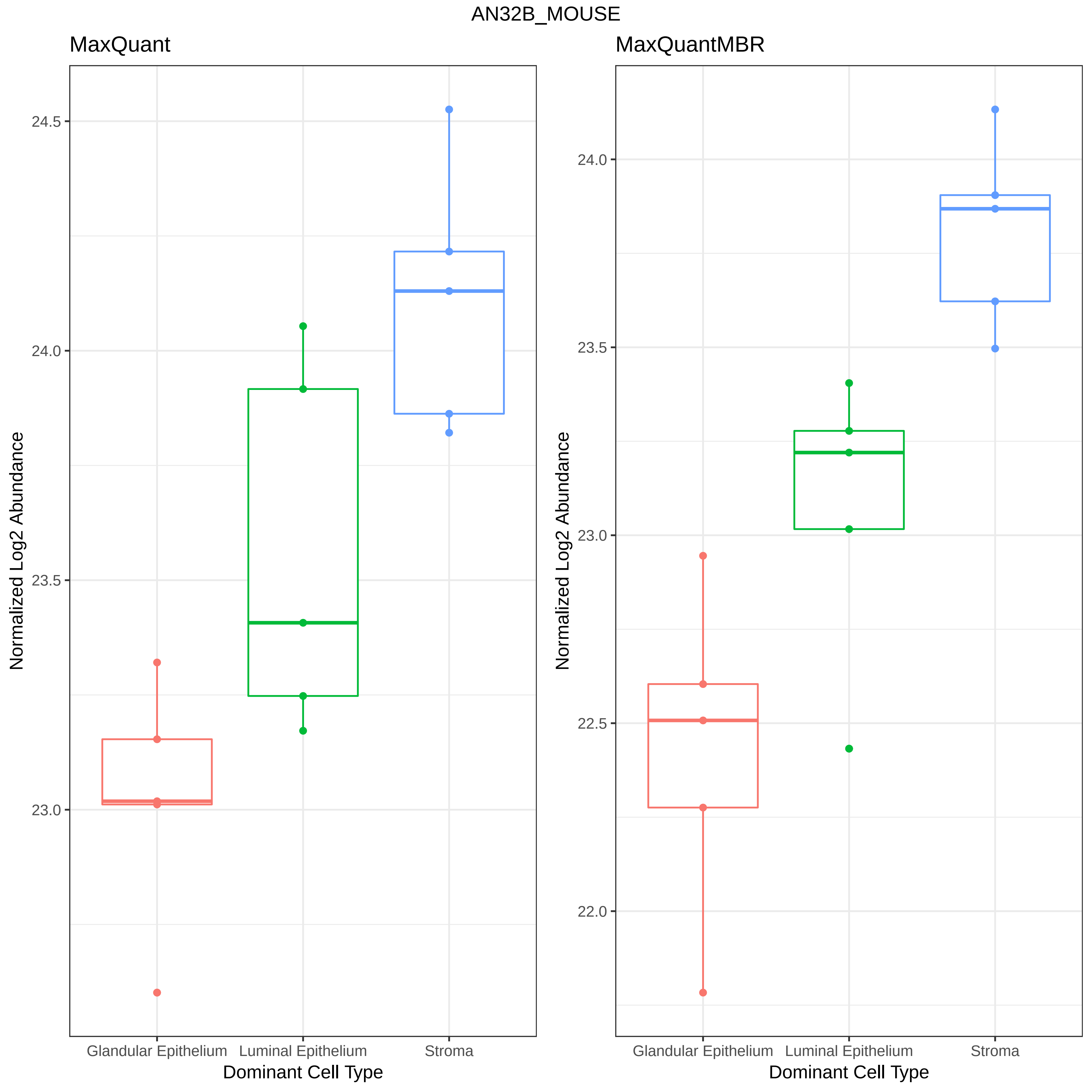

AN32B_MOUSE
MaxQuant
MaxQuantMBR
Normalized Log2 Abundance
24.5
24.0
23.5
23.0
22.5
22.0
Glandular Epithelium
Luminal Epithelium
Stroma
Dominant Cell Type

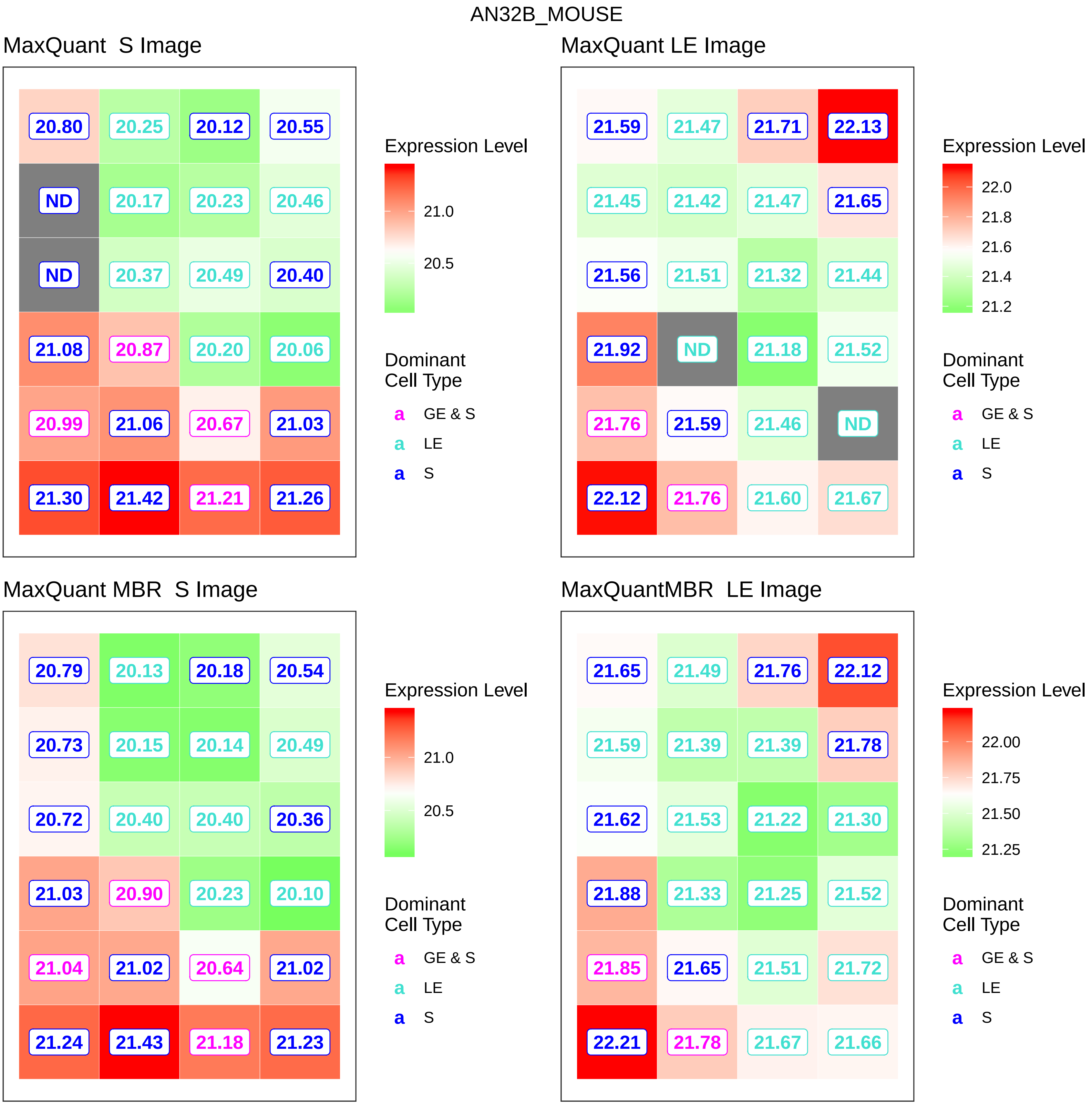
AN32B_MOUSE
MaxQuant S Image
20.80 20.25 20.12 20.55
ND 20.17 20.23 20.46
ND 20.37 20.49 20.40
21.08 20.87 20.20 20.06
20.99 21.06 20.67 21.03
21.30 21.42 21.21 21.26
Expression Level
21.0
20.5
Dominant Cell Type
a GE & S
a LE
a S
MaxQuant LE Image
21.59 21.47 21.71 22.13
21.45 21.42 21.47 21.65
21.56 21.51 21.32 21.44
21.92 ND 21.18 21.52
21.76 21.59 21.46 ND
22.12 21.76 21.60 21.67
Expression Level
22.0
21.8
21.6
21.4
21.2
Dominant Cell Type
a GE & S
a LE
a S
MaxQuant MBR S Image
20.79 20.13 20.18 20.54
20.73 20.15 20.14 20.49
20.72 20.40 20.40 20.36
21.03 20.90 20.23 20.10
21.04 21.02 20.64 21.02
21.24 21.43 21.18 21.23
Expression Level
21.0
20.5
Dominant Cell Type
a GE & S
a LE
a S
MaxQuantMBR LE Image
21.65 21.49 21.76 22.12
21.59 21.39 21.39 21.78
21.62 21.53 21.22 21.30
21.88 21.33 21.25 21.52
21.85 21.65 21.51 21.72
22.21 21.78 21.67 21.66
Expression Level
22.00
21.75
21.50
21.25
Dominant Cell Type
a GE & S
a LE
a S

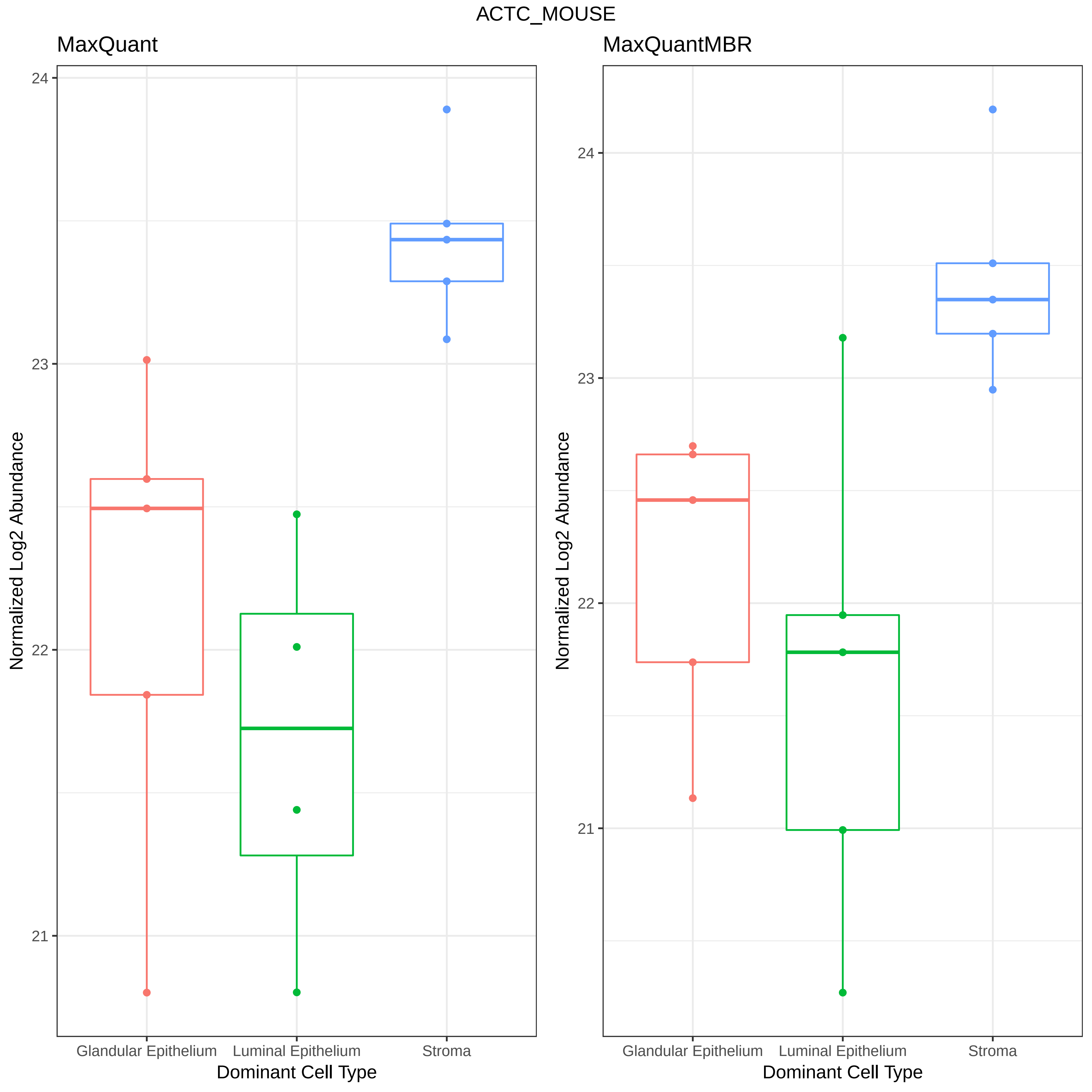
ACTC_MOUSE
MaxQuant
MaxQuantMBR
Normalized Log2 Abundance
24
23
22
21
Glandular Epithelium
Luminal Epithelium
Stroma
Dominant Cell Type

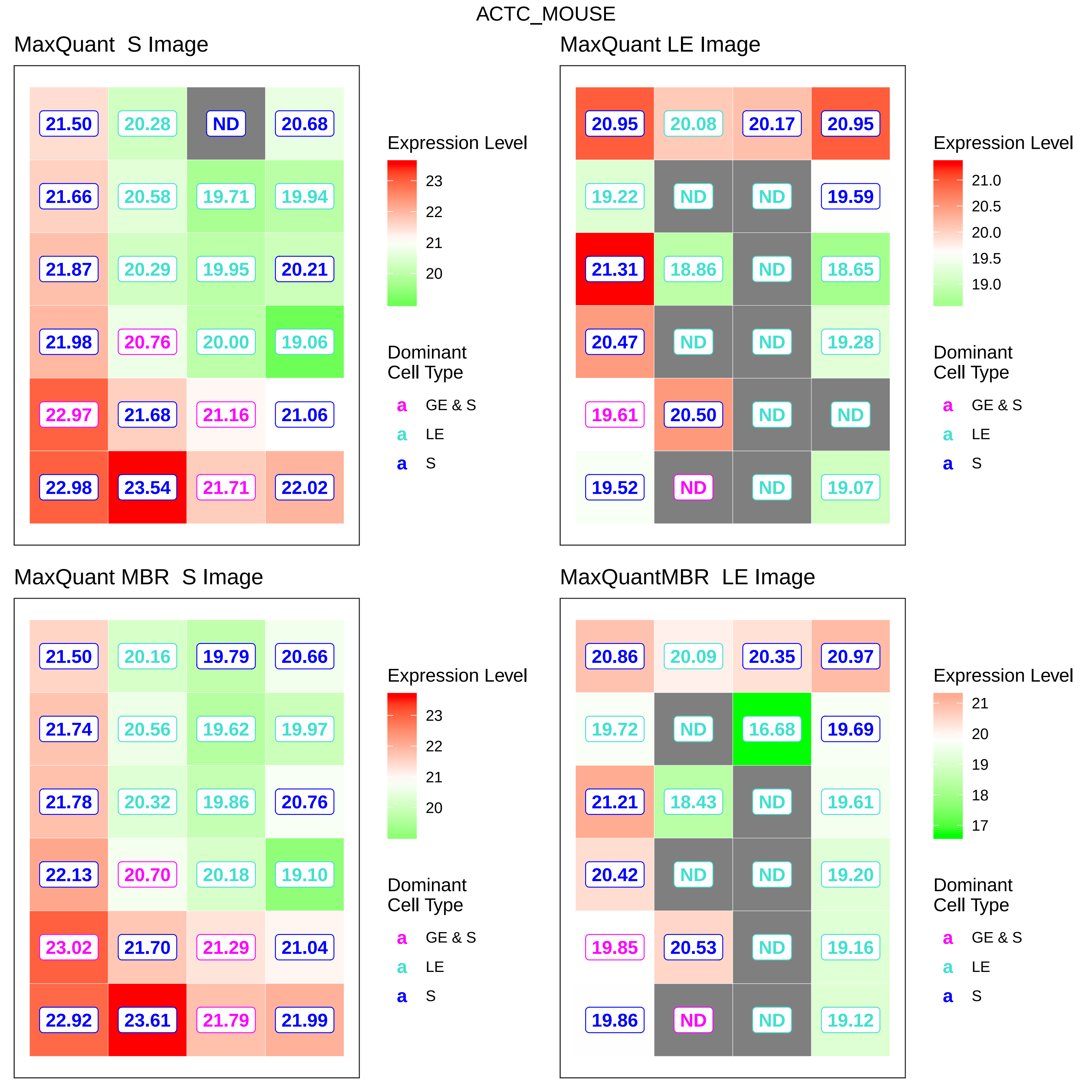
ACTC_MOUSE
MaxQuant S Image
21.50 20.28 ND 20.68
21.66 20.58 19.71 19.94
21.87 20.29 19.95 20.21
21.98 20.76 20.00 19.06
22.97 21.68 21.16 21.06
22.98 23.54 21.71 22.02
Expression Level
23
22
21
20
Dominant Cell Type
a GE & S
a LE
a S
MaxQuant LE Image
20.95 20.08 20.17 20.95
19.22 ND ND 19.59
21.31 18.86 ND 18.65
20.47 ND ND 19.28
19.61 20.50 ND ND
19.52 ND ND 19.07
Expression Level
21.0
20.5
20.0
19.5
19.0
Dominant Cell Type
a GE & S
a LE
a S
MaxQuant MBR S Image
21.50 20.16 19.79 20.66
21.74 20.56 19.62 19.97
21.78 20.32 19.86 20.76
22.13 20.70 20.18 19.10
23.02 21.70 21.29 21.04
22.92 23.61 21.79 21.99
Expression Level
23
22
21
20
Dominant Cell Type
a GE & S
a LE
a S
MaxQuantMBR LE Image
20.86 20.09 20.35 20.97
19.72 ND 16.68 19.69
21.21 18.43 ND 19.61
20.42 ND ND 19.20
19.85 20.53 ND 19.16
19.86 ND ND 19.12
Expression Level
21
20
19
18
17
Dominant Cell Type
a GE & S
a LE
a S

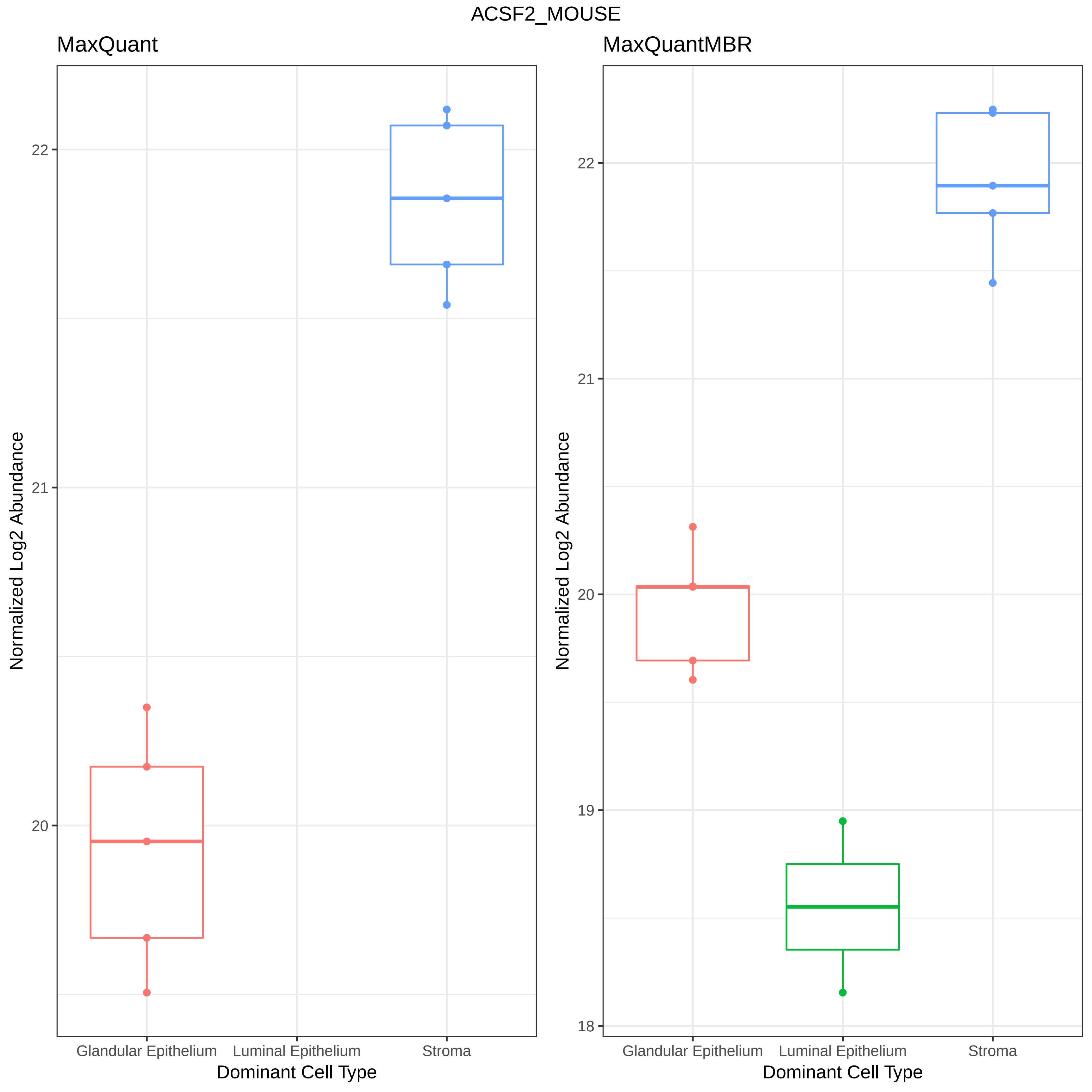
ACSF2_MOUSE
MaxQuant
MaxQuantMBR
Normalized Log2 Abundance
22
21
20
19
18
Glandular Epithelium
Luminal Epithelium
Stroma
Dominant Cell Type

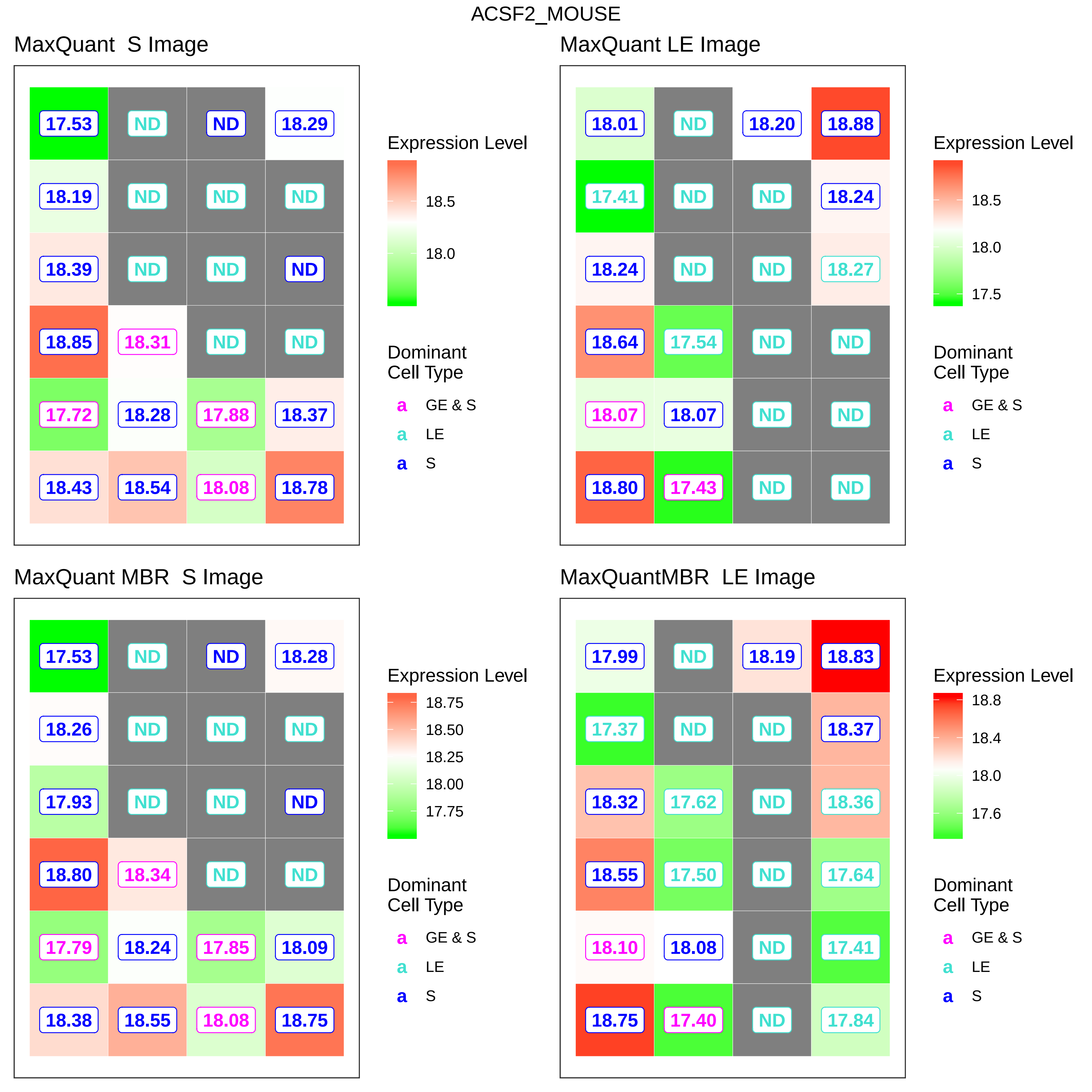

ACSF2_MOUSE
MaxQuant S Image
17.53
ND
ND
18.29
18.19
ND
ND
ND
18.39
ND
ND
ND
18.85
18.31
ND
ND
17.72
18.28
17.88
18.37
18.43
18.54
18.08
18.78
Expression Level
18.5
18.0
Dominant Cell Type
a GE & S
a LE
a S
MaxQuant LE Image
18.01
ND
18.20
18.88
17.41
ND
ND
18.24
18.24
ND
ND
18.27
18.64
17.54
ND
ND
18.07
18.07
ND
ND
18.80
17.43
ND
ND
Expression Level
18.5
18.0
17.5
Dominant Cell Type
a GE & S
a LE
a S
MaxQuant MBR S Image
17.53
ND
ND
18.28
18.26
ND
ND
ND
17.93
ND
ND
ND
18.80
18.34
ND
ND
17.79
18.24
17.85
18.09
18.38
18.55
18.08
18.75
Expression Level
18.75
18.50
18.25
18.00
17.75
Dominant Cell Type
a GE & S
a LE
a S
MaxQuantMBR LE Image
17.99
ND
18.19
18.83
17.37
ND
ND
18.37
18.32
17.62
ND
18.36
18.55
17.50
ND
17.64
18.10
18.08
ND
17.41
18.75
17.40
ND
17.84
Expression Level
18.8
18.4
18.0
17.6
Dominant Cell Type
a GE & S
a LE
a S

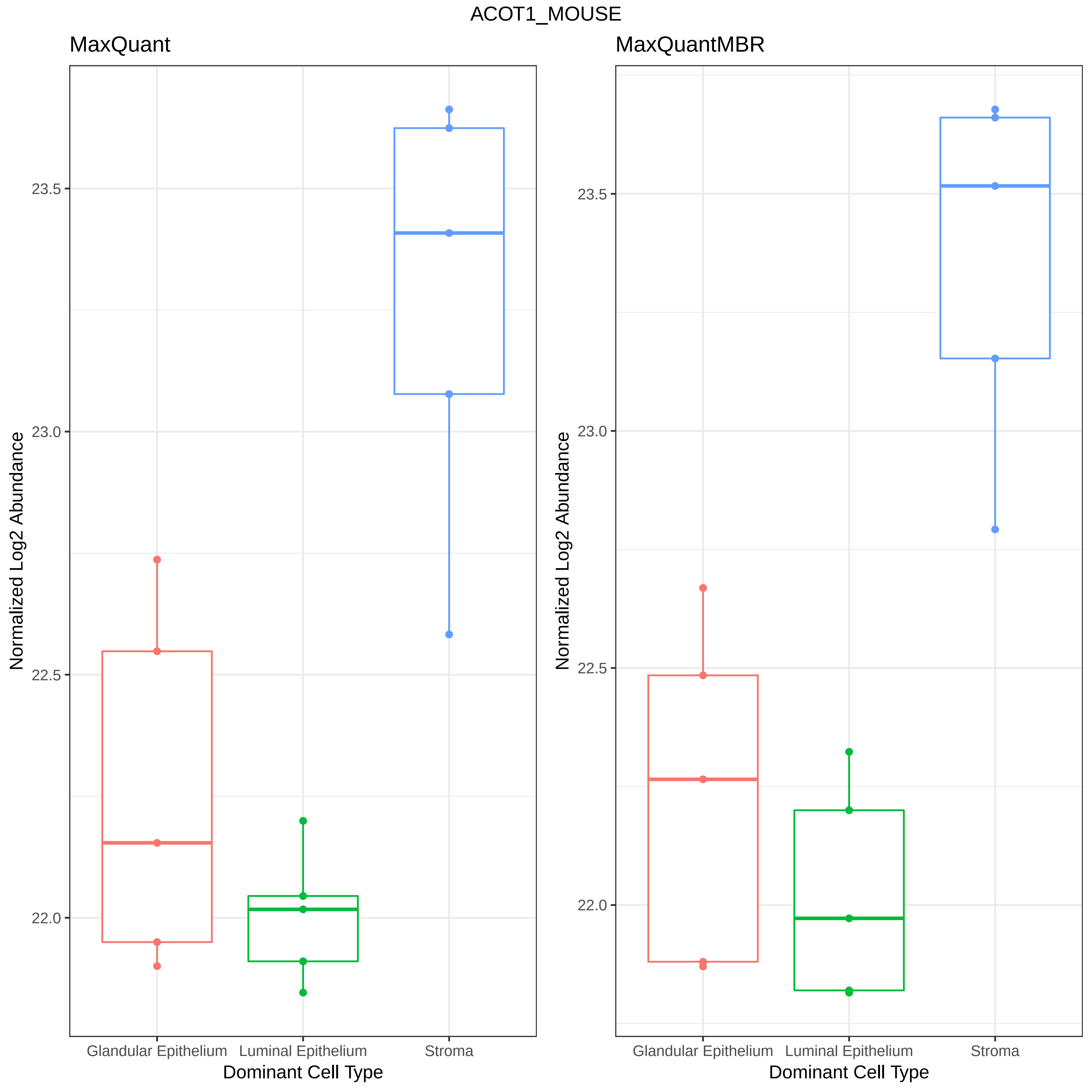

ACOT1_MOUSE
MaxQuant
MaxQuantMBR
Normalized Log2 Abundance
23.5
23.0
22.5
22.0
Glandular Epithelium
Luminal Epithelium
Stroma
Dominant Cell Type

ACOT1_MOUSE

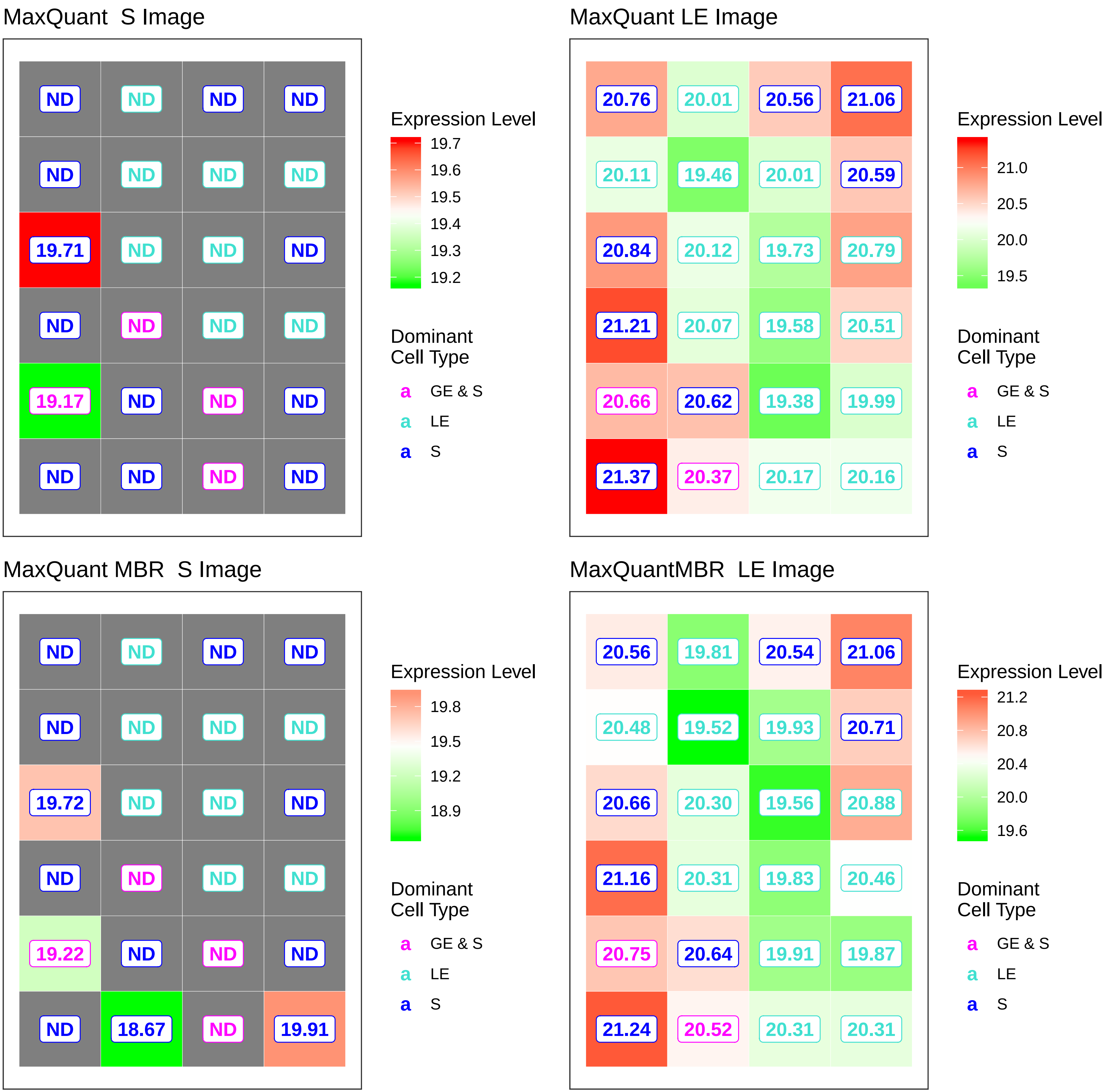
MaxQuant S Image
ND ND ND ND
ND ND ND ND
19.71 ND ND ND
ND ND ND ND
19.17 ND ND ND
ND ND ND ND
Expression Level
19.7
19.6
19.5
19.4
19.3
19.2
Dominant Cell Type
a GE & S
a LE
a S
MaxQuant LE Image
20.76 20.01 20.56 21.06
20.11 19.46 20.01 20.59
20.84 20.12 19.73 20.79
21.21 20.07 19.58 20.51
20.66 20.62 19.38 19.99
21.37 20.37 20.17 20.16
Expression Level
21.0
20.5
20.0
19.5
Dominant Cell Type
a GE & S
a LE
a S
MaxQuant MBR S Image
ND ND ND ND
ND ND ND ND
19.72 ND ND ND
ND ND ND ND
19.22 ND ND ND
ND 18.67 ND 19.91
Expression Level
19.8
19.5
19.2
18.9
Dominant Cell Type
a GE & S
a LE
a S
MaxQuantMBR LE Image
20.56 19.81 20.54 21.06
20.48 19.52 19.93 20.71
20.66 20.30 19.56 20.88
21.16 20.31 19.83 20.46
20.75 20.64 19.91 19.87
21.24 20.52 20.31 20.31
Expression Level
21.2
20.8
20.4
20.0
19.6
Dominant Cell Type
a GE & S
a LE
a S

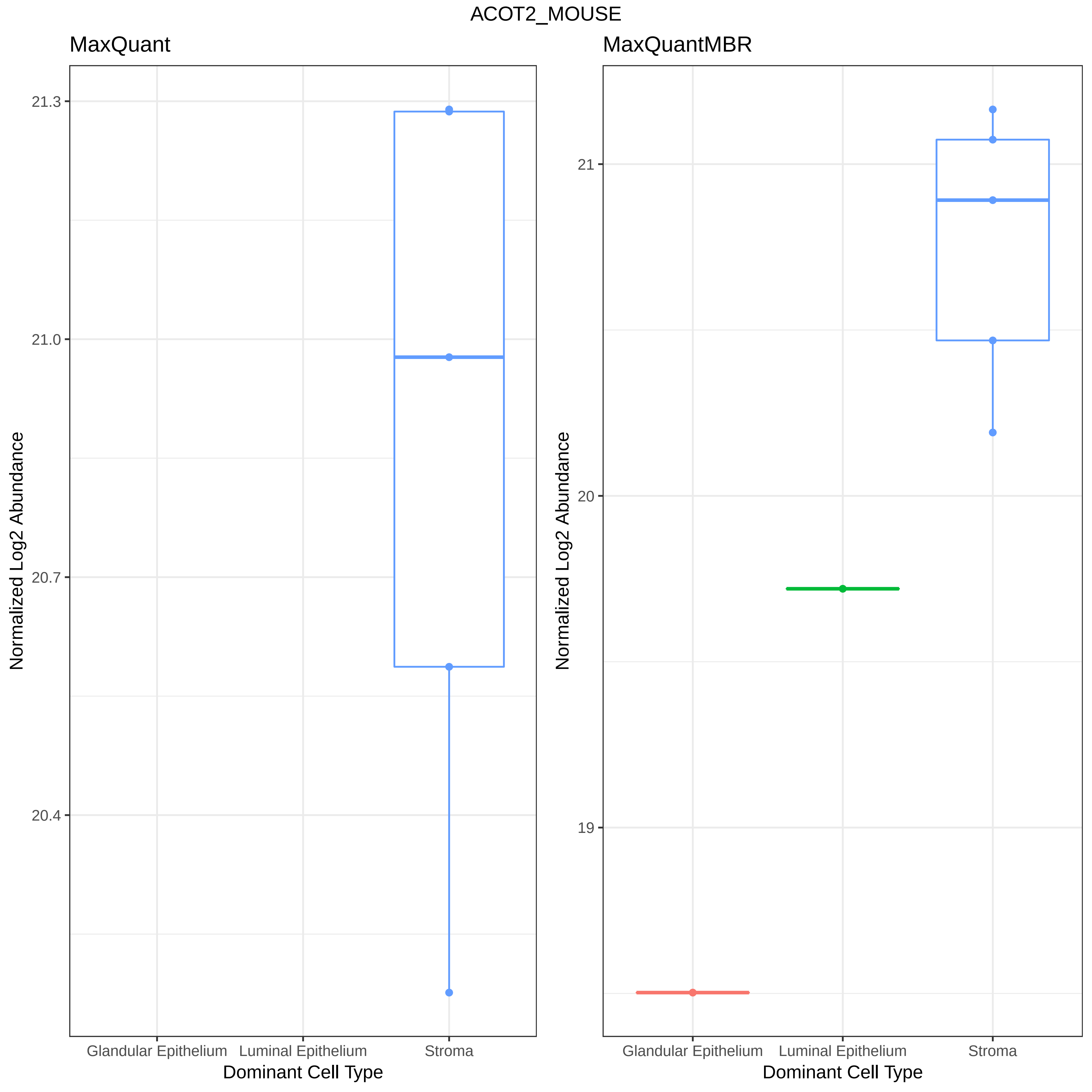
ACOT2_MOUSE
MaxQuant
MaxQuantMBR
Normalized Log2 Abundance
21.3
21.0
20.7
20.4
21
20
19
Glandular Epithelium
Luminal Epithelium
Stroma
Dominant Cell Type

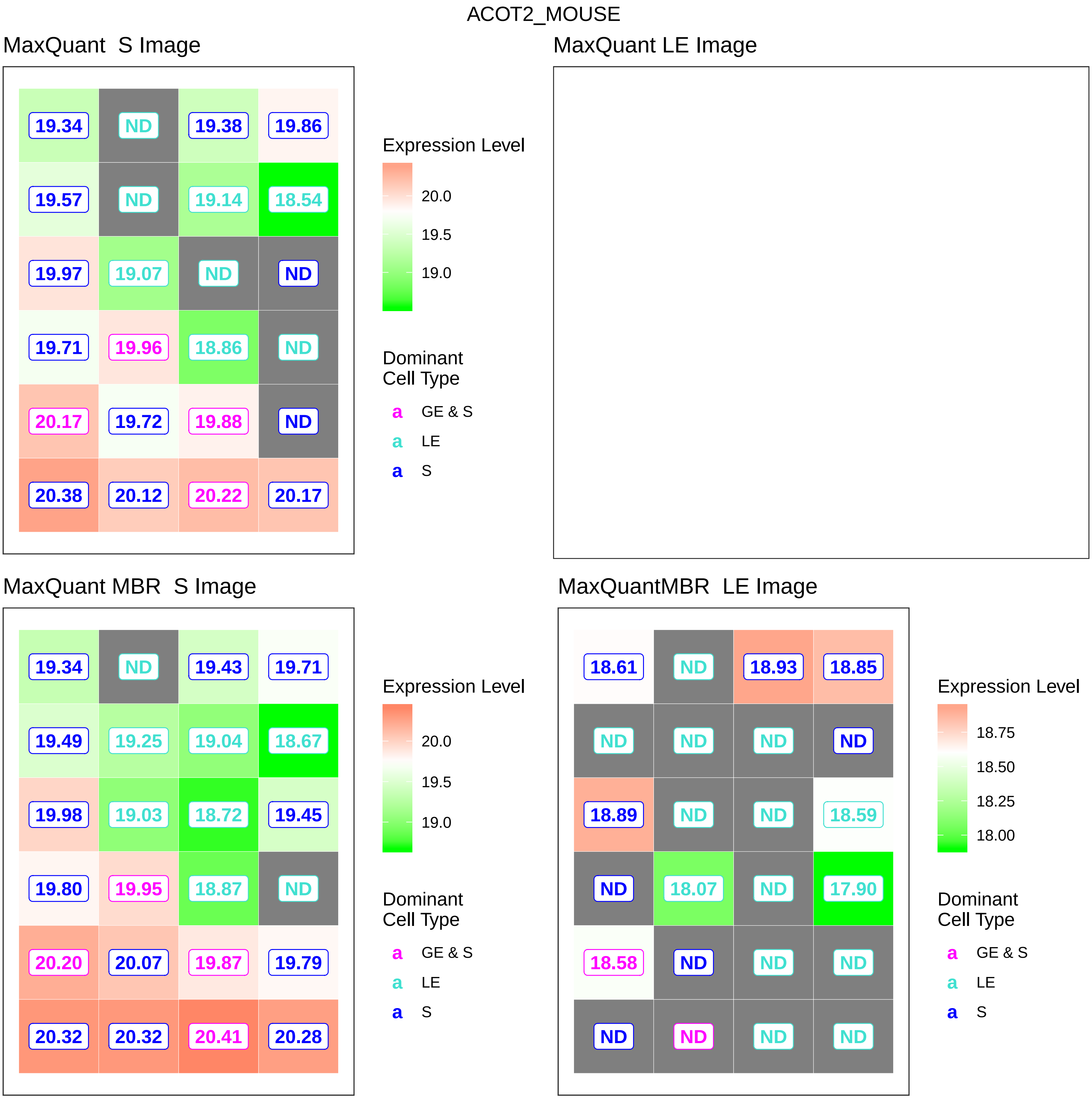
ACOT2_MOUSE
MaxQuant S Image
19.34 ND 19.38 19.86
19.57 ND 19.14 18.54
19.97 19.07 ND ND
19.71 19.96 18.86 ND
20.17 19.72 19.88 ND
20.38 20.12 20.22 20.17
Expression Level
20.0
19.5
19.0
Dominant Cell Type
a GE & S
a LE
a S
MaxQuant LE Image
MaxQuant MBR S Image
19.34 ND 19.43 19.71
19.49 19.25 19.04 18.67
19.98 19.03 18.72 19.45
19.80 19.95 18.87 ND
20.20 20.07 19.87 19.79
20.32 20.32 20.41 20.28
Expression Level
20.0
19.5
19.0
Dominant Cell Type
a GE & S
a LE
a S
MaxQuantMBR LE Image
18.61 ND 18.93 18.85
ND ND ND ND
18.89 ND ND 18.59
ND 18.07 ND 17.90
18.58 ND ND ND
ND ND ND ND
Expression Level
18.75
18.50
18.25
18.00
Dominant Cell Type
a GE & S
a LE
a S

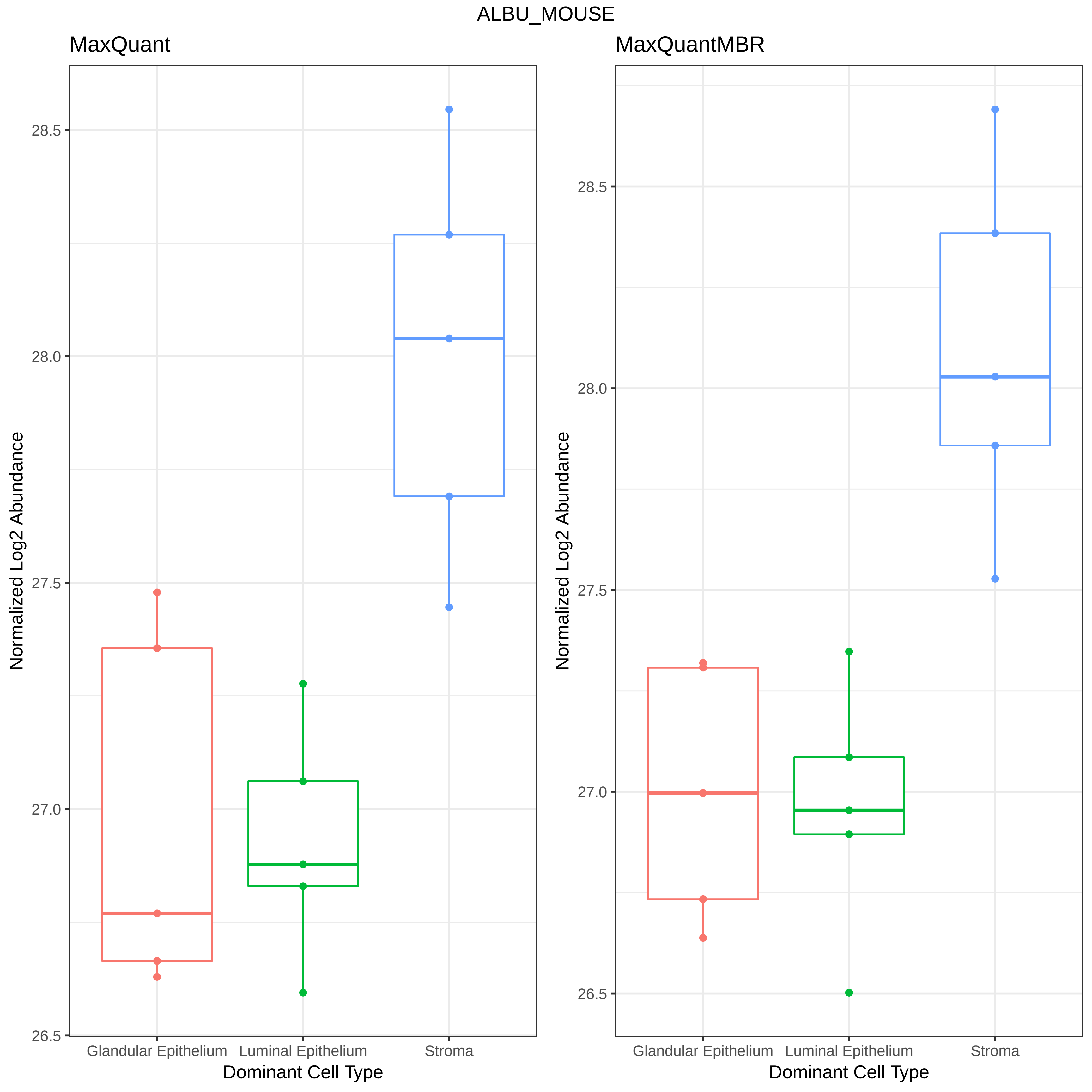
ALBU_MOUSE
MaxQuant
MaxQuantMBR
Normalized Log2 Abundance
28.5
28.0
27.5
27.0
26.5
Glandular Epithelium
Luminal Epithelium
Stroma
Dominant Cell Type

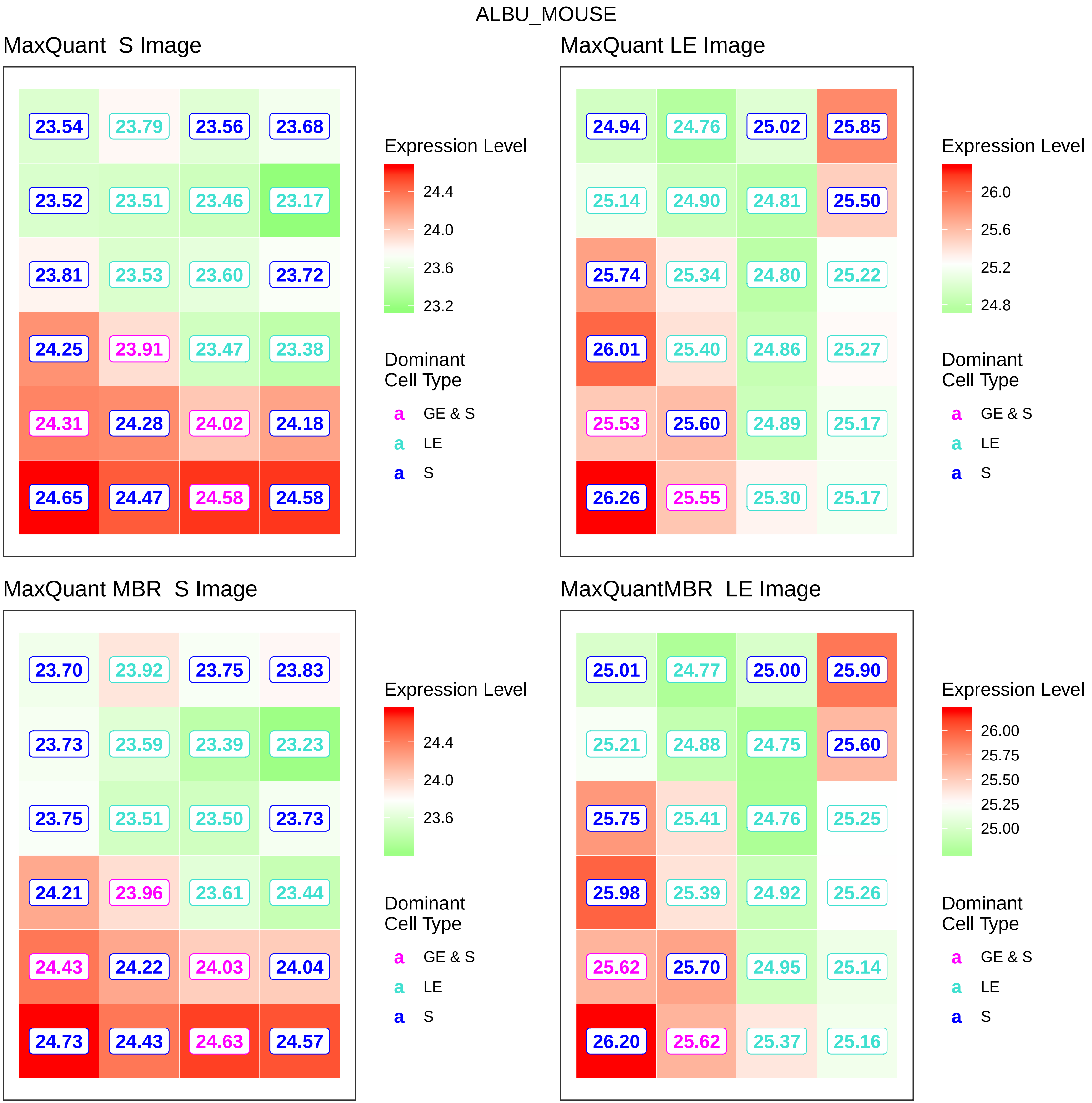
ALBU_MOUSE
MaxQuant S Image
23.54 23.79 23.56 23.68
23.52 23.51 23.46 23.17
23.81 23.53 23.60 23.72
24.25 23.91 23.47 23.38
24.31 24.28 24.02 24.18
24.65 24.47 24.58 24.58
Expression Level
24.4
24.0
23.6
23.2
Dominant Cell Type
a GE & S
a LE
a S
MaxQuant LE Image
24.94 24.76 25.02 25.85
25.14 24.90 24.81 25.50
25.74 25.34 24.80 25.22
26.01 25.40 24.86 25.27
25.53 25.60 24.89 25.17
26.26 25.55 25.30 25.17
Expression Level
26.0
25.6
25.2
24.8
Dominant Cell Type
a GE & S
a LE
a S
MaxQuant MBR S Image
23.70 23.92 23.75 23.83
23.73 23.59 23.39 23.23
23.75 23.51 23.50 23.73
24.21 23.96 23.61 23.44
24.43 24.22 24.03 24.04
24.73 24.43 24.63 24.57
Expression Level
24.4
24.0
23.6
Dominant Cell Type
a GE & S
a LE
a S
MaxQuantMBR LE Image
25.01 24.77 25.00 25.90
25.21 24.88 24.75 25.60
25.75 25.41 24.76 25.25
25.98 25.39 24.92 25.26
25.62 25.70 24.95 25.14
26.20 25.62 25.37 25.16
Expression Level
26.00
25.75
25.50
25.25
25.00
Dominant Cell Type
a GE & S
a LE
a S

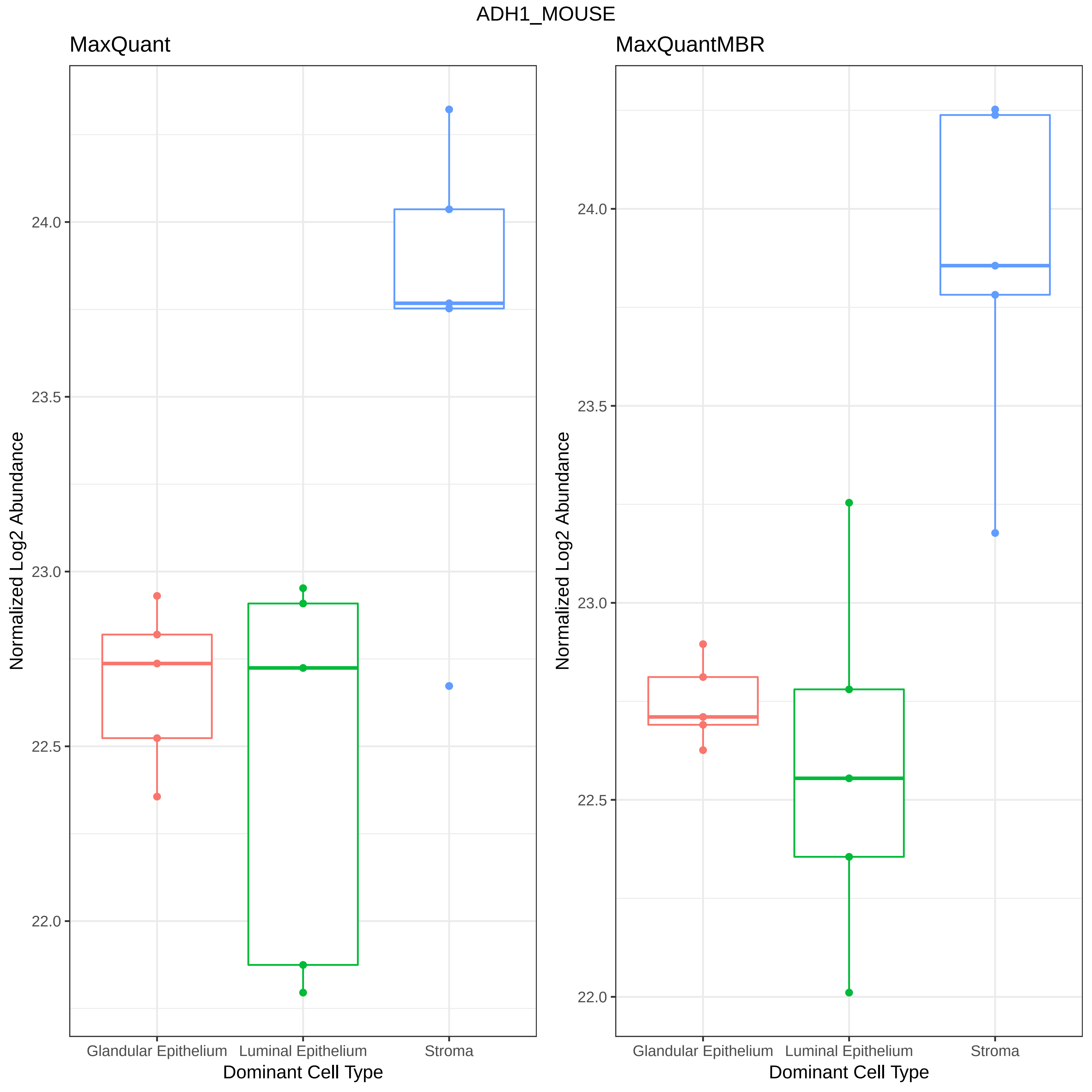
ADH1_MOUSE
MaxQuant
MaxQuantMBR
Normalized Log2 Abundance
Dominant Cell Type
Glandular Epithelium
Luminal Epithelium
Stroma
22.0
22.5
23.0
23.5
24.0

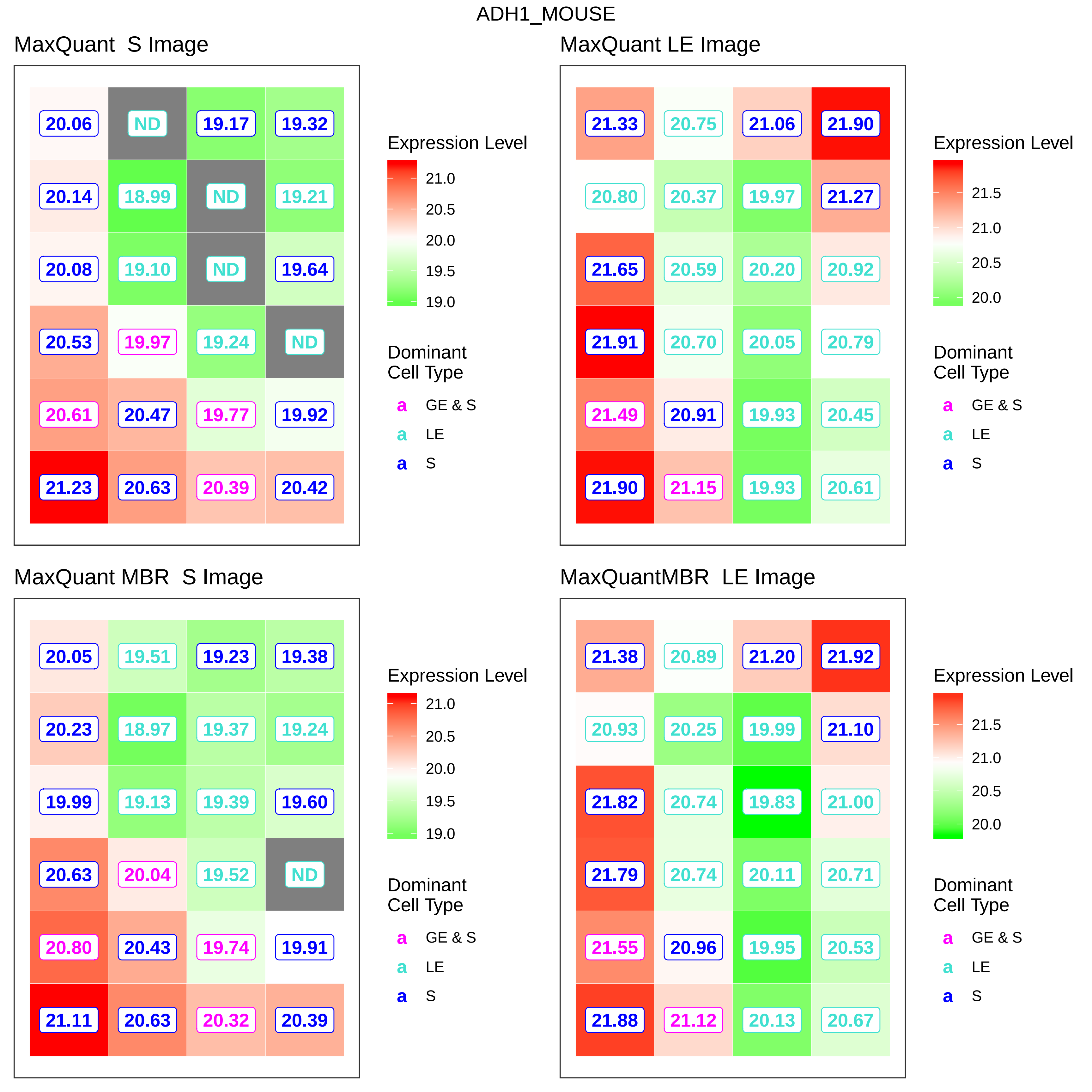
ADH1_MOUSE
MaxQuant S Image
20.06 ND 19.17 19.32
20.14 18.99 ND 19.21
20.08 19.10 ND 19.64
20.53 19.97 19.24 ND
20.61 20.47 19.77 19.92
21.23 20.63 20.39 20.42
Expression Level
21.0
20.5
20.0
19.5
19.0
Dominant Cell Type
a GE & S
a LE
a S
MaxQuant LE Image
21.33 20.75 21.06 21.90
20.80 20.37 19.97 21.27
21.65 20.59 20.20 20.92
21.91 20.70 20.05 20.79
21.49 20.91 19.93 20.45
21.90 21.15 19.93 20.61
Expression Level
21.5
21.0
20.5
20.0
Dominant Cell Type
a GE & S
a LE
a S
MaxQuant MBR S Image
20.05 19.51 19.23 19.38
20.23 18.97 19.37 19.24
19.99 19.13 19.39 19.60
20.63 20.04 19.52 ND
20.80 20.43 19.74 19.91
21.11 20.63 20.32 20.39
Expression Level
21.0
20.5
20.0
19.5
19.0
Dominant Cell Type
a GE & S
a LE
a S
MaxQuantMBR LE Image
21.38 20.89 21.20 21.92
20.93 20.25 19.99 21.10
21.82 20.74 19.83 21.00
21.79 20.74 20.11 20.71
21.55 20.96 19.95 20.53
21.88 21.12 20.13 20.67
Expression Level
21.5
21.0
20.5
20.0
Dominant Cell Type
a GE & S
a LE
a S

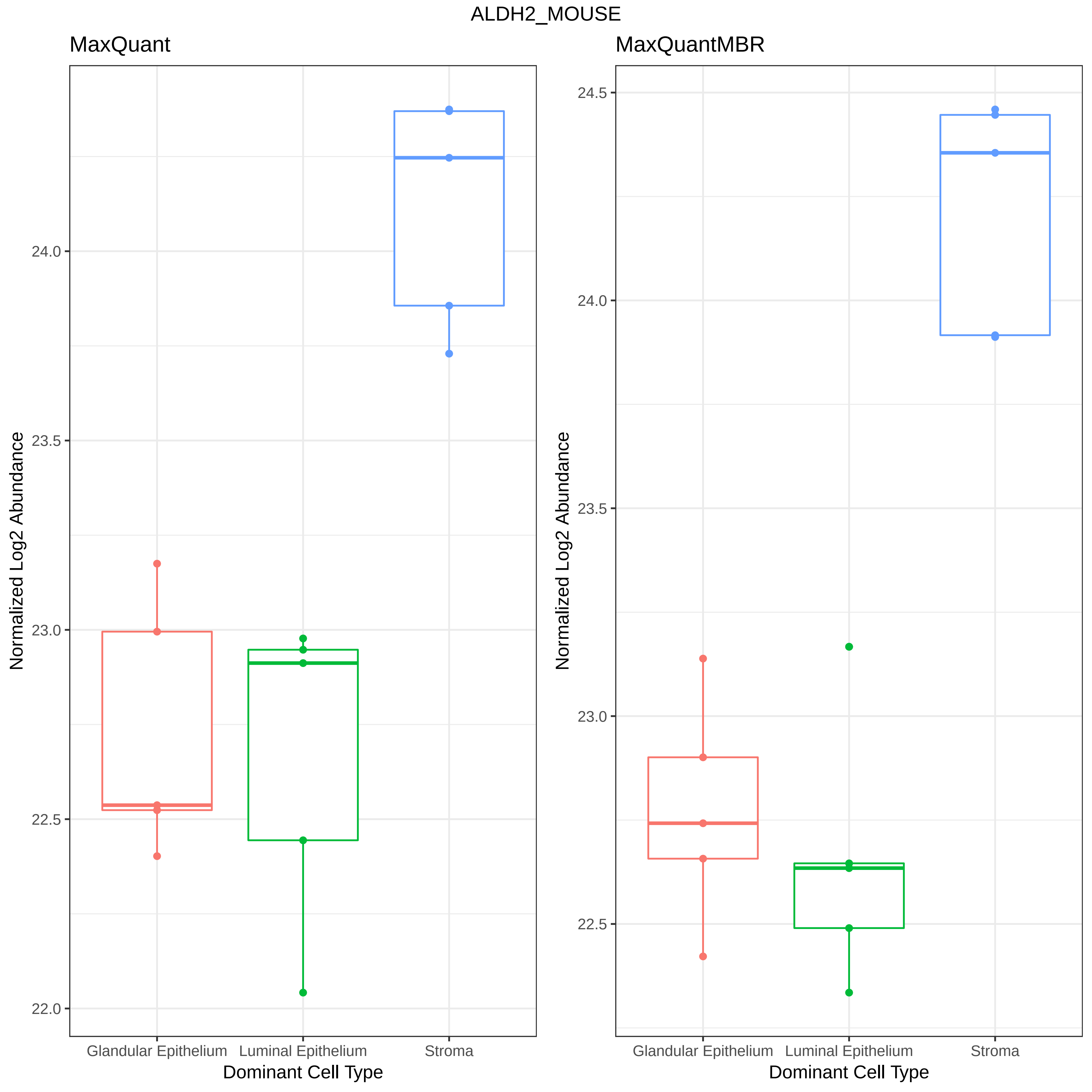

ALDH2_MOUSE
MaxQuant
MaxQuantMBR
Normalized Log2 Abundance
24.0
23.5
23.0
22.5
22.0
24.5
Glandular Epithelium
Luminal Epithelium
Stroma
Dominant Cell Type

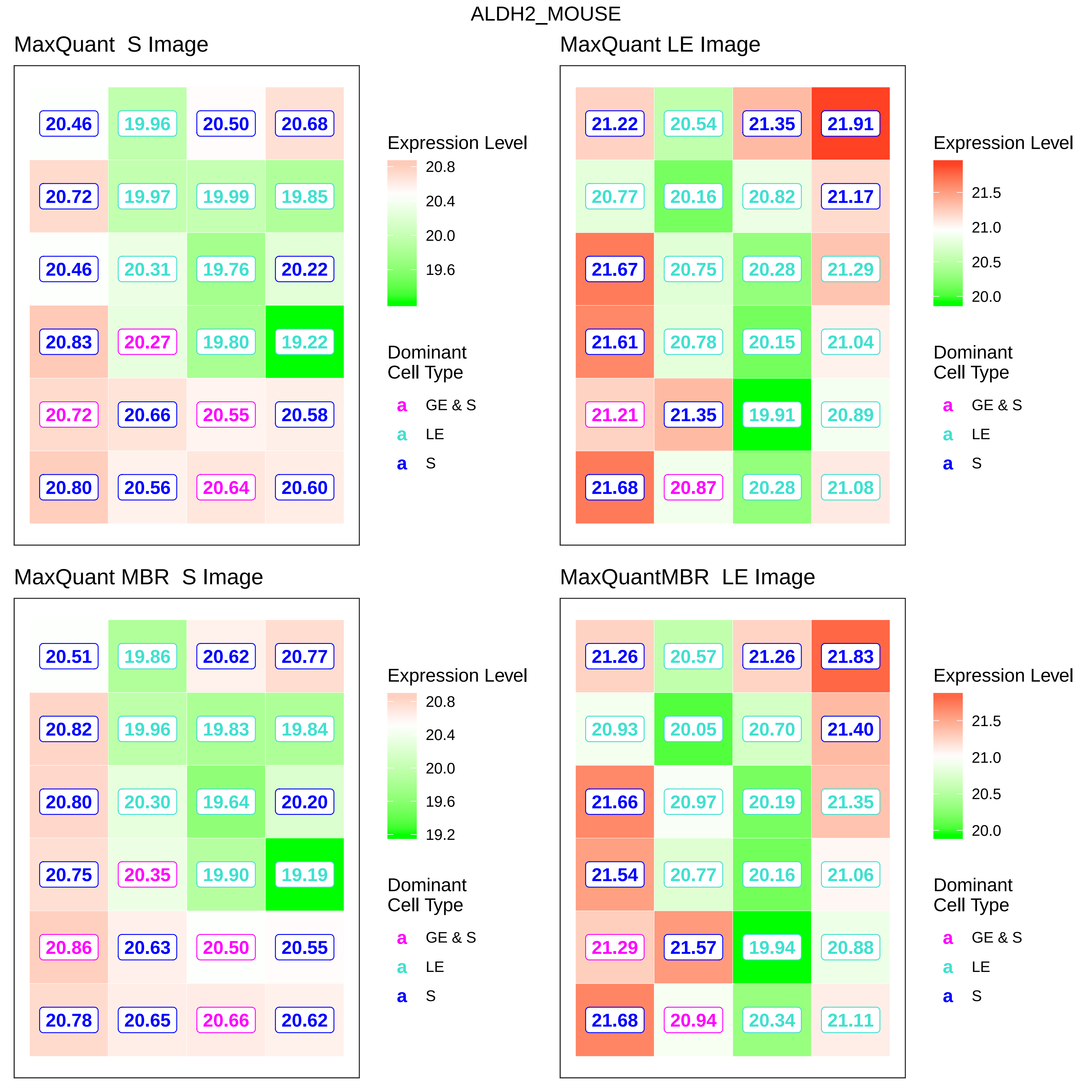
ALDH2_MOUSE
MaxQuant S Image
20.46 19.96 20.50 20.68
20.72 19.97 19.99 19.85
20.46 20.31 19.76 20.22
20.83 20.27 19.80 19.22
20.72 20.66 20.55 20.58
20.80 20.56 20.64 20.60
Expression Level
20.8
20.4
20.0
19.6
Dominant Cell Type
a GE & S
a LE
a S
MaxQuant LE Image
21.22 20.54 21.35 21.91
20.77 20.16 20.82 21.17
21.67 20.75 20.28 21.29
21.61 20.78 20.15 21.04
21.21 21.35 19.91 20.89
21.68 20.87 20.28 21.08
Expression Level
21.5
21.0
20.5
20.0
Dominant Cell Type
a GE & S
a LE
a S
MaxQuant MBR S Image
20.51 19.86 20.62 20.77
20.82 19.96 19.83 19.84
20.80 20.30 19.64 20.20
20.75 20.35 19.90 19.19
20.86 20.63 20.50 20.55
20.78 20.65 20.66 20.62
Expression Level
20.8
20.4
20.0
19.6
19.2
Dominant Cell Type
a GE & S
a LE
a S
MaxQuantMBR LE Image
21.26 20.57 21.26 21.83
20.93 20.05 20.70 21.40
21.66 20.97 20.19 21.35
21.54 20.77 20.16 21.06
21.29 21.57 19.94 20.88
21.68 20.94 20.34 21.11
Expression Level
21.5
21.0
20.5
20.0
Dominant Cell Type
a GE & S
a LE
a S

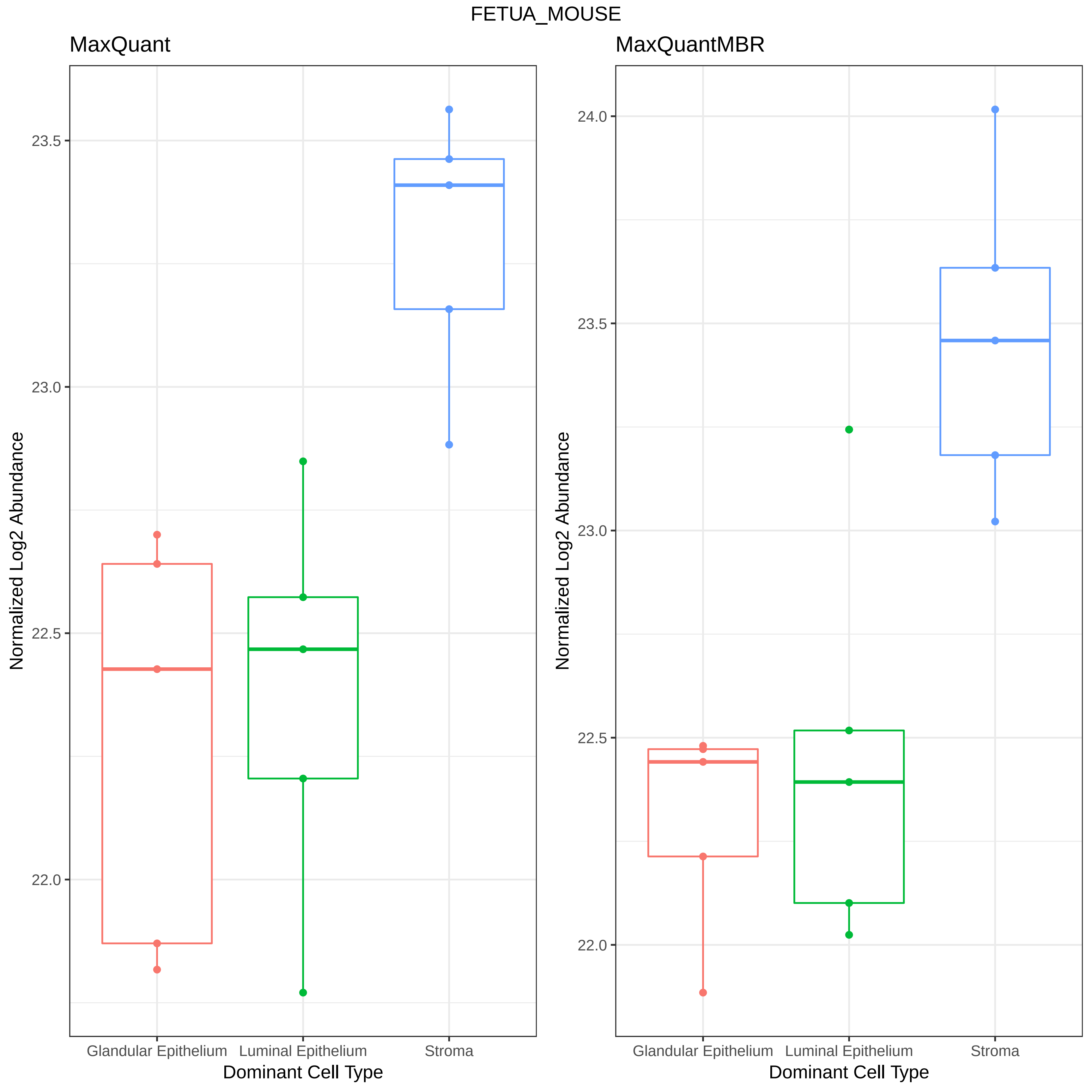
FETUA_MOUSE
MaxQuant
MaxQuantMBR
Normalized Log2 Abundance
23.5
23.0
22.5
22.0
24.0
23.5
23.0
22.5
22.0
Glandular Epithelium
Luminal Epithelium
Stroma
Dominant Cell Type

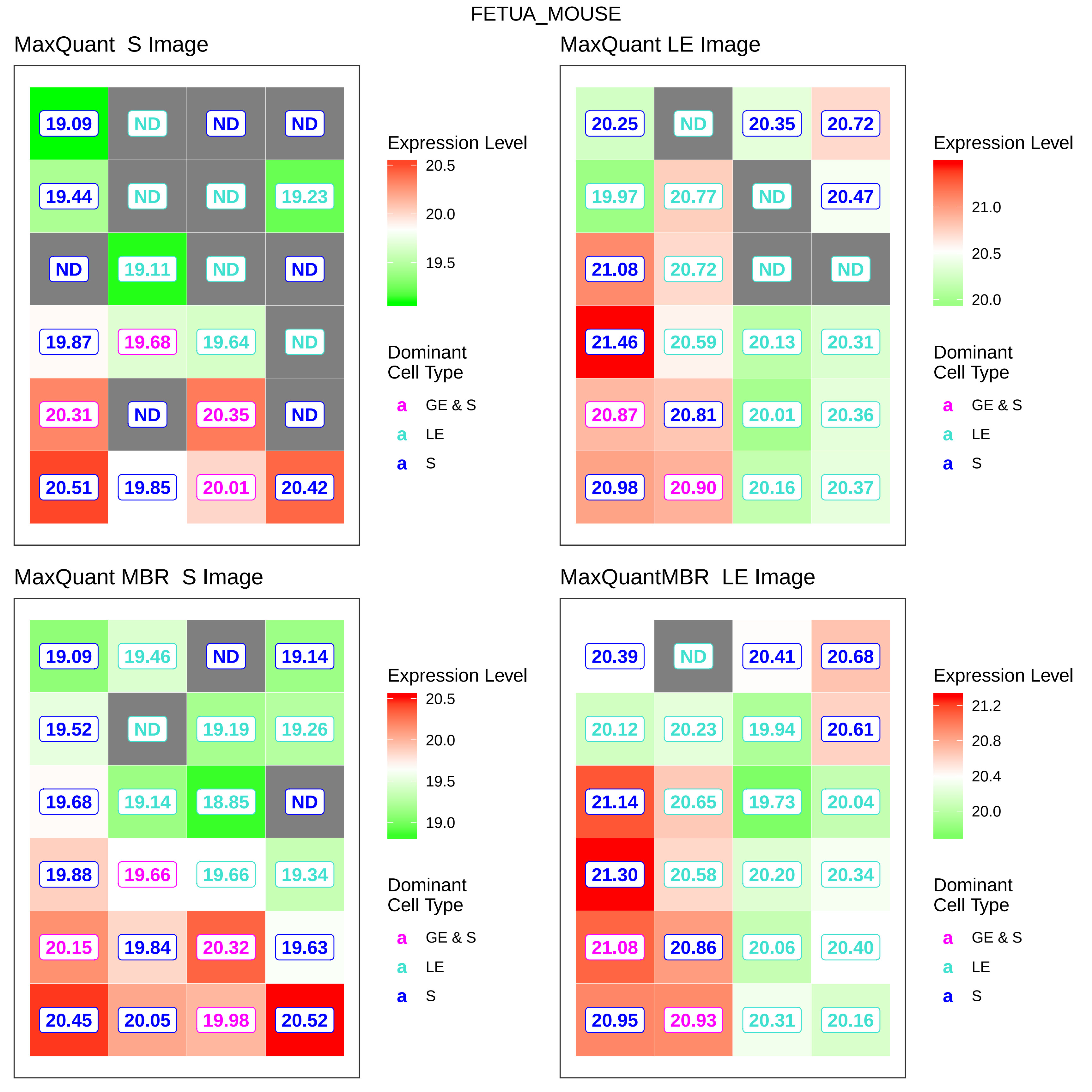
FETUA_MOUSE
MaxQuant S Image
19.09 ND ND ND
19.44 ND ND 19.23
ND 19.11 ND ND
19.87 19.68 19.64 ND
20.31 ND 20.35 ND
20.51 19.85 20.01 20.42
Expression Level
20.5
20.0
19.5
Dominant Cell Type
a GE & S
a LE
a S
MaxQuant LE Image
20.25 ND 20.35 20.72
19.97 20.77 ND 20.47
21.08 20.72 ND ND
21.46 20.59 20.13 20.31
20.87 20.81 20.01 20.36
20.98 20.90 20.16 20.37
Expression Level
21.0
20.5
20.0
Dominant Cell Type
a GE & S
a LE
a S
MaxQuant MBR S Image
19.09 19.46 ND 19.14
19.52 ND 19.19 19.26
19.68 19.14 18.85 ND
19.88 19.66 19.66 19.34
20.15 19.84 20.32 19.63
20.45 20.05 19.98 20.52
Expression Level
20.5
20.0
19.5
19.0
Dominant Cell Type
a GE & S
a LE
a S
MaxQuantMBR LE Image
20.39 ND 20.41 20.68
20.12 20.23 19.94 20.61
21.14 20.65 19.73 20.04
21.30 20.58 20.20 20.34
21.08 20.86 20.06 20.40
20.95 20.93 20.31 20.16
Expression Level
21.2
20.8
20.4
20.0
Dominant Cell Type
a GE & S
a LE
a S

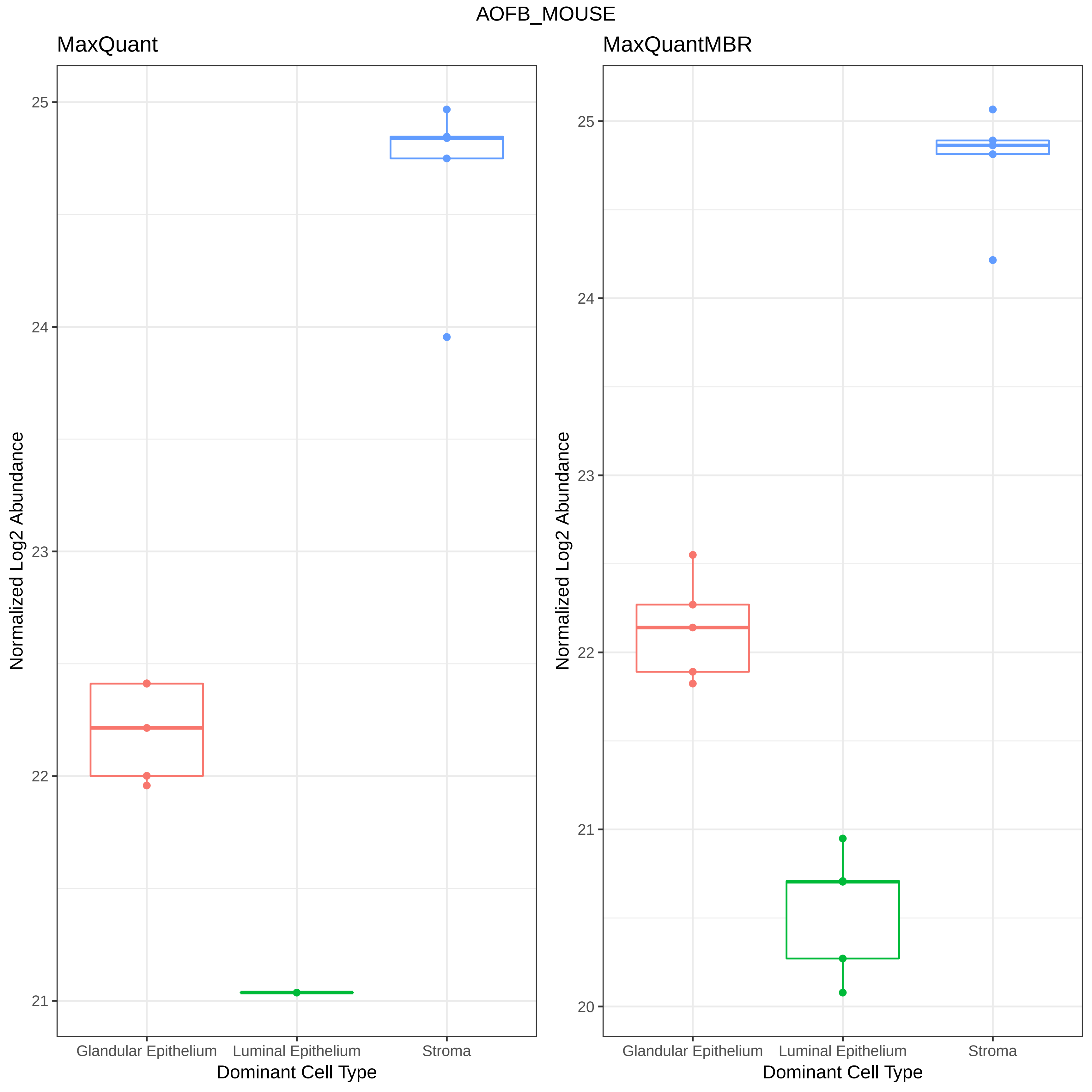

AOFB_MOUSE
MaxQuant
MaxQuantMBR
Normalized Log2 Abundance
25
24
23
22
21
20
Glandular Epithelium
Luminal Epithelium
Stroma
Dominant Cell Type

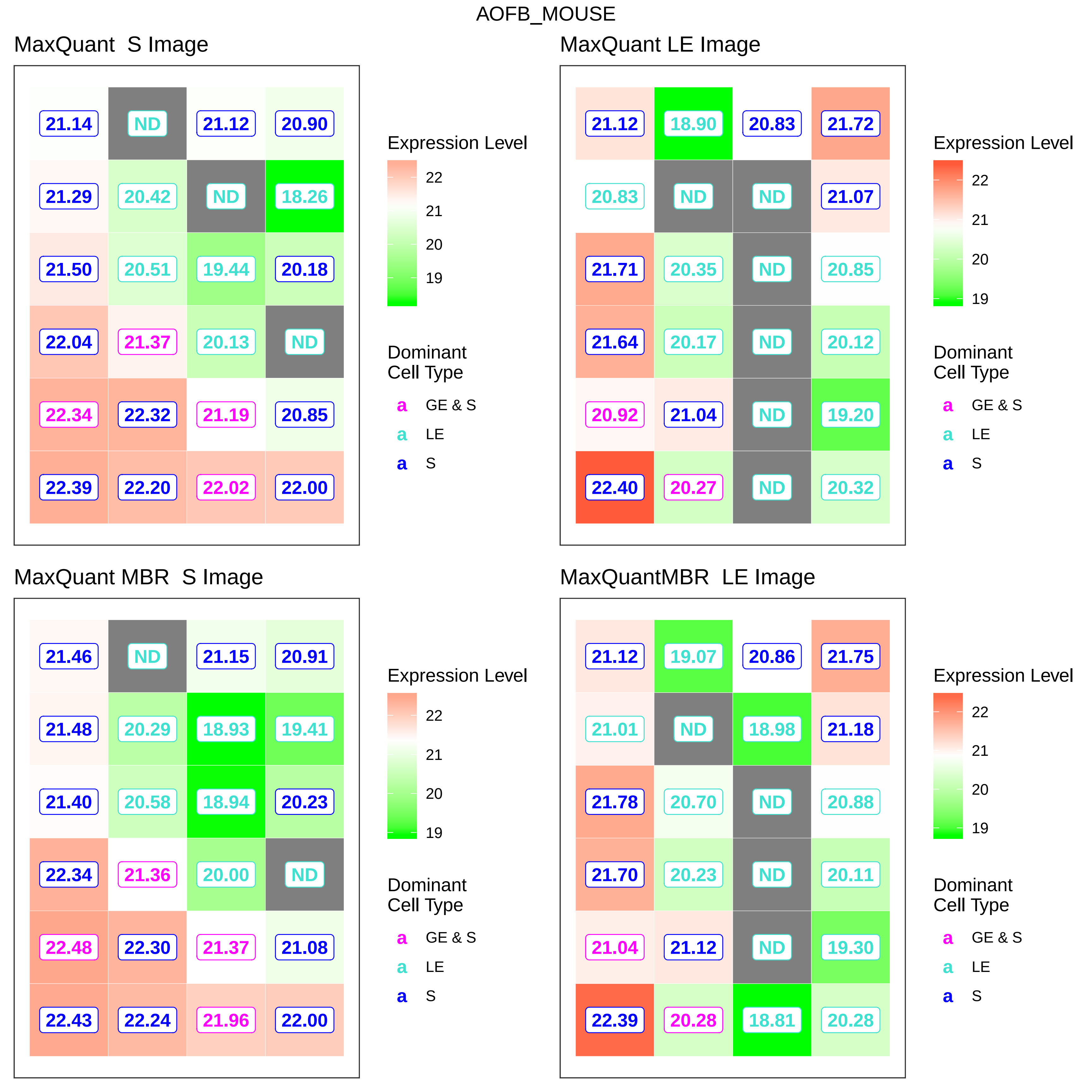
AOFB_MOUSE
MaxQuant S Image
21.14 ND 21.12 20.90
21.29 20.42 ND 18.26
21.50 20.51 19.44 20.18
22.04 21.37 20.13 ND
22.34 22.32 21.19 20.85
22.39 22.20 22.02 22.00
Expression Level
22
21
20
19
Dominant Cell Type
a GE & S
a LE
a S
MaxQuant LE Image
21.12 18.90 20.83 21.72
20.83 ND ND 21.07
21.71 20.35 ND 20.85
21.64 20.17 ND 20.12
20.92 21.04 ND 19.20
22.40 20.27 ND 20.32
Expression Level
22
21
20
19
Dominant Cell Type
a GE & S
a LE
a S
MaxQuant MBR S Image
21.46 ND 21.15 20.91
21.48 20.29 18.93 19.41
21.40 20.58 18.94 20.23
22.34 21.36 20.00 ND
22.48 22.30 21.37 21.08
22.43 22.24 21.96 22.00
Expression Level
22
21
20
19
Dominant Cell Type
a GE & S
a LE
a S
MaxQuantMBR LE Image
21.12 19.07 20.86 21.75
21.01 ND 18.98 21.18
21.78 20.70 ND 20.88
21.70 20.23 ND 20.11
21.04 21.12 ND 19.30
22.39 20.28 18.81 20.28
Expression Level
22
21
20
19
Dominant Cell Type
a GE & S
a LE
a S

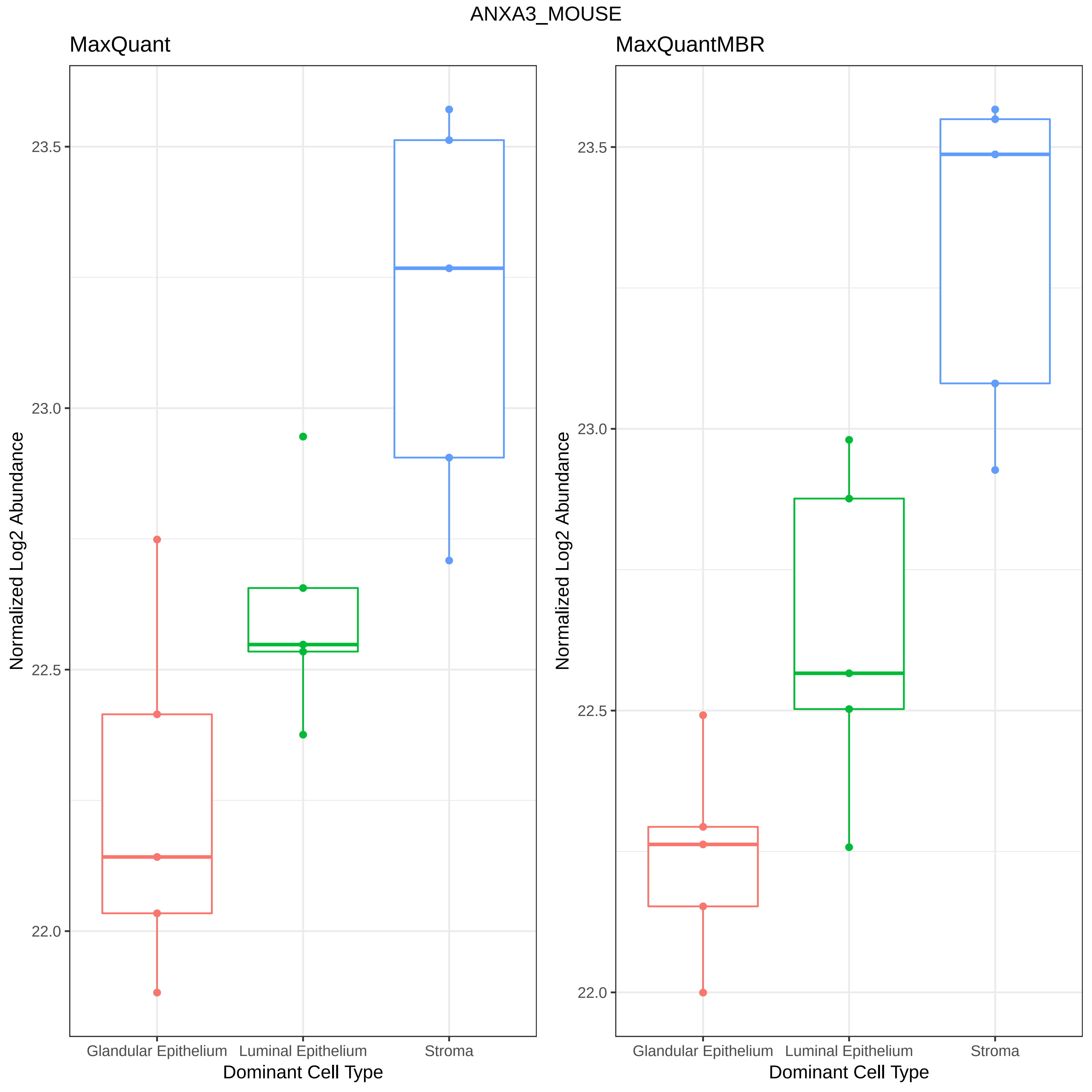

ANXA3_MOUSE
MaxQuant
MaxQuantMBR
Normalized Log2 Abundance
23.5
23.0
22.5
22.0
Glandular Epithelium
Luminal Epithelium
Stroma
Dominant Cell Type

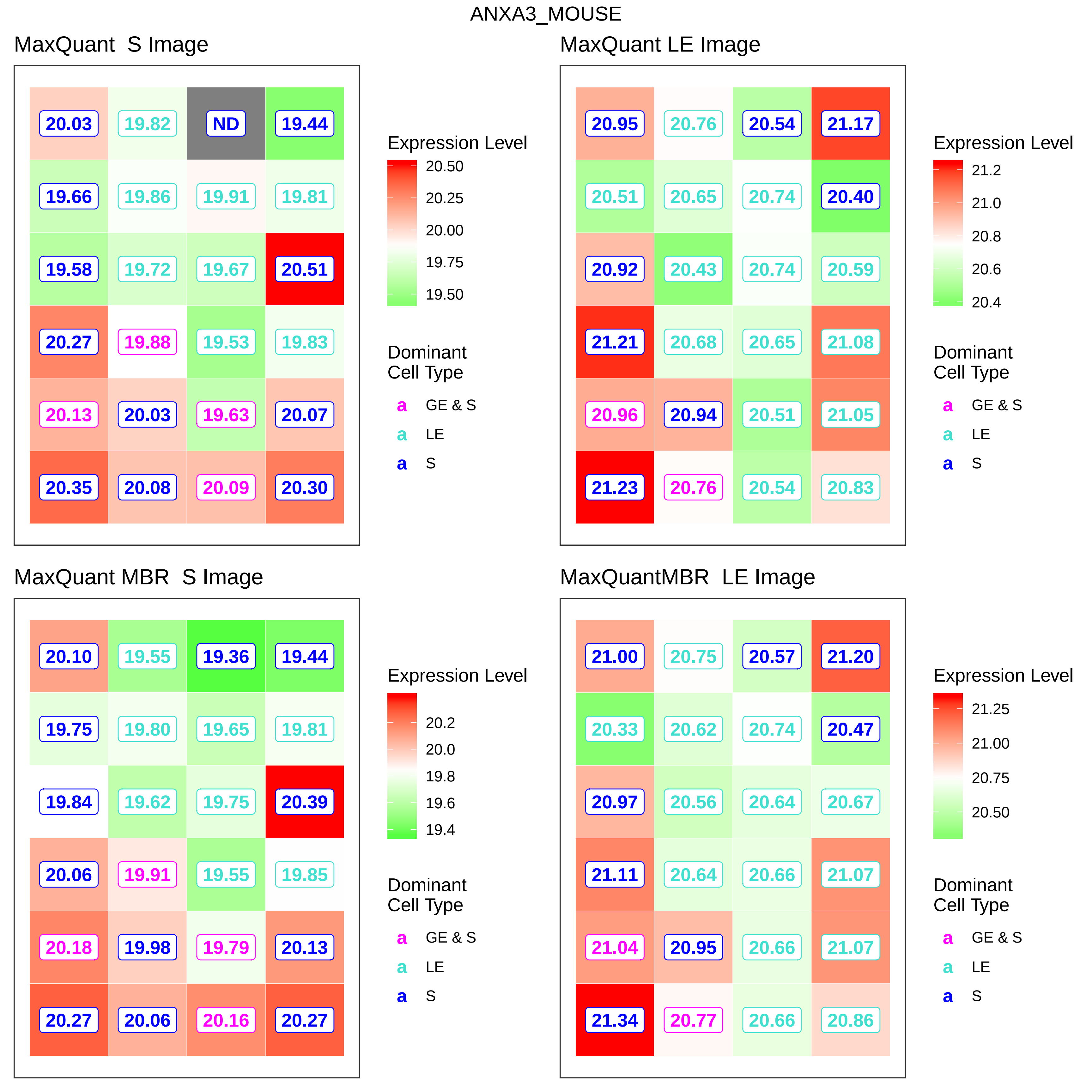
ANXA3_MOUSE
MaxQuant S Image
20.03 19.82 ND 19.44
19.66 19.86 19.91 19.81
19.58 19.72 19.67 20.51
20.27 19.88 19.53 19.83
20.13 20.03 19.63 20.07
20.35 20.08 20.09 20.30
Expression Level
20.50
20.25
20.00
19.75
19.50
Dominant Cell Type
a GE & S
a LE
a S
MaxQuant LE Image
20.95 20.76 20.54 21.17
20.51 20.65 20.74 20.40
20.92 20.43 20.74 20.59
21.21 20.68 20.65 21.08
20.96 20.94 20.51 21.05
21.23 20.76 20.54 20.83
Expression Level
21.2
21.0
20.8
20.6
20.4
Dominant Cell Type
a GE & S
a LE
a S
MaxQuant MBR S Image
20.10 19.55 19.36 19.44
19.75 19.80 19.65 19.81
19.84 19.62 19.75 20.39
20.06 19.91 19.55 19.85
20.18 19.98 19.79 20.13
20.27 20.06 20.16 20.27
Expression Level
20.2
20.0
19.8
19.6
19.4
Dominant Cell Type
a GE & S
a LE
a S
MaxQuantMBR LE Image
21.00 20.75 20.57 21.20
20.33 20.62 20.74 20.47
20.97 20.56 20.64 20.67
21.11 20.64 20.66 21.07
21.04 20.95 20.66 21.07
21.34 20.77 20.66 20.86
Expression Level
21.25
21.00
20.75
20.50
Dominant Cell Type
a GE & S
a LE
a S

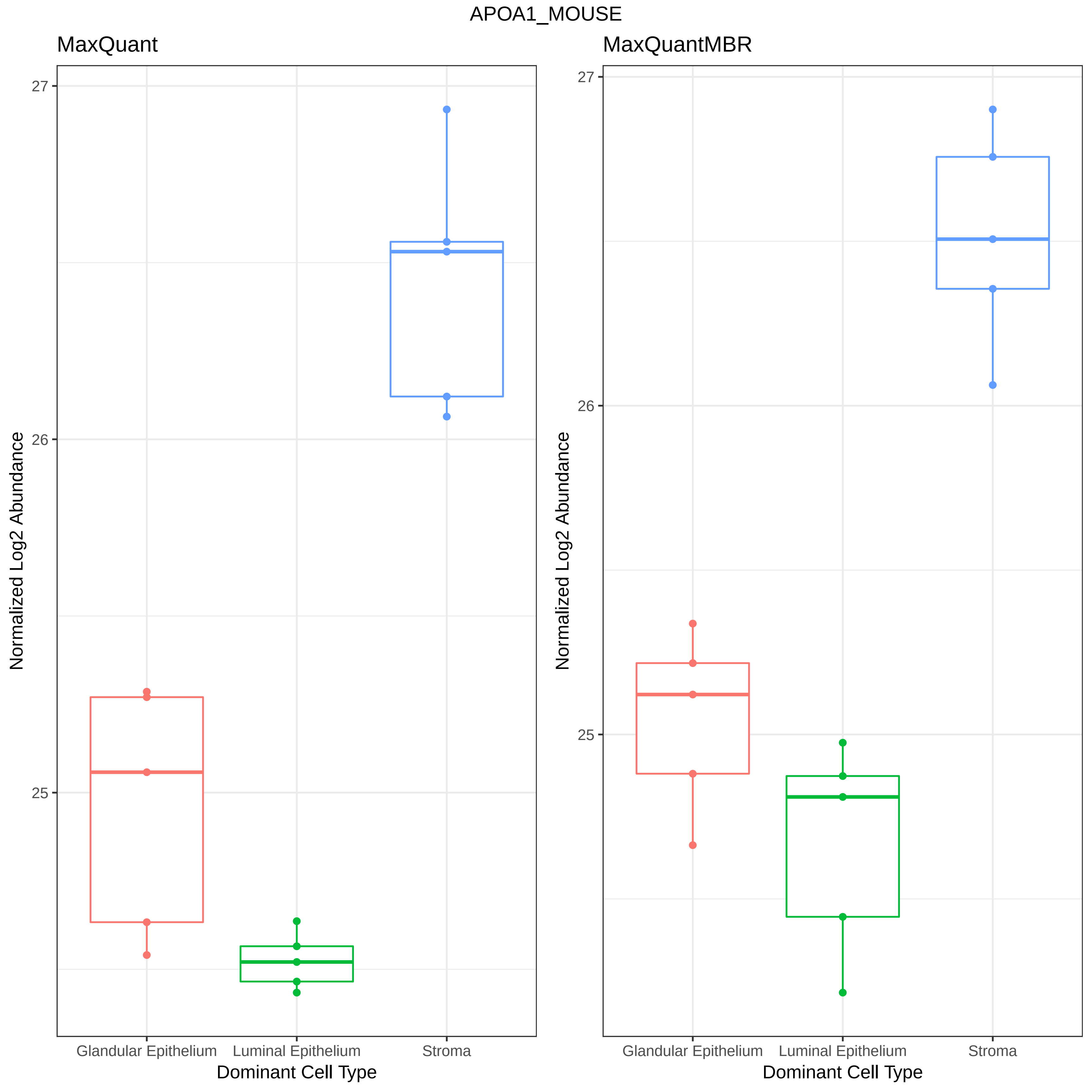
APOA1_MOUSE
MaxQuant
MaxQuantMBR
27
26
25
Normalized Log2 Abundance
Glandular Epithelium
Luminal Epithelium
Stroma
Dominant Cell Type

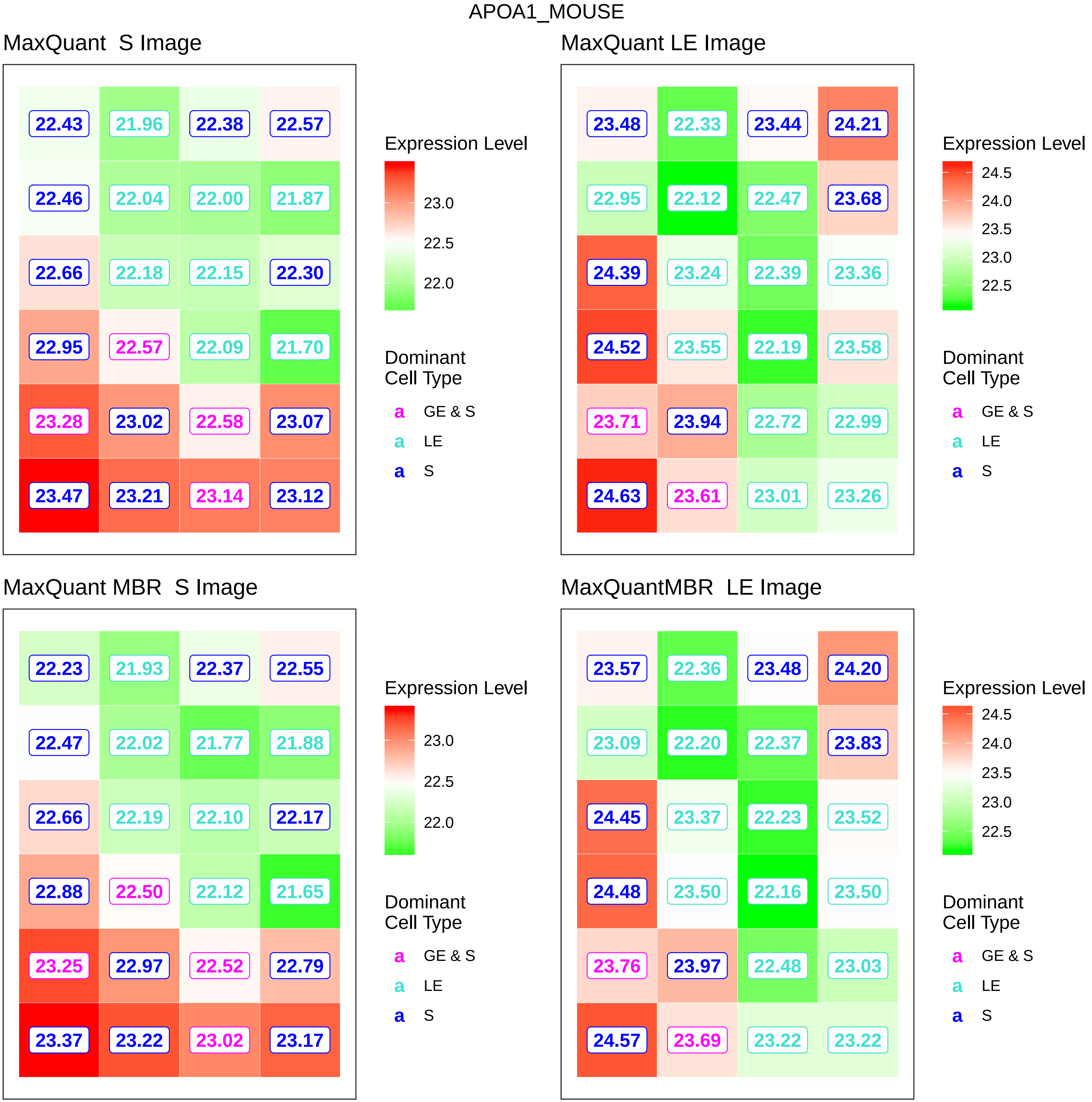
APOA1_MOUSE
MaxQuant S Image
22.43 21.96 22.38 22.57
22.46 22.04 22.00 21.87
22.66 22.18 22.15 22.30
22.95 22.57 22.09 21.70
23.28 23.02 22.58 23.07
23.47 23.21 23.14 23.12
Expression Level
23.0
22.5
22.0
Dominant Cell Type
a GE & S
a LE
a S
MaxQuant LE Image
23.48 22.33 23.44 24.21
22.95 22.12 22.47 23.68
24.39 23.24 22.39 23.36
24.52 23.55 22.19 23.58
23.71 23.94 22.72 22.99
24.63 23.61 23.01 23.26
Expression Level
24.5
24.0
23.5
23.0
22.5
Dominant Cell Type
a GE & S
a LE
a S
MaxQuant MBR S Image
22.23 21.93 22.37 22.55
22.47 22.02 21.77 21.88
22.66 22.19 22.10 22.17
22.88 22.50 22.12 21.65
23.25 22.97 22.52 22.79
23.37 23.22 23.02 23.17
Expression Level
23.0
22.5
22.0
Dominant Cell Type
a GE & S
a LE
a S
MaxQuantMBR LE Image
23.57 22.36 23.48 24.20
23.09 22.20 22.37 23.83
24.45 23.37 22.23 23.52
24.48 23.50 22.16 23.50
23.76 23.97 22.48 23.03
24.57 23.69 23.22 23.22
Expression Level
24.5
24.0
23.5
23.0
22.5
Dominant Cell Type
a GE & S
a LE
a S

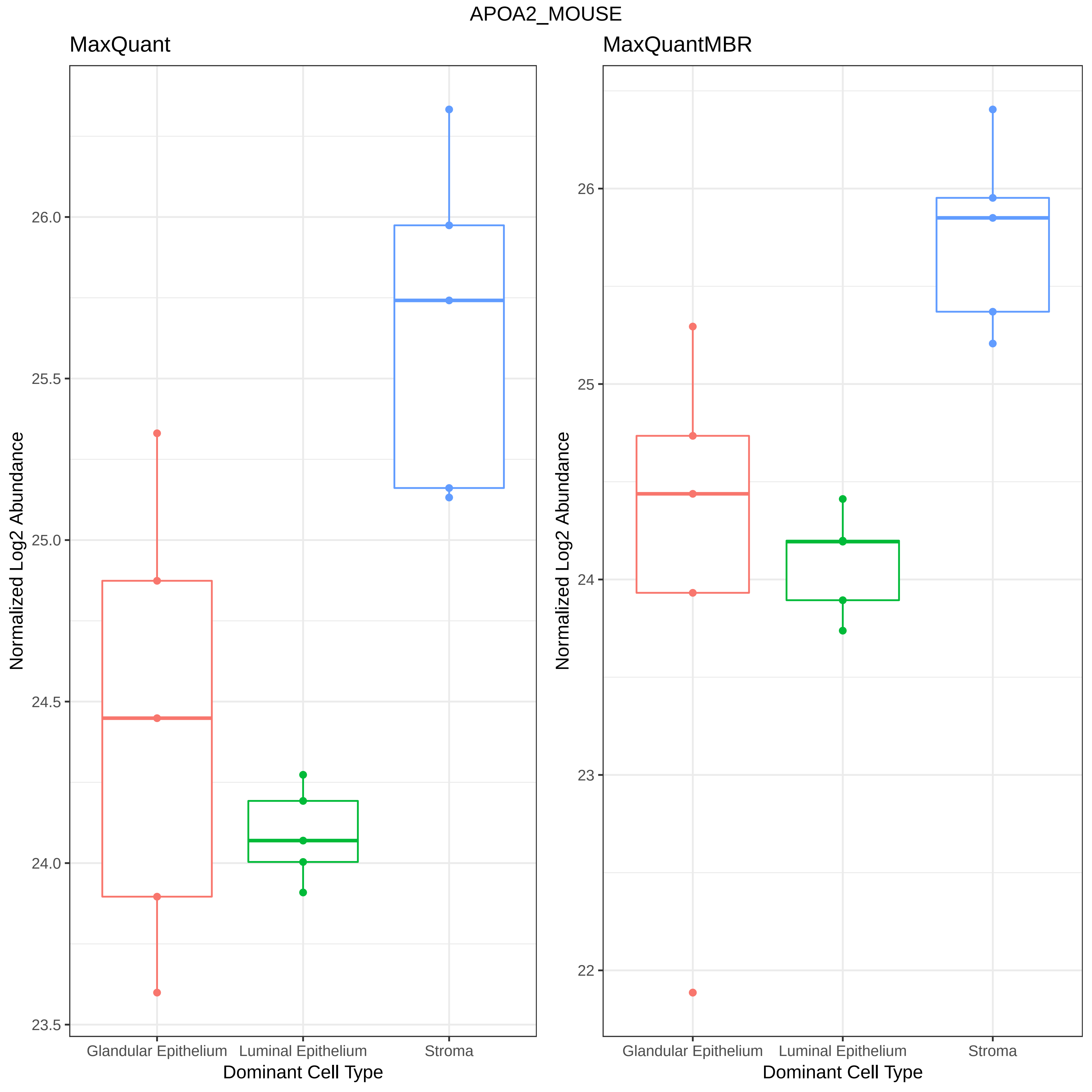
APOA2_MOUSE
MaxQuant
MaxQuantMBR
Normalized Log2 Abundance
Dominant Cell Type
Glandular Epithelium
Luminal Epithelium
Stroma
23.5
24.0
24.5
25.0
25.5
26.0
22
23
24
25
26

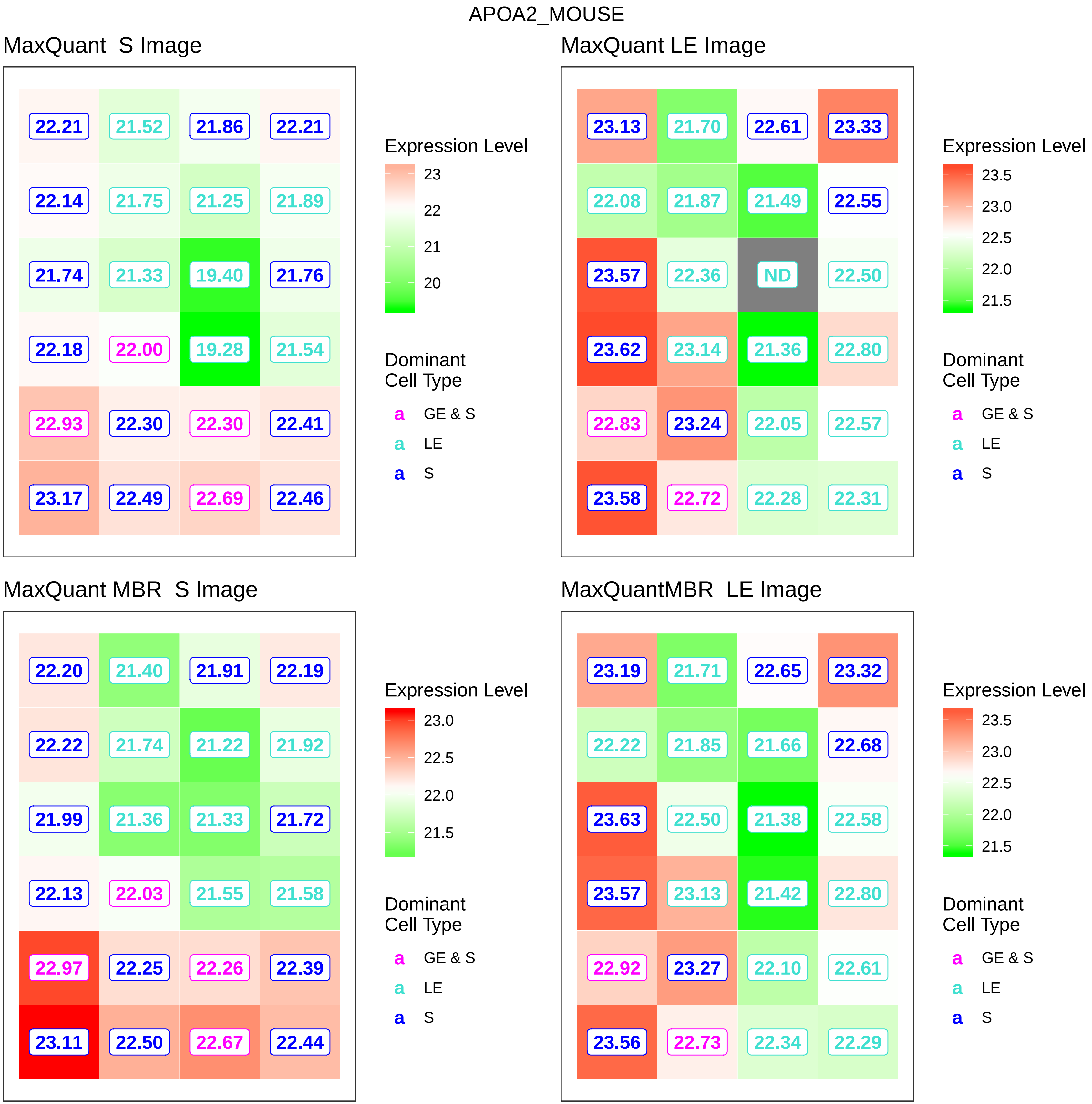
APOA2_MOUSE
MaxQuant S Image
22.21 21.52 21.86 22.21
22.14 21.75 21.25 21.89
21.74 21.33 19.40 21.76
22.18 22.00 19.28 21.54
22.93 22.30 22.30 22.41
23.17 22.49 22.69 22.46
Expression Level
23
22
21
20
Dominant Cell Type
a GE & S
a LE
a S
MaxQuant LE Image
23.13 21.70 22.61 23.33
22.08 21.87 21.49 22.55
23.57 22.36 ND 22.50
23.62 23.14 21.36 22.80
22.83 23.24 22.05 22.57
23.58 22.72 22.28 22.31
Expression Level
23.5
23.0
22.5
22.0
21.5
Dominant Cell Type
a GE & S
a LE
a S
MaxQuant MBR S Image
22.20 21.40 21.91 22.19
22.22 21.74 21.22 21.92
21.99 21.36 21.33 21.72
22.13 22.03 21.55 21.58
22.97 22.25 22.26 22.39
23.11 22.50 22.67 22.44
Expression Level
23.0
22.5
22.0
21.5
Dominant Cell Type
a GE & S
a LE
a S
MaxQuantMBR LE Image
23.19 21.71 22.65 23.32
22.22 21.85 21.66 22.68
23.63 22.50 21.38 22.58
23.57 23.13 21.42 22.80
22.92 23.27 22.10 22.61
23.56 22.73 22.34 22.29
Expression Level
23.5
23.0
22.5
22.0
21.5
Dominant Cell Type
a GE & S
a LE
a S

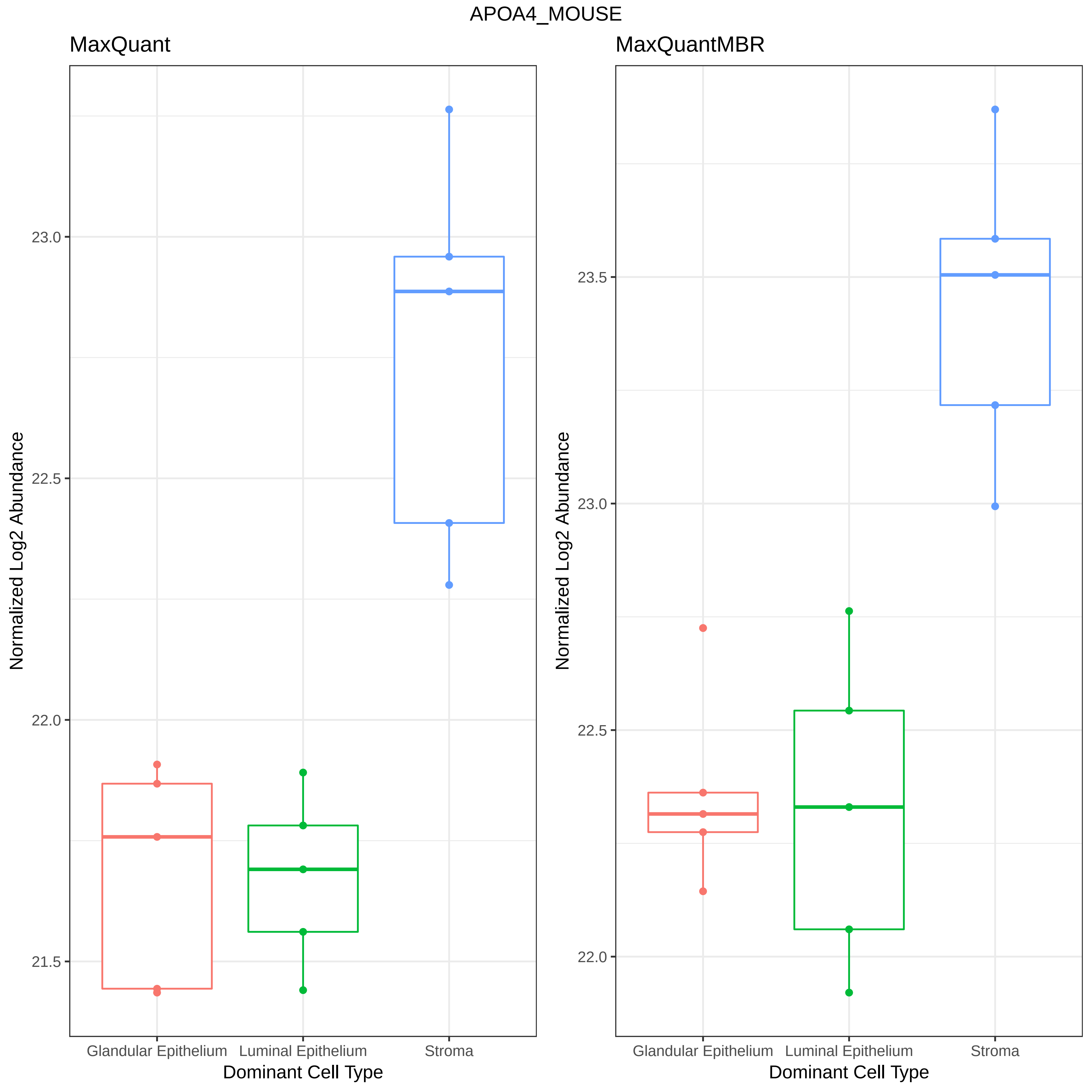
APOA4_MOUSE
MaxQuant
MaxQuantMBR
Normalized Log2 Abundance
23.0
22.5
22.0
21.5
23.5
Glandular Epithelium
Luminal Epithelium
Stroma
Dominant Cell Type

APOA4_MOUSE

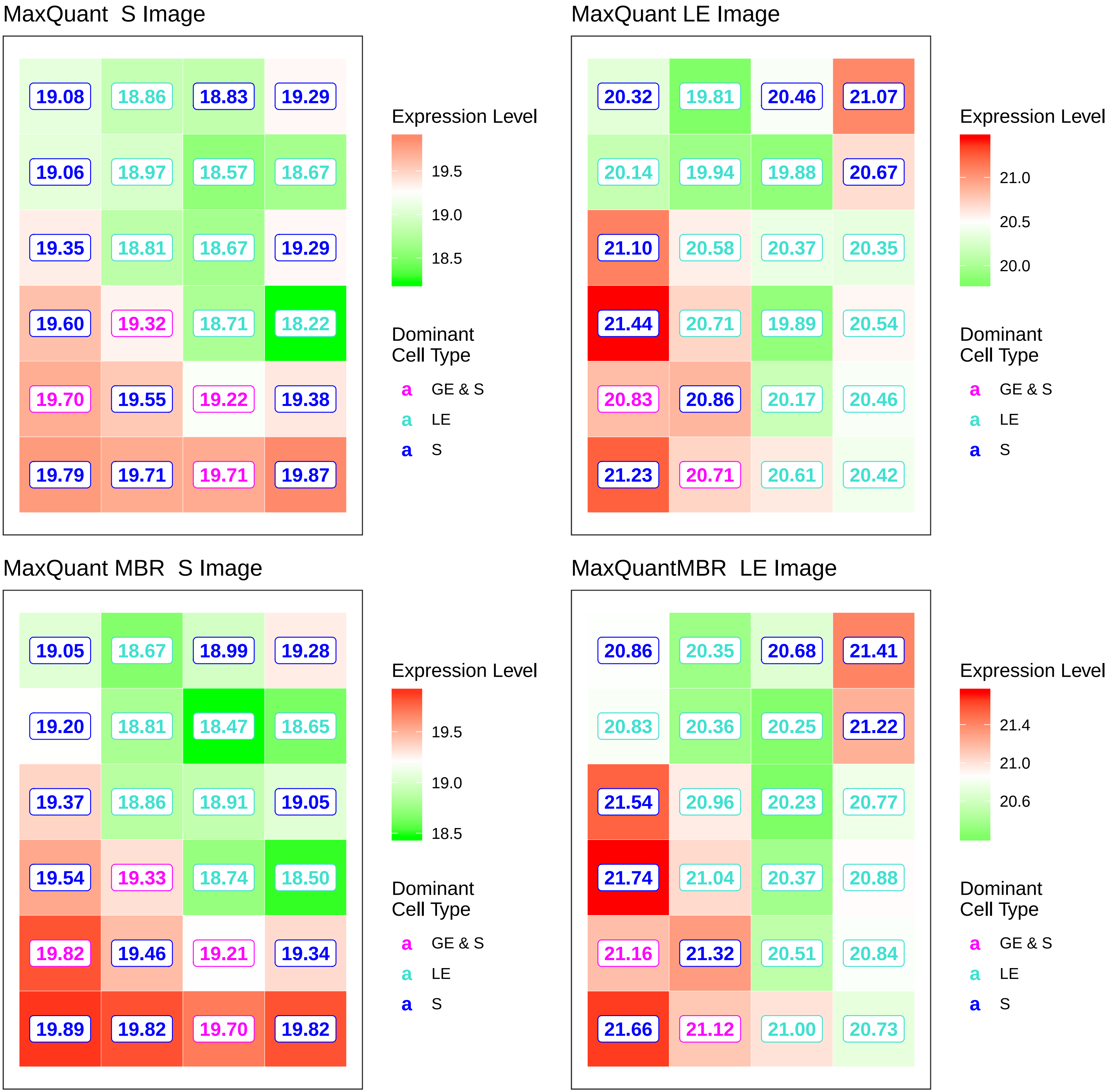
MaxQuant S Image
19.08
18.86
18.83
19.29
19.06
18.97
18.57
18.67
19.35
18.81
18.67
19.29
19.60
19.32
18.71
18.22
19.70
19.55
19.22
19.38
19.79
19.71
19.71
19.87
Expression Level
19.5
19.0
18.5
Dominant Cell Type
a GE & S
a LE
a S
MaxQuant LE Image
20.32
19.81
20.46
21.07
20.14
19.94
19.88
20.67
21.10
20.58
20.37
20.35
21.44
20.71
19.89
20.54
20.83
20.86
20.17
20.46
21.23
20.71
20.61
20.42
Expression Level
21.0
20.5
20.0
Dominant Cell Type
a GE & S
a LE
a S
MaxQuant MBR S Image
19.05
18.67
18.99
19.28
19.20
18.81
18.47
18.65
19.37
18.86
18.91
19.05
19.54
19.33
18.74
18.50
19.82
19.46
19.21
19.34
19.89
19.82
19.70
19.82
Expression Level
19.5
19.0
18.5
Dominant Cell Type
a GE & S
a LE
a S
MaxQuantMBR LE Image
20.86
20.35
20.68
21.41
20.83
20.36
20.25
21.22
21.54
20.96
20.23
20.77
21.74
21.04
20.37
20.88
21.16
21.32
20.51
20.84
21.66
21.12
21.00
20.73
Expression Level
21.4
21.0
20.6
Dominant Cell Type
a GE & S
a LE
a S

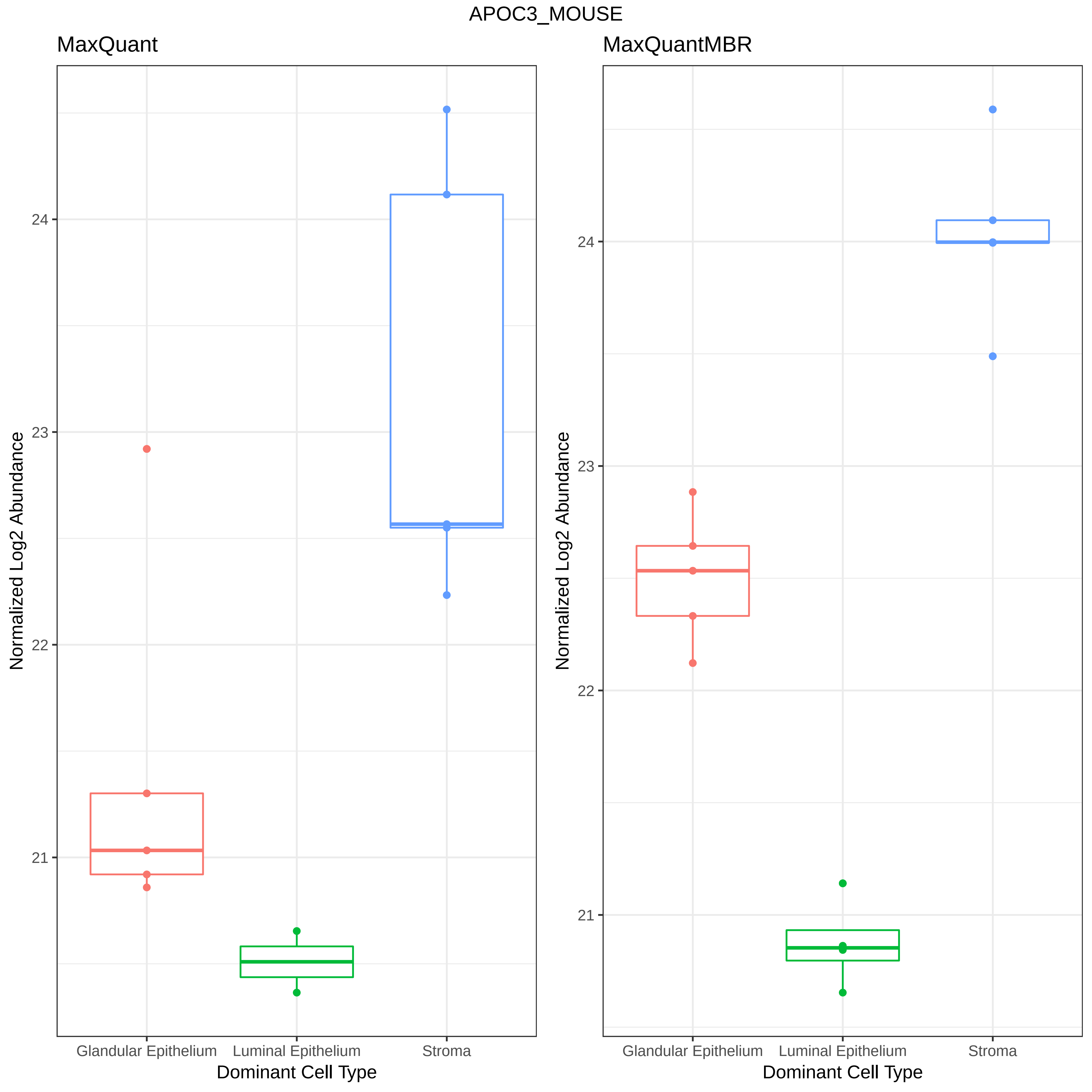
APOC3_MOUSE
MaxQuant
MaxQuantMBR
Normalized Log2 Abundance
24
23
22
21
Glandular Epithelium
Luminal Epithelium
Stroma
Dominant Cell Type

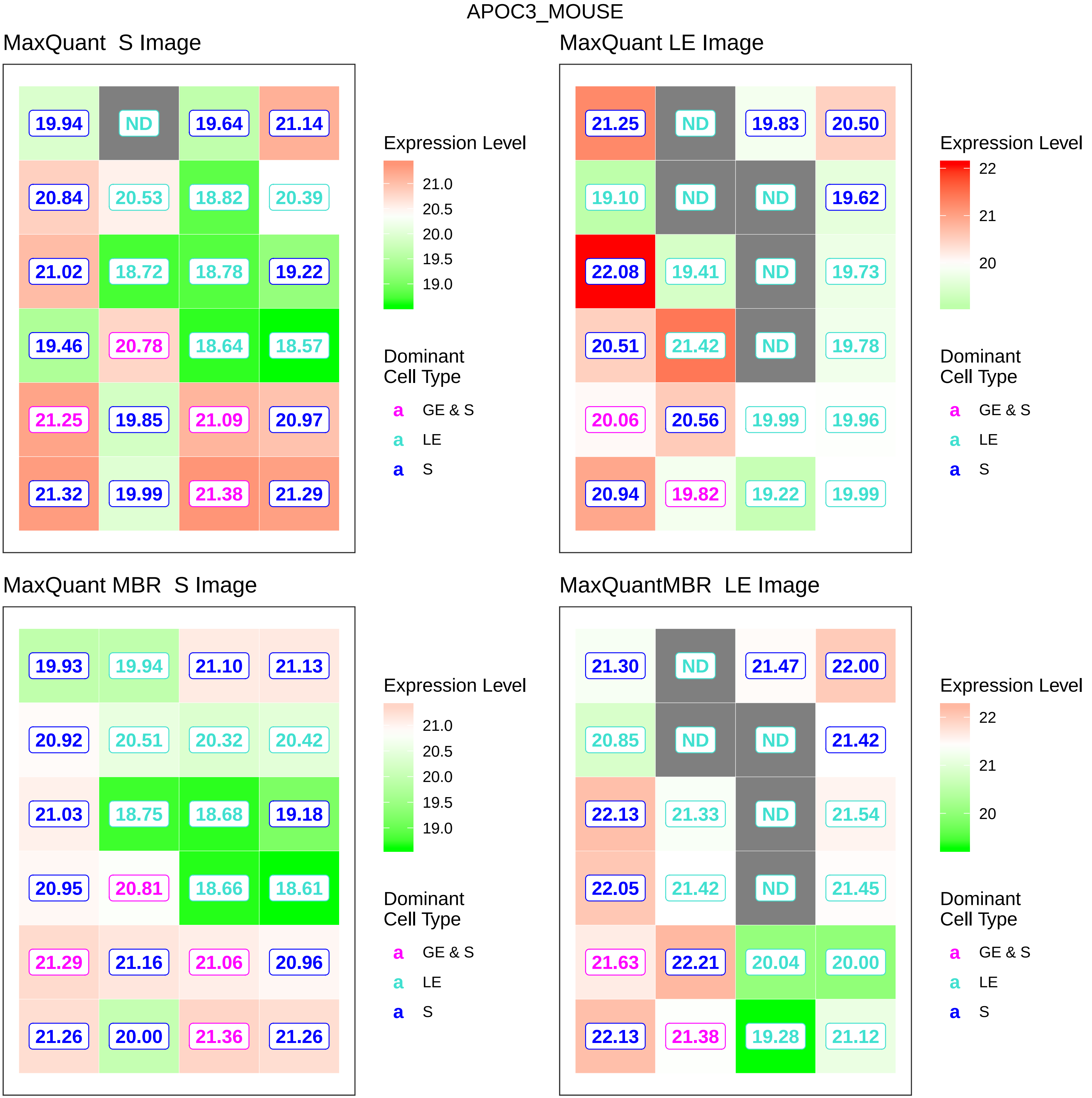
APOC3_MOUSE
MaxQuant S Image
19.94 ND 19.64 21.14
20.84 20.53 18.82 20.39
21.02 18.72 18.78 19.22
19.46 20.78 18.64 18.57
21.25 19.85 21.09 20.97
21.32 19.99 21.38 21.29
Expression Level
21.0
20.5
20.0
19.5
19.0
Dominant Cell Type
a GE & S
a LE
a S
MaxQuant LE Image
21.25 ND 19.83 20.50
19.10 ND ND 19.62
22.08 19.41 ND 19.73
20.51 21.42 ND 19.78
20.06 20.56 19.99 19.96
20.94 19.82 19.22 19.99
Expression Level
22
21
20
Dominant Cell Type
a GE & S
a LE
a S
MaxQuant MBR S Image
19.93 19.94 21.10 21.13
20.92 20.51 20.32 20.42
21.03 18.75 18.68 19.18
20.95 20.81 18.66 18.61
21.29 21.16 21.06 20.96
21.26 20.00 21.36 21.26
Expression Level
21.0
20.5
20.0
19.5
19.0
Dominant Cell Type
a GE & S
a LE
a S
MaxQuantMBR LE Image
21.30 ND 21.47 22.00
20.85 ND ND 21.42
22.13 21.33 ND 21.54
22.05 21.42 ND 21.45
21.63 22.21 20.04 20.00
22.13 21.38 19.28 21.12
Expression Level
22
21
20
Dominant Cell Type
a GE & S
a LE
a S

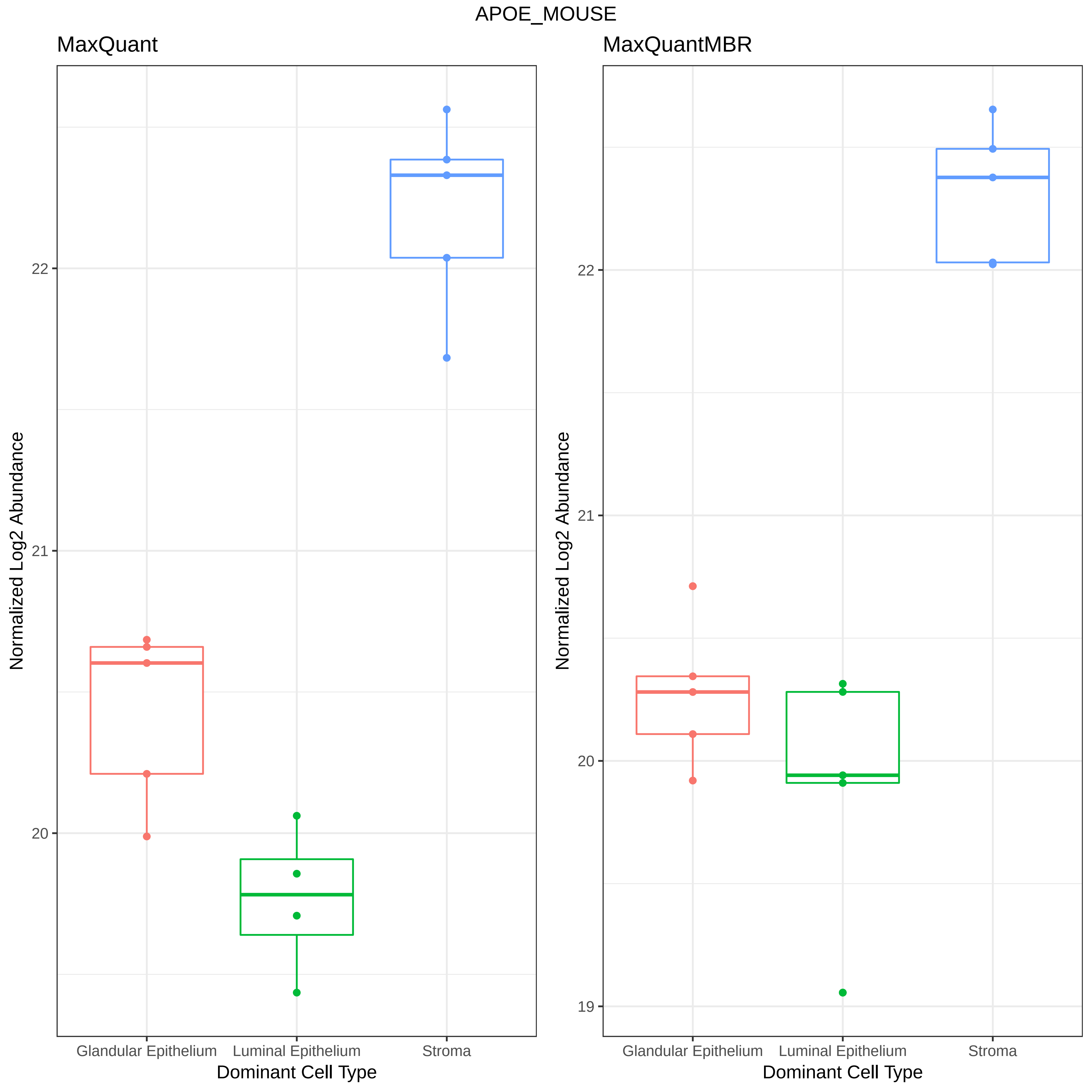

APOE_MOUSE
MaxQuant
MaxQuantMBR
Normalized Log2 Abundance
22
21
20
19
Glandular Epithelium
Luminal Epithelium
Stroma
Dominant Cell Type

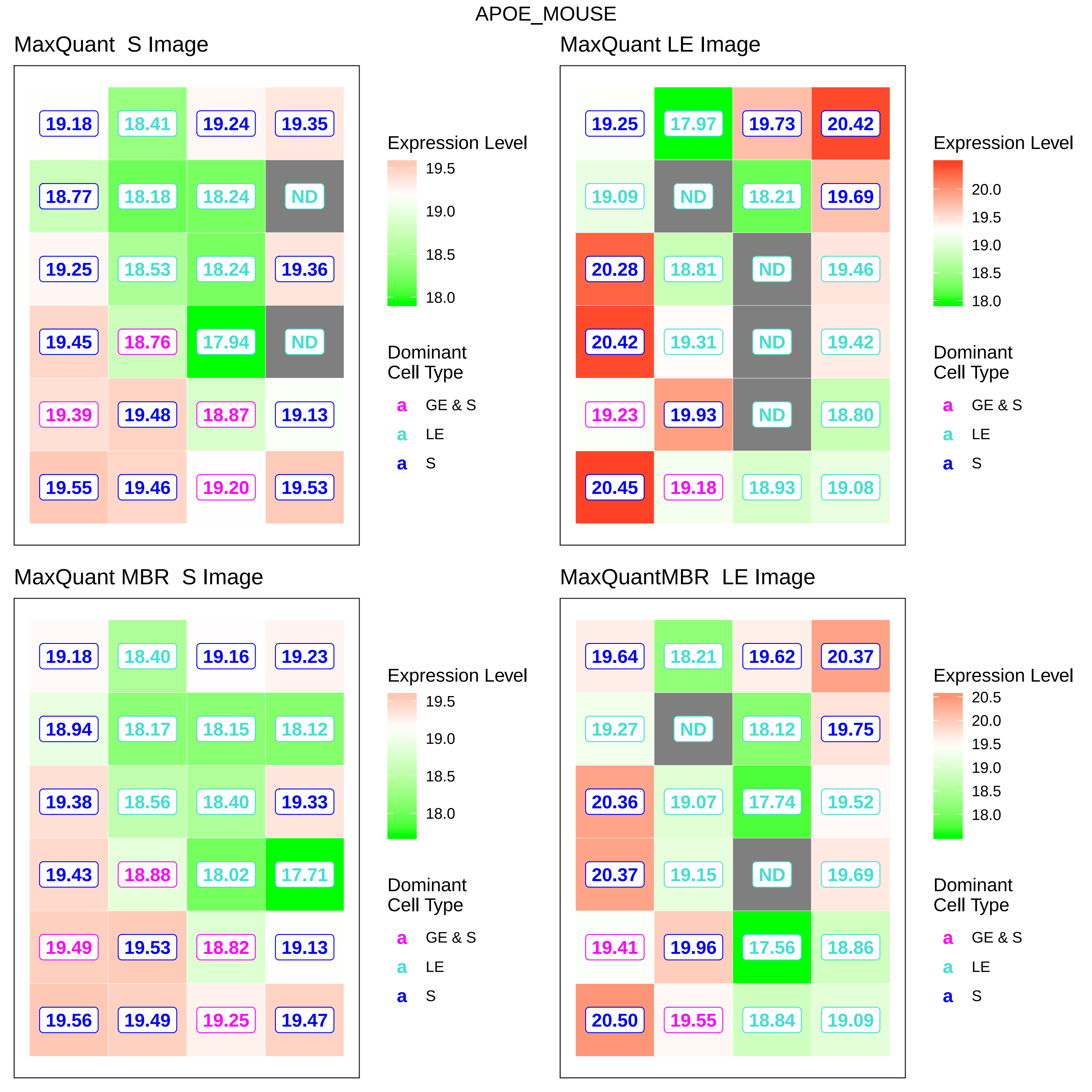
APOE_MOUSE
MaxQuant S Image
19.18 18.41 19.24 19.35
18.77 18.18 18.24 ND
19.25 18.53 18.24 19.36
19.45 18.76 17.94 ND
19.39 19.48 18.87 19.13
19.55 19.46 19.20 19.53
Expression Level
19.5
19.0
18.5
18.0
Dominant Cell Type
a GE & S
a LE
a S
MaxQuant LE Image
19.25 17.97 19.73 20.42
19.09 ND 18.21 19.69
20.28 18.81 ND 19.46
20.42 19.31 ND 19.42
19.23 19.93 ND 18.80
20.45 19.18 18.93 19.08
Expression Level
20.0
19.5
19.0
18.5
18.0
Dominant Cell Type
a GE & S
a LE
a S
MaxQuant MBR S Image
19.18 18.40 19.16 19.23
18.94 18.17 18.15 18.12
19.38 18.56 18.40 19.33
19.43 18.88 18.02 17.71
19.49 19.53 18.82 19.13
19.56 19.49 19.25 19.47
Expression Level
19.5
19.0
18.5
18.0
Dominant Cell Type
a GE & S
a LE
a S
MaxQuantMBR LE Image
19.64 18.21 19.62 20.37
19.27 ND 18.12 19.75
20.36 19.07 17.74 19.52
20.37 19.15 ND 19.69
19.41 19.96 17.56 18.86
20.50 19.55 18.84 19.09
Expression Level
20.5
20.0
19.5
19.0
18.5
18.0
Dominant Cell Type
a GE & S
a LE
a S

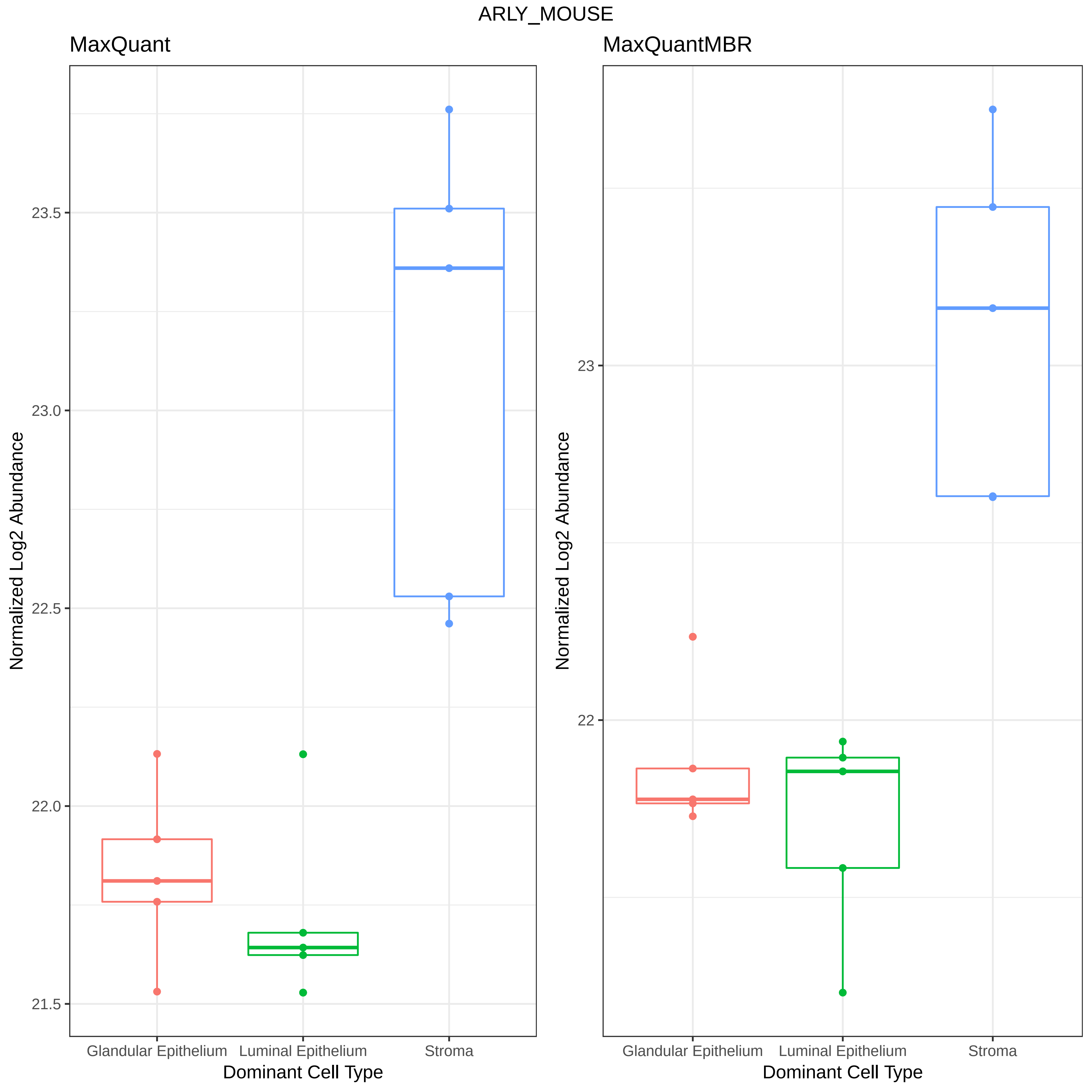
ARLY_MOUSE
MaxQuant
MaxQuantMBR
Normalized Log2 Abundance
23.5
23.0
22.5
22.0
21.5
23
22
Glandular Epithelium
Luminal Epithelium
Stroma
Dominant Cell Type

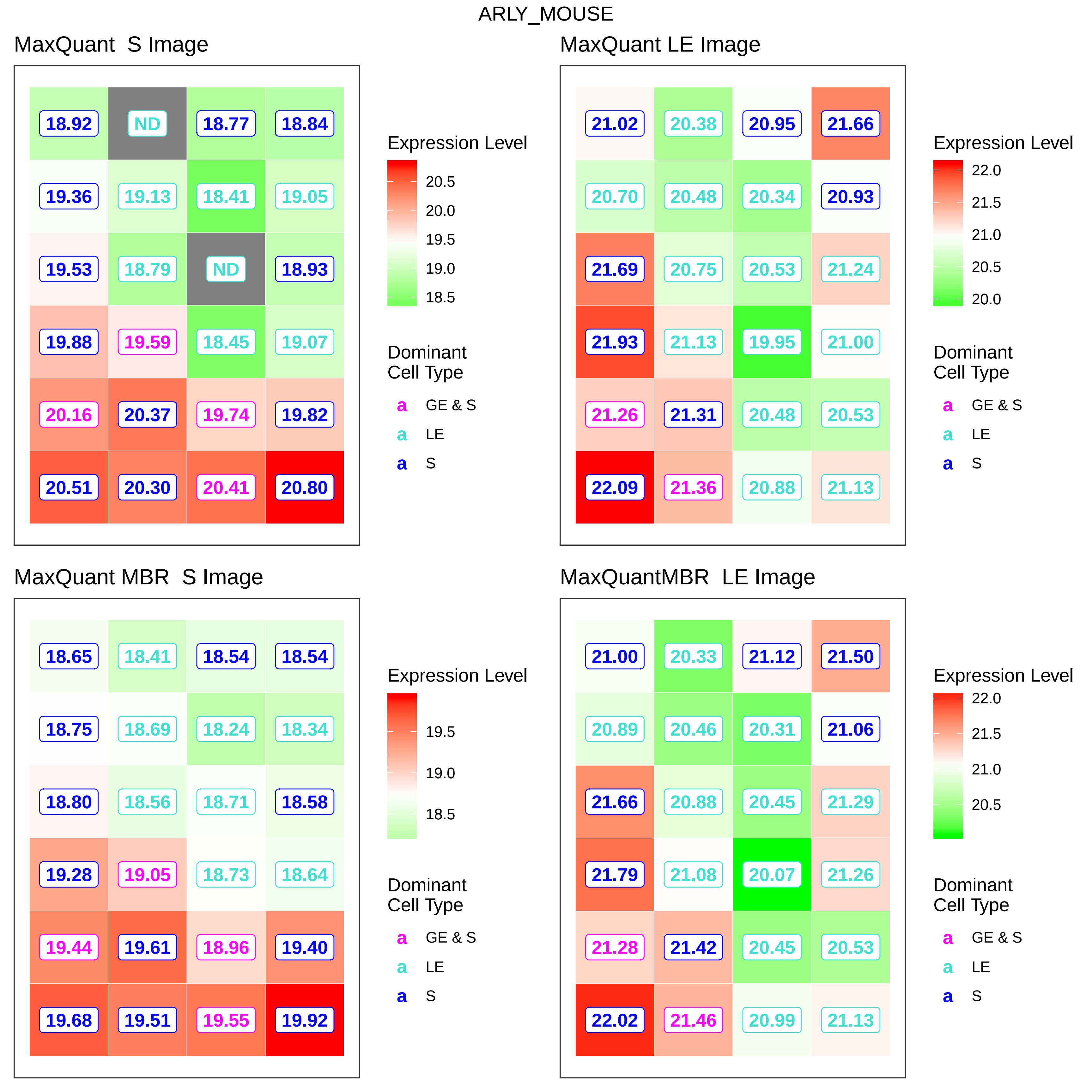
ARLY_MOUSE
MaxQuant S Image
18.92 ND 18.77 18.84
19.36 19.13 18.41 19.05
19.53 18.79 ND 18.93
19.88 19.59 18.45 19.07
20.16 20.37 19.74 19.82
20.51 20.30 20.41 20.80
Expression Level
20.5
20.0
19.5
19.0
18.5
Dominant Cell Type
a GE & S
a LE
a S
MaxQuant LE Image
21.02 20.38 20.95 21.66
20.70 20.48 20.34 20.93
21.69 20.75 20.53 21.24
21.93 21.13 19.95 21.00
21.26 21.31 20.48 20.53
22.09 21.36 20.88 21.13
Expression Level
22.0
21.5
21.0
20.5
20.0
Dominant Cell Type
a GE & S
a LE
a S
MaxQuant MBR S Image
18.65 18.41 18.54 18.54
18.75 18.69 18.24 18.34
18.80 18.56 18.71 18.58
19.28 19.05 18.73 18.64
19.44 19.61 18.96 19.40
19.68 19.51 19.55 19.92
Expression Level
19.5
19.0
18.5
Dominant Cell Type
a GE & S
a LE
a S
MaxQuantMBR LE Image
21.00 20.33 21.12 21.50
20.89 20.46 20.31 21.06
21.66 20.88 20.45 21.29
21.79 21.08 20.07 21.26
21.28 21.42 20.45 20.53
22.02 21.46 20.99 21.13
Expression Level
22.0
21.5
21.0
20.5
Dominant Cell Type
a GE & S
a LE
a S

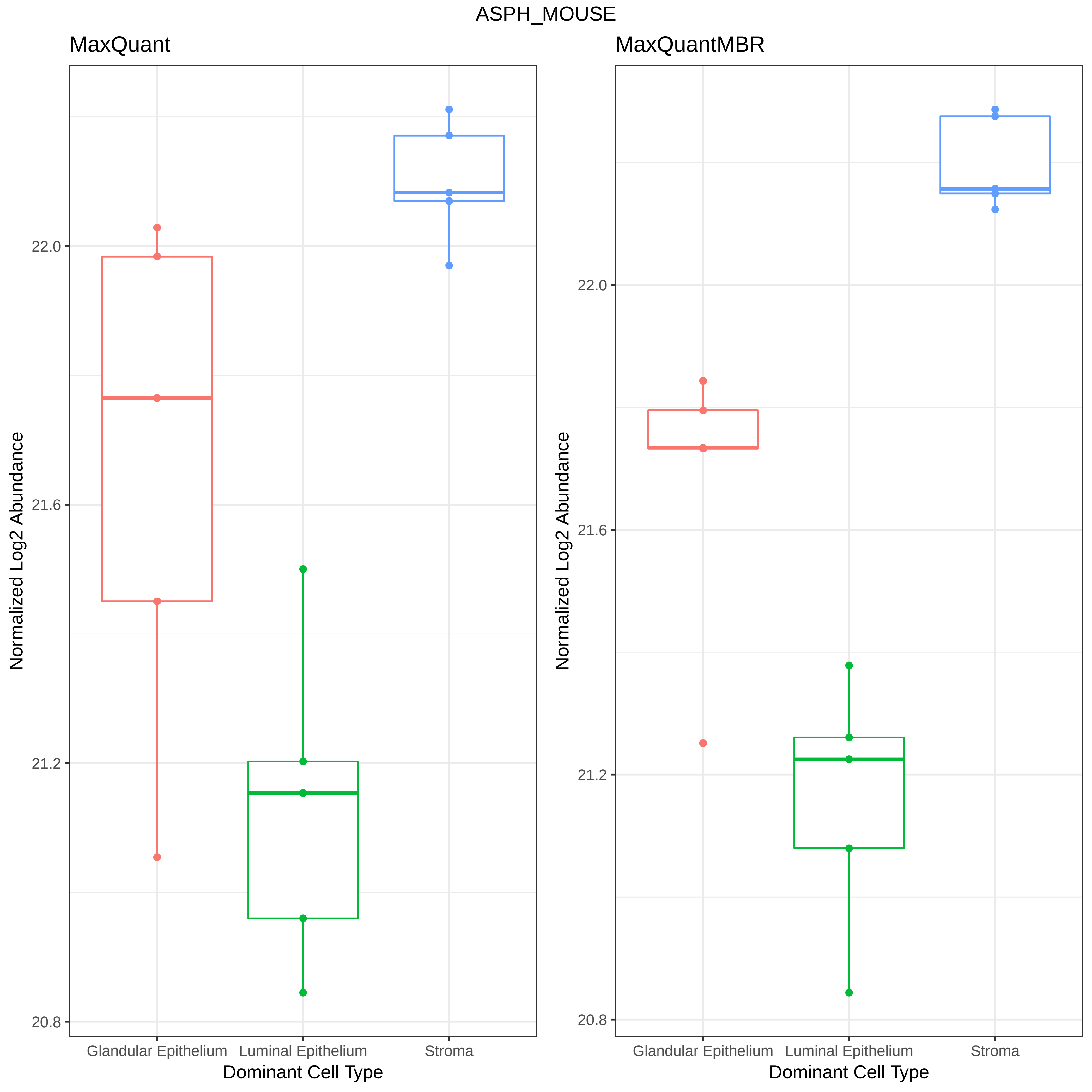
ASPH_MOUSE
MaxQuant
MaxQuantMBR
Normalized Log2 Abundance
22.0
21.6
21.2
20.8
Glandular Epithelium
Luminal Epithelium
Stroma
Dominant Cell Type

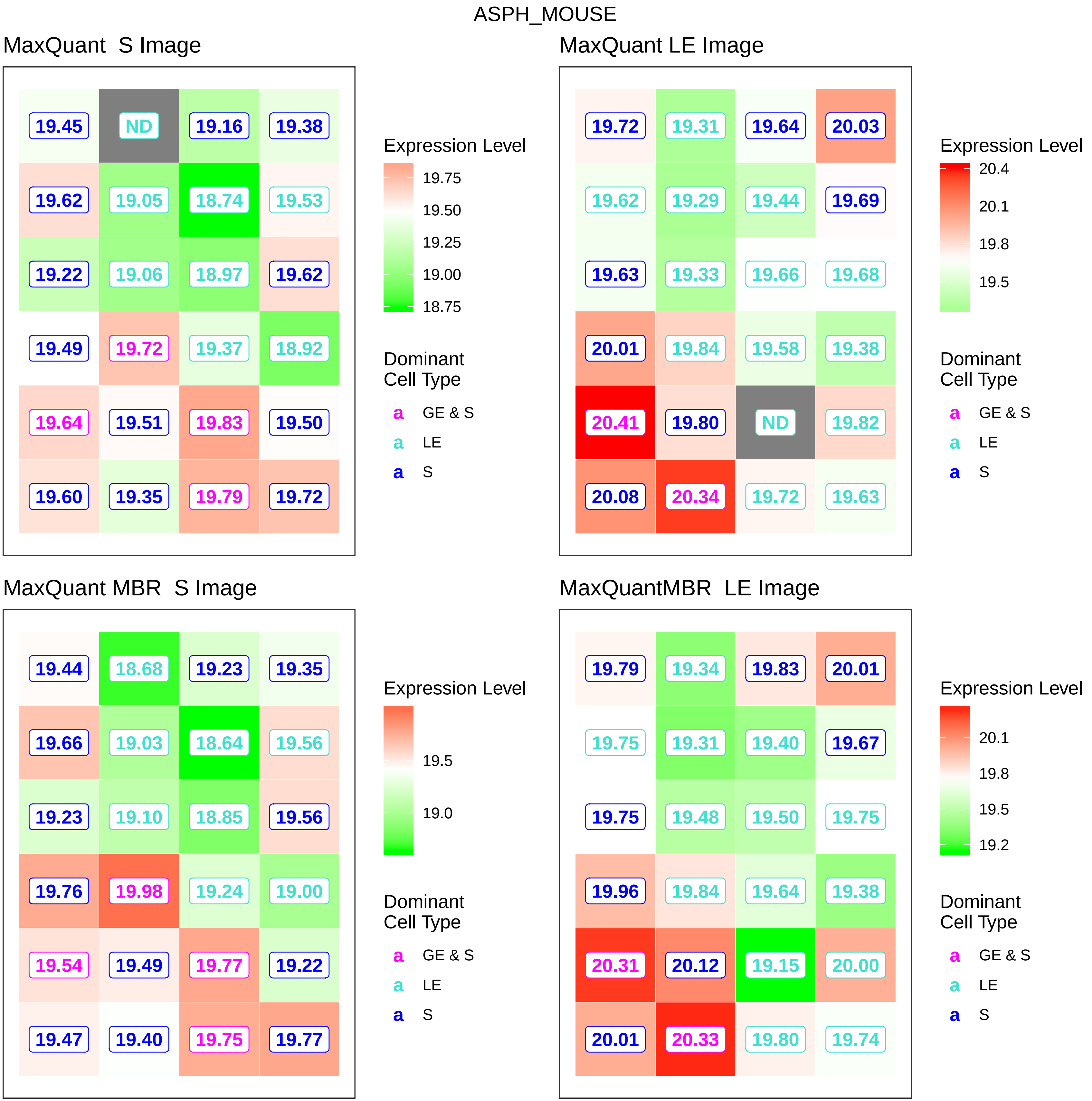
ASPH_MOUSE
MaxQuant S Image
19.45 ND 19.16 19.38
19.62 19.05 18.74 19.53
19.22 19.06 18.97 19.62
19.49 19.72 19.37 18.92
19.64 19.51 19.83 19.50
19.60 19.35 19.79 19.72
Expression Level
19.75
19.50
19.25
19.00
18.75
Dominant Cell Type
a GE & S
a LE
a S
MaxQuant LE Image
19.72 19.31 19.64 20.03
19.62 19.29 19.44 19.69
19.63 19.33 19.66 19.68
20.01 19.84 19.58 19.38
20.41 19.80 ND 19.82
20.08 20.34 19.72 19.63
Expression Level
20.4
20.1
19.8
19.5
Dominant Cell Type
a GE & S
a LE
a S
MaxQuant MBR S Image
19.44 18.68 19.23 19.35
19.66 19.03 18.64 19.56
19.23 19.10 18.85 19.56
19.76 19.98 19.24 19.00
19.54 19.49 19.77 19.22
19.47 19.40 19.75 19.77
Expression Level
19.5
19.0
Dominant Cell Type
a GE & S
a LE
a S
MaxQuantMBR LE Image
19.79 19.34 19.83 20.01
19.75 19.31 19.40 19.67
19.75 19.48 19.50 19.75
19.96 19.84 19.64 19.38
20.31 20.12 19.15 20.00
20.01 20.33 19.80 19.74
Expression Level
20.1
19.8
19.5
19.2
Dominant Cell Type
a GE & S
a LE
a S

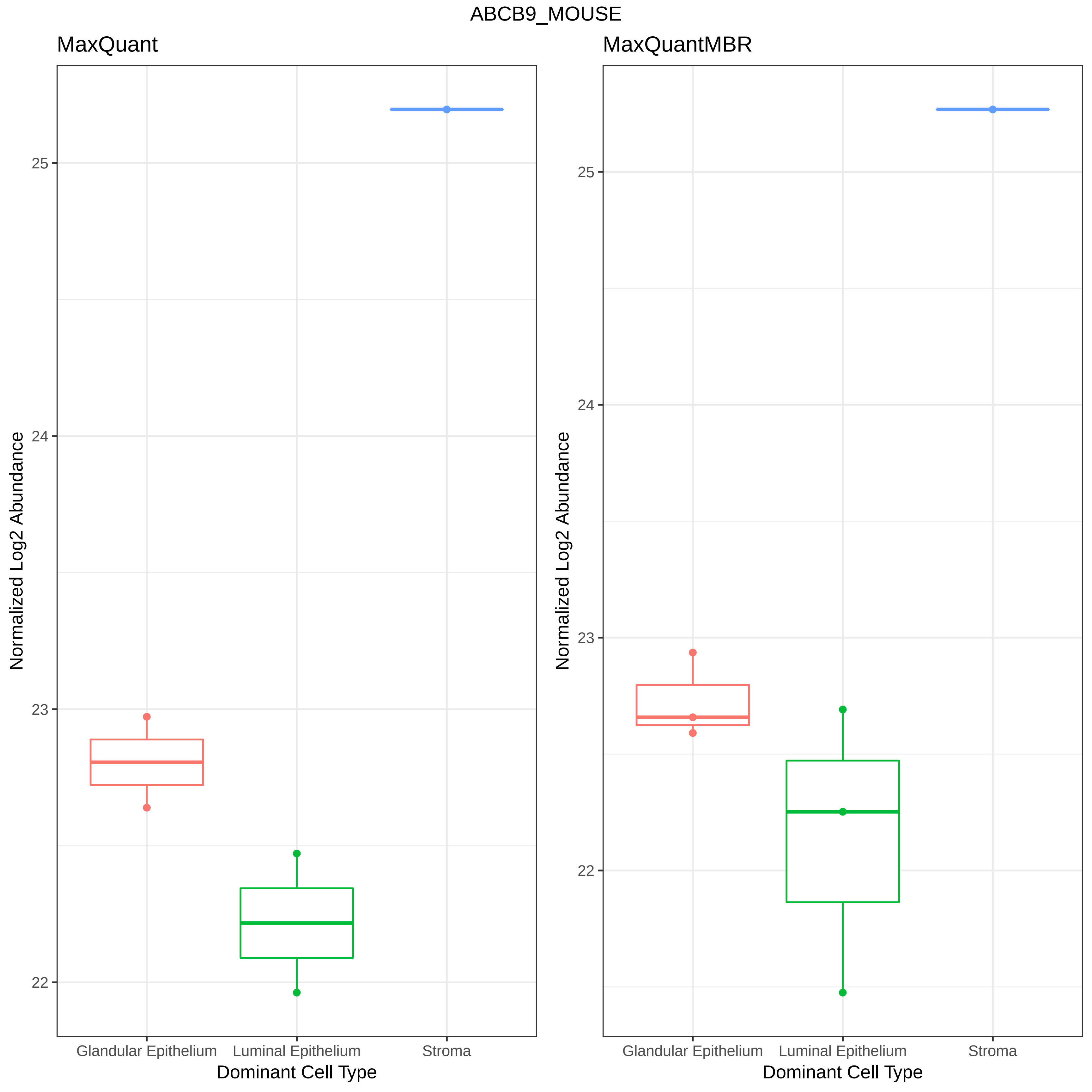

ABCB9_MOUSE
MaxQuant
MaxQuantMBR
Normalized Log2 Abundance
25
24
23
22
Glandular Epithelium
Luminal Epithelium
Stroma
Dominant Cell Type

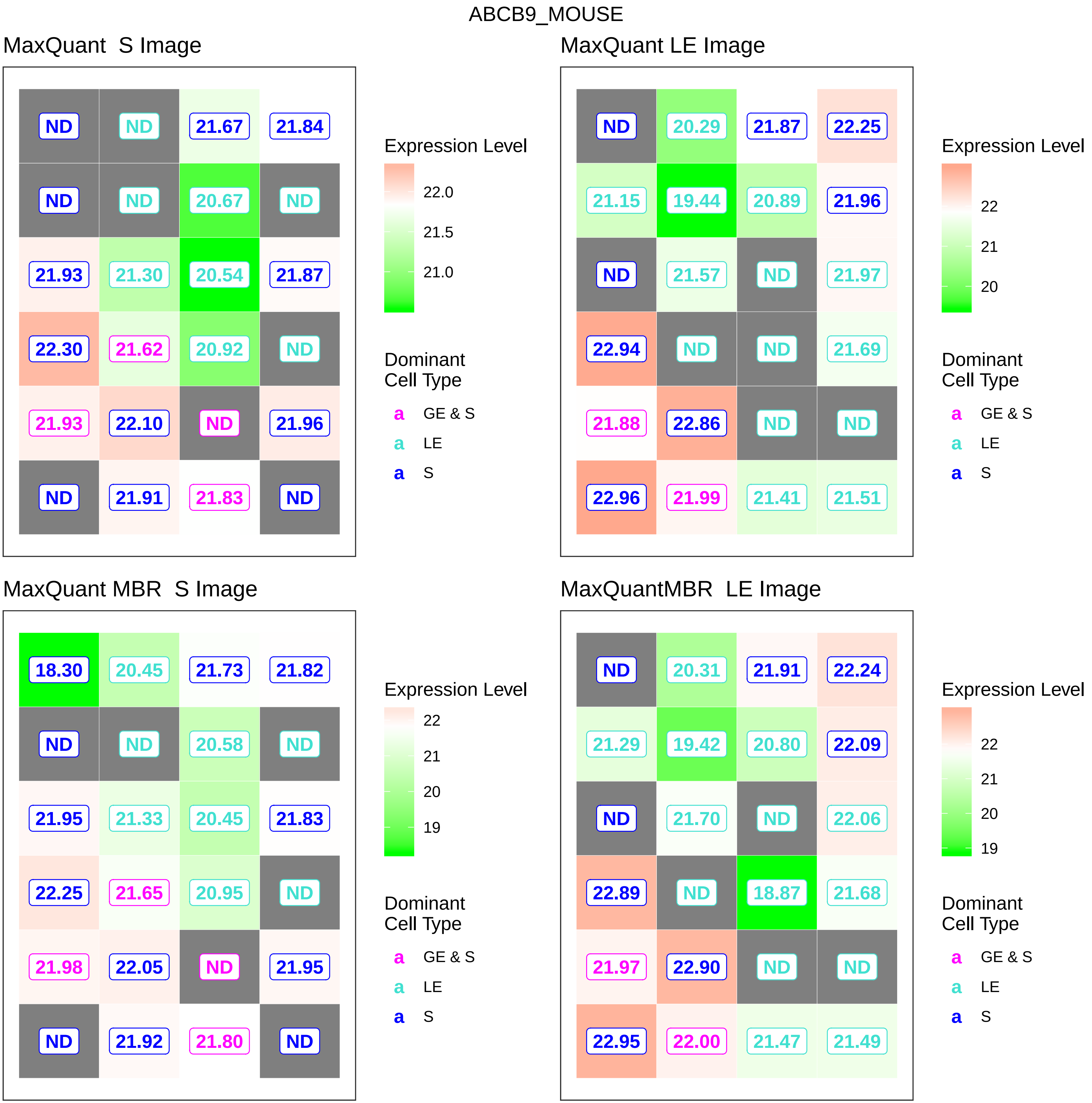
ABCB9_MOUSE
MaxQuant S Image
ND ND 21.67 21.84
ND ND 20.67 ND
21.93 21.30 20.54 21.87
22.30 21.62 20.92 ND
21.93 22.10 ND 21.96
ND 21.91 21.83 ND
Expression Level
22.0
21.5
21.0
Dominant Cell Type
a GE & S
a LE
a S
MaxQuant LE Image
ND 20.29 21.87 22.25
21.15 19.44 20.89 21.96
ND 21.57 ND 21.97
22.94 ND ND 21.69
21.88 22.86 ND ND
22.96 21.99 21.41 21.51
Expression Level
22
21
20
Dominant Cell Type
a GE & S
a LE
a S
MaxQuant MBR S Image
18.30 20.45 21.73 21.82
ND ND 20.58 ND
21.95 21.33 20.45 21.83
22.25 21.65 20.95 ND
21.98 22.05 ND 21.95
ND 21.92 21.80 ND
Expression Level
22
21
20
19
Dominant Cell Type
a GE & S
a LE
a S
MaxQuantMBR LE Image
ND 20.31 21.91 22.24
21.29 19.42 20.80 22.09
ND 21.70 ND 22.06
22.89 ND 18.87 21.68
21.97 22.90 ND ND
22.95 22.00 21.47 21.49
Expression Level
22
21
20
19
Dominant Cell Type
a GE & S
a LE
a S

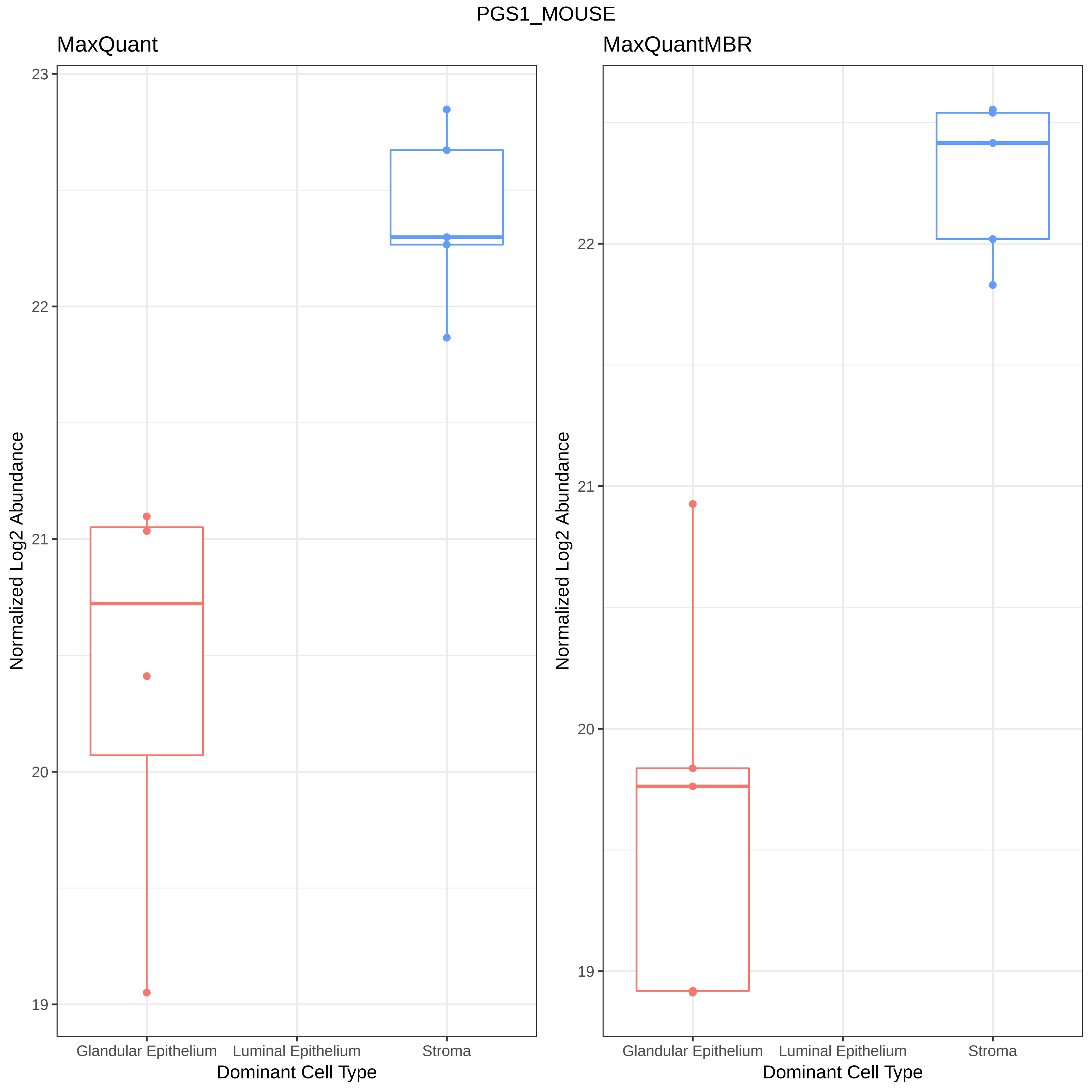
PGS1_MOUSE
MaxQuant
MaxQuantMBR
Normalized Log2 Abundance
23
22
21
20
19
Glandular Epithelium
Luminal Epithelium
Stroma
Dominant Cell Type

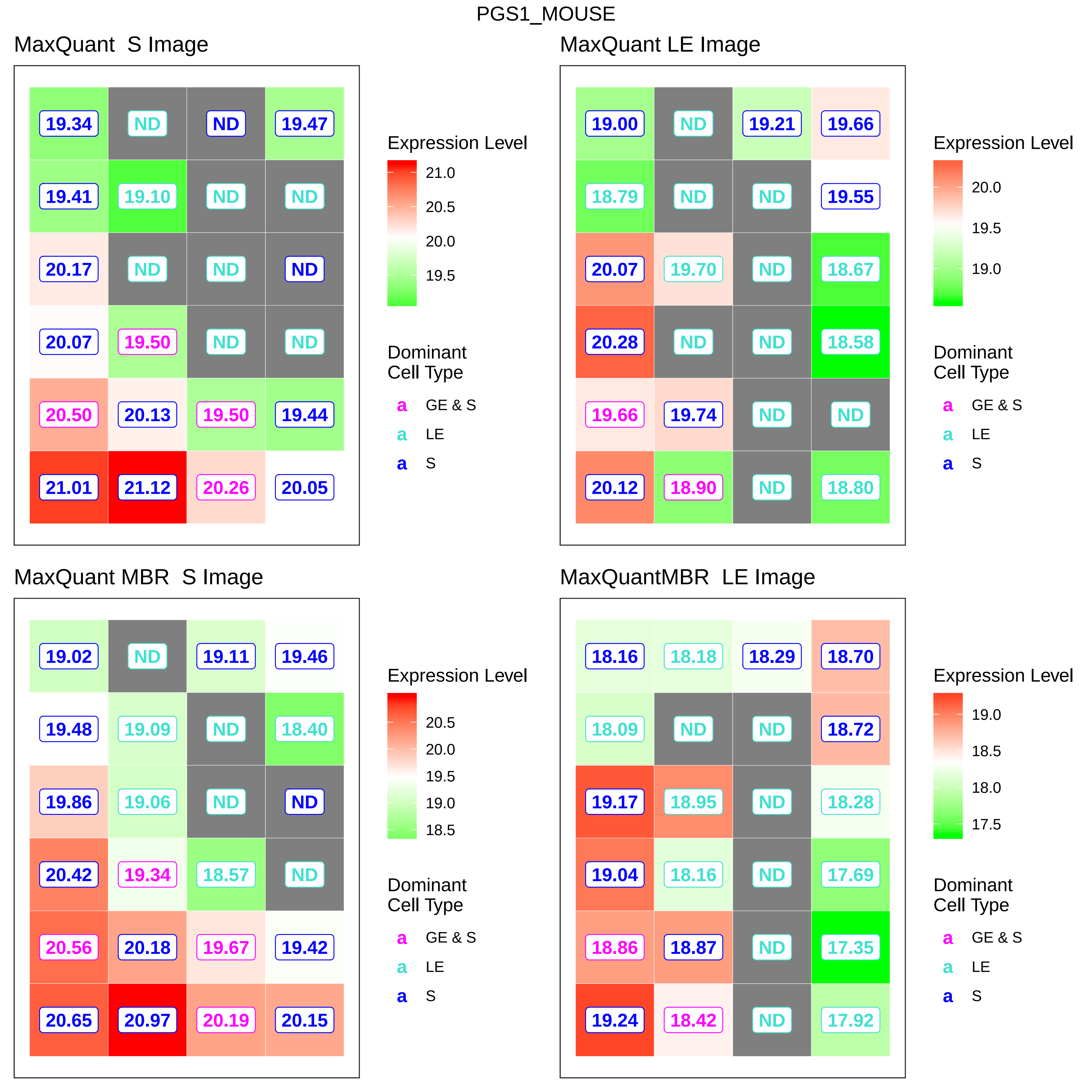
PGS1_MOUSE
MaxQuant S Image
19.34 ND ND 19.47
19.41 19.10 ND ND
20.17 ND ND ND
20.07 19.50 ND ND
20.50 20.13 19.50 19.44
21.01 21.12 20.26 20.05
Expression Level
21.0
20.5
20.0
19.5
Dominant Cell Type
a GE & S
a LE
a S
MaxQuant LE Image
19.00 ND 19.21 19.66
18.79 ND ND 19.55
20.07 19.70 ND 18.67
20.28 ND ND 18.58
19.66 19.74 ND ND
20.12 18.90 ND 18.80
Expression Level
20.0
19.5
19.0
Dominant Cell Type
a GE & S
a LE
a S
MaxQuant MBR S Image
19.02 ND 19.11 19.46
19.48 19.09 ND 18.40
19.86 19.06 ND ND
20.42 19.34 18.57 ND
20.56 20.18 19.67 19.42
20.65 20.97 20.19 20.15
Expression Level
20.5
20.0
19.5
19.0
18.5
Dominant Cell Type
a GE & S
a LE
a S
MaxQuantMBR LE Image
18.16 18.18 18.29 18.70
18.09 ND ND 18.72
19.17 18.95 ND 18.28
19.04 18.16 ND 17.69
18.86 18.87 ND 17.35
19.24 18.42 ND 17.92
Expression Level
19.0
18.5
18.0
17.5
Dominant Cell Type
a GE & S
a LE
a S

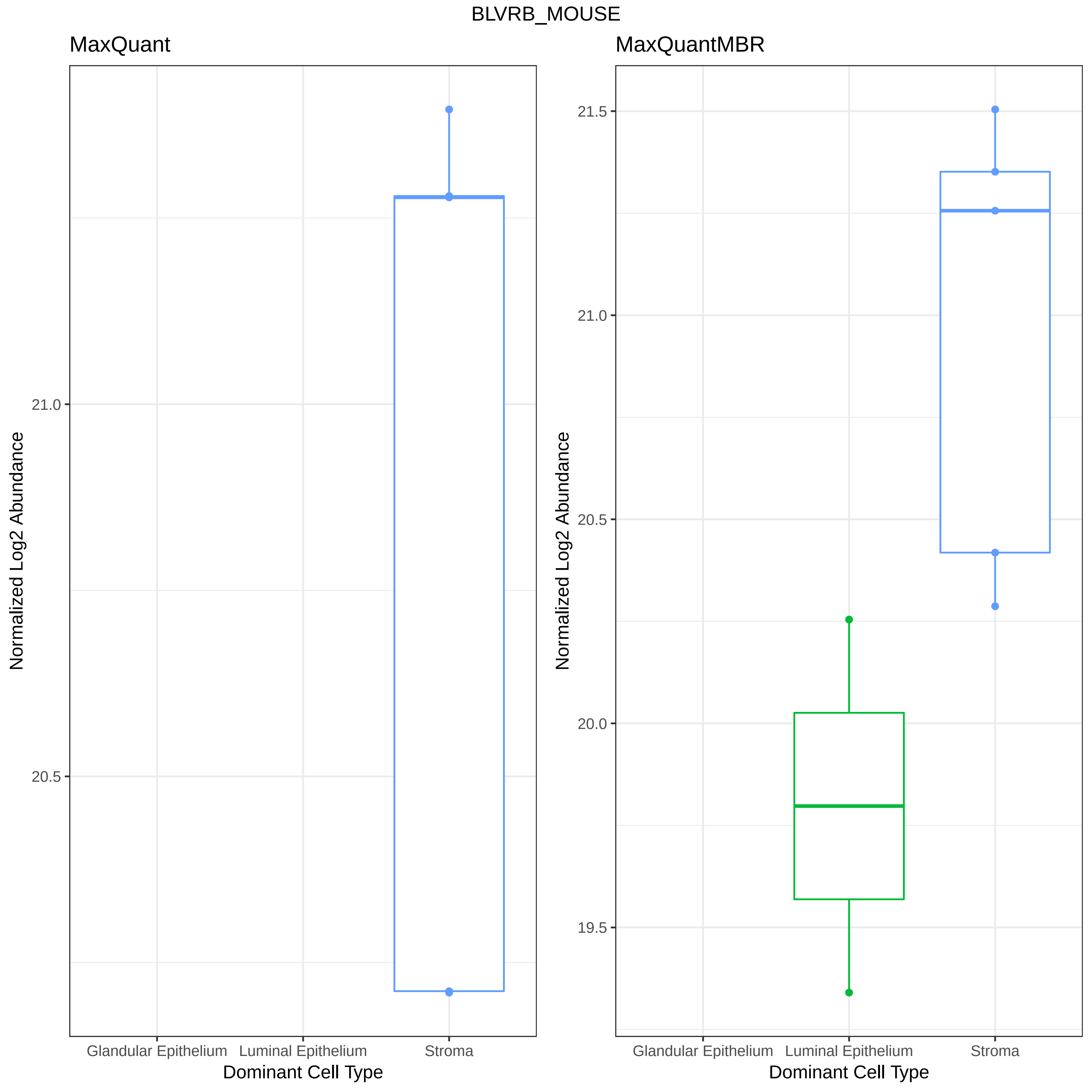
BLVRB_MOUSE
MaxQuant
MaxQuantMBR
21.0
20.5
21.5
21.0
20.5
20.0
19.5
Normalized Log2 Abundance
Normalized Log2 Abundance
Glandular Epithelium
Luminal Epithelium
Stroma
Glandular Epithelium
Luminal Epithelium
Stroma
Dominant Cell Type
Dominant Cell Type

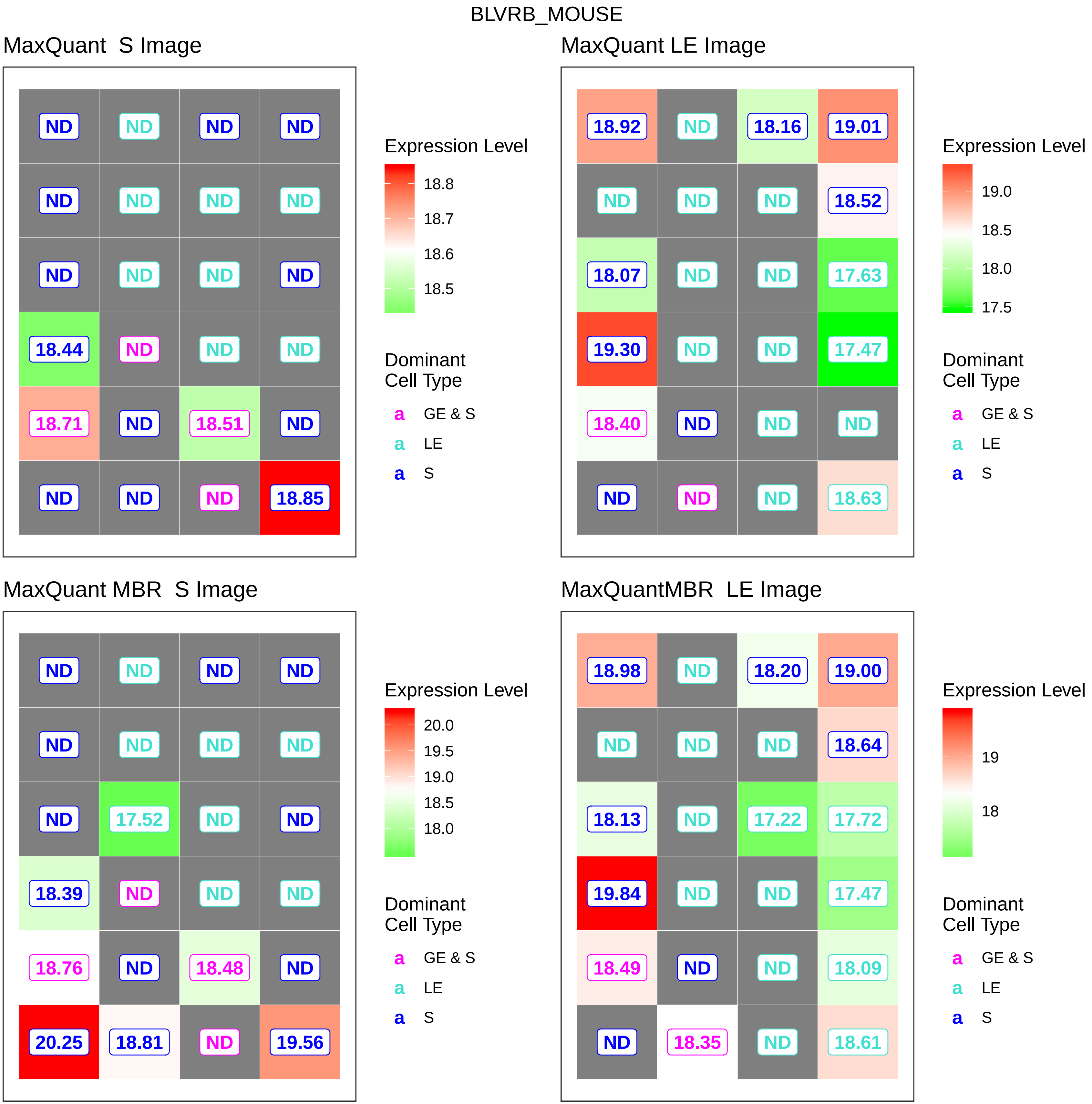
BLVRB_MOUSE
MaxQuant S Image
ND ND ND ND
ND ND ND ND
ND ND ND ND
18.44 ND ND ND
18.71 ND 18.51 ND
ND ND ND 18.85
Expression Level
18.8
18.7
18.6
18.5
Dominant Cell Type
a GE & S
a LE
a S
MaxQuant LE Image
18.92 ND 18.16 19.01
ND ND ND 18.52
18.07 ND ND 17.63
19.30 ND ND 17.47
18.40 ND ND ND
ND ND ND 18.63
Expression Level
19.0
18.5
18.0
17.5
Dominant Cell Type
a GE & S
a LE
a S
MaxQuant MBR S Image
ND ND ND ND
ND ND ND ND
ND 17.52 ND ND
18.39 ND ND ND
18.76 ND 18.48 ND
20.25 18.81 ND 19.56
Expression Level
20.0
19.5
19.0
18.5
18.0
Dominant Cell Type
a GE & S
a LE
a S
MaxQuantMBR LE Image
18.98 ND 18.20 19.00
ND ND ND 18.64
18.13 ND 17.22 17.72
19.84 ND ND 17.47
18.49 ND ND 18.09
ND 18.35 ND 18.61
Expression Level
19
18
Dominant Cell Type
a GE & S
a LE
a S

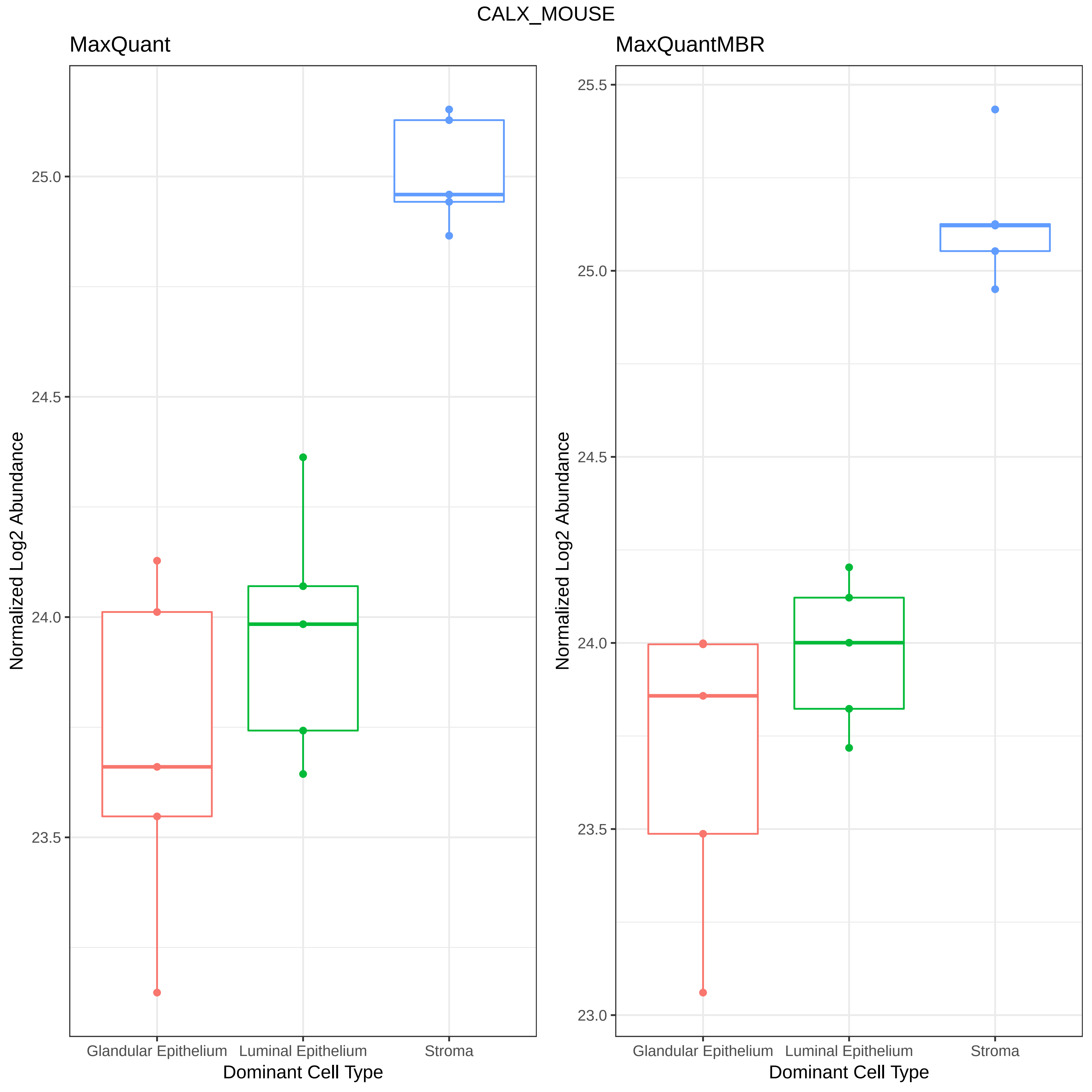

CALX_MOUSE
MaxQuant
MaxQuantMBR
Normalized Log2 Abundance
25.0
24.5
24.0
23.5
25.5
23.0
Glandular Epithelium
Luminal Epithelium
Stroma
Dominant Cell Type

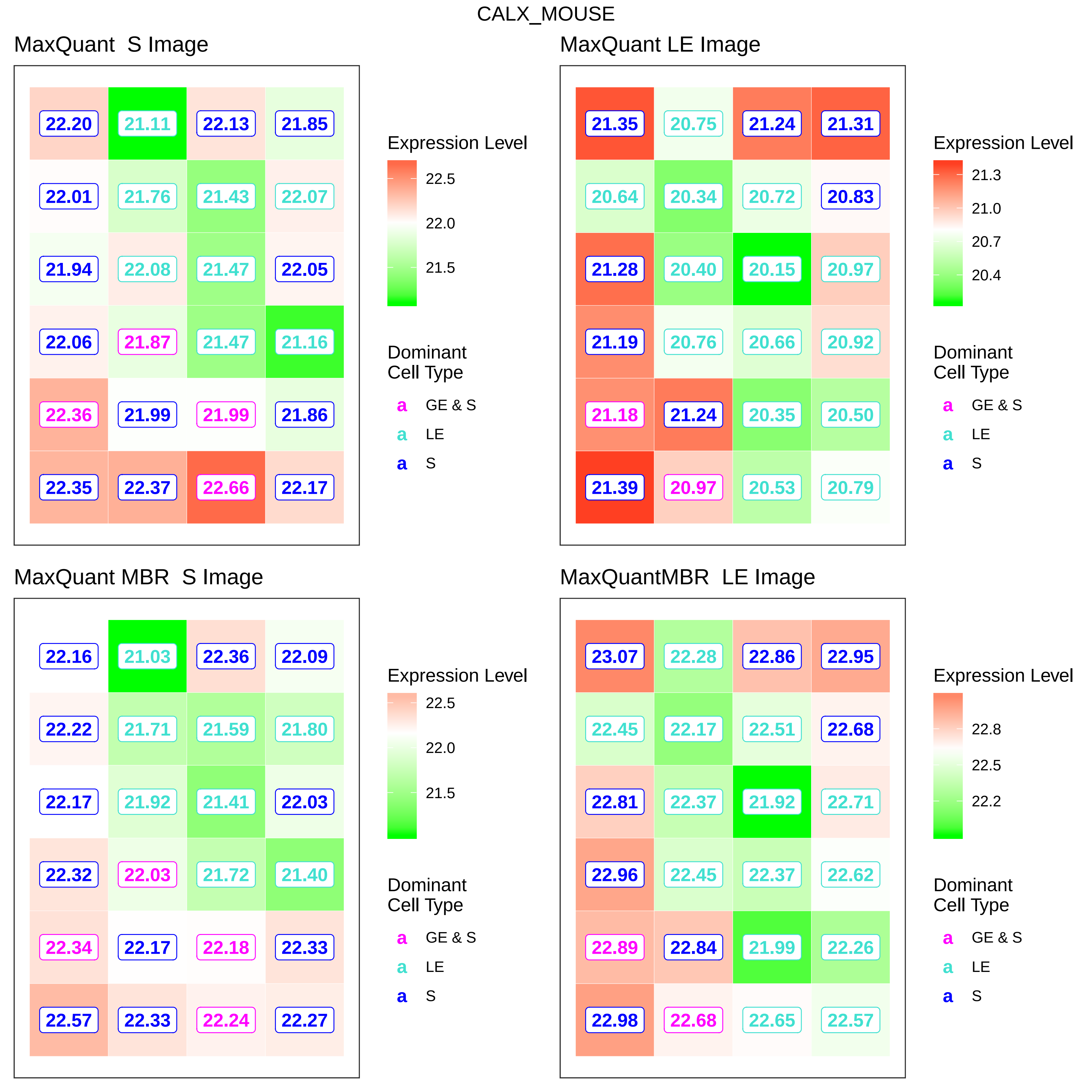
CALX_MOUSE
MaxQuant S Image
22.20 21.11 22.13 21.85
22.01 21.76 21.43 22.07
21.94 22.08 21.47 22.05
22.06 21.87 21.47 21.16
22.36 21.99 21.99 21.86
22.35 22.37 22.66 22.17
Expression Level
22.5
22.0
21.5
Dominant Cell Type
a GE & S
a LE
a S
MaxQuant LE Image
21.35 20.75 21.24 21.31
20.64 20.34 20.72 20.83
21.28 20.40 20.15 20.97
21.19 20.76 20.66 20.92
21.18 21.24 20.35 20.50
21.39 20.97 20.53 20.79
Expression Level
21.3
21.0
20.7
20.4
Dominant Cell Type
a GE & S
a LE
a S
MaxQuant MBR S Image
22.16 21.03 22.36 22.09
22.22 21.71 21.59 21.80
22.17 21.92 21.41 22.03
22.32 22.03 21.72 21.40
22.34 22.17 22.18 22.33
22.57 22.33 22.24 22.27
Expression Level
22.5
22.0
21.5
Dominant Cell Type
a GE & S
a LE
a S
MaxQuantMBR LE Image
23.07 22.28 22.86 22.95
22.45 22.17 22.51 22.68
22.81 22.37 21.92 22.71
22.96 22.45 22.37 22.62
22.89 22.84 21.99 22.26
22.98 22.68 22.65 22.57
Expression Level
22.8
22.5
22.2
Dominant Cell Type
a GE & S
a LE
a S

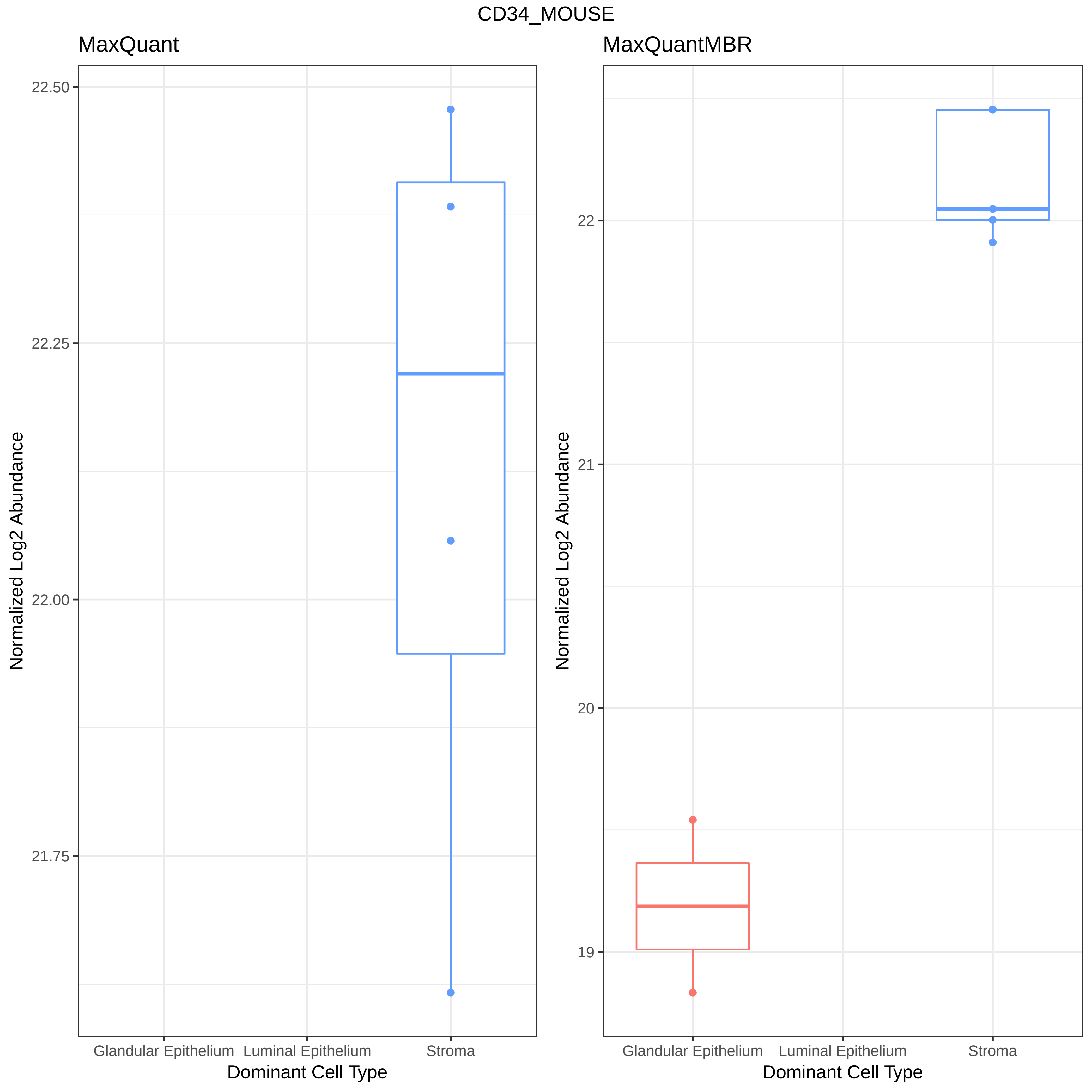

CD34_MOUSE
MaxQuant
MaxQuantMBR
Normalized Log2 Abundance
22.50
22.25
22.00
21.75
22
21
20
19
Glandular Epithelium
Luminal Epithelium
Stroma
Dominant Cell Type

CD34_MOUSE

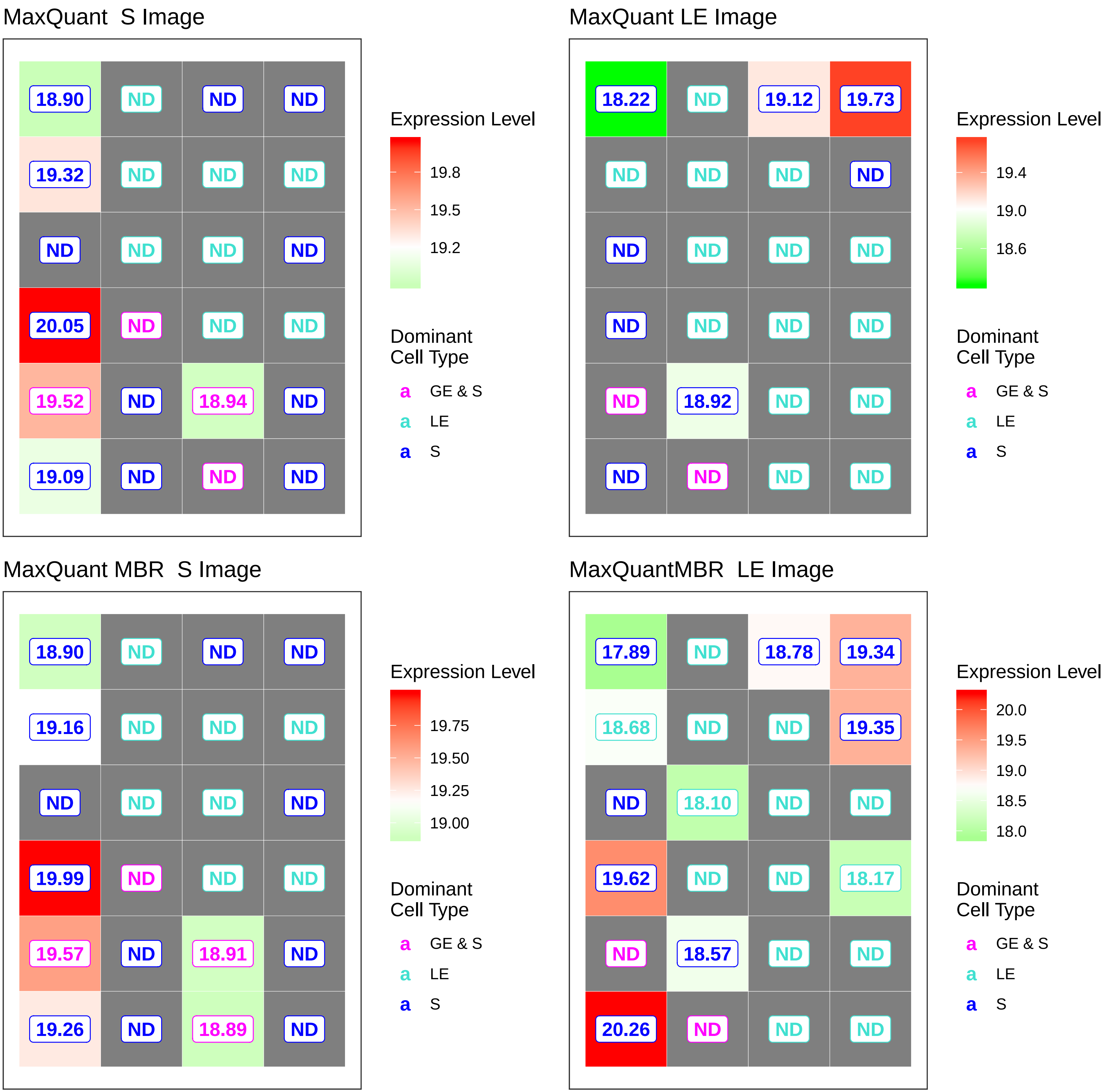
MaxQuant S Image
18.90 ND ND ND
19.32 ND ND ND
ND ND ND ND
20.05 ND ND ND
19.52 ND 18.94 ND
19.09 ND ND ND
Expression Level
19.8
19.5
19.2
Dominant Cell Type
a GE & S
a LE
a S
MaxQuant LE Image
18.22 ND 19.12 19.73
ND ND ND ND
ND ND ND ND
ND ND ND ND
ND 18.92 ND ND
ND ND ND ND
Expression Level
19.4
19.0
18.6
Dominant Cell Type
a GE & S
a LE
a S
MaxQuant MBR S Image
18.90 ND ND ND
19.16 ND ND ND
ND ND ND ND
19.99 ND ND ND
19.57 ND 18.91 ND
19.26 ND 18.89 ND
Expression Level
19.75
19.50
19.25
19.00
Dominant Cell Type
a GE & S
a LE
a S
MaxQuantMBR LE Image
17.89 ND 18.78 19.34
18.68 ND ND 19.35
ND 18.10 ND ND
19.62 ND ND 18.17
ND 18.57 ND ND
20.26 ND ND ND
Expression Level
20.0
19.5
19.0
18.5
18.0
Dominant Cell Type
a GE & S
a LE
a S

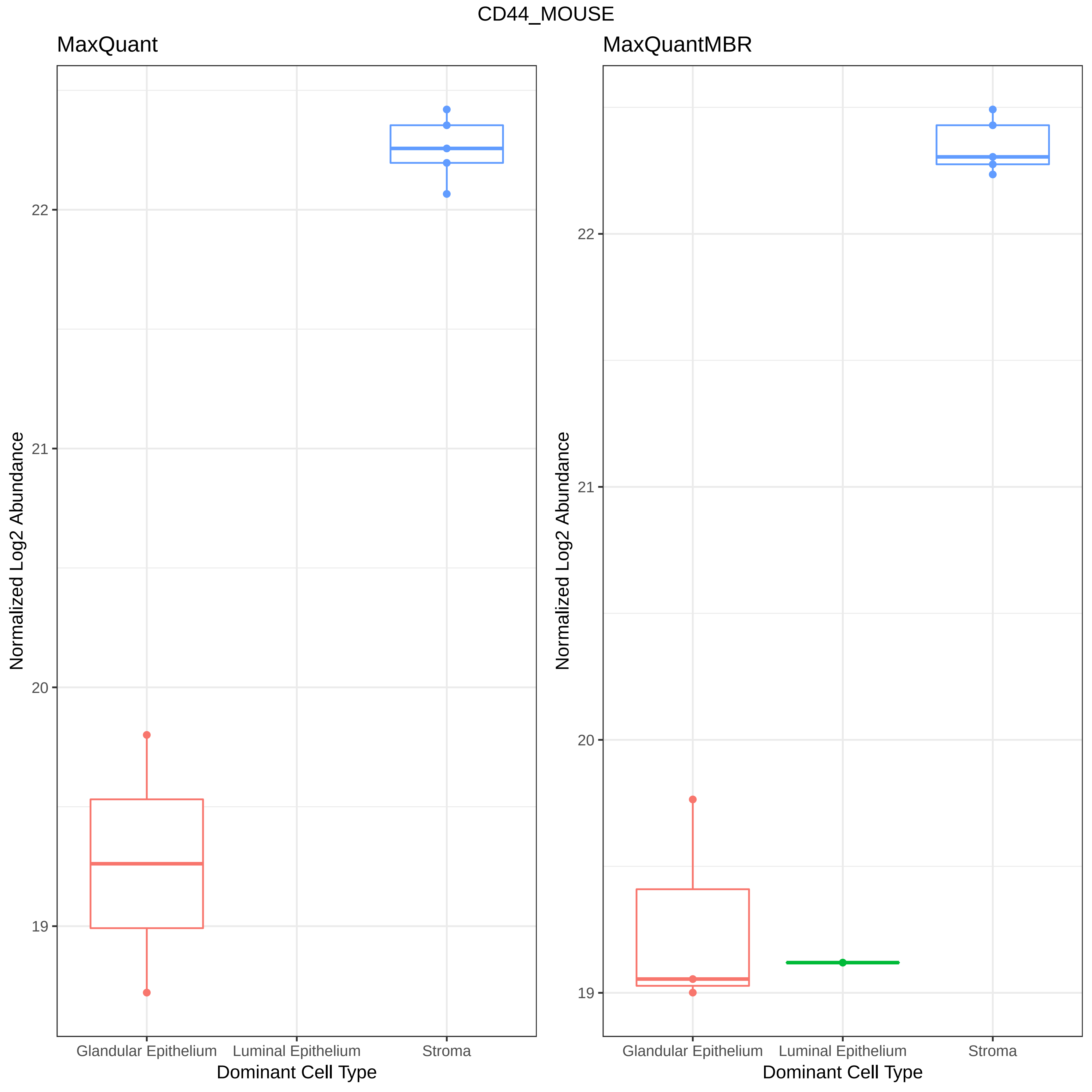
CD44_MOUSE
MaxQuant
MaxQuantMBR
Normalized Log2 Abundance
22
21
20
19
Glandular Epithelium
Luminal Epithelium
Stroma
Dominant Cell Type

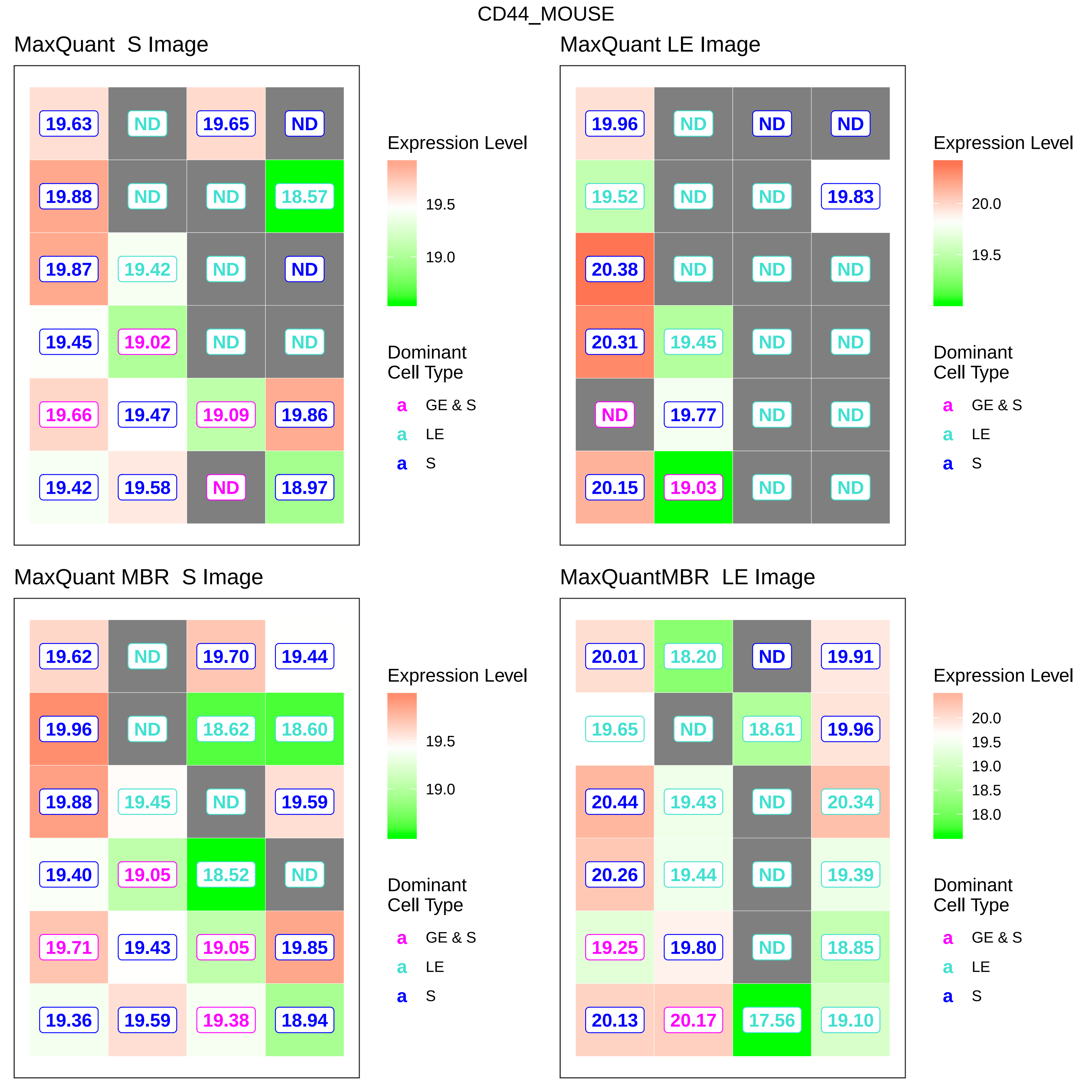
CD44_MOUSE
MaxQuant S Image
19.63
ND
19.65
ND
19.88
ND
ND
18.57
19.87
19.42
ND
ND
19.45
19.02
ND
ND
19.66
19.47
19.09
19.86
19.42
19.58
ND
18.97
Expression Level
19.5
19.0
Dominant
Cell Type
a GE & S
a LE
a S
MaxQuant LE Image
19.96
ND
ND
ND
19.52
ND
ND
19.83
20.38
ND
ND
ND
20.31
19.45
ND
ND
ND
19.77
ND
ND
20.15
19.03
ND
ND
Expression Level
20.0
19.5
Dominant
Cell Type
a GE & S
a LE
a S
MaxQuant MBR S Image
19.62
ND
19.70
19.44
19.96
ND
18.62
18.60
19.88
19.45
ND
19.59
19.40
19.05
18.52
ND
19.71
19.43
19.05
19.85
19.36
19.59
19.38
18.94
Expression Level
19.5
19.0
Dominant
Cell Type
a GE & S
a LE
a S
MaxQuantMBR LE Image
20.01
18.20
ND
19.91
19.65
ND
18.61
19.96
20.44
19.43
ND
20.34
20.26
19.44
ND
19.39
19.25
19.80
ND
18.85
20.13
20.17
17.56
19.10
Expression Level
20.0
19.5
19.0
18.5
18.0
Dominant
Cell Type
a GE & S
a LE
a S

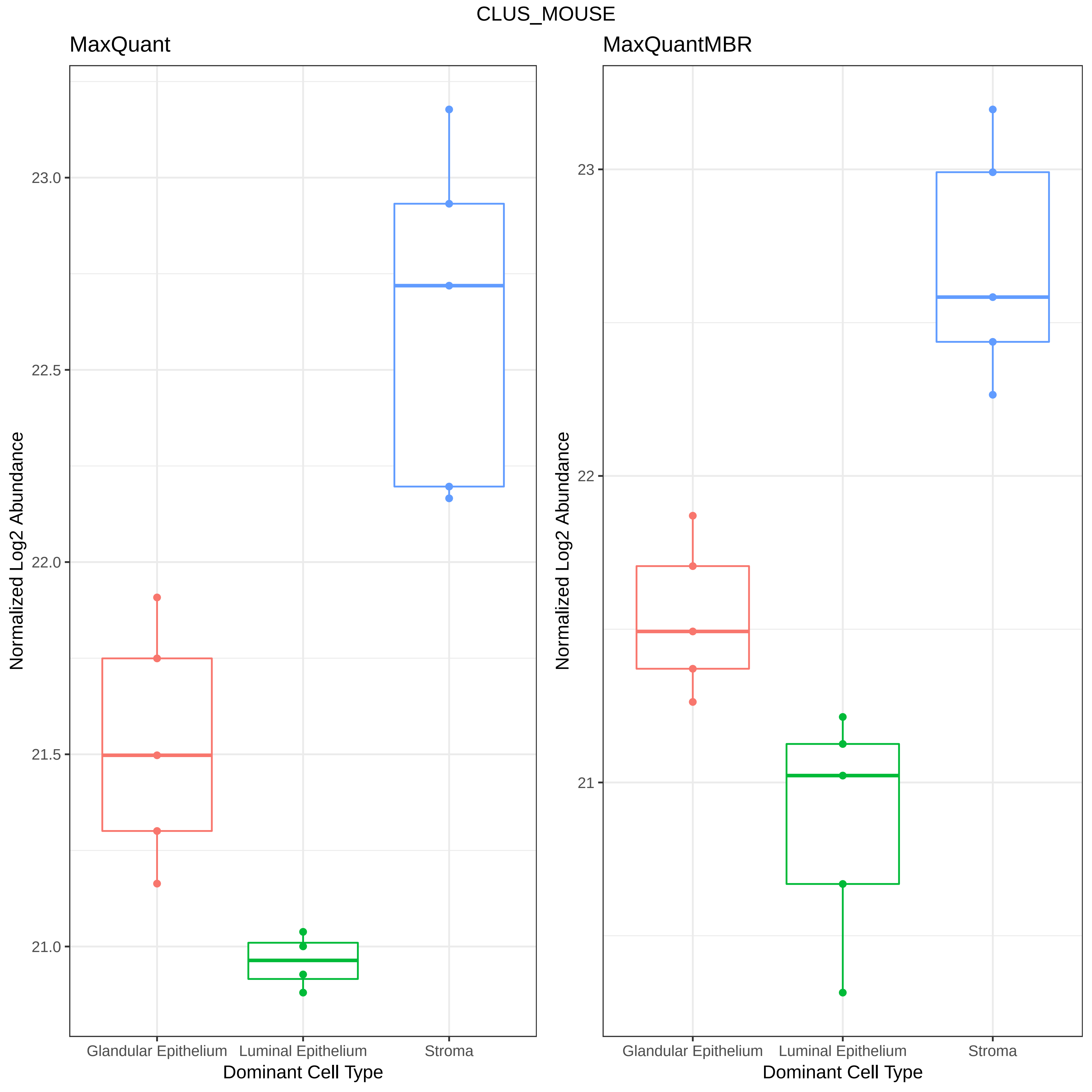

CLUS_MOUSE
MaxQuant
MaxQuantMBR
Normalized Log2 Abundance
23.0
22.5
22.0
21.5
21.0
23
22
21
Glandular Epithelium
Luminal Epithelium
Stroma
Dominant Cell Type

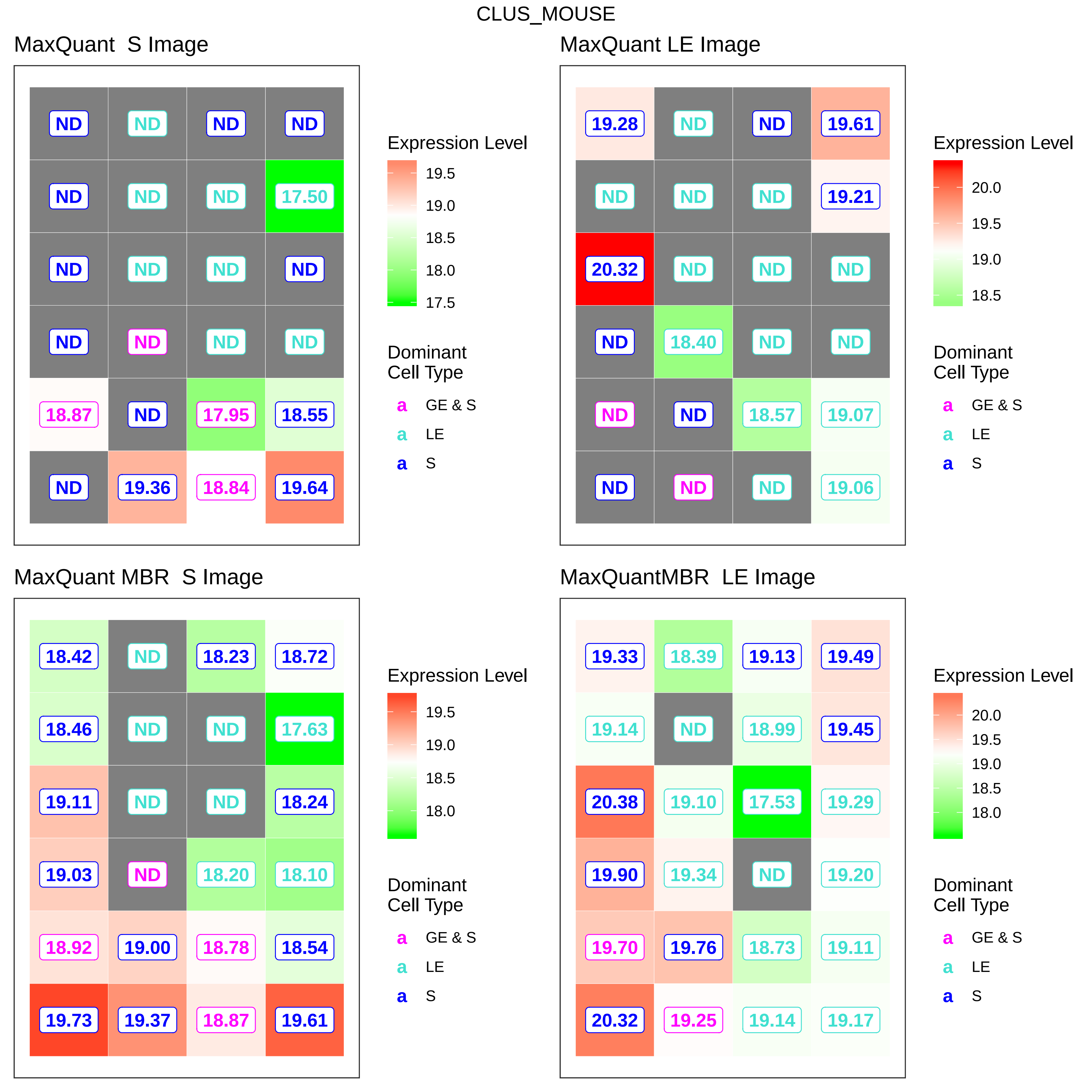
CLUS_MOUSE
MaxQuant S Image
MaxQuant LE Image
MaxQuant MBR S Image
MaxQuantMBR LE Image
Expression Level
Dominant Cell Type
GE & S
LE
S
ND
19.28
19.61
19.21
20.32
18.40
18.57
19.07
19.06
17.50
18.87
17.95
18.55
19.36
18.84
19.64
18.42
18.23
18.72
18.46
17.63
19.11
18.24
19.03
18.20
18.10
18.92
19.00
18.78
18.54
19.73
19.37
18.87
19.61
19.33
18.39
19.13
19.49
19.14
18.99
19.45
20.38
19.10
17.53
19.29
19.90
19.34
19.20
19.70
19.76
18.73
19.11
20.32
19.25
19.14
19.17

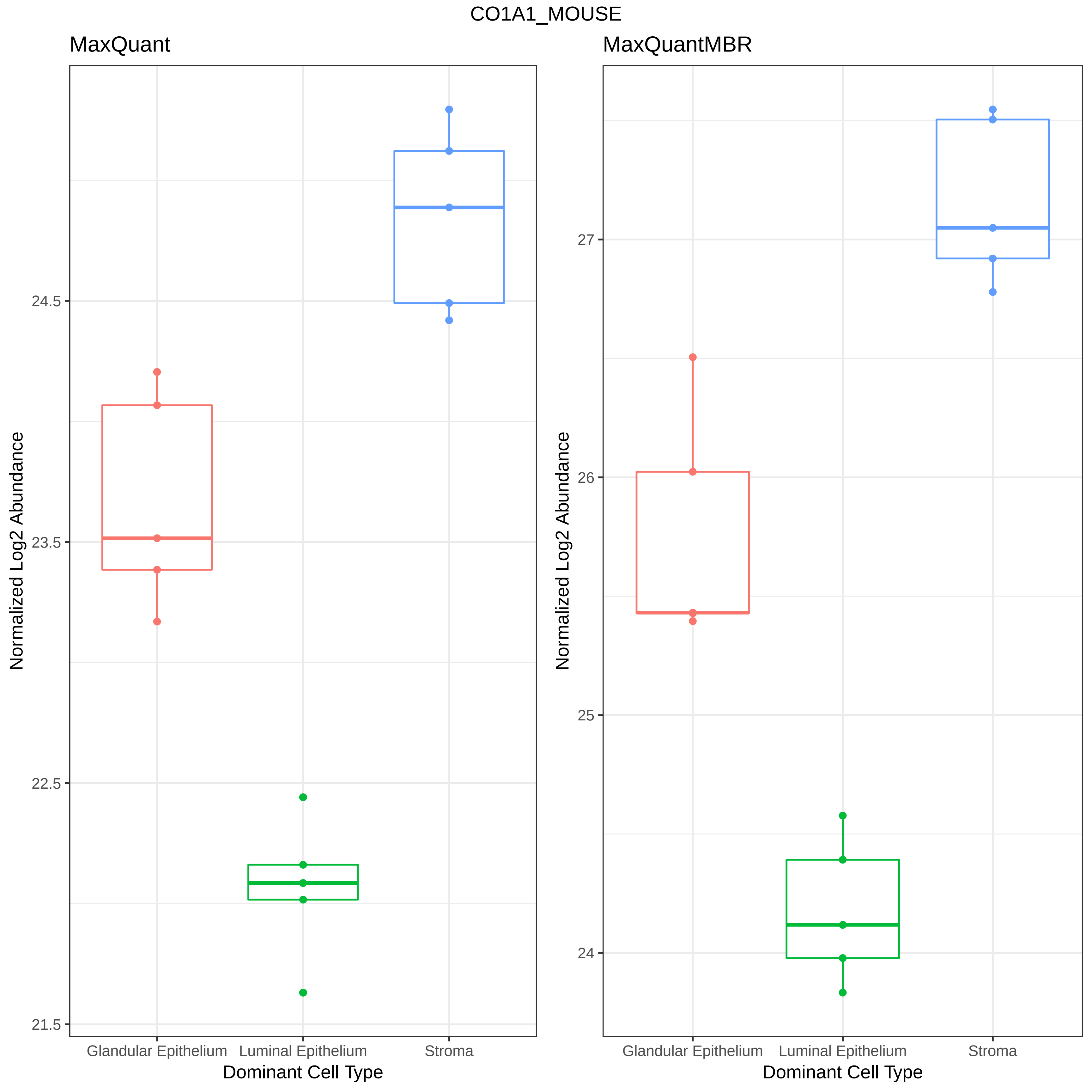

CO1A1_MOUSE
MaxQuant
MaxQuantMBR
Normalized Log2 Abundance
24.5
23.5
22.5
21.5
27
26
25
24
Glandular Epithelium
Luminal Epithelium
Stroma
Dominant Cell Type

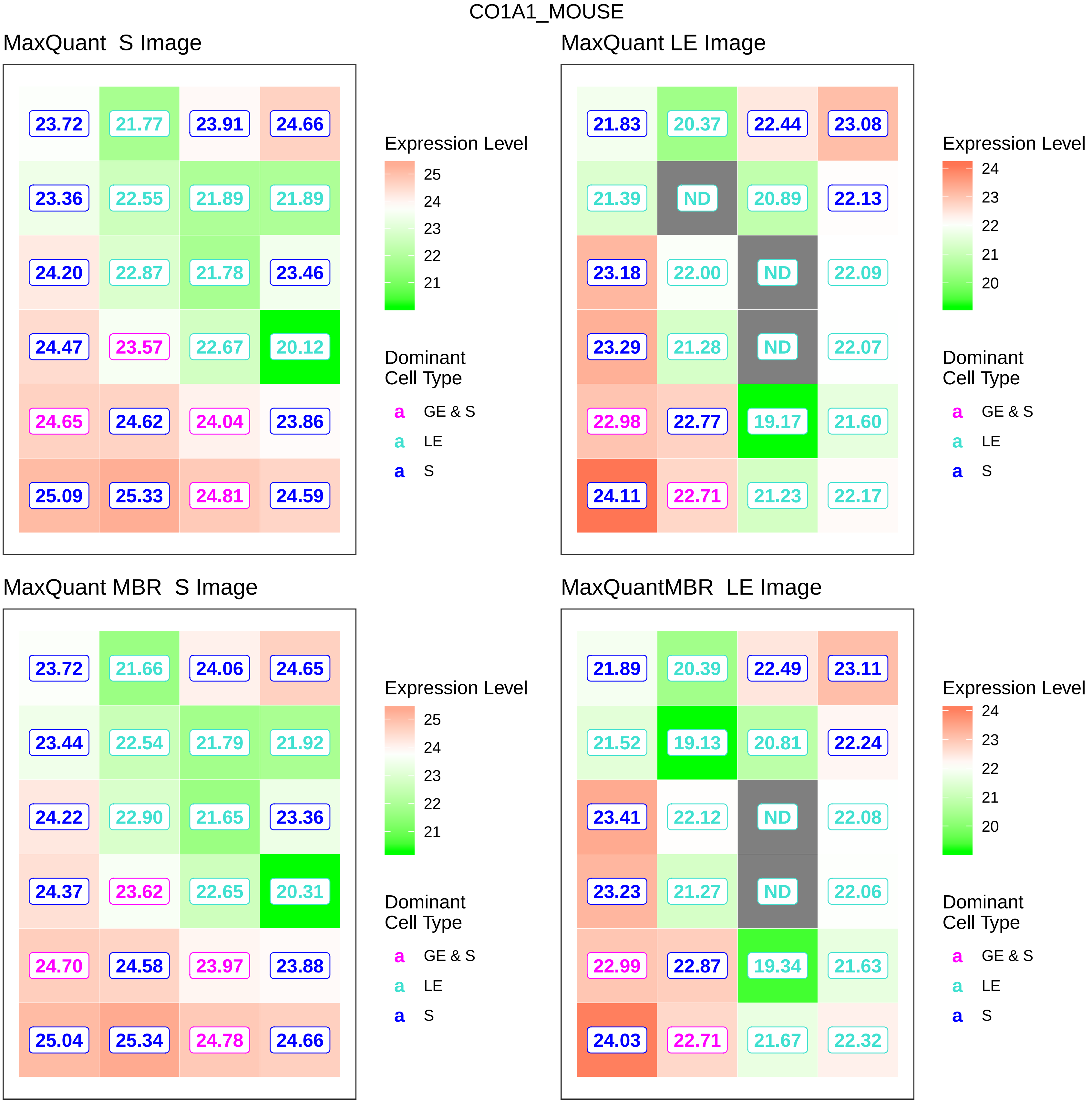
CO1A1_MOUSE
MaxQuant S Image
23.72 21.77 23.91 24.66
23.36 22.55 21.89 21.89
24.20 22.87 21.78 23.46
24.47 23.57 22.67 20.12
24.65 24.62 24.04 23.86
25.09 25.33 24.81 24.59
Expression Level
25
24
23
22
21
Dominant Cell Type
a GE & S
a LE
a S
MaxQuant LE Image
21.83 20.37 22.44 23.08
21.39 ND 20.89 22.13
23.18 22.00 ND 22.09
23.29 21.28 ND 22.07
22.98 22.77 19.17 21.60
24.11 22.71 21.23 22.17
Expression Level
24
23
22
21
20
Dominant Cell Type
a GE & S
a LE
a S
MaxQuant MBR S Image
23.72 21.66 24.06 24.65
23.44 22.54 21.79 21.92
24.22 22.90 21.65 23.36
24.37 23.62 22.65 20.31
24.70 24.58 23.97 23.88
25.04 25.34 24.78 24.66
Expression Level
25
24
23
22
21
Dominant Cell Type
a GE & S
a LE
a S
MaxQuantMBR LE Image
21.89 20.39 22.49 23.11
21.52 19.13 20.81 22.24
23.41 22.12 ND 22.08
23.23 21.27 ND 22.06
22.99 22.87 19.34 21.63
24.03 22.71 21.67 22.32
Expression Level
24
23
22
21
20
Dominant Cell Type
a GE & S
a LE
a S

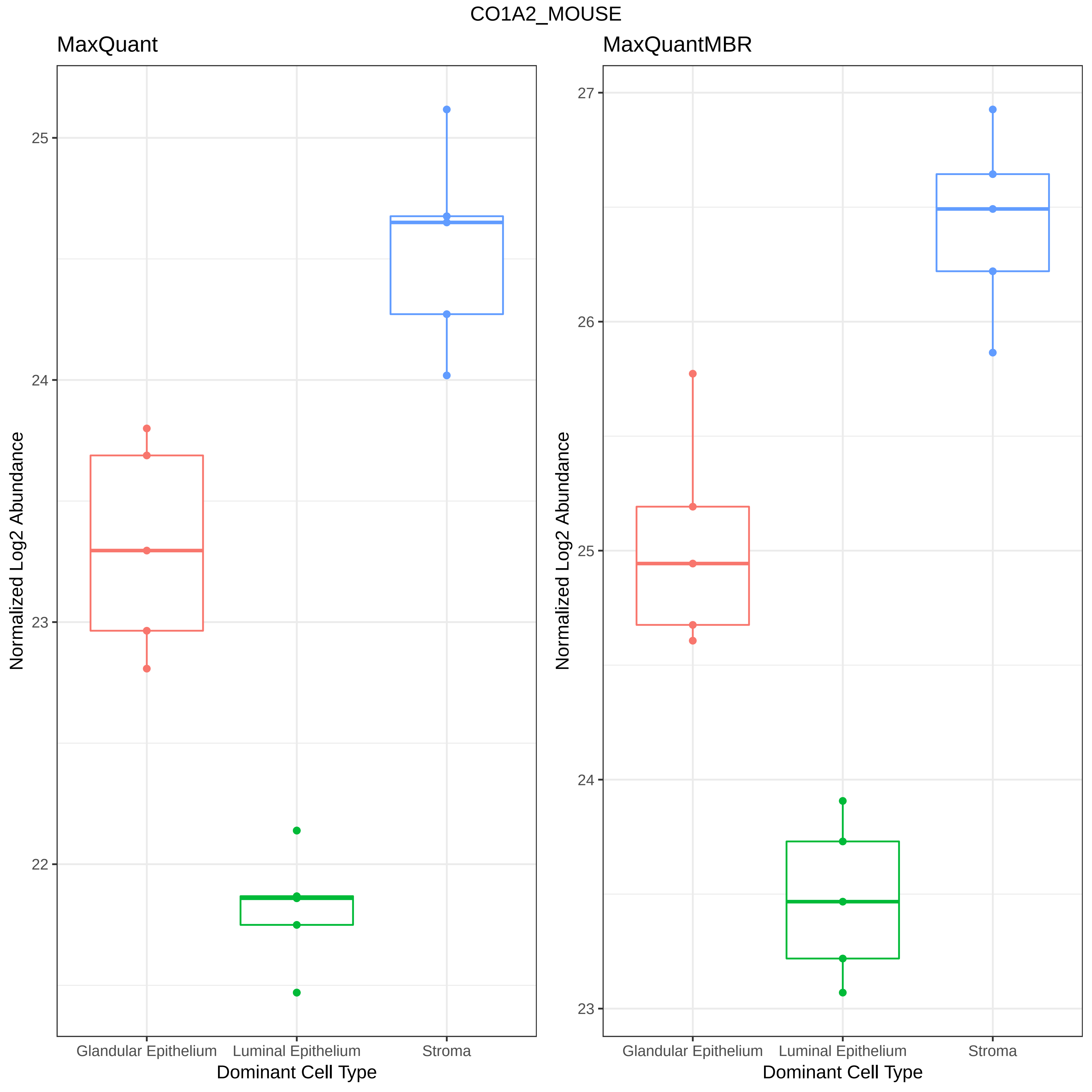

CO1A2_MOUSE
MaxQuant
MaxQuantMBR
Normalized Log2 Abundance
25
24
23
22
27
26
25
24
23
Glandular Epithelium
Luminal Epithelium
Stroma
Dominant Cell Type

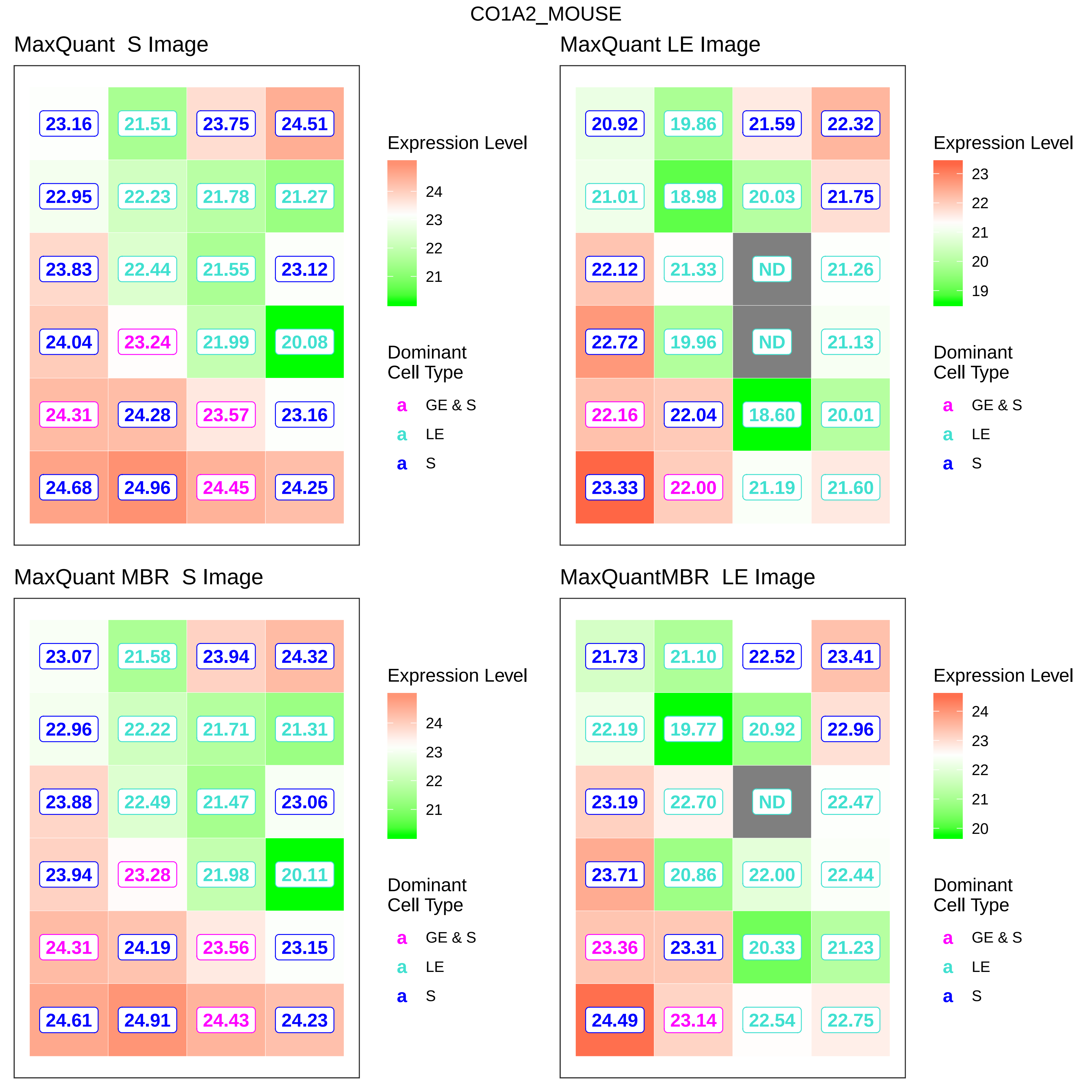
CO1A2_MOUSE
MaxQuant S Image
23.16 21.51 23.75 24.51
22.95 22.23 21.78 21.27
23.83 22.44 21.55 23.12
24.04 23.24 21.99 20.08
24.31 24.28 23.57 23.16
24.68 24.96 24.45 24.25
Expression Level
24
23
22
21
Dominant Cell Type
a GE & S
a LE
a S
MaxQuant LE Image
20.92 19.86 21.59 22.32
21.01 18.98 20.03 21.75
22.12 21.33 ND 21.26
22.72 19.96 ND 21.13
22.16 22.04 18.60 20.01
23.33 22.00 21.19 21.60
Expression Level
23
22
21
20
19
Dominant Cell Type
a GE & S
a LE
a S
MaxQuant MBR S Image
23.07 21.58 23.94 24.32
22.96 22.22 21.71 21.31
23.88 22.49 21.47 23.06
23.94 23.28 21.98 20.11
24.31 24.19 23.56 23.15
24.61 24.91 24.43 24.23
Expression Level
24
23
22
21
Dominant Cell Type
a GE & S
a LE
a S
MaxQuantMBR LE Image
21.73 21.10 22.52 23.41
22.19 19.77 20.92 22.96
23.19 22.70 ND 22.47
23.71 20.86 22.00 22.44
23.36 23.31 20.33 21.23
24.49 23.14 22.54 22.75
Expression Level
24
23
22
21
20
Dominant Cell Type
a GE & S
a LE
a S

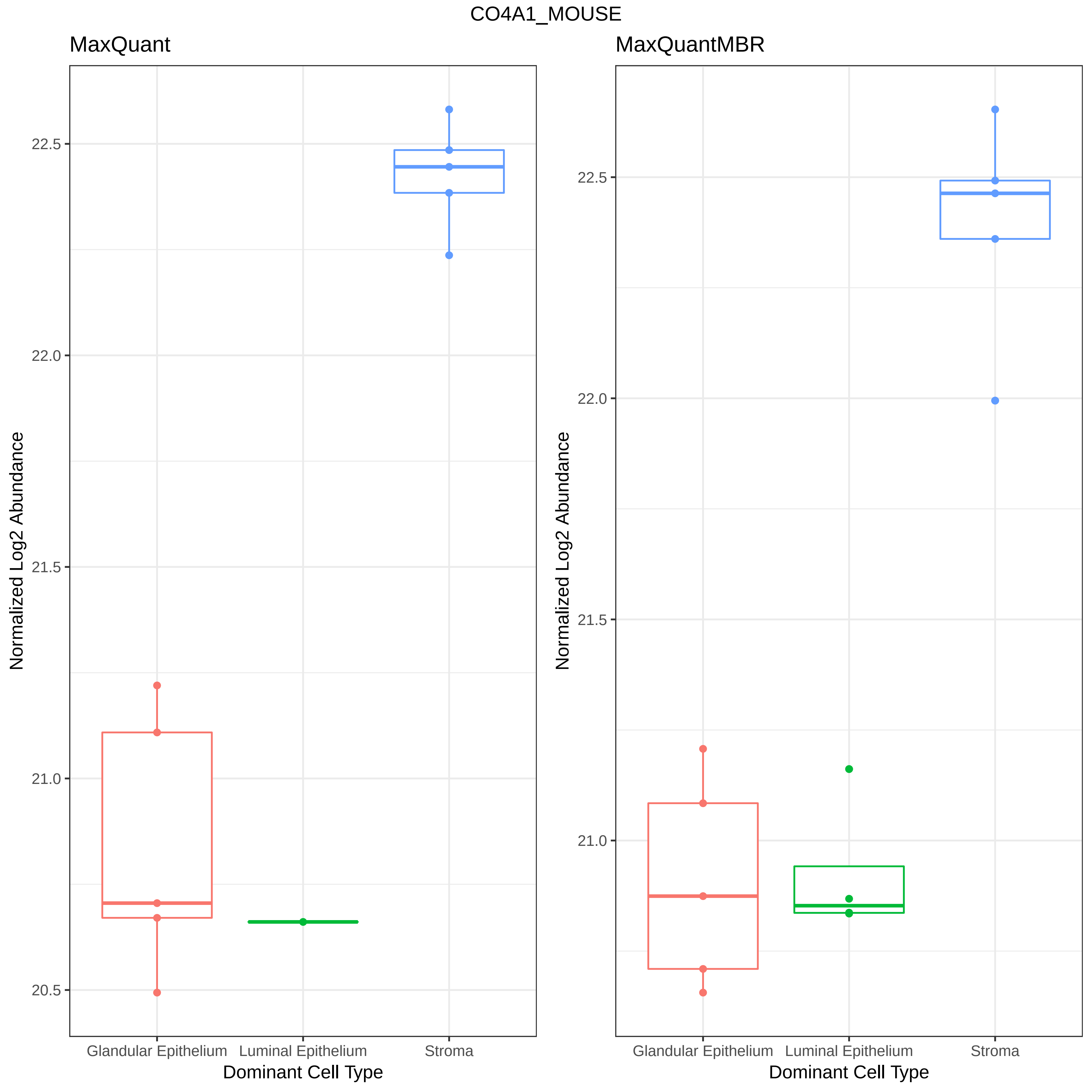

CO4A1_MOUSE
MaxQuant
MaxQuantMBR
Normalized Log2 Abundance
22.5
22.0
21.5
21.0
20.5
Glandular Epithelium
Luminal Epithelium
Stroma
Dominant Cell Type

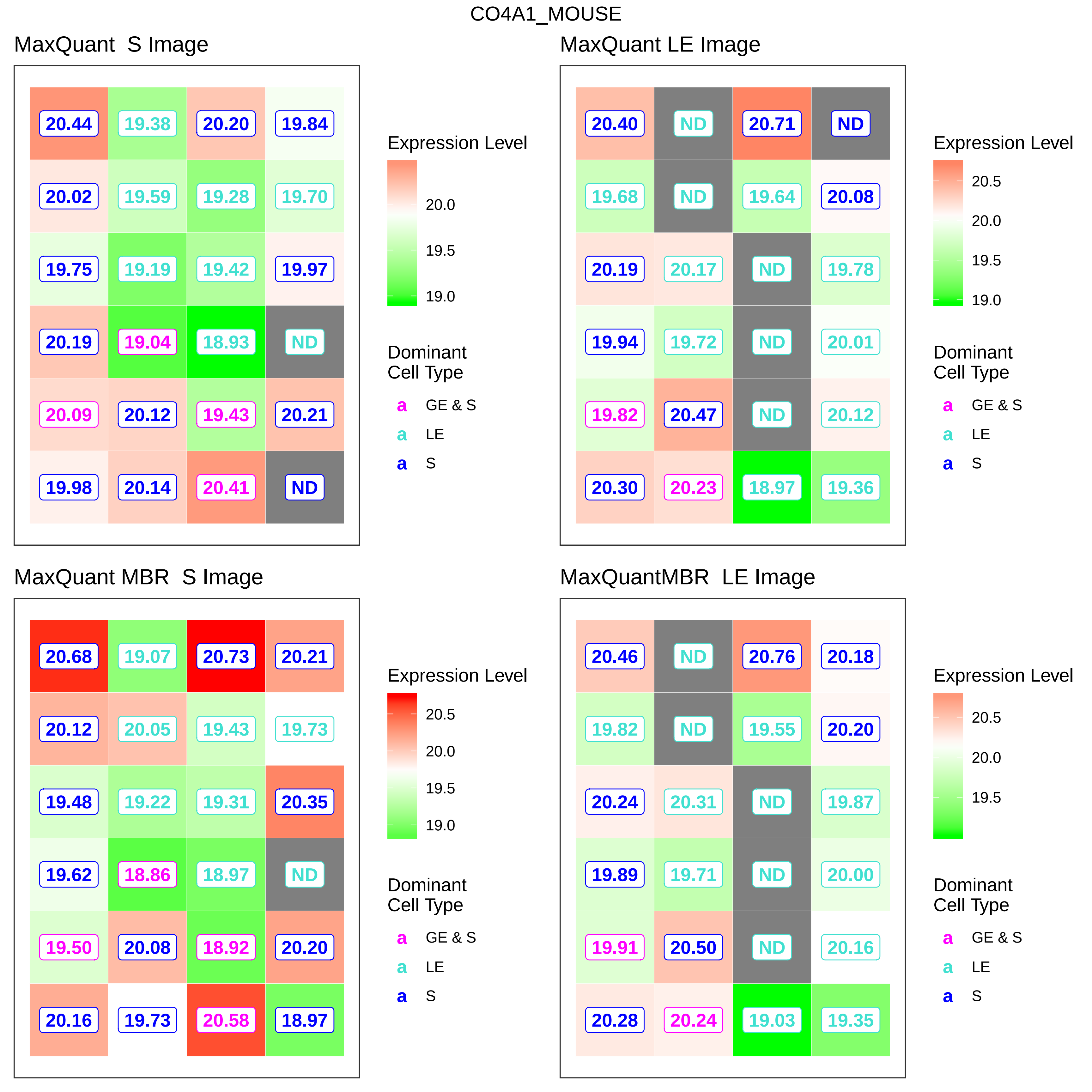

CO4A1_MOUSE
MaxQuant S Image
20.44 19.38 20.20 19.84
20.02 19.59 19.28 19.70
19.75 19.19 19.42 19.97
20.19 19.04 18.93 ND
20.09 20.12 19.43 20.21
19.98 20.14 20.41 ND
Expression Level
20.0
19.5
19.0
Dominant Cell Type
a GE & S
a LE
a S
MaxQuant LE Image
20.40 ND 20.71 ND
19.68 ND 19.64 20.08
20.19 20.17 ND 19.78
19.94 19.72 ND 20.01
19.82 20.47 ND 20.12
20.30 20.23 18.97 19.36
Expression Level
20.5
20.0
19.5
19.0
Dominant Cell Type
a GE & S
a LE
a S
MaxQuant MBR S Image
20.68 19.07 20.73 20.21
20.12 20.05 19.43 19.73
19.48 19.22 19.31 20.35
19.62 18.86 18.97 ND
19.50 20.08 18.92 20.20
20.16 19.73 20.58 18.97
Expression Level
20.5
20.0
19.5
19.0
Dominant Cell Type
a GE & S
a LE
a S
MaxQuantMBR LE Image
20.46 ND 20.76 20.18
19.82 ND 19.55 20.20
20.24 20.31 ND 19.87
19.89 19.71 ND 20.00
19.91 20.50 ND 20.16
20.28 20.24 19.03 19.35
Expression Level
20.5
20.0
19.5
Dominant Cell Type
a GE & S
a LE
a S

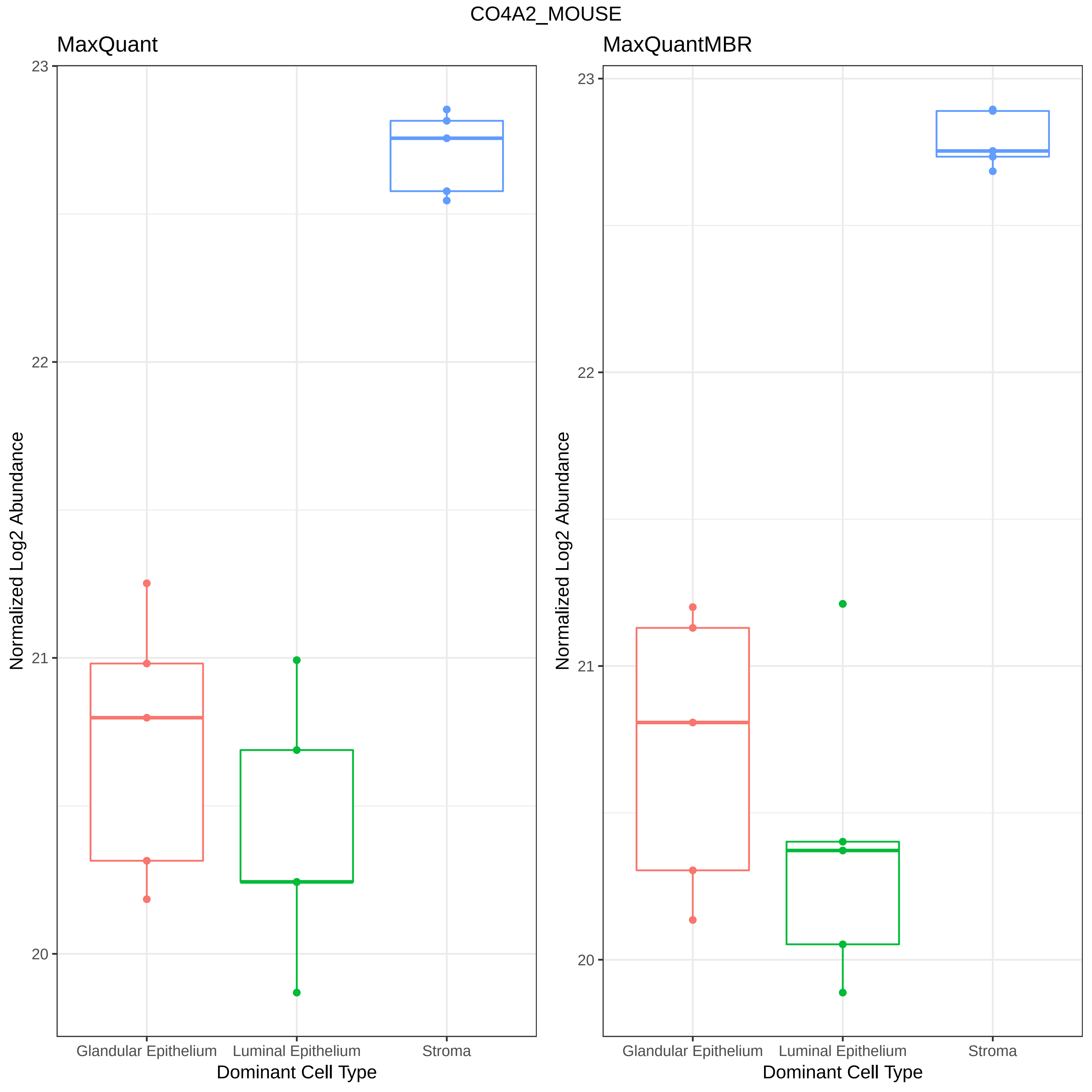
CO4A2_MOUSE
MaxQuant
MaxQuantMBR
Normalized Log2 Abundance
23
22
21
20
Glandular Epithelium
Luminal Epithelium
Stroma
Dominant Cell Type

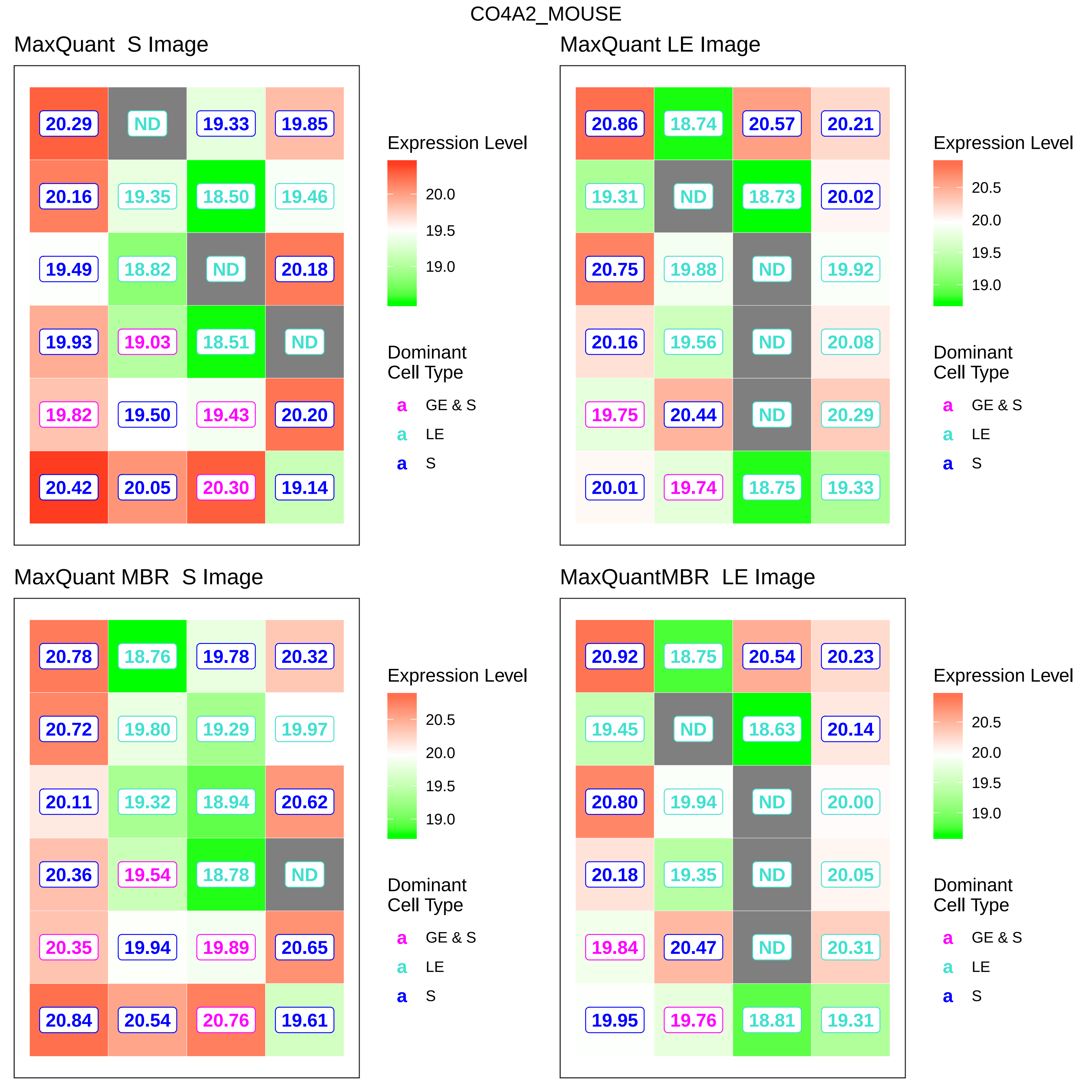
CO4A2_MOUSE
MaxQuant S Image
20.29 ND 19.33 19.85
20.16 19.35 18.50 19.46
19.49 18.82 ND 20.18
19.93 19.03 18.51 ND
19.82 19.50 19.43 20.20
20.42 20.05 20.30 19.14
Expression Level
20.0
19.5
19.0
Dominant Cell Type
a GE & S
a LE
a S
MaxQuant LE Image
20.86 18.74 20.57 20.21
19.31 ND 18.73 20.02
20.75 19.88 ND 19.92
20.16 19.56 ND 20.08
19.75 20.44 ND 20.29
20.01 19.74 18.75 19.33
Expression Level
20.5
20.0
19.5
19.0
Dominant Cell Type
a GE & S
a LE
a S
MaxQuant MBR S Image
20.78 18.76 19.78 20.32
20.72 19.80 19.29 19.97
20.11 19.32 18.94 20.62
20.36 19.54 18.78 ND
20.35 19.94 19.89 20.65
20.84 20.54 20.76 19.61
Expression Level
20.5
20.0
19.5
19.0
Dominant Cell Type
a GE & S
a LE
a S
MaxQuantMBR LE Image
20.92 18.75 20.54 20.23
19.45 ND 18.63 20.14
20.80 19.94 ND 20.00
20.18 19.35 ND 20.05
19.84 20.47 ND 20.31
19.95 19.76 18.81 19.31
Expression Level
20.5
20.0
19.5
19.0
Dominant Cell Type
a GE & S
a LE
a S

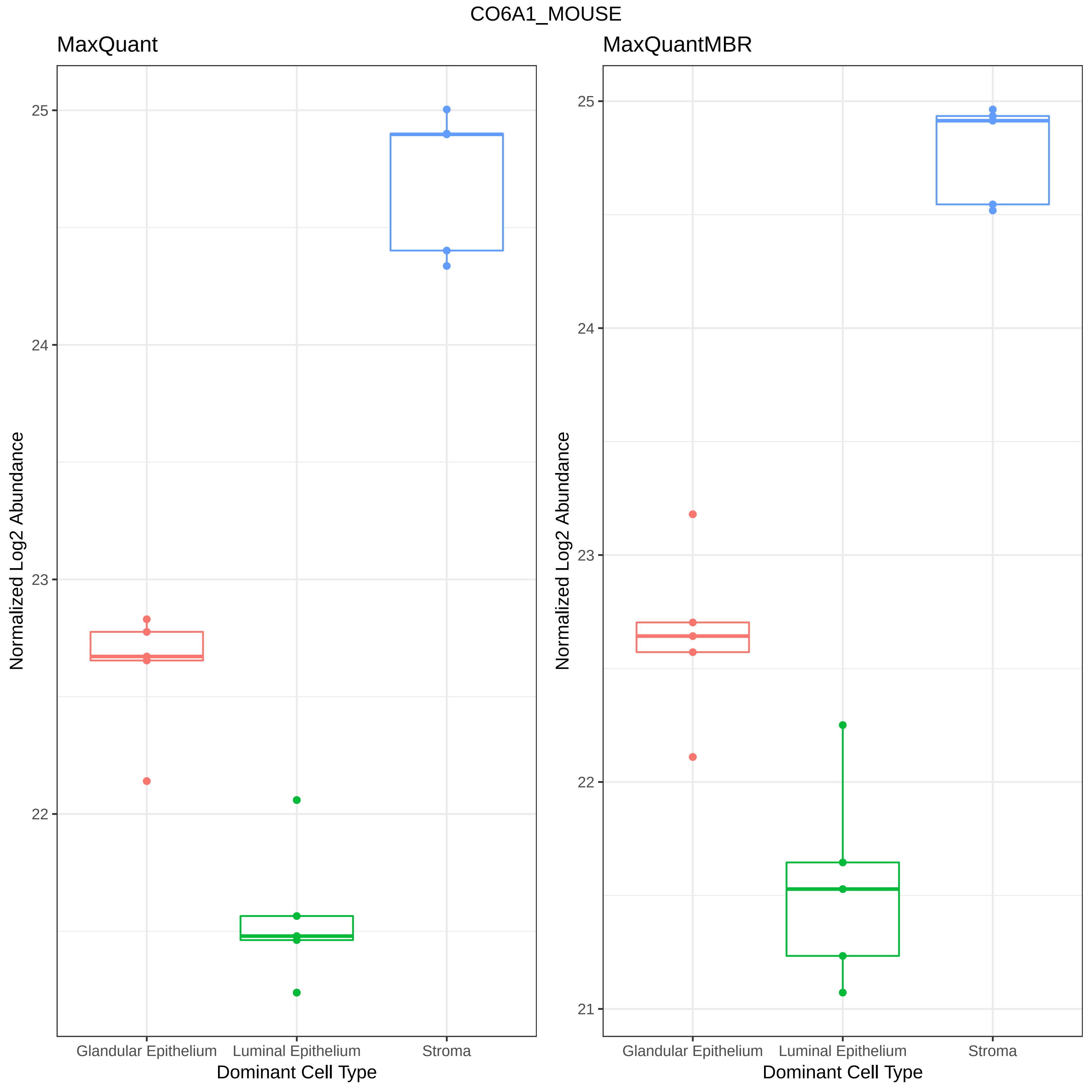

CO6A1_MOUSE
MaxQuant
MaxQuantMBR
Normalized Log2 Abundance
25
24
23
22
21
Glandular Epithelium
Luminal Epithelium
Stroma
Dominant Cell Type

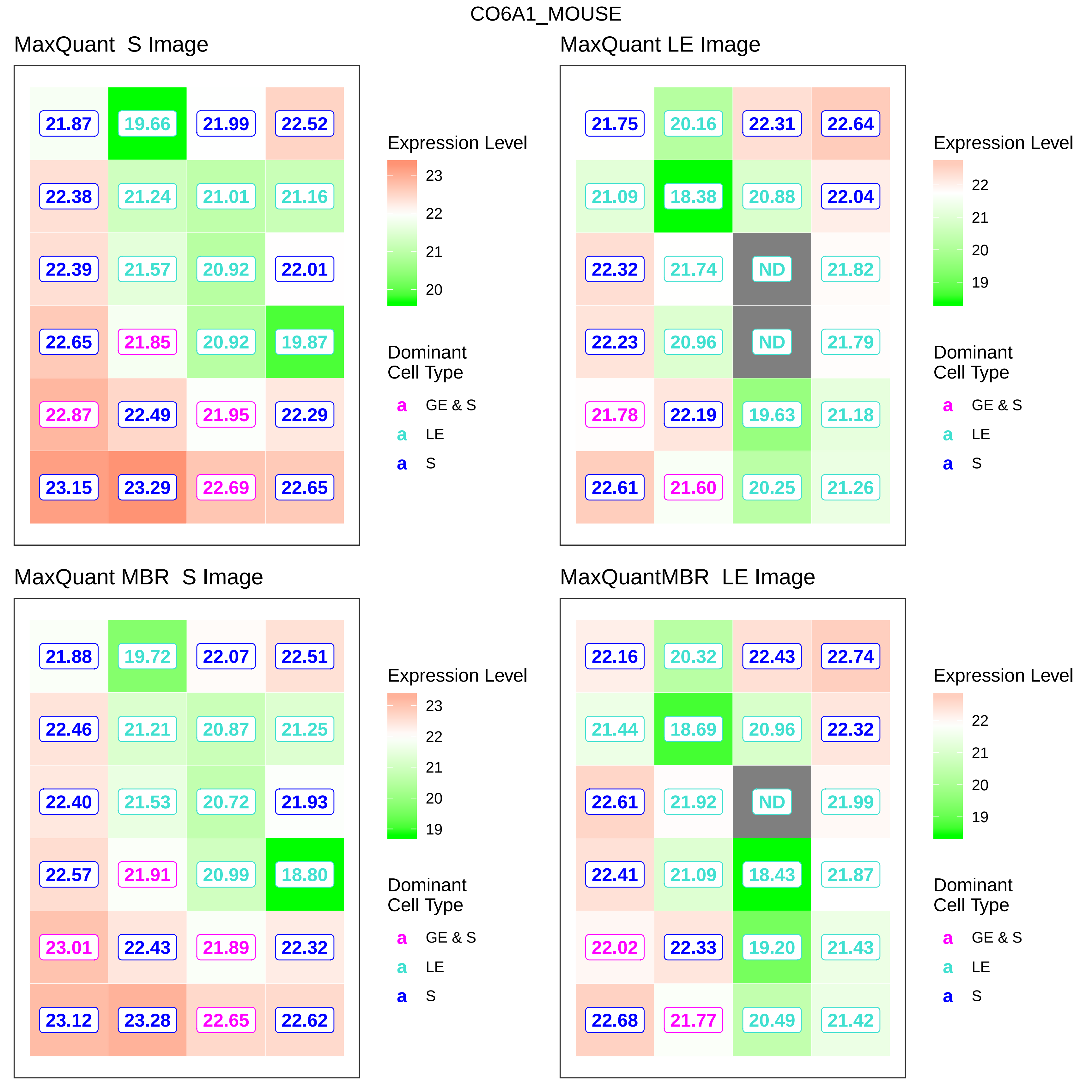

CO6A1_MOUSE
MaxQuant S Image
21.87 19.66 21.99 22.52
22.38 21.24 21.01 21.16
22.39 21.57 20.92 22.01
22.65 21.85 20.92 19.87
22.87 22.49 21.95 22.29
23.15 23.29 22.69 22.65
Expression Level
23
22
21
20
Dominant Cell Type
a GE & S
a LE
a S
MaxQuant LE Image
21.75 20.16 22.31 22.64
21.09 18.38 20.88 22.04
22.32 21.74 ND 21.82
22.23 20.96 ND 21.79
21.78 22.19 19.63 21.18
22.61 21.60 20.25 21.26
Expression Level
22
21
20
19
Dominant Cell Type
a GE & S
a LE
a S
MaxQuant MBR S Image
21.88 19.72 22.07 22.51
22.46 21.21 20.87 21.25
22.40 21.53 20.72 21.93
22.57 21.91 20.99 18.80
23.01 22.43 21.89 22.32
23.12 23.28 22.65 22.62
Expression Level
23
22
21
20
19
Dominant Cell Type
a GE & S
a LE
a S
MaxQuantMBR LE Image
22.16 20.32 22.43 22.74
21.44 18.69 20.96 22.32
22.61 21.92 ND 21.99
22.41 21.09 18.43 21.87
22.02 22.33 19.20 21.43
22.68 21.77 20.49 21.42
Expression Level
22
21
20
19
Dominant Cell Type
a GE & S
a LE
a S

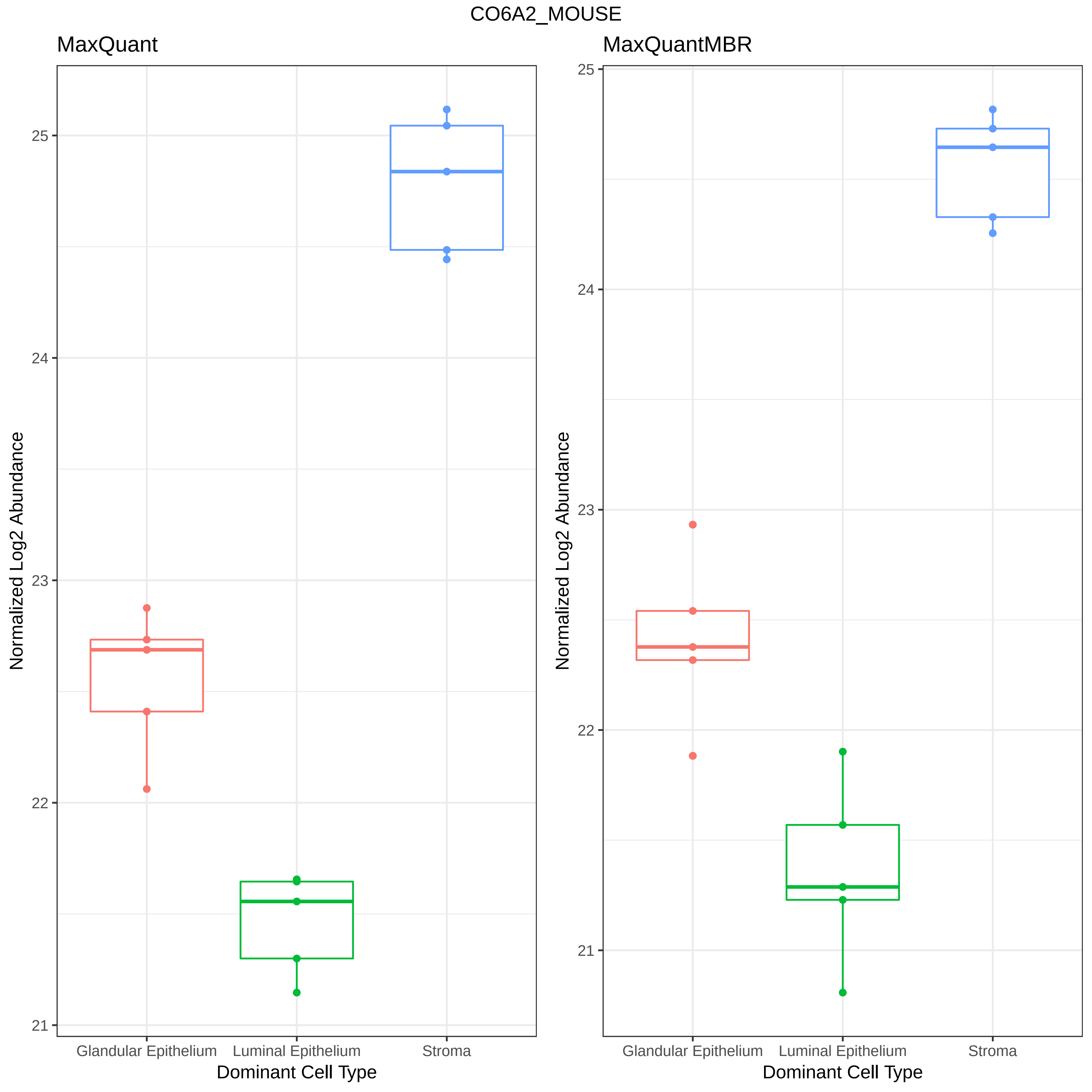

CO6A2_MOUSE
MaxQuant
MaxQuantMBR
Normalized Log2 Abundance
25
24
23
22
21
Glandular Epithelium
Luminal Epithelium
Stroma
Dominant Cell Type

CO6A2_MOUSE

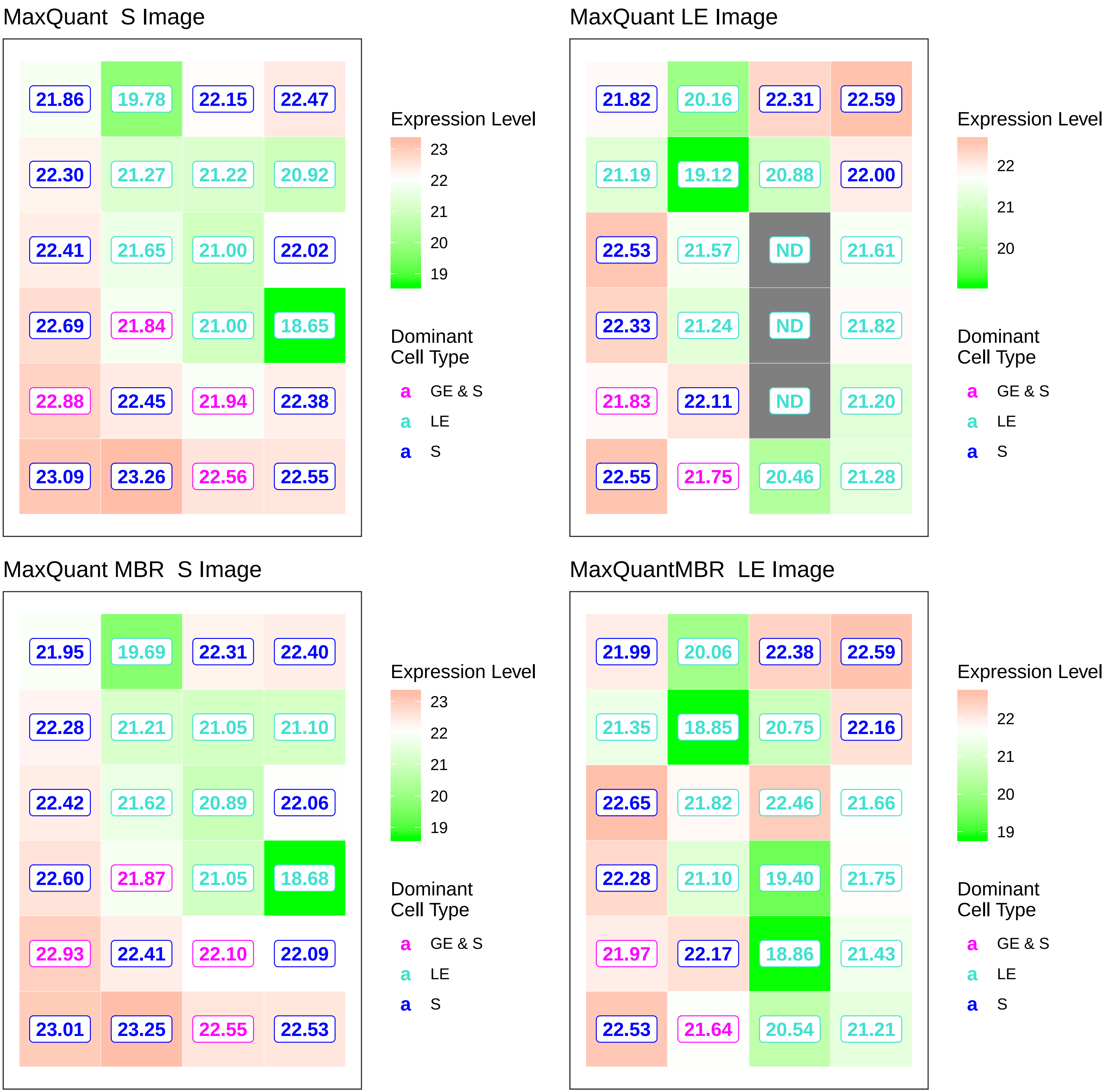
MaxQuant S Image
21.86 19.78 22.15 22.47
22.30 21.27 21.22 20.92
22.41 21.65 21.00 22.02
22.69 21.84 21.00 18.65
22.88 22.45 21.94 22.38
23.09 23.26 22.56 22.55
Expression Level
23
22
21
20
19
Dominant Cell Type
a GE & S
a LE
a S
MaxQuant LE Image
21.82 20.16 22.31 22.59
21.19 19.12 20.88 22.00
22.53 21.57 ND 21.61
22.33 21.24 ND 21.82
21.83 22.11 ND 21.20
22.55 21.75 20.46 21.28
Expression Level
22
21
20
Dominant Cell Type
a GE & S
a LE
a S
MaxQuant MBR S Image
21.95 19.69 22.31 22.40
22.28 21.21 21.05 21.10
22.42 21.62 20.89 22.06
22.60 21.87 21.05 18.68
22.93 22.41 22.10 22.09
23.01 23.25 22.55 22.53
Expression Level
23
22
21
20
19
Dominant Cell Type
a GE & S
a LE
a S
MaxQuantMBR LE Image
21.99 20.06 22.38 22.59
21.35 18.85 20.75 22.16
22.65 21.82 22.46 21.66
22.28 21.10 19.40 21.75
21.97 22.17 18.86 21.43
22.53 21.64 20.54 21.21
Expression Level
22
21
20
19
Dominant Cell Type
a GE & S
a LE
a S

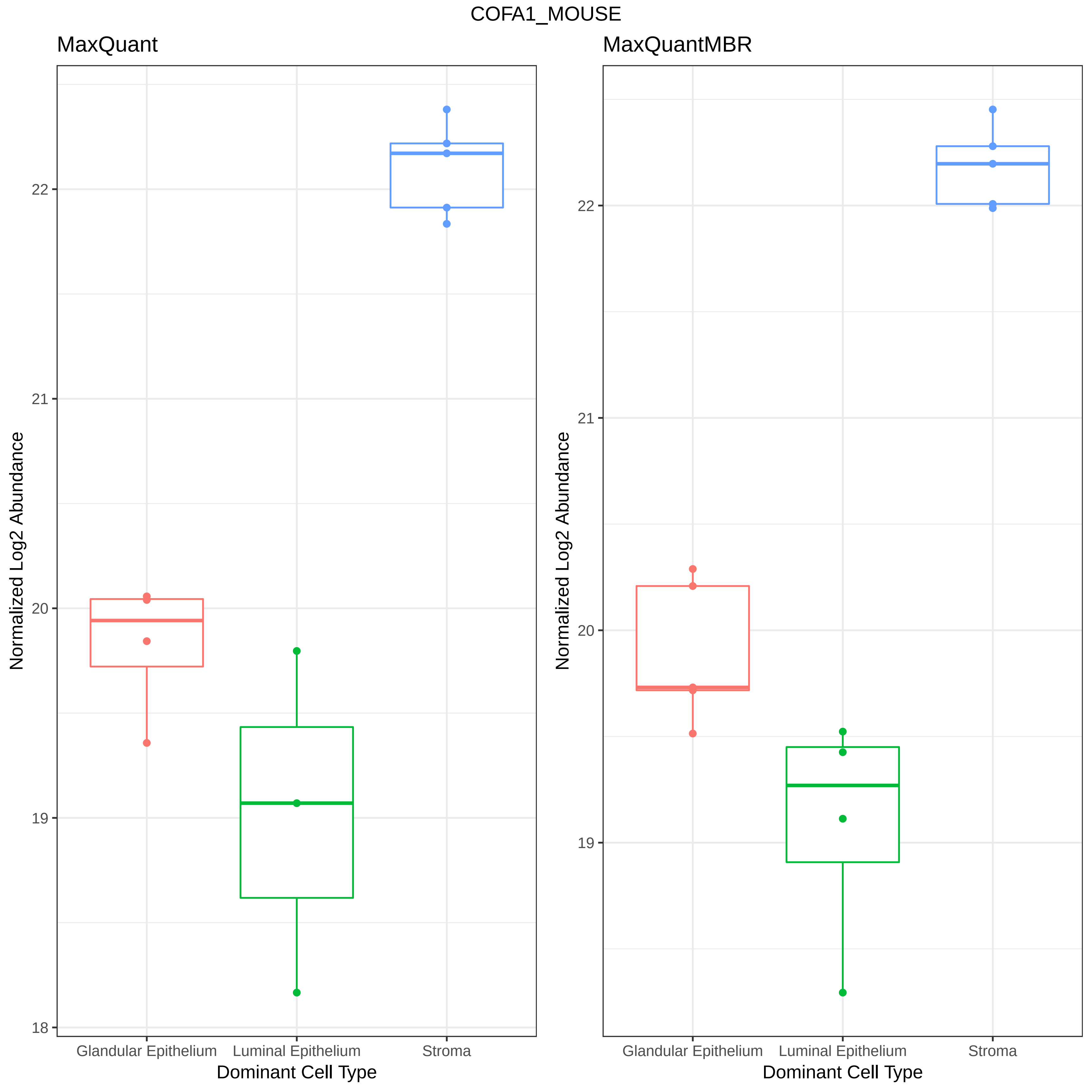
COFA1_MOUSE
MaxQuant
MaxQuantMBR
Normalized Log2 Abundance
22
21
20
19
18
Glandular Epithelium
Luminal Epithelium
Stroma
Dominant Cell Type

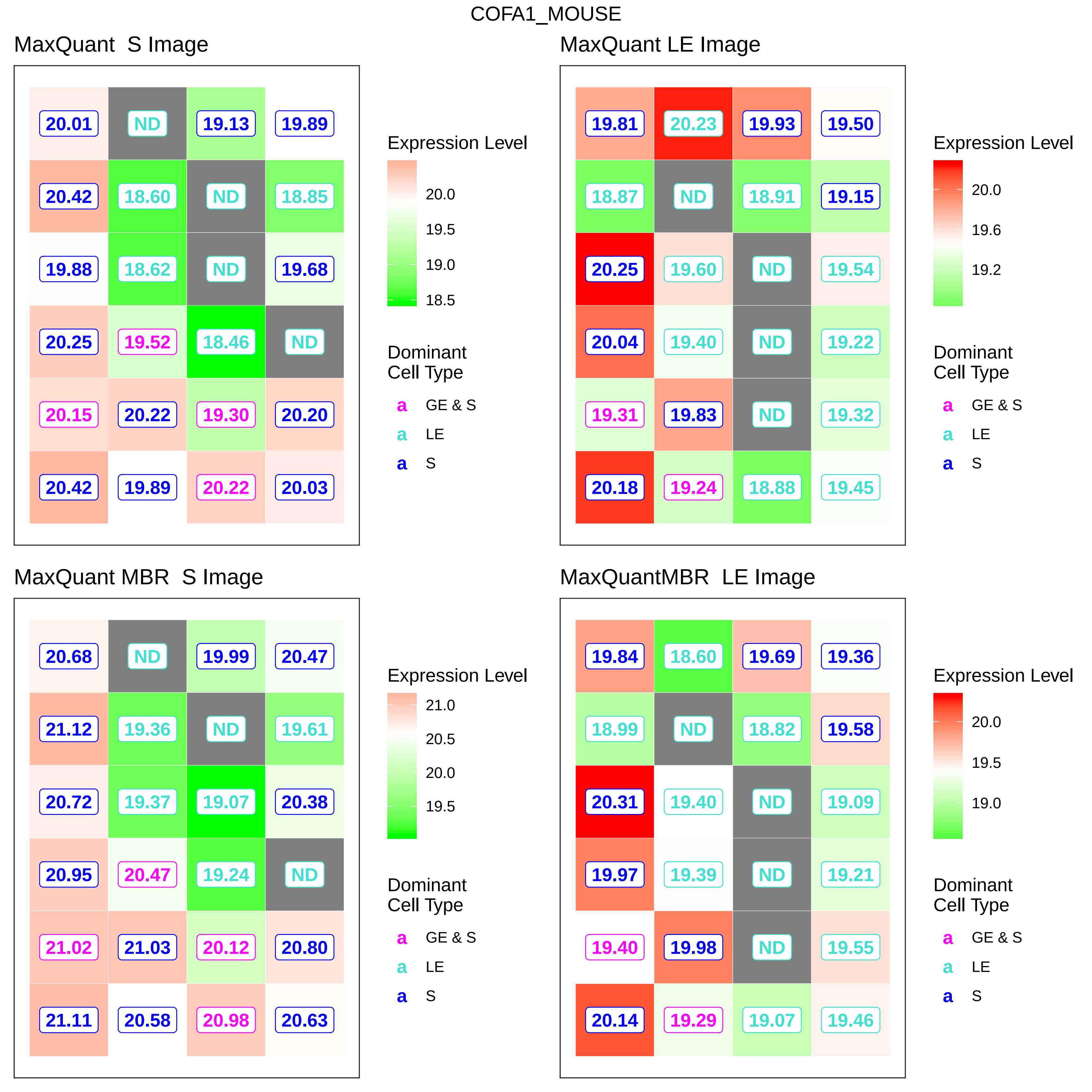
COFA1_MOUSE
MaxQuant S Image
20.01 ND 19.13 19.89
20.42 18.60 ND 18.85
19.88 18.62 ND 19.68
20.25 19.52 18.46 ND
20.15 20.22 19.30 20.20
20.42 19.89 20.22 20.03
Expression Level
20.0
19.5
19.0
18.5
Dominant Cell Type
a GE & S
a LE
a S
MaxQuant LE Image
19.81 20.23 19.93 19.50
18.87 ND 18.91 19.15
20.25 19.60 ND 19.54
20.04 19.40 ND 19.22
19.31 19.83 ND 19.32
20.18 19.24 18.88 19.45
Expression Level
20.0
19.6
19.2
Dominant Cell Type
a GE & S
a LE
a S
MaxQuant MBR S Image
20.68 ND 19.99 20.47
21.12 19.36 ND 19.61
20.72 19.37 19.07 20.38
20.95 20.47 19.24 ND
21.02 21.03 20.12 20.80
21.11 20.58 20.98 20.63
Expression Level
21.0
20.5
20.0
19.5
Dominant Cell Type
a GE & S
a LE
a S
MaxQuantMBR LE Image
19.84 18.60 19.69 19.36
18.99 ND 18.82 19.58
20.31 19.40 ND 19.09
19.97 19.39 ND 19.21
19.40 19.98 ND 19.55
20.14 19.29 19.07 19.46
Expression Level
20.0
19.5
19.0
Dominant Cell Type
a GE & S
a LE
a S

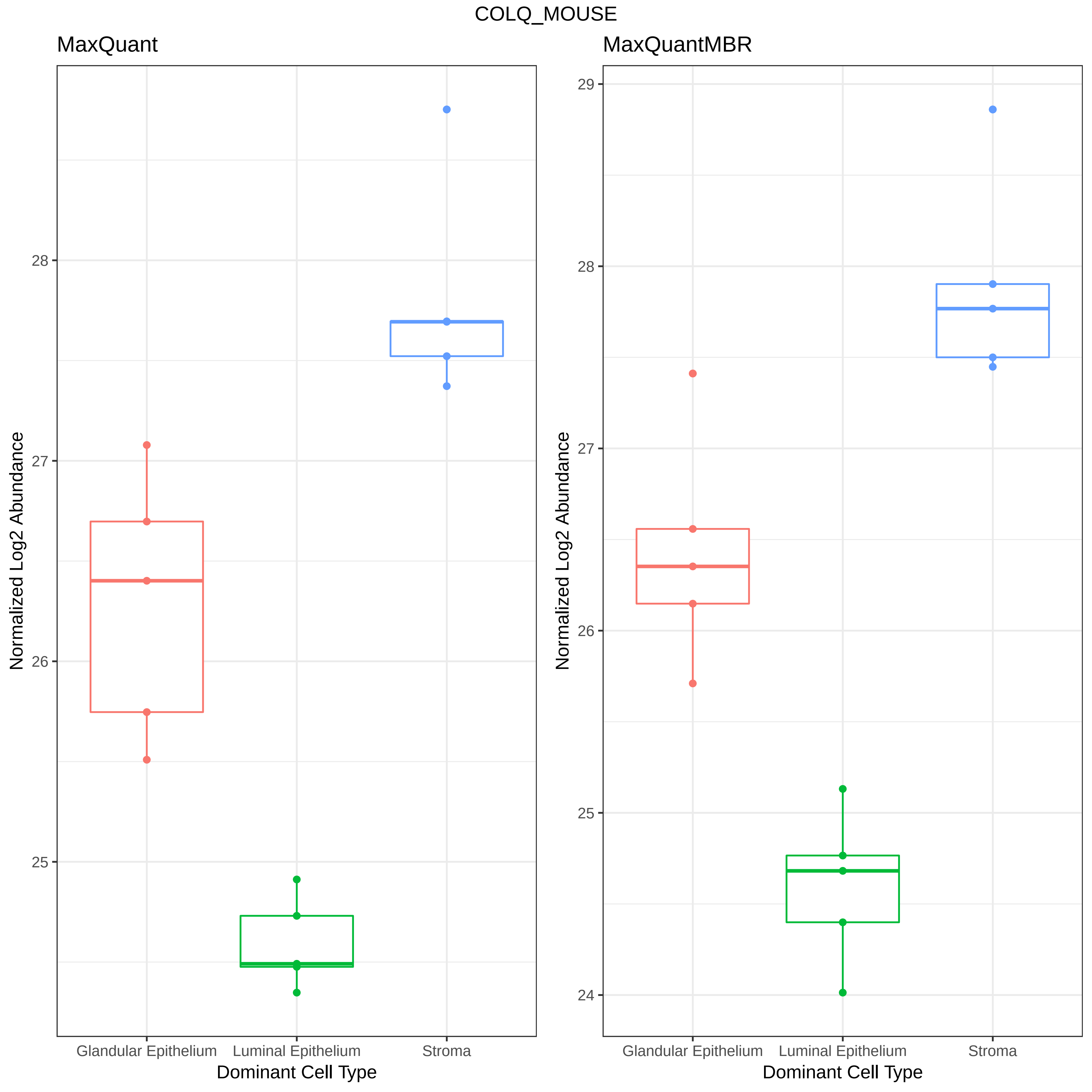
COLQ_MOUSE
MaxQuant
MaxQuantMBR
Normalized Log2 Abundance
28
27
26
25
29
24
Glandular Epithelium
Luminal Epithelium
Stroma
Dominant Cell Type

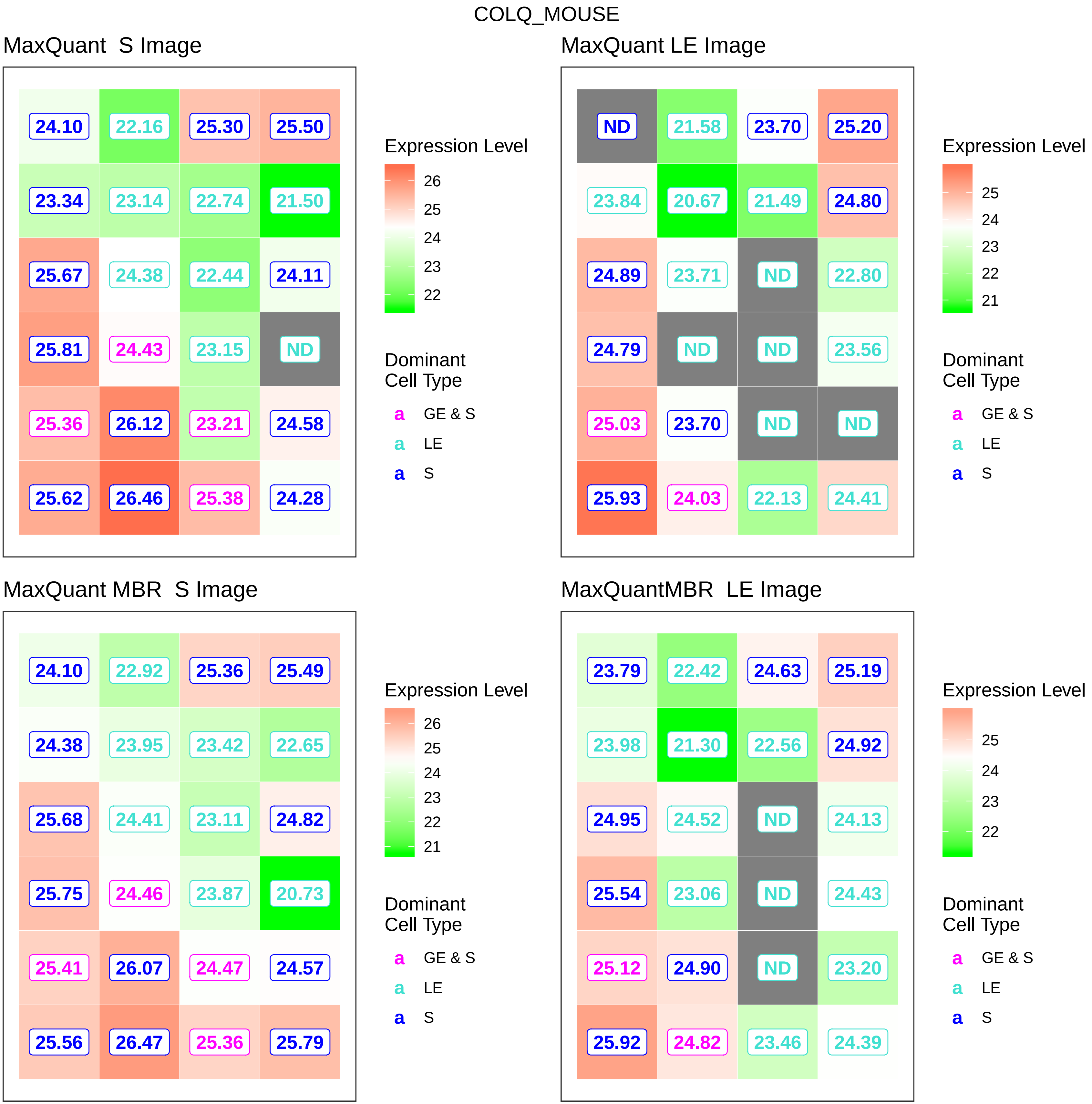
COLQ_MOUSE
MaxQuant S Image
24.10 22.16 25.30 25.50
23.34 23.14 22.74 21.50
25.67 24.38 22.44 24.11
25.81 24.43 23.15 ND
25.36 26.12 23.21 24.58
25.62 26.46 25.38 24.28
Expression Level
26
25
24
23
22
Dominant Cell Type
a GE & S
a LE
a S
MaxQuant LE Image
ND 21.58 23.70 25.20
23.84 20.67 21.49 24.80
24.89 23.71 ND 22.80
24.79 ND ND 23.56
25.03 23.70 ND ND
25.93 24.03 22.13 24.41
Expression Level
25
24
23
22
21
Dominant Cell Type
a GE & S
a LE
a S
MaxQuant MBR S Image
24.10 22.92 25.36 25.49
24.38 23.95 23.42 22.65
25.68 24.41 23.11 24.82
25.75 24.46 23.87 20.73
25.41 26.07 24.47 24.57
25.56 26.47 25.36 25.79
Expression Level
26
25
24
23
22
21
Dominant Cell Type
a GE & S
a LE
a S
MaxQuantMBR LE Image
23.79 22.42 24.63 25.19
23.98 21.30 22.56 24.92
24.95 24.52 ND 24.13
25.54 23.06 ND 24.43
25.12 24.90 ND 23.20
25.92 24.82 23.46 24.39
Expression Level
25
24
23
22
Dominant Cell Type
a GE & S
a LE
a S

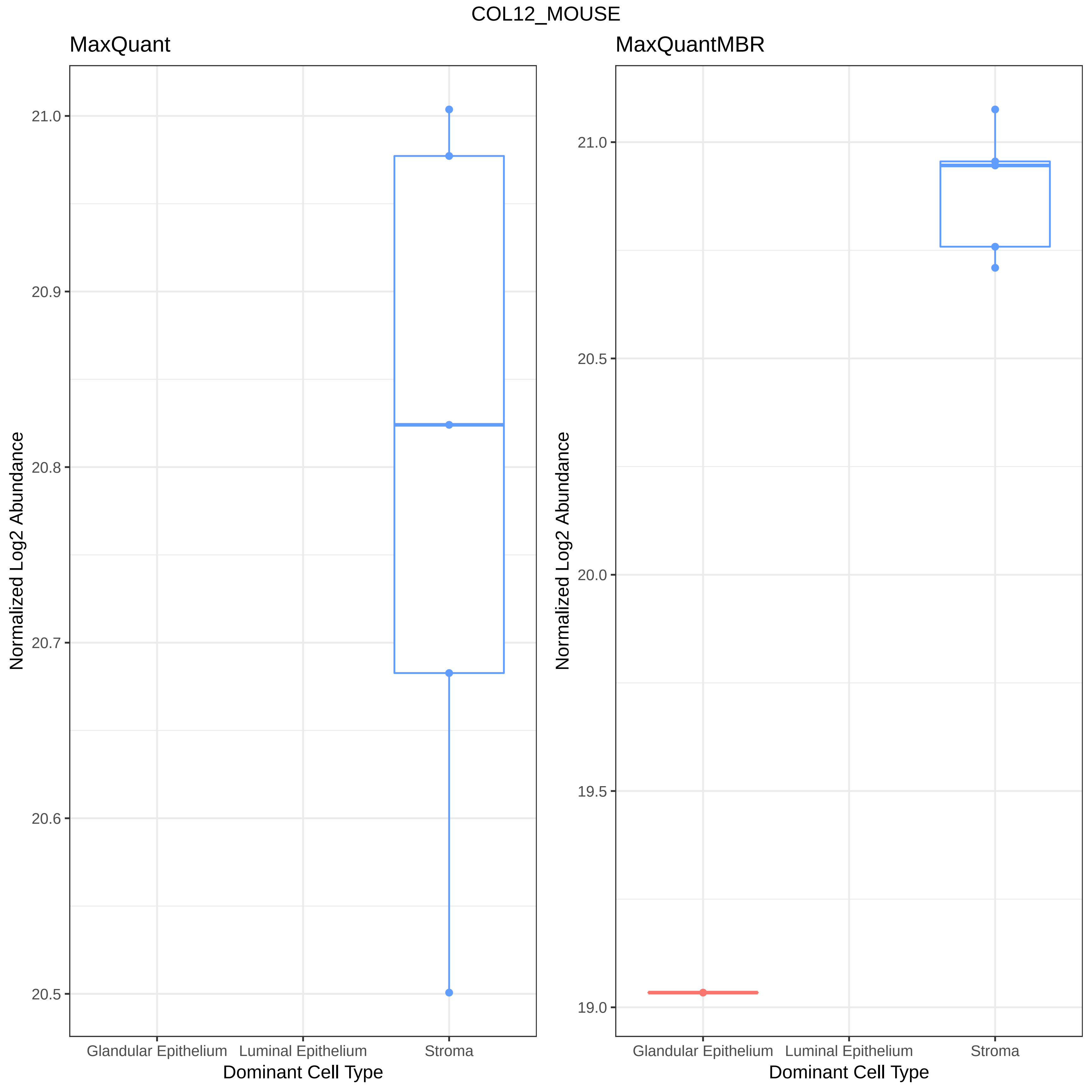
COL12_MOUSE
MaxQuant
MaxQuantMBR
Normalized Log2 Abundance
21.0
20.9
20.8
20.7
20.6
20.5
21.0
20.5
20.0
19.5
19.0
Glandular Epithelium
Luminal Epithelium
Stroma
Dominant Cell Type

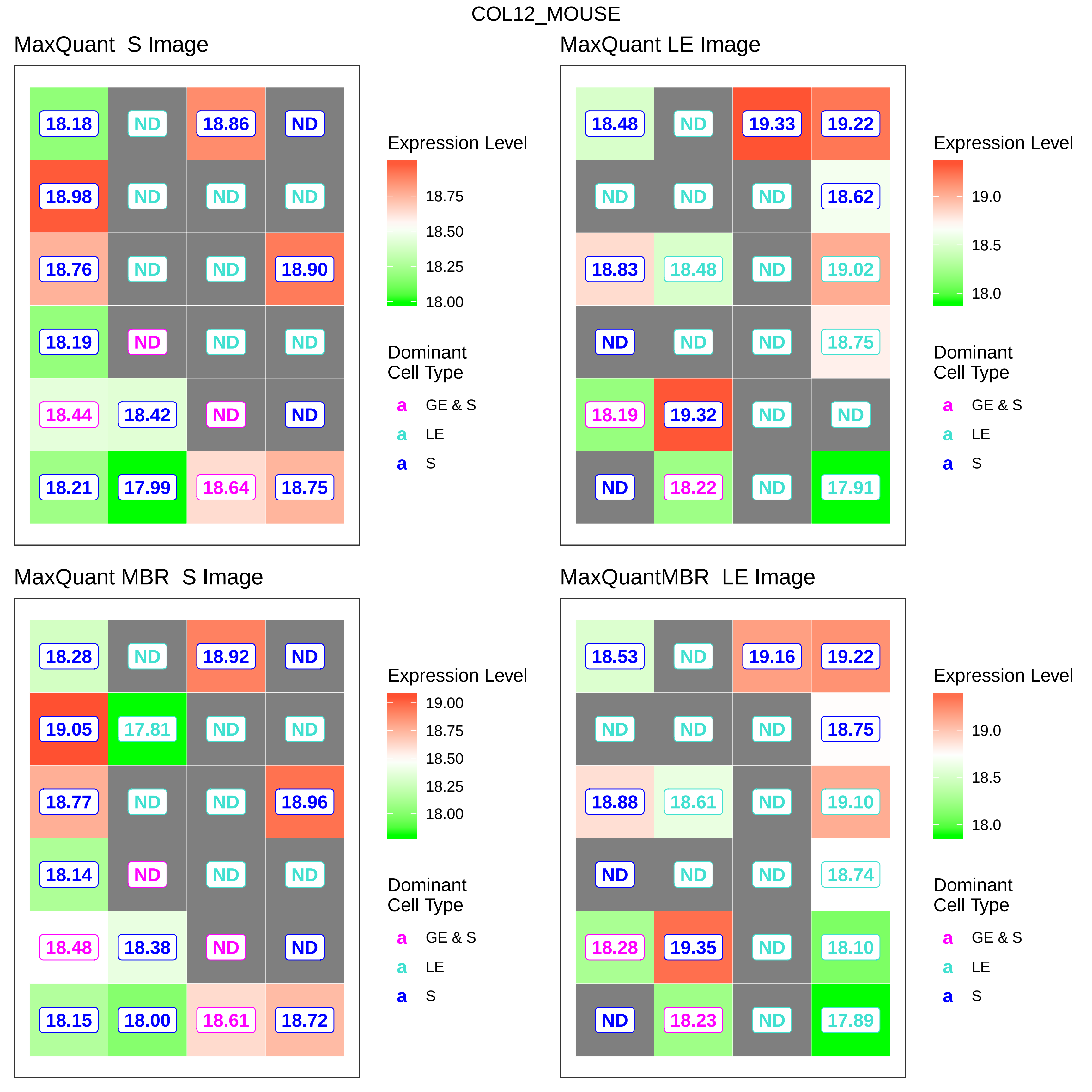
COL12_MOUSE
MaxQuant S Image
18.18
ND
18.86
ND
18.98
ND
ND
ND
18.76
ND
ND
18.90
18.19
ND
ND
ND
18.44
18.42
ND
ND
18.21
17.99
18.64
18.75
Expression Level
18.75
18.50
18.25
18.00
Dominant Cell Type
a GE & S
a LE
a S
MaxQuant LE Image
18.48
ND
19.33
19.22
ND
ND
ND
18.62
18.83
18.48
ND
19.02
ND
ND
ND
18.75
18.19
19.32
ND
ND
ND
18.22
ND
17.91
Expression Level
19.0
18.5
18.0
Dominant Cell Type
a GE & S
a LE
a S
MaxQuant MBR S Image
18.28
ND
18.92
ND
19.05
17.81
ND
ND
18.77
ND
ND
18.96
18.14
ND
ND
ND
18.48
18.38
ND
ND
18.15
18.00
18.61
18.72
Expression Level
19.00
18.75
18.50
18.25
18.00
Dominant Cell Type
a GE & S
a LE
a S
MaxQuantMBR LE Image
18.53
ND
19.16
19.22
ND
ND
ND
18.75
18.88
18.61
ND
19.10
ND
ND
ND
18.74
18.28
19.35
ND
18.10
ND
18.23
ND
17.89
Expression Level
19.0
18.5
18.0
Dominant Cell Type
a GE & S
a LE
a S

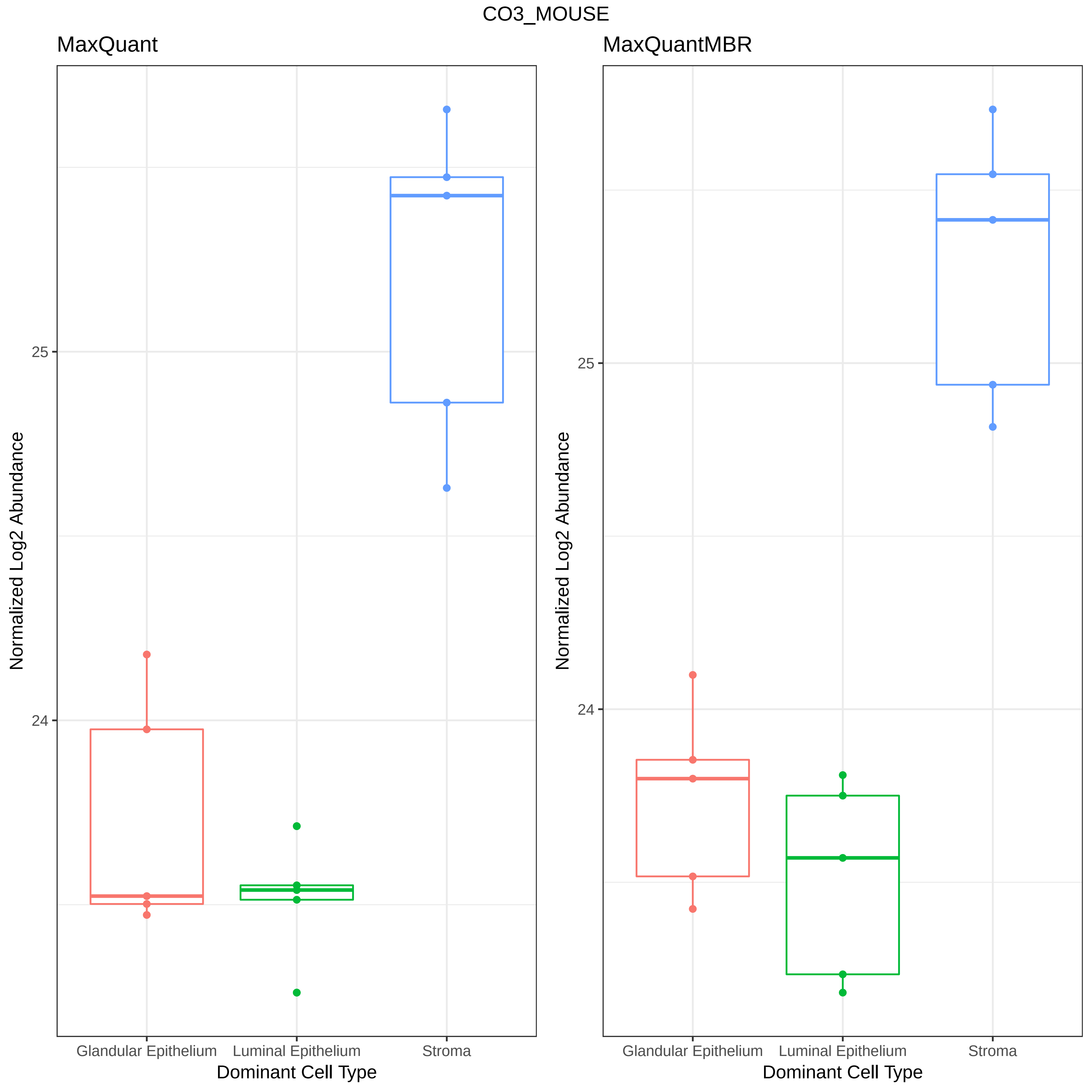

CO3_MOUSE
MaxQuant
MaxQuantMBR
Normalized Log2 Abundance
25
24
Glandular Epithelium
Luminal Epithelium
Stroma
Dominant Cell Type

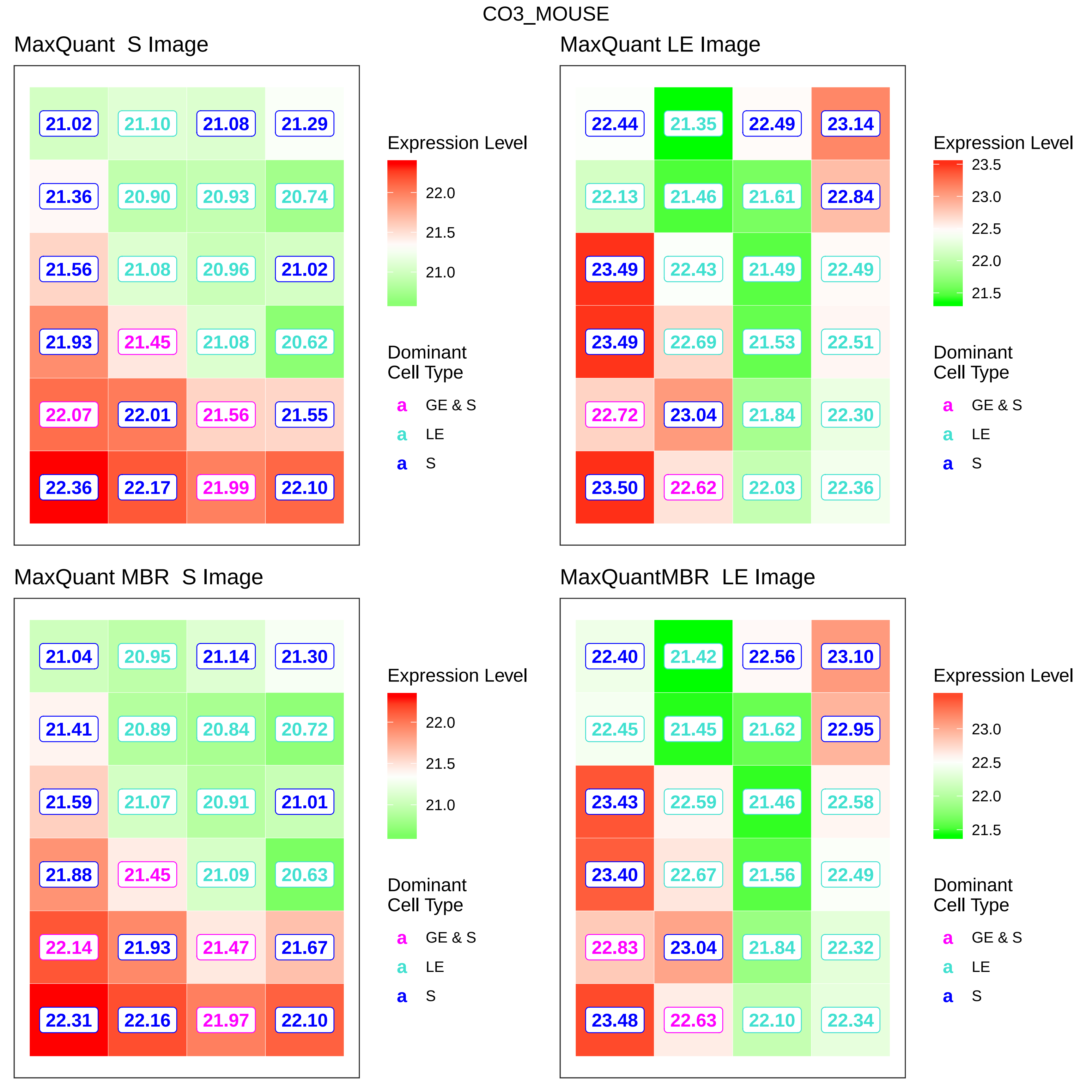

CO3_MOUSE
MaxQuant S Image
21.02 21.10 21.08 21.29
21.36 20.90 20.93 20.74
21.56 21.08 20.96 21.02
21.93 21.45 21.08 20.62
22.07 22.01 21.56 21.55
22.36 22.17 21.99 22.10
Expression Level
22.0
21.5
21.0
Dominant Cell Type
a GE & S
a LE
a S
MaxQuant LE Image
22.44 21.35 22.49 23.14
22.13 21.46 21.61 22.84
23.49 22.43 21.49 22.49
23.49 22.69 21.53 22.51
22.72 23.04 21.84 22.30
23.50 22.62 22.03 22.36
Expression Level
23.5
23.0
22.5
22.0
21.5
Dominant Cell Type
a GE & S
a LE
a S
MaxQuant MBR S Image
21.04 20.95 21.14 21.30
21.41 20.89 20.84 20.72
21.59 21.07 20.91 21.01
21.88 21.45 21.09 20.63
22.14 21.93 21.47 21.67
22.31 22.16 21.97 22.10
Expression Level
22.0
21.5
21.0
Dominant Cell Type
a GE & S
a LE
a S
MaxQuantMBR LE Image
22.40 21.42 22.56 23.10
22.45 21.45 21.62 22.95
23.43 22.59 21.46 22.58
23.40 22.67 21.56 22.49
22.83 23.04 21.84 22.32
23.48 22.63 22.10 22.34
Expression Level
23.0
22.5
22.0
21.5
Dominant Cell Type
a GE & S
a LE
a S

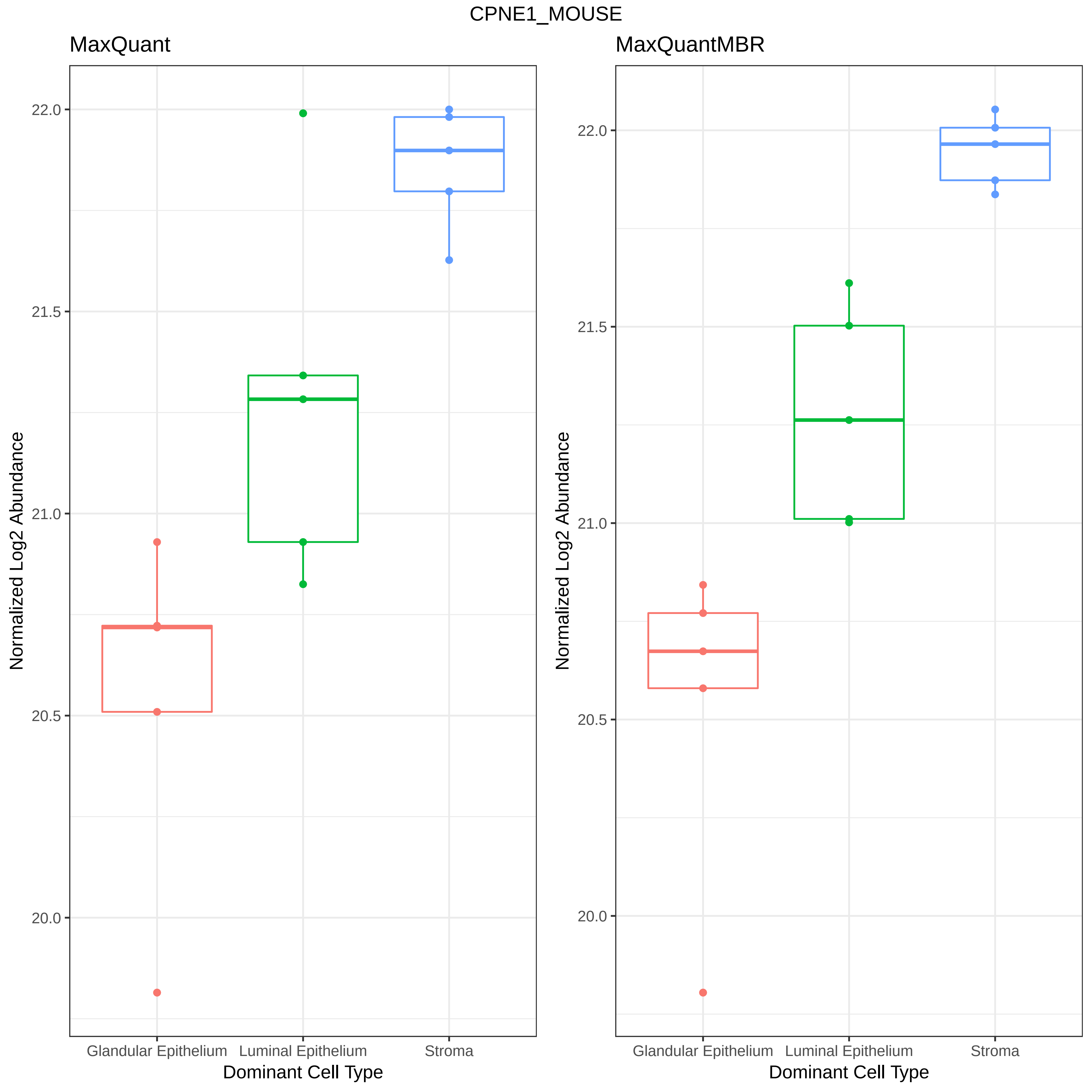

CPNE1_MOUSE
MaxQuant
MaxQuantMBR
Normalized Log2 Abundance
22.0
21.5
21.0
20.5
20.0
Glandular Epithelium
Luminal Epithelium
Stroma
Dominant Cell Type

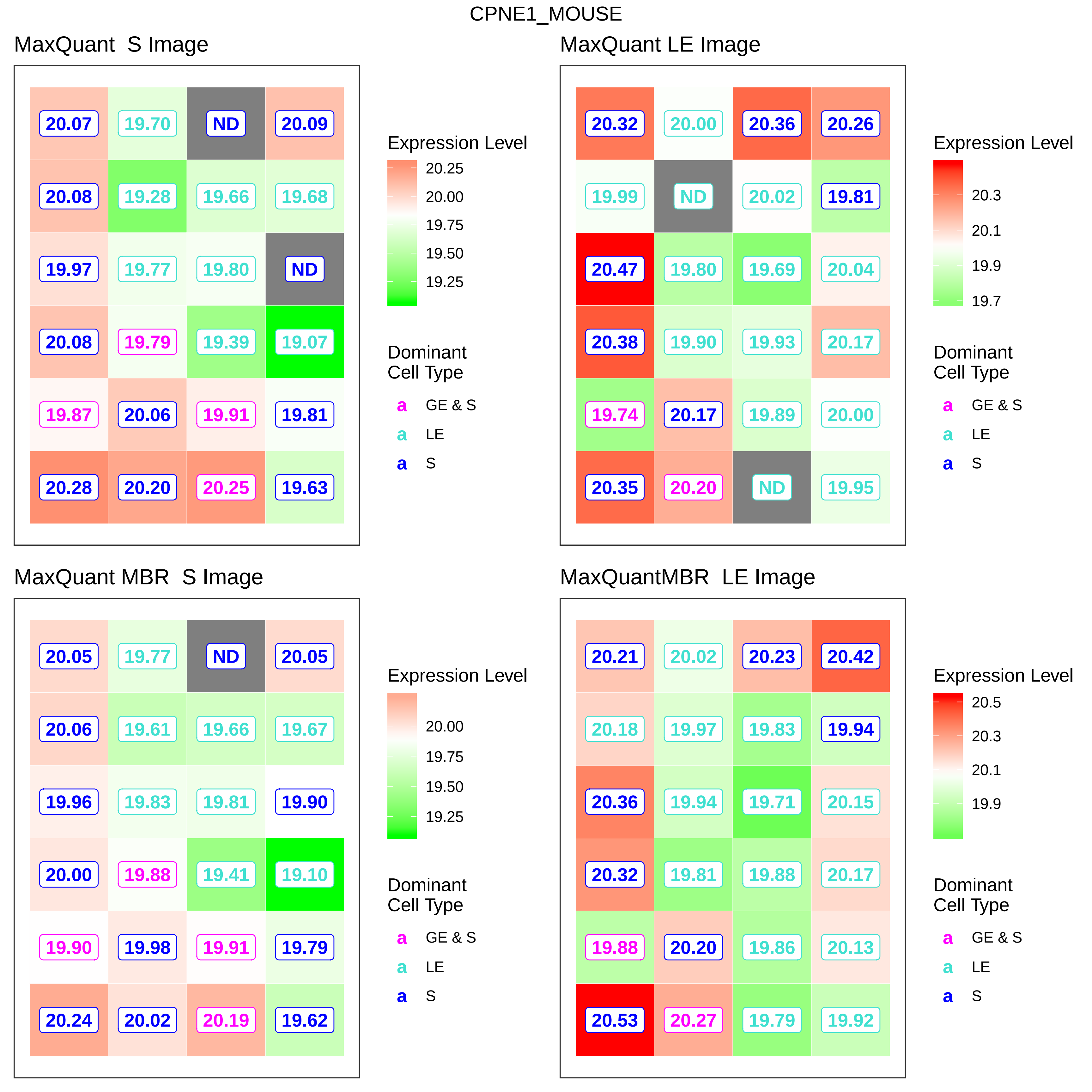
CPNE1_MOUSE
MaxQuant S Image
20.07 19.70 ND 20.09
20.08 19.28 19.66 19.68
19.97 19.77 19.80 ND
20.08 19.79 19.39 19.07
19.87 20.06 19.91 19.81
20.28 20.20 20.25 19.63
Expression Level
20.25
20.00
19.75
19.50
19.25
Dominant Cell Type
a GE & S
a LE
a S
MaxQuant LE Image
20.32 20.00 20.36 20.26
19.99 ND 20.02 19.81
20.47 19.80 19.69 20.04
20.38 19.90 19.93 20.17
19.74 20.17 19.89 20.00
20.35 20.20 ND 19.95
Expression Level
20.3
20.1
19.9
19.7
Dominant Cell Type
a GE & S
a LE
a S
MaxQuant MBR S Image
20.05 19.77 ND 20.05
20.06 19.61 19.66 19.67
19.96 19.83 19.81 19.90
20.00 19.88 19.41 19.10
19.90 19.98 19.91 19.79
20.24 20.02 20.19 19.62
Expression Level
20.00
19.75
19.50
19.25
Dominant Cell Type
a GE & S
a LE
a S
MaxQuantMBR LE Image
20.21 20.02 20.23 20.42
20.18 19.97 19.83 19.94
20.36 19.94 19.71 20.15
20.32 19.81 19.88 20.17
19.88 20.20 19.86 20.13
20.53 20.27 19.79 19.92
Expression Level
20.5
20.3
20.1
19.9
Dominant Cell Type
a GE & S
a LE
a S

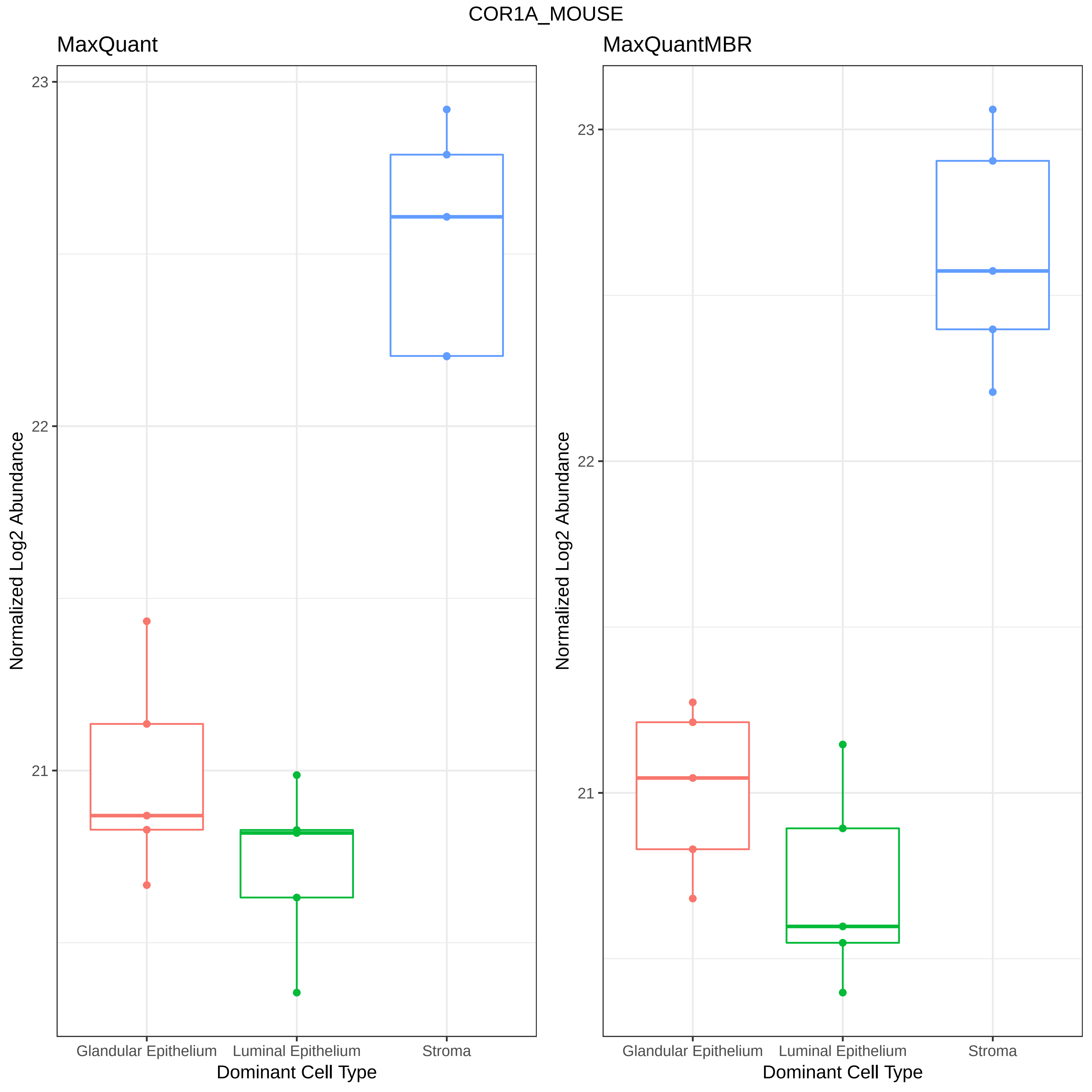
COR1A_MOUSE
MaxQuant
MaxQuantMBR
Normalized Log2 Abundance
23
22
21
Glandular Epithelium
Luminal Epithelium
Stroma
Dominant Cell Type

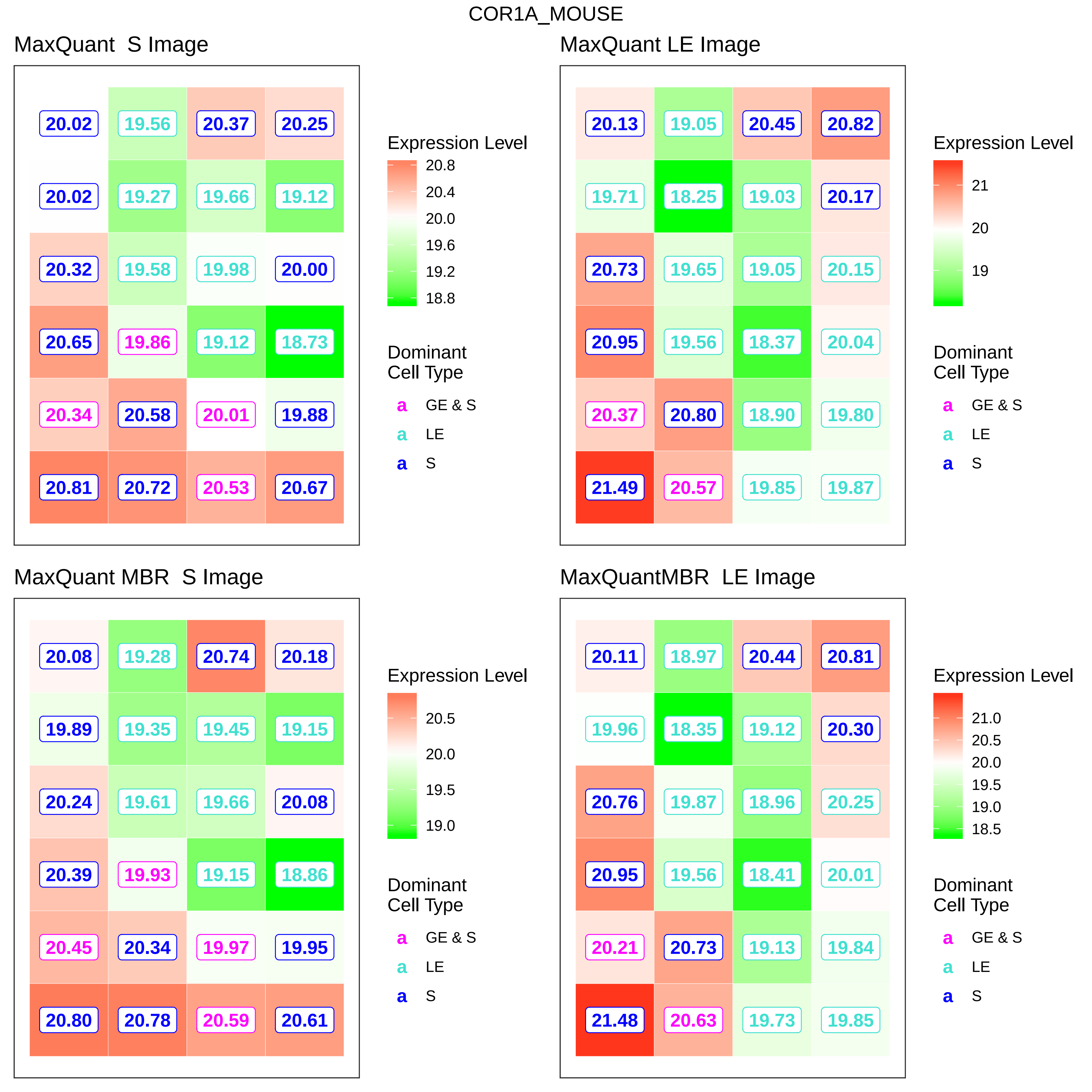
COR1A_MOUSE
MaxQuant S Image
20.02 19.56 20.37 20.25
20.02 19.27 19.66 19.12
20.32 19.58 19.98 20.00
20.65 19.86 19.12 18.73
20.34 20.58 20.01 19.88
20.81 20.72 20.53 20.67
Expression Level
20.8
20.4
20.0
19.6
19.2
18.8
Dominant Cell Type
a GE & S
a LE
a S
MaxQuant LE Image
20.13 19.05 20.45 20.82
19.71 18.25 19.03 20.17
20.73 19.65 19.05 20.15
20.95 19.56 18.37 20.04
20.37 20.80 18.90 19.80
21.49 20.57 19.85 19.87
Expression Level
21
20
19
Dominant Cell Type
a GE & S
a LE
a S
MaxQuant MBR S Image
20.08 19.28 20.74 20.18
19.89 19.35 19.45 19.15
20.24 19.61 19.66 20.08
20.39 19.93 19.15 18.86
20.45 20.34 19.97 19.95
20.80 20.78 20.59 20.61
Expression Level
20.5
20.0
19.5
19.0
Dominant Cell Type
a GE & S
a LE
a S
MaxQuantMBR LE Image
20.11 18.97 20.44 20.81
19.96 18.35 19.12 20.30
20.76 19.87 18.96 20.25
20.95 19.56 18.41 20.01
20.21 20.73 19.13 19.84
21.48 20.63 19.73 19.85
Expression Level
21.0
20.5
20.0
19.5
19.0
18.5
Dominant Cell Type
a GE & S
a LE
a S

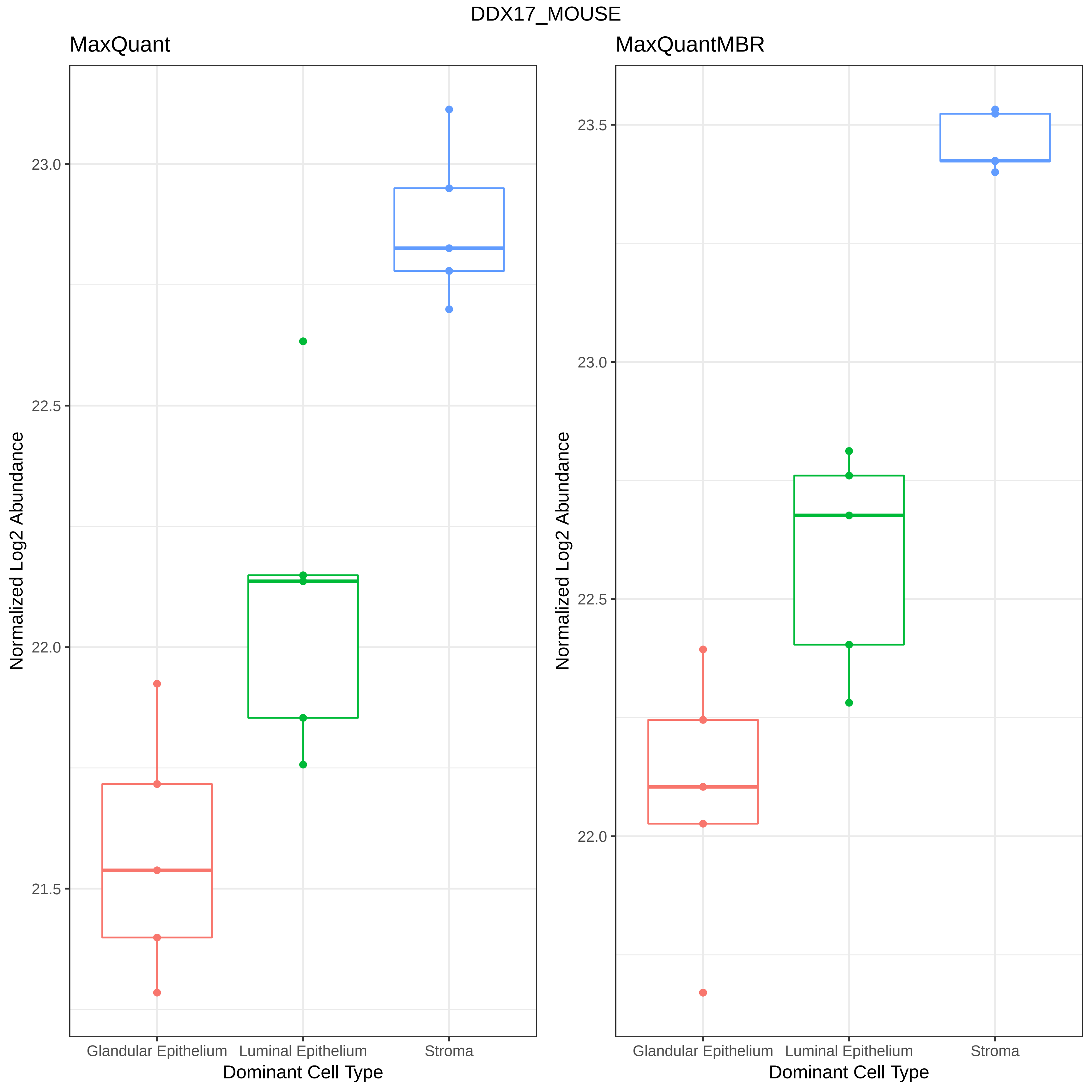

DDX17_MOUSE
MaxQuant
MaxQuantMBR
Normalized Log2 Abundance
23.0
22.5
22.0
21.5
23.5
23.0
22.5
22.0
Glandular Epithelium
Luminal Epithelium
Stroma
Dominant Cell Type

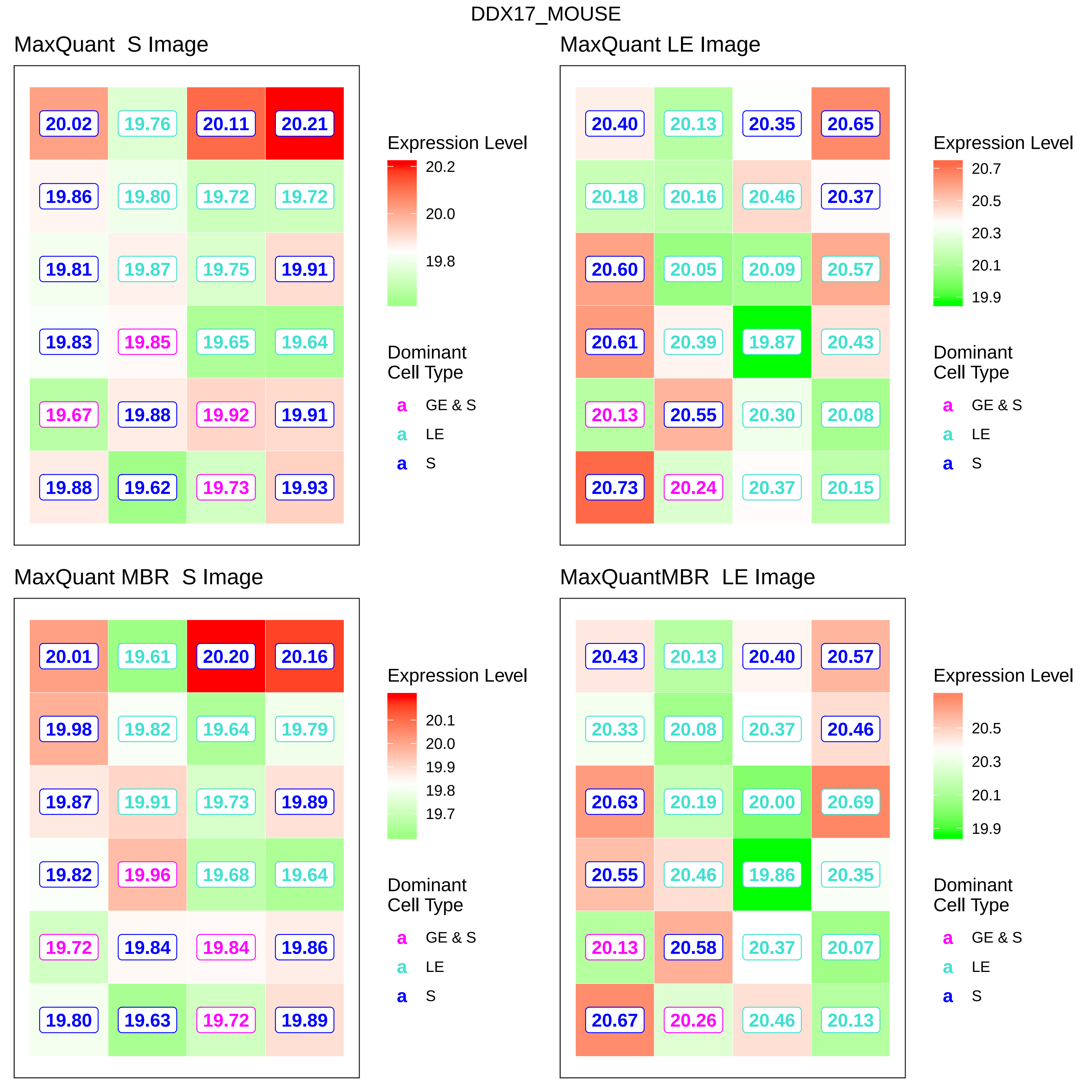
DDX17_MOUSE
MaxQuant S Image
20.02 19.76 20.11 20.21
19.86 19.80 19.72 19.72
19.81 19.87 19.75 19.91
19.83 19.85 19.65 19.64
19.67 19.88 19.92 19.91
19.88 19.62 19.73 19.93
Expression Level
20.2
20.0
19.8
Dominant Cell Type
a GE & S
a LE
a S
MaxQuant LE Image
20.40 20.13 20.35 20.65
20.18 20.16 20.46 20.37
20.60 20.05 20.09 20.57
20.61 20.39 19.87 20.43
20.13 20.55 20.30 20.08
20.73 20.24 20.37 20.15
Expression Level
20.7
20.5
20.3
20.1
19.9
Dominant Cell Type
a GE & S
a LE
a S
MaxQuant MBR S Image
20.01 19.61 20.20 20.16
19.98 19.82 19.64 19.79
19.87 19.91 19.73 19.89
19.82 19.96 19.68 19.64
19.72 19.84 19.84 19.86
19.80 19.63 19.72 19.89
Expression Level
20.1
20.0
19.9
19.8
19.7
Dominant Cell Type
a GE & S
a LE
a S
MaxQuantMBR LE Image
20.43 20.13 20.40 20.57
20.33 20.08 20.37 20.46
20.63 20.19 20.00 20.69
20.55 20.46 19.86 20.35
20.13 20.58 20.37 20.07
20.67 20.26 20.46 20.13
Expression Level
20.5
20.3
20.1
19.9
Dominant Cell Type
a GE & S
a LE
a S

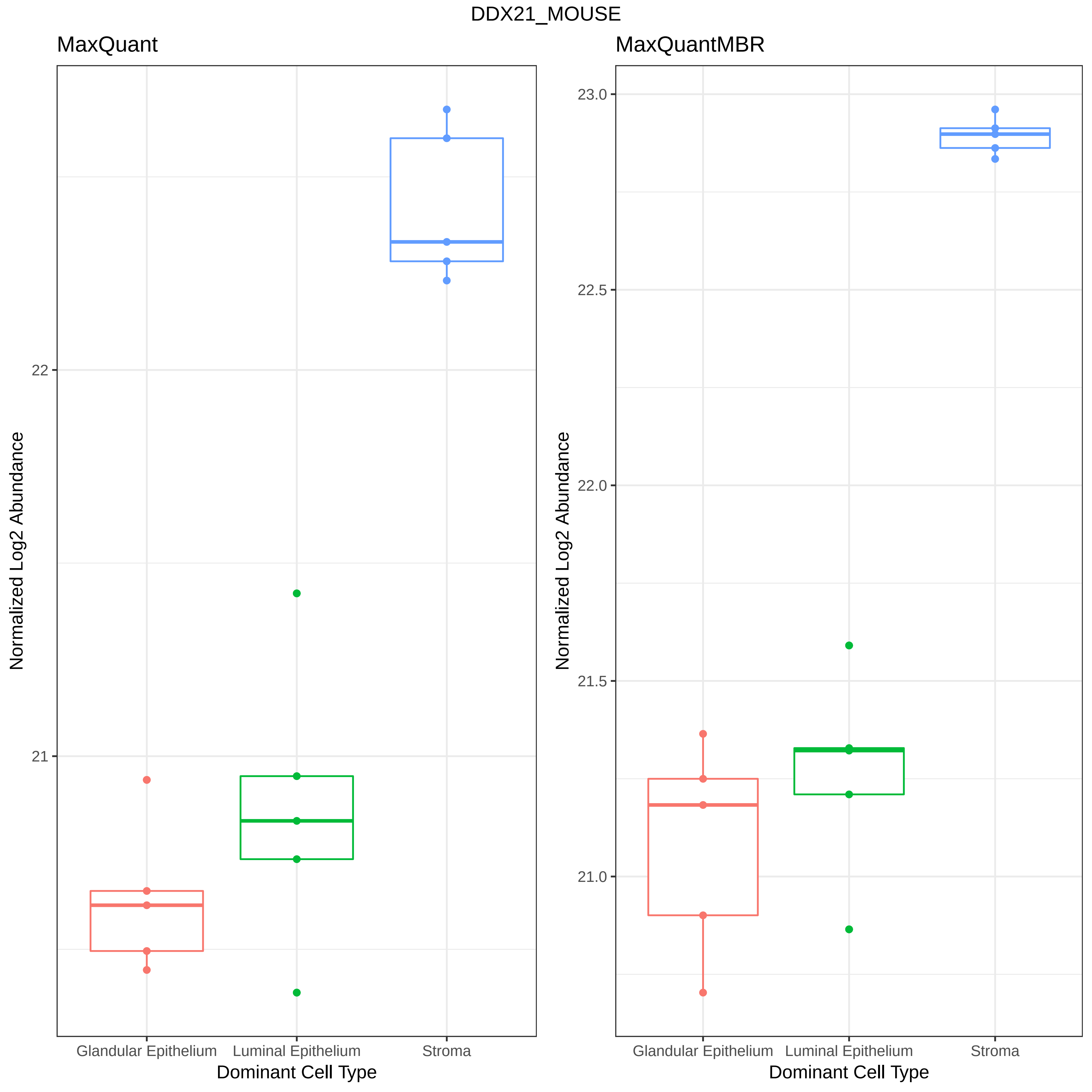

DDX21_MOUSE
MaxQuant
MaxQuantMBR
Normalized Log2 Abundance
22
21
23.0
22.5
22.0
21.5
21.0
Glandular Epithelium
Luminal Epithelium
Stroma
Dominant Cell Type

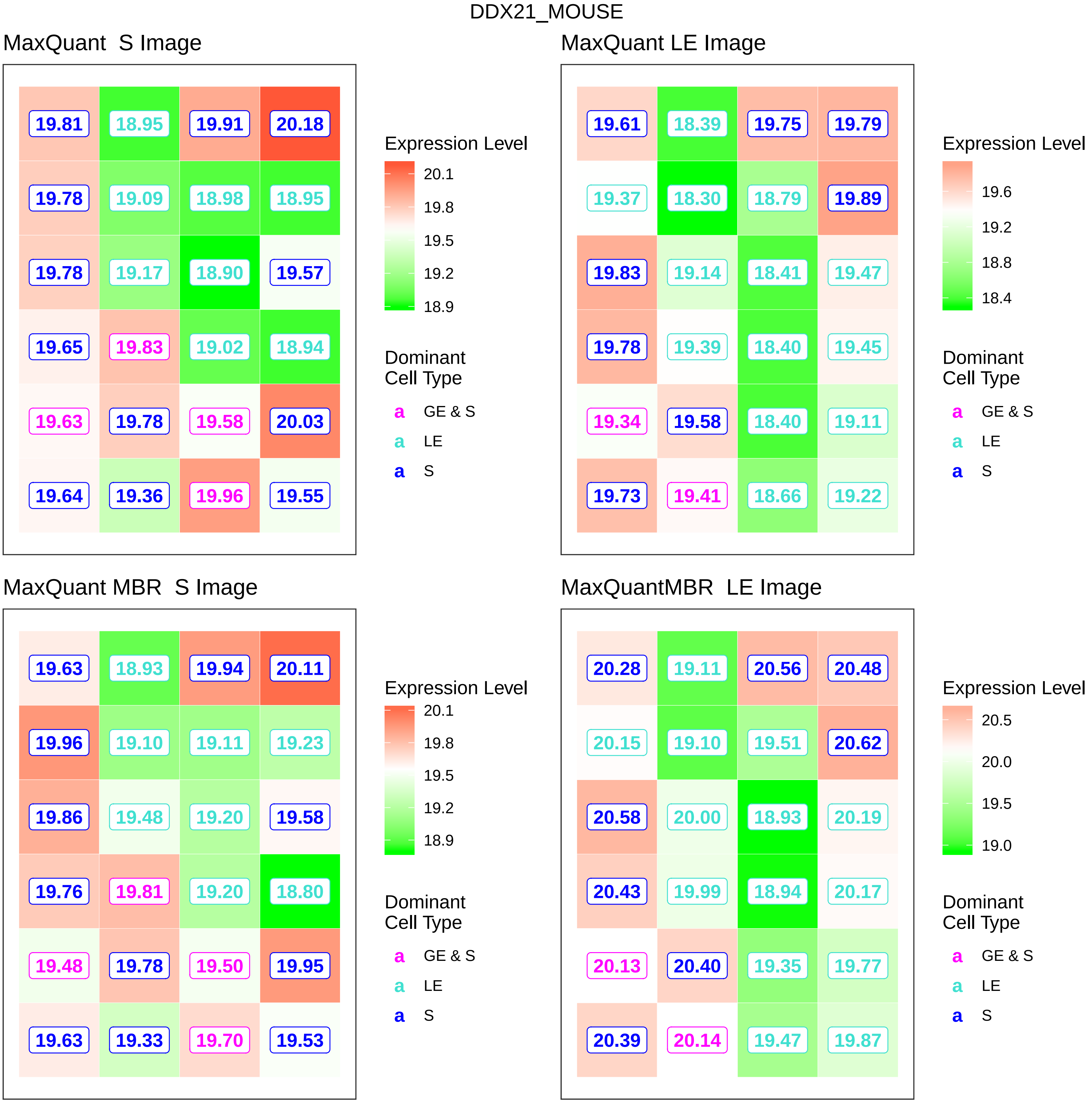
DDX21_MOUSE
MaxQuant S Image
19.81 18.95 19.91 20.18
19.78 19.09 18.98 18.95
19.78 19.17 18.90 19.57
19.65 19.83 19.02 18.94
19.63 19.78 19.58 20.03
19.64 19.36 19.96 19.55
Expression Level
20.1
19.8
19.5
19.2
18.9
Dominant Cell Type
a GE & S
a LE
a S
MaxQuant LE Image
19.61 18.39 19.75 19.79
19.37 18.30 18.79 19.89
19.83 19.14 18.41 19.47
19.78 19.39 18.40 19.45
19.34 19.58 18.40 19.11
19.73 19.41 18.66 19.22
Expression Level
19.6
19.2
18.8
18.4
Dominant Cell Type
a GE & S
a LE
a S
MaxQuant MBR S Image
19.63 18.93 19.94 20.11
19.96 19.10 19.11 19.23
19.86 19.48 19.20 19.58
19.76 19.81 19.20 18.80
19.48 19.78 19.50 19.95
19.63 19.33 19.70 19.53
Expression Level
20.1
19.8
19.5
19.2
18.9
Dominant Cell Type
a GE & S
a LE
a S
MaxQuantMBR LE Image
20.28 19.11 20.56 20.48
20.15 19.10 19.51 20.62
20.58 20.00 18.93 20.19
20.43 19.99 18.94 20.17
20.13 20.40 19.35 19.77
20.39 20.14 19.47 19.87
Expression Level
20.5
20.0
19.5
19.0
Dominant Cell Type
a GE & S
a LE
a S

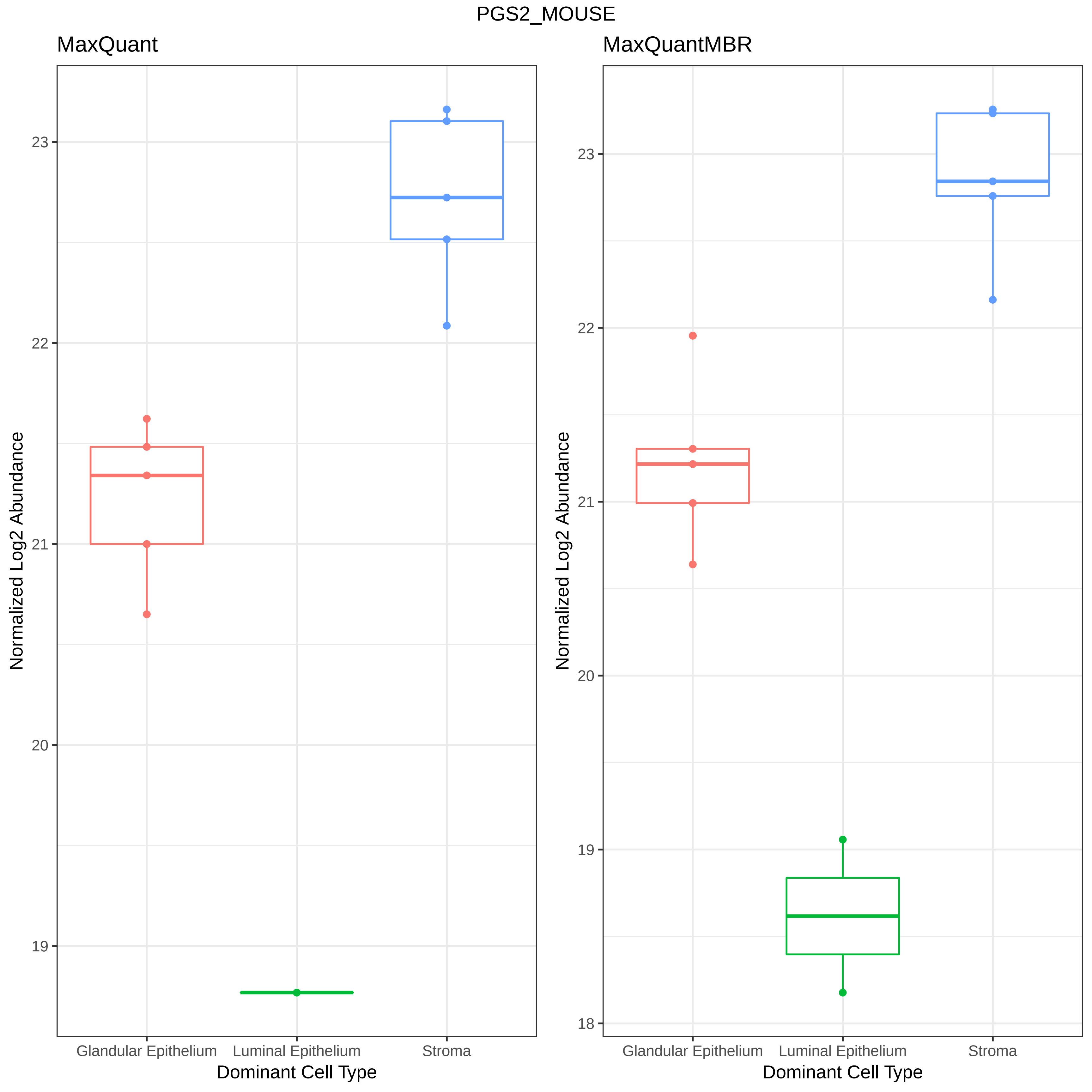
PGS2_MOUSE
MaxQuant
MaxQuantMBR
Normalized Log2 Abundance
23
22
21
20
19
18
Glandular Epithelium
Luminal Epithelium
Stroma
Dominant Cell Type

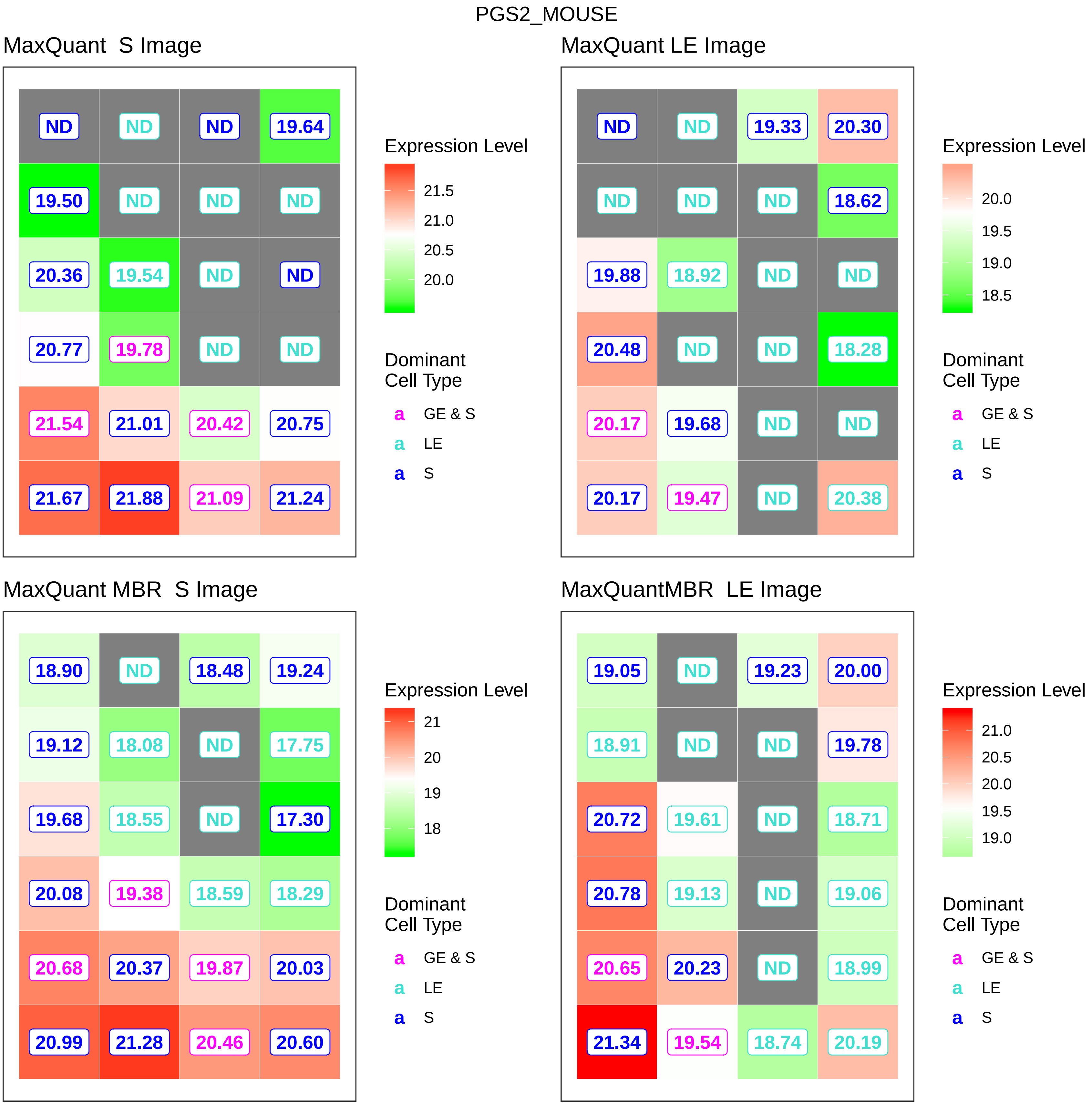
PGS2_MOUSE
MaxQuant S Image
ND ND ND 19.64
19.50 ND ND ND
20.36 19.54 ND ND
20.77 19.78 ND ND
21.54 21.01 20.42 20.75
21.67 21.88 21.09 21.24
Expression Level
21.5 21.0 20.5 20.0
Dominant Cell Type
a GE & S
a LE
a S
MaxQuant LE Image
ND ND 19.33 20.30
ND ND ND 18.62
19.88 18.92 ND ND
20.48 ND ND 18.28
20.17 19.68 ND ND
20.17 19.47 ND 20.38
Expression Level
20.0 19.5 19.0 18.5
Dominant Cell Type
a GE & S
a LE
a S
MaxQuant MBR S Image
18.90 ND 18.48 19.24
19.12 18.08 ND 17.75
19.68 18.55 ND 17.30
20.08 19.38 18.59 18.29
20.68 20.37 19.87 20.03
20.99 21.28 20.46 20.60
Expression Level
21 20 19 18
Dominant Cell Type
a GE & S
a LE
a S
MaxQuantMBR LE Image
19.05 ND 19.23 20.00
18.91 ND ND 19.78
20.72 19.61 ND 18.71
20.78 19.13 ND 19.06
20.65 20.23 ND 18.99
21.34 19.54 18.74 20.19
Expression Level
21.0 20.5 20.0 19.5 19.0
Dominant Cell Type
a GE & S
a LE
a S

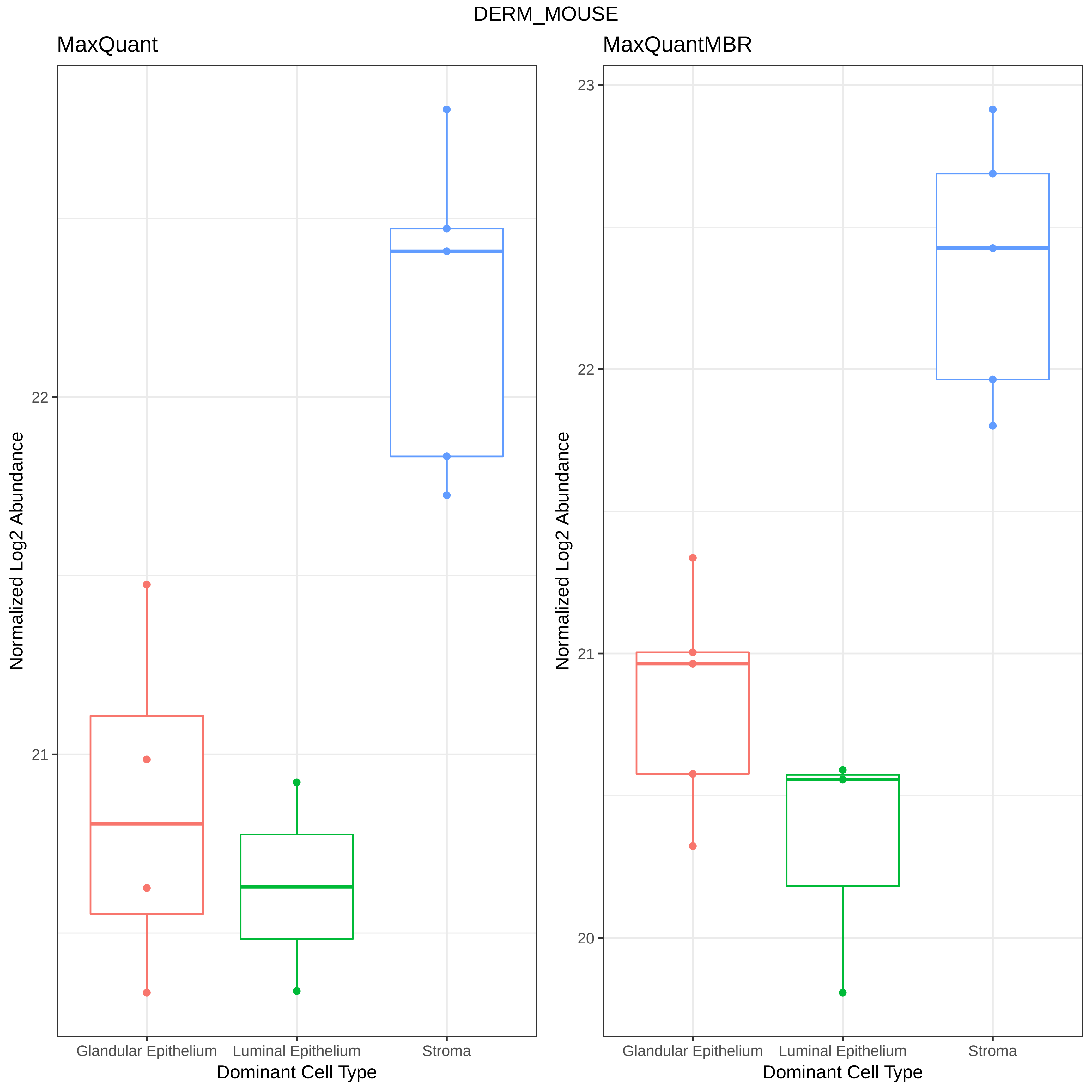
DERM_MOUSE
MaxQuant
MaxQuantMBR
Normalized Log2 Abundance
22
21
23
20
Glandular Epithelium
Luminal Epithelium
Stroma
Dominant Cell Type

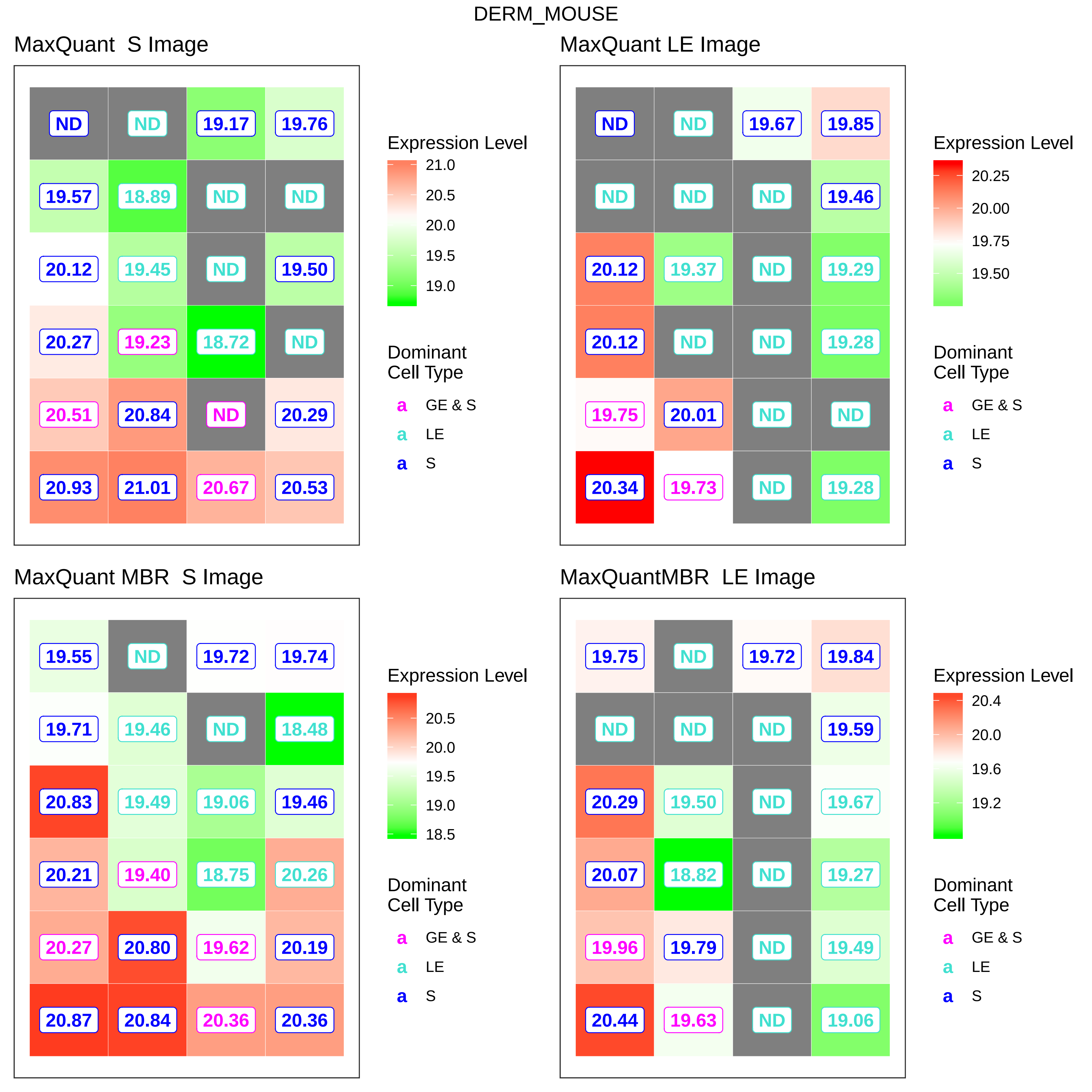
DERM_MOUSE
MaxQuant S Image
ND ND 19.17 19.76
19.57 18.89 ND ND
20.12 19.45 ND 19.50
20.27 19.23 18.72 ND
20.51 20.84 ND 20.29
20.93 21.01 20.67 20.53
Expression Level
21.0
20.5
20.0
19.5
19.0
Dominant Cell Type
a GE & S
a LE
a S
MaxQuant LE Image
ND ND 19.67 19.85
ND ND ND 19.46
20.12 19.37 ND 19.29
20.12 ND ND 19.28
19.75 20.01 ND ND
20.34 19.73 ND 19.28
Expression Level
20.25
20.00
19.75
19.50
Dominant Cell Type
a GE & S
a LE
a S
MaxQuant MBR S Image
19.55 ND 19.72 19.74
19.71 19.46 ND 18.48
20.83 19.49 19.06 19.46
20.21 19.40 18.75 20.26
20.27 20.80 19.62 20.19
20.87 20.84 20.36 20.36
Expression Level
20.5
20.0
19.5
19.0
18.5
Dominant Cell Type
a GE & S
a LE
a S
MaxQuantMBR LE Image
19.75 ND 19.72 19.84
ND ND ND 19.59
20.29 19.50 ND 19.67
20.07 18.82 ND 19.27
19.96 19.79 ND 19.49
20.44 19.63 ND 19.06
Expression Level
20.4
20.0
19.6
19.2
Dominant Cell Type
a GE & S
a LE
a S

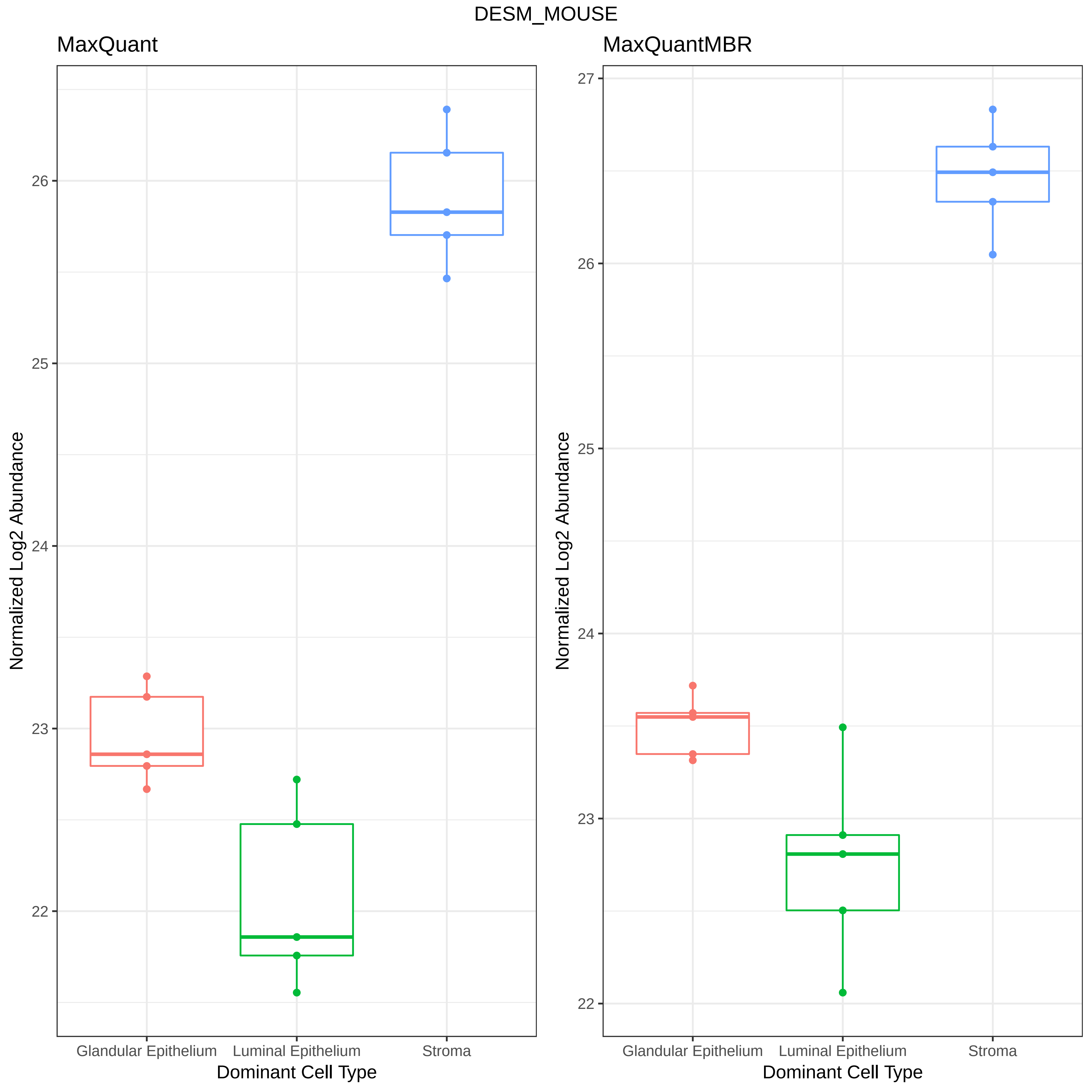
DESM_MOUSE
MaxQuant
MaxQuantMBR
Normalized Log2 Abundance
22
23
24
25
26
27
Glandular Epithelium
Luminal Epithelium
Stroma
Dominant Cell Type

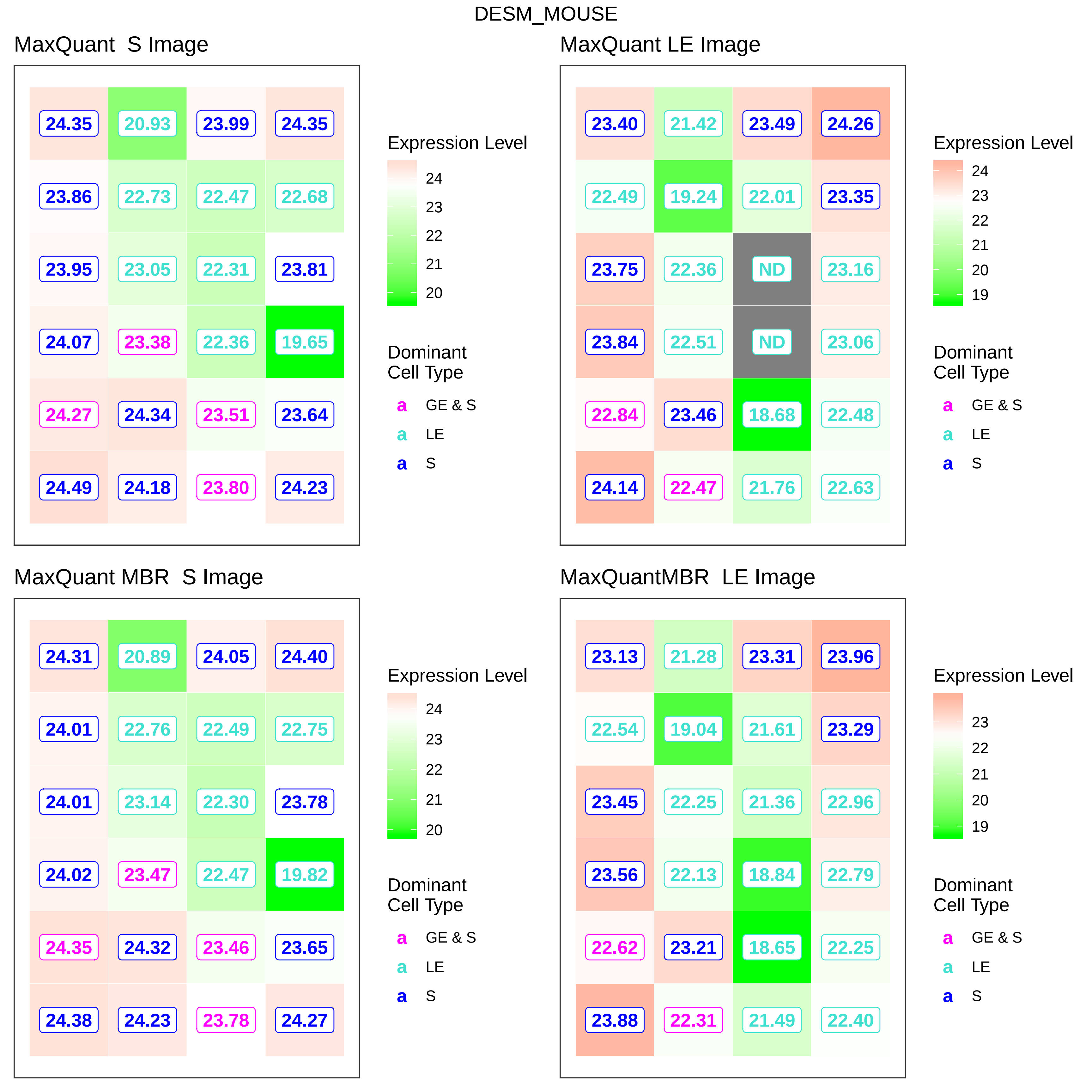

DESM_MOUSE
MaxQuant S Image
24.35 20.93 23.99 24.35
23.86 22.73 22.47 22.68
23.95 23.05 22.31 23.81
24.07 23.38 22.36 19.65
24.27 24.34 23.51 23.64
24.49 24.18 23.80 24.23
Expression Level
24 23 22 21 20
Dominant Cell Type
a GE & S
a LE
a S
MaxQuant LE Image
23.40 21.42 23.49 24.26
22.49 19.24 22.01 23.35
23.75 22.36 ND 23.16
23.84 22.51 ND 23.06
22.84 23.46 18.68 22.48
24.14 22.47 21.76 22.63
Expression Level
24 23 22 21 20 19
Dominant Cell Type
a GE & S
a LE
a S
MaxQuant MBR S Image
24.31 20.89 24.05 24.40
24.01 22.76 22.49 22.75
24.01 23.14 22.30 23.78
24.02 23.47 22.47 19.82
24.35 24.32 23.46 23.65
24.38 24.23 23.78 24.27
Expression Level
24 23 22 21 20
Dominant Cell Type
a GE & S
a LE
a S
MaxQuantMBR LE Image
23.13 21.28 23.31 23.96
22.54 19.04 21.61 23.29
23.45 22.25 21.36 22.96
23.56 22.13 18.84 22.79
22.62 23.21 18.65 22.25
23.88 22.31 21.49 22.40
Expression Level
23 22 21 20 19
Dominant Cell Type
a GE & S
a LE
a S

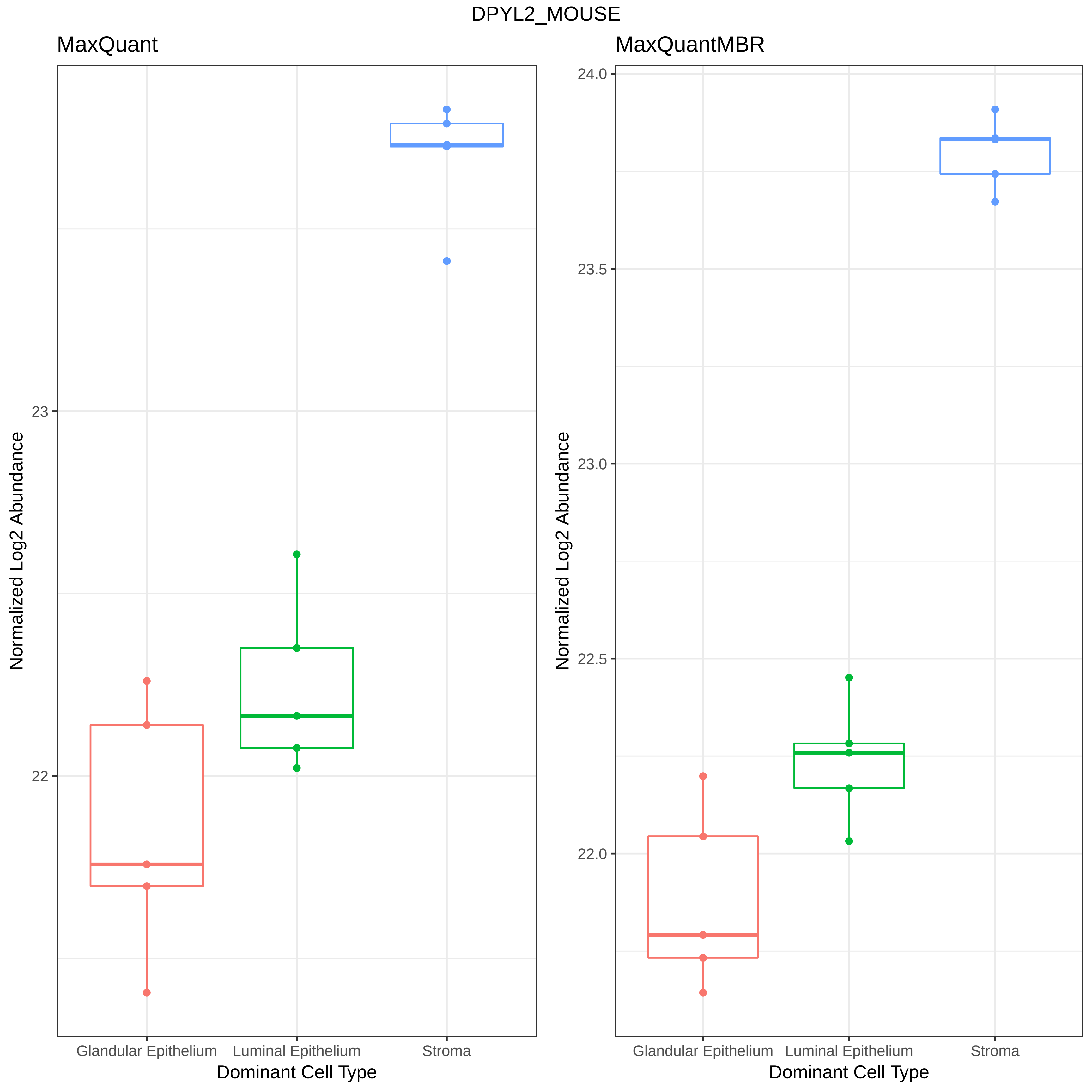

DPYL2_MOUSE
MaxQuant
MaxQuantMBR
Normalized Log2 Abundance
23
22
24.0
23.5
23.0
22.5
22.0
Glandular Epithelium
Luminal Epithelium
Stroma
Dominant Cell Type

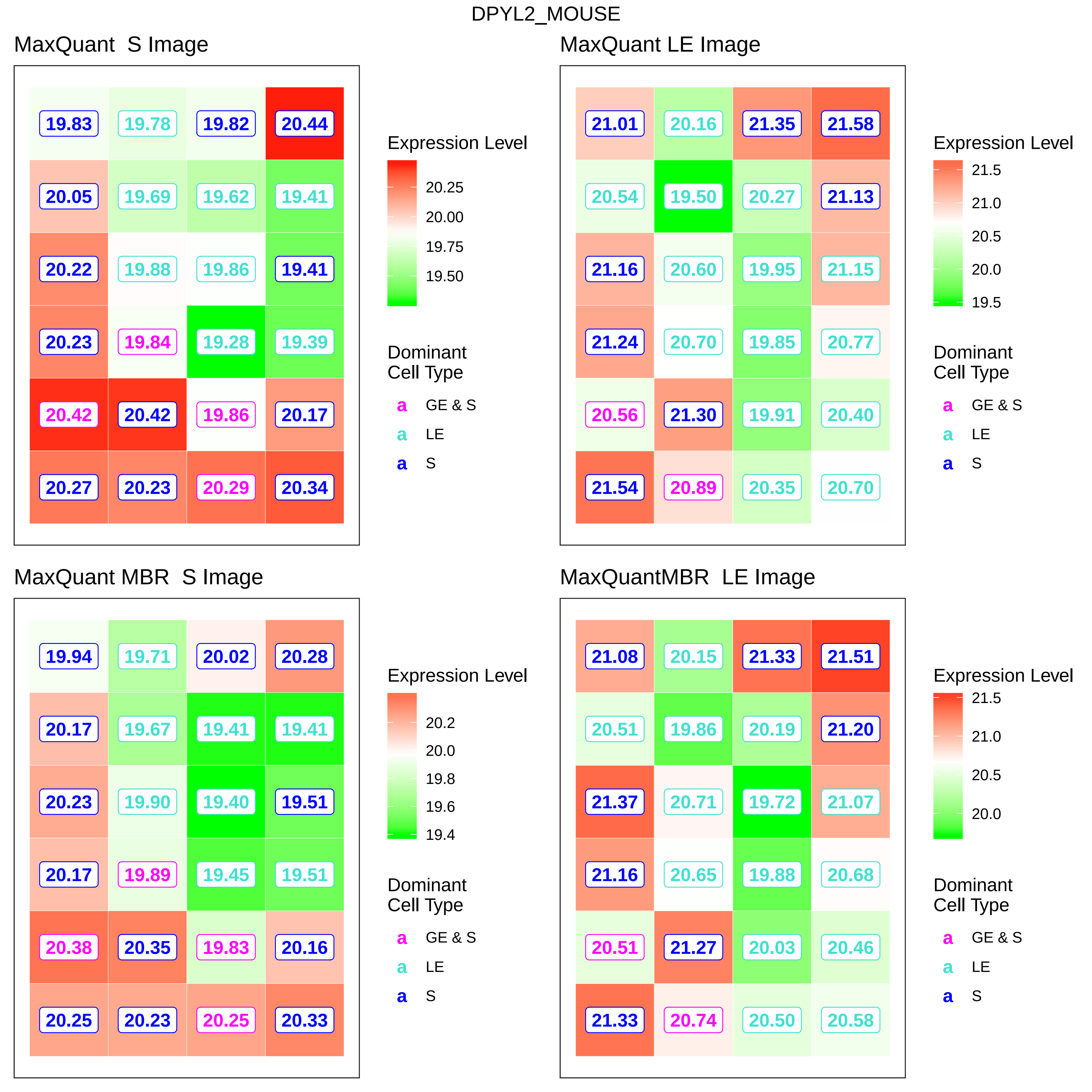
DPYL2_MOUSE
MaxQuant S Image
19.83 19.78 19.82 20.44
20.05 19.69 19.62 19.41
20.22 19.88 19.86 19.41
20.23 19.84 19.28 19.39
20.42 20.42 19.86 20.17
20.27 20.23 20.29 20.34
Expression Level
20.25
20.00
19.75
19.50
Dominant Cell Type
a GE & S
a LE
a S
MaxQuant LE Image
21.01 20.16 21.35 21.58
20.54 19.50 20.27 21.13
21.16 20.60 19.95 21.15
21.24 20.70 19.85 20.77
20.56 21.30 19.91 20.40
21.54 20.89 20.35 20.70
Expression Level
21.5
21.0
20.5
20.0
19.5
Dominant Cell Type
a GE & S
a LE
a S
MaxQuant MBR S Image
19.94 19.71 20.02 20.28
20.17 19.67 19.41 19.41
20.23 19.90 19.40 19.51
20.17 19.89 19.45 19.51
20.38 20.35 19.83 20.16
20.25 20.23 20.25 20.33
Expression Level
20.2
20.0
19.8
19.6
19.4
Dominant Cell Type
a GE & S
a LE
a S
MaxQuantMBR LE Image
21.08 20.15 21.33 21.51
20.51 19.86 20.19 21.20
21.37 20.71 19.72 21.07
21.16 20.65 19.88 20.68
20.51 21.27 20.03 20.46
21.33 20.74 20.50 20.58
Expression Level
21.5
21.0
20.5
20.0
Dominant Cell Type
a GE & S
a LE
a S

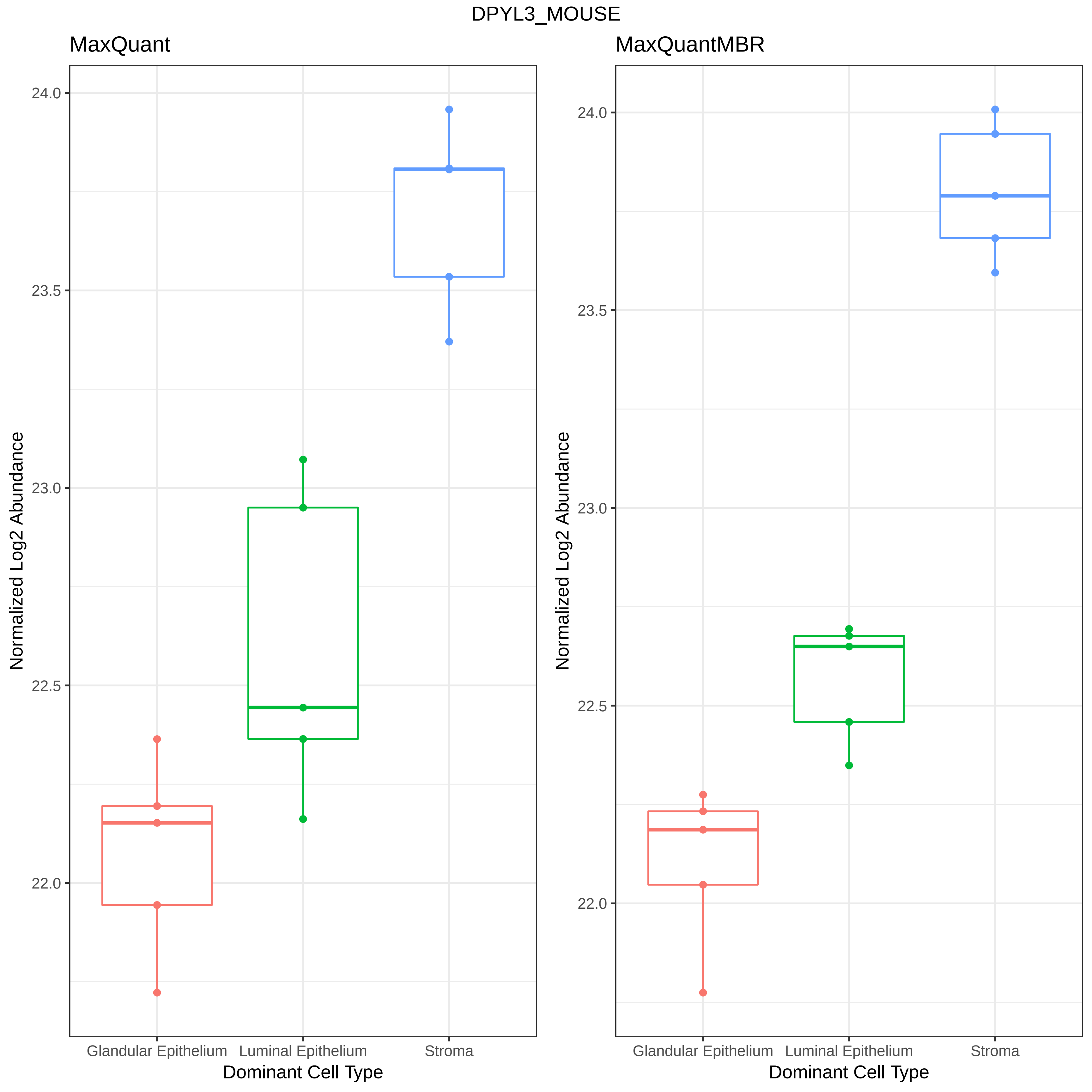
DPYL3_MOUSE
MaxQuant
MaxQuantMBR
Normalized Log2 Abundance
24.0
23.5
23.0
22.5
22.0
Glandular Epithelium
Luminal Epithelium
Stroma
Dominant Cell Type

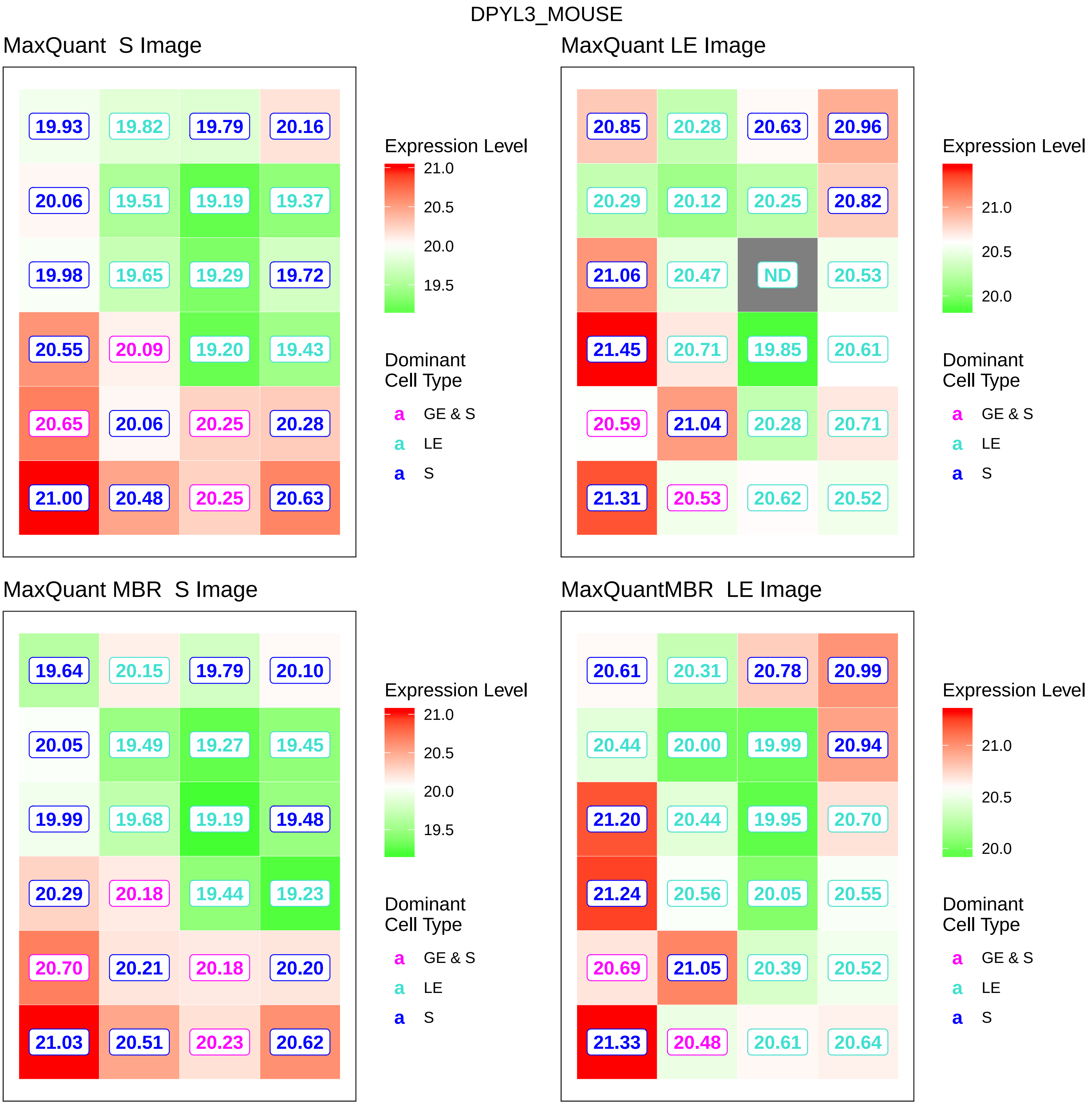

DPYL3_MOUSE
MaxQuant S Image
19.93 19.82 19.79 20.16
20.06 19.51 19.19 19.37
19.98 19.65 19.29 19.72
20.55 20.09 19.20 19.43
20.65 20.06 20.25 20.28
21.00 20.48 20.25 20.63
Expression Level
21.0
20.5
20.0
19.5
Dominant Cell Type
a GE & S
a LE
a S
MaxQuant LE Image
20.85 20.28 20.63 20.96
20.29 20.12 20.25 20.82
21.06 20.47 ND 20.53
21.45 20.71 19.85 20.61
20.59 21.04 20.28 20.71
21.31 20.53 20.62 20.52
Expression Level
21.0
20.5
20.0
Dominant Cell Type
a GE & S
a LE
a S
MaxQuant MBR S Image
19.64 20.15 19.79 20.10
20.05 19.49 19.27 19.45
19.99 19.68 19.19 19.48
20.29 20.18 19.44 19.23
20.70 20.21 20.18 20.20
21.03 20.51 20.23 20.62
Expression Level
21.0
20.5
20.0
19.5
Dominant Cell Type
a GE & S
a LE
a S
MaxQuantMBR LE Image
20.61 20.31 20.78 20.99
20.44 20.00 19.99 20.94
21.20 20.44 19.95 20.70
21.24 20.56 20.05 20.55
20.69 21.05 20.39 20.52
21.33 20.48 20.61 20.64
Expression Level
21.0
20.5
20.0
Dominant Cell Type
a GE & S
a LE
a S

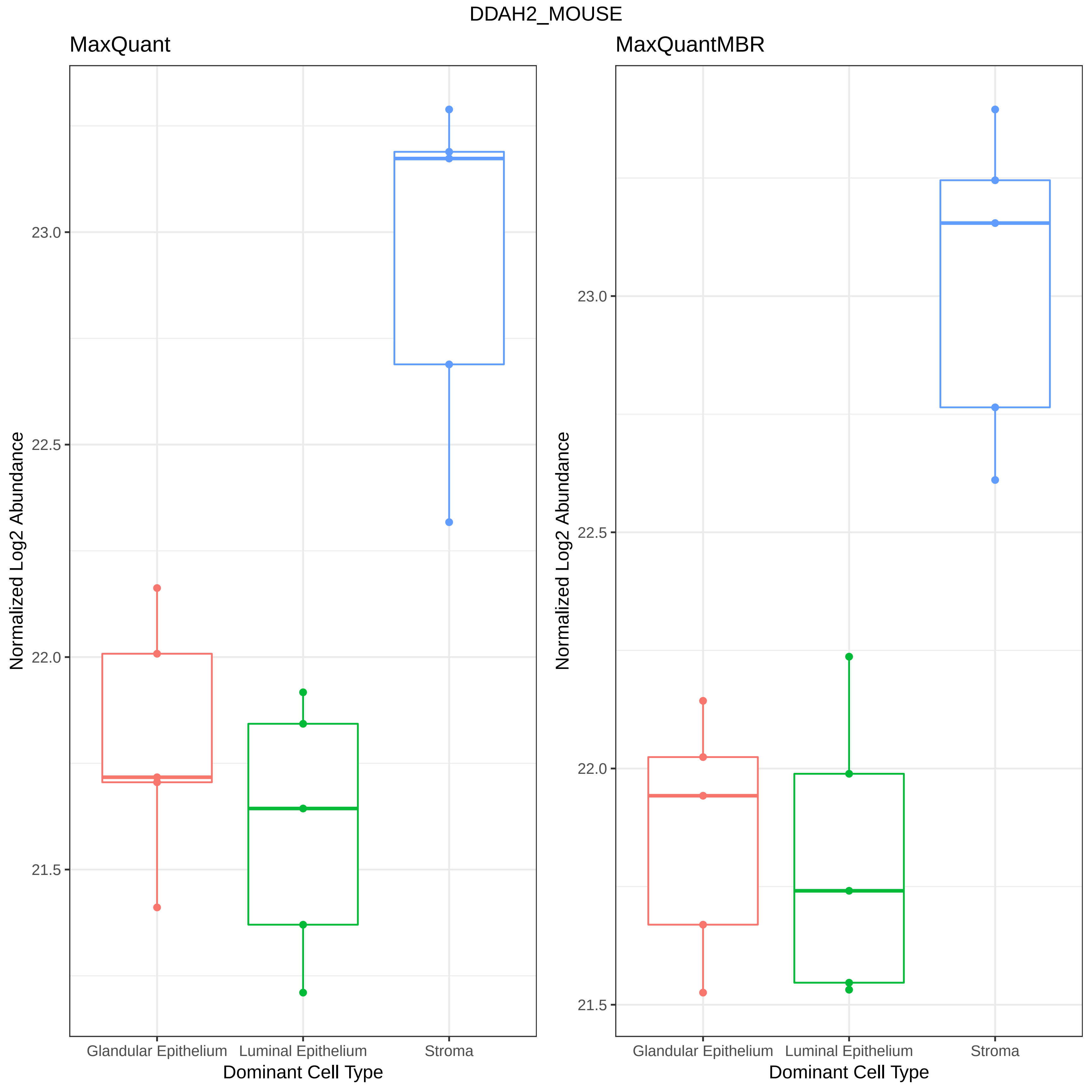
DDAH2_MOUSE
MaxQuant
MaxQuantMBR
Normalized Log2 Abundance
23.0
22.5
22.0
21.5
Glandular Epithelium
Luminal Epithelium
Stroma
Dominant Cell Type

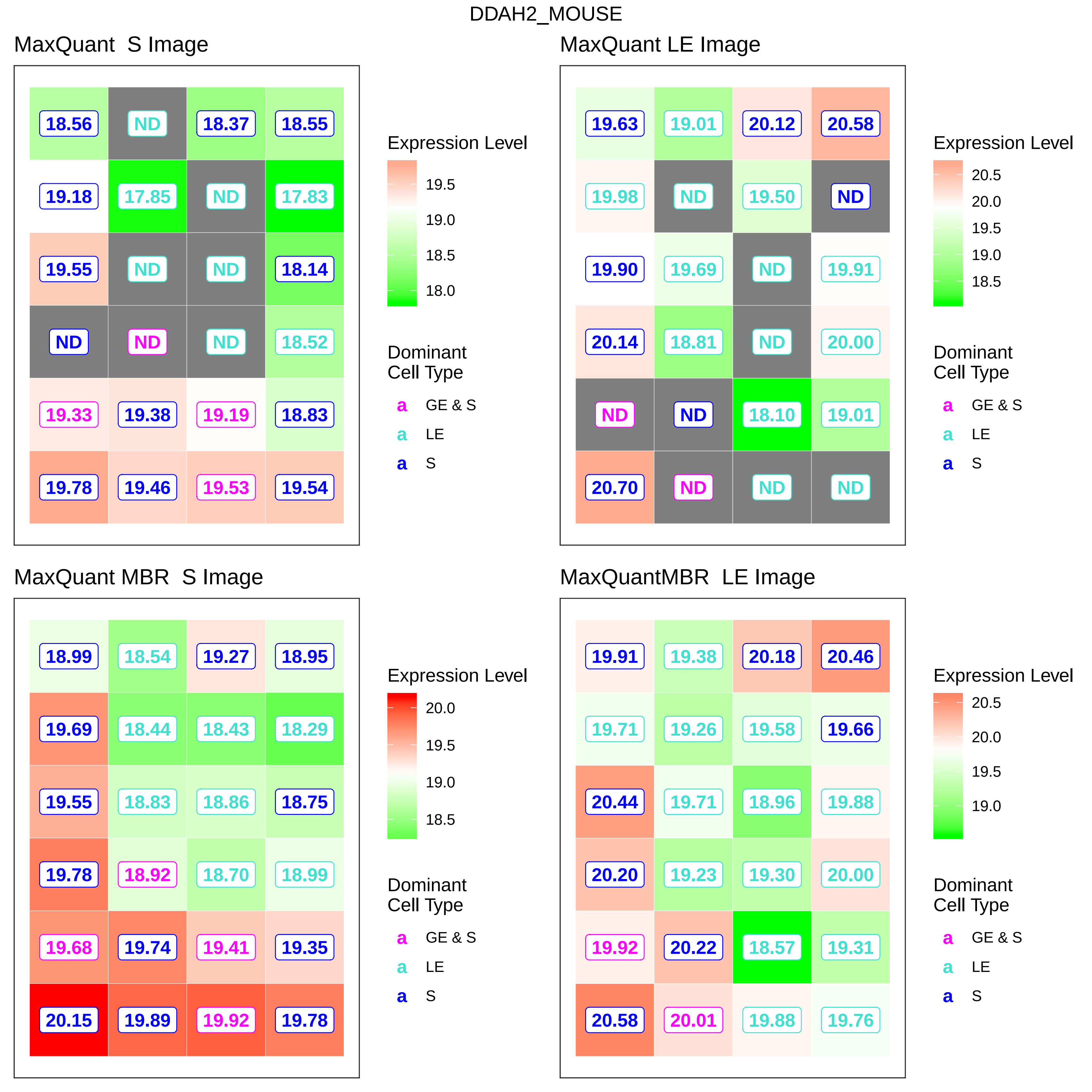
DDAH2_MOUSE
MaxQuant S Image
18.56 ND 18.37 18.55
19.18 17.85 ND 17.83
19.55 ND ND 18.14
ND ND ND 18.52
19.33 19.38 19.19 18.83
19.78 19.46 19.53 19.54
Expression Level
19.5
19.0
18.5
18.0
Dominant Cell Type
a GE & S
a LE
a S
MaxQuant LE Image
19.63 19.01 20.12 20.58
19.98 ND 19.50 ND
19.90 19.69 ND 19.91
20.14 18.81 ND 20.00
ND ND 18.10 19.01
20.70 ND ND ND
Expression Level
20.5
20.0
19.5
19.0
18.5
Dominant Cell Type
a GE & S
a LE
a S
MaxQuant MBR S Image
18.99 18.54 19.27 18.95
19.69 18.44 18.43 18.29
19.55 18.83 18.86 18.75
19.78 18.92 18.70 18.99
19.68 19.74 19.41 19.35
20.15 19.89 19.92 19.78
Expression Level
20.0
19.5
19.0
18.5
Dominant Cell Type
a GE & S
a LE
a S
MaxQuantMBR LE Image
19.91 19.38 20.18 20.46
19.71 19.26 19.58 19.66
20.44 19.71 18.96 19.88
20.20 19.23 19.30 20.00
19.92 20.22 18.57 19.31
20.58 20.01 19.88 19.76
Expression Level
20.5
20.0
19.5
19.0
Dominant Cell Type
a GE & S
a LE
a S

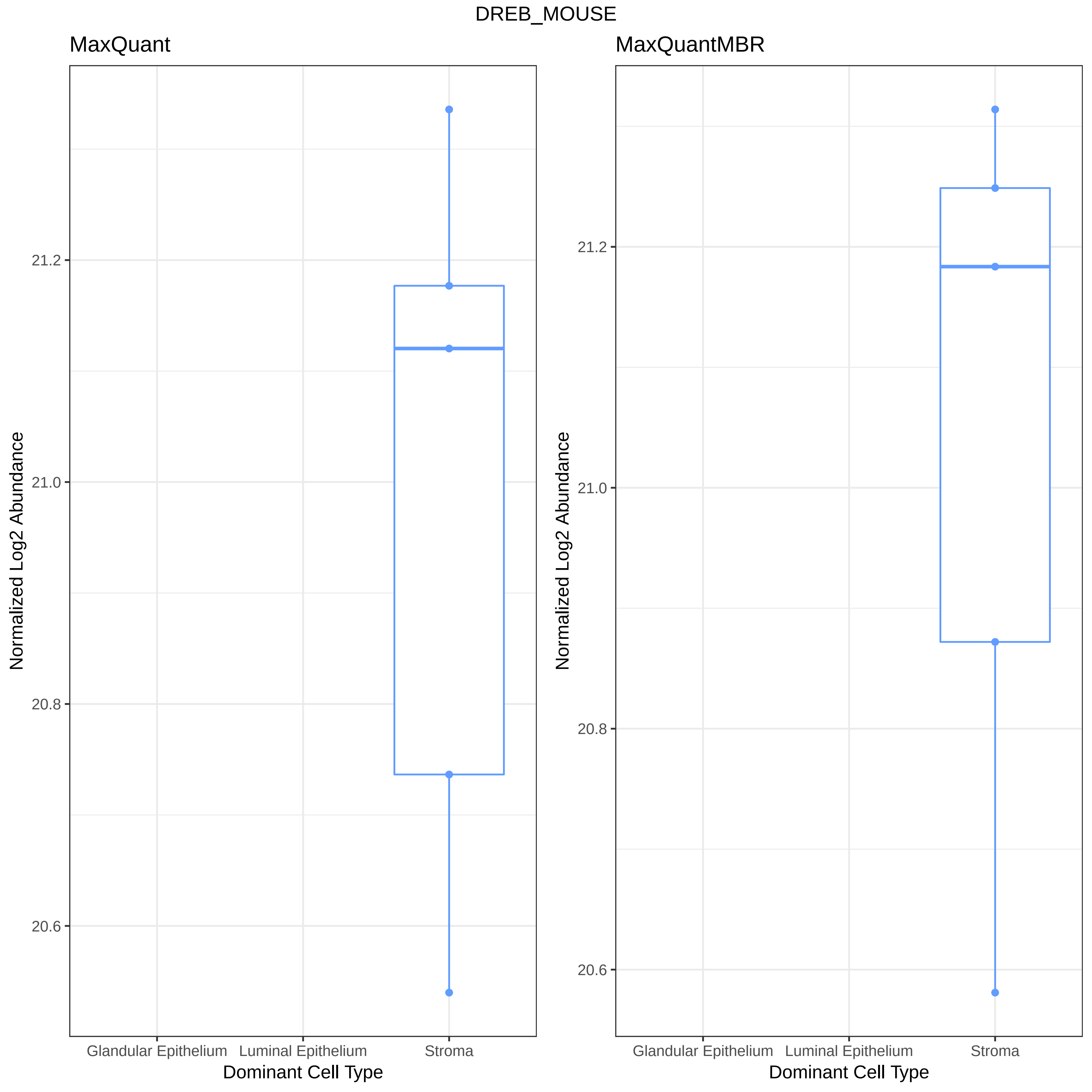
DREB_MOUSE
MaxQuant
MaxQuantMBR
Normalized Log2 Abundance
21.2
21.0
20.8
20.6
Glandular Epithelium
Luminal Epithelium
Stroma
Dominant Cell Type

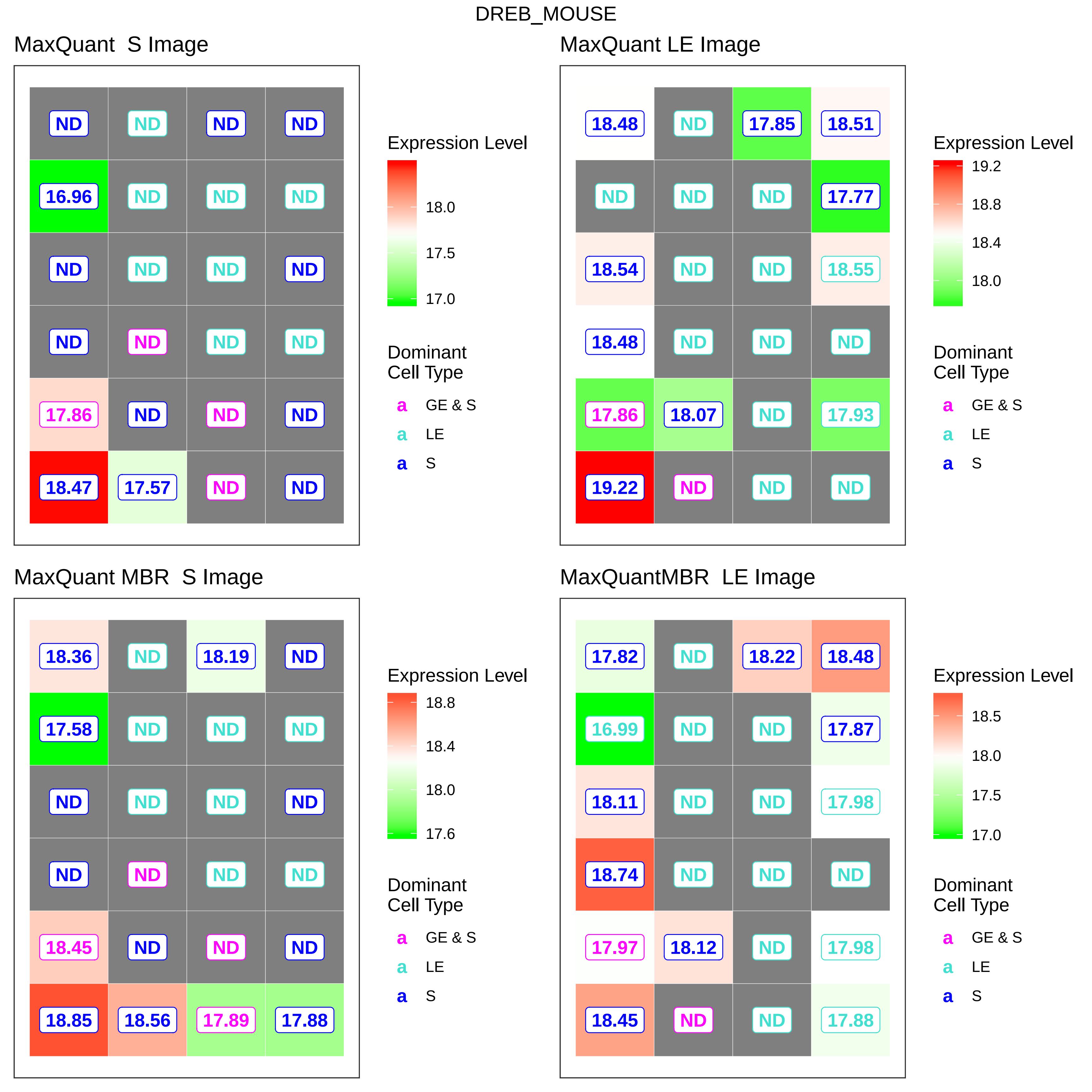

DREB_MOUSE
MaxQuant S Image
ND ND ND ND
16.96 ND ND ND
ND ND ND ND
ND ND ND ND
17.86 ND ND ND
18.47 17.57 ND ND
Expression Level
18.0
17.5
17.0
Dominant Cell Type
a GE & S
a LE
a S
MaxQuant LE Image
18.48 ND 17.85 18.51
ND ND ND 17.77
18.54 ND ND 18.55
18.48 ND ND ND
17.86 18.07 ND 17.93
19.22 ND ND ND
Expression Level
19.2
18.8
18.4
18.0
Dominant Cell Type
a GE & S
a LE
a S
MaxQuant MBR S Image
18.36 ND 18.19 ND
17.58 ND ND ND
ND ND ND ND
ND ND ND ND
18.45 ND ND ND
18.85 18.56 17.89 17.88
Expression Level
18.8
18.4
18.0
17.6
Dominant Cell Type
a GE & S
a LE
a S
MaxQuantMBR LE Image
17.82 ND 18.22 18.48
16.99 ND ND 17.87
18.11 ND ND 17.98
18.74 ND ND ND
17.97 18.12 ND 17.98
18.45 ND ND 17.88
Expression Level
18.5
18.0
17.5
17.0
Dominant Cell Type
a GE & S
a LE
a S

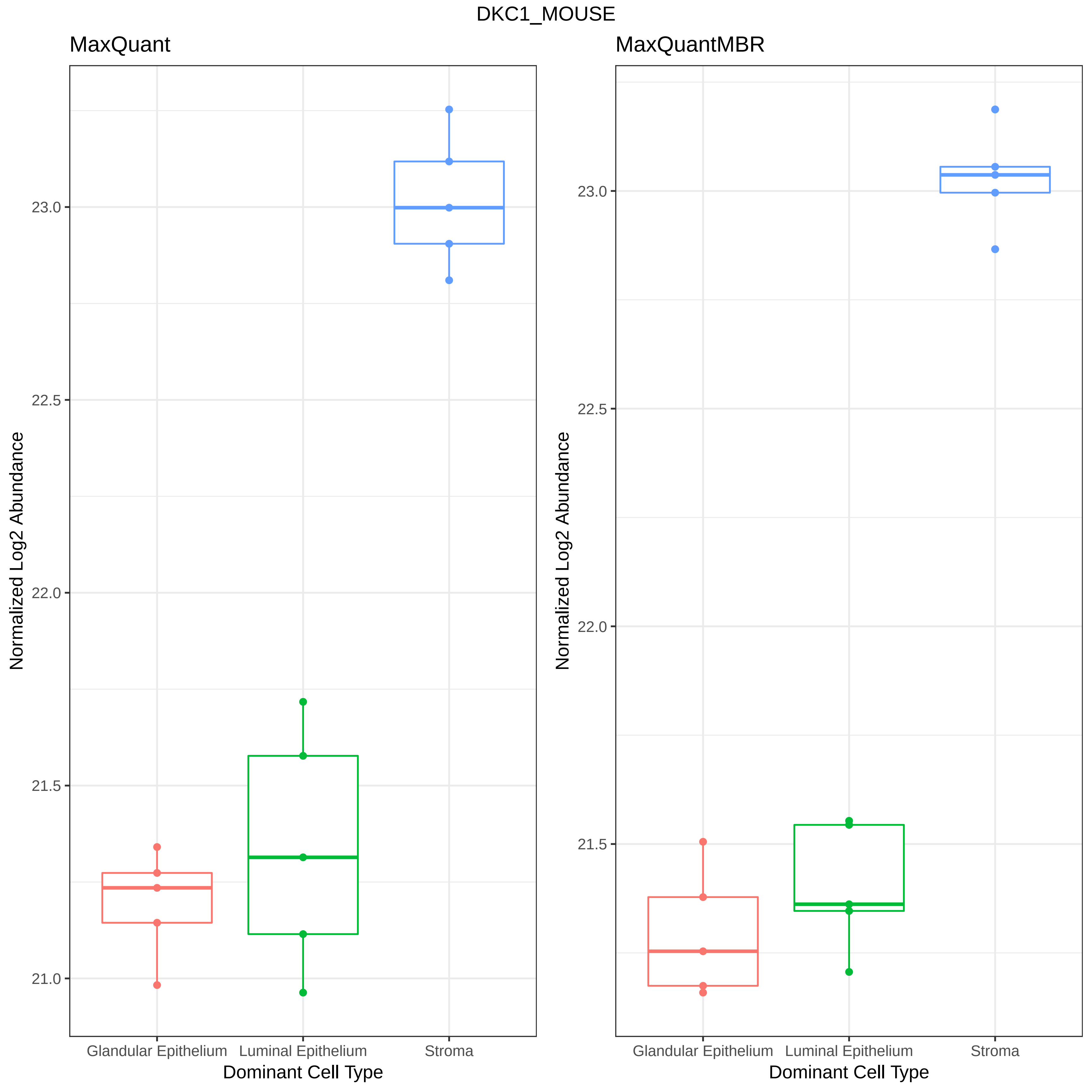

DKC1_MOUSE
MaxQuant
MaxQuantMBR
Normalized Log2 Abundance
Dominant Cell Type
Glandular Epithelium
Luminal Epithelium
Stroma
21.0
21.5
22.0
22.5
23.0

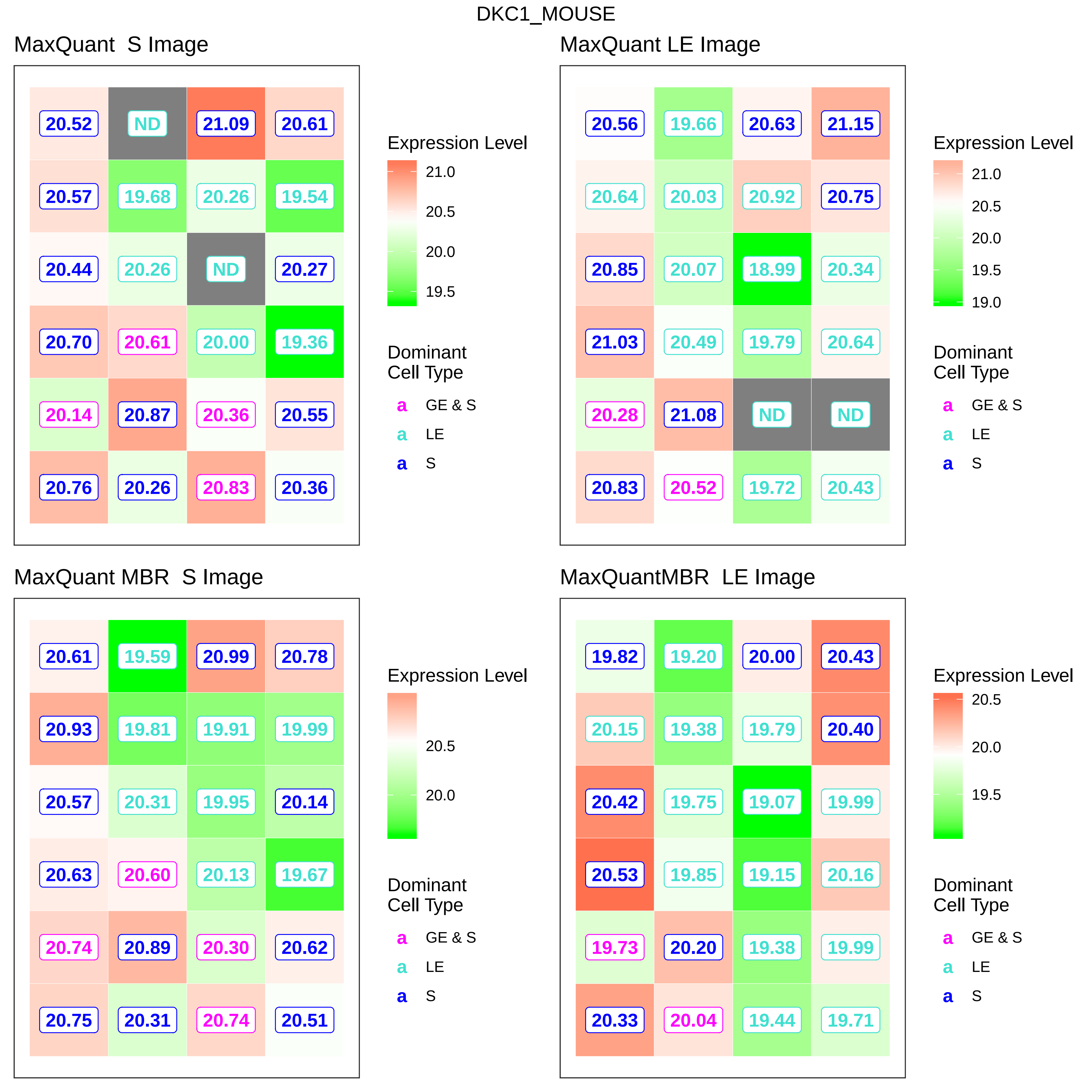
DKC1_MOUSE
MaxQuant S Image
20.52 ND 21.09 20.61
20.57 19.68 20.26 19.54
20.44 20.26 ND 20.27
20.70 20.61 20.00 19.36
20.14 20.87 20.36 20.55
20.76 20.26 20.83 20.36
Expression Level
21.0
20.5
20.0
19.5
Dominant Cell Type
a GE & S
a LE
a S
MaxQuant LE Image
20.56 19.66 20.63 21.15
20.64 20.03 20.92 20.75
20.85 20.07 18.99 20.34
21.03 20.49 19.79 20.64
20.28 21.08 ND ND
20.83 20.52 19.72 20.43
Expression Level
21.0
20.5
20.0
19.5
19.0
Dominant Cell Type
a GE & S
a LE
a S
MaxQuant MBR S Image
20.61 19.59 20.99 20.78
20.93 19.81 19.91 19.99
20.57 20.31 19.95 20.14
20.63 20.60 20.13 19.67
20.74 20.89 20.30 20.62
20.75 20.31 20.74 20.51
Expression Level
20.5
20.0
Dominant Cell Type
a GE & S
a LE
a S
MaxQuantMBR LE Image
19.82 19.20 20.00 20.43
20.15 19.38 19.79 20.40
20.42 19.75 19.07 19.99
20.53 19.85 19.15 20.16
19.73 20.20 19.38 19.99
20.33 20.04 19.44 19.71
Expression Level
20.5
20.0
19.5
Dominant Cell Type
a GE & S
a LE
a S

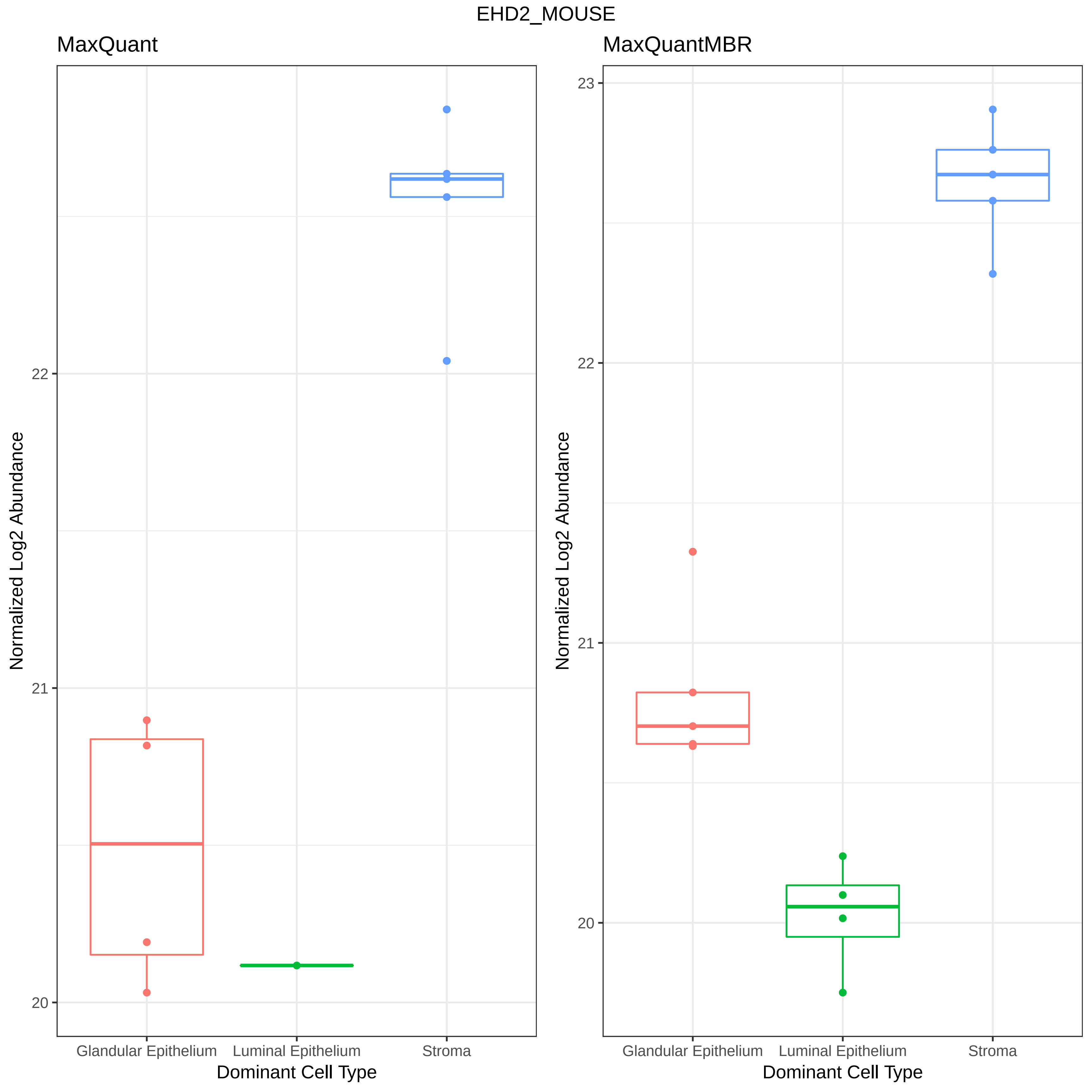
EHD2_MOUSE
MaxQuant
MaxQuantMBR
Normalized Log2 Abundance
Dominant Cell Type
Glandular Epithelium
Luminal Epithelium
Stroma
20
21
22
23

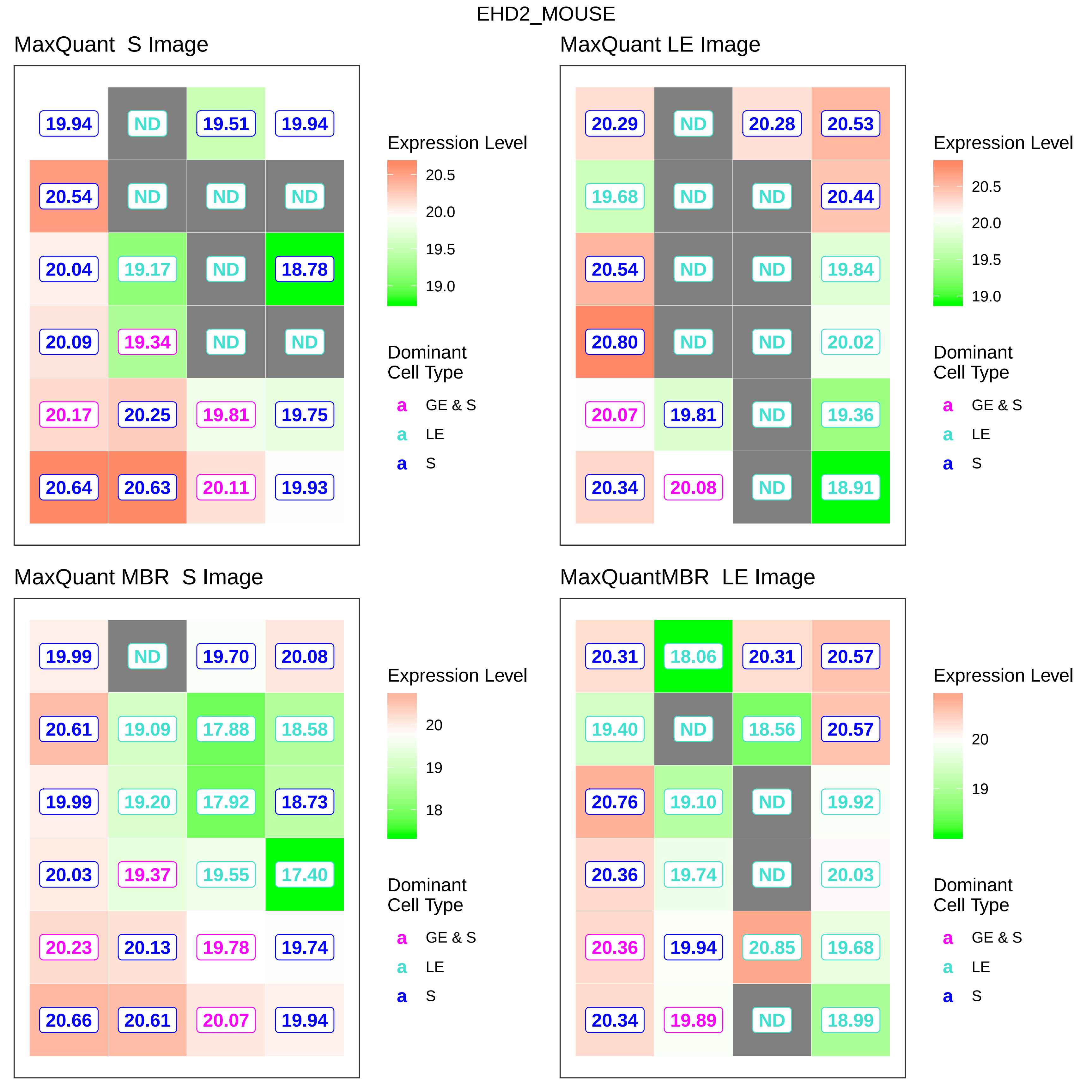

EHD2_MOUSE
MaxQuant S Image
19.94 ND 19.51 19.94
20.54 ND ND ND
20.04 19.17 ND 18.78
20.09 19.34 ND ND
20.17 20.25 19.81 19.75
20.64 20.63 20.11 19.93
Expression Level
20.5
20.0
19.5
19.0
Dominant Cell Type
a GE & S
a LE
a S
MaxQuant LE Image
20.29 ND 20.28 20.53
19.68 ND ND 20.44
20.54 ND ND 19.84
20.80 ND ND 20.02
20.07 19.81 ND 19.36
20.34 20.08 ND 18.91
Expression Level
20.5
20.0
19.5
19.0
Dominant Cell Type
a GE & S
a LE
a S
MaxQuant MBR S Image
19.99 ND 19.70 20.08
20.61 19.09 17.88 18.58
19.99 19.20 17.92 18.73
20.03 19.37 19.55 17.40
20.23 20.13 19.78 19.74
20.66 20.61 20.07 19.94
Expression Level
20
19
18
Dominant Cell Type
a GE & S
a LE
a S
MaxQuantMBR LE Image
20.31 18.06 20.31 20.57
19.40 ND 18.56 20.57
20.76 19.10 ND 19.92
20.36 19.74 ND 20.03
20.36 19.94 20.85 19.68
20.34 19.89 ND 18.99
Expression Level
20
19
Dominant Cell Type
a GE & S
a LE
a S

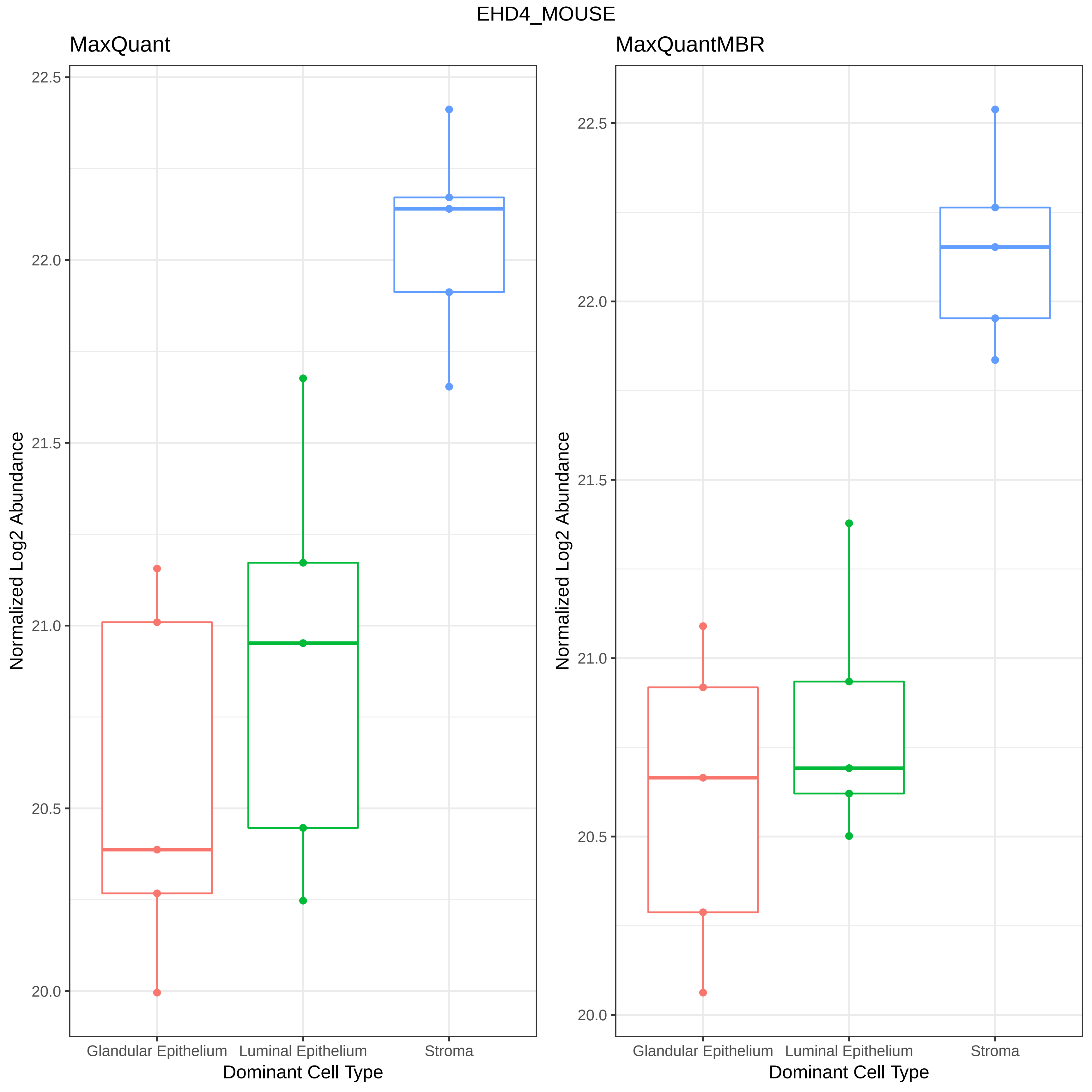
EHD4_MOUSE
MaxQuant
MaxQuantMBR
Normalized Log2 Abundance
22.5
22.0
21.5
21.0
20.5
20.0
Glandular Epithelium
Luminal Epithelium
Stroma
Dominant Cell Type

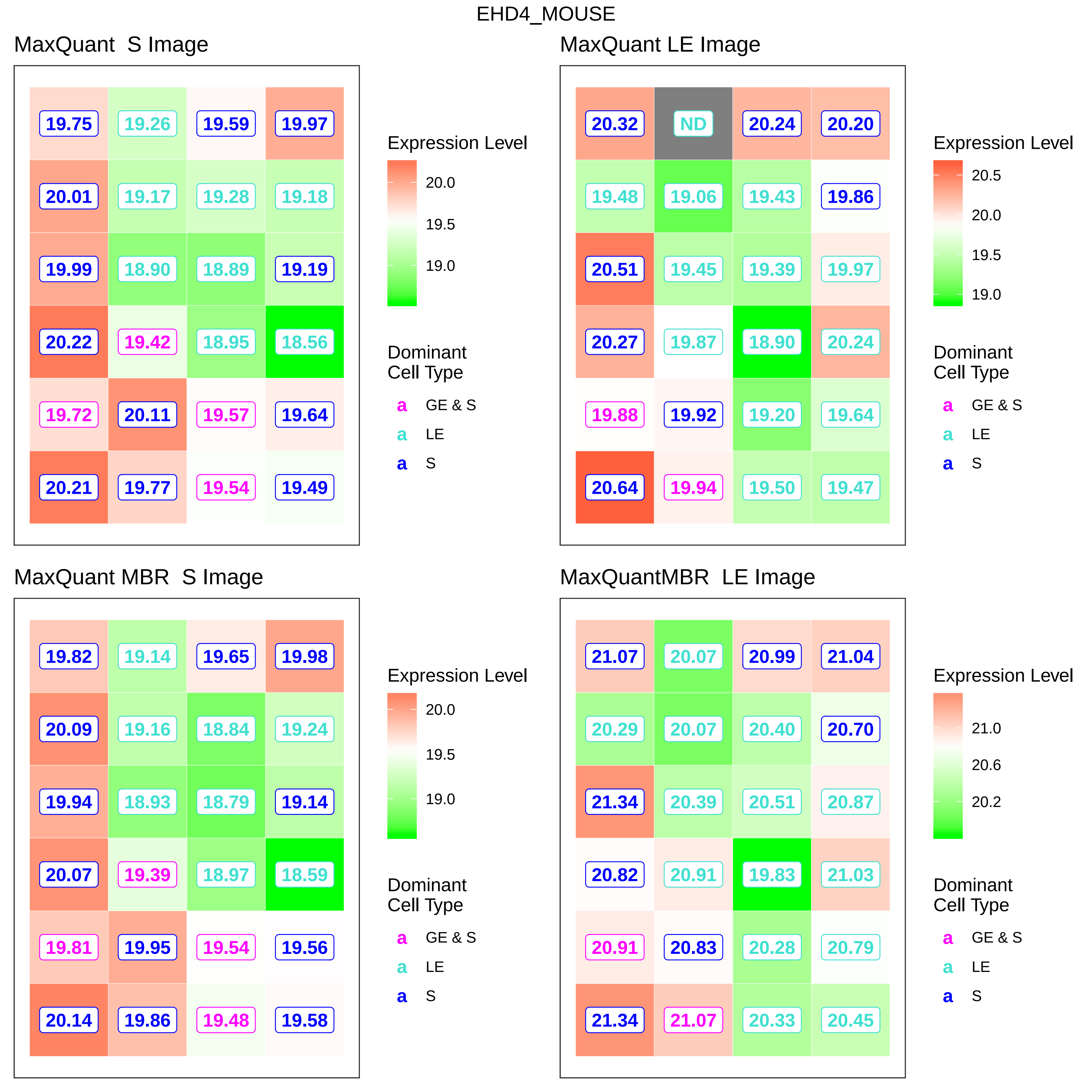
EHD4_MOUSE
MaxQuant S Image
19.75 19.26 19.59 19.97
20.01 19.17 19.28 19.18
19.99 18.90 18.89 19.19
20.22 19.42 18.95 18.56
19.72 20.11 19.57 19.64
20.21 19.77 19.54 19.49
Expression Level
20.0
19.5
19.0
Dominant Cell Type
GE & S
LE
S
MaxQuant LE Image
20.32 ND 20.24 20.20
19.48 19.06 19.43 19.86
20.51 19.45 19.39 19.97
20.27 19.87 18.90 20.24
19.88 19.92 19.20 19.64
20.64 19.94 19.50 19.47
Expression Level
20.5
20.0
19.5
19.0
Dominant Cell Type
GE & S
LE
S
MaxQuant MBR S Image
19.82 19.14 19.65 19.98
20.09 19.16 18.84 19.24
19.94 18.93 18.79 19.14
20.07 19.39 18.97 18.59
19.81 19.95 19.54 19.56
20.14 19.86 19.48 19.58
Expression Level
20.0
19.5
19.0
Dominant Cell Type
GE & S
LE
S
MaxQuantMBR LE Image
21.07 20.07 20.99 21.04
20.29 20.07 20.40 20.70
21.34 20.39 20.51 20.87
20.82 20.91 19.83 21.03
20.91 20.83 20.28 20.79
21.34 21.07 20.33 20.45
Expression Level
21.0
20.6
20.2
Dominant Cell Type
GE & S
LE
S

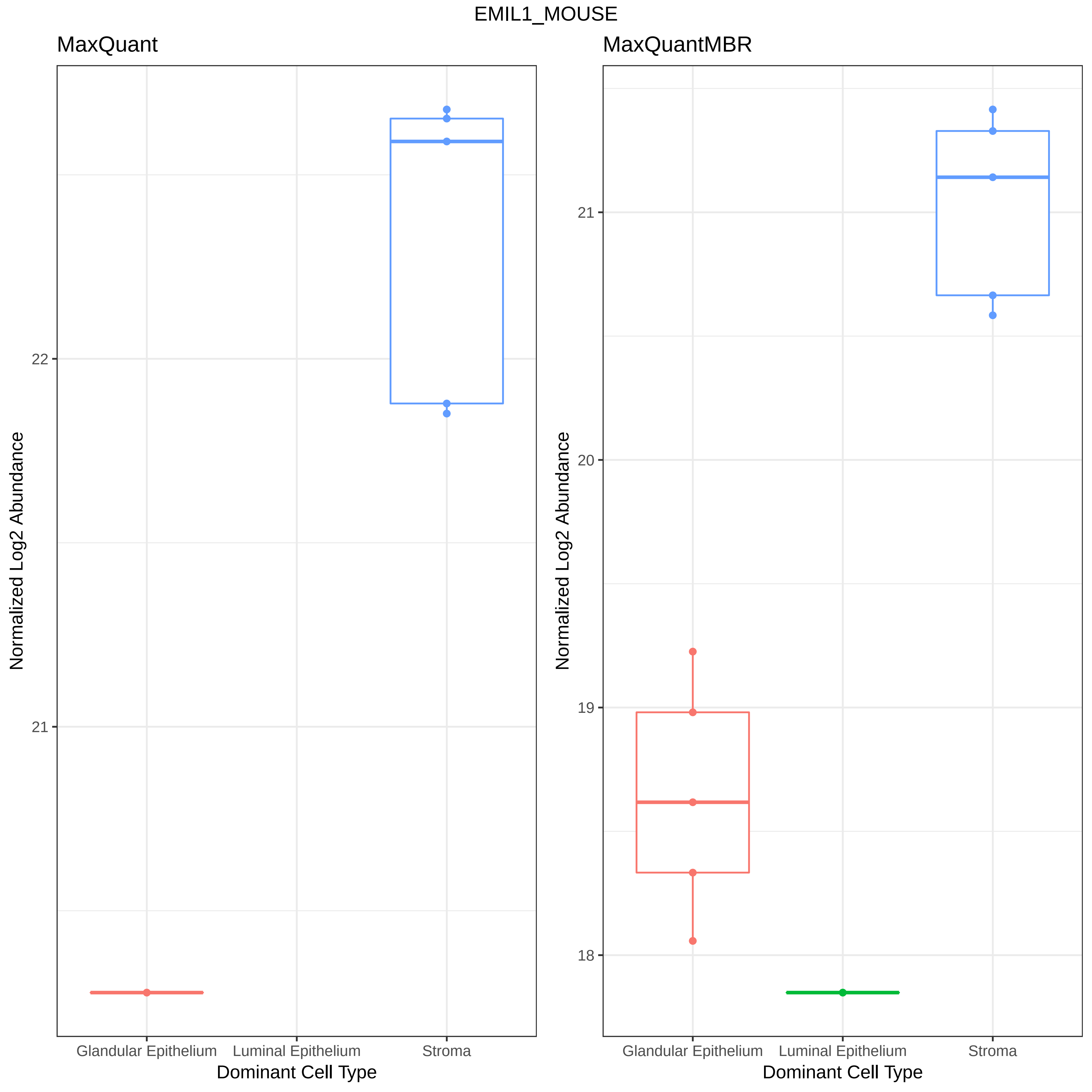
EMIL1_MOUSE
MaxQuant
MaxQuantMBR
Normalized Log2 Abundance
22
21
21
20
19
18
Glandular Epithelium
Luminal Epithelium
Stroma
Dominant Cell Type

EMIL1_MOUSE

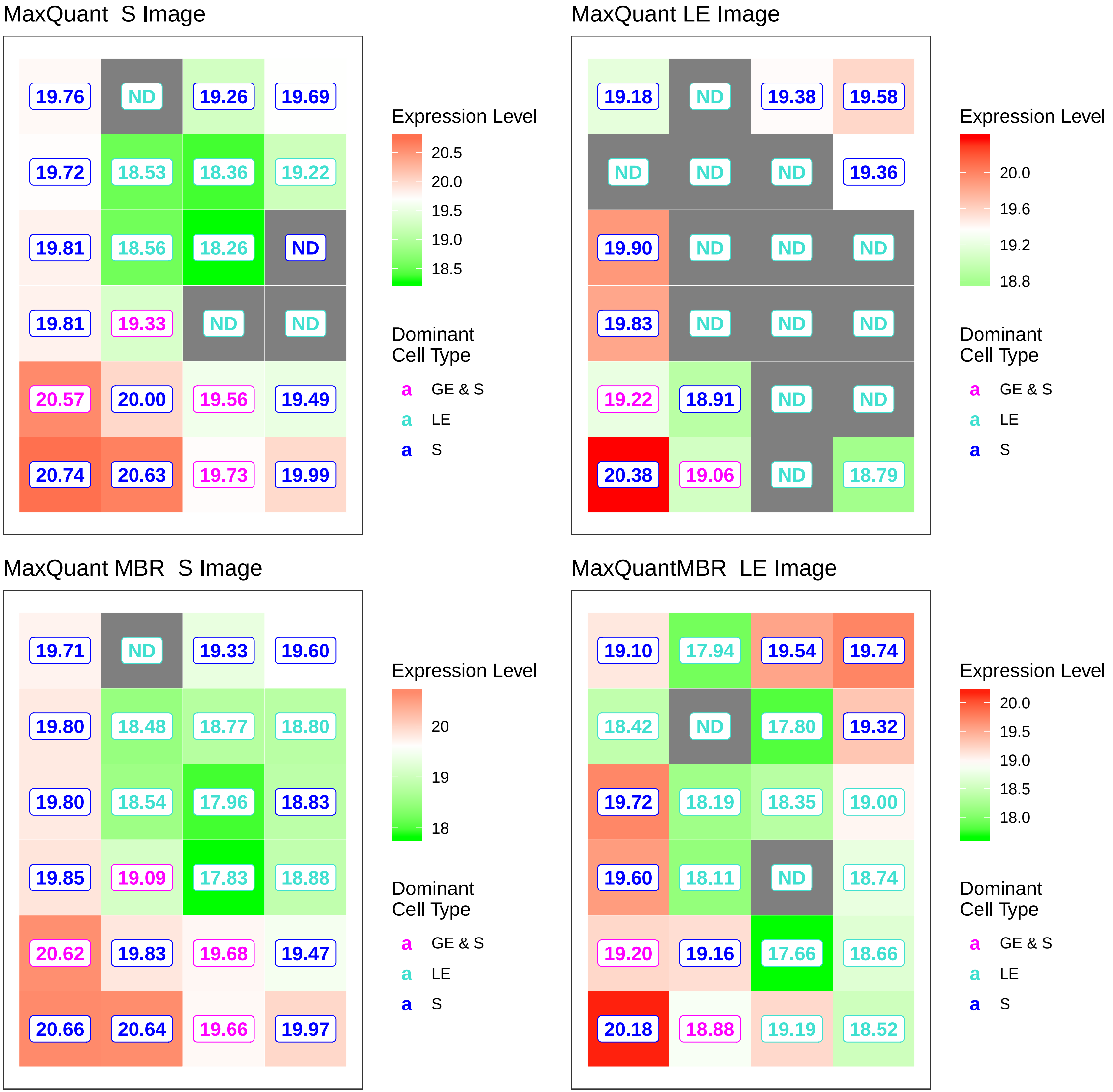
MaxQuant S Image
19.76 ND 19.26 19.69
19.72 18.53 18.36 19.22
19.81 18.56 18.26 ND
19.81 19.33 ND ND
20.57 20.00 19.56 19.49
20.74 20.63 19.73 19.99
Expression Level
20.5
20.0
19.5
19.0
18.5
Dominant Cell Type
a GE & S
a LE
a S
MaxQuant LE Image
19.18 ND 19.38 19.58
ND ND ND 19.36
19.90 ND ND ND
19.83 ND ND ND
19.22 18.91 ND ND
20.38 19.06 ND 18.79
Expression Level
20.0
19.6
19.2
18.8
Dominant Cell Type
a GE & S
a LE
a S
MaxQuant MBR S Image
19.71 ND 19.33 19.60
19.80 18.48 18.77 18.80
19.80 18.54 17.96 18.83
19.85 19.09 17.83 18.88
20.62 19.83 19.68 19.47
20.66 20.64 19.66 19.97
Expression Level
20
19
18
Dominant Cell Type
a GE & S
a LE
a S
MaxQuantMBR LE Image
19.10 17.94 19.54 19.74
18.42 ND 17.80 19.32
19.72 18.19 18.35 19.00
19.60 18.11 ND 18.74
19.20 19.16 17.66 18.66
20.18 18.88 19.19 18.52
Expression Level
20.0
19.5
19.0
18.5
18.0
Dominant Cell Type
a GE & S
a LE
a S

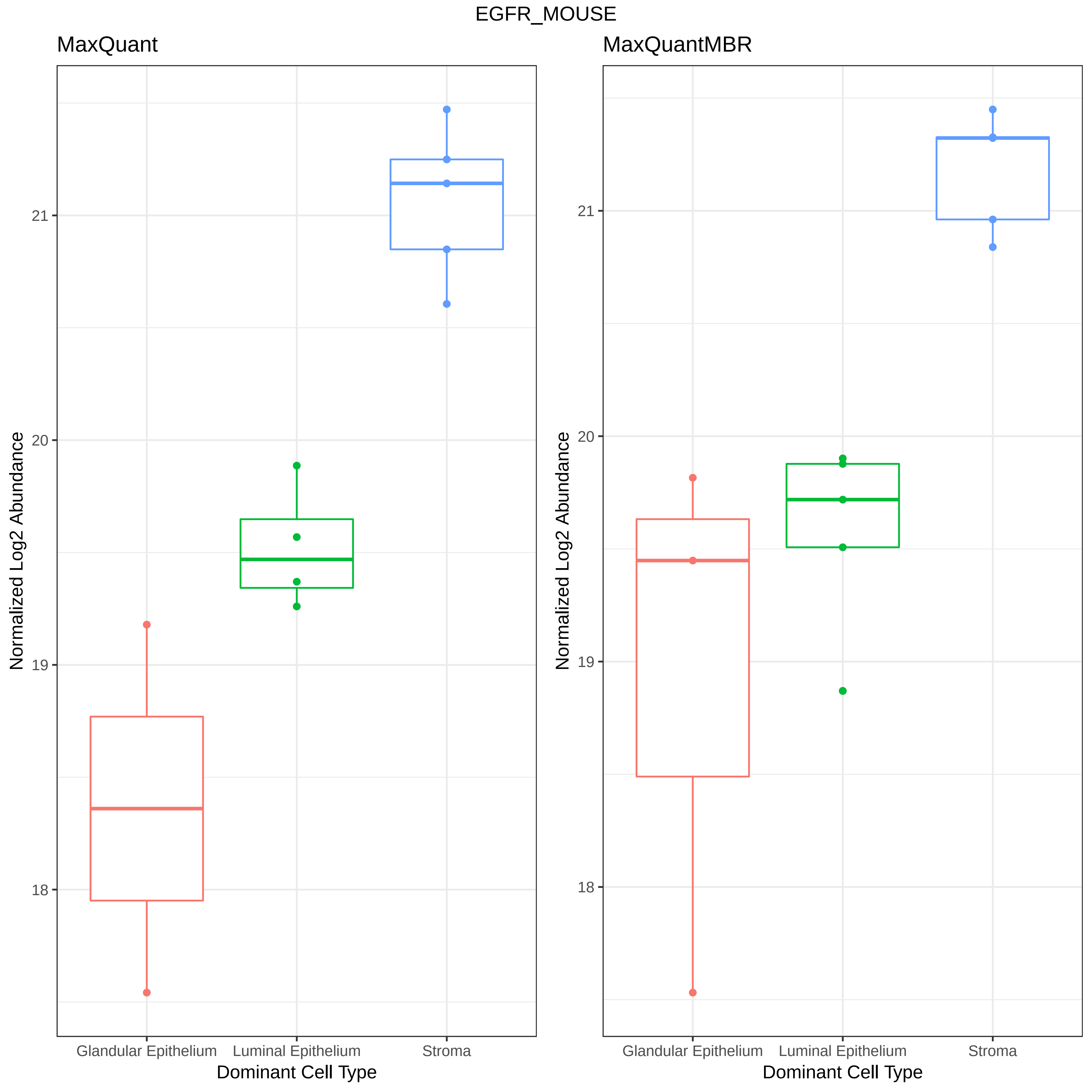
EGFR_MOUSE
MaxQuant
MaxQuantMBR
Normalized Log2 Abundance
21
20
19
18
Glandular Epithelium
Luminal Epithelium
Stroma
Dominant Cell Type

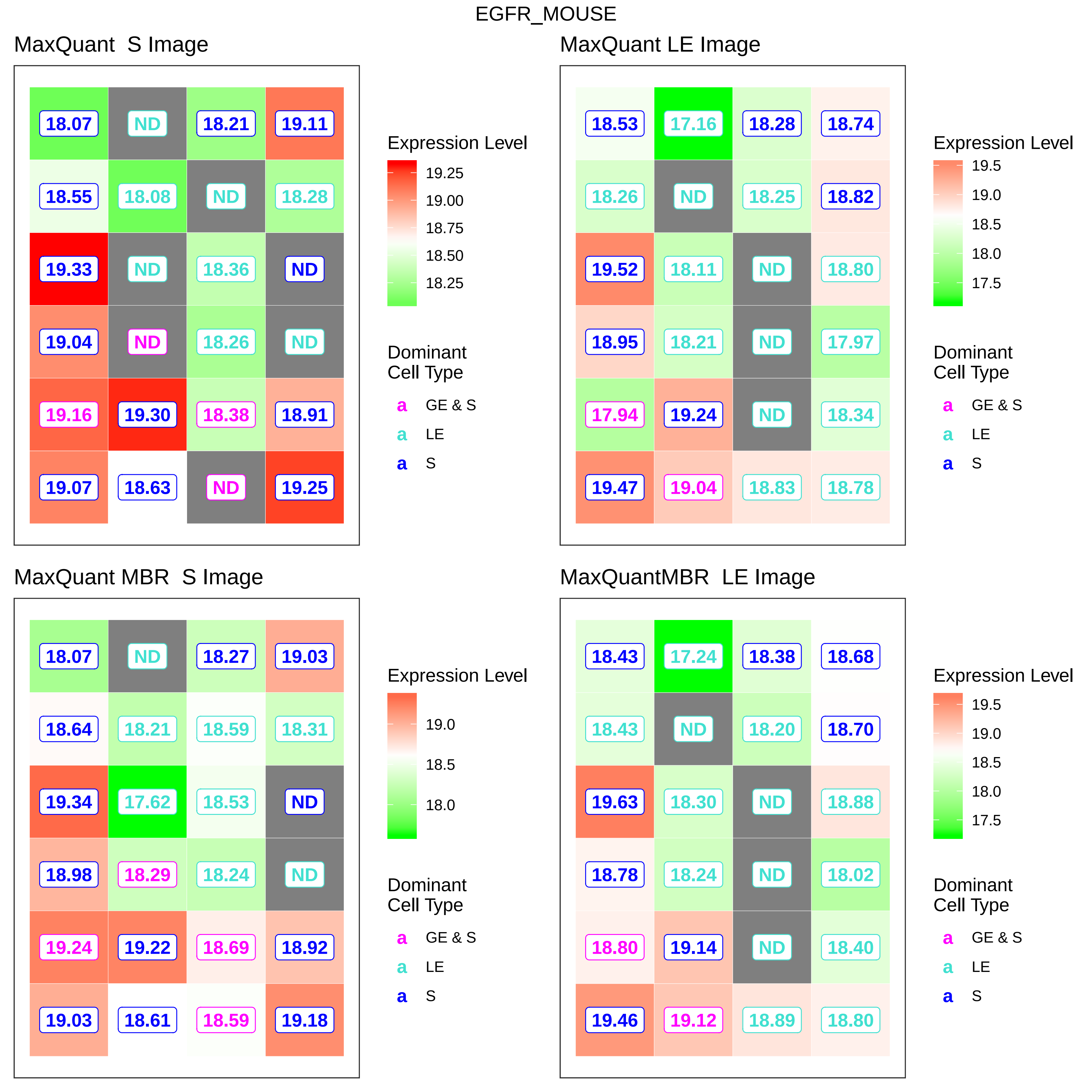
EGFR_MOUSE
MaxQuant S Image
18.07 ND 18.21 19.11
18.55 18.08 ND 18.28
19.33 ND 18.36 ND
19.04 ND 18.26 ND
19.16 19.30 18.38 18.91
19.07 18.63 ND 19.25
Expression Level
19.25
19.00
18.75
18.50
18.25
Dominant Cell Type
a GE & S
a LE
a S
MaxQuant LE Image
18.53 17.16 18.28 18.74
18.26 ND 18.25 18.82
19.52 18.11 ND 18.80
18.95 18.21 ND 17.97
17.94 19.24 ND 18.34
19.47 19.04 18.83 18.78
Expression Level
19.5
19.0
18.5
18.0
17.5
Dominant Cell Type
a GE & S
a LE
a S
MaxQuant MBR S Image
18.07 ND 18.27 19.03
18.64 18.21 18.59 18.31
19.34 17.62 18.53 ND
18.98 18.29 18.24 ND
19.24 19.22 18.69 18.92
19.03 18.61 18.59 19.18
Expression Level
19.0
18.5
18.0
Dominant Cell Type
a GE & S
a LE
a S
MaxQuantMBR LE Image
18.43 17.24 18.38 18.68
18.43 ND 18.20 18.70
19.63 18.30 ND 18.88
18.78 18.24 ND 18.02
18.80 19.14 ND 18.40
19.46 19.12 18.89 18.80
Expression Level
19.5
19.0
18.5
18.0
17.5
Dominant Cell Type
a GE & S
a LE
a S

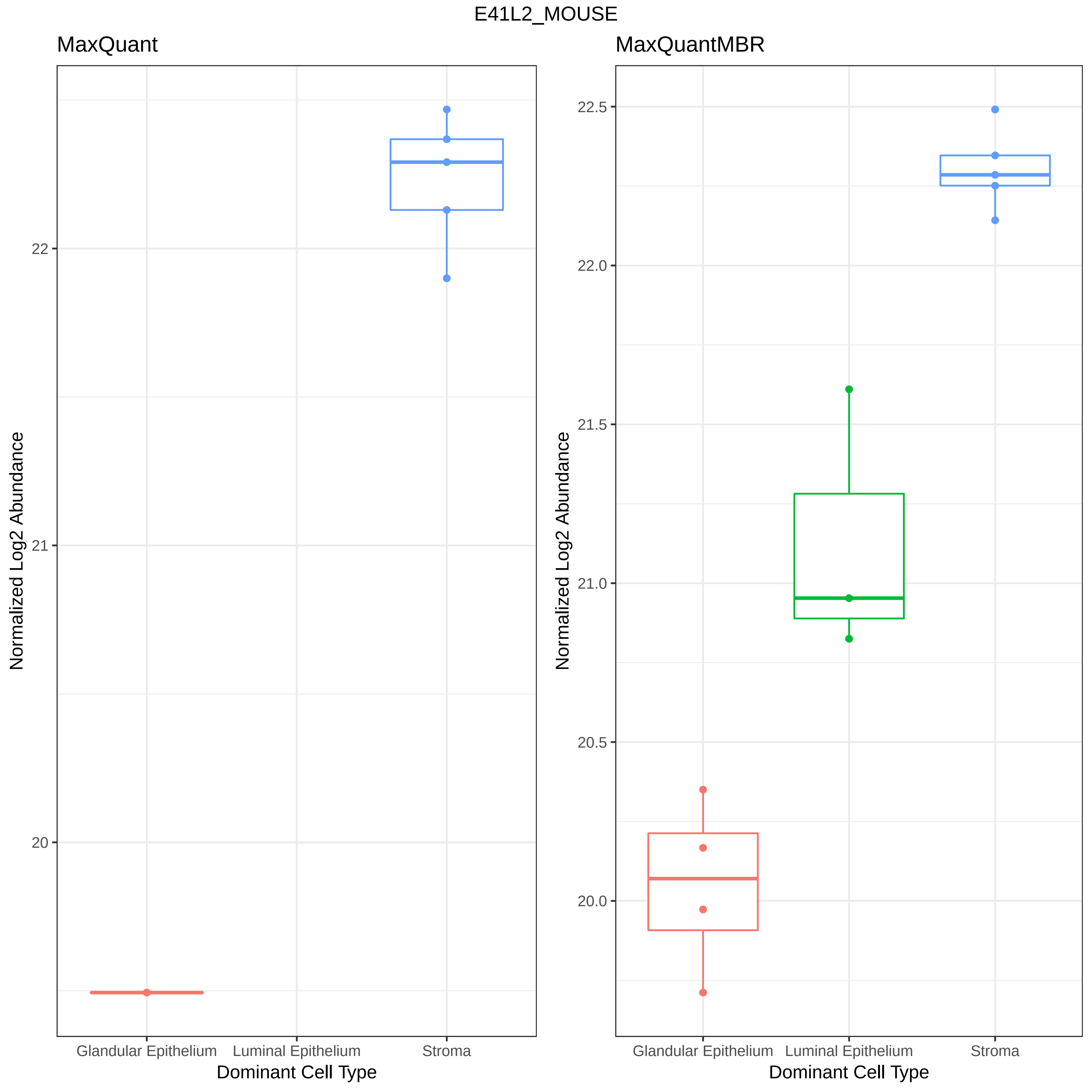

E41L2_MOUSE
MaxQuant
MaxQuantMBR
Normalized Log2 Abundance
22
21
20
22.5
22.0
21.5
21.0
20.5
20.0
Glandular Epithelium
Luminal Epithelium
Stroma
Dominant Cell Type

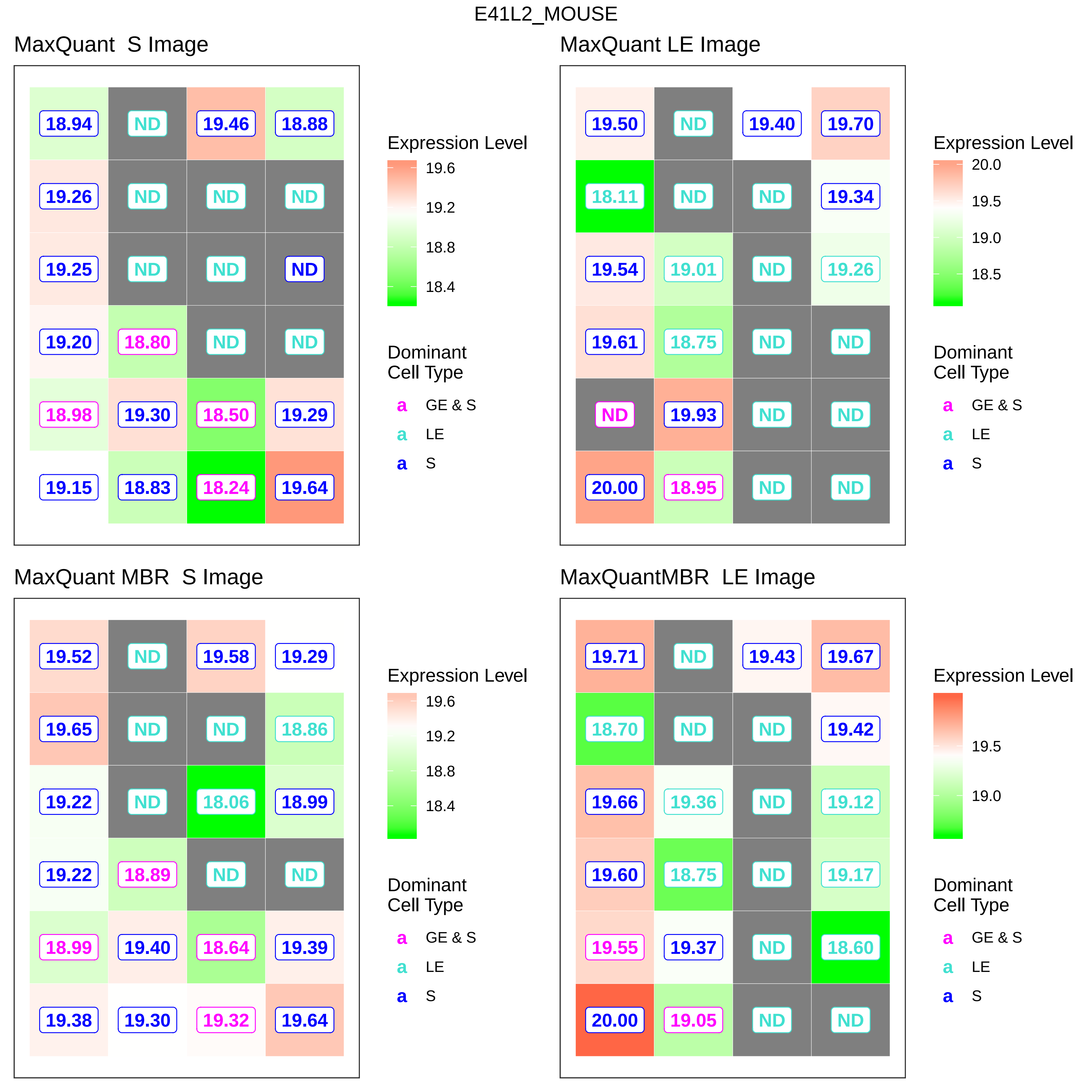
E41L2_MOUSE
MaxQuant S Image
18.94
ND
19.46
18.88
19.26
ND
ND
ND
19.25
ND
ND
ND
19.20
18.80
ND
ND
18.98
19.30
18.50
19.29
19.15
18.83
18.24
19.64
Expression Level
19.6
19.2
18.8
18.4
Dominant Cell Type
a GE & S
a LE
a S
MaxQuant LE Image
19.50
ND
19.40
19.70
18.11
ND
ND
19.34
19.54
19.01
ND
19.26
19.61
18.75
ND
ND
ND
19.93
ND
ND
20.00
18.95
ND
ND
Expression Level
20.0
19.5
19.0
18.5
Dominant Cell Type
a GE & S
a LE
a S
MaxQuant MBR S Image
19.52
ND
19.58
19.29
19.65
ND
ND
18.86
19.22
ND
18.06
18.99
19.22
18.89
ND
ND
18.99
19.40
18.64
19.39
19.38
19.30
19.32
19.64
Expression Level
19.6
19.2
18.8
18.4
Dominant Cell Type
a GE & S
a LE
a S
MaxQuantMBR LE Image
19.71
ND
19.43
19.67
18.70
ND
ND
19.42
19.66
19.36
ND
19.12
19.60
18.75
ND
19.17
19.55
19.37
ND
18.60
20.00
19.05
ND
ND
Expression Level
19.5
19.0
Dominant Cell Type
a GE & S
a LE
a S

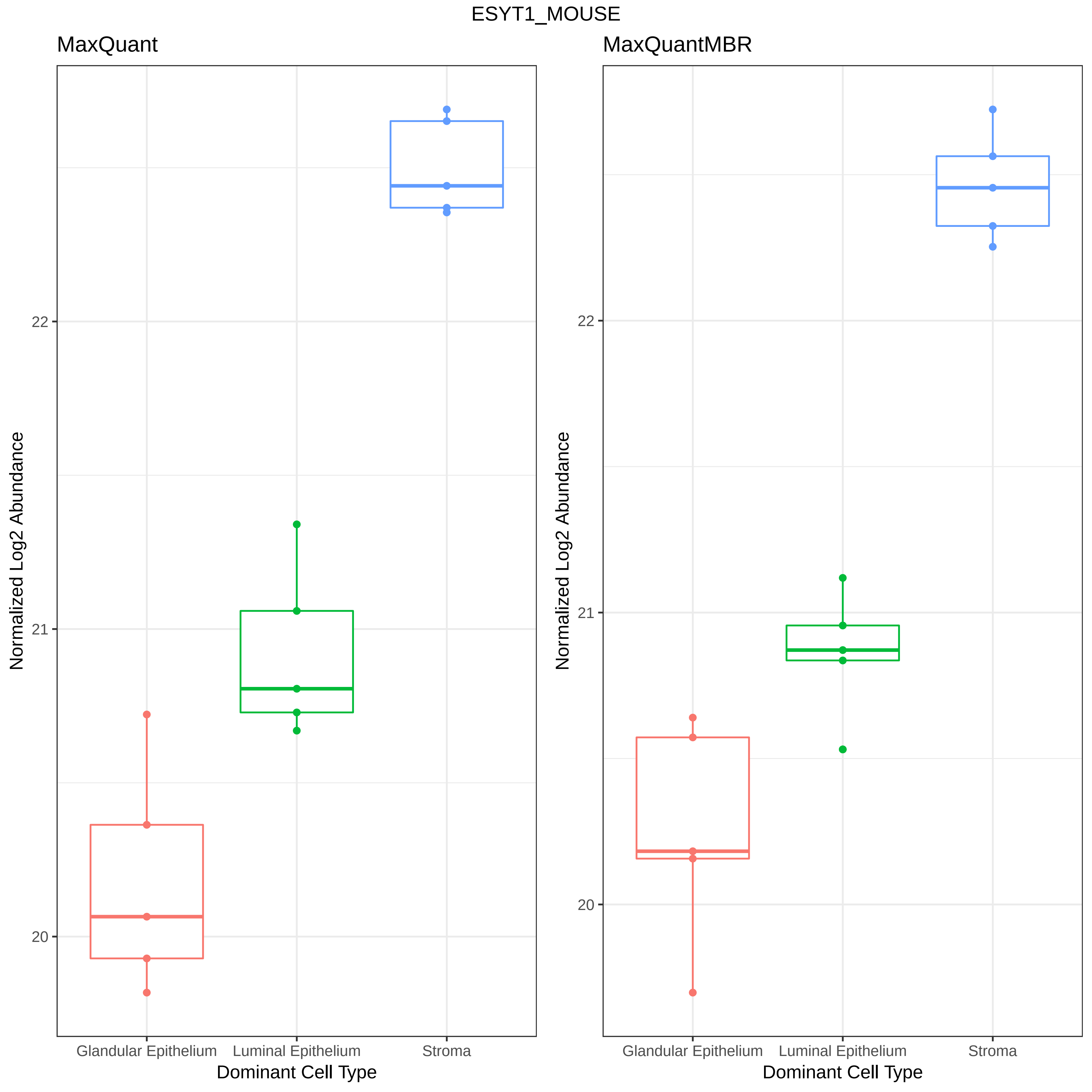
ESYT1_MOUSE
MaxQuant
MaxQuantMBR
Normalized Log2 Abundance
22
21
20
Glandular Epithelium
Luminal Epithelium
Stroma
Dominant Cell Type

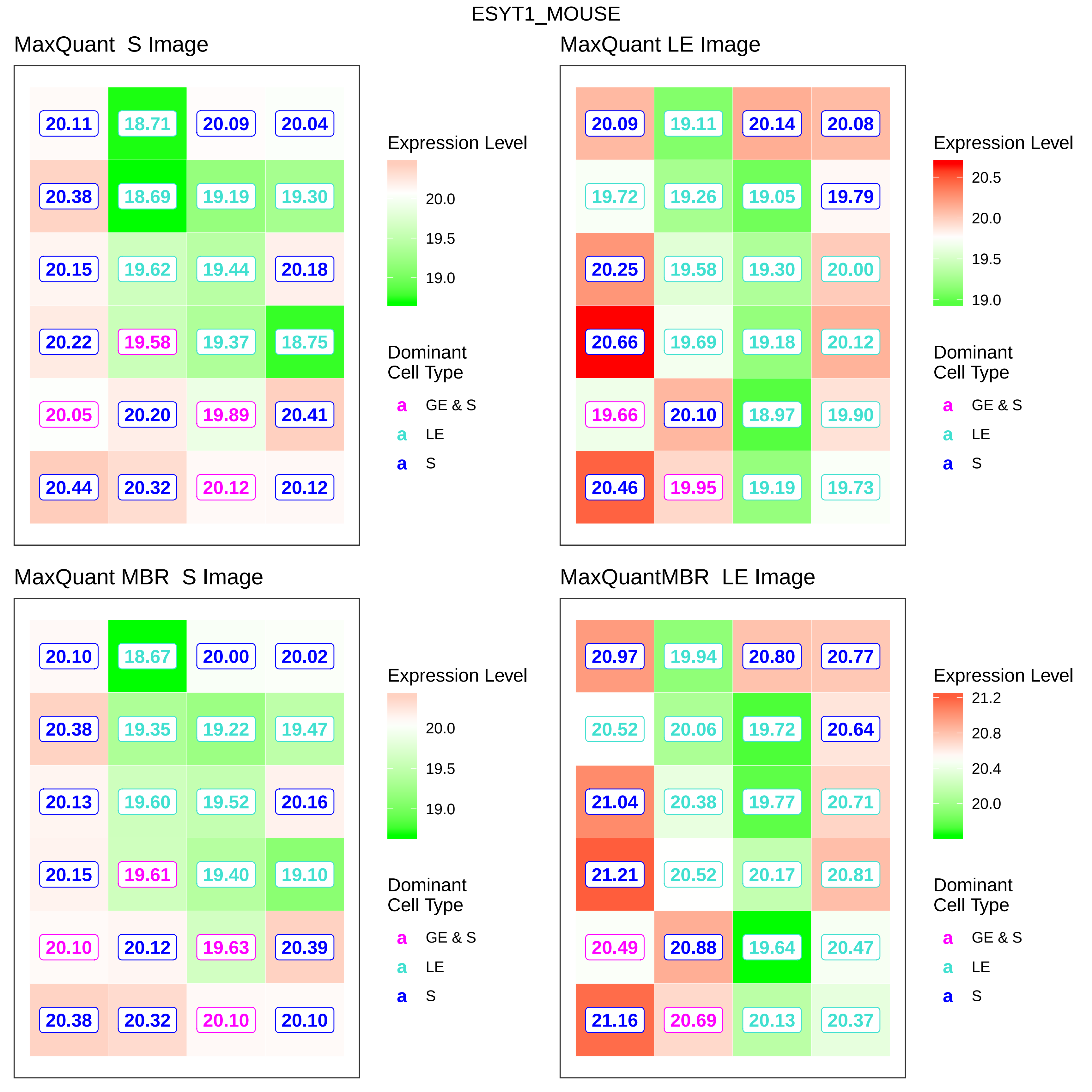

ESYT1_MOUSE
MaxQuant S Image
20.11 18.71 20.09 20.04
20.38 18.69 19.19 19.30
20.15 19.62 19.44 20.18
20.22 19.58 19.37 18.75
20.05 20.20 19.89 20.41
20.44 20.32 20.12 20.12
Expression Level
20.0
19.5
19.0
Dominant Cell Type
a GE & S
a LE
a S
MaxQuant LE Image
20.09 19.11 20.14 20.08
19.72 19.26 19.05 19.79
20.25 19.58 19.30 20.00
20.66 19.69 19.18 20.12
19.66 20.10 18.97 19.90
20.46 19.95 19.19 19.73
Expression Level
20.5
20.0
19.5
19.0
Dominant Cell Type
a GE & S
a LE
a S
MaxQuant MBR S Image
20.10 18.67 20.00 20.02
20.38 19.35 19.22 19.47
20.13 19.60 19.52 20.16
20.15 19.61 19.40 19.10
20.10 20.12 19.63 20.39
20.38 20.32 20.10 20.10
Expression Level
20.0
19.5
19.0
Dominant Cell Type
a GE & S
a LE
a S
MaxQuantMBR LE Image
20.97 19.94 20.80 20.77
20.52 20.06 19.72 20.64
21.04 20.38 19.77 20.71
21.21 20.52 20.17 20.81
20.49 20.88 19.64 20.47
21.16 20.69 20.13 20.37
Expression Level
21.2
20.8
20.4
20.0
Dominant Cell Type
a GE & S
a LE
a S

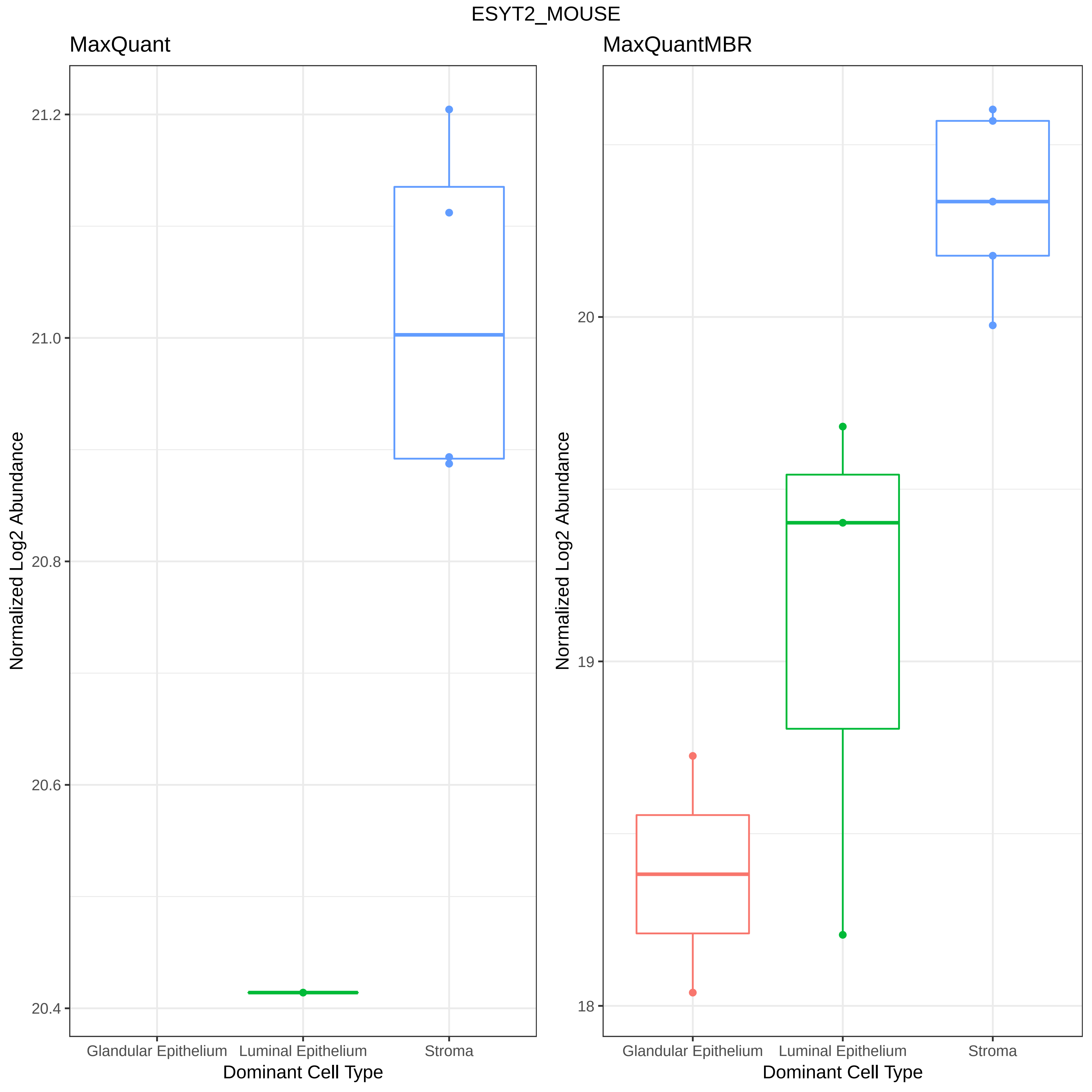
ESYT2_MOUSE
MaxQuant
MaxQuantMBR
Normalized Log2 Abundance
21.2
21.0
20.8
20.6
20.4
20
19
18
Glandular Epithelium
Luminal Epithelium
Stroma
Dominant Cell Type

ESYT2_MOUSE

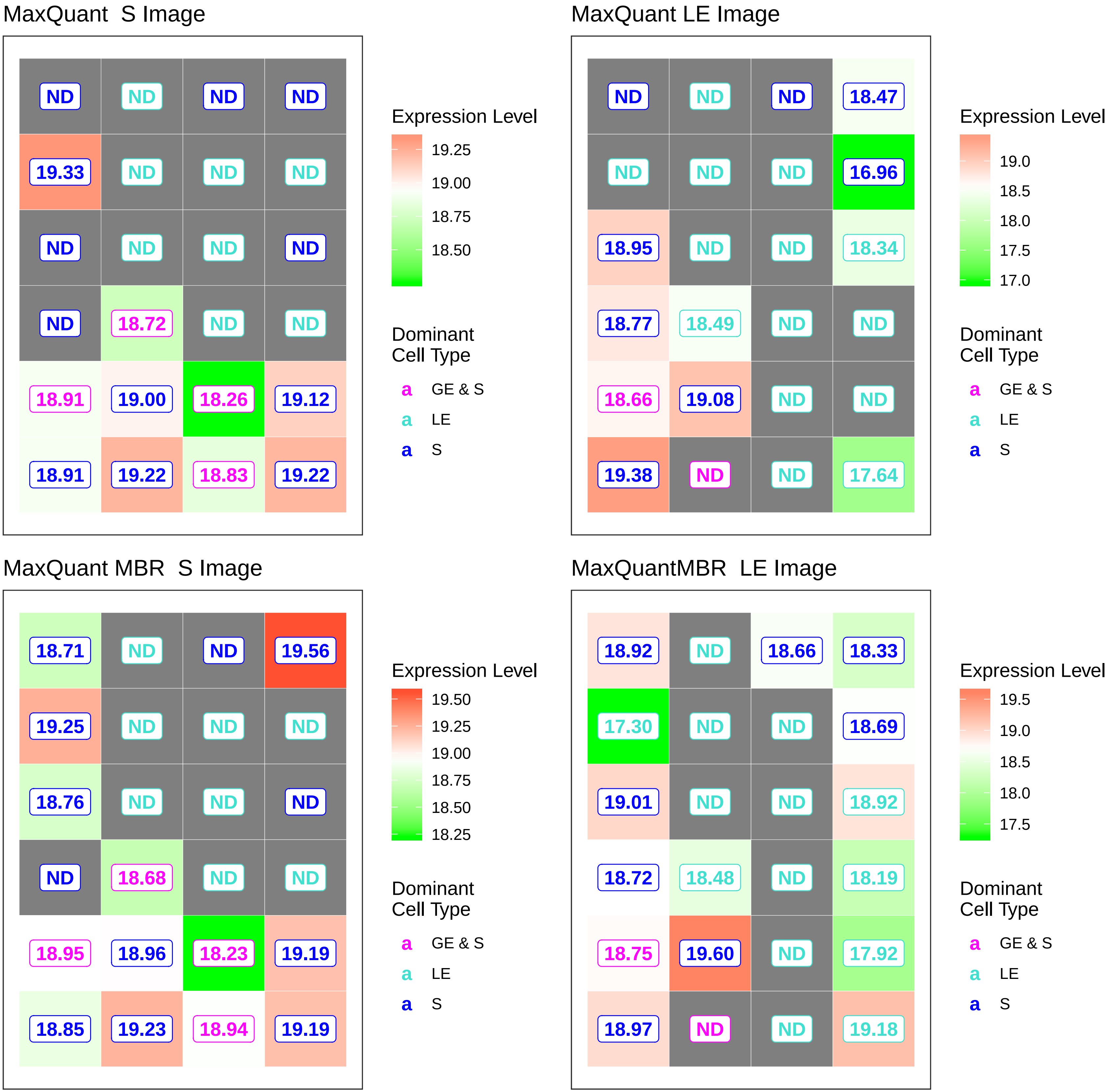
MaxQuant S Image
ND ND ND ND
19.33 ND ND ND
ND ND ND ND
ND 18.72 ND ND
18.91 19.00 18.26 19.12
18.91 19.22 18.83 19.22
Expression Level
19.25
19.00
18.75
18.50
Dominant Cell Type
a GE & S
a LE
a S
MaxQuant LE Image
ND ND ND 18.47
ND ND ND 16.96
18.95 ND ND 18.34
18.77 18.49 ND ND
18.66 19.08 ND ND
19.38 ND ND 17.64
Expression Level
19.0
18.5
18.0
17.5
17.0
Dominant Cell Type
a GE & S
a LE
a S
MaxQuant MBR S Image
18.71 ND ND 19.56
19.25 ND ND ND
18.76 ND ND ND
ND 18.68 ND ND
18.95 18.96 18.23 19.19
18.85 19.23 18.94 19.19
Expression Level
19.50
19.25
19.00
18.75
18.50
18.25
Dominant Cell Type
a GE & S
a LE
a S
MaxQuantMBR LE Image
18.92 ND 18.66 18.33
17.30 ND ND 18.69
19.01 ND ND 18.92
18.72 18.48 ND 18.19
18.75 19.60 ND 17.92
18.97 ND ND 19.18
Expression Level
19.5
19.0
18.5
18.0
17.5
Dominant Cell Type
a GE & S
a LE
a S

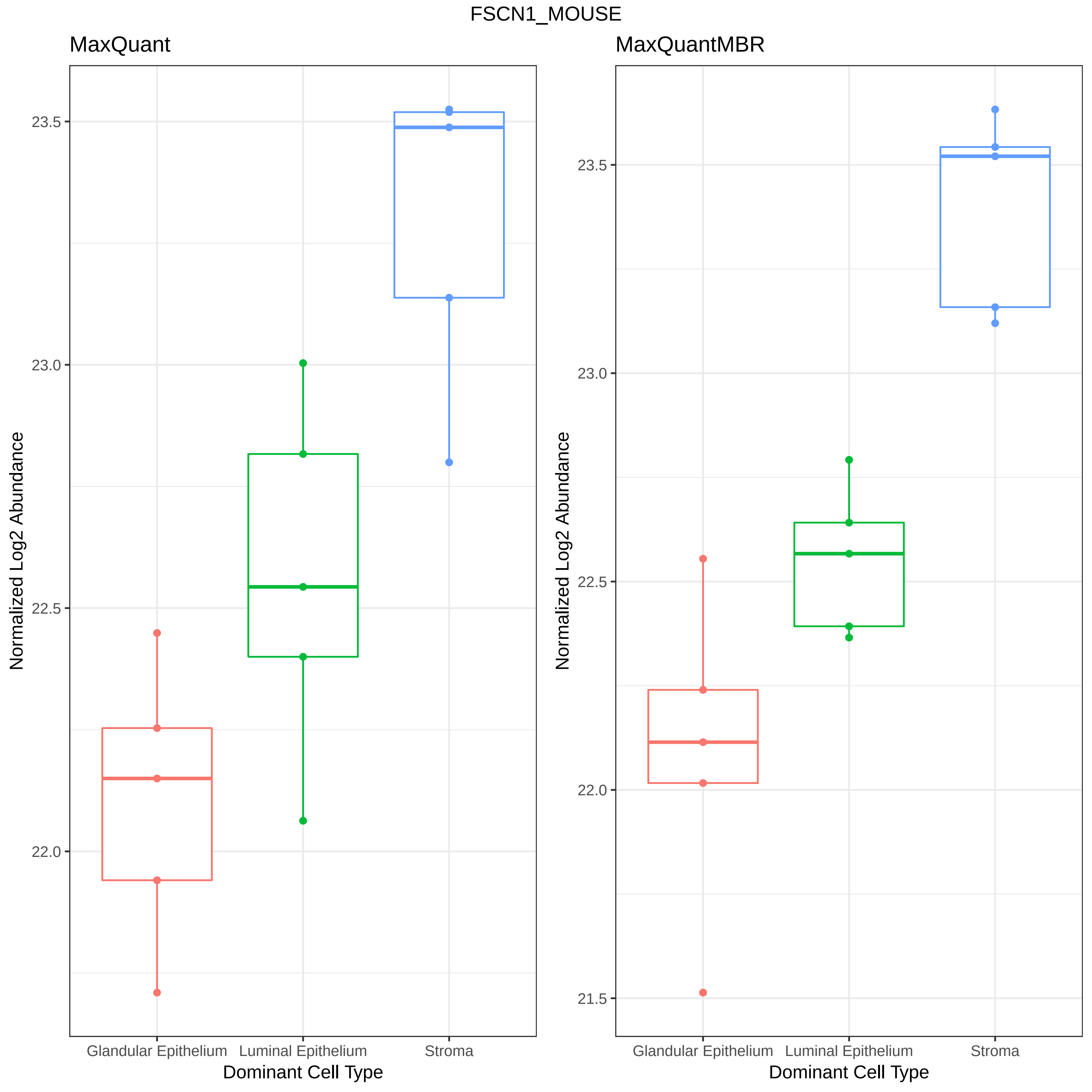
FSCN1_MOUSE
MaxQuant
MaxQuantMBR
Normalized Log2 Abundance
23.5
23.0
22.5
22.0
21.5
Glandular Epithelium
Luminal Epithelium
Stroma
Dominant Cell Type

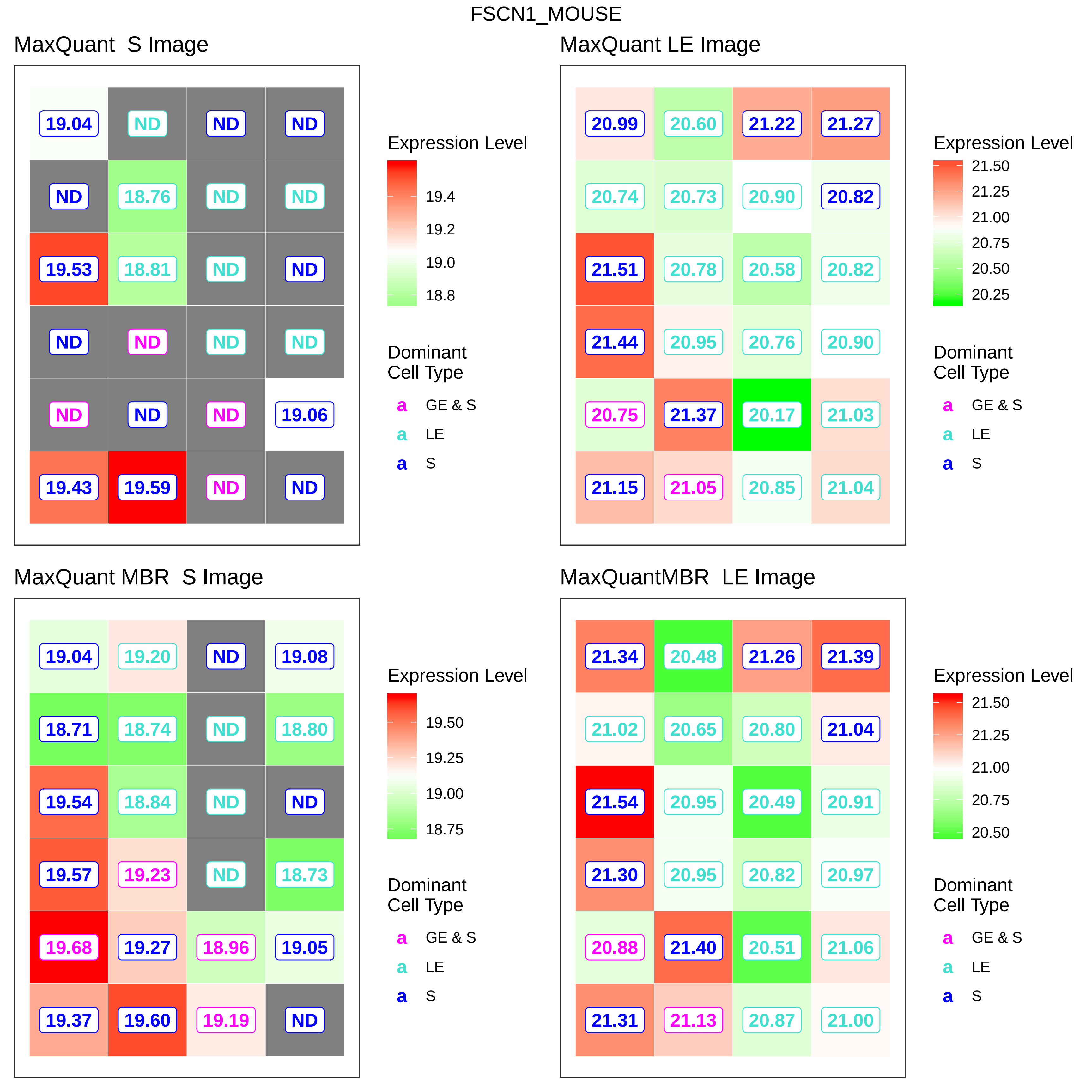
FSCN1_MOUSE
MaxQuant S Image
19.04 ND ND ND
ND 18.76 ND ND
19.53 18.81 ND ND
ND ND ND ND
ND ND ND 19.06
19.43 19.59 ND ND
Expression Level
19.4
19.2
19.0
18.8
Dominant Cell Type
a GE & S
a LE
a S
MaxQuant LE Image
20.99 20.60 21.22 21.27
20.74 20.73 20.90 20.82
21.51 20.78 20.58 20.82
21.44 20.95 20.76 20.90
20.75 21.37 20.17 21.03
21.15 21.05 20.85 21.04
Expression Level
21.50
21.25
21.00
20.75
20.50
20.25
Dominant Cell Type
a GE & S
a LE
a S
MaxQuant MBR S Image
19.04 19.20 ND 19.08
18.71 18.74 ND 18.80
19.54 18.84 ND ND
19.57 19.23 ND 18.73
19.68 19.27 18.96 19.05
19.37 19.60 19.19 ND
Expression Level
19.50
19.25
19.00
18.75
Dominant Cell Type
a GE & S
a LE
a S
MaxQuantMBR LE Image
21.34 20.48 21.26 21.39
21.02 20.65 20.80 21.04
21.54 20.95 20.49 20.91
21.30 20.95 20.82 20.97
20.88 21.40 20.51 21.06
21.31 21.13 20.87 21.00
Expression Level
21.50
21.25
21.00
20.75
20.50
Dominant Cell Type
a GE & S
a LE
a S

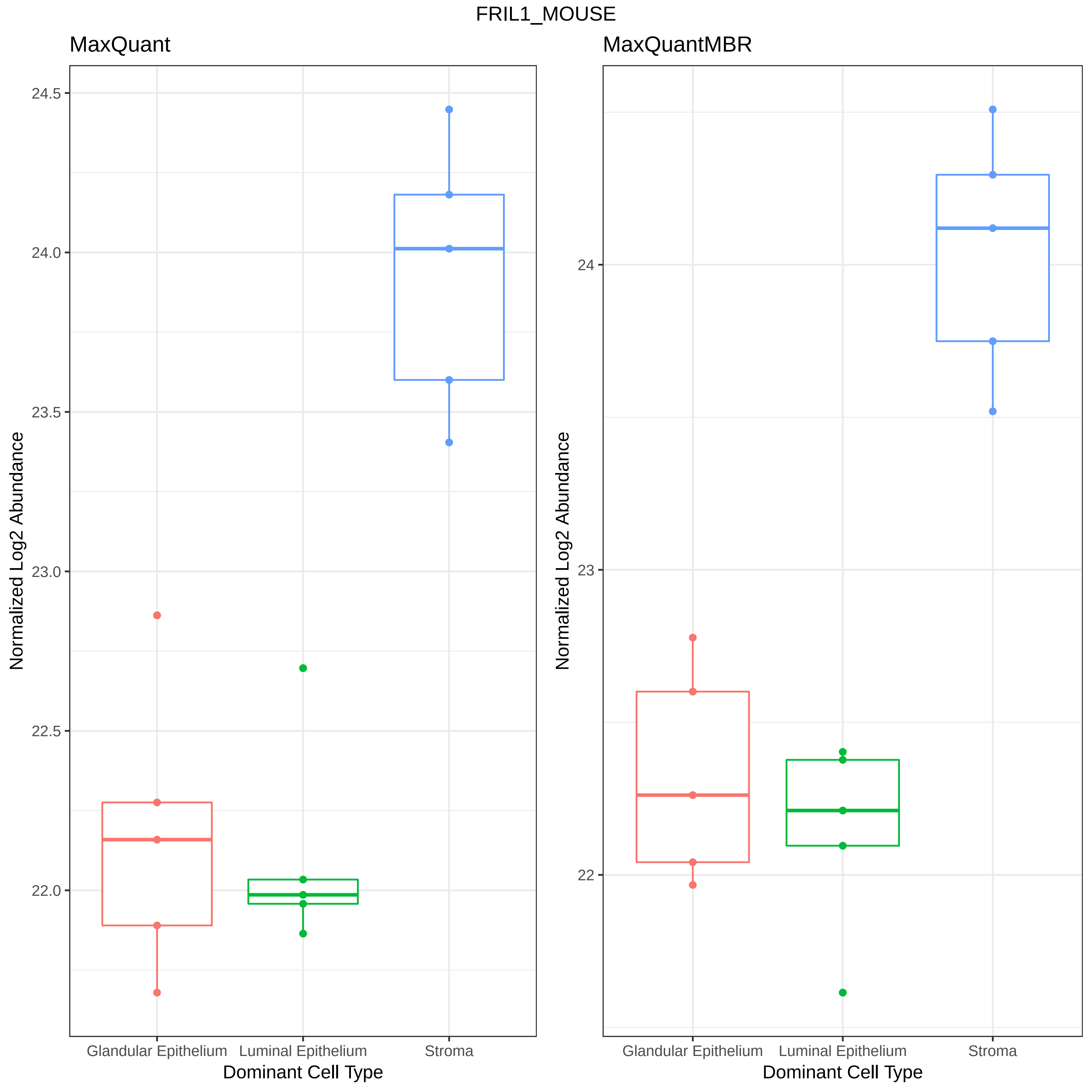

FRIL1_MOUSE
MaxQuant
MaxQuantMBR
Normalized Log2 Abundance
24.5
24.0
23.5
23.0
22.5
22.0
24
23
22
Glandular Epithelium
Luminal Epithelium
Stroma
Dominant Cell Type

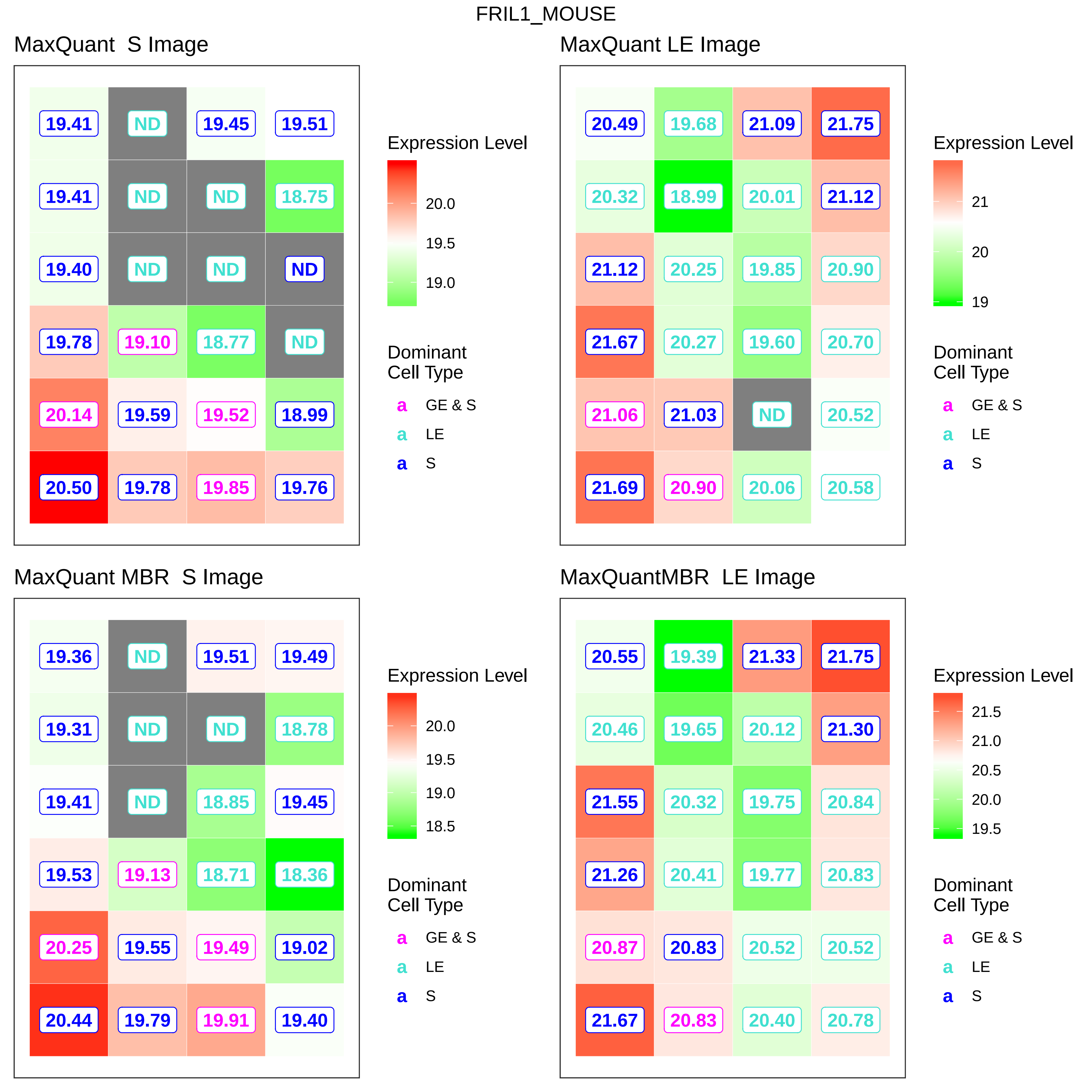
FRIL1_MOUSE
MaxQuant S Image
19.41
ND
19.45
19.51
19.41
ND
ND
18.75
19.40
ND
ND
ND
19.78
19.10
18.77
ND
20.14
19.59
19.52
18.99
20.50
19.78
19.85
19.76
Expression Level
20.0
19.5
19.0
Dominant Cell Type
a GE & S
a LE
a S
MaxQuant LE Image
20.49
19.68
21.09
21.75
20.32
18.99
20.01
21.12
21.12
20.25
19.85
20.90
21.67
20.27
19.60
20.70
21.06
21.03
ND
20.52
21.69
20.90
20.06
20.58
Expression Level
21
20
19
Dominant Cell Type
a GE & S
a LE
a S
MaxQuant MBR S Image
19.36
ND
19.51
19.49
19.31
ND
ND
18.78
19.41
ND
18.85
19.45
19.53
19.13
18.71
18.36
20.25
19.55
19.49
19.02
20.44
19.79
19.91
19.40
Expression Level
20.0
19.5
19.0
18.5
Dominant Cell Type
a GE & S
a LE
a S
MaxQuantMBR LE Image
20.55
19.39
21.33
21.75
20.46
19.65
20.12
21.30
21.55
20.32
19.75
20.84
21.26
20.41
19.77
20.83
20.87
20.83
20.52
20.52
21.67
20.83
20.40
20.78
Expression Level
21.5
21.0
20.5
20.0
19.5
Dominant Cell Type
a GE & S
a LE
a S

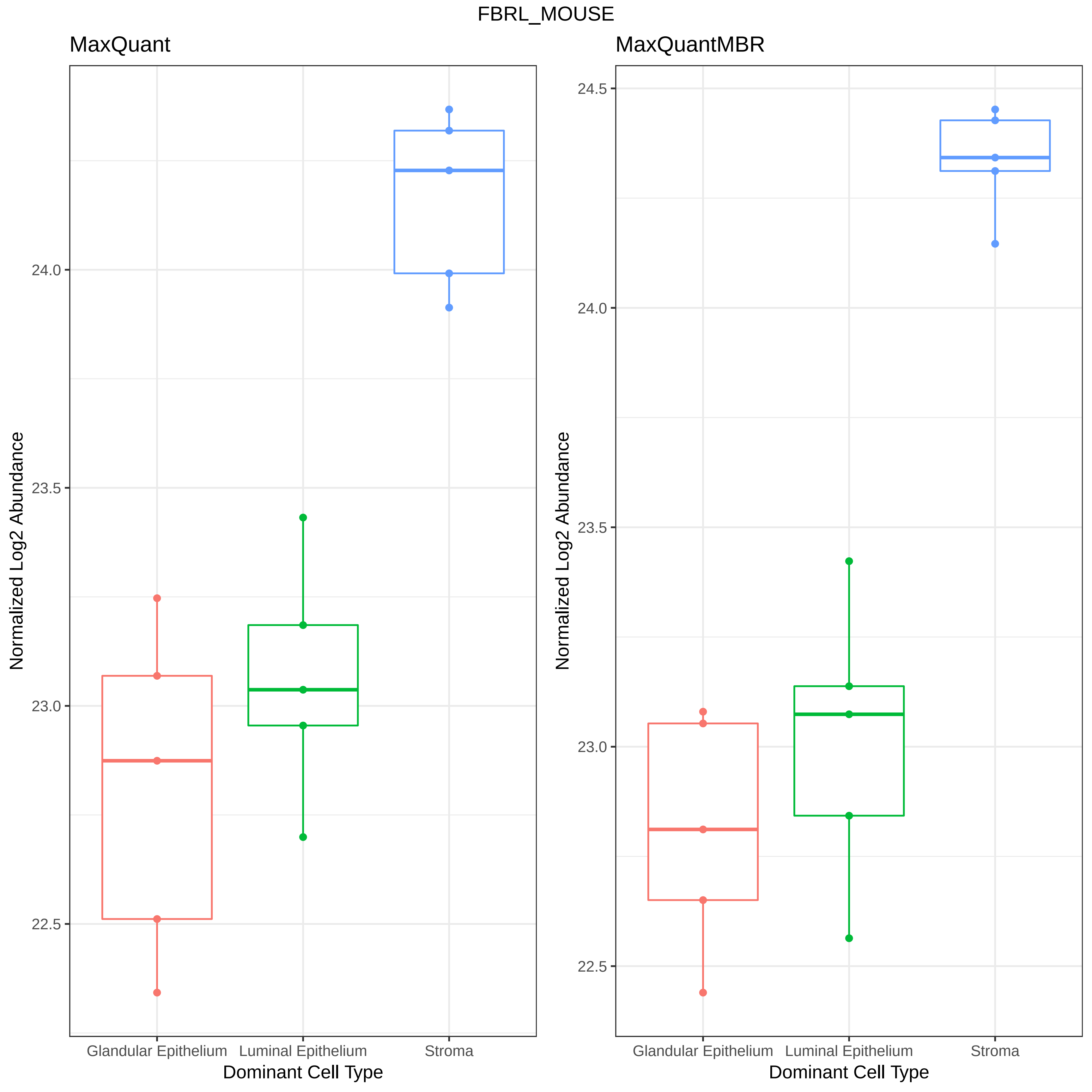
FBRL_MOUSE
MaxQuant
MaxQuantMBR
Normalized Log2 Abundance
24.5
24.0
23.5
23.0
22.5
Glandular Epithelium
Luminal Epithelium
Stroma
Dominant Cell Type

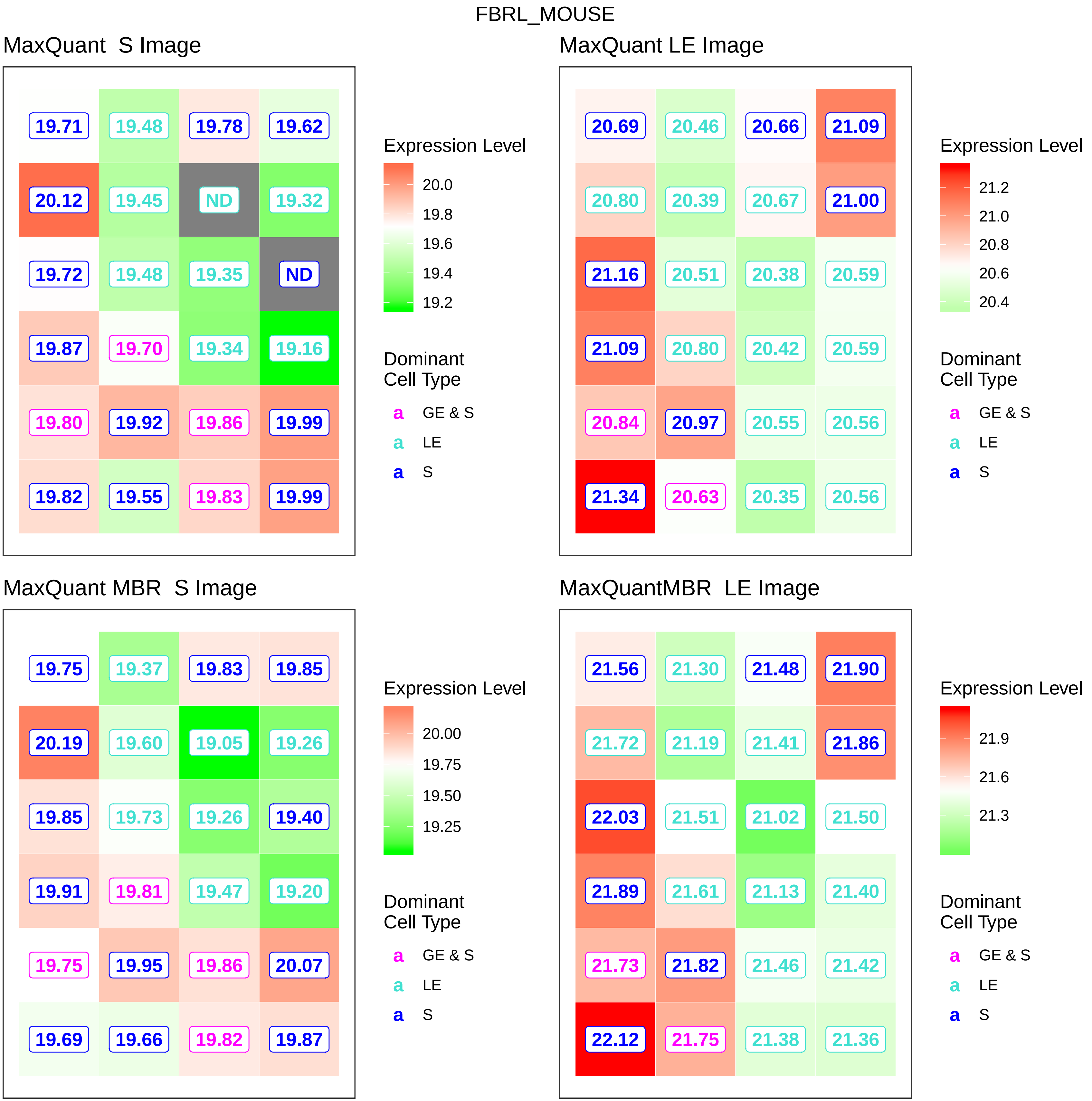
FBRL_MOUSE
MaxQuant S Image
19.71 19.48 19.78 19.62
20.12 19.45 ND 19.32
19.72 19.48 19.35 ND
19.87 19.70 19.34 19.16
19.80 19.92 19.86 19.99
19.82 19.55 19.83 19.99
Expression Level
20.0
19.8
19.6
19.4
19.2
Dominant Cell Type
a GE & S
a LE
a S
MaxQuant LE Image
20.69 20.46 20.66 21.09
20.80 20.39 20.67 21.00
21.16 20.51 20.38 20.59
21.09 20.80 20.42 20.59
20.84 20.97 20.55 20.56
21.34 20.63 20.35 20.56
Expression Level
21.2
21.0
20.8
20.6
20.4
Dominant Cell Type
a GE & S
a LE
a S
MaxQuant MBR S Image
19.75 19.37 19.83 19.85
20.19 19.60 19.05 19.26
19.85 19.73 19.26 19.40
19.91 19.81 19.47 19.20
19.75 19.95 19.86 20.07
19.69 19.66 19.82 19.87
Expression Level
20.00
19.75
19.50
19.25
Dominant Cell Type
a GE & S
a LE
a S
MaxQuantMBR LE Image
21.56 21.30 21.48 21.90
21.72 21.19 21.41 21.86
22.03 21.51 21.02 21.50
21.89 21.61 21.13 21.40
21.73 21.82 21.46 21.42
22.12 21.75 21.38 21.36
Expression Level
21.9
21.6
21.3
Dominant Cell Type
a GE & S
a LE
a S

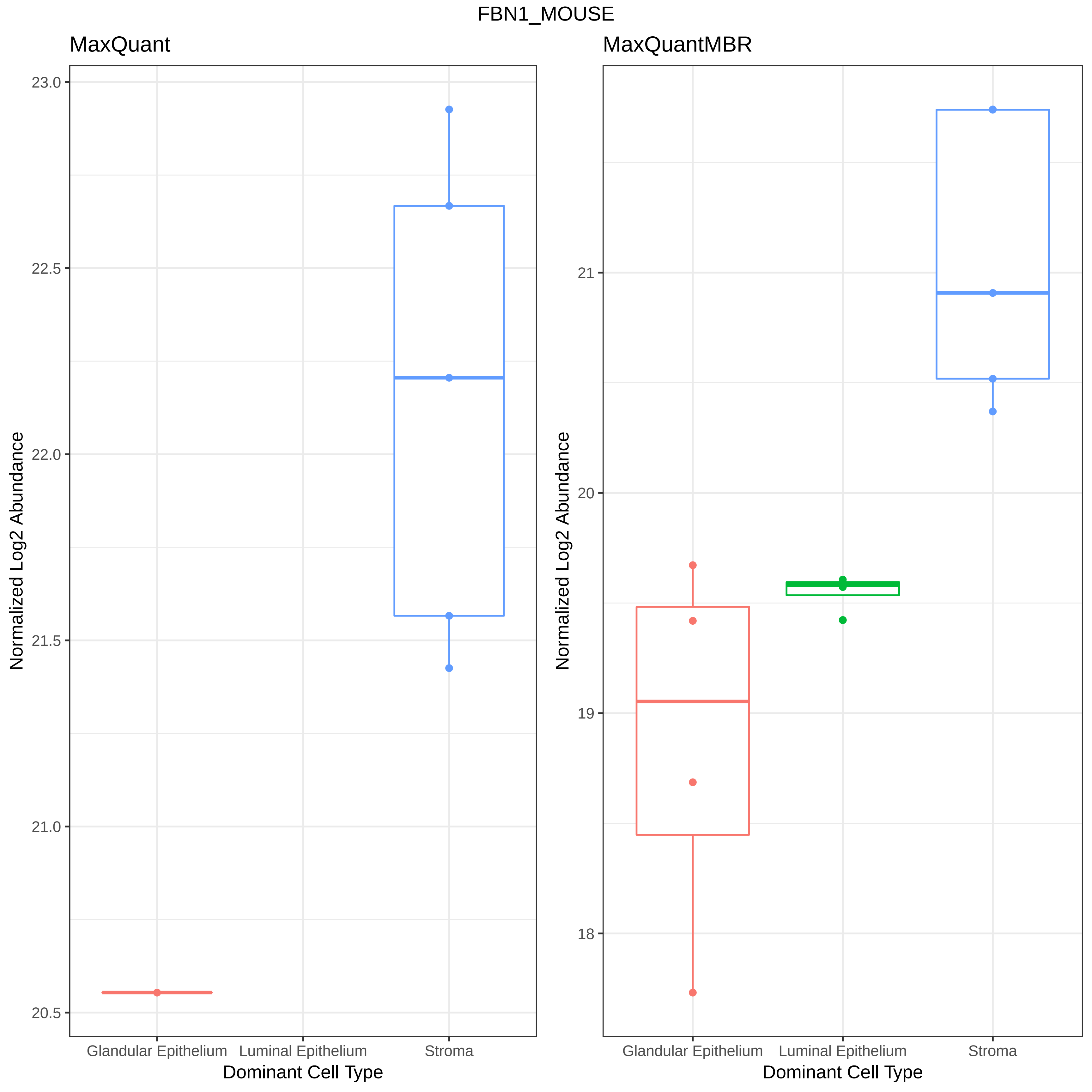

FBN1_MOUSE
MaxQuant
MaxQuantMBR
Normalized Log2 Abundance
Dominant Cell Type
23.0
22.5
22.0
21.5
21.0
20.5
21
20
19
18
Glandular Epithelium
Luminal Epithelium
Stroma

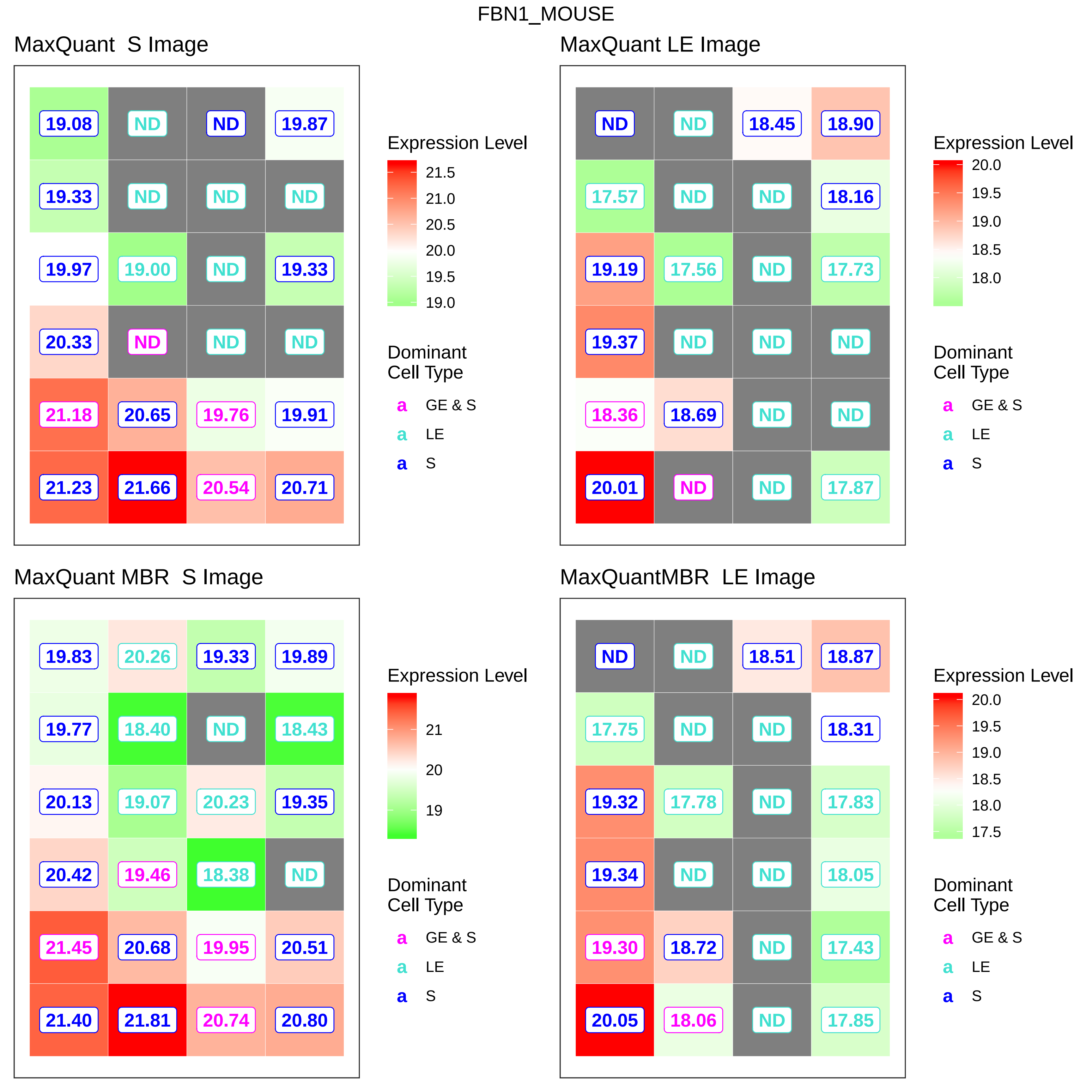
FBN1_MOUSE
MaxQuant S Image
19.08 ND ND 19.87
19.33 ND ND ND
19.97 19.00 ND 19.33
20.33 ND ND ND
21.18 20.65 19.76 19.91
21.23 21.66 20.54 20.71
Expression Level
21.5 21.0 20.5 20.0 19.5 19.0
Dominant Cell Type
a GE & S
a LE
a S
MaxQuant LE Image
ND ND 18.45 18.90
17.57 ND ND 18.16
19.19 17.56 ND 17.73
19.37 ND ND ND
18.36 18.69 ND ND
20.01 ND ND 17.87
Expression Level
20.0 19.5 19.0 18.5 18.0
Dominant Cell Type
a GE & S
a LE
a S
MaxQuant MBR S Image
19.83 20.26 19.33 19.89
19.77 18.40 ND 18.43
20.13 19.07 20.23 19.35
20.42 19.46 18.38 ND
21.45 20.68 19.95 20.51
21.40 21.81 20.74 20.80
Expression Level
21 20 19
Dominant Cell Type
a GE & S
a LE
a S
MaxQuantMBR LE Image
ND ND 18.51 18.87
17.75 ND ND 18.31
19.32 17.78 ND 17.83
19.34 ND ND 18.05
19.30 18.72 ND 17.43
20.05 18.06 ND 17.85
Expression Level
20.0 19.5 19.0 18.5 18.0 17.5
Dominant Cell Type
a GE & S
a LE
a S

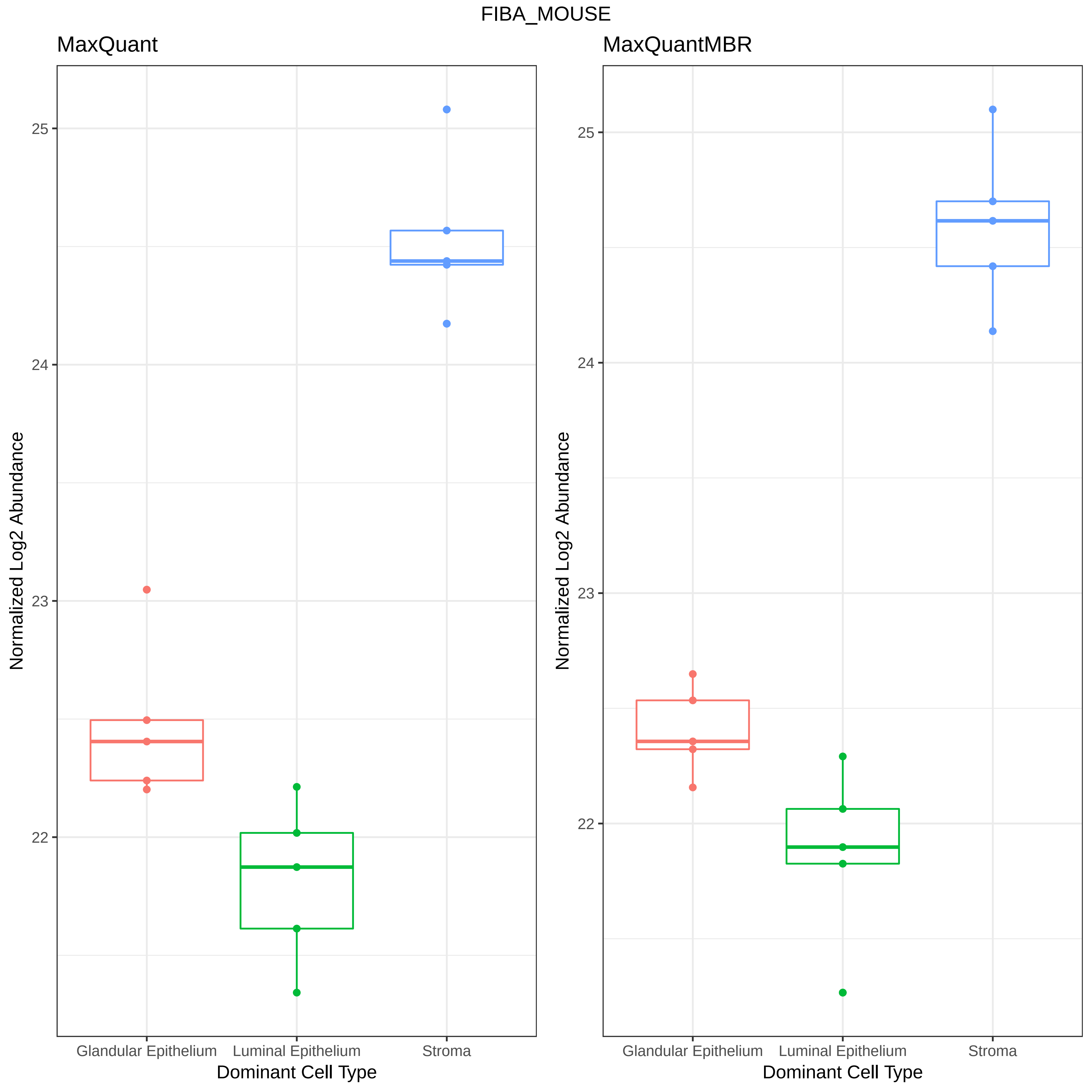
FIBA_MOUSE
MaxQuant
MaxQuantMBR
Normalized Log2 Abundance
25
24
23
22
Glandular Epithelium
Luminal Epithelium
Stroma
Dominant Cell Type

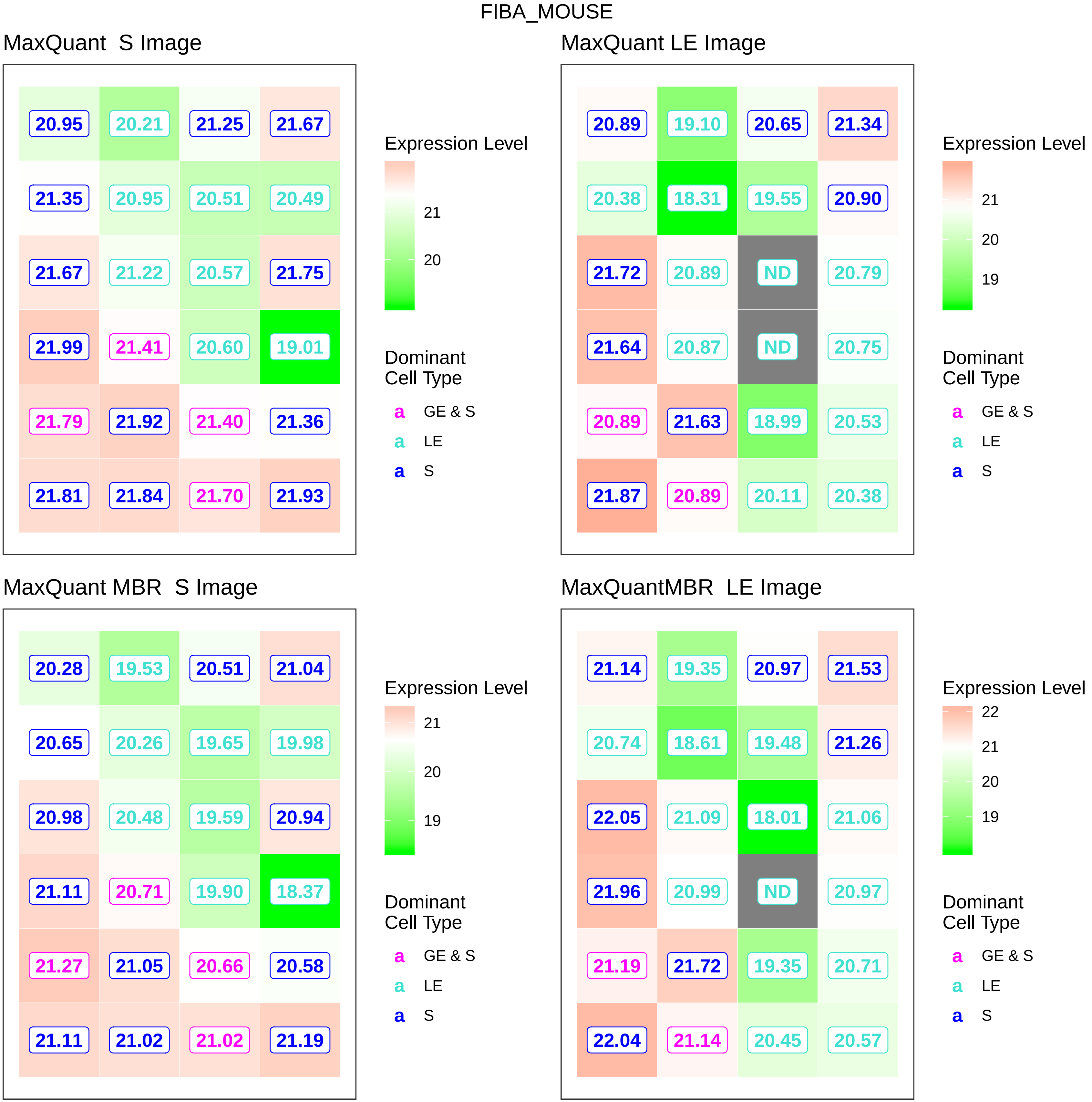

FIBA_MOUSE
MaxQuant S Image
20.95 20.21 21.25 21.67
21.35 20.95 20.51 20.49
21.67 21.22 20.57 21.75
21.99 21.41 20.60 19.01
21.79 21.92 21.40 21.36
21.81 21.84 21.70 21.93
Expression Level
21
20
Dominant Cell Type
a GE & S
a LE
a S
MaxQuant LE Image
20.89 19.10 20.65 21.34
20.38 18.31 19.55 20.90
21.72 20.89 ND 20.79
21.64 20.87 ND 20.75
20.89 21.63 18.99 20.53
21.87 20.89 20.11 20.38
Expression Level
21
20
19
Dominant Cell Type
a GE & S
a LE
a S
MaxQuant MBR S Image
20.28 19.53 20.51 21.04
20.65 20.26 19.65 19.98
20.98 20.48 19.59 20.94
21.11 20.71 19.90 18.37
21.27 21.05 20.66 20.58
21.11 21.02 21.02 21.19
Expression Level
21
20
19
Dominant Cell Type
a GE & S
a LE
a S
MaxQuantMBR LE Image
21.14 19.35 20.97 21.53
20.74 18.61 19.48 21.26
22.05 21.09 18.01 21.06
21.96 20.99 ND 20.97
21.19 21.72 19.35 20.71
22.04 21.14 20.45 20.57
Expression Level
22
21
20
19
Dominant Cell Type
a GE & S
a LE
a S

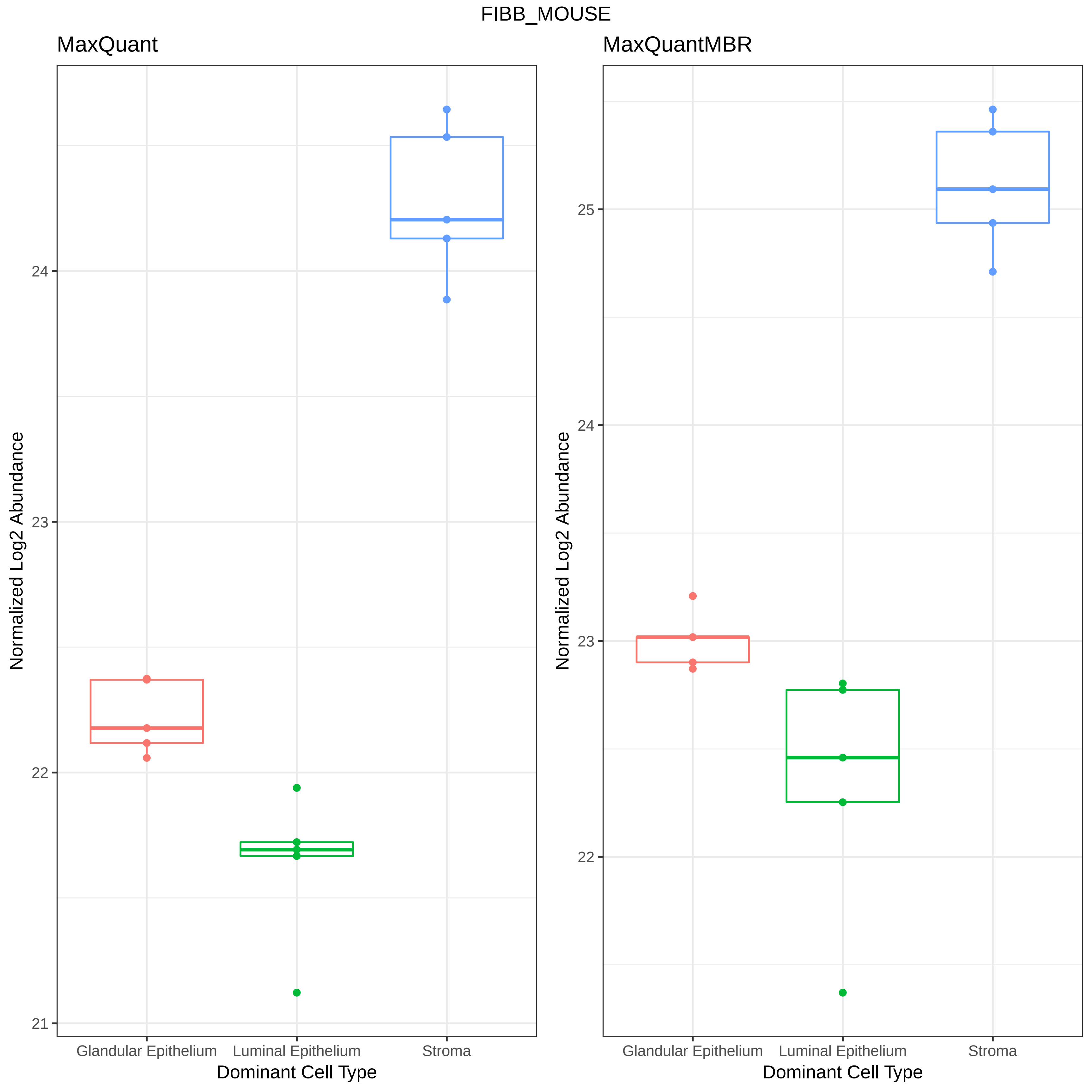
FIBB_MOUSE
MaxQuant
MaxQuantMBR
Normalized Log2 Abundance
21
22
23
24
25
Glandular Epithelium
Luminal Epithelium
Stroma
Dominant Cell Type

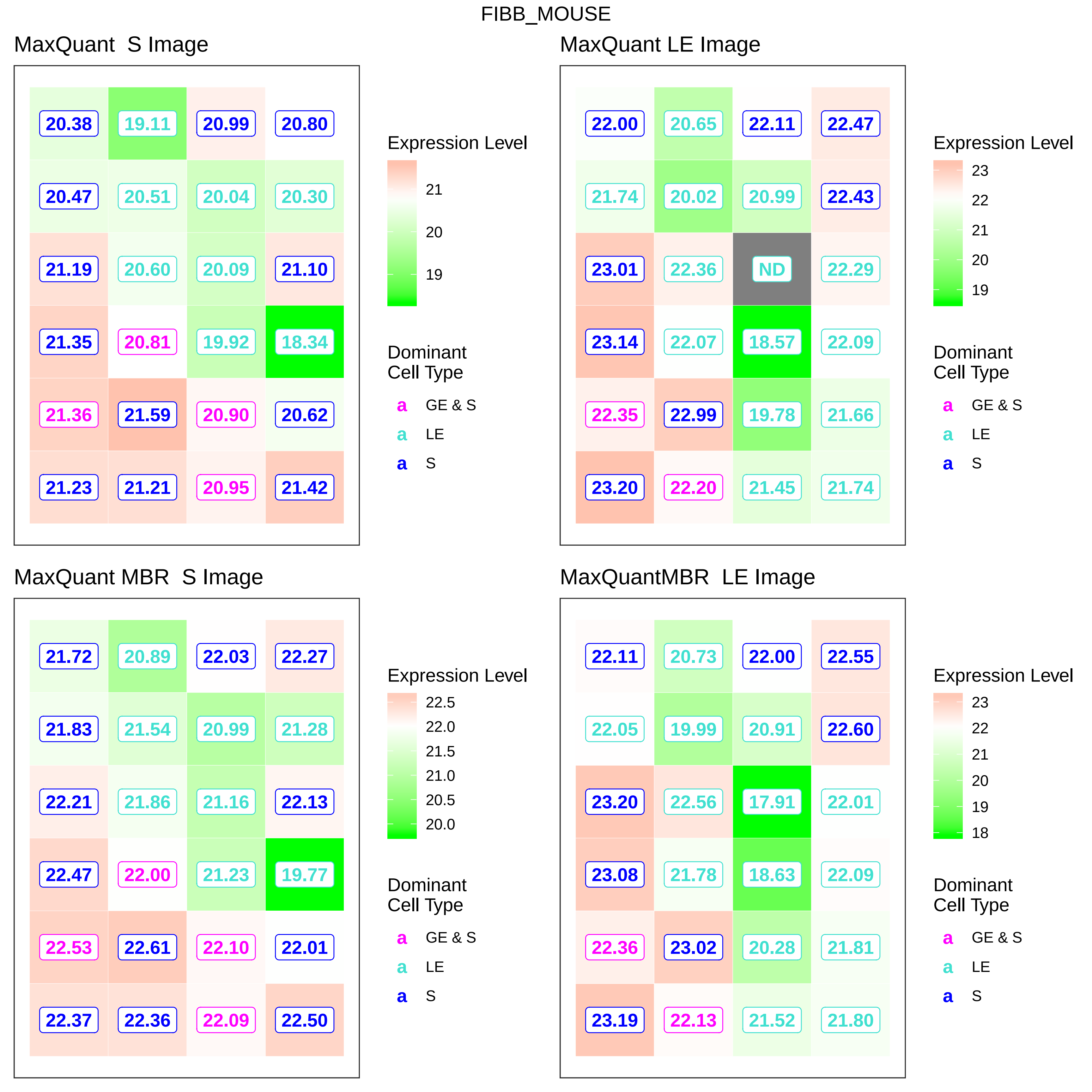
FIBB_MOUSE
MaxQuant S Image
20.38 19.11 20.99 20.80
20.47 20.51 20.04 20.30
21.19 20.60 20.09 21.10
21.35 20.81 19.92 18.34
21.36 21.59 20.90 20.62
21.23 21.21 20.95 21.42
Expression Level
21
20
19
Dominant Cell Type
a GE & S
a LE
a S
MaxQuant LE Image
22.00 20.65 22.11 22.47
21.74 20.02 20.99 22.43
23.01 22.36 ND 22.29
23.14 22.07 18.57 22.09
22.35 22.99 19.78 21.66
23.20 22.20 21.45 21.74
Expression Level
23
22
21
20
19
Dominant Cell Type
a GE & S
a LE
a S
MaxQuant MBR S Image
21.72 20.89 22.03 22.27
21.83 21.54 20.99 21.28
22.21 21.86 21.16 22.13
22.47 22.00 21.23 19.77
22.53 22.61 22.10 22.01
22.37 22.36 22.09 22.50
Expression Level
22.5
22.0
21.5
21.0
20.5
20.0
Dominant Cell Type
a GE & S
a LE
a S
MaxQuantMBR LE Image
22.11 20.73 22.00 22.55
22.05 19.99 20.91 22.60
23.20 22.56 17.91 22.01
23.08 21.78 18.63 22.09
22.36 23.02 20.28 21.81
23.19 22.13 21.52 21.80
Expression Level
23
22
21
20
19
18
Dominant Cell Type
a GE & S
a LE
a S

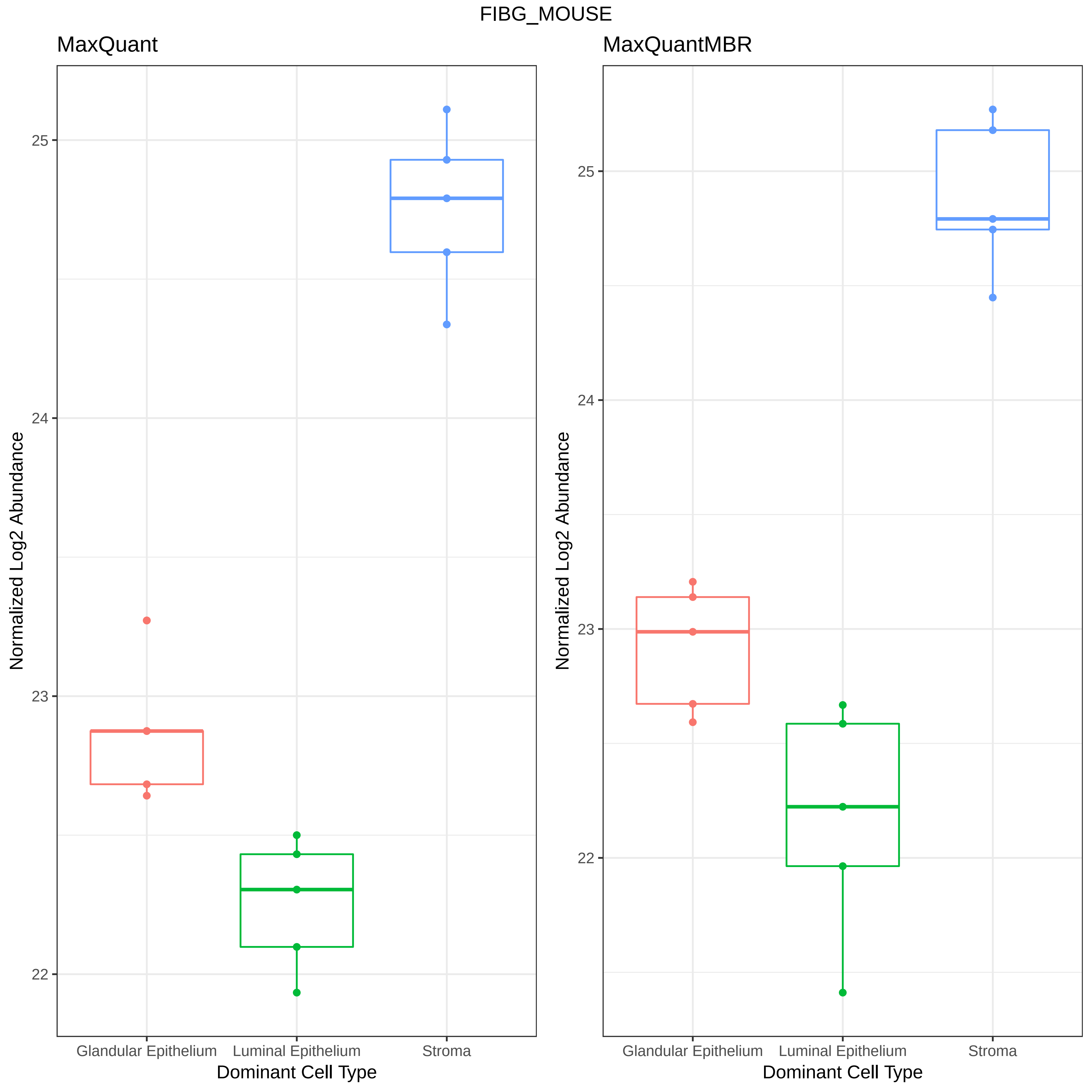
FIBG_MOUSE
MaxQuant
MaxQuantMBR
Normalized Log2 Abundance
25
24
23
22
Glandular Epithelium
Luminal Epithelium
Stroma
Dominant Cell Type

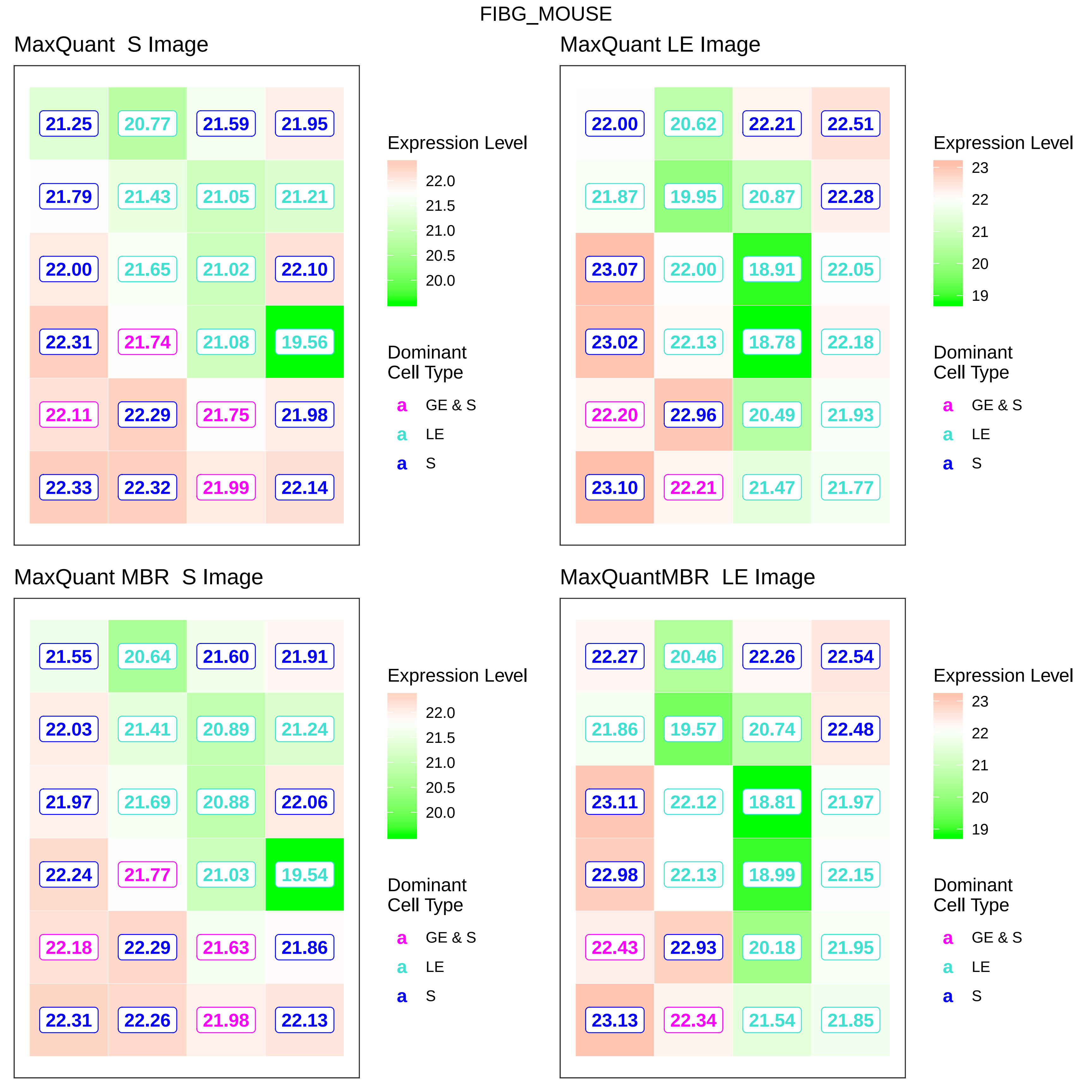
FIBG_MOUSE
MaxQuant S Image
21.25 20.77 21.59 21.95
21.79 21.43 21.05 21.21
22.00 21.65 21.02 22.10
22.31 21.74 21.08 19.56
22.11 22.29 21.75 21.98
22.33 22.32 21.99 22.14
Expression Level
22.0
21.5
21.0
20.5
20.0
Dominant Cell Type
a GE & S
a LE
a S
MaxQuant LE Image
22.00 20.62 22.21 22.51
21.87 19.95 20.87 22.28
23.07 22.00 18.91 22.05
23.02 22.13 18.78 22.18
22.20 22.96 20.49 21.93
23.10 22.21 21.47 21.77
Expression Level
23
22
21
20
19
Dominant Cell Type
a GE & S
a LE
a S
MaxQuant MBR S Image
21.55 20.64 21.60 21.91
22.03 21.41 20.89 21.24
21.97 21.69 20.88 22.06
22.24 21.77 21.03 19.54
22.18 22.29 21.63 21.86
22.31 22.26 21.98 22.13
Expression Level
22.0
21.5
21.0
20.5
20.0
Dominant Cell Type
a GE & S
a LE
a S
MaxQuantMBR LE Image
22.27 20.46 22.26 22.54
21.86 19.57 20.74 22.48
23.11 22.12 18.81 21.97
22.98 22.13 18.99 22.15
22.43 22.93 20.18 21.95
23.13 22.34 21.54 21.85
Expression Level
23
22
21
20
19
Dominant Cell Type
a GE & S
a LE
a S

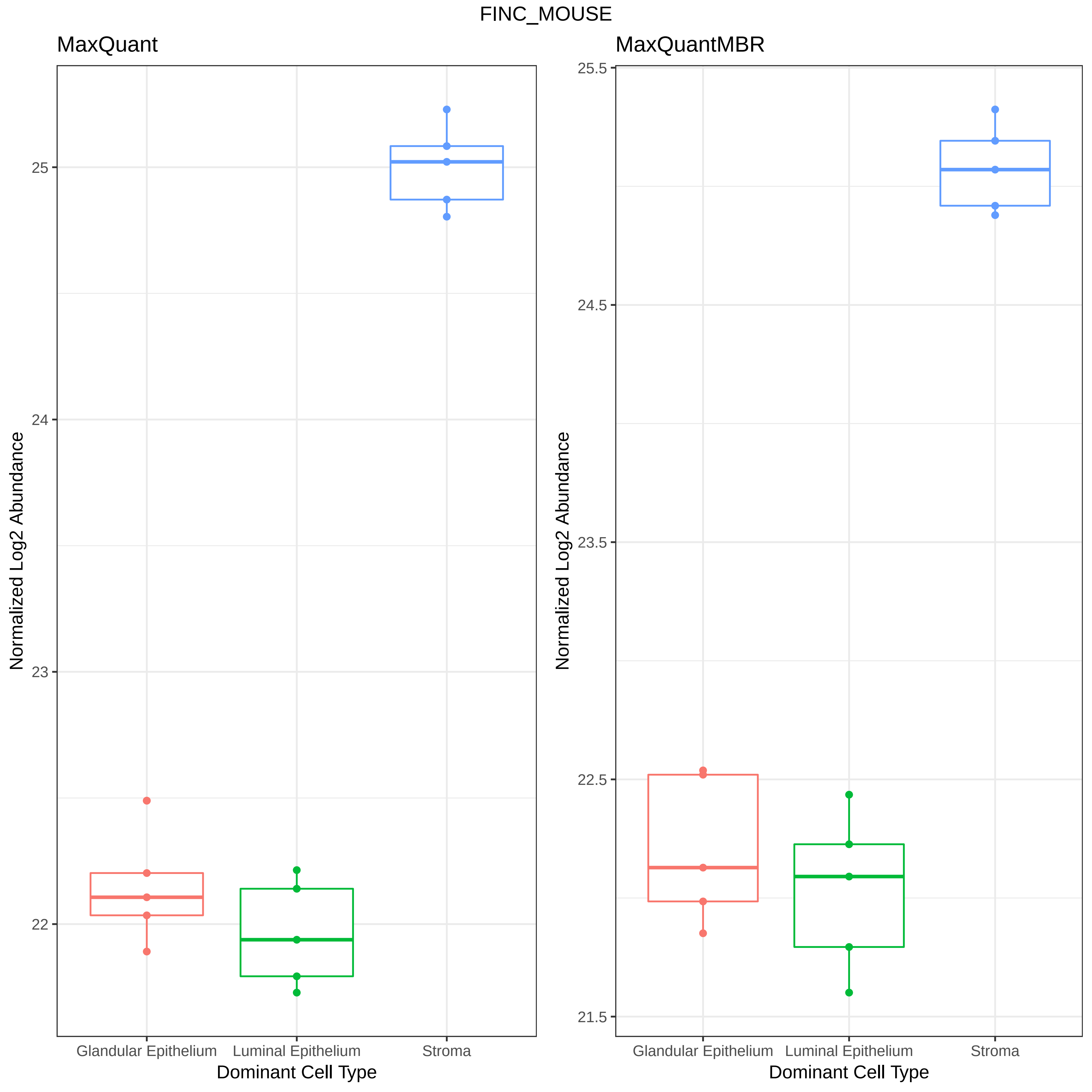

FINC_MOUSE
MaxQuant
MaxQuantMBR
Normalized Log2 Abundance
25
24
23
22
25.5
24.5
23.5
22.5
21.5
Glandular Epithelium
Luminal Epithelium
Stroma
Dominant Cell Type

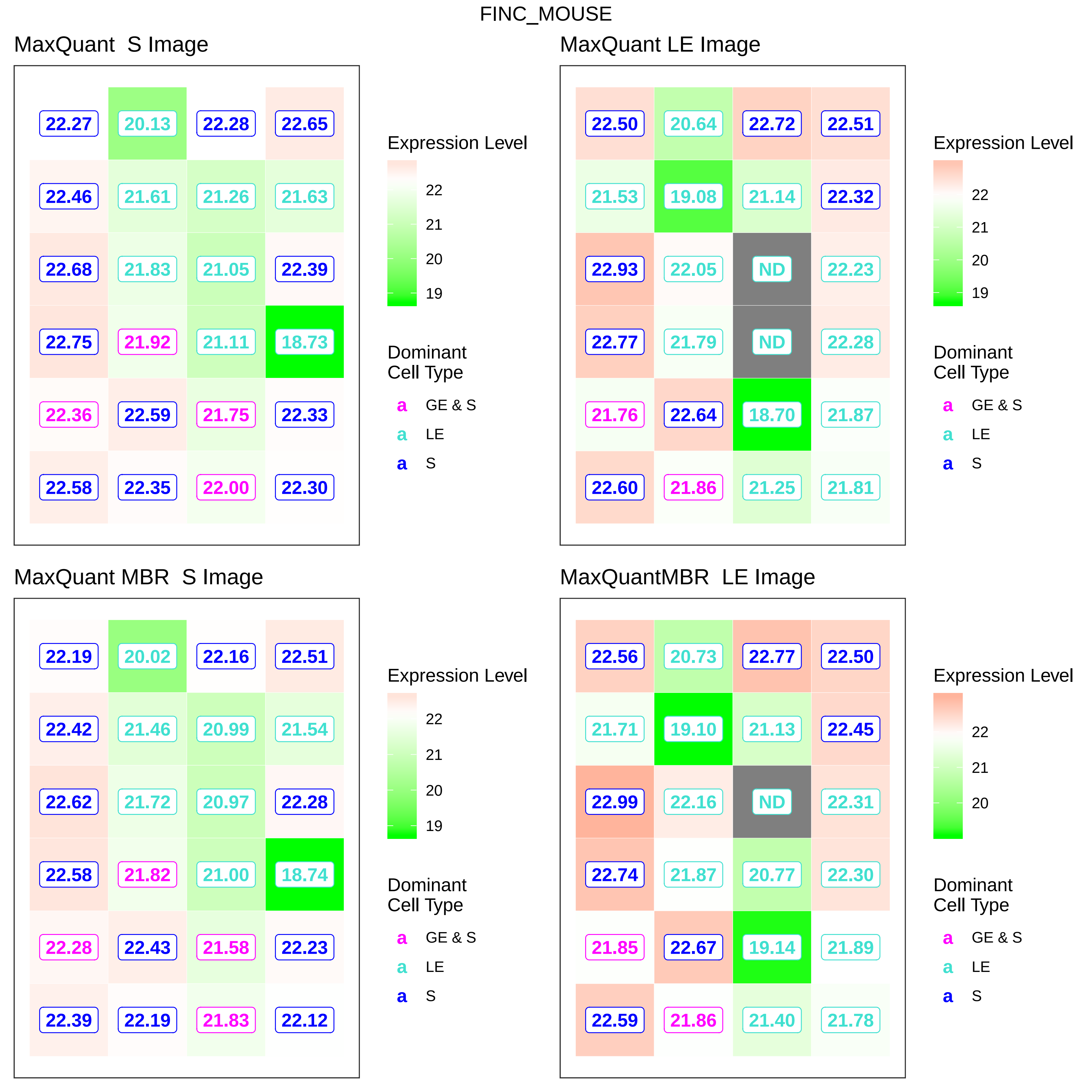
FINC_MOUSE
MaxQuant S Image
22.27 20.13 22.28 22.65
22.46 21.61 21.26 21.63
22.68 21.83 21.05 22.39
22.75 21.92 21.11 18.73
22.36 22.59 21.75 22.33
22.58 22.35 22.00 22.30
Expression Level
22
21
20
19
Dominant Cell Type
a GE & S
a LE
a S
MaxQuant LE Image
22.50 20.64 22.72 22.51
21.53 19.08 21.14 22.32
22.93 22.05 ND 22.23
22.77 21.79 ND 22.28
21.76 22.64 18.70 21.87
22.60 21.86 21.25 21.81
Expression Level
22
21
20
19
Dominant Cell Type
a GE & S
a LE
a S
MaxQuant MBR S Image
22.19 20.02 22.16 22.51
22.42 21.46 20.99 21.54
22.62 21.72 20.97 22.28
22.58 21.82 21.00 18.74
22.28 22.43 21.58 22.23
22.39 22.19 21.83 22.12
Expression Level
22
21
20
19
Dominant Cell Type
a GE & S
a LE
a S
MaxQuantMBR LE Image
22.56 20.73 22.77 22.50
21.71 19.10 21.13 22.45
22.99 22.16 ND 22.31
22.74 21.87 20.77 22.30
21.85 22.67 19.14 21.89
22.59 21.86 21.40 21.78
Expression Level
22
21
20
Dominant Cell Type
a GE & S
a LE
a S

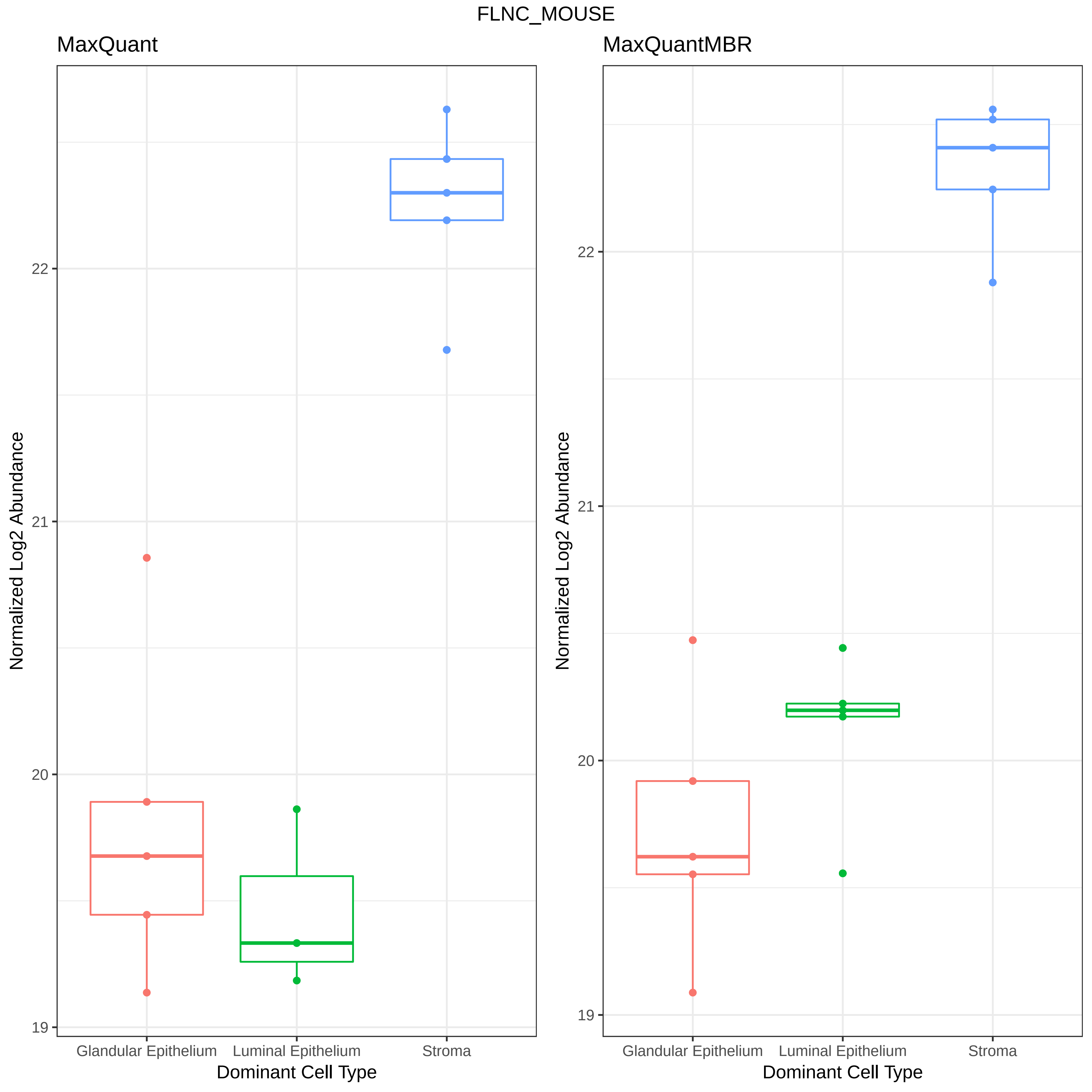

FLNC_MOUSE
MaxQuant
MaxQuantMBR
Normalized Log2 Abundance
22
21
20
19
Glandular Epithelium
Luminal Epithelium
Stroma
Dominant Cell Type

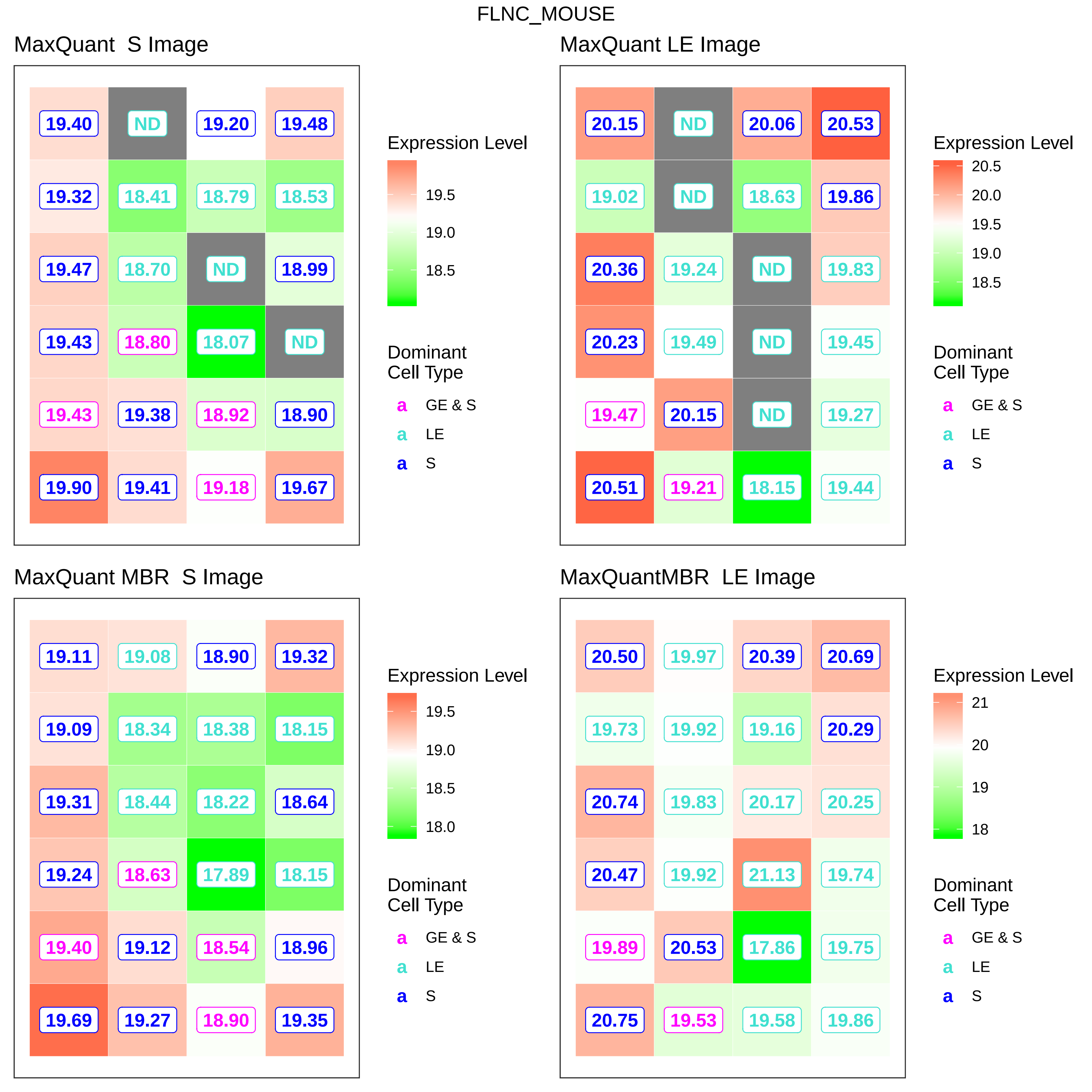
FLNC_MOUSE
MaxQuant S Image
19.40
ND
19.20
19.48
19.32
18.41
18.79
18.53
19.47
18.70
ND
18.99
19.43
18.80
18.07
ND
19.43
19.38
18.92
18.90
19.90
19.41
19.18
19.67
Expression Level
19.5
19.0
18.5
Dominant Cell Type
a GE & S
a LE
a S
MaxQuant LE Image
20.15
ND
20.06
20.53
19.02
ND
18.63
19.86
20.36
19.24
ND
19.83
20.23
19.49
ND
19.45
19.47
20.15
ND
19.27
20.51
19.21
18.15
19.44
Expression Level
20.5
20.0
19.5
19.0
18.5
Dominant Cell Type
a GE & S
a LE
a S
MaxQuant MBR S Image
19.11
19.08
18.90
19.32
19.09
18.34
18.38
18.15
19.31
18.44
18.22
18.64
19.24
18.63
17.89
18.15
19.40
19.12
18.54
18.96
19.69
19.27
18.90
19.35
Expression Level
19.5
19.0
18.5
18.0
Dominant Cell Type
a GE & S
a LE
a S
MaxQuantMBR LE Image
20.50
19.97
20.39
20.69
19.73
19.92
19.16
20.29
20.74
19.83
20.17
20.25
20.47
19.92
21.13
19.74
19.89
20.53
17.86
19.75
20.75
19.53
19.58
19.86
Expression Level
21
20
19
18
Dominant Cell Type
a GE & S
a LE
a S

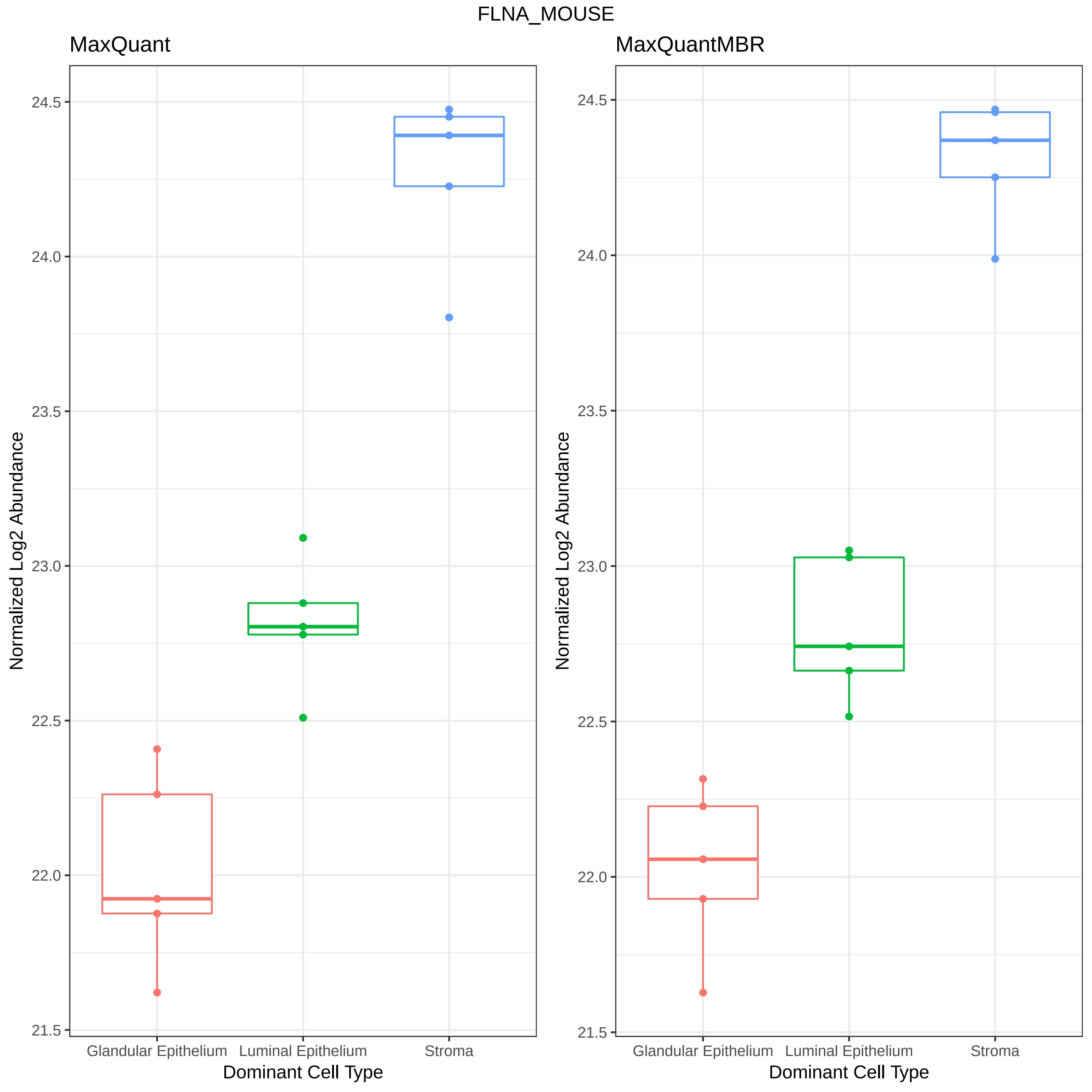
FLNA_MOUSE
MaxQuant
MaxQuantMBR
Normalized Log2 Abundance
24.5
24.0
23.5
23.0
22.5
22.0
21.5
Glandular Epithelium
Luminal Epithelium
Stroma
Dominant Cell Type

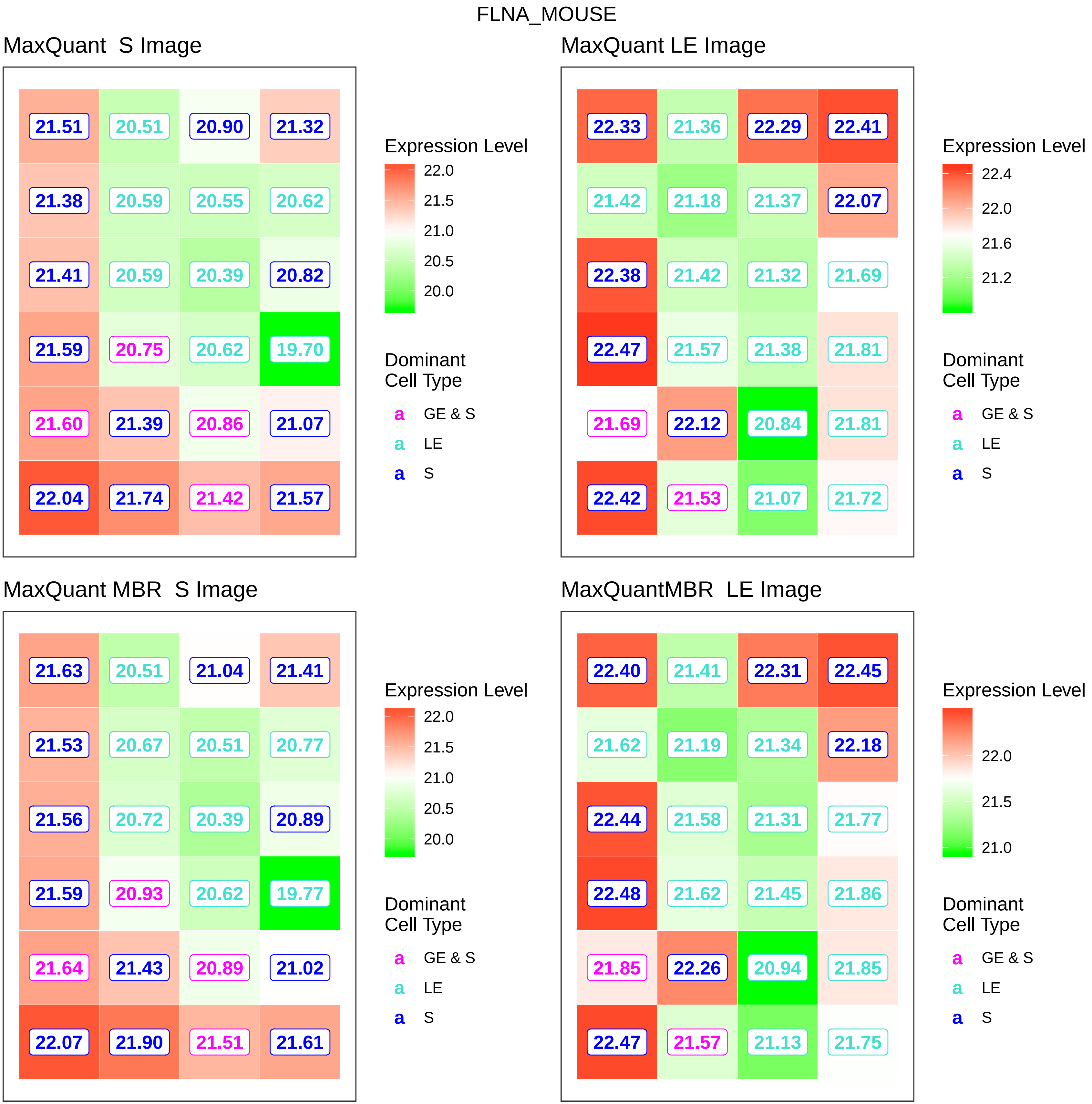
FLNA_MOUSE
MaxQuant S Image
21.51 20.51 20.90 21.32
21.38 20.59 20.55 20.62
21.41 20.59 20.39 20.82
21.59 20.75 20.62 19.70
21.60 21.39 20.86 21.07
22.04 21.74 21.42 21.57
Expression Level
22.0
21.5
21.0
20.5
20.0
Dominant Cell Type
a GE & S
a LE
a S
MaxQuant LE Image
22.33 21.36 22.29 22.41
21.42 21.18 21.37 22.07
22.38 21.42 21.32 21.69
22.47 21.57 21.38 21.81
21.69 22.12 20.84 21.81
22.42 21.53 21.07 21.72
Expression Level
22.4
22.0
21.6
21.2
Dominant Cell Type
a GE & S
a LE
a S
MaxQuant MBR S Image
21.63 20.51 21.04 21.41
21.53 20.67 20.51 20.77
21.56 20.72 20.39 20.89
21.59 20.93 20.62 19.77
21.64 21.43 20.89 21.02
22.07 21.90 21.51 21.61
Expression Level
22.0
21.5
21.0
20.5
20.0
Dominant Cell Type
a GE & S
a LE
a S
MaxQuantMBR LE Image
22.40 21.41 22.31 22.45
21.62 21.19 21.34 22.18
22.44 21.58 21.31 21.77
22.48 21.62 21.45 21.86
21.85 22.26 20.94 21.85
22.47 21.57 21.13 21.75
Expression Level
22.0
21.5
21.0
Dominant Cell Type
a GE & S
a LE
a S

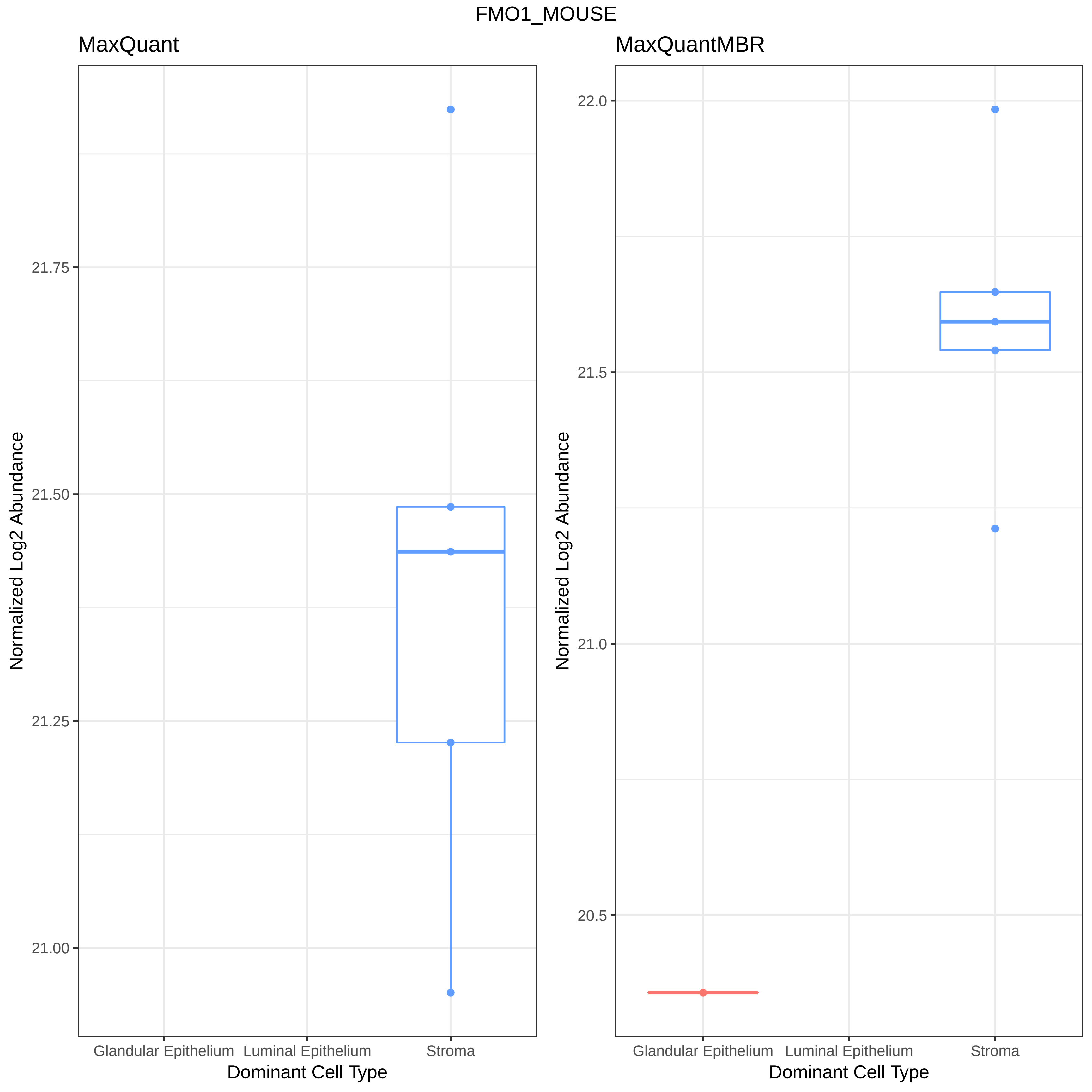
FMO1_MOUSE
MaxQuant
MaxQuantMBR
Normalized Log2 Abundance
21.75
21.50
21.25
21.00
22.0
21.5
21.0
20.5
Glandular Epithelium
Luminal Epithelium
Stroma
Dominant Cell Type

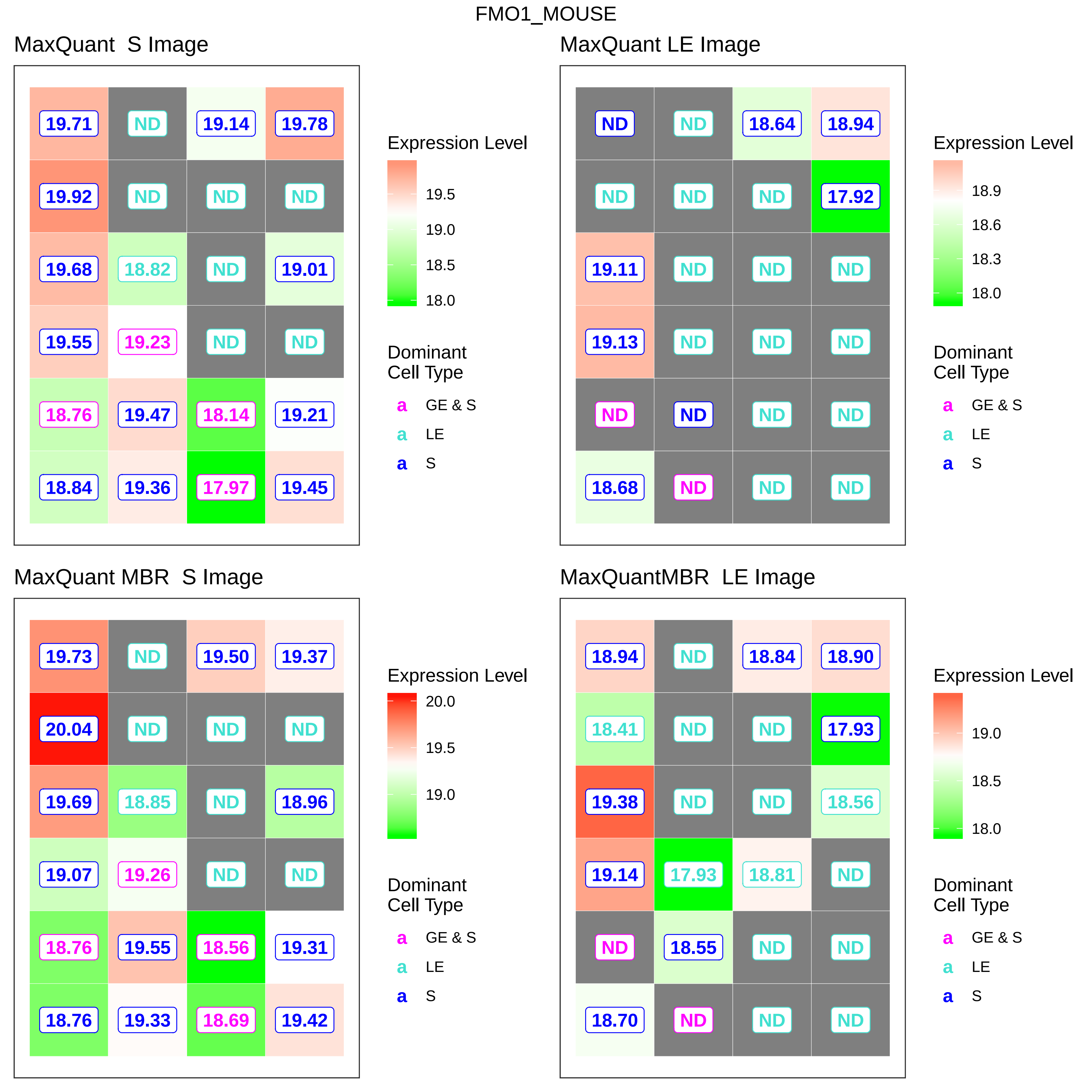

FMO1_MOUSE
MaxQuant S Image
19.71 ND 19.14 19.78
19.92 ND ND ND
19.68 18.82 ND 19.01
19.55 19.23 ND ND
18.76 19.47 18.14 19.21
18.84 19.36 17.97 19.45
Expression Level
19.5
19.0
18.5
18.0
Dominant Cell Type
a GE & S
a LE
a S
MaxQuant LE Image
ND ND 18.64 18.94
ND ND ND 17.92
19.11 ND ND ND
19.13 ND ND ND
ND ND ND ND
18.68 ND ND ND
Expression Level
18.9
18.6
18.3
18.0
Dominant Cell Type
a GE & S
a LE
a S
MaxQuant MBR S Image
19.73 ND 19.50 19.37
20.04 ND ND ND
19.69 18.85 ND 18.96
19.07 19.26 ND ND
18.76 19.55 18.56 19.31
18.76 19.33 18.69 19.42
Expression Level
20.0
19.5
19.0
Dominant Cell Type
a GE & S
a LE
a S
MaxQuantMBR LE Image
18.94 ND 18.84 18.90
18.41 ND ND 17.93
19.38 ND ND 18.56
19.14 17.93 18.81 ND
ND 18.55 ND ND
18.70 ND ND ND
Expression Level
19.0
18.5
18.0
Dominant Cell Type
a GE & S
a LE
a S

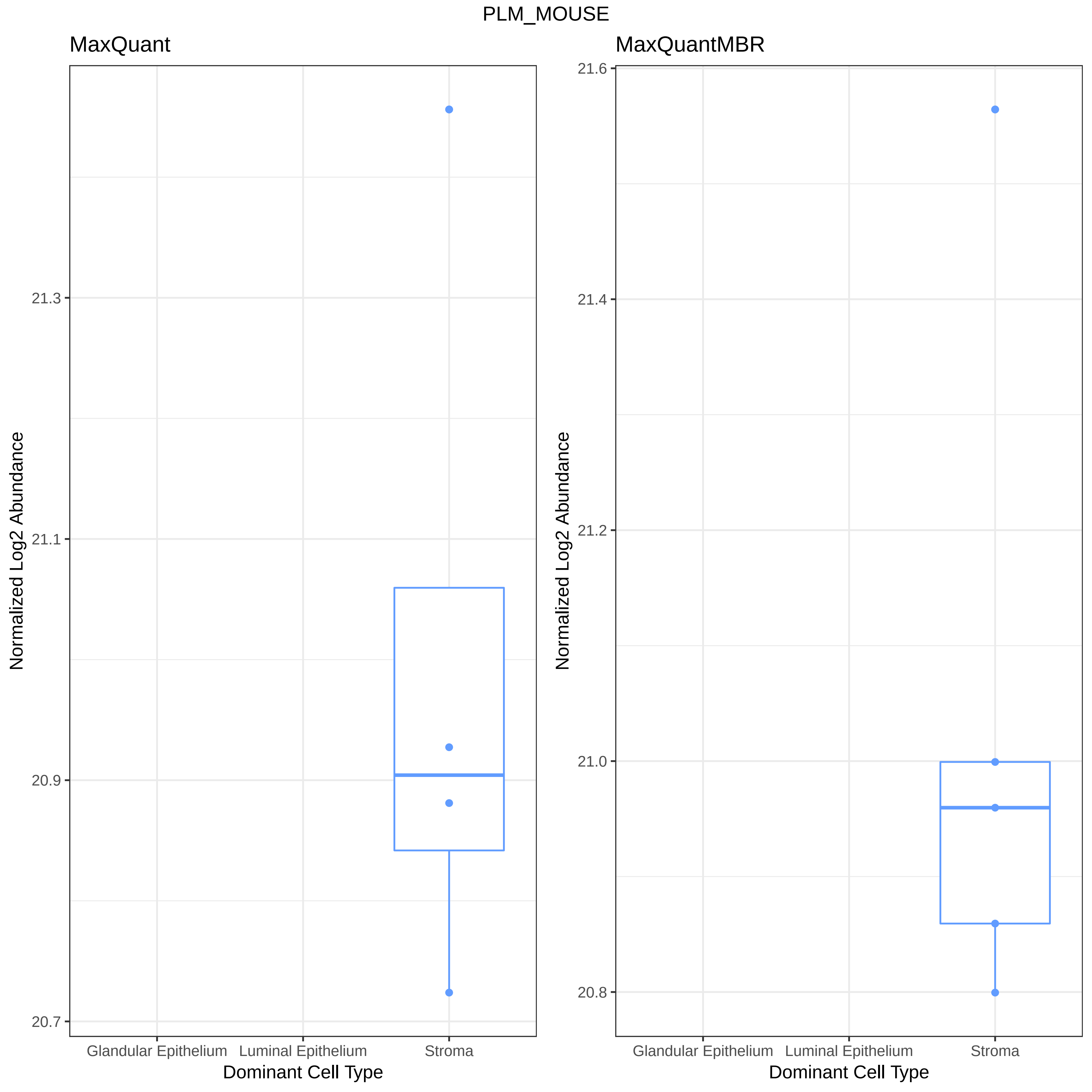
PLM_MOUSE
MaxQuant
MaxQuantMBR
Normalized Log2 Abundance
21.3
21.1
20.9
20.7
21.6
21.4
21.2
21.0
20.8
Glandular Epithelium
Luminal Epithelium
Stroma
Dominant Cell Type

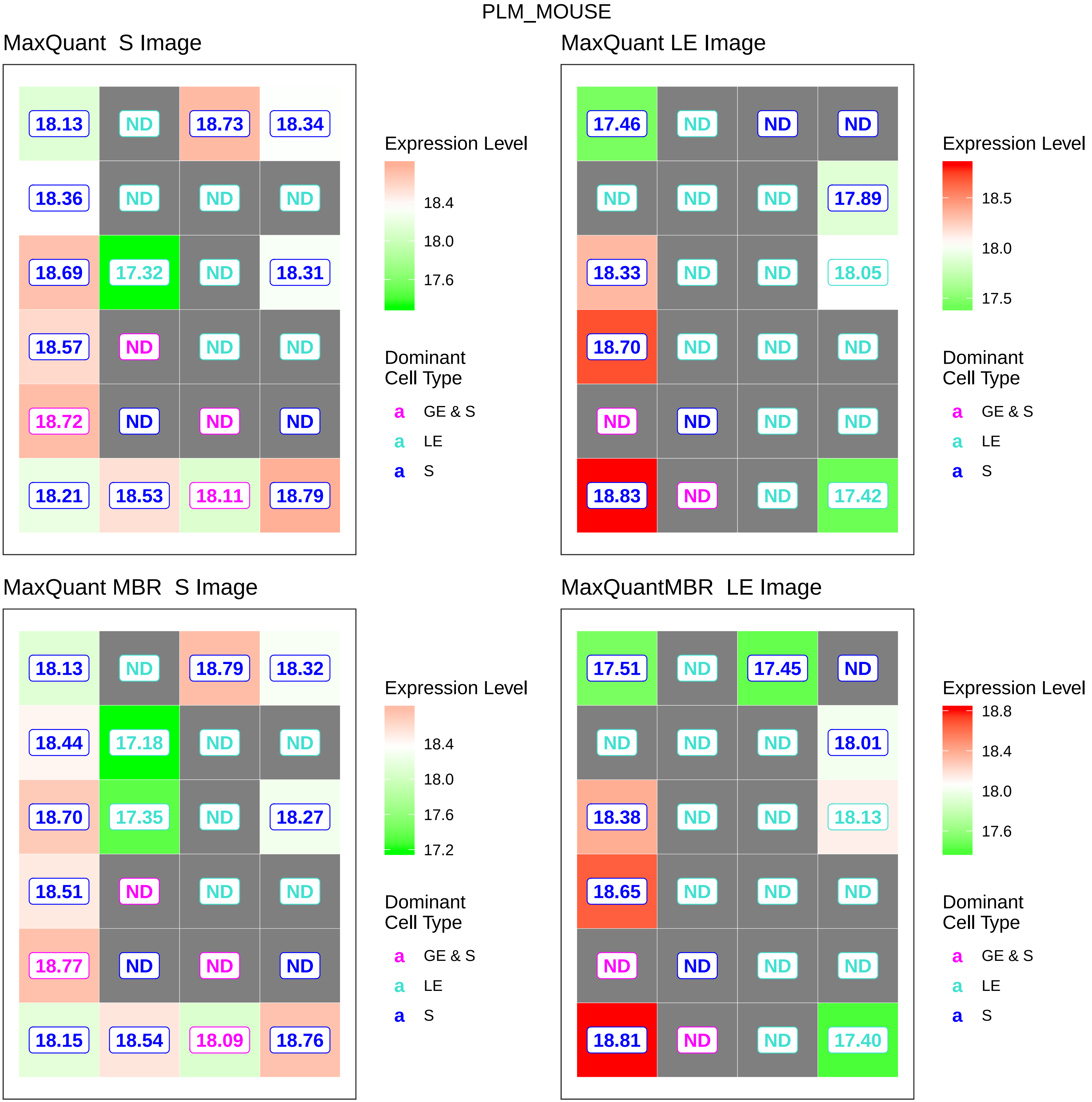
PLM_MOUSE
MaxQuant S Image
18.13 ND 18.73 18.34
18.36 ND ND ND
18.69 17.32 ND 18.31
18.57 ND ND ND
18.72 ND ND ND
18.21 18.53 18.11 18.79
Expression Level
18.4
18.0
17.6
Dominant Cell Type
a GE & S
a LE
a S
MaxQuant LE Image
17.46 ND ND ND
ND ND ND 17.89
18.33 ND ND 18.05
18.70 ND ND ND
ND ND ND ND
18.83 ND ND 17.42
Expression Level
18.5
18.0
17.5
Dominant Cell Type
a GE & S
a LE
a S
MaxQuant MBR S Image
18.13 ND 18.79 18.32
18.44 17.18 ND ND
18.70 17.35 ND 18.27
18.51 ND ND ND
18.77 ND ND ND
18.15 18.54 18.09 18.76
Expression Level
18.4
18.0
17.6
17.2
Dominant Cell Type
a GE & S
a LE
a S
MaxQuantMBR LE Image
17.51 ND 17.45 ND
ND ND ND 18.01
18.38 ND ND 18.13
18.65 ND ND ND
ND ND ND ND
18.81 ND ND 17.40
Expression Level
18.8
18.4
18.0
17.6
Dominant Cell Type
a GE & S
a LE
a S

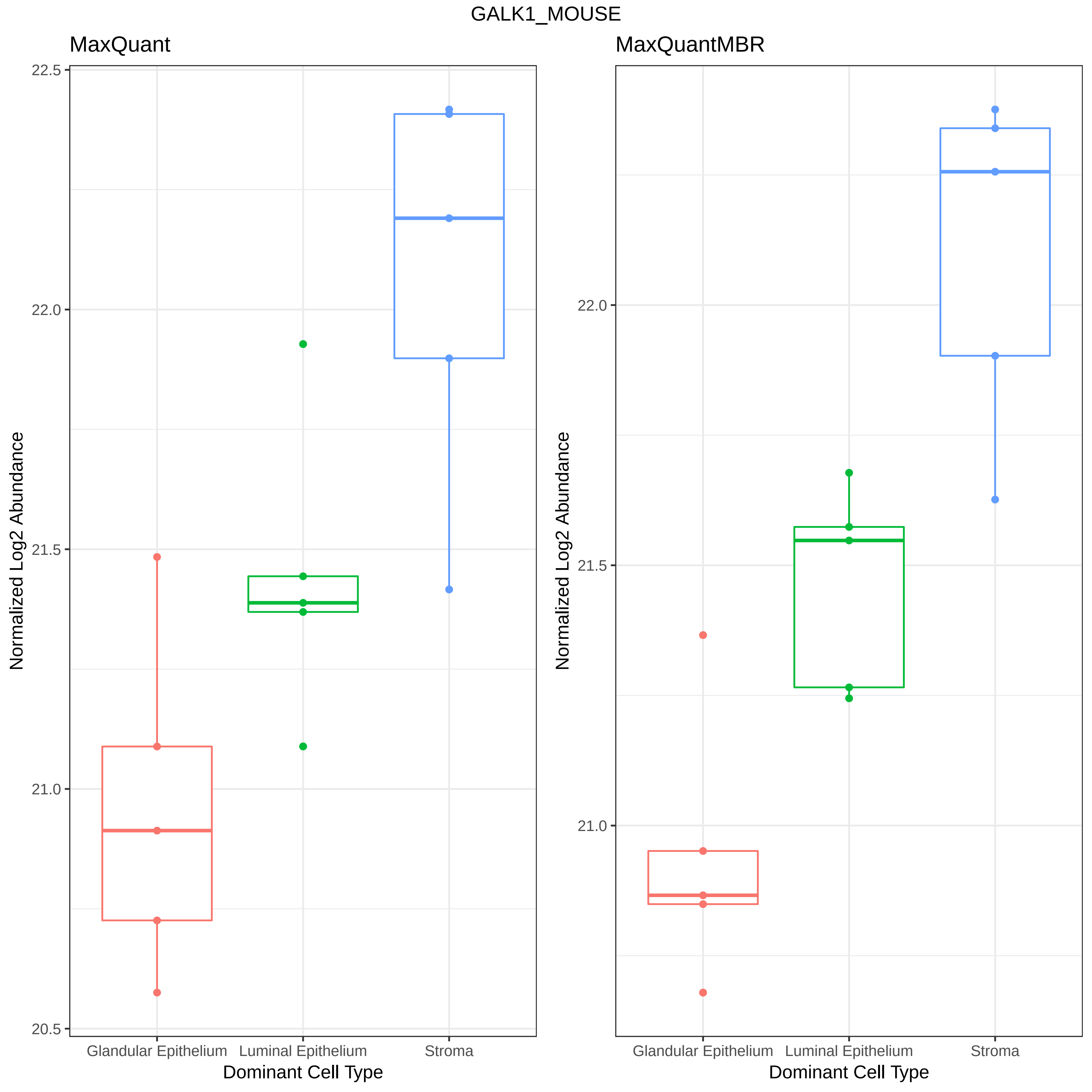
GALK1_MOUSE
MaxQuant
MaxQuantMBR
Normalized Log2 Abundance
22.5
22.0
21.5
21.0
20.5
Glandular Epithelium
Luminal Epithelium
Stroma
Dominant Cell Type

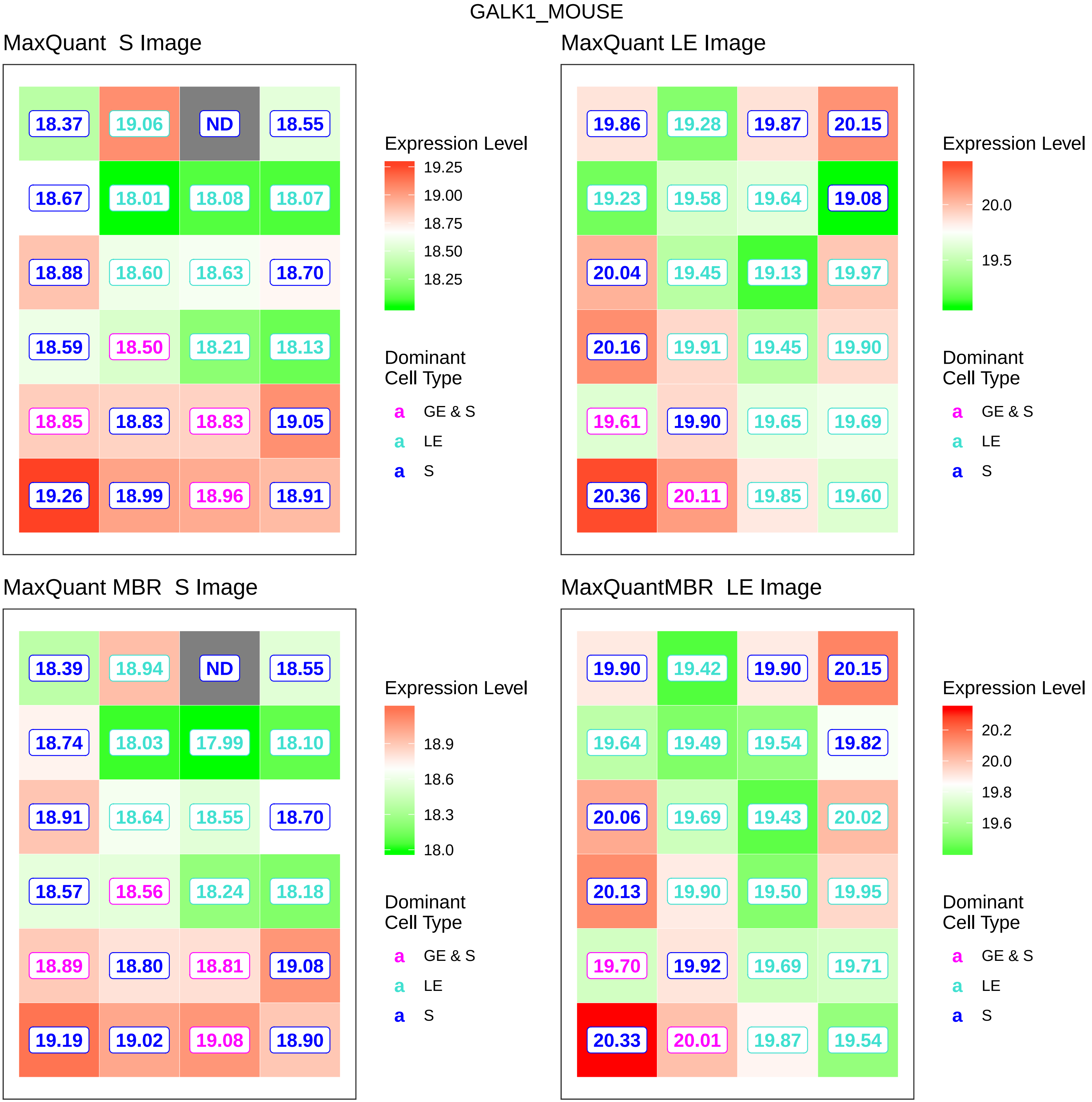
GALK1_MOUSE
MaxQuant S Image
18.37 19.06 ND 18.55
18.67 18.01 18.08 18.07
18.88 18.60 18.63 18.70
18.59 18.50 18.21 18.13
18.85 18.83 18.83 19.05
19.26 18.99 18.96 18.91
Expression Level
19.25
19.00
18.75
18.50
18.25
Dominant Cell Type
a GE & S
a LE
a S
MaxQuant LE Image
19.86 19.28 19.87 20.15
19.23 19.58 19.64 19.08
20.04 19.45 19.13 19.97
20.16 19.91 19.45 19.90
19.61 19.90 19.65 19.69
20.36 20.11 19.85 19.60
Expression Level
20.0
19.5
Dominant Cell Type
a GE & S
a LE
a S
MaxQuant MBR S Image
18.39 18.94 ND 18.55
18.74 18.03 17.99 18.10
18.91 18.64 18.55 18.70
18.57 18.56 18.24 18.18
18.89 18.80 18.81 19.08
19.19 19.02 19.08 18.90
Expression Level
18.9
18.6
18.3
18.0
Dominant Cell Type
a GE & S
a LE
a S
MaxQuantMBR LE Image
19.90 19.42 19.90 20.15
19.64 19.49 19.54 19.82
20.06 19.69 19.43 20.02
20.13 19.90 19.50 19.95
19.70 19.92 19.69 19.71
20.33 20.01 19.87 19.54
Expression Level
20.2
20.0
19.8
19.6
Dominant Cell Type
a GE & S
a LE
a S

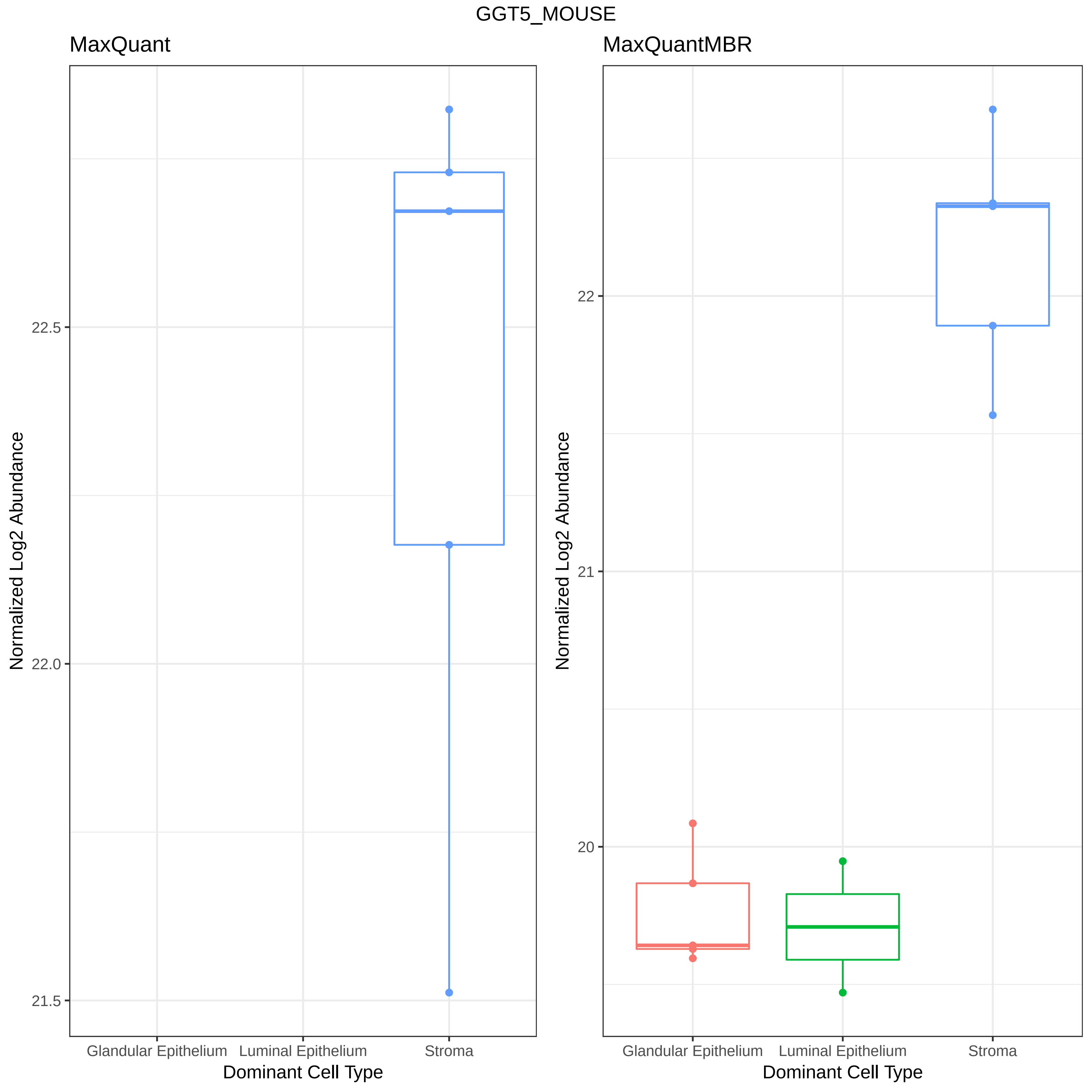
GGT5_MOUSE
MaxQuant
MaxQuantMBR
Normalized Log2 Abundance
22.5
22.0
21.5
22
21
20
Glandular Epithelium
Luminal Epithelium
Stroma
Dominant Cell Type

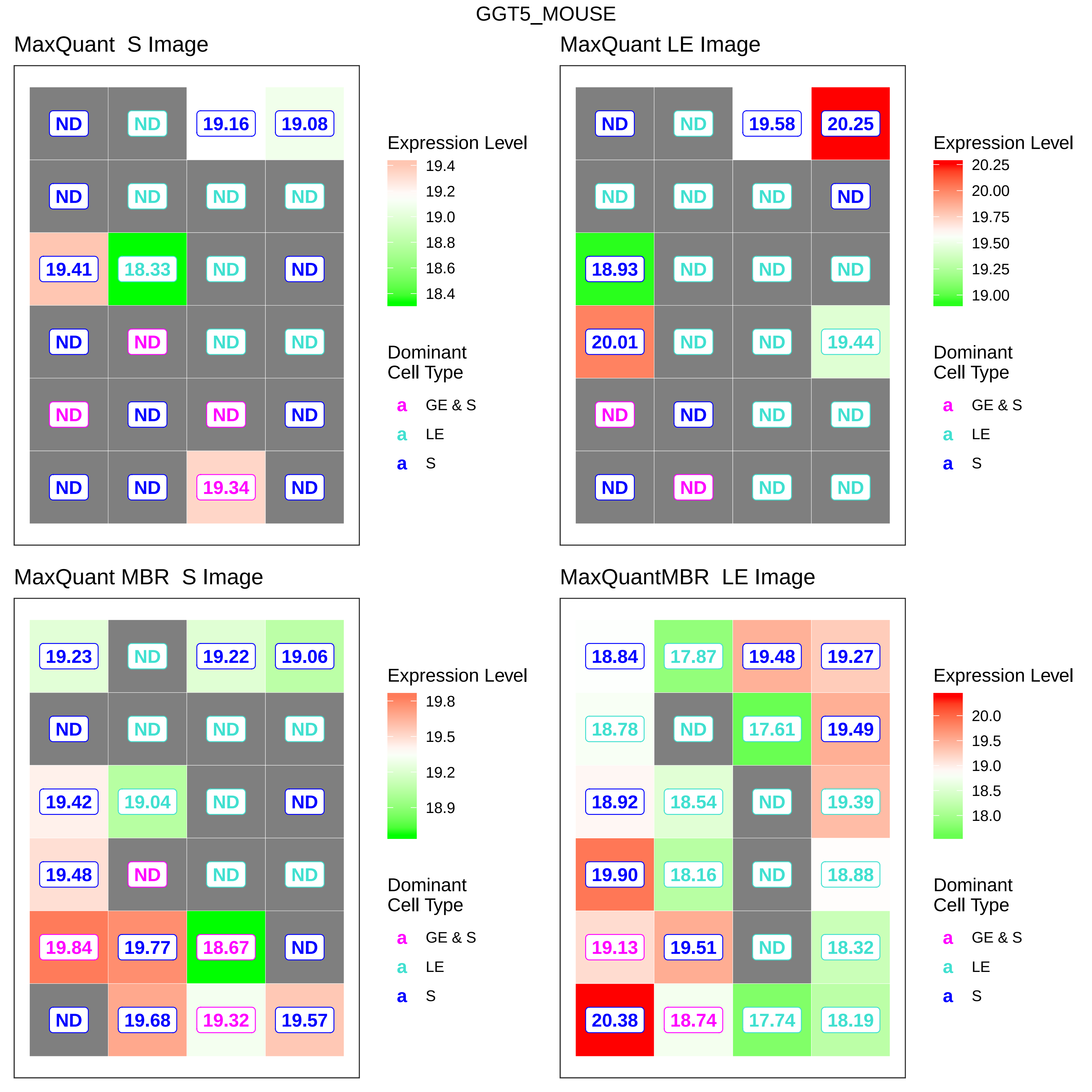
GGT5_MOUSE
MaxQuant S Image
ND ND 19.16 19.08
ND ND ND ND
19.41 18.33 ND ND
ND ND ND ND
ND ND ND ND
ND ND 19.34 ND
Expression Level
19.4
19.2
19.0
18.8
18.6
18.4
Dominant Cell Type
a GE & S
a LE
a S
MaxQuant LE Image
ND ND 19.58 20.25
ND ND ND ND
18.93 ND ND ND
20.01 ND ND 19.44
ND ND ND ND
ND ND ND ND
Expression Level
20.25
20.00
19.75
19.50
19.25
19.00
Dominant Cell Type
a GE & S
a LE
a S
MaxQuant MBR S Image
19.23 ND 19.22 19.06
ND ND ND ND
19.42 19.04 ND ND
19.48 ND ND ND
19.84 19.77 18.67 ND
ND 19.68 19.32 19.57
Expression Level
19.8
19.5
19.2
18.9
Dominant Cell Type
a GE & S
a LE
a S
MaxQuantMBR LE Image
18.84 17.87 19.48 19.27
18.78 ND 17.61 19.49
18.92 18.54 ND 19.39
19.90 18.16 ND 18.88
19.13 19.51 ND 18.32
20.38 18.74 17.74 18.19
Expression Level
20.0
19.5
19.0
18.5
18.0
Dominant Cell Type
a GE & S
a LE
a S

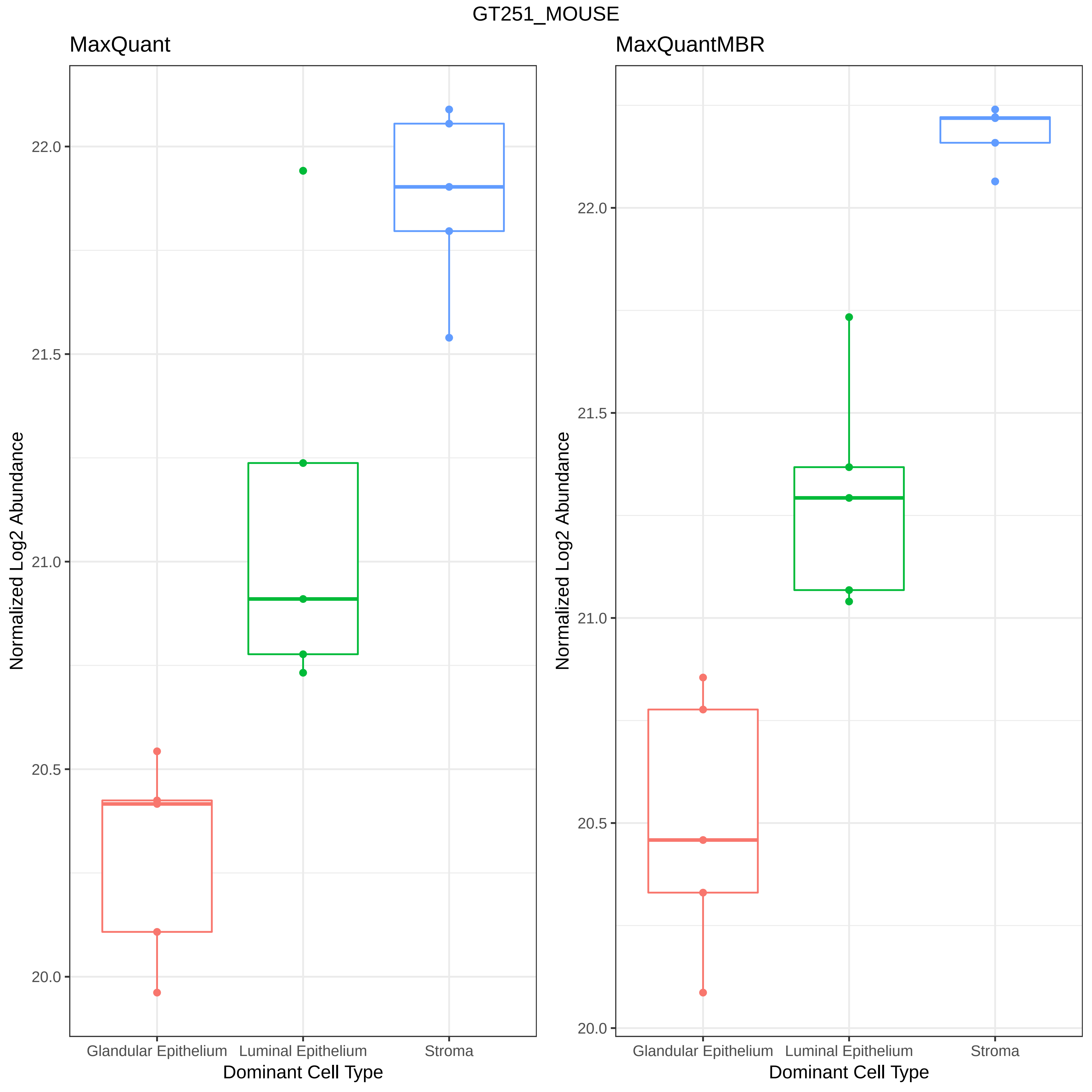

GT251_MOUSE
MaxQuant
MaxQuantMBR
Normalized Log2 Abundance
22.0
21.5
21.0
20.5
20.0
Glandular Epithelium
Luminal Epithelium
Stroma
Dominant Cell Type

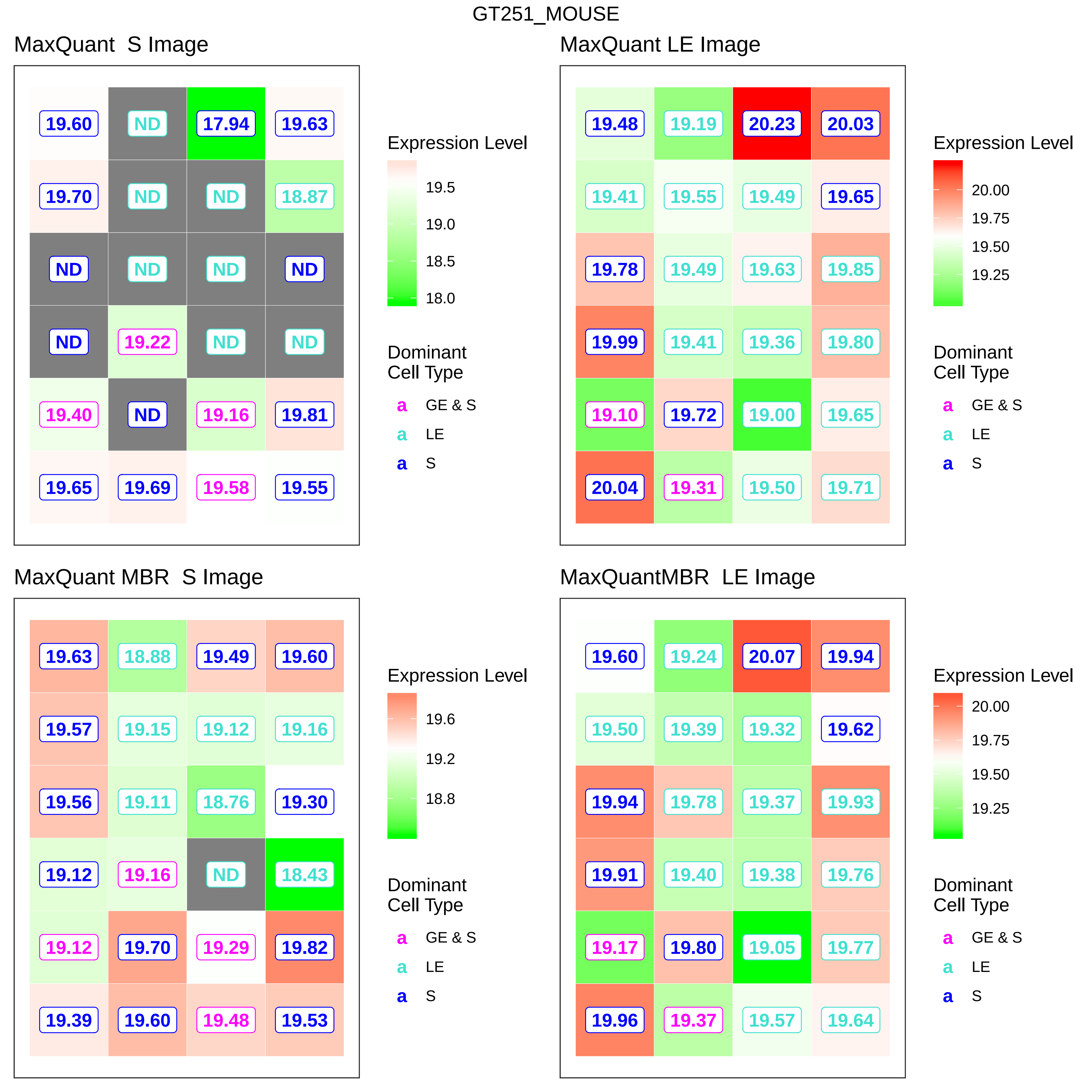

GT251_MOUSE
MaxQuant S Image
19.60 ND 17.94 19.63
19.70 ND ND 18.87
ND ND ND ND
ND 19.22 ND ND
19.40 ND 19.16 19.81
19.65 19.69 19.58 19.55
Expression Level
19.5
19.0
18.5
18.0
Dominant Cell Type
a GE & S
a LE
a S
MaxQuant LE Image
19.48 19.19 20.23 20.03
19.41 19.55 19.49 19.65
19.78 19.49 19.63 19.85
19.99 19.41 19.36 19.80
19.10 19.72 19.00 19.65
20.04 19.31 19.50 19.71
Expression Level
20.00
19.75
19.50
19.25
Dominant Cell Type
a GE & S
a LE
a S
MaxQuant MBR S Image
19.63 18.88 19.49 19.60
19.57 19.15 19.12 19.16
19.56 19.11 18.76 19.30
19.12 19.16 ND 18.43
19.12 19.70 19.29 19.82
19.39 19.60 19.48 19.53
Expression Level
19.6
19.2
18.8
Dominant Cell Type
a GE & S
a LE
a S
MaxQuantMBR LE Image
19.60 19.24 20.07 19.94
19.50 19.39 19.32 19.62
19.94 19.78 19.37 19.93
19.91 19.40 19.38 19.76
19.17 19.80 19.05 19.77
19.96 19.37 19.57 19.64
Expression Level
20.00
19.75
19.50
19.25
Dominant Cell Type
a GE & S
a LE
a S

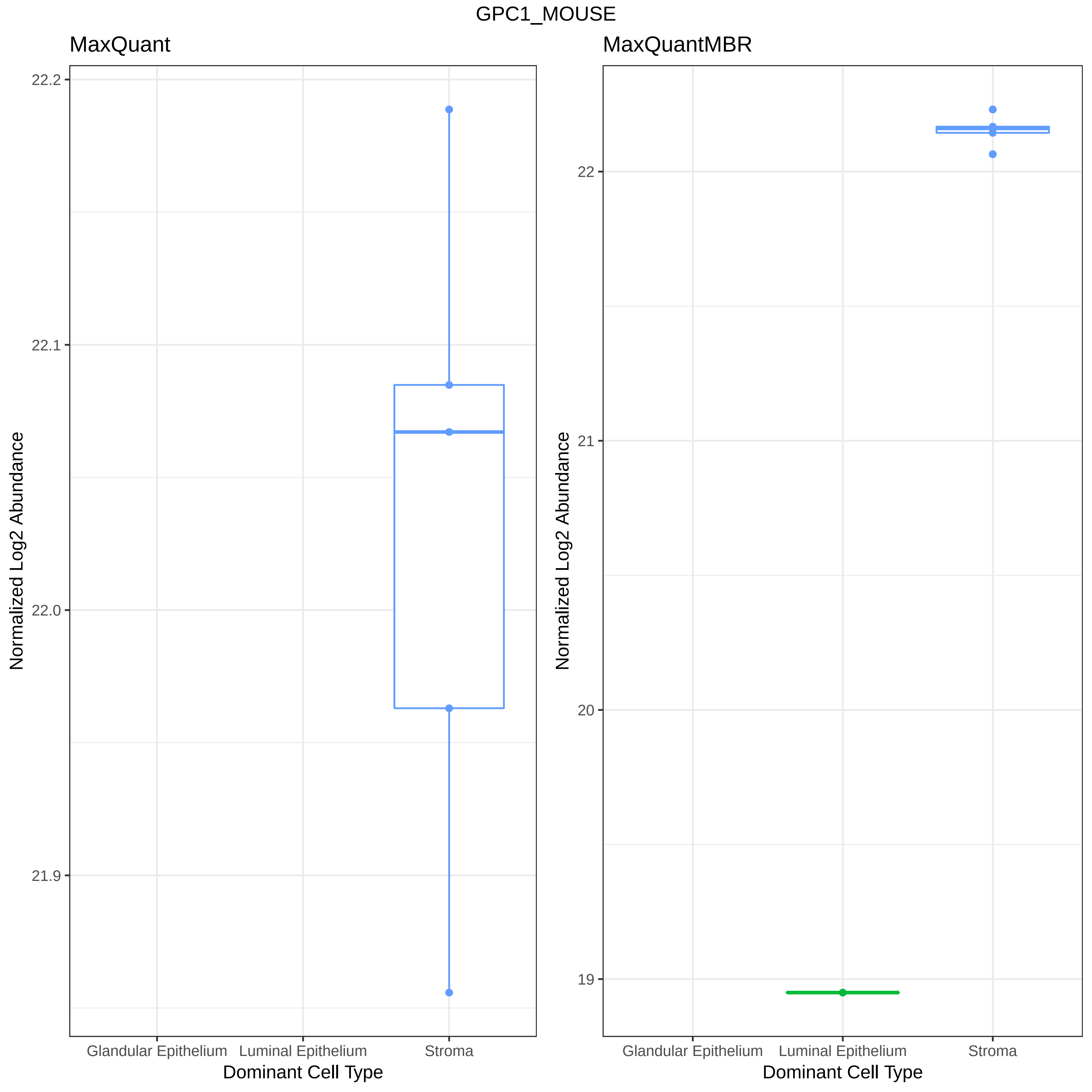

GPC1_MOUSE
MaxQuant
MaxQuantMBR
Normalized Log2 Abundance
22.2
22.1
22.0
21.9
22
21
20
19
Glandular Epithelium
Luminal Epithelium
Stroma
Dominant Cell Type

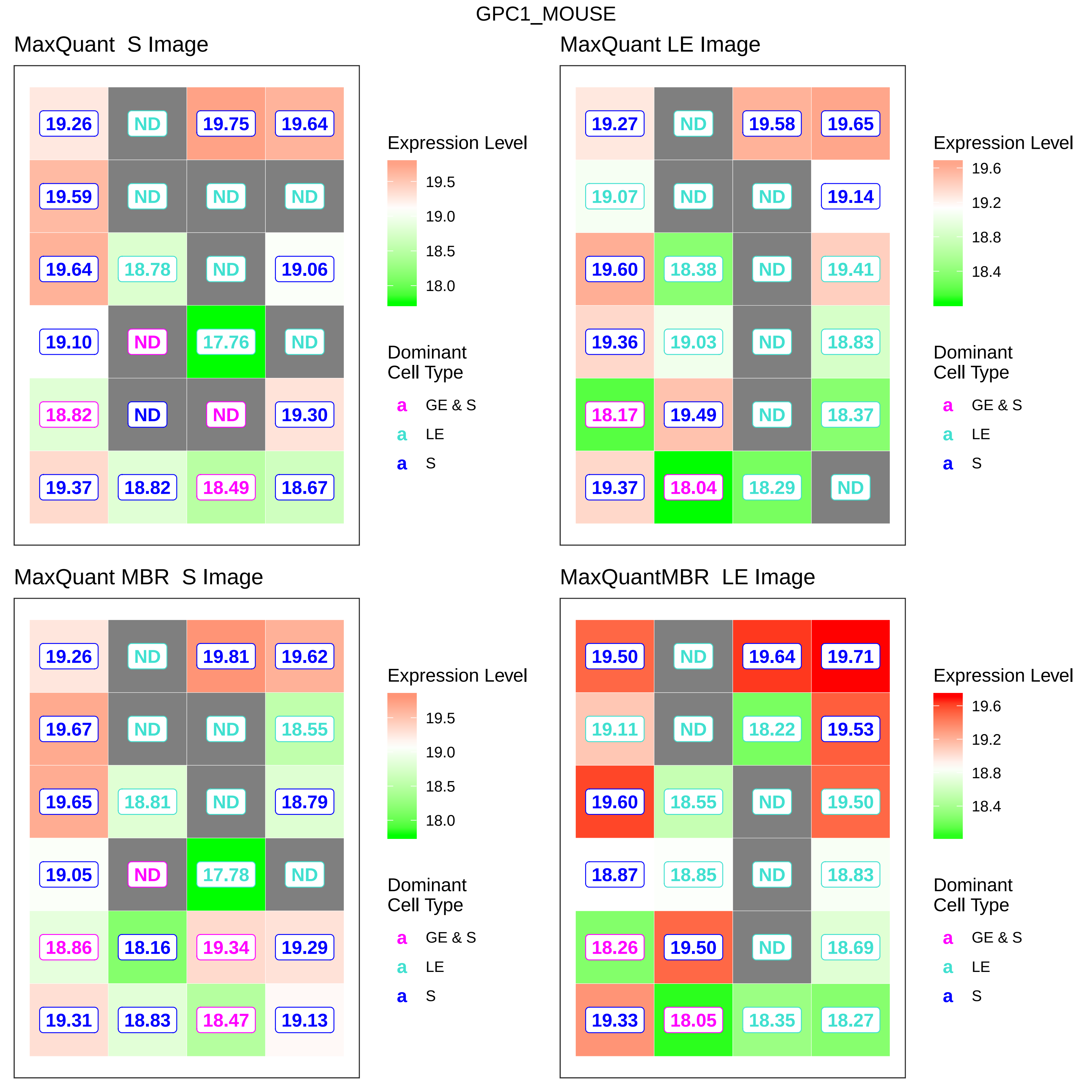
GPC1_MOUSE
MaxQuant S Image
19.26 ND 19.75 19.64
19.59 ND ND ND
19.64 18.78 ND 19.06
19.10 ND 17.76 ND
18.82 ND ND 19.30
19.37 18.82 18.49 18.67
Expression Level
19.5
19.0
18.5
18.0
Dominant Cell Type
a GE & S
a LE
a S
MaxQuant LE Image
19.27 ND 19.58 19.65
19.07 ND ND 19.14
19.60 18.38 ND 19.41
19.36 19.03 ND 18.83
18.17 19.49 ND 18.37
19.37 18.04 18.29 ND
Expression Level
19.6
19.2
18.8
18.4
Dominant Cell Type
a GE & S
a LE
a S
MaxQuant MBR S Image
19.26 ND 19.81 19.62
19.67 ND ND 18.55
19.65 18.81 ND 18.79
19.05 ND 17.78 ND
18.86 18.16 19.34 19.29
19.31 18.83 18.47 19.13
Expression Level
19.5
19.0
18.5
18.0
Dominant Cell Type
a GE & S
a LE
a S
MaxQuantMBR LE Image
19.50 ND 19.64 19.71
19.11 ND 18.22 19.53
19.60 18.55 ND 19.50
18.87 18.85 ND 18.83
18.26 19.50 ND 18.69
19.33 18.05 18.35 18.27
Expression Level
19.6
19.2
18.8
18.4
Dominant Cell Type
a GE & S
a LE
a S

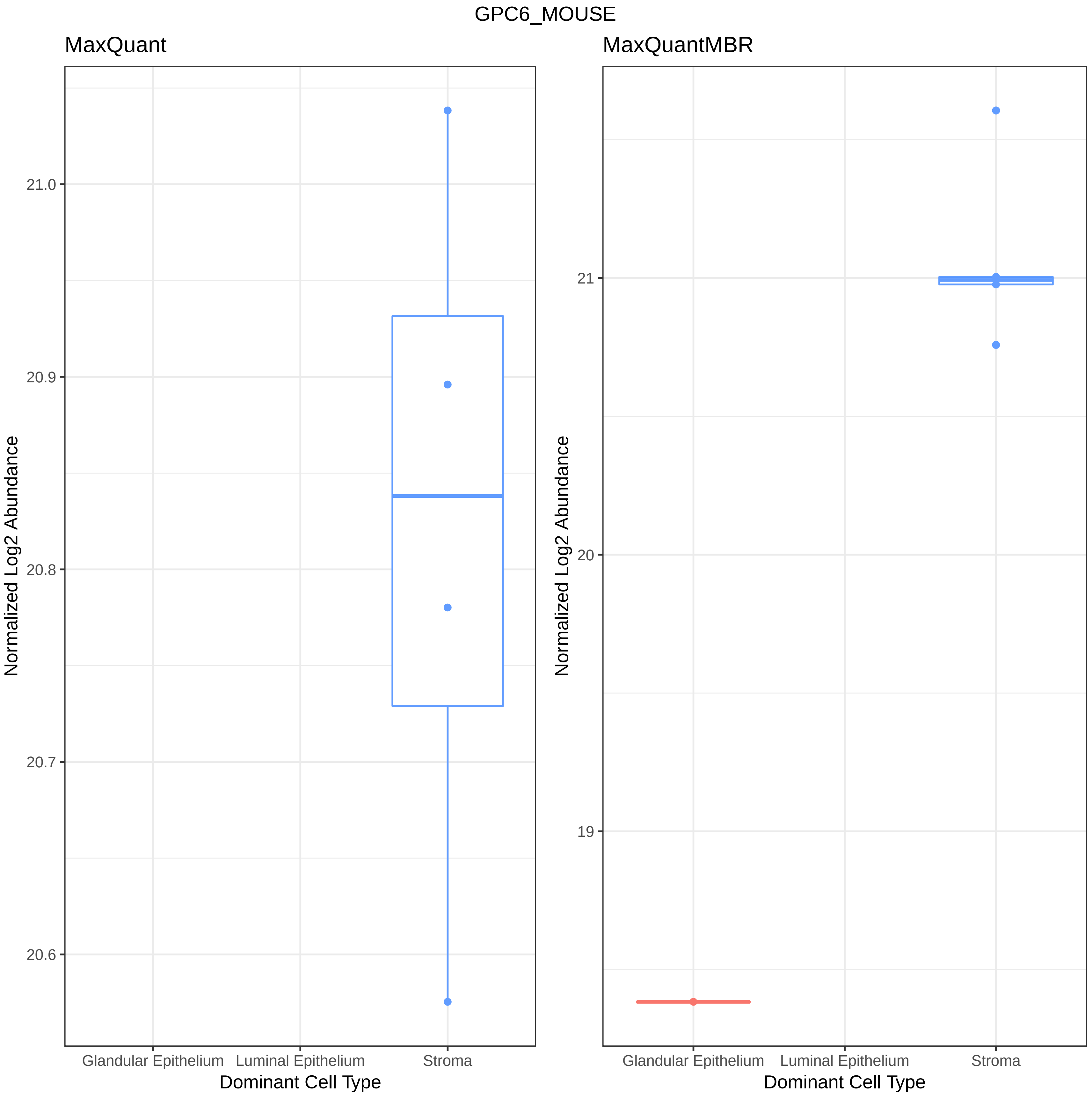
GPC6_MOUSE
MaxQuant
MaxQuantMBR
Normalized Log2 Abundance
21.0
20.9
20.8
20.7
20.6
21
20
19
Glandular Epithelium
Luminal Epithelium
Stroma
Dominant Cell Type

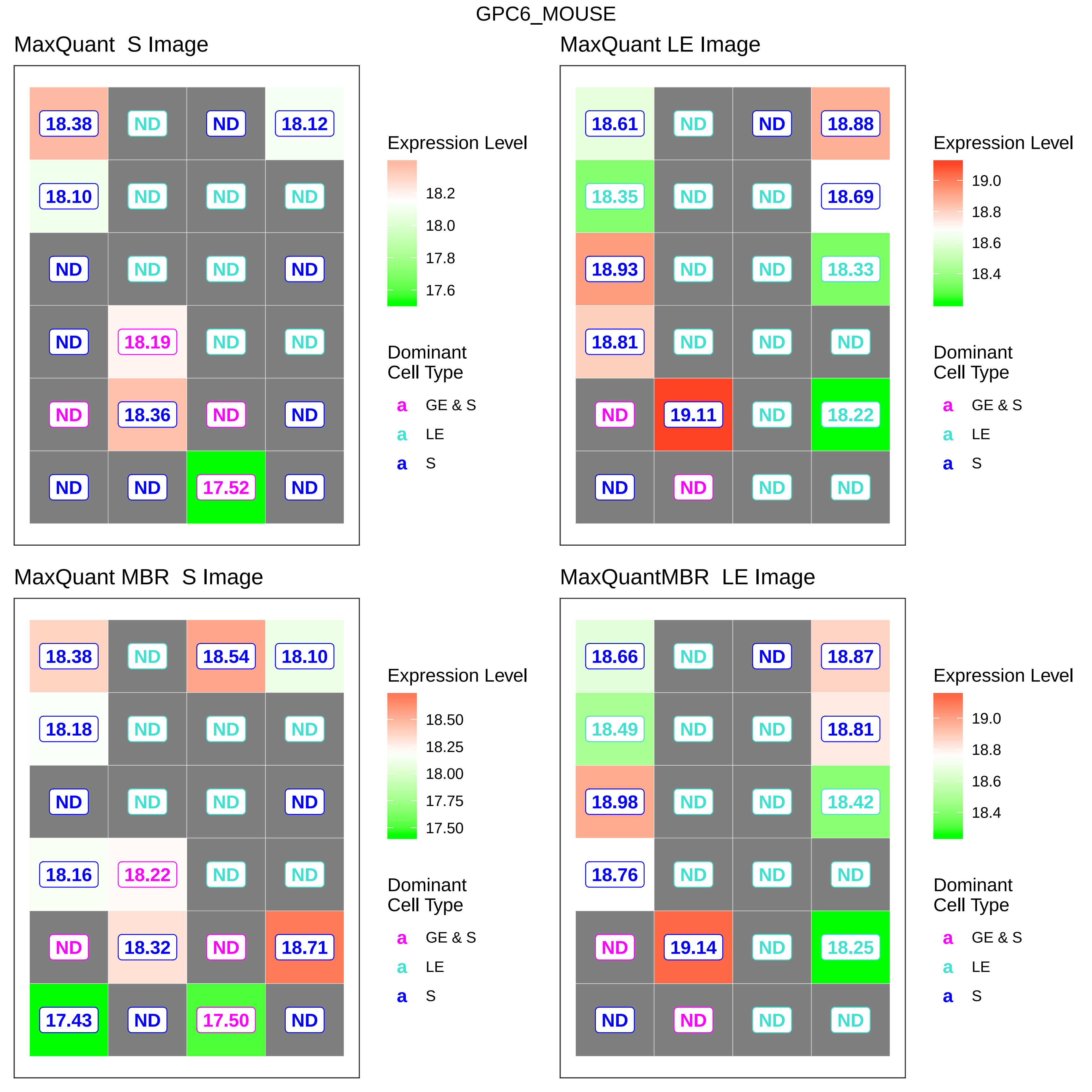

GPC6_MOUSE
MaxQuant S Image
18.38 ND ND 18.12
18.10 ND ND ND
ND ND ND ND
ND 18.19 ND ND
ND 18.36 ND ND
ND ND 17.52 ND
Expression Level
18.2
18.0
17.8
17.6
Dominant Cell Type
a GE & S
a LE
a S
MaxQuant LE Image
18.61 ND ND 18.88
18.35 ND ND 18.69
18.93 ND ND 18.33
18.81 ND ND ND
ND 19.11 ND 18.22
ND ND ND ND
Expression Level
19.0
18.8
18.6
18.4
Dominant Cell Type
a GE & S
a LE
a S
MaxQuant MBR S Image
18.38 ND 18.54 18.10
18.18 ND ND ND
ND ND ND ND
18.16 18.22 ND ND
ND 18.32 ND 18.71
17.43 ND 17.50 ND
Expression Level
18.50
18.25
18.00
17.75
17.50
Dominant Cell Type
a GE & S
a LE
a S
MaxQuantMBR LE Image
18.66 ND ND 18.87
18.49 ND ND 18.81
18.98 ND ND 18.42
18.76 ND ND ND
ND 19.14 ND 18.25
ND ND ND ND
Expression Level
19.0
18.8
18.6
18.4
Dominant Cell Type
a GE & S
a LE
a S

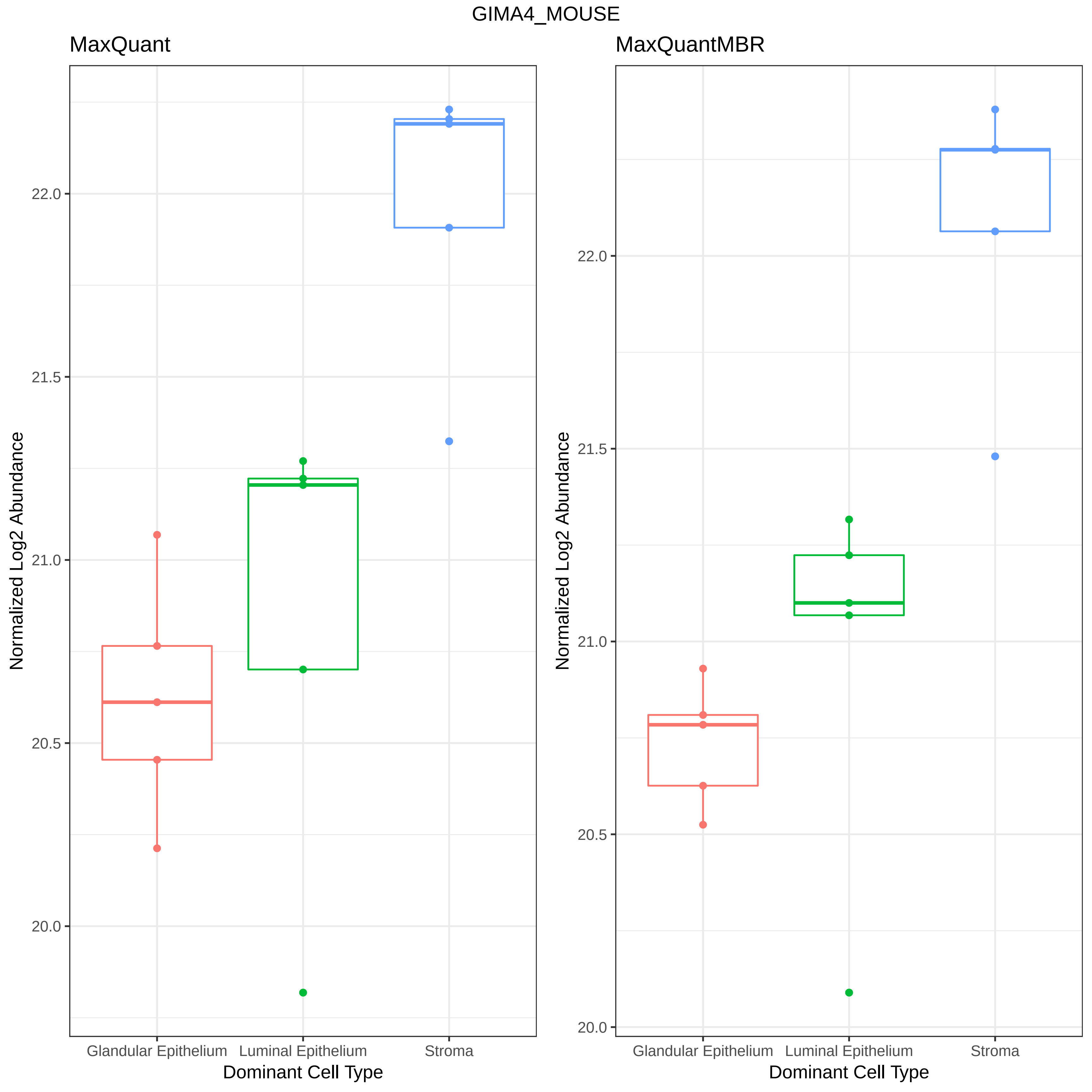

GIMA4_MOUSE
MaxQuant
MaxQuantMBR
Normalized Log2 Abundance
Dominant Cell Type
Glandular Epithelium
Luminal Epithelium
Stroma
20.0
20.5
21.0
21.5
22.0

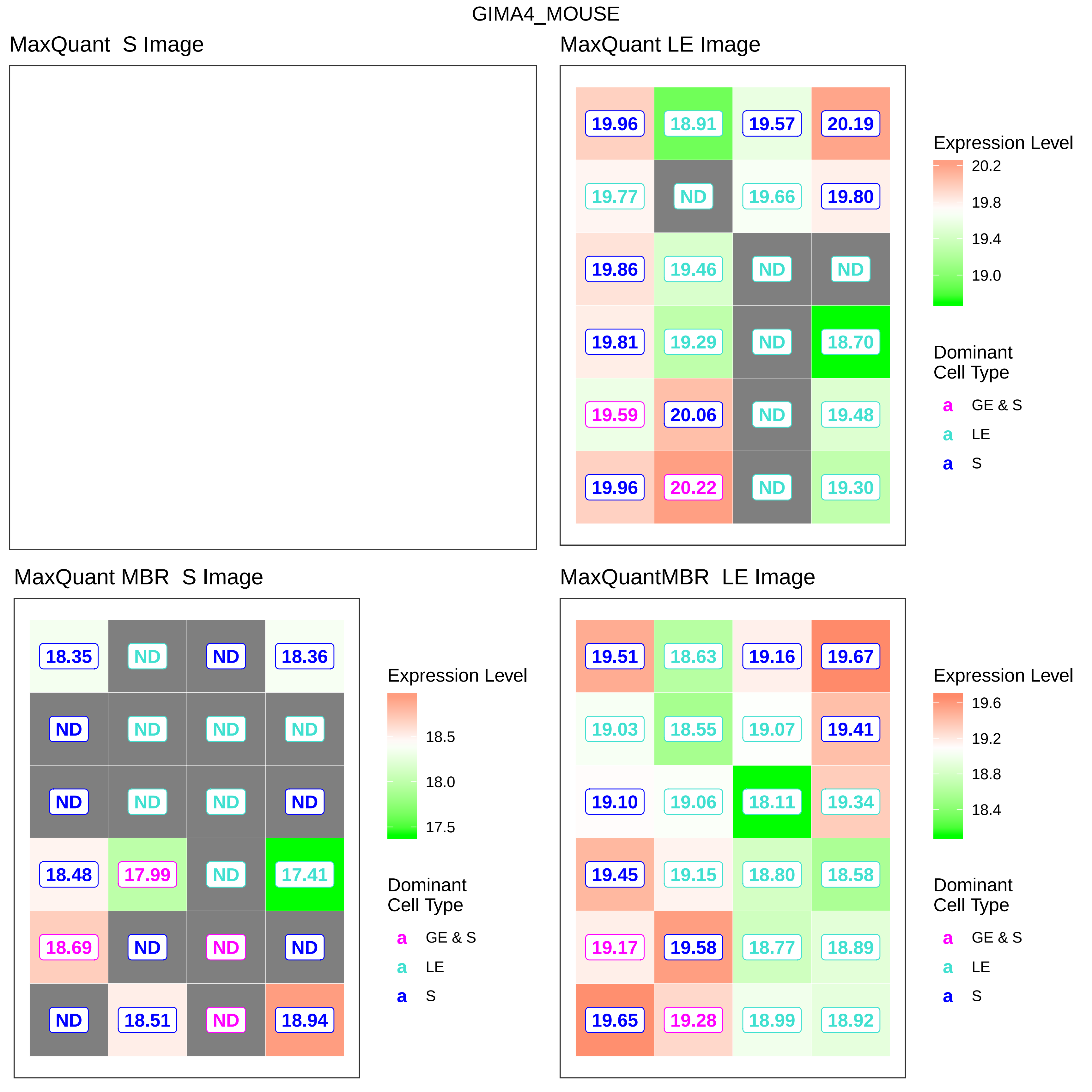
GIMA4_MOUSE
MaxQuant S Image
MaxQuant LE Image
19.96 18.91 19.57 20.19
19.77 ND 19.66 19.80
19.86 19.46 ND ND
19.81 19.29 ND 18.70
19.59 20.06 ND 19.48
19.96 20.22 ND 19.30
Expression Level
20.2
19.8
19.4
19.0
Dominant Cell Type
a GE & S
a LE
a S
MaxQuant MBR S Image
18.35 ND ND 18.36
ND ND ND ND
ND ND ND ND
18.48 17.99 ND 17.41
18.69 ND ND ND
ND 18.51 ND 18.94
Expression Level
18.5
18.0
17.5
Dominant Cell Type
a GE & S
a LE
a S
MaxQuantMBR LE Image
19.51 18.63 19.16 19.67
19.03 18.55 19.07 19.41
19.10 19.06 18.11 19.34
19.45 19.15 18.80 18.58
19.17 19.58 18.77 18.89
19.65 19.28 18.99 18.92
Expression Level
19.6
19.2
18.8
18.4
Dominant Cell Type
a GE & S
a LE
a S

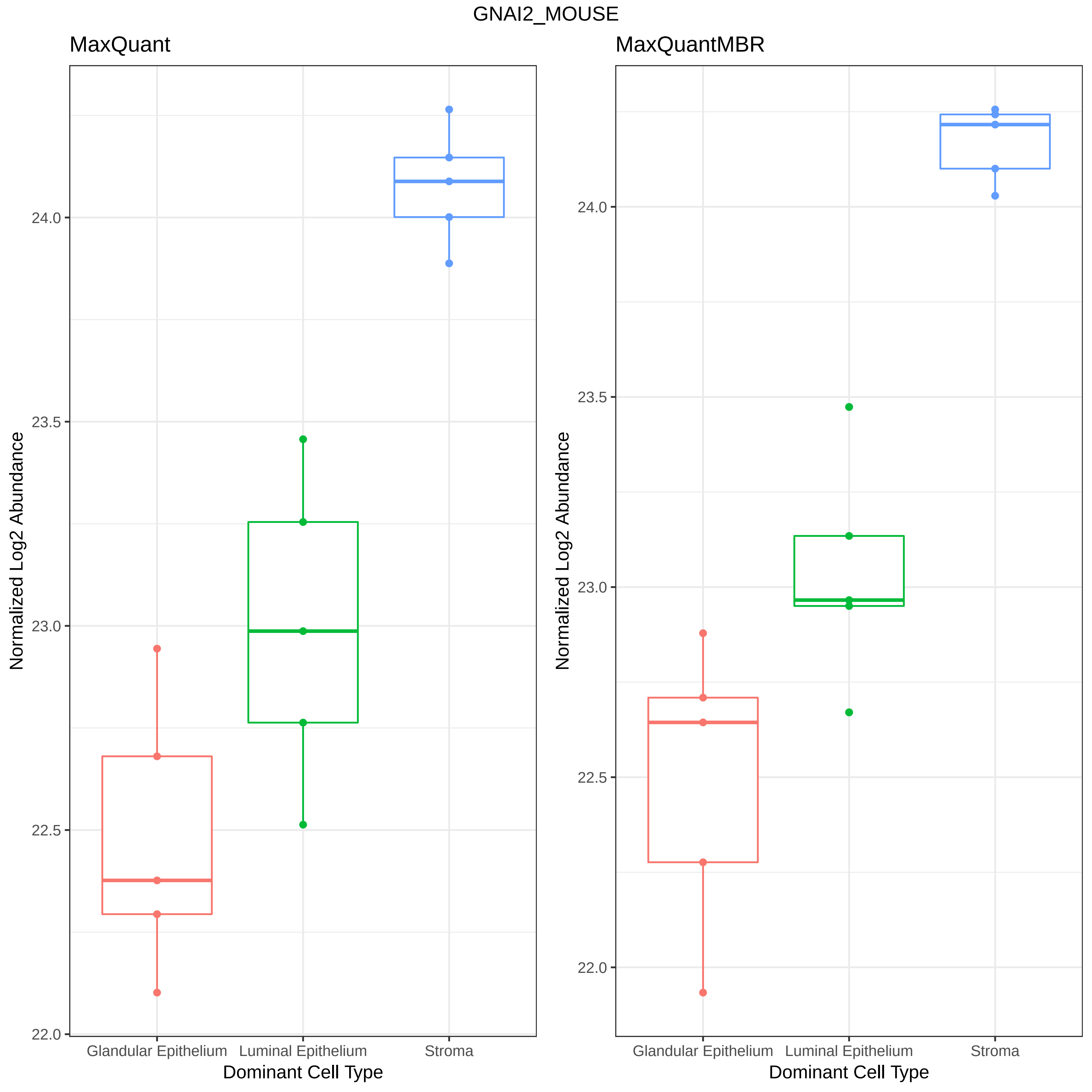
GNAI2_MOUSE
MaxQuant
MaxQuantMBR
Normalized Log2 Abundance
24.0
23.5
23.0
22.5
22.0
Glandular Epithelium
Luminal Epithelium
Stroma
Dominant Cell Type

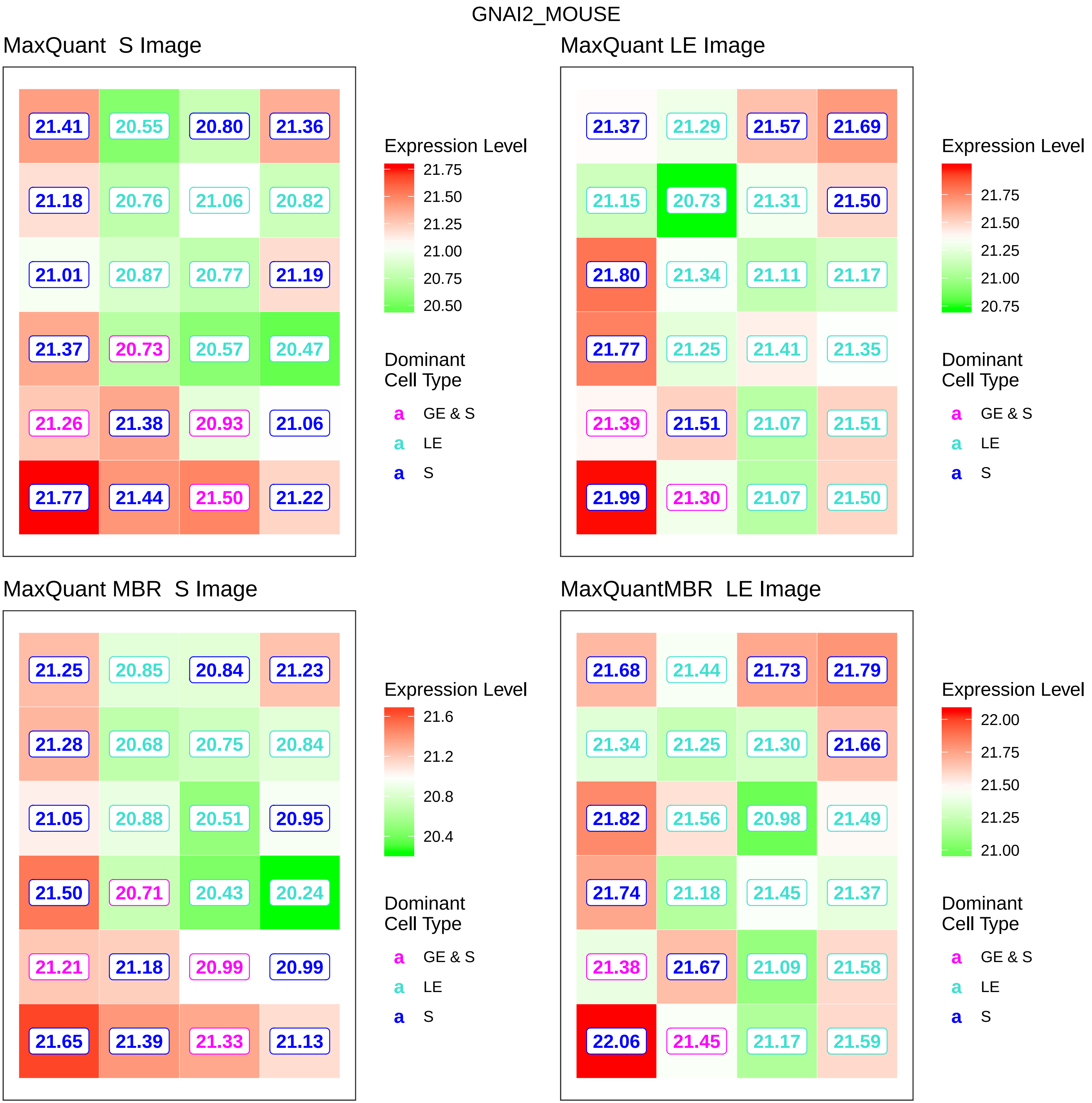

GNAI2_MOUSE
MaxQuant S Image
21.41 20.55 20.80 21.36
21.18 20.76 21.06 20.82
21.01 20.87 20.77 21.19
21.37 20.73 20.57 20.47
21.26 21.38 20.93 21.06
21.77 21.44 21.50 21.22
Expression Level
21.75
21.50
21.25
21.00
20.75
20.50
Dominant Cell Type
a GE & S
a LE
a S
MaxQuant LE Image
21.37 21.29 21.57 21.69
21.15 20.73 21.31 21.50
21.80 21.34 21.11 21.17
21.77 21.25 21.41 21.35
21.39 21.51 21.07 21.51
21.99 21.30 21.07 21.50
Expression Level
21.75
21.50
21.25
21.00
20.75
Dominant Cell Type
a GE & S
a LE
a S
MaxQuant MBR S Image
21.25 20.85 20.84 21.23
21.28 20.68 20.75 20.84
21.05 20.88 20.51 20.95
21.50 20.71 20.43 20.24
21.21 21.18 20.99 20.99
21.65 21.39 21.33 21.13
Expression Level
21.6
21.2
20.8
20.4
Dominant Cell Type
a GE & S
a LE
a S
MaxQuantMBR LE Image
21.68 21.44 21.73 21.79
21.34 21.25 21.30 21.66
21.82 21.56 20.98 21.49
21.74 21.18 21.45 21.37
21.38 21.67 21.09 21.58
22.06 21.45 21.17 21.59
Expression Level
22.00
21.75
21.50
21.25
21.00
Dominant Cell Type
a GE & S
a LE
a S

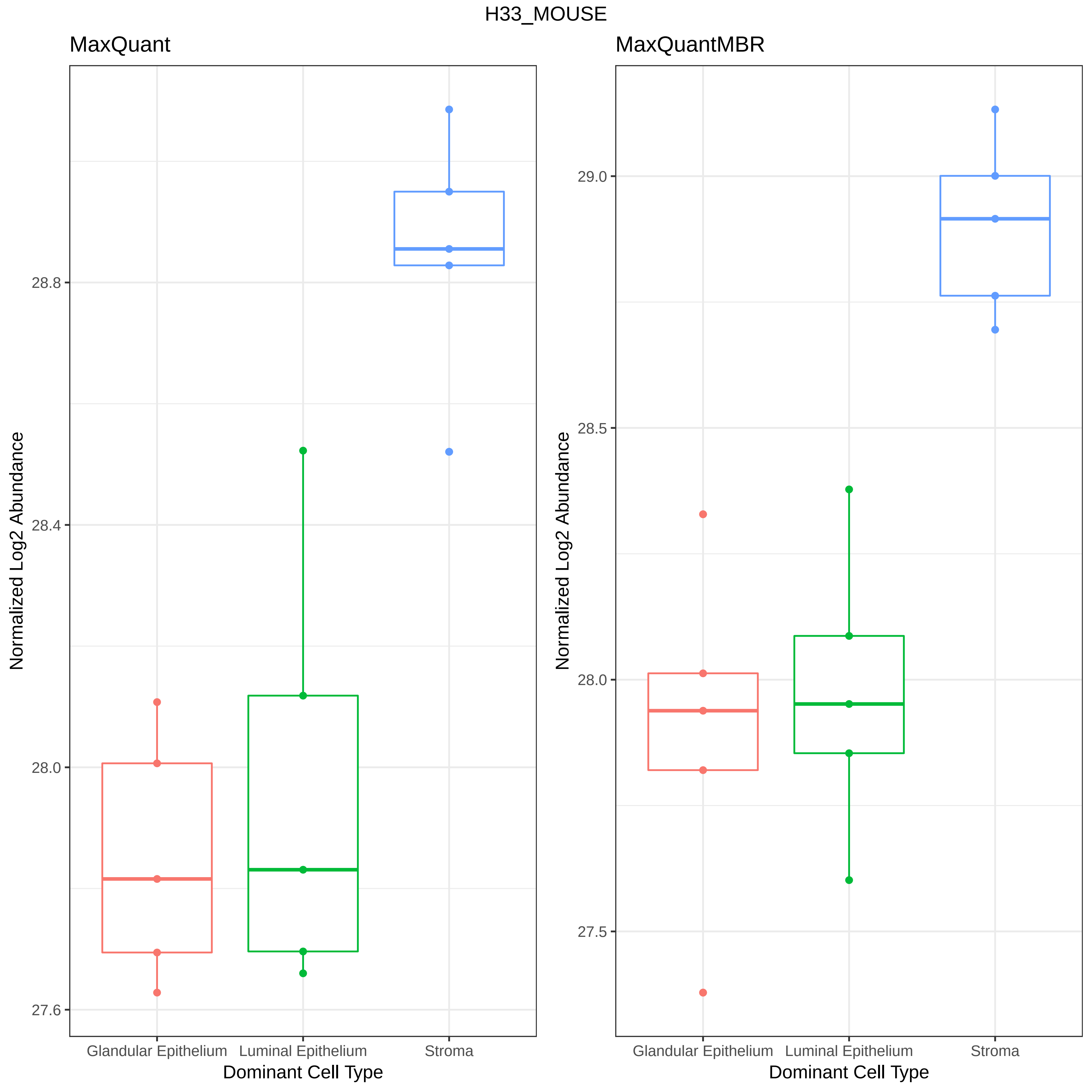
H33_MOUSE
MaxQuant
MaxQuantMBR
Normalized Log2 Abundance
28.8
28.4
28.0
27.6
29.0
28.5
28.0
27.5
Glandular Epithelium
Luminal Epithelium
Stroma
Dominant Cell Type

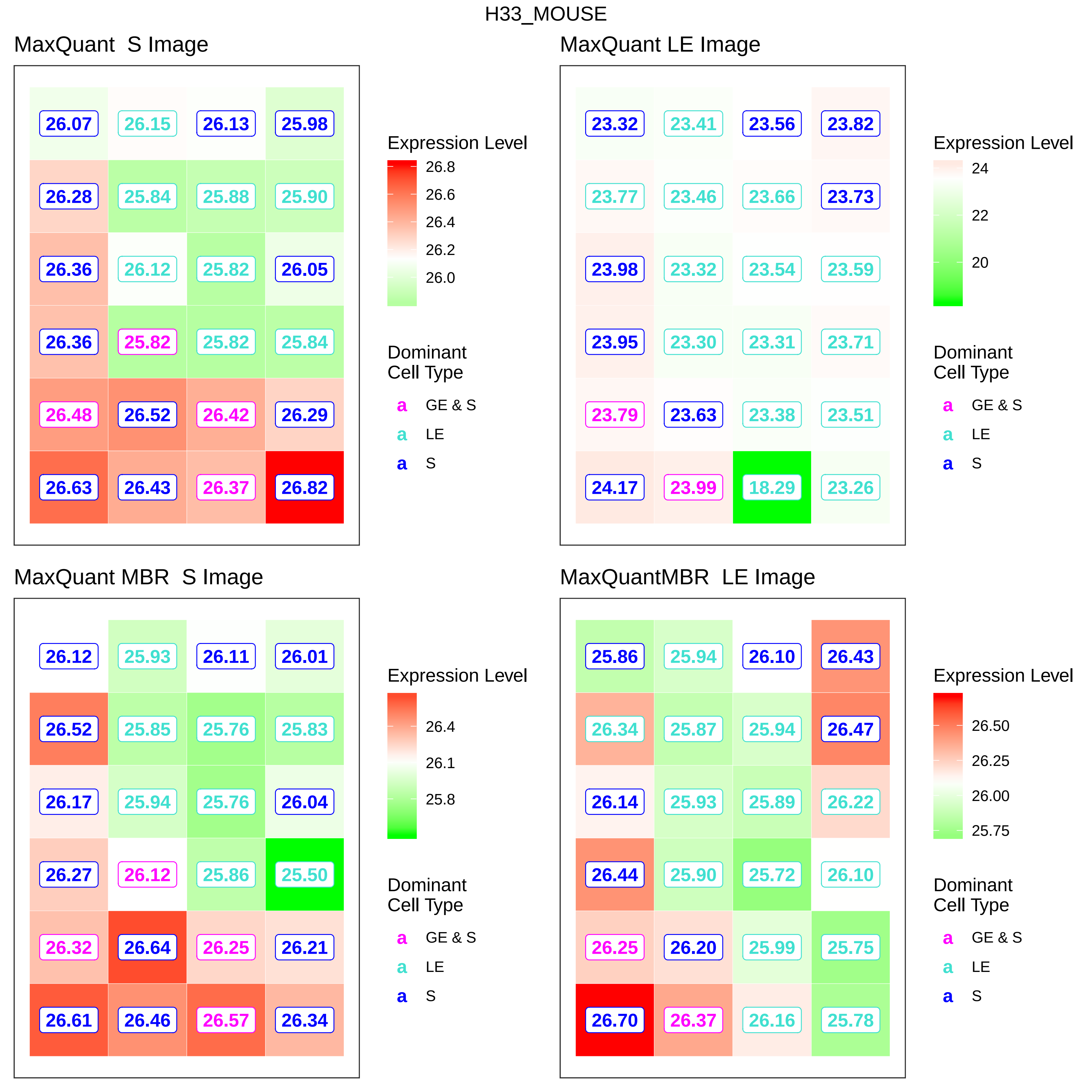
H33_MOUSE
MaxQuant S Image
26.07 26.15 26.13 25.98
26.28 25.84 25.88 25.90
26.36 26.12 25.82 26.05
26.36 25.82 25.82 25.84
26.48 26.52 26.42 26.29
26.63 26.43 26.37 26.82
Expression Level
26.8
26.6
26.4
26.2
26.0
Dominant Cell Type
a GE & S
a LE
a S
MaxQuant LE Image
23.32 23.41 23.56 23.82
23.77 23.46 23.66 23.73
23.98 23.32 23.54 23.59
23.95 23.30 23.31 23.71
23.79 23.63 23.38 23.51
24.17 23.99 18.29 23.26
Expression Level
24
22
20
Dominant Cell Type
a GE & S
a LE
a S
MaxQuant MBR S Image
26.12 25.93 26.11 26.01
26.52 25.85 25.76 25.83
26.17 25.94 25.76 26.04
26.27 26.12 25.86 25.50
26.32 26.64 26.25 26.21
26.61 26.46 26.57 26.34
Expression Level
26.4
26.1
25.8
Dominant Cell Type
a GE & S
a LE
a S
MaxQuantMBR LE Image
25.86 25.94 26.10 26.43
26.34 25.87 25.94 26.47
26.14 25.93 25.89 26.22
26.44 25.90 25.72 26.10
26.25 26.20 25.99 25.75
26.70 26.37 26.16 25.78
Expression Level
26.50
26.25
26.00
25.75
Dominant Cell Type
a GE & S
a LE
a S

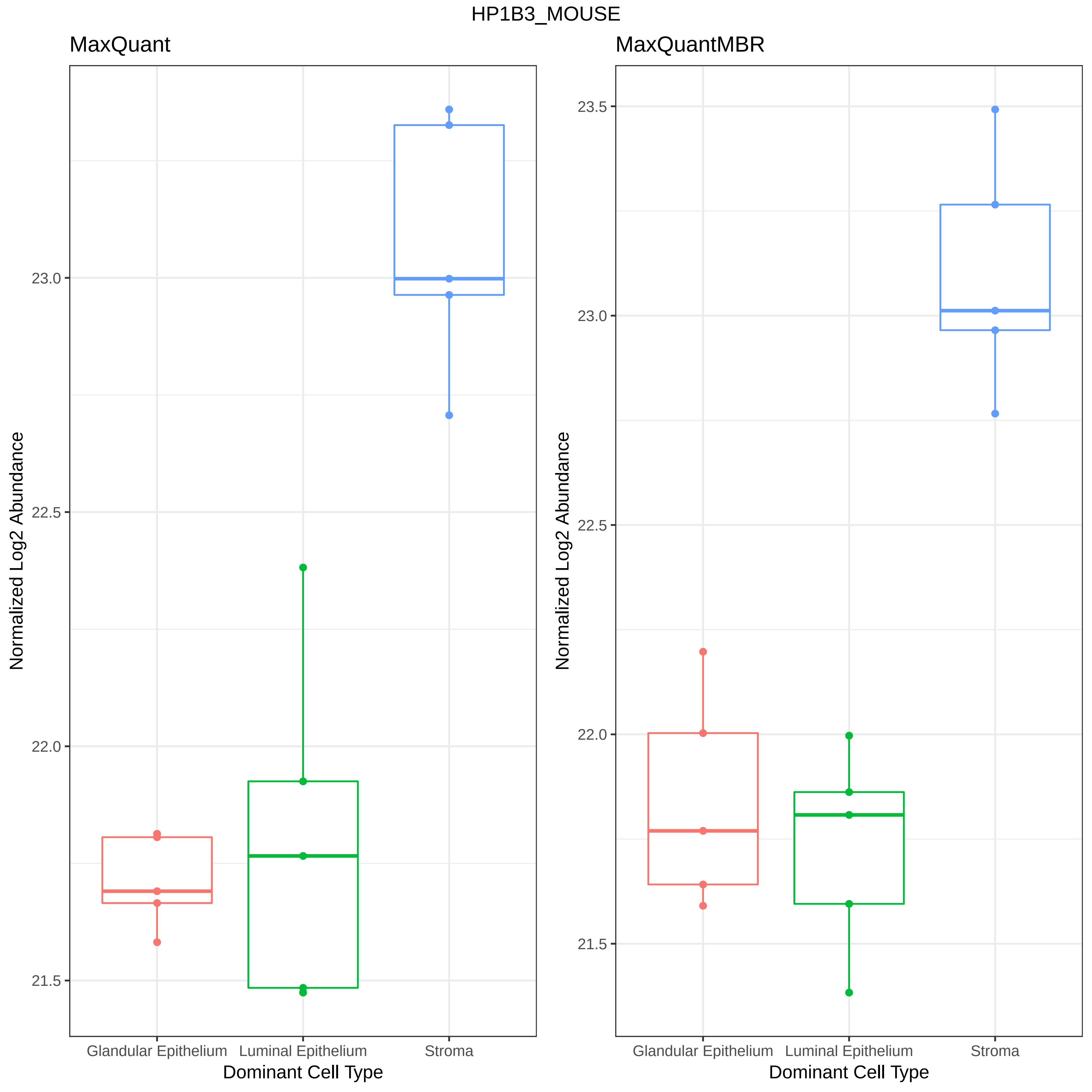

HP1B3_MOUSE
MaxQuant
MaxQuantMBR
Normalized Log2 Abundance
23.0
22.5
22.0
21.5
23.5
Glandular Epithelium
Luminal Epithelium
Stroma
Dominant Cell Type

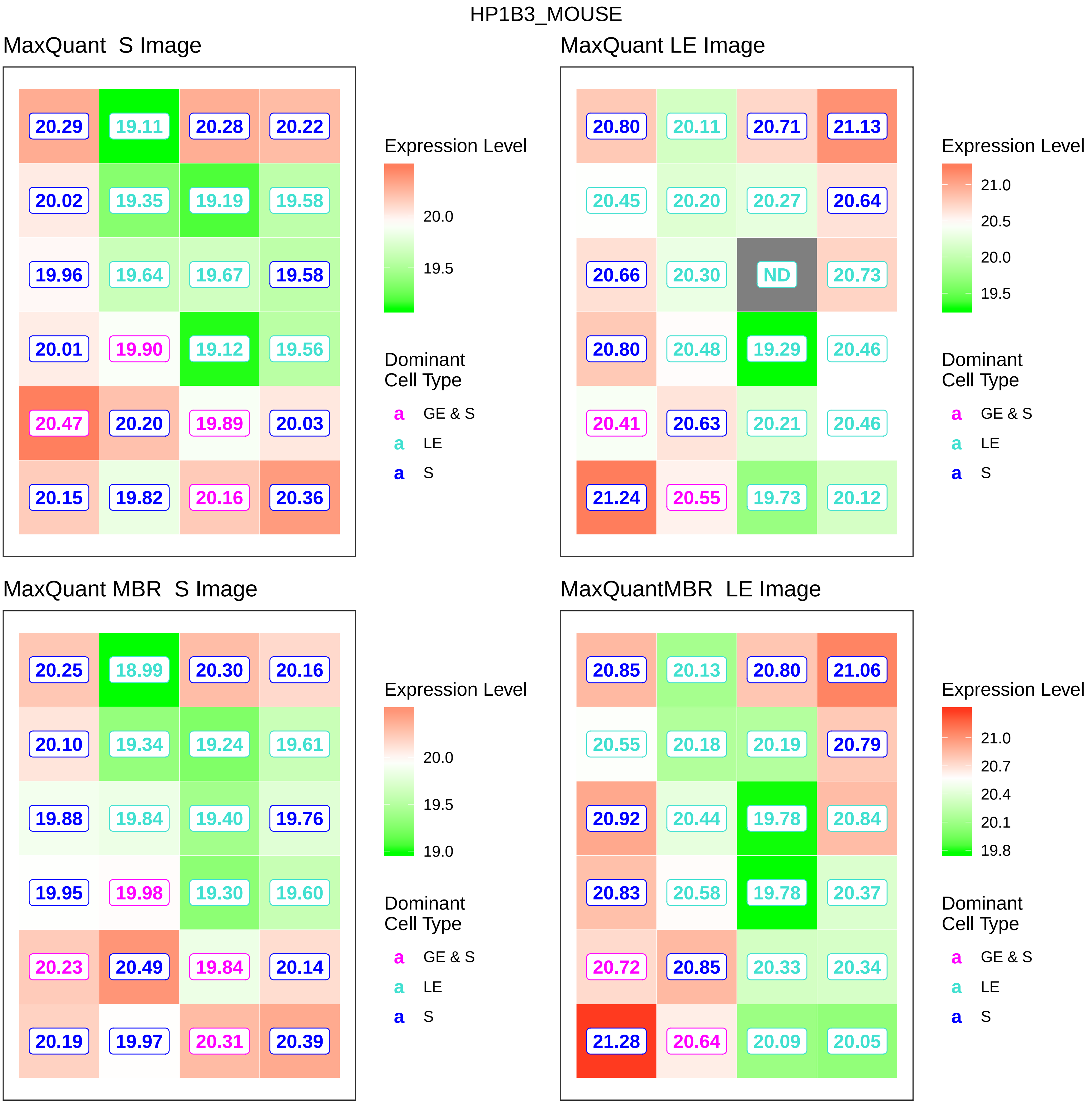
HP1B3_MOUSE
MaxQuant S Image
20.29 19.11 20.28 20.22
20.02 19.35 19.19 19.58
19.96 19.64 19.67 19.58
20.01 19.90 19.12 19.56
20.47 20.20 19.89 20.03
20.15 19.82 20.16 20.36
Expression Level
20.0
19.5
Dominant Cell Type
a GE & S
a LE
a S
MaxQuant LE Image
20.80 20.11 20.71 21.13
20.45 20.20 20.27 20.64
20.66 20.30 ND 20.73
20.80 20.48 19.29 20.46
20.41 20.63 20.21 20.46
21.24 20.55 19.73 20.12
Expression Level
21.0
20.5
20.0
19.5
Dominant Cell Type
a GE & S
a LE
a S
MaxQuant MBR S Image
20.25 18.99 20.30 20.16
20.10 19.34 19.24 19.61
19.88 19.84 19.40 19.76
19.95 19.98 19.30 19.60
20.23 20.49 19.84 20.14
20.19 19.97 20.31 20.39
Expression Level
20.0
19.5
19.0
Dominant Cell Type
a GE & S
a LE
a S
MaxQuantMBR LE Image
20.85 20.13 20.80 21.06
20.55 20.18 20.19 20.79
20.92 20.44 19.78 20.84
20.83 20.58 19.78 20.37
20.72 20.85 20.33 20.34
21.28 20.64 20.09 20.05
Expression Level
21.0
20.7
20.4
20.1
19.8
Dominant Cell Type
a GE & S
a LE
a S

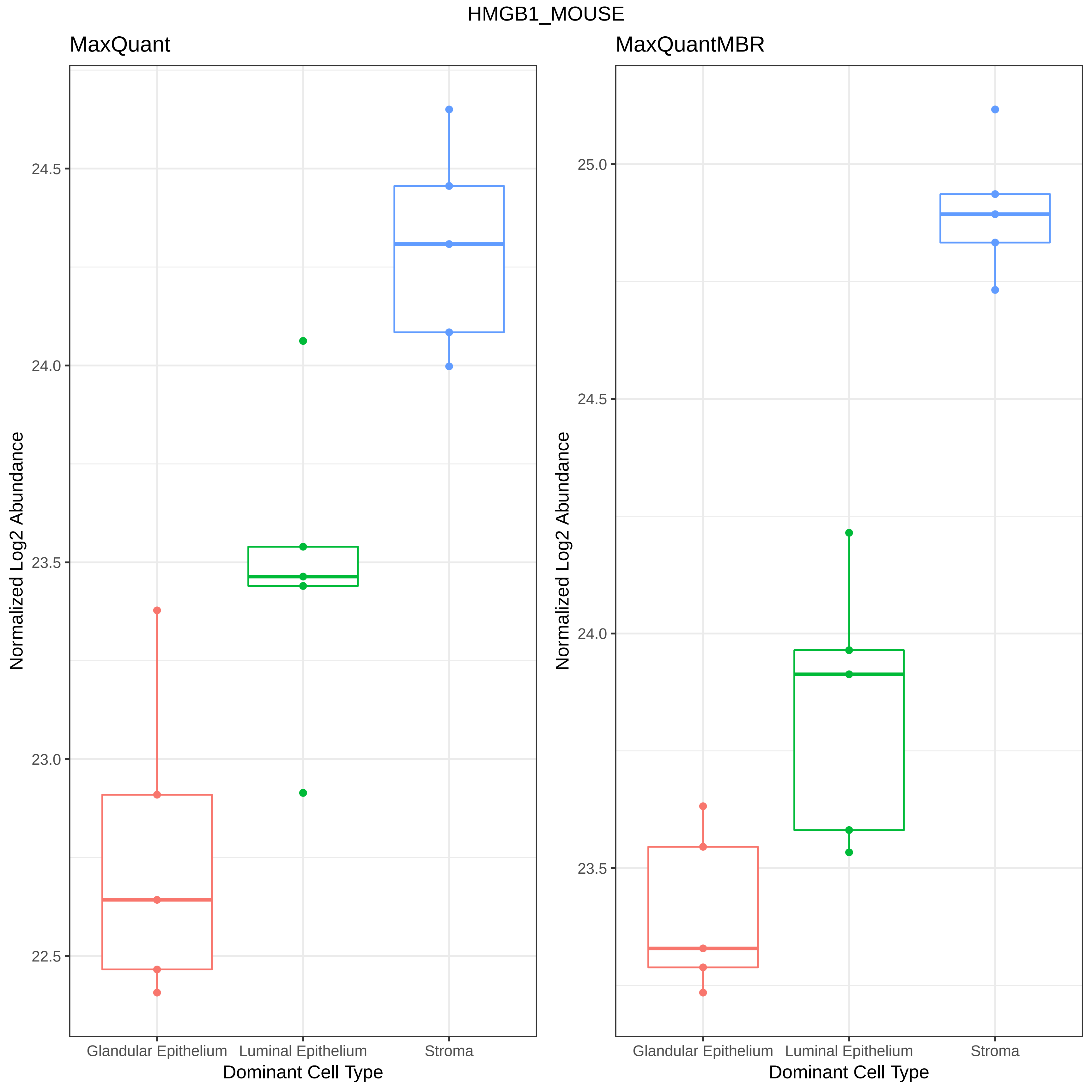
HMGB1_MOUSE
MaxQuant
MaxQuantMBR
Normalized Log2 Abundance
24.5
24.0
23.5
23.0
22.5
25.0
Glandular Epithelium
Luminal Epithelium
Stroma
Dominant Cell Type

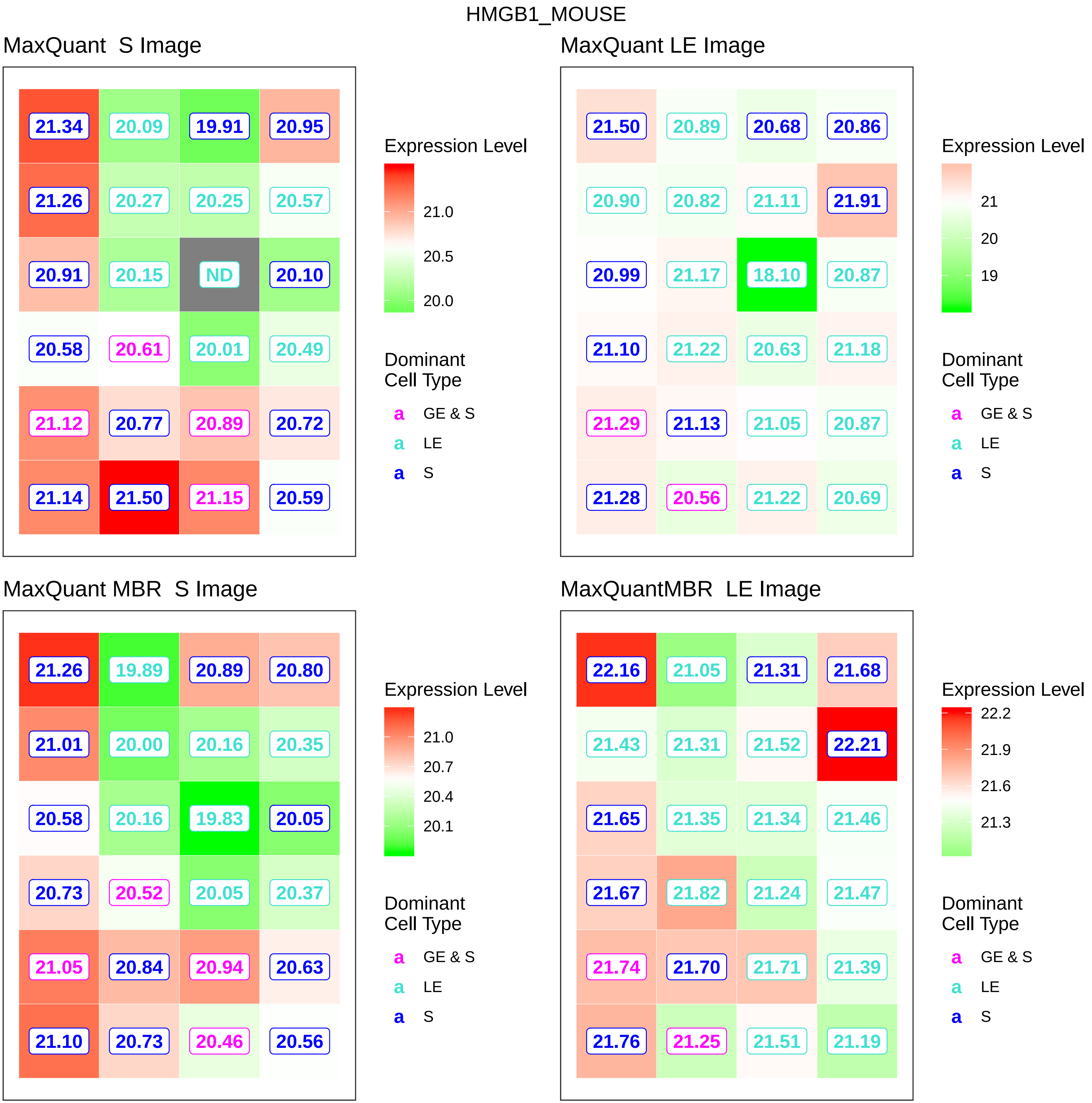
HMGB1_MOUSE
MaxQuant S Image
21.34
20.09
19.91
20.95
21.26
20.27
20.25
20.57
20.91
20.15
ND
20.10
20.58
20.61
20.01
20.49
21.12
20.77
20.89
20.72
21.14
21.50
21.15
20.59
Expression Level
21.0
20.5
20.0
Dominant Cell Type
a GE & S
a LE
a S
MaxQuant LE Image
21.50
20.89
20.68
20.86
20.90
20.82
21.11
21.91
20.99
21.17
18.10
20.87
21.10
21.22
20.63
21.18
21.29
21.13
21.05
20.87
21.28
20.56
21.22
20.69
Expression Level
21
20
19
Dominant Cell Type
a GE & S
a LE
a S
MaxQuant MBR S Image
21.26
19.89
20.89
20.80
21.01
20.00
20.16
20.35
20.58
20.16
19.83
20.05
20.73
20.52
20.05
20.37
21.05
20.84
20.94
20.63
21.10
20.73
20.46
20.56
Expression Level
21.0
20.7
20.4
20.1
Dominant Cell Type
a GE & S
a LE
a S
MaxQuantMBR LE Image
22.16
21.05
21.31
21.68
21.43
21.31
21.52
22.21
21.65
21.35
21.34
21.46
21.67
21.82
21.24
21.47
21.74
21.70
21.71
21.39
21.76
21.25
21.51
21.19
Expression Level
22.2
21.9
21.6
21.3
Dominant Cell Type
a GE & S
a LE
a S

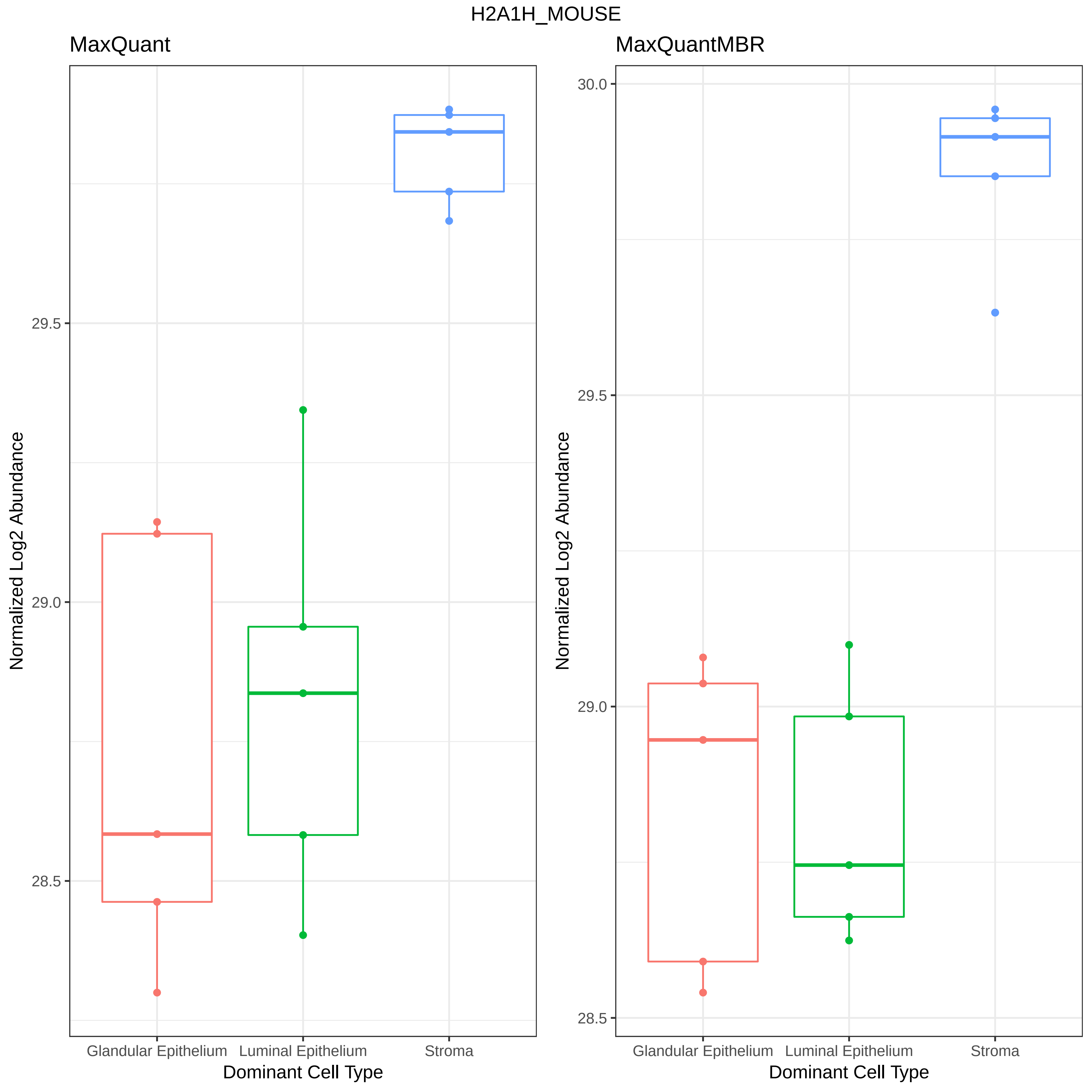
H2A1H_MOUSE
MaxQuant
MaxQuantMBR
29.5
29.0
28.5
30.0
Normalized Log2 Abundance
Glandular Epithelium
Luminal Epithelium
Stroma
Dominant Cell Type

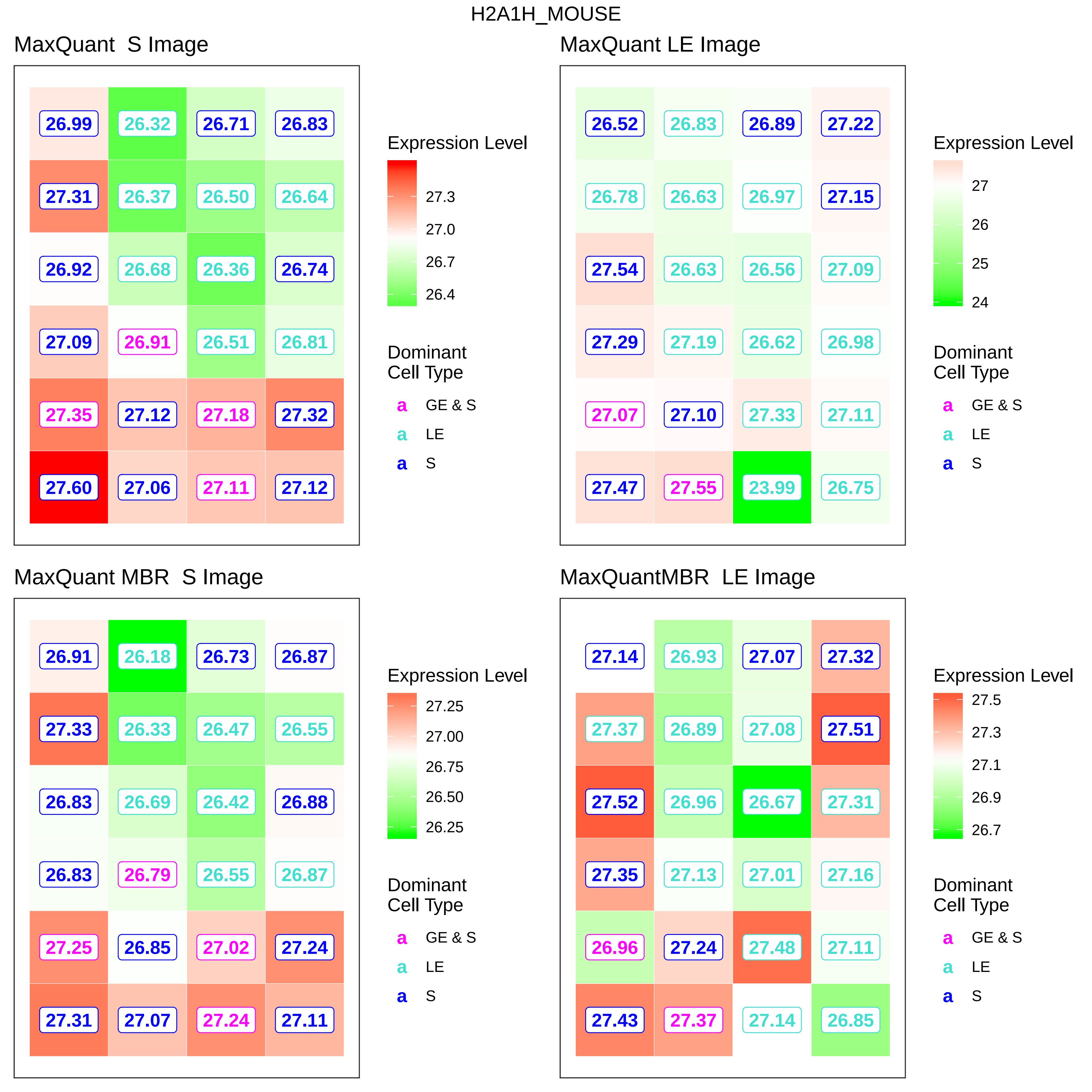

H2A1H_MOUSE
MaxQuant S Image
26.99 26.32 26.71 26.83
27.31 26.37 26.50 26.64
26.92 26.68 26.36 26.74
27.09 26.91 26.51 26.81
27.35 27.12 27.18 27.32
27.60 27.06 27.11 27.12
Expression Level
27.3
27.0
26.7
26.4
Dominant Cell Type
a GE & S
a LE
a S
MaxQuant LE Image
26.52 26.83 26.89 27.22
26.78 26.63 26.97 27.15
27.54 26.63 26.56 27.09
27.29 27.19 26.62 26.98
27.07 27.10 27.33 27.11
27.47 27.55 23.99 26.75
Expression Level
27
26
25
24
Dominant Cell Type
a GE & S
a LE
a S
MaxQuant MBR S Image
26.91 26.18 26.73 26.87
27.33 26.33 26.47 26.55
26.83 26.69 26.42 26.88
26.83 26.79 26.55 26.87
27.25 26.85 27.02 27.24
27.31 27.07 27.24 27.11
Expression Level
27.25
27.00
26.75
26.50
26.25
Dominant Cell Type
a GE & S
a LE
a S
MaxQuantMBR LE Image
27.14 26.93 27.07 27.32
27.37 26.89 27.08 27.51
27.52 26.96 26.67 27.31
27.35 27.13 27.01 27.16
26.96 27.24 27.48 27.11
27.43 27.37 27.14 26.85
Expression Level
27.5
27.3
27.1
26.9
26.7
Dominant Cell Type
a GE & S
a LE
a S

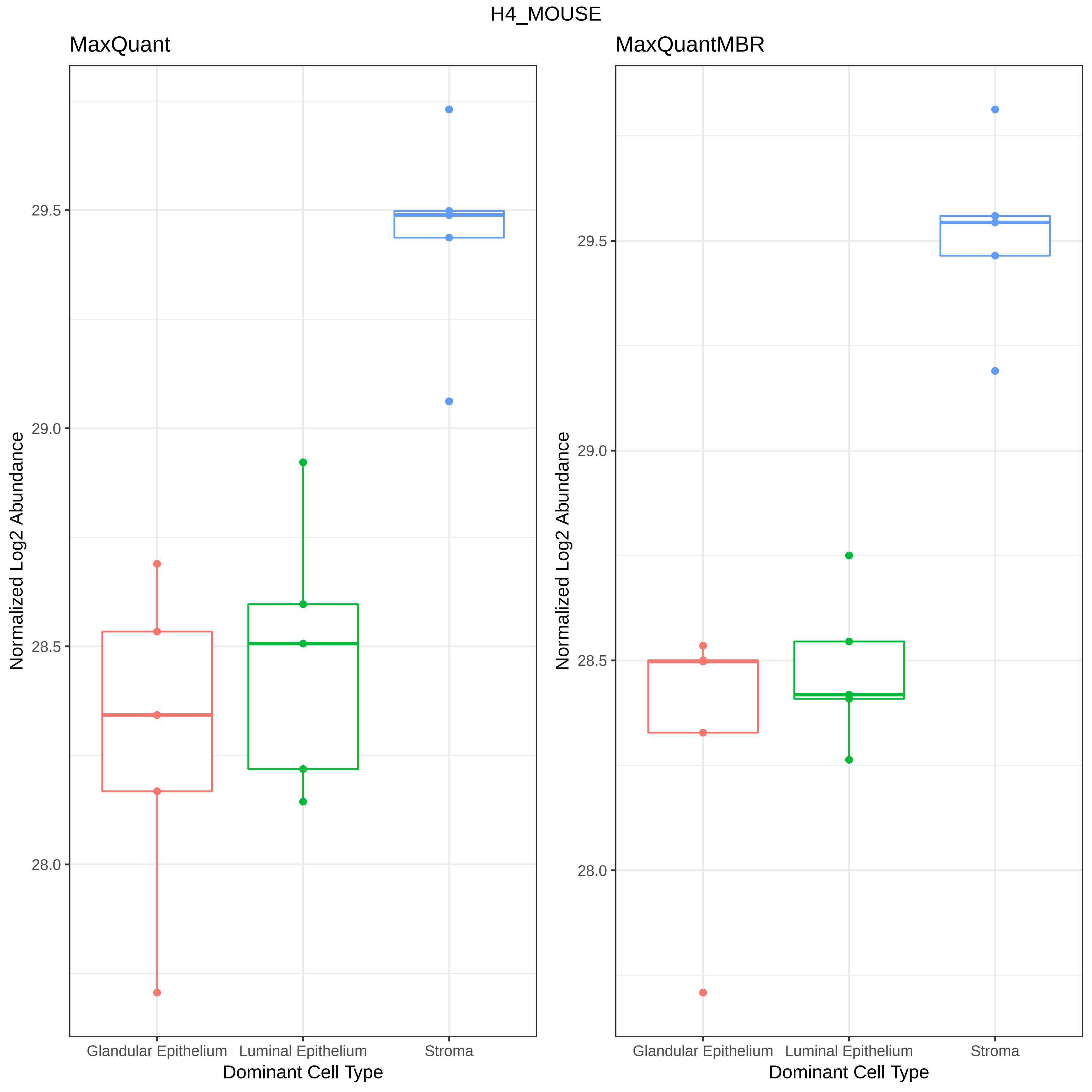
H4_MOUSE
MaxQuant
MaxQuantMBR
Normalized Log2 Abundance
29.5
29.0
28.5
28.0
Glandular Epithelium
Luminal Epithelium
Stroma
Dominant Cell Type

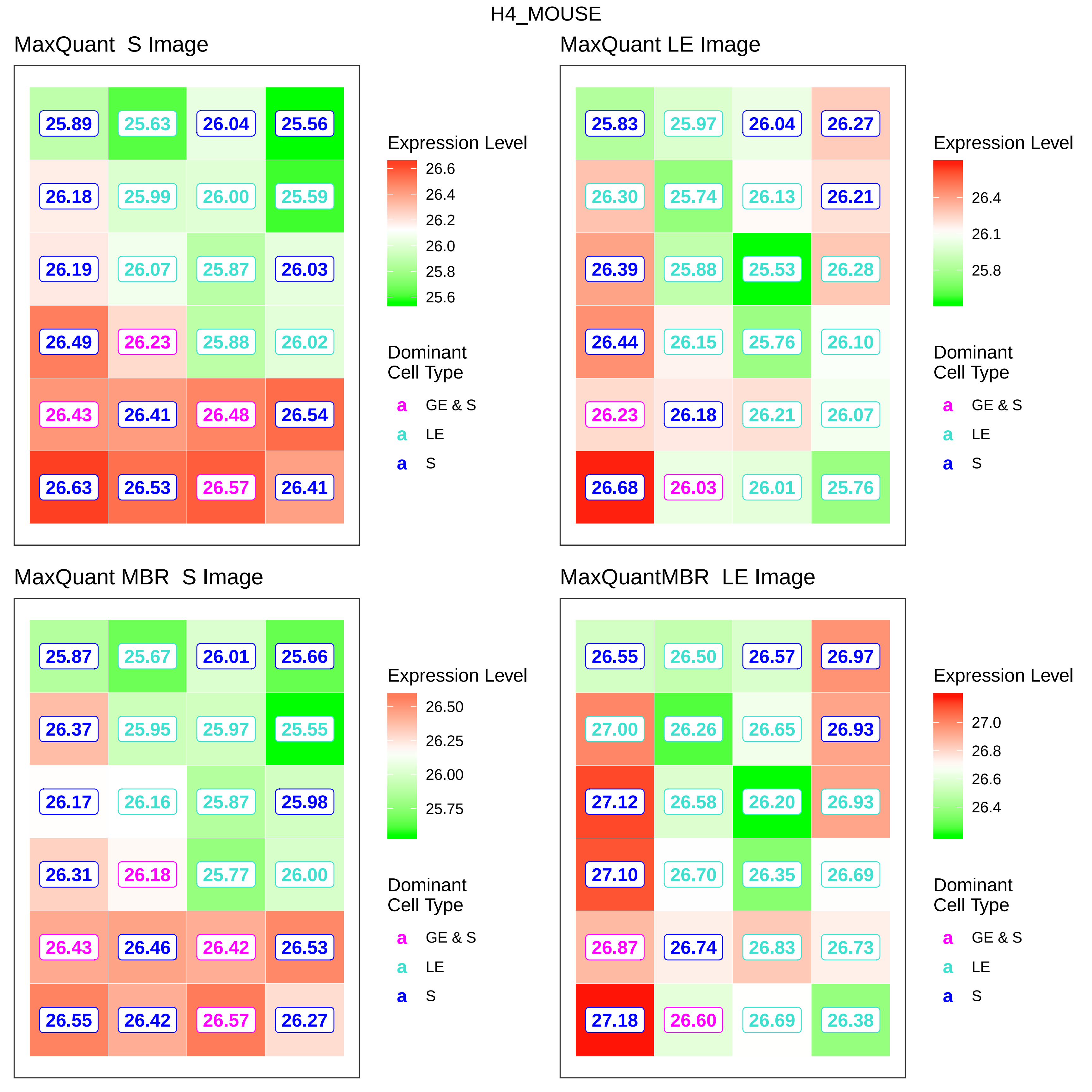

H4_MOUSE
MaxQuant S Image
25.89 25.63 26.04 25.56
26.18 25.99 26.00 25.59
26.19 26.07 25.87 26.03
26.49 26.23 25.88 26.02
26.43 26.41 26.48 26.54
26.63 26.53 26.57 26.41
Expression Level
26.6 26.4 26.2 26.0 25.8 25.6
Dominant Cell Type
a GE & S
a LE
a S
MaxQuant LE Image
25.83 25.97 26.04 26.27
26.30 25.74 26.13 26.21
26.39 25.88 25.53 26.28
26.44 26.15 25.76 26.10
26.23 26.18 26.21 26.07
26.68 26.03 26.01 25.76
Expression Level
26.4 26.1 25.8
Dominant Cell Type
a GE & S
a LE
a S
MaxQuant MBR S Image
25.87 25.67 26.01 25.66
26.37 25.95 25.97 25.55
26.17 26.16 25.87 25.98
26.31 26.18 25.77 26.00
26.43 26.46 26.42 26.53
26.55 26.42 26.57 26.27
Expression Level
26.50 26.25 26.00 25.75
Dominant Cell Type
a GE & S
a LE
a S
MaxQuantMBR LE Image
26.55 26.50 26.57 26.97
27.00 26.26 26.65 26.93
27.12 26.58 26.20 26.93
27.10 26.70 26.35 26.69
26.87 26.74 26.83 26.73
27.18 26.60 26.69 26.38
Expression Level
27.0 26.8 26.6 26.4
Dominant Cell Type
a GE & S
a LE
a S

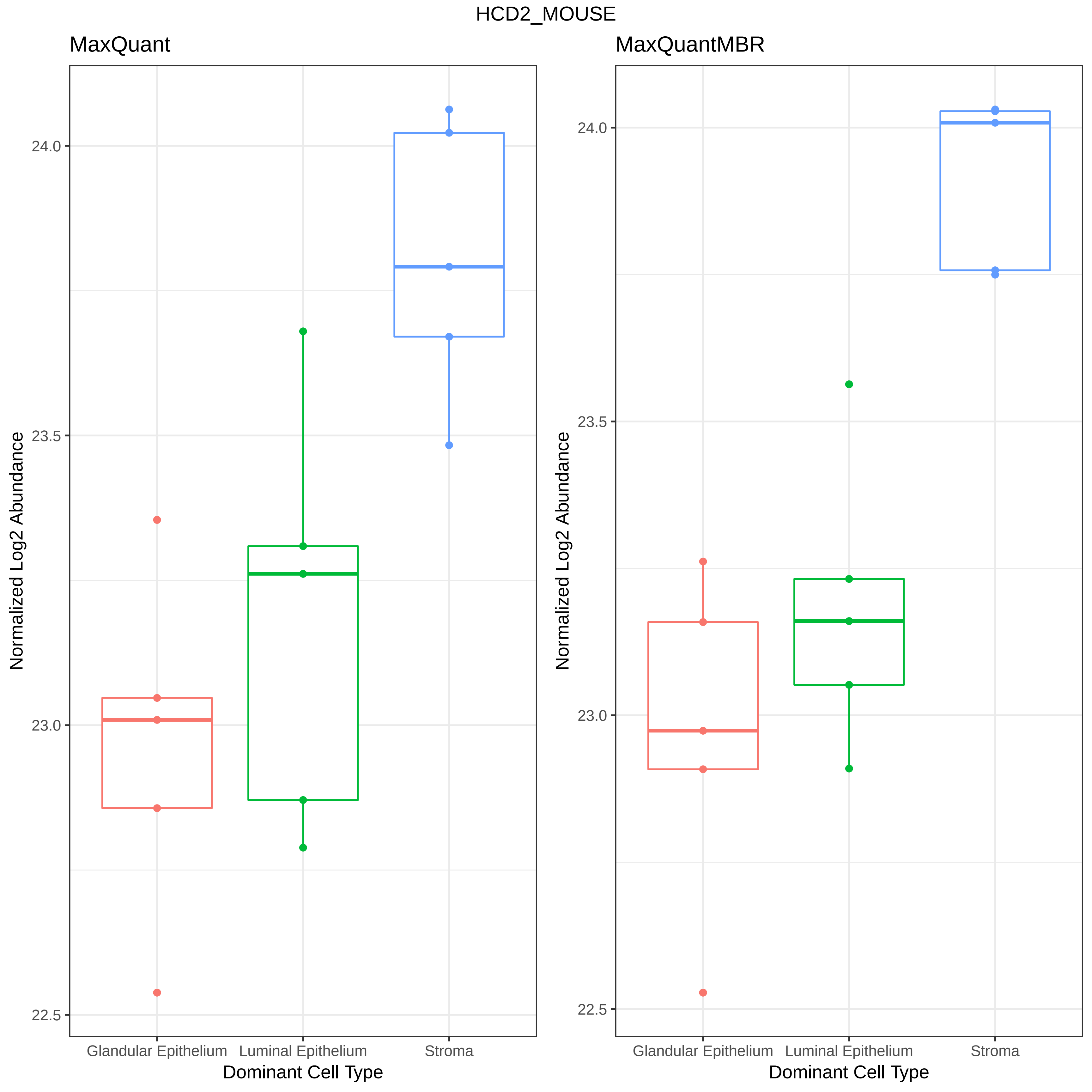

HCD2_MOUSE
MaxQuant
MaxQuantMBR
Normalized Log2 Abundance
24.0
23.5
23.0
22.5
Glandular Epithelium
Luminal Epithelium
Stroma
Dominant Cell Type

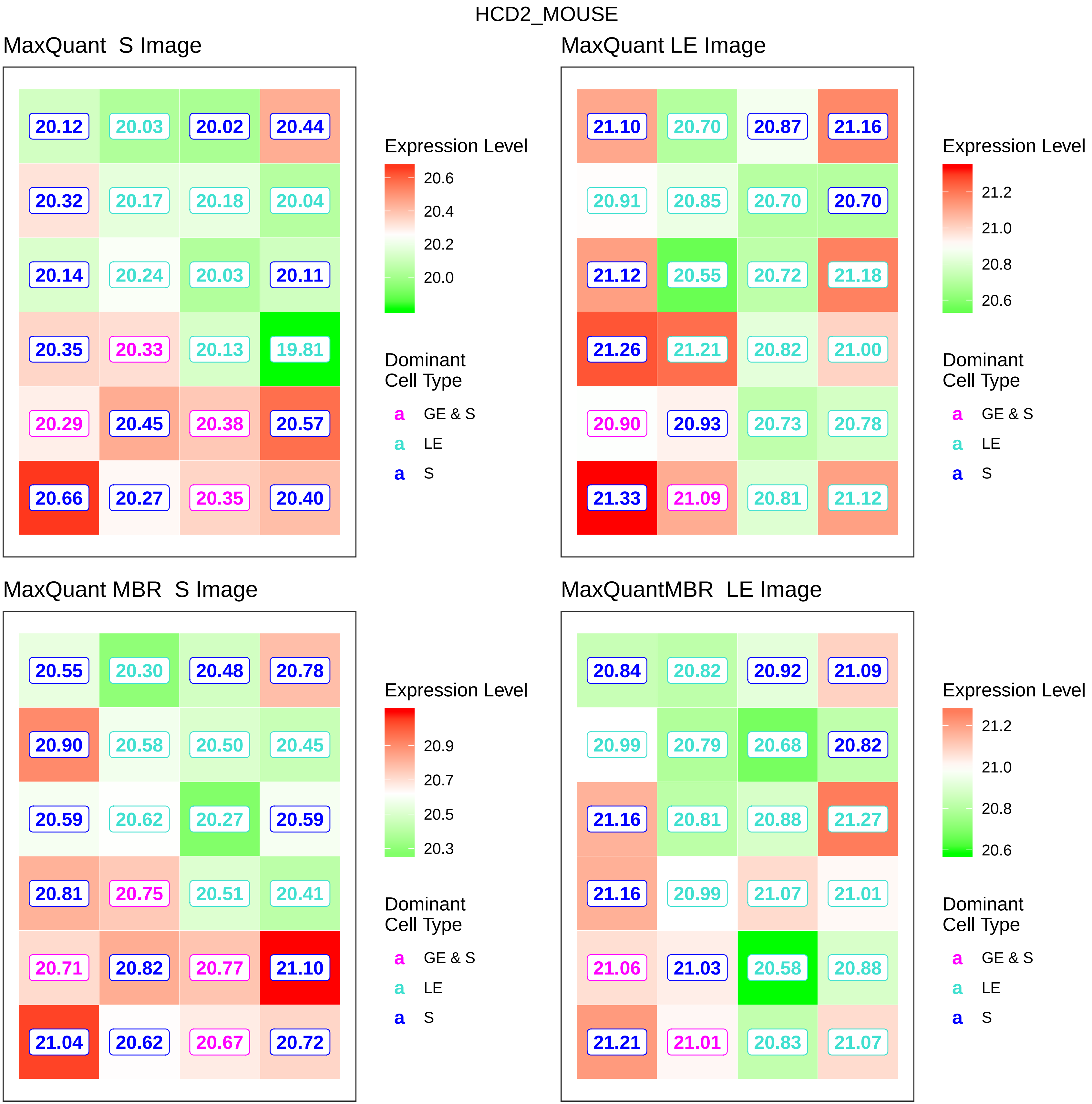
HCD2_MOUSE
MaxQuant S Image
20.12 20.03 20.02 20.44
20.32 20.17 20.18 20.04
20.14 20.24 20.03 20.11
20.35 20.33 20.13 19.81
20.29 20.45 20.38 20.57
20.66 20.27 20.35 20.40
Expression Level
20.6
20.4
20.2
20.0
Dominant Cell Type
a GE & S
a LE
a S
MaxQuant LE Image
21.10 20.70 20.87 21.16
20.91 20.85 20.70 20.70
21.12 20.55 20.72 21.18
21.26 21.21 20.82 21.00
20.90 20.93 20.73 20.78
21.33 21.09 20.81 21.12
Expression Level
21.2
21.0
20.8
20.6
Dominant Cell Type
a GE & S
a LE
a S
MaxQuant MBR S Image
20.55 20.30 20.48 20.78
20.90 20.58 20.50 20.45
20.59 20.62 20.27 20.59
20.81 20.75 20.51 20.41
20.71 20.82 20.77 21.10
21.04 20.62 20.67 20.72
Expression Level
20.9
20.7
20.5
20.3
Dominant Cell Type
a GE & S
a LE
a S
MaxQuantMBR LE Image
20.84 20.82 20.92 21.09
20.99 20.79 20.68 20.82
21.16 20.81 20.88 21.27
21.16 20.99 21.07 21.01
21.06 21.03 20.58 20.88
21.21 21.01 20.83 21.07
Expression Level
21.2
21.0
20.8
20.6
Dominant Cell Type
a GE & S
a LE
a S

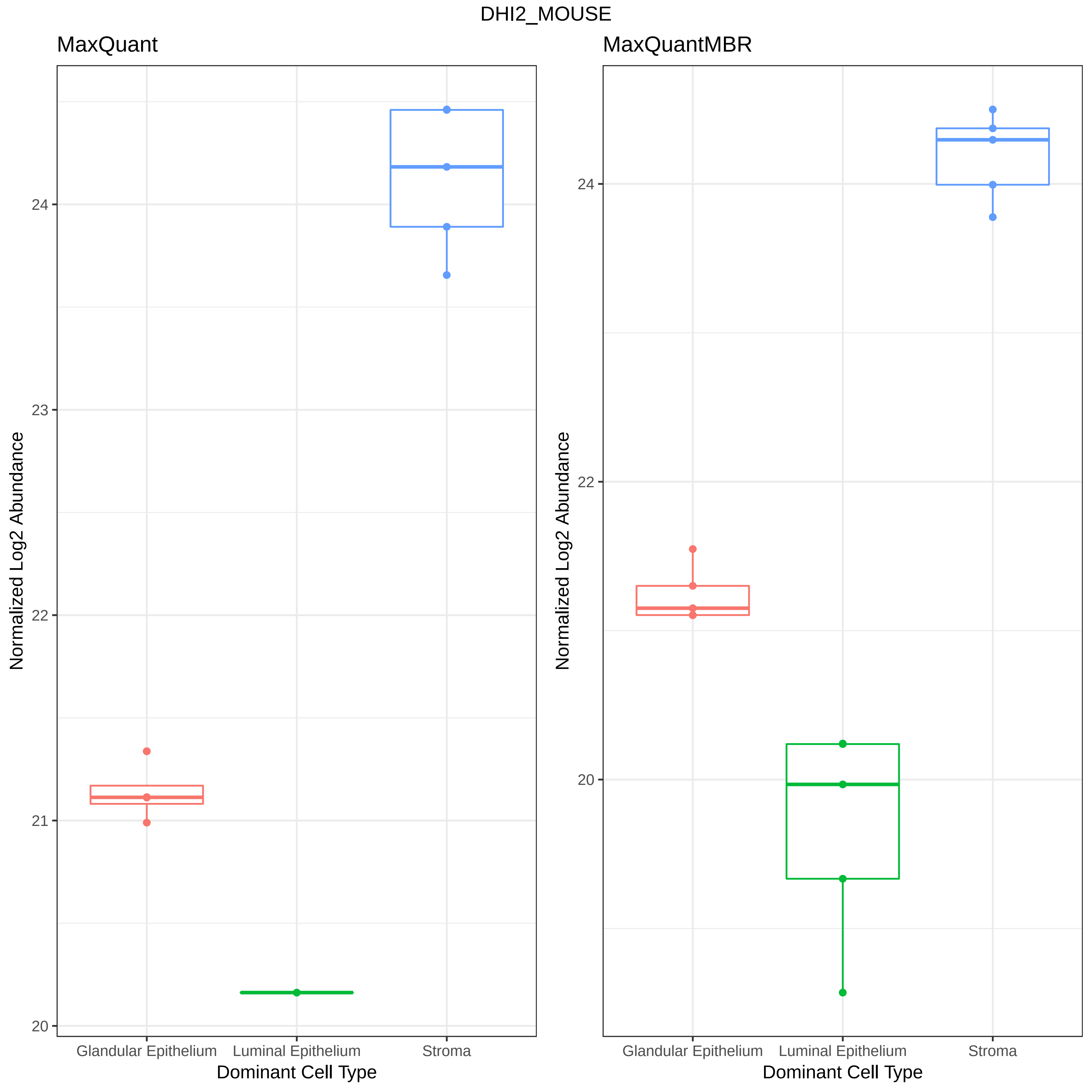
DHI2_MOUSE
MaxQuant
MaxQuantMBR
Normalized Log2 Abundance
24
23
22
21
20
Glandular Epithelium
Luminal Epithelium
Stroma
Dominant Cell Type

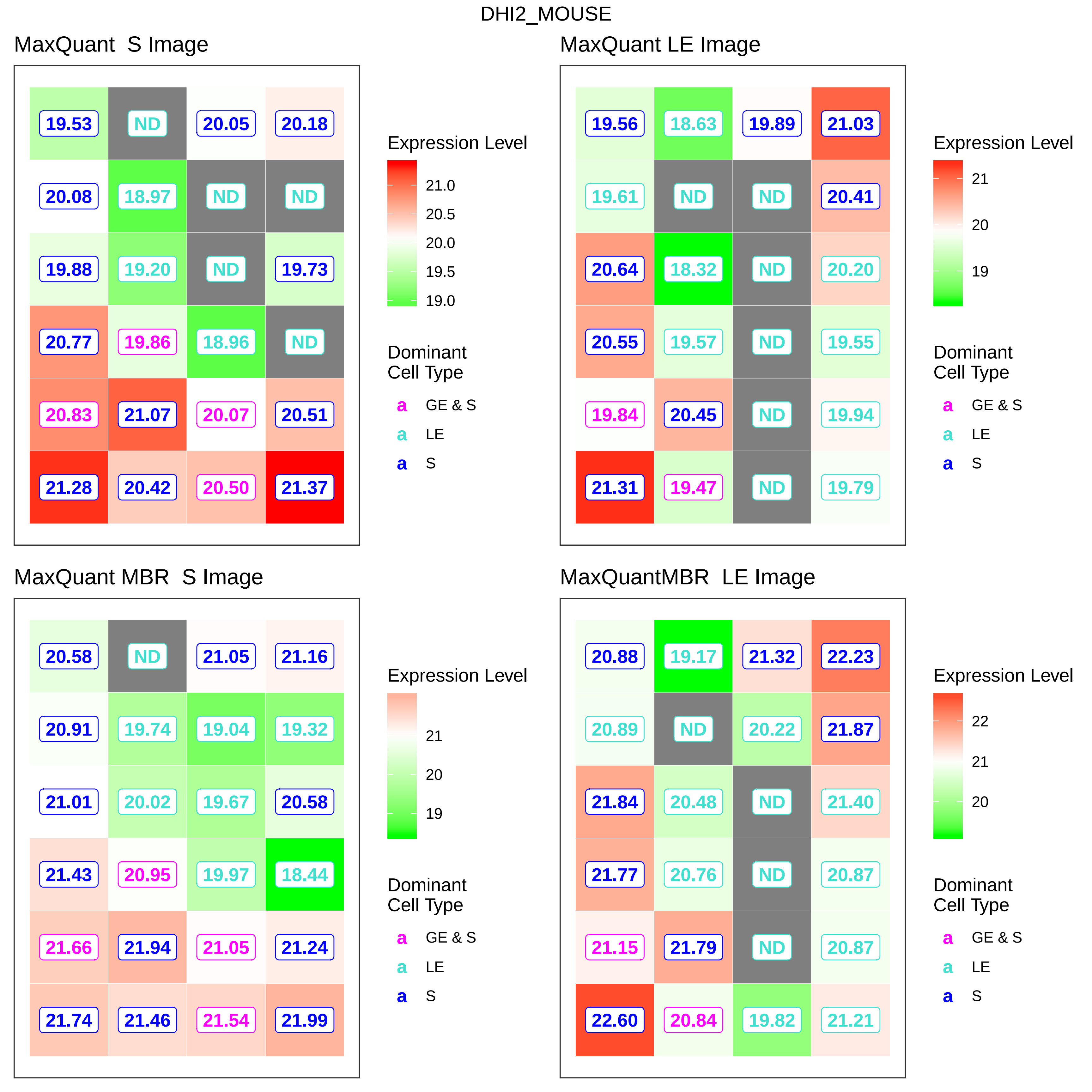
DHI2_MOUSE
MaxQuant S Image
19.53
ND
20.05
20.18
20.08
18.97
ND
ND
19.88
19.20
ND
19.73
20.77
19.86
18.96
ND
20.83
21.07
20.07
20.51
21.28
20.42
20.50
21.37
Expression Level
21.0
20.5
20.0
19.5
19.0
Dominant Cell Type
a GE & S
a LE
a S
MaxQuant LE Image
19.56
18.63
19.89
21.03
19.61
ND
ND
20.41
20.64
18.32
ND
20.20
20.55
19.57
ND
19.55
19.84
20.45
ND
19.94
21.31
19.47
ND
19.79
Expression Level
21
20
19
Dominant Cell Type
a GE & S
a LE
a S
MaxQuant MBR S Image
20.58
ND
21.05
21.16
20.91
19.74
19.04
19.32
21.01
20.02
19.67
20.58
21.43
20.95
19.97
18.44
21.66
21.94
21.05
21.24
21.74
21.46
21.54
21.99
Expression Level
21
20
19
Dominant Cell Type
a GE & S
a LE
a S
MaxQuantMBR LE Image
20.88
19.17
21.32
22.23
20.89
ND
20.22
21.87
21.84
20.48
ND
21.40
21.77
20.76
ND
20.87
21.15
21.79
ND
20.87
22.60
20.84
19.82
21.21
Expression Level
22
21
20
Dominant Cell Type
a GE & S
a LE
a S

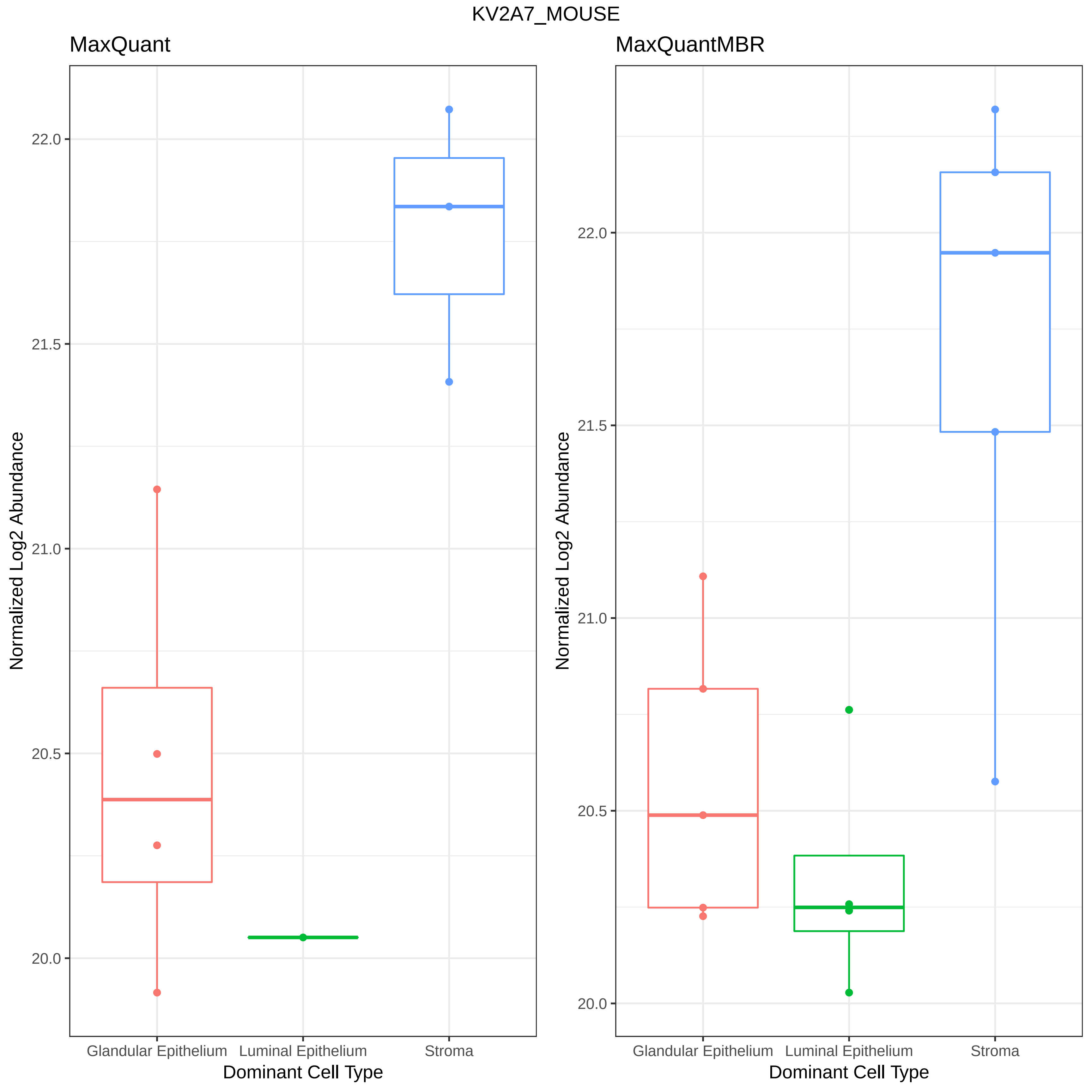
KV2A7_MOUSE
MaxQuant
MaxQuantMBR
Normalized Log2 Abundance
22.0
21.5
21.0
20.5
20.0
Glandular Epithelium
Luminal Epithelium
Stroma
Dominant Cell Type

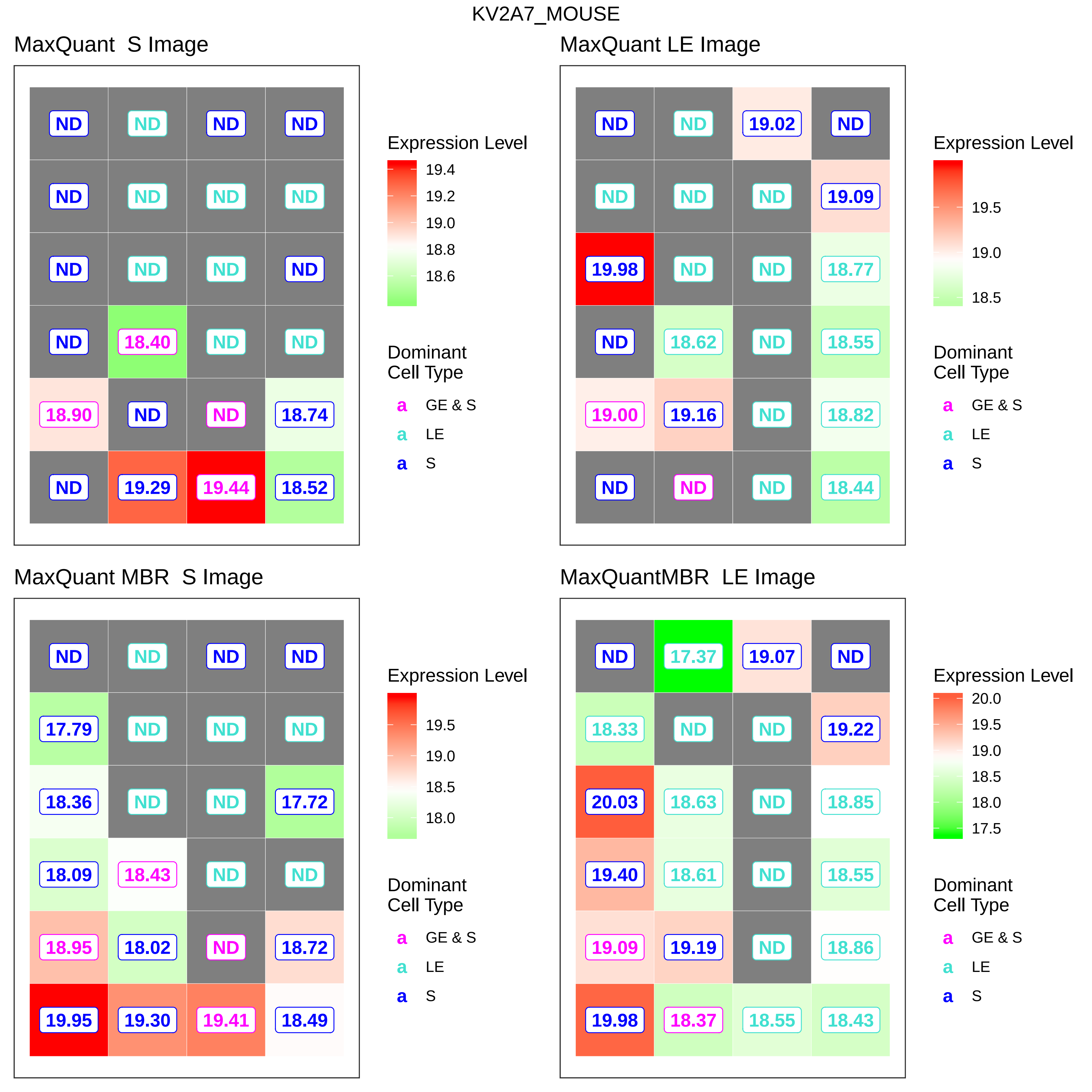

KV2A7_MOUSE
MaxQuant S Image
ND ND ND ND
ND ND ND ND
ND ND ND ND
ND 18.40 ND ND
18.90 ND ND 18.74
ND 19.29 19.44 18.52
Expression Level
19.4
19.2
19.0
18.8
18.6
Dominant Cell Type
a GE & S
a LE
a S
MaxQuant LE Image
ND ND 19.02 ND
ND ND ND 19.09
19.98 ND ND 18.77
ND 18.62 ND 18.55
19.00 19.16 ND 18.82
ND ND ND 18.44
Expression Level
19.5
19.0
18.5
Dominant Cell Type
a GE & S
a LE
a S
MaxQuant MBR S Image
ND ND ND ND
17.79 ND ND ND
18.36 ND ND 17.72
18.09 18.43 ND ND
18.95 18.02 ND 18.72
19.95 19.30 19.41 18.49
Expression Level
19.5
19.0
18.5
18.0
Dominant Cell Type
a GE & S
a LE
a S
MaxQuantMBR LE Image
ND 17.37 19.07 ND
18.33 ND ND 19.22
20.03 18.63 ND 18.85
19.40 18.61 ND 18.55
19.09 19.19 ND 18.86
19.98 18.37 18.55 18.43
Expression Level
20.0
19.5
19.0
18.5
18.0
17.5
Dominant Cell Type
a GE & S
a LE
a S

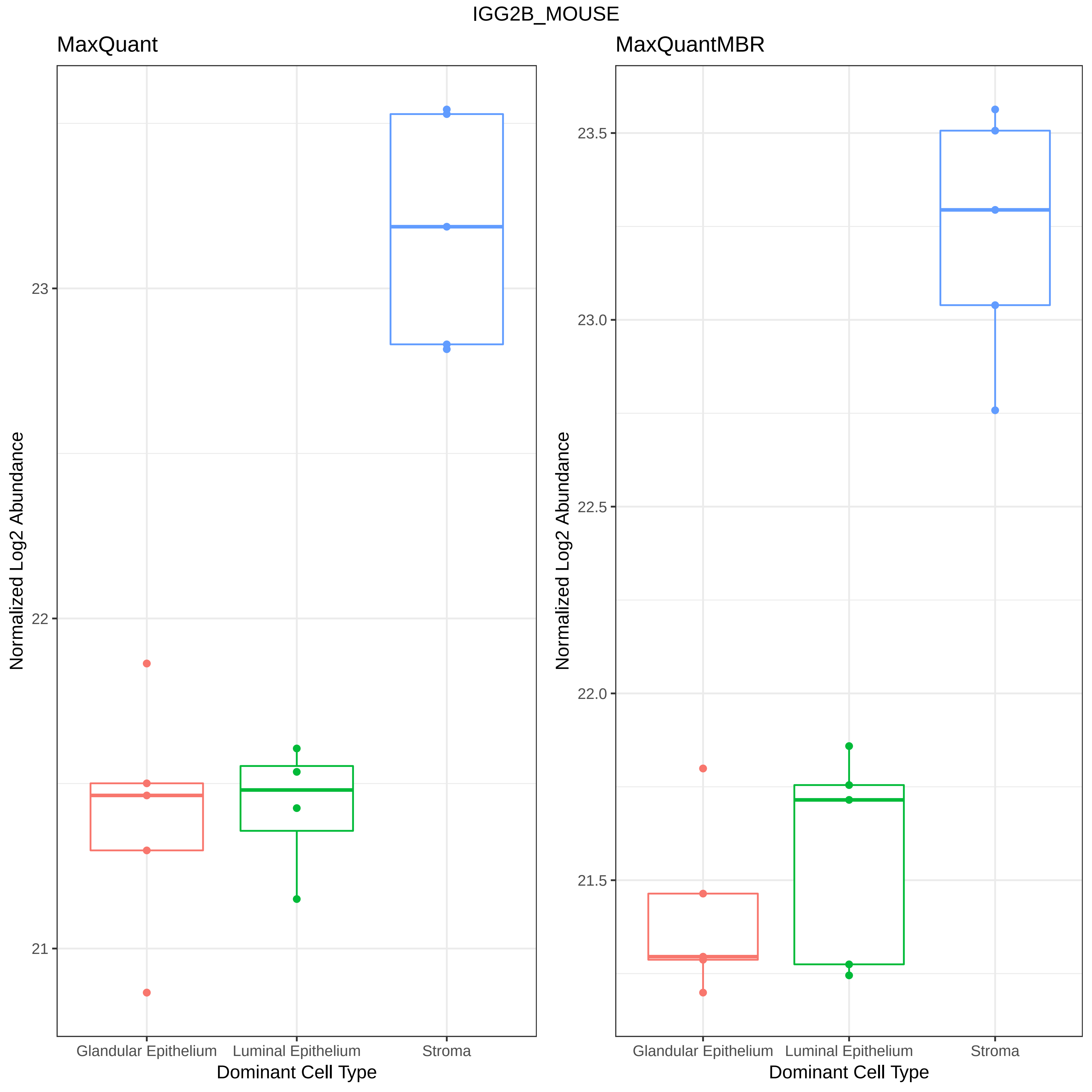
IGG2B_MOUSE
MaxQuant
MaxQuantMBR
Normalized Log2 Abundance
23
22
21
23.5
23.0
22.5
22.0
21.5
Glandular Epithelium
Luminal Epithelium
Stroma
Dominant Cell Type

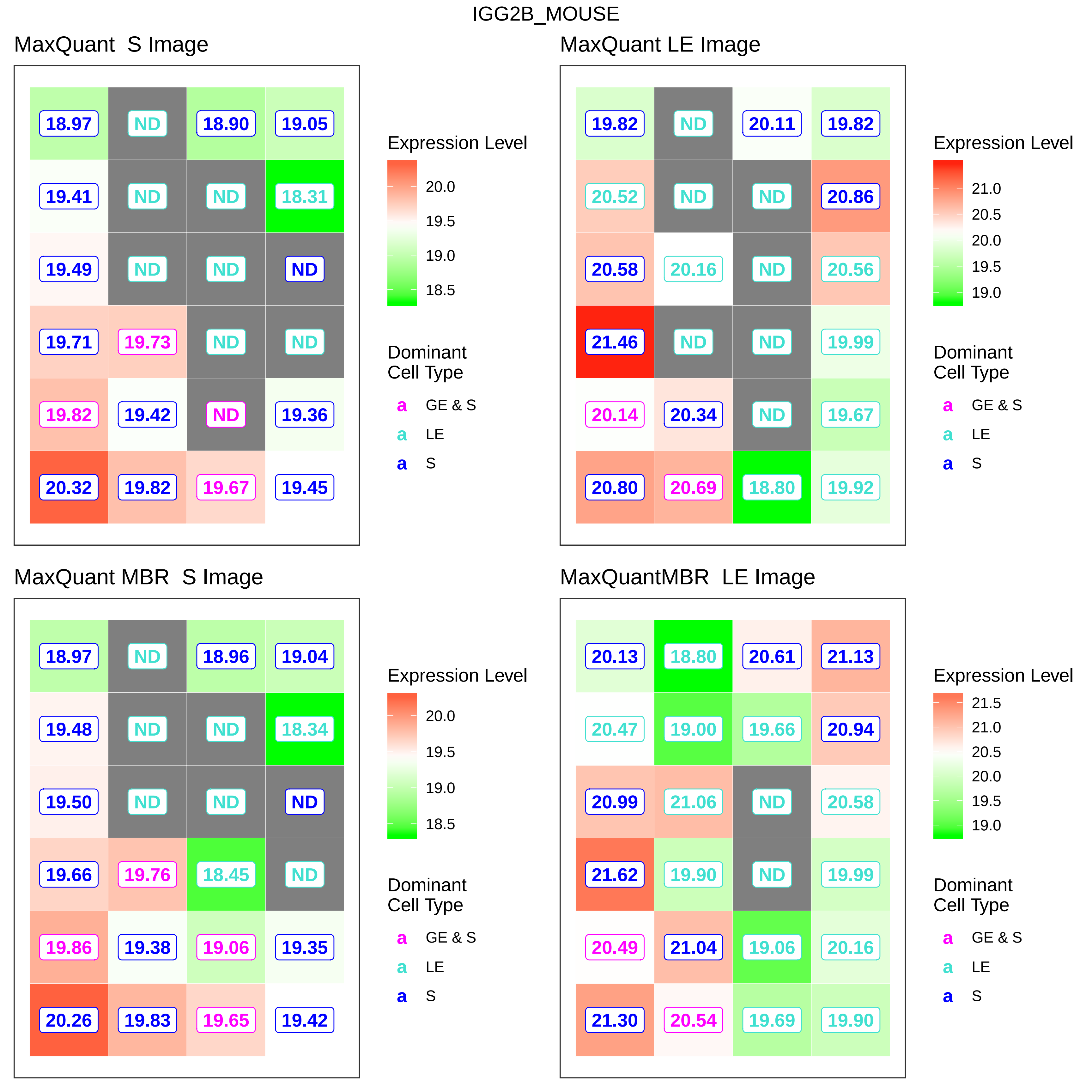

IGG2B_MOUSE
MaxQuant S Image
18.97 ND 18.90 19.05
19.41 ND ND 18.31
19.49 ND ND ND
19.71 19.73 ND ND
19.82 19.42 ND 19.36
20.32 19.82 19.67 19.45
Expression Level
20.0
19.5
19.0
18.5
Dominant Cell Type
a GE & S
a LE
a S
MaxQuant LE Image
19.82 ND 20.11 19.82
20.52 ND ND 20.86
20.58 20.16 ND 20.56
21.46 ND ND 19.99
20.14 20.34 ND 19.67
20.80 20.69 18.80 19.92
Expression Level
21.0
20.5
20.0
19.5
19.0
Dominant Cell Type
a GE & S
a LE
a S
MaxQuant MBR S Image
18.97 ND 18.96 19.04
19.48 ND ND 18.34
19.50 ND ND ND
19.66 19.76 18.45 ND
19.86 19.38 19.06 19.35
20.26 19.83 19.65 19.42
Expression Level
20.0
19.5
19.0
18.5
Dominant Cell Type
a GE & S
a LE
a S
MaxQuantMBR LE Image
20.13 18.80 20.61 21.13
20.47 19.00 19.66 20.94
20.99 21.06 ND 20.58
21.62 19.90 ND 19.99
20.49 21.04 19.06 20.16
21.30 20.54 19.69 19.90
Expression Level
21.5
21.0
20.5
20.0
19.5
19.0
Dominant Cell Type
a GE & S
a LE
a S

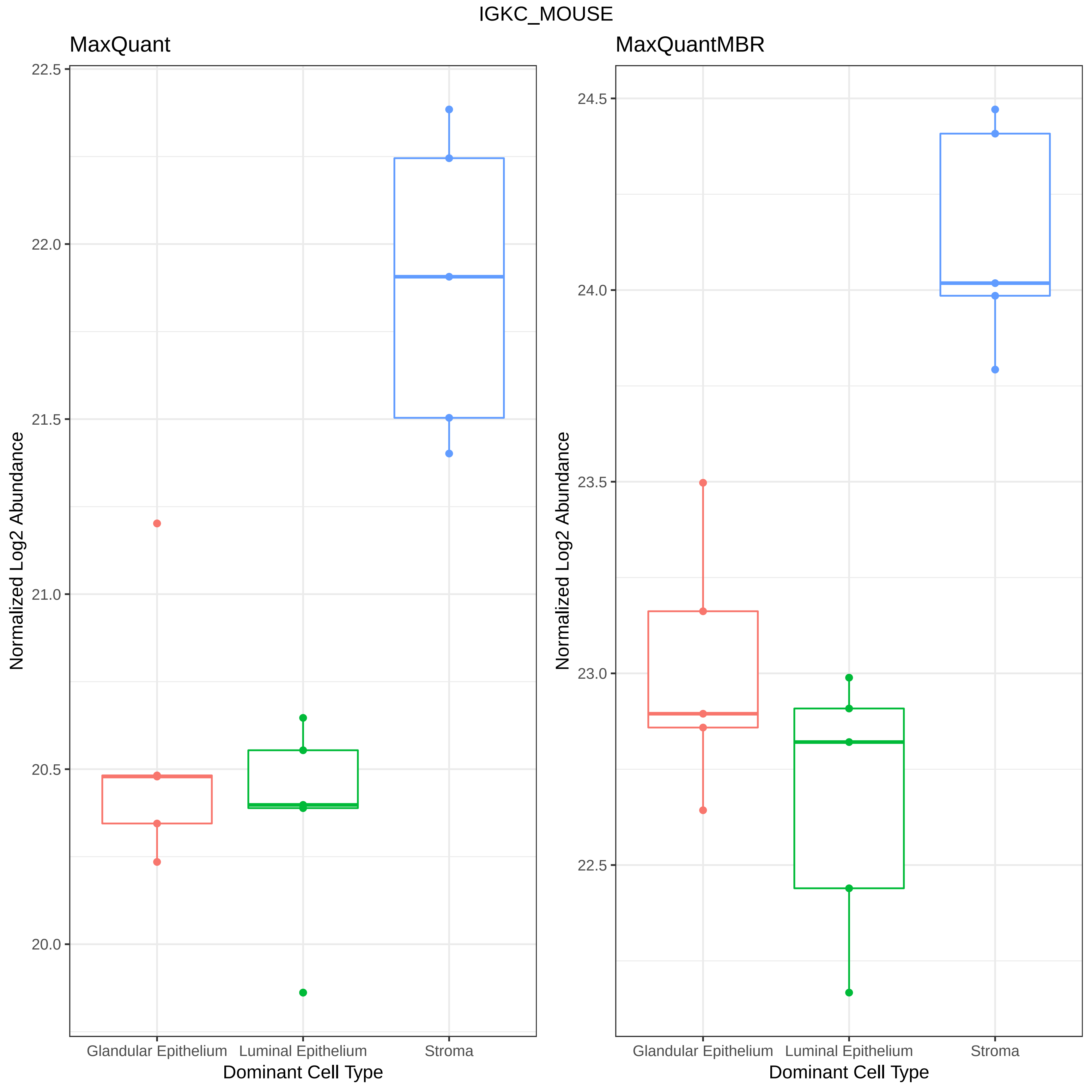

IGKC_MOUSE
MaxQuant
MaxQuantMBR
Normalized Log2 Abundance
22.5
22.0
21.5
21.0
20.5
20.0
24.5
24.0
23.5
23.0
22.5
Glandular Epithelium
Luminal Epithelium
Stroma
Dominant Cell Type

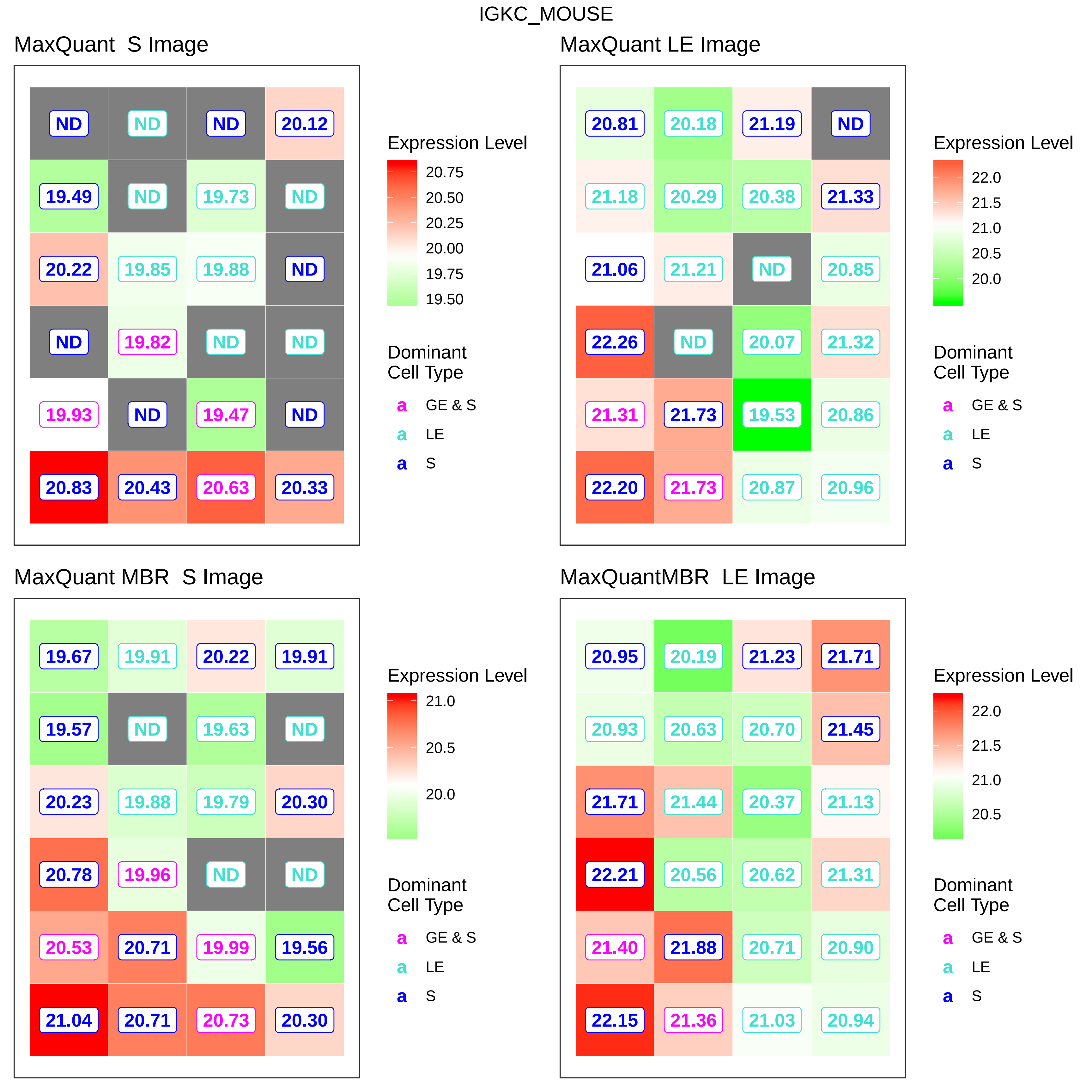
IGKC_MOUSE
MaxQuant S Image
ND
ND
ND
20.12
19.49
ND
19.73
ND
20.22
19.85
19.88
ND
ND
19.82
ND
ND
19.93
ND
19.47
ND
20.83
20.43
20.63
20.33
Expression Level
20.75
20.50
20.25
20.00
19.75
19.50
Dominant Cell Type
a GE & S
a LE
a S
MaxQuant LE Image
20.81
20.18
21.19
ND
21.18
20.29
20.38
21.33
21.06
21.21
ND
20.85
22.26
ND
20.07
21.32
21.31
21.73
19.53
20.86
22.20
21.73
20.87
20.96
Expression Level
22.0
21.5
21.0
20.5
20.0
Dominant Cell Type
a GE & S
a LE
a S
MaxQuant MBR S Image
19.67
19.91
20.22
19.91
19.57
ND
19.63
ND
20.23
19.88
19.79
20.30
20.78
19.96
ND
ND
20.53
20.71
19.99
19.56
21.04
20.71
20.73
20.30
Expression Level
21.0
20.5
20.0
Dominant Cell Type
a GE & S
a LE
a S
MaxQuantMBR LE Image
20.95
20.19
21.23
21.71
20.93
20.63
20.70
21.45
21.71
21.44
20.37
21.13
22.21
20.56
20.62
21.31
21.40
21.88
20.71
20.90
22.15
21.36
21.03
20.94
Expression Level
22.0
21.5
21.0
20.5
Dominant Cell Type
a GE & S
a LE
a S

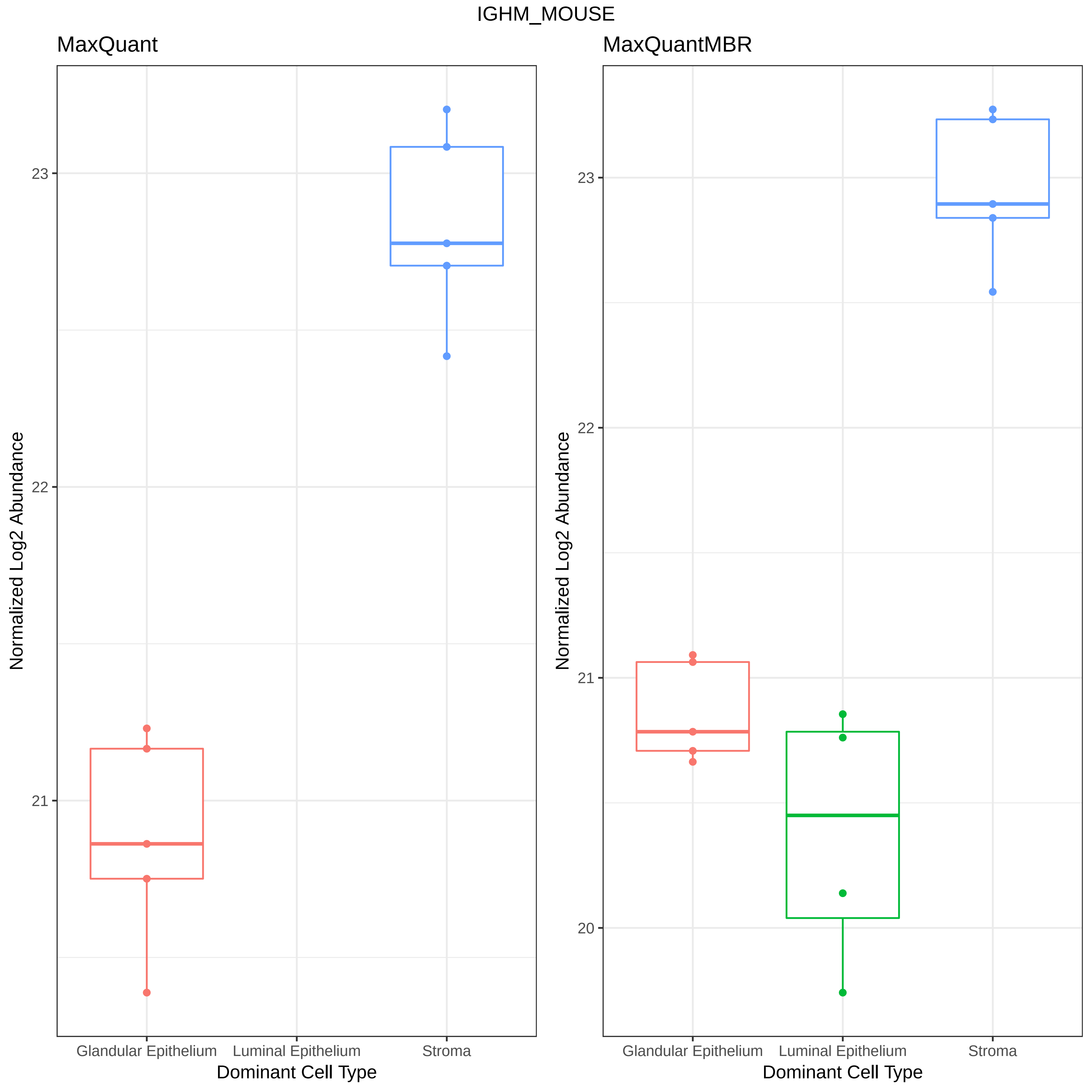

IGHM_MOUSE
MaxQuant
MaxQuantMBR
Normalized Log2 Abundance
23
22
21
20
Glandular Epithelium
Luminal Epithelium
Stroma
Dominant Cell Type

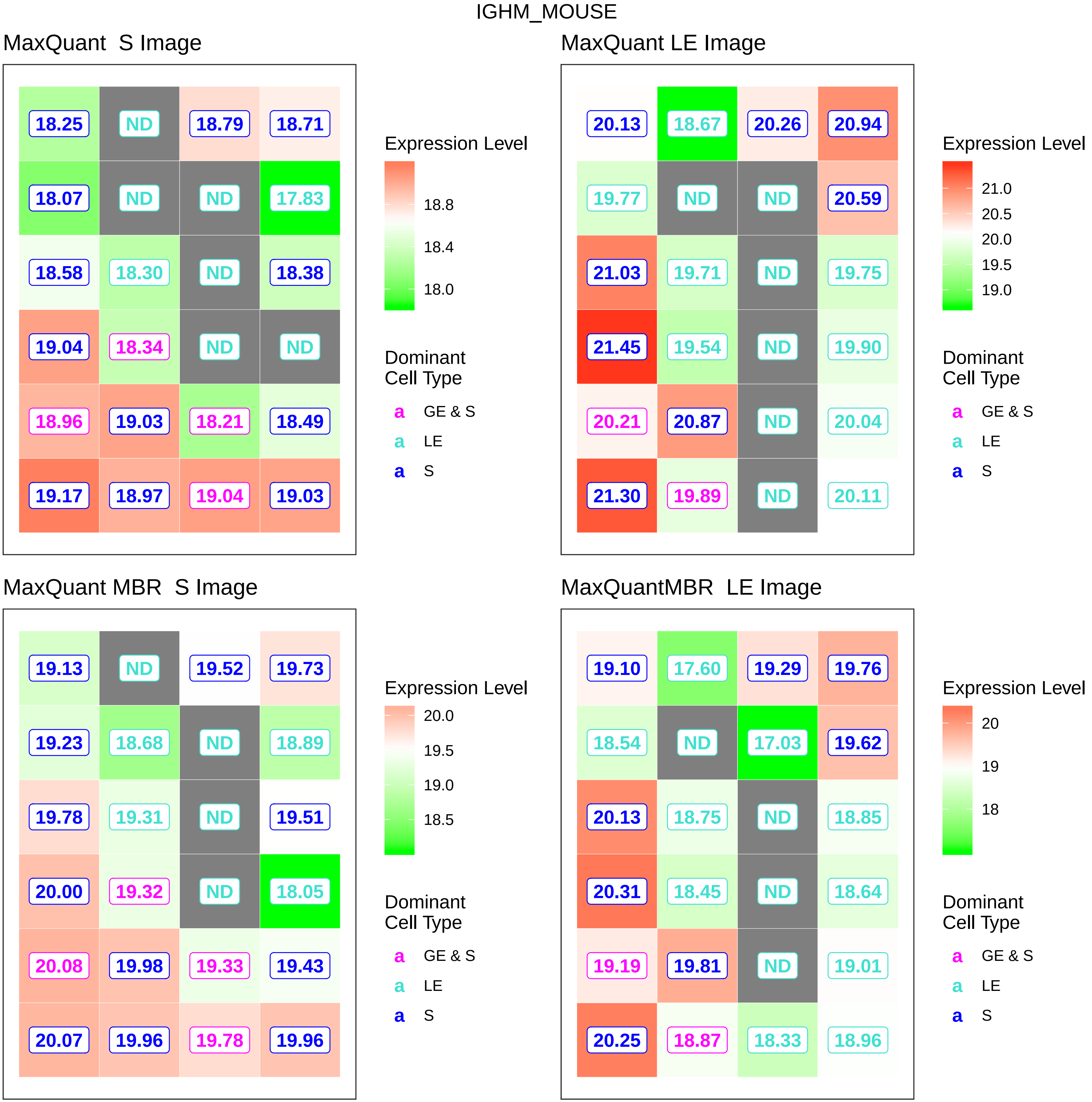
IGHM_MOUSE
MaxQuant S Image
18.25 ND 18.79 18.71
18.07 ND ND 17.83
18.58 18.30 ND 18.38
19.04 18.34 ND ND
18.96 19.03 18.21 18.49
19.17 18.97 19.04 19.03
Expression Level
18.8
18.4
18.0
Dominant Cell Type
a GE & S
a LE
a S
MaxQuant LE Image
20.13 18.67 20.26 20.94
19.77 ND ND 20.59
21.03 19.71 ND 19.75
21.45 19.54 ND 19.90
20.21 20.87 ND 20.04
21.30 19.89 ND 20.11
Expression Level
21.0
20.5
20.0
19.5
19.0
Dominant Cell Type
a GE & S
a LE
a S
MaxQuant MBR S Image
19.13 ND 19.52 19.73
19.23 18.68 ND 18.89
19.78 19.31 ND 19.51
20.00 19.32 ND 18.05
20.08 19.98 19.33 19.43
20.07 19.96 19.78 19.96
Expression Level
20.0
19.5
19.0
18.5
Dominant Cell Type
a GE & S
a LE
a S
MaxQuantMBR LE Image
19.10 17.60 19.29 19.76
18.54 ND 17.03 19.62
20.13 18.75 ND 18.85
20.31 18.45 ND 18.64
19.19 19.81 ND 19.01
20.25 18.87 18.33 18.96
Expression Level
20
19
18
Dominant Cell Type
a GE & S
a LE
a S

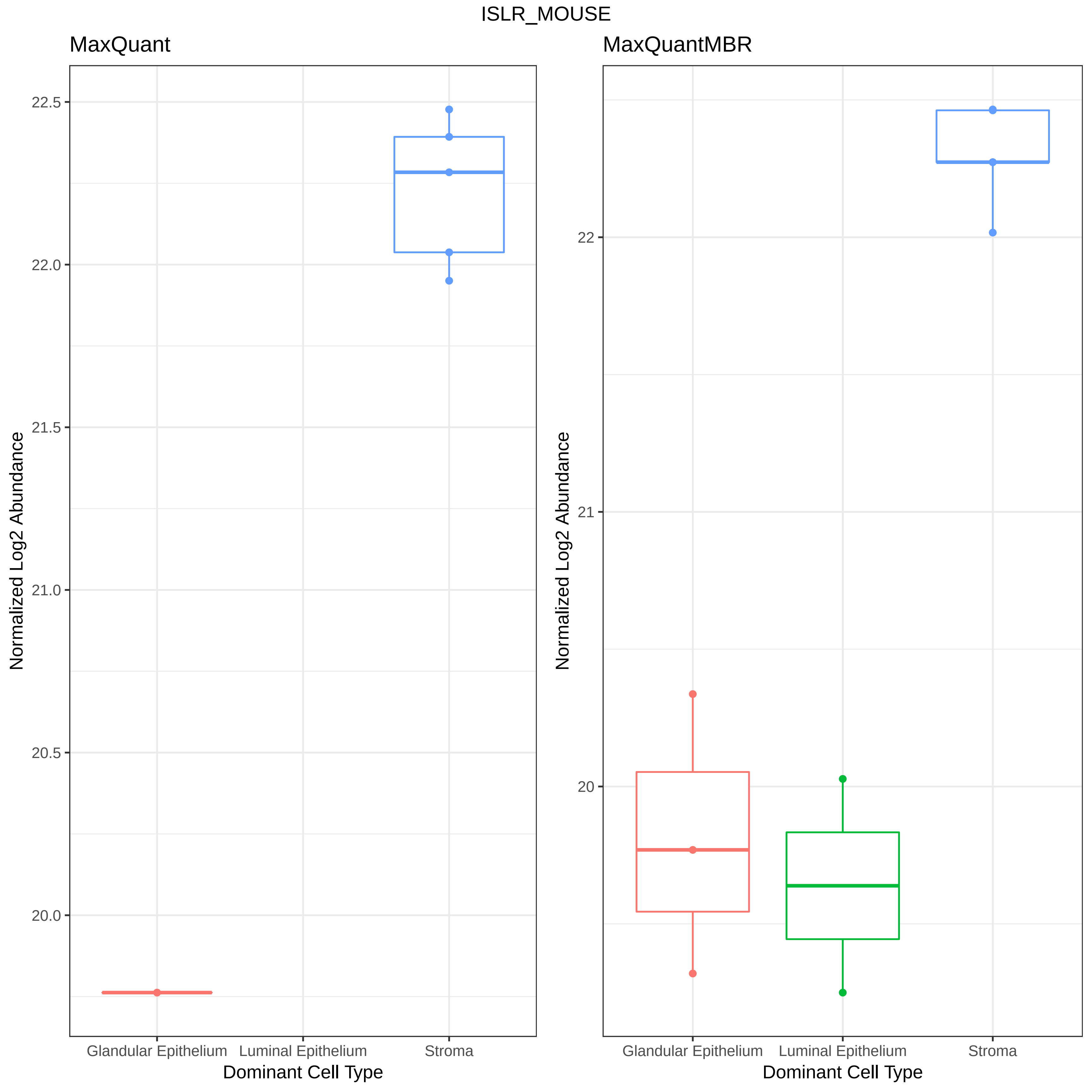

ISLR_MOUSE
MaxQuant
MaxQuantMBR
Normalized Log2 Abundance
22.5
22.0
21.5
21.0
20.5
20.0
22
21
20
Glandular Epithelium
Luminal Epithelium
Stroma
Dominant Cell Type

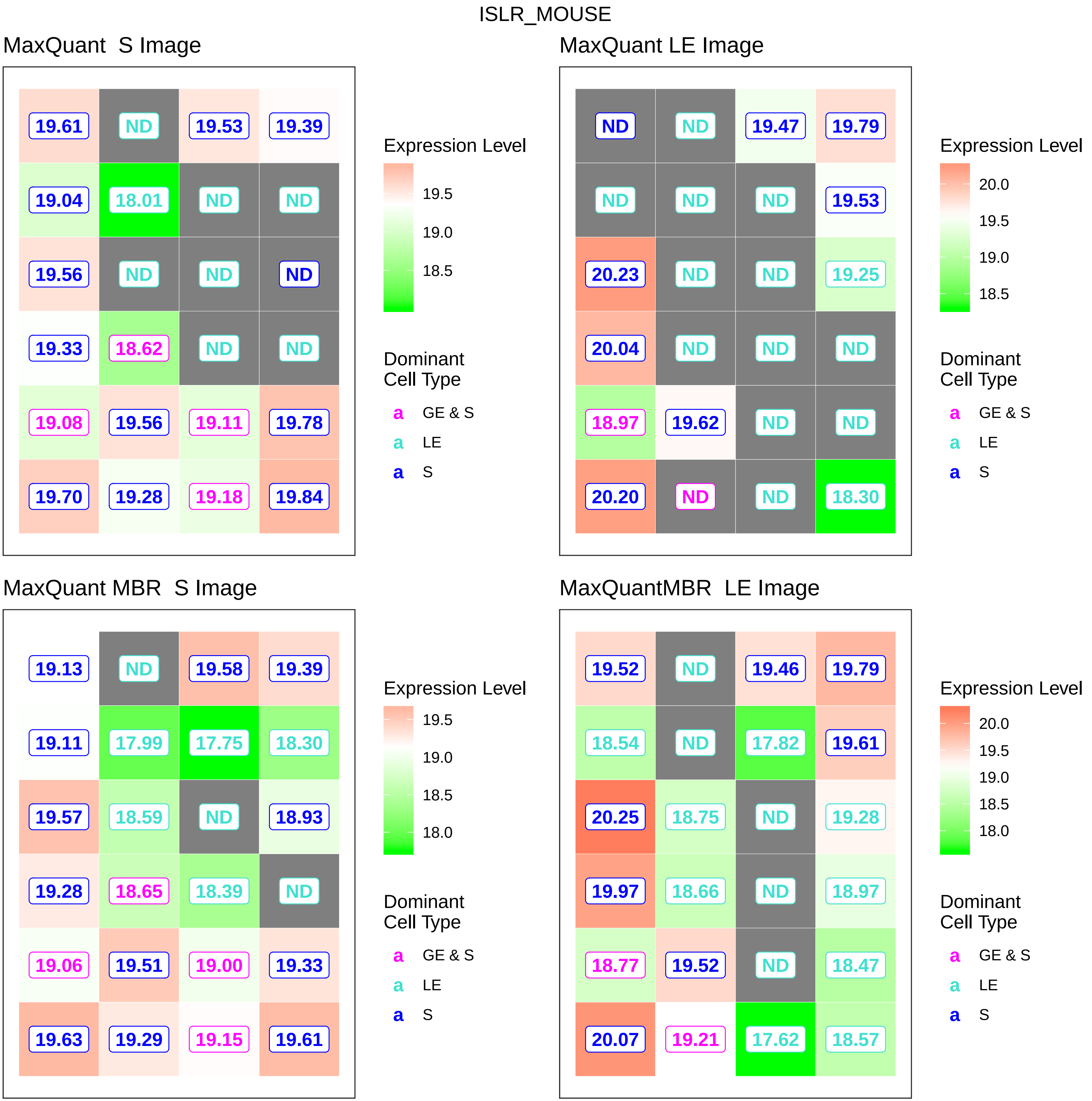

ISLR_MOUSE
MaxQuant S Image
19.61 ND 19.53 19.39
19.04 18.01 ND ND
19.56 ND ND ND
19.33 18.62 ND ND
19.08 19.56 19.11 19.78
19.70 19.28 19.18 19.84
Expression Level
19.5
19.0
18.5
Dominant Cell Type
a GE & S
a LE
a S
MaxQuant LE Image
ND ND 19.47 19.79
ND ND ND 19.53
20.23 ND ND 19.25
20.04 ND ND ND
18.97 19.62 ND ND
20.20 ND ND 18.30
Expression Level
20.0
19.5
19.0
18.5
Dominant Cell Type
a GE & S
a LE
a S
MaxQuant MBR S Image
19.13 ND 19.58 19.39
19.11 17.99 17.75 18.30
19.57 18.59 ND 18.93
19.28 18.65 18.39 ND
19.06 19.51 19.00 19.33
19.63 19.29 19.15 19.61
Expression Level
19.5
19.0
18.5
18.0
Dominant Cell Type
a GE & S
a LE
a S
MaxQuantMBR LE Image
19.52 ND 19.46 19.79
18.54 ND 17.82 19.61
20.25 18.75 ND 19.28
19.97 18.66 ND 18.97
18.77 19.52 ND 18.47
20.07 19.21 17.62 18.57
Expression Level
20.0
19.5
19.0
18.5
18.0
Dominant Cell Type
a GE & S
a LE
a S

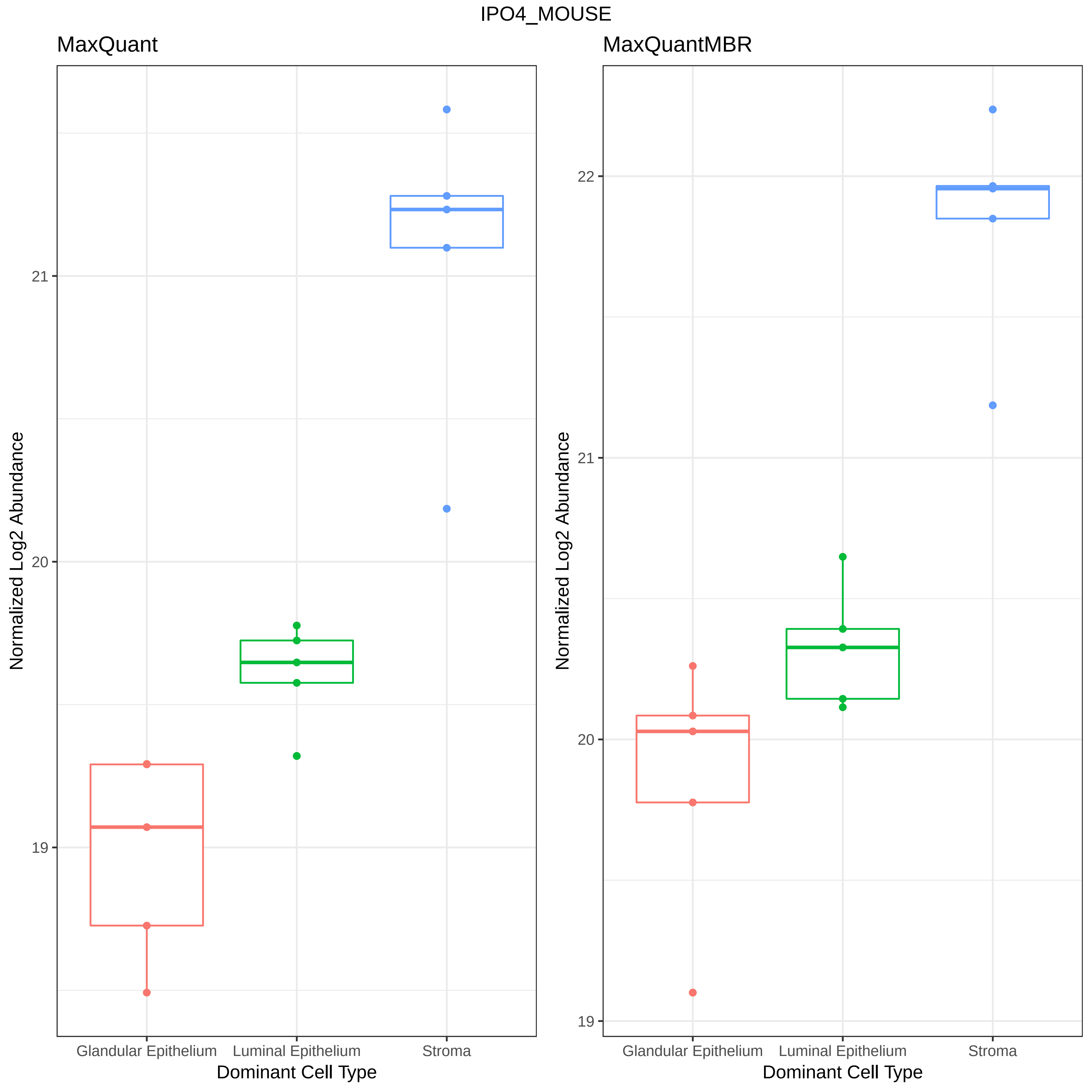

IPO4_MOUSE
MaxQuant
MaxQuantMBR
Normalized Log2 Abundance
21
20
19
22
Glandular Epithelium
Luminal Epithelium
Stroma
Dominant Cell Type

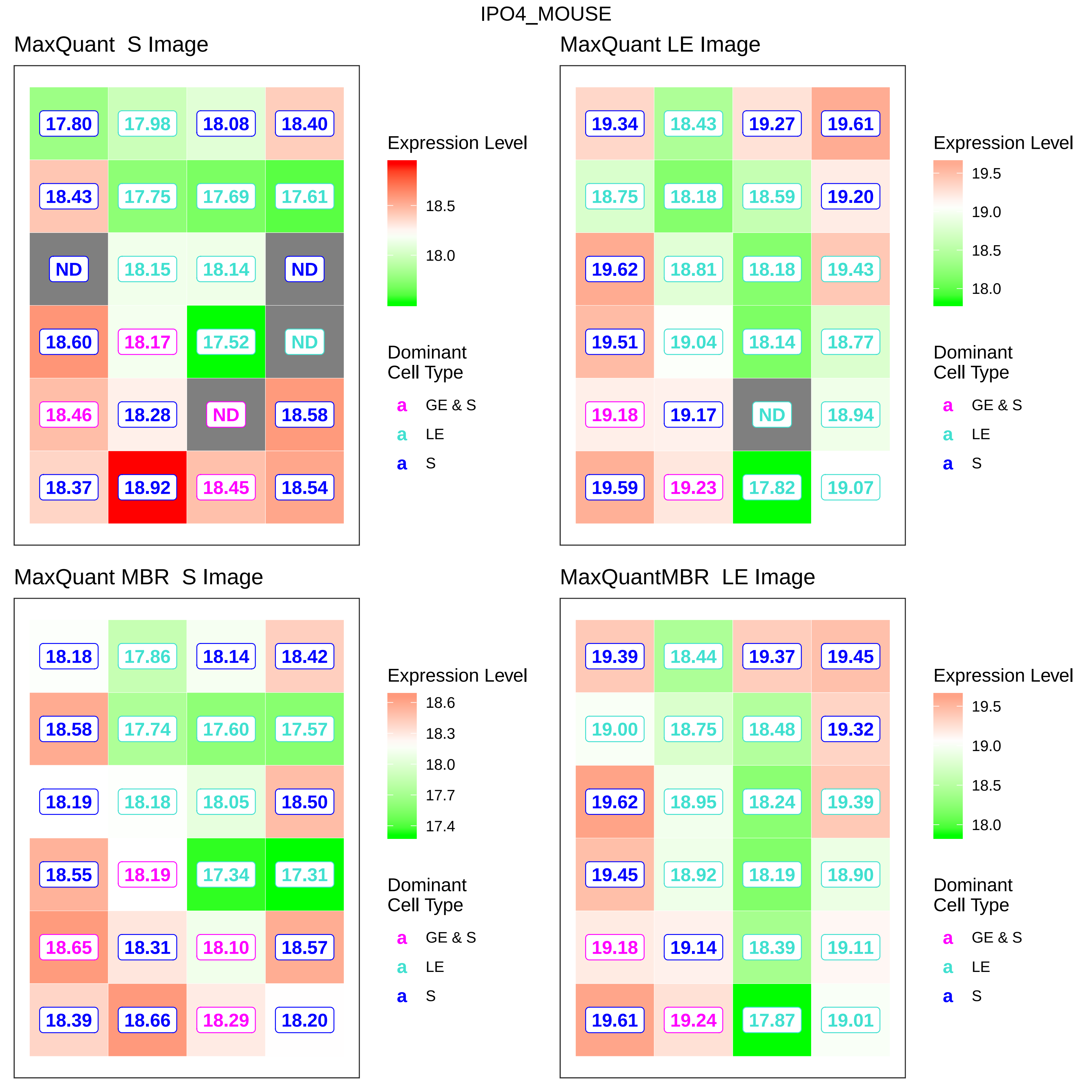
IPO4_MOUSE
MaxQuant S Image
17.80 17.98 18.08 18.40
18.43 17.75 17.69 17.61
ND 18.15 18.14 ND
18.60 18.17 17.52 ND
18.46 18.28 ND 18.58
18.37 18.92 18.45 18.54
Expression Level
18.5
18.0
Dominant Cell Type
a GE & S
a LE
a S
MaxQuant LE Image
19.34 18.43 19.27 19.61
18.75 18.18 18.59 19.20
19.62 18.81 18.18 19.43
19.51 19.04 18.14 18.77
19.18 19.17 ND 18.94
19.59 19.23 17.82 19.07
Expression Level
19.5
19.0
18.5
18.0
Dominant Cell Type
a GE & S
a LE
a S
MaxQuant MBR S Image
18.18 17.86 18.14 18.42
18.58 17.74 17.60 17.57
18.19 18.18 18.05 18.50
18.55 18.19 17.34 17.31
18.65 18.31 18.10 18.57
18.39 18.66 18.29 18.20
Expression Level
18.6
18.3
18.0
17.7
17.4
Dominant Cell Type
a GE & S
a LE
a S
MaxQuantMBR LE Image
19.39 18.44 19.37 19.45
19.00 18.75 18.48 19.32
19.62 18.95 18.24 19.39
19.45 18.92 18.19 18.90
19.18 19.14 18.39 19.11
19.61 19.24 17.87 19.01
Expression Level
19.5
19.0
18.5
18.0
Dominant Cell Type
a GE & S
a LE
a S

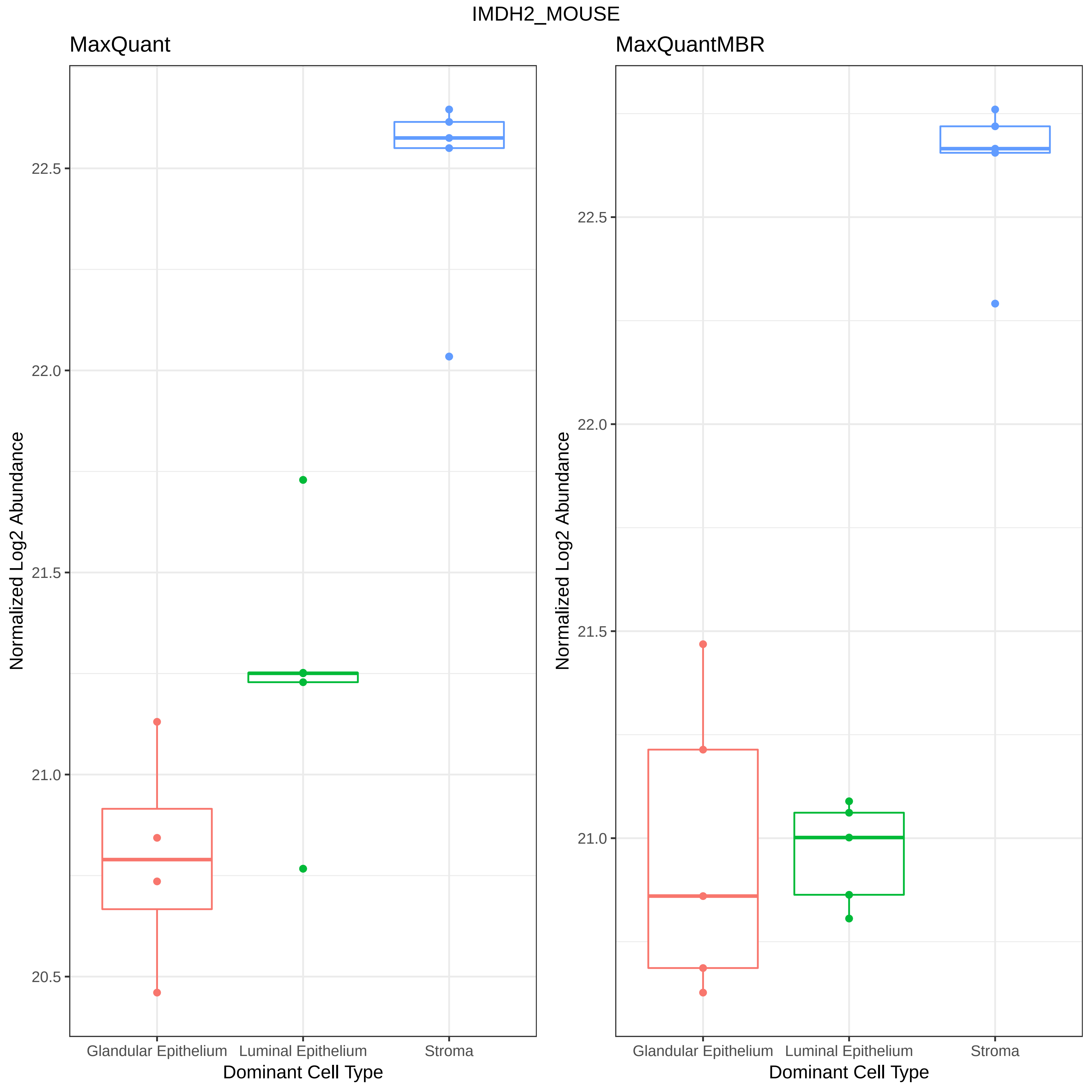
IMDH2_MOUSE
MaxQuant
MaxQuantMBR
Normalized Log2 Abundance
22.5
22.0
21.5
21.0
20.5
Glandular Epithelium
Luminal Epithelium
Stroma
Dominant Cell Type

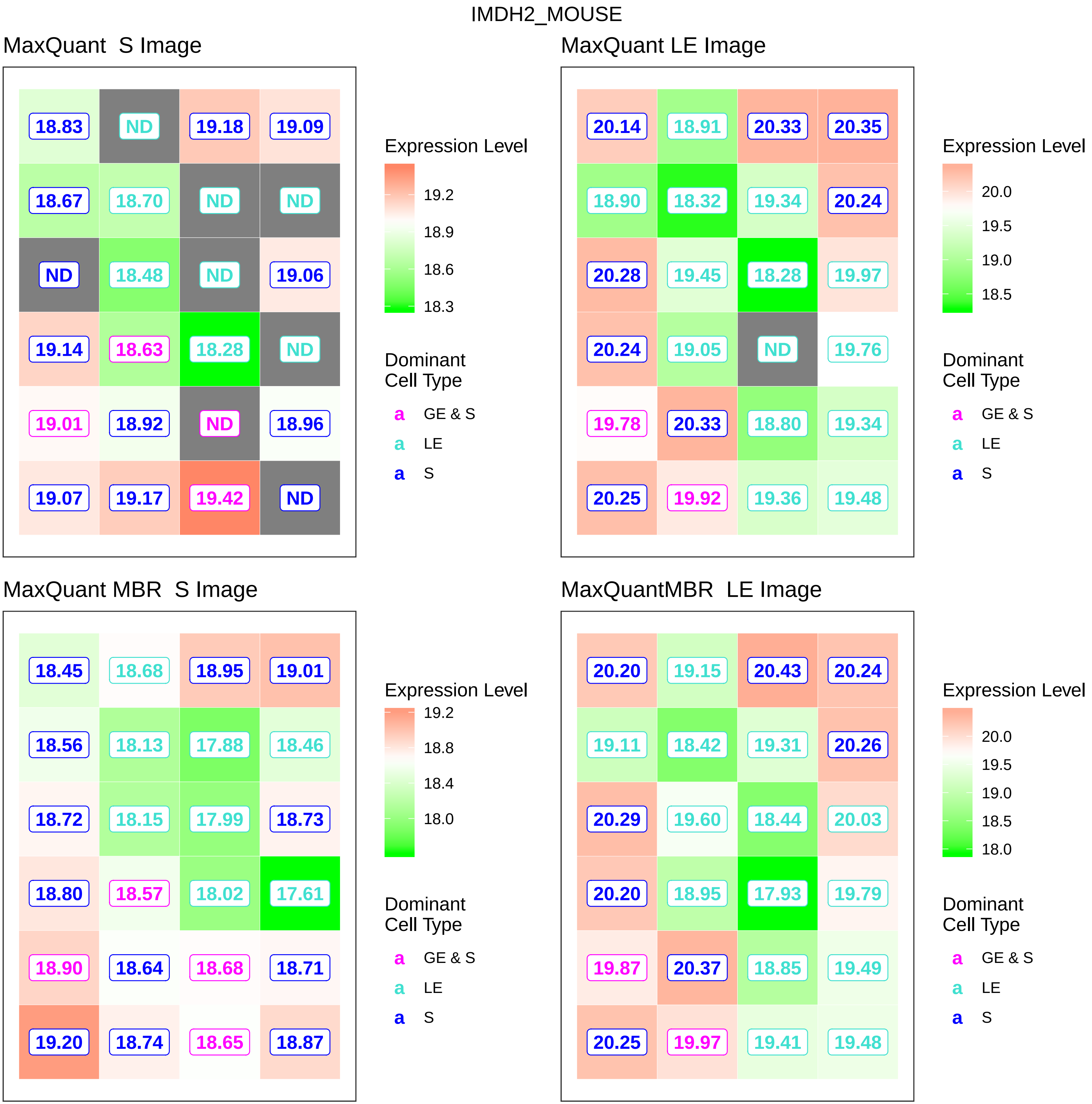
IMDH2_MOUSE
MaxQuant S Image
18.83 ND 19.18 19.09
18.67 18.70 ND ND
ND 18.48 ND 19.06
19.14 18.63 18.28 ND
19.01 18.92 ND 18.96
19.07 19.17 19.42 ND
Expression Level
19.2
18.9
18.6
18.3
Dominant Cell Type
a GE & S
a LE
a S
MaxQuant LE Image
20.14 18.91 20.33 20.35
18.90 18.32 19.34 20.24
20.28 19.45 18.28 19.97
20.24 19.05 ND 19.76
19.78 20.33 18.80 19.34
20.25 19.92 19.36 19.48
Expression Level
20.0
19.5
19.0
18.5
Dominant Cell Type
a GE & S
a LE
a S
MaxQuant MBR S Image
18.45 18.68 18.95 19.01
18.56 18.13 17.88 18.46
18.72 18.15 17.99 18.73
18.80 18.57 18.02 17.61
18.90 18.64 18.68 18.71
19.20 18.74 18.65 18.87
Expression Level
19.2
18.8
18.4
18.0
Dominant Cell Type
a GE & S
a LE
a S
MaxQuantMBR LE Image
20.20 19.15 20.43 20.24
19.11 18.42 19.31 20.26
20.29 19.60 18.44 20.03
20.20 18.95 17.93 19.79
19.87 20.37 18.85 19.49
20.25 19.97 19.41 19.48
Expression Level
20.0
19.5
19.0
18.5
18.0
Dominant Cell Type
a GE & S
a LE
a S

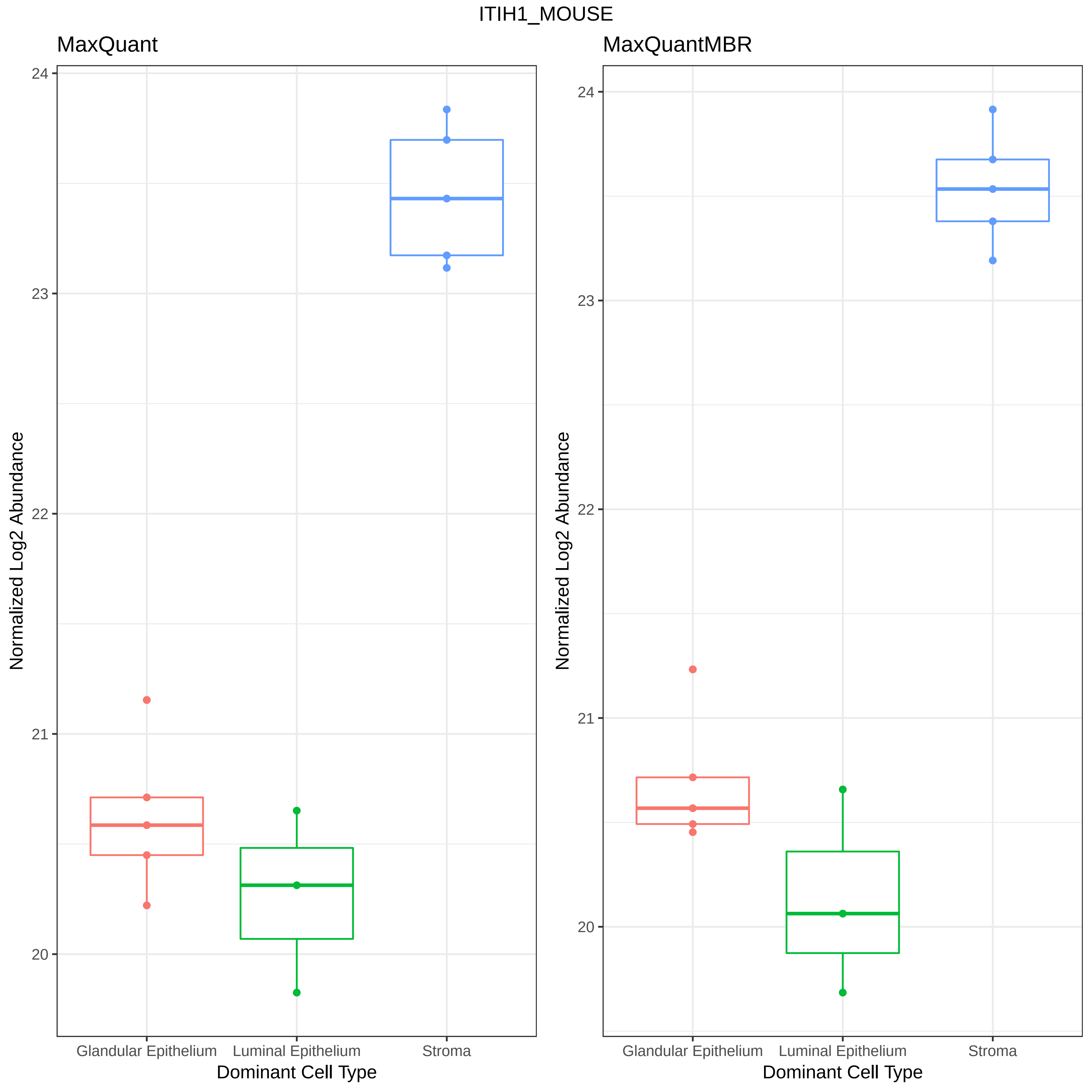

ITIH1_MOUSE
MaxQuant
MaxQuantMBR
Normalized Log2 Abundance
24
23
22
21
20
Glandular Epithelium
Luminal Epithelium
Stroma
Dominant Cell Type

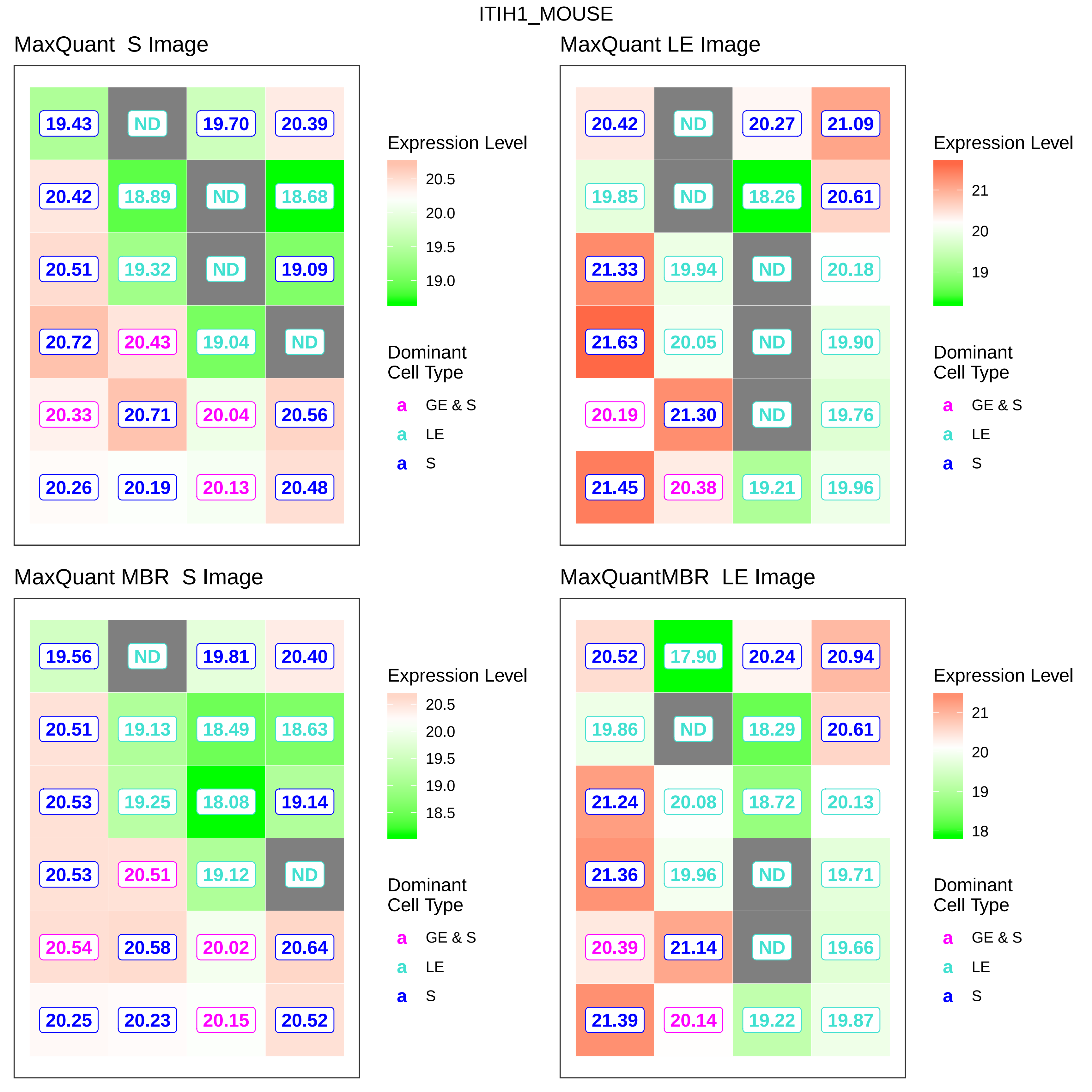
ITIH1_MOUSE
MaxQuant S Image
19.43 ND 19.70 20.39
20.42 18.89 ND 18.68
20.51 19.32 ND 19.09
20.72 20.43 19.04 ND
20.33 20.71 20.04 20.56
20.26 20.19 20.13 20.48
Expression Level
20.5
20.0
19.5
19.0
Dominant Cell Type
a GE & S
a LE
a S
MaxQuant LE Image
20.42 ND 20.27 21.09
19.85 ND 18.26 20.61
21.33 19.94 ND 20.18
21.63 20.05 ND 19.90
20.19 21.30 ND 19.76
21.45 20.38 19.21 19.96
Expression Level
21
20
19
Dominant Cell Type
a GE & S
a LE
a S
MaxQuant MBR S Image
19.56 ND 19.81 20.40
20.51 19.13 18.49 18.63
20.53 19.25 18.08 19.14
20.53 20.51 19.12 ND
20.54 20.58 20.02 20.64
20.25 20.23 20.15 20.52
Expression Level
20.5
20.0
19.5
19.0
18.5
Dominant Cell Type
a GE & S
a LE
a S
MaxQuantMBR LE Image
20.52 17.90 20.24 20.94
19.86 ND 18.29 20.61
21.24 20.08 18.72 20.13
21.36 19.96 ND 19.71
20.39 21.14 ND 19.66
21.39 20.14 19.22 19.87
Expression Level
21
20
19
18
Dominant Cell Type
a GE & S
a LE
a S

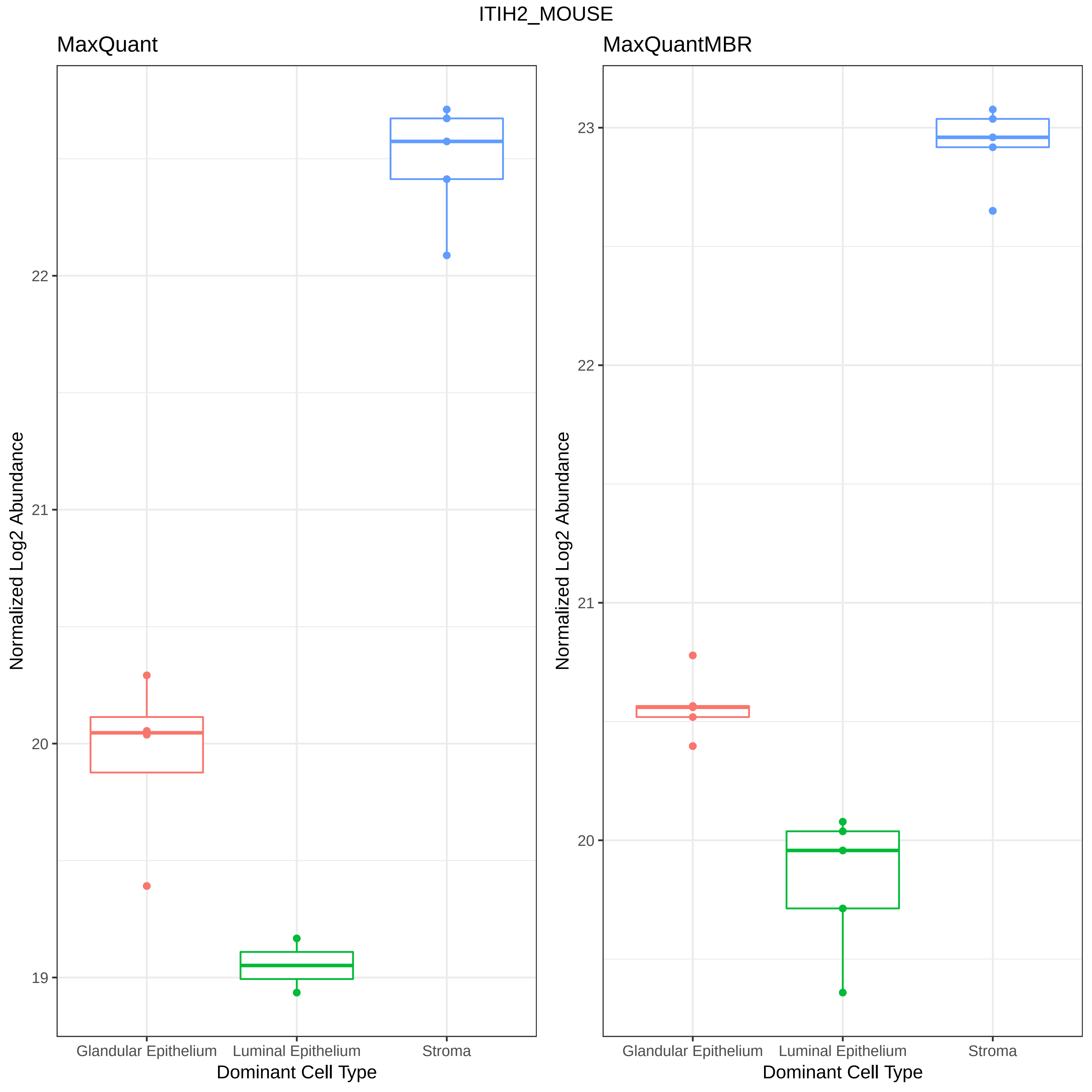

ITIH2_MOUSE
MaxQuant
MaxQuantMBR
Normalized Log2 Abundance
22
21
20
19
23
Glandular Epithelium
Luminal Epithelium
Stroma
Dominant Cell Type

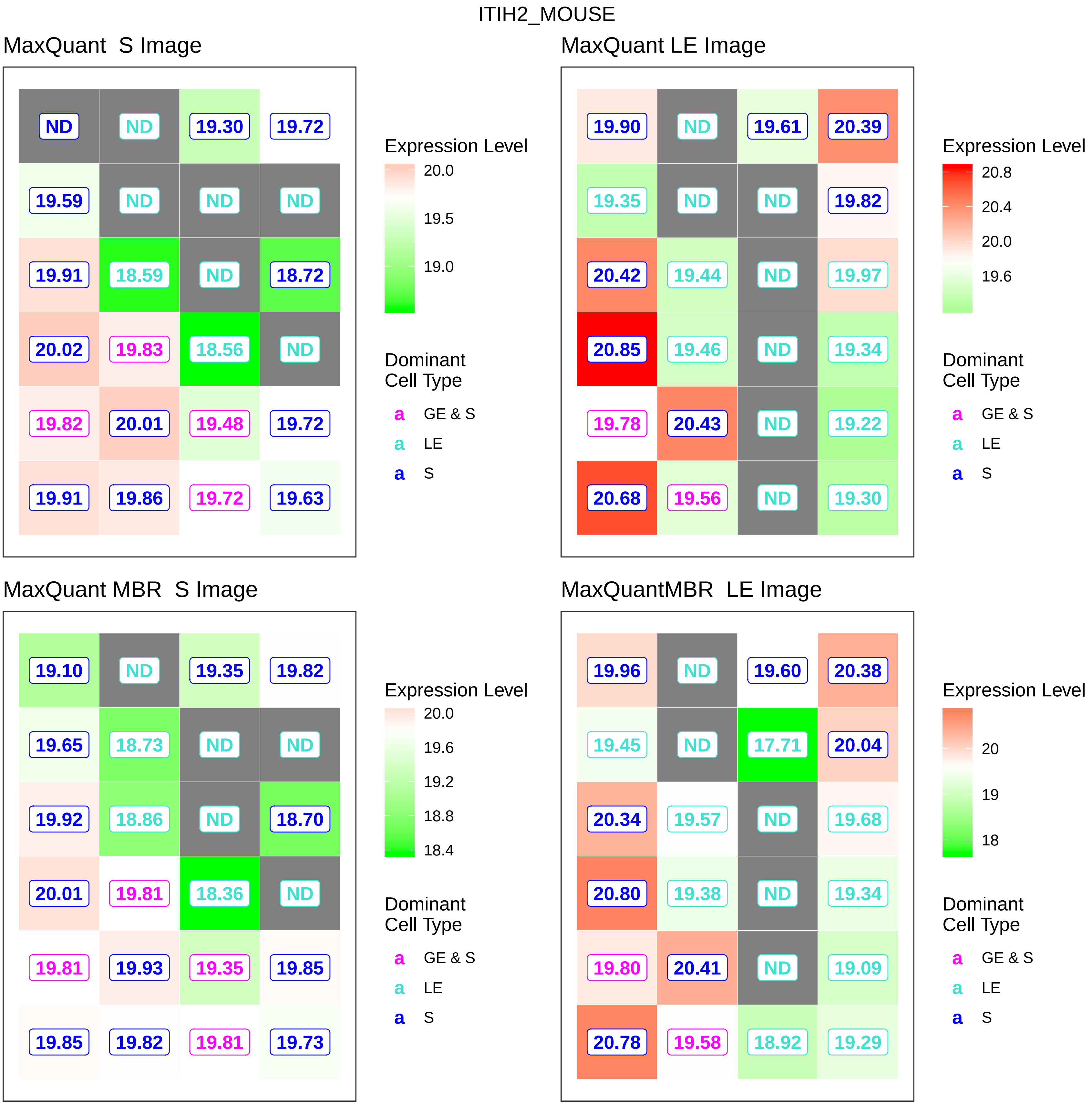
ITIH2_MOUSE
MaxQuant S Image
ND ND 19.30 19.72
19.59 ND ND ND
19.91 18.59 ND 18.72
20.02 19.83 18.56 ND
19.82 20.01 19.48 19.72
19.91 19.86 19.72 19.63
Expression Level
20.0
19.5
19.0
Dominant Cell Type
a GE & S
a LE
a S
MaxQuant LE Image
19.90 ND 19.61 20.39
19.35 ND ND 19.82
20.42 19.44 ND 19.97
20.85 19.46 ND 19.34
19.78 20.43 ND 19.22
20.68 19.56 ND 19.30
Expression Level
20.8
20.4
20.0
19.6
Dominant Cell Type
a GE & S
a LE
a S
MaxQuant MBR S Image
19.10 ND 19.35 19.82
19.65 18.73 ND ND
19.92 18.86 ND 18.70
20.01 19.81 18.36 ND
19.81 19.93 19.35 19.85
19.85 19.82 19.81 19.73
Expression Level
20.0
19.6
19.2
18.8
18.4
Dominant Cell Type
a GE & S
a LE
a S
MaxQuantMBR LE Image
19.96 ND 19.60 20.38
19.45 ND 17.71 20.04
20.34 19.57 ND 19.68
20.80 19.38 ND 19.34
19.80 20.41 ND 19.09
20.78 19.58 18.92 19.29
Expression Level
20
19
18
Dominant Cell Type
a GE & S
a LE
a S

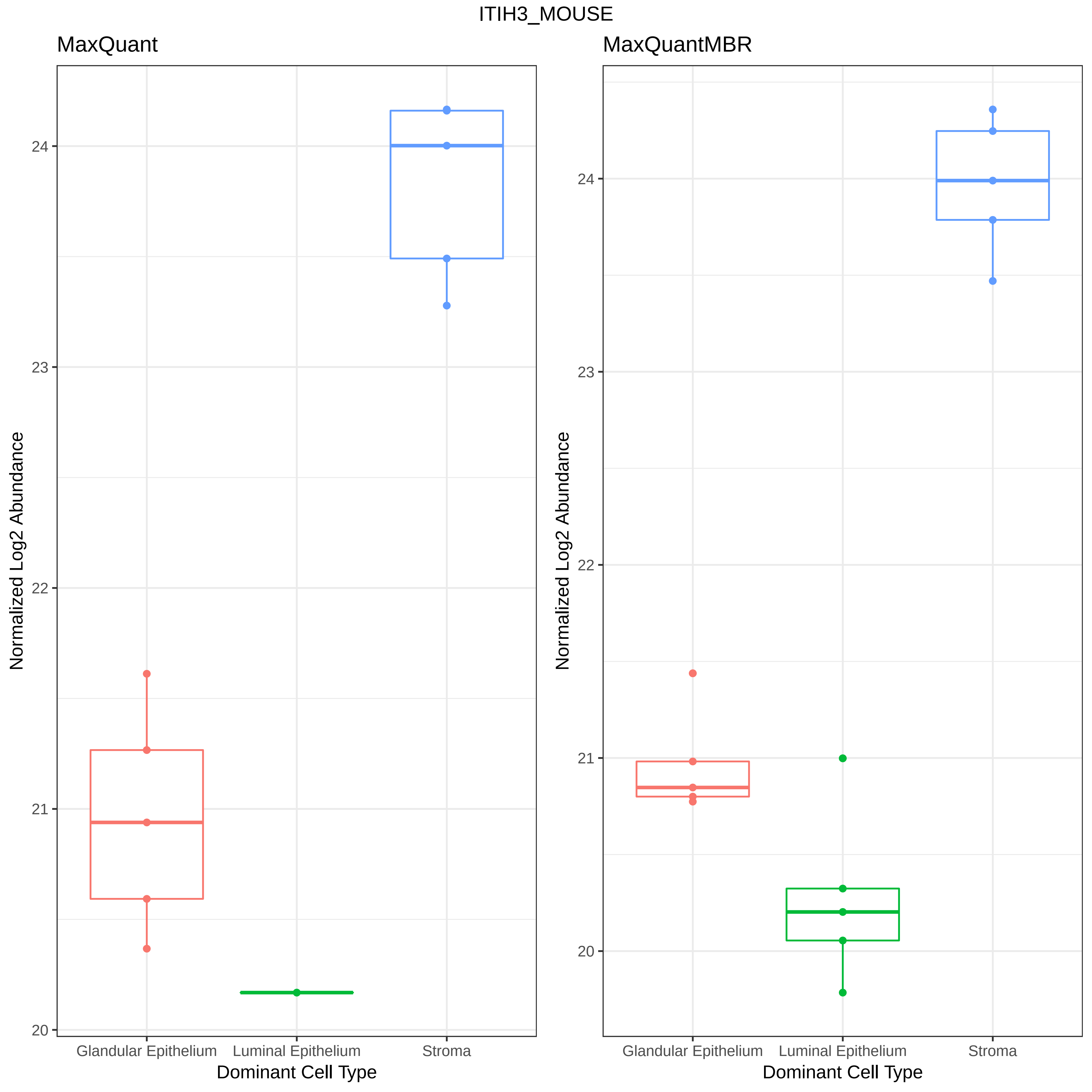

ITIH3_MOUSE
MaxQuant
MaxQuantMBR
Normalized Log2 Abundance
24
23
22
21
20
Glandular Epithelium
Luminal Epithelium
Stroma
Dominant Cell Type

ITIH3_MOUSE

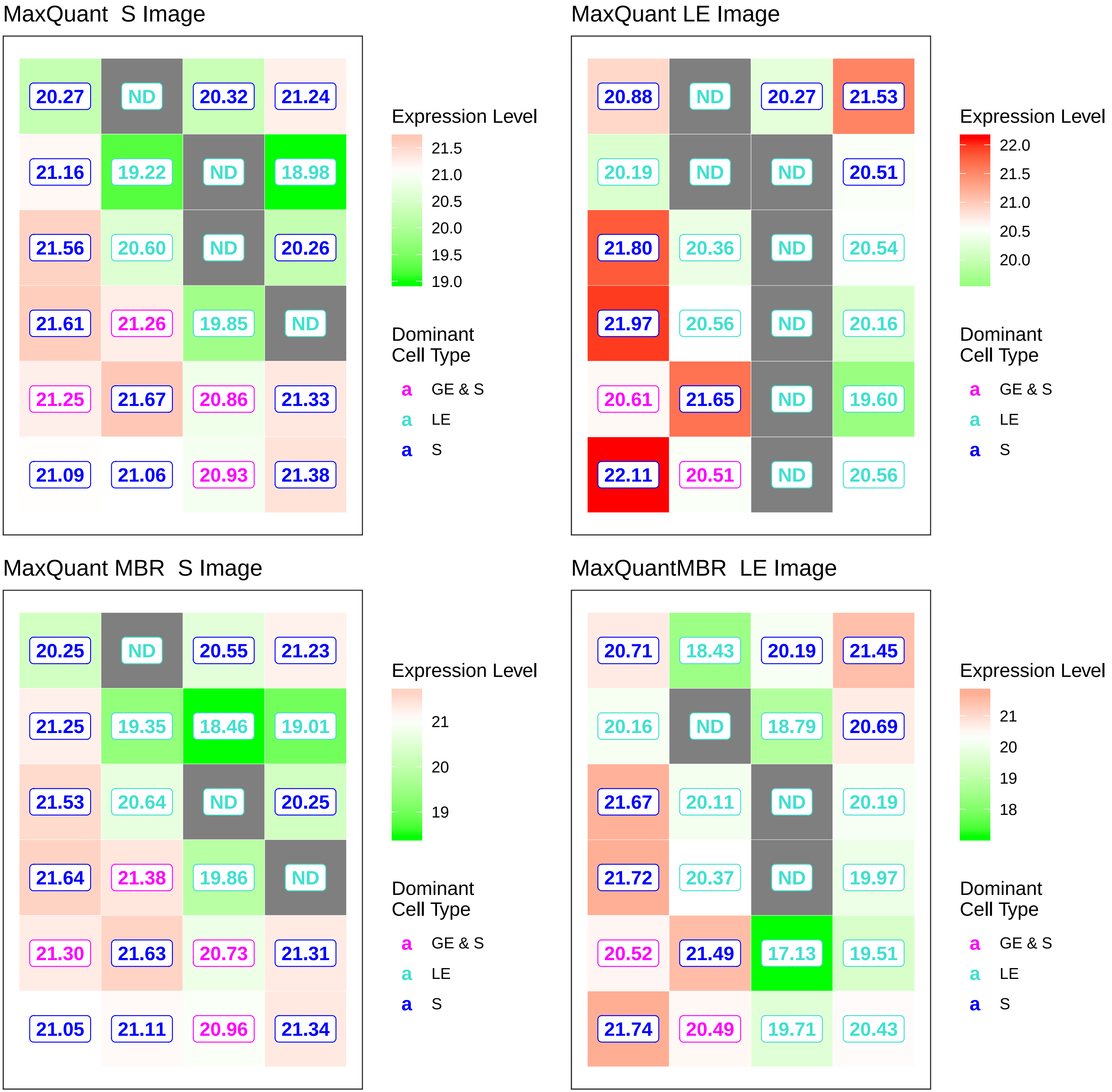

MaxQuant S Image
20.27 ND 20.32 21.24
21.16 19.22 ND 18.98
21.56 20.60 ND 20.26
21.61 21.26 19.85 ND
21.25 21.67 20.86 21.33
21.09 21.06 20.93 21.38
Expression Level
21.5
21.0
20.5
20.0
19.5
19.0
Dominant Cell Type
a GE & S
a LE
a S
MaxQuant LE Image
20.88 ND 20.27 21.53
20.19 ND ND 20.51
21.80 20.36 ND 20.54
21.97 20.56 ND 20.16
20.61 21.65 ND 19.60
22.11 20.51 ND 20.56
Expression Level
22.0
21.5
21.0
20.5
20.0
Dominant Cell Type
a GE & S
a LE
a S
MaxQuant MBR S Image
20.25 ND 20.55 21.23
21.25 19.35 18.46 19.01
21.53 20.64 ND 20.25
21.64 21.38 19.86 ND
21.30 21.63 20.73 21.31
21.05 21.11 20.96 21.34
Expression Level
21
20
19
Dominant Cell Type
a GE & S
a LE
a S
MaxQuantMBR LE Image
20.71 18.43 20.19 21.45
20.16 ND 18.79 20.69
21.67 20.11 ND 20.19
21.72 20.37 ND 19.97
20.52 21.49 17.13 19.51
21.74 20.49 19.71 20.43
Expression Level
21
20
19
18
Dominant Cell Type
a GE & S
a LE
a S

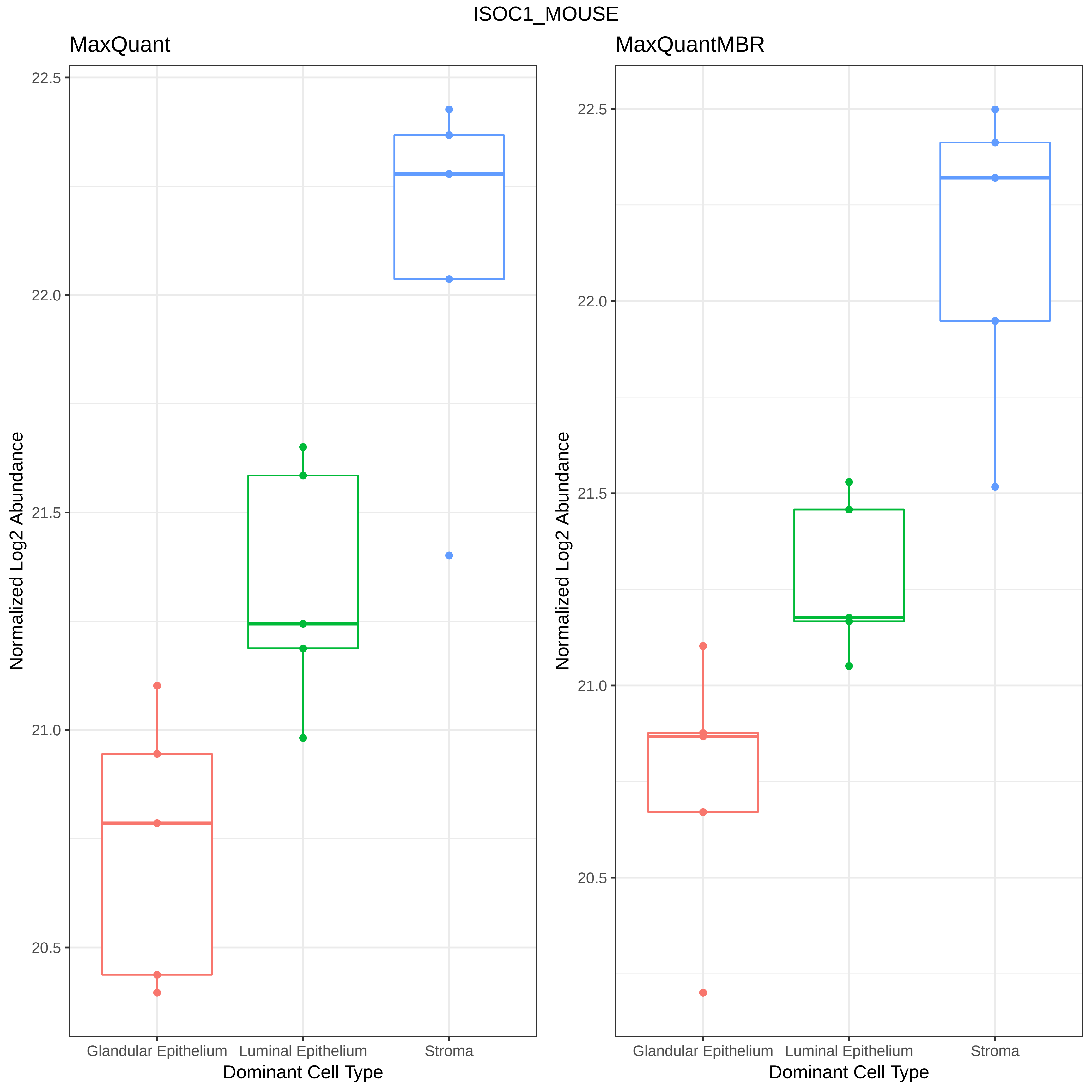
ISOC1_MOUSE
MaxQuant
MaxQuantMBR
Normalized Log2 Abundance
Dominant Cell Type
Glandular Epithelium
Luminal Epithelium
Stroma
20.5
21.0
21.5
22.0
22.5

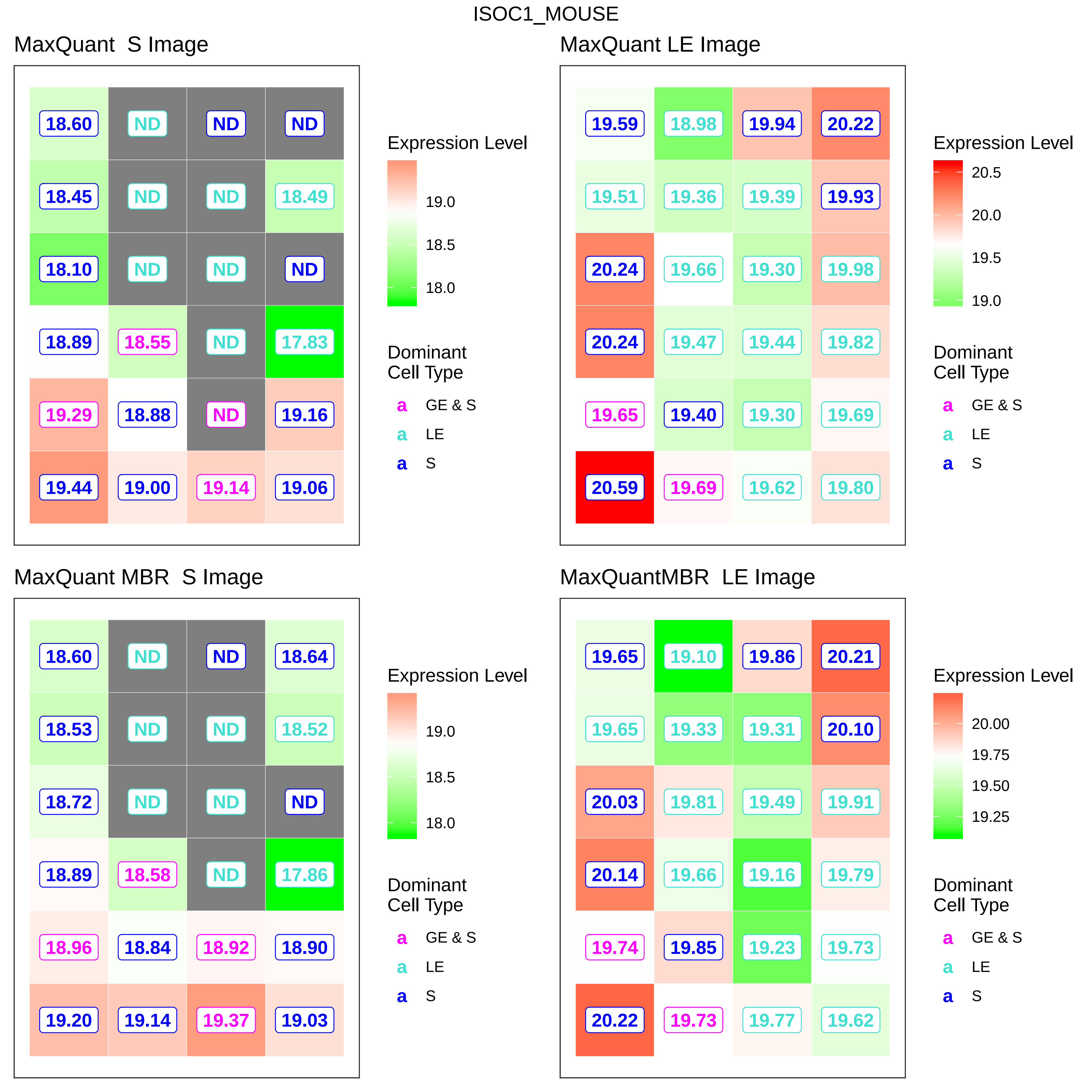
ISOC1_MOUSE
MaxQuant S Image
18.60 ND ND ND
18.45 ND ND 18.49
18.10 ND ND ND
18.89 18.55 ND 17.83
19.29 18.88 ND 19.16
19.44 19.00 19.14 19.06
Expression Level
19.0
18.5
18.0
Dominant Cell Type
a GE & S
a LE
a S
MaxQuant LE Image
19.59 18.98 19.94 20.22
19.51 19.36 19.39 19.93
20.24 19.66 19.30 19.98
20.24 19.47 19.44 19.82
19.65 19.40 19.30 19.69
20.59 19.69 19.62 19.80
Expression Level
20.5
20.0
19.5
19.0
Dominant Cell Type
a GE & S
a LE
a S
MaxQuant MBR S Image
18.60 ND ND 18.64
18.53 ND ND 18.52
18.72 ND ND ND
18.89 18.58 ND 17.86
18.96 18.84 18.92 18.90
19.20 19.14 19.37 19.03
Expression Level
19.0
18.5
18.0
Dominant Cell Type
a GE & S
a LE
a S
MaxQuantMBR LE Image
19.65 19.10 19.86 20.21
19.65 19.33 19.31 20.10
20.03 19.81 19.49 19.91
20.14 19.66 19.16 19.79
19.74 19.85 19.23 19.73
20.22 19.73 19.77 19.62
Expression Level
20.00
19.75
19.50
19.25
Dominant Cell Type
a GE & S
a LE
a S

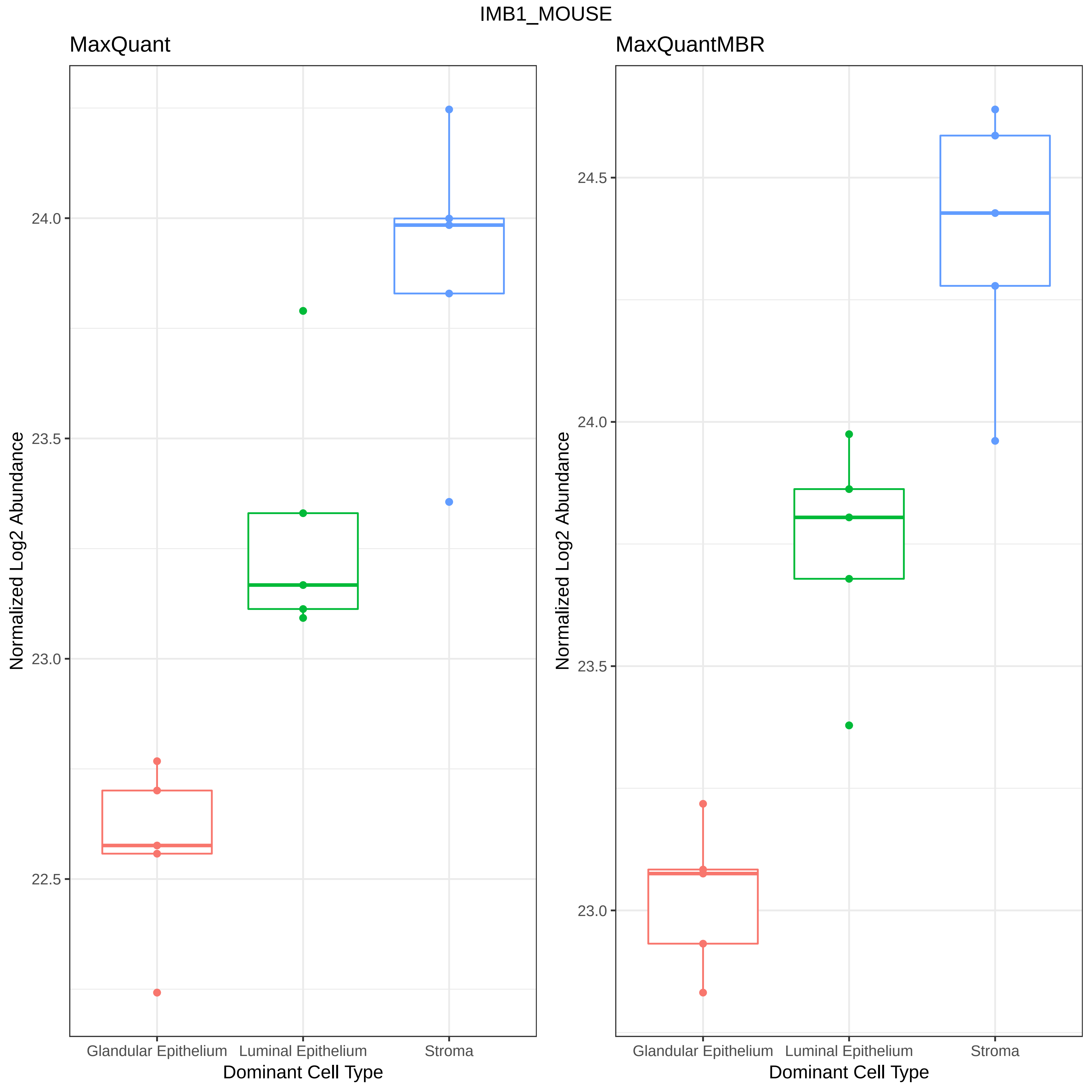
IMB1_MOUSE
MaxQuant
MaxQuantMBR
Normalized Log2 Abundance
24.0
23.5
23.0
22.5
24.5
24.0
23.5
23.0
Glandular Epithelium
Luminal Epithelium
Stroma
Dominant Cell Type

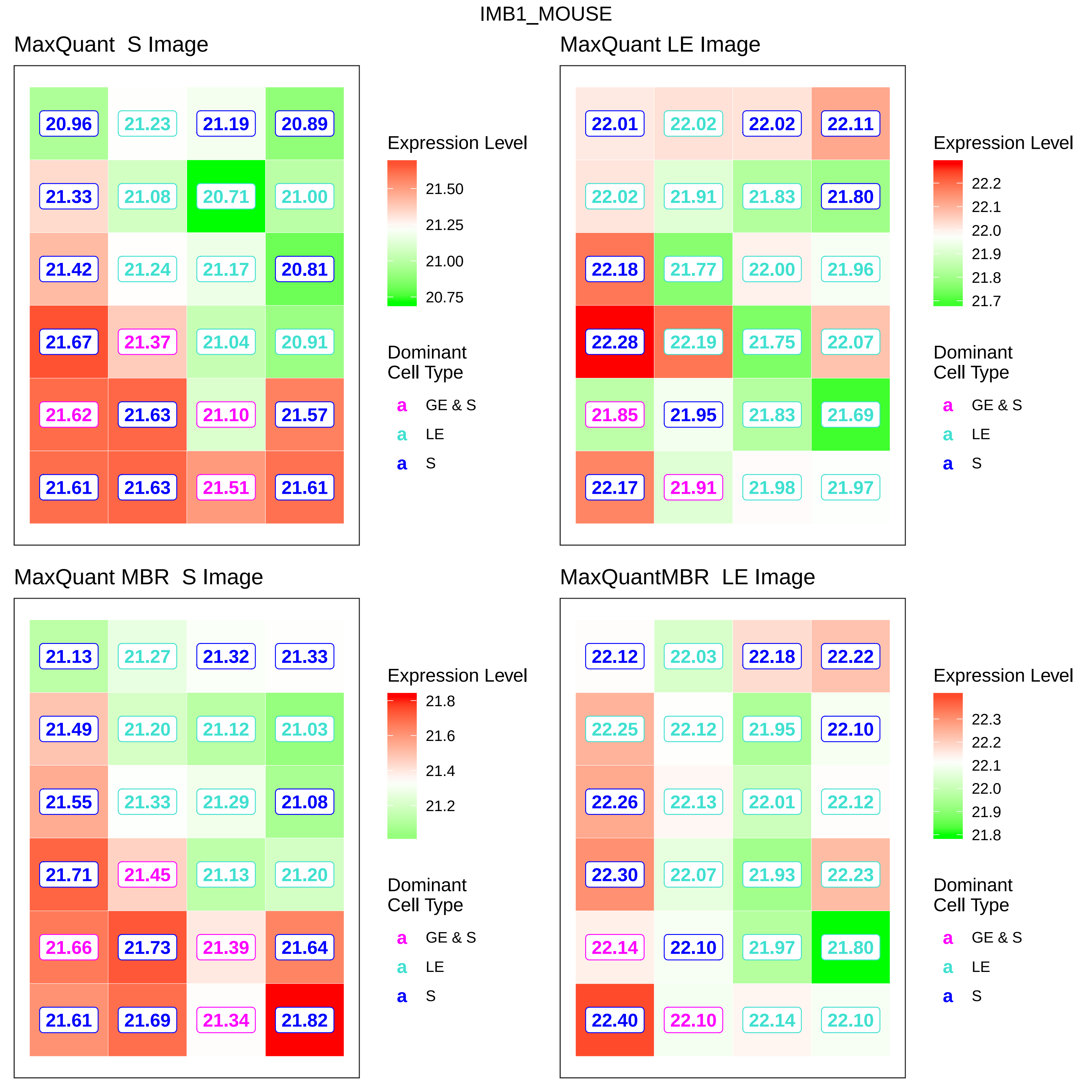
IMB1_MOUSE
MaxQuant S Image
20.96 21.23 21.19 20.89
21.33 21.08 20.71 21.00
21.42 21.24 21.17 20.81
21.67 21.37 21.04 20.91
21.62 21.63 21.10 21.57
21.61 21.63 21.51 21.61
Expression Level
21.50
21.25
21.00
20.75
Dominant Cell Type
a GE & S
a LE
a S
MaxQuant LE Image
22.01 22.02 22.02 22.11
22.02 21.91 21.83 21.80
22.18 21.77 22.00 21.96
22.28 22.19 21.75 22.07
21.85 21.95 21.83 21.69
22.17 21.91 21.98 21.97
Expression Level
22.2
22.1
22.0
21.9
21.8
21.7
Dominant Cell Type
a GE & S
a LE
a S
MaxQuant MBR S Image
21.13 21.27 21.32 21.33
21.49 21.20 21.12 21.03
21.55 21.33 21.29 21.08
21.71 21.45 21.13 21.20
21.66 21.73 21.39 21.64
21.61 21.69 21.34 21.82
Expression Level
21.8
21.6
21.4
21.2
Dominant Cell Type
a GE & S
a LE
a S
MaxQuantMBR LE Image
22.12 22.03 22.18 22.22
22.25 22.12 21.95 22.10
22.26 22.13 22.01 22.12
22.30 22.07 21.93 22.23
22.14 22.10 21.97 21.80
22.40 22.10 22.14 22.10
Expression Level
22.3
22.2
22.1
22.0
21.9
21.8
Dominant Cell Type
a GE & S
a LE
a S

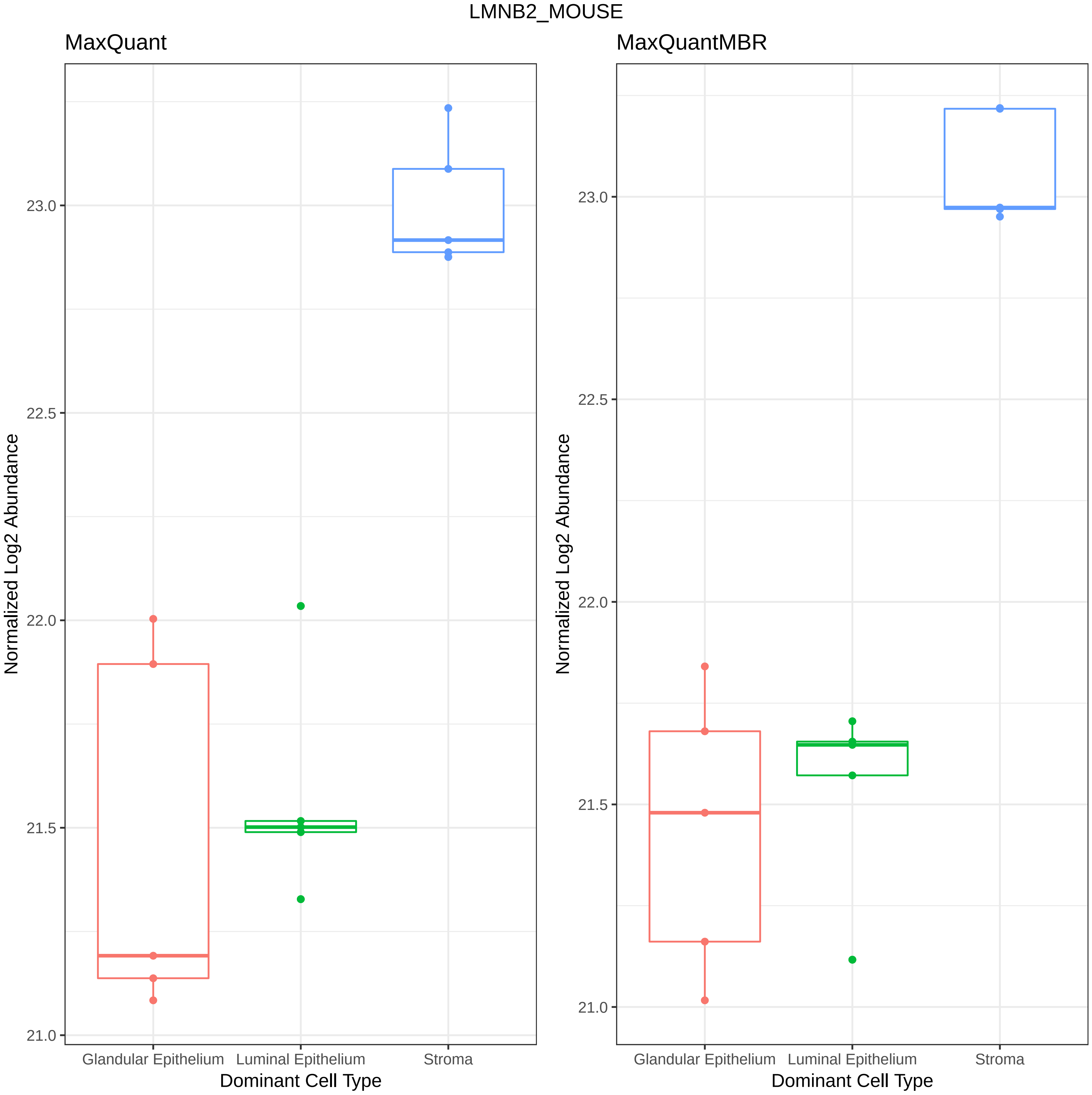

LMNB2_MOUSE
MaxQuant
MaxQuantMBR
Normalized Log2 Abundance
23.0
22.5
22.0
21.5
21.0
Glandular Epithelium
Luminal Epithelium
Stroma
Dominant Cell Type

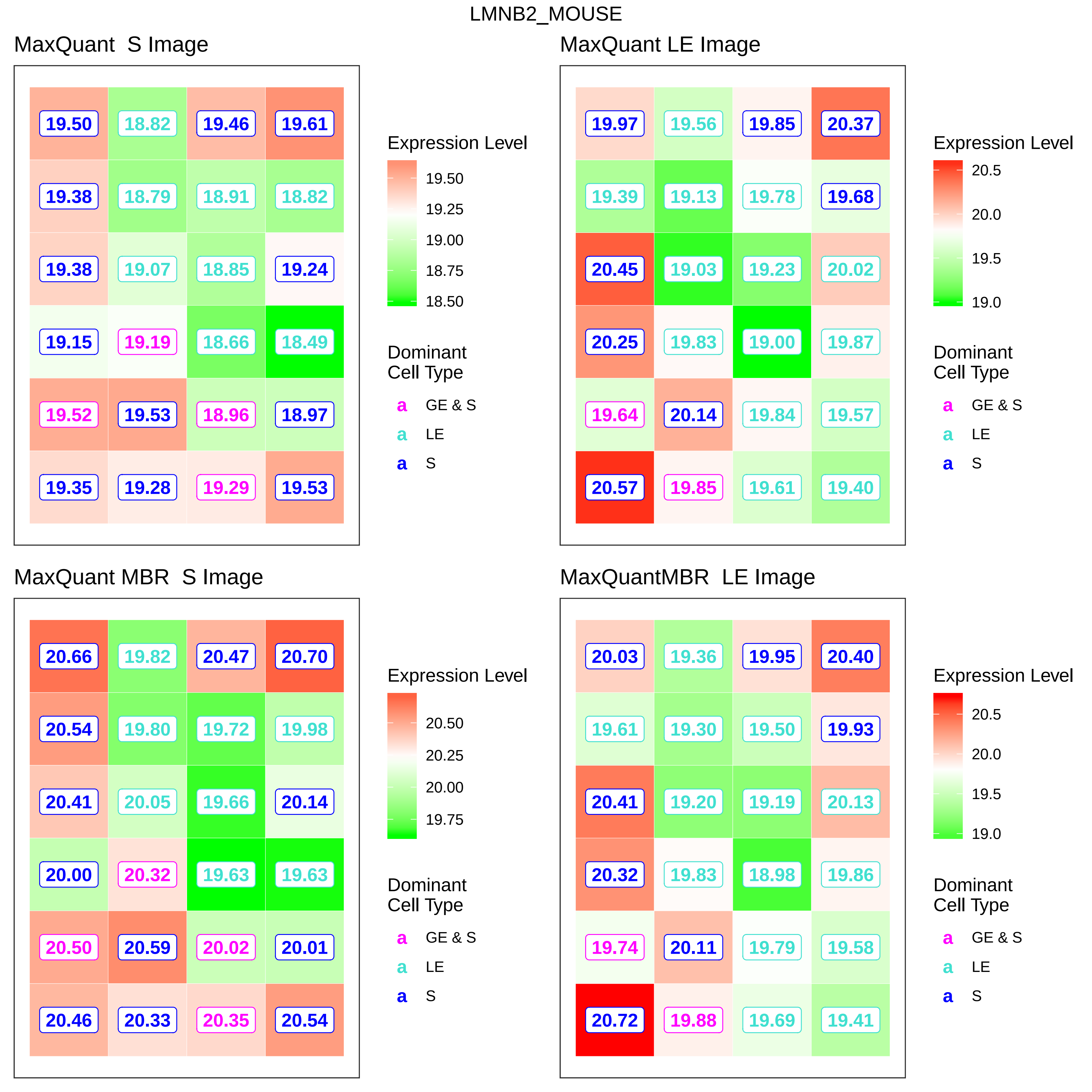
LMNB2_MOUSE
MaxQuant S Image
19.50 18.82 19.46 19.61
19.38 18.79 18.91 18.82
19.38 19.07 18.85 19.24
19.15 19.19 18.66 18.49
19.52 19.53 18.96 18.97
19.35 19.28 19.29 19.53
Expression Level
19.50
19.25
19.00
18.75
18.50
Dominant Cell Type
a GE & S
a LE
a S
MaxQuant LE Image
19.97 19.56 19.85 20.37
19.39 19.13 19.78 19.68
20.45 19.03 19.23 20.02
20.25 19.83 19.00 19.87
19.64 20.14 19.84 19.57
20.57 19.85 19.61 19.40
Expression Level
20.5
20.0
19.5
19.0
Dominant Cell Type
a GE & S
a LE
a S
MaxQuant MBR S Image
20.66 19.82 20.47 20.70
20.54 19.80 19.72 19.98
20.41 20.05 19.66 20.14
20.00 20.32 19.63 19.63
20.50 20.59 20.02 20.01
20.46 20.33 20.35 20.54
Expression Level
20.50
20.25
20.00
19.75
Dominant Cell Type
a GE & S
a LE
a S
MaxQuantMBR LE Image
20.03 19.36 19.95 20.40
19.61 19.30 19.50 19.93
20.41 19.20 19.19 20.13
20.32 19.83 18.98 19.86
19.74 20.11 19.79 19.58
20.72 19.88 19.69 19.41
Expression Level
20.5
20.0
19.5
19.0
Dominant Cell Type
a GE & S
a LE
a S

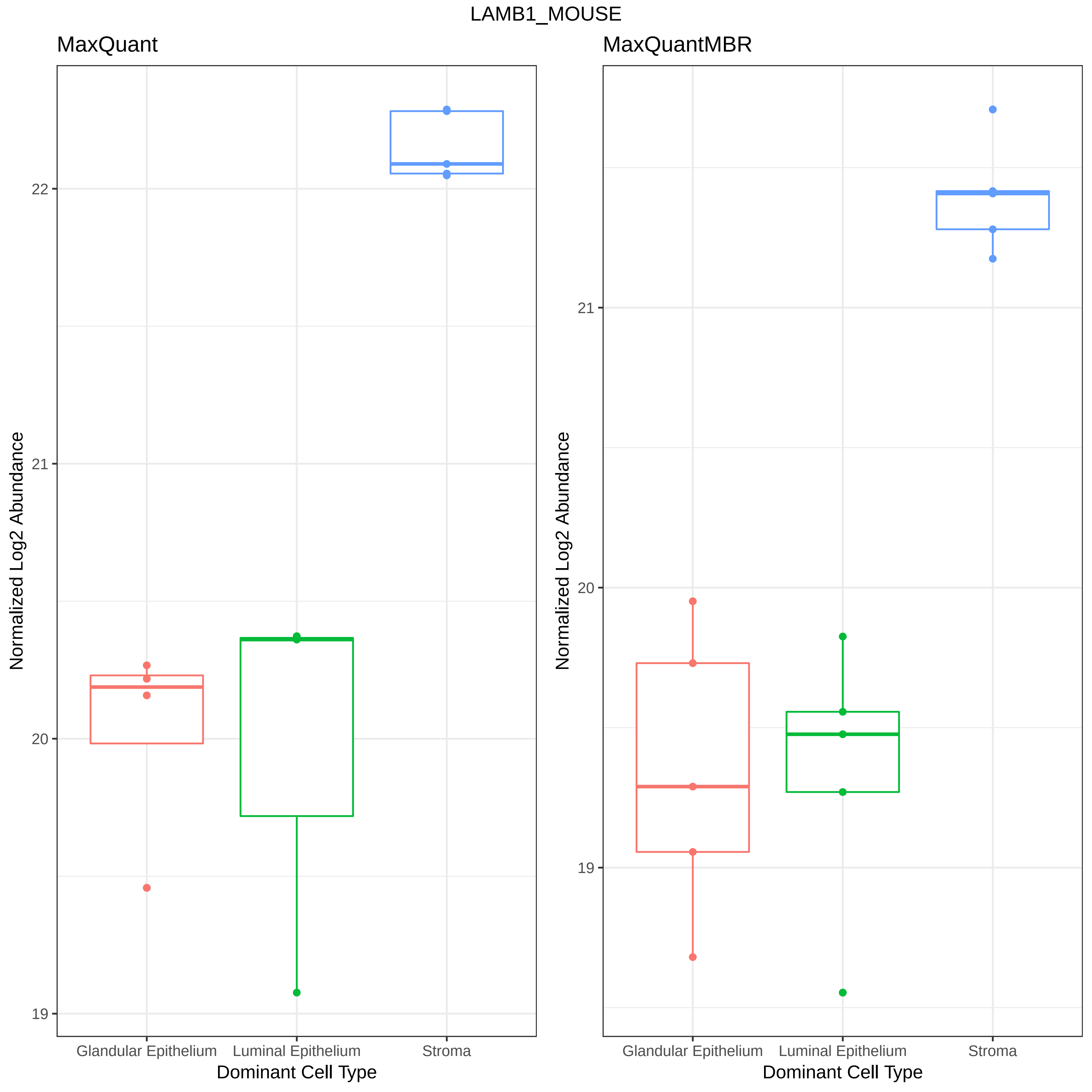

LAMB1_MOUSE
MaxQuant
MaxQuantMBR
Normalized Log2 Abundance
22
21
20
19
Glandular Epithelium
Luminal Epithelium
Stroma
Dominant Cell Type

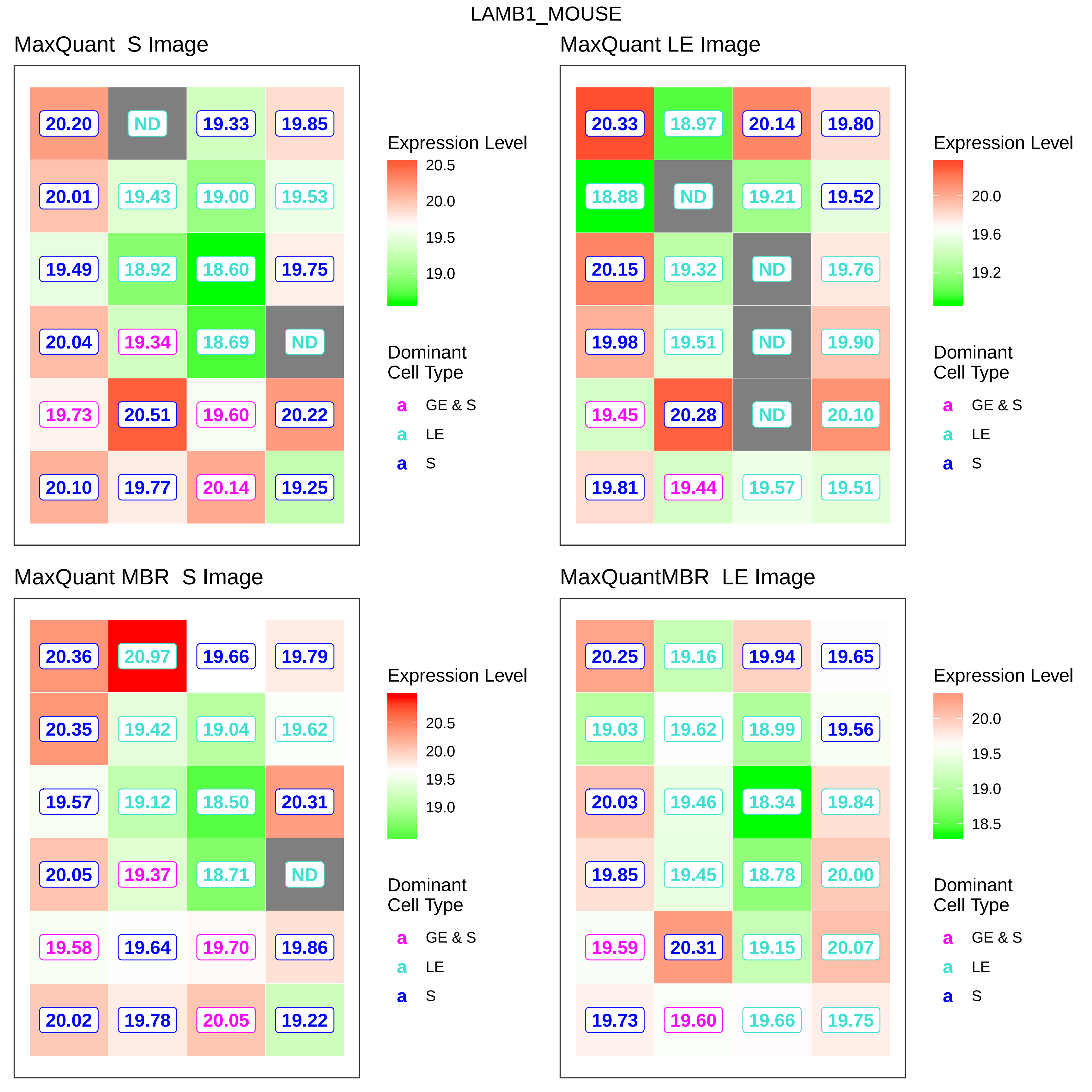
LAMB1_MOUSE
MaxQuant S Image
20.20 ND 19.33 19.85
20.01 19.43 19.00 19.53
19.49 18.92 18.60 19.75
20.04 19.34 18.69 ND
19.73 20.51 19.60 20.22
20.10 19.77 20.14 19.25
Expression Level
20.5
20.0
19.5
19.0
Dominant Cell Type
a GE & S
a LE
a S
MaxQuant LE Image
20.33 18.97 20.14 19.80
18.88 ND 19.21 19.52
20.15 19.32 ND 19.76
19.98 19.51 ND 19.90
19.45 20.28 ND 20.10
19.81 19.44 19.57 19.51
Expression Level
20.0
19.6
19.2
Dominant Cell Type
a GE & S
a LE
a S
MaxQuant MBR S Image
20.36 20.97 19.66 19.79
20.35 19.42 19.04 19.62
19.57 19.12 18.50 20.31
20.05 19.37 18.71 ND
19.58 19.64 19.70 19.86
20.02 19.78 20.05 19.22
Expression Level
20.5
20.0
19.5
19.0
Dominant Cell Type
a GE & S
a LE
a S
MaxQuantMBR LE Image
20.25 19.16 19.94 19.65
19.03 19.62 18.99 19.56
20.03 19.46 18.34 19.84
19.85 19.45 18.78 20.00
19.59 20.31 19.15 20.07
19.73 19.60 19.66 19.75
Expression Level
20.0
19.5
19.0
18.5
Dominant Cell Type
a GE & S
a LE
a S

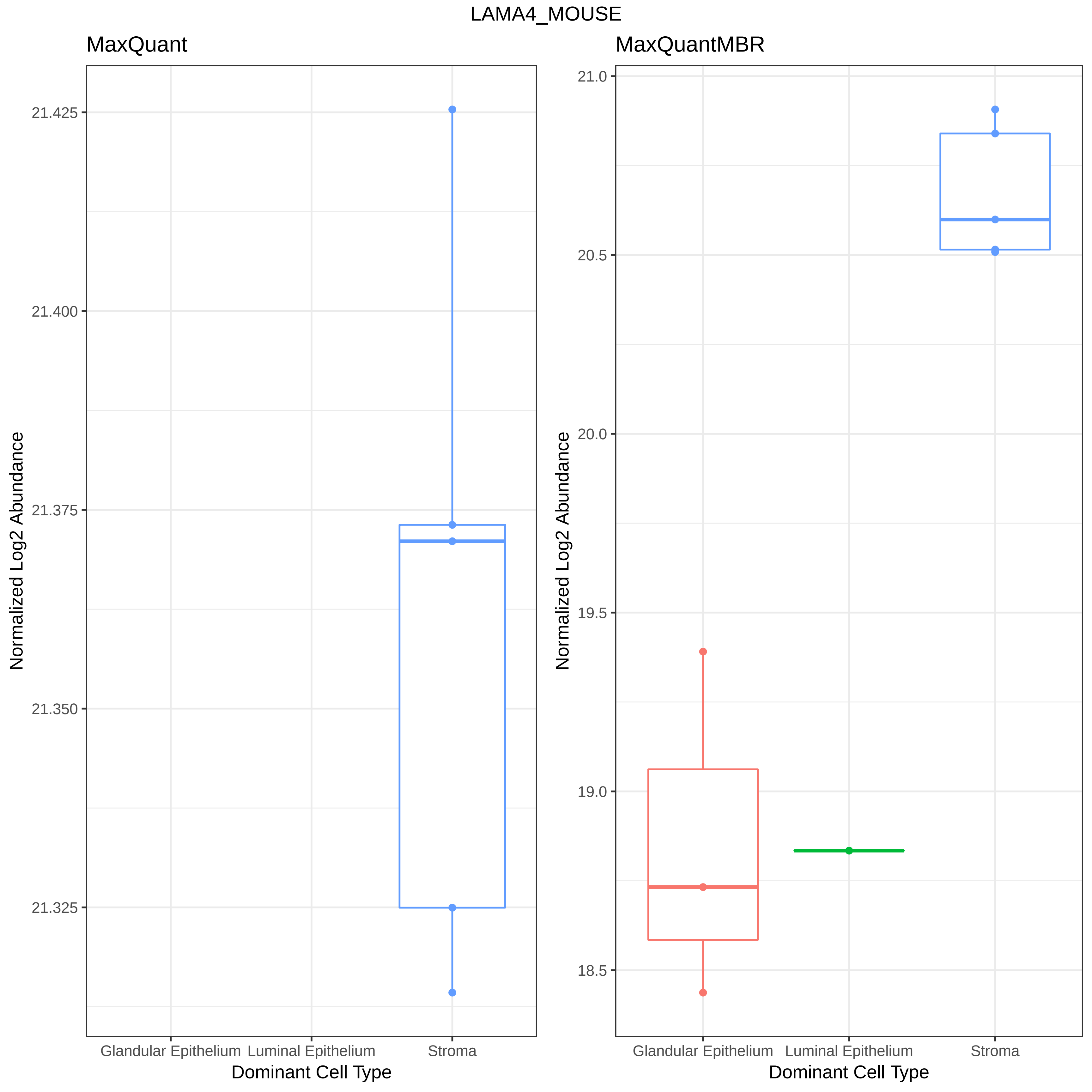

LAMA4_MOUSE
MaxQuant
MaxQuantMBR
21.425
21.400
21.375
21.350
21.325
21.0
20.5
20.0
19.5
19.0
18.5
Normalized Log2 Abundance
Normalized Log2 Abundance
Glandular Epithelium
Luminal Epithelium
Stroma
Glandular Epithelium
Luminal Epithelium
Stroma
Dominant Cell Type
Dominant Cell Type

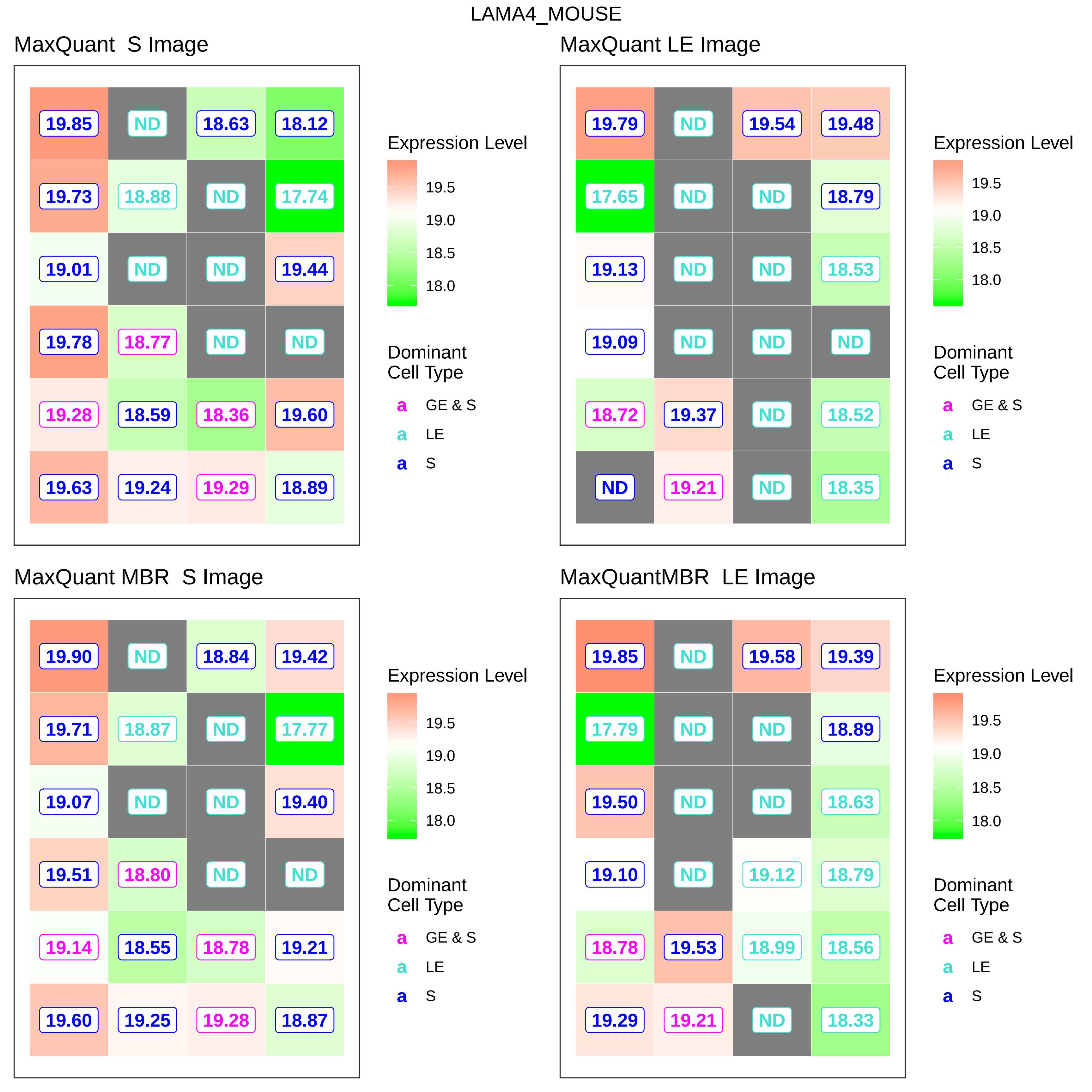

LAMA4_MOUSE
MaxQuant S Image
19.85 ND 18.63 18.12
19.73 18.88 ND 17.74
19.01 ND ND 19.44
19.78 18.77 ND ND
19.28 18.59 18.36 19.60
19.63 19.24 19.29 18.89
Expression Level
19.5
19.0
18.5
18.0
Dominant Cell Type
a GE & S
a LE
a S
MaxQuant LE Image
19.79 ND 19.54 19.48
17.65 ND ND 18.79
19.13 ND ND 18.53
19.09 ND ND ND
18.72 19.37 ND 18.52
ND 19.21 ND 18.35
Expression Level
19.5
19.0
18.5
18.0
Dominant Cell Type
a GE & S
a LE
a S
MaxQuant MBR S Image
19.90 ND 18.84 19.42
19.71 18.87 ND 17.77
19.07 ND ND 19.40
19.51 18.80 ND ND
19.14 18.55 18.78 19.21
19.60 19.25 19.28 18.87
Expression Level
19.5
19.0
18.5
18.0
Dominant Cell Type
a GE & S
a LE
a S
MaxQuantMBR LE Image
19.85 ND 19.58 19.39
17.79 ND ND 18.89
19.50 ND ND 18.63
19.10 ND 19.12 18.79
18.78 19.53 18.99 18.56
19.29 19.21 ND 18.33
Expression Level
19.5
19.0
18.5
18.0
Dominant Cell Type
a GE & S
a LE
a S

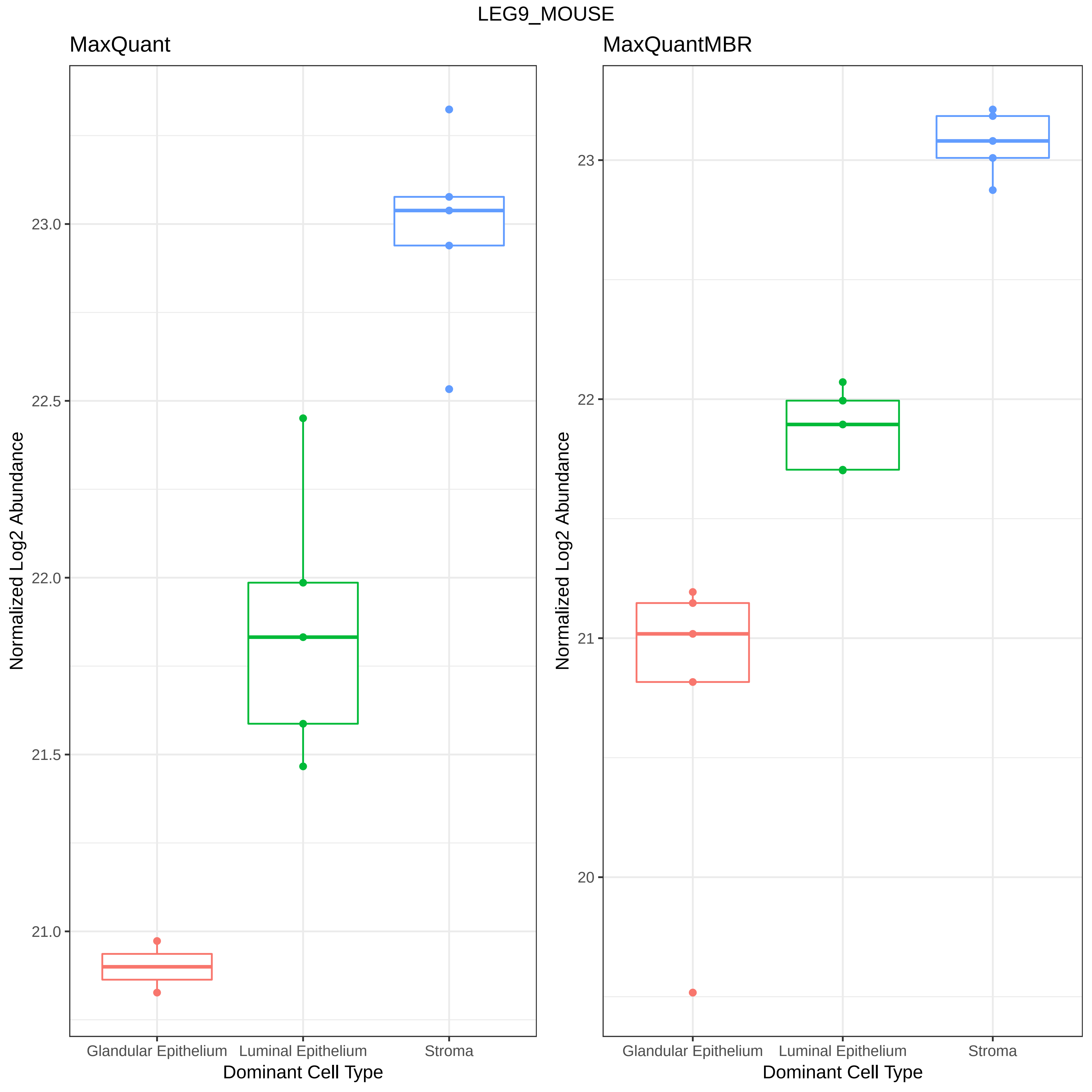
LEG9_MOUSE
MaxQuant
MaxQuantMBR
Normalized Log2 Abundance
23.0
22.5
22.0
21.5
21.0
23
22
21
20
Glandular Epithelium
Luminal Epithelium
Stroma
Dominant Cell Type

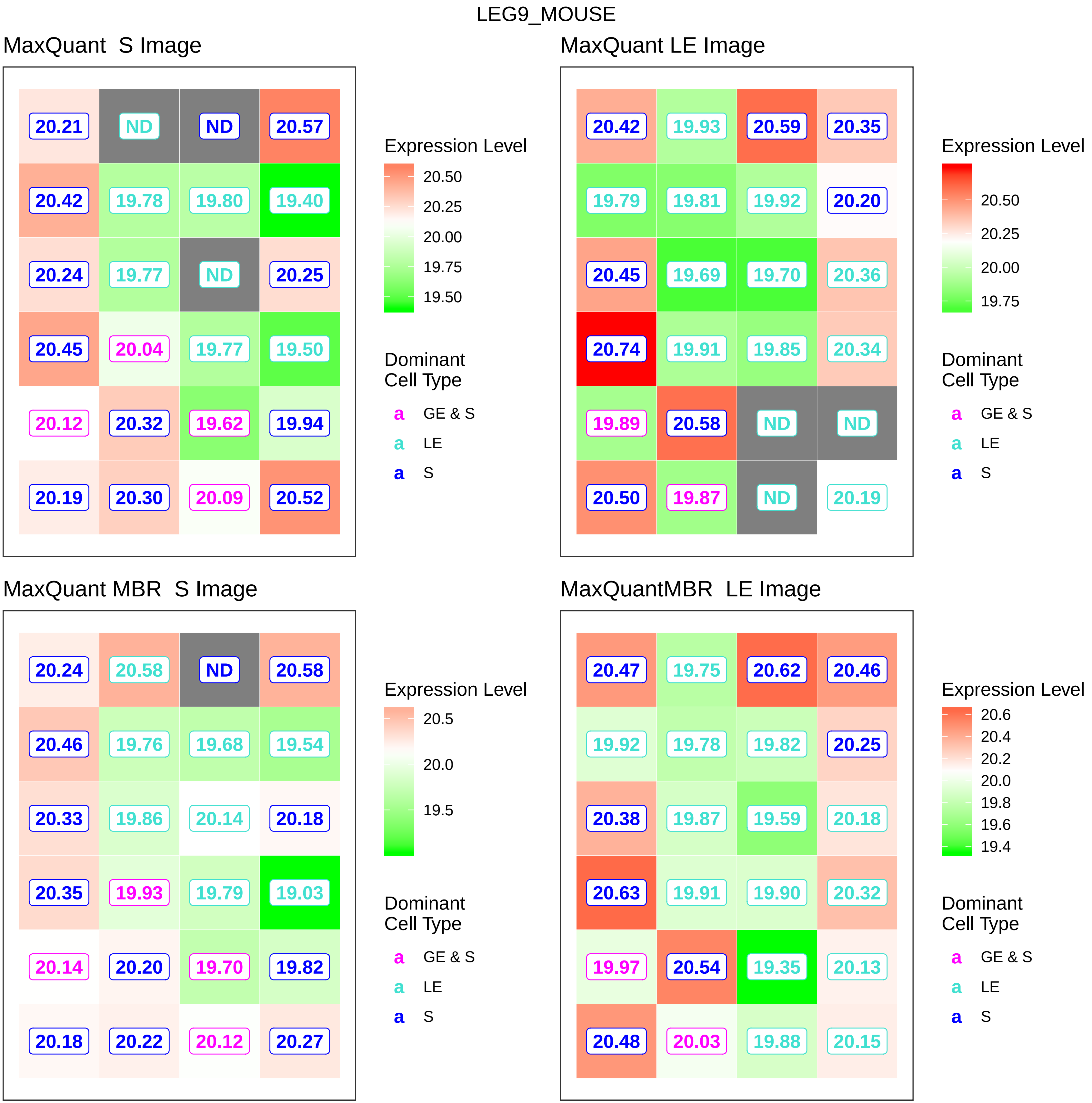
LEG9_MOUSE
MaxQuant S Image
20.21 ND ND 20.57
20.42 19.78 19.80 19.40
20.24 19.77 ND 20.25
20.45 20.04 19.77 19.50
20.12 20.32 19.62 19.94
20.19 20.30 20.09 20.52
Expression Level
20.50
20.25
20.00
19.75
19.50
Dominant Cell Type
a GE & S
a LE
a S
MaxQuant LE Image
20.42 19.93 20.59 20.35
19.79 19.81 19.92 20.20
20.45 19.69 19.70 20.36
20.74 19.91 19.85 20.34
19.89 20.58 ND ND
20.50 19.87 ND 20.19
Expression Level
20.50
20.25
20.00
19.75
Dominant Cell Type
a GE & S
a LE
a S
MaxQuant MBR S Image
20.24 20.58 ND 20.58
20.46 19.76 19.68 19.54
20.33 19.86 20.14 20.18
20.35 19.93 19.79 19.03
20.14 20.20 19.70 19.82
20.18 20.22 20.12 20.27
Expression Level
20.5
20.0
19.5
Dominant Cell Type
a GE & S
a LE
a S
MaxQuantMBR LE Image
20.47 19.75 20.62 20.46
19.92 19.78 19.82 20.25
20.38 19.87 19.59 20.18
20.63 19.91 19.90 20.32
19.97 20.54 19.35 20.13
20.48 20.03 19.88 20.15
Expression Level
20.6
20.4
20.2
20.0
19.8
19.6
19.4
Dominant Cell Type
a GE & S
a LE
a S

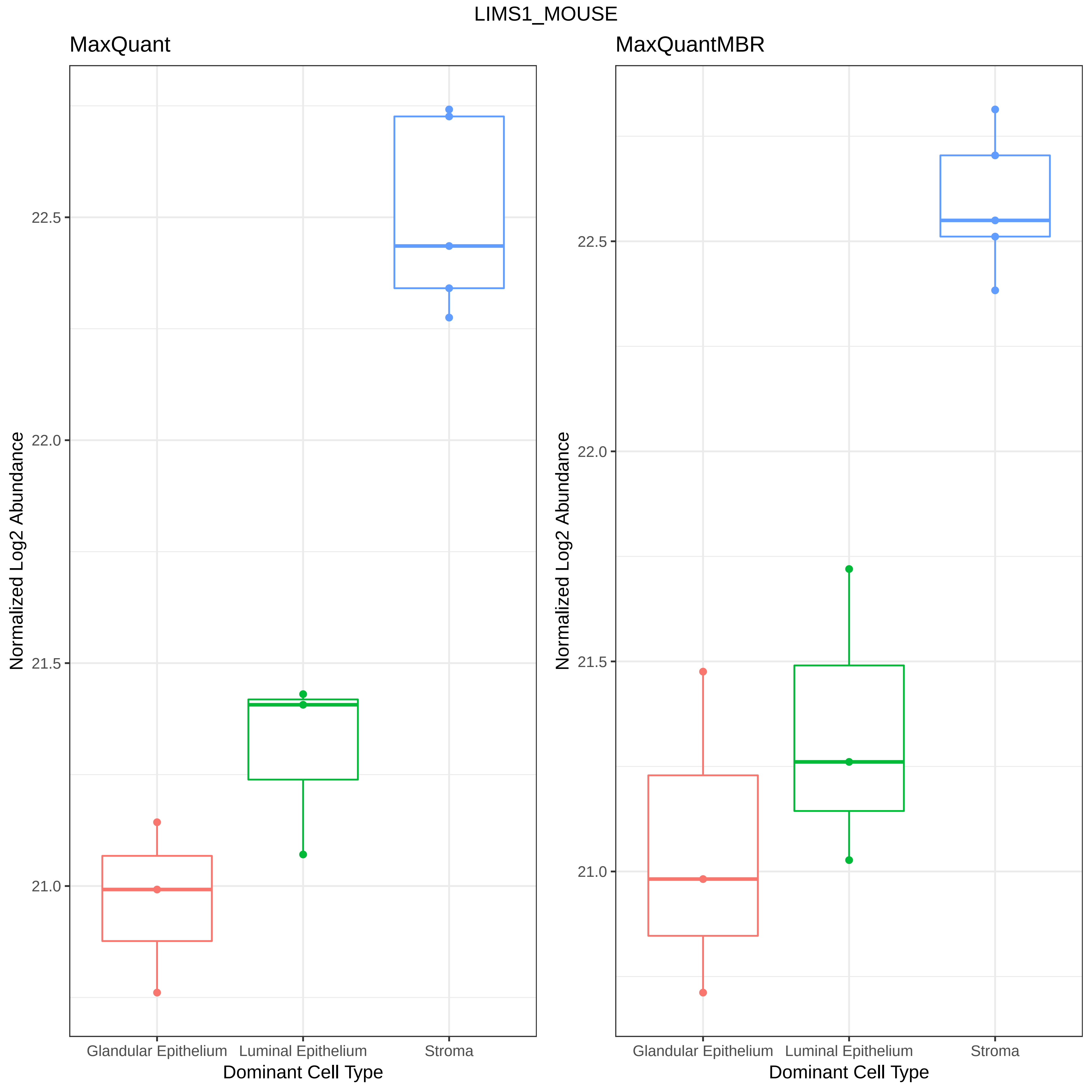
LIMS1_MOUSE
MaxQuant
MaxQuantMBR
Normalized Log2 Abundance
22.5
22.0
21.5
21.0
Glandular Epithelium
Luminal Epithelium
Stroma
Dominant Cell Type

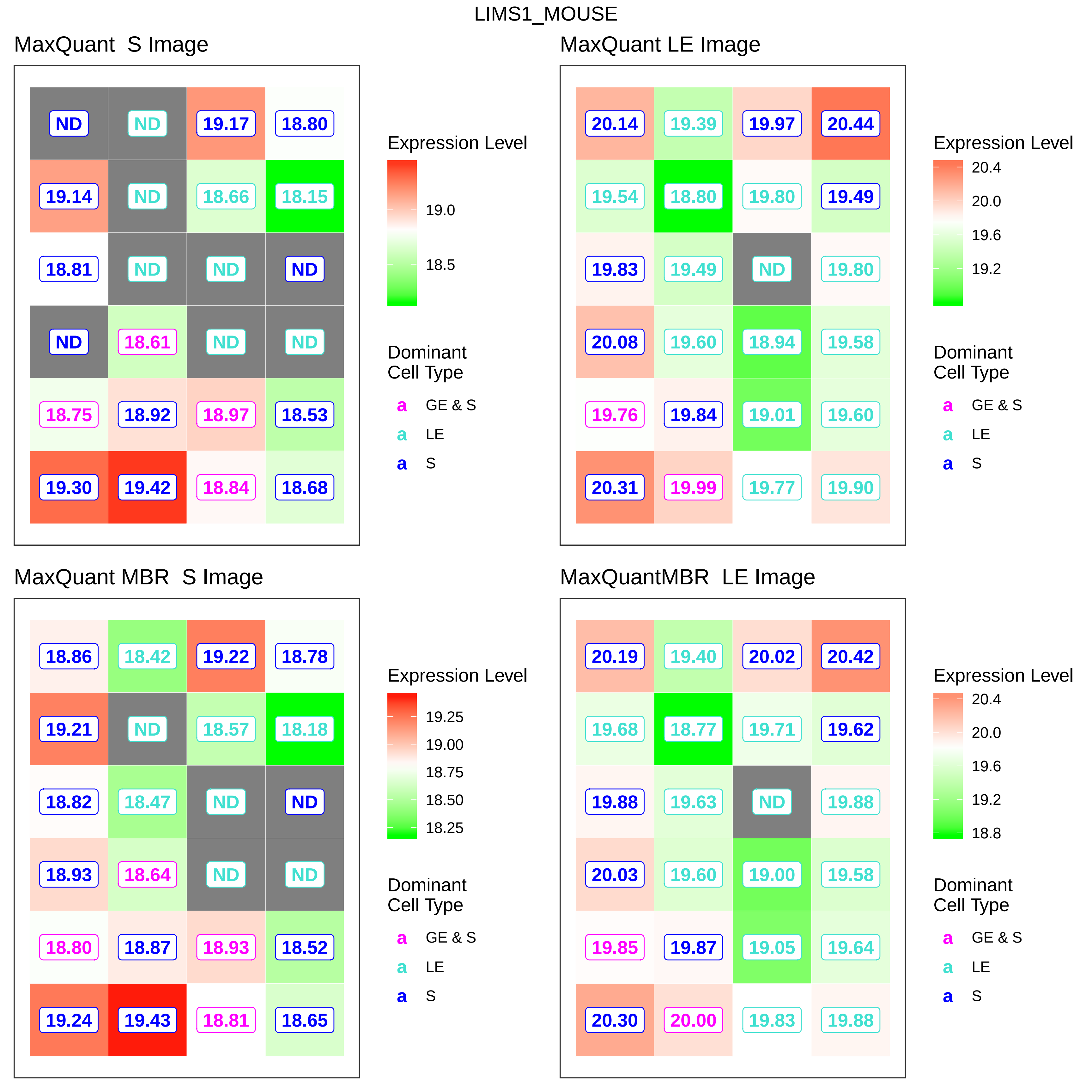

LIMS1_MOUSE
MaxQuant S Image
ND ND 19.17 18.80
19.14 ND 18.66 18.15
18.81 ND ND ND
ND 18.61 ND ND
18.75 18.92 18.97 18.53
19.30 19.42 18.84 18.68
Expression Level
19.0
18.5
Dominant Cell Type
a GE & S
a LE
a S
MaxQuant LE Image
20.14 19.39 19.97 20.44
19.54 18.80 19.80 19.49
19.83 19.49 ND 19.80
20.08 19.60 18.94 19.58
19.76 19.84 19.01 19.60
20.31 19.99 19.77 19.90
Expression Level
20.4
20.0
19.6
19.2
Dominant Cell Type
a GE & S
a LE
a S
MaxQuant MBR S Image
18.86 18.42 19.22 18.78
19.21 ND 18.57 18.18
18.82 18.47 ND ND
18.93 18.64 ND ND
18.80 18.87 18.93 18.52
19.24 19.43 18.81 18.65
Expression Level
19.25
19.00
18.75
18.50
18.25
Dominant Cell Type
a GE & S
a LE
a S
MaxQuantMBR LE Image
20.19 19.40 20.02 20.42
19.68 18.77 19.71 19.62
19.88 19.63 ND 19.88
20.03 19.60 19.00 19.58
19.85 19.87 19.05 19.64
20.30 20.00 19.83 19.88
Expression Level
20.4
20.0
19.6
19.2
18.8
Dominant Cell Type
a GE & S
a LE
a S

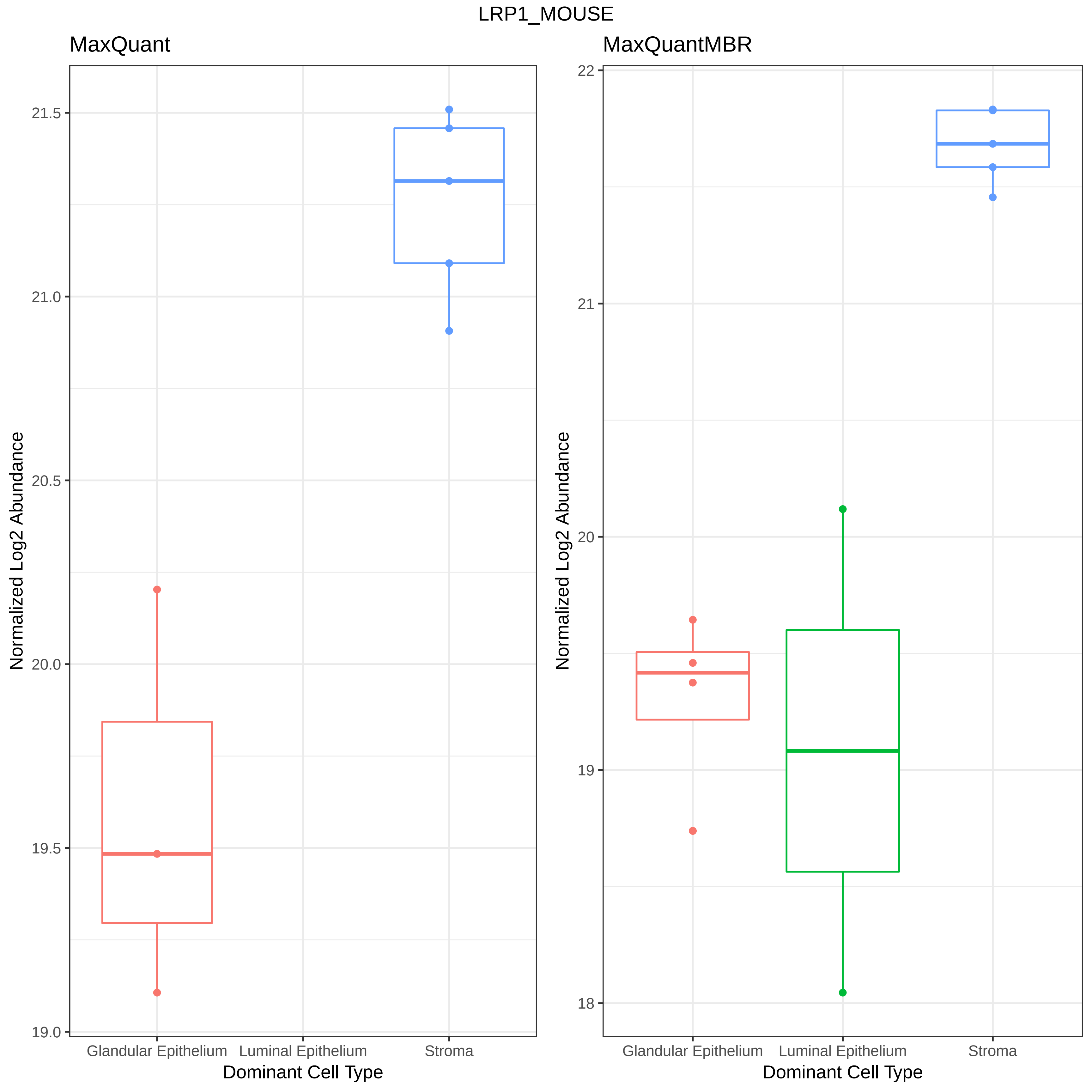
LRP1_MOUSE
MaxQuant
MaxQuantMBR
Normalized Log2 Abundance
Dominant Cell Type
Glandular Epithelium
Luminal Epithelium
Stroma
19.0
19.5
20.0
20.5
21.0
21.5
18
19
20
21
22

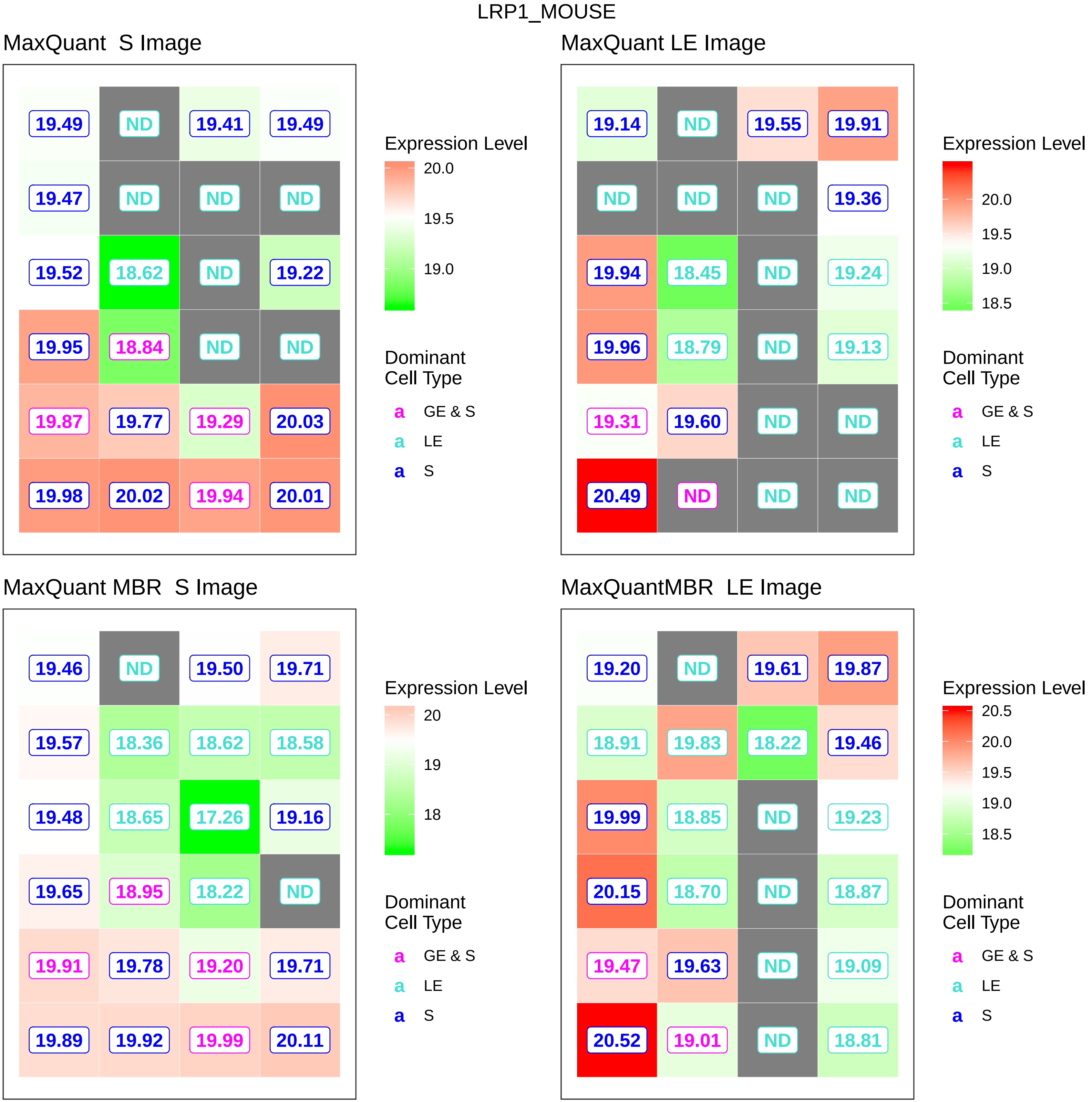
LRP1_MOUSE
MaxQuant S Image
19.49 ND 19.41 19.49
19.47 ND ND ND
19.52 18.62 ND 19.22
19.95 18.84 ND ND
19.87 19.77 19.29 20.03
19.98 20.02 19.94 20.01
Expression Level
20.0
19.5
19.0
Dominant Cell Type
a GE & S
a LE
a S
MaxQuant LE Image
19.14 ND 19.55 19.91
ND ND ND 19.36
19.94 18.45 ND 19.24
19.96 18.79 ND 19.13
19.31 19.60 ND ND
20.49 ND ND ND
Expression Level
20.0
19.5
19.0
18.5
Dominant Cell Type
a GE & S
a LE
a S
MaxQuant MBR S Image
19.46 ND 19.50 19.71
19.57 18.36 18.62 18.58
19.48 18.65 17.26 19.16
19.65 18.95 18.22 ND
19.91 19.78 19.20 19.71
19.89 19.92 19.99 20.11
Expression Level
20
19
18
Dominant Cell Type
a GE & S
a LE
a S
MaxQuantMBR LE Image
19.20 ND 19.61 19.87
18.91 19.83 18.22 19.46
19.99 18.85 ND 19.23
20.15 18.70 ND 18.87
19.47 19.63 ND 19.09
20.52 19.01 ND 18.81
Expression Level
20.5
20.0
19.5
19.0
18.5
Dominant Cell Type
a GE & S
a LE
a S

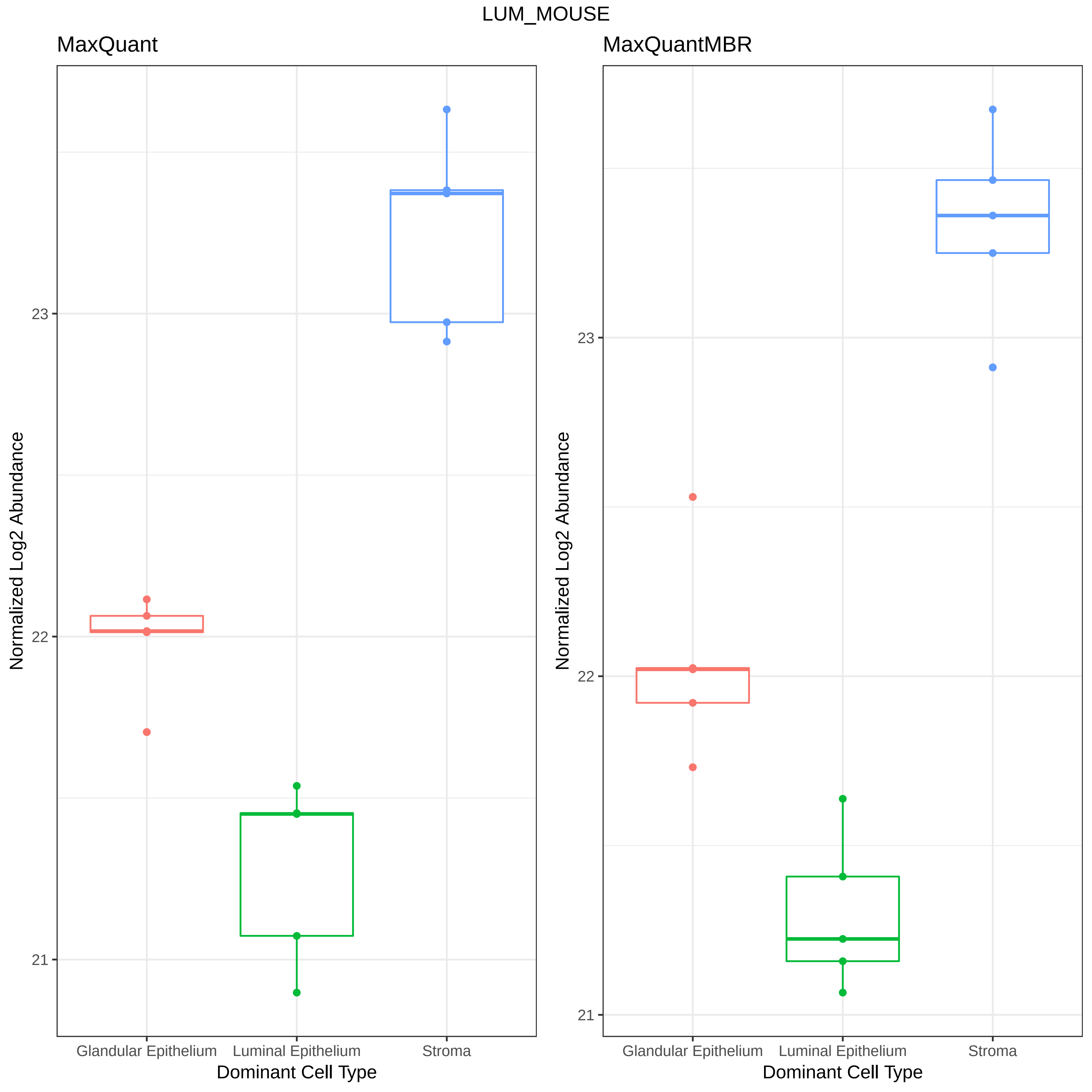
LUM_MOUSE
MaxQuant
MaxQuantMBR
Normalized Log2 Abundance
23
22
21
Glandular Epithelium
Luminal Epithelium
Stroma
Dominant Cell Type

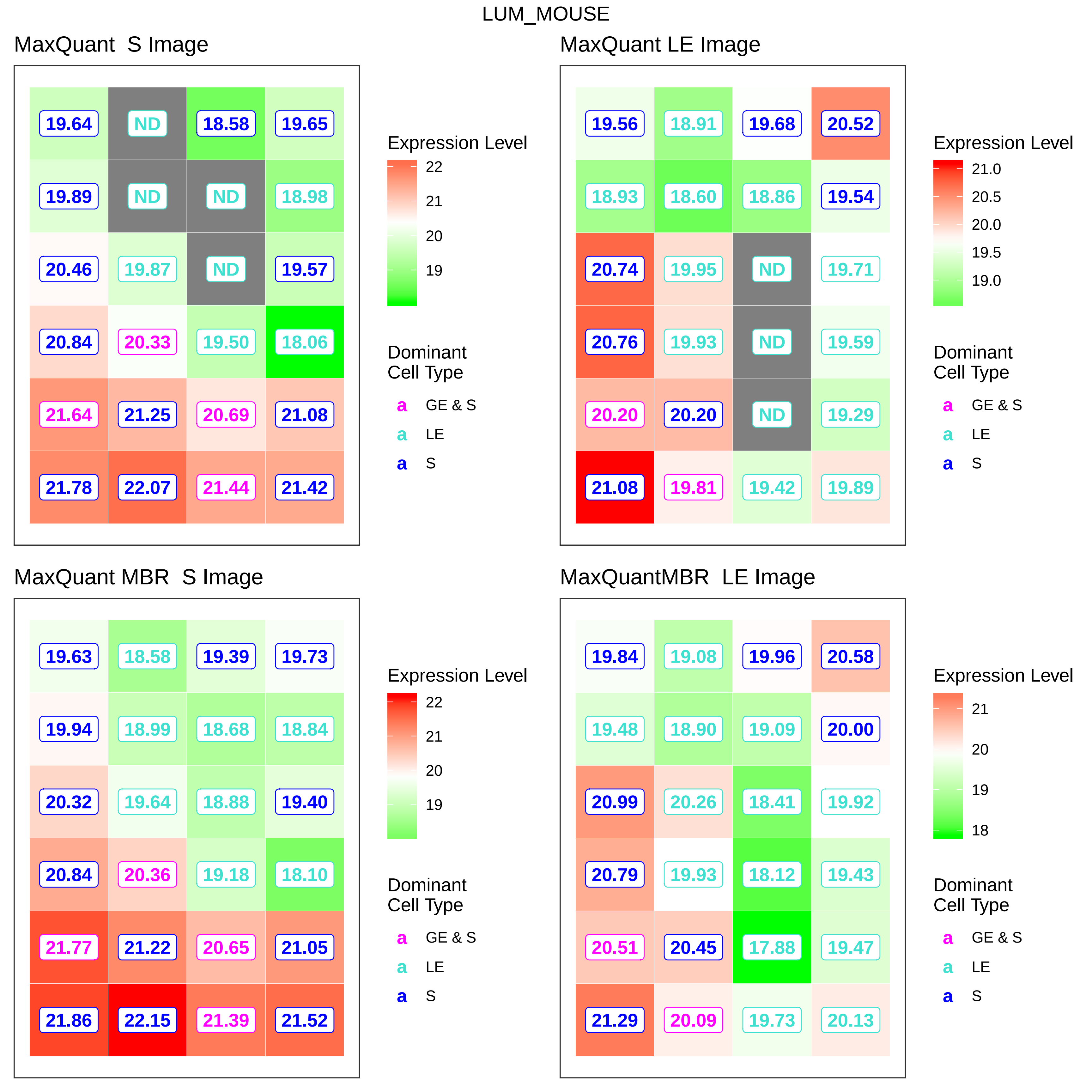
LUM_MOUSE
MaxQuant S Image
19.64 ND 18.58 19.65
19.89 ND ND 18.98
20.46 19.87 ND 19.57
20.84 20.33 19.50 18.06
21.64 21.25 20.69 21.08
21.78 22.07 21.44 21.42
Expression Level
22
21
20
19
Dominant Cell Type
a GE & S
a LE
a S
MaxQuant LE Image
19.56 18.91 19.68 20.52
18.93 18.60 18.86 19.54
20.74 19.95 ND 19.71
20.76 19.93 ND 19.59
20.20 20.20 ND 19.29
21.08 19.81 19.42 19.89
Expression Level
21.0
20.5
20.0
19.5
19.0
Dominant Cell Type
a GE & S
a LE
a S
MaxQuant MBR S Image
19.63 18.58 19.39 19.73
19.94 18.99 18.68 18.84
20.32 19.64 18.88 19.40
20.84 20.36 19.18 18.10
21.77 21.22 20.65 21.05
21.86 22.15 21.39 21.52
Expression Level
22
21
20
19
Dominant Cell Type
a GE & S
a LE
a S
MaxQuantMBR LE Image
19.84 19.08 19.96 20.58
19.48 18.90 19.09 20.00
20.99 20.26 18.41 19.92
20.79 19.93 18.12 19.43
20.51 20.45 17.88 19.47
21.29 20.09 19.73 20.13
Expression Level
21
20
19
18
Dominant Cell Type
a GE & S
a LE
a S

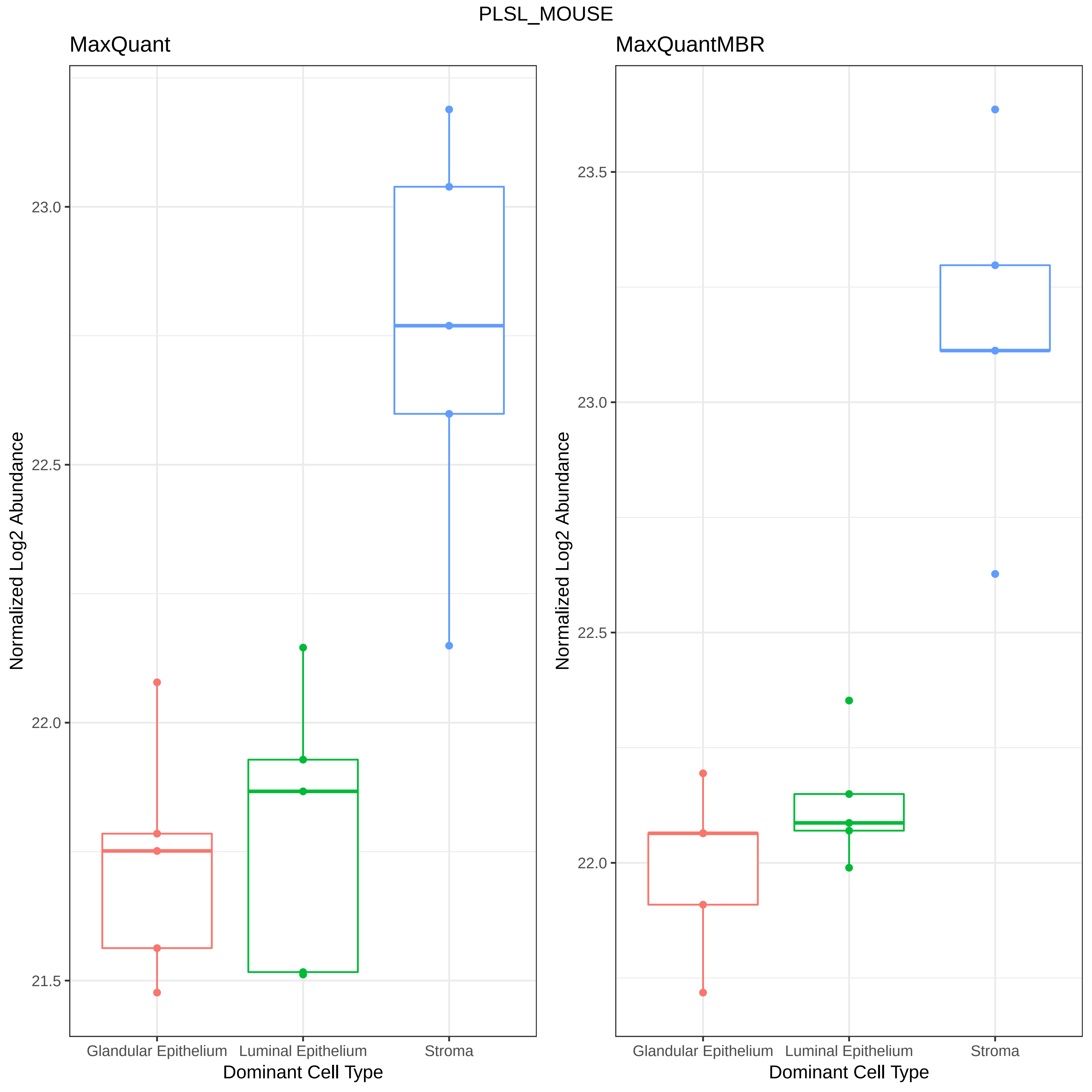
PLSL_MOUSE
MaxQuant
MaxQuantMBR
Normalized Log2 Abundance
Dominant Cell Type
Glandular Epithelium
Luminal Epithelium
Stroma
21.5
22.0
22.5
23.0
23.5

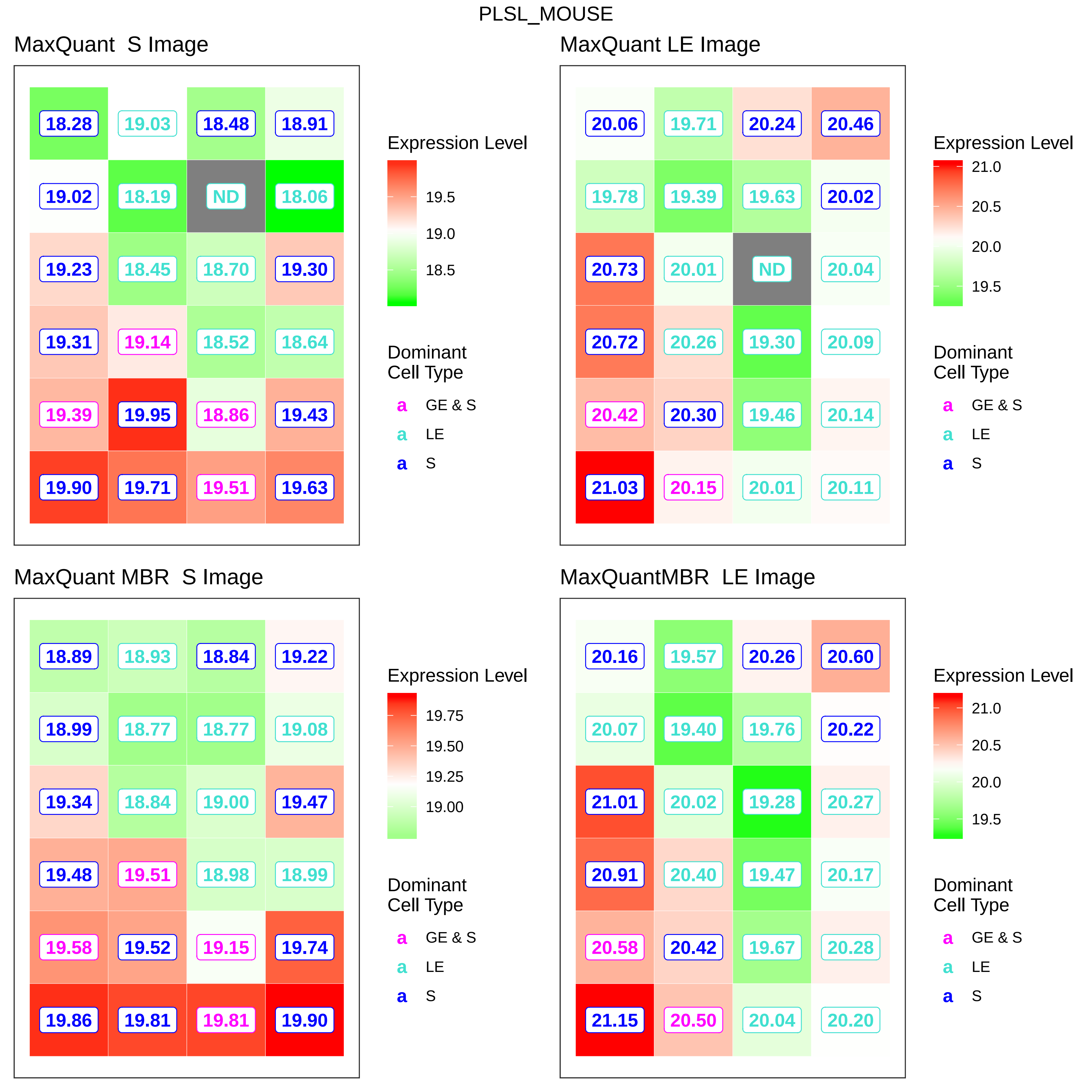

PLSL_MOUSE
MaxQuant S Image
18.28 19.03 18.48 18.91
19.02 18.19 ND 18.06
19.23 18.45 18.70 19.30
19.31 19.14 18.52 18.64
19.39 19.95 18.86 19.43
19.90 19.71 19.51 19.63
Expression Level
19.5
19.0
18.5
Dominant Cell Type
a GE & S
a LE
a S
MaxQuant LE Image
20.06 19.71 20.24 20.46
19.78 19.39 19.63 20.02
20.73 20.01 ND 20.04
20.72 20.26 19.30 20.09
20.42 20.30 19.46 20.14
21.03 20.15 20.01 20.11
Expression Level
21.0
20.5
20.0
19.5
Dominant Cell Type
a GE & S
a LE
a S
MaxQuant MBR S Image
18.89 18.93 18.84 19.22
18.99 18.77 18.77 19.08
19.34 18.84 19.00 19.47
19.48 19.51 18.98 18.99
19.58 19.52 19.15 19.74
19.86 19.81 19.81 19.90
Expression Level
19.75
19.50
19.25
19.00
Dominant Cell Type
a GE & S
a LE
a S
MaxQuantMBR LE Image
20.16 19.57 20.26 20.60
20.07 19.40 19.76 20.22
21.01 20.02 19.28 20.27
20.91 20.40 19.47 20.17
20.58 20.42 19.67 20.28
21.15 20.50 20.04 20.20
Expression Level
21.0
20.5
20.0
19.5
Dominant Cell Type
a GE & S
a LE
a S

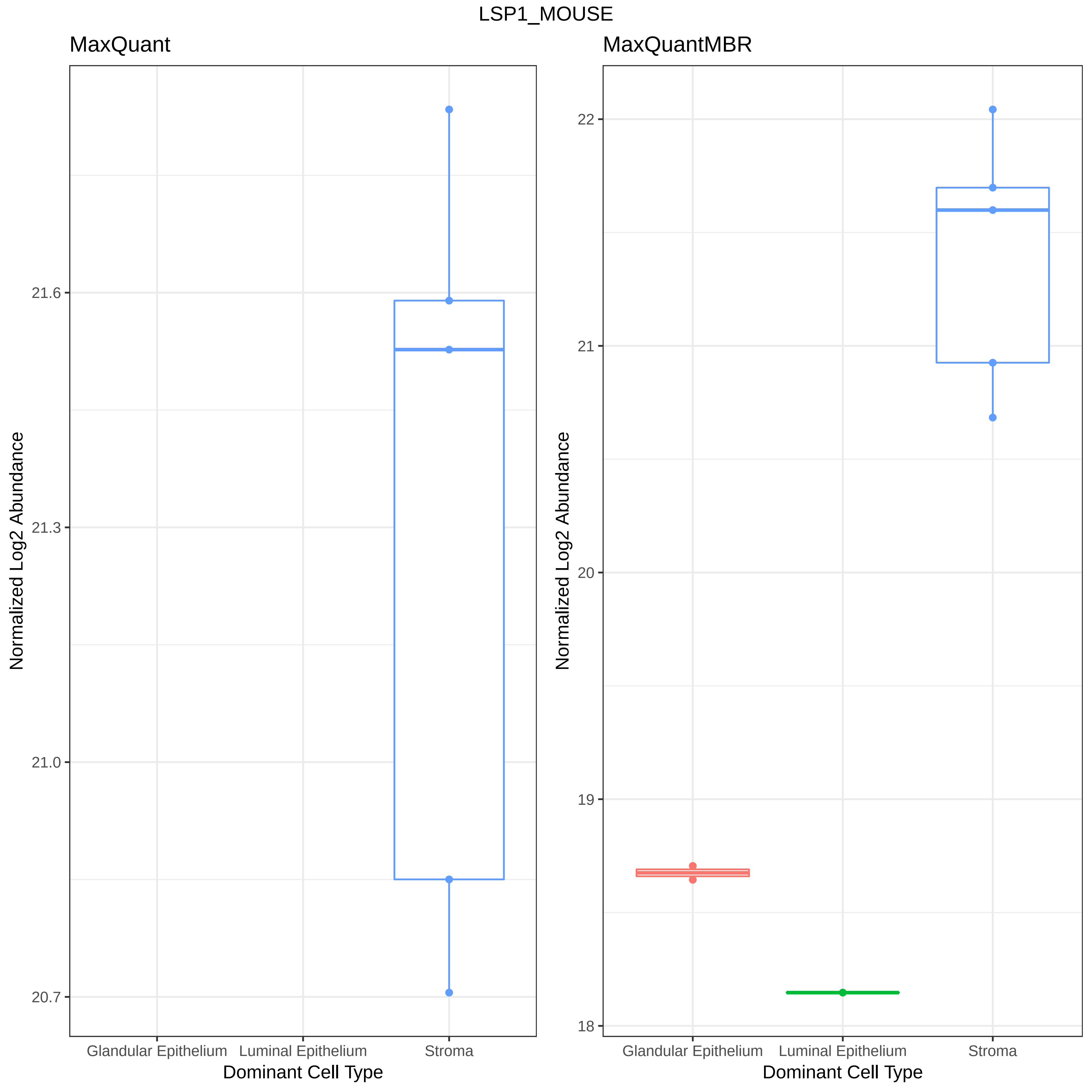
LSP1_MOUSE
MaxQuant
MaxQuantMBR
Normalized Log2 Abundance
21.6
21.3
21.0
20.7
22
21
20
19
18
Glandular Epithelium
Luminal Epithelium
Stroma
Dominant Cell Type

LSP1_MOUSE

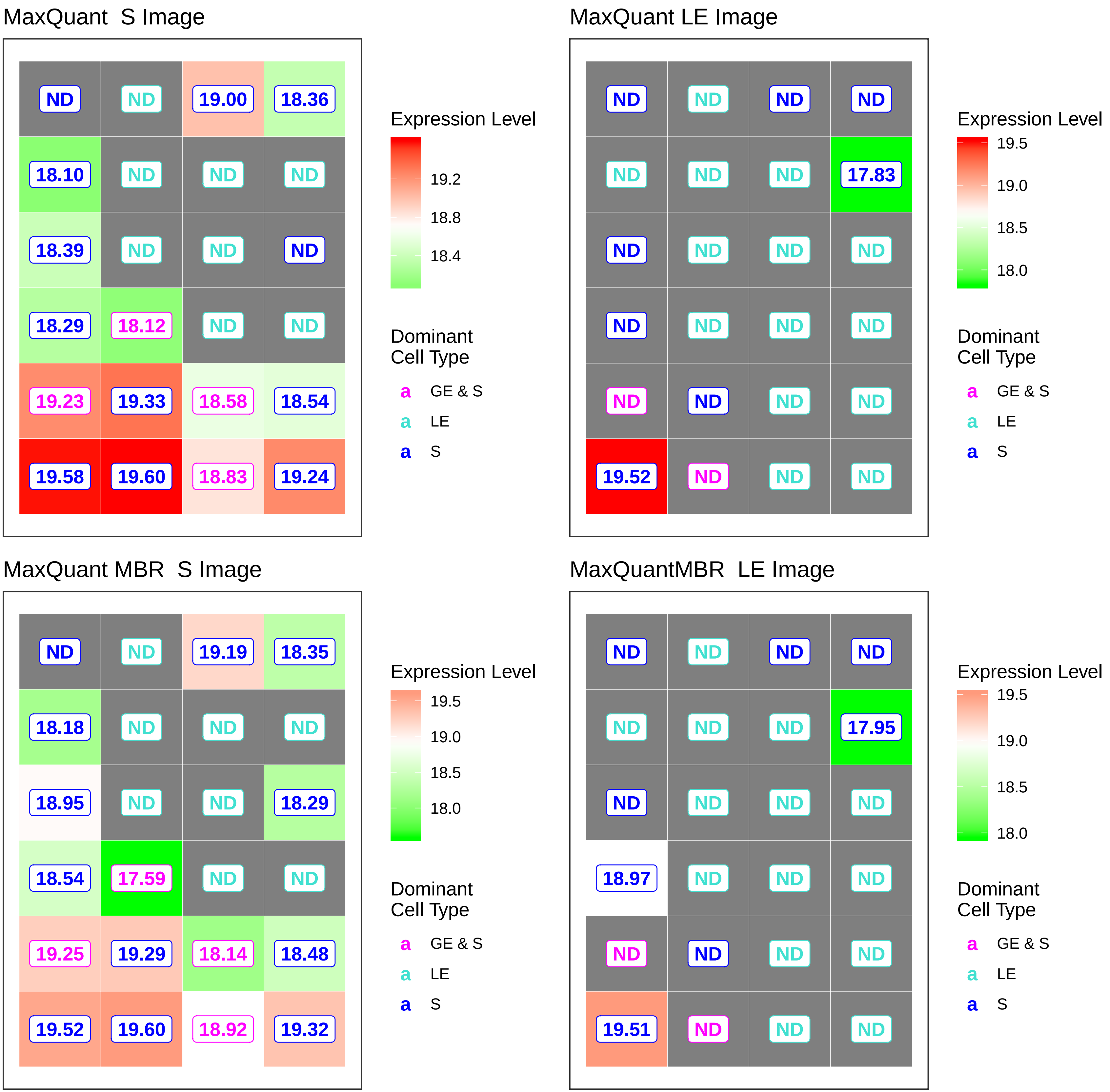

MaxQuant S Image
ND ND 19.00 18.36
18.10 ND ND ND
18.39 ND ND ND
18.29 18.12 ND ND
19.23 19.33 18.58 18.54
19.58 19.60 18.83 19.24
Expression Level
19.2
18.8
18.4
Dominant Cell Type
a GE & S
a LE
a S
MaxQuant LE Image
ND ND ND ND
ND ND ND 17.83
ND ND ND ND
ND ND ND ND
ND ND ND ND
19.52 ND ND ND
Expression Level
19.5
19.0
18.5
18.0
Dominant Cell Type
a GE & S
a LE
a S
MaxQuant MBR S Image
ND ND 19.19 18.35
18.18 ND ND ND
18.95 ND ND 18.29
18.54 17.59 ND ND
19.25 19.29 18.14 18.48
19.52 19.60 18.92 19.32
Expression Level
19.5
19.0
18.5
18.0
Dominant Cell Type
a GE & S
a LE
a S
MaxQuantMBR LE Image
ND ND ND ND
ND ND ND 17.95
ND ND ND ND
18.97 ND ND ND
ND ND ND ND
19.51 ND ND ND
Expression Level
19.5
19.0
18.5
18.0
Dominant Cell Type
a GE & S
a LE
a S

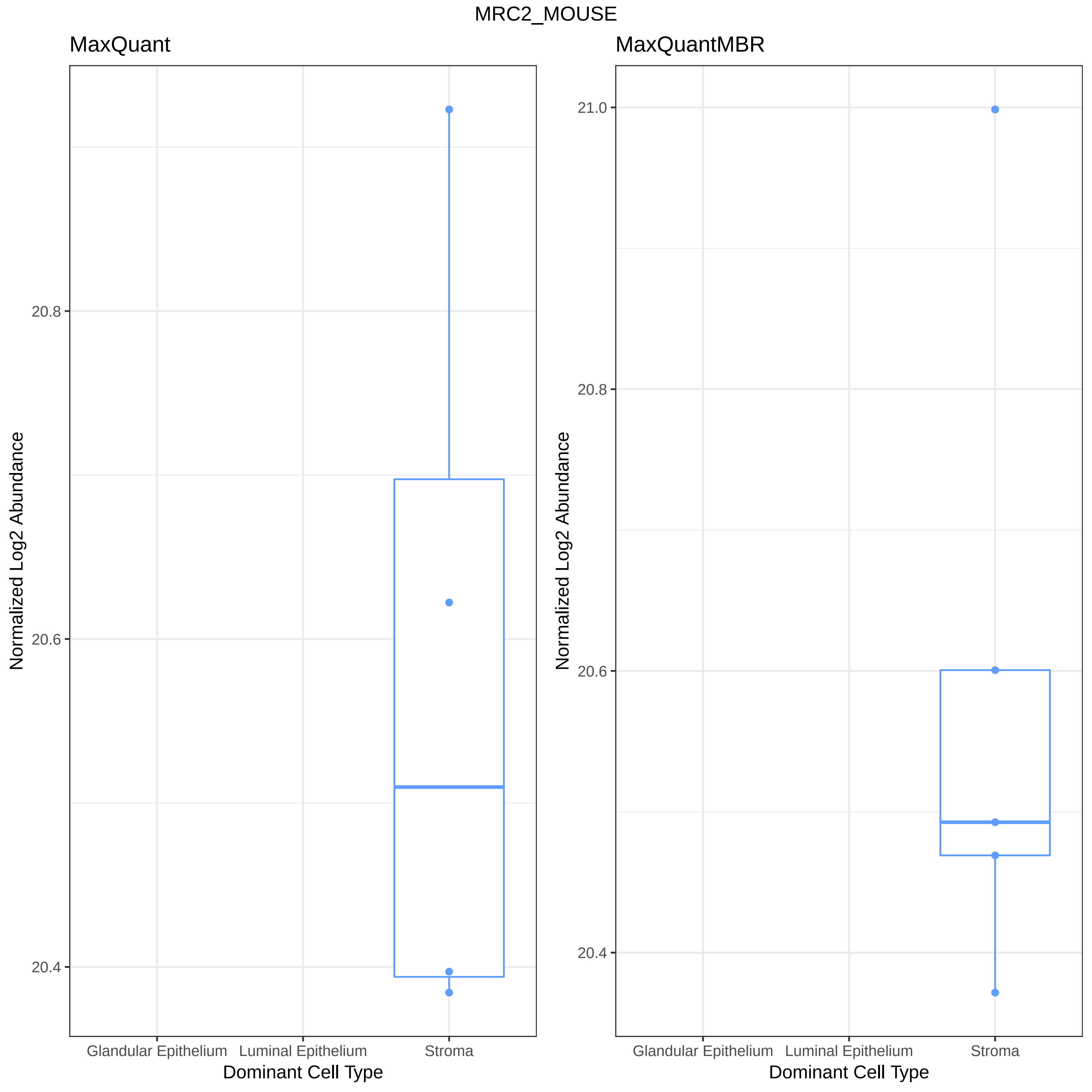
MRC2_MOUSE
MaxQuant
MaxQuantMBR
Normalized Log2 Abundance
20.4
20.6
20.8
21.0
Glandular Epithelium
Luminal Epithelium
Stroma
Dominant Cell Type

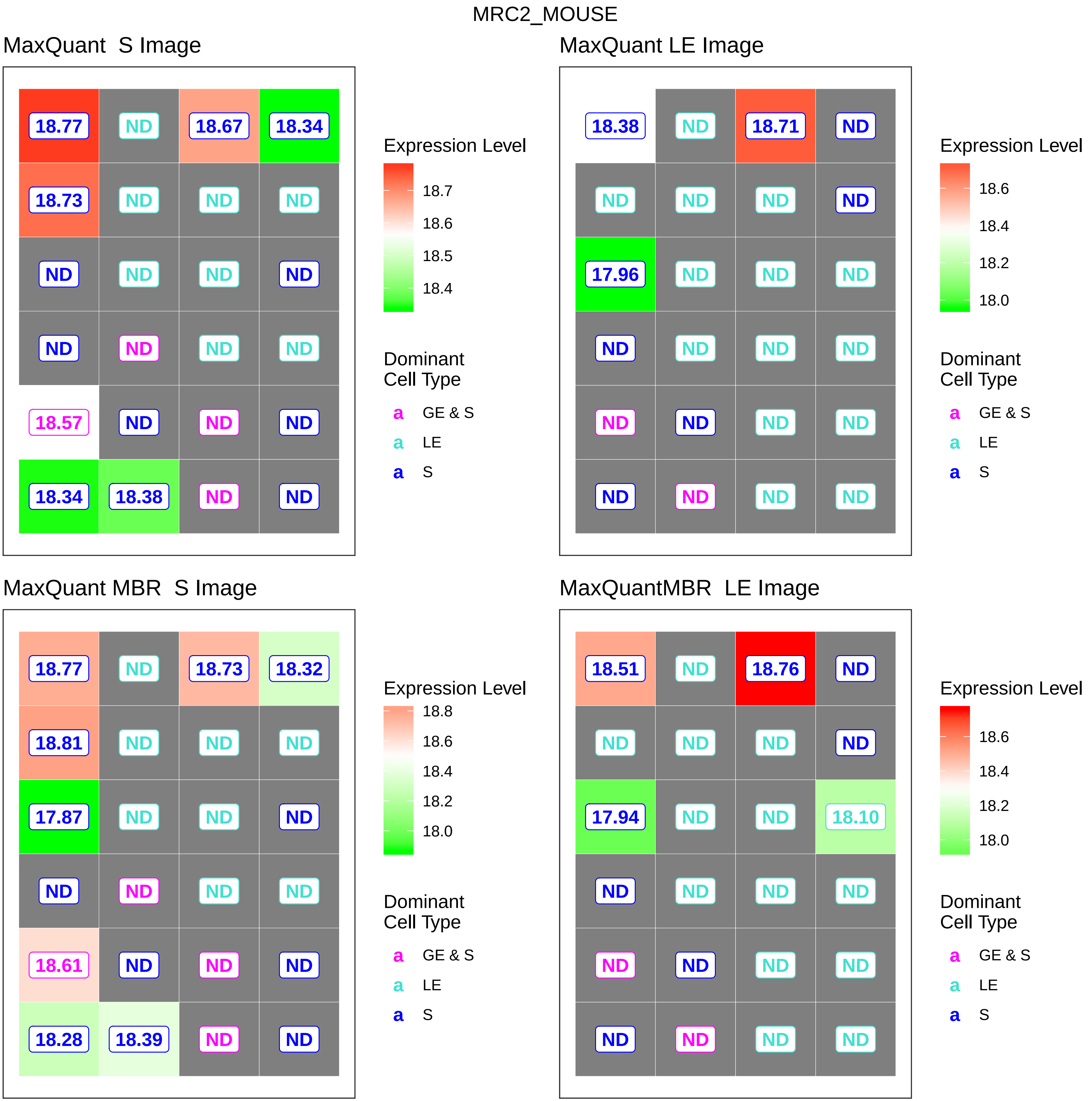
MRC2_MOUSE
MaxQuant S Image
18.77 ND 18.67 18.34
18.73 ND ND ND
ND ND ND ND
ND ND ND ND
18.57 ND ND ND
18.34 18.38 ND ND
Expression Level
18.7
18.6
18.5
18.4
Dominant Cell Type
a GE & S
a LE
a S
MaxQuant LE Image
18.38 ND 18.71 ND
ND ND ND ND
17.96 ND ND ND
ND ND ND ND
ND ND ND ND
ND ND ND ND
Expression Level
18.6
18.4
18.2
18.0
Dominant Cell Type
a GE & S
a LE
a S
MaxQuant MBR S Image
18.77 ND 18.73 18.32
18.81 ND ND ND
17.87 ND ND ND
ND ND ND ND
18.61 ND ND ND
18.28 18.39 ND ND
Expression Level
18.8
18.6
18.4
18.2
18.0
Dominant Cell Type
a GE & S
a LE
a S
MaxQuantMBR LE Image
18.51 ND 18.76 ND
ND ND ND ND
17.94 ND ND 18.10
ND ND ND ND
ND ND ND ND
ND ND ND ND
Expression Level
18.6
18.4
18.2
18.0
Dominant Cell Type
a GE & S
a LE
a S

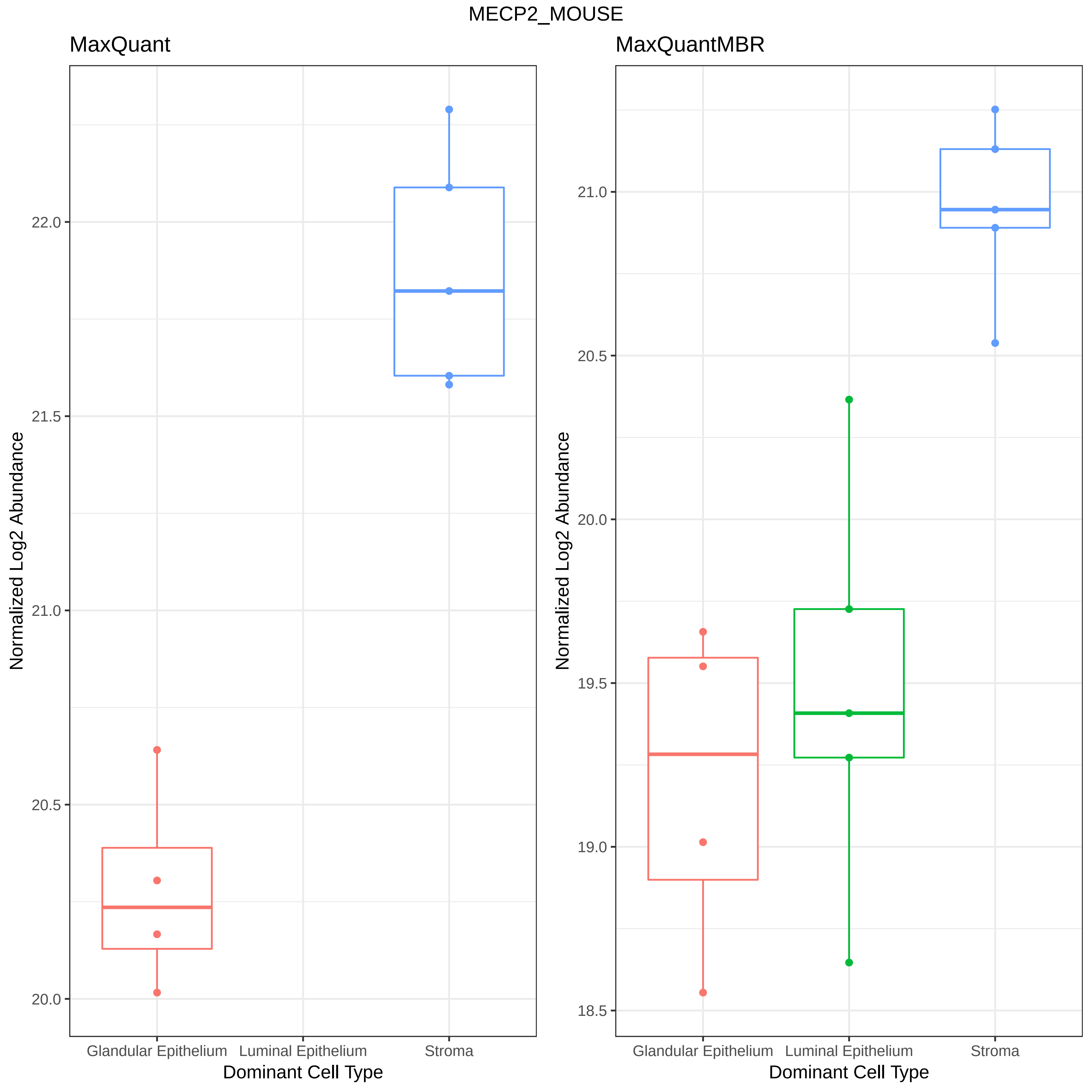
MECP2_MOUSE
MaxQuant
MaxQuantMBR
Normalized Log2 Abundance
22.0
21.5
21.0
20.5
20.0
21.0
20.5
20.0
19.5
19.0
18.5
Glandular Epithelium
Luminal Epithelium
Stroma
Dominant Cell Type

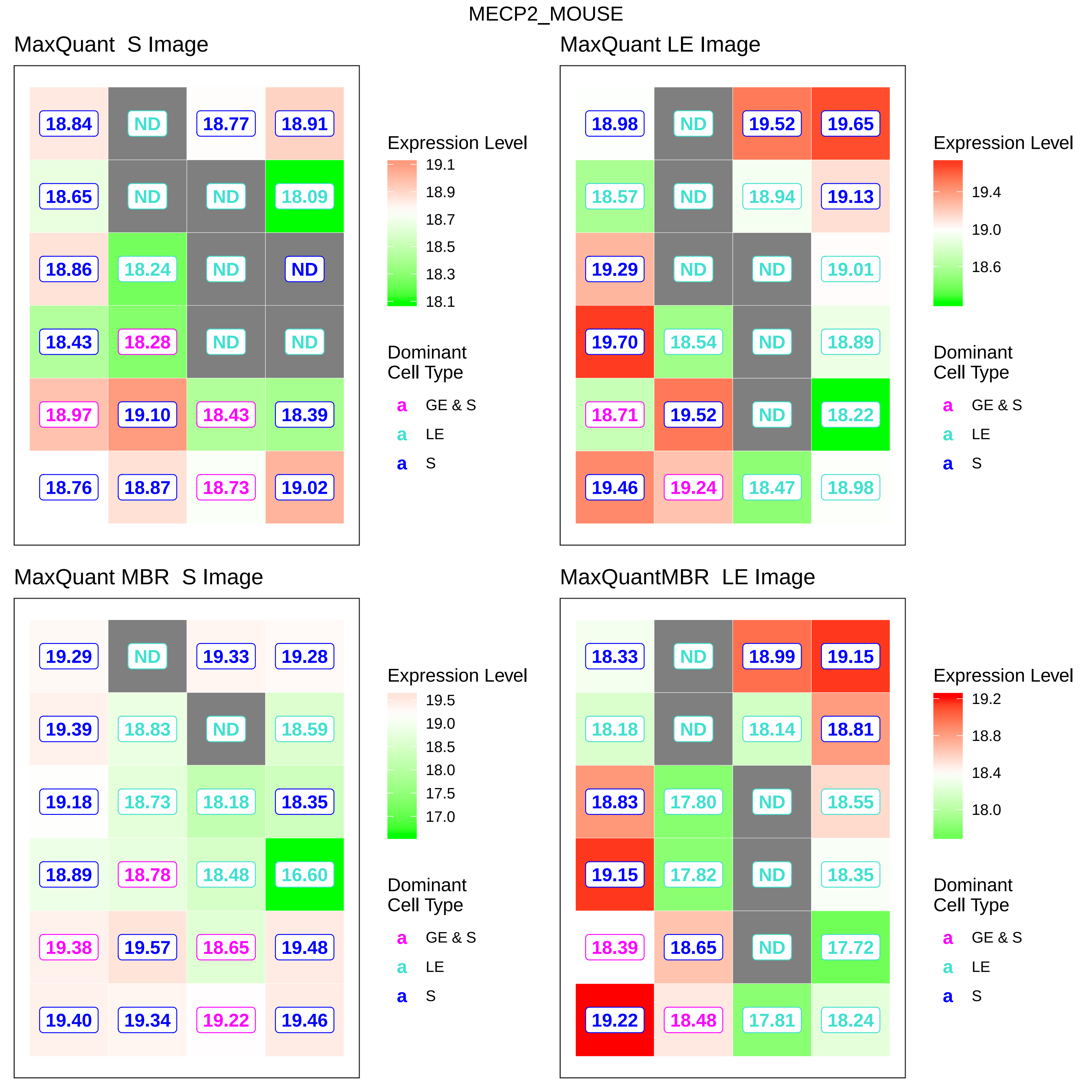
MECP2_MOUSE
MaxQuant S Image
18.84 ND 18.77 18.91
18.65 ND ND 18.09
18.86 18.24 ND ND
18.43 18.28 ND ND
18.97 19.10 18.43 18.39
18.76 18.87 18.73 19.02
Expression Level
19.1 18.9 18.7 18.5 18.3 18.1
Dominant Cell Type
a GE & S
a LE
a S
MaxQuant LE Image
18.98 ND 19.52 19.65
18.57 ND 18.94 19.13
19.29 ND ND 19.01
19.70 18.54 ND 18.89
18.71 19.52 ND 18.22
19.46 19.24 18.47 18.98
Expression Level
19.4 19.0 18.6
Dominant Cell Type
a GE & S
a LE
a S
MaxQuant MBR S Image
19.29 ND 19.33 19.28
19.39 18.83 ND 18.59
19.18 18.73 18.18 18.35
18.89 18.78 18.48 16.60
19.38 19.57 18.65 19.48
19.40 19.34 19.22 19.46
Expression Level
19.5 19.0 18.5 18.0 17.5 17.0
Dominant Cell Type
a GE & S
a LE
a S
MaxQuantMBR LE Image
18.33 ND 18.99 19.15
18.18 ND 18.14 18.81
18.83 17.80 ND 18.55
19.15 17.82 ND 18.35
18.39 18.65 ND 17.72
19.22 18.48 17.81 18.24
Expression Level
19.2 18.8 18.4 18.0
Dominant Cell Type
a GE & S
a LE
a S

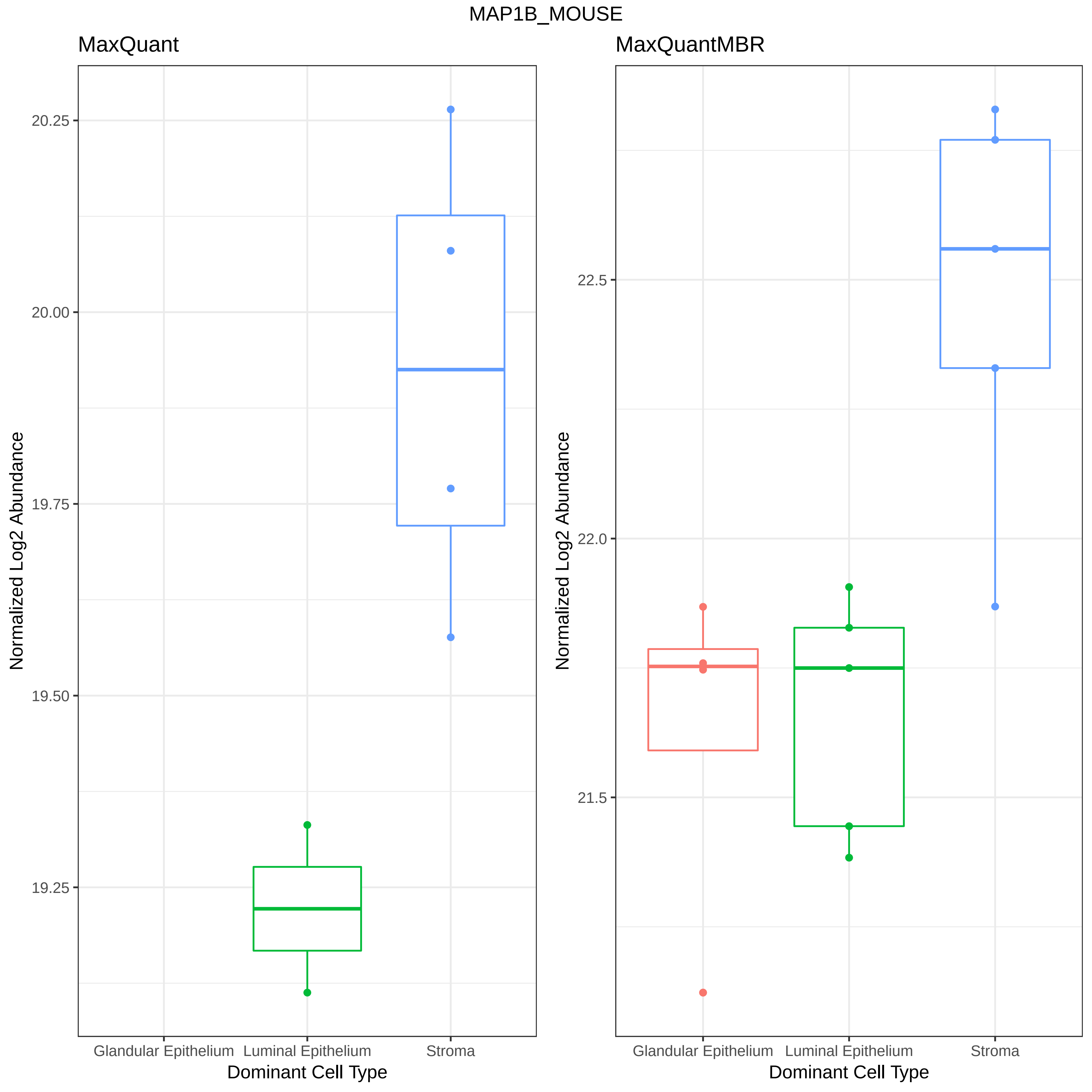
MAP1B_MOUSE
MaxQuant
MaxQuantMBR
Normalized Log2 Abundance
20.25
20.00
19.75
19.50
19.25
22.5
22.0
21.5
Glandular Epithelium
Luminal Epithelium
Stroma
Dominant Cell Type

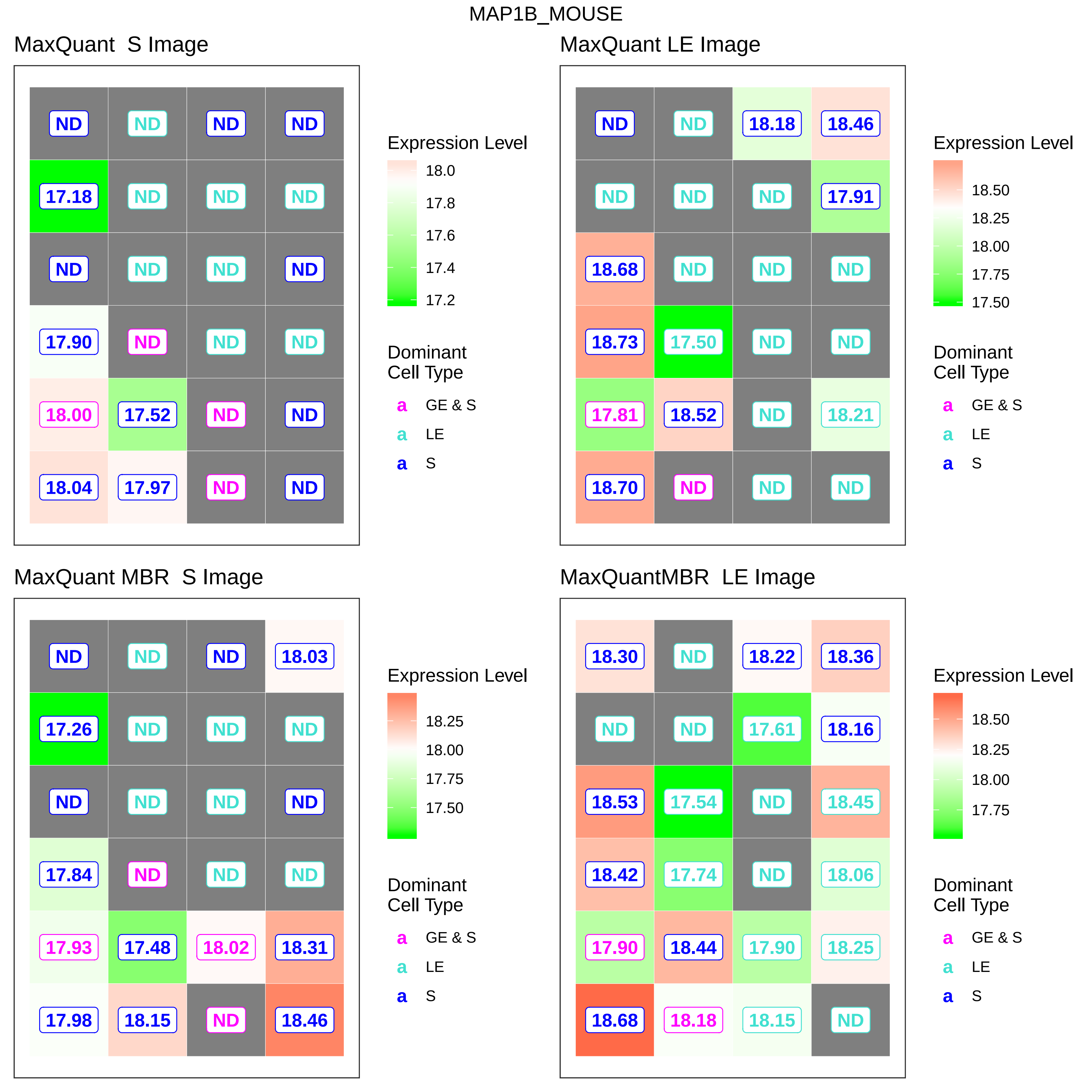

MAP1B_MOUSE
MaxQuant S Image
ND ND ND ND
17.18 ND ND ND
ND ND ND ND
17.90 ND ND ND
18.00 17.52 ND ND
18.04 17.97 ND ND
Expression Level
18.0
17.8
17.6
17.4
17.2
Dominant Cell Type
a GE & S
a LE
a S
MaxQuant LE Image
ND ND 18.18 18.46
ND ND ND 17.91
18.68 ND ND ND
18.73 17.50 ND ND
17.81 18.52 ND 18.21
18.70 ND ND ND
Expression Level
18.50
18.25
18.00
17.75
17.50
Dominant Cell Type
a GE & S
a LE
a S
MaxQuant MBR S Image
ND ND ND 18.03
17.26 ND ND ND
ND ND ND ND
17.84 ND ND ND
17.93 17.48 18.02 18.31
17.98 18.15 ND 18.46
Expression Level
18.25
18.00
17.75
17.50
Dominant Cell Type
a GE & S
a LE
a S
MaxQuantMBR LE Image
18.30 ND 18.22 18.36
ND ND 17.61 18.16
18.53 17.54 ND 18.45
18.42 17.74 ND 18.06
17.90 18.44 17.90 18.25
18.68 18.18 18.15 ND
Expression Level
18.50
18.25
18.00
17.75
Dominant Cell Type
a GE & S
a LE
a S

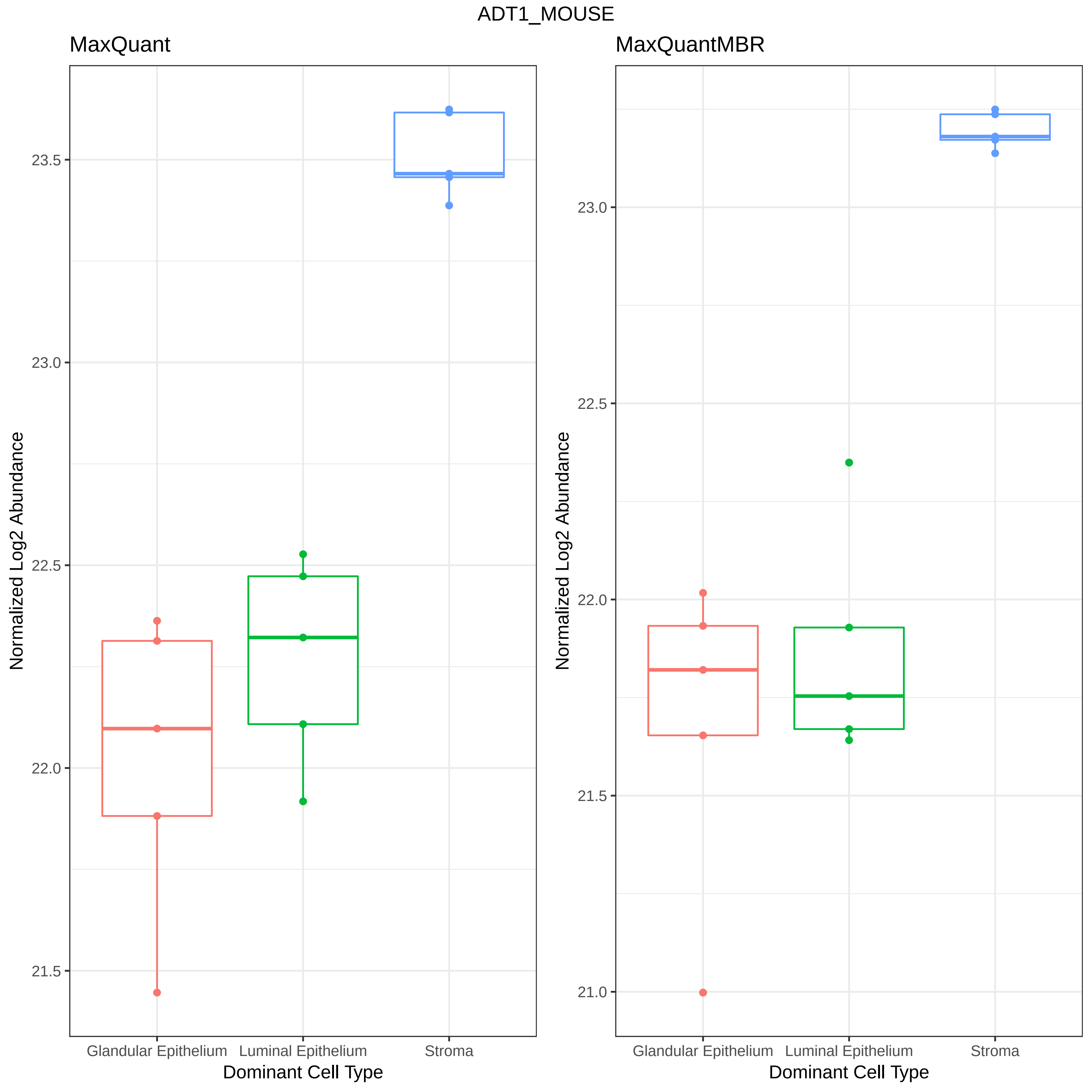

ADT1_MOUSE
MaxQuant
MaxQuantMBR
Normalized Log2 Abundance
23.5
23.0
22.5
22.0
21.5
21.0
Glandular Epithelium
Luminal Epithelium
Stroma
Dominant Cell Type

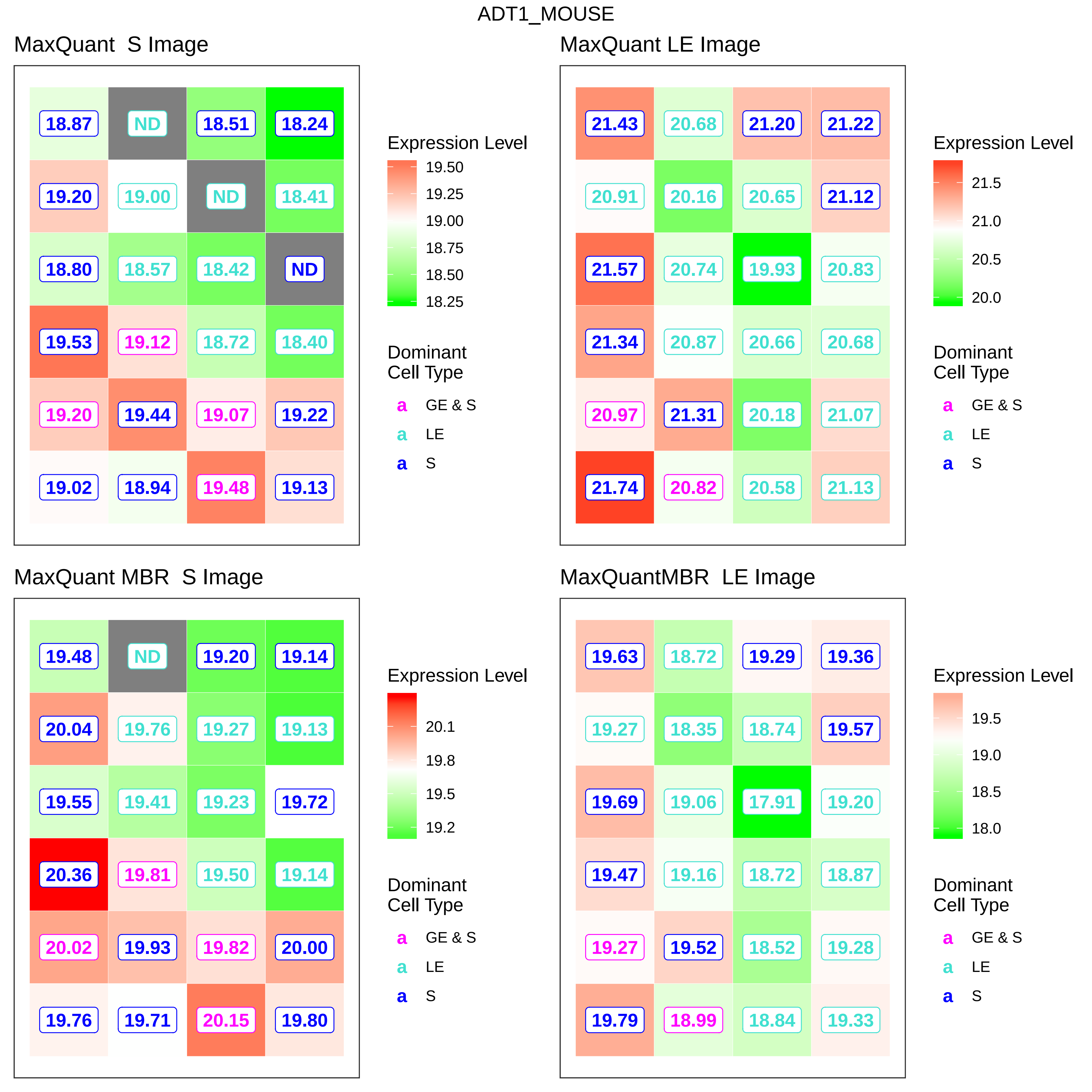
ADT1_MOUSE
MaxQuant S Image
18.87 ND 18.51 18.24
19.20 19.00 ND 18.41
18.80 18.57 18.42 ND
19.53 19.12 18.72 18.40
19.20 19.44 19.07 19.22
19.02 18.94 19.48 19.13
Expression Level
19.50
19.25
19.00
18.75
18.50
18.25
Dominant Cell Type
a GE & S
a LE
a S
MaxQuant LE Image
21.43 20.68 21.20 21.22
20.91 20.16 20.65 21.12
21.57 20.74 19.93 20.83
21.34 20.87 20.66 20.68
20.97 21.31 20.18 21.07
21.74 20.82 20.58 21.13
Expression Level
21.5
21.0
20.5
20.0
Dominant Cell Type
a GE & S
a LE
a S
MaxQuant MBR S Image
19.48 ND 19.20 19.14
20.04 19.76 19.27 19.13
19.55 19.41 19.23 19.72
20.36 19.81 19.50 19.14
20.02 19.93 19.82 20.00
19.76 19.71 20.15 19.80
Expression Level
20.1
19.8
19.5
19.2
Dominant Cell Type
a GE & S
a LE
a S
MaxQuantMBR LE Image
19.63 18.72 19.29 19.36
19.27 18.35 18.74 19.57
19.69 19.06 17.91 19.20
19.47 19.16 18.72 18.87
19.27 19.52 18.52 19.28
19.79 18.99 18.84 19.33
Expression Level
19.5
19.0
18.5
18.0
Dominant Cell Type
a GE & S
a LE
a S

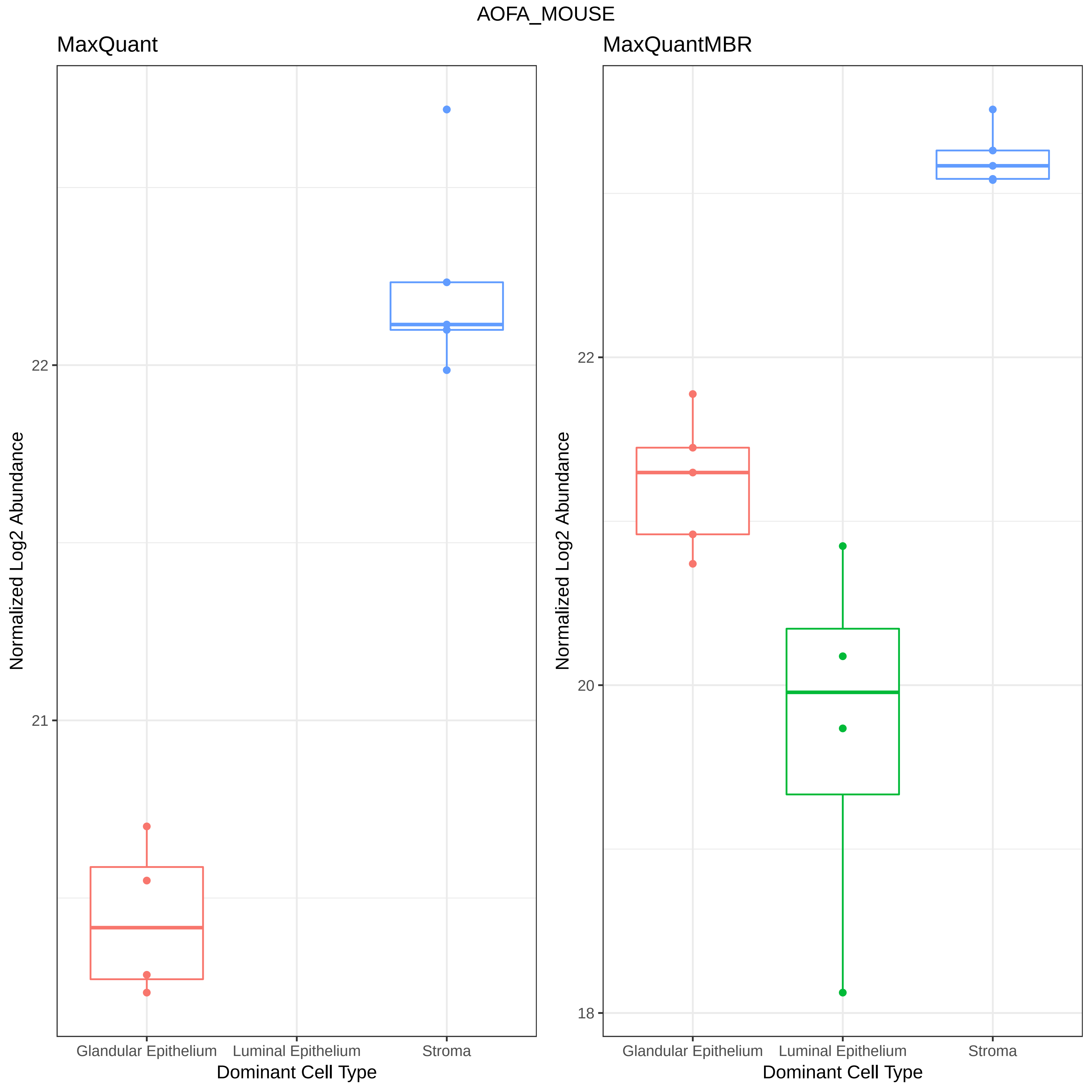
AOFA_MOUSE
MaxQuant
MaxQuantMBR
Normalized Log2 Abundance
22
21
20
18
Glandular Epithelium
Luminal Epithelium
Stroma
Dominant Cell Type

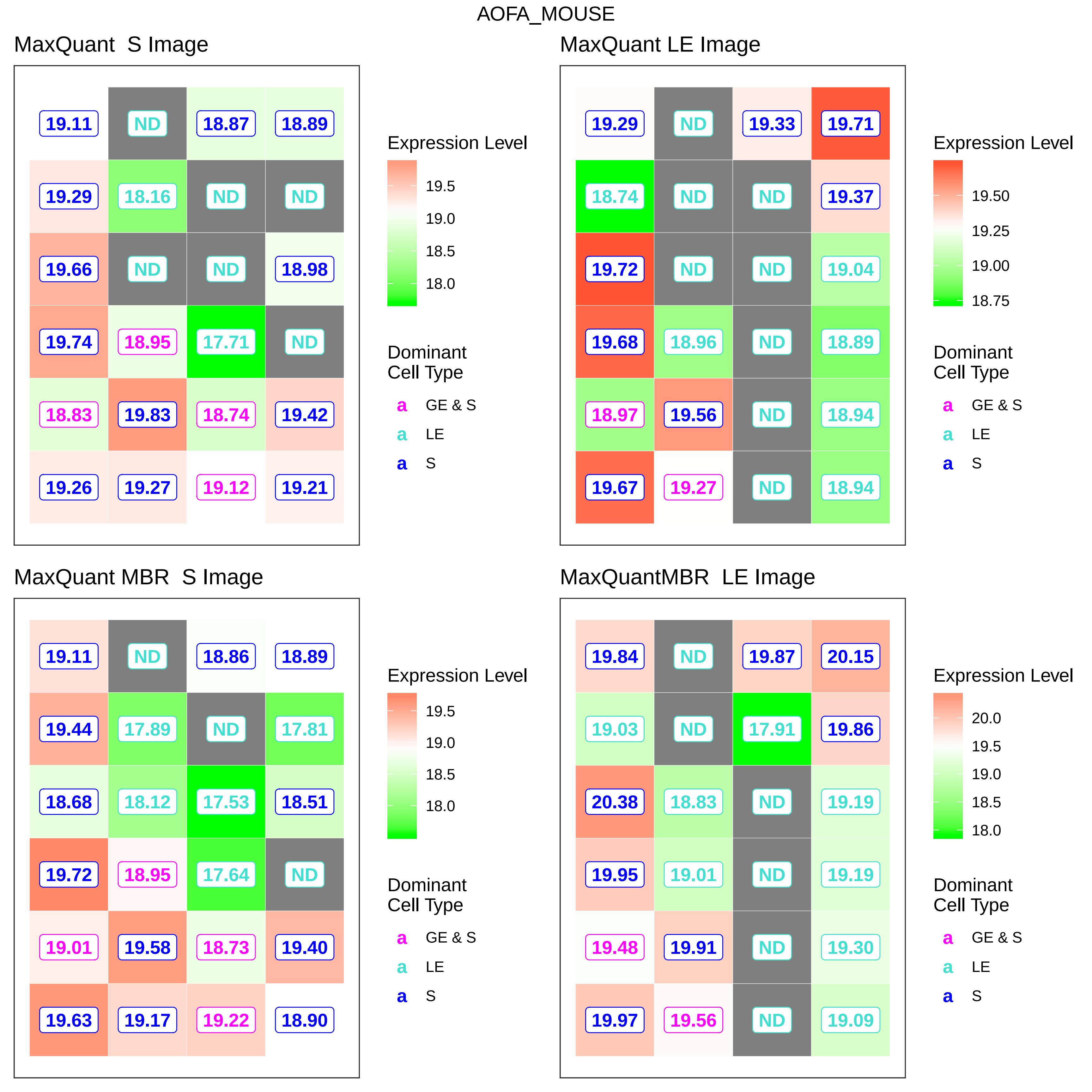

AOFA_MOUSE
MaxQuant S Image
19.11 ND 18.87 18.89
19.29 18.16 ND ND
19.66 ND ND 18.98
19.74 18.95 17.71 ND
18.83 19.83 18.74 19.42
19.26 19.27 19.12 19.21
Expression Level
19.5
19.0
18.5
18.0
Dominant Cell Type
a GE & S
a LE
a S
MaxQuant LE Image
19.29 ND 19.33 19.71
18.74 ND ND 19.37
19.72 ND ND 19.04
19.68 18.96 ND 18.89
18.97 19.56 ND 18.94
19.67 19.27 ND 18.94
Expression Level
19.50
19.25
19.00
18.75
Dominant Cell Type
a GE & S
a LE
a S
MaxQuant MBR S Image
19.11 ND 18.86 18.89
19.44 17.89 ND 17.81
18.68 18.12 17.53 18.51
19.72 18.95 17.64 ND
19.01 19.58 18.73 19.40
19.63 19.17 19.22 18.90
Expression Level
19.5
19.0
18.5
18.0
Dominant Cell Type
a GE & S
a LE
a S
MaxQuantMBR LE Image
19.84 ND 19.87 20.15
19.03 ND 17.91 19.86
20.38 18.83 ND 19.19
19.95 19.01 ND 19.19
19.48 19.91 ND 19.30
19.97 19.56 ND 19.09
Expression Level
20.0
19.5
19.0
18.5
18.0
Dominant Cell Type
a GE & S
a LE
a S

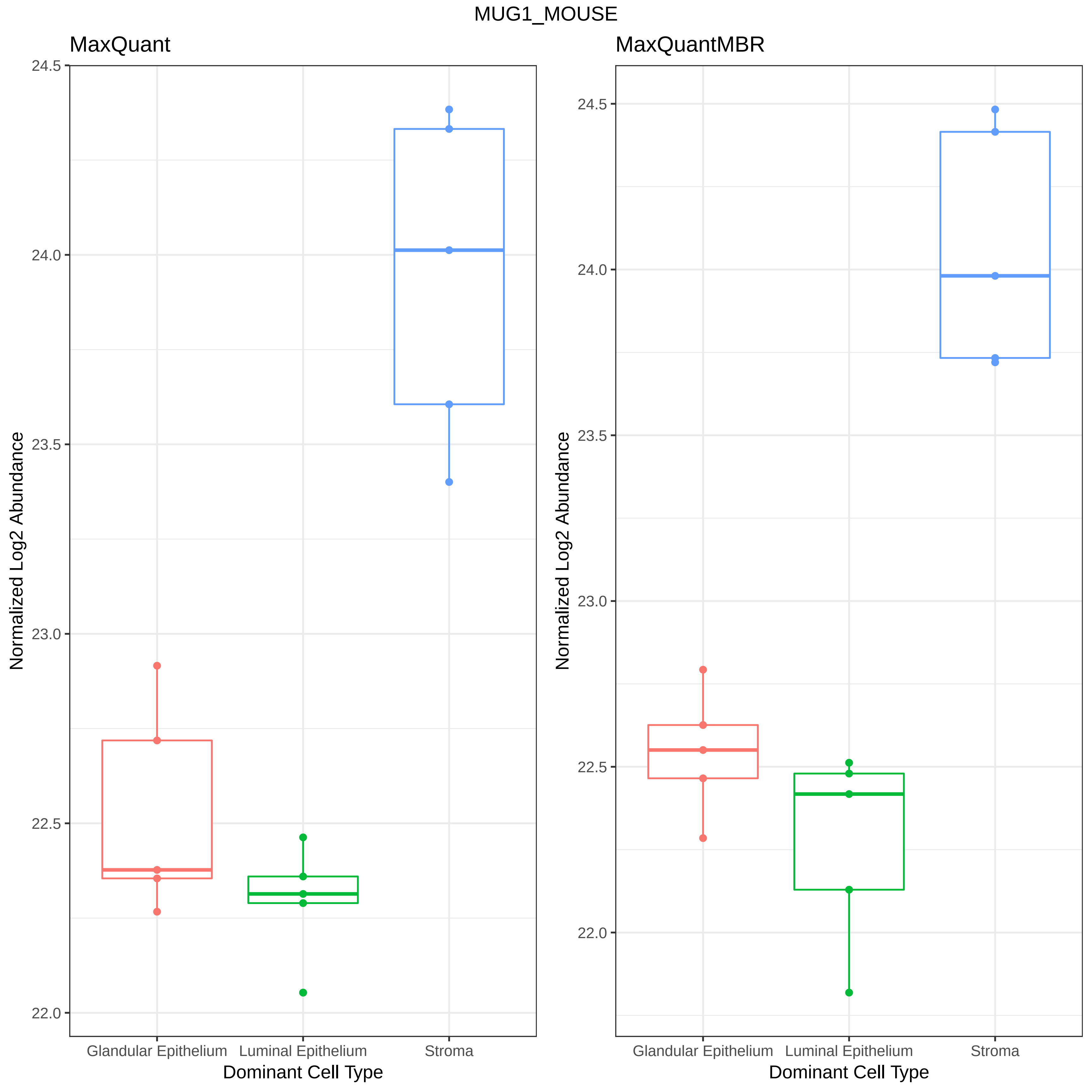
MUG1_MOUSE
MaxQuant
MaxQuantMBR
Normalized Log2 Abundance
24.5
24.0
23.5
23.0
22.5
22.0
Glandular Epithelium
Luminal Epithelium
Stroma
Dominant Cell Type

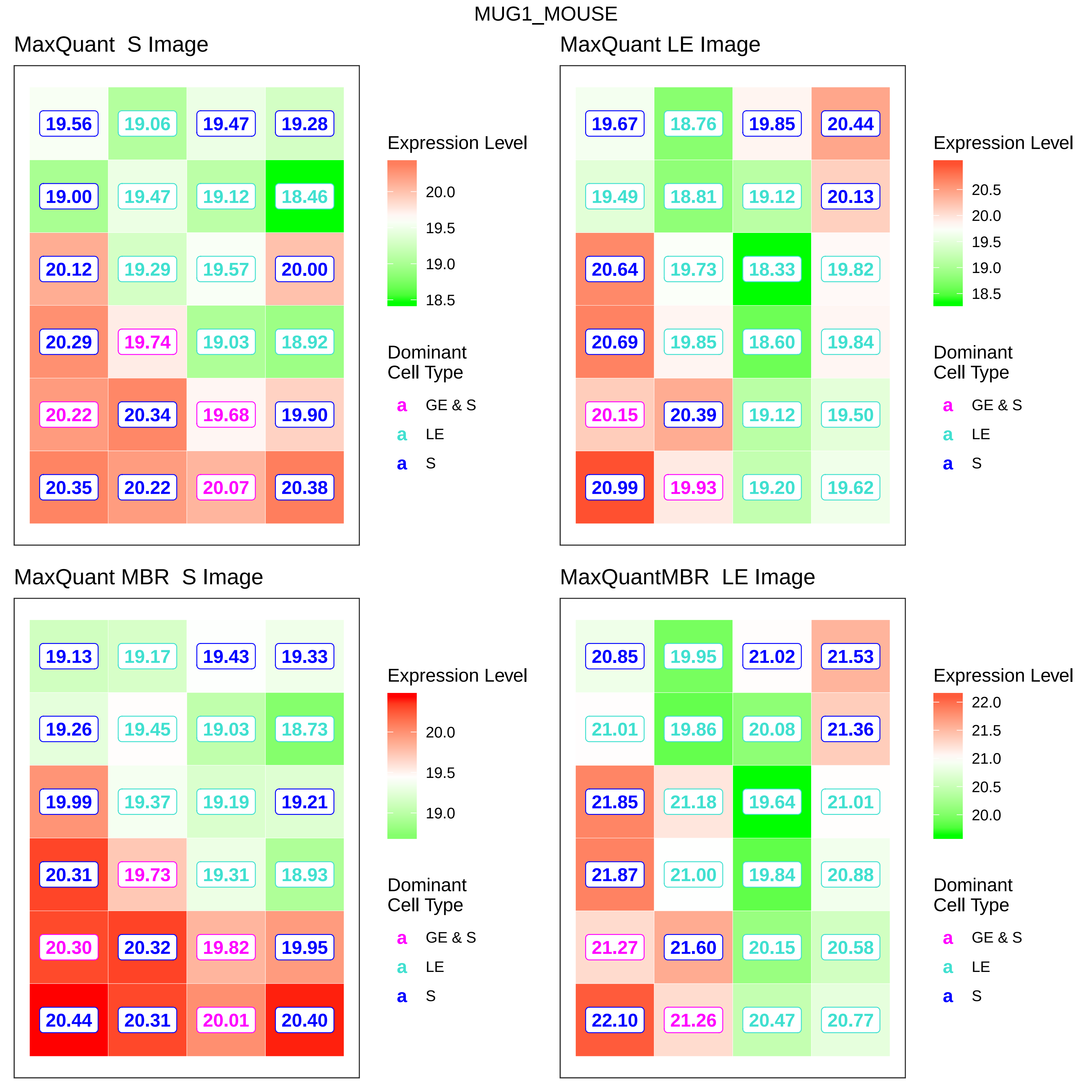
MUG1_MOUSE
MaxQuant S Image
19.56 19.06 19.47 19.28
19.00 19.47 19.12 18.46
20.12 19.29 19.57 20.00
20.29 19.74 19.03 18.92
20.22 20.34 19.68 19.90
20.35 20.22 20.07 20.38
Expression Level
20.0
19.5
19.0
18.5
Dominant Cell Type
a GE & S
a LE
a S
MaxQuant LE Image
19.67 18.76 19.85 20.44
19.49 18.81 19.12 20.13
20.64 19.73 18.33 19.82
20.69 19.85 18.60 19.84
20.15 20.39 19.12 19.50
20.99 19.93 19.20 19.62
Expression Level
20.5
20.0
19.5
19.0
18.5
Dominant Cell Type
a GE & S
a LE
a S
MaxQuant MBR S Image
19.13 19.17 19.43 19.33
19.26 19.45 19.03 18.73
19.99 19.37 19.19 19.21
20.31 19.73 19.31 18.93
20.30 20.32 19.82 19.95
20.44 20.31 20.01 20.40
Expression Level
20.0
19.5
19.0
Dominant Cell Type
a GE & S
a LE
a S
MaxQuantMBR LE Image
20.85 19.95 21.02 21.53
21.01 19.86 20.08 21.36
21.85 21.18 19.64 21.01
21.87 21.00 19.84 20.88
21.27 21.60 20.15 20.58
22.10 21.26 20.47 20.77
Expression Level
22.0
21.5
21.0
20.5
20.0
Dominant Cell Type
a GE & S
a LE
a S

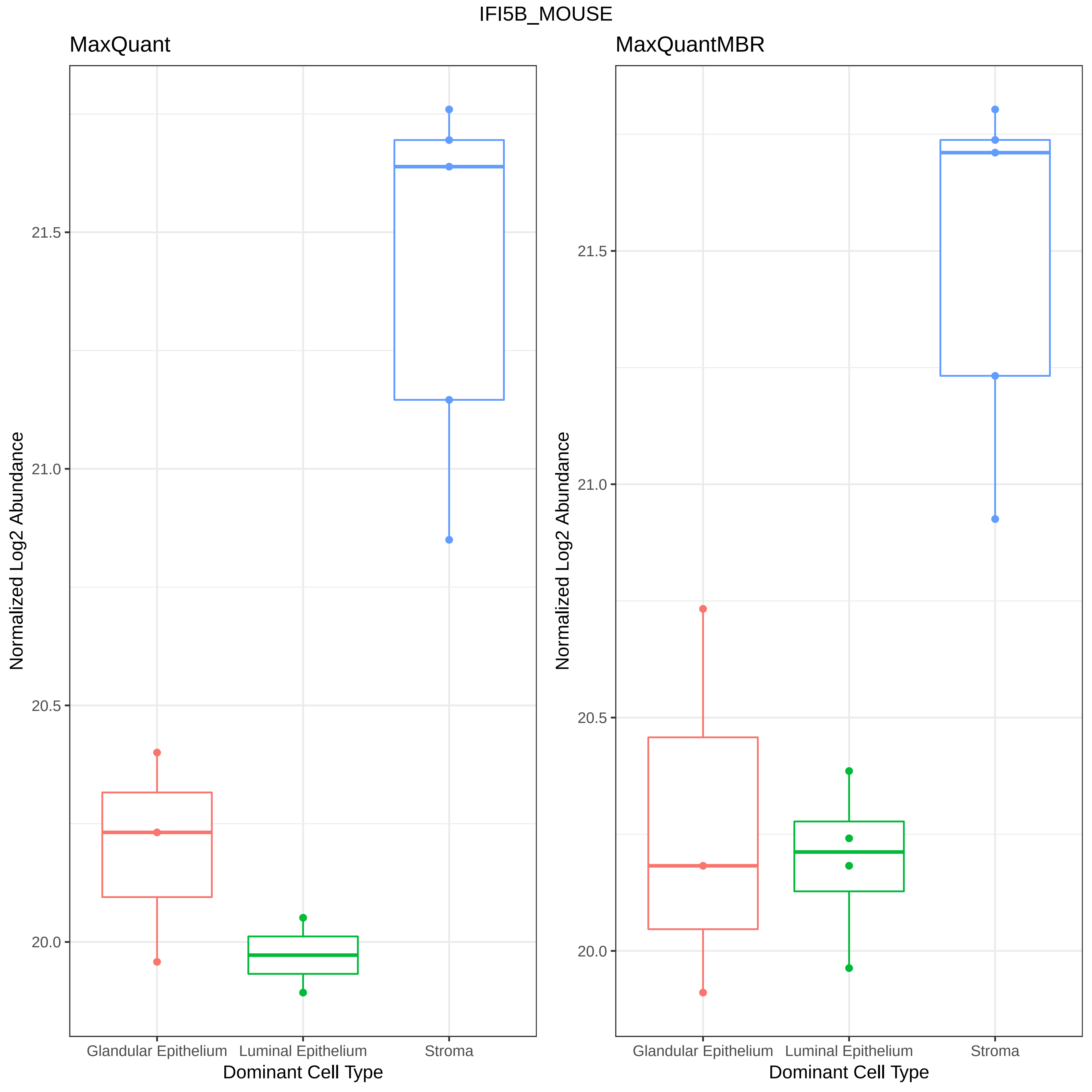
IFI5B_MOUSE
MaxQuant
MaxQuantMBR
Normalized Log2 Abundance
21.5
21.0
20.5
20.0
Glandular Epithelium
Luminal Epithelium
Stroma
Dominant Cell Type

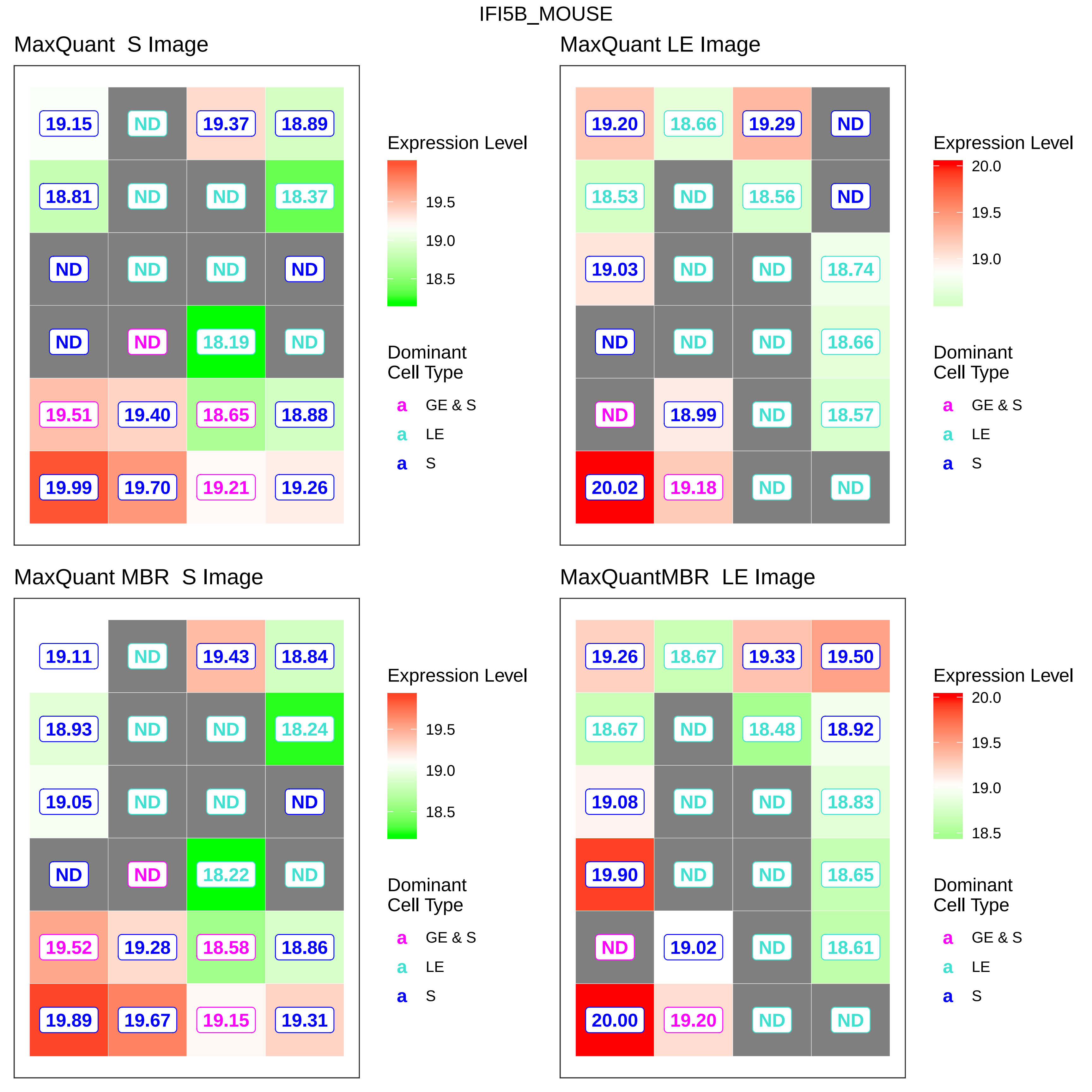

IFI5B_MOUSE
MaxQuant S Image
19.15 ND 19.37 18.89
18.81 ND ND 18.37
ND ND ND ND
ND ND 18.19 ND
19.51 19.40 18.65 18.88
19.99 19.70 19.21 19.26
Expression Level
19.5
19.0
18.5
Dominant Cell Type
a GE & S
a LE
a S
MaxQuant LE Image
19.20 18.66 19.29 ND
18.53 ND 18.56 ND
19.03 ND ND 18.74
ND ND ND 18.66
ND 18.99 ND 18.57
20.02 19.18 ND ND
Expression Level
20.0
19.5
19.0
Dominant Cell Type
a GE & S
a LE
a S
MaxQuant MBR S Image
19.11 ND 19.43 18.84
18.93 ND ND 18.24
19.05 ND ND ND
ND ND 18.22 ND
19.52 19.28 18.58 18.86
19.89 19.67 19.15 19.31
Expression Level
19.5
19.0
18.5
Dominant Cell Type
a GE & S
a LE
a S
MaxQuantMBR LE Image
19.26 18.67 19.33 19.50
18.67 ND 18.48 18.92
19.08 ND ND 18.83
19.90 ND ND 18.65
ND 19.02 ND 18.61
20.00 19.20 ND ND
Expression Level
20.0
19.5
19.0
18.5
Dominant Cell Type
a GE & S
a LE
a S

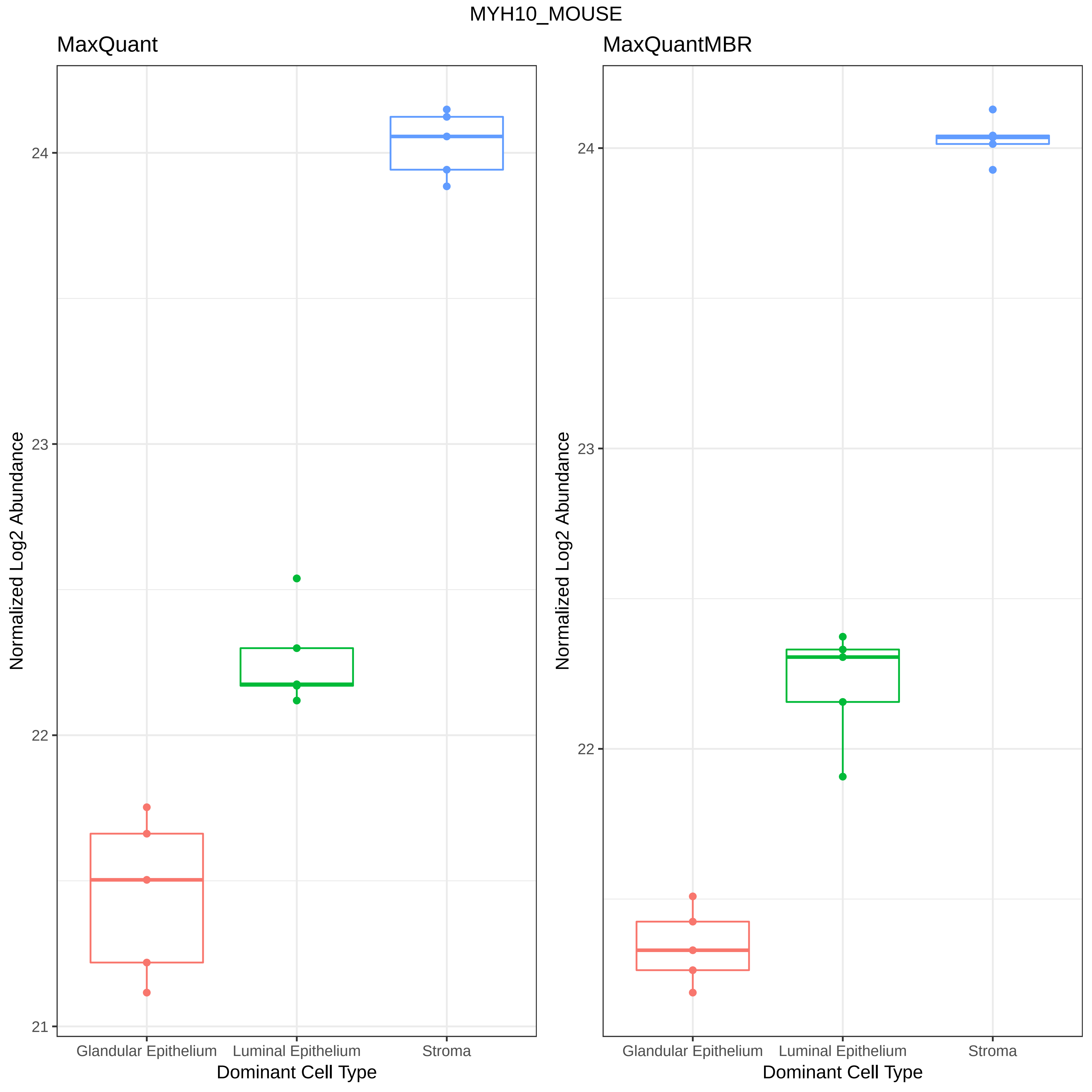

MYH10_MOUSE
MaxQuant
MaxQuantMBR
Normalized Log2 Abundance
24
23
22
21
Glandular Epithelium
Luminal Epithelium
Stroma
Dominant Cell Type

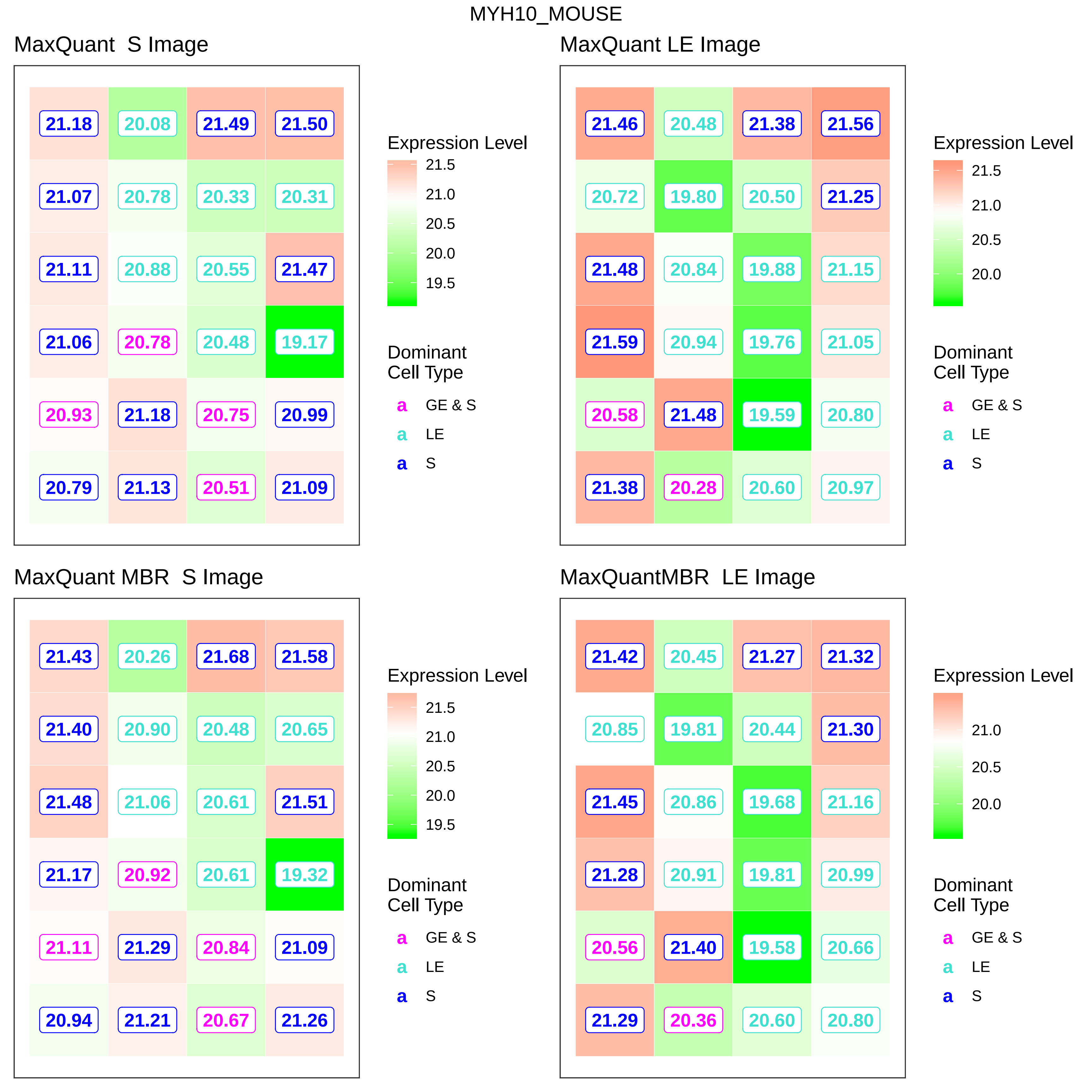
MYH10_MOUSE
MaxQuant S Image
21.18 20.08 21.49 21.50
21.07 20.78 20.33 20.31
21.11 20.88 20.55 21.47
21.06 20.78 20.48 19.17
20.93 21.18 20.75 20.99
20.79 21.13 20.51 21.09
Expression Level
21.5
21.0
20.5
20.0
19.5
Dominant Cell Type
a GE & S
a LE
a S
MaxQuant LE Image
21.46 20.48 21.38 21.56
20.72 19.80 20.50 21.25
21.48 20.84 19.88 21.15
21.59 20.94 19.76 21.05
20.58 21.48 19.59 20.80
21.38 20.28 20.60 20.97
Expression Level
21.5
21.0
20.5
20.0
Dominant Cell Type
a GE & S
a LE
a S
MaxQuant MBR S Image
21.43 20.26 21.68 21.58
21.40 20.90 20.48 20.65
21.48 21.06 20.61 21.51
21.17 20.92 20.61 19.32
21.11 21.29 20.84 21.09
20.94 21.21 20.67 21.26
Expression Level
21.5
21.0
20.5
20.0
19.5
Dominant Cell Type
a GE & S
a LE
a S
MaxQuantMBR LE Image
21.42 20.45 21.27 21.32
20.85 19.81 20.44 21.30
21.45 20.86 19.68 21.16
21.28 20.91 19.81 20.99
20.56 21.40 19.58 20.66
21.29 20.36 20.60 20.80
Expression Level
21.0
20.5
20.0
Dominant Cell Type
a GE & S
a LE
a S

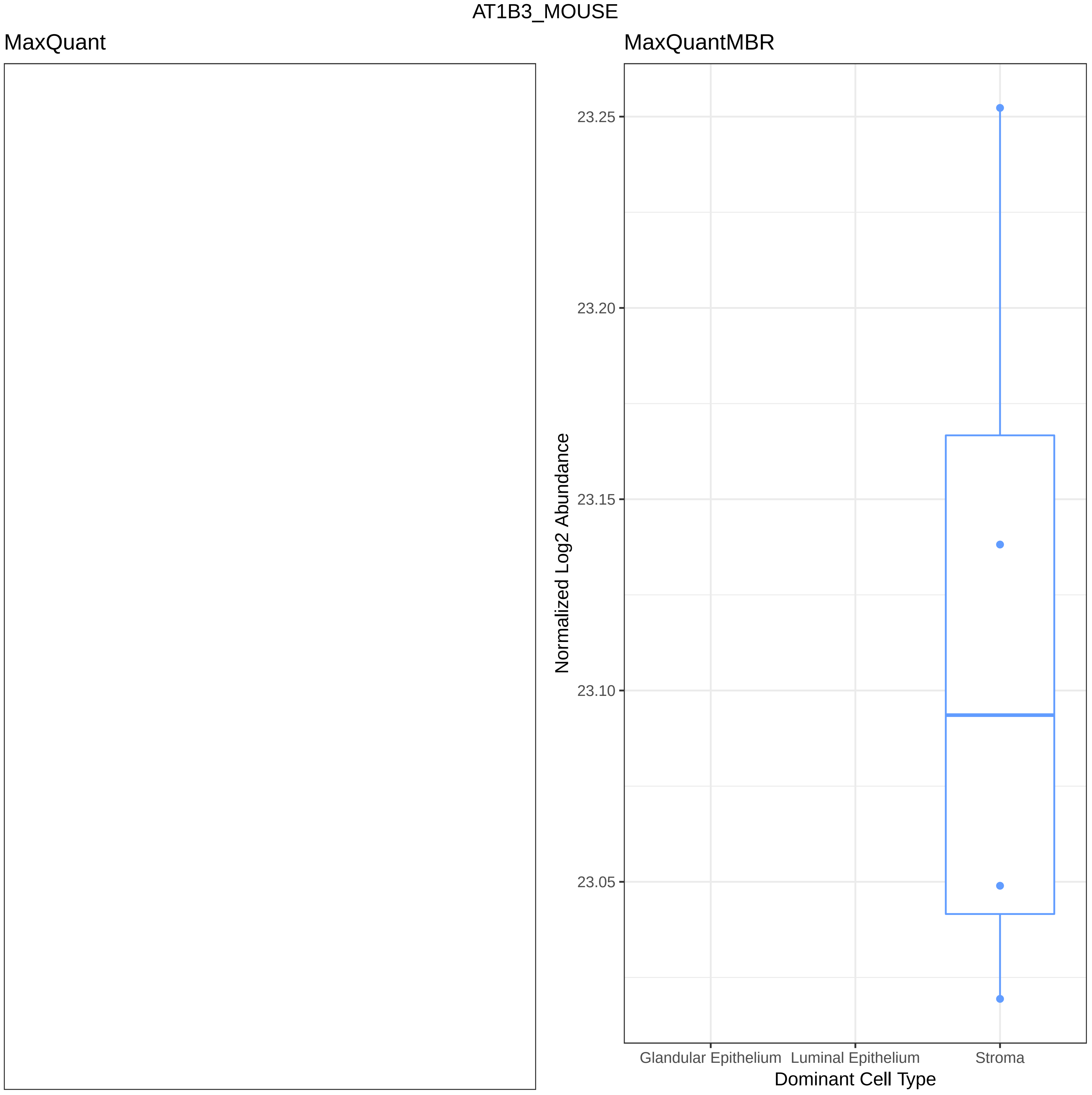
AT1B3_MOUSE
MaxQuant
MaxQuantMBR
Normalized Log2 Abundance
23.25
23.20
23.15
23.10
23.05
Glandular Epithelium
Luminal Epithelium
Stroma
Dominant Cell Type

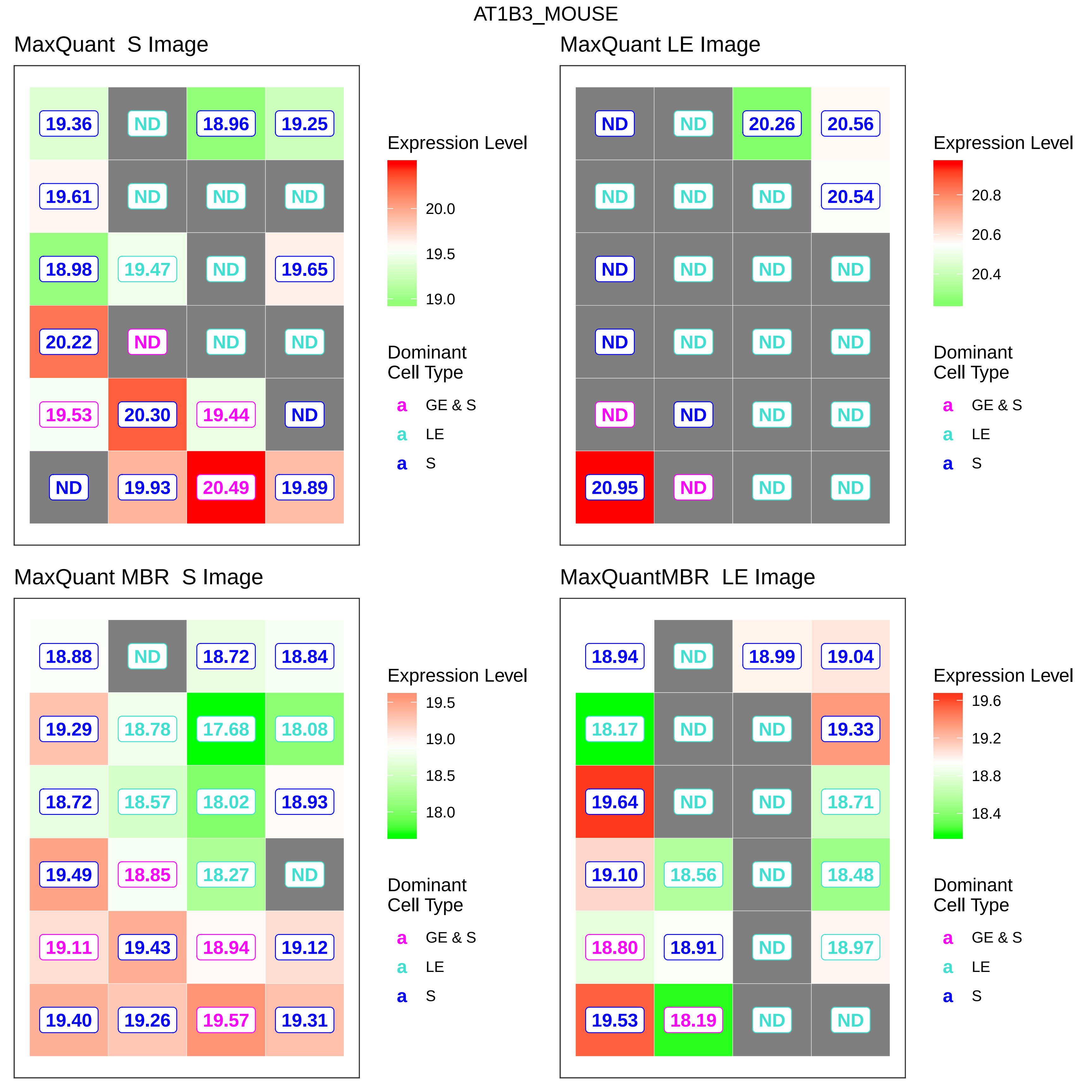
AT1B3_MOUSE
MaxQuant S Image
19.36 ND 18.96 19.25
19.61 ND ND ND
18.98 19.47 ND 19.65
20.22 ND ND ND
19.53 20.30 19.44 ND
ND 19.93 20.49 19.89
Expression Level
20.0
19.5
19.0
Dominant Cell Type
a GE & S
a LE
a S
MaxQuant LE Image
ND ND 20.26 20.56
ND ND ND 20.54
ND ND ND ND
ND ND ND ND
ND ND ND ND
20.95 ND ND ND
Expression Level
20.8
20.6
20.4
Dominant Cell Type
a GE & S
a LE
a S
MaxQuant MBR S Image
18.88 ND 18.72 18.84
19.29 18.78 17.68 18.08
18.72 18.57 18.02 18.93
19.49 18.85 18.27 ND
19.11 19.43 18.94 19.12
19.40 19.26 19.57 19.31
Expression Level
19.5
19.0
18.5
18.0
Dominant Cell Type
a GE & S
a LE
a S
MaxQuantMBR LE Image
18.94 ND 18.99 19.04
18.17 ND ND 19.33
19.64 ND ND 18.71
19.10 18.56 ND 18.48
18.80 18.91 ND 18.97
19.53 18.19 ND ND
Expression Level
19.6
19.2
18.8
18.4
Dominant Cell Type
a GE & S
a LE
a S

NALD2_MOUSE

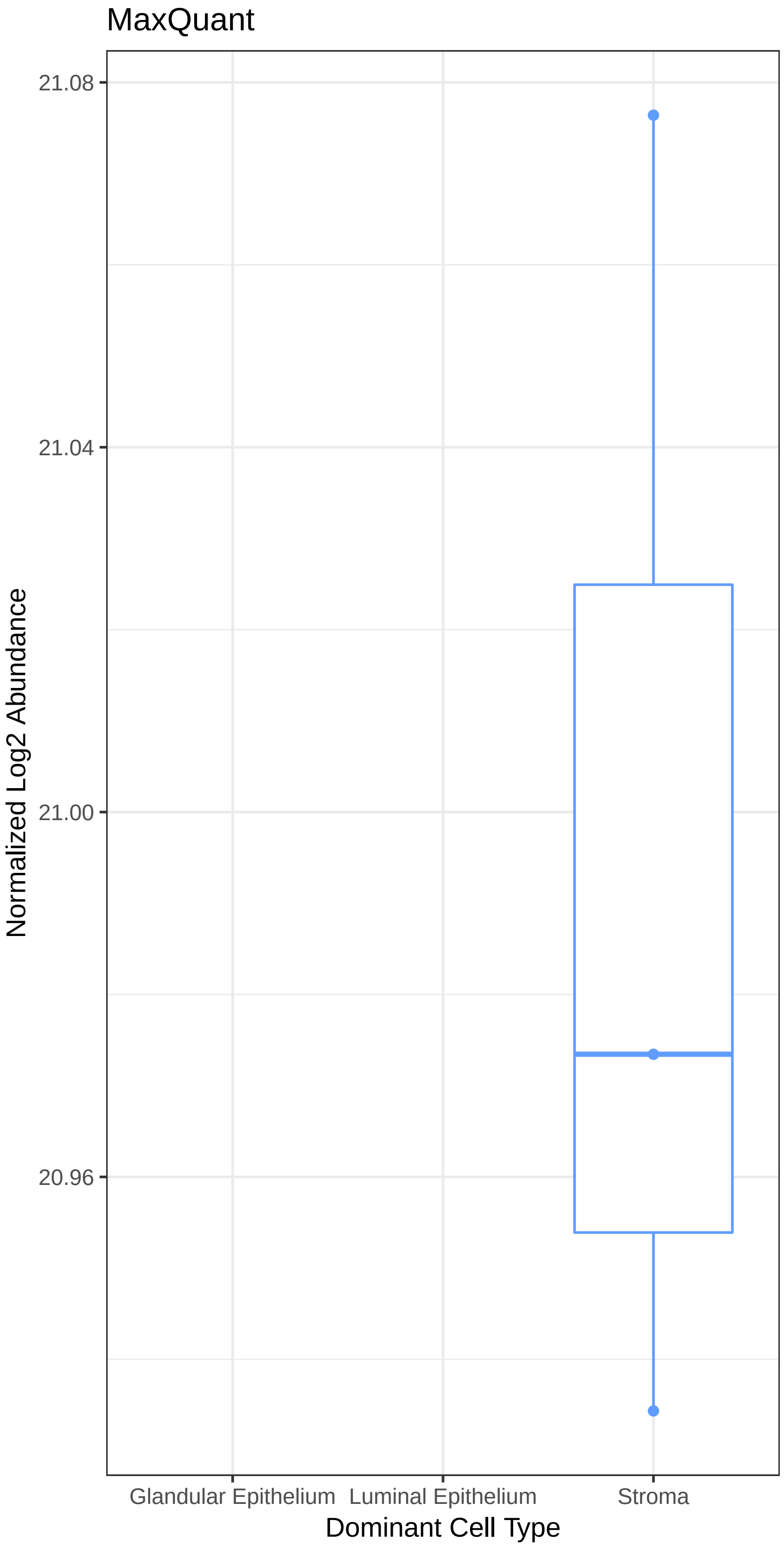

MaxQuant
21.08
21.04
21.00
20.96
Normalized Log2 Abundance
Glandular Epithelium
Luminal Epithelium
Stroma
Dominant Cell Type

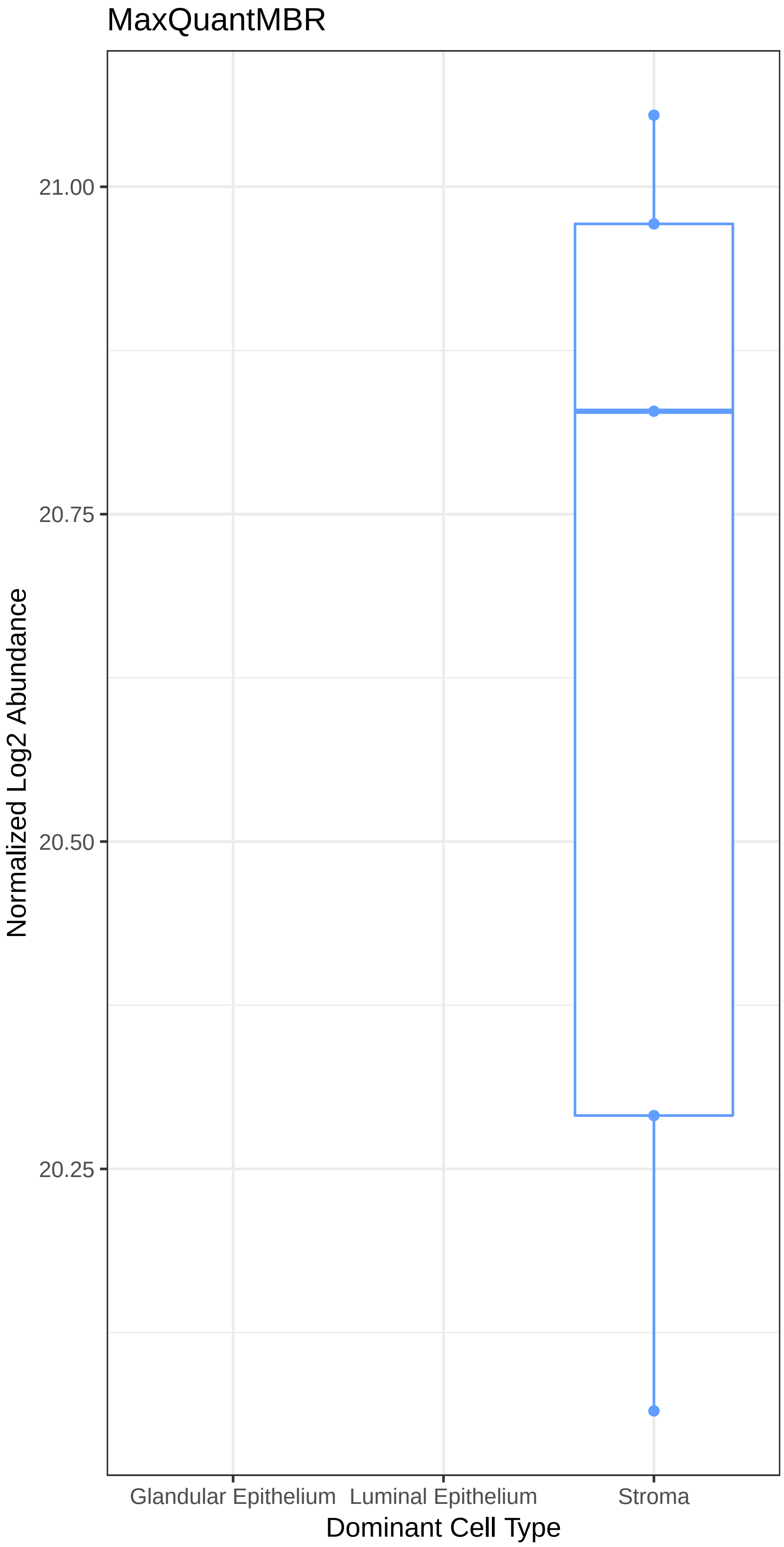

MaxQuantMBR
21.00
20.75
20.50
20.25
Normalized Log2 Abundance
Glandular Epithelium
Luminal Epithelium
Stroma
Dominant Cell Type

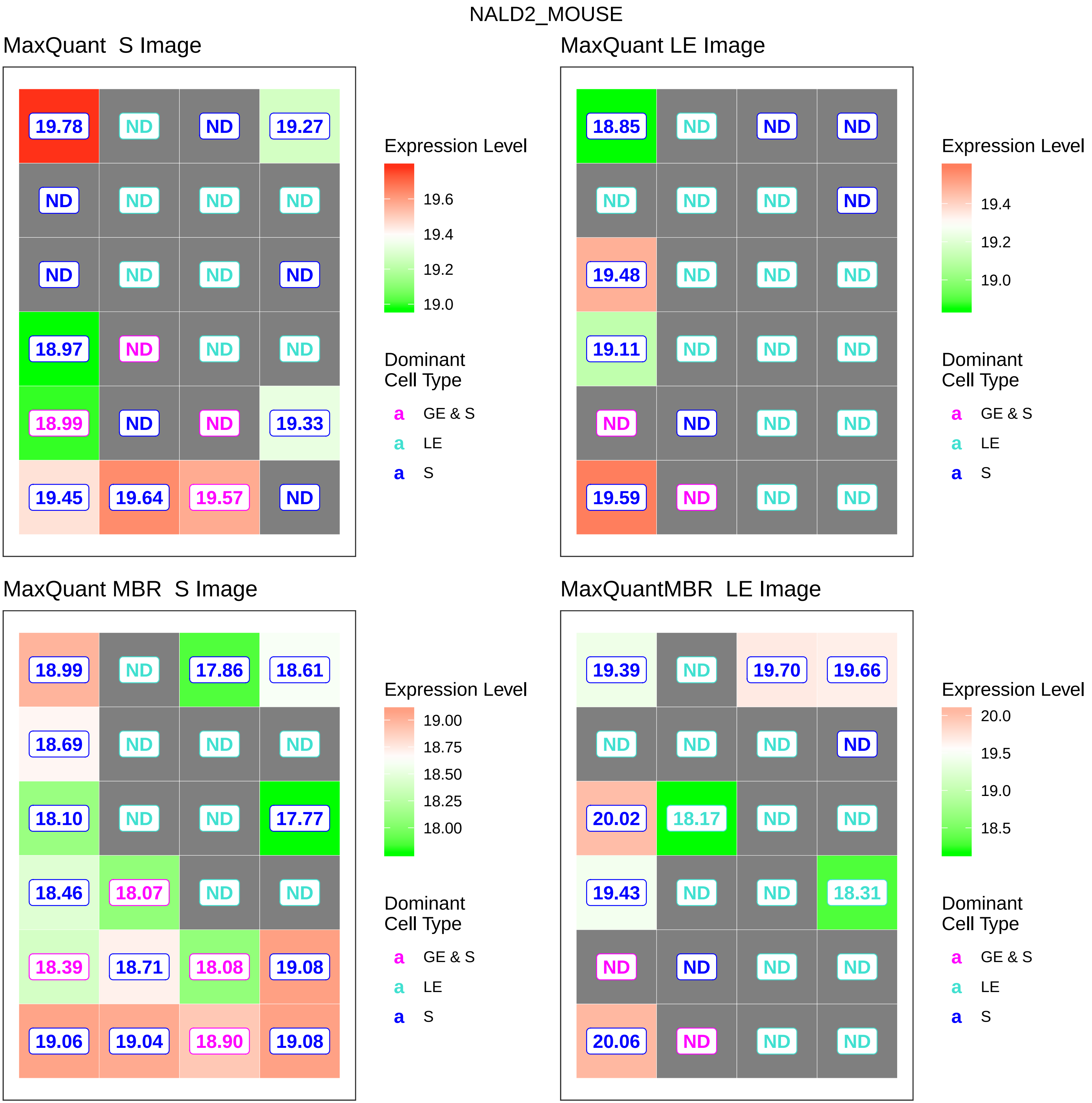
NALD2_MOUSE
MaxQuant S Image
19.78
ND
ND
19.27
ND
ND
ND
ND
ND
ND
ND
ND
18.97
ND
ND
ND
18.99
ND
ND
19.33
19.45
19.64
19.57
ND
Expression Level
19.6
19.4
19.2
19.0
Dominant Cell Type
a GE & S
a LE
a S
MaxQuant LE Image
18.85
ND
ND
ND
ND
ND
ND
ND
19.48
ND
ND
ND
19.11
ND
ND
ND
ND
ND
ND
ND
19.59
ND
ND
ND
Expression Level
19.4
19.2
19.0
Dominant Cell Type
a GE & S
a LE
a S
MaxQuant MBR S Image
18.99
ND
17.86
18.61
18.69
ND
ND
ND
18.10
ND
ND
17.77
18.46
18.07
ND
ND
18.39
18.71
18.08
19.08
19.06
19.04
18.90
19.08
Expression Level
19.00
18.75
18.50
18.25
18.00
Dominant Cell Type
a GE & S
a LE
a S
MaxQuantMBR LE Image
19.39
ND
19.70
19.66
ND
ND
ND
ND
20.02
18.17
ND
ND
19.43
ND
ND
18.31
ND
ND
ND
ND
20.06
ND
ND
ND
Expression Level
20.0
19.5
19.0
18.5
Dominant Cell Type
a GE & S
a LE
a S

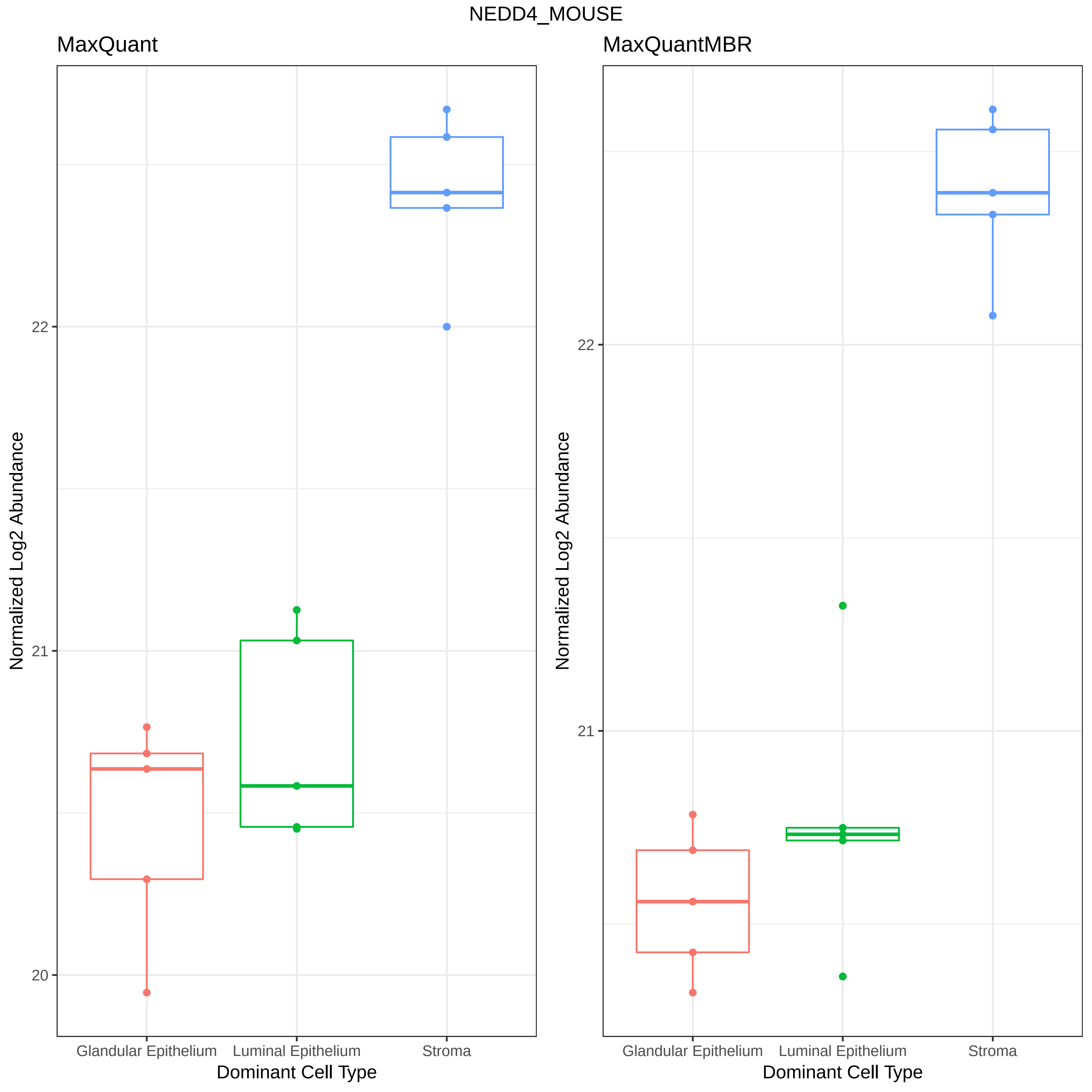
NEDD4_MOUSE
MaxQuant
MaxQuantMBR
Normalized Log2 Abundance
22
21
20
Glandular Epithelium
Luminal Epithelium
Stroma
Dominant Cell Type

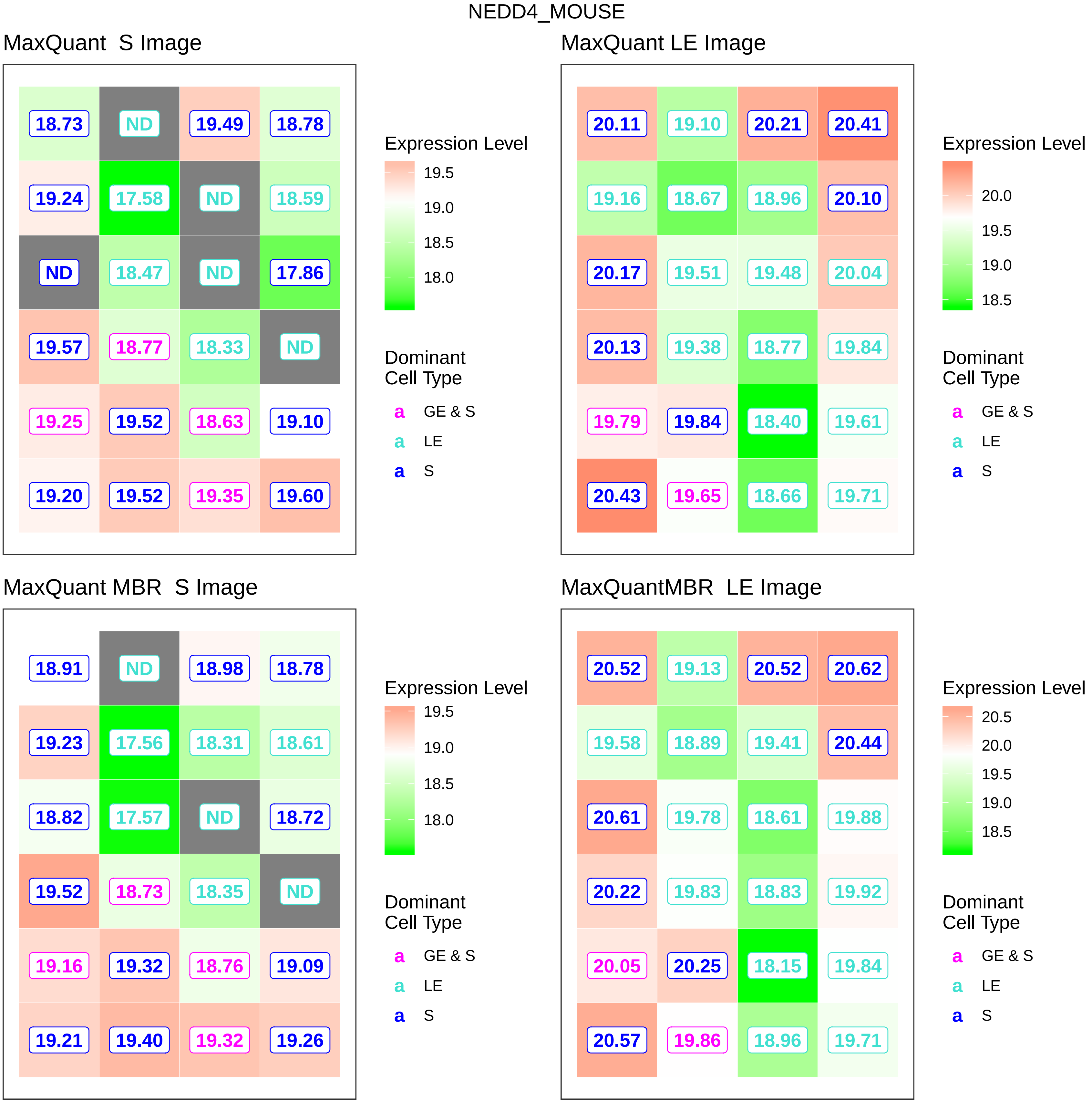
NEDD4_MOUSE
MaxQuant S Image
18.73 ND 19.49 18.78
19.24 17.58 ND 18.59
ND 18.47 ND 17.86
19.57 18.77 18.33 ND
19.25 19.52 18.63 19.10
19.20 19.52 19.35 19.60
Expression Level
19.5
19.0
18.5
18.0
Dominant Cell Type
a GE & S
a LE
a S
MaxQuant LE Image
20.11 19.10 20.21 20.41
19.16 18.67 18.96 20.10
20.17 19.51 19.48 20.04
20.13 19.38 18.77 19.84
19.79 19.84 18.40 19.61
20.43 19.65 18.66 19.71
Expression Level
20.0
19.5
19.0
18.5
Dominant Cell Type
a GE & S
a LE
a S
MaxQuant MBR S Image
18.91 ND 18.98 18.78
19.23 17.56 18.31 18.61
18.82 17.57 ND 18.72
19.52 18.73 18.35 ND
19.16 19.32 18.76 19.09
19.21 19.40 19.32 19.26
Expression Level
19.5
19.0
18.5
18.0
Dominant Cell Type
a GE & S
a LE
a S
MaxQuantMBR LE Image
20.52 19.13 20.52 20.62
19.58 18.89 19.41 20.44
20.61 19.78 18.61 19.88
20.22 19.83 18.83 19.92
20.05 20.25 18.15 19.84
20.57 19.86 18.96 19.71
Expression Level
20.5
20.0
19.5
19.0
18.5
Dominant Cell Type
a GE & S
a LE
a S

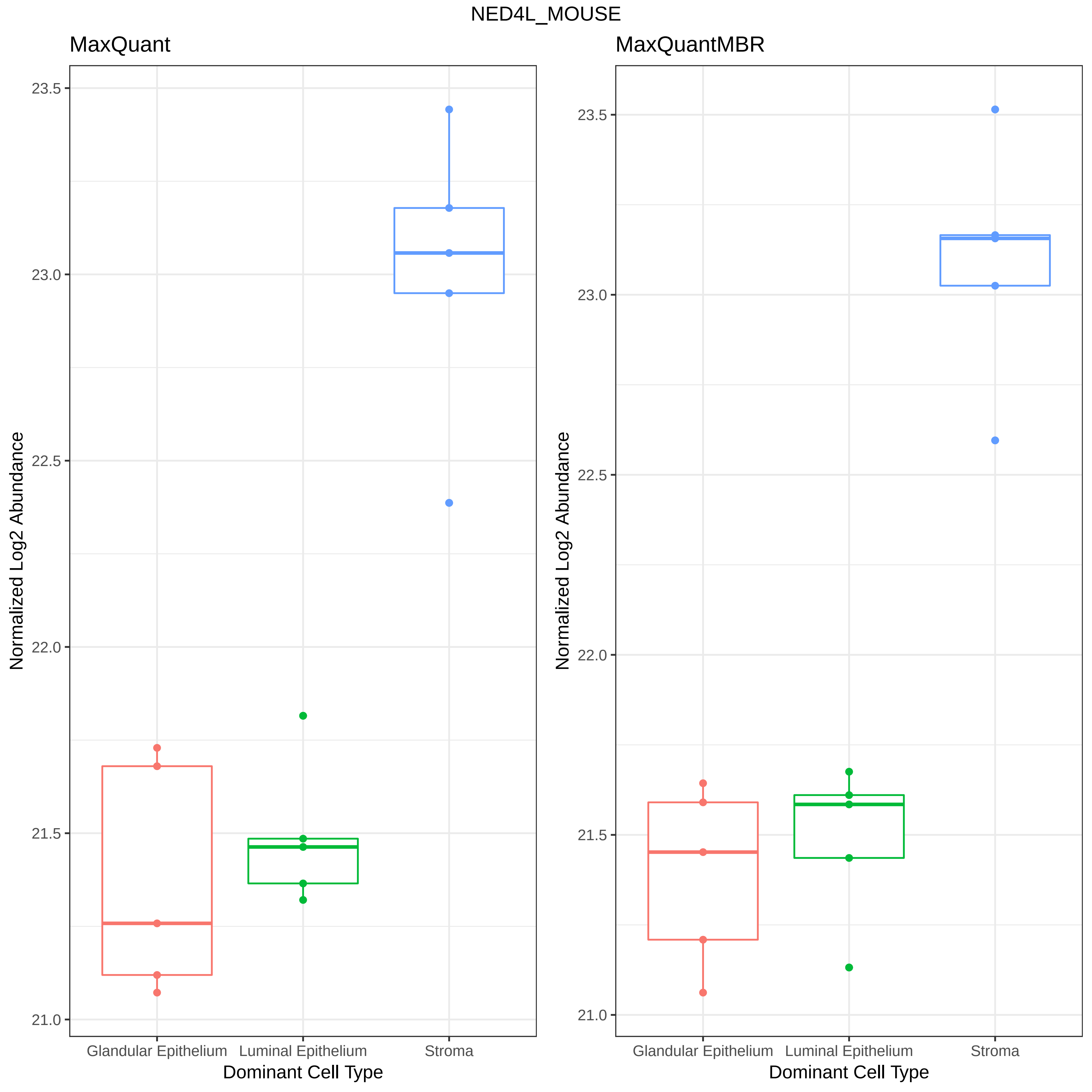

NED4L_MOUSE
MaxQuant
MaxQuantMBR
Normalized Log2 Abundance
23.5
23.0
22.5
22.0
21.5
21.0
Glandular Epithelium
Luminal Epithelium
Stroma
Dominant Cell Type

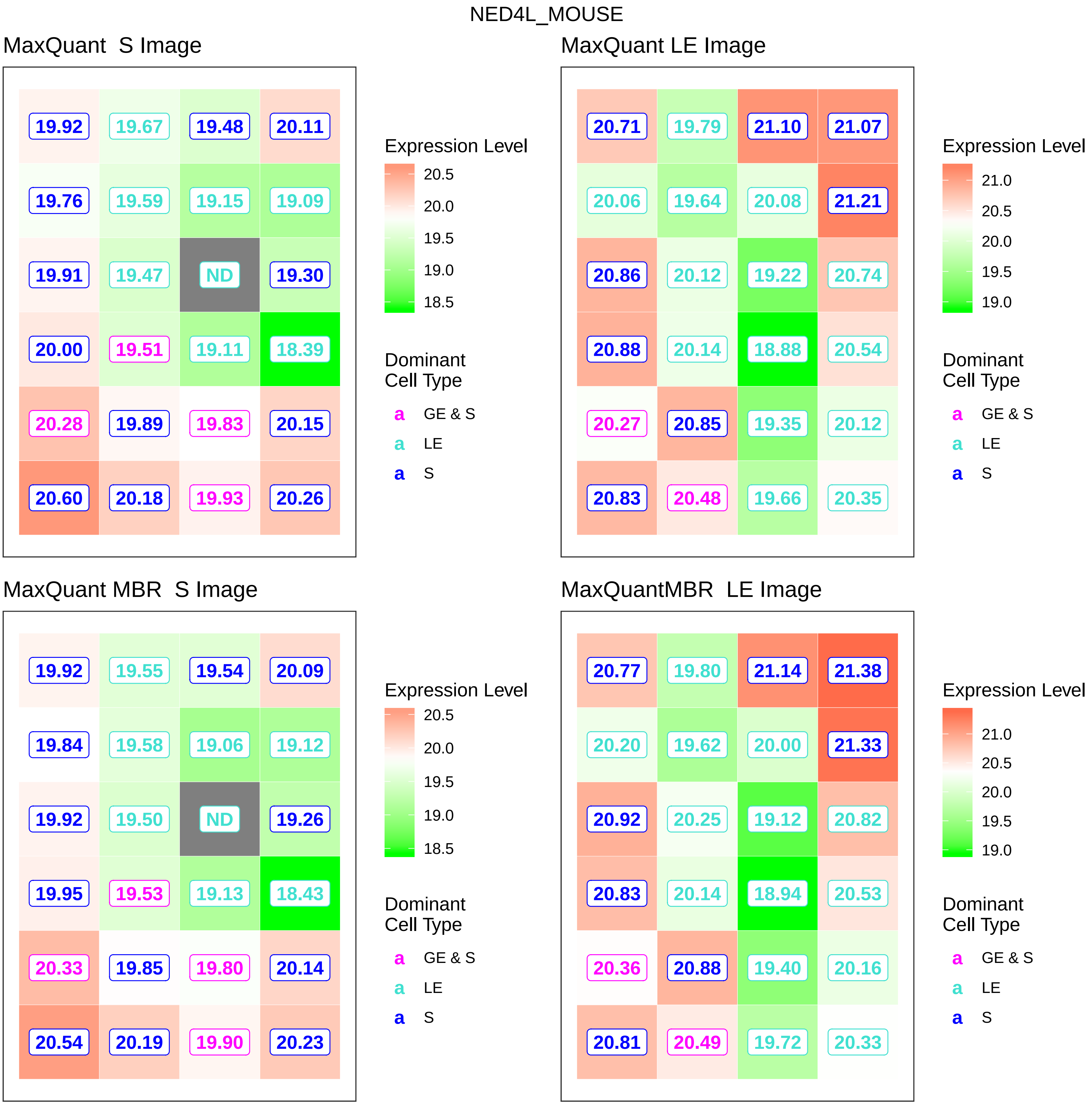

NED4L_MOUSE
MaxQuant S Image
19.92 19.67 19.48 20.11
19.76 19.59 19.15 19.09
19.91 19.47 ND 19.30
20.00 19.51 19.11 18.39
20.28 19.89 19.83 20.15
20.60 20.18 19.93 20.26
Expression Level
20.5
20.0
19.5
19.0
18.5
Dominant Cell Type
a GE & S
a LE
a S
MaxQuant LE Image
20.71 19.79 21.10 21.07
20.06 19.64 20.08 21.21
20.86 20.12 19.22 20.74
20.88 20.14 18.88 20.54
20.27 20.85 19.35 20.12
20.83 20.48 19.66 20.35
Expression Level
21.0
20.5
20.0
19.5
19.0
Dominant Cell Type
a GE & S
a LE
a S
MaxQuant MBR S Image
19.92 19.55 19.54 20.09
19.84 19.58 19.06 19.12
19.92 19.50 ND 19.26
19.95 19.53 19.13 18.43
20.33 19.85 19.80 20.14
20.54 20.19 19.90 20.23
Expression Level
20.5
20.0
19.5
19.0
18.5
Dominant Cell Type
a GE & S
a LE
a S
MaxQuantMBR LE Image
20.77 19.80 21.14 21.38
20.20 19.62 20.00 21.33
20.92 20.25 19.12 20.82
20.83 20.14 18.94 20.53
20.36 20.88 19.40 20.16
20.81 20.49 19.72 20.33
Expression Level
21.0
20.5
20.0
19.5
19.0
Dominant Cell Type
a GE & S
a LE
a S

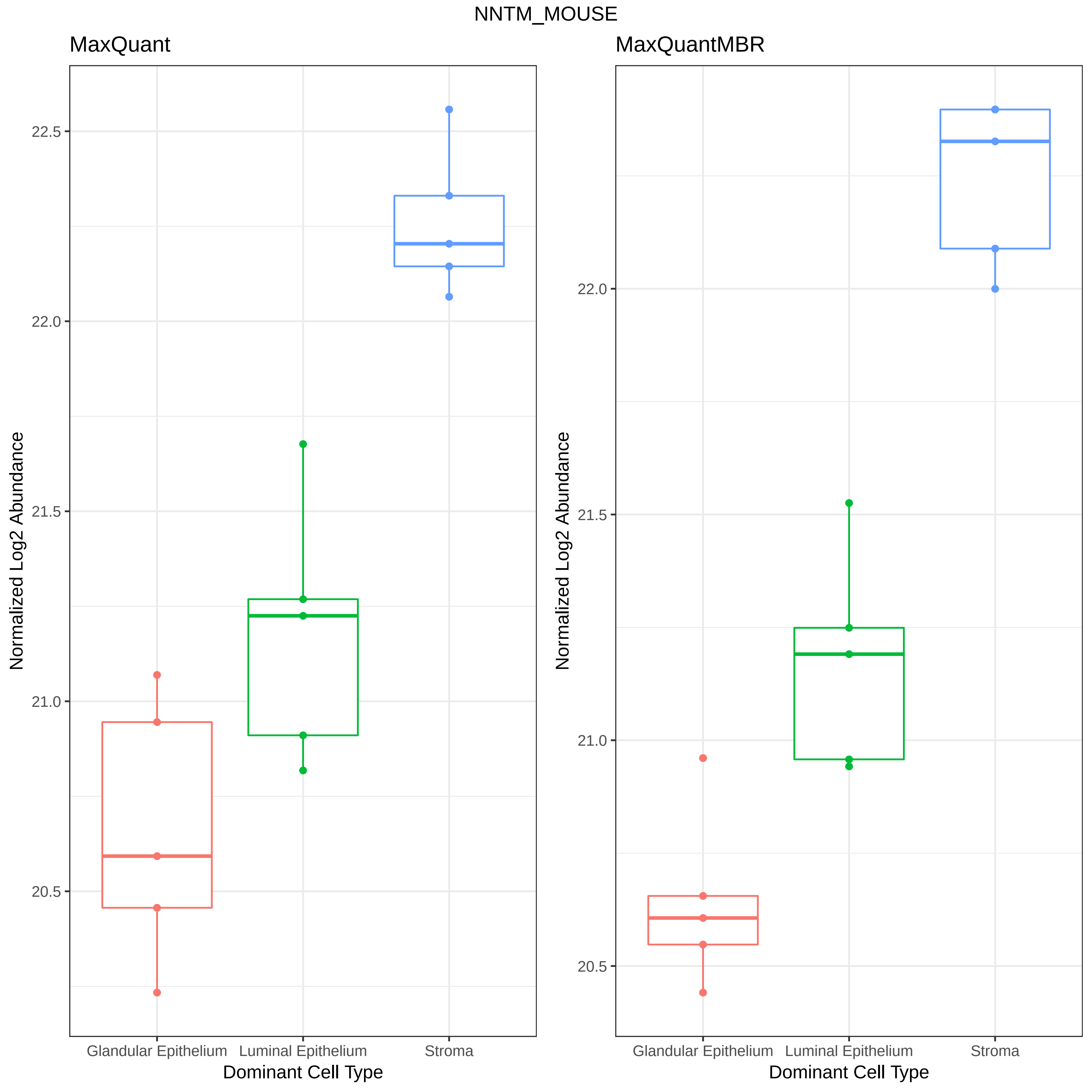
NNTM_MOUSE
MaxQuant
MaxQuantMBR
Normalized Log2 Abundance
22.5
22.0
21.5
21.0
20.5
Glandular Epithelium
Luminal Epithelium
Stroma
Dominant Cell Type

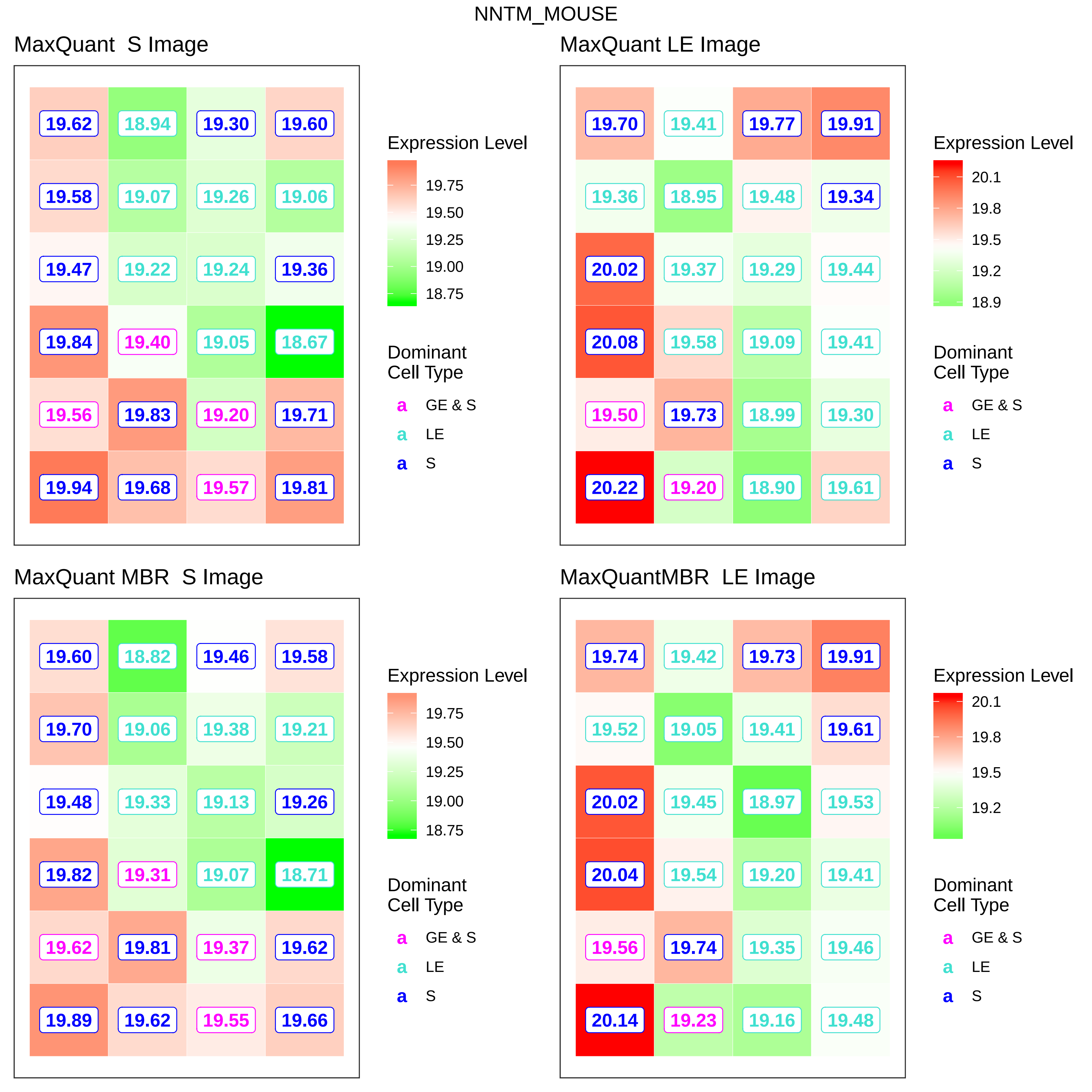
NNTM_MOUSE
MaxQuant S Image
19.62 18.94 19.30 19.60
19.58 19.07 19.26 19.06
19.47 19.22 19.24 19.36
19.84 19.40 19.05 18.67
19.56 19.83 19.20 19.71
19.94 19.68 19.57 19.81
Expression Level
19.75
19.50
19.25
19.00
18.75
Dominant Cell Type
a GE & S
a LE
a S
MaxQuant LE Image
19.70 19.41 19.77 19.91
19.36 18.95 19.48 19.34
20.02 19.37 19.29 19.44
20.08 19.58 19.09 19.41
19.50 19.73 18.99 19.30
20.22 19.20 18.90 19.61
Expression Level
20.1
19.8
19.5
19.2
18.9
Dominant Cell Type
a GE & S
a LE
a S
MaxQuant MBR S Image
19.60 18.82 19.46 19.58
19.70 19.06 19.38 19.21
19.48 19.33 19.13 19.26
19.82 19.31 19.07 18.71
19.62 19.81 19.37 19.62
19.89 19.62 19.55 19.66
Expression Level
19.75
19.50
19.25
19.00
18.75
Dominant Cell Type
a GE & S
a LE
a S
MaxQuantMBR LE Image
19.74 19.42 19.73 19.91
19.52 19.05 19.41 19.61
20.02 19.45 18.97 19.53
20.04 19.54 19.20 19.41
19.56 19.74 19.35 19.46
20.14 19.23 19.16 19.48
Expression Level
20.1
19.8
19.5
19.2
Dominant Cell Type
a GE & S
a LE
a S

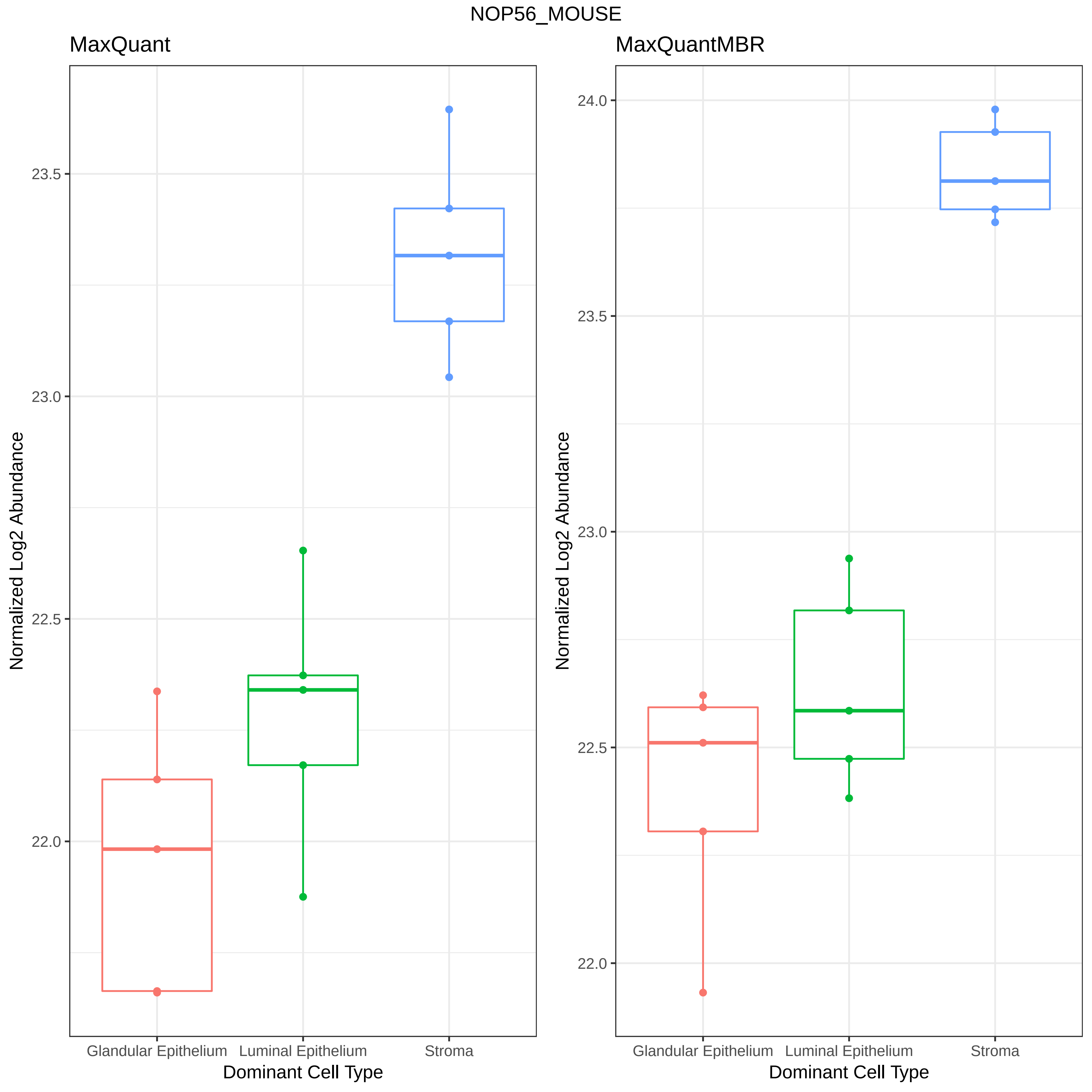
NOP56_MOUSE
MaxQuant
MaxQuantMBR
Normalized Log2 Abundance
23.5
23.0
22.5
22.0
24.0
Glandular Epithelium
Luminal Epithelium
Stroma
Dominant Cell Type

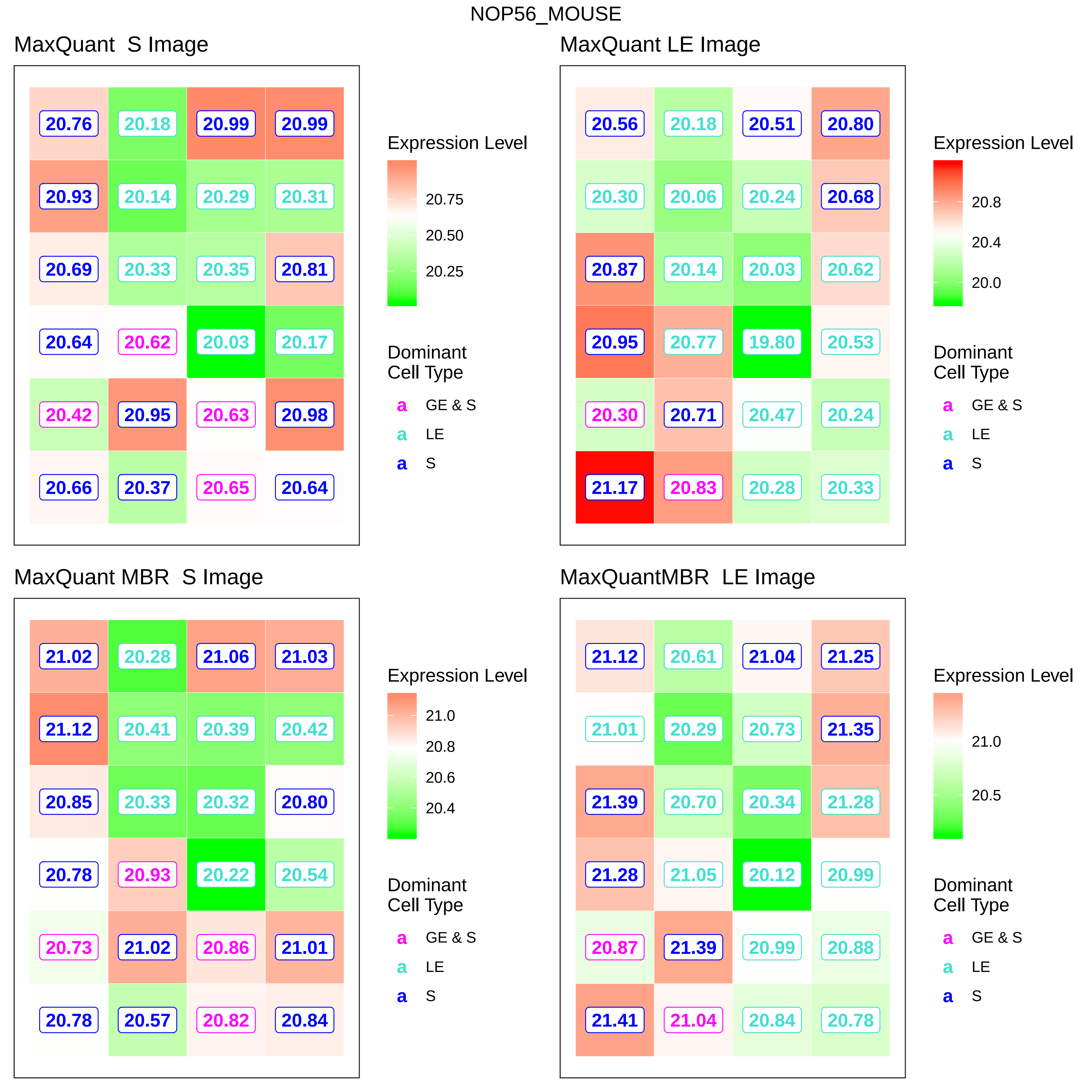
NOP56_MOUSE
MaxQuant S Image
20.76 20.18 20.99 20.99
20.93 20.14 20.29 20.31
20.69 20.33 20.35 20.81
20.64 20.62 20.03 20.17
20.42 20.95 20.63 20.98
20.66 20.37 20.65 20.64
Expression Level
20.75
20.50
20.25
Dominant Cell Type
a GE & S
a LE
a S
MaxQuant LE Image
20.56 20.18 20.51 20.80
20.30 20.06 20.24 20.68
20.87 20.14 20.03 20.62
20.95 20.77 19.80 20.53
20.30 20.71 20.47 20.24
21.17 20.83 20.28 20.33
Expression Level
20.8
20.4
20.0
Dominant Cell Type
a GE & S
a LE
a S
MaxQuant MBR S Image
21.02 20.28 21.06 21.03
21.12 20.41 20.39 20.42
20.85 20.33 20.32 20.80
20.78 20.93 20.22 20.54
20.73 21.02 20.86 21.01
20.78 20.57 20.82 20.84
Expression Level
21.0
20.8
20.6
20.4
Dominant Cell Type
a GE & S
a LE
a S
MaxQuantMBR LE Image
21.12 20.61 21.04 21.25
21.01 20.29 20.73 21.35
21.39 20.70 20.34 21.28
21.28 21.05 20.12 20.99
20.87 21.39 20.99 20.88
21.41 21.04 20.84 20.78
Expression Level
21.0
20.5
Dominant Cell Type
a GE & S
a LE
a S

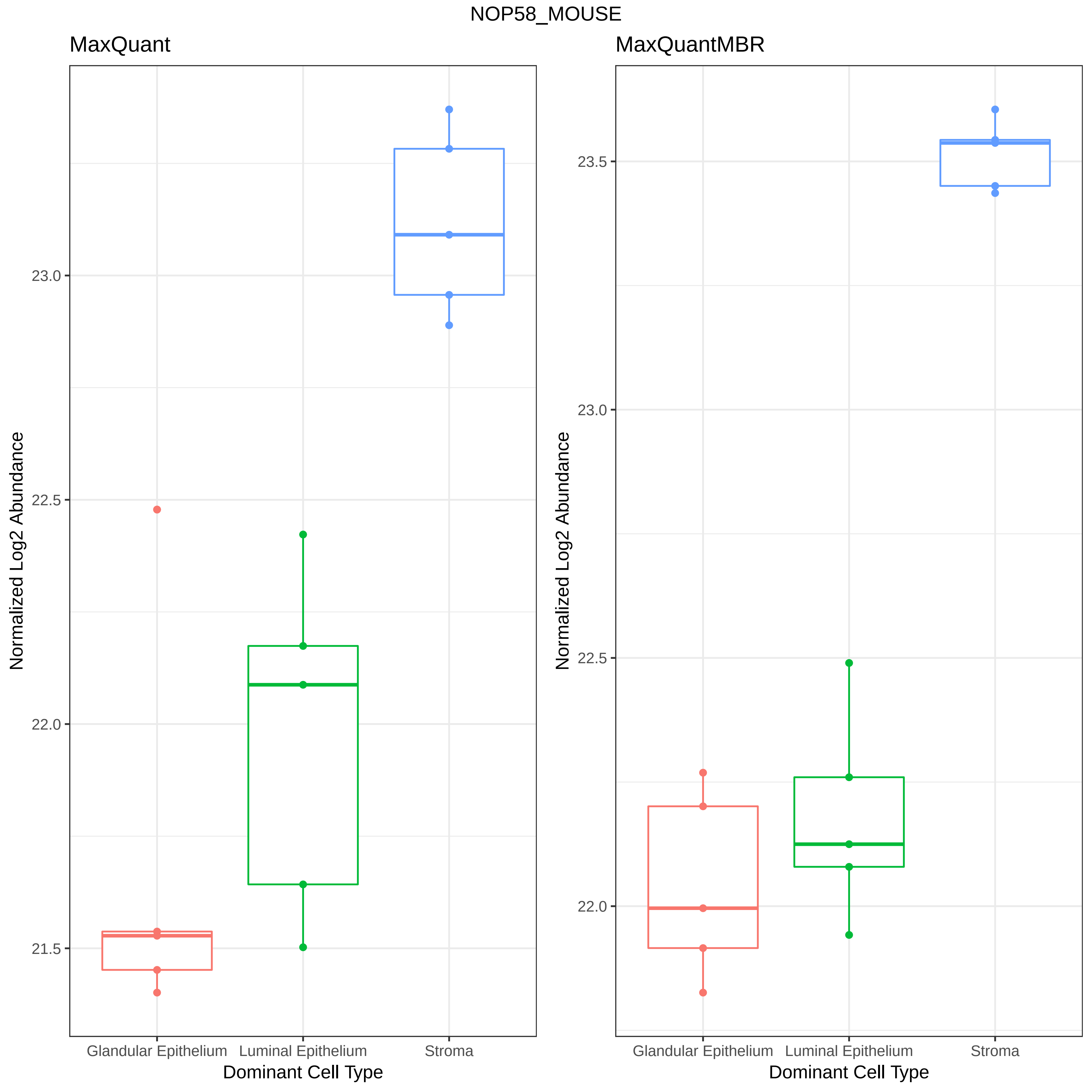
NOP58_MOUSE
MaxQuant
MaxQuantMBR
Normalized Log2 Abundance
23.0
22.5
22.0
21.5
23.5
Glandular Epithelium
Luminal Epithelium
Stroma
Dominant Cell Type

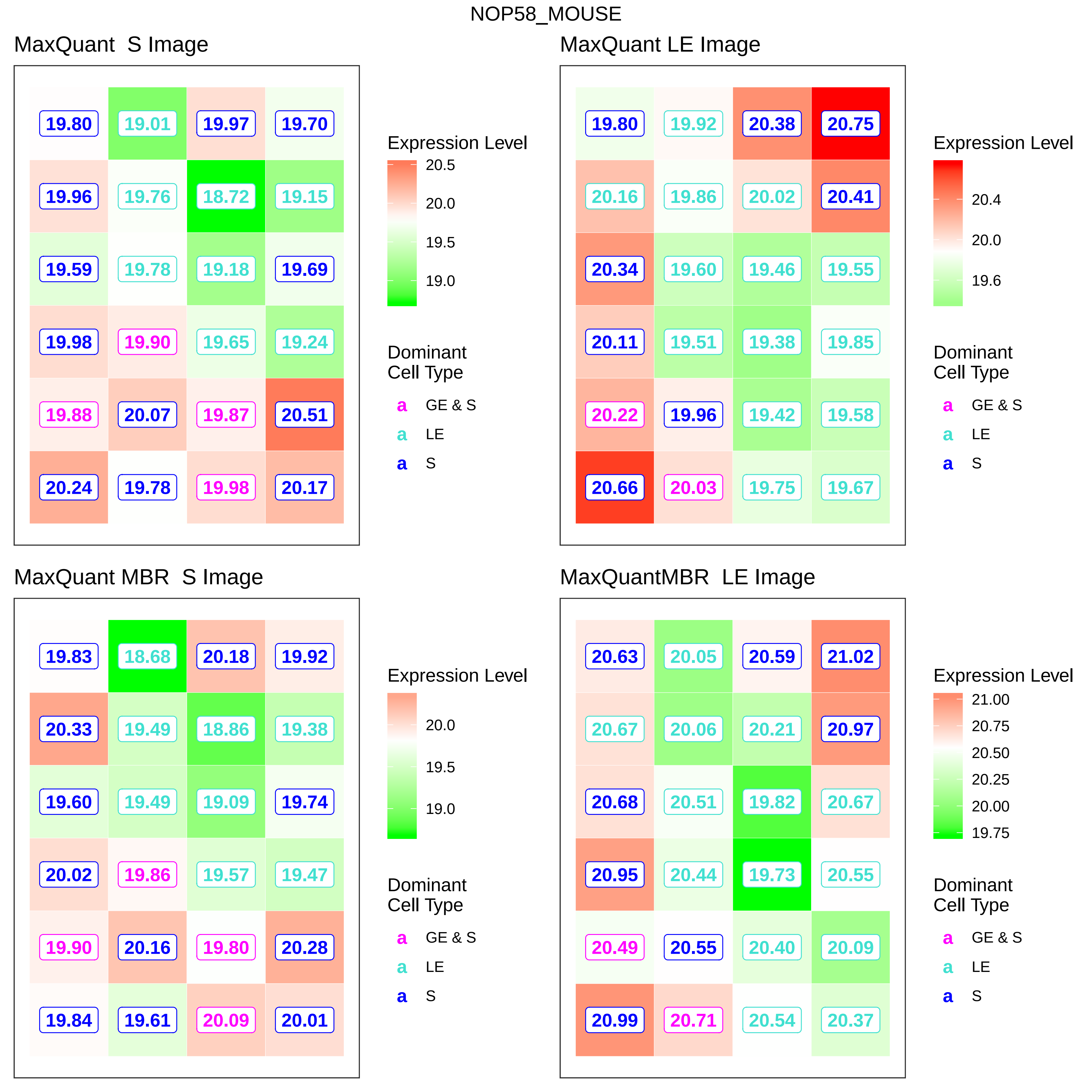
NOP58_MOUSE
MaxQuant S Image
19.80 19.01 19.97 19.70
19.96 19.76 18.72 19.15
19.59 19.78 19.18 19.69
19.98 19.90 19.65 19.24
19.88 20.07 19.87 20.51
20.24 19.78 19.98 20.17
Expression Level
20.5
20.0
19.5
19.0
Dominant Cell Type
a GE & S
a LE
a S
MaxQuant LE Image
19.80 19.92 20.38 20.75
20.16 19.86 20.02 20.41
20.34 19.60 19.46 19.55
20.11 19.51 19.38 19.85
20.22 19.96 19.42 19.58
20.66 20.03 19.75 19.67
Expression Level
20.4
20.0
19.6
Dominant Cell Type
a GE & S
a LE
a S
MaxQuant MBR S Image
19.83 18.68 20.18 19.92
20.33 19.49 18.86 19.38
19.60 19.49 19.09 19.74
20.02 19.86 19.57 19.47
19.90 20.16 19.80 20.28
19.84 19.61 20.09 20.01
Expression Level
20.0
19.5
19.0
Dominant Cell Type
a GE & S
a LE
a S
MaxQuantMBR LE Image
20.63 20.05 20.59 21.02
20.67 20.06 20.21 20.97
20.68 20.51 19.82 20.67
20.95 20.44 19.73 20.55
20.49 20.55 20.40 20.09
20.99 20.71 20.54 20.37
Expression Level
21.00
20.75
20.50
20.25
20.00
19.75
Dominant Cell Type
a GE & S
a LE
a S

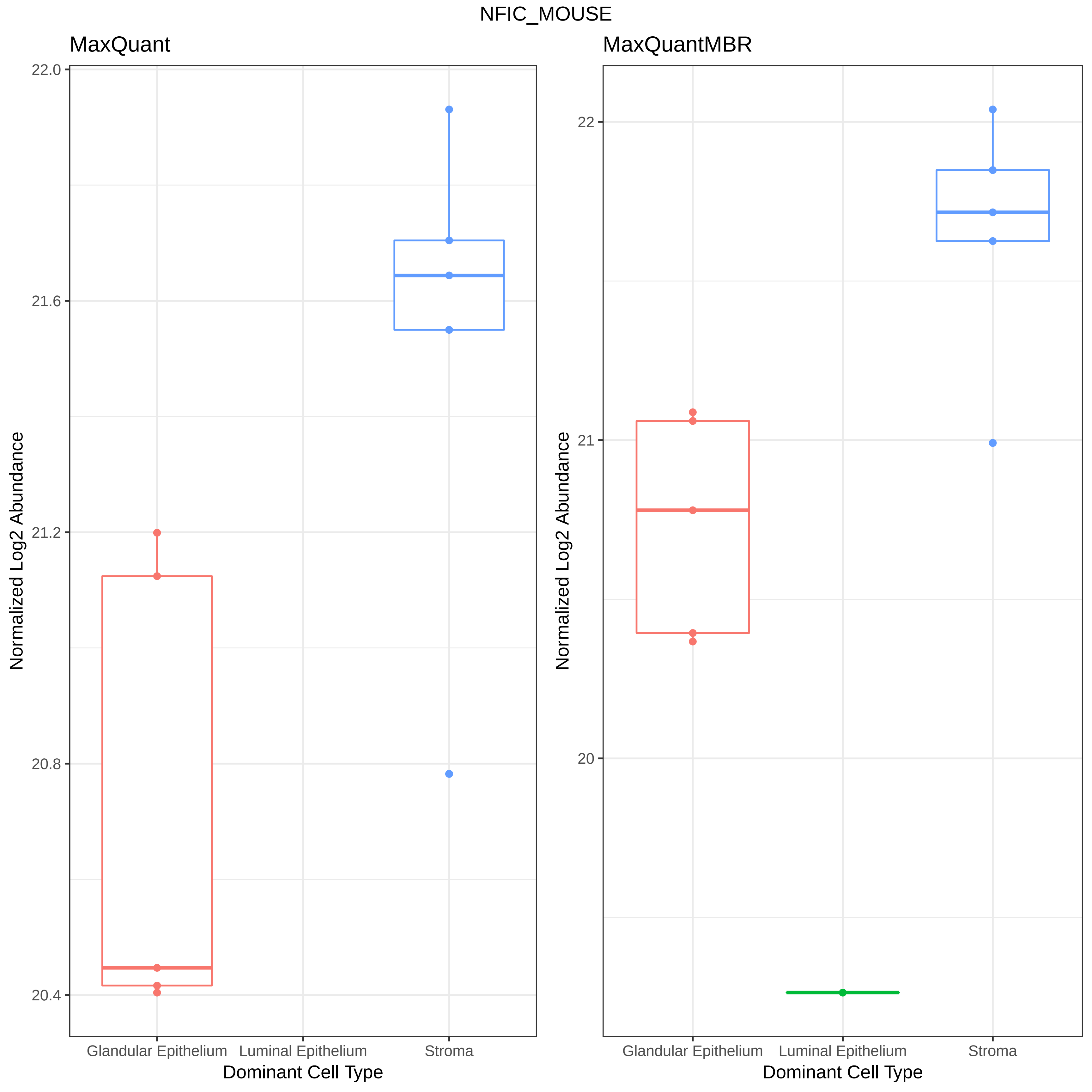
NFIC_MOUSE
MaxQuant
MaxQuantMBR
22.0
21.6
21.2
20.8
20.4
22
21
20
Normalized Log2 Abundance
Glandular Epithelium
Luminal Epithelium
Stroma
Dominant Cell Type

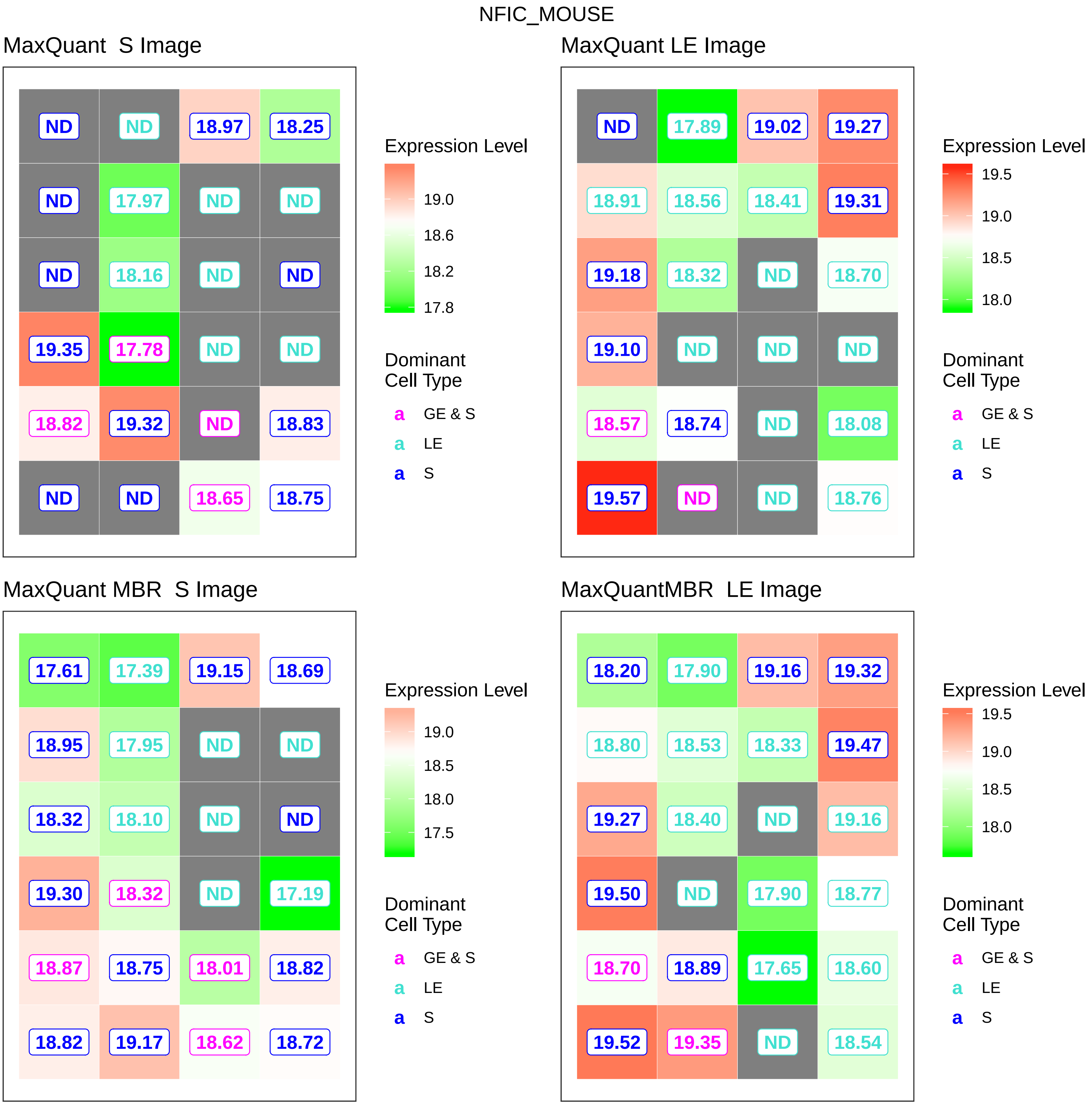

NFIC_MOUSE
MaxQuant S Image
ND ND 18.97 18.25
ND 17.97 ND ND
ND 18.16 ND ND
19.35 17.78 ND ND
18.82 19.32 ND 18.83
ND ND 18.65 18.75
Expression Level
19.0
18.6
18.2
17.8
Dominant Cell Type
a GE & S
a LE
a S
MaxQuant LE Image
ND 17.89 19.02 19.27
18.91 18.56 18.41 19.31
19.18 18.32 ND 18.70
19.10 ND ND ND
18.57 18.74 ND 18.08
19.57 ND ND 18.76
Expression Level
19.5
19.0
18.5
18.0
Dominant Cell Type
a GE & S
a LE
a S
MaxQuant MBR S Image
17.61 17.39 19.15 18.69
18.95 17.95 ND ND
18.32 18.10 ND ND
19.30 18.32 ND 17.19
18.87 18.75 18.01 18.82
18.82 19.17 18.62 18.72
Expression Level
19.0
18.5
18.0
17.5
Dominant Cell Type
a GE & S
a LE
a S
MaxQuantMBR LE Image
18.20 17.90 19.16 19.32
18.80 18.53 18.33 19.47
19.27 18.40 ND 19.16
19.50 ND 17.90 18.77
18.70 18.89 17.65 18.60
19.52 19.35 ND 18.54
Expression Level
19.5
19.0
18.5
18.0
Dominant Cell Type
a GE & S
a LE
a S

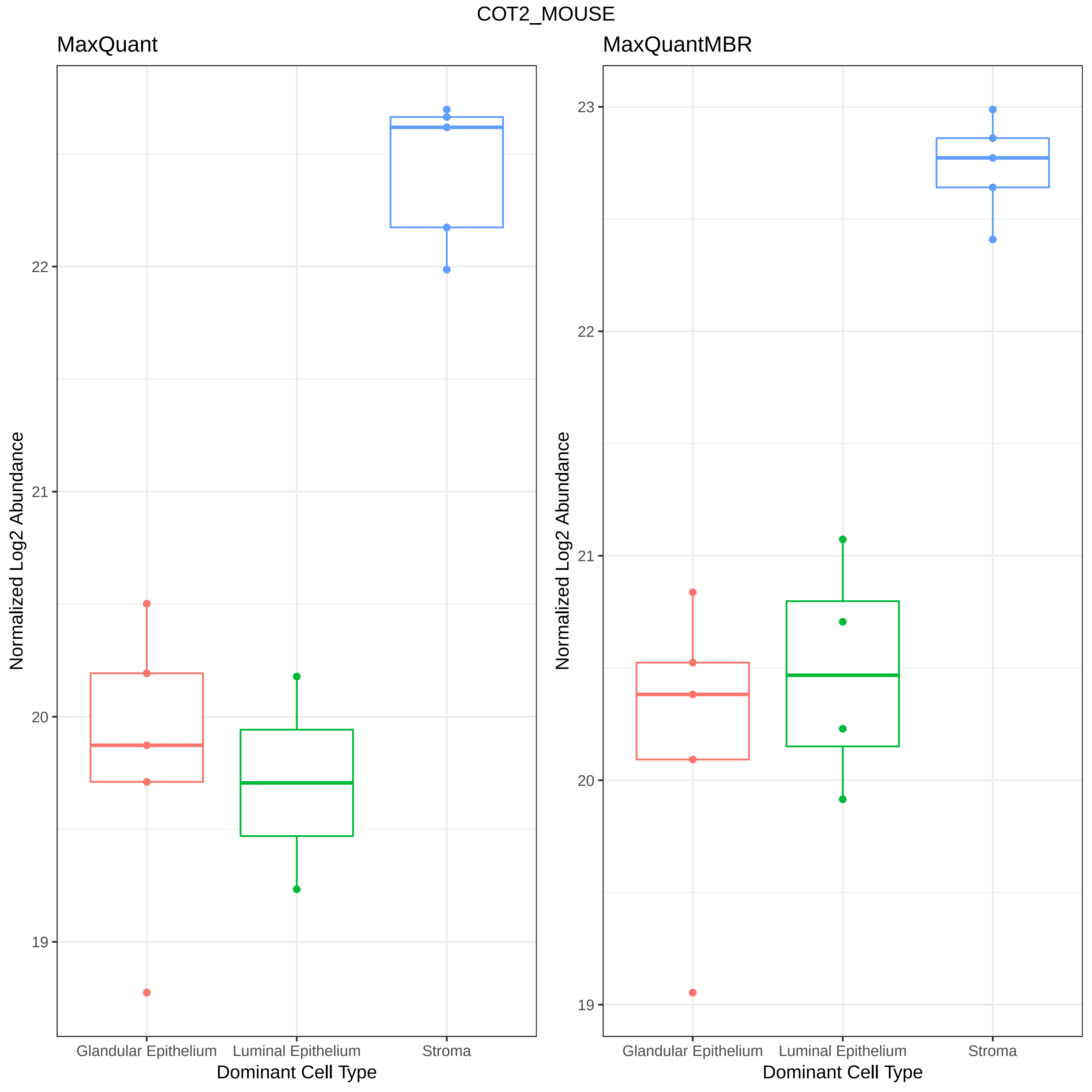
COT2_MOUSE
MaxQuant
MaxQuantMBR
Normalized Log2 Abundance
19
20
21
22
23
Glandular Epithelium
Luminal Epithelium
Stroma
Dominant Cell Type

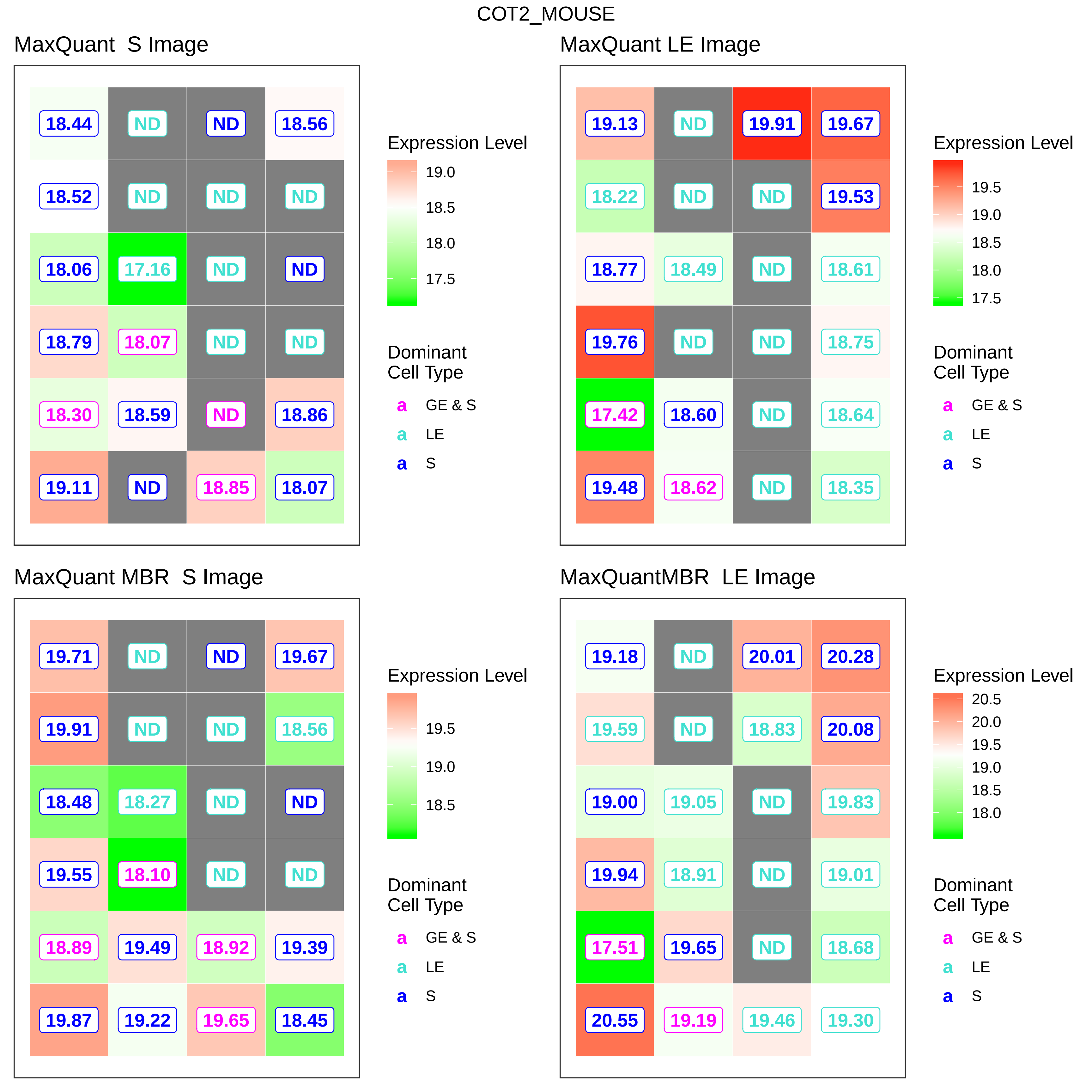
COT2_MOUSE
MaxQuant S Image
18.44 ND ND 18.56
18.52 ND ND ND
18.06 17.16 ND ND
18.79 18.07 ND ND
18.30 18.59 ND 18.86
19.11 ND 18.85 18.07
Expression Level
19.0
18.5
18.0
17.5
Dominant Cell Type
a GE & S
a LE
a S
MaxQuant LE Image
19.13 ND 19.91 19.67
18.22 ND ND 19.53
18.77 18.49 ND 18.61
19.76 ND ND 18.75
17.42 18.60 ND 18.64
19.48 18.62 ND 18.35
Expression Level
19.5
19.0
18.5
18.0
17.5
Dominant Cell Type
a GE & S
a LE
a S
MaxQuant MBR S Image
19.71 ND ND 19.67
19.91 ND ND 18.56
18.48 18.27 ND ND
19.55 18.10 ND ND
18.89 19.49 18.92 19.39
19.87 19.22 19.65 18.45
Expression Level
19.5
19.0
18.5
Dominant Cell Type
a GE & S
a LE
a S
MaxQuantMBR LE Image
19.18 ND 20.01 20.28
19.59 ND 18.83 20.08
19.00 19.05 ND 19.83
19.94 18.91 ND 19.01
17.51 19.65 ND 18.68
20.55 19.19 19.46 19.30
Expression Level
20.5
20.0
19.5
19.0
18.5
18.0
Dominant Cell Type
a GE & S
a LE
a S

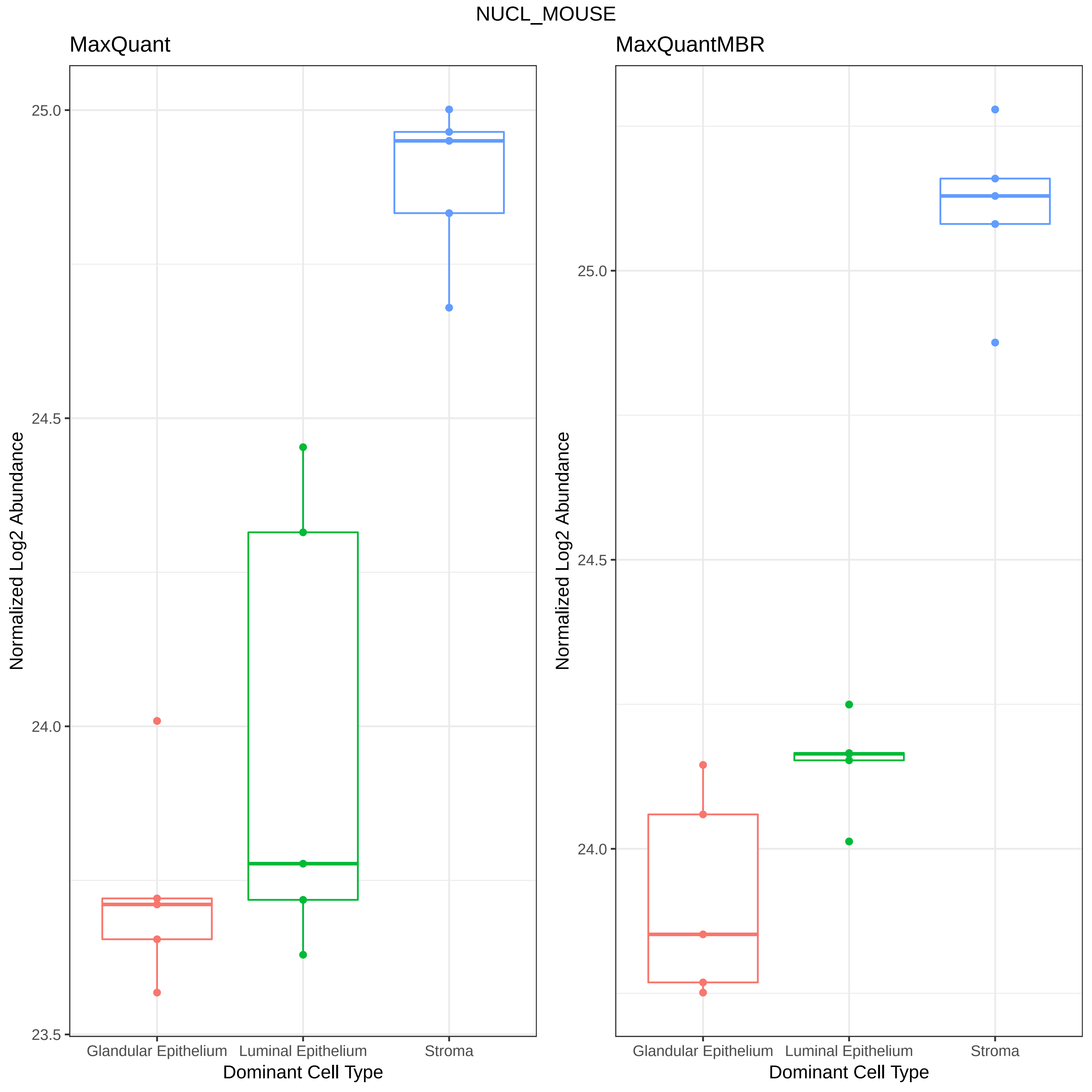
NUCL_MOUSE
MaxQuant
MaxQuantMBR
Normalized Log2 Abundance
25.0
24.5
24.0
23.5
Glandular Epithelium
Luminal Epithelium
Stroma
Dominant Cell Type

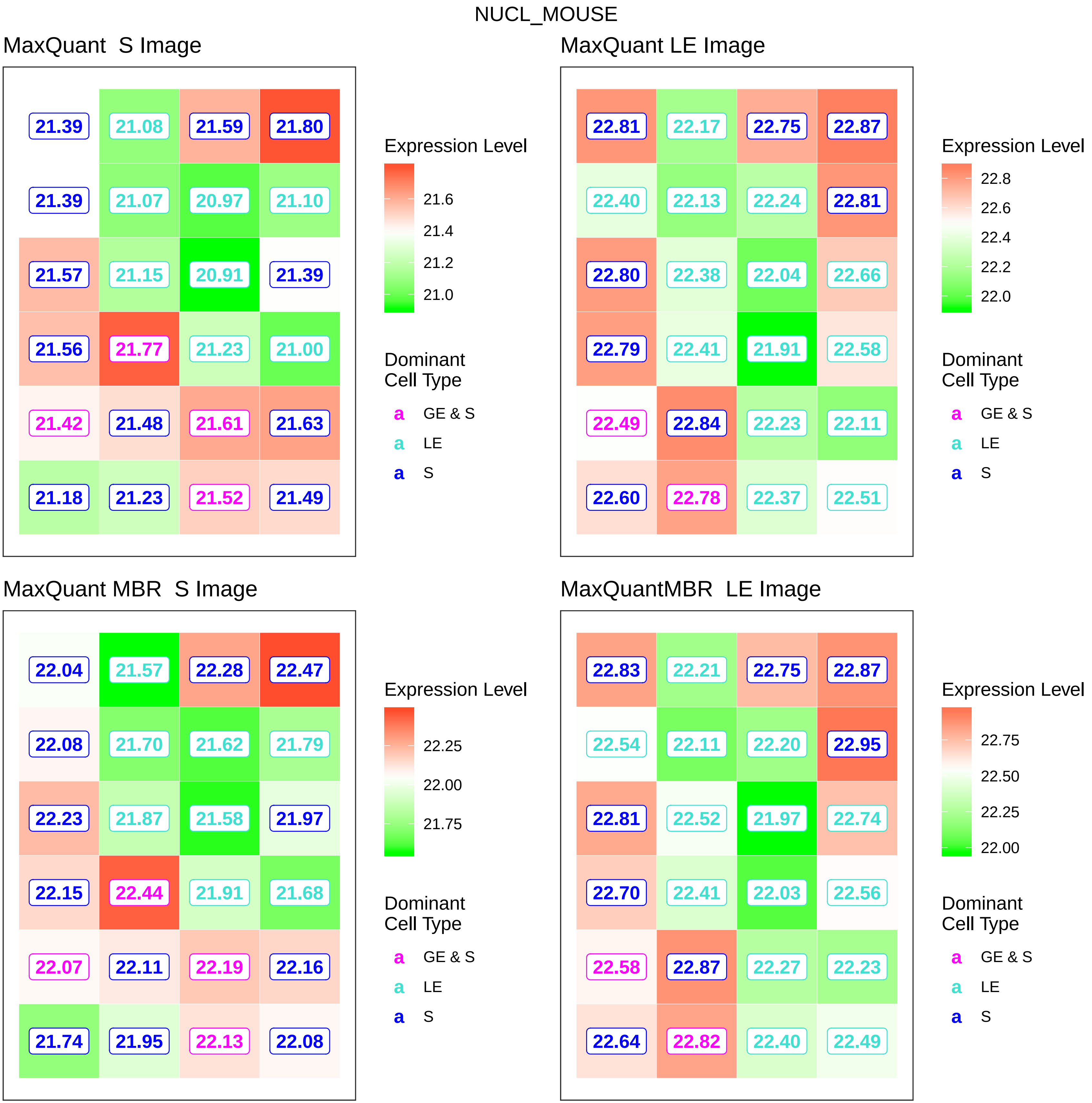
NUCL_MOUSE
MaxQuant S Image
21.39 21.08 21.59 21.80
21.39 21.07 20.97 21.10
21.57 21.15 20.91 21.39
21.56 21.77 21.23 21.00
21.42 21.48 21.61 21.63
21.18 21.23 21.52 21.49
Expression Level
21.6
21.4
21.2
21.0
Dominant Cell Type
a GE & S
a LE
a S
MaxQuant LE Image
22.81 22.17 22.75 22.87
22.40 22.13 22.24 22.81
22.80 22.38 22.04 22.66
22.79 22.41 21.91 22.58
22.49 22.84 22.23 22.11
22.60 22.78 22.37 22.51
Expression Level
22.8
22.6
22.4
22.2
22.0
Dominant Cell Type
a GE & S
a LE
a S
MaxQuant MBR S Image
22.04 21.57 22.28 22.47
22.08 21.70 21.62 21.79
22.23 21.87 21.58 21.97
22.15 22.44 21.91 21.68
22.07 22.11 22.19 22.16
21.74 21.95 22.13 22.08
Expression Level
22.25
22.00
21.75
Dominant Cell Type
a GE & S
a LE
a S
MaxQuantMBR LE Image
22.83 22.21 22.75 22.87
22.54 22.11 22.20 22.95
22.81 22.52 21.97 22.74
22.70 22.41 22.03 22.56
22.58 22.87 22.27 22.23
22.64 22.82 22.40 22.49
Expression Level
22.75
22.50
22.25
22.00
Dominant Cell Type
a GE & S
a LE
a S

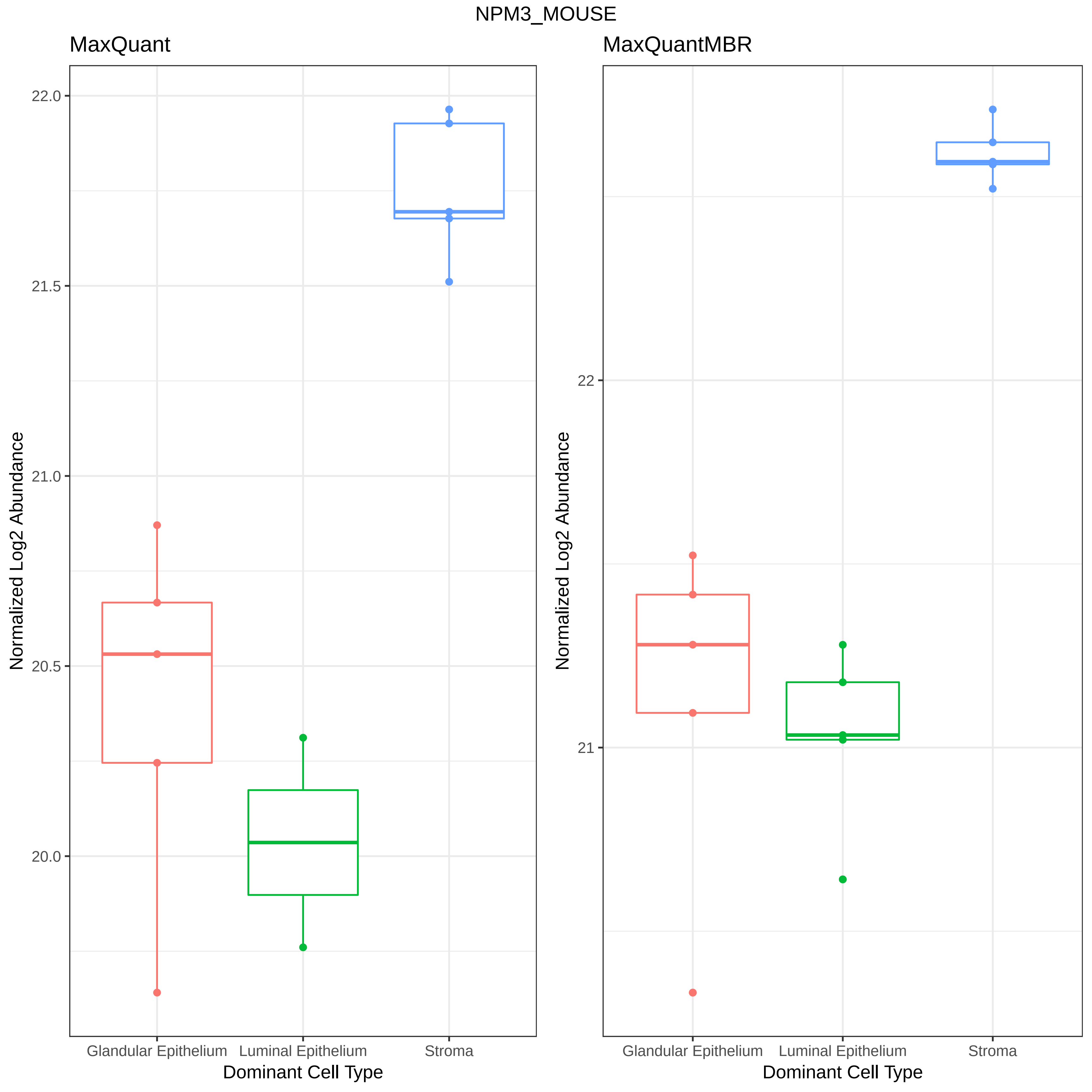

NPM3_MOUSE
MaxQuant
MaxQuantMBR
22.0
21.5
21.0
20.5
20.0
22
21
Normalized Log2 Abundance
Normalized Log2 Abundance
Glandular Epithelium
Luminal Epithelium
Stroma
Glandular Epithelium
Luminal Epithelium
Stroma
Dominant Cell Type
Dominant Cell Type

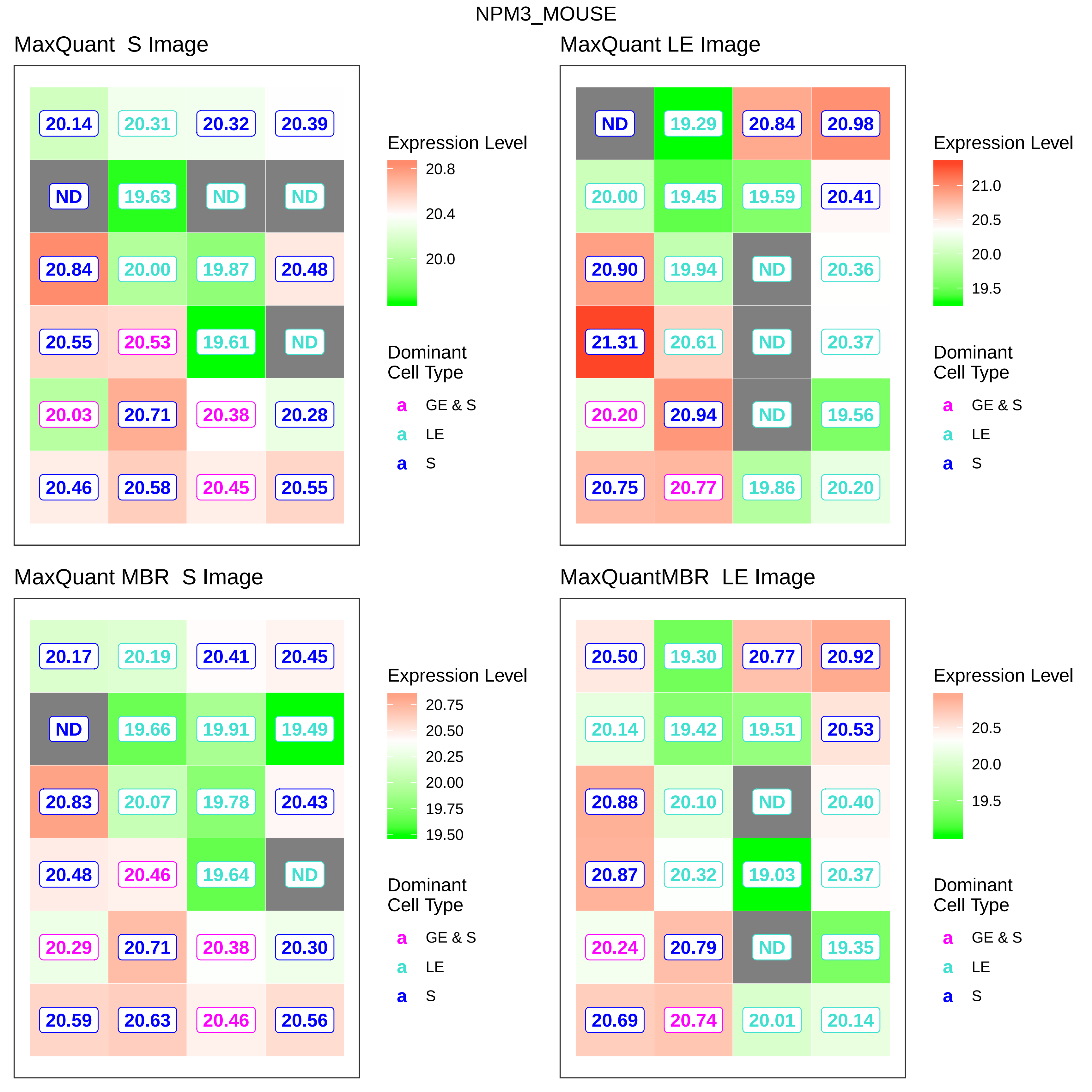

NPM3_MOUSE
MaxQuant S Image
20.14 20.31 20.32 20.39
ND 19.63 ND ND
20.84 20.00 19.87 20.48
20.55 20.53 19.61 ND
20.03 20.71 20.38 20.28
20.46 20.58 20.45 20.55
Expression Level
20.8
20.4
20.0
Dominant Cell Type
a GE & S
a LE
a S
MaxQuant LE Image
ND 19.29 20.84 20.98
20.00 19.45 19.59 20.41
20.90 19.94 ND 20.36
21.31 20.61 ND 20.37
20.20 20.94 ND 19.56
20.75 20.77 19.86 20.20
Expression Level
21.0
20.5
20.0
19.5
Dominant Cell Type
a GE & S
a LE
a S
MaxQuant MBR S Image
20.17 20.19 20.41 20.45
ND 19.66 19.91 19.49
20.83 20.07 19.78 20.43
20.48 20.46 19.64 ND
20.29 20.71 20.38 20.30
20.59 20.63 20.46 20.56
Expression Level
20.75
20.50
20.25
20.00
19.75
19.50
Dominant Cell Type
a GE & S
a LE
a S
MaxQuantMBR LE Image
20.50 19.30 20.77 20.92
20.14 19.42 19.51 20.53
20.88 20.10 ND 20.40
20.87 20.32 19.03 20.37
20.24 20.79 ND 19.35
20.69 20.74 20.01 20.14
Expression Level
20.5
20.0
19.5
Dominant Cell Type
a GE & S
a LE
a S

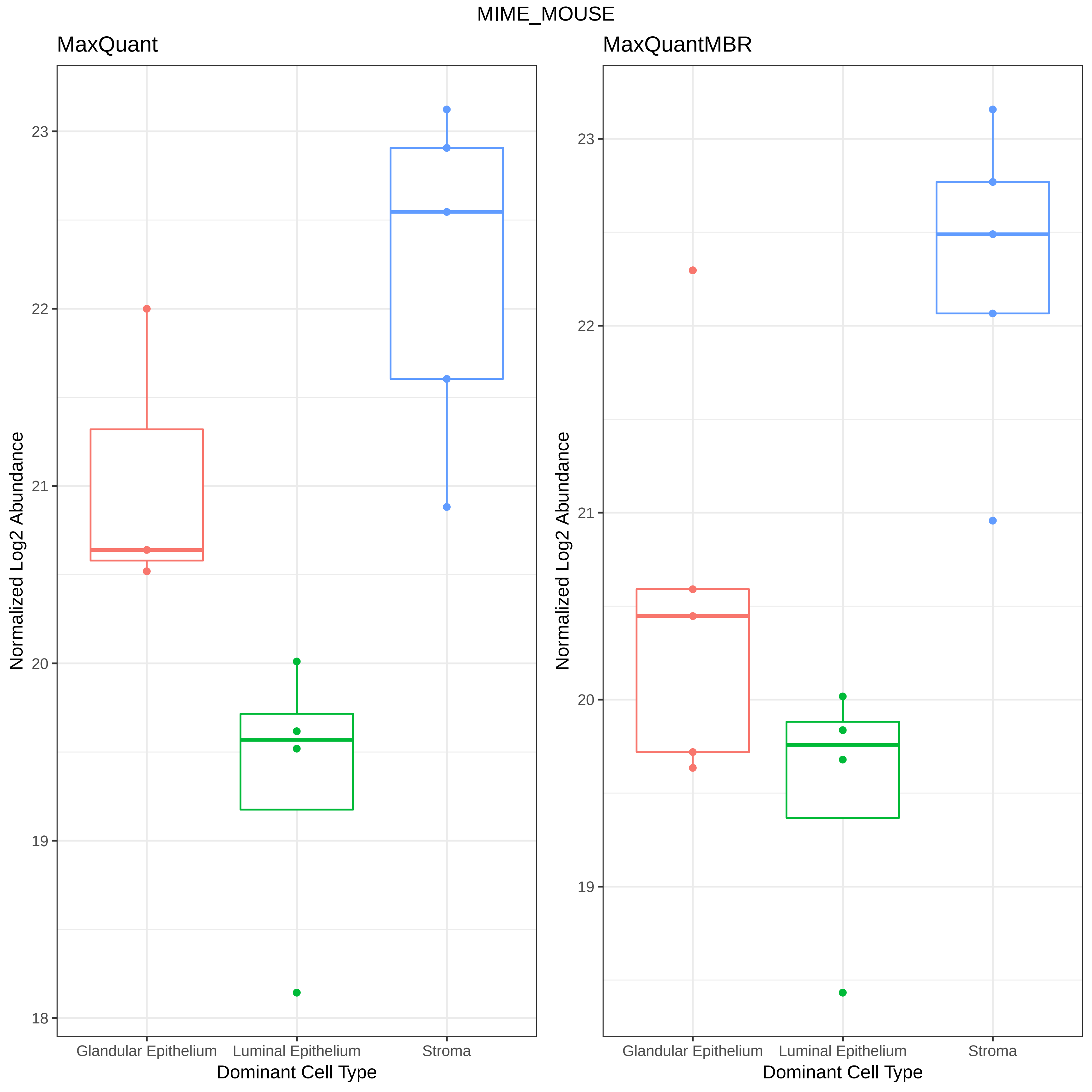
MIME_MOUSE
MaxQuant
MaxQuantMBR
Normalized Log2 Abundance
23
22
21
20
19
18
Glandular Epithelium
Luminal Epithelium
Stroma
Dominant Cell Type

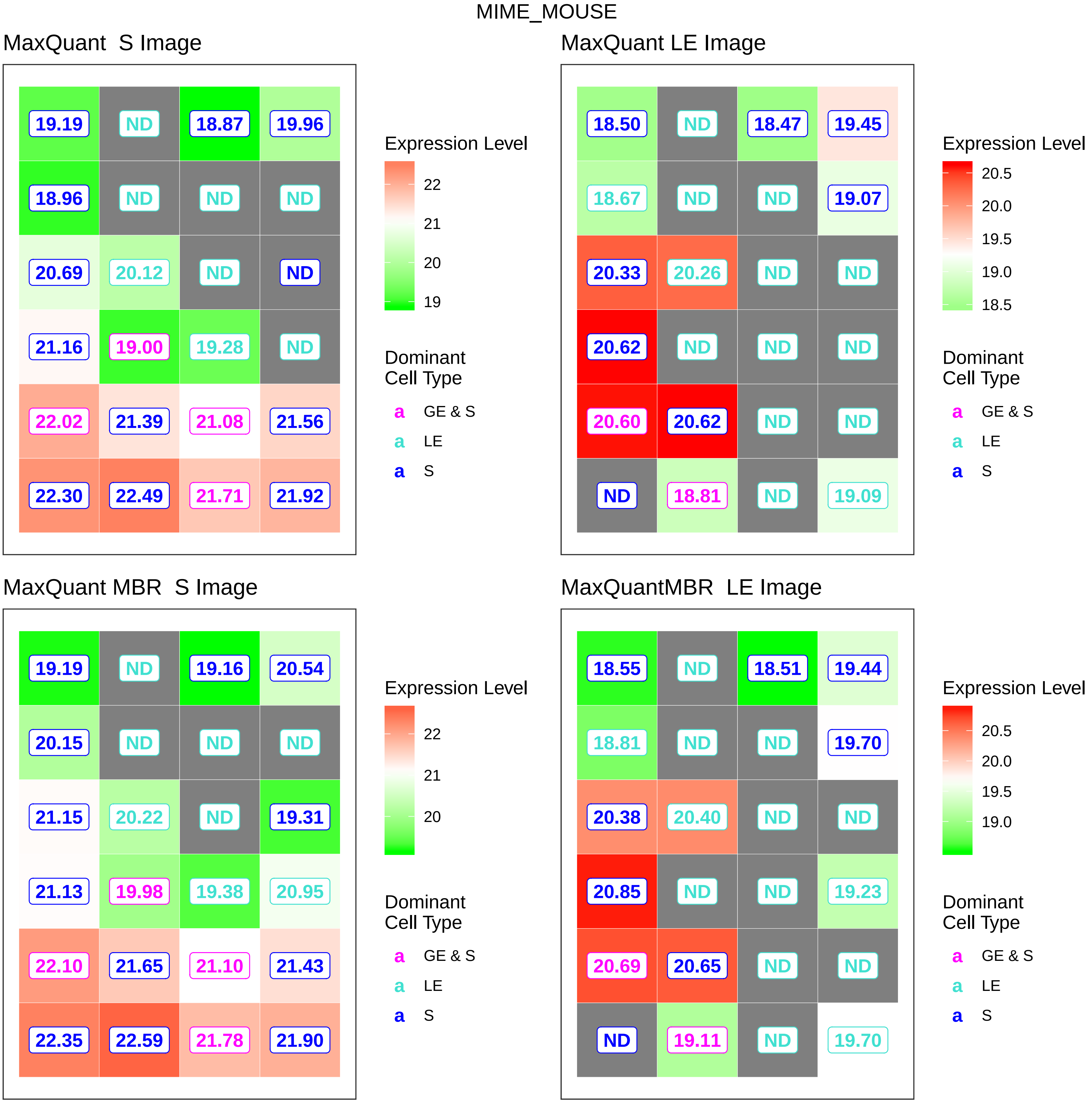
MIME_MOUSE
MaxQuant S Image
19.19 ND 18.87 19.96
18.96 ND ND ND
20.69 20.12 ND ND
21.16 19.00 19.28 ND
22.02 21.39 21.08 21.56
22.30 22.49 21.71 21.92
Expression Level
22
21
20
19
Dominant Cell Type
a GE & S
a LE
a S
MaxQuant LE Image
18.50 ND 18.47 19.45
18.67 ND ND 19.07
20.33 20.26 ND ND
20.62 ND ND ND
20.60 20.62 ND ND
ND 18.81 ND 19.09
Expression Level
20.5
20.0
19.5
19.0
18.5
Dominant Cell Type
a GE & S
a LE
a S
MaxQuant MBR S Image
19.19 ND 19.16 20.54
20.15 ND ND ND
21.15 20.22 ND 19.31
21.13 19.98 19.38 20.95
22.10 21.65 21.10 21.43
22.35 22.59 21.78 21.90
Expression Level
22
21
20
Dominant Cell Type
a GE & S
a LE
a S
MaxQuantMBR LE Image
18.55 ND 18.51 19.44
18.81 ND ND 19.70
20.38 20.40 ND ND
20.85 ND ND 19.23
20.69 20.65 ND ND
ND 19.11 ND 19.70
Expression Level
20.5
20.0
19.5
19.0
Dominant Cell Type
a GE & S
a LE
a S

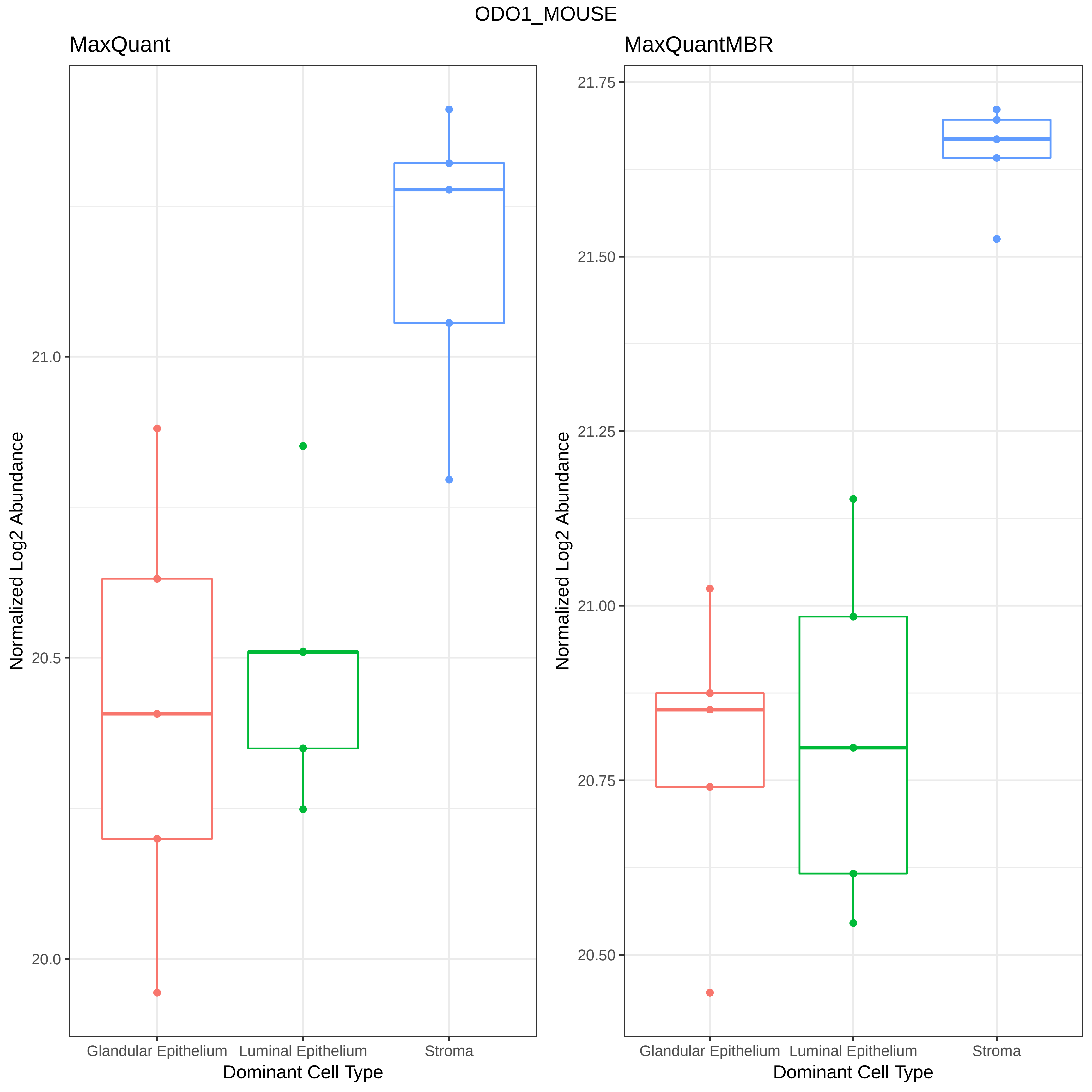

ODO1_MOUSE
MaxQuant
MaxQuantMBR
Normalized Log2 Abundance
21.0
20.5
20.0
21.75
21.50
21.25
21.00
20.75
20.50
Glandular Epithelium
Luminal Epithelium
Stroma
Dominant Cell Type

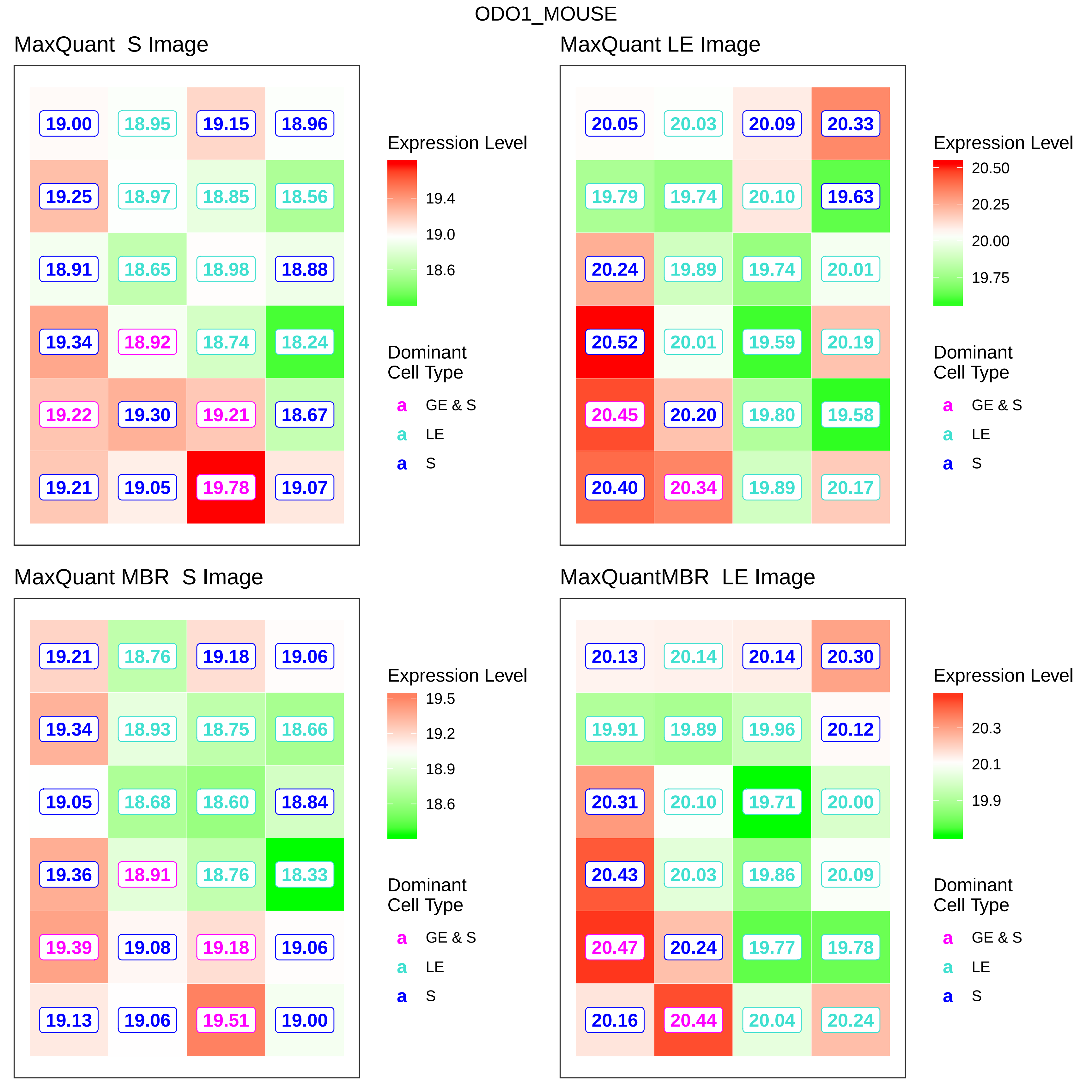
ODO1_MOUSE
MaxQuant S Image
19.00 18.95 19.15 18.96
19.25 18.97 18.85 18.56
18.91 18.65 18.98 18.88
19.34 18.92 18.74 18.24
19.22 19.30 19.21 18.67
19.21 19.05 19.78 19.07
Expression Level
19.4
19.0
18.6
Dominant Cell Type
a GE & S
a LE
a S
MaxQuant LE Image
20.05 20.03 20.09 20.33
19.79 19.74 20.10 19.63
20.24 19.89 19.74 20.01
20.52 20.01 19.59 20.19
20.45 20.20 19.80 19.58
20.40 20.34 19.89 20.17
Expression Level
20.50
20.25
20.00
19.75
Dominant Cell Type
a GE & S
a LE
a S
MaxQuant MBR S Image
19.21 18.76 19.18 19.06
19.34 18.93 18.75 18.66
19.05 18.68 18.60 18.84
19.36 18.91 18.76 18.33
19.39 19.08 19.18 19.06
19.13 19.06 19.51 19.00
Expression Level
19.5
19.2
18.9
18.6
Dominant Cell Type
a GE & S
a LE
a S
MaxQuantMBR LE Image
20.13 20.14 20.14 20.30
19.91 19.89 19.96 20.12
20.31 20.10 19.71 20.00
20.43 20.03 19.86 20.09
20.47 20.24 19.77 19.78
20.16 20.44 20.04 20.24
Expression Level
20.3
20.1
19.9
Dominant Cell Type
a GE & S
a LE
a S

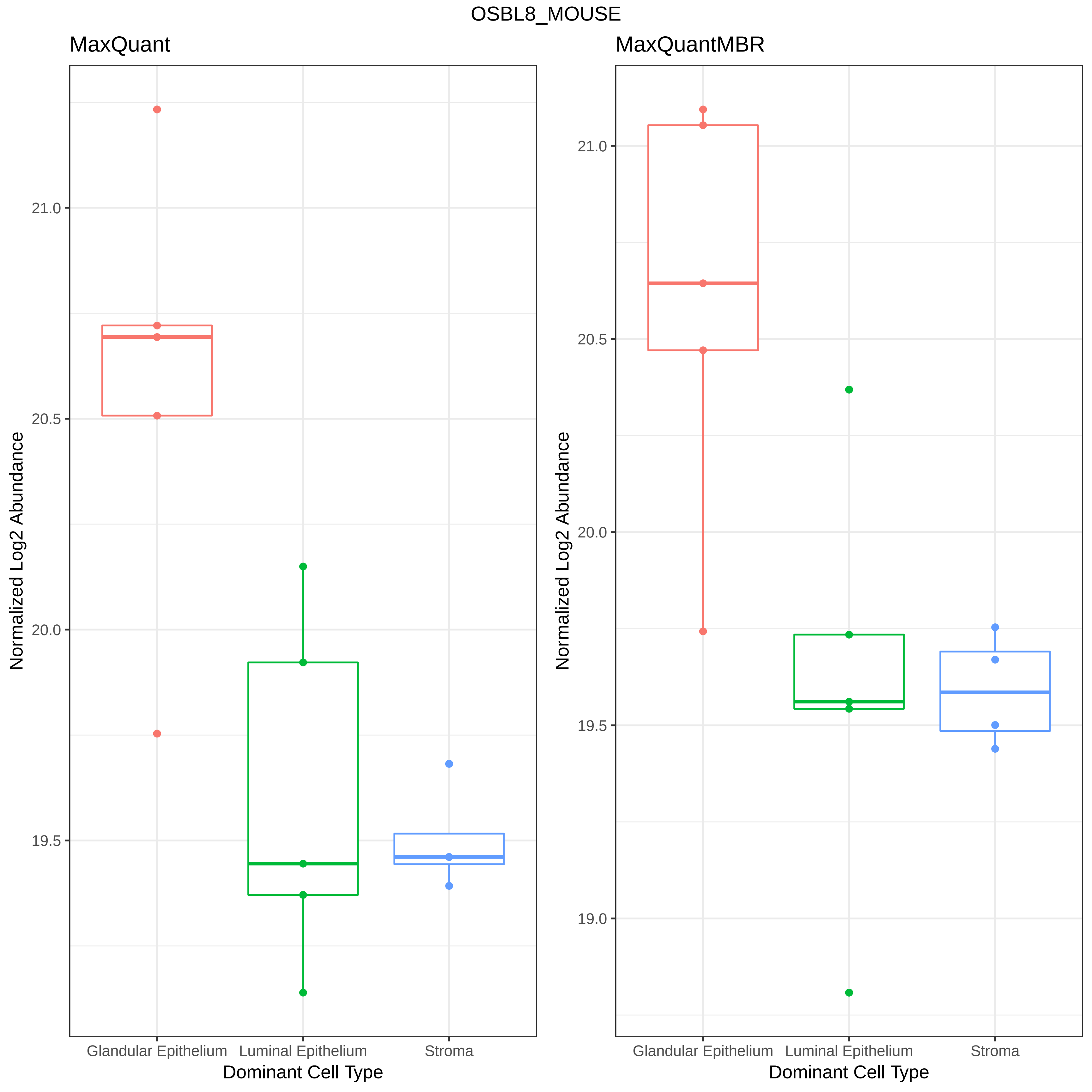
OSBL8_MOUSE
MaxQuant
MaxQuantMBR
Normalized Log2 Abundance
21.0
20.5
20.0
19.5
19.0
Glandular Epithelium
Luminal Epithelium
Stroma
Dominant Cell Type

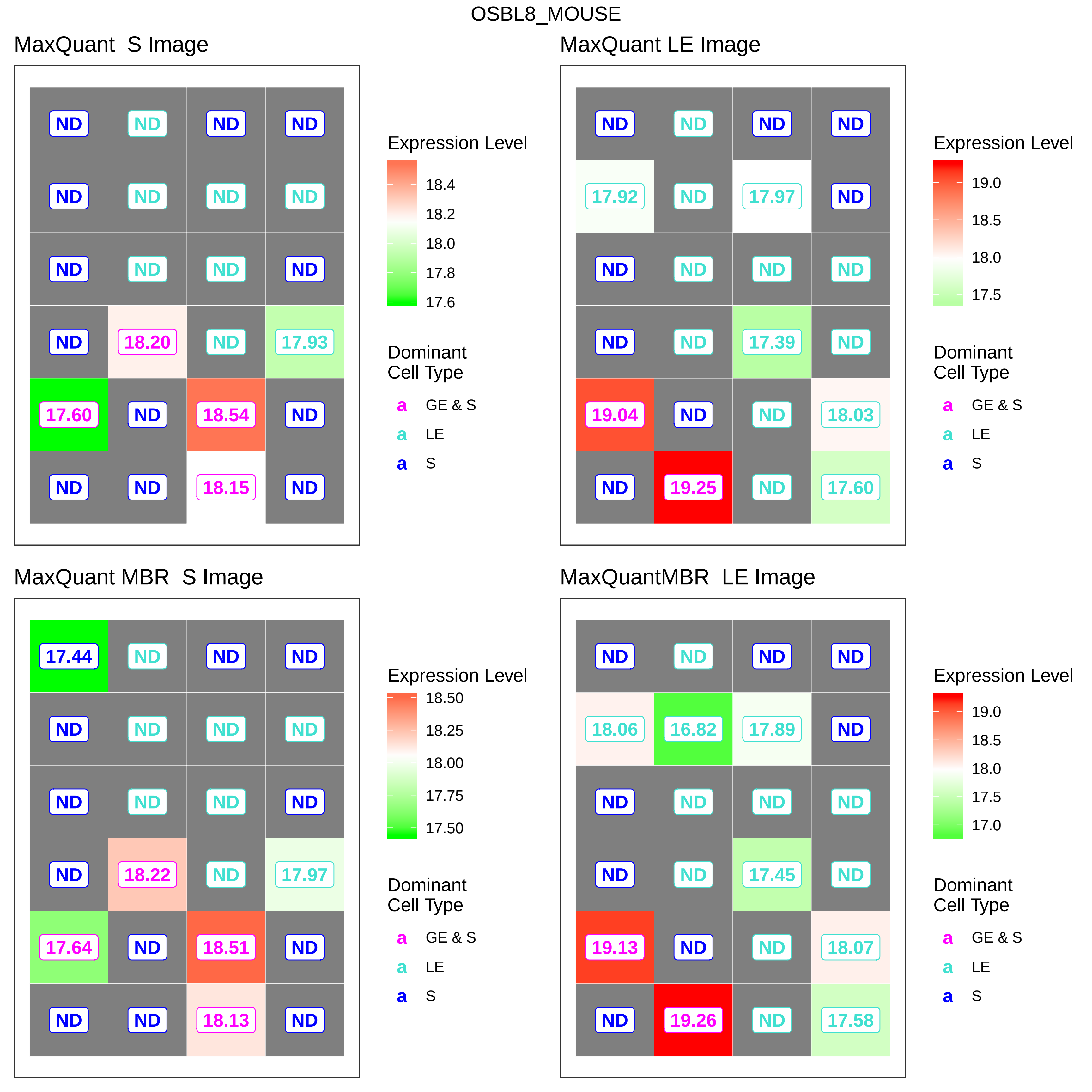
OSBL8_MOUSE
MaxQuant S Image
ND ND ND ND
ND ND ND ND
ND ND ND ND
ND 18.20 ND 17.93
17.60 ND 18.54 ND
ND ND 18.15 ND
Expression Level
18.4
18.2
18.0
17.8
17.6
Dominant Cell Type
a GE & S
a LE
a S
MaxQuant LE Image
ND ND ND ND
17.92 ND 17.97 ND
ND ND ND ND
ND ND 17.39 ND
19.04 ND ND 18.03
ND 19.25 ND 17.60
Expression Level
19.0
18.5
18.0
17.5
Dominant Cell Type
a GE & S
a LE
a S
MaxQuant MBR S Image
17.44 ND ND ND
ND ND ND ND
ND ND ND ND
ND 18.22 ND 17.97
17.64 ND 18.51 ND
ND ND 18.13 ND
Expression Level
18.50
18.25
18.00
17.75
17.50
Dominant Cell Type
a GE & S
a LE
a S
MaxQuantMBR LE Image
ND ND ND ND
18.06 16.82 17.89 ND
ND ND ND ND
ND ND 17.45 ND
19.13 ND ND 18.07
ND 19.26 ND 17.58
Expression Level
19.0
18.5
18.0
17.5
17.0
Dominant Cell Type
a GE & S
a LE
a S

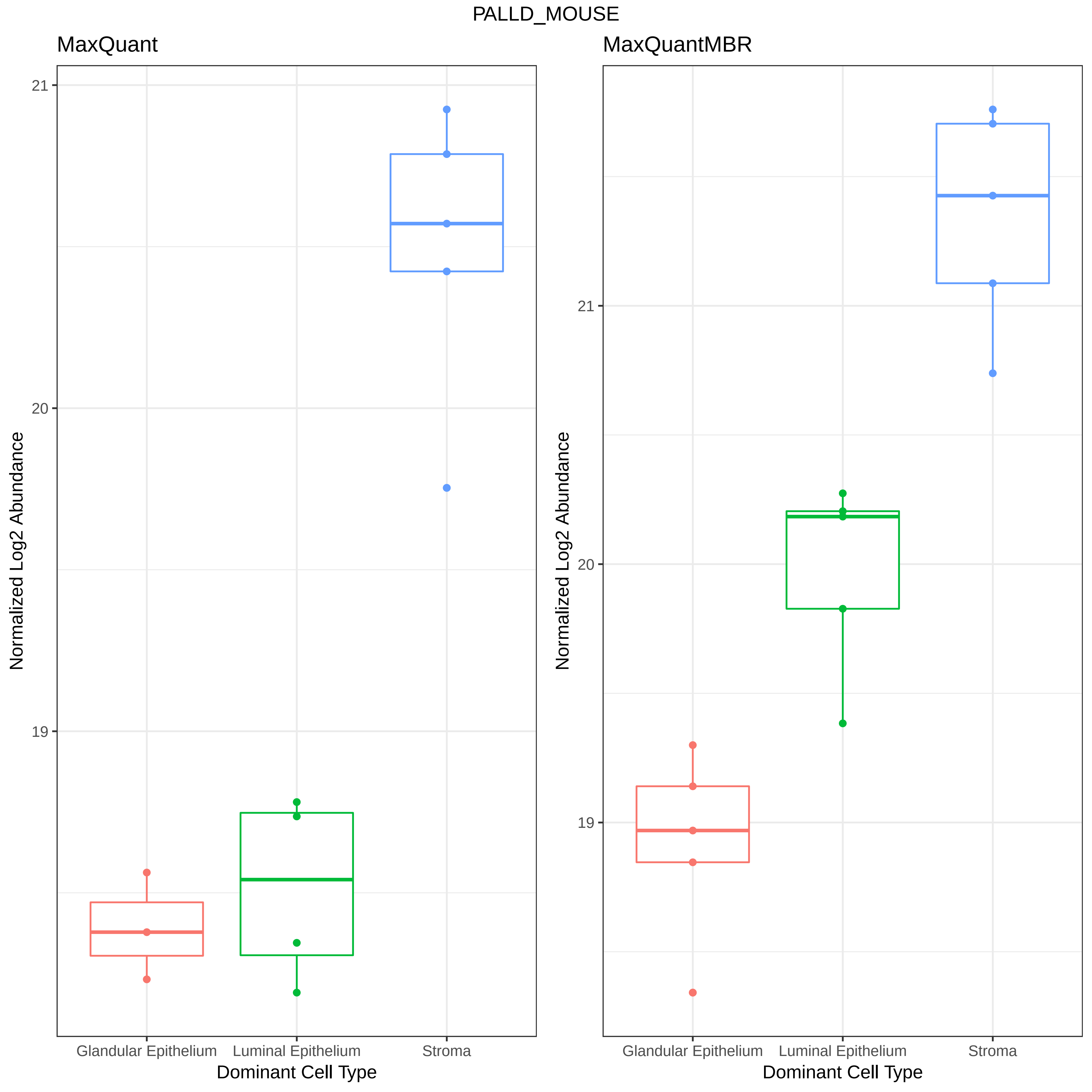

PALLD_MOUSE
MaxQuant
MaxQuantMBR
21
20
19
Normalized Log2 Abundance
Glandular Epithelium
Luminal Epithelium
Stroma
Dominant Cell Type

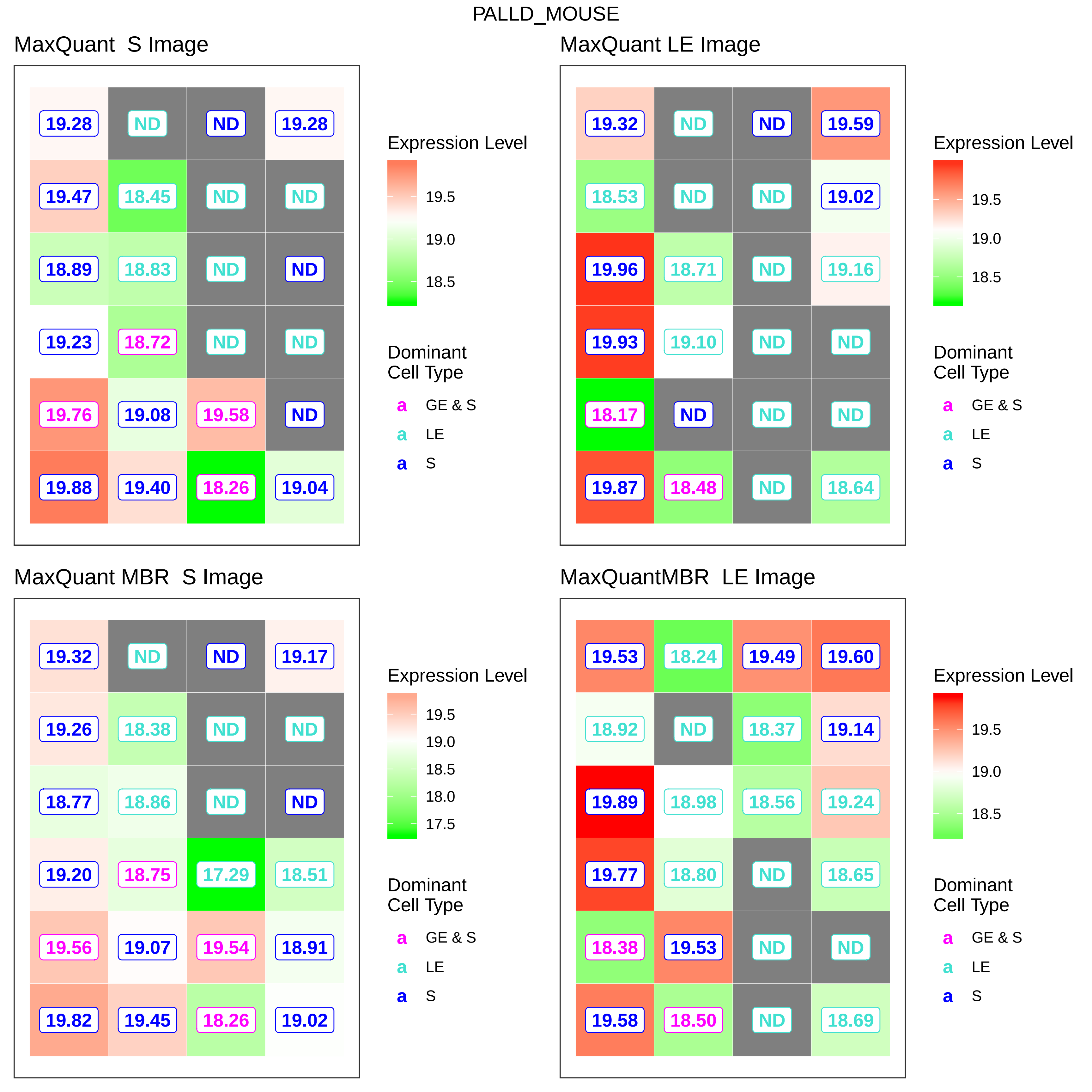
PALLD_MOUSE
MaxQuant S Image
19.28 ND ND 19.28
19.47 18.45 ND ND
18.89 18.83 ND ND
19.23 18.72 ND ND
19.76 19.08 19.58 ND
19.88 19.40 18.26 19.04
Expression Level
19.5
19.0
18.5
Dominant Cell Type
a GE & S
a LE
a S
MaxQuant LE Image
19.32 ND ND 19.59
18.53 ND ND 19.02
19.96 18.71 ND 19.16
19.93 19.10 ND ND
18.17 ND ND ND
19.87 18.48 ND 18.64
Expression Level
19.5
19.0
18.5
Dominant Cell Type
a GE & S
a LE
a S
MaxQuant MBR S Image
19.32 ND ND 19.17
19.26 18.38 ND ND
18.77 18.86 ND ND
19.20 18.75 17.29 18.51
19.56 19.07 19.54 18.91
19.82 19.45 18.26 19.02
Expression Level
19.5
19.0
18.5
18.0
17.5
Dominant Cell Type
a GE & S
a LE
a S
MaxQuantMBR LE Image
19.53 18.24 19.49 19.60
18.92 ND 18.37 19.14
19.89 18.98 18.56 19.24
19.77 18.80 ND 18.65
18.38 19.53 ND ND
19.58 18.50 ND 18.69
Expression Level
19.5
19.0
18.5
Dominant Cell Type
a GE & S
a LE
a S

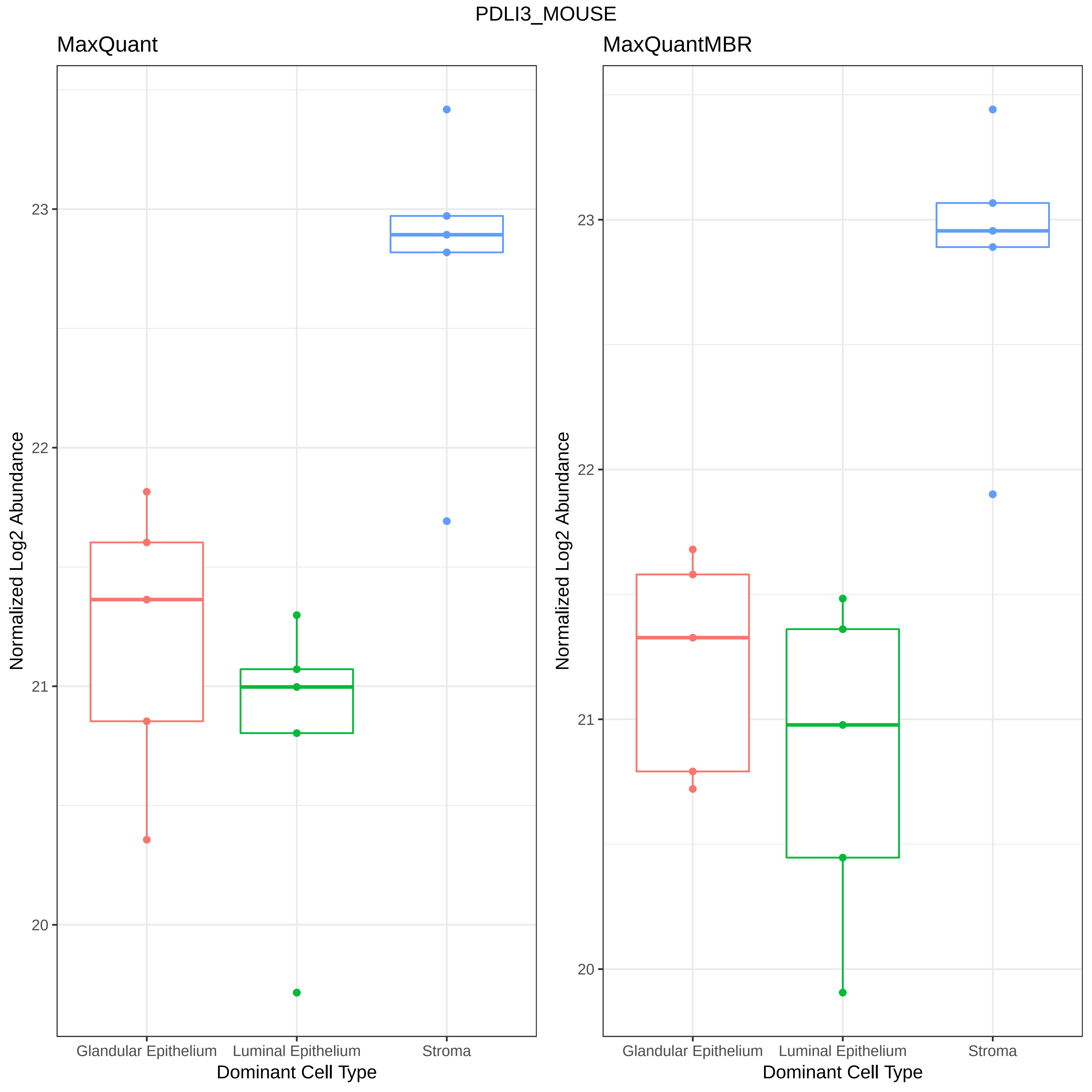

PDLI3_MOUSE
MaxQuant
MaxQuantMBR
Normalized Log2 Abundance
23
22
21
20
Glandular Epithelium
Luminal Epithelium
Stroma
Dominant Cell Type

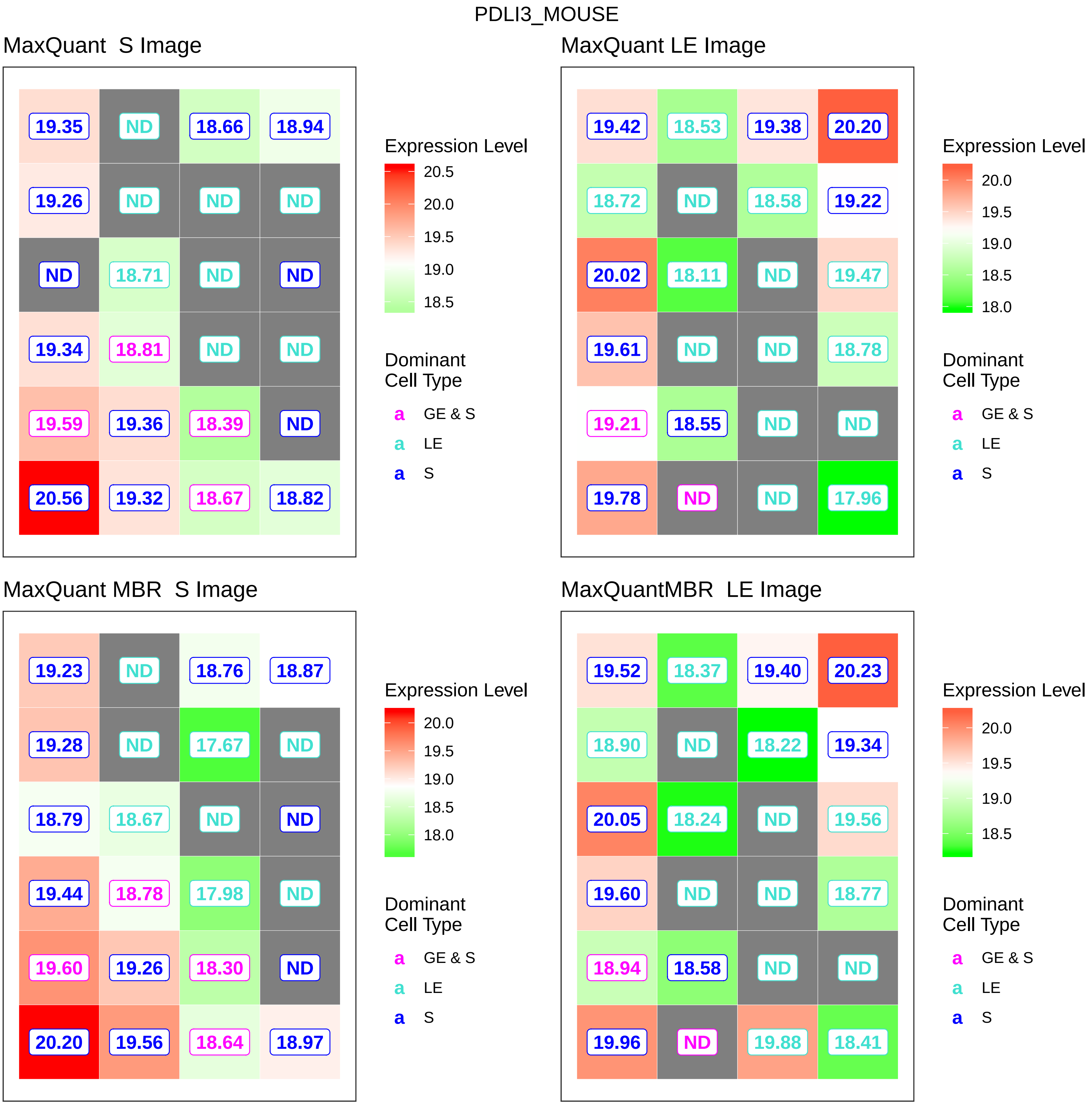
PDLI3_MOUSE
MaxQuant S Image
19.35 ND 18.66 18.94
19.26 ND ND ND
ND 18.71 ND ND
19.34 18.81 ND ND
19.59 19.36 18.39 ND
20.56 19.32 18.67 18.82
Expression Level
20.5
20.0
19.5
19.0
18.5
Dominant Cell Type
a GE & S
a LE
a S
MaxQuant LE Image
19.42 18.53 19.38 20.20
18.72 ND 18.58 19.22
20.02 18.11 ND 19.47
19.61 ND ND 18.78
19.21 18.55 ND ND
19.78 ND ND 17.96
Expression Level
20.0
19.5
19.0
18.5
18.0
Dominant Cell Type
a GE & S
a LE
a S
MaxQuant MBR S Image
19.23 ND 18.76 18.87
19.28 ND 17.67 ND
18.79 18.67 ND ND
19.44 18.78 17.98 ND
19.60 19.26 18.30 ND
20.20 19.56 18.64 18.97
Expression Level
20.0
19.5
19.0
18.5
18.0
Dominant Cell Type
a GE & S
a LE
a S
MaxQuantMBR LE Image
19.52 18.37 19.40 20.23
18.90 ND 18.22 19.34
20.05 18.24 ND 19.56
19.60 ND ND 18.77
18.94 18.58 ND ND
19.96 ND 19.88 18.41
Expression Level
20.0
19.5
19.0
18.5
Dominant Cell Type
a GE & S
a LE
a S

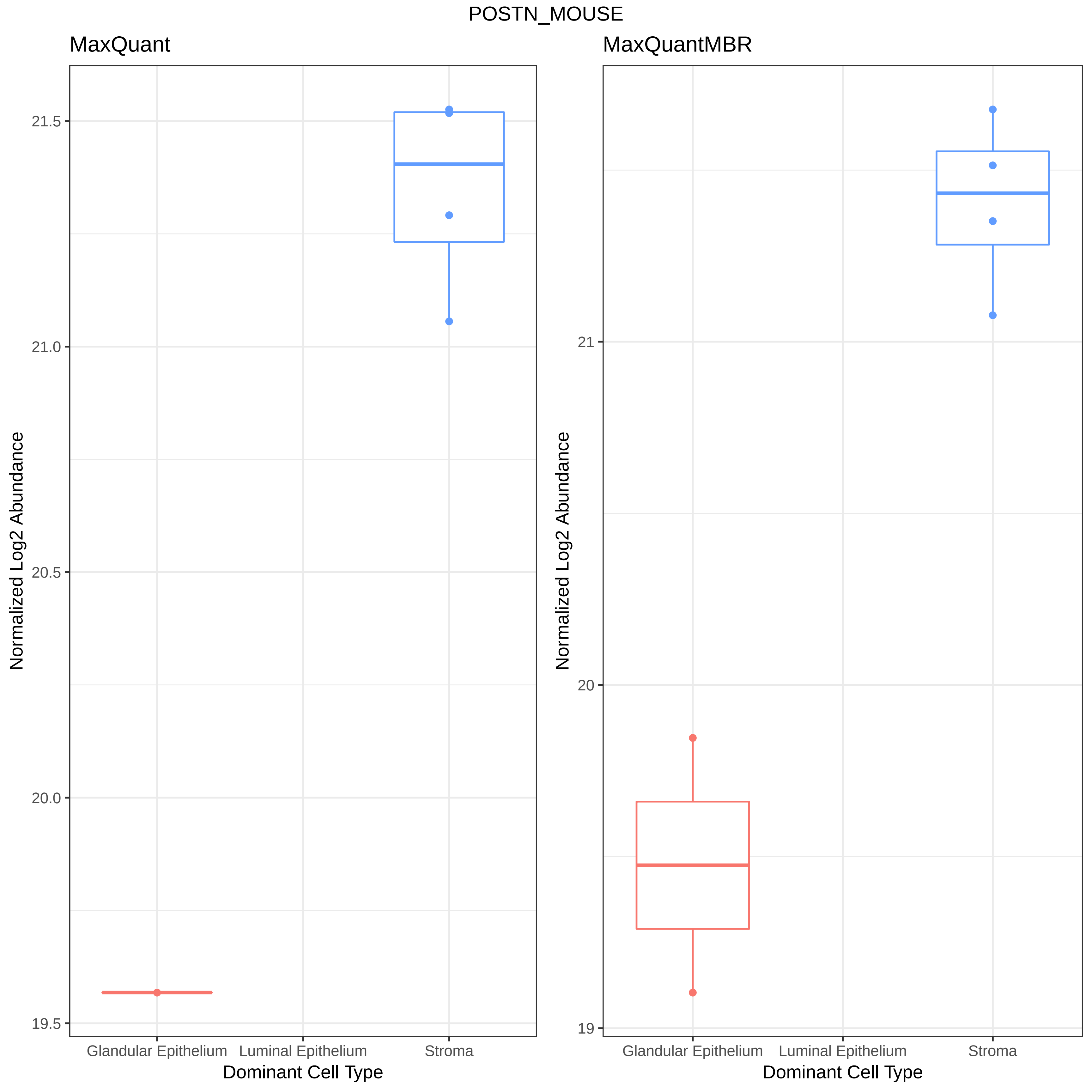

POSTN_MOUSE
MaxQuant
MaxQuantMBR
Normalized Log2 Abundance
21.5
21.0
20.5
20.0
19.5
21
20
19
Glandular Epithelium
Luminal Epithelium
Stroma
Dominant Cell Type

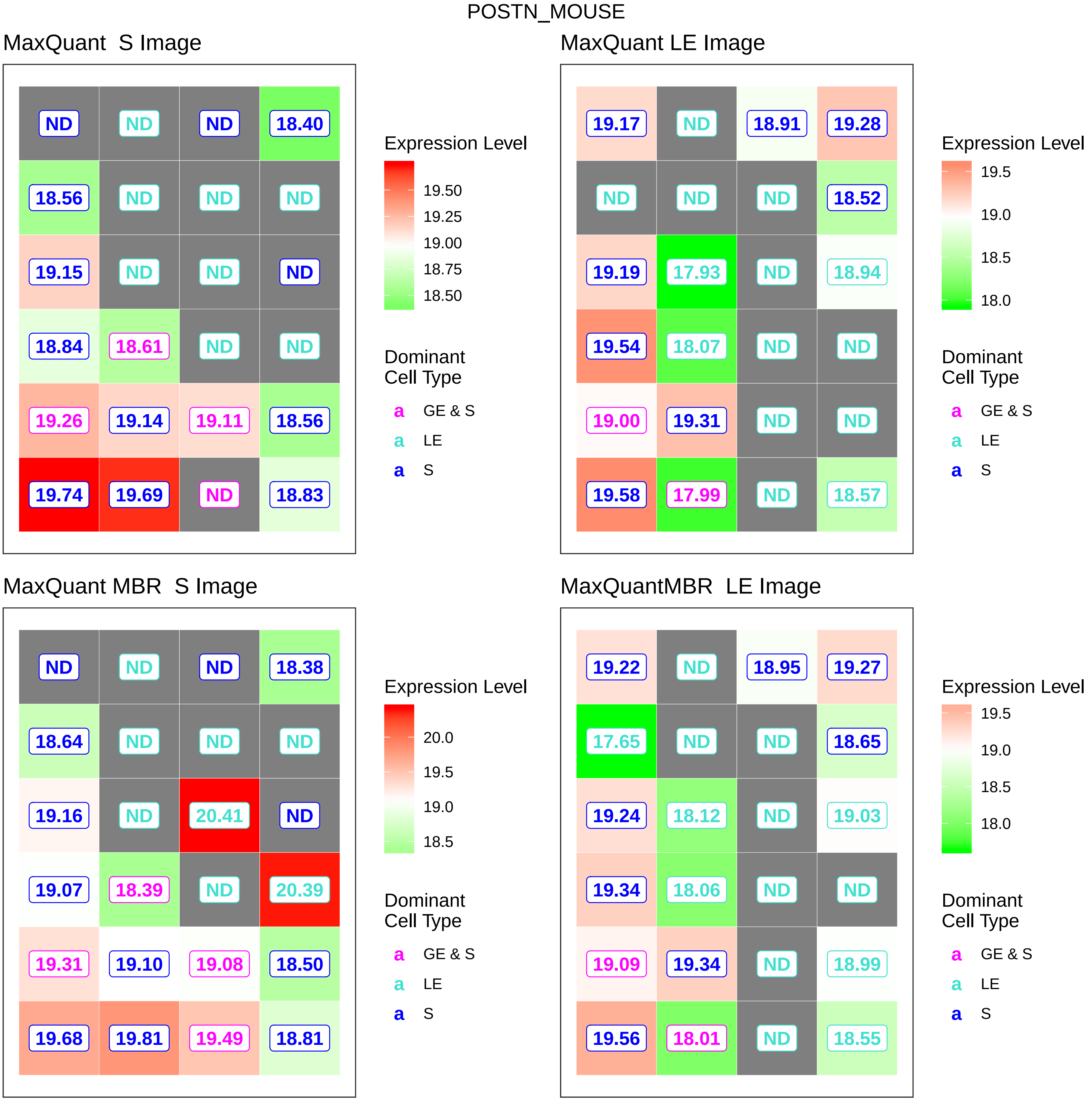
POSTN_MOUSE
MaxQuant S Image
ND ND ND 18.40
18.56 ND ND ND
19.15 ND ND ND
18.84 18.61 ND ND
19.26 19.14 19.11 18.56
19.74 19.69 ND 18.83
Expression Level
19.50
19.25
19.00
18.75
18.50
Dominant Cell Type
a GE & S
a LE
a S
MaxQuant LE Image
19.17 ND 18.91 19.28
ND ND ND 18.52
19.19 17.93 ND 18.94
19.54 18.07 ND ND
19.00 19.31 ND ND
19.58 17.99 ND 18.57
Expression Level
19.5
19.0
18.5
18.0
Dominant Cell Type
a GE & S
a LE
a S
MaxQuant MBR S Image
ND ND ND 18.38
18.64 ND ND ND
19.16 ND 20.41 ND
19.07 18.39 ND 20.39
19.31 19.10 19.08 18.50
19.68 19.81 19.49 18.81
Expression Level
20.0
19.5
19.0
18.5
Dominant Cell Type
a GE & S
a LE
a S
MaxQuantMBR LE Image
19.22 ND 18.95 19.27
17.65 ND ND 18.65
19.24 18.12 ND 19.03
19.34 18.06 ND ND
19.09 19.34 ND 18.99
19.56 18.01 ND 18.55
Expression Level
19.5
19.0
18.5
18.0
Dominant Cell Type
a GE & S
a LE
a S

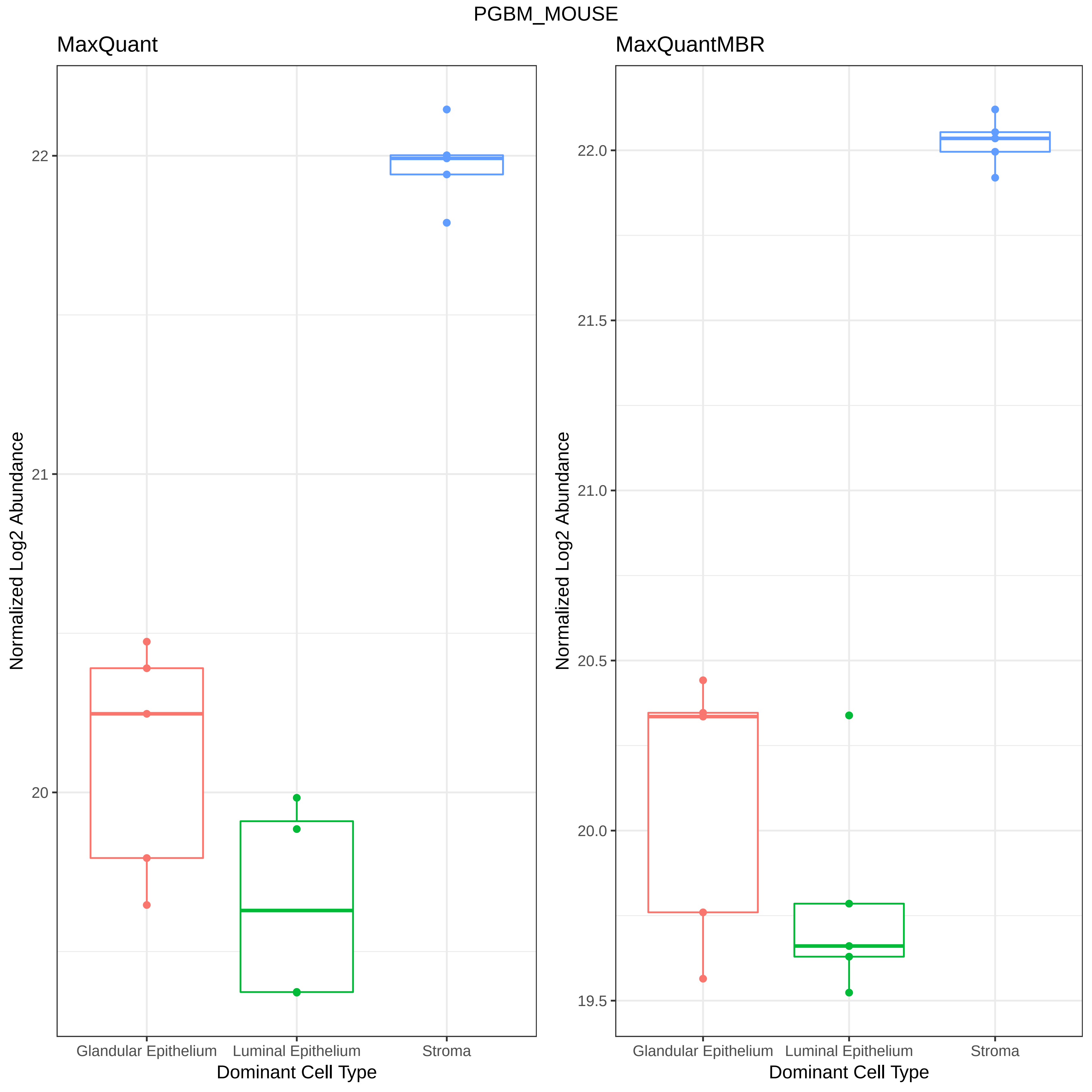

PGBM_MOUSE
MaxQuant
MaxQuantMBR
Normalized Log2 Abundance
22
21
20
22.0
21.5
21.0
20.5
20.0
19.5
Glandular Epithelium
Luminal Epithelium
Stroma
Dominant Cell Type

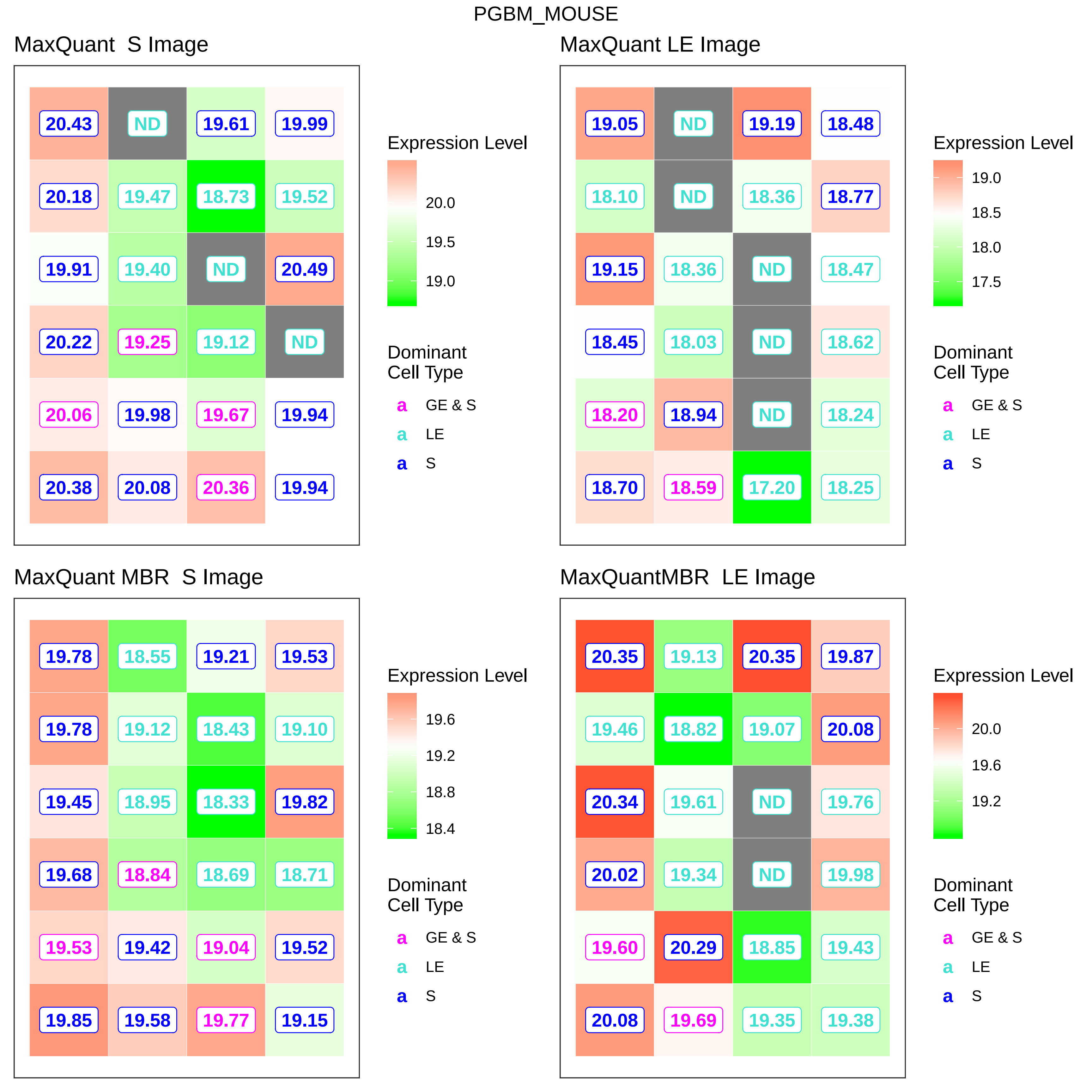
PGBM_MOUSE
MaxQuant S Image
20.43 ND 19.61 19.99
20.18 19.47 18.73 19.52
19.91 19.40 ND 20.49
20.22 19.25 19.12 ND
20.06 19.98 19.67 19.94
20.38 20.08 20.36 19.94
Expression Level
20.0
19.5
19.0
Dominant Cell Type
a GE & S
a LE
a S
MaxQuant LE Image
19.05 ND 19.19 18.48
18.10 ND 18.36 18.77
19.15 18.36 ND 18.47
18.45 18.03 ND 18.62
18.20 18.94 ND 18.24
18.70 18.59 17.20 18.25
Expression Level
19.0
18.5
18.0
17.5
Dominant Cell Type
a GE & S
a LE
a S
MaxQuant MBR S Image
19.78 18.55 19.21 19.53
19.78 19.12 18.43 19.10
19.45 18.95 18.33 19.82
19.68 18.84 18.69 18.71
19.53 19.42 19.04 19.52
19.85 19.58 19.77 19.15
Expression Level
19.6
19.2
18.8
18.4
Dominant Cell Type
a GE & S
a LE
a S
MaxQuantMBR LE Image
20.35 19.13 20.35 19.87
19.46 18.82 19.07 20.08
20.34 19.61 ND 19.76
20.02 19.34 ND 19.98
19.60 20.29 18.85 19.43
20.08 19.69 19.35 19.38
Expression Level
20.0
19.6
19.2
Dominant Cell Type
a GE & S
a LE
a S

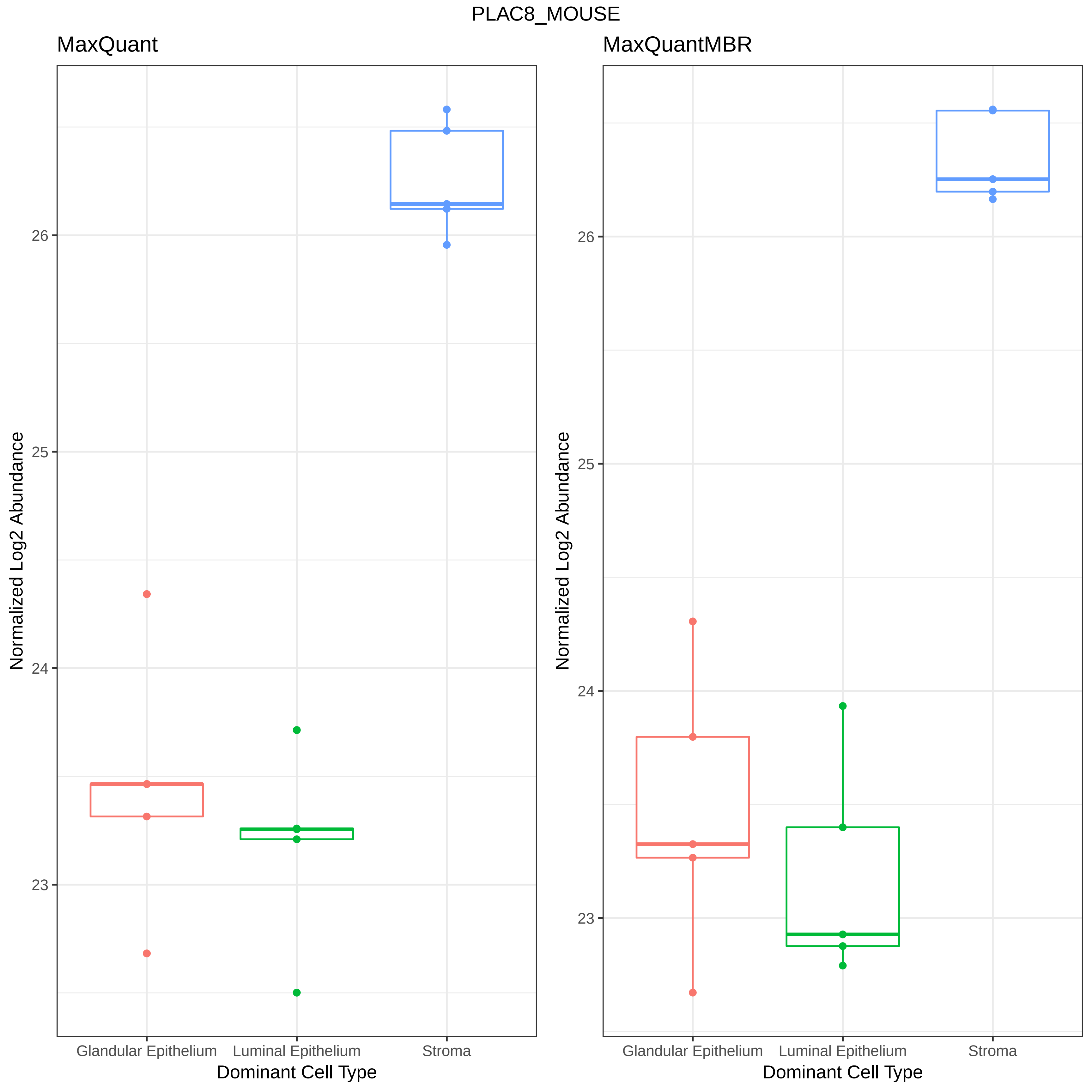
PLAC8_MOUSE
MaxQuant
MaxQuantMBR
Normalized Log2 Abundance
26
25
24
23
Glandular Epithelium
Luminal Epithelium
Stroma
Dominant Cell Type

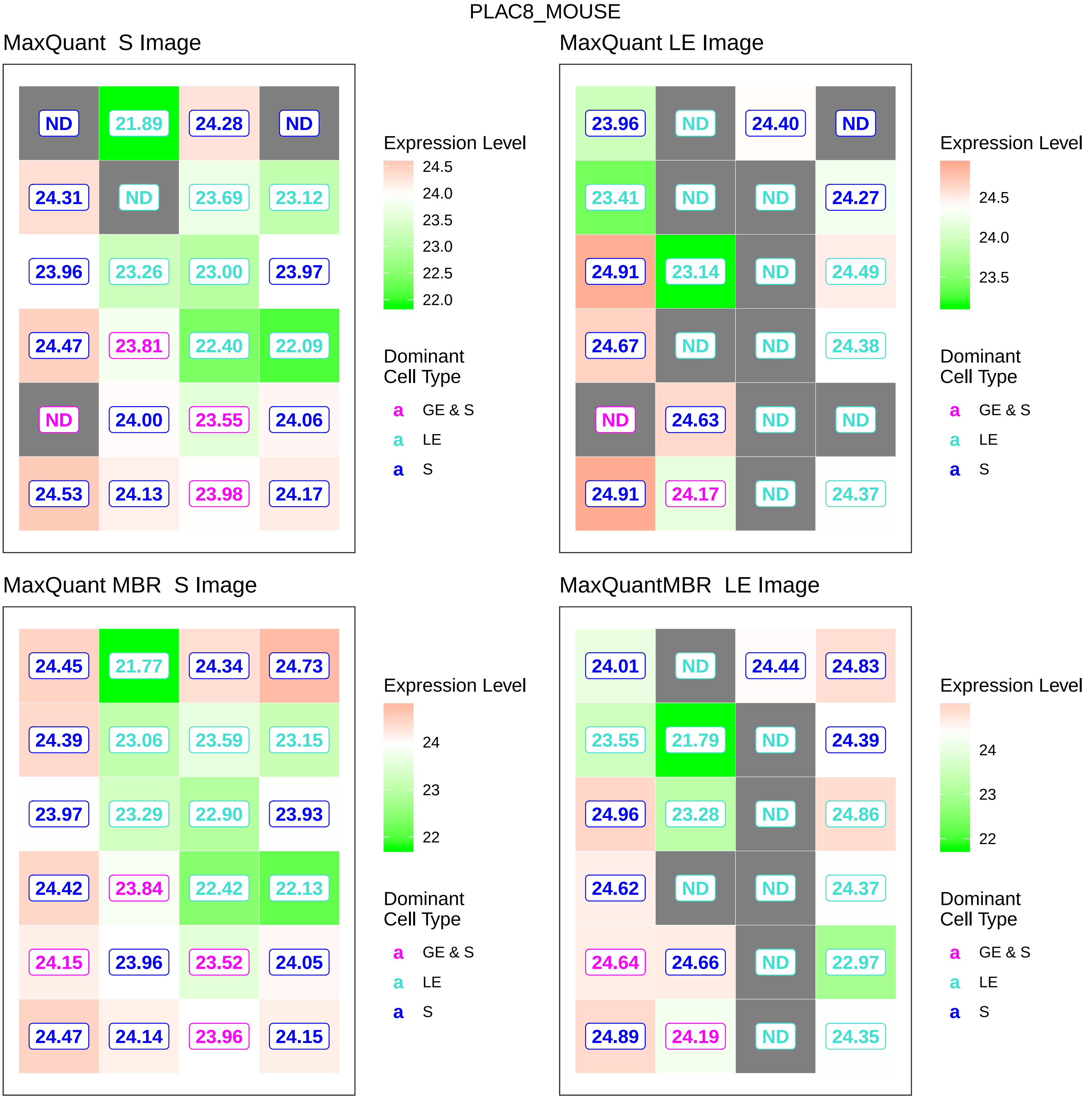

PLAC8_MOUSE
MaxQuant S Image
ND 21.89 24.28 ND
24.31 ND 23.69 23.12
23.96 23.26 23.00 23.97
24.47 23.81 22.40 22.09
ND 24.00 23.55 24.06
24.53 24.13 23.98 24.17
Expression Level
24.5
24.0
23.5
23.0
22.5
22.0
Dominant Cell Type
a GE & S
a LE
a S
MaxQuant LE Image
23.96 ND 24.40 ND
23.41 ND ND 24.27
24.91 23.14 ND 24.49
24.67 ND ND 24.38
ND 24.63 ND ND
24.91 24.17 ND 24.37
Expression Level
24.5
24.0
23.5
Dominant Cell Type
a GE & S
a LE
a S
MaxQuant MBR S Image
24.45 21.77 24.34 24.73
24.39 23.06 23.59 23.15
23.97 23.29 22.90 23.93
24.42 23.84 22.42 22.13
24.15 23.96 23.52 24.05
24.47 24.14 23.96 24.15
Expression Level
24
23
22
Dominant Cell Type
a GE & S
a LE
a S
MaxQuantMBR LE Image
24.01 ND 24.44 24.83
23.55 21.79 ND 24.39
24.96 23.28 ND 24.86
24.62 ND ND 24.37
24.64 24.66 ND 22.97
24.89 24.19 ND 24.35
Expression Level
24
23
22
Dominant Cell Type
a GE & S
a LE
a S

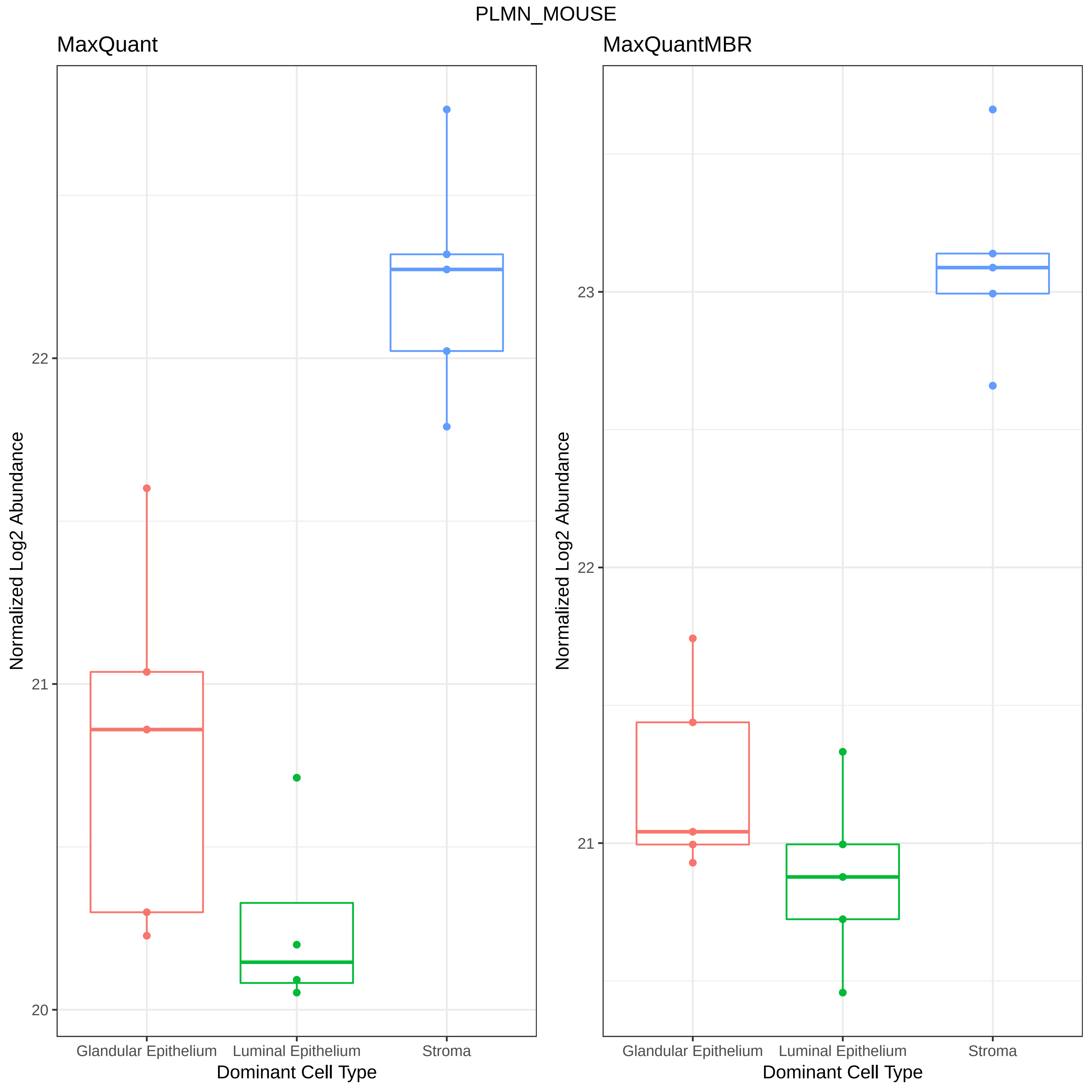

PLMN_MOUSE
MaxQuant
MaxQuantMBR
Normalized Log2 Abundance
22
21
20
23
Glandular Epithelium
Luminal Epithelium
Stroma
Dominant Cell Type

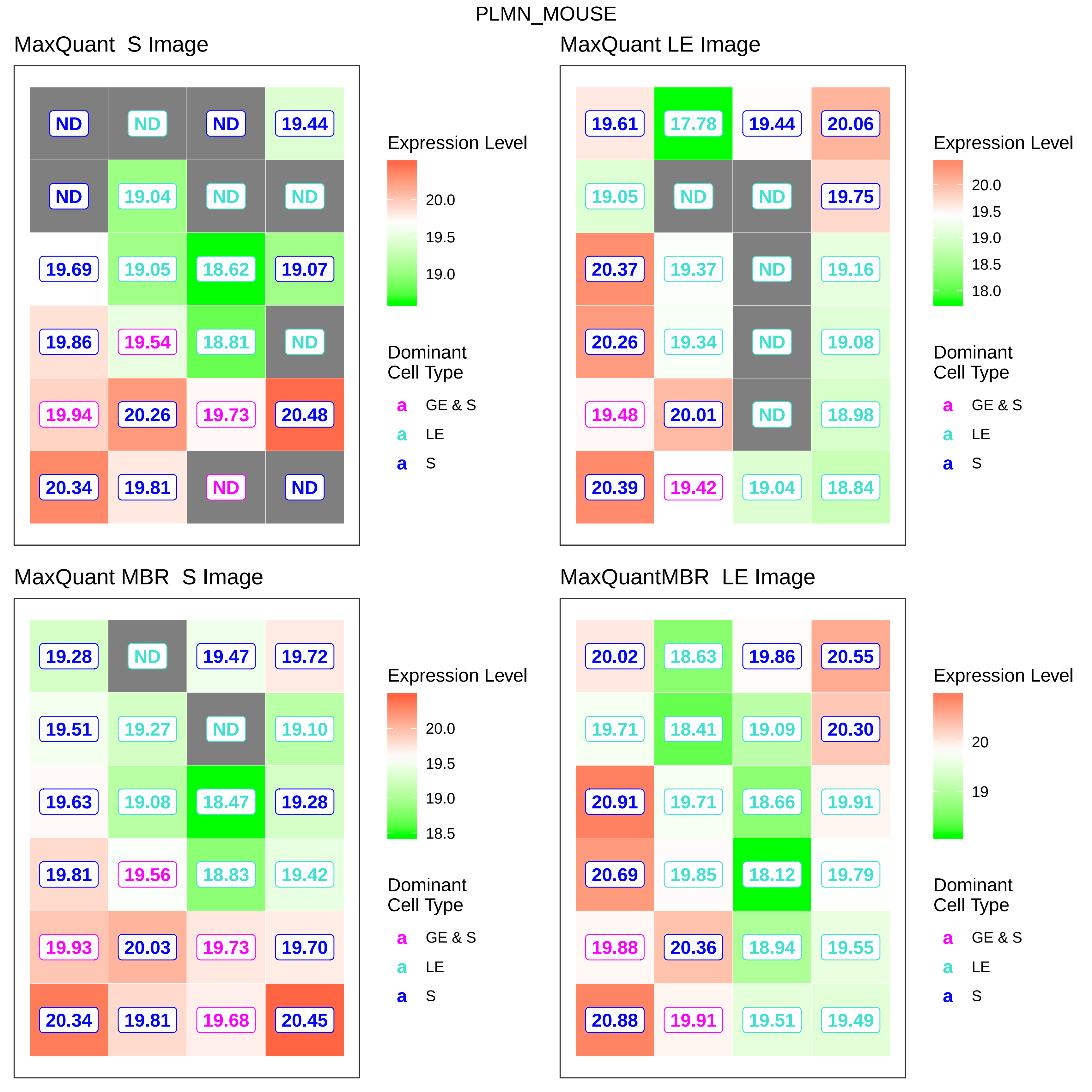
PLMN_MOUSE
MaxQuant S Image
ND ND ND 19.44
ND 19.04 ND ND
19.69 19.05 18.62 19.07
19.86 19.54 18.81 ND
19.94 20.26 19.73 20.48
20.34 19.81 ND ND
Expression Level
20.0
19.5
19.0
Dominant Cell Type
a GE & S
a LE
a S
MaxQuant LE Image
19.61 17.78 19.44 20.06
19.05 ND ND 19.75
20.37 19.37 ND 19.16
20.26 19.34 ND 19.08
19.48 20.01 ND 18.98
20.39 19.42 19.04 18.84
Expression Level
20.0
19.5
19.0
18.5
18.0
Dominant Cell Type
a GE & S
a LE
a S
MaxQuant MBR S Image
19.28 ND 19.47 19.72
19.51 19.27 ND 19.10
19.63 19.08 18.47 19.28
19.81 19.56 18.83 19.42
19.93 20.03 19.73 19.70
20.34 19.81 19.68 20.45
Expression Level
20.0
19.5
19.0
18.5
Dominant Cell Type
a GE & S
a LE
a S
MaxQuantMBR LE Image
20.02 18.63 19.86 20.55
19.71 18.41 19.09 20.30
20.91 19.71 18.66 19.91
20.69 19.85 18.12 19.79
19.88 20.36 18.94 19.55
20.88 19.91 19.51 19.49
Expression Level
20
19
Dominant Cell Type
a GE & S
a LE
a S

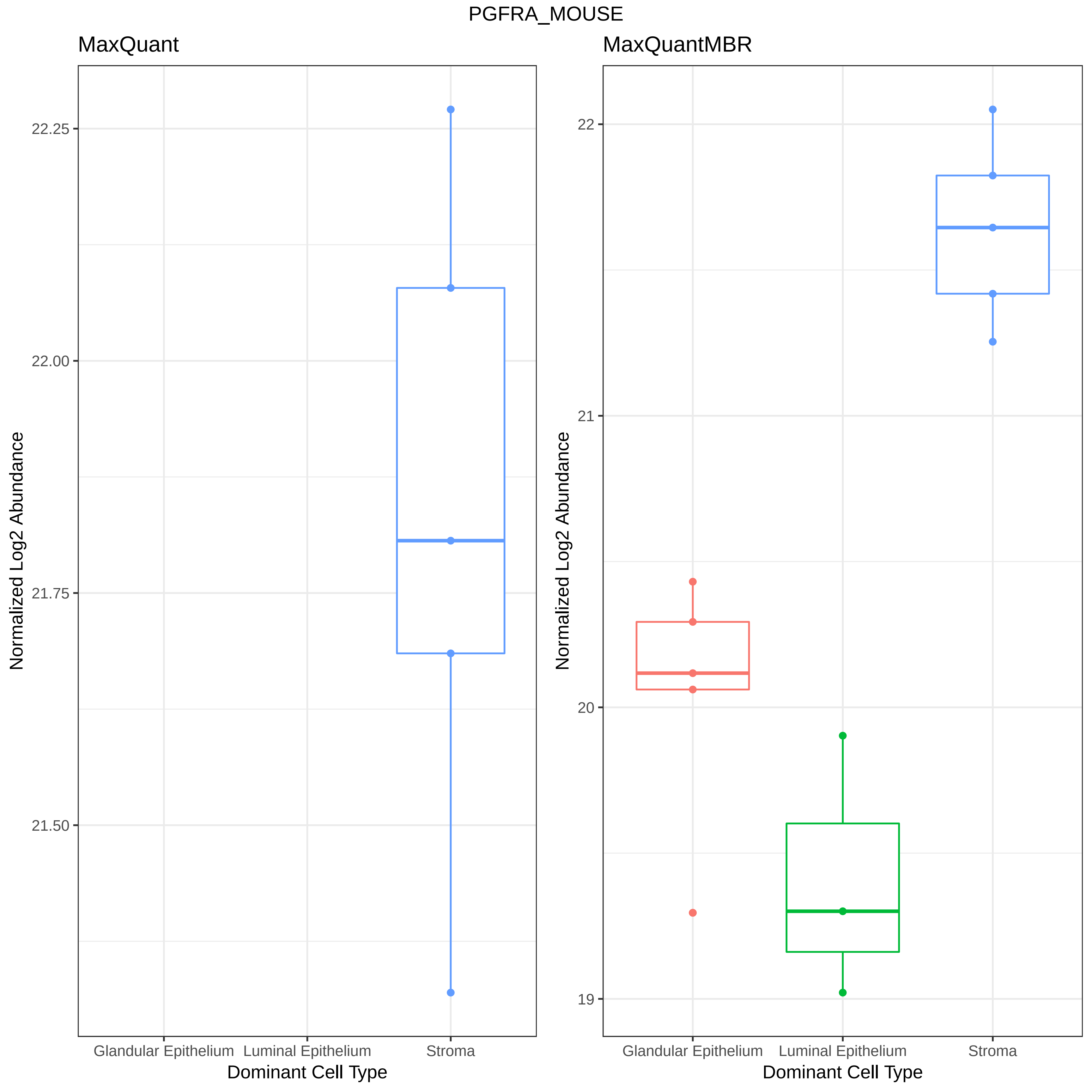
PGFRA_MOUSE
MaxQuant
MaxQuantMBR
22.25
22.00
21.75
21.50
22
21
20
19
Normalized Log2 Abundance
Normalized Log2 Abundance
Glandular Epithelium
Luminal Epithelium
Stroma
Glandular Epithelium
Luminal Epithelium
Stroma
Dominant Cell Type
Dominant Cell Type

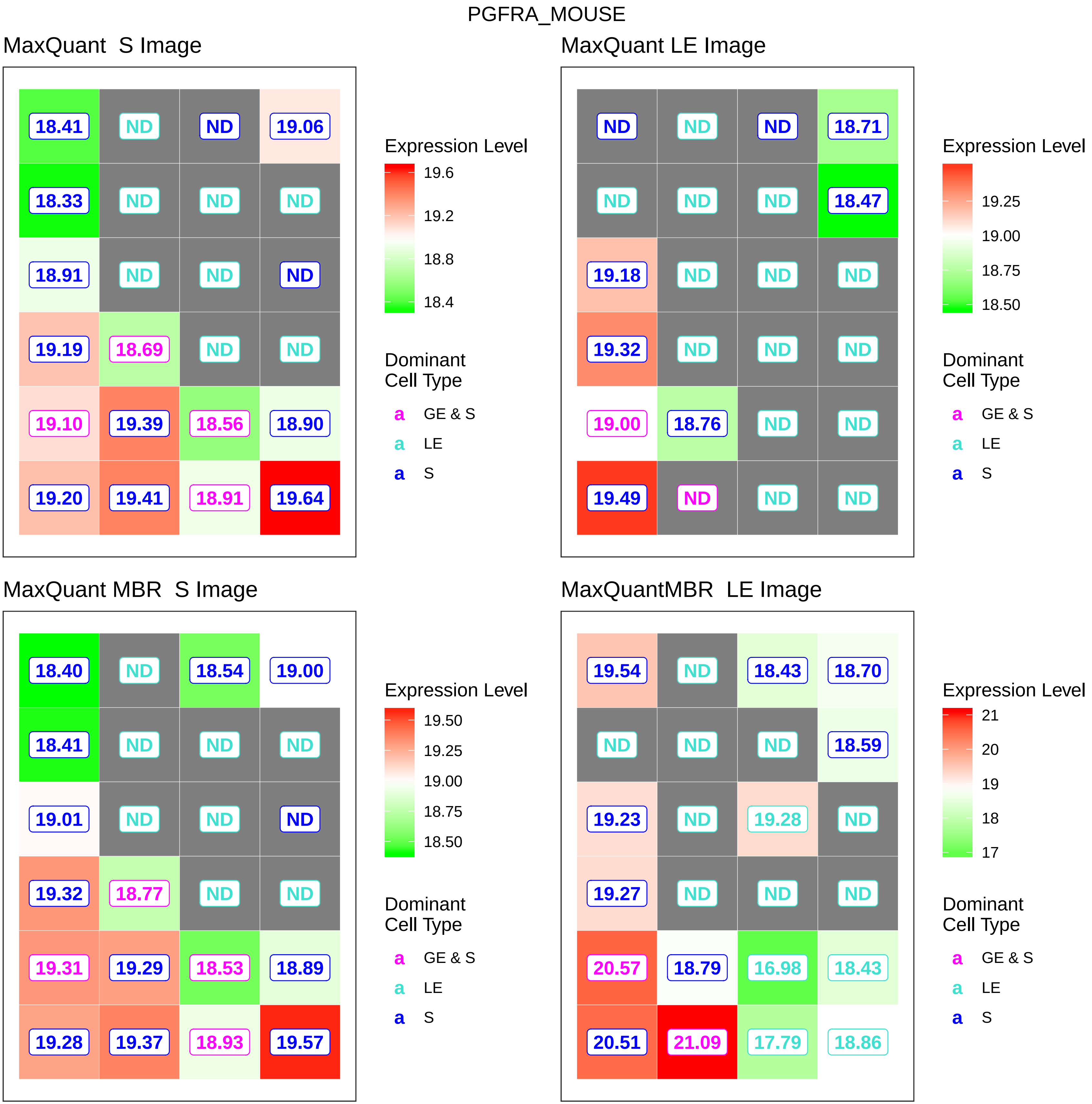
PGFRA_MOUSE
MaxQuant S Image
18.41 ND ND 19.06
18.33 ND ND ND
18.91 ND ND ND
19.19 18.69 ND ND
19.10 19.39 18.56 18.90
19.20 19.41 18.91 19.64
Expression Level
19.6
19.2
18.8
18.4
Dominant Cell Type
a GE & S
a LE
a S
MaxQuant LE Image
ND ND ND 18.71
ND ND ND 18.47
19.18 ND ND ND
19.32 ND ND ND
19.00 18.76 ND ND
19.49 ND ND ND
Expression Level
19.25
19.00
18.75
18.50
Dominant Cell Type
a GE & S
a LE
a S
MaxQuant MBR S Image
18.40 ND 18.54 19.00
18.41 ND ND ND
19.01 ND ND ND
19.32 18.77 ND ND
19.31 19.29 18.53 18.89
19.28 19.37 18.93 19.57
Expression Level
19.50
19.25
19.00
18.75
18.50
Dominant Cell Type
a GE & S
a LE
a S
MaxQuantMBR LE Image
19.54 ND 18.43 18.70
ND ND ND 18.59
19.23 ND 19.28 ND
19.27 ND ND ND
20.57 18.79 16.98 18.43
20.51 21.09 17.79 18.86
Expression Level
21
20
19
18
17
Dominant Cell Type
a GE & S
a LE
a S

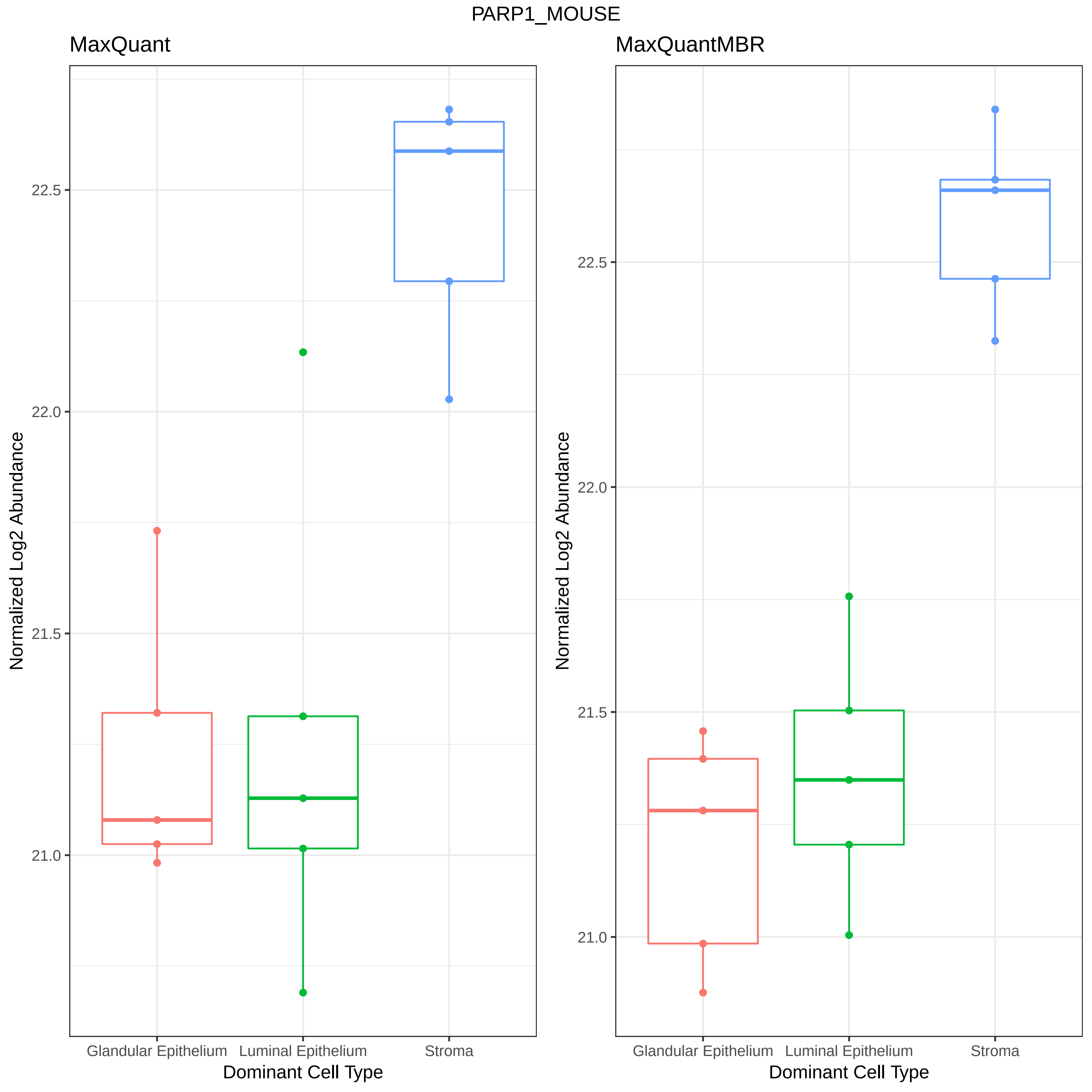
PARP1_MOUSE
MaxQuant
MaxQuantMBR
Normalized Log2 Abundance
22.5
22.0
21.5
21.0
Glandular Epithelium
Luminal Epithelium
Stroma
Dominant Cell Type

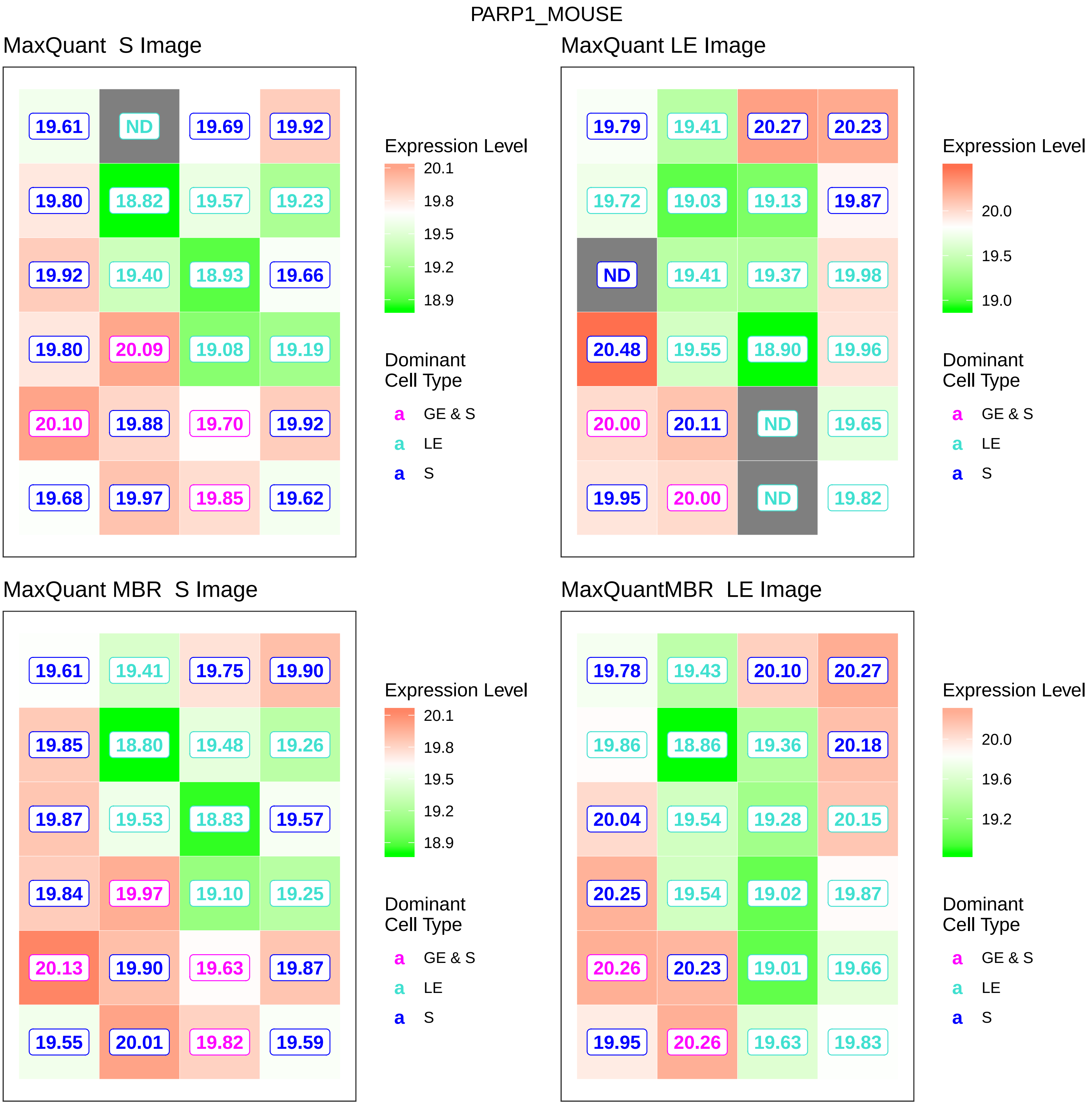
PARP1_MOUSE
MaxQuant S Image
19.61 ND 19.69 19.92
19.80 18.82 19.57 19.23
19.92 19.40 18.93 19.66
19.80 20.09 19.08 19.19
20.10 19.88 19.70 19.92
19.68 19.97 19.85 19.62
Expression Level
20.1
19.8
19.5
19.2
18.9
Dominant Cell Type
a GE & S
a LE
a S
MaxQuant LE Image
19.79 19.41 20.27 20.23
19.72 19.03 19.13 19.87
ND 19.41 19.37 19.98
20.48 19.55 18.90 19.96
20.00 20.11 ND 19.65
19.95 20.00 ND 19.82
Expression Level
20.0
19.5
19.0
Dominant Cell Type
a GE & S
a LE
a S
MaxQuant MBR S Image
19.61 19.41 19.75 19.90
19.85 18.80 19.48 19.26
19.87 19.53 18.83 19.57
19.84 19.97 19.10 19.25
20.13 19.90 19.63 19.87
19.55 20.01 19.82 19.59
Expression Level
20.1
19.8
19.5
19.2
18.9
Dominant Cell Type
a GE & S
a LE
a S
MaxQuantMBR LE Image
19.78 19.43 20.10 20.27
19.86 18.86 19.36 20.18
20.04 19.54 19.28 20.15
20.25 19.54 19.02 19.87
20.26 20.23 19.01 19.66
19.95 20.26 19.63 19.83
Expression Level
20.0
19.6
19.2
Dominant Cell Type
a GE & S
a LE
a S

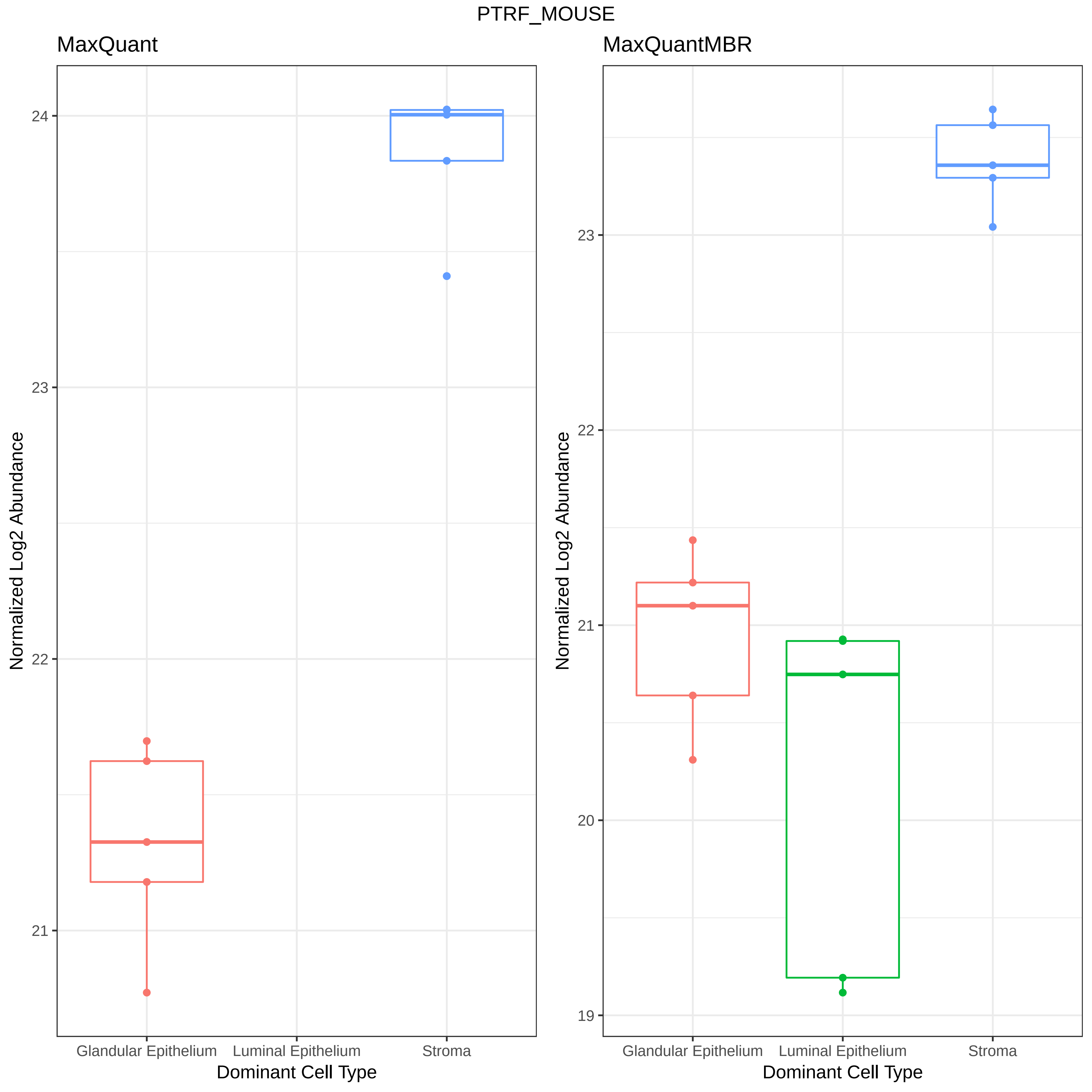

PTRF_MOUSE
MaxQuant
MaxQuantMBR
Normalized Log2 Abundance
24
23
22
21
20
19
Glandular Epithelium
Luminal Epithelium
Stroma
Dominant Cell Type

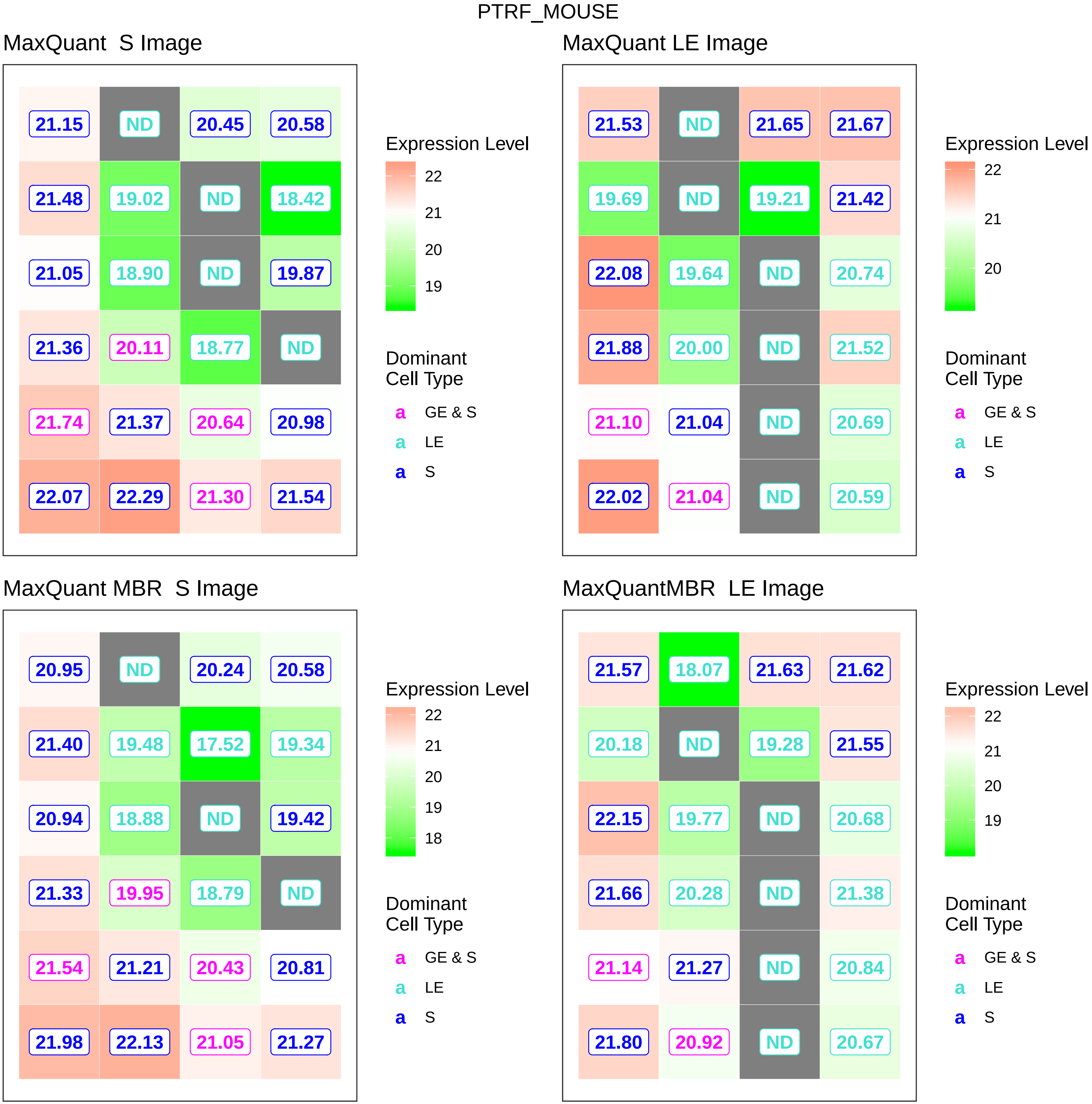
PTRF_MOUSE
MaxQuant S Image
21.15 ND 20.45 20.58
21.48 19.02 ND 18.42
21.05 18.90 ND 19.87
21.36 20.11 18.77 ND
21.74 21.37 20.64 20.98
22.07 22.29 21.30 21.54
Expression Level
22
21
20
19
Dominant Cell Type
a GE & S
a LE
a S
MaxQuant LE Image
21.53 ND 21.65 21.67
19.69 ND 19.21 21.42
22.08 19.64 ND 20.74
21.88 20.00 ND 21.52
21.10 21.04 ND 20.69
22.02 21.04 ND 20.59
Expression Level
22
21
20
Dominant Cell Type
a GE & S
a LE
a S
MaxQuant MBR S Image
20.95 ND 20.24 20.58
21.40 19.48 17.52 19.34
20.94 18.88 ND 19.42
21.33 19.95 18.79 ND
21.54 21.21 20.43 20.81
21.98 22.13 21.05 21.27
Expression Level
22
21
20
19
18
Dominant Cell Type
a GE & S
a LE
a S
MaxQuantMBR LE Image
21.57 18.07 21.63 21.62
20.18 ND 19.28 21.55
22.15 19.77 ND 20.68
21.66 20.28 ND 21.38
21.14 21.27 ND 20.84
21.80 20.92 ND 20.67
Expression Level
22
21
20
19
Dominant Cell Type
a GE & S
a LE
a S

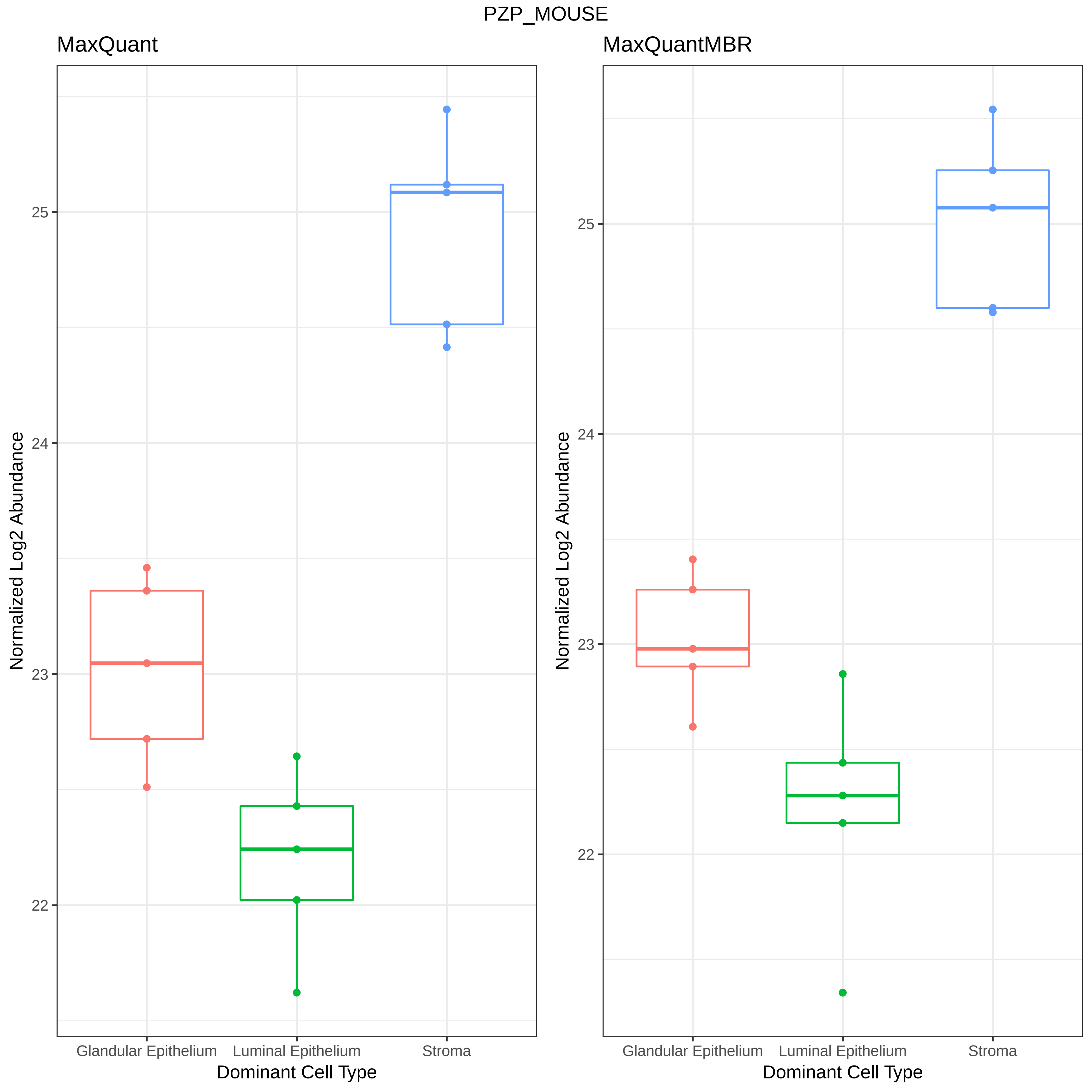

PZP_MOUSE
MaxQuant
MaxQuantMBR
Normalized Log2 Abundance
25
24
23
22
Glandular Epithelium
Luminal Epithelium
Stroma
Dominant Cell Type

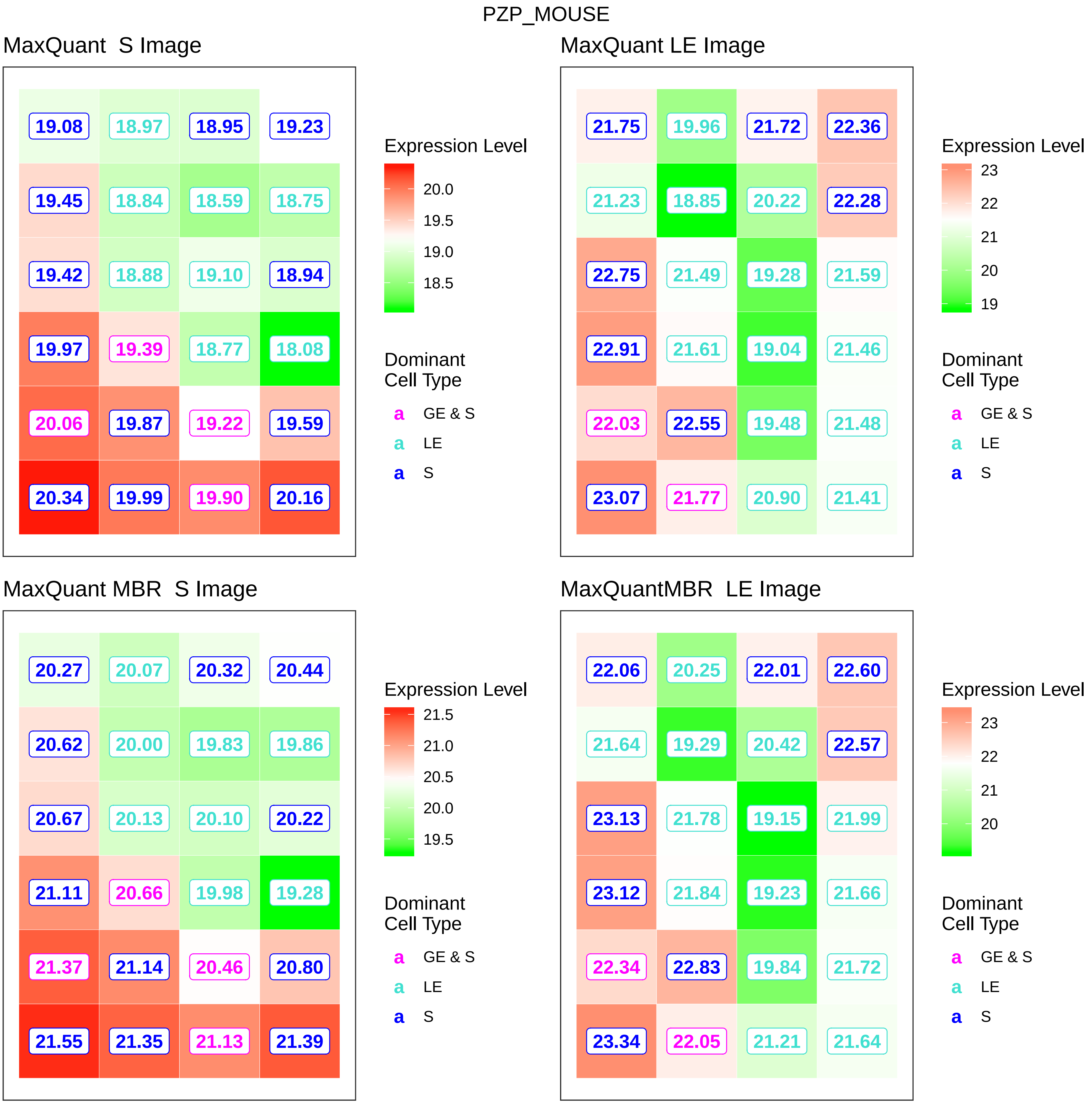
PZP_MOUSE
MaxQuant S Image
19.08 18.97 18.95 19.23
19.45 18.84 18.59 18.75
19.42 18.88 19.10 18.94
19.97 19.39 18.77 18.08
20.06 19.87 19.22 19.59
20.34 19.99 19.90 20.16
Expression Level
20.0
19.5
19.0
18.5
Dominant Cell Type
a GE & S
a LE
a S
MaxQuant LE Image
21.75 19.96 21.72 22.36
21.23 18.85 20.22 22.28
22.75 21.49 19.28 21.59
22.91 21.61 19.04 21.46
22.03 22.55 19.48 21.48
23.07 21.77 20.90 21.41
Expression Level
23
22
21
20
19
Dominant Cell Type
a GE & S
a LE
a S
MaxQuant MBR S Image
20.27 20.07 20.32 20.44
20.62 20.00 19.83 19.86
20.67 20.13 20.10 20.22
21.11 20.66 19.98 19.28
21.37 21.14 20.46 20.80
21.55 21.35 21.13 21.39
Expression Level
21.5
21.0
20.5
20.0
19.5
Dominant Cell Type
a GE & S
a LE
a S
MaxQuantMBR LE Image
22.06 20.25 22.01 22.60
21.64 19.29 20.42 22.57
23.13 21.78 19.15 21.99
23.12 21.84 19.23 21.66
22.34 22.83 19.84 21.72
23.34 22.05 21.21 21.64
Expression Level
23
22
21
20
Dominant Cell Type
a GE & S
a LE
a S

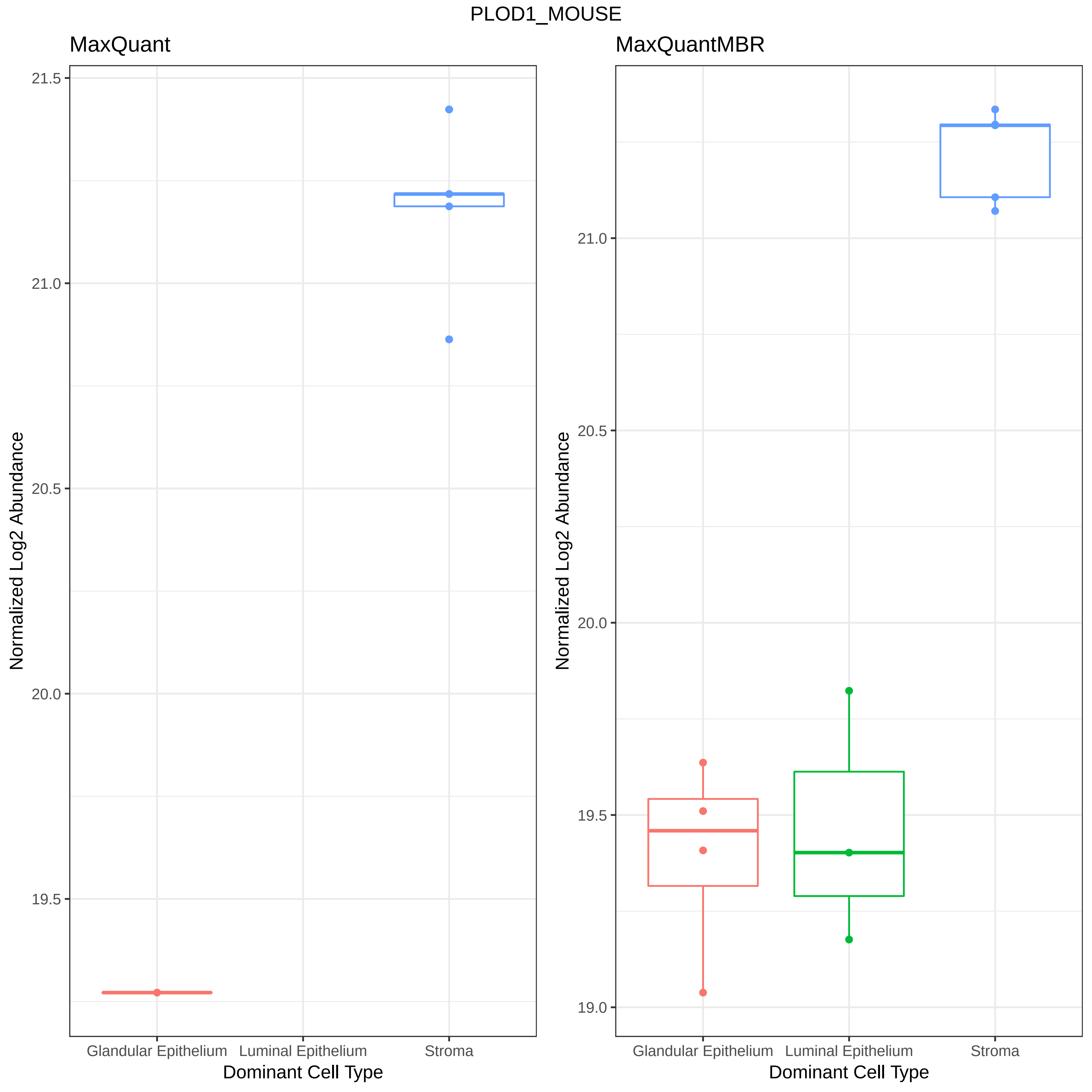
PLOD1_MOUSE
MaxQuant
MaxQuantMBR
Normalized Log2 Abundance
21.5
21.0
20.5
20.0
19.5
19.0
Glandular Epithelium
Luminal Epithelium
Stroma
Dominant Cell Type

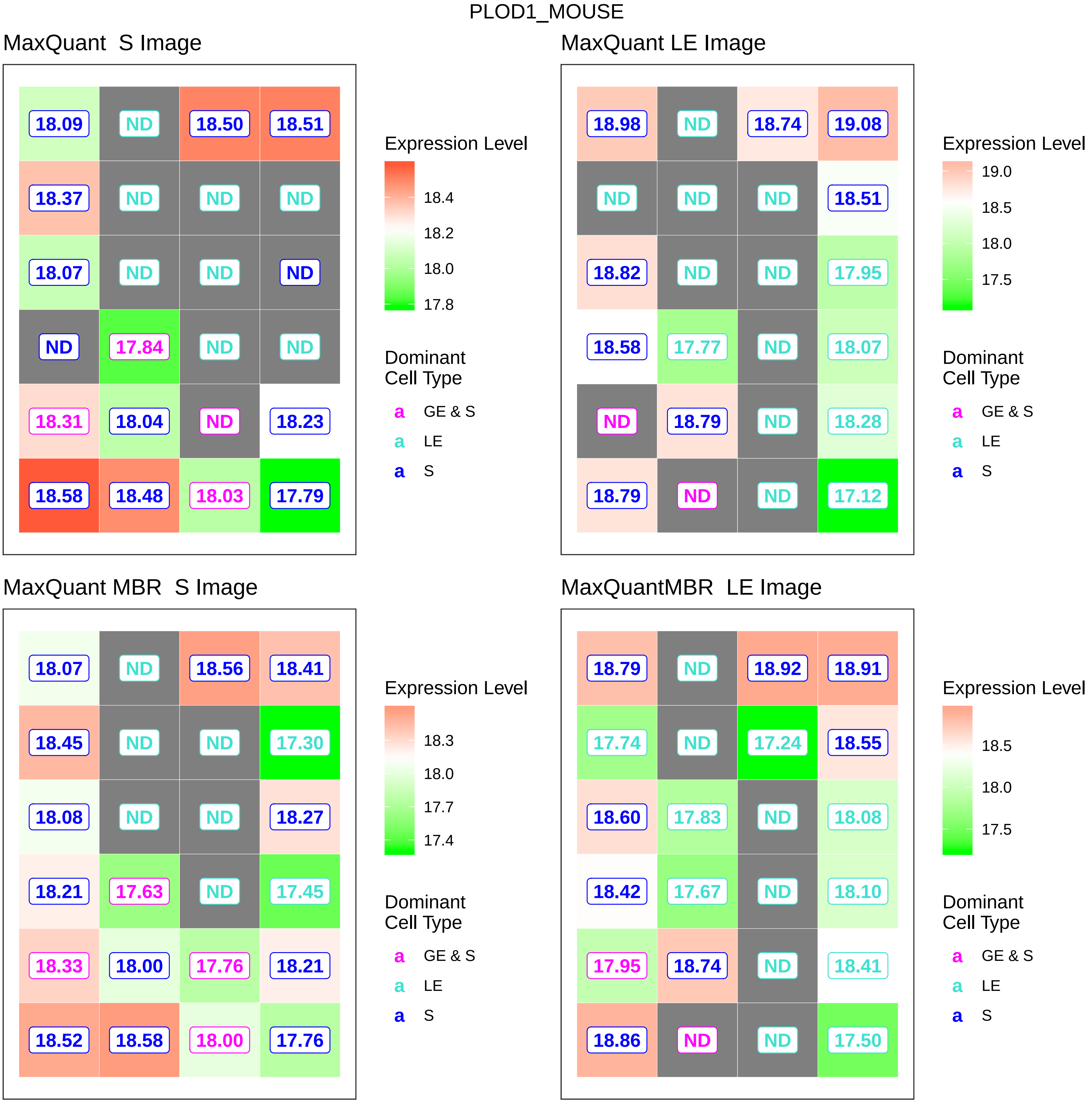
PLOD1_MOUSE
MaxQuant S Image
18.09 ND 18.50 18.51
18.37 ND ND ND
18.07 ND ND ND
ND 17.84 ND ND
18.31 18.04 ND 18.23
18.58 18.48 18.03 17.79
Expression Level
18.4
18.2
18.0
17.8
Dominant Cell Type
a GE & S
a LE
a S
MaxQuant LE Image
18.98 ND 18.74 19.08
ND ND ND 18.51
18.82 ND ND 17.95
18.58 17.77 ND 18.07
ND 18.79 ND 18.28
18.79 ND ND 17.12
Expression Level
19.0
18.5
18.0
17.5
Dominant Cell Type
a GE & S
a LE
a S
MaxQuant MBR S Image
18.07 ND 18.56 18.41
18.45 ND ND 17.30
18.08 ND ND 18.27
18.21 17.63 ND 17.45
18.33 18.00 17.76 18.21
18.52 18.58 18.00 17.76
Expression Level
18.3
18.0
17.7
17.4
Dominant Cell Type
a GE & S
a LE
a S
MaxQuantMBR LE Image
18.79 ND 18.92 18.91
17.74 ND 17.24 18.55
18.60 17.83 ND 18.08
18.42 17.67 ND 18.10
17.95 18.74 ND 18.41
18.86 ND ND 17.50
Expression Level
18.5
18.0
17.5
Dominant Cell Type
a GE & S
a LE
a S

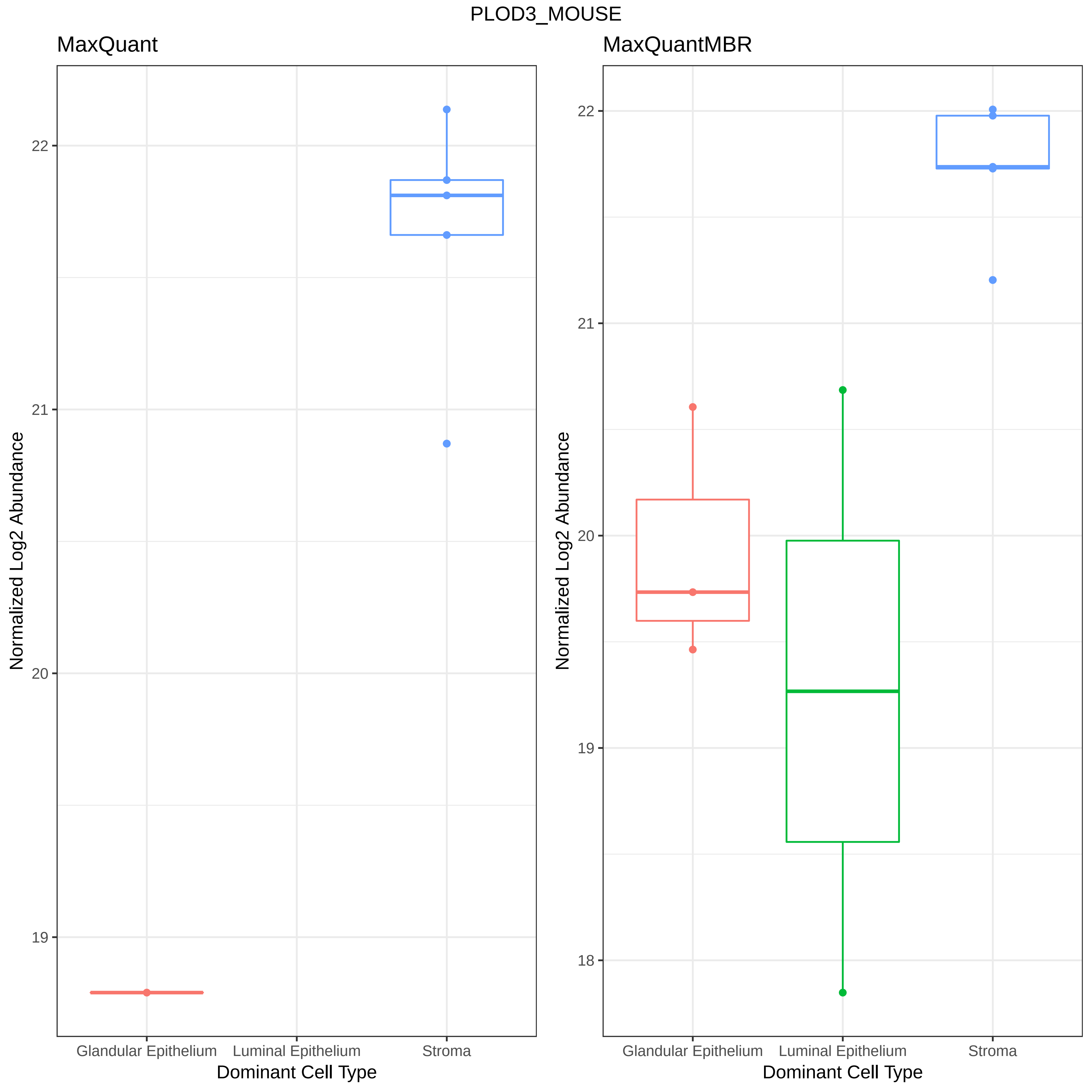
PLOD3_MOUSE
MaxQuant
MaxQuantMBR
Normalized Log2 Abundance
22
21
20
19
18
Glandular Epithelium
Luminal Epithelium
Stroma
Dominant Cell Type

PLOD3_MOUSE

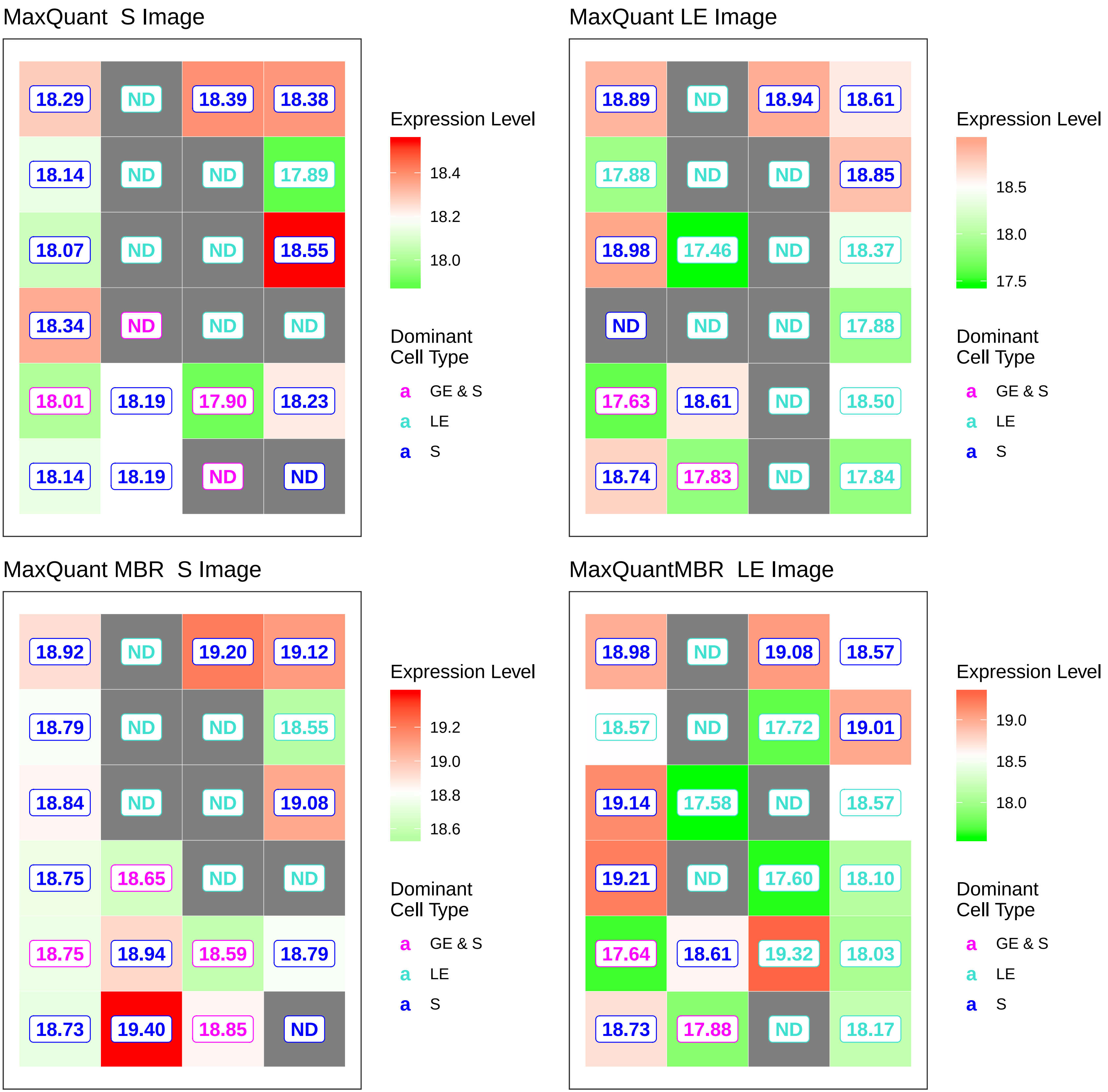
MaxQuant S Image
18.29 ND 18.39 18.38
18.14 ND ND 17.89
18.07 ND ND 18.55
18.34 ND ND ND
18.01 18.19 17.90 18.23
18.14 18.19 ND ND
Expression Level
18.4
18.2
18.0
Dominant Cell Type
a GE & S
a LE
a S
MaxQuant LE Image
18.89 ND 18.94 18.61
17.88 ND ND 18.85
18.98 17.46 ND 18.37
ND ND ND 17.88
17.63 18.61 ND 18.50
18.74 17.83 ND 17.84
Expression Level
18.5
18.0
17.5
Dominant Cell Type
a GE & S
a LE
a S
MaxQuant MBR S Image
18.92 ND 19.20 19.12
18.79 ND ND 18.55
18.84 ND ND 19.08
18.75 18.65 ND ND
18.75 18.94 18.59 18.79
18.73 19.40 18.85 ND
Expression Level
19.2
19.0
18.8
18.6
Dominant Cell Type
a GE & S
a LE
a S
MaxQuantMBR LE Image
18.98 ND 19.08 18.57
18.57 ND 17.72 19.01
19.14 17.58 ND 18.57
19.21 ND 17.60 18.10
17.64 18.61 19.32 18.03
18.73 17.88 ND 18.17
Expression Level
19.0
18.5
18.0
Dominant Cell Type
a GE & S
a LE
a S

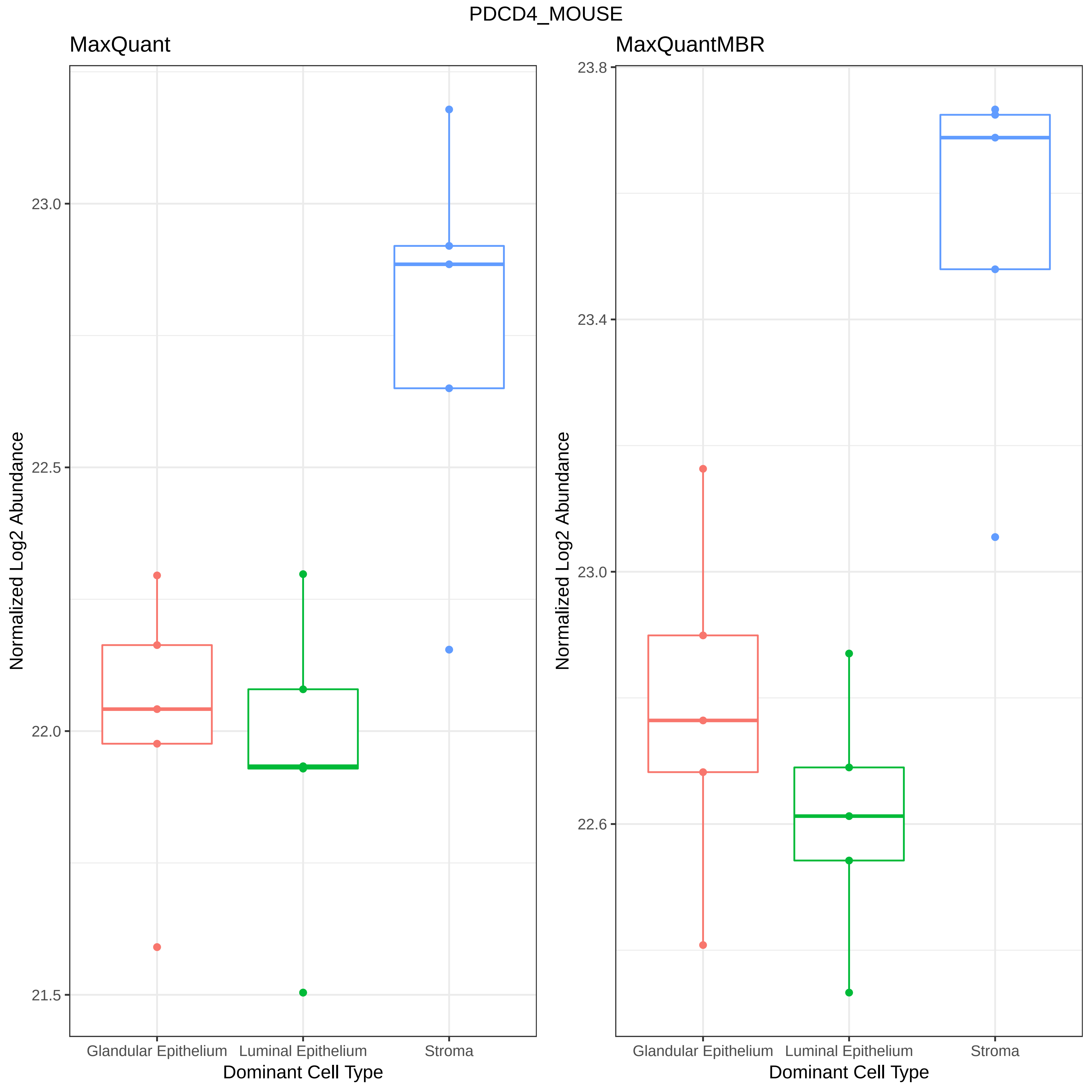
PDCD4_MOUSE
MaxQuant
MaxQuantMBR
Normalized Log2 Abundance
23.0
22.5
22.0
21.5
23.8
23.4
23.0
22.6
Glandular Epithelium
Luminal Epithelium
Stroma
Dominant Cell Type

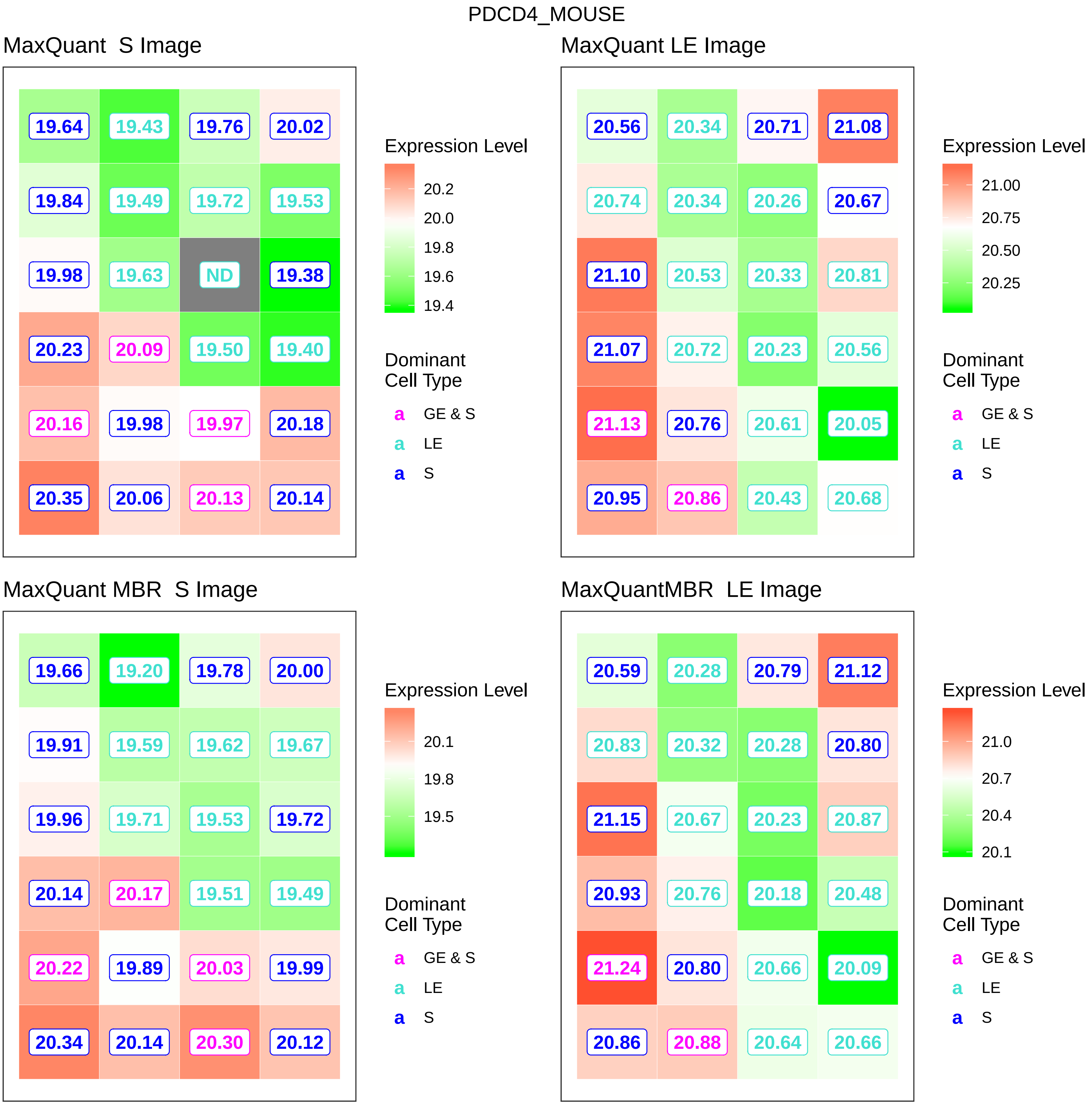
PDCD4_MOUSE
MaxQuant S Image
19.64 19.43 19.76 20.02
19.84 19.49 19.72 19.53
19.98 19.63 ND 19.38
20.23 20.09 19.50 19.40
20.16 19.98 19.97 20.18
20.35 20.06 20.13 20.14
Expression Level
20.2
20.0
19.8
19.6
19.4
Dominant Cell Type
a GE & S
a LE
a S
MaxQuant LE Image
20.56 20.34 20.71 21.08
20.74 20.34 20.26 20.67
21.10 20.53 20.33 20.81
21.07 20.72 20.23 20.56
21.13 20.76 20.61 20.05
20.95 20.86 20.43 20.68
Expression Level
21.00
20.75
20.50
20.25
Dominant Cell Type
a GE & S
a LE
a S
MaxQuant MBR S Image
19.66 19.20 19.78 20.00
19.91 19.59 19.62 19.67
19.96 19.71 19.53 19.72
20.14 20.17 19.51 19.49
20.22 19.89 20.03 19.99
20.34 20.14 20.30 20.12
Expression Level
20.1
19.8
19.5
Dominant Cell Type
a GE & S
a LE
a S
MaxQuantMBR LE Image
20.59 20.28 20.79 21.12
20.83 20.32 20.28 20.80
21.15 20.67 20.23 20.87
20.93 20.76 20.18 20.48
21.24 20.80 20.66 20.09
20.86 20.88 20.64 20.66
Expression Level
21.0
20.7
20.4
20.1
Dominant Cell Type
a GE & S
a LE
a S

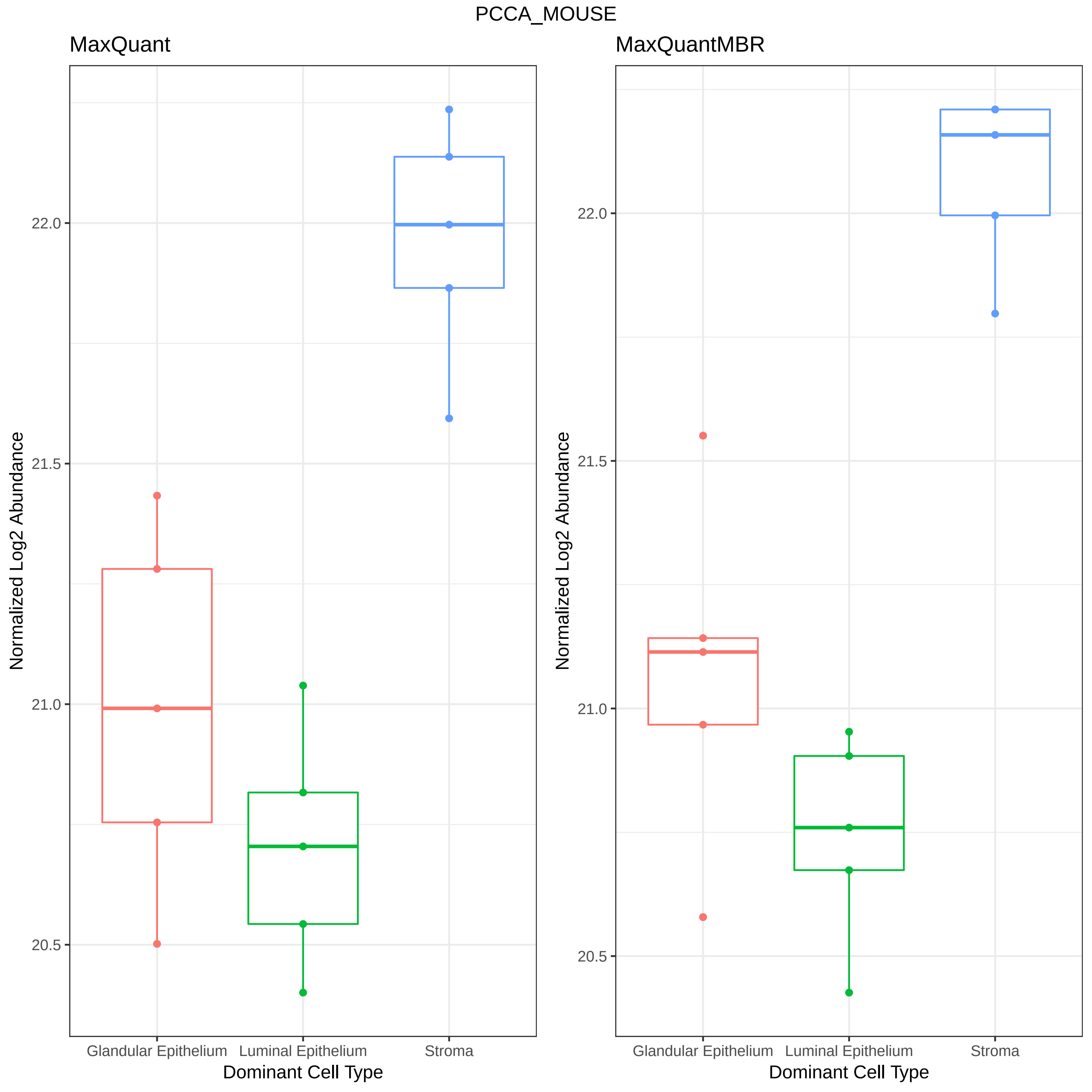
PCCA_MOUSE
MaxQuant
MaxQuantMBR
Normalized Log2 Abundance
22.0
21.5
21.0
20.5
Glandular Epithelium
Luminal Epithelium
Stroma
Dominant Cell Type

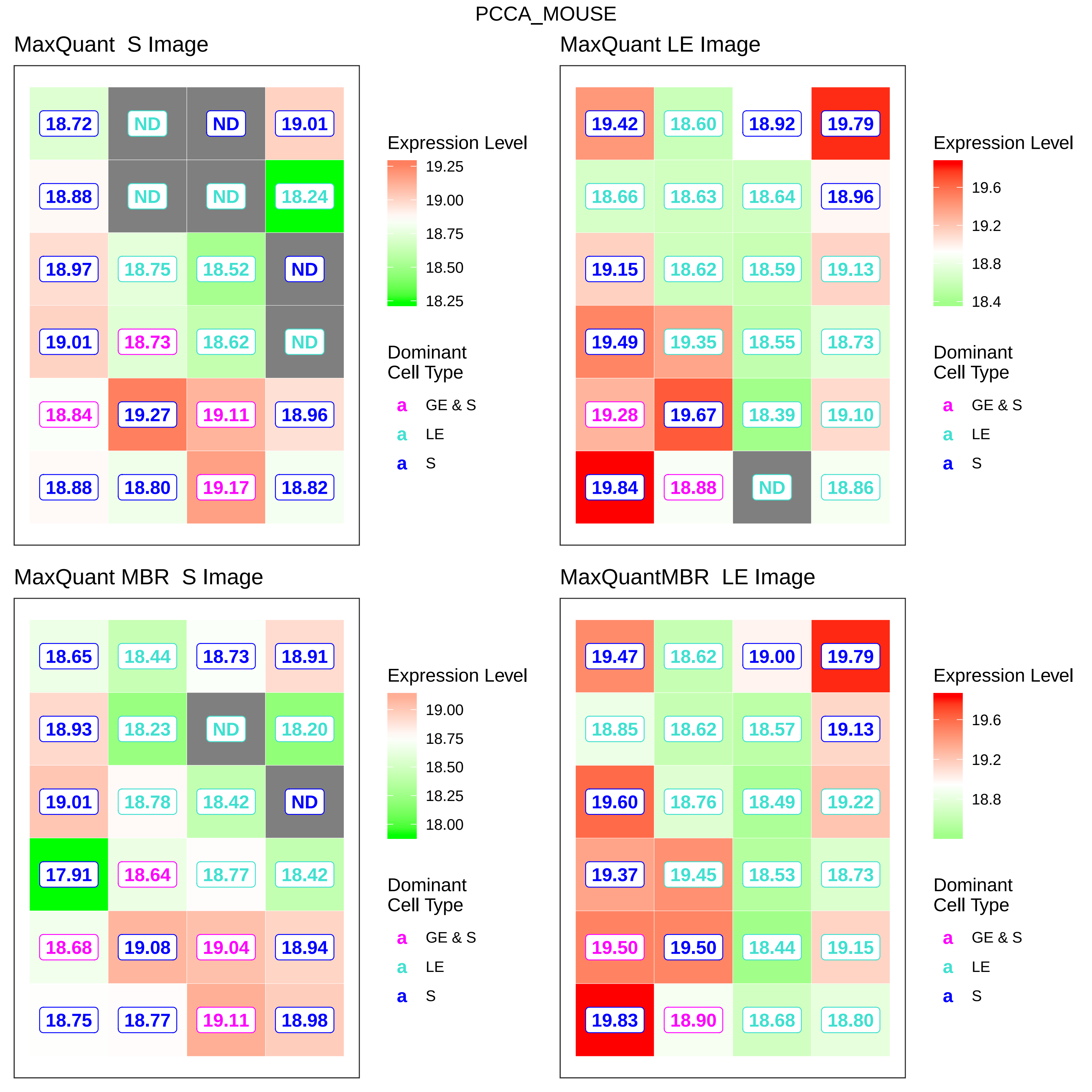

PCCA_MOUSE
MaxQuant S Image
18.72 ND ND 19.01
18.88 ND ND 18.24
18.97 18.75 18.52 ND
19.01 18.73 18.62 ND
18.84 19.27 19.11 18.96
18.88 18.80 19.17 18.82
Expression Level
19.25
19.00
18.75
18.50
18.25
Dominant Cell Type
a GE & S
a LE
a S
MaxQuant LE Image
19.42 18.60 18.92 19.79
18.66 18.63 18.64 18.96
19.15 18.62 18.59 19.13
19.49 19.35 18.55 18.73
19.28 19.67 18.39 19.10
19.84 18.88 ND 18.86
Expression Level
19.6
19.2
18.8
18.4
Dominant Cell Type
a GE & S
a LE
a S
MaxQuant MBR S Image
18.65 18.44 18.73 18.91
18.93 18.23 ND 18.20
19.01 18.78 18.42 ND
17.91 18.64 18.77 18.42
18.68 19.08 19.04 18.94
18.75 18.77 19.11 18.98
Expression Level
19.00
18.75
18.50
18.25
18.00
Dominant Cell Type
a GE & S
a LE
a S
MaxQuantMBR LE Image
19.47 18.62 19.00 19.79
18.85 18.62 18.57 19.13
19.60 18.76 18.49 19.22
19.37 19.45 18.53 18.73
19.50 19.50 18.44 19.15
19.83 18.90 18.68 18.80
Expression Level
19.6
19.2
18.8
Dominant Cell Type
a GE & S
a LE
a S

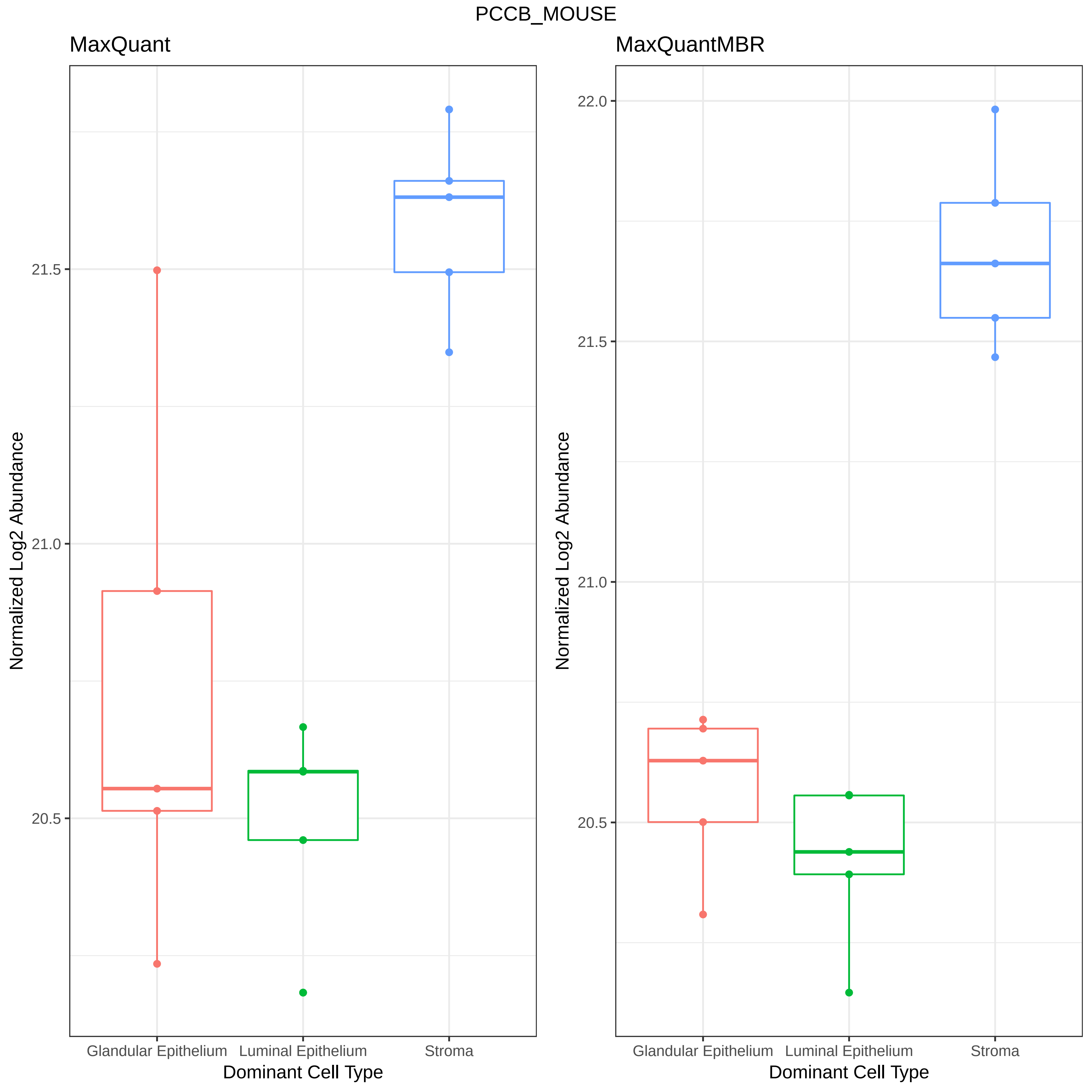
PCCB_MOUSE
MaxQuant
MaxQuantMBR
Normalized Log2 Abundance
21.5
21.0
20.5
22.0
21.5
21.0
20.5
Glandular Epithelium
Luminal Epithelium
Stroma
Glandular Epithelium
Luminal Epithelium
Stroma
Dominant Cell Type
Dominant Cell Type

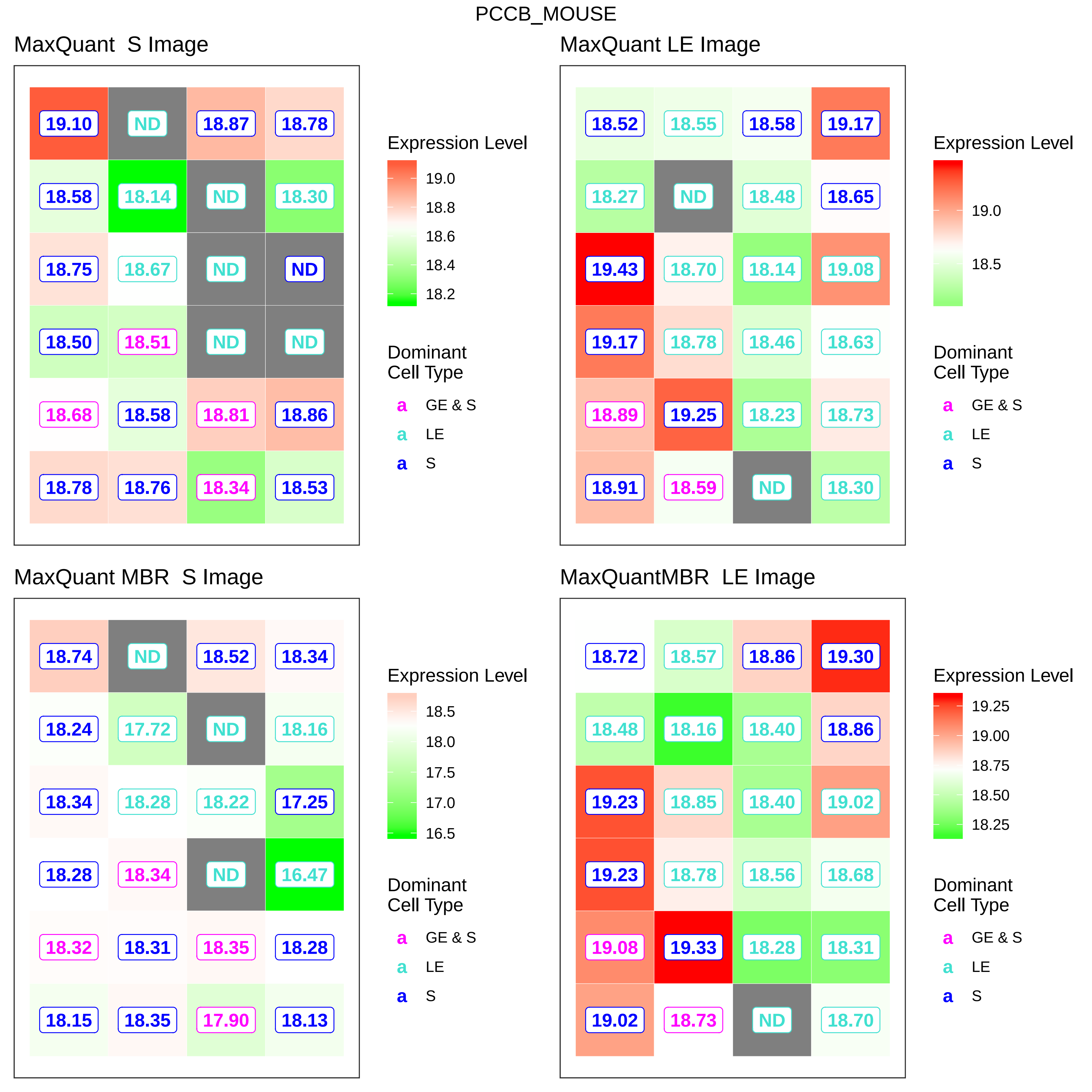
PCCB_MOUSE
MaxQuant S Image
19.10
ND
18.87
18.78
18.58
18.14
ND
18.30
18.75
18.67
ND
ND
18.50
18.51
ND
ND
18.68
18.58
18.81
18.86
18.78
18.76
18.34
18.53
Expression Level
19.0
18.8
18.6
18.4
18.2
Dominant Cell Type
a GE & S
a LE
a S
MaxQuant LE Image
18.52
18.55
18.58
19.17
18.27
ND
18.48
18.65
19.43
18.70
18.14
19.08
19.17
18.78
18.46
18.63
18.89
19.25
18.23
18.73
18.91
18.59
ND
18.30
Expression Level
19.0
18.5
Dominant Cell Type
a GE & S
a LE
a S
MaxQuant MBR S Image
18.74
ND
18.52
18.34
18.24
17.72
ND
18.16
18.34
18.28
18.22
17.25
18.28
18.34
ND
16.47
18.32
18.31
18.35
18.28
18.15
18.35
17.90
18.13
Expression Level
18.5
18.0
17.5
17.0
16.5
Dominant Cell Type
a GE & S
a LE
a S
MaxQuantMBR LE Image
18.72
18.57
18.86
19.30
18.48
18.16
18.40
18.86
19.23
18.85
18.40
19.02
19.23
18.78
18.56
18.68
19.08
19.33
18.28
18.31
19.02
18.73
ND
18.70
Expression Level
19.25
19.00
18.75
18.50
18.25
Dominant Cell Type
a GE & S
a LE
a S

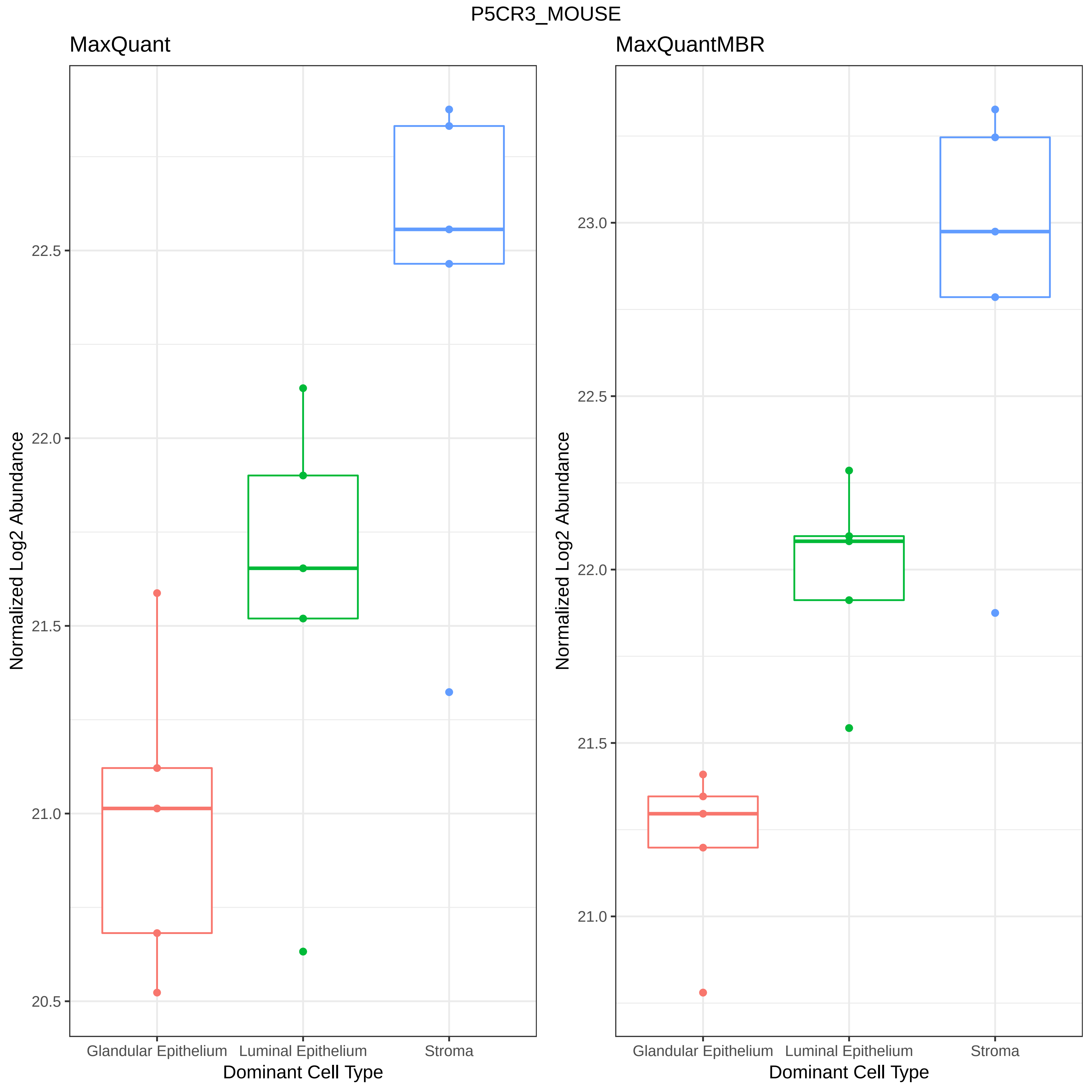

P5CR3_MOUSE
MaxQuant
MaxQuantMBR
Normalized Log2 Abundance
22.5
22.0
21.5
21.0
20.5
23.0
22.5
22.0
21.5
21.0
Glandular Epithelium
Luminal Epithelium
Stroma
Dominant Cell Type

P5CR3_MOUSE

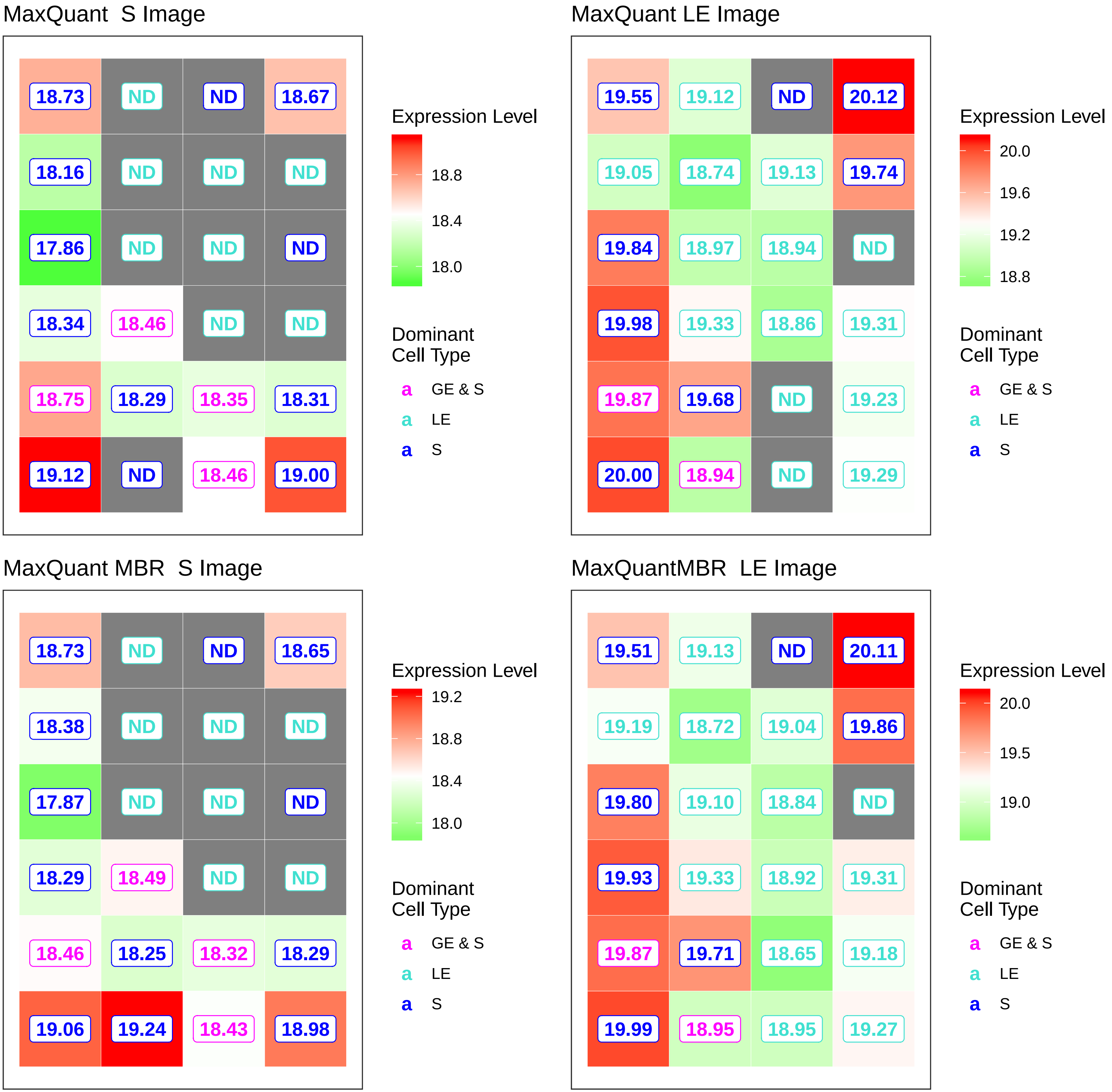
MaxQuant S Image
18.73 ND ND 18.67
18.16 ND ND ND
17.86 ND ND ND
18.34 18.46 ND ND
18.75 18.29 18.35 18.31
19.12 ND 18.46 19.00
Expression Level
18.8
18.4
18.0
Dominant Cell Type
a GE & S
a LE
a S
MaxQuant LE Image
19.55 19.12 ND 20.12
19.05 18.74 19.13 19.74
19.84 18.97 18.94 ND
19.98 19.33 18.86 19.31
19.87 19.68 ND 19.23
20.00 18.94 ND 19.29
Expression Level
20.0
19.6
19.2
18.8
Dominant Cell Type
a GE & S
a LE
a S
MaxQuant MBR S Image
18.73 ND ND 18.65
18.38 ND ND ND
17.87 ND ND ND
18.29 18.49 ND ND
18.46 18.25 18.32 18.29
19.06 19.24 18.43 18.98
Expression Level
19.2
18.8
18.4
18.0
Dominant Cell Type
a GE & S
a LE
a S
MaxQuantMBR LE Image
19.51 19.13 ND 20.11
19.19 18.72 19.04 19.86
19.80 19.10 18.84 ND
19.93 19.33 18.92 19.31
19.87 19.71 18.65 19.18
19.99 18.95 18.95 19.27
Expression Level
20.0
19.5
19.0
Dominant Cell Type
a GE & S
a LE
a S

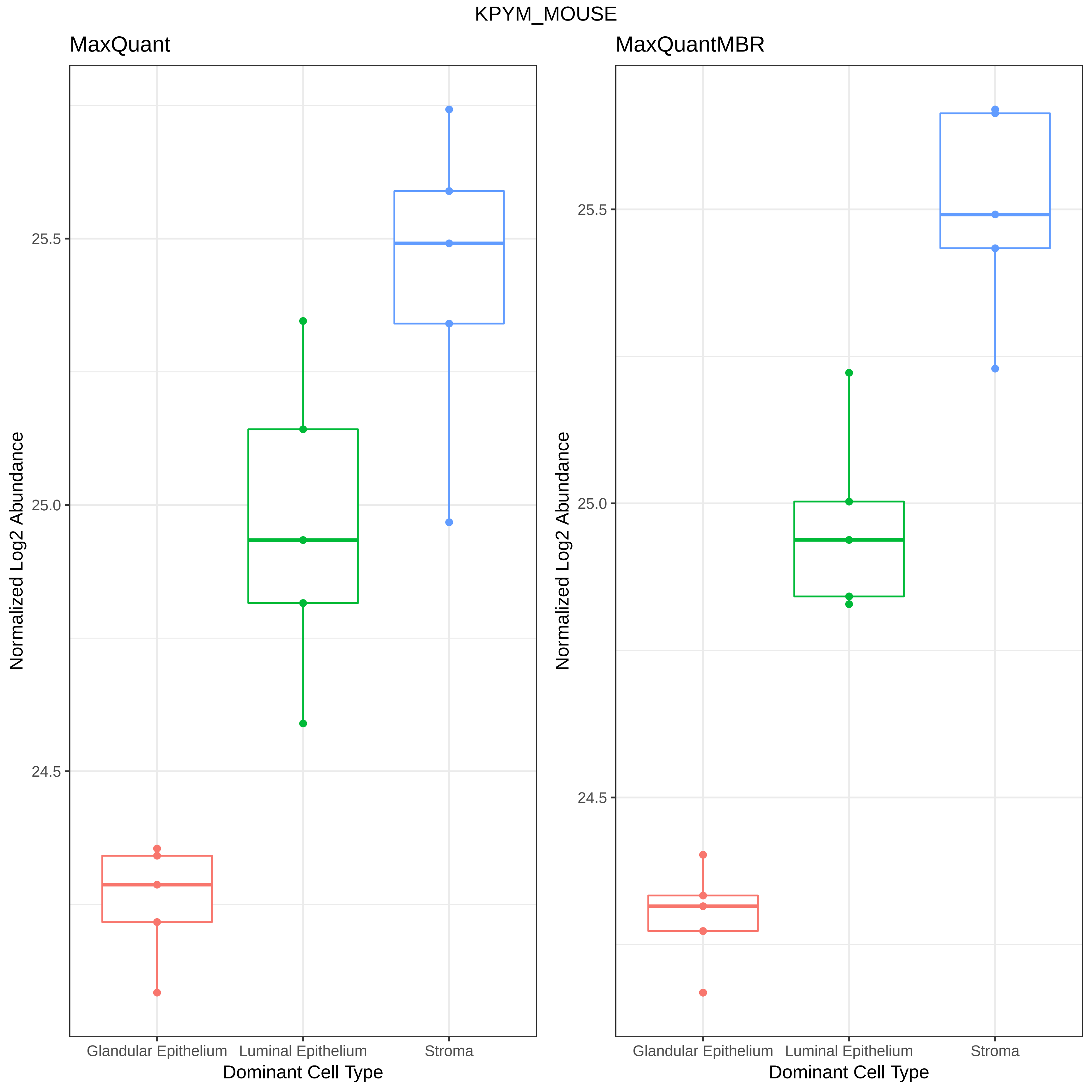
KPYM_MOUSE
MaxQuant
MaxQuantMBR
Normalized Log2 Abundance
25.5
25.0
24.5
Glandular Epithelium
Luminal Epithelium
Stroma
Dominant Cell Type

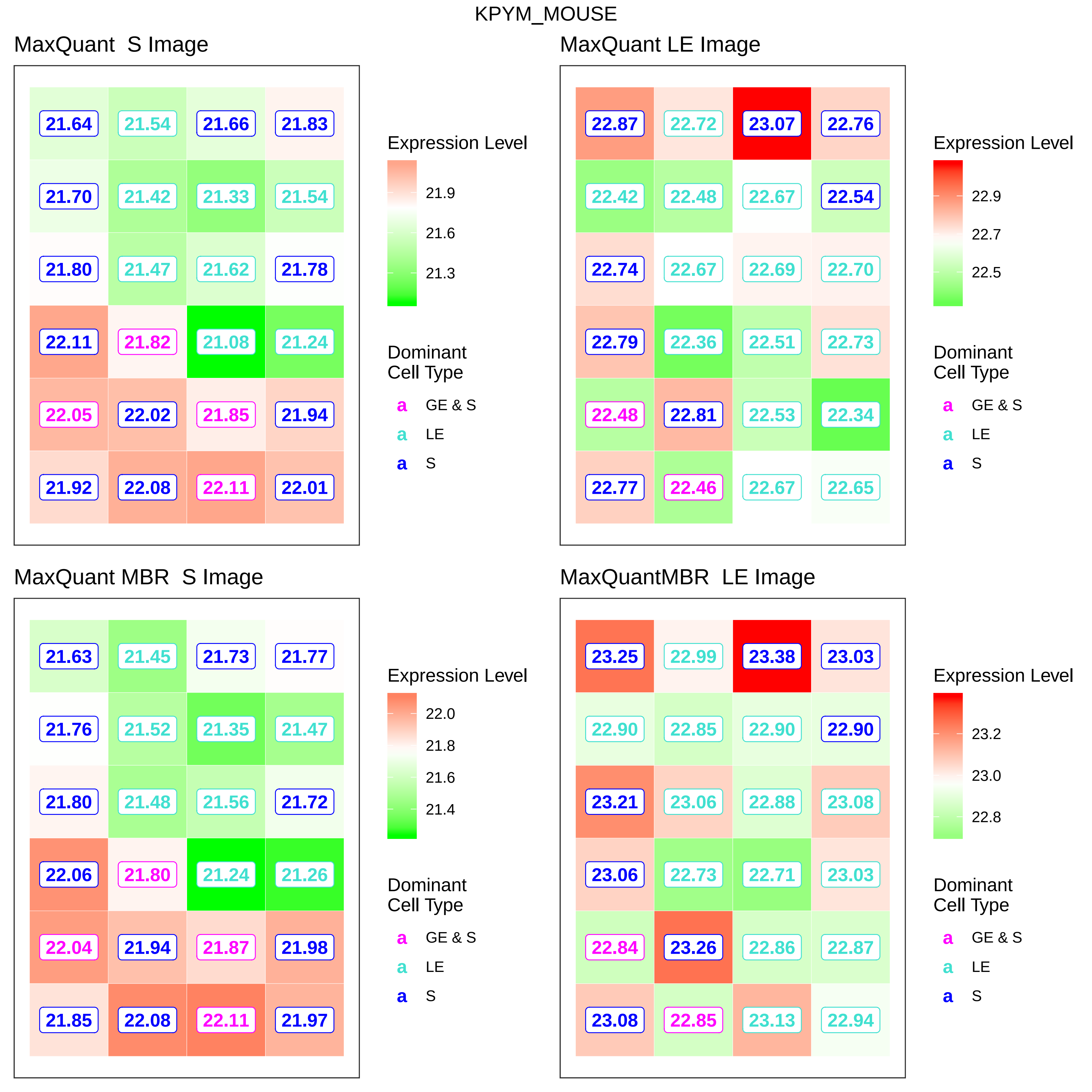
KPYM_MOUSE
MaxQuant S Image
21.64 21.54 21.66 21.83
21.70 21.42 21.33 21.54
21.80 21.47 21.62 21.78
22.11 21.82 21.08 21.24
22.05 22.02 21.85 21.94
21.92 22.08 22.11 22.01
Expression Level
21.9
21.6
21.3
Dominant Cell Type
a GE & S
a LE
a S
MaxQuant LE Image
22.87 22.72 23.07 22.76
22.42 22.48 22.67 22.54
22.74 22.67 22.69 22.70
22.79 22.36 22.51 22.73
22.48 22.81 22.53 22.34
22.77 22.46 22.67 22.65
Expression Level
22.9
22.7
22.5
Dominant Cell Type
a GE & S
a LE
a S
MaxQuant MBR S Image
21.63 21.45 21.73 21.77
21.76 21.52 21.35 21.47
21.80 21.48 21.56 21.72
22.06 21.80 21.24 21.26
22.04 21.94 21.87 21.98
21.85 22.08 22.11 21.97
Expression Level
22.0
21.8
21.6
21.4
Dominant Cell Type
a GE & S
a LE
a S
MaxQuantMBR LE Image
23.25 22.99 23.38 23.03
22.90 22.85 22.90 22.90
23.21 23.06 22.88 23.08
23.06 22.73 22.71 23.03
22.84 23.26 22.86 22.87
23.08 22.85 23.13 22.94
Expression Level
23.2
23.0
22.8
Dominant Cell Type
a GE & S
a LE
a S

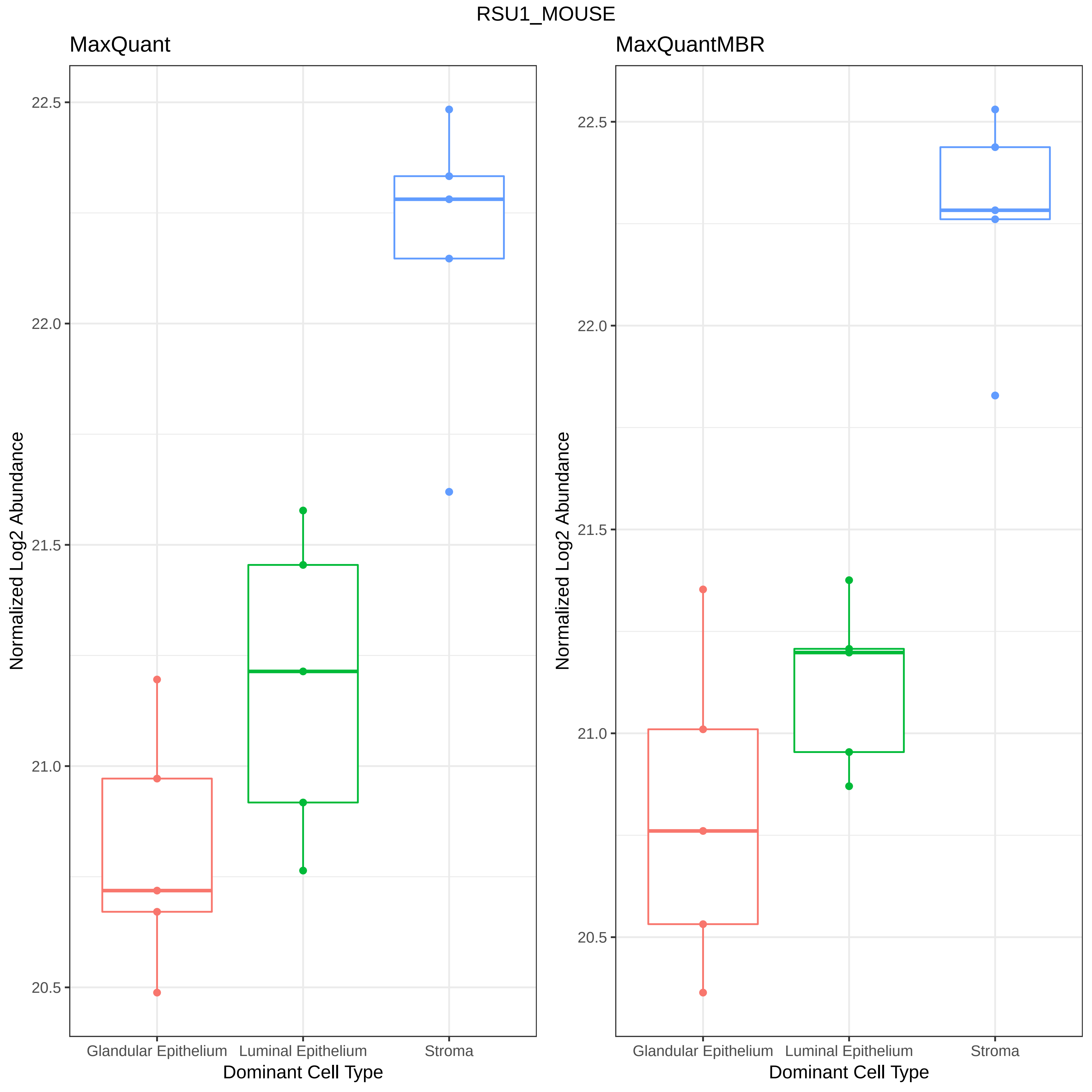

RSU1_MOUSE
MaxQuant
MaxQuantMBR
Normalized Log2 Abundance
22.5
22.0
21.5
21.0
20.5
Glandular Epithelium
Luminal Epithelium
Stroma
Dominant Cell Type

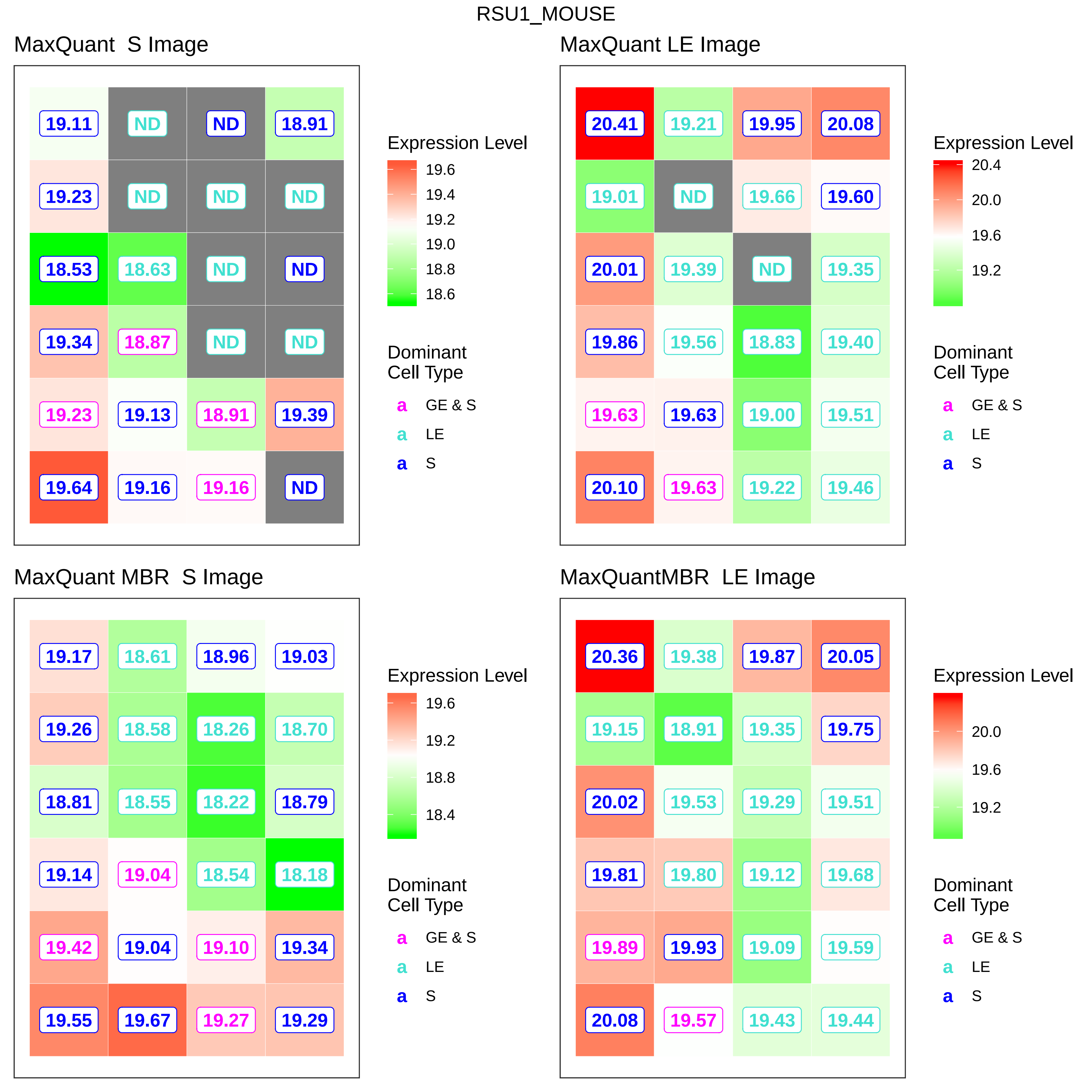

RSU1_MOUSE
MaxQuant S Image
19.11 ND ND 18.91
19.23 ND ND ND
18.53 18.63 ND ND
19.34 18.87 ND ND
19.23 19.13 18.91 19.39
19.64 19.16 19.16 ND
Expression Level
19.6
19.4
19.2
19.0
18.8
18.6
Dominant Cell Type
a GE & S
a LE
a S
MaxQuant LE Image
20.41 19.21 19.95 20.08
19.01 ND 19.66 19.60
20.01 19.39 ND 19.35
19.86 19.56 18.83 19.40
19.63 19.63 19.00 19.51
20.10 19.63 19.22 19.46
Expression Level
20.4
20.0
19.6
19.2
Dominant Cell Type
a GE & S
a LE
a S
MaxQuant MBR S Image
19.17 18.61 18.96 19.03
19.26 18.58 18.26 18.70
18.81 18.55 18.22 18.79
19.14 19.04 18.54 18.18
19.42 19.04 19.10 19.34
19.55 19.67 19.27 19.29
Expression Level
19.6
19.2
18.8
18.4
Dominant Cell Type
a GE & S
a LE
a S
MaxQuantMBR LE Image
20.36 19.38 19.87 20.05
19.15 18.91 19.35 19.75
20.02 19.53 19.29 19.51
19.81 19.80 19.12 19.68
19.89 19.93 19.09 19.59
20.08 19.57 19.43 19.44
Expression Level
20.0
19.6
19.2
Dominant Cell Type
a GE & S
a LE
a S

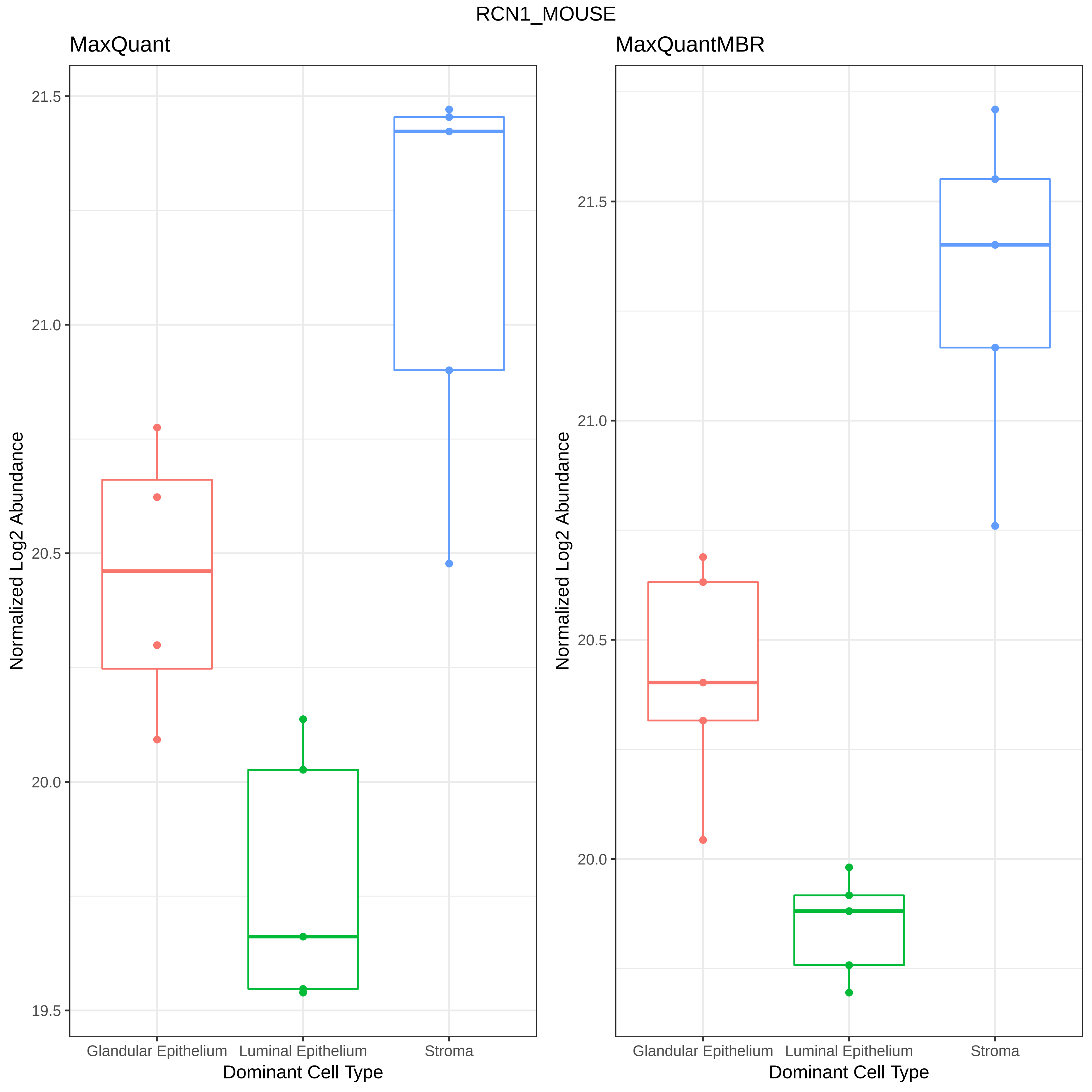
RCN1_MOUSE
MaxQuant
MaxQuantMBR
Normalized Log2 Abundance
21.5
21.0
20.5
20.0
19.5
Glandular Epithelium
Luminal Epithelium
Stroma
Dominant Cell Type

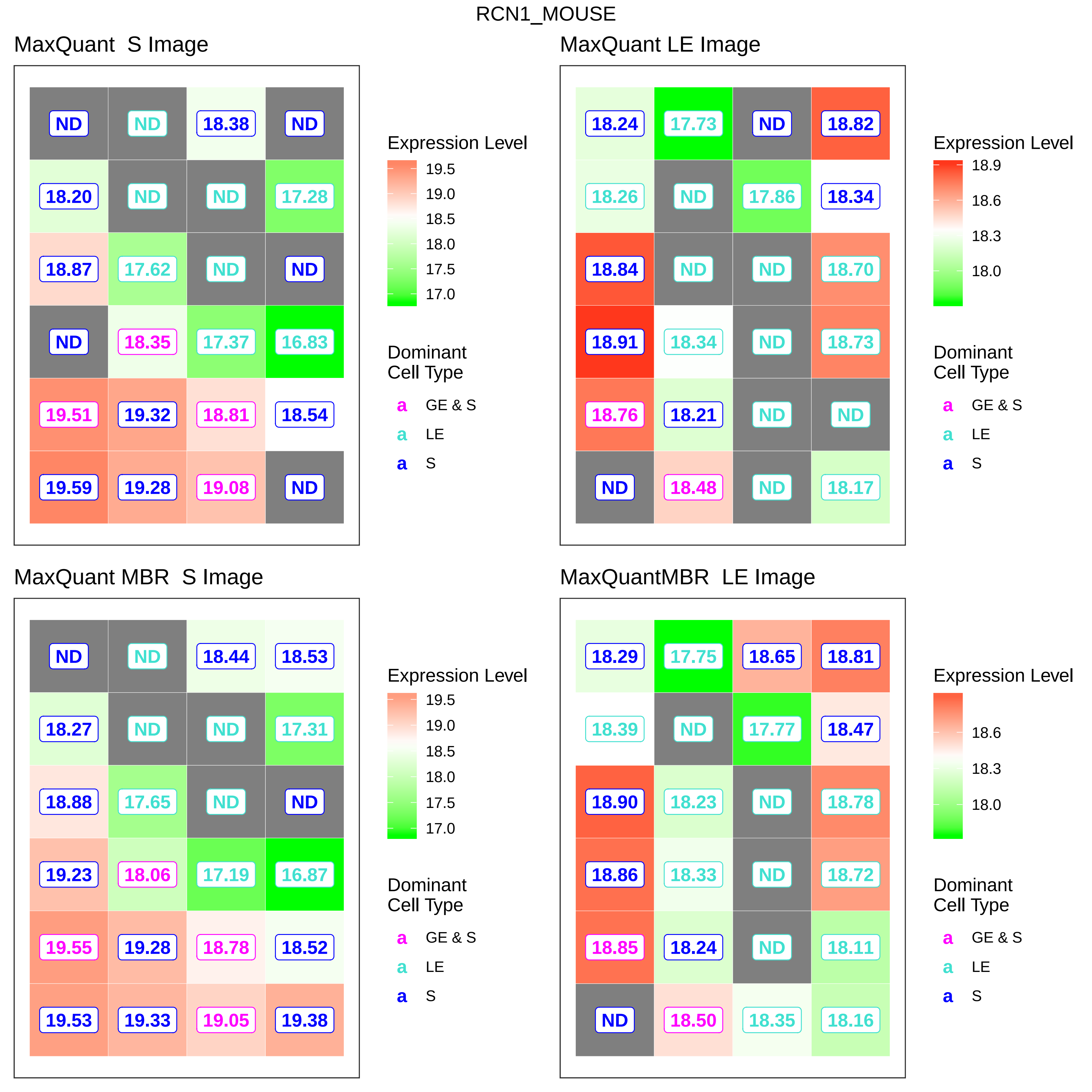

RCN1_MOUSE
MaxQuant S Image
ND
ND
18.38
ND
18.20
ND
ND
17.28
18.87
17.62
ND
ND
ND
18.35
17.37
16.83
19.51
19.32
18.81
18.54
19.59
19.28
19.08
ND
Expression Level
19.5
19.0
18.5
18.0
17.5
17.0
Dominant Cell Type
a GE & S
a LE
a S
MaxQuant LE Image
18.24
17.73
ND
18.82
18.26
ND
17.86
18.34
18.84
ND
ND
18.70
18.91
18.34
ND
18.73
18.76
18.21
ND
ND
ND
18.48
ND
18.17
Expression Level
18.9
18.6
18.3
18.0
Dominant Cell Type
a GE & S
a LE
a S
MaxQuant MBR S Image
ND
ND
18.44
18.53
18.27
ND
ND
17.31
18.88
17.65
ND
ND
19.23
18.06
17.19
16.87
19.55
19.28
18.78
18.52
19.53
19.33
19.05
19.38
Expression Level
19.5
19.0
18.5
18.0
17.5
17.0
Dominant Cell Type
a GE & S
a LE
a S
MaxQuantMBR LE Image
18.29
17.75
18.65
18.81
18.39
ND
17.77
18.47
18.90
18.23
ND
18.78
18.86
18.33
ND
18.72
18.85
18.24
ND
18.11
ND
18.50
18.35
18.16
Expression Level
18.6
18.3
18.0
Dominant Cell Type
a GE & S
a LE
a S

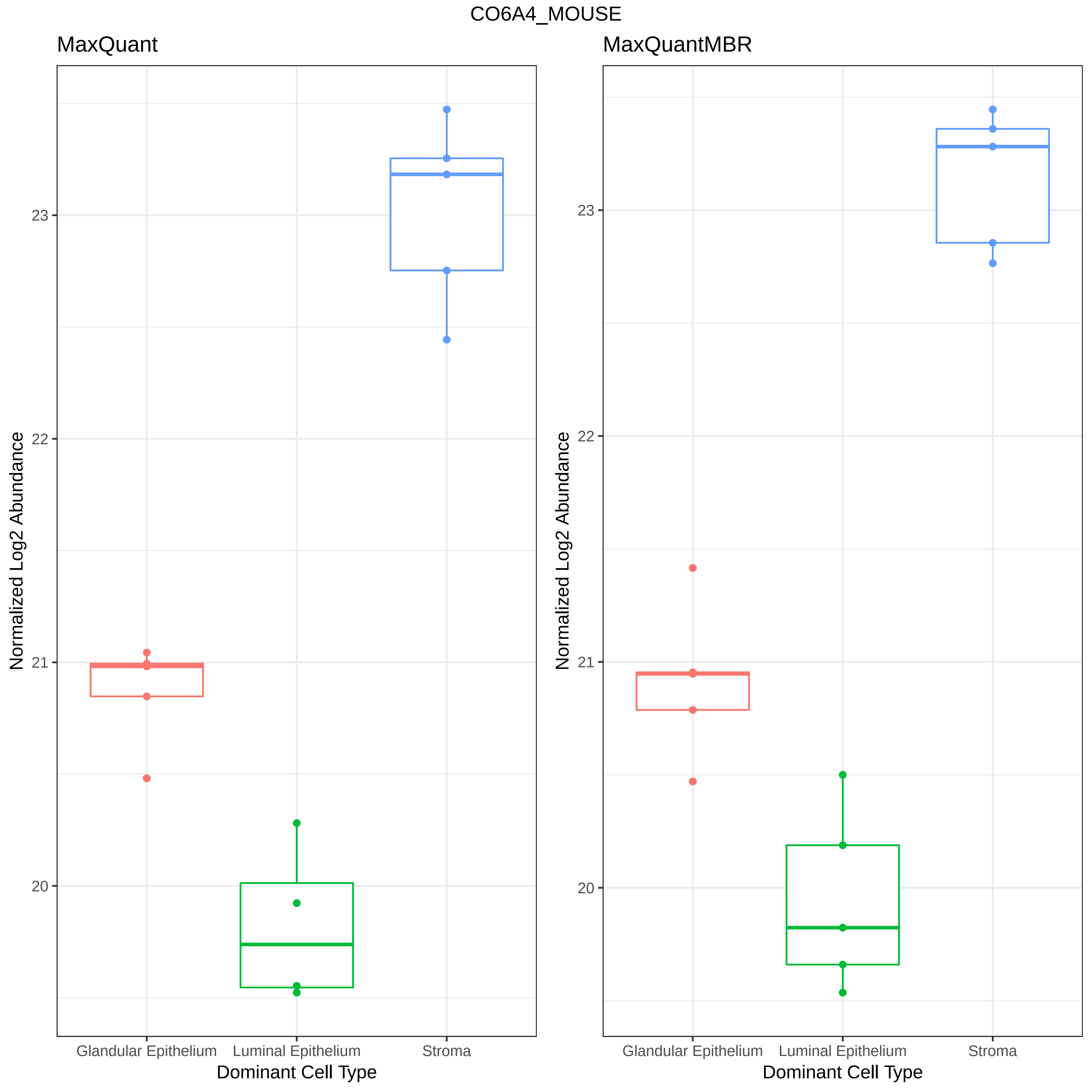

CO6A4_MOUSE
MaxQuant
MaxQuantMBR
Normalized Log2 Abundance
23
22
21
20
Glandular Epithelium
Luminal Epithelium
Stroma
Dominant Cell Type

CO6A4_MOUSE

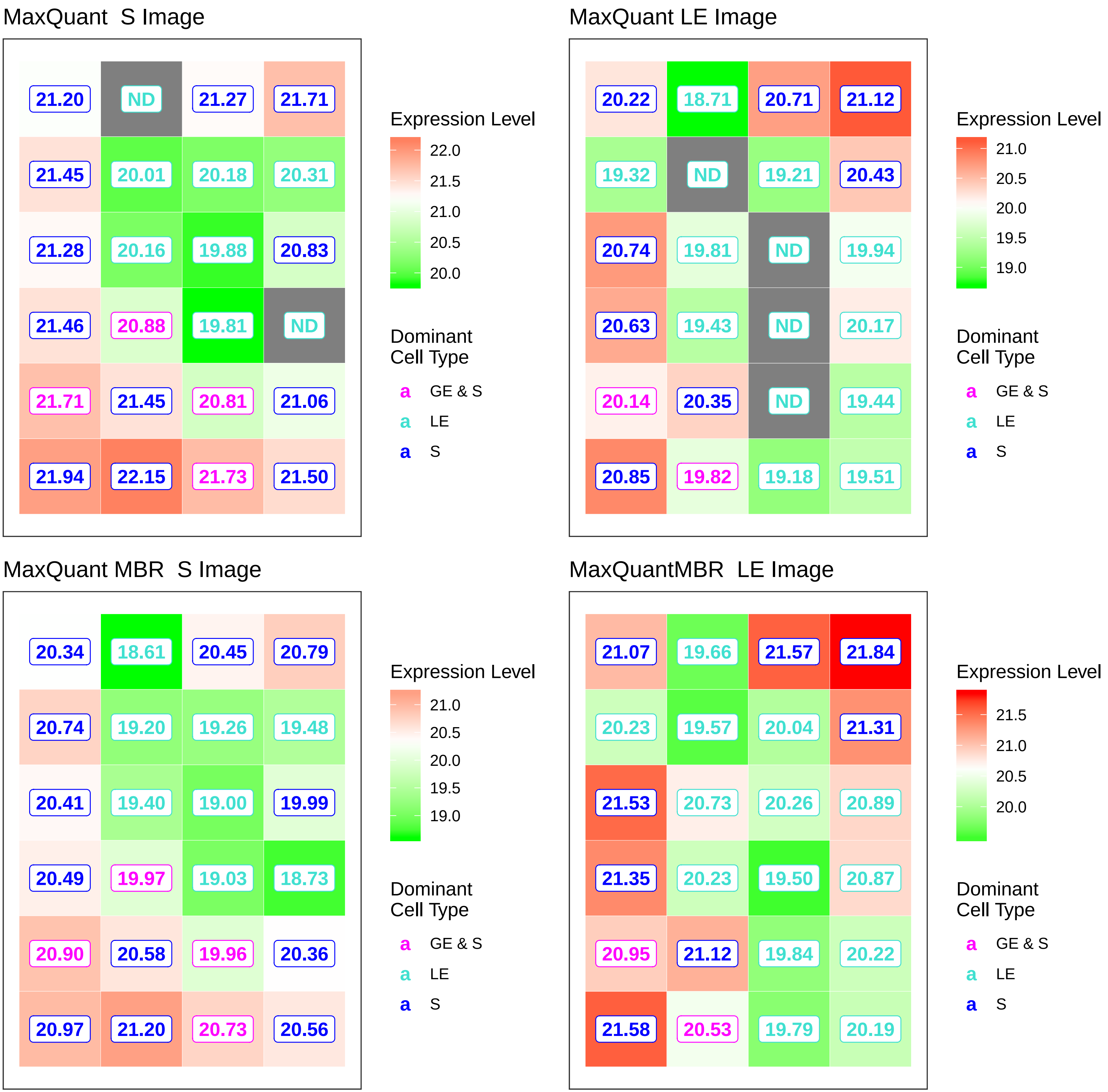
MaxQuant S Image
21.20 ND 21.27 21.71
21.45 20.01 20.18 20.31
21.28 20.16 19.88 20.83
21.46 20.88 19.81 ND
21.71 21.45 20.81 21.06
21.94 22.15 21.73 21.50
Expression Level
22.0
21.5
21.0
20.5
20.0
Dominant Cell Type
a GE & S
a LE
a S
MaxQuant LE Image
20.22 18.71 20.71 21.12
19.32 ND 19.21 20.43
20.74 19.81 ND 19.94
20.63 19.43 ND 20.17
20.14 20.35 ND 19.44
20.85 19.82 19.18 19.51
Expression Level
21.0
20.5
20.0
19.5
19.0
Dominant Cell Type
a GE & S
a LE
a S
MaxQuant MBR S Image
20.34 18.61 20.45 20.79
20.74 19.20 19.26 19.48
20.41 19.40 19.00 19.99
20.49 19.97 19.03 18.73
20.90 20.58 19.96 20.36
20.97 21.20 20.73 20.56
Expression Level
21.0
20.5
20.0
19.5
19.0
Dominant Cell Type
a GE & S
a LE
a S
MaxQuantMBR LE Image
21.07 19.66 21.57 21.84
20.23 19.57 20.04 21.31
21.53 20.73 20.26 20.89
21.35 20.23 19.50 20.87
20.95 21.12 19.84 20.22
21.58 20.53 19.79 20.19
Expression Level
21.5
21.0
20.5
20.0
Dominant Cell Type
a GE & S
a LE
a S

CO6A6_MOUSE

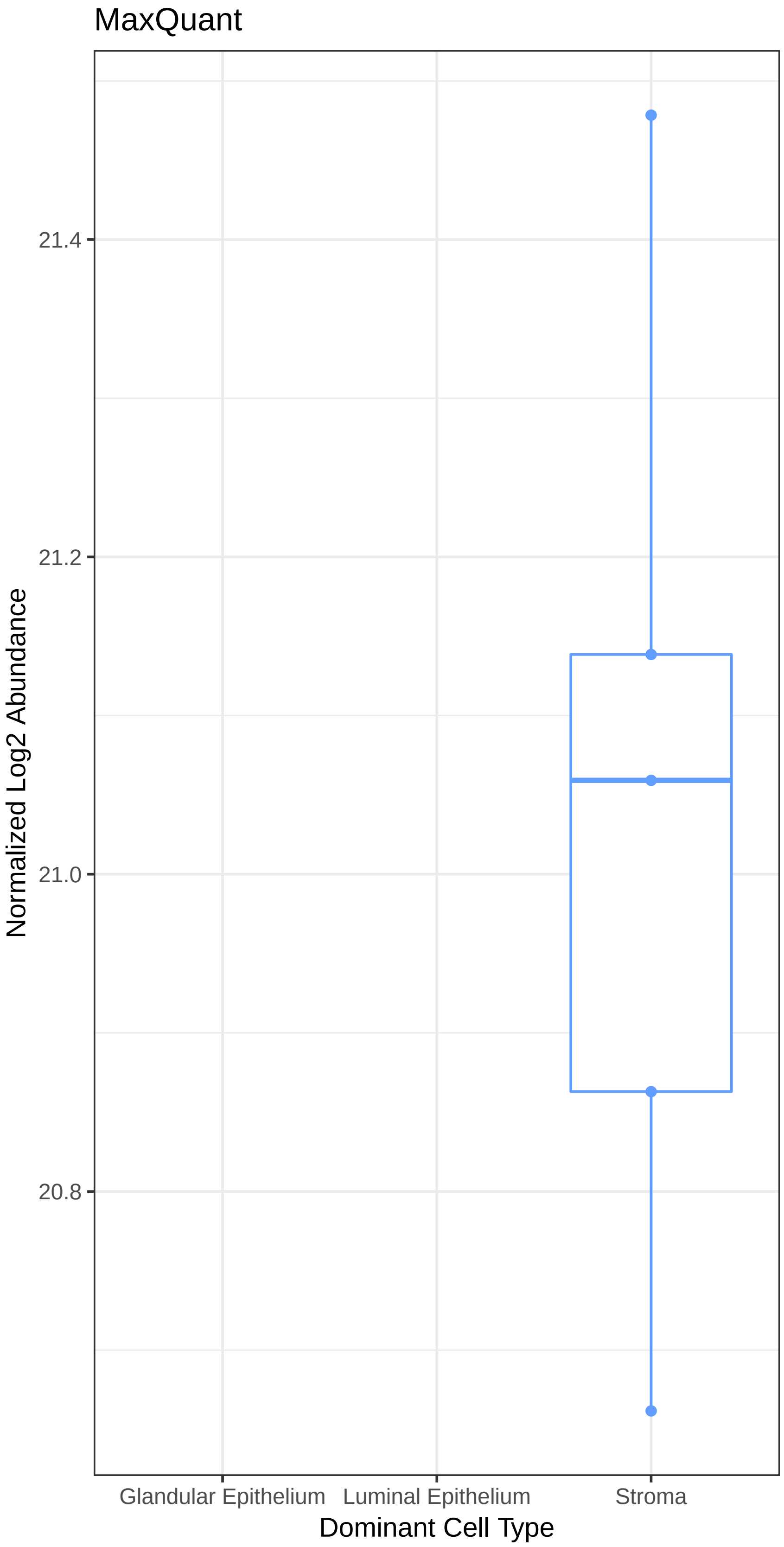

MaxQuant
Normalized Log2 Abundance
21.4
21.2
21.0
20.8
Glandular Epithelium
Luminal Epithelium
Stroma
Dominant Cell Type

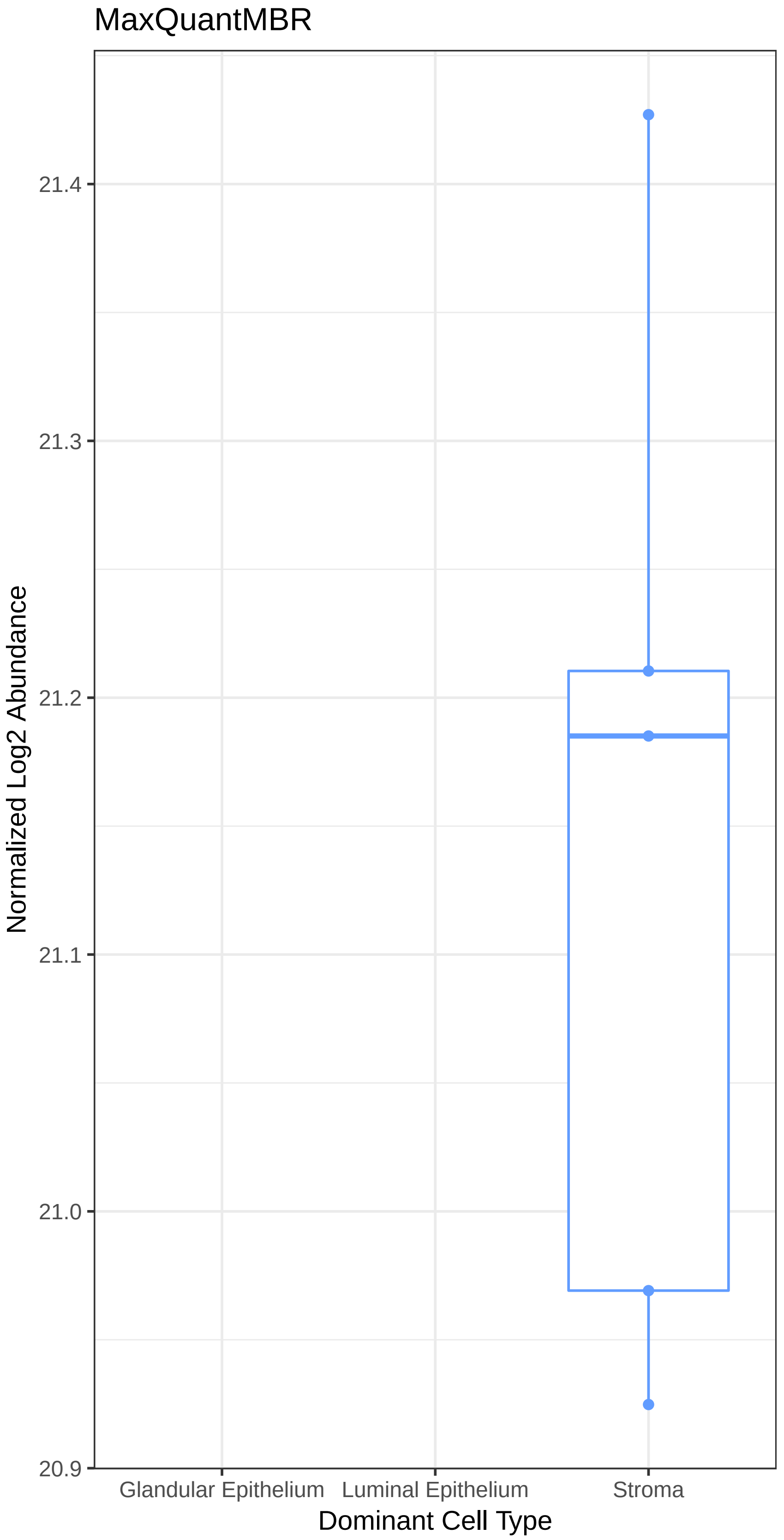

MaxQuantMBR
Normalized Log2 Abundance
21.4
21.3
21.2
21.1
21.0
20.9
Glandular Epithelium
Luminal Epithelium
Stroma
Dominant Cell Type

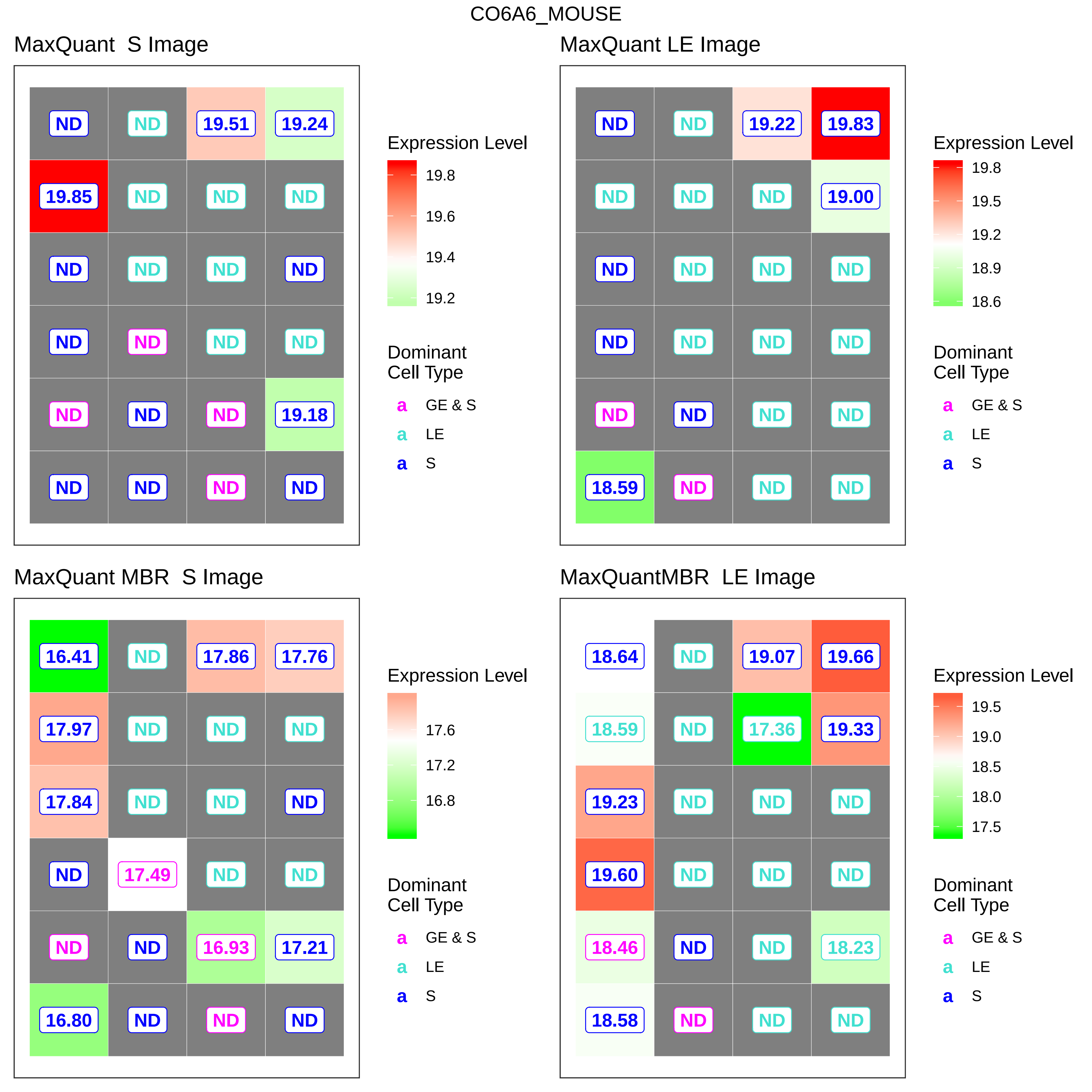
CO6A6_MOUSE
MaxQuant S Image
ND ND 19.51 19.24
19.85 ND ND ND
ND ND ND ND
ND ND ND ND
ND ND ND 19.18
ND ND ND ND
Expression Level
19.8
19.6
19.4
19.2
Dominant Cell Type
a GE & S
a LE
a S
MaxQuant LE Image
ND ND 19.22 19.83
ND ND ND 19.00
ND ND ND ND
ND ND ND ND
ND ND ND ND
18.59 ND ND ND
Expression Level
19.8
19.5
19.2
18.9
18.6
Dominant Cell Type
a GE & S
a LE
a S
MaxQuant MBR S Image
16.41 ND 17.86 17.76
17.97 ND ND ND
17.84 ND ND ND
ND 17.49 ND ND
ND ND 16.93 17.21
16.80 ND ND ND
Expression Level
17.6
17.2
16.8
Dominant Cell Type
a GE & S
a LE
a S
MaxQuantMBR LE Image
18.64 ND 19.07 19.66
18.59 ND 17.36 19.33
19.23 ND ND ND
19.60 ND ND ND
18.46 ND ND 18.23
18.58 ND ND ND
Expression Level
19.5
19.0
18.5
18.0
17.5
Dominant Cell Type
a GE & S
a LE
a S

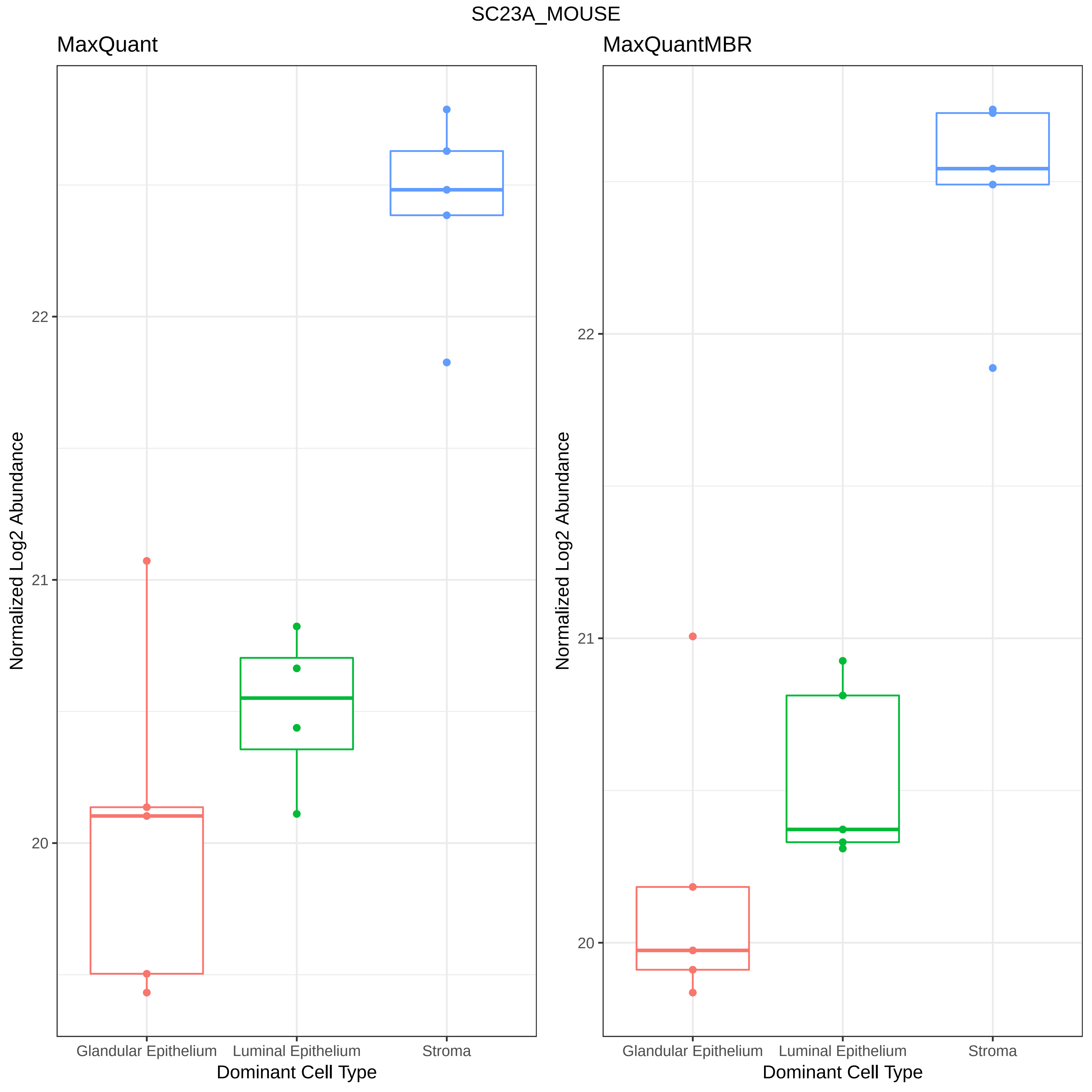

SC23A_MOUSE
MaxQuant
MaxQuantMBR
Normalized Log2 Abundance
22
21
20
Glandular Epithelium
Luminal Epithelium
Stroma
Dominant Cell Type

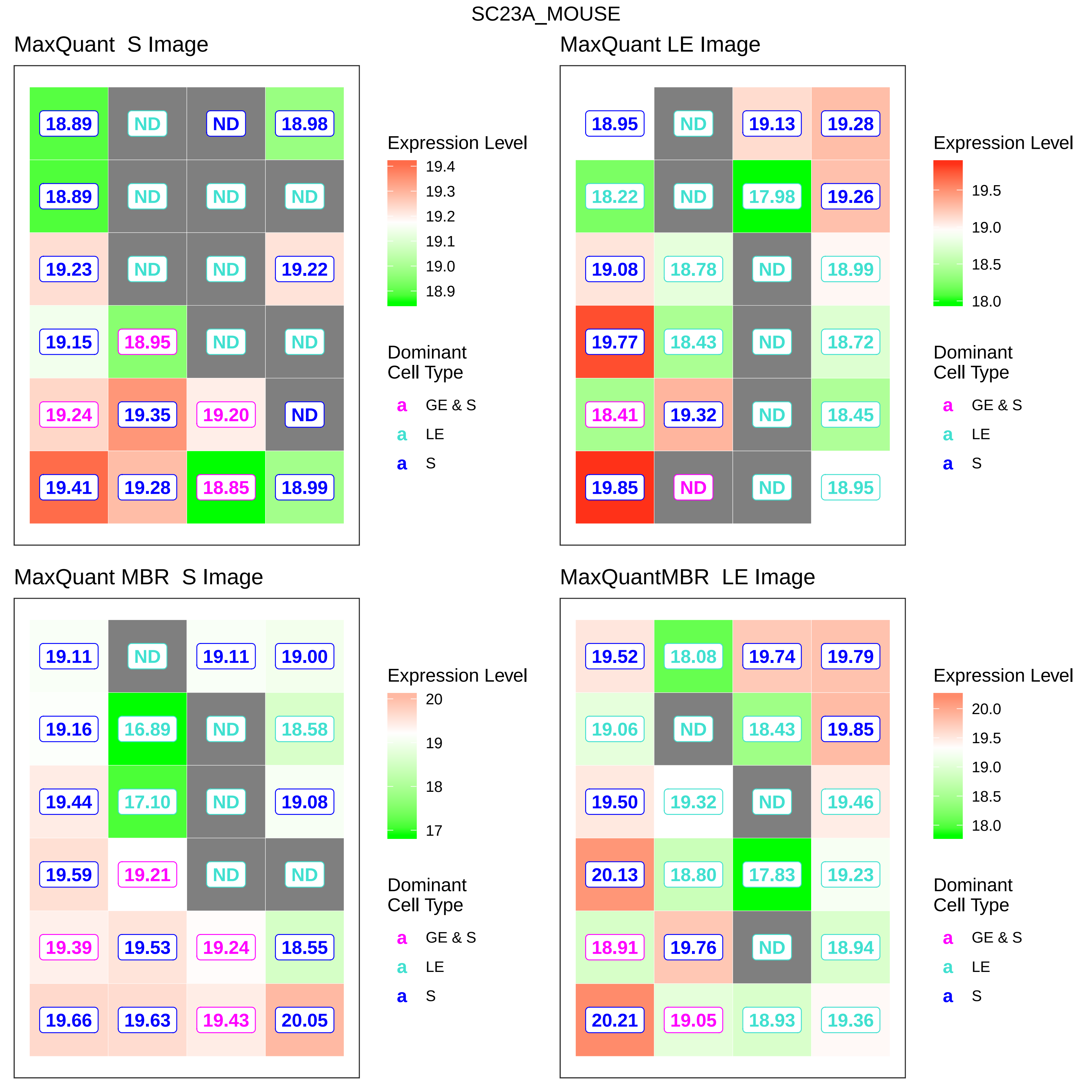

SC23A_MOUSE
MaxQuant S Image
18.89 ND ND 18.98
18.89 ND ND ND
19.23 ND ND 19.22
19.15 18.95 ND ND
19.24 19.35 19.20 ND
19.41 19.28 18.85 18.99
Expression Level
19.4
19.3
19.2
19.1
19.0
18.9
Dominant Cell Type
a GE & S
a LE
a S
MaxQuant LE Image
18.95 ND 19.13 19.28
18.22 ND 17.98 19.26
19.08 18.78 ND 18.99
19.77 18.43 ND 18.72
18.41 19.32 ND 18.45
19.85 ND ND 18.95
Expression Level
19.5
19.0
18.5
18.0
Dominant Cell Type
a GE & S
a LE
a S
MaxQuant MBR S Image
19.11 ND 19.11 19.00
19.16 16.89 ND 18.58
19.44 17.10 ND 19.08
19.59 19.21 ND ND
19.39 19.53 19.24 18.55
19.66 19.63 19.43 20.05
Expression Level
20
19
18
17
Dominant Cell Type
a GE & S
a LE
a S
MaxQuantMBR LE Image
19.52 18.08 19.74 19.79
19.06 ND 18.43 19.85
19.50 19.32 ND 19.46
20.13 18.80 17.83 19.23
18.91 19.76 ND 18.94
20.21 19.05 18.93 19.36
Expression Level
20.0
19.5
19.0
18.5
18.0
Dominant Cell Type
a GE & S
a LE
a S

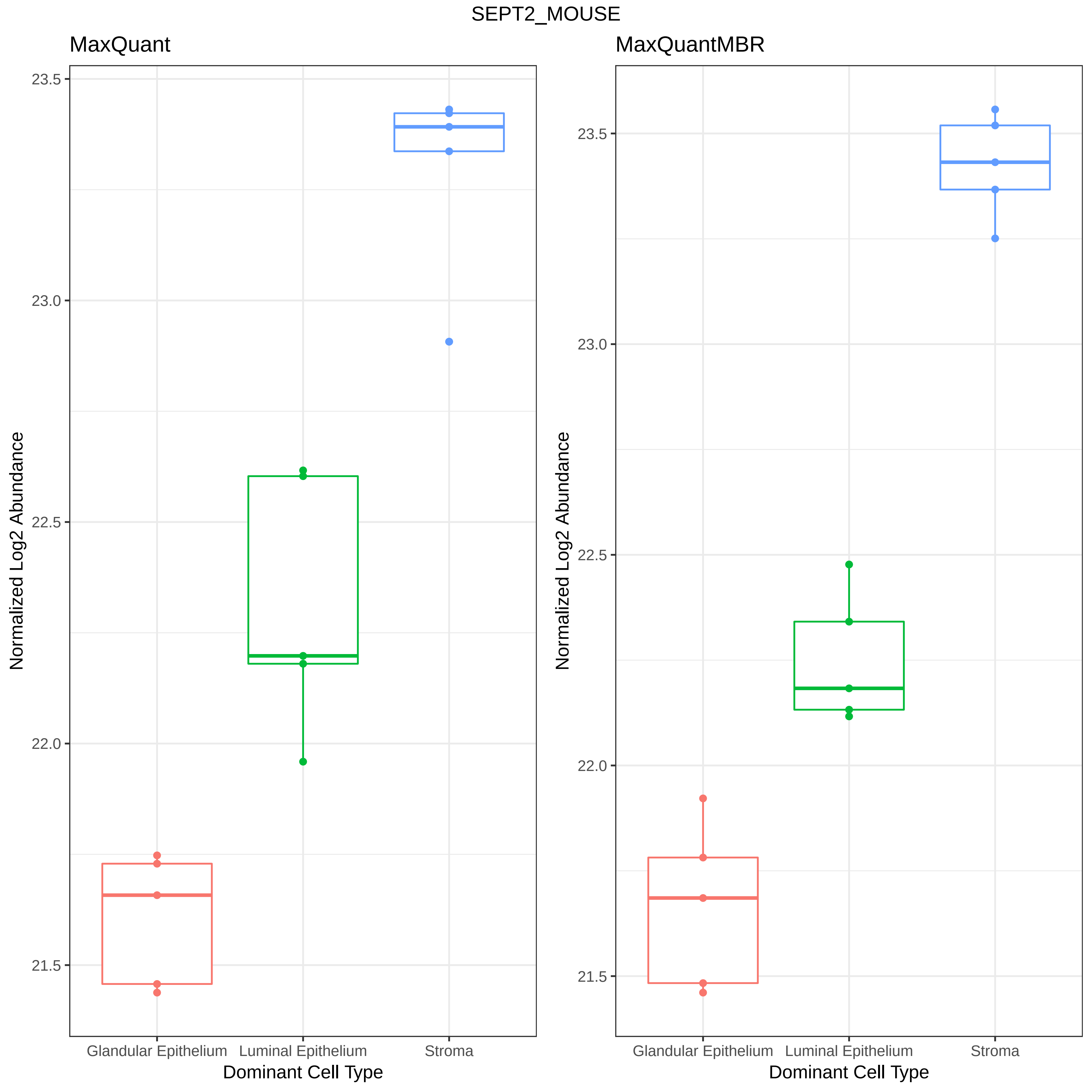

SEPT2_MOUSE
MaxQuant
MaxQuantMBR
Normalized Log2 Abundance
23.5
23.0
22.5
22.0
21.5
Glandular Epithelium
Luminal Epithelium
Stroma
Dominant Cell Type

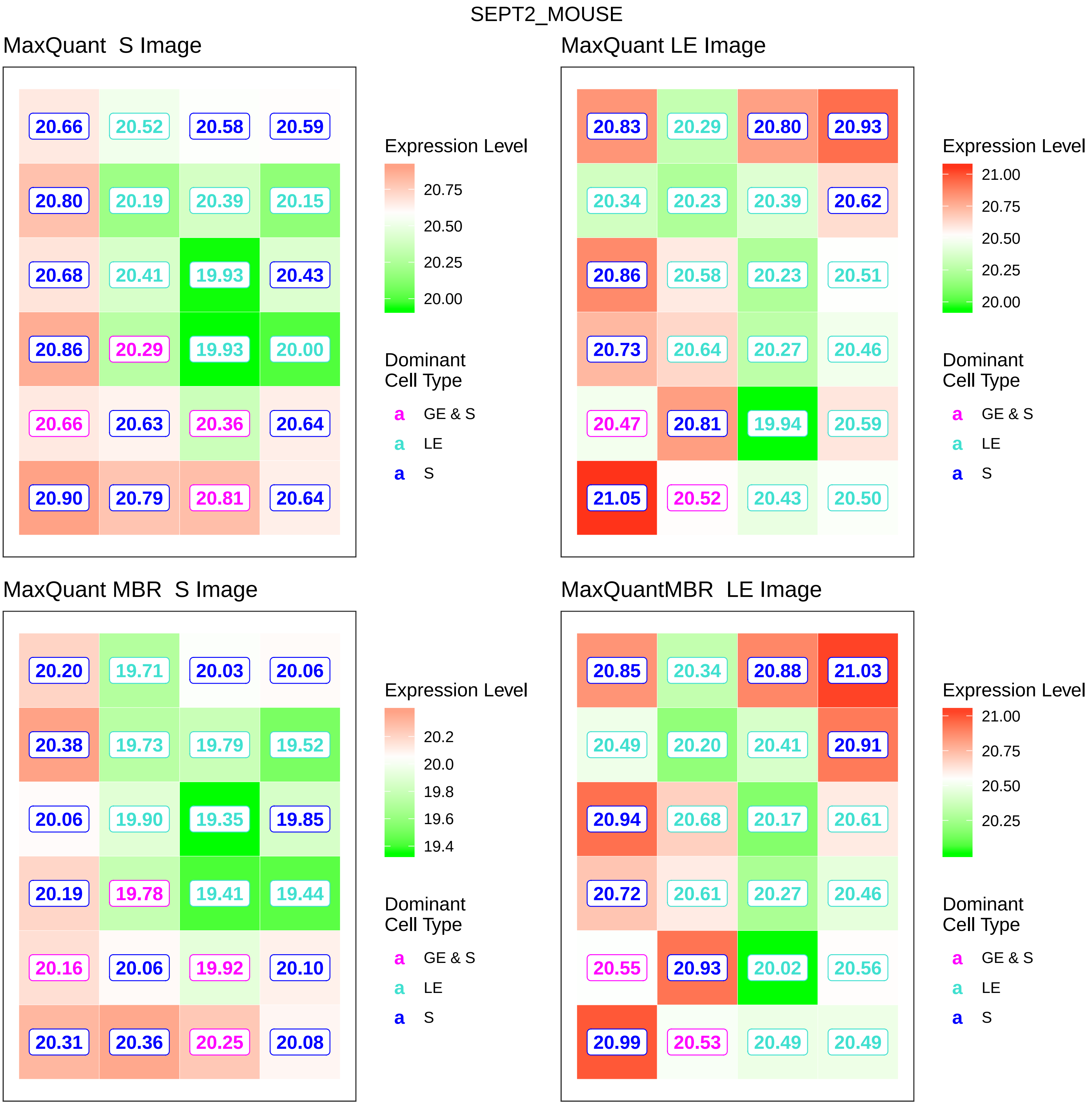
SEPT2_MOUSE
MaxQuant S Image
20.66 20.52 20.58 20.59
20.80 20.19 20.39 20.15
20.68 20.41 19.93 20.43
20.86 20.29 19.93 20.00
20.66 20.63 20.36 20.64
20.90 20.79 20.81 20.64
Expression Level
20.75
20.50
20.25
20.00
Dominant Cell Type
a GE & S
a LE
a S
MaxQuant LE Image
20.83 20.29 20.80 20.93
20.34 20.23 20.39 20.62
20.86 20.58 20.23 20.51
20.73 20.64 20.27 20.46
20.47 20.81 19.94 20.59
21.05 20.52 20.43 20.50
Expression Level
21.00
20.75
20.50
20.25
20.00
Dominant Cell Type
a GE & S
a LE
a S
MaxQuant MBR S Image
20.20 19.71 20.03 20.06
20.38 19.73 19.79 19.52
20.06 19.90 19.35 19.85
20.19 19.78 19.41 19.44
20.16 20.06 19.92 20.10
20.31 20.36 20.25 20.08
Expression Level
20.2
20.0
19.8
19.6
19.4
Dominant Cell Type
a GE & S
a LE
a S
MaxQuantMBR LE Image
20.85 20.34 20.88 21.03
20.49 20.20 20.41 20.91
20.94 20.68 20.17 20.61
20.72 20.61 20.27 20.46
20.55 20.93 20.02 20.56
20.99 20.53 20.49 20.49
Expression Level
21.00
20.75
20.50
20.25
Dominant Cell Type
a GE & S
a LE
a S

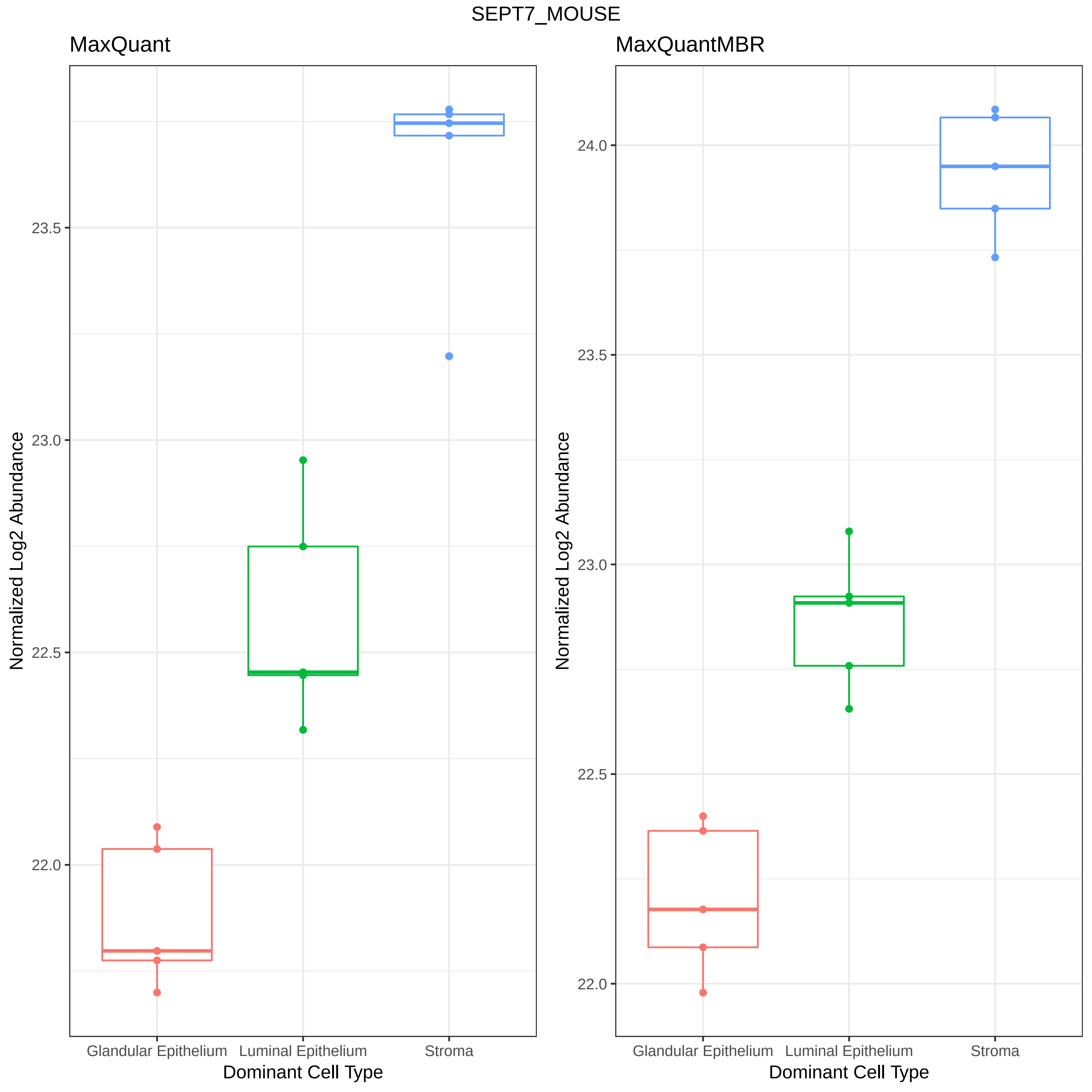

SEPT7_MOUSE
MaxQuant
MaxQuantMBR
Normalized Log2 Abundance
23.5
23.0
22.5
22.0
24.0
Glandular Epithelium
Luminal Epithelium
Stroma
Dominant Cell Type

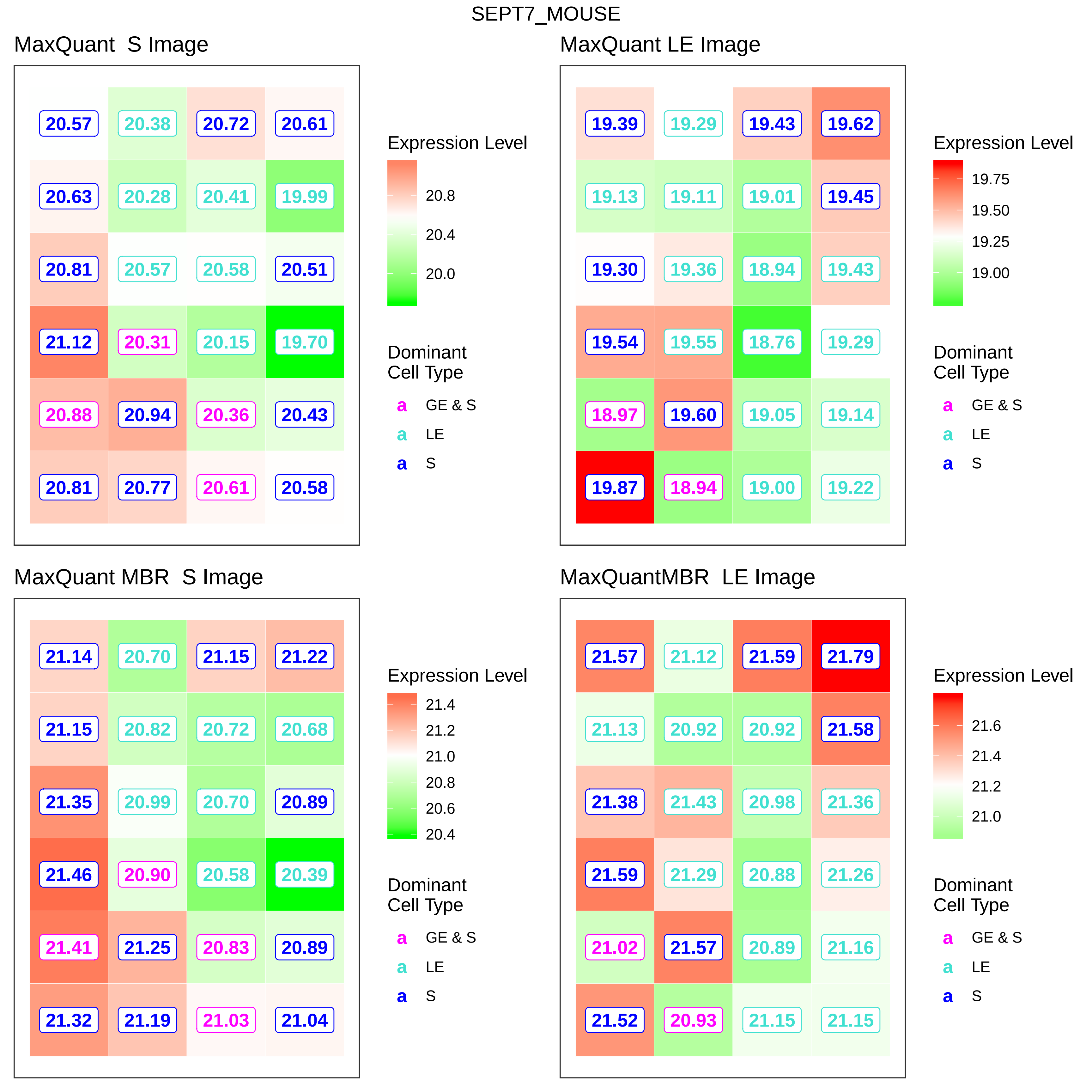
SEPT7_MOUSE
MaxQuant S Image
20.57 20.38 20.72 20.61
20.63 20.28 20.41 19.99
20.81 20.57 20.58 20.51
21.12 20.31 20.15 19.70
20.88 20.94 20.36 20.43
20.81 20.77 20.61 20.58
Expression Level
20.8
20.4
20.0
Dominant Cell Type
a GE & S
a LE
a S
MaxQuant LE Image
19.39 19.29 19.43 19.62
19.13 19.11 19.01 19.45
19.30 19.36 18.94 19.43
19.54 19.55 18.76 19.29
18.97 19.60 19.05 19.14
19.87 18.94 19.00 19.22
Expression Level
19.75
19.50
19.25
19.00
Dominant Cell Type
a GE & S
a LE
a S
MaxQuant MBR S Image
21.14 20.70 21.15 21.22
21.15 20.82 20.72 20.68
21.35 20.99 20.70 20.89
21.46 20.90 20.58 20.39
21.41 21.25 20.83 20.89
21.32 21.19 21.03 21.04
Expression Level
21.4
21.2
21.0
20.8
20.6
20.4
Dominant Cell Type
a GE & S
a LE
a S
MaxQuantMBR LE Image
21.57 21.12 21.59 21.79
21.13 20.92 20.92 21.58
21.38 21.43 20.98 21.36
21.59 21.29 20.88 21.26
21.02 21.57 20.89 21.16
21.52 20.93 21.15 21.15
Expression Level
21.6
21.4
21.2
21.0
Dominant Cell Type
a GE & S
a LE
a S

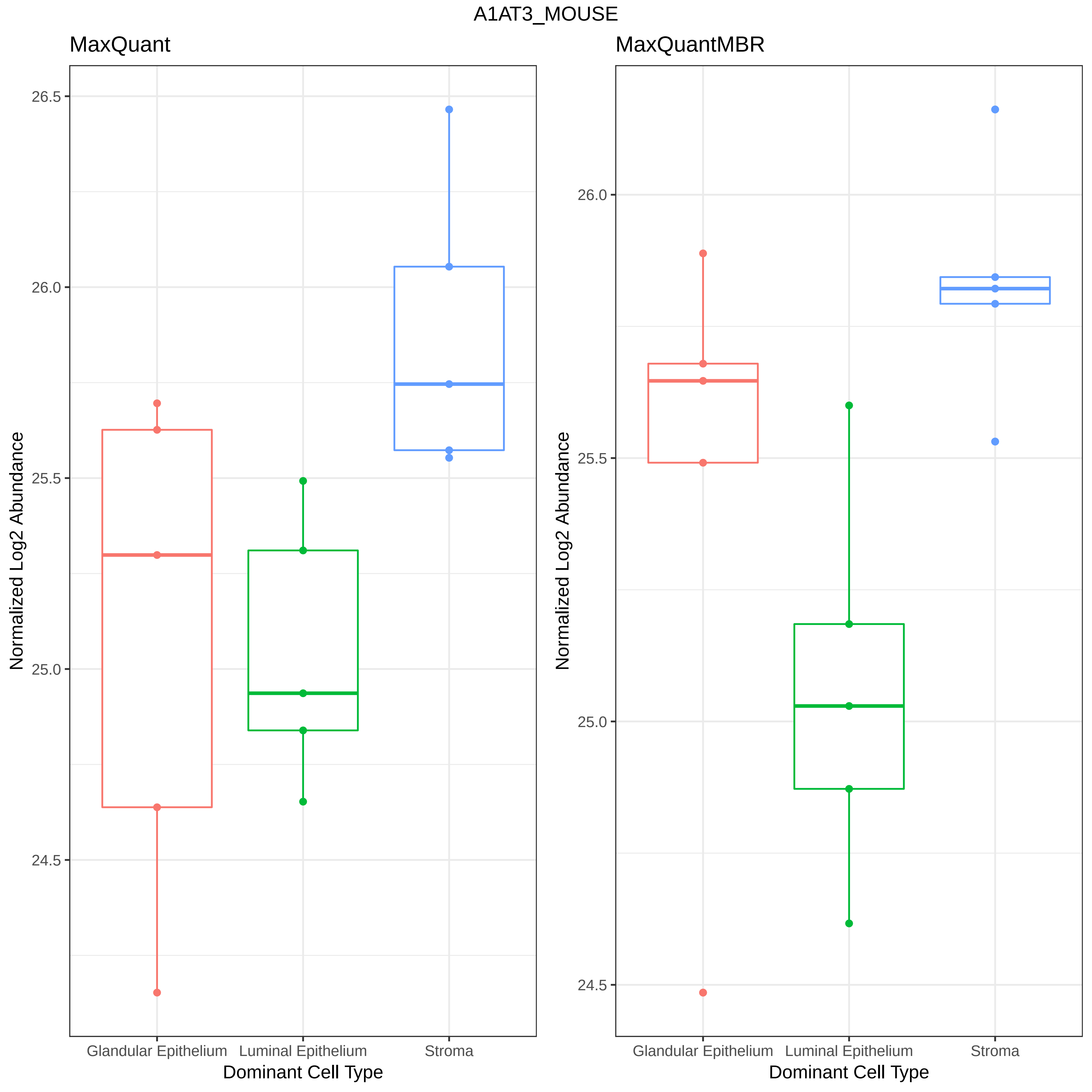

A1AT3_MOUSE
MaxQuant
MaxQuantMBR
Normalized Log2 Abundance
26.5
26.0
25.5
25.0
24.5
Glandular Epithelium
Luminal Epithelium
Stroma
Dominant Cell Type

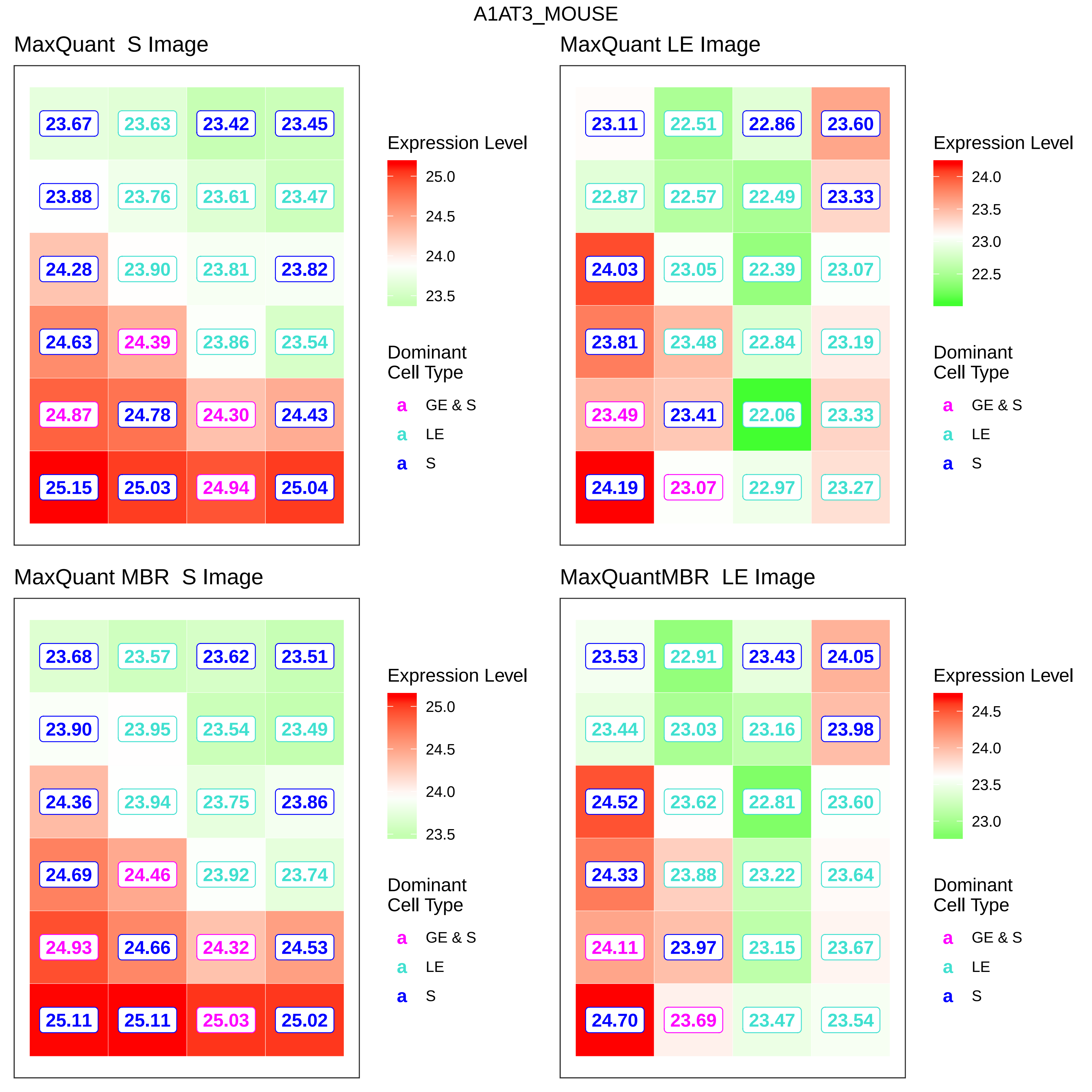

A1AT3_MOUSE
MaxQuant S Image
23.67 23.63 23.42 23.45
23.88 23.76 23.61 23.47
24.28 23.90 23.81 23.82
24.63 24.39 23.86 23.54
24.87 24.78 24.30 24.43
25.15 25.03 24.94 25.04
Expression Level
25.0
24.5
24.0
23.5
Dominant Cell Type
a GE & S
a LE
a S
MaxQuant LE Image
23.11 22.51 22.86 23.60
22.87 22.57 22.49 23.33
24.03 23.05 22.39 23.07
23.81 23.48 22.84 23.19
23.49 23.41 22.06 23.33
24.19 23.07 22.97 23.27
Expression Level
24.0
23.5
23.0
22.5
Dominant Cell Type
a GE & S
a LE
a S
MaxQuant MBR S Image
23.68 23.57 23.62 23.51
23.90 23.95 23.54 23.49
24.36 23.94 23.75 23.86
24.69 24.46 23.92 23.74
24.93 24.66 24.32 24.53
25.11 25.11 25.03 25.02
Expression Level
25.0
24.5
24.0
23.5
Dominant Cell Type
a GE & S
a LE
a S
MaxQuantMBR LE Image
23.53 22.91 23.43 24.05
23.44 23.03 23.16 23.98
24.52 23.62 22.81 23.60
24.33 23.88 23.22 23.64
24.11 23.97 23.15 23.67
24.70 23.69 23.47 23.54
Expression Level
24.5
24.0
23.5
23.0
Dominant Cell Type
a GE & S
a LE
a S

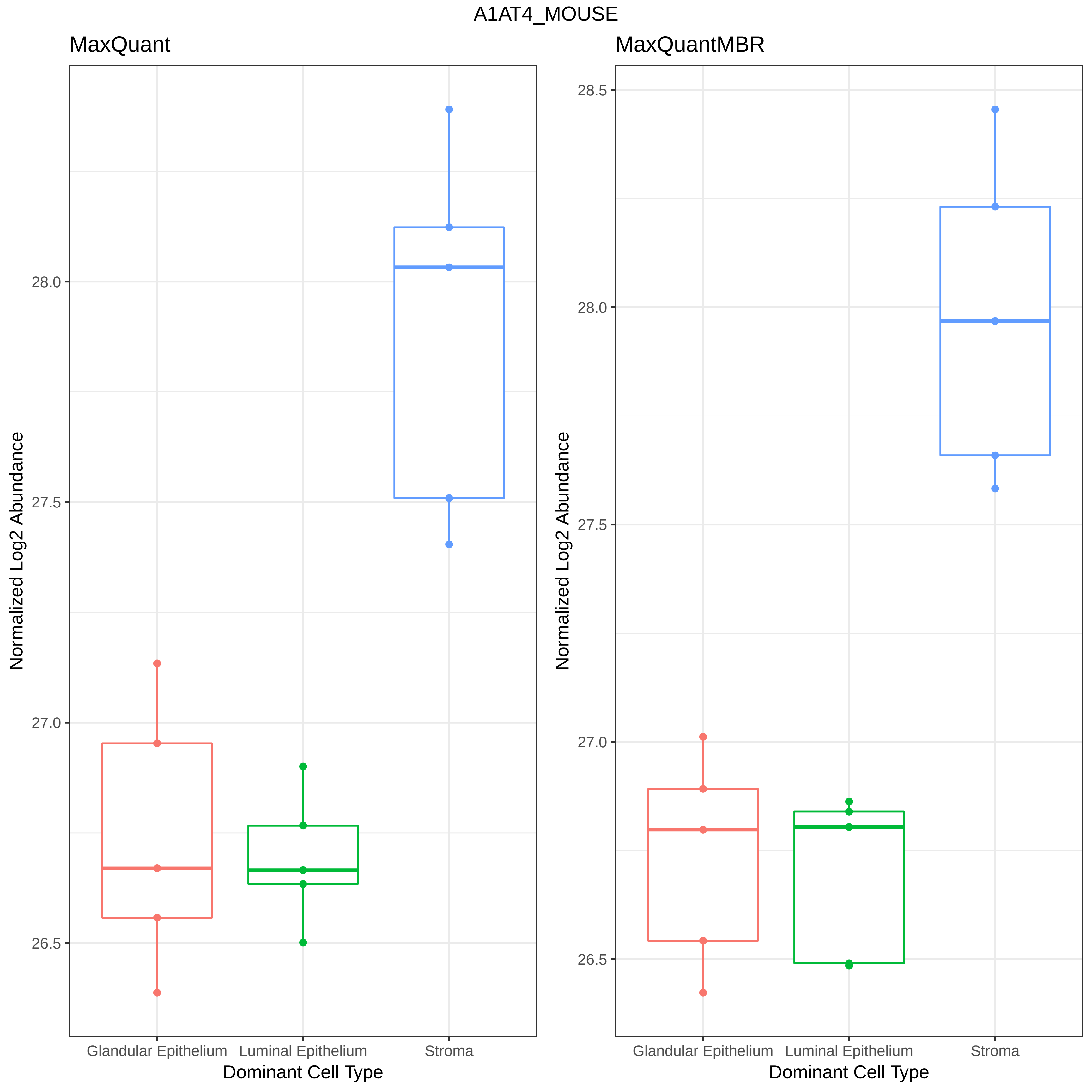
A1AT4_MOUSE
MaxQuant
MaxQuantMBR
Normalized Log2 Abundance
28.5
28.0
27.5
27.0
26.5
Glandular Epithelium
Luminal Epithelium
Stroma
Dominant Cell Type

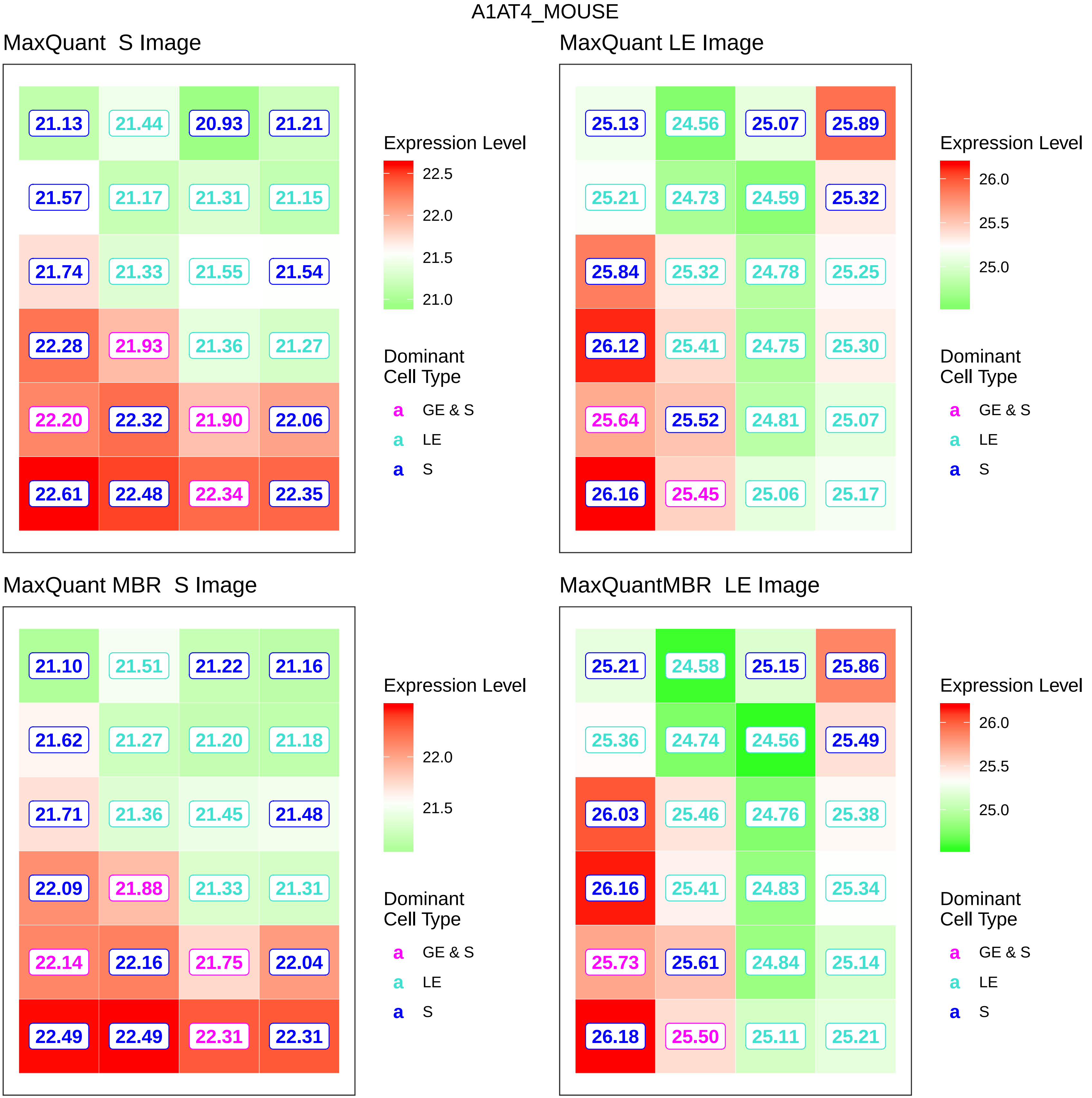
A1AT4_MOUSE
MaxQuant S Image
21.13 21.44 20.93 21.21
21.57 21.17 21.31 21.15
21.74 21.33 21.55 21.54
22.28 21.93 21.36 21.27
22.20 22.32 21.90 22.06
22.61 22.48 22.34 22.35
Expression Level
22.5
22.0
21.5
21.0
Dominant Cell Type
a GE & S
a LE
a S
MaxQuant LE Image
25.13 24.56 25.07 25.89
25.21 24.73 24.59 25.32
25.84 25.32 24.78 25.25
26.12 25.41 24.75 25.30
25.64 25.52 24.81 25.07
26.16 25.45 25.06 25.17
Expression Level
26.0
25.5
25.0
Dominant Cell Type
a GE & S
a LE
a S
MaxQuant MBR S Image
21.10 21.51 21.22 21.16
21.62 21.27 21.20 21.18
21.71 21.36 21.45 21.48
22.09 21.88 21.33 21.31
22.14 22.16 21.75 22.04
22.49 22.49 22.31 22.31
Expression Level
22.0
21.5
Dominant Cell Type
a GE & S
a LE
a S
MaxQuantMBR LE Image
25.21 24.58 25.15 25.86
25.36 24.74 24.56 25.49
26.03 25.46 24.76 25.38
26.16 25.41 24.83 25.34
25.73 25.61 24.84 25.14
26.18 25.50 25.11 25.21
Expression Level
26.0
25.5
25.0
Dominant Cell Type
a GE & S
a LE
a S

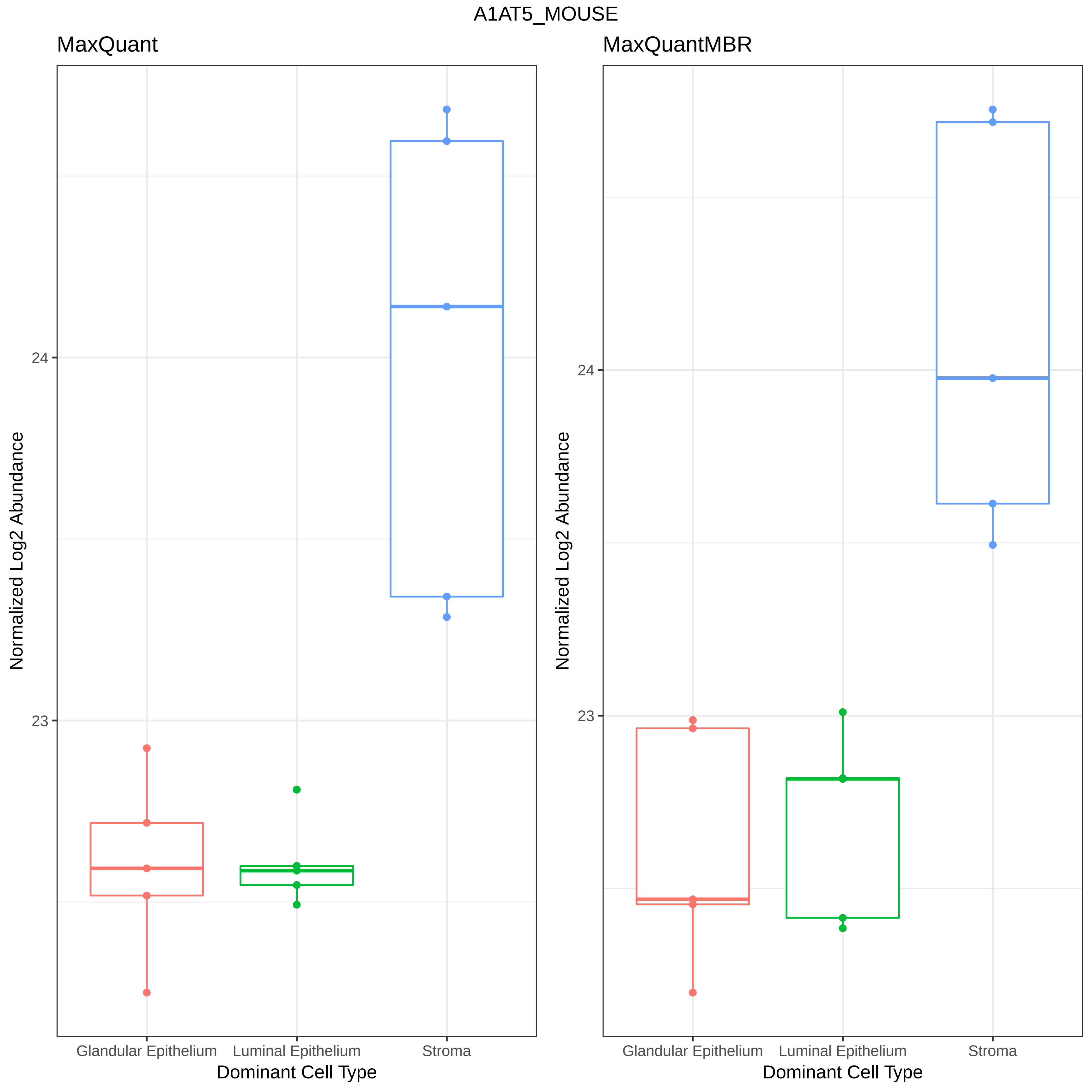
A1AT5_MOUSE
MaxQuant
MaxQuantMBR
Normalized Log2 Abundance
24
23
Glandular Epithelium
Luminal Epithelium
Stroma
Dominant Cell Type

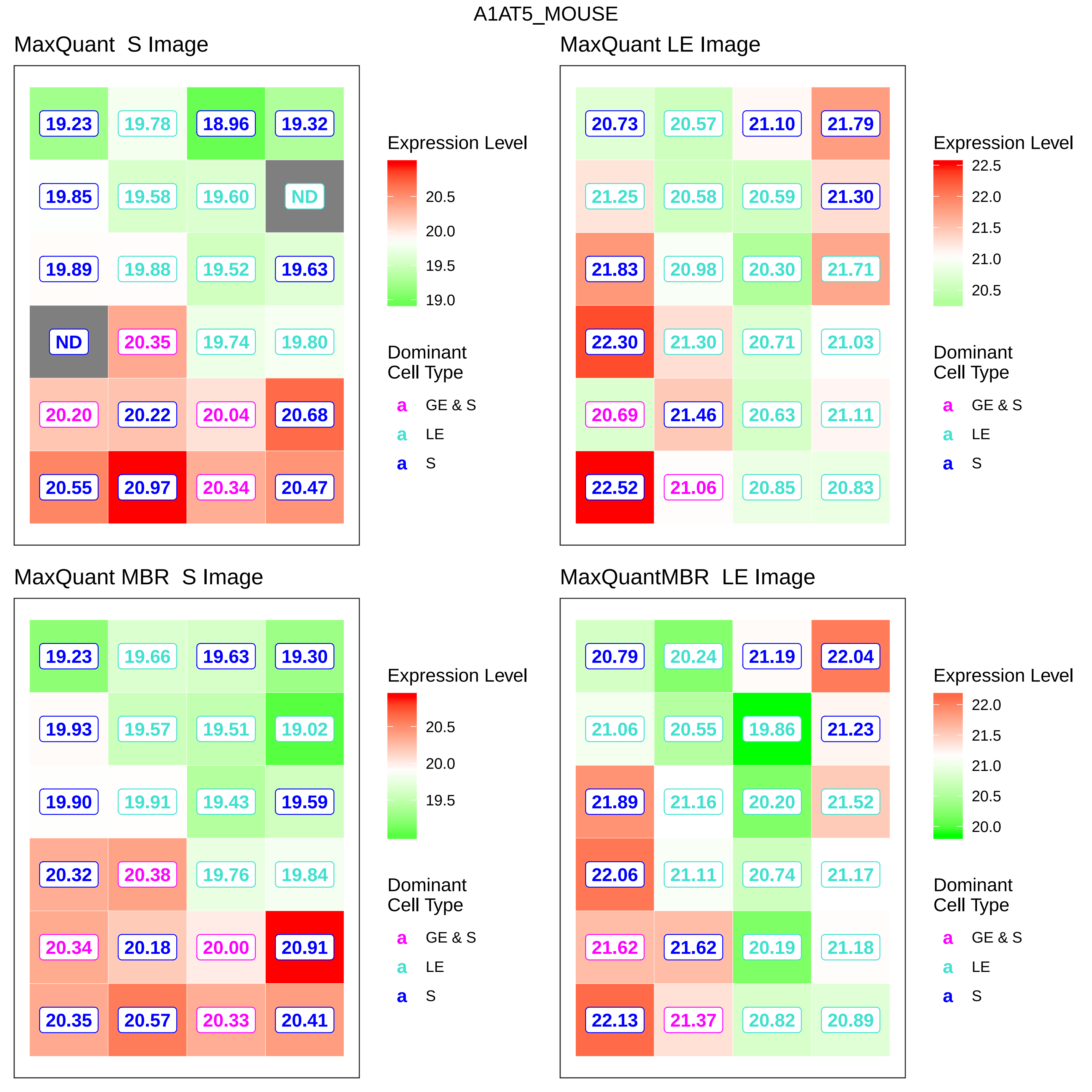
A1AT5_MOUSE
MaxQuant S Image
19.23 19.78 18.96 19.32
19.85 19.58 19.60 ND
19.89 19.88 19.52 19.63
ND 20.35 19.74 19.80
20.20 20.22 20.04 20.68
20.55 20.97 20.34 20.47
Expression Level
20.5
20.0
19.5
19.0
Dominant Cell Type
a GE & S
a LE
a S
MaxQuant LE Image
20.73 20.57 21.10 21.79
21.25 20.58 20.59 21.30
21.83 20.98 20.30 21.71
22.30 21.30 20.71 21.03
20.69 21.46 20.63 21.11
22.52 21.06 20.85 20.83
Expression Level
22.5
22.0
21.5
21.0
20.5
Dominant Cell Type
a GE & S
a LE
a S
MaxQuant MBR S Image
19.23 19.66 19.63 19.30
19.93 19.57 19.51 19.02
19.90 19.91 19.43 19.59
20.32 20.38 19.76 19.84
20.34 20.18 20.00 20.91
20.35 20.57 20.33 20.41
Expression Level
20.5
20.0
19.5
Dominant Cell Type
a GE & S
a LE
a S
MaxQuantMBR LE Image
20.79 20.24 21.19 22.04
21.06 20.55 19.86 21.23
21.89 21.16 20.20 21.52
22.06 21.11 20.74 21.17
21.62 21.62 20.19 21.18
22.13 21.37 20.82 20.89
Expression Level
22.0
21.5
21.0
20.5
20.0
Dominant Cell Type
a GE & S
a LE
a S

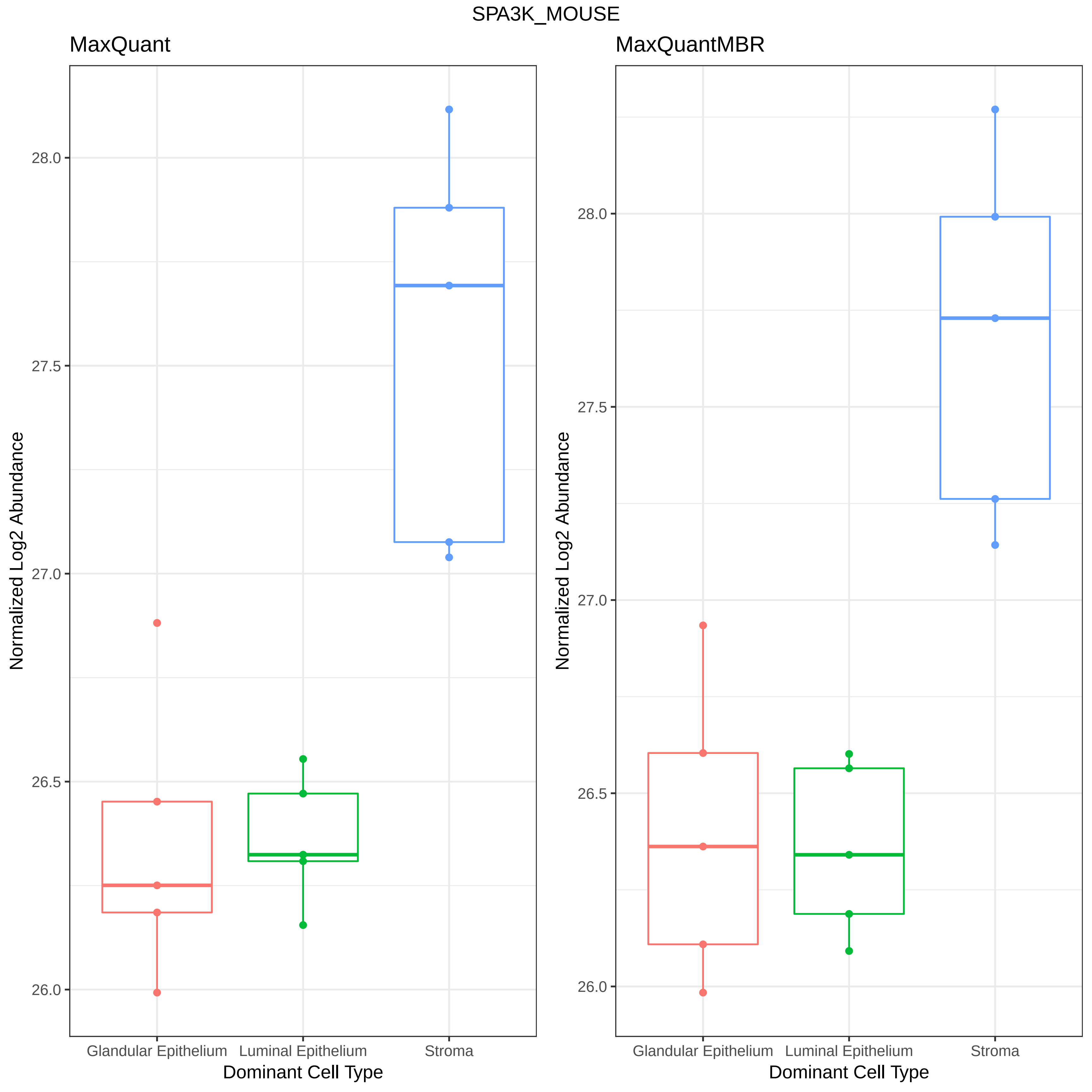
SPA3K_MOUSE
MaxQuant
MaxQuantMBR
Normalized Log2 Abundance
28.0
27.5
27.0
26.5
26.0
Glandular Epithelium
Luminal Epithelium
Stroma
Dominant Cell Type

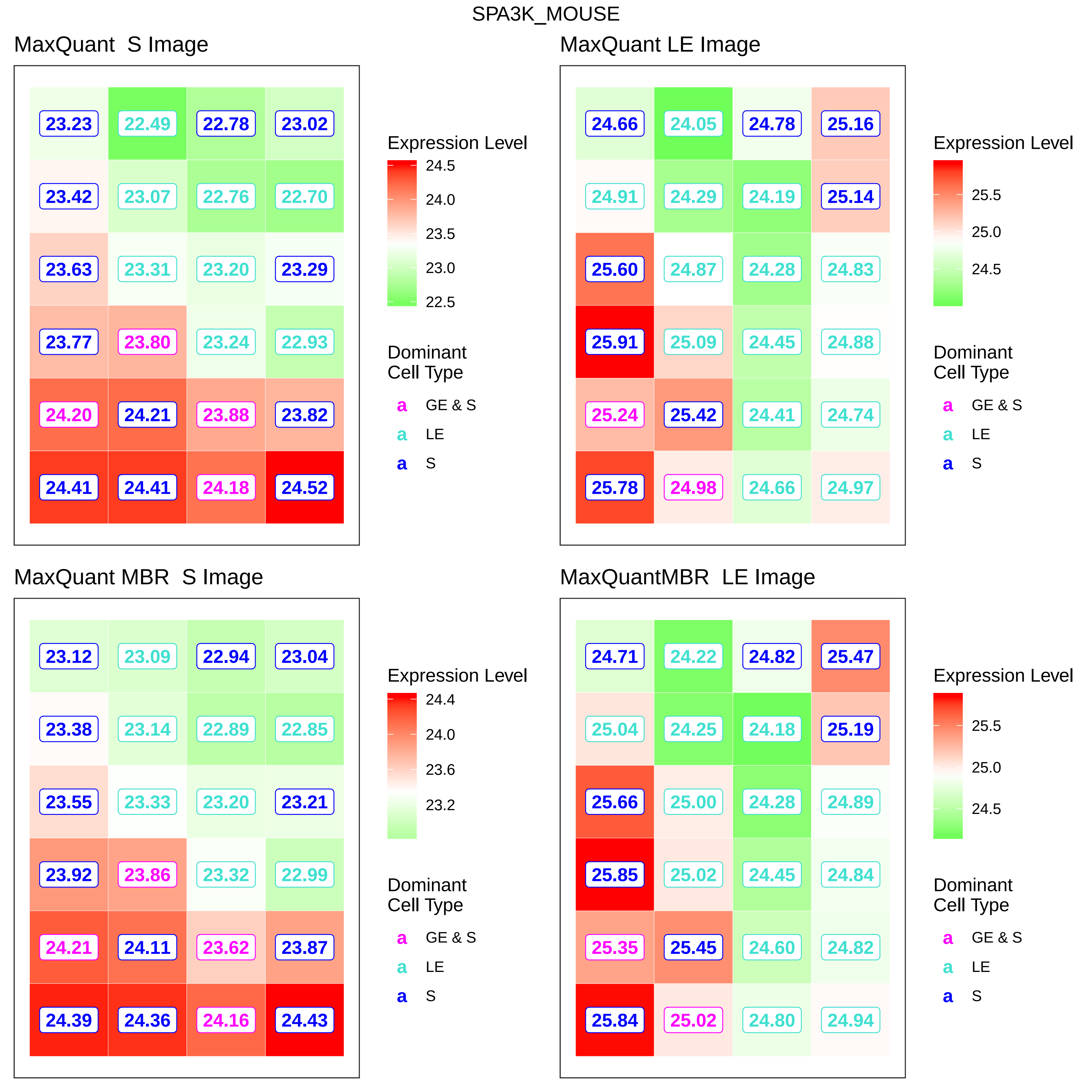
SPA3K_MOUSE
MaxQuant S Image
23.23 22.49 22.78 23.02
23.42 23.07 22.76 22.70
23.63 23.31 23.20 23.29
23.77 23.80 23.24 22.93
24.20 24.21 23.88 23.82
24.41 24.41 24.18 24.52
Expression Level
24.5
24.0
23.5
23.0
22.5
Dominant Cell Type
a GE & S
a LE
a S
MaxQuant LE Image
24.66 24.05 24.78 25.16
24.91 24.29 24.19 25.14
25.60 24.87 24.28 24.83
25.91 25.09 24.45 24.88
25.24 25.42 24.41 24.74
25.78 24.98 24.66 24.97
Expression Level
25.5
25.0
24.5
Dominant Cell Type
a GE & S
a LE
a S
MaxQuant MBR S Image
23.12 23.09 22.94 23.04
23.38 23.14 22.89 22.85
23.55 23.33 23.20 23.21
23.92 23.86 23.32 22.99
24.21 24.11 23.62 23.87
24.39 24.36 24.16 24.43
Expression Level
24.4
24.0
23.6
23.2
Dominant Cell Type
a GE & S
a LE
a S
MaxQuantMBR LE Image
24.71 24.22 24.82 25.47
25.04 24.25 24.18 25.19
25.66 25.00 24.28 24.89
25.85 25.02 24.45 24.84
25.35 25.45 24.60 24.82
25.84 25.02 24.80 24.94
Expression Level
25.5
25.0
24.5
Dominant Cell Type
a GE & S
a LE
a S

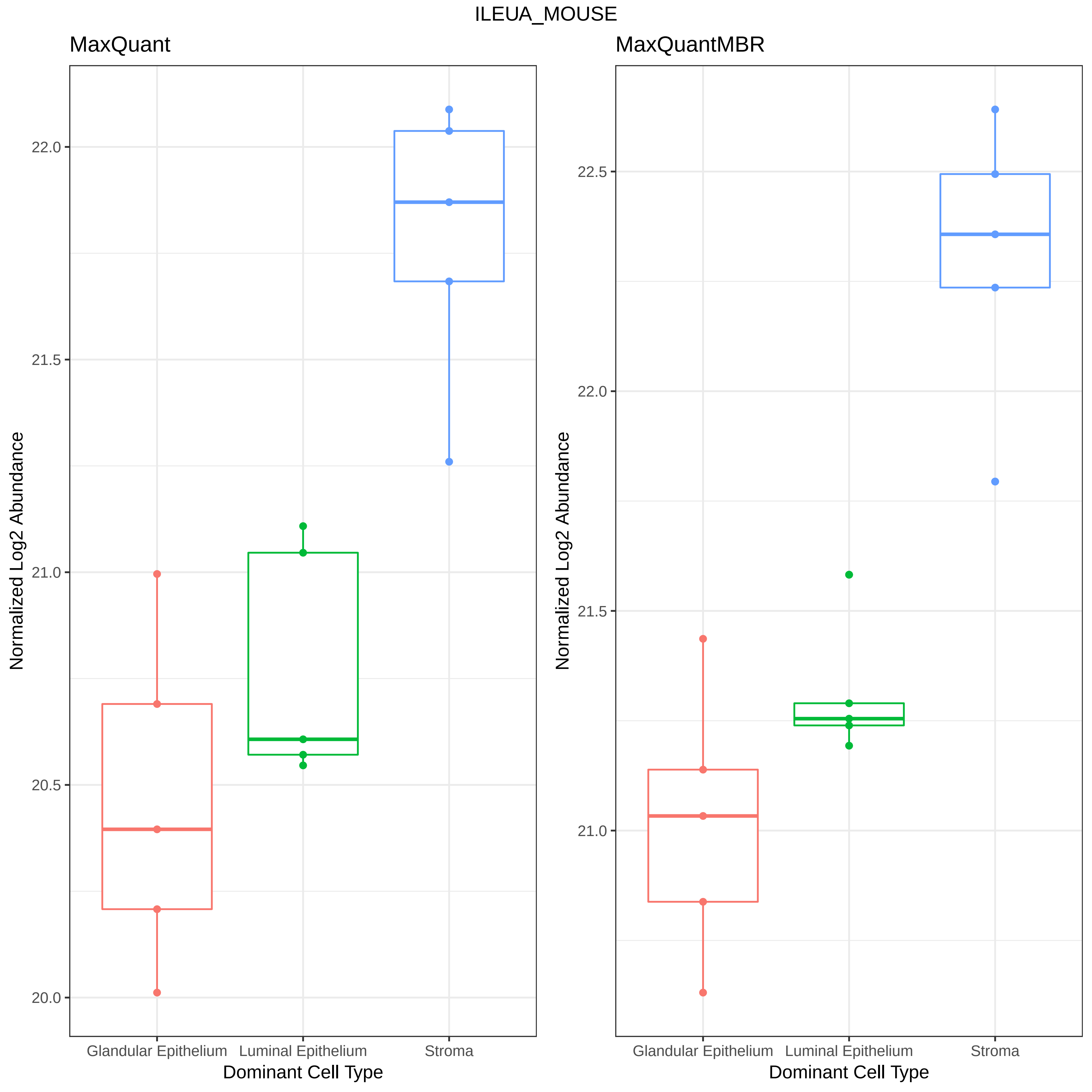

ILEUA_MOUSE
MaxQuant
MaxQuantMBR
Normalized Log2 Abundance
22.0
21.5
21.0
20.5
20.0
22.5
Glandular Epithelium
Luminal Epithelium
Stroma
Dominant Cell Type

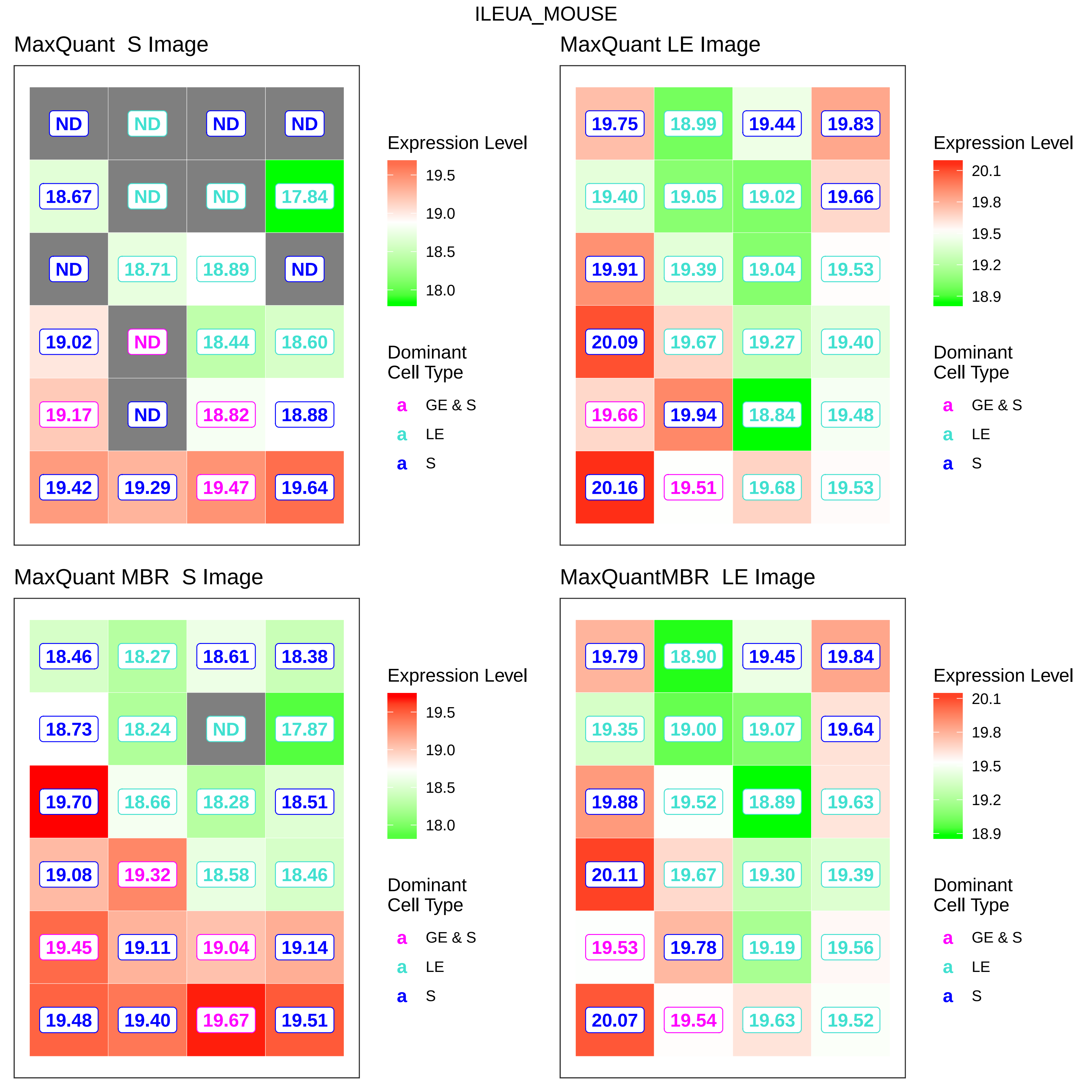
ILEUA_MOUSE
MaxQuant S Image
ND ND ND ND
18.67 ND ND 17.84
ND 18.71 18.89 ND
19.02 ND 18.44 18.60
19.17 ND 18.82 18.88
19.42 19.29 19.47 19.64
Expression Level
19.5
19.0
18.5
18.0
Dominant Cell Type
a GE & S
a LE
a S
MaxQuant LE Image
19.75 18.99 19.44 19.83
19.40 19.05 19.02 19.66
19.91 19.39 19.04 19.53
20.09 19.67 19.27 19.40
19.66 19.94 18.84 19.48
20.16 19.51 19.68 19.53
Expression Level
20.1
19.8
19.5
19.2
18.9
Dominant Cell Type
a GE & S
a LE
a S
MaxQuant MBR S Image
18.46 18.27 18.61 18.38
18.73 18.24 ND 17.87
19.70 18.66 18.28 18.51
19.08 19.32 18.58 18.46
19.45 19.11 19.04 19.14
19.48 19.40 19.67 19.51
Expression Level
19.5
19.0
18.5
18.0
Dominant Cell Type
a GE & S
a LE
a S
MaxQuantMBR LE Image
19.79 18.90 19.45 19.84
19.35 19.00 19.07 19.64
19.88 19.52 18.89 19.63
20.11 19.67 19.30 19.39
19.53 19.78 19.19 19.56
20.07 19.54 19.63 19.52
Expression Level
20.1
19.8
19.5
19.2
18.9
Dominant Cell Type
a GE & S
a LE
a S

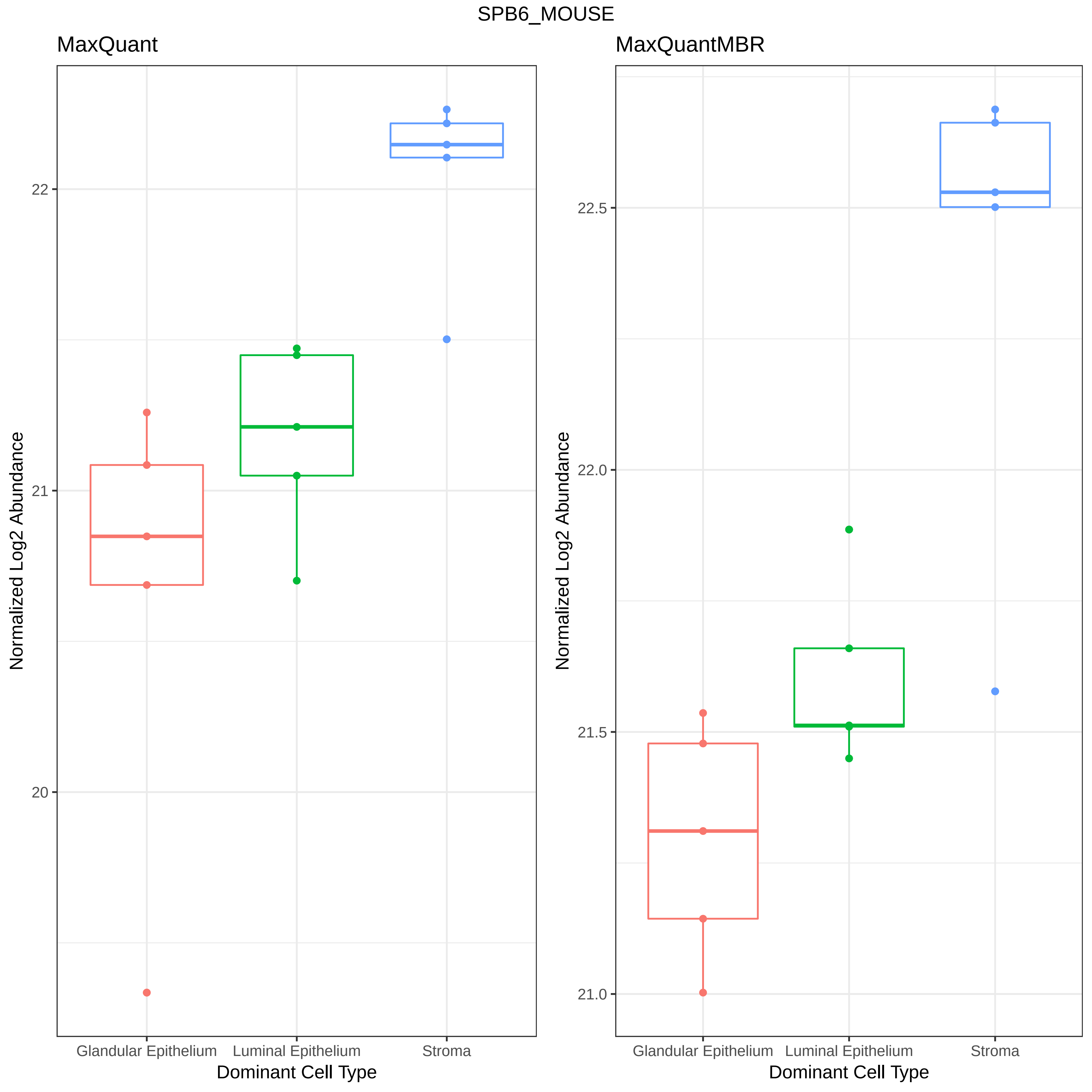
SPB6_MOUSE
MaxQuant
MaxQuantMBR
Normalized Log2 Abundance
22
21
20
22.5
22.0
21.5
21.0
Glandular Epithelium
Luminal Epithelium
Stroma
Dominant Cell Type

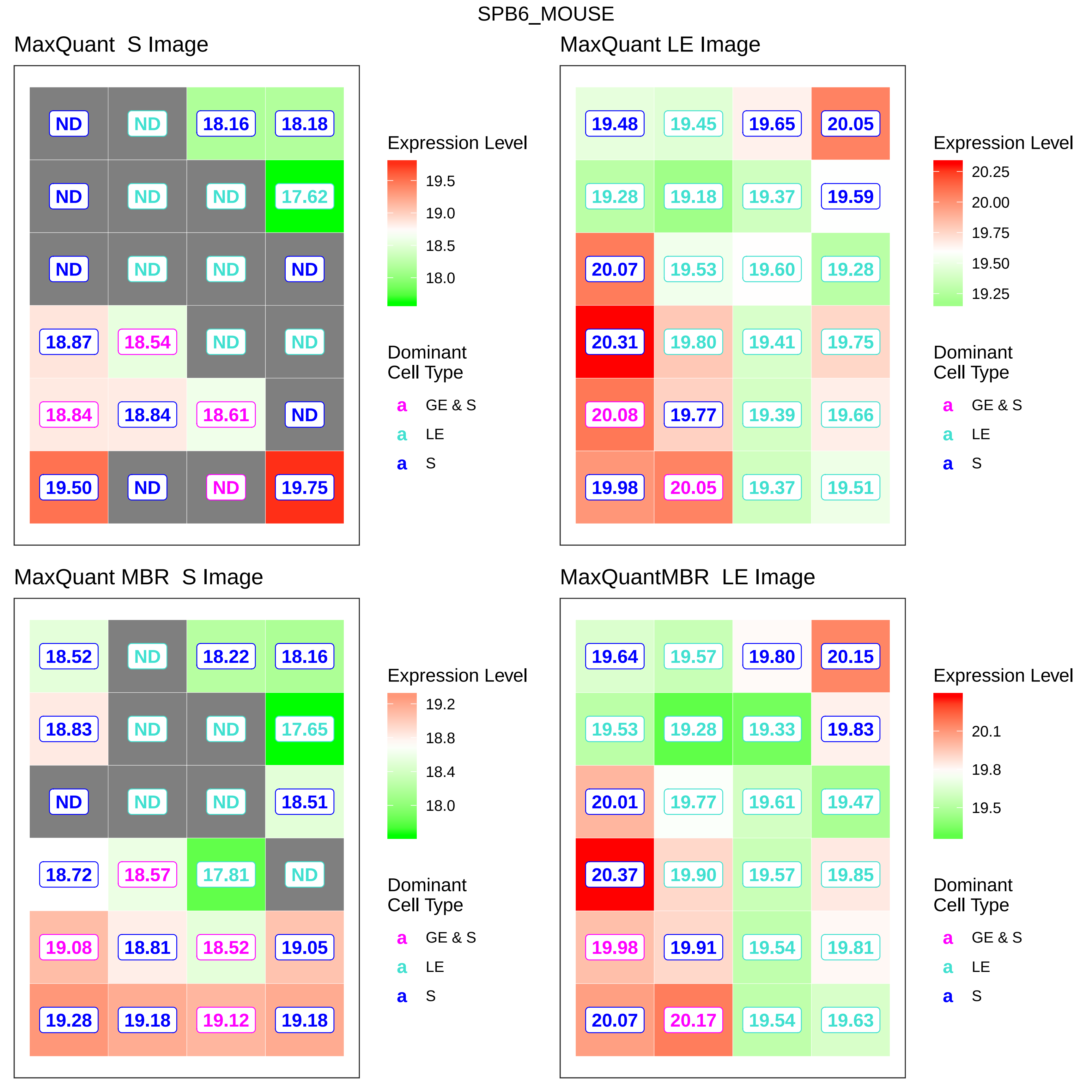
SPB6_MOUSE
MaxQuant S Image
ND ND 18.16 18.18
ND ND ND 17.62
ND ND ND ND
18.87 18.54 ND ND
18.84 18.84 18.61 ND
19.50 ND ND 19.75
Expression Level
19.5
19.0
18.5
18.0
Dominant Cell Type
a GE & S
a LE
a S
MaxQuant LE Image
19.48 19.45 19.65 20.05
19.28 19.18 19.37 19.59
20.07 19.53 19.60 19.28
20.31 19.80 19.41 19.75
20.08 19.77 19.39 19.66
19.98 20.05 19.37 19.51
Expression Level
20.25
20.00
19.75
19.50
19.25
Dominant Cell Type
a GE & S
a LE
a S
MaxQuant MBR S Image
18.52 ND 18.22 18.16
18.83 ND ND 17.65
ND ND ND 18.51
18.72 18.57 17.81 ND
19.08 18.81 18.52 19.05
19.28 19.18 19.12 19.18
Expression Level
19.2
18.8
18.4
18.0
Dominant Cell Type
a GE & S
a LE
a S
MaxQuantMBR LE Image
19.64 19.57 19.80 20.15
19.53 19.28 19.33 19.83
20.01 19.77 19.61 19.47
20.37 19.90 19.57 19.85
19.98 19.91 19.54 19.81
20.07 20.17 19.54 19.63
Expression Level
20.1
19.8
19.5
Dominant Cell Type
a GE & S
a LE
a S

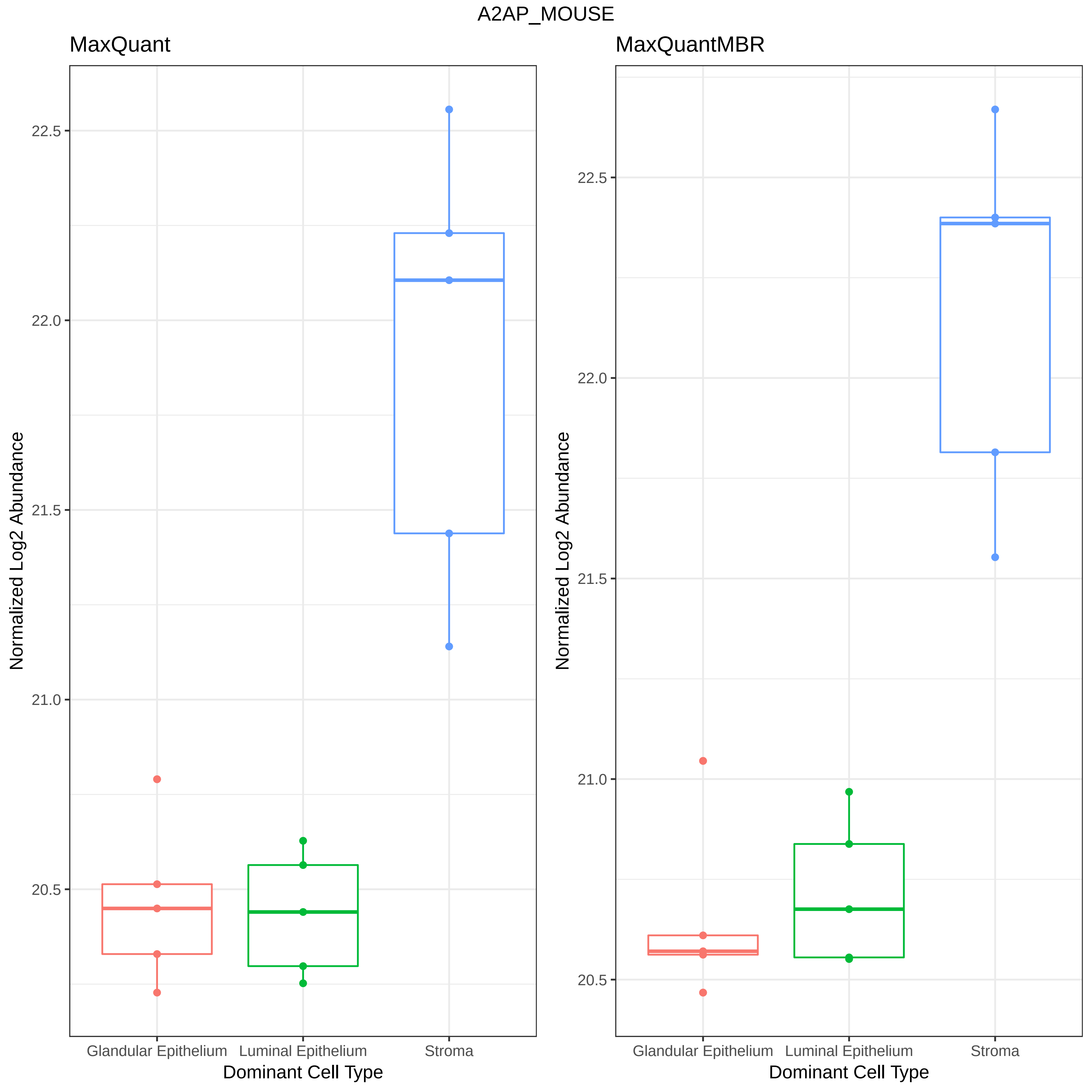

A2AP_MOUSE
MaxQuant
MaxQuantMBR
Normalized Log2 Abundance
22.5
22.0
21.5
21.0
20.5
Glandular Epithelium
Luminal Epithelium
Stroma
Dominant Cell Type

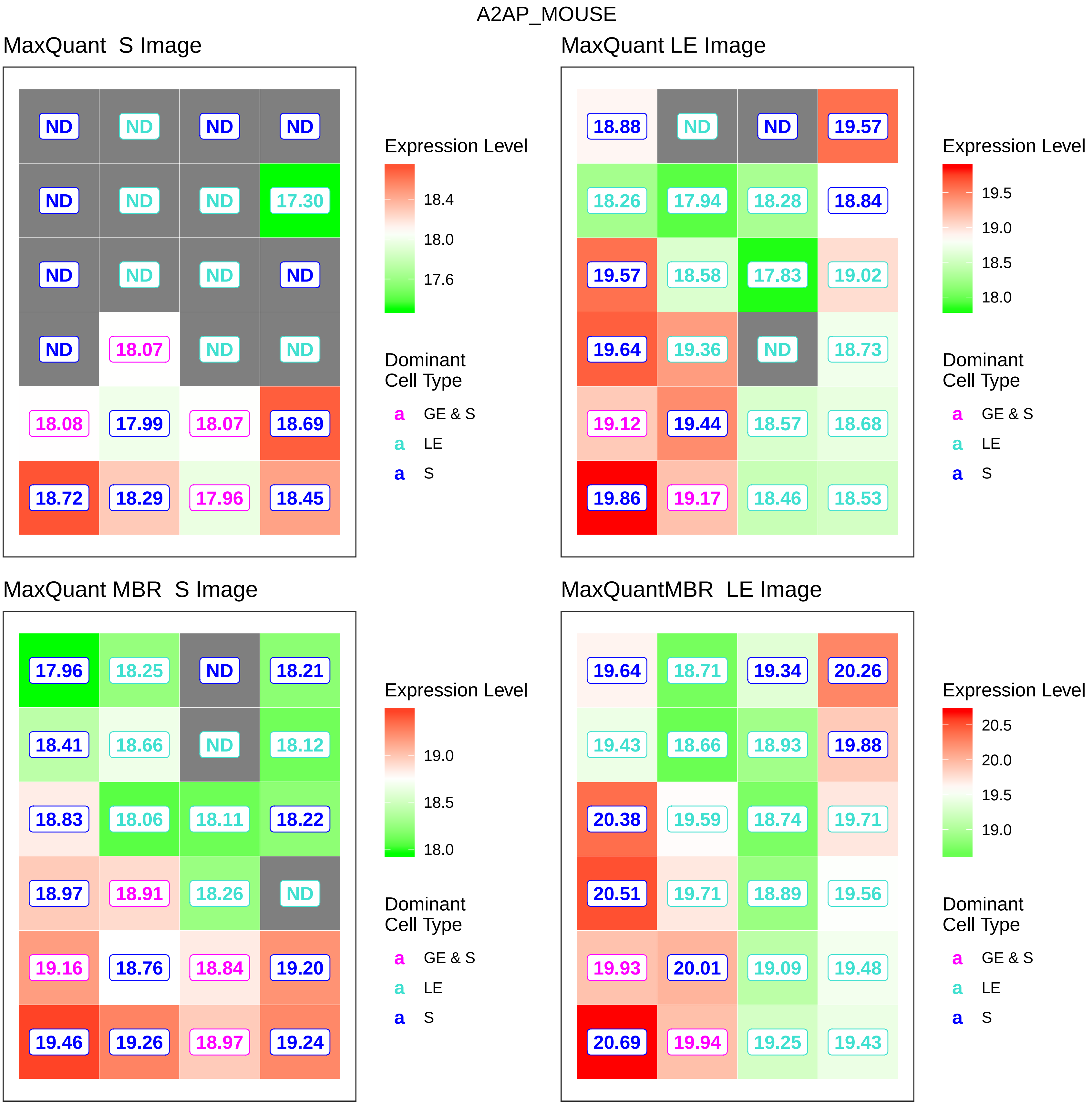
A2AP_MOUSE
MaxQuant S Image
ND ND ND ND
ND ND ND 17.30
ND ND ND ND
ND 18.07 ND ND
18.08 17.99 18.07 18.69
18.72 18.29 17.96 18.45
Expression Level
18.4
18.0
17.6
Dominant Cell Type
a GE & S
a LE
a S
MaxQuant LE Image
18.88 ND ND 19.57
18.26 17.94 18.28 18.84
19.57 18.58 17.83 19.02
19.64 19.36 ND 18.73
19.12 19.44 18.57 18.68
19.86 19.17 18.46 18.53
Expression Level
19.5
19.0
18.5
18.0
Dominant Cell Type
a GE & S
a LE
a S
MaxQuant MBR S Image
17.96 18.25 ND 18.21
18.41 18.66 ND 18.12
18.83 18.06 18.11 18.22
18.97 18.91 18.26 ND
19.16 18.76 18.84 19.20
19.46 19.26 18.97 19.24
Expression Level
19.0
18.5
18.0
Dominant Cell Type
a GE & S
a LE
a S
MaxQuantMBR LE Image
19.64 18.71 19.34 20.26
19.43 18.66 18.93 19.88
20.38 19.59 18.74 19.71
20.51 19.71 18.89 19.56
19.93 20.01 19.09 19.48
20.69 19.94 19.25 19.43
Expression Level
20.5
20.0
19.5
19.0
Dominant Cell Type
a GE & S
a LE
a S

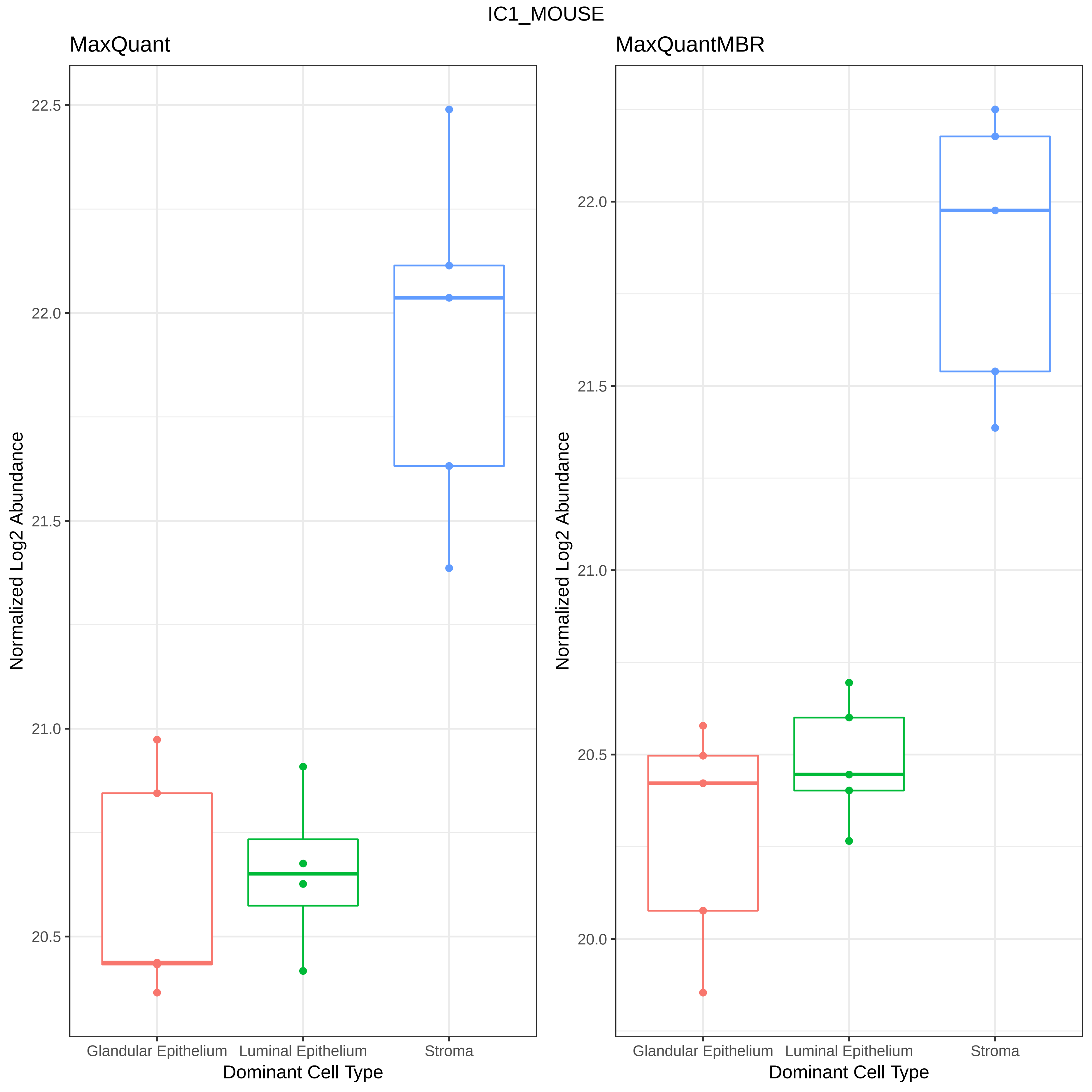
IC1_MOUSE
MaxQuant
MaxQuantMBR
Normalized Log2 Abundance
22.5
22.0
21.5
21.0
20.5
20.0
Glandular Epithelium
Luminal Epithelium
Stroma
Dominant Cell Type

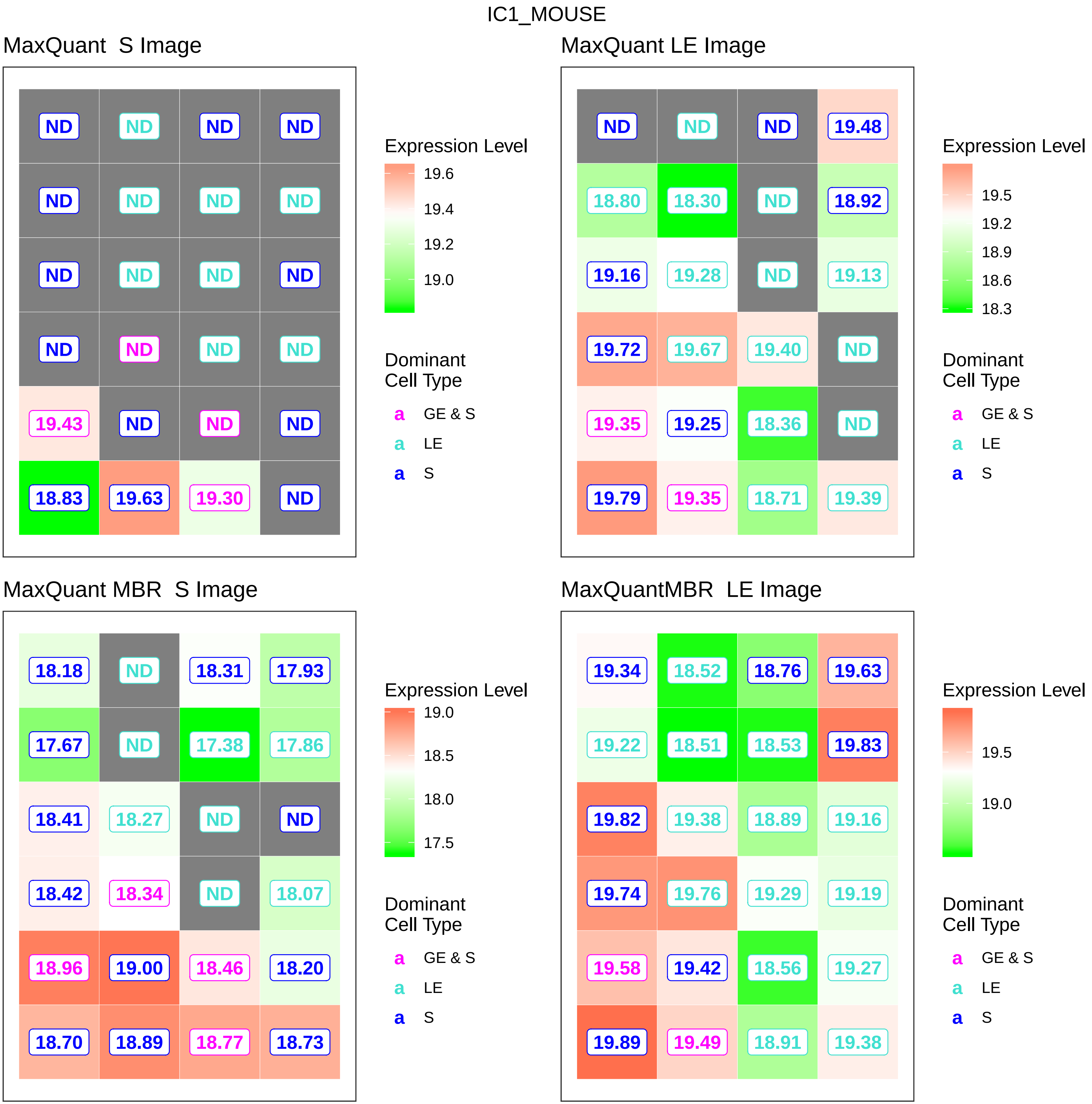
IC1_MOUSE
MaxQuant S Image
ND ND ND ND
ND ND ND ND
ND ND ND ND
ND ND ND ND
19.43 ND ND ND
18.83 19.63 19.30 ND
Expression Level
19.6
19.4
19.2
19.0
Dominant Cell Type
a GE & S
a LE
a S
MaxQuant LE Image
ND ND ND 19.48
18.80 18.30 ND 18.92
19.16 19.28 ND 19.13
19.72 19.67 19.40 ND
19.35 19.25 18.36 ND
19.79 19.35 18.71 19.39
Expression Level
19.5
19.2
18.9
18.6
18.3
Dominant Cell Type
a GE & S
a LE
a S
MaxQuant MBR S Image
18.18 ND 18.31 17.93
17.67 ND 17.38 17.86
18.41 18.27 ND ND
18.42 18.34 ND 18.07
18.96 19.00 18.46 18.20
18.70 18.89 18.77 18.73
Expression Level
19.0
18.5
18.0
17.5
Dominant Cell Type
a GE & S
a LE
a S
MaxQuantMBR LE Image
19.34 18.52 18.76 19.63
19.22 18.51 18.53 19.83
19.82 19.38 18.89 19.16
19.74 19.76 19.29 19.19
19.58 19.42 18.56 19.27
19.89 19.49 18.91 19.38
Expression Level
19.5
19.0
Dominant Cell Type
a GE & S
a LE
a S

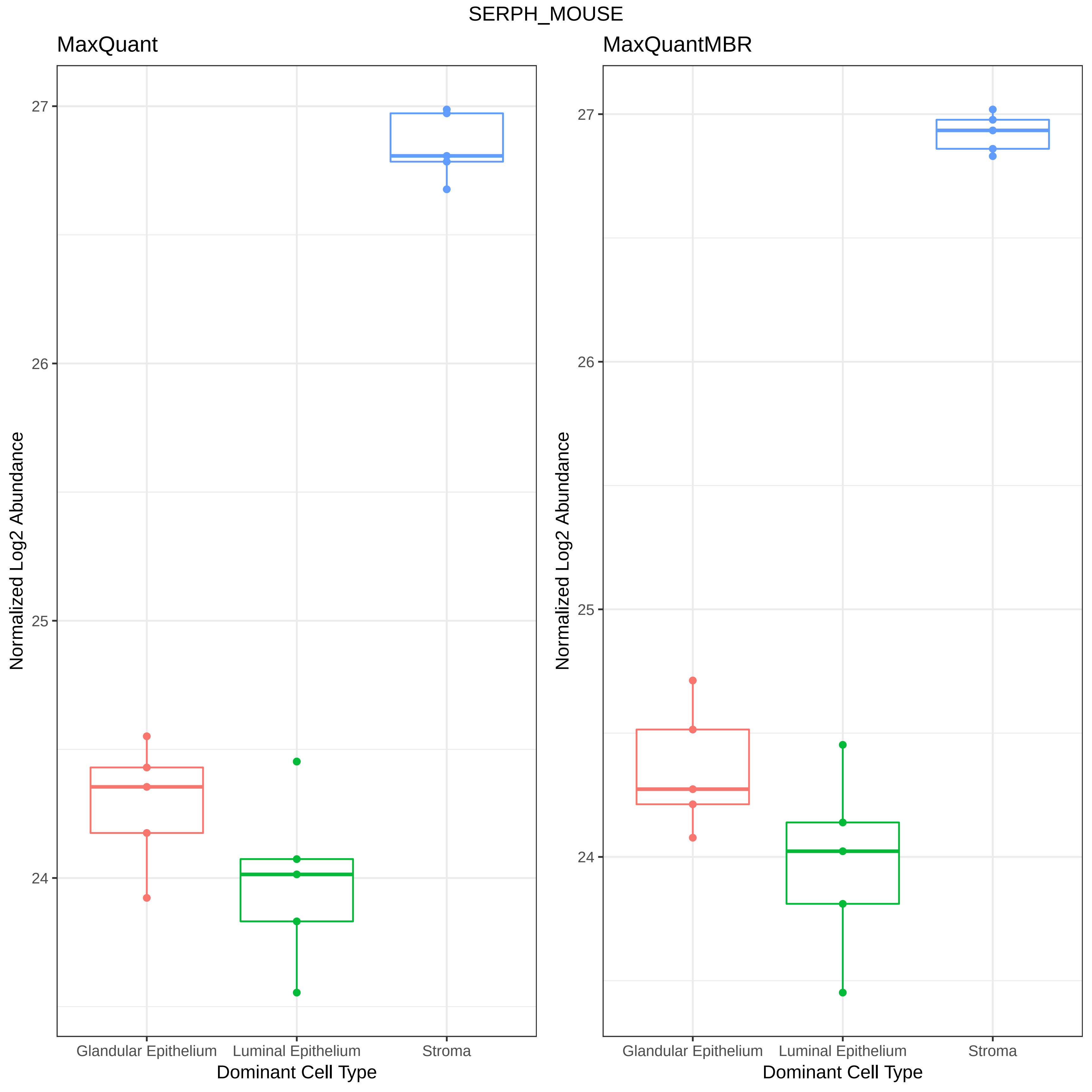

SERPH_MOUSE
MaxQuant
MaxQuantMBR
27
26
25
24
Normalized Log2 Abundance
Glandular Epithelium
Luminal Epithelium
Stroma
Dominant Cell Type

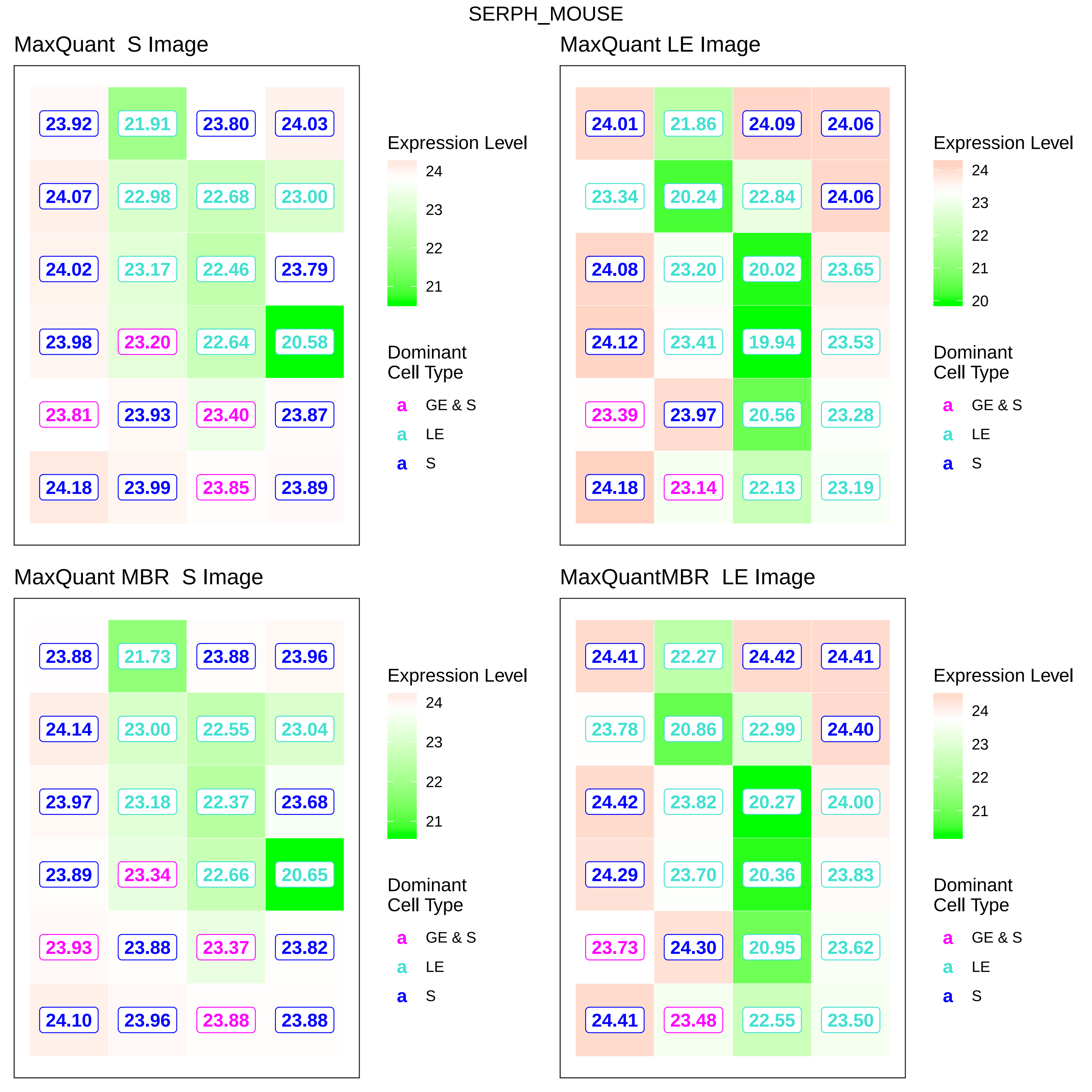

SERPH_MOUSE
MaxQuant S Image
23.92 21.91 23.80 24.03
24.07 22.98 22.68 23.00
24.02 23.17 22.46 23.79
23.98 23.20 22.64 20.58
23.81 23.93 23.40 23.87
24.18 23.99 23.85 23.89
Expression Level
24
23
22
21
Dominant Cell Type
a GE & S
a LE
a S
MaxQuant LE Image
24.01 21.86 24.09 24.06
23.34 20.24 22.84 24.06
24.08 23.20 20.02 23.65
24.12 23.41 19.94 23.53
23.39 23.97 20.56 23.28
24.18 23.14 22.13 23.19
Expression Level
24
23
22
21
20
Dominant Cell Type
a GE & S
a LE
a S
MaxQuant MBR S Image
23.88 21.73 23.88 23.96
24.14 23.00 22.55 23.04
23.97 23.18 22.37 23.68
23.89 23.34 22.66 20.65
23.93 23.88 23.37 23.82
24.10 23.96 23.88 23.88
Expression Level
24
23
22
21
Dominant Cell Type
a GE & S
a LE
a S
MaxQuantMBR LE Image
24.41 22.27 24.42 24.41
23.78 20.86 22.99 24.40
24.42 23.82 20.27 24.00
24.29 23.70 20.36 23.83
23.73 24.30 20.95 23.62
24.41 23.48 22.55 23.50
Expression Level
24
23
22
21
Dominant Cell Type
a GE & S
a LE
a S

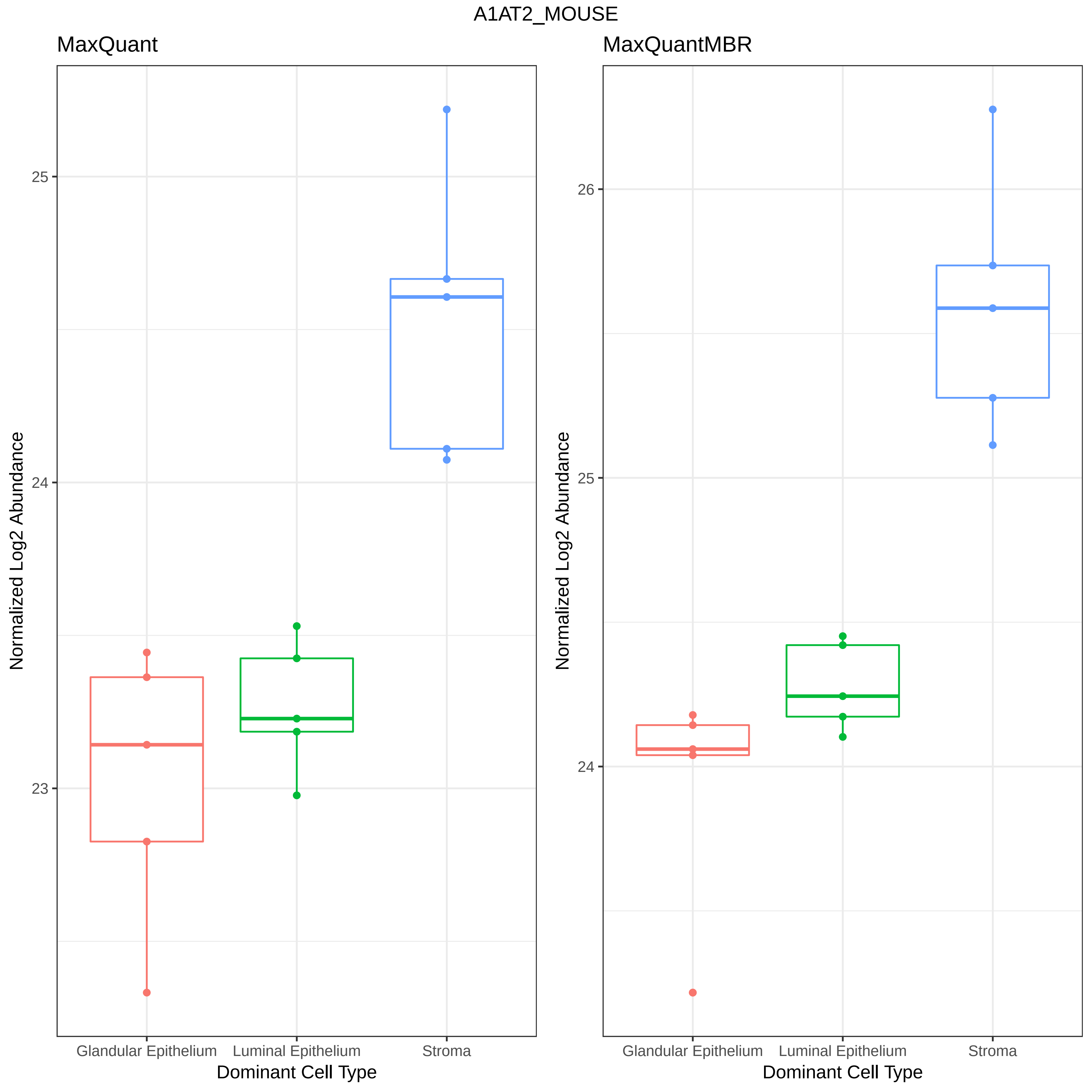
A1AT2_MOUSE
MaxQuant
MaxQuantMBR
Normalized Log2 Abundance
25
24
23
26
25
24
Glandular Epithelium
Luminal Epithelium
Stroma
Dominant Cell Type
Glandular Epithelium
Luminal Epithelium
Stroma
Dominant Cell Type

A1AT2_MOUSE

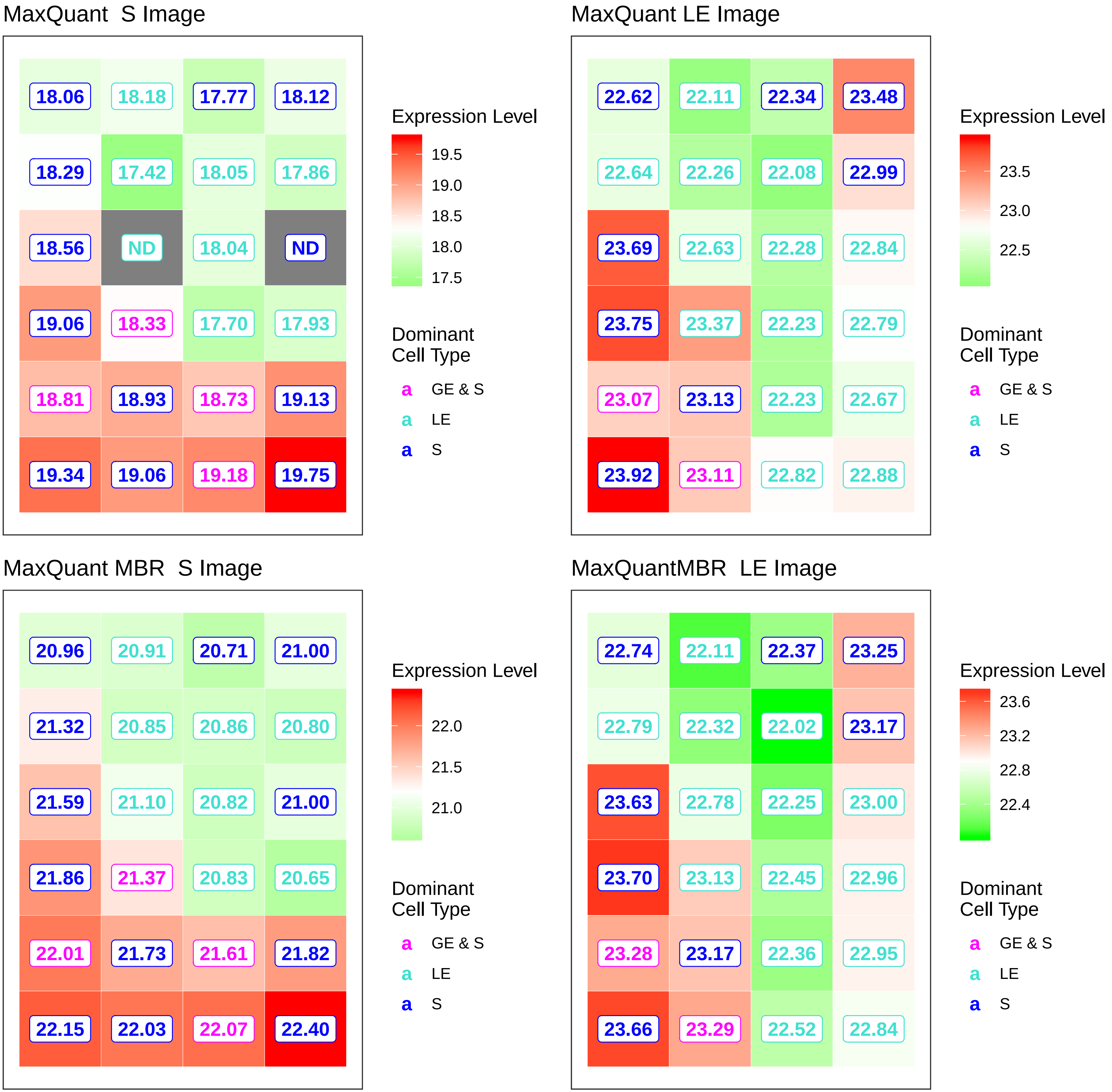
MaxQuant S Image
18.06 18.18 17.77 18.12
18.29 17.42 18.05 17.86
18.56 ND 18.04 ND
19.06 18.33 17.70 17.93
18.81 18.93 18.73 19.13
19.34 19.06 19.18 19.75
Expression Level
19.5
19.0
18.5
18.0
17.5
Dominant Cell Type
a GE & S
a LE
a S
MaxQuant LE Image
22.62 22.11 22.34 23.48
22.64 22.26 22.08 22.99
23.69 22.63 22.28 22.84
23.75 23.37 22.23 22.79
23.07 23.13 22.23 22.67
23.92 23.11 22.82 22.88
Expression Level
23.5
23.0
22.5
Dominant Cell Type
a GE & S
a LE
a S
MaxQuant MBR S Image
20.96 20.91 20.71 21.00
21.32 20.85 20.86 20.80
21.59 21.10 20.82 21.00
21.86 21.37 20.83 20.65
22.01 21.73 21.61 21.82
22.15 22.03 22.07 22.40
Expression Level
22.0
21.5
21.0
Dominant Cell Type
a GE & S
a LE
a S
MaxQuantMBR LE Image
22.74 22.11 22.37 23.25
22.79 22.32 22.02 23.17
23.63 22.78 22.25 23.00
23.70 23.13 22.45 22.96
23.28 23.17 22.36 22.95
23.66 23.29 22.52 22.84
Expression Level
23.6
23.2
22.8
22.4
Dominant Cell Type
a GE & S
a LE
a S

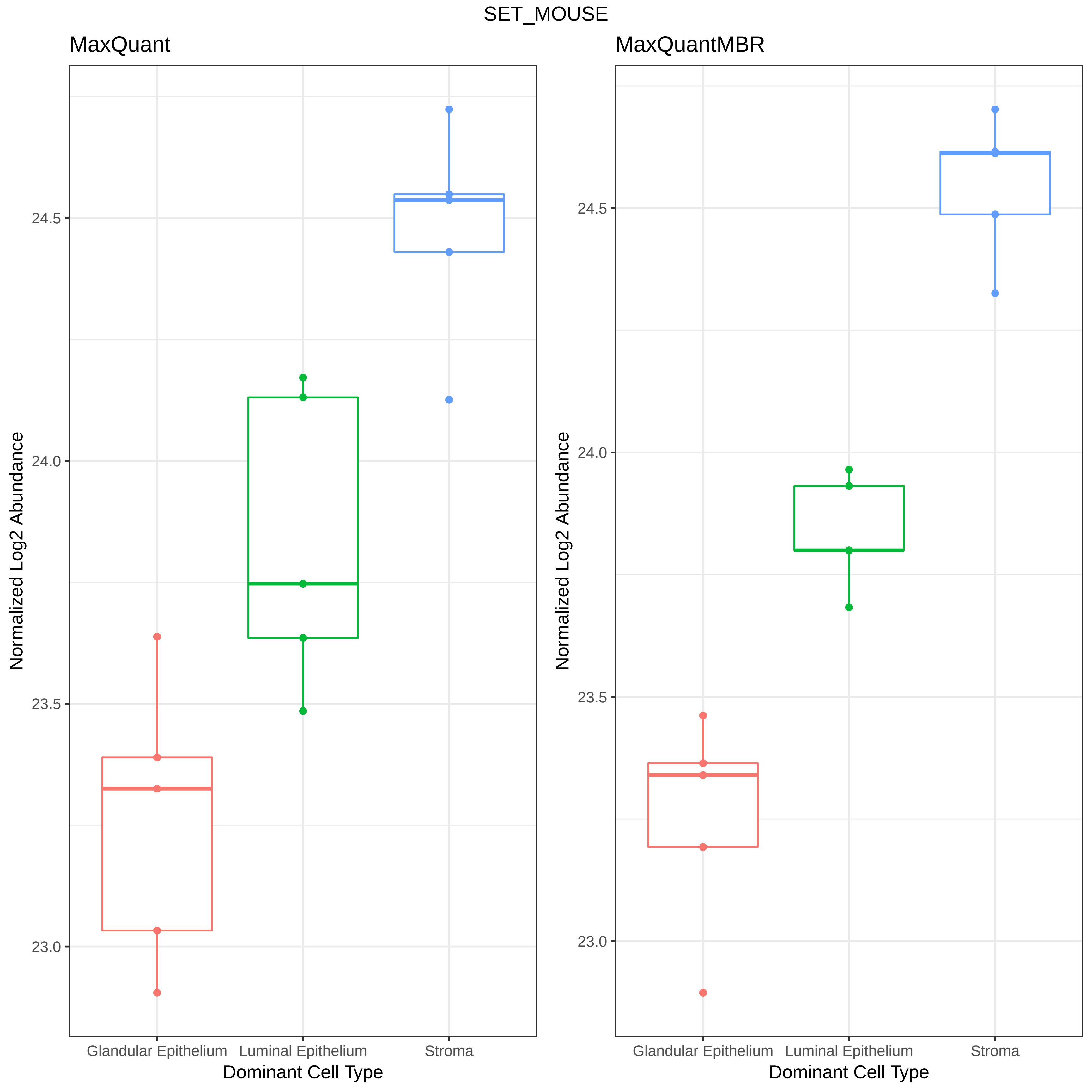
SET_MOUSE
MaxQuant
MaxQuantMBR
Normalized Log2 Abundance
23.0
23.5
24.0
24.5
Glandular Epithelium
Luminal Epithelium
Stroma
Dominant Cell Type

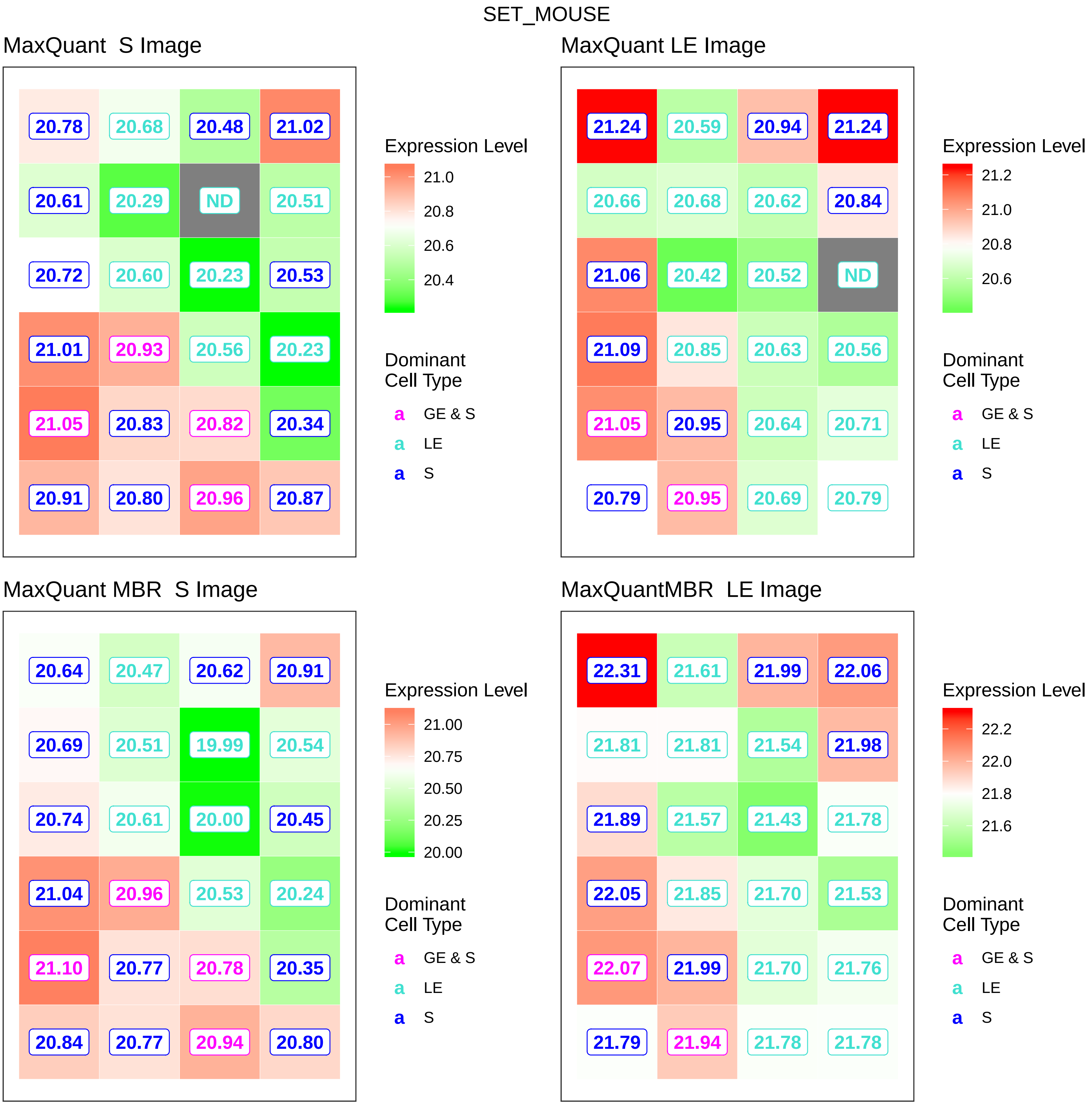
SET_MOUSE
MaxQuant S Image
20.78 20.68 20.48 21.02
20.61 20.29 ND 20.51
20.72 20.60 20.23 20.53
21.01 20.93 20.56 20.23
21.05 20.83 20.82 20.34
20.91 20.80 20.96 20.87
Expression Level
21.0
20.8
20.6
20.4
Dominant Cell Type
a GE & S
a LE
a S
MaxQuant LE Image
21.24 20.59 20.94 21.24
20.66 20.68 20.62 20.84
21.06 20.42 20.52 ND
21.09 20.85 20.63 20.56
21.05 20.95 20.64 20.71
20.79 20.95 20.69 20.79
Expression Level
21.2
21.0
20.8
20.6
Dominant Cell Type
a GE & S
a LE
a S
MaxQuant MBR S Image
20.64 20.47 20.62 20.91
20.69 20.51 19.99 20.54
20.74 20.61 20.00 20.45
21.04 20.96 20.53 20.24
21.10 20.77 20.78 20.35
20.84 20.77 20.94 20.80
Expression Level
21.00
20.75
20.50
20.25
20.00
Dominant Cell Type
a GE & S
a LE
a S
MaxQuantMBR LE Image
22.31 21.61 21.99 22.06
21.81 21.81 21.54 21.98
21.89 21.57 21.43 21.78
22.05 21.85 21.70 21.53
22.07 21.99 21.70 21.76
21.79 21.94 21.78 21.78
Expression Level
22.2
22.0
21.8
21.6
Dominant Cell Type
a GE & S
a LE
a S

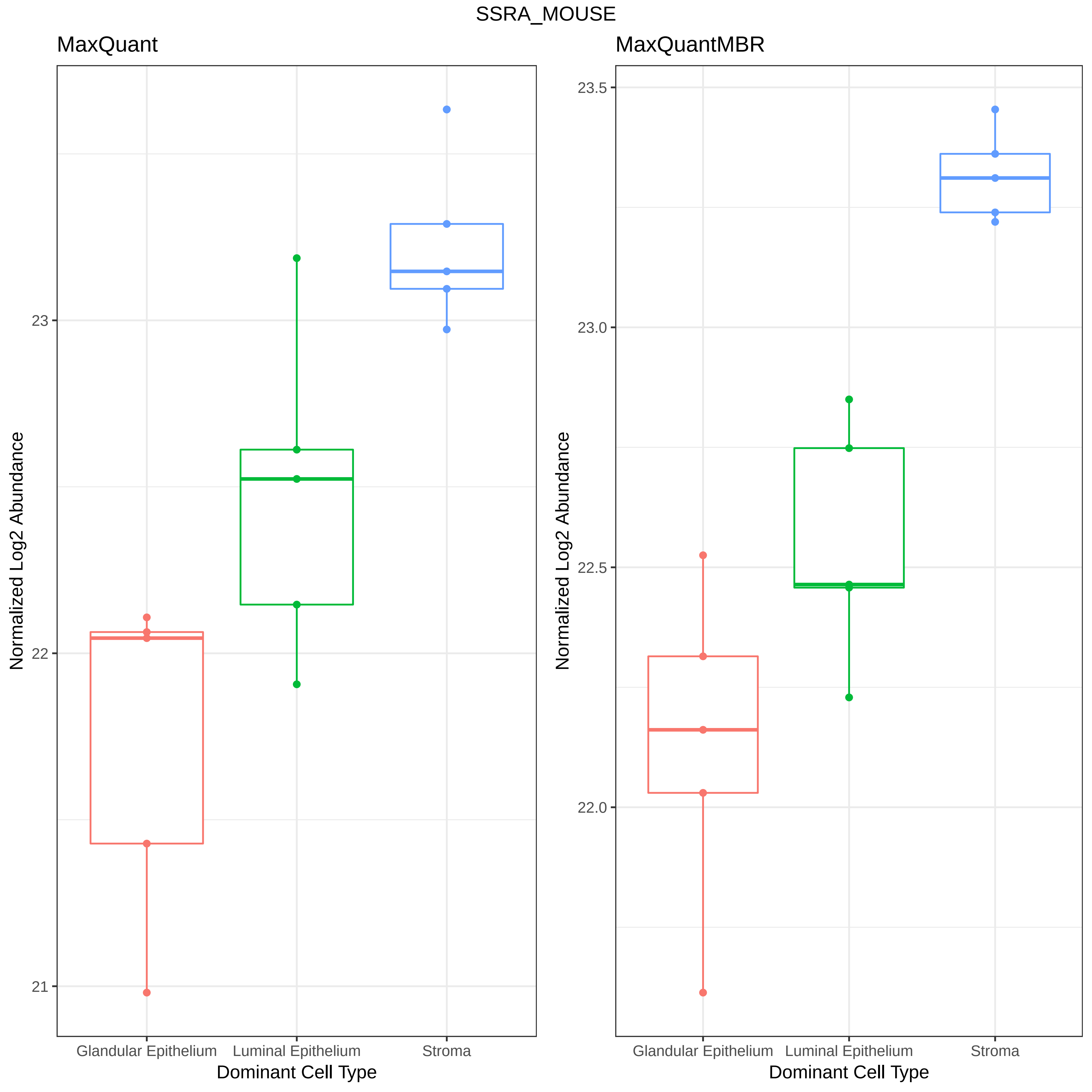

SSRA_MOUSE
MaxQuant
MaxQuantMBR
Normalized Log2 Abundance
23
22
21
23.5
23.0
22.5
22.0
Glandular Epithelium
Luminal Epithelium
Stroma
Dominant Cell Type

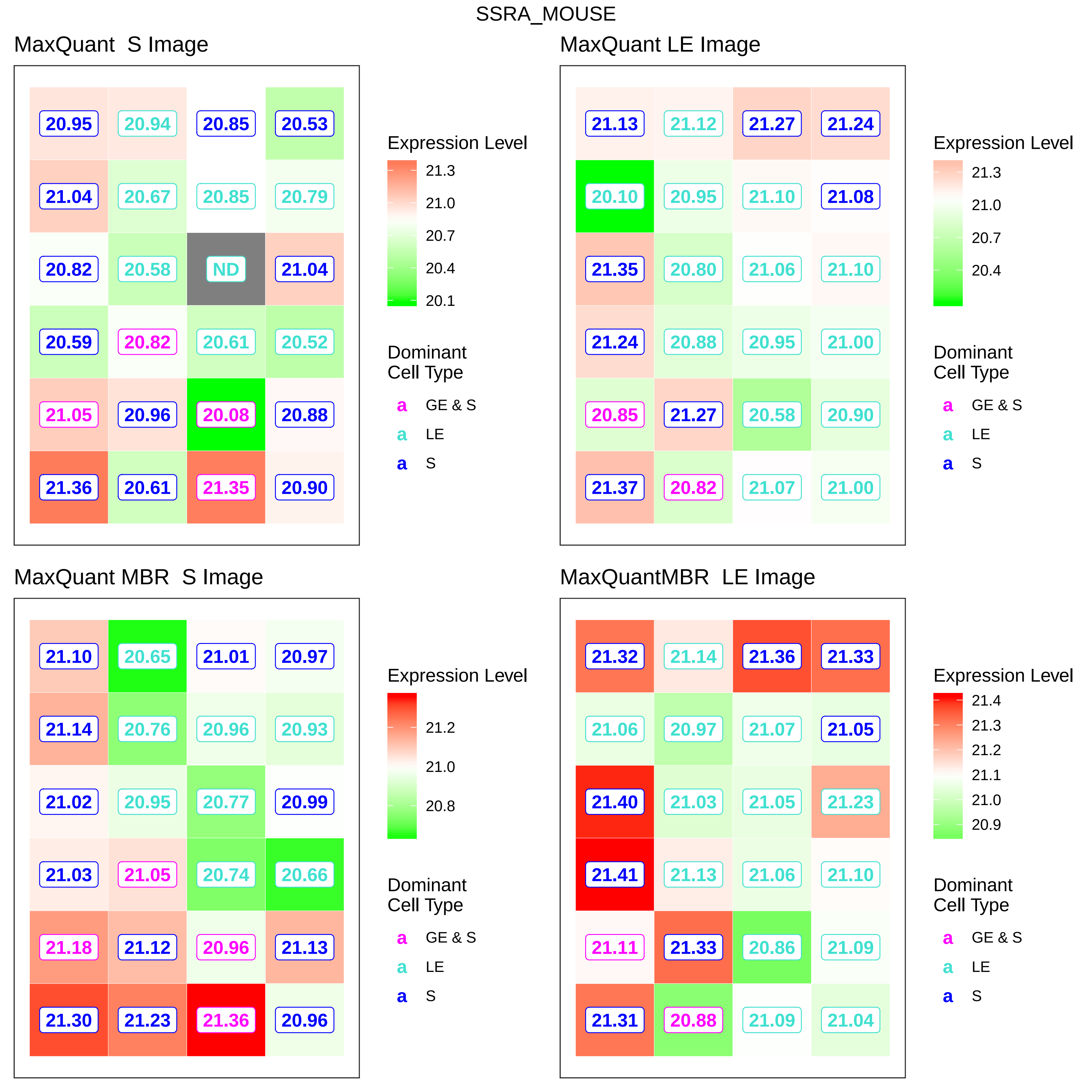
SSRA_MOUSE
MaxQuant S Image
20.95 20.94 20.85 20.53
21.04 20.67 20.85 20.79
20.82 20.58 ND 21.04
20.59 20.82 20.61 20.52
21.05 20.96 20.08 20.88
21.36 20.61 21.35 20.90
Expression Level
21.3
21.0
20.7
20.4
20.1
Dominant Cell Type
a GE & S
a LE
a S
MaxQuant LE Image
21.13 21.12 21.27 21.24
20.10 20.95 21.10 21.08
21.35 20.80 21.06 21.10
21.24 20.88 20.95 21.00
20.85 21.27 20.58 20.90
21.37 20.82 21.07 21.00
Expression Level
21.3
21.0
20.7
20.4
Dominant Cell Type
a GE & S
a LE
a S
MaxQuant MBR S Image
21.10 20.65 21.01 20.97
21.14 20.76 20.96 20.93
21.02 20.95 20.77 20.99
21.03 21.05 20.74 20.66
21.18 21.12 20.96 21.13
21.30 21.23 21.36 20.96
Expression Level
21.2
21.0
20.8
Dominant Cell Type
a GE & S
a LE
a S
MaxQuantMBR LE Image
21.32 21.14 21.36 21.33
21.06 20.97 21.07 21.05
21.40 21.03 21.05 21.23
21.41 21.13 21.06 21.10
21.11 21.33 20.86 21.09
21.31 20.88 21.09 21.04
Expression Level
21.4
21.3
21.2
21.1
21.0
20.9
Dominant Cell Type
a GE & S
a LE
a S

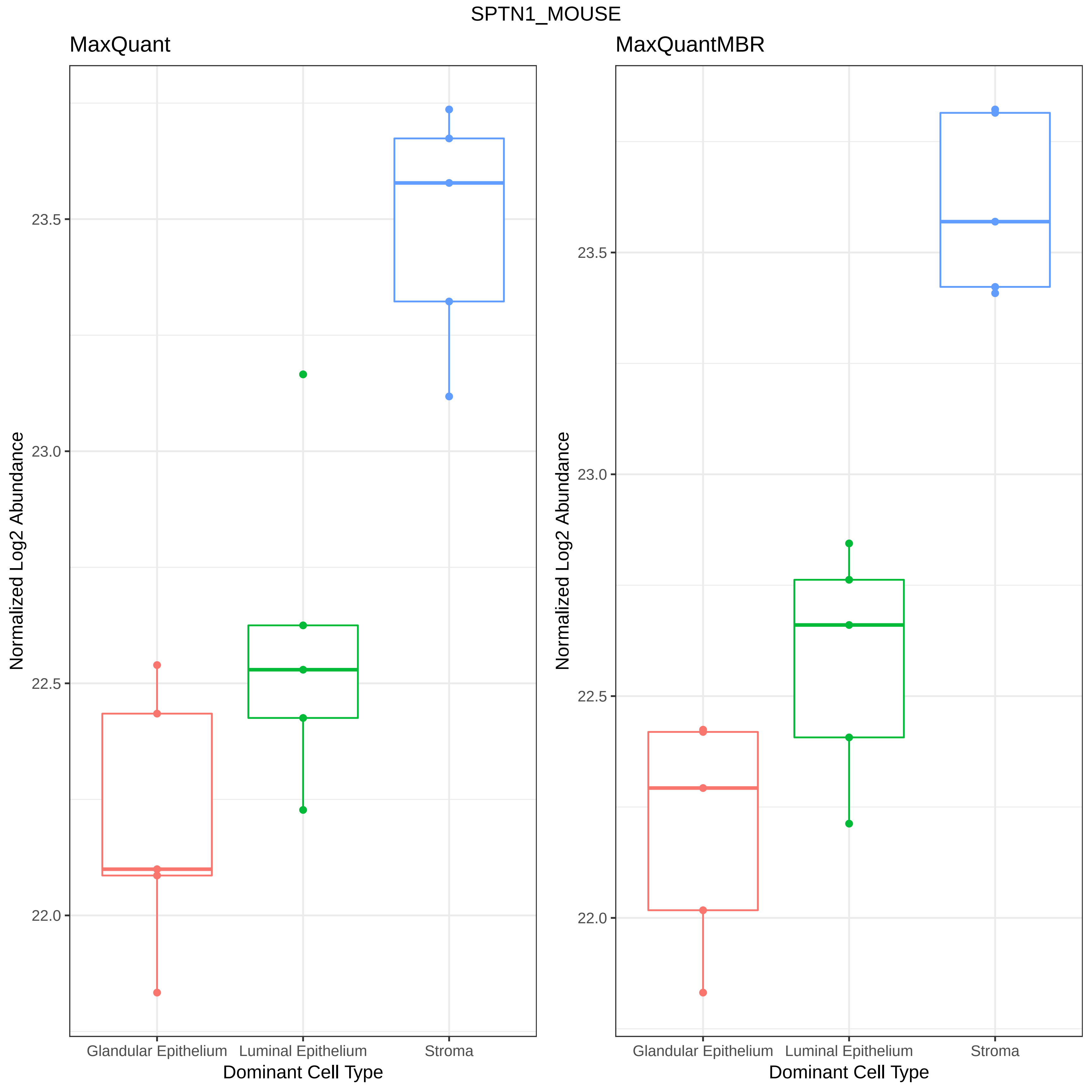
SPTN1_MOUSE
MaxQuant
MaxQuantMBR
Normalized Log2 Abundance
23.5
23.0
22.5
22.0
Glandular Epithelium
Luminal Epithelium
Stroma
Dominant Cell Type

SPTN1_MOUSE

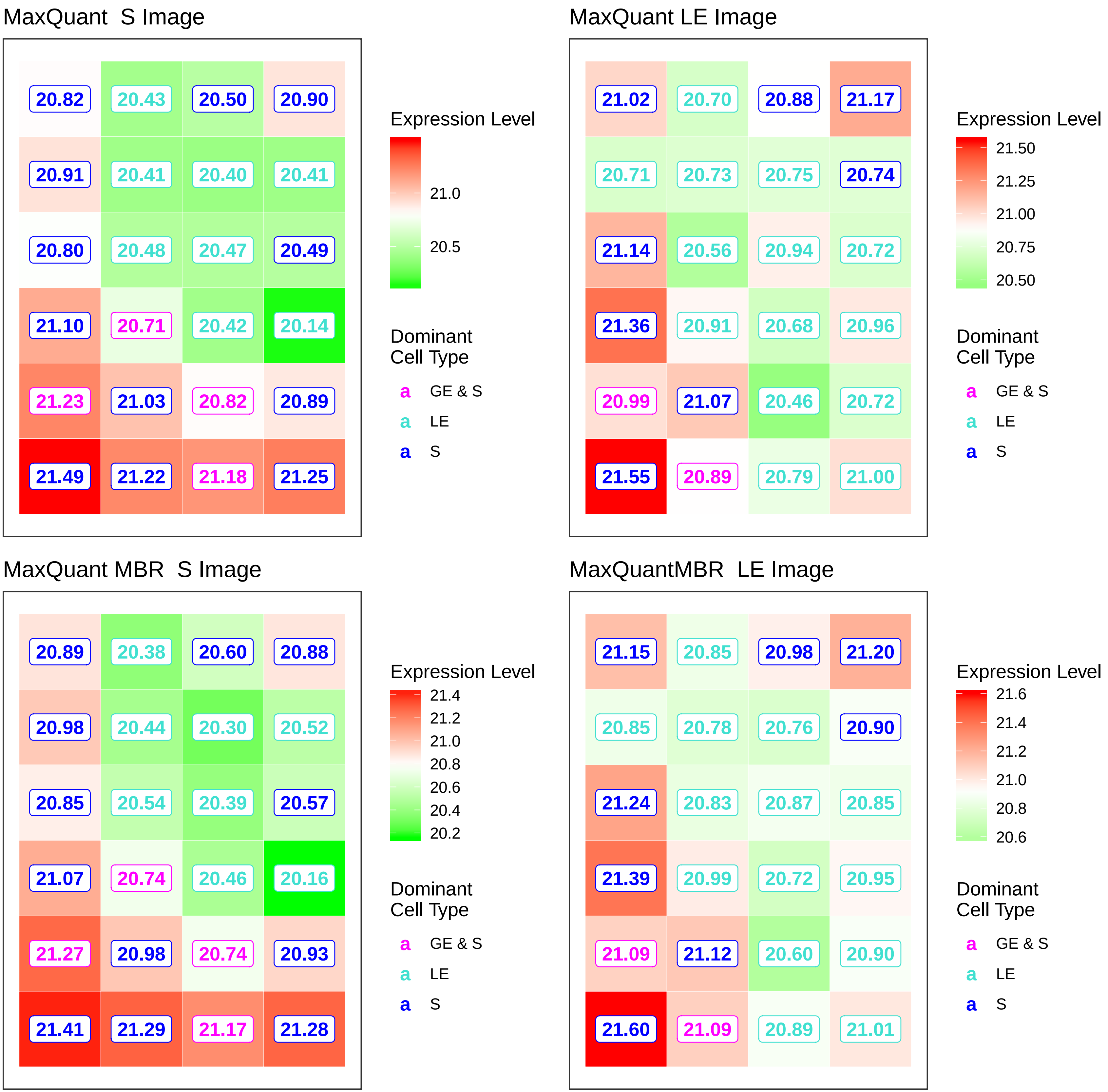
MaxQuant S Image
20.82 20.43 20.50 20.90
20.91 20.41 20.40 20.41
20.80 20.48 20.47 20.49
21.10 20.71 20.42 20.14
21.23 21.03 20.82 20.89
21.49 21.22 21.18 21.25
Expression Level
21.0
20.5
Dominant Cell Type
a GE & S
a LE
a S
MaxQuant LE Image
21.02 20.70 20.88 21.17
20.71 20.73 20.75 20.74
21.14 20.56 20.94 20.72
21.36 20.91 20.68 20.96
20.99 21.07 20.46 20.72
21.55 20.89 20.79 21.00
Expression Level
21.50
21.25
21.00
20.75
20.50
Dominant Cell Type
a GE & S
a LE
a S
MaxQuant MBR S Image
20.89 20.38 20.60 20.88
20.98 20.44 20.30 20.52
20.85 20.54 20.39 20.57
21.07 20.74 20.46 20.16
21.27 20.98 20.74 20.93
21.41 21.29 21.17 21.28
Expression Level
21.4
21.2
21.0
20.8
20.6
20.4
20.2
Dominant Cell Type
a GE & S
a LE
a S
MaxQuantMBR LE Image
21.15 20.85 20.98 21.20
20.85 20.78 20.76 20.90
21.24 20.83 20.87 20.85
21.39 20.99 20.72 20.95
21.09 21.12 20.60 20.90
21.60 21.09 20.89 21.01
Expression Level
21.6
21.4
21.2
21.0
20.8
20.6
Dominant Cell Type
a GE & S
a LE
a S

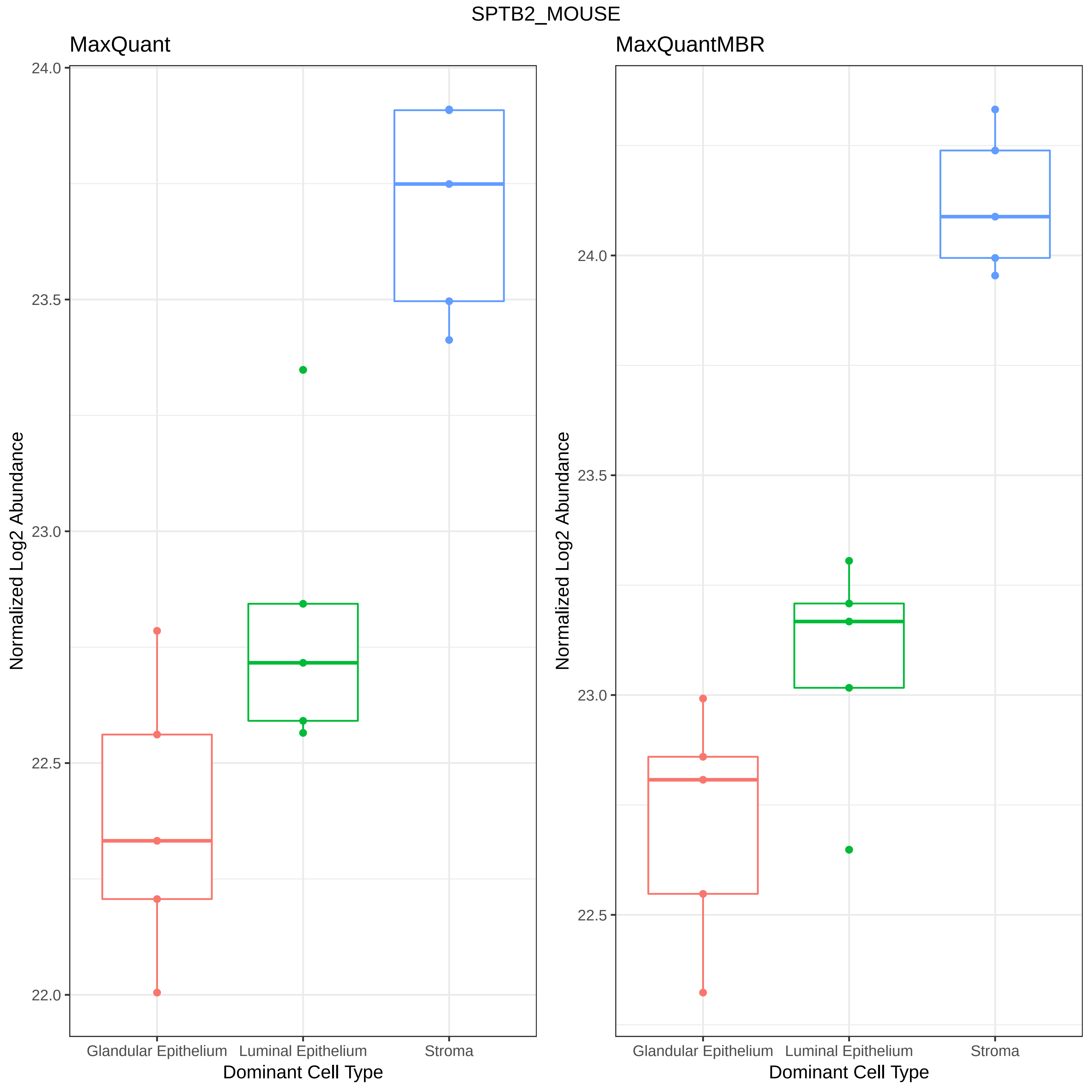
SPTB2_MOUSE
MaxQuant
MaxQuantMBR
Normalized Log2 Abundance
24.0
23.5
23.0
22.5
22.0
Glandular Epithelium
Luminal Epithelium
Stroma
Dominant Cell Type

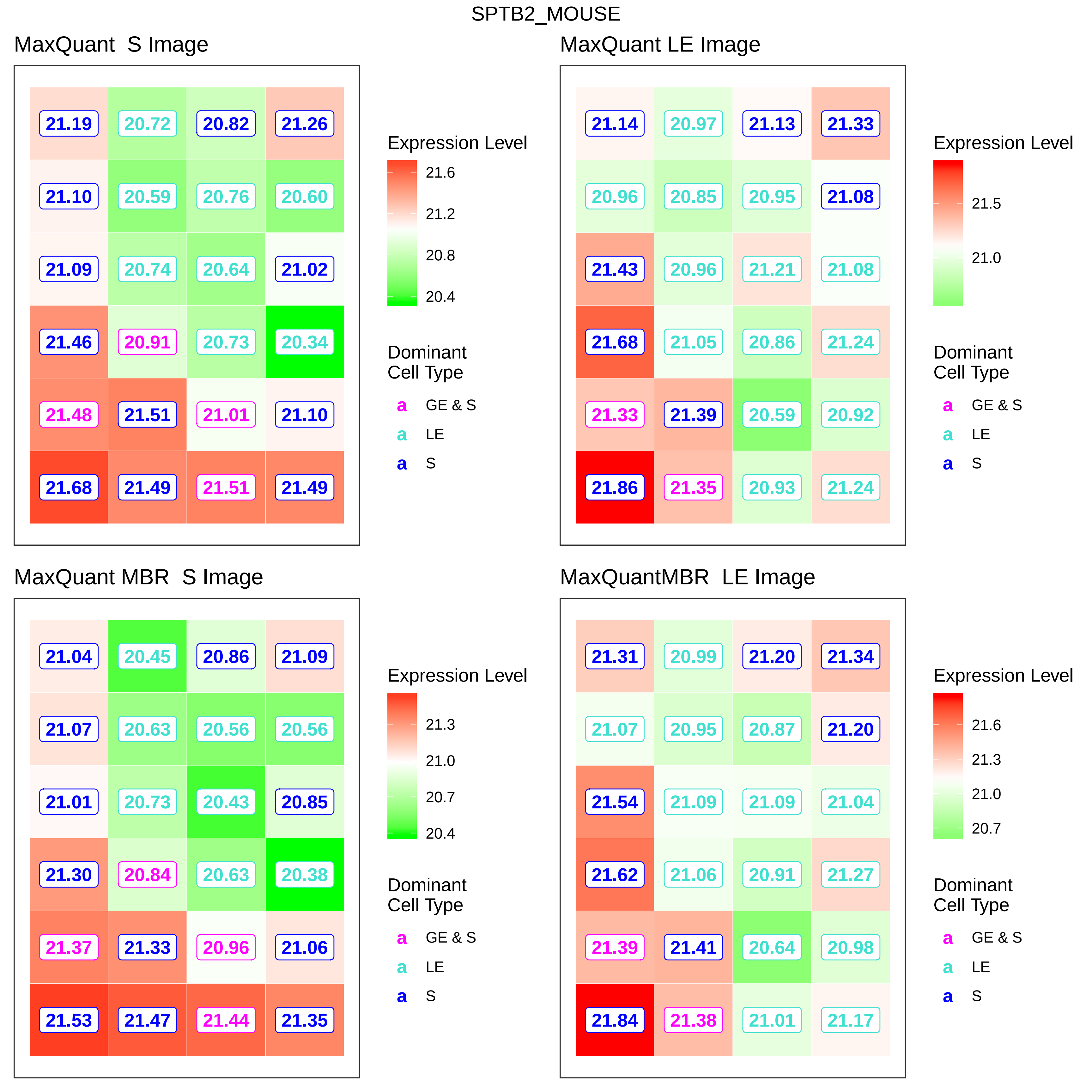
SPTB2_MOUSE
MaxQuant S Image
21.19 20.72 20.82 21.26
21.10 20.59 20.76 20.60
21.09 20.74 20.64 21.02
21.46 20.91 20.73 20.34
21.48 21.51 21.01 21.10
21.68 21.49 21.51 21.49
Expression Level
21.6
21.2
20.8
20.4
Dominant Cell Type
a GE & S
a LE
a S
MaxQuant LE Image
21.14 20.97 21.13 21.33
20.96 20.85 20.95 21.08
21.43 20.96 21.21 21.08
21.68 21.05 20.86 21.24
21.33 21.39 20.59 20.92
21.86 21.35 20.93 21.24
Expression Level
21.5
21.0
Dominant Cell Type
a GE & S
a LE
a S
MaxQuant MBR S Image
21.04 20.45 20.86 21.09
21.07 20.63 20.56 20.56
21.01 20.73 20.43 20.85
21.30 20.84 20.63 20.38
21.37 21.33 20.96 21.06
21.53 21.47 21.44 21.35
Expression Level
21.3
21.0
20.7
20.4
Dominant Cell Type
a GE & S
a LE
a S
MaxQuantMBR LE Image
21.31 20.99 21.20 21.34
21.07 20.95 20.87 21.20
21.54 21.09 21.09 21.04
21.62 21.06 20.91 21.27
21.39 21.41 20.64 20.98
21.84 21.38 21.01 21.17
Expression Level
21.6
21.3
21.0
20.7
Dominant Cell Type
a GE & S
a LE
a S

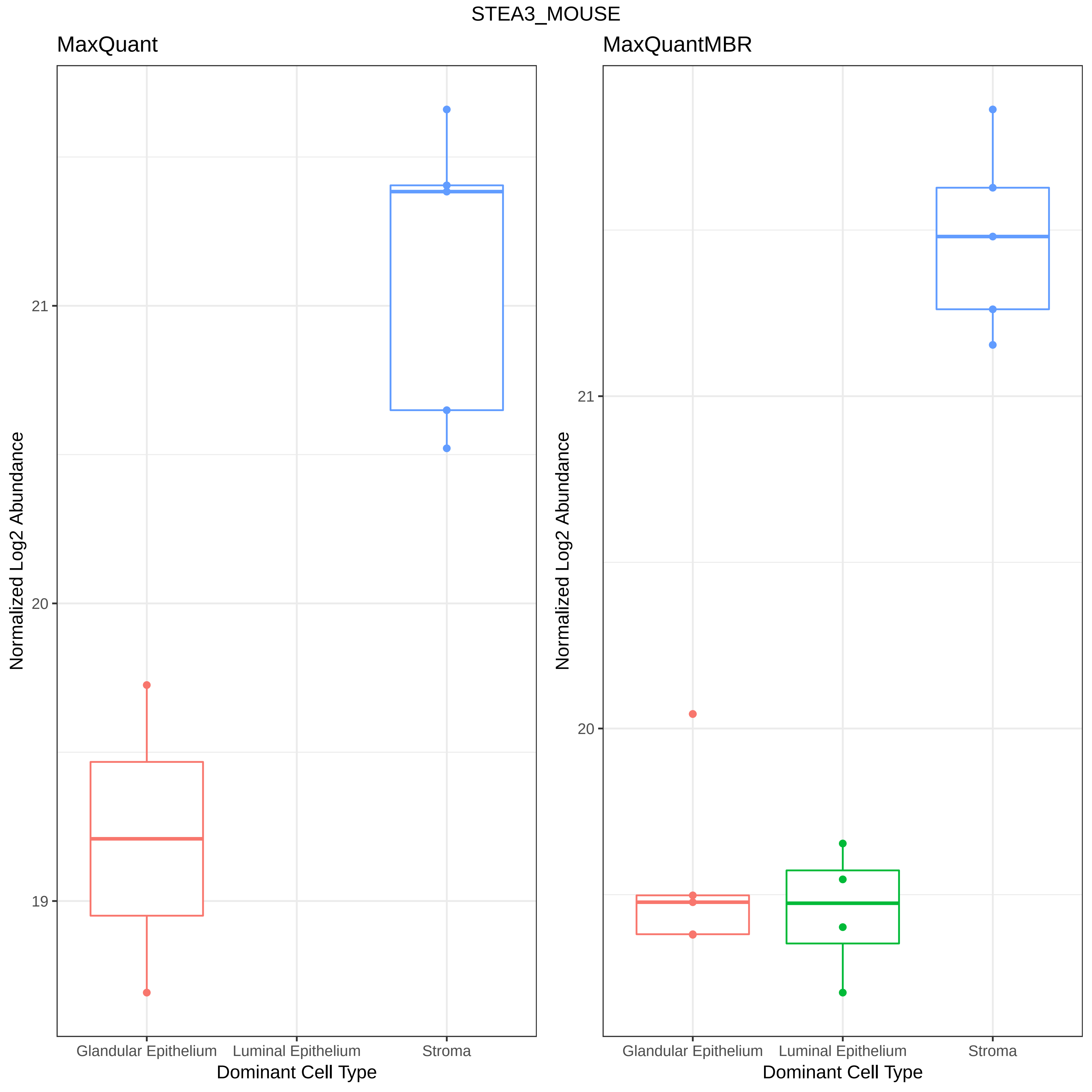

STEA3_MOUSE
MaxQuant
MaxQuantMBR
Normalized Log2 Abundance
21
20
19
Glandular Epithelium
Luminal Epithelium
Stroma
Dominant Cell Type

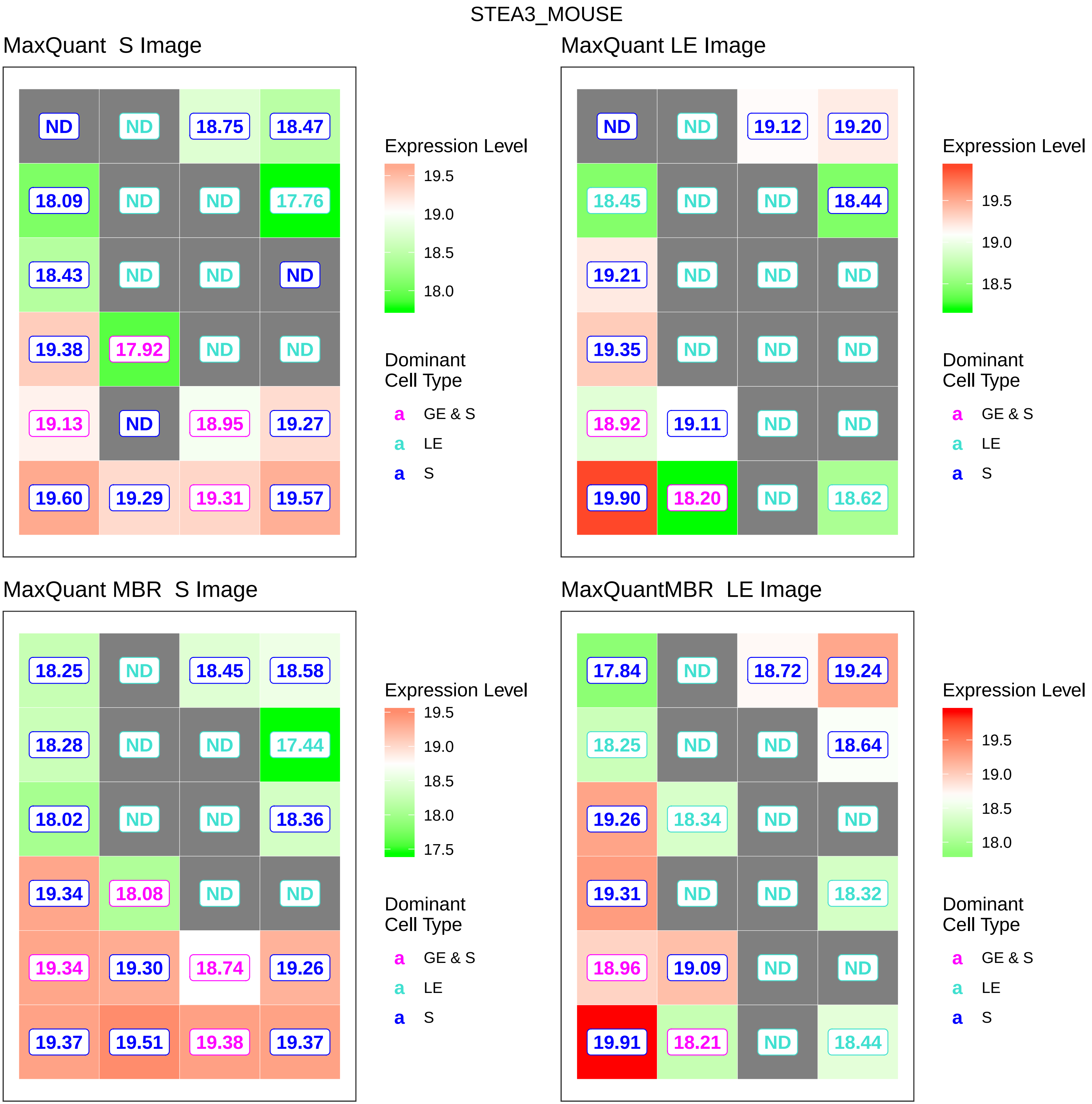
STEA3_MOUSE
MaxQuant S Image
ND ND 18.75 18.47
18.09 ND ND 17.76
18.43 ND ND ND
19.38 17.92 ND ND
19.13 ND 18.95 19.27
19.60 19.29 19.31 19.57
Expression Level
19.5
19.0
18.5
18.0
Dominant Cell Type
a GE & S
a LE
a S
MaxQuant LE Image
ND ND 19.12 19.20
18.45 ND ND 18.44
19.21 ND ND ND
19.35 ND ND ND
18.92 19.11 ND ND
19.90 18.20 ND 18.62
Expression Level
19.5
19.0
18.5
Dominant Cell Type
a GE & S
a LE
a S
MaxQuant MBR S Image
18.25 ND 18.45 18.58
18.28 ND ND 17.44
18.02 ND ND 18.36
19.34 18.08 ND ND
19.34 19.30 18.74 19.26
19.37 19.51 19.38 19.37
Expression Level
19.5
19.0
18.5
18.0
17.5
Dominant Cell Type
a GE & S
a LE
a S
MaxQuantMBR LE Image
17.84 ND 18.72 19.24
18.25 ND ND 18.64
19.26 18.34 ND ND
19.31 ND ND 18.32
18.96 19.09 ND ND
19.91 18.21 ND 18.44
Expression Level
19.5
19.0
18.5
18.0
Dominant Cell Type
a GE & S
a LE
a S

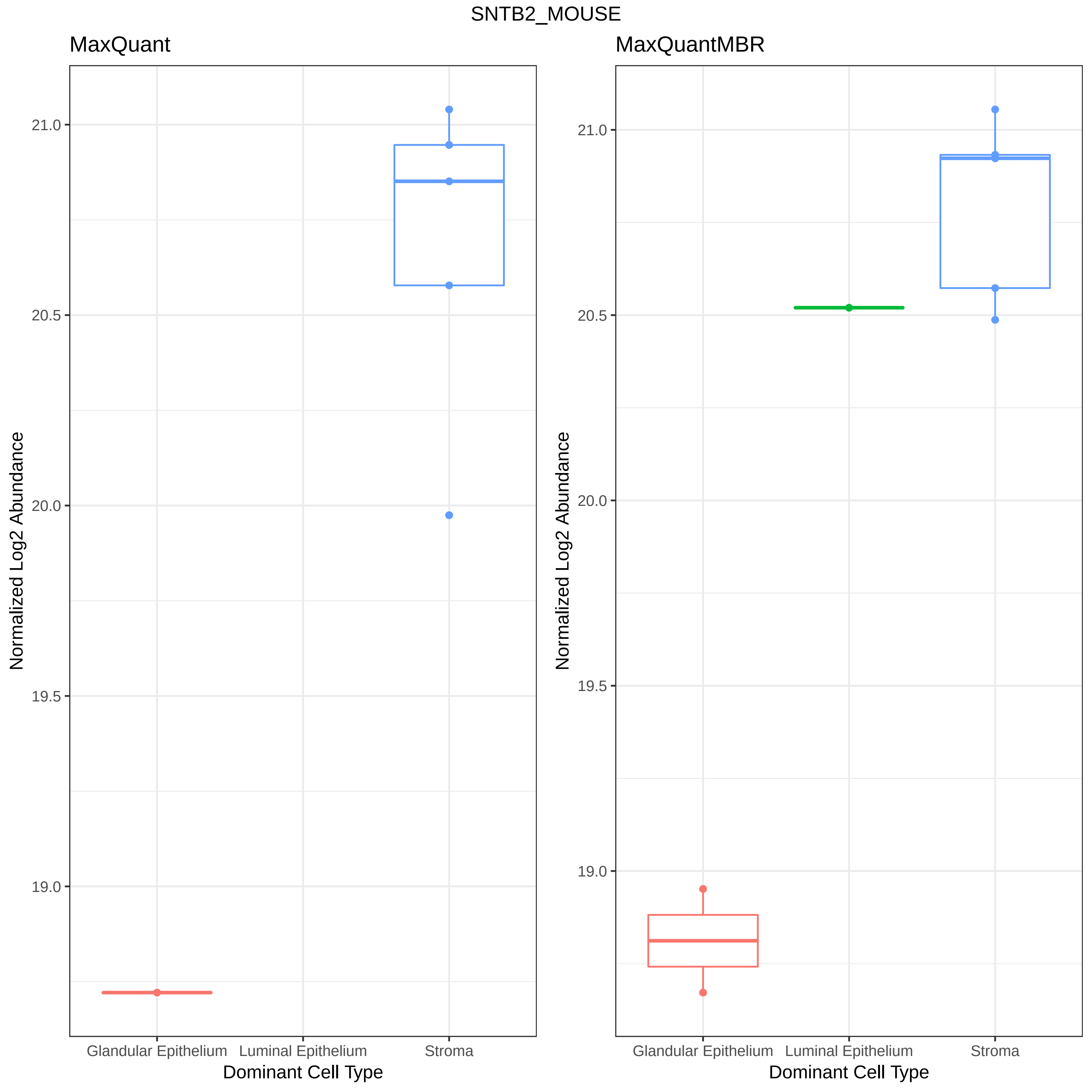
SNTB2_MOUSE
MaxQuant
MaxQuantMBR
Normalized Log2 Abundance
21.0
20.5
20.0
19.5
19.0
Glandular Epithelium
Luminal Epithelium
Stroma
Dominant Cell Type

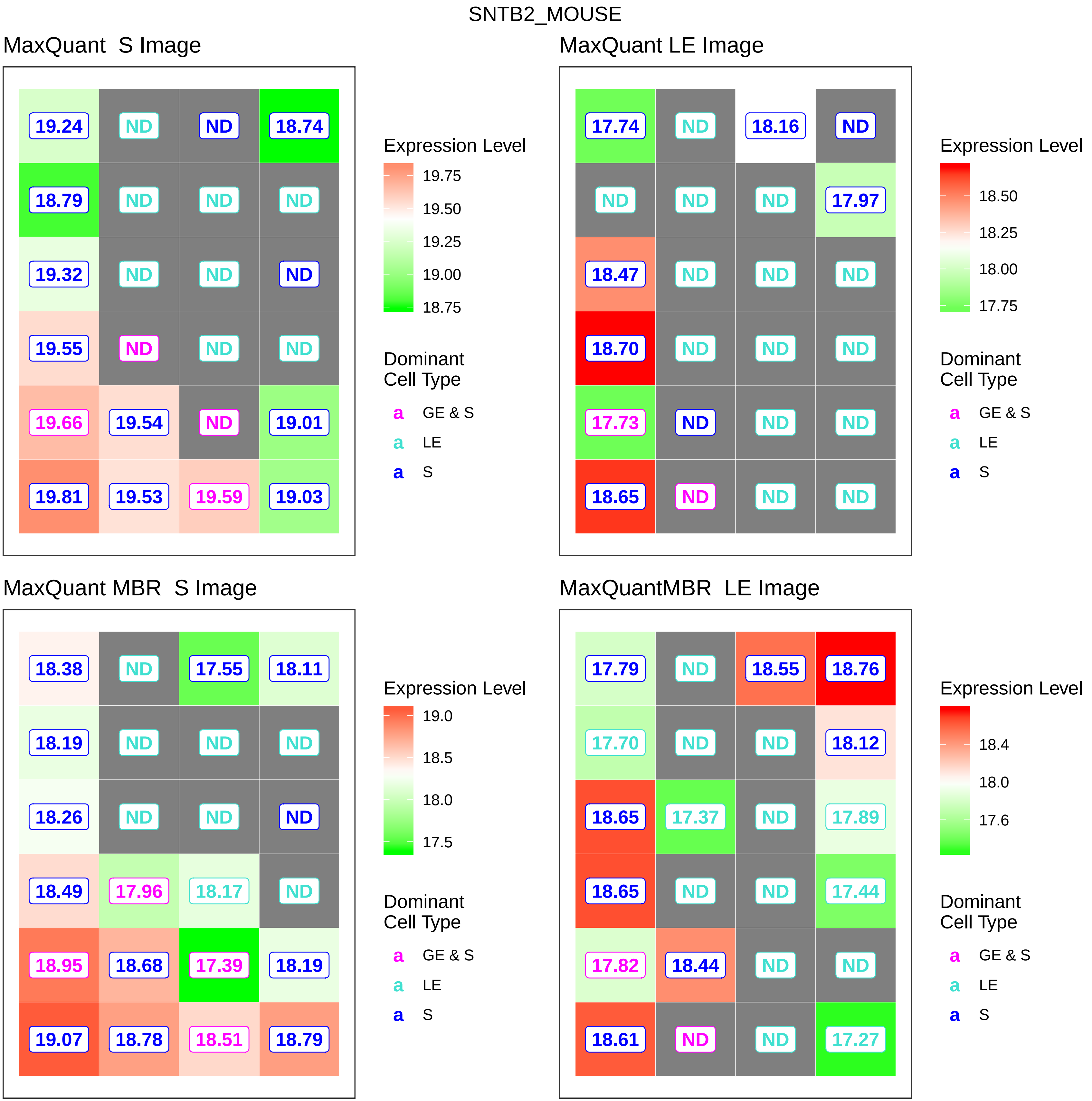
SNTB2_MOUSE
MaxQuant S Image
19.24 ND ND 18.74
18.79 ND ND ND
19.32 ND ND ND
19.55 ND ND ND
19.66 19.54 ND 19.01
19.81 19.53 19.59 19.03
Expression Level
19.75
19.50
19.25
19.00
18.75
Dominant Cell Type
a GE & S
a LE
a S
MaxQuant LE Image
17.74 ND 18.16 ND
ND ND ND 17.97
18.47 ND ND ND
18.70 ND ND ND
17.73 ND ND ND
18.65 ND ND ND
Expression Level
18.50
18.25
18.00
17.75
Dominant Cell Type
a GE & S
a LE
a S
MaxQuant MBR S Image
18.38 ND 17.55 18.11
18.19 ND ND ND
18.26 ND ND ND
18.49 17.96 18.17 ND
18.95 18.68 17.39 18.19
19.07 18.78 18.51 18.79
Expression Level
19.0
18.5
18.0
17.5
Dominant Cell Type
a GE & S
a LE
a S
MaxQuantMBR LE Image
17.79 ND 18.55 18.76
17.70 ND ND 18.12
18.65 17.37 ND 17.89
18.65 ND ND 17.44
17.82 18.44 ND ND
18.61 ND ND 17.27
Expression Level
18.4
18.0
17.6
Dominant Cell Type
a GE & S
a LE
a S

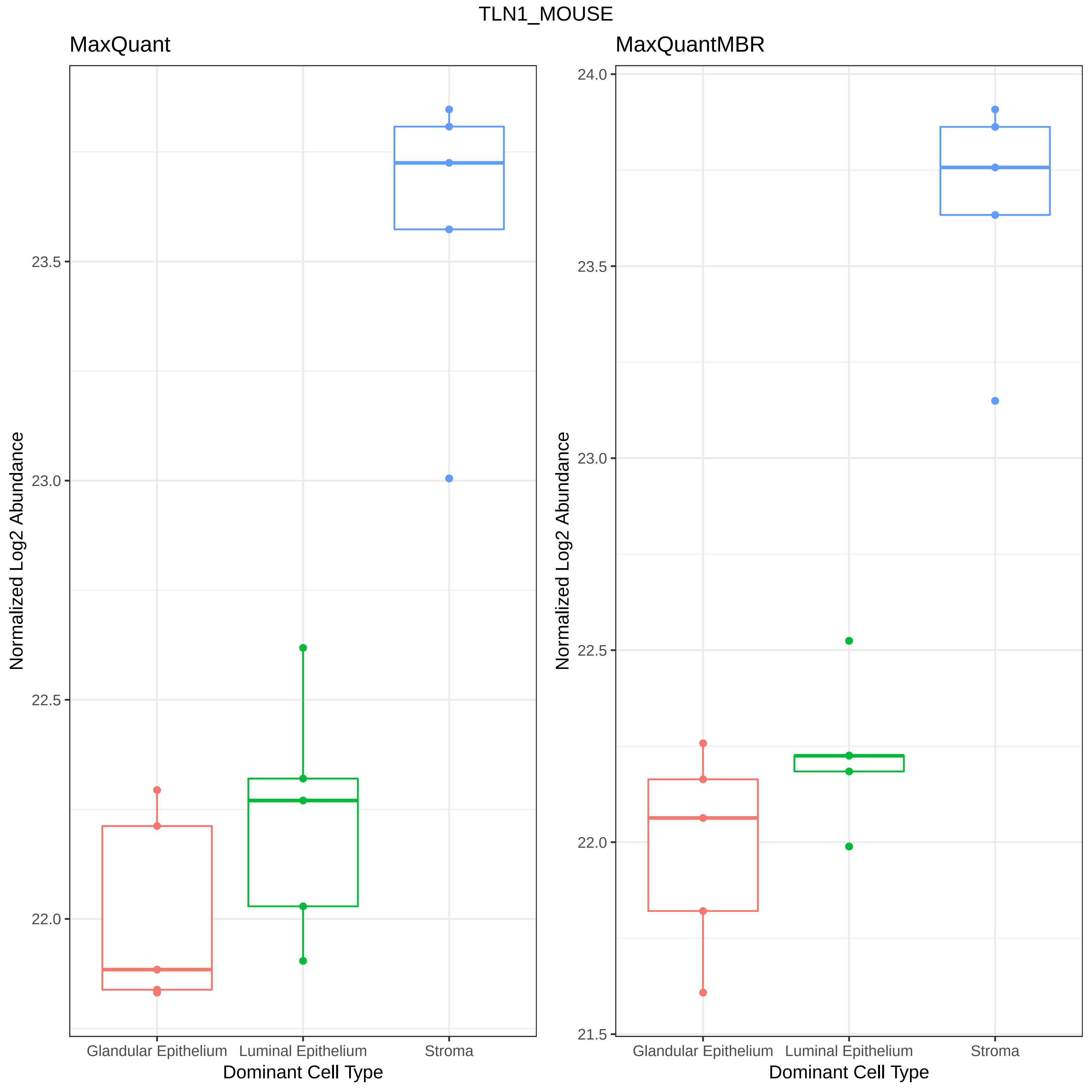

TLN1_MOUSE
MaxQuant
MaxQuantMBR
Normalized Log2 Abundance
23.5
23.0
22.5
22.0
24.0
21.5
Glandular Epithelium
Luminal Epithelium
Stroma
Dominant Cell Type

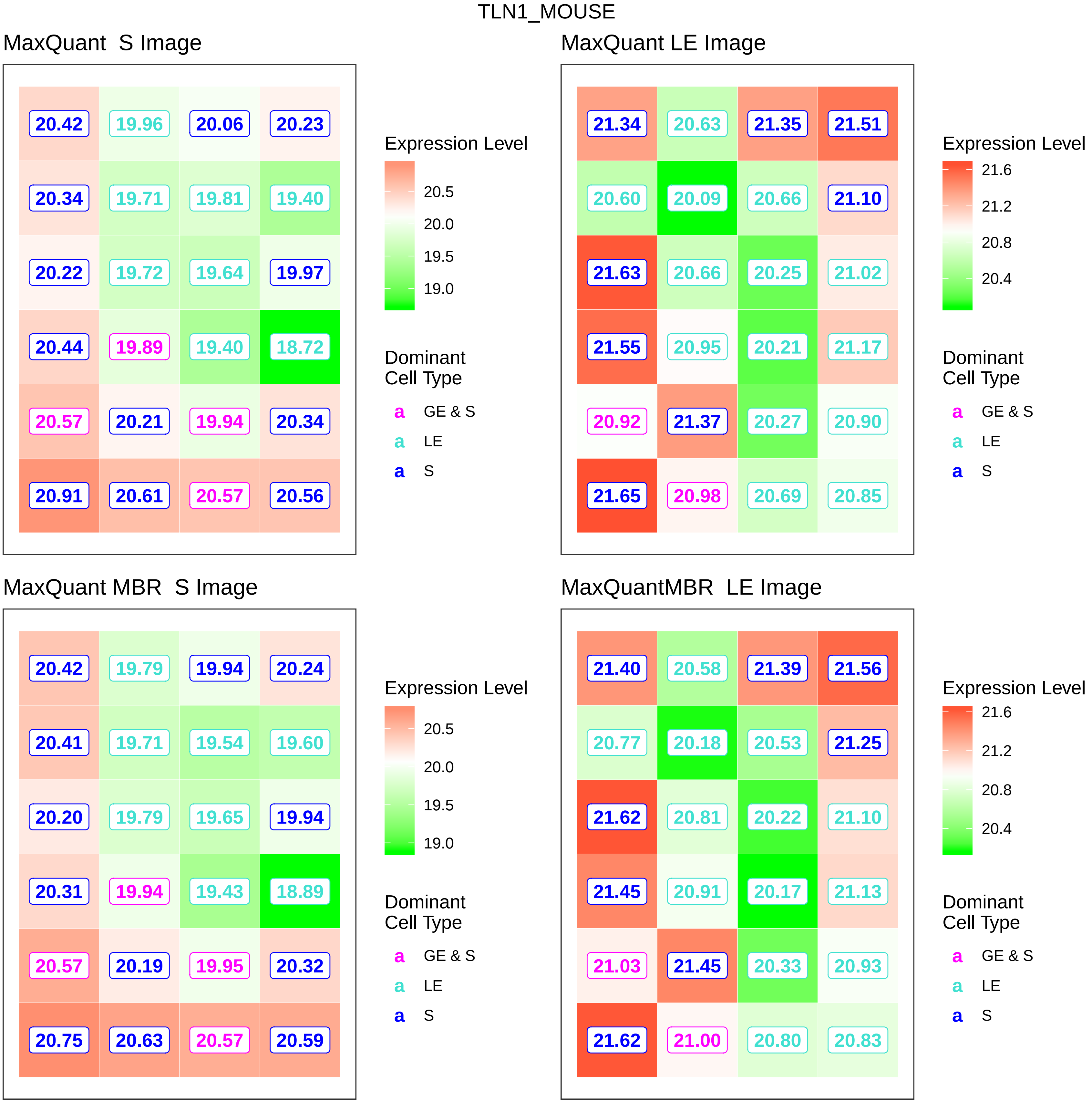
TLN1_MOUSE
MaxQuant S Image
20.42 19.96 20.06 20.23
20.34 19.71 19.81 19.40
20.22 19.72 19.64 19.97
20.44 19.89 19.40 18.72
20.57 20.21 19.94 20.34
20.91 20.61 20.57 20.56
Expression Level
20.5
20.0
19.5
19.0
Dominant Cell Type
a GE & S
a LE
a S
MaxQuant LE Image
21.34 20.63 21.35 21.51
20.60 20.09 20.66 21.10
21.63 20.66 20.25 21.02
21.55 20.95 20.21 21.17
20.92 21.37 20.27 20.90
21.65 20.98 20.69 20.85
Expression Level
21.6
21.2
20.8
20.4
Dominant Cell Type
a GE & S
a LE
a S
MaxQuant MBR S Image
20.42 19.79 19.94 20.24
20.41 19.71 19.54 19.60
20.20 19.79 19.65 19.94
20.31 19.94 19.43 18.89
20.57 20.19 19.95 20.32
20.75 20.63 20.57 20.59
Expression Level
20.5
20.0
19.5
19.0
Dominant Cell Type
a GE & S
a LE
a S
MaxQuantMBR LE Image
21.40 20.58 21.39 21.56
20.77 20.18 20.53 21.25
21.62 20.81 20.22 21.10
21.45 20.91 20.17 21.13
21.03 21.45 20.33 20.93
21.62 21.00 20.80 20.83
Expression Level
21.6
21.2
20.8
20.4
Dominant Cell Type
a GE & S
a LE
a S

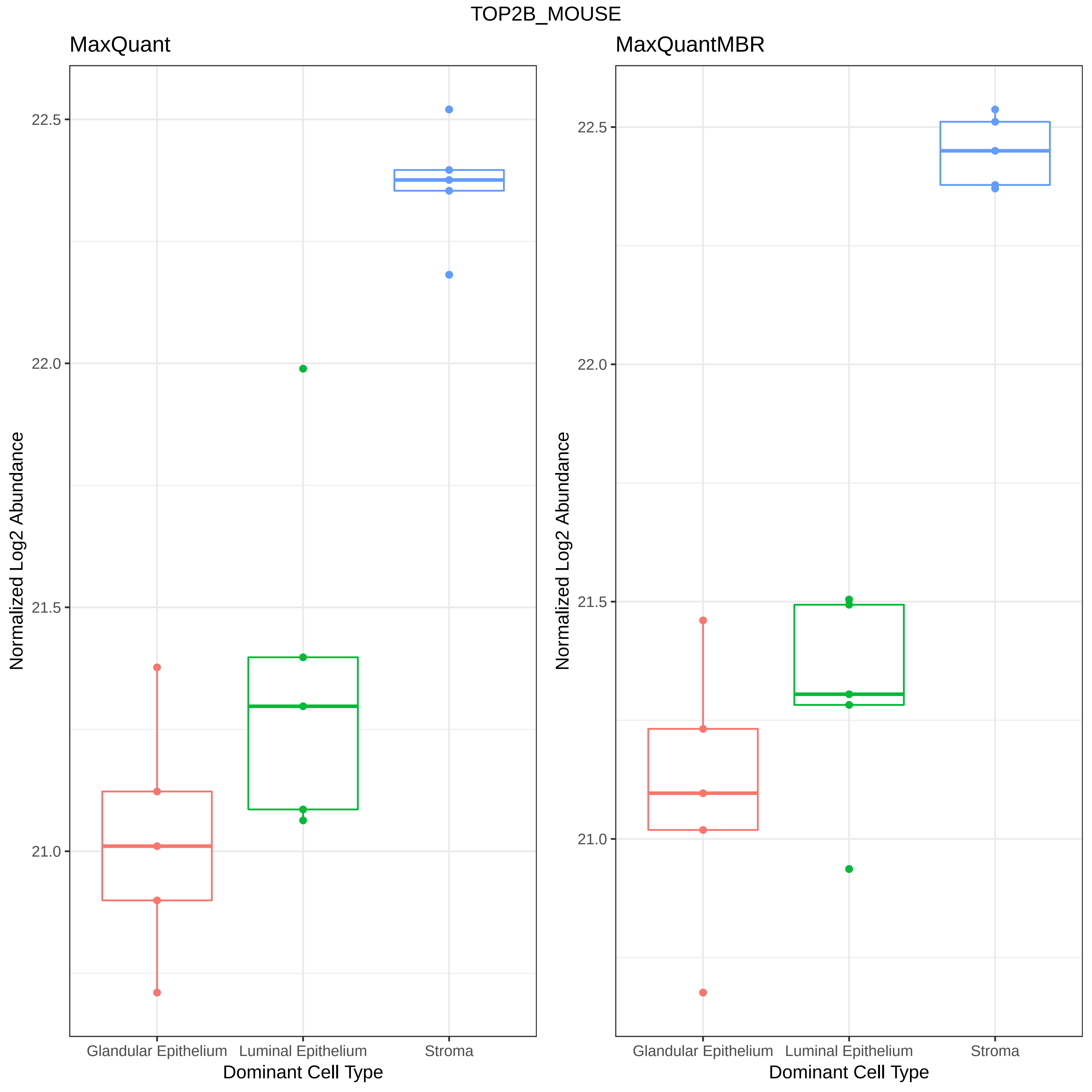
TOP2B_MOUSE
MaxQuant
MaxQuantMBR
Normalized Log2 Abundance
22.5
22.0
21.5
21.0
Glandular Epithelium
Luminal Epithelium
Stroma
Dominant Cell Type

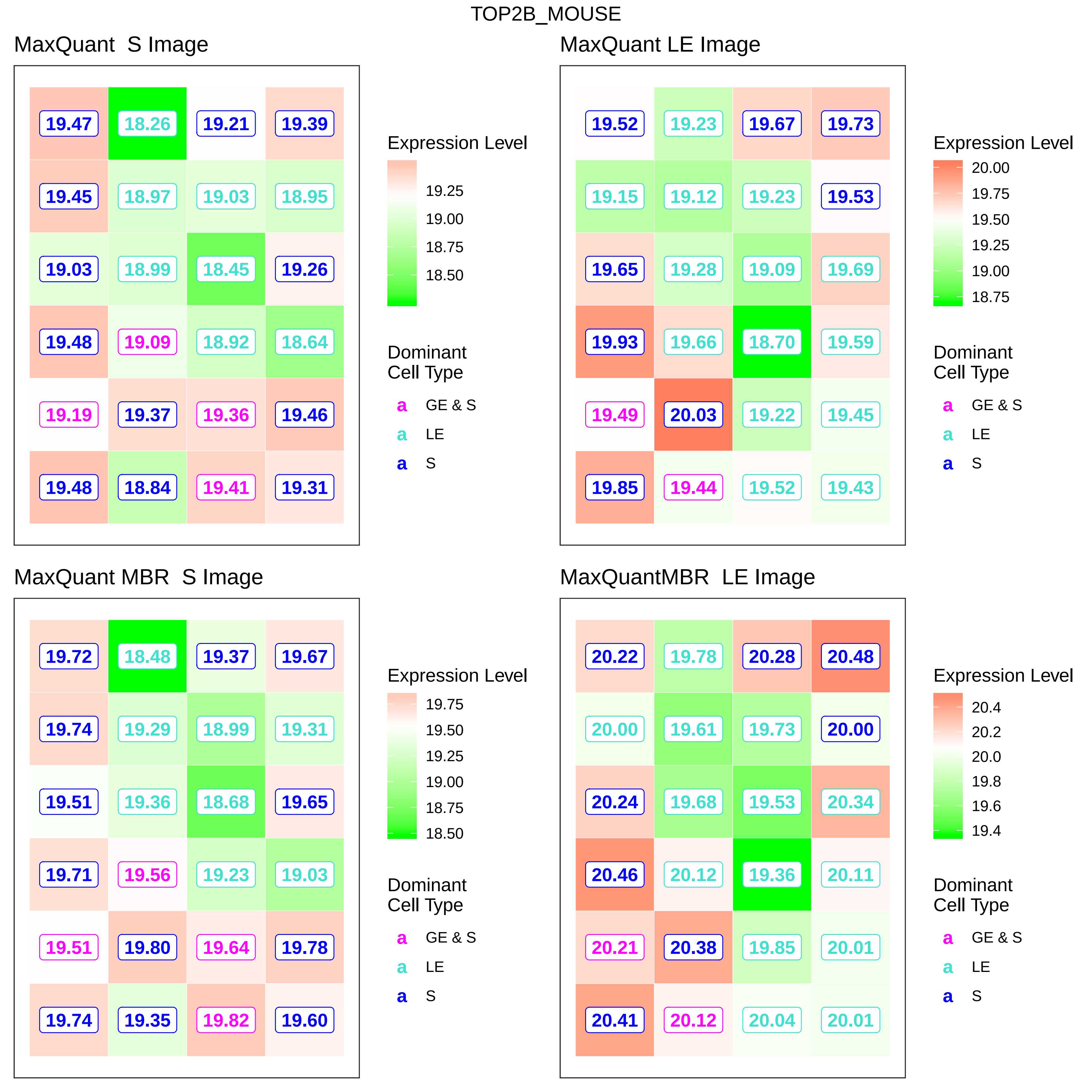
TOP2B_MOUSE
MaxQuant S Image
19.47 18.26 19.21 19.39
19.45 18.97 19.03 18.95
19.03 18.99 18.45 19.26
19.48 19.09 18.92 18.64
19.19 19.37 19.36 19.46
19.48 18.84 19.41 19.31
Expression Level
19.25
19.00
18.75
18.50
Dominant Cell Type
a GE & S
a LE
a S
MaxQuant LE Image
19.52 19.23 19.67 19.73
19.15 19.12 19.23 19.53
19.65 19.28 19.09 19.69
19.93 19.66 18.70 19.59
19.49 20.03 19.22 19.45
19.85 19.44 19.52 19.43
Expression Level
20.00
19.75
19.50
19.25
19.00
18.75
Dominant Cell Type
a GE & S
a LE
a S
MaxQuant MBR S Image
19.72 18.48 19.37 19.67
19.74 19.29 18.99 19.31
19.51 19.36 18.68 19.65
19.71 19.56 19.23 19.03
19.51 19.80 19.64 19.78
19.74 19.35 19.82 19.60
Expression Level
19.75
19.50
19.25
19.00
18.75
18.50
Dominant Cell Type
a GE & S
a LE
a S
MaxQuantMBR LE Image
20.22 19.78 20.28 20.48
20.00 19.61 19.73 20.00
20.24 19.68 19.53 20.34
20.46 20.12 19.36 20.11
20.21 20.38 19.85 20.01
20.41 20.12 20.04 20.01
Expression Level
20.4
20.2
20.0
19.8
19.6
19.4
Dominant Cell Type
a GE & S
a LE
a S

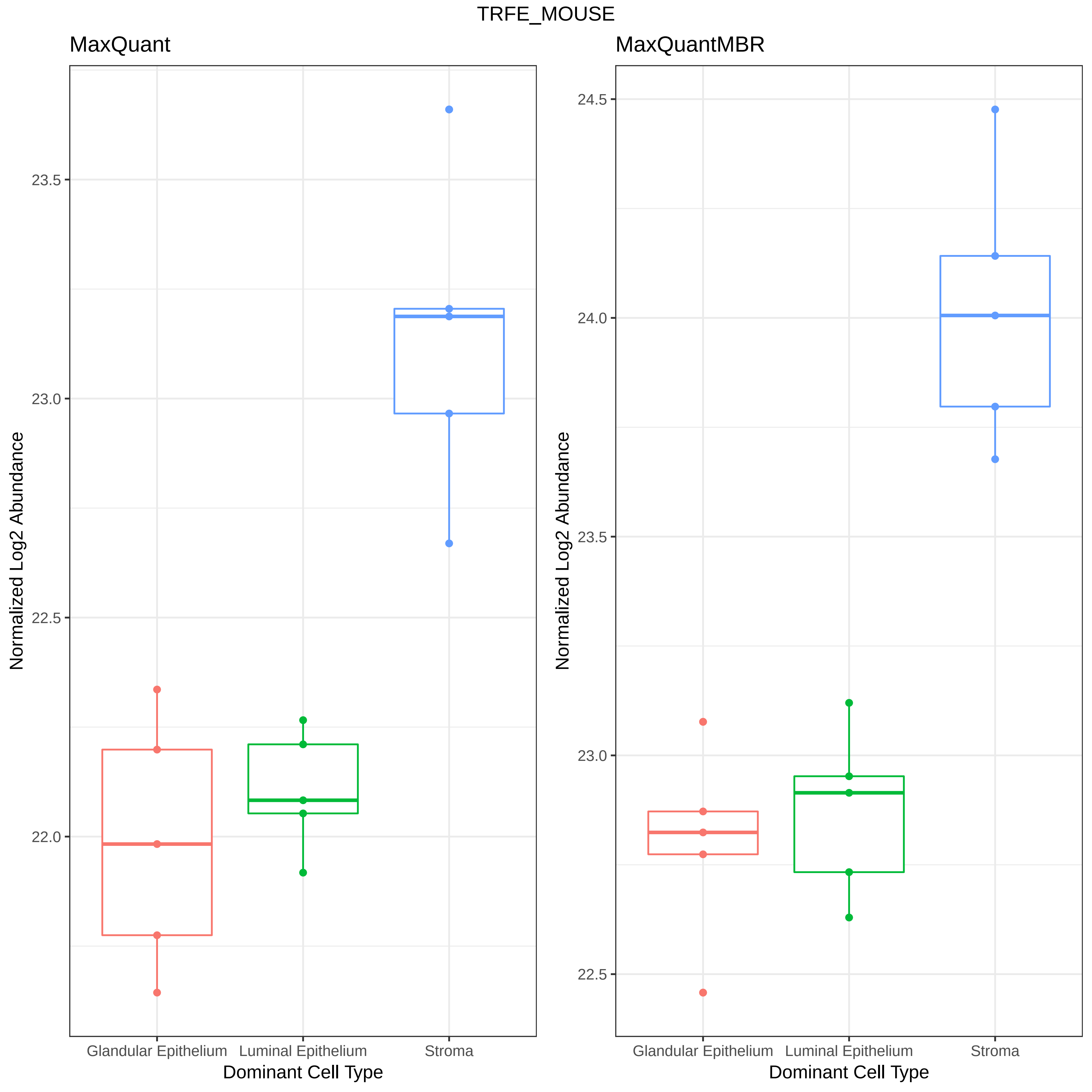

TRFE_MOUSE
MaxQuant
MaxQuantMBR
Normalized Log2 Abundance
23.5
23.0
22.5
22.0
24.5
24.0
Glandular Epithelium
Luminal Epithelium
Stroma
Dominant Cell Type

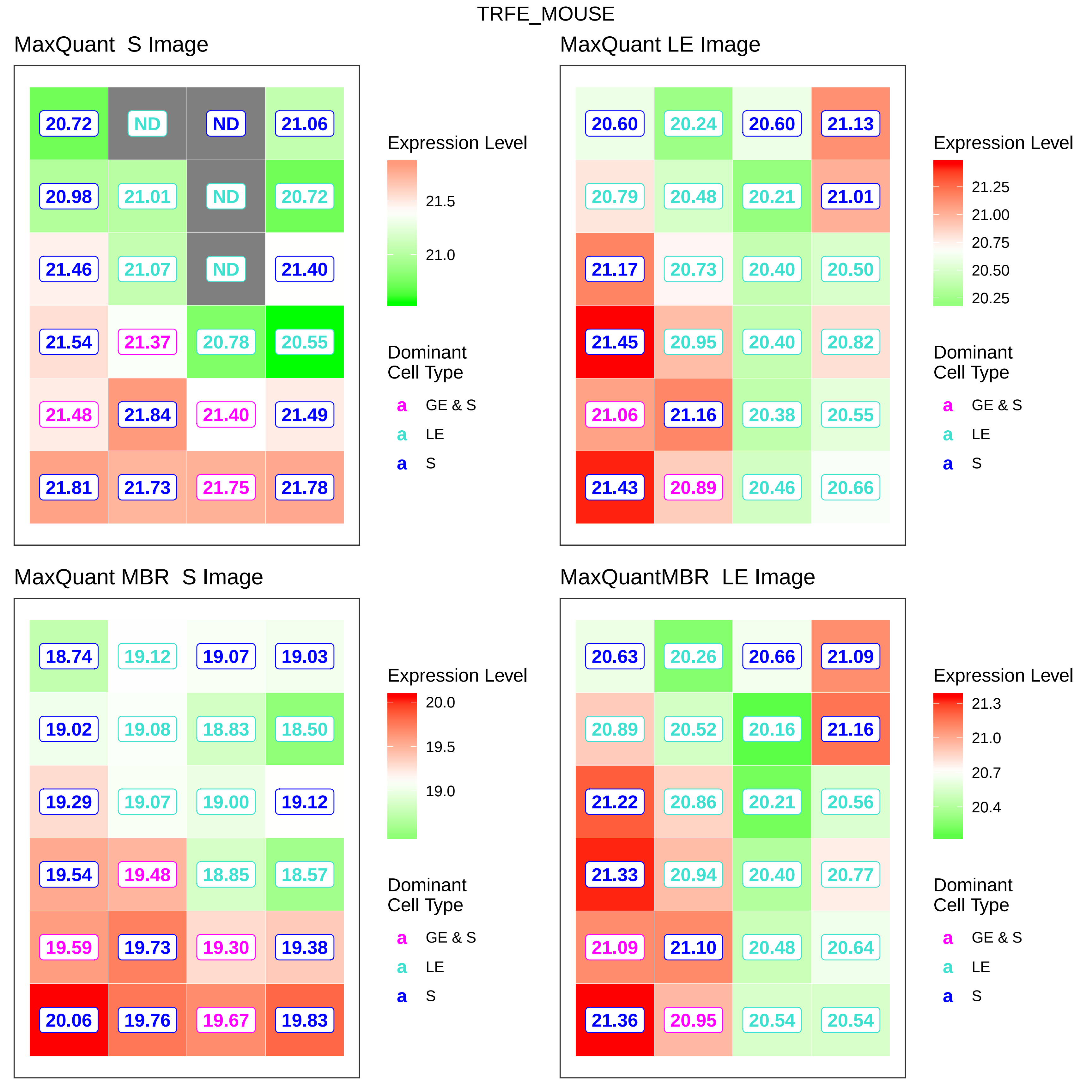
TRFE_MOUSE
MaxQuant S Image
20.72 ND ND 21.06
20.98 21.01 ND 20.72
21.46 21.07 ND 21.40
21.54 21.37 20.78 20.55
21.48 21.84 21.40 21.49
21.81 21.73 21.75 21.78
Expression Level
21.5
21.0
Dominant Cell Type
a GE & S
a LE
a S
MaxQuant LE Image
20.60 20.24 20.60 21.13
20.79 20.48 20.21 21.01
21.17 20.73 20.40 20.50
21.45 20.95 20.40 20.82
21.06 21.16 20.38 20.55
21.43 20.89 20.46 20.66
Expression Level
21.25
21.00
20.75
20.50
20.25
Dominant Cell Type
a GE & S
a LE
a S
MaxQuant MBR S Image
18.74 19.12 19.07 19.03
19.02 19.08 18.83 18.50
19.29 19.07 19.00 19.12
19.54 19.48 18.85 18.57
19.59 19.73 19.30 19.38
20.06 19.76 19.67 19.83
Expression Level
20.0
19.5
19.0
Dominant Cell Type
a GE & S
a LE
a S
MaxQuantMBR LE Image
20.63 20.26 20.66 21.09
20.89 20.52 20.16 21.16
21.22 20.86 20.21 20.56
21.33 20.94 20.40 20.77
21.09 21.10 20.48 20.64
21.36 20.95 20.54 20.54
Expression Level
21.3
21.0
20.7
20.4
Dominant Cell Type
a GE & S
a LE
a S

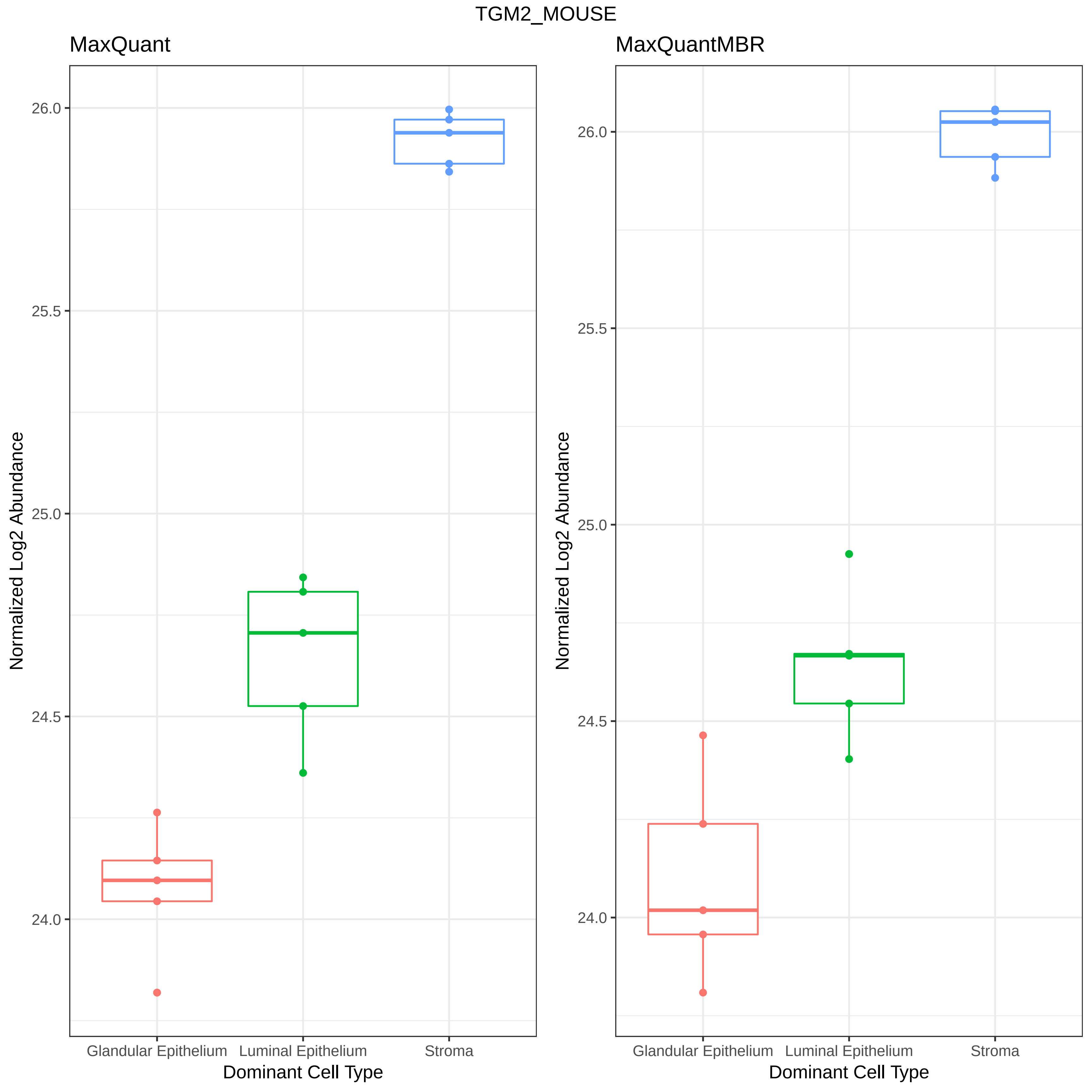

TGM2_MOUSE
MaxQuant
MaxQuantMBR
Normalized Log2 Abundance
26.0
25.5
25.0
24.5
24.0
Glandular Epithelium
Luminal Epithelium
Stroma
Dominant Cell Type

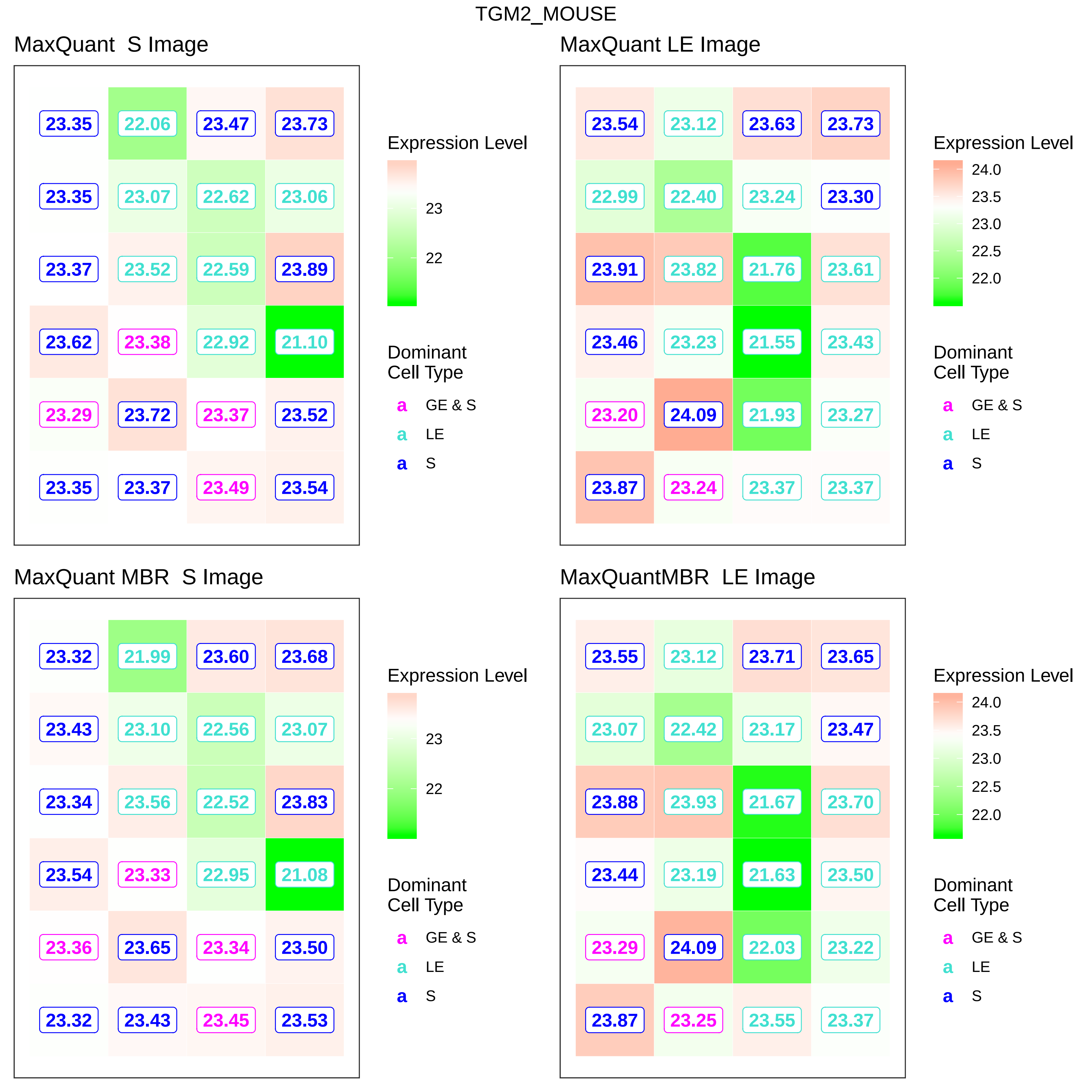
TGM2_MOUSE
MaxQuant S Image
23.35 22.06 23.47 23.73
23.35 23.07 22.62 23.06
23.37 23.52 22.59 23.89
23.62 23.38 22.92 21.10
23.29 23.72 23.37 23.52
23.35 23.37 23.49 23.54
Expression Level
23
22
Dominant Cell Type
a GE & S
a LE
a S
MaxQuant LE Image
23.54 23.12 23.63 23.73
22.99 22.40 23.24 23.30
23.91 23.82 21.76 23.61
23.46 23.23 21.55 23.43
23.20 24.09 21.93 23.27
23.87 23.24 23.37 23.37
Expression Level
24.0
23.5
23.0
22.5
22.0
Dominant Cell Type
a GE & S
a LE
a S
MaxQuant MBR S Image
23.32 21.99 23.60 23.68
23.43 23.10 22.56 23.07
23.34 23.56 22.52 23.83
23.54 23.33 22.95 21.08
23.36 23.65 23.34 23.50
23.32 23.43 23.45 23.53
Expression Level
23
22
Dominant Cell Type
a GE & S
a LE
a S
MaxQuantMBR LE Image
23.55 23.12 23.71 23.65
23.07 22.42 23.17 23.47
23.88 23.93 21.67 23.70
23.44 23.19 21.63 23.50
23.29 24.09 22.03 23.22
23.87 23.25 23.55 23.37
Expression Level
24.0
23.5
23.0
22.5
22.0
Dominant Cell Type
a GE & S
a LE
a S

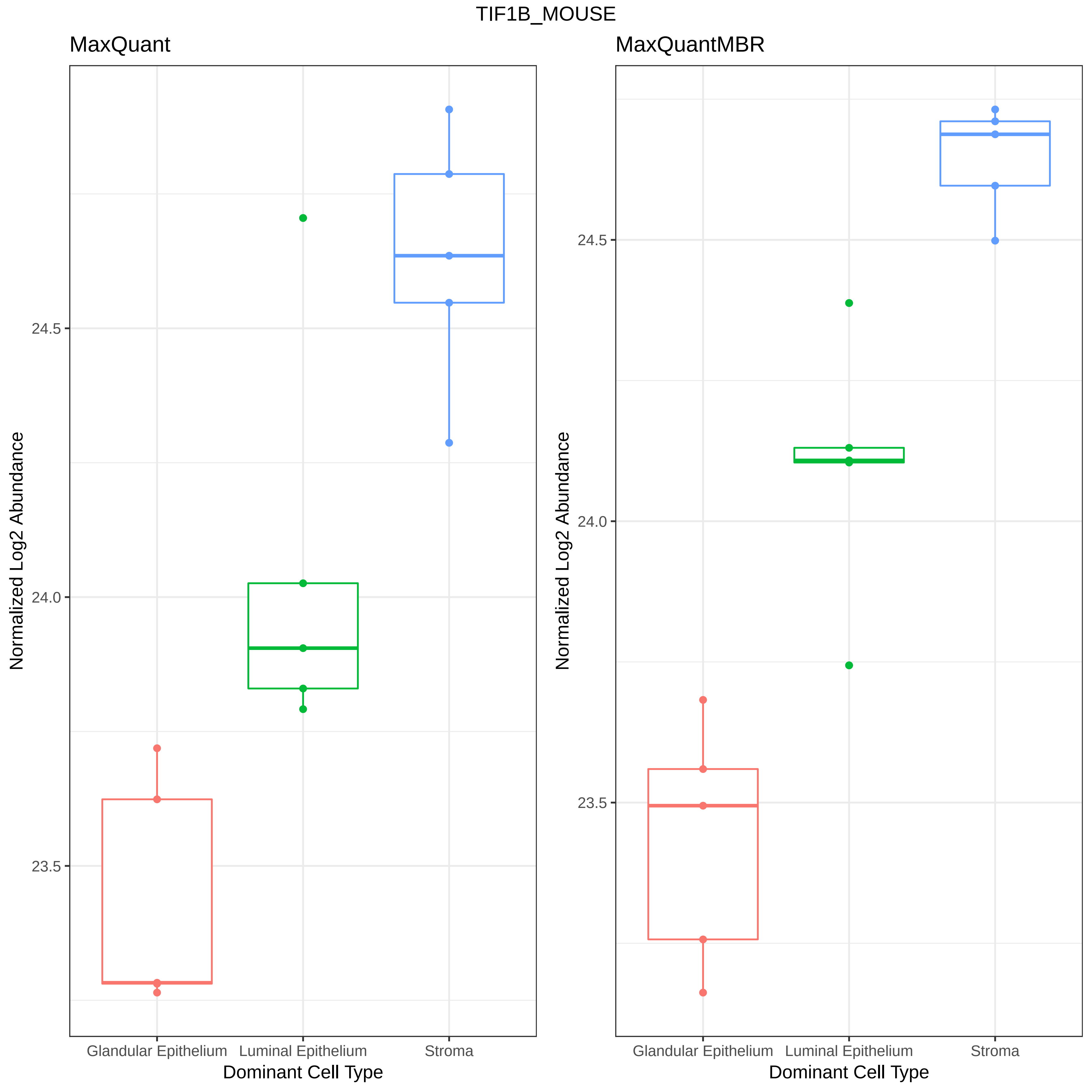

TIF1B_MOUSE
MaxQuant
MaxQuantMBR
Normalized Log2 Abundance
24.5
24.0
23.5
Glandular Epithelium
Luminal Epithelium
Stroma
Dominant Cell Type

TIF1B_MOUSE

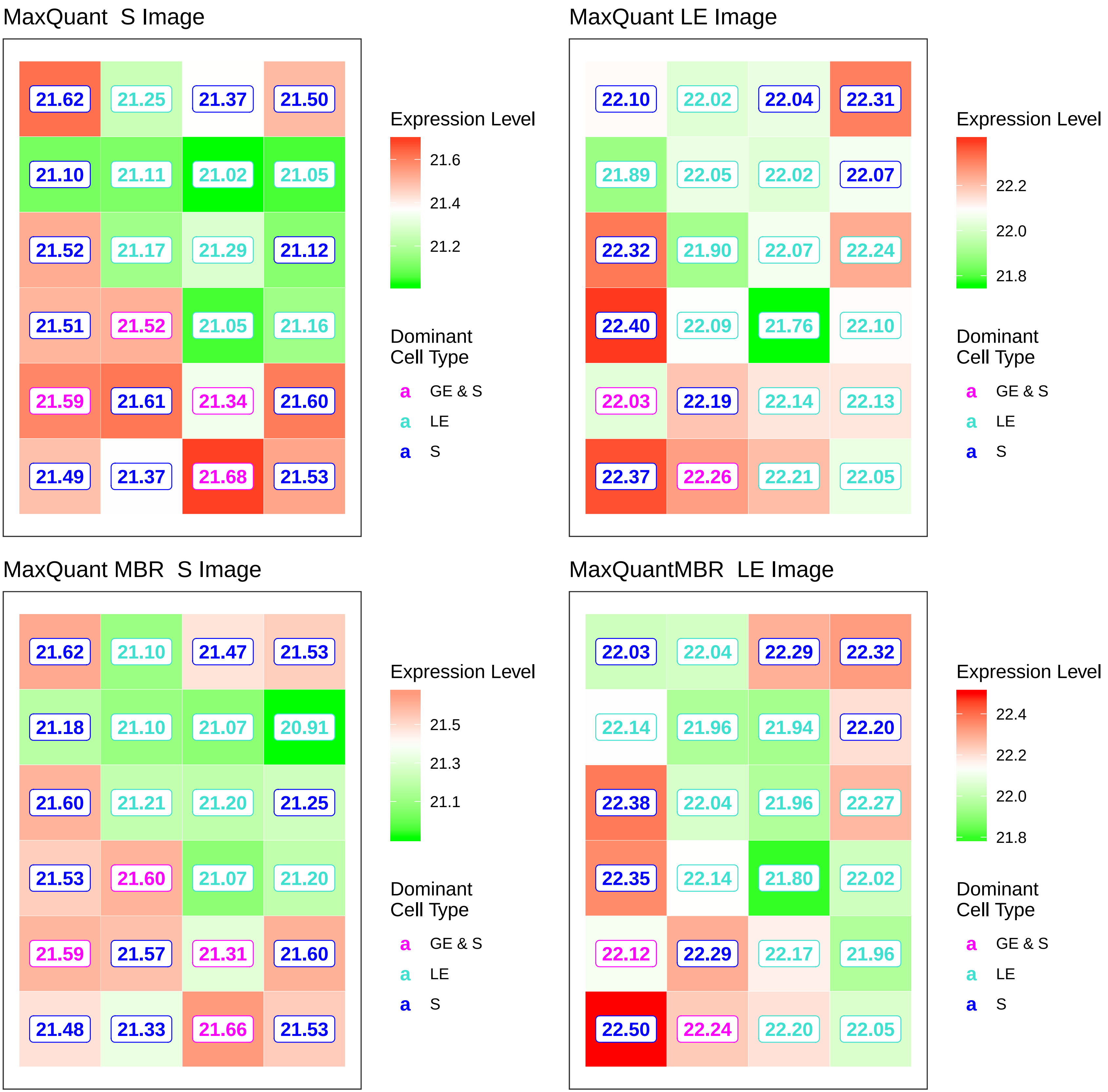
MaxQuant S Image
21.62
21.25
21.37
21.50
21.10
21.11
21.02
21.05
21.52
21.17
21.29
21.12
21.51
21.52
21.05
21.16
21.59
21.61
21.34
21.60
21.49
21.37
21.68
21.53
Expression Level
21.6
21.4
21.2
Dominant Cell Type
a GE & S
a LE
a S
MaxQuant LE Image
22.10
22.02
22.04
22.31
21.89
22.05
22.02
22.07
22.32
21.90
22.07
22.24
22.40
22.09
21.76
22.10
22.03
22.19
22.14
22.13
22.37
22.26
22.21
22.05
Expression Level
22.2
22.0
21.8
Dominant Cell Type
a GE & S
a LE
a S
MaxQuant MBR S Image
21.62
21.10
21.47
21.53
21.18
21.10
21.07
20.91
21.60
21.21
21.20
21.25
21.53
21.60
21.07
21.20
21.59
21.57
21.31
21.60
21.48
21.33
21.66
21.53
Expression Level
21.5
21.3
21.1
Dominant Cell Type
a GE & S
a LE
a S
MaxQuantMBR LE Image
22.03
22.04
22.29
22.32
22.14
21.96
21.94
22.20
22.38
22.04
21.96
22.27
22.35
22.14
21.80
22.02
22.12
22.29
22.17
21.96
22.50
22.24
22.20
22.05
Expression Level
22.4
22.2
22.0
21.8
Dominant Cell Type
a GE & S
a LE
a S

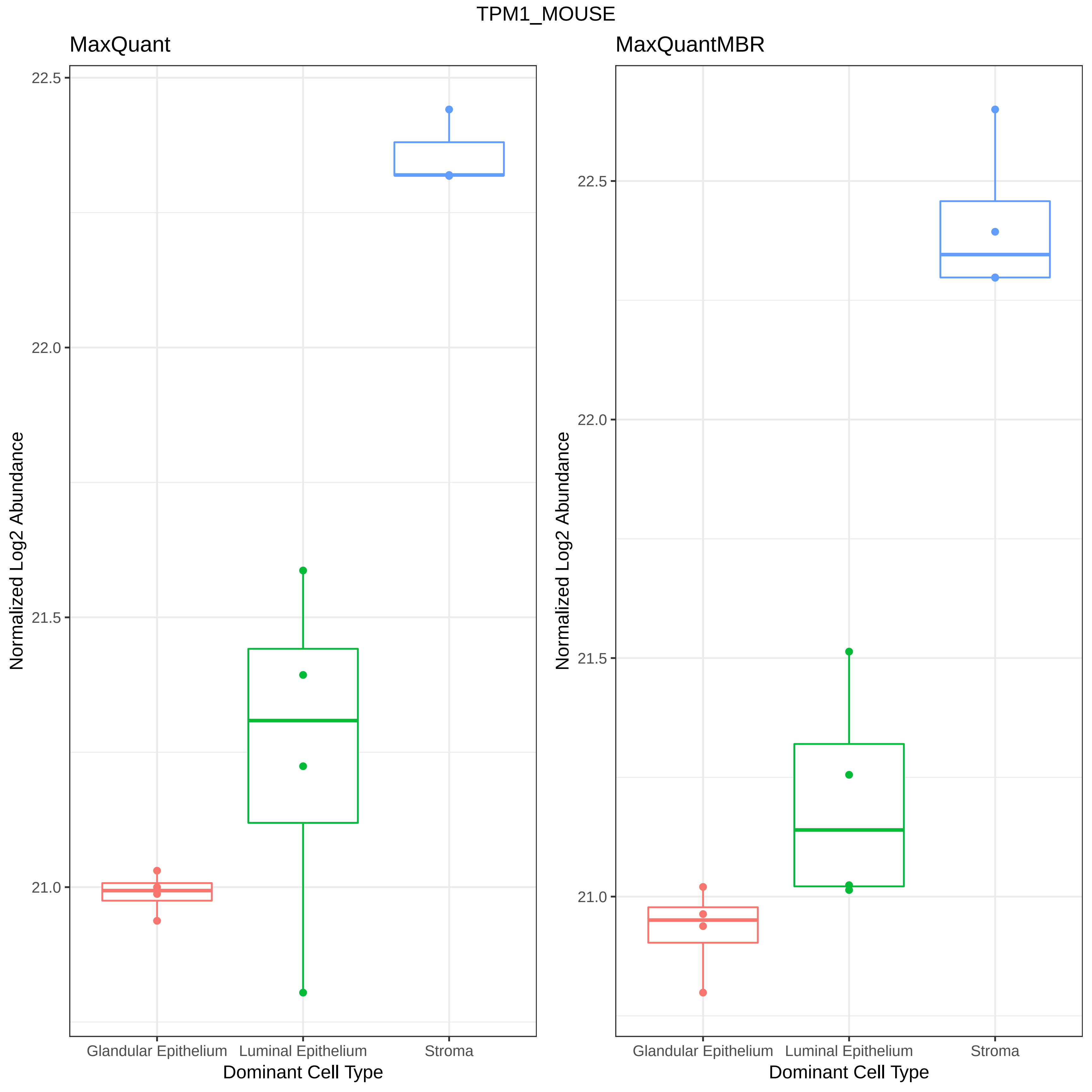

TPM1_MOUSE
MaxQuant
MaxQuantMBR
22.5
22.0
21.5
21.0
Normalized Log2 Abundance
Glandular Epithelium
Luminal Epithelium
Stroma
Dominant Cell Type

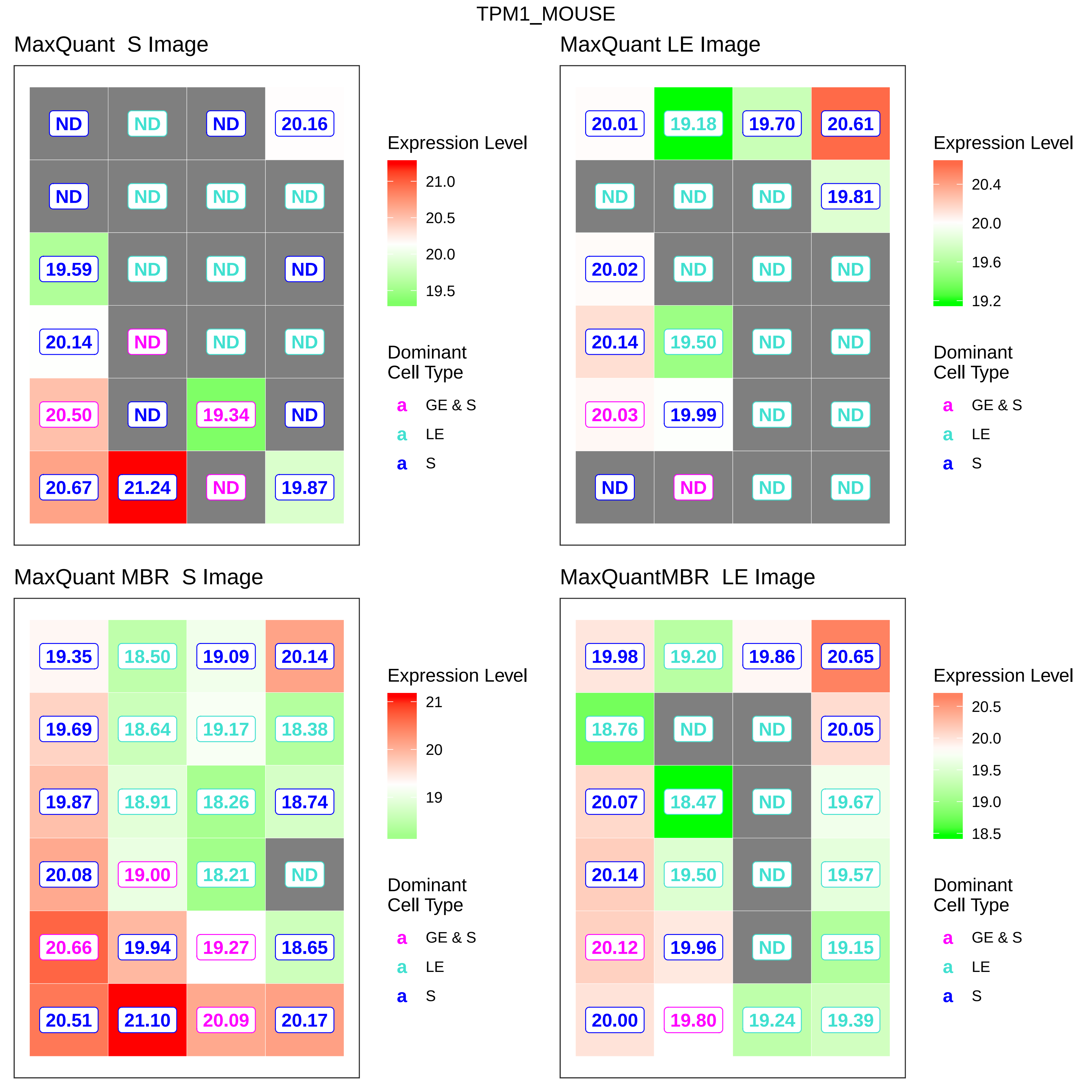
TPM1_MOUSE
MaxQuant S Image
ND ND ND 20.16
ND ND ND ND
19.59 ND ND ND
20.14 ND ND ND
20.50 ND 19.34 ND
20.67 21.24 ND 19.87
Expression Level
21.0
20.5
20.0
19.5
Dominant Cell Type
a GE & S
a LE
a S
MaxQuant LE Image
20.01 19.18 19.70 20.61
ND ND ND 19.81
20.02 ND ND ND
20.14 19.50 ND ND
20.03 19.99 ND ND
ND ND ND ND
Expression Level
20.4
20.0
19.6
19.2
Dominant Cell Type
a GE & S
a LE
a S
MaxQuant MBR S Image
19.35 18.50 19.09 20.14
19.69 18.64 19.17 18.38
19.87 18.91 18.26 18.74
20.08 19.00 18.21 ND
20.66 19.94 19.27 18.65
20.51 21.10 20.09 20.17
Expression Level
21
20
19
Dominant Cell Type
a GE & S
a LE
a S
MaxQuantMBR LE Image
19.98 19.20 19.86 20.65
18.76 ND ND 20.05
20.07 18.47 ND 19.67
20.14 19.50 ND 19.57
20.12 19.96 ND 19.15
20.00 19.80 19.24 19.39
Expression Level
20.5
20.0
19.5
19.0
18.5
Dominant Cell Type
a GE & S
a LE
a S

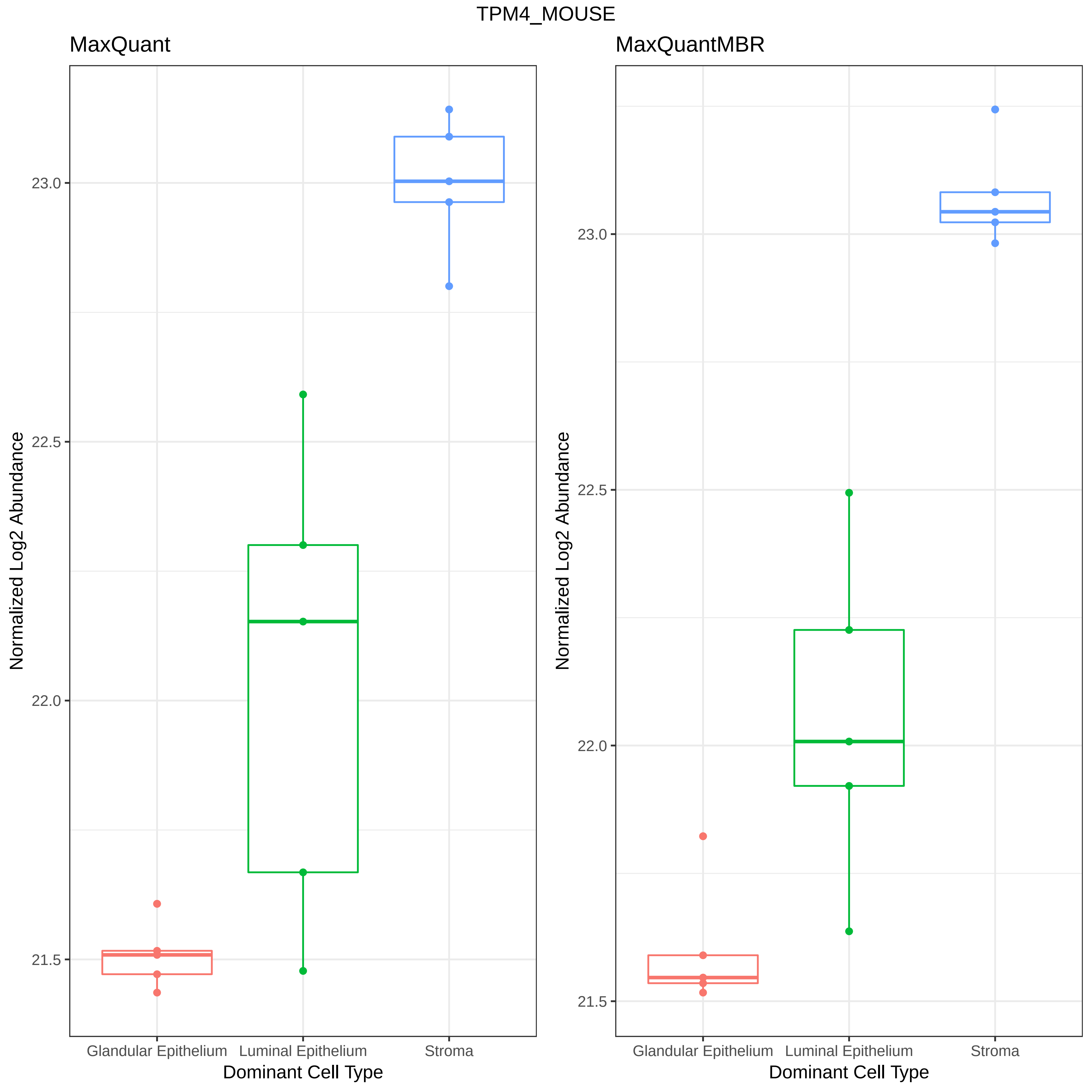

TPM4_MOUSE
MaxQuant
MaxQuantMBR
Normalized Log2 Abundance
23.0
22.5
22.0
21.5
Glandular Epithelium
Luminal Epithelium
Stroma
Dominant Cell Type

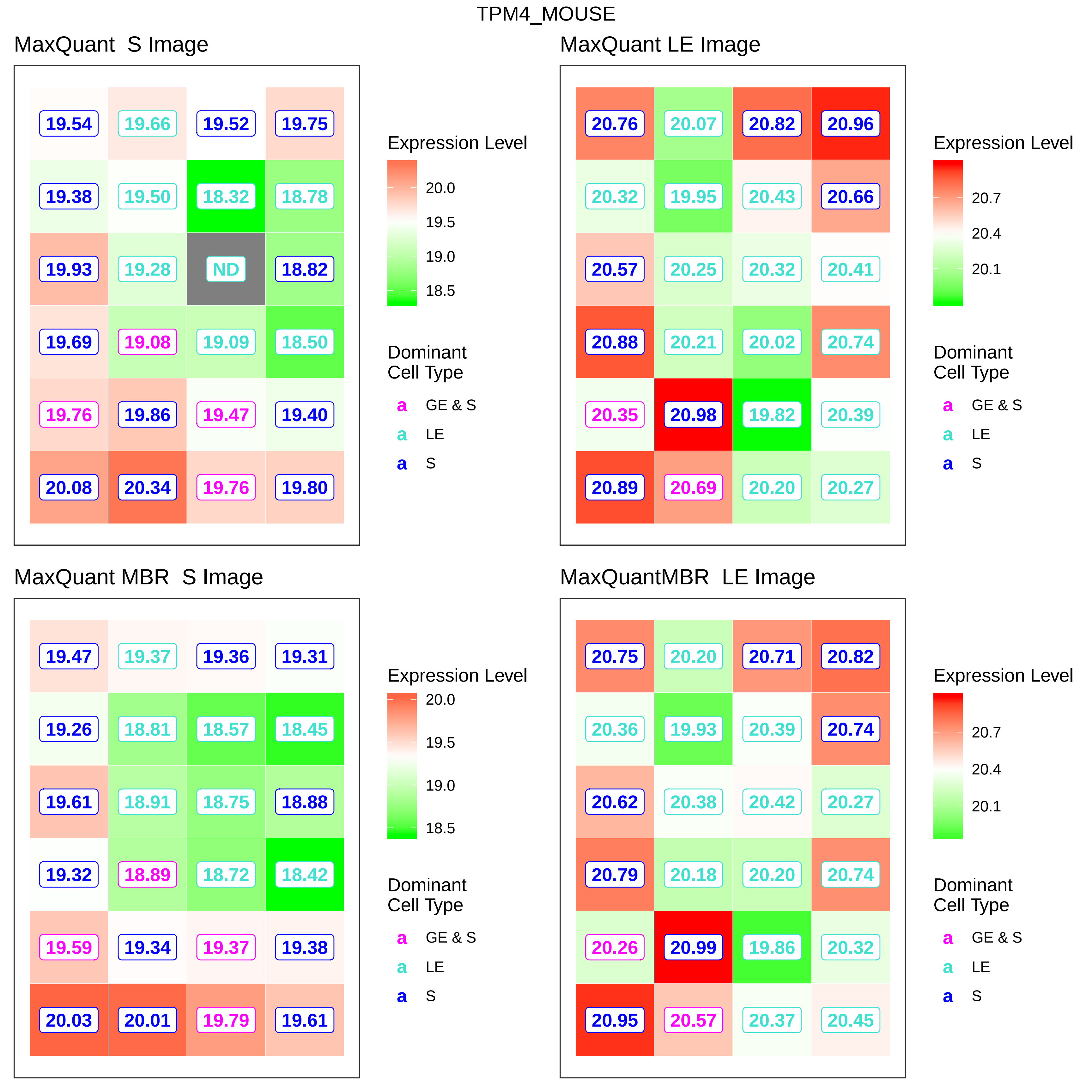
TPM4_MOUSE
MaxQuant S Image
19.54 19.66 19.52 19.75
19.38 19.50 18.32 18.78
19.93 19.28 ND 18.82
19.69 19.08 19.09 18.50
19.76 19.86 19.47 19.40
20.08 20.34 19.76 19.80
Expression Level
20.0
19.5
19.0
18.5
Dominant Cell Type
a GE & S
a LE
a S
MaxQuant LE Image
20.76 20.07 20.82 20.96
20.32 19.95 20.43 20.66
20.57 20.25 20.32 20.41
20.88 20.21 20.02 20.74
20.35 20.98 19.82 20.39
20.89 20.69 20.20 20.27
Expression Level
20.7
20.4
20.1
Dominant Cell Type
a GE & S
a LE
a S
MaxQuant MBR S Image
19.47 19.37 19.36 19.31
19.26 18.81 18.57 18.45
19.61 18.91 18.75 18.88
19.32 18.89 18.72 18.42
19.59 19.34 19.37 19.38
20.03 20.01 19.79 19.61
Expression Level
20.0
19.5
19.0
18.5
Dominant Cell Type
a GE & S
a LE
a S
MaxQuantMBR LE Image
20.75 20.20 20.71 20.82
20.36 19.93 20.39 20.74
20.62 20.38 20.42 20.27
20.79 20.18 20.20 20.74
20.26 20.99 19.86 20.32
20.95 20.57 20.37 20.45
Expression Level
20.7
20.4
20.1
Dominant Cell Type
a GE & S
a LE
a S

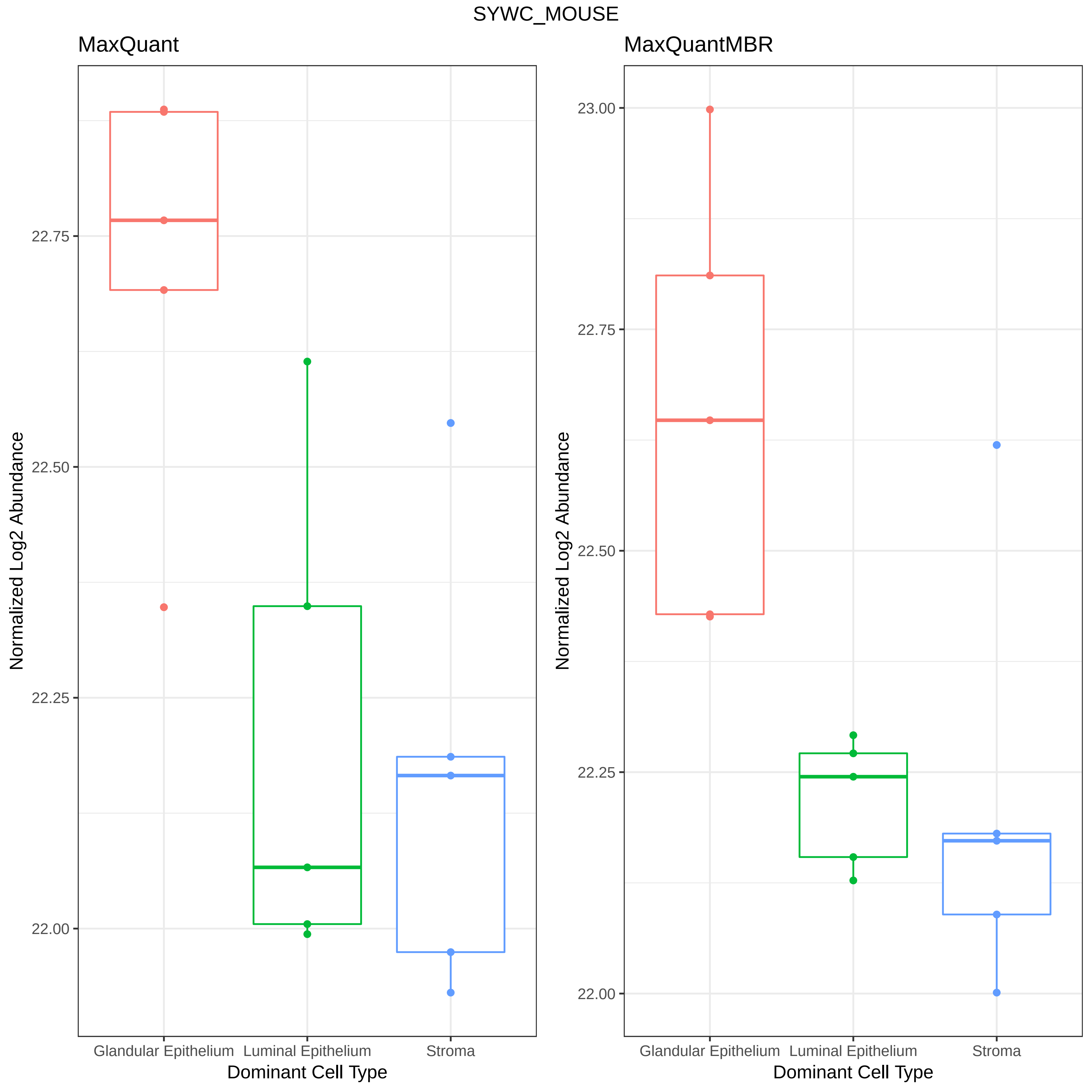

SYWC_MOUSE
MaxQuant
MaxQuantMBR
Normalized Log2 Abundance
22.75
22.50
22.25
22.00
23.00
22.75
22.50
22.25
22.00
Glandular Epithelium
Luminal Epithelium
Stroma
Glandular Epithelium
Luminal Epithelium
Stroma
Dominant Cell Type
Dominant Cell Type

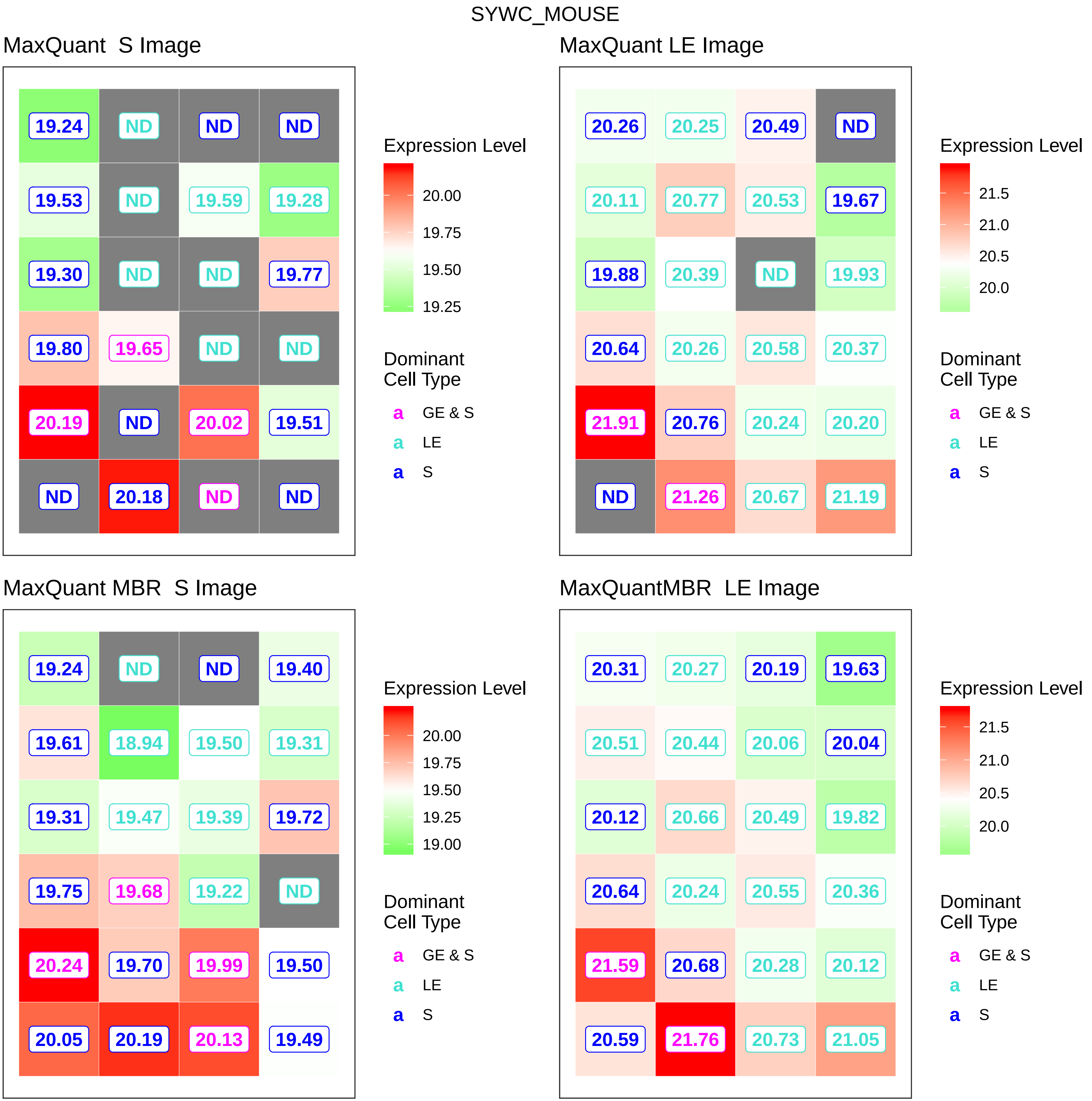
SYWC_MOUSE
MaxQuant S Image
19.24 ND ND ND
19.53 ND 19.59 19.28
19.30 ND ND 19.77
19.80 19.65 ND ND
20.19 ND 20.02 19.51
ND 20.18 ND ND
Expression Level
20.00
19.75
19.50
19.25
Dominant Cell Type
a GE & S
a LE
a S
MaxQuant LE Image
20.26 20.25 20.49 ND
20.11 20.77 20.53 19.67
19.88 20.39 ND 19.93
20.64 20.26 20.58 20.37
21.91 20.76 20.24 20.20
ND 21.26 20.67 21.19
Expression Level
21.5
21.0
20.5
20.0
Dominant Cell Type
a GE & S
a LE
a S
MaxQuant MBR S Image
19.24 ND ND 19.40
19.61 18.94 19.50 19.31
19.31 19.47 19.39 19.72
19.75 19.68 19.22 ND
20.24 19.70 19.99 19.50
20.05 20.19 20.13 19.49
Expression Level
20.00
19.75
19.50
19.25
19.00
Dominant Cell Type
a GE & S
a LE
a S
MaxQuantMBR LE Image
20.31 20.27 20.19 19.63
20.51 20.44 20.06 20.04
20.12 20.66 20.49 19.82
20.64 20.24 20.55 20.36
21.59 20.68 20.28 20.12
20.59 21.76 20.73 21.05
Expression Level
21.5
21.0
20.5
20.0
Dominant Cell Type
a GE & S
a LE
a S

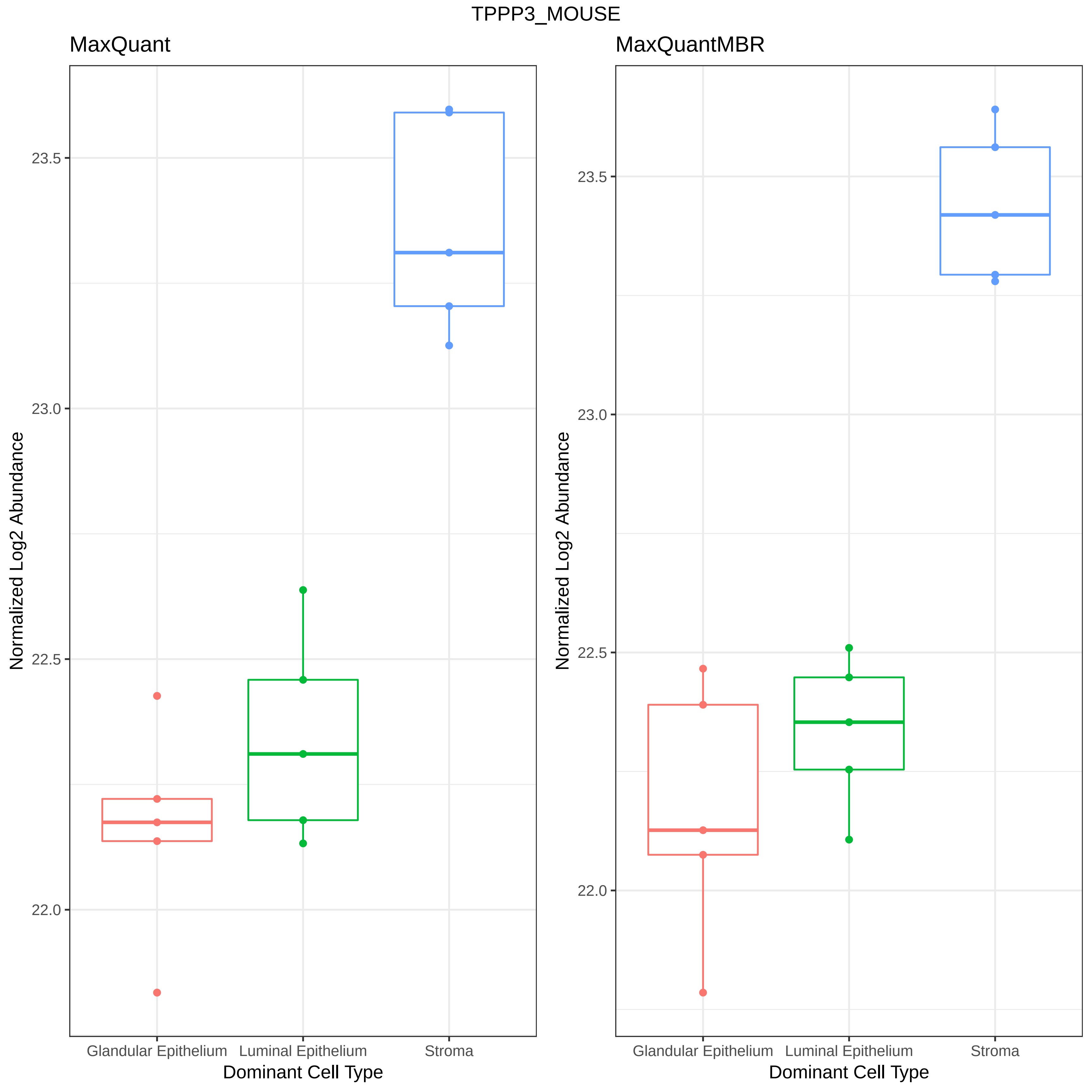
TPPP3_MOUSE
MaxQuant
MaxQuantMBR
Normalized Log2 Abundance
23.5
23.0
22.5
22.0
Glandular Epithelium
Luminal Epithelium
Stroma
Dominant Cell Type

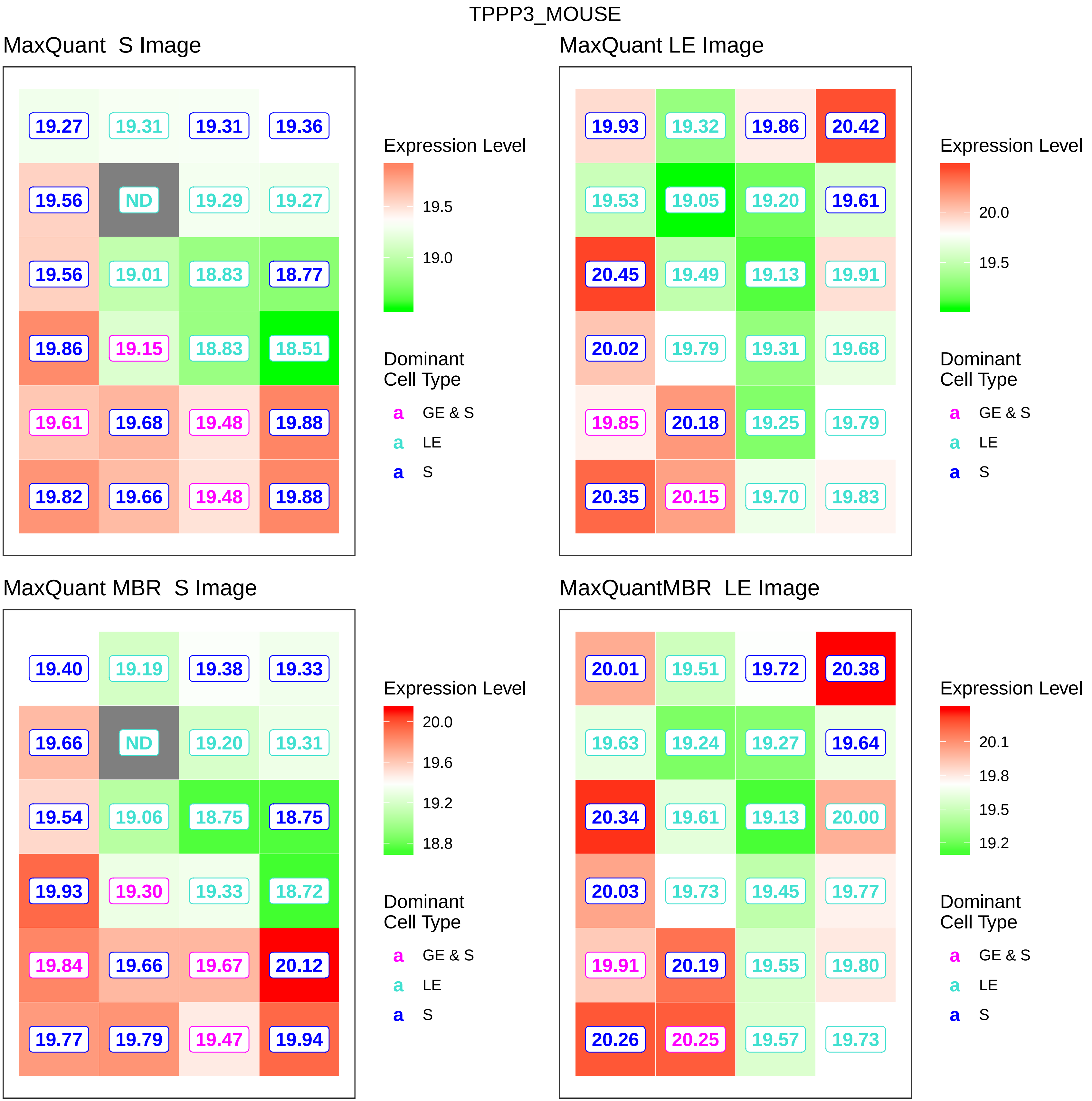
TPPP3_MOUSE
MaxQuant S Image
19.27 19.31 19.31 19.36
19.56 ND 19.29 19.27
19.56 19.01 18.83 18.77
19.86 19.15 18.83 18.51
19.61 19.68 19.48 19.88
19.82 19.66 19.48 19.88
Expression Level
19.5
19.0
Dominant Cell Type
a GE & S
a LE
a S
MaxQuant LE Image
19.93 19.32 19.86 20.42
19.53 19.05 19.20 19.61
20.45 19.49 19.13 19.91
20.02 19.79 19.31 19.68
19.85 20.18 19.25 19.79
20.35 20.15 19.70 19.83
Expression Level
20.0
19.5
Dominant Cell Type
a GE & S
a LE
a S
MaxQuant MBR S Image
19.40 19.19 19.38 19.33
19.66 ND 19.20 19.31
19.54 19.06 18.75 18.75
19.93 19.30 19.33 18.72
19.84 19.66 19.67 20.12
19.77 19.79 19.47 19.94
Expression Level
20.0
19.6
19.2
18.8
Dominant Cell Type
a GE & S
a LE
a S
MaxQuantMBR LE Image
20.01 19.51 19.72 20.38
19.63 19.24 19.27 19.64
20.34 19.61 19.13 20.00
20.03 19.73 19.45 19.77
19.91 20.19 19.55 19.80
20.26 20.25 19.57 19.73
Expression Level
20.1
19.8
19.5
19.2
Dominant Cell Type
a GE & S
a LE
a S

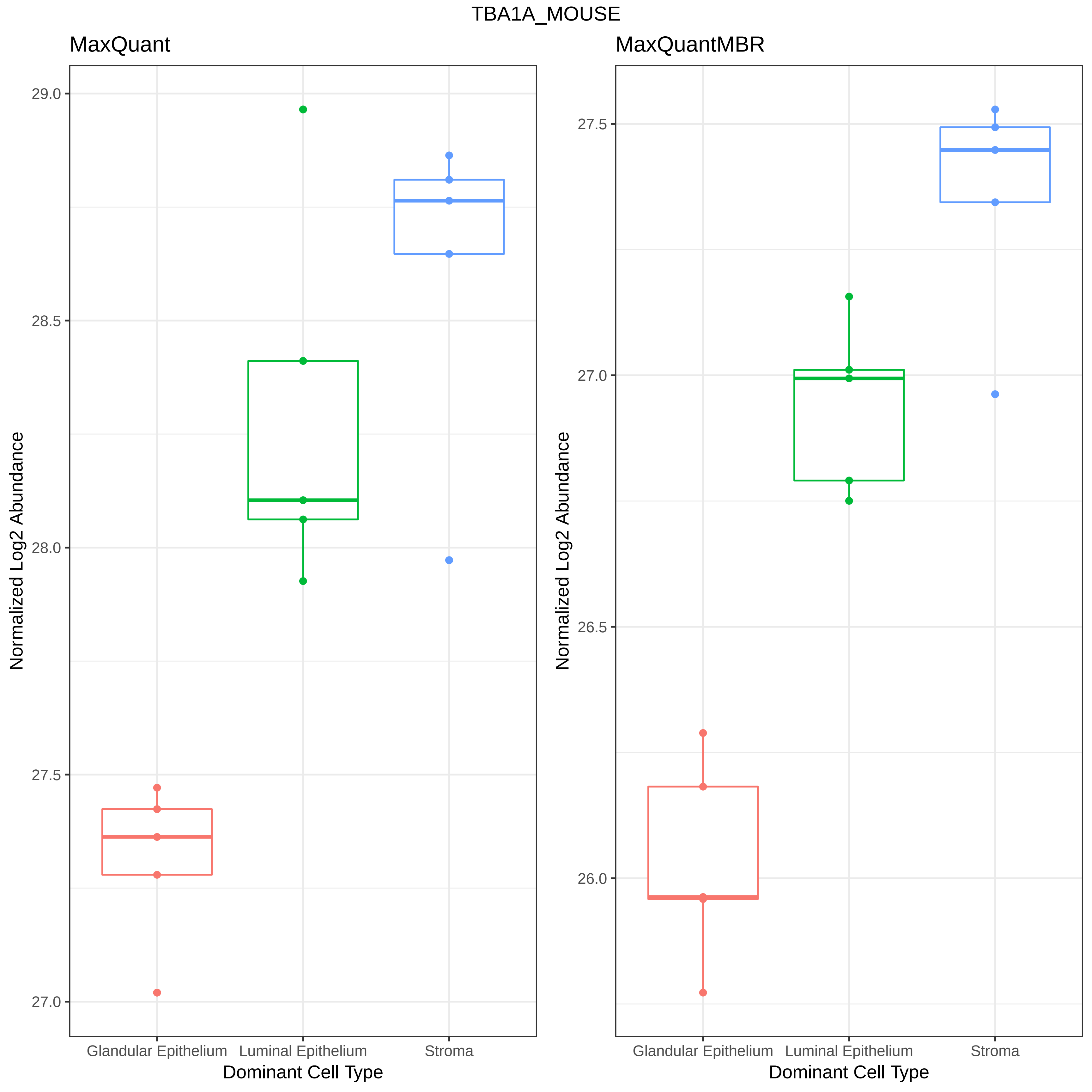

TBA1A_MOUSE
MaxQuant
MaxQuantMBR
Normalized Log2 Abundance
29.0
28.5
28.0
27.5
27.0
26.5
26.0
Glandular Epithelium
Luminal Epithelium
Stroma
Dominant Cell Type

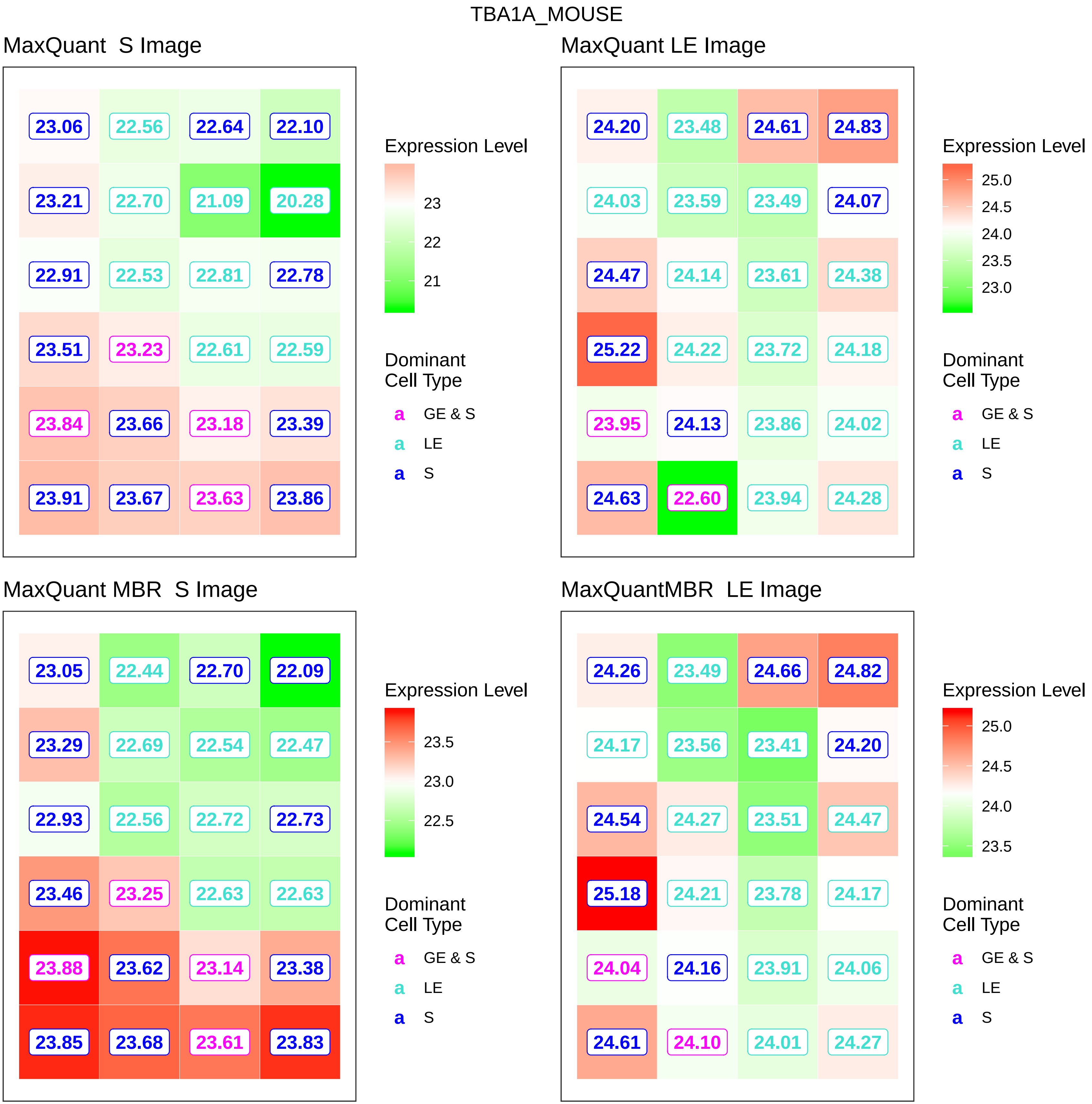
TBA1A_MOUSE
MaxQuant S Image
23.06 22.56 22.64 22.10
23.21 22.70 21.09 20.28
22.91 22.53 22.81 22.78
23.51 23.23 22.61 22.59
23.84 23.66 23.18 23.39
23.91 23.67 23.63 23.86
Expression Level
23
22
21
Dominant Cell Type
a GE & S
a LE
a S
MaxQuant LE Image
24.20 23.48 24.61 24.83
24.03 23.59 23.49 24.07
24.47 24.14 23.61 24.38
25.22 24.22 23.72 24.18
23.95 24.13 23.86 24.02
24.63 22.60 23.94 24.28
Expression Level
25.0
24.5
24.0
23.5
23.0
Dominant Cell Type
a GE & S
a LE
a S
MaxQuant MBR S Image
23.05 22.44 22.70 22.09
23.29 22.69 22.54 22.47
22.93 22.56 22.72 22.73
23.46 23.25 22.63 22.63
23.88 23.62 23.14 23.38
23.85 23.68 23.61 23.83
Expression Level
23.5
23.0
22.5
Dominant Cell Type
a GE & S
a LE
a S
MaxQuantMBR LE Image
24.26 23.49 24.66 24.82
24.17 23.56 23.41 24.20
24.54 24.27 23.51 24.47
25.18 24.21 23.78 24.17
24.04 24.16 23.91 24.06
24.61 24.10 24.01 24.27
Expression Level
25.0
24.5
24.0
23.5
Dominant Cell Type
a GE & S
a LE
a S

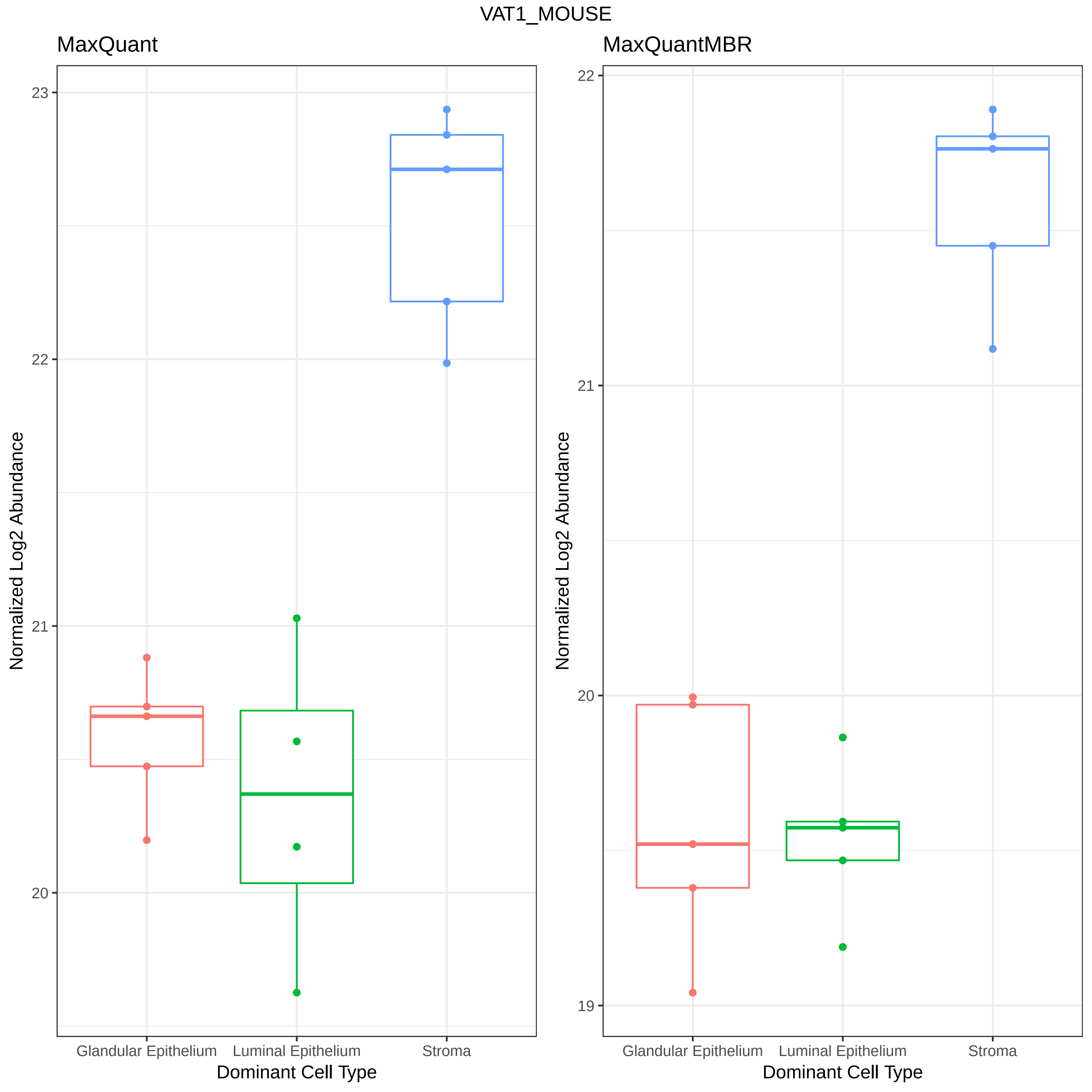
VAT1_MOUSE
MaxQuant
MaxQuantMBR
Normalized Log2 Abundance
23
22
21
20
19
Glandular Epithelium
Luminal Epithelium
Stroma
Dominant Cell Type

VAT1_MOUSE

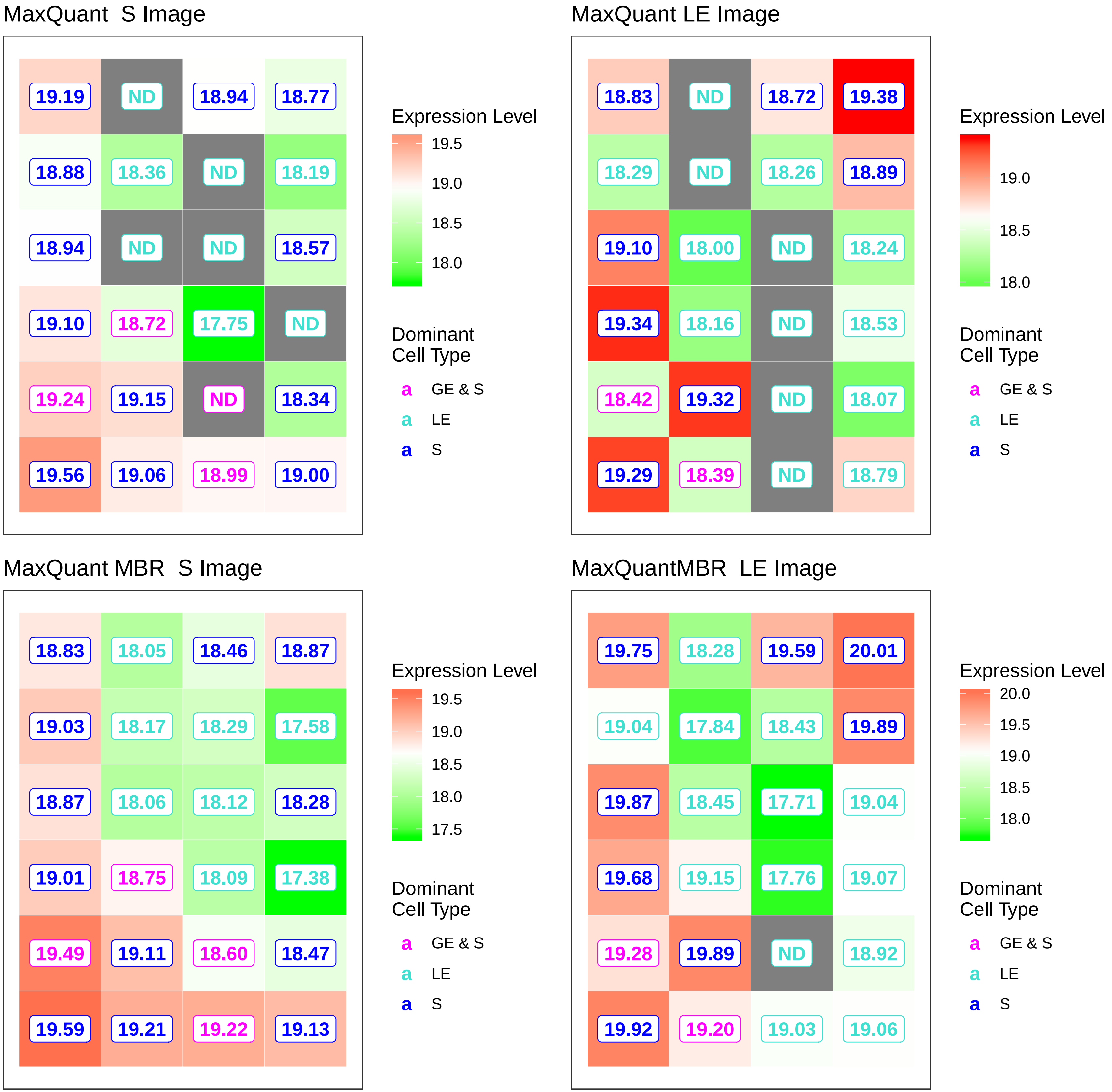
MaxQuant S Image
19.19
ND
18.94
18.77
18.88
18.36
ND
18.19
18.94
ND
ND
18.57
19.10
18.72
17.75
ND
19.24
19.15
ND
18.34
19.56
19.06
18.99
19.00
Expression Level
19.5
19.0
18.5
18.0
Dominant Cell Type
a GE & S
a LE
a S
MaxQuant LE Image
18.83
ND
18.72
19.38
18.29
ND
18.26
18.89
19.10
18.00
ND
18.24
19.34
18.16
ND
18.53
18.42
19.32
ND
18.07
19.29
18.39
ND
18.79
Expression Level
19.0
18.5
18.0
Dominant Cell Type
a GE & S
a LE
a S
MaxQuant MBR S Image
18.83
18.05
18.46
18.87
19.03
18.17
18.29
17.58
18.87
18.06
18.12
18.28
19.01
18.75
18.09
17.38
19.49
19.11
18.60
18.47
19.59
19.21
19.22
19.13
Expression Level
19.5
19.0
18.5
18.0
17.5
Dominant Cell Type
a GE & S
a LE
a S
MaxQuantMBR LE Image
19.75
18.28
19.59
20.01
19.04
17.84
18.43
19.89
19.87
18.45
17.71
19.04
19.68
19.15
17.76
19.07
19.28
19.89
ND
18.92
19.92
19.20
19.03
19.06
Expression Level
20.0
19.5
19.0
18.5
18.0
Dominant Cell Type
a GE & S
a LE
a S
