# Supplementary material for: Automated mass spectrometry imaging of over 2000 proteins from tissue sections at 100-μm spatial resolution
Source: Nat Commun. 2020 Jan 7;11:8. doi: 10.1038/s41467-019-13858-z (PMC6946663; doi:10.1038/s41467-019-13858-z)
Supplement: Supplementary file 2 — Peer Review File [file 41467_2019_13858_MOESM2_ESM.pdf]

**Editorial Note:** Parts of this peer review file have been redacted as indicated to remove third-party material where no permission to publish could be obtained.

Reviewers' comments:

Reviewer #1 (Remarks to the Author):

Work by Piehowski et al. entitled Automated mass spectrometry imaging of over 2000 proteins from tissue sections at 100-μm spatial resolution describes a novel method in the field of imaging mass spectrometry (IMS). The work is novel and warrants publication.

Specifically they demonstrate an automated IMS method using label-free quantitative (LFQ) proteomics to analyze tissue voxels that incorporates prior innovations from Prof Kelly et al. on single cell proteomics using the NanoPOTS device. Here this is coupled with laser capture microdissection (LCM) for automated sample collection and processing and coupled this to custom NanoLC system to create proteome maps via voxels provided by the LCM. This process produced quantitative cell-type-specific images for >2000 proteins at 100-μm spatial resolution across mouse uterine tissue sections preparing for blastocyst implantation. The data is then analyzed using Trelliscope – software for processing and analyzing very large datasets (as generated here) first as subsets and then in total by combining the subsets.

The field of single cell proteomics has taken off in recent years with NanoPOTS by Kelly et al. and other publications. The combination of this concept that avoids adsorptive loss of proteins or peptides during processing for LCMSMS with IMS of uterine tissue will be of broad interest to the community.

On a more subjective note, do you feel that the paper will influence thinking in the field? While the NanoPOTS for the community to take up NanoPOTS directly us currently limitied due to the technical complexity of the devices involved, this report's use of NanoPOTS will influence how other think about doing such IMS experiments. It will surely lead to further developments that perhaps use of methods to avoid adsorptive losses but this report shows the way forward.

This reviewer has no further comments other than the report is well prepared and the English is adequate as are the references for understanding and reproducing the work. Publication as is recommended.

Reviewer #2 (Remarks to the Author):

The manuscript describes the application of the nanoPOTS protocol for imaging mass spectrometry based on division of the tissue into voxels, followed by LC-MS/MS of each voxel. The work is a clear development of approaches developed in the lab.

Voxel-based IMS (Petyuk et al Mapping Protein Abundance Patterns in the Brain Using Voxelation Combined with Liquid Chromatography and Mass Spectrometry. Methods. 2010 Feb; 50(2): 77).

NanoPOTS (Zhu et al. Nanodroplet processing platform for deep and quantitative proteome profiling of 10–100 mammalian cells. Nature Communications, volume 9, Article number: 882 (2018)).

In this regard the use of nanoPOTS is a natural progression of the unique technologies developed at PNNL, and certainly represents an interesting case study of what may be achieved using the

nanoPOTS platform. For example the spatial resolution reported here, 100um pixel, is comparable to that reported by MALDI imaging mass spectrometry 5 to 10 years ago. Many MALDI imaging experiments still use a similar spatial resolution in order to maintain throughput, but 20 um spatial resolution is now routinely available. It may be argued that the authors' pooling of "3-5 sections for each of the 15 samples" represents a loss of resolution, however that would be unfair as the depth resolution is still within the 100um lateral resolution.

The authors state that "our 100um voxel size perfectly captured the LE cells lining both sides of the uterine cavity". Clearly the authors' meaning of the word perfect differs from this reviewer. At best one could argue that it is sufficient to capture the broad histoarchitecture of the tissue, albeit from from perfectly.

The main problem this reviewer has with the current manuscript is that it does not offer anything novel. It is the nanoPOTS applied to voxelization. Yes, more images of proteins are reported, but the images are awful, consisting of just 24 pixels. The reason they consist of so few pixels is the very long pixel analysis time of the voxelization approach. approximately 2hrs per LC-MS/MS run, means a single 24 pixel image would require 2 days of continuous measurement time (not including any QC runs). Accordingly the utility of the approach, for imaging, is minimal because its low throughput is far from sufficient. One could argue that it could generate reference datasets, in the same manner as the Allen Brain Atlas for the transcriptome. However, a single 1cm2 tissue section imaged by nanoPOTS at 100um voxel size would require at least 3 yr measurement time. For biomedical research one could analyze a small number of pixels, e.g. 24 as used here. However, a small patient series of just 50 patients would require 100 days continuous measurement time (not including instrument QC or maintenance).

Several groups are now actively combining imaging mass spectrometry with laser capture microdissection and high performance LC-MS/MS, in order to dig deeper into the underlying biology indicated by the imaging mass spectrometry experiment. nanoPOTS has the potential to be able to better match the length scales of the imaging mass spectrometry experiment, than existing reports to date. However that is not the subject of the current manuscript.
In short, while undoubtedly technically excellent, the manuscript lacks novelty. There are many application areas that could benefit from the nanoPOTS technology. In this reviewer's opinion the authors would be better advised identifying suitable applications rather than shoehorning nanoPOTS as a new imaging mass spectrometry platform, for which it is simply unsuited.

Response to Referees for "Automated mass spectrometry imaging of over 2000 proteins from tissue sections at 100-µm spatial resolution (NCOMMS-18-34816-T)"

A single matrix assisted laser deposition ionization (MALDI) imaging mass spectrometry (IMS) experiment can produce thousands of ion images, providing molecular context to classical histological analysis, yet in order to identify the proteins, fragmentation data is often collected in time consuming separate experiments with lower spatial resolution either directly from tissue [1] or by liquid chromatography-tandem MS (LC–MS/MS) analyses following extraction [2].

The most recent review by Dr. Richard Caprioli [3], the developer of MALDI IMS and a leader in the field, states "Protein identification is crucial for the biological contextualization of molecular imaging data. However, gas-phase fragmentation efficiency of MALDI generated proteins presents significant challenges, making protein identification directly from tissue difficult." This review then highlights the secondary experiments that are required to identify MALDI proteins and peptides. **Our foundational manuscript illustrates how our novel nanoPOTS imaging platform provides unprecedented biological insight into the proteomic changes across heterogeneous tissue sections by yielding quantitative data and confident peptide identification in a single experiment**.

The below figure, taken directly from Dr. Caprioli's recent review [3], details these secondary protein identification experiments. These secondary analyses would need to be performed multiple times to identify proteins unique to distinct areas of heterogeneous tissues. These secondary measurements are at ≥250 µm resolution except for laser capture microdissection (LCM) which is listed at single cell. Robust identifications from proteomic analysis of LCM single cells can only be achieved with a microfluidic system such as nanoPOTS.

This comprehensive review was not published when we submitted our manuscript and we should have added more information and references to our introduction. We feel if we had done this in our first draft, Reviewer 2 would have been more enthusiastic about the development of our nanoPOTS proteomic imaging technology.

Our introduction now states "*A single matrix-assisted laser deposition/ionization (MALDI) IMS experiment can produce thousands of ion images, providing molecular context to classical histological analysis, yet in order to identify the proteins, fragmentation data is often collected in separate experiments (reviewed in [6]) either directly from tissue or by liquid chromatography-tandem MS (LC–MS/MS) following extraction.[7,8] Protein coverage using IMS can be improved through on-tissue digestion, but confident in situ MS/MS peptide identification remains challenging due to low signal-to-noise ratios and high spectral complexity which impede database identifications. To increase the number of identified peptides, researchers have coupled IMS with LC-MS, where one tissue section is analyzed by IMS, while an adjacent section is homogenized and analyzed by LC-MS/MS.[9-11] However, linking these two MS modalities is challenging due to the high complexity of mammalian tissue sections, which has led to false-positive assignments.[12] As a result, there is currently no IMS technology capable of in-depth proteome imaging.*"

[redacted]

Reviewer 2 comments in black followed by our responses in blue

The manuscript describes the application of the nanoPOTS protocol for imaging mass spectrometry based on division of the tissue into voxels, followed by LC-MS/MS of each voxel. The work is a clear development of approaches developed in the lab.

Voxel-based IMS (Petyuk et al Mapping Protein Abundance Patterns in the Brain Using Voxelation Combined with Liquid Chromatography and Mass Spectrometry. Methods. 2010 Feb; 50(2): 77).

Response: The 2010 paper studied 1 mm x 1 mm x 1 mm tissue voxels that were obtained by manual dissection, whereas the present work achieved greater proteome coverage for 100 µm x100 µm x 12 µm voxels containing >8,000X less tissue. In addition, the present workflow is compatible with automated microdissection of histological thin sections that are routinely obtained in the clinic. As such, the comparison with the previous work studying 4-orders-of-magnitude larger samples highlights the magnitude of the present advance.

NanoPOTS (Zhu et al. Nanodroplet processing platform for deep and quantitative proteome profiling of 10–100 mammalian cells. Nature Communications, volume 9, Article number: 882 (2018)).

In this regard the use of nanoPOTS is a natural progression of the unique technologies developed at PNNL, and certainly represents an interesting case study of what may be achieved using the nanoPOTS platform. For example the spatial resolution reported here, 100um pixel, is comparable to that reported by MALDI imaging mass spectrometry 5 to 10 years ago. Many MALDI imaging experiments still use a similar spatial resolution in order to maintain throughput, but 20 um spatial resolution is now routinely available.
Response: As mentioned above, MALDI does NOT have 20 µm spatial resolution for protein imaging, it merely uses a 20 µm probe. Due to low ionization efficiency of most proteins/peptides, the effective spatial resolution of MALDI experiments is significantly lower [4]. Further, MALDI imaging does not provide protein or peptide identifications [3]; >2,000 proteins were identified in our nanoPOTS experiment without the need for orthogonal proteomics experiments. In addition, our peptide identifications are representative of the exact tissue piece being analyzed, giving reliable, accurate peptide identifications. MALDI utilizes *in situ* ionization which provides speed and resolution; however, every *in situ* pixel contains a unique composition of biomolecules which influences their ionization efficiency in an unpredictable manner, precluding accurate quantification. Our approach utilizes high resolution LC separations to mitigate these effects, providing highly accurate relative quantification unobtainable with MALDI.

It may be argued that the authors' pooling of "3-5 sections for each of the 15 samples" represents a loss of resolution, however that would be unfair as the depth resolution is still within the 100um lateral resolution.
Responses:
All nanoPOTS proteomic imagining results presented in the manuscript represent individual **unpooled 100 µm x 100 µm x 12 µm voxels**.
We also validated all of our images with robust statistical comparisons (p-values <0.05 for Tukey-adjusted ANOVA or a Holm-adjusted g-test) across 15 samples (n=5 for each our 3 cell types) **in a secondary non-imaging study where we pooled "3-5 sections for each of the 15 samples"** to obtain 100-200 ng of material for each sample.

The authors state that "our 100um voxel size perfectly captured the LE cells lining both sides of the uterine cavity". Clearly the authors' meaning of the word perfect differs from this reviewer. At best one could argue that it is sufficient to capture the broad histoarchitecture of the tissue, albeit from perfectly.
Response: We agree perfectly does not belong in the sentence. Our revised manuscript now states "our 100 µm voxel size was sufficient to capture the LE cells lining both sides of the uterine cavity".

The main problem this reviewer has with the current manuscript is that it does not offer anything novel. It is the nanoPOTS applied to voxelization. Yes, more images of proteins are reported, but the images are awful, consisting of just 24 pixels. The reason they consist of so few pixels is the very long pixel analysis

time of the voxelization approach. approximately 2hrs per LC-MS/MS run, means a single 24 pixel image would require 2 days of continuous measurement time (not including any QC runs). Accordingly the utility of the approach, for imaging, is minimal because its low throughput is far from sufficient. One could argue that it could generate reference datasets, in the same manner as the Allen Brain Atlas for the transcriptome. However, a single 1cm2 tissue section imaged by nanoPOTS at 100um voxel size would require at least 3 yr measurement time. For biomedical research one could analyze a small number of pixels, e.g. 24 as used here. However, a small patient series of
just 50 patients would require 100 days continuous measurement time (not including instrument QC or maintenance).

Responses:

We agree that increased throughput and resolution will always be beneficial. However, as the only current approach capable of such measurements, the work might be better judged relative to existing techniques. We would argue that members of the imaging mass spectrometry community would not find our 24 pixel images “awful” since this manuscript presents a significant advancement in the IMS field.  As clearly demonstrated in our manuscript, nanoPOTS imaging does not rely on secondary measurements of limited effectiveness to identify proteins and it was able to provide accurate quantitative imaging data for over >2,000 proteins.  We also developed a custom open-source software platform for interactive, facile visualization of protein images and statistically significant results.

Although our current nanoPOTS experiments are comparable in time to the orthogonal proteomic measurements required for identification of proteins/peptides observed in MALDI experiments [3] we agree these measurements are slow. The current goal of our nanoPOTS imaging approach is to provide in-depth analyses that can produce mechanistic insights into biological function and disease states. Ongoing instrumentation developments at PNNL such as Structures for Lossless Ion Manipulation (SLIM) for rapid gas-phase separations will provide a future path forward to achieving faster analyses. In addition, a recent LC system (Evosep One) has already achieve 200 LC-MS runs per day, without sacrificing in-depth proteome coverage [5]. We expect more technical developments that facilitate high-throughput proteomics to emerge in the near future.

Our manuscript clearly shows nanoPOTS proteome imaging of tissue heterogeneity with unprecedented spatial resolution and depth of coverage. Additional advantages of our nanoPOTS proteomic imaging platform is it is automated and therefore the time consuming LC-MS portion of our analyses can be performed unattended and we do not have any matrix effects that are inherent to MALDI measurements.

Several groups are now actively combining imaging mass spectrometry with laser capture microdissection and high performance LC-MS/MS, in order to dig deeper into the underlying biology indicated by the imaging mass spectrometry experiment. nanoPOTS has the potential to be able to better match the length scales of the imaging mass spectrometry experiment, than existing reports to date. However that is not the subject of the current manuscript.

Response: Again these are orthogonal experiments need to not only dig deeper into the data from the imaging mass spectrometry experiment, they are often needed to accurately annotate

the proteins/peptides identified by mass alone in the imaging mass spectrometry experiments especially across heterogeneous tissues where a protein may only exist in a limited amount of cells of a particular cell type.

In short, while undoubtedly technically excellent, the manuscript lacks novelty. There are many application areas that could benefit from the nanoPOTS technology. In this reviewer's opinion the authors would be better advised identifying suitable applications rather than shoehorning nanoPOTS as a new imaging mass spectrometry platform, for which it is simply unsuited.

Response: We are not shoehorning nanoPOTS, nanoPOTS proteomic imaging is a powerful platform that provides accurate peptide identifications for thousands of proteins directly from the imaged regions measuring only 100 µm x 100 µm x 12 µm, which negates the need for inaccurate reference databases currently used by other IMS technologies for inferring protein/peptide identifications since they annotate these molecules by mass alone. Further, LC-MS/MS measurements provide 4 orders of magnitude in dynamic range with accurate relative quantification greatly expanding the range of proteins that can be imaged. Even with reference libraries MALDI is limited to imaging at most the top 10% most abundant proteins in a sample, which are often not the proteins of interest to the biological researchers.

References Cited


1 Stauber, J. *et al.* On-tissue protein identification and imaging by MALDI-Ion mobility mass spectrometry. *Journal of the American Society for Mass Spectrometry* **21**, 338-347, doi:10.1016/j.jasms.2009.09.016 (2010).

2 Zhu, Y. *et al.* Spatially resolved proteome mapping of laser capture microdissected tissue with automated sample transfer to nanodroplets. *Molecular & Cellular Proteomics*, doi:10.1074/mcp.TIR118.000686 (2018).

3 Ryan, D. J., Spraggins, J. M. & Caprioli, R. M. Protein identification strategies in MALDI imaging mass spectrometry: a brief review. *Current Opinion in Chemical Biology* **48**, 64-72, doi:https://doi.org/10.1016/j.cbpa.2018.10.023 (2019).

4 van de Ven, S. M. W. Y. *et al.* Protein biomarkers on tissue as imaged via MALDI mass spectrometry: A systematic approach to study the limits of detection. *PROTEOMICS* **16**, 1660-1669, doi:doi:10.1002/pmic.201500515 (2016).

5 Bache, N. *et al.* A Novel LC System Embeds Analytes in Pre-formed Gradients for Rapid, Ultra-robust Proteomics. *Molecular & Cellular Proteomics* **17**, 2284-2296, doi:10.1074/mcp.TIR118.000853 (2018).
